# A high throughput method for identifying personalized tumor-associated antigens

**DOI:** 10.18632/oncotarget.118

**Published:** 2010-06-27

**Authors:** Yurij Ionov

**Affiliations:** Department of Cancer Genetics, Roswell Park Cancer Institute, Buffalo, NY

**Keywords:** Random peptide phage display library, tumor-associated antigens, colon cancer, cancer immunodiagnostics, personalized medicine

## Abstract

Circulating autoantibodies against tumor-associated antigens (TAAs) and their pattern of glycosylation can be used as diagnostic indicators of cancer. Using random peptide library screening, we identified patient-specific sets of peptides recognized by colon cancer patients' serum IgG and IgM antibodies. We demonstrate a strategy for analyzing BLAST search results for identifying tumor-associated antigens represented by peptides that mimic sequential epitopes. Statistical analysis of the frequency with which the proteins are retrieved by BLAST homology searching and an estimation of the probability of a match by chance can identify the proteins that are the real targets of the immune response against tumors. In addition, we observed an over-expression of the mRNA for the match-producing protein only in the corresponding tumor sample, out of fourteen tumor and normal samples analyzed. This observation confirms that personalized tumor-associated antigens can be identified by BLAST homology search following random peptide library screening on cancer patient's serum antibodies.

## INTRODUCTION

Tumor cells, often characterized by altered expression of proteins and their glycosylation patterns, induce humoral and cellular immune responses in the autologous host [1-6]. Circulating antibodies against tumor-associated antigens (TAAs) are a strong indicator of cancer and, due to a long half-life and high concentration in the blood, are easier to detect than the proteins they recognize. TAAs have usually been identified by using cancer patients' serum antibodies to screen cDNA libraries derived from autologous or heterologous tumors. However, screening cDNA libraries, besides being laborious and time consuming, fails to identify TAAs generated by aberrant glycosylation of cell membrane glycoproteins and glycolipids [7].

An alternative to using cDNA expression libraries for TAA identification is to screen Random Peptide Phage Display Libraries (RPPDLs) with serum antibodies from cancer patients. RPPDLs have been widely utilized to map protein interaction sites. Peptide libraries were first used for the assessment of antibody specificity [8]. In this assay, a RPPDL is incubated with a target antibody. Subsequently, phage bound to the antibody are eluted and amplified in host bacteria. This process, termed “biopanning,” can be repeated several times in order to obtain an enriched population of the best binders. Bound peptides are identified upon phage DNA sequencing. The amino acid sequences of the peptides binding to cancer-specific serum antibodies can mimic sequential and conformational epitopes of protein antigens [9] as well as carbohydrate epitopes of glycoproteins or glycolipids. The only drawback of RPPDL screening on serum antibodies is the lack of immediate information on the identities of the real antigens that are mimicked by the antibodybinding peptides. Meanwhile, establishing these identities is important for the design of personalized immunoassay tests for reliable and specific detection of cancer recurrency

It has been demonstrated that the identity of the antigens mimicked by peptides obtained from RPPDL screening can be determined using a proteomic approach [10]. RPPDL screening on serum antibodies from a prostate cancer patient identified a peptide motif associated with serum antibody reactivity that positively correlated with progression of prostate cancer. A glucose-regulated protein (GRP78), a member of the heat-shock protein family, recognized by antiserum raised against the selected peptide was identified through a series of biochemical approaches, including electrophoretic fractionation and mass spectrometry analysis. The peptide recognized by the prostate cancer patient's antibodies appeared to mimic a conformational epitope within the GRP78 protein. The large volume of work required for identifying TAAs based on peptides mimicking conformational epitopes diminishes the efficiency of RPPDL screening for development of personalized immunoassay tests. Our work demonstrates that for peptides that mimic sequential epitopes, the corresponding TAAs can be easily identified by protein database homology searches using the basic local alignment search tool (BLAST).

## RESULTS

### Biopanning of peptide libraries on antibodies from colorectal cancer patient sera

**Fig. 1:**
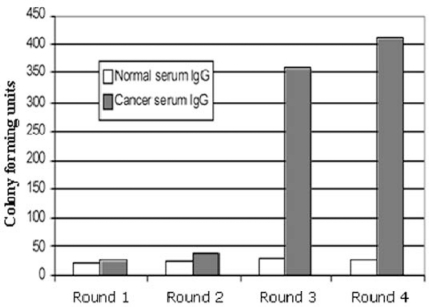
Enrichment of RPPD library with phage particles that bind specifically to IgG antibodies from the serum of CRC patient A after four rounds of biopanning. After each round of pre-adsorption on an excess of normal serum IgG and affinity selection on patient A IgG, amplified phage particles were purified and quantified. Equal amounts of phage (10^7^ cfu) were incubated for 1 hour with normal serum IgG and cancer patient A IgG immobilized on protein G agarose. After washing out unbound phage, the remaining phage were eluted and used to infect bacteria. Aliquots of infected bacteria were spread on LB plates containing tetracycline. The next day, the number of colonies on plates corresponding to normal IgG and cancer patient A's IgG were quantified.

To identify peptide sequences recognized by cancer-specific serum antibodies, we used a mixture of several peptide libraries of variable peptide length for screening on IgG antibodies from the serum samples of seven colorectal cancer (CRC) patients. Four cycles of selection/amplification were performed on each serum sample to achieve enrichment in antibody-binding phage. Each cycle consisted of an initial step of library preadsorption on normal immunoglobulins, followed by an affinity selection on antibodies from the cancer patient serum and amplification of the phage particles thus selected in bacteria, as described [10]. Serum samples from four CRC patients, hereafter referred to as patients A, B, C, and D, were enrichment-positive, implying recovery of phage particles reacting with cancer-specific antibodies; sera pooled from 24 healthy donors served as the negative control. Two sera positive for enrichment on IgG antibodies were also tested for enrichment on IgM antibodies. Each serum gave rise to phage enrichment, as the phage bound the corresponding IgM antibodies but not the IgM antibodies present in the control. Figure 1 shows the enrichment of phage particles that specifically bind to IgG antibodies from a CRC patient serum after four rounds of successful selection. We also performed library biopanning with sera obtained from four healthy donors. No phage enrichment was observed as compared to the control serum.

For each positive serum, the enriched phage population typically contained several phage groups with identical inserts. Some groups included sequences that were different but contained overlapping fragments (motifs), usually four or five amino acids in length. Several singularly represented inserts were also recovered. The peptide sequences derived from phage binding to IgG antibodies were different from those binding to IgM antibodies, from the same serum. Table 1 shows the sequences of peptides identified for each positive serum. We also sequenced 11 inserts from phage isolated after one round of biopanning with IgG from a healthy individual. All sequences were different.

**Table 1:** Peptide sequences selected from RPPDLs and the corresponding matching over-represented proteins

Patient	Ab type	Peptide sequences from “biopanning” selected phages			Overrepresented proteins identified by BLAST search
A	IgG	TGVRGQRISQ(9)	QNPGETSKMN(6)^a^	KYRWYK(3)^a^	^a^NP_055982.2 **bifunctional arginine demethylase and lysyl-hydroxylase JMJD6 (2)**
A	IgM	AVHFPD DLITPGD (2) AEPPFEF (2) PSKAAYVV^a^ (3)	**QD**LY**SSA** (3) **QD**IF**SSA** (3) MSSVMTY^a^	FQSPK^b^ (2) ASHQNRP FRQAAS	^a^NP_078966.2 **Mucin 16 (2)** ^b^NP_002025.2 **Fucosyltransferase 5** ^b^NP_000140.1 **Fucosyltransferase 3** ^b^NP_000141.1 **Fucosyltransferase 6**
B	IgG	FSRRAQQVGAK(3) DHNRSM SHNRVSN K**K**GM**GHH**GNG	**K**AY**GHH**L**S**AE(2) GLGVGHK SYSGYWHSWI FGA**K**SH**GHH**R	KSNKCF M**K**QS**GHH**R**S**E WTRRPYDELIV	
C	IgG	KENGR**SPTH**S(10) **GR**S**NKSG**	**SPTH**P(5)	**GR**R**NKSG**	
D	IgG	V**PWSK**PWWTQ GHNNHNRHHP^a^ **SNV**R**SF**DNPI ANT**PWSK**TL (2) IPLPPPSRPF ^b^ (2) HNTRNWTLPP(1)	**SNVISY**PDV GN**PWSK**QINI VNTTSYNMRP(2) L**PWSK**LSSPS SNVKNYMAIP QLHPHNLHSP	TLHTTHSPFK^a^ NYEPVPRGAR^d^ TDAA**PWSK**VT(2) GKSLHGSHHP **SNVISF**RHAS **TNVISY**TPLY(3)^a^	^a^NP_078966.2 **Mucin 16 (2)** ^b^NP_002025.2 **Fucosyltransferase 5** ^d^NP_002024.1 **Fucosyltransferase 4**
D	IgM	QSLDHSSC^a^ (5) LNPQSPRD(4) YSWRAT(4) NERSEAR^a^ HFHHLAVRGR PQGWLGW GTVEPD PTRWGARLVK	GGRWNR(2) PETTDK(2) PGHVRGTLGR^a^(2) YVDTLSKL RGQSLA^c^ AVRRPD (4) QRLAAGFH QLAETLF	GRKTELF^b^ YLASPFE^c^ FRVARAA RTGRMWR LVYPQQ VVGLVP^c^(2) QIQLSGG^c^ VKNRGR	^a^NP_001122080.1 **mitogen-activated protein kinase binding protein 1 (3)** ^b^NP_078966.2 **Mucin 16 (2)** ^c^NP_001156729.1 **plexin B3 (4)**
Normal control	IgG	DIRLSAQL SWSGYYTY TNGVHHGR GLYNFMGK	RRTDYLLNG DPTVSESS^a^ APQGYLFK ESSTKSE	NQHLILSVG SIAAAVH RANKEPAT	^a^NP_078966.2 **Mucin 16**

All database-retrieved proteins presented in the table are of Homo sapiens origin.

1 Numbers in parentheses after the peptide sequences indicate the number of identical copies of the same phage identified after selection and sequencing.

2 Numbers in parenthses after the protein names indicate the number of times the protein was retrieved by BLAST homology search on different peptide sequences.

3 Highlighted in bold are conserved motifs present in different phage.

4 Superscript letters relate peptide sequences with the proteins retrieved by BLAST. Accession numbers provided correspond to the NCBI entry references.

### Antibodies against the selected peptides can be detected in cancer patient sera by ELISA

**Fig. 2:**
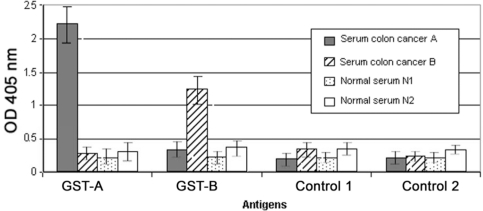
ELISA assay for binding of serum IgG antibodies to peptides isolated from RPPD libraries. Purified GSTpeptide fusion antigens were used to coat ELISA microplates. After blocking with 3% BSA in PBS the antigens were incubated overnight with cancer patient and normal individual sera diluted 1:200 with 3% BSA in PBS. The binding was detected using goat anti-human IgG antibodies conjugated with alkaline phosphatase and p-NPP as a substrate. GST-A and GST-B are TGVRGQRISQ and MKQSGHHRSE peptides expressed as GST-fusion proteins. As control peptides we used arbitrarily chosen peptides isolated from phage libraries and expressed as GST-fusion proteins. The average of three measurements is shown.

The multiple cycles of target binding and amplification of the bound phage in bacteria make RPPDL biopanning a sensitive assay capable of detecting very low-affinity interactions, which can be otherwise undetectable by standard immunoassay tests, such as an enzyme-linked immunosorbent assay (ELISA). To evaluate the suitability of the peptides selected from RPPDL screening for developing a simple personalized immunoassay test, we cloned the cDNAs encoding two peptides, TGVRGQRISQ (patient A) and MKQSGHHRSE (patient B), in a glutathione S-transferase (GST) fusion vector. We purified the proteins expressed in bacteria in order to screen the cancer patients' and healthy donors' sera by ELISA. Patient A's peptide was present in nine of the 18 phage particles isolated after selection on his own IgG. Likewise, patient B's peptide was selected on IgG antibodies. This peptide had a KXXGHH motif, which was found in five of the 14 phage particles isolated. Two randomly chosen peptides from unselected libraries were also expressed as GST fusions to be used as controls for non-specific binding. As seen in Figure 2, the GST-fused peptides isolated from phage libraries by affinity selection on cancer sera bind IgG antibodies from their corresponding cancer sera in the ELISA.

### Protein database searching using BLAST can identify proteins represented by selected peptides

The phage-displayed peptides bound by antibodies in the sera of cancer patients can represent sequential or conformational epitopes of protein antigens as well as peptide mimics of carbohydrate antigens. Assuming that among 70 peptide sequences identified by RPPDL biopanning on serum antibodies there were those representing sequential epitopes, we performed BLAST searching of the human reference protein database (refseq_ protein) for proteins containing sequence fragments identical or nearly identical to the sequences of the identified peptides. Since the lengths of serum antibody-recognized motifs were usually between four and six amino acids, a large number of proteins can produce the exact match to peptide sequences of such a short length solely by chance. Indeed, for each peptide sequence, BLAST homology searches using parameters adjusted to a short input sequence usually retrieved an average of 120 proteins. Supplementary tables S1-S7 show the lists of proteins detected by BLAST searching for each peptide. Some proteins were overrepresented. Generally, the most overrepresented proteins had the largest size. For example, mucin 16 (also known as CA125 ovarian cancer antigen) and nesprin-1 (14,507 and 8,749 amino acids, respectively) produced matches to 7 and 5 peptides, respectively. Multiple matches of phage insert sequences to such large proteins can be explained by recognition by antibodies with different specificities and/or by a higher probability of matches produced by pure chance. To estimate the probability of a peptide-protein match being produced by chance, we performed a BLAST search for 70 cancer sera-specific phage insert sequences in reverse orientation (i.e. “spelt backwards”) and calculated the length-normalized match frequency for the most overrepresented proteins, which produced four or more matches to different peptides. Given that the probability of a match being produced by chance is directly proportional to the length of the protein, we estimated that the frequency of a match by chance to a protein of 1,000 amino acids is f = 0.02 ± 0.01. This estimate indicates that the seven and five matches to mucin and nesprin-1, respectively, detected among 70 phage inserts analyzed were due to chance rather than to recognition by antibodies with different specificities. However, based on this estimate, the probability of the 403-amino acid bifunctional arginine demethylase and lysyl-hydroxylase 6 (JMJD6; accession number NP_055982.2) to produce matches by chance to two out of three peptides corresponding to IgG antibodies of patient A is p < 0.001. Since no peptide sequence corresponding to antibodies from other cancer patients or from a healthy control produced any matches to the JMJD6 protein, the p-value calculated by Fisher's exact test is p = 0.0009, suggesting that the two peptides mimic real sequential epitopes of the protein.

The 813-amino acid protein glycogen phosphorylase (liver form isoform 2; accession number NP_001157412.1) produced matches to five different peptides obtained on the IgG antibodies of patient D. However, all the peptides contained the common PWSK motif, which produced the match to the protein, thus negating the significance of the fact that the matches were multiple. The protein plexin B3 (accession number NP_001156729.1) produced matches to four out of 24 peptides corresponding to the IgM antibodies of patient D. All peptide sequences were different, without any common motifs. No other peptide corresponding to other cancer patients or healthy donor antibodies produced a match to this protein, thus producing a p-value of 0.006 by Fisher's exact test.

**Fig. 3:**
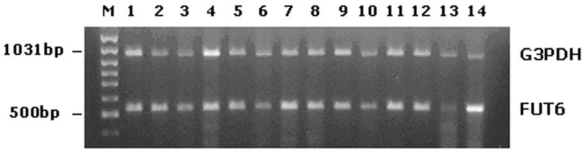
Simultaneous RT-PCR amplification of FUT6 and G3PDH gene fragments from normal colon tissue and colon tumor tissue mRNA. PCR products were electrophoresed on a 1% agarose gel. Numbers 1, 3, 5, 7, 9 and 11 are RT-PCR products from normal colon samples mRNA. Numbers 2, 4, 6, 8, 10 and 12 are RT-PCR products from corresponding colon tumor samples. Number 13 is the PCR product obtained from a cDNA of normal colon obtained from Clontech. Number 14 is the RT-PCR product obtained from the tumor of colon cancer patient A.

The FUT6 and FUT4 proteins (359 and 530 amino acids, respectively) of the fucosyltransferase protein family produced matches to two peptides corresponding to IgG antibodies of patient D. FUT6 also produced a match to a peptide obtained on IgM antibodies of patient A. FUT3 and FUT5 are highly homologous to FUT6 and, therefore, also produced the identical match to the peptide. An elevated level of serum fucosyltransferase activity involved in the synthesis of Lewis histo-blood group system antigens is known to be associated with colorectal and other cancers [11-13]. Since the total tumor RNA was available for patient A, we analyzed the FUT6 mRNA level in patient A's tumor and in other colon tumor and normal tissue samples. An RT-PCR analysis showed that FUT6 mRNA was over-expressed only in patent's A tumor but not in any other of the 13 colon tumor and normal tissues (Figure 3).

## DISCUSSION

We have demonstrated that patient-specific peptides recognized by serum IgG or IgM antibodies can be identified using RPPD libraries. For some of the peptides representing sequential epitopes, the corresponding proteins can be identified using BLAST homology searches and statistical analysis. Multiple matches to different peptide sequences corresponding to antibodies from the same cancer patient strongly indicate the presence of an immune response against the protein retrieved by BLAST.

One problem in using serum antibodies and RPPDL biopanning for developing personalized immunoassay tests is to distinguish the peptides associated with tumor growth from those representing the patient's immune response to other agents unrelated to cancer. For the peptides mimicking the sequential epitopes the proteins that likely induced a tumor-associated antibody response can be identified by performing gene expression analysis, if the RNA from the patient's tumor is available. The proteins overrepresented in the BLAST homology search, whose gene expression is up-regulated at the mRNA level in the tumor, are likely to represent personalized tumor-associated antigens produced by protein overexpression. By combining two high-throughput technologies, combinatorial peptide library biopanning on serum IgG or IgM antibodies and microarray-based gene expression analysis, the peptides representing sequential epitopes of the subset of tumor-associated antigens for a cancer patient can be easily and quickly identified. We demonstrated that the RPPDL biopanning-derived peptides can be used in a simple ELISA test for detecting serum antibody reactivities.

Due to the short lifespan of the IgM-producing plasma cells, the peptides selected by biopanning on serum IgM antibodies should be advantageous for designing an immunoassay test for detecting recurrences or metastases. A follow-up study using cancer patients' serum samples taken at different time-points is required to test whether the titer of IgM antibodies against RPPDL biopanning-selected peptides vanishes after surgical removal of the primary tumor and reappears upon tumor recurrence.

Only four out of seven serum samples were positive for the enrichment of RPPDL with cancer patient's antibody specific phage. The set of selected peptides was unique for each cancer patient. It is possible that the method of selection for antibodybinding peptides, which included an initial step of preadsorption of the phage libraries on normal immunoglobulins, prevented the identification of epitopes which are differentially recognized but shared between healthy donors and cancer patients' antibodies. Antibody reactivities against such epitopes can be common for many cancer patients and might be used for developing general immunoassay tests for cancer detection. With the advance of next generation sequencing technology, it is now possible to sequence all the phage that bind to cancer patients and to normal controls' antibodies in order to identify all possible differentially recognized peptides that can be used for designing personalized or general immunoassay tests for detecting recurrences or metastases.

## MATERIALS AND METHODS

### Cancer patient sera

The study was approved by the Institutional Review Board. All patients provided written informed consent, as required by the Institutional Review Board and the Food and Drug Administration and in line with the Helsinki Declaration. Sera from CRC patients were procured by the Midwestern and Eastern divisions of the Cooperative Human Tissue Network (CHTN) of the NCI. All sera were drawn from patients at the time of surgery

### Random peptide phage display libraries

The mixture of 5-mer, 6-mer, 7-mer, 8-mer, 9- mer, and 10-mer phage display peptide libraries were kindly provided by Drs. Renata Pasqualini and Wadih Arap. The libraries were constructed as described [8,10].

### Isolation of phage binding antibodies from colon cancer patient sera

We used a mixture of several libraries for the affinity selection of IgG and IgM immunoglobulins from cancer patient sera. In order to select peptides specific to antibodies from each serum, we developed a four-round procedure of phage panning. Each round of the protocol consisted of an initial step of preadsorption of the phage libraries on normal immunoglobulins, followed by affinity selection of the antibodies present in the patient serum and amplification of the phage particles eluted from the antibodies in bacteria, as described [10]. IgG or IgM antibodies from a CRC patient were immobilized on protein G (Gibco BRL) or anti-human IgM (Sigma) agarose, respectively. The immobilized antibodies were then washed in PBS and pre-blocked with 3% bovine serum albumin (BSA) in PBS for 30 min. 50 îl of colon cancer patient serum was incubated with 50 îl of BSA-pre-blocked protein G agarose beads for 30 min. In the control experiment, 50 îl of serum from a healthy individual was used. Likewise, 200 îl of pooled human sera from 24 healthy individuals were incubated with 200 îl of BSA-pre-blocked protein G agarose beads for 30 min, which were used to preadsorb the phage libraries on normal IgG. After incubation, the beads were washed in PBS four times. We resuspended 10[11] colony forming units (cfu) of phage in 200 îl of 3% BSA/0.05% Tween 20/PBS solution. The suspensions were incubated with immobilized IgG antibodies from the pool of healthy donor sera for 2h at 4° C. The phages unbound to normal IgG antibodies were split into equal volumes. One volume was transferred to protein G agarose beads with immobilized IgG antibodies from the serum of a CRC patient, and the other volume was transferred to the same amount of protein G agarose beads with immobilized IgG antibodies from control healthy individuals. These phages were incubated for 2h at 4° C. The unbound phage was removed by 10 washes with 3% BSA/0.05% Tween 20/PBS buffer solution. The bound phages were eluted with 100 îl of 0.1M glycine buffer, pH 2.2, containing 1 mg/ml BSA and 0.1 mg/ml of phenol red indicator. The eluted phage solution was neutralized by the addition of 1/10 volume of 1M Tris buffer, pH 9. The neutralized phage was incubated with 1 ml of starved, competent E. coli K91kan (OD600=1-2) for 20 min at room temperature without shaking, followed by the addition of 10 ml of LB medium containing 0.2 îg/ml of tetracycline. After incubation at room temperature for 20min, 10 to 100-îl aliquots from each sample were spread on agar plates containing tetracycline at 40 îg/ml. The colonies were counted after 12 h. The remaining infected bacteria were grown overnight at 37° C in the presence of 40 îg/ml tetracycline. The amplified phage were purified from the bacterial supernatant, quantified and subjected to the next round of selection. The amount of IgG or IgM immunoglobulins from healthy individuals used for preadsorption in each round of selection was fourfold higher than that from cancer patients. The binding results were quantified as the number of cfu produced by bacteria infected with the phage bound to normal and cancer patient sera antibodies. The biopanning procedure was considered successful when the number of cfu obtained after the fourth round of selection was at least twofold higher than those binding to control antibodies from healthy individuals.

### Sequencing of phage DNA inserts

The colonies derived from the patient antibodies were grown overnight and the phage was purified by double precipitation with polyethylene-glycol. Phage DNAs were purified using Strataclean Resin (Stratagene) and sequenced with the primer 5′-CCCTCATAGTTAAGCGTAACG-3′. Sequencing was performed using the D-Rhodamine ABI prism automatic sequencing system (Perkin Elmer), as described in the manufacturer's protocol.

### Expression of Peptides

PCR amplifications of the peptide-encoding DNA inserts were performed with phage ssDNA and the primers 5′-AGGCTCGAGGATCCTCGGCCGACGGGGCT-3′ (sense) and 5′-AGGTCTAGAATTCGCCCCAGCGGCCCC-3′ (antisense). The template was purified with phage with Strataclean resin (Stratagene). The PCR conditions included 35 cycles consisting of 1 minute at 95° C for denaturation, 1 minute at 53° C for annealing, and 1 minute at 72° C for extension. The PCR products were ethanol-precipitated in the presence of glycogen (1 îl of a 20 mg/ml stock solution; Boehringer-Mannheim) and then digested with BamHI and EcoRI. The fragments were then ligated into the same restriction sites of the pGEX2TK vector (Promega). Bl-21 E. Coli cells were transformed with the constructs, and the fusion proteins were purified using gluthatione coupled beads following the manufacturer's protocol (Sigma).

### ELISA

10 îg/ml solution of purified GST fusion proteins in 0.1M NaHCO3 was used to coat multi-well plates (50 îl per well). After washing with 0.05% Tween 20/PBS (washing buffer) and blocking with 3% BSA/0.05% Tween 20/PBS (blocking buffer), the plates were incubated overnight at 4° C with cancer patients' and healthy individuals' sera diluted 1/200 in blocking buffer. The plates were then washed and incubated for 2h at 4° C with anti-human alkaline phosphatase-conjugated antibodies (GIBCO) diluted in blocking buffer. The plates were then washed and developed using p-NPP (SIGMA) as a substrate.

### Protein database search

Homology searches with the peptide sequences isolated through library panning on IgG and IgM antibodies were performed using the BLAST module available on-line from NCBI (URL: http://blast.ncbi.nlm.nih.gov/Blast.cgi). The searches were performed against the refseq_protein database. The parameters of the BLAST module were adjusted for an optimal short motif homology search, and the searches were limited to human sequences.

### RT-PCR

A multiplex RT-PCR assay was performed using a Clontech RT-PCR kit. 2 îg of DNAase-treated RNA from normal and colon tumor tissues were used with random hexamers to synthesize the first strand. Control G3PDH and FUT6 gene fragments were amplified in a single PCR reaction. The primers used for amplification of the FUT6 cDNA fragment were: 5′- ATGGATCCCCTGGGCCCGGCCA-3′ (sense) and 5′-CCGTAGGGCGTGAAGATGT-3′ (antisense). The primers used for amplification of a 983-bp fragment of the control G3PDH gene were supplied with the RT-PCR kit. PCR conditions were 35 rounds each consisting of 1 min at 95° C, 1 min at 58° C, and 4 min at 72° C; the PCR reactions were concluded with a final extention step of 10 min at 72° C. PCR products were visualized by electrophoresis on a 1% agarose gel.

## SUPPLEMENTAL TABLES

Supplemental Table 1

Supplemental Table 2

Supplemental Table 3

Supplemental Table 4

Supplemental Table 5

Supplemental Table 6

Supplemental Table 7

## Abbreviations used

BSA: bovine serum albumin
CRC: colorectal cancer
ELISA: enzyme-linked immunosorbent assay
GST: glutathione S-transferase
pNPP: pnitrophenyl phosphate
RPPDL: random peptide phage displayed library
RT-PCR: reverse transcription-polymerase chain reaction
TAA: tumor-associated antigen
