# Supplementary material for: A high throughput method for identifying personalized tumor-associated antigens
Source: Oncotarget. 2010 Jun 27;1(2):148–55. doi: 10.18632/oncotarget.118 (PMC2920534; doi:10.18632/oncotarget.118)
Supplement: Supplemental Table 1 [file oncotarget-01-148-s001.doc]

**IgG Patient A**

| **Accession** | **Proteins with a match to TGVRGQRISQ peptide** | **[Max score](http://blast.ncbi.nlm.nih.gov/Blast.cgi?CMD=Get&ALIGNMENTS=100&ALIGNMENT_VIEW=Pairwise&CDD_SEARCH_STATE=1&DATABASE_SORT=0&DESCRIPTIONS=100&ENTREZ_QUERY=txid9606 %5BORGN%5D&FIRST_QUERY_NUM=0&FORMAT_OBJECT=Alignment&FORMAT_PAGE_TARGET=&FORMAT_TYPE=HTML&GET_SEQUENCE=yes&I_THRESH=&MASK_CHAR=2&MASK_COLOR=1&NEW_DESIGN=on&NEW_VIEW=yes&NUM_OVERVIEW=100&OLD_BLAST=false&PAGE=Proteins&QUERY_INDEX=0&QUERY_NUMBER=0&RESULTS_PAGE_TARGET=&RID=SZWVP0JX01N&SHOW_LINKOUT=yes&SHOW_OVERVIEW=yes&STEP_NUMBER=&WORD_SIZE=2&DISPLAY_SORT=1&HSP_SORT=1" \l "sort_mark)** | **[Total score](http://blast.ncbi.nlm.nih.gov/Blast.cgi?CMD=Get&ALIGNMENTS=100&ALIGNMENT_VIEW=Pairwise&CDD_SEARCH_STATE=1&DATABASE_SORT=0&DESCRIPTIONS=100&ENTREZ_QUERY=txid9606 %5BORGN%5D&FIRST_QUERY_NUM=0&FORMAT_OBJECT=Alignment&FORMAT_PAGE_TARGET=&FORMAT_TYPE=HTML&GET_SEQUENCE=yes&I_THRESH=&MASK_CHAR=2&MASK_COLOR=1&NEW_DESIGN=on&NEW_VIEW=yes&NUM_OVERVIEW=100&OLD_BLAST=false&PAGE=Proteins&QUERY_INDEX=0&QUERY_NUMBER=0&RESULTS_PAGE_TARGET=&RID=SZWVP0JX01N&SHOW_LINKOUT=yes&SHOW_OVERVIEW=yes&STEP_NUMBER=&WORD_SIZE=2&DISPLAY_SORT=2&HSP_SORT=1" \l "sort_mark)** | **[Query coverage](http://blast.ncbi.nlm.nih.gov/Blast.cgi?CMD=Get&ALIGNMENTS=100&ALIGNMENT_VIEW=Pairwise&CDD_SEARCH_STATE=1&DATABASE_SORT=0&DESCRIPTIONS=100&ENTREZ_QUERY=txid9606 %5BORGN%5D&FIRST_QUERY_NUM=0&FORMAT_OBJECT=Alignment&FORMAT_PAGE_TARGET=&FORMAT_TYPE=HTML&GET_SEQUENCE=yes&I_THRESH=&MASK_CHAR=2&MASK_COLOR=1&NEW_DESIGN=on&NEW_VIEW=yes&NUM_OVERVIEW=100&OLD_BLAST=false&PAGE=Proteins&QUERY_INDEX=0&QUERY_NUMBER=0&RESULTS_PAGE_TARGET=&RID=SZWVP0JX01N&SHOW_LINKOUT=yes&SHOW_OVERVIEW=yes&STEP_NUMBER=&WORD_SIZE=2&DISPLAY_SORT=4&HSP_SORT=0" \l "sort_mark)** | **[E value](http://blast.ncbi.nlm.nih.gov/Blast.cgi?CMD=Get&ALIGNMENTS=100&ALIGNMENT_VIEW=Pairwise&CDD_SEARCH_STATE=1&DATABASE_SORT=0&DESCRIPTIONS=100&ENTREZ_QUERY=txid9606 %5BORGN%5D&FIRST_QUERY_NUM=0&FORMAT_OBJECT=Alignment&FORMAT_PAGE_TARGET=&FORMAT_TYPE=HTML&GET_SEQUENCE=yes&I_THRESH=&MASK_CHAR=2&MASK_COLOR=1&NEW_DESIGN=on&NEW_VIEW=yes&NUM_OVERVIEW=100&OLD_BLAST=false&PAGE=Proteins&QUERY_INDEX=0&QUERY_NUMBER=0&RESULTS_PAGE_TARGET=&RID=SZWVP0JX01N&SHOW_LINKOUT=yes&SHOW_OVERVIEW=yes&STEP_NUMBER=&WORD_SIZE=2&DISPLAY_SORT=0&HSP_SORT=0" \l "sort_mark)** |
| --- | --- | --- | --- | --- | --- |
| [NP_056199.2](http://www.ncbi.nlm.nih.gov/entrez/query.fcgi?cmd=Retrieve&db=Protein&list_uids=47578107&dopt=GenPept&RID=SZWVP0JX01N&log$=prottop&blast_rank=1) | delangin isoform B [Homo sapiens] | [22.3](http://blast.ncbi.nlm.nih.gov/Blast.cgi#47578107%2347578107) | 22.3 | 90% | 34 |
| [NP_001138462.1](http://www.ncbi.nlm.nih.gov/entrez/query.fcgi?cmd=Retrieve&db=Protein&list_uids=222418587&dopt=GenPept&RID=SZWVP0JX01N&log$=prottop&blast_rank=2) | hypothetical protein LOC57495 [Homo sapiens] | [21.8](http://blast.ncbi.nlm.nih.gov/Blast.cgi#222418587%23222418587) | 21.8 | 60% | 46 |
| [NP_065805.2](http://www.ncbi.nlm.nih.gov/entrez/query.fcgi?cmd=Retrieve&db=Protein&list_uids=186928844&dopt=GenPept&RID=SZWVP0JX01N&log$=prottop&blast_rank=3) | Cdc42 GTPase-activating protein [Homo sapiens] | [21.0](http://blast.ncbi.nlm.nih.gov/Blast.cgi#186928844%23186928844) | 21.0 | 70% | 83 |
| [NP_004913.2](http://www.ncbi.nlm.nih.gov/entrez/query.fcgi?cmd=Retrieve&db=Protein&list_uids=38373669&dopt=GenPept&RID=SZWVP0JX01N&log$=prottop&blast_rank=4) | SEC24-related protein C [Homo sapiens] >ref|NP_940999.1| SEC24-related protein C [Homo sapiens] | [21.0](http://blast.ncbi.nlm.nih.gov/Blast.cgi#38373669%2338373669) | 21.0 | 60% | 83 |
| [XP_002344653.1](http://www.ncbi.nlm.nih.gov/entrez/query.fcgi?cmd=Retrieve&db=Protein&list_uids=239755648&dopt=GenPept&RID=SZWVP0JX01N&log$=prottop&blast_rank=5) | PREDICTED: hypothetical protein [Homo sapiens] | [20.6](http://blast.ncbi.nlm.nih.gov/Blast.cgi#239755648%23239755648) | 20.6 | 70% | 111 |
| [NP_004571.2](http://www.ncbi.nlm.nih.gov/entrez/query.fcgi?cmd=Retrieve&db=Protein&list_uids=19923264&dopt=GenPept&RID=SZWVP0JX01N&log$=prottop&blast_rank=6) | Ras-related protein Rab-27A [Homo sapiens] >ref|NP_899057.1| Ras-related protein Rab-27A [Homo sapiens] >ref|NP_899058.1| Ras-related protein Rab-27A [Homo sapiens] >ref|NP_899059.1| Ras-related protein Rab-27A [Homo sapiens] | [20.6](http://blast.ncbi.nlm.nih.gov/Blast.cgi#19923264%2319923264) | 20.6 | 80% | 111 |
| [XP_002345510.1](http://www.ncbi.nlm.nih.gov/entrez/query.fcgi?cmd=Retrieve&db=Protein&list_uids=239757646&dopt=GenPept&RID=SZWVP0JX01N&log$=prottop&blast_rank=7) | PREDICTED: similar to phosphatidylinositol 4-kinase type 3 alpha [Homo sapiens] | [20.2](http://blast.ncbi.nlm.nih.gov/Blast.cgi#239757646%23239757646) | 35.2 | 70% | 149 |
| [NP_699193.2](http://www.ncbi.nlm.nih.gov/entrez/query.fcgi?cmd=Retrieve&db=Protein&list_uids=281427284&dopt=GenPept&RID=SZWVP0JX01N&log$=prottop&blast_rank=8) | inactive serine protease 35 precursor [Homo sapiens] >ref|NP_001163894.1| inactive serine protease 35 precursor [Homo sapiens] | [19.7](http://blast.ncbi.nlm.nih.gov/Blast.cgi#281427284%23281427284) | 19.7 | 70% | 200 |
| [NP_005924.2](http://www.ncbi.nlm.nih.gov/entrez/query.fcgi?cmd=Retrieve&db=Protein&list_uids=56550039&dopt=GenPept&RID=SZWVP0JX01N&log$=prottop&blast_rank=9) | myeloid/lymphoid or mixed-lineage leukemia protein [Homo sapiens] | [19.7](http://blast.ncbi.nlm.nih.gov/Blast.cgi#56550039%2356550039) | 35.6 | 80% | 200 |
| [NP_940888.2](http://www.ncbi.nlm.nih.gov/entrez/query.fcgi?cmd=Retrieve&db=Protein&list_uids=50053872&dopt=GenPept&RID=SZWVP0JX01N&log$=prottop&blast_rank=10) | ribosomal protein L7-like 1 [Homo sapiens] | [19.7](http://blast.ncbi.nlm.nih.gov/Blast.cgi#50053872%2350053872) | 31.8 | 80% | 200 |
| [NP_006606.1](http://www.ncbi.nlm.nih.gov/entrez/query.fcgi?cmd=Retrieve&db=Protein&list_uids=5729758&dopt=GenPept&RID=SZWVP0JX01N&log$=prottop&blast_rank=11) | calpain 9 isoform 1 [Homo sapiens] | [19.7](http://blast.ncbi.nlm.nih.gov/Blast.cgi#5729758%235729758) | 19.7 | 80% | 200 |
| [NP_057536.1](http://www.ncbi.nlm.nih.gov/entrez/query.fcgi?cmd=Retrieve&db=Protein&list_uids=7705383&dopt=GenPept&RID=SZWVP0JX01N&log$=prottop&blast_rank=12) | calpain 9 isoform 2 [Homo sapiens] | [19.7](http://blast.ncbi.nlm.nih.gov/Blast.cgi#7705383%237705383) | 19.7 | 80% | 200 |
| [NP_056261.4](http://www.ncbi.nlm.nih.gov/entrez/query.fcgi?cmd=Retrieve&db=Protein&list_uids=262359929&dopt=GenPept&RID=SZWVP0JX01N&log$=prottop&blast_rank=13) | transcription factor ELYS [Homo sapiens] | [19.3](http://blast.ncbi.nlm.nih.gov/Blast.cgi#262359929%23262359929) | 19.3 | 50% | 268 |
| [NP_114157.3](http://www.ncbi.nlm.nih.gov/entrez/query.fcgi?cmd=Retrieve&db=Protein&list_uids=221219020&dopt=GenPept&RID=SZWVP0JX01N&log$=prottop&blast_rank=14) | NYD-SP11 protein [Homo sapiens] | [19.3](http://blast.ncbi.nlm.nih.gov/Blast.cgi#221219020%23221219020) | 34.4 | 70% | 268 |
| [NP_001092909.1](http://www.ncbi.nlm.nih.gov/entrez/query.fcgi?cmd=Retrieve&db=Protein&list_uids=150456460&dopt=GenPept&RID=SZWVP0JX01N&log$=prottop&blast_rank=15) | EPH receptor A10 isofom 3 [Homo sapiens] | [19.3](http://blast.ncbi.nlm.nih.gov/Blast.cgi#150456460%23150456460) | 19.3 | 50% | 268 |
| [NP_001092093.1](http://www.ncbi.nlm.nih.gov/entrez/query.fcgi?cmd=Retrieve&db=Protein&list_uids=148833506&dopt=GenPept&RID=SZWVP0JX01N&log$=prottop&blast_rank=16) | obscurin, cytoskeletal calmodulin and titin-interacting RhoGEF isoform b [Homo sapiens] | [19.3](http://blast.ncbi.nlm.nih.gov/Blast.cgi#148833506%23148833506) | 44.3 | 70% | 268 |
| [NP_775912.2](http://www.ncbi.nlm.nih.gov/entrez/query.fcgi?cmd=Retrieve&db=Protein&list_uids=150456469&dopt=GenPept&RID=SZWVP0JX01N&log$=prottop&blast_rank=17) | EPH receptor A10 isoform 2 [Homo sapiens] | [19.3](http://blast.ncbi.nlm.nih.gov/Blast.cgi#150456469%23150456469) | 19.3 | 50% | 268 |
| [NP_001123617.1](http://www.ncbi.nlm.nih.gov/entrez/query.fcgi?cmd=Retrieve&db=Protein&list_uids=194306653&dopt=GenPept&RID=SZWVP0JX01N&log$=prottop&blast_rank=18) | Yes-associated protein 1, 65kDa isoform 1 [Homo sapiens] | [19.3](http://blast.ncbi.nlm.nih.gov/Blast.cgi#194306653%23194306653) | 29.3 | 60% | 268 |
| [NP_002582.3](http://www.ncbi.nlm.nih.gov/entrez/query.fcgi?cmd=Retrieve&db=Protein&list_uids=187281517&dopt=GenPept&RID=SZWVP0JX01N&log$=prottop&blast_rank=19) | cytosolic phosphoenolpyruvate carboxykinase 1 [Homo sapiens] | [19.3](http://blast.ncbi.nlm.nih.gov/Blast.cgi#187281517%23187281517) | 19.3 | 50% | 268 |
| [NP_071895.3](http://www.ncbi.nlm.nih.gov/entrez/query.fcgi?cmd=Retrieve&db=Protein&list_uids=190341097&dopt=GenPept&RID=SZWVP0JX01N&log$=prottop&blast_rank=20) | rhomboid family 1 [Homo sapiens] | [19.3](http://blast.ncbi.nlm.nih.gov/Blast.cgi#190341097%23190341097) | 19.3 | 50% | 268 |
| [NP_653168.2](http://www.ncbi.nlm.nih.gov/entrez/query.fcgi?cmd=Retrieve&db=Protein&list_uids=36030946&dopt=GenPept&RID=SZWVP0JX01N&log$=prottop&blast_rank=21) | angel homolog 2 [Homo sapiens] | [19.3](http://blast.ncbi.nlm.nih.gov/Blast.cgi#36030946%2336030946) | 19.3 | 50% | 268 |
| [NP_443075.2](http://www.ncbi.nlm.nih.gov/entrez/query.fcgi?cmd=Retrieve&db=Protein&list_uids=58331253&dopt=GenPept&RID=SZWVP0JX01N&log$=prottop&blast_rank=22) | obscurin, cytoskeletal calmodulin and titin-interacting RhoGEF isoform a [Homo sapiens] | [19.3](http://blast.ncbi.nlm.nih.gov/Blast.cgi#58331253%2358331253) | 60.7 | 70% | 268 |
| [NP_705841.2](http://www.ncbi.nlm.nih.gov/entrez/query.fcgi?cmd=Retrieve&db=Protein&list_uids=87116681&dopt=GenPept&RID=SZWVP0JX01N&log$=prottop&blast_rank=23) | lysophosphatidylcholine acyltransferase 4 [Homo sapiens] | [19.3](http://blast.ncbi.nlm.nih.gov/Blast.cgi#87116681%2387116681) | 19.3 | 70% | 268 |
| [NP_055048.1](http://www.ncbi.nlm.nih.gov/entrez/query.fcgi?cmd=Retrieve&db=Protein&list_uids=7657671&dopt=GenPept&RID=SZWVP0JX01N&log$=prottop&blast_rank=24) | upstream binding transcription factor, RNA polymerase I isoform a [Homo sapiens] | [19.3](http://blast.ncbi.nlm.nih.gov/Blast.cgi#7657671%237657671) | 19.3 | 50% | 268 |
| [NP_803173.1](http://www.ncbi.nlm.nih.gov/entrez/query.fcgi?cmd=Retrieve&db=Protein&list_uids=28933465&dopt=GenPept&RID=SZWVP0JX01N&log$=prottop&blast_rank=25) | syntaxin 12 [Homo sapiens] | [19.3](http://blast.ncbi.nlm.nih.gov/Blast.cgi#28933465%2328933465) | 19.3 | 50% | 268 |
| [NP_037437.2](http://www.ncbi.nlm.nih.gov/entrez/query.fcgi?cmd=Retrieve&db=Protein&list_uids=28373101&dopt=GenPept&RID=SZWVP0JX01N&log$=prottop&blast_rank=26) | ST8 alpha-N-acetyl-neuraminide alpha-2,8-sialyltransferase 5 [Homo sapiens] | [19.3](http://blast.ncbi.nlm.nih.gov/Blast.cgi#28373101%2328373101) | 19.3 | 80% | 268 |
| [NP_001070152.1](http://www.ncbi.nlm.nih.gov/entrez/query.fcgi?cmd=Retrieve&db=Protein&list_uids=115529449&dopt=GenPept&RID=SZWVP0JX01N&log$=prottop&blast_rank=27) | upstream binding transcription factor, RNA polymerase I isoform b [Homo sapiens] >ref|NP_001070151.1| upstream binding transcription factor, RNA polymerase I isoform b [Homo sapiens] | [19.3](http://blast.ncbi.nlm.nih.gov/Blast.cgi#115529449%23115529449) | 19.3 | 50% | 268 |
| [NP_006452.3](http://www.ncbi.nlm.nih.gov/entrez/query.fcgi?cmd=Retrieve&db=Protein&list_uids=73623035&dopt=GenPept&RID=SZWVP0JX01N&log$=prottop&blast_rank=28) | sperm associated antigen 5 [Homo sapiens] | [19.3](http://blast.ncbi.nlm.nih.gov/Blast.cgi#73623035%2373623035) | 19.3 | 50% | 268 |
| [NP_003489.1](http://www.ncbi.nlm.nih.gov/entrez/query.fcgi?cmd=Retrieve&db=Protein&list_uids=4507117&dopt=GenPept&RID=SZWVP0JX01N&log$=prottop&blast_rank=29) | Stannin [Homo sapiens] | [19.3](http://blast.ncbi.nlm.nih.gov/Blast.cgi#4507117%234507117) | 19.3 | 50% | 268 |
| [NP_858059.1](http://www.ncbi.nlm.nih.gov/entrez/query.fcgi?cmd=Retrieve&db=Protein&list_uids=32307150&dopt=GenPept&RID=SZWVP0JX01N&log$=prottop&blast_rank=30) | O-linked GlcNAc transferase isoform 2 [Homo sapiens] | [19.3](http://blast.ncbi.nlm.nih.gov/Blast.cgi#32307150%2332307150) | 19.3 | 70% | 268 |
| [NP_005041.1](http://www.ncbi.nlm.nih.gov/entrez/query.fcgi?cmd=Retrieve&db=Protein&list_uids=4826958&dopt=GenPept&RID=SZWVP0JX01N&log$=prottop&blast_rank=31) | ATP-binding cassette, sub-family D, member 4 [Homo sapiens] | [19.3](http://blast.ncbi.nlm.nih.gov/Blast.cgi#4826958%234826958) | 19.3 | 50% | 268 |
| [NP_858058.1](http://www.ncbi.nlm.nih.gov/entrez/query.fcgi?cmd=Retrieve&db=Protein&list_uids=32307148&dopt=GenPept&RID=SZWVP0JX01N&log$=prottop&blast_rank=32) | O-linked GlcNAc transferase isoform 1 [Homo sapiens] | [19.3](http://blast.ncbi.nlm.nih.gov/Blast.cgi#32307148%2332307148) | 19.3 | 70% | 268 |
| [NP_006097.1](http://www.ncbi.nlm.nih.gov/entrez/query.fcgi?cmd=Retrieve&db=Protein&list_uids=5174751&dopt=GenPept&RID=SZWVP0JX01N&log$=prottop&blast_rank=33) | Yes-associated protein 1, 65kDa isoform 2 [Homo sapiens] | [19.3](http://blast.ncbi.nlm.nih.gov/Blast.cgi#5174751%235174751) | 29.3 | 60% | 268 |
| [NP_057324.2](http://www.ncbi.nlm.nih.gov/entrez/query.fcgi?cmd=Retrieve&db=Protein&list_uids=33598924&dopt=GenPept&RID=SZWVP0JX01N&log$=prottop&blast_rank=34) | scavenger receptor class A, member 3 isoform 1 [Homo sapiens] | [19.3](http://blast.ncbi.nlm.nih.gov/Blast.cgi#33598924%2333598924) | 33.1 | 100% | 268 |
| [NP_878185.1](http://www.ncbi.nlm.nih.gov/entrez/query.fcgi?cmd=Retrieve&db=Protein&list_uids=33598922&dopt=GenPept&RID=SZWVP0JX01N&log$=prottop&blast_rank=35) | scavenger receptor class A, member 3 isoform 2 [Homo sapiens] | [19.3](http://blast.ncbi.nlm.nih.gov/Blast.cgi#33598922%2333598922) | 19.3 | 50% | 268 |
| [XP_002342650.1](http://www.ncbi.nlm.nih.gov/entrez/query.fcgi?cmd=Retrieve&db=Protein&list_uids=239742782&dopt=GenPept&RID=SZWVP0JX01N&log$=prottop&blast_rank=36) | PREDICTED: hypothetical protein XP_002342650 [Homo sapiens] >ref|XP_002346785.1| PREDICTED: hypothetical protein XP_002346785 [Homo sapiens] >ref|XP_002345914.1| PREDICTED: similar to leucocyte elastase inhibitor [Homo sapiens] | [18.9](http://blast.ncbi.nlm.nih.gov/Blast.cgi#239742782%23239742782) | 18.9 | 50% | 359 |
| [NP_116256.2](http://www.ncbi.nlm.nih.gov/entrez/query.fcgi?cmd=Retrieve&db=Protein&list_uids=118918413&dopt=GenPept&RID=SZWVP0JX01N&log$=prottop&blast_rank=37) | MICAL C-terminal like [Homo sapiens] | [18.9](http://blast.ncbi.nlm.nih.gov/Blast.cgi#118918413%23118918413) | 18.9 | 60% | 359 |
| [NP_542410.2](http://www.ncbi.nlm.nih.gov/entrez/query.fcgi?cmd=Retrieve&db=Protein&list_uids=111118968&dopt=GenPept&RID=SZWVP0JX01N&log$=prottop&blast_rank=38) | collagen, type XI, alpha 2 isoform 3 preproprotein [Homo sapiens] | [18.9](http://blast.ncbi.nlm.nih.gov/Blast.cgi#111118968%23111118968) | 33.5 | 70% | 359 |
| [NP_001035090.1](http://www.ncbi.nlm.nih.gov/entrez/query.fcgi?cmd=Retrieve&db=Protein&list_uids=90819237&dopt=GenPept&RID=SZWVP0JX01N&log$=prottop&blast_rank=39) | myeloid/lymphoid or mixed-lineage leukemia (trithorax homolog, Drosophila); translocated to, 4 isoform 1 [Homo sapiens] | [18.9](http://blast.ncbi.nlm.nih.gov/Blast.cgi#90819237%2390819237) | 34.8 | 60% | 359 |
| [NP_001035089.1](http://www.ncbi.nlm.nih.gov/entrez/query.fcgi?cmd=Retrieve&db=Protein&list_uids=90819233&dopt=GenPept&RID=SZWVP0JX01N&log$=prottop&blast_rank=40) | myeloid/lymphoid or mixed-lineage leukemia (trithorax homolog, Drosophila); translocated to, 4 isoform 2 [Homo sapiens] | [18.9](http://blast.ncbi.nlm.nih.gov/Blast.cgi#90819233%2390819233) | 34.8 | 60% | 359 |
| [NP_542412.2](http://www.ncbi.nlm.nih.gov/entrez/query.fcgi?cmd=Retrieve&db=Protein&list_uids=111118972&dopt=GenPept&RID=SZWVP0JX01N&log$=prottop&blast_rank=41) | collagen, type XI, alpha 2 isoform 2 preproprotein [Homo sapiens] | [18.9](http://blast.ncbi.nlm.nih.gov/Blast.cgi#111118972%23111118972) | 33.5 | 70% | 359 |
| [NP_001014449.1](http://www.ncbi.nlm.nih.gov/entrez/query.fcgi?cmd=Retrieve&db=Protein&list_uids=62241022&dopt=GenPept&RID=SZWVP0JX01N&log$=prottop&blast_rank=42) | DEAD (Asp-Glu-Ala-As) box polypeptide 19 isoform 3 [Homo sapiens] | [18.9](http://blast.ncbi.nlm.nih.gov/Blast.cgi#62241022%2362241022) | 18.9 | 70% | 359 |
| [NP_542411.2](http://www.ncbi.nlm.nih.gov/entrez/query.fcgi?cmd=Retrieve&db=Protein&list_uids=111118970&dopt=GenPept&RID=SZWVP0JX01N&log$=prottop&blast_rank=43) | collagen, type XI, alpha 2 isoform 1 preproprotein [Homo sapiens] | [18.9](http://blast.ncbi.nlm.nih.gov/Blast.cgi#111118970%23111118970) | 33.5 | 70% | 359 |
| [NP_003446.2](http://www.ncbi.nlm.nih.gov/entrez/query.fcgi?cmd=Retrieve&db=Protein&list_uids=56699475&dopt=GenPept&RID=SZWVP0JX01N&log$=prottop&blast_rank=44) | zinc finger protein 202 [Homo sapiens] | [18.9](http://blast.ncbi.nlm.nih.gov/Blast.cgi#56699475%2356699475) | 43.9 | 50% | 359 |
| [NP_005927.2](http://www.ncbi.nlm.nih.gov/entrez/query.fcgi?cmd=Retrieve&db=Protein&list_uids=90819231&dopt=GenPept&RID=SZWVP0JX01N&log$=prottop&blast_rank=45) | myeloid/lymphoid or mixed-lineage leukemia (trithorax homolog, Drosophila); translocated to, 4 isoform 3 [Homo sapiens] | [18.9](http://blast.ncbi.nlm.nih.gov/Blast.cgi#90819231%2390819231) | 34.8 | 60% | 359 |
| [NP_001014451.1](http://www.ncbi.nlm.nih.gov/entrez/query.fcgi?cmd=Retrieve&db=Protein&list_uids=62241024&dopt=GenPept&RID=SZWVP0JX01N&log$=prottop&blast_rank=46) | DEAD (Asp-Glu-Ala-As) box polypeptide 19 isoform 2 [Homo sapiens] | [18.9](http://blast.ncbi.nlm.nih.gov/Blast.cgi#62241024%2362241024) | 18.9 | 70% | 359 |
| [NP_009198.4](http://www.ncbi.nlm.nih.gov/entrez/query.fcgi?cmd=Retrieve&db=Protein&list_uids=187608784&dopt=GenPept&RID=SZWVP0JX01N&log$=prottop&blast_rank=47) | transmembrane channel-like 6 [Homo sapiens] >ref|NP_001120670.1| transmembrane channel-like 6 [Homo sapiens] | [18.9](http://blast.ncbi.nlm.nih.gov/Blast.cgi#187608784%23187608784) | 33.5 | 80% | 359 |
| [NP_116244.1](http://www.ncbi.nlm.nih.gov/entrez/query.fcgi?cmd=Retrieve&db=Protein&list_uids=14249584&dopt=GenPept&RID=SZWVP0JX01N&log$=prottop&blast_rank=48) | hematopoietic SH2 domain containing [Homo sapiens] | [18.9](http://blast.ncbi.nlm.nih.gov/Blast.cgi#14249584%2314249584) | 18.9 | 70% | 359 |
| [NP_060378.1](http://www.ncbi.nlm.nih.gov/entrez/query.fcgi?cmd=Retrieve&db=Protein&list_uids=8923582&dopt=GenPept&RID=SZWVP0JX01N&log$=prottop&blast_rank=49) | zinc finger protein 446 [Homo sapiens] | [18.9](http://blast.ncbi.nlm.nih.gov/Blast.cgi#8923582%238923582) | 18.9 | 50% | 359 |
| [NP_060802.1](http://www.ncbi.nlm.nih.gov/entrez/query.fcgi?cmd=Retrieve&db=Protein&list_uids=8922886&dopt=GenPept&RID=SZWVP0JX01N&log$=prottop&blast_rank=50) | DDX19-like protein [Homo sapiens] | [18.9](http://blast.ncbi.nlm.nih.gov/Blast.cgi#8922886%238922886) | 18.9 | 70% | 359 |
| [NP_000848.1](http://www.ncbi.nlm.nih.gov/entrez/query.fcgi?cmd=Retrieve&db=Protein&list_uids=4504215&dopt=GenPept&RID=SZWVP0JX01N&log$=prottop&blast_rank=51) | guanylate cyclase 1, soluble, beta 3 [Homo sapiens] | [18.9](http://blast.ncbi.nlm.nih.gov/Blast.cgi#4504215%234504215) | 18.9 | 70% | 359 |
| [NP_009173.1](http://www.ncbi.nlm.nih.gov/entrez/query.fcgi?cmd=Retrieve&db=Protein&list_uids=6005743&dopt=GenPept&RID=SZWVP0JX01N&log$=prottop&blast_rank=52) | DEAD (Asp-Glu-Ala-As) box polypeptide 19 isoform 1 [Homo sapiens] | [18.9](http://blast.ncbi.nlm.nih.gov/Blast.cgi#6005743%236005743) | 18.9 | 70% | 359 |
| [NP_004938.1](http://www.ncbi.nlm.nih.gov/entrez/query.fcgi?cmd=Retrieve&db=Protein&list_uids=31415870&dopt=GenPept&RID=SZWVP0JX01N&log$=prottop&blast_rank=53) | dedicator of cytokinesis 3 [Homo sapiens] | [18.9](http://blast.ncbi.nlm.nih.gov/Blast.cgi#31415870%2331415870) | 18.9 | 50% | 359 |
| [NP_003566.1](http://www.ncbi.nlm.nih.gov/entrez/query.fcgi?cmd=Retrieve&db=Protein&list_uids=31657109&dopt=GenPept&RID=SZWVP0JX01N&log$=prottop&blast_rank=54) | zinc finger protein 282 [Homo sapiens] | [18.9](http://blast.ncbi.nlm.nih.gov/Blast.cgi#31657109%2331657109) | 18.9 | 50% | 359 |
| [NP_001161881.1](http://www.ncbi.nlm.nih.gov/entrez/query.fcgi?cmd=Retrieve&db=Protein&list_uids=270288802&dopt=GenPept&RID=SZWVP0JX01N&log$=prottop&blast_rank=55) | regulating synaptic membrane exocytosis 1 isoform 4 [Homo sapiens] | [18.5](http://blast.ncbi.nlm.nih.gov/Blast.cgi#270288802%23270288802) | 18.5 | 60% | 482 |
| [NP_001161880.1](http://www.ncbi.nlm.nih.gov/entrez/query.fcgi?cmd=Retrieve&db=Protein&list_uids=270288800&dopt=GenPept&RID=SZWVP0JX01N&log$=prottop&blast_rank=56) | regulating synaptic membrane exocytosis 1 isoform 3 [Homo sapiens] | [18.5](http://blast.ncbi.nlm.nih.gov/Blast.cgi#270288800%23270288800) | 18.5 | 60% | 482 |
| [NP_001161879.1](http://www.ncbi.nlm.nih.gov/entrez/query.fcgi?cmd=Retrieve&db=Protein&list_uids=270288798&dopt=GenPept&RID=SZWVP0JX01N&log$=prottop&blast_rank=57) | regulating synaptic membrane exocytosis 1 isoform 2 [Homo sapiens] | [18.5](http://blast.ncbi.nlm.nih.gov/Blast.cgi#270288798%23270288798) | 18.5 | 60% | 482 |
| [XP_002345254.1](http://www.ncbi.nlm.nih.gov/entrez/query.fcgi?cmd=Retrieve&db=Protein&list_uids=239757352&dopt=GenPept&RID=SZWVP0JX01N&log$=prottop&blast_rank=58) | PREDICTED: similar to LMARRLC1A [Homo sapiens] | [18.5](http://blast.ncbi.nlm.nih.gov/Blast.cgi#239757352%23239757352) | 18.5 | 60% | 482 |
| [XP_002348032.1](http://www.ncbi.nlm.nih.gov/entrez/query.fcgi?cmd=Retrieve&db=Protein&list_uids=239751859&dopt=GenPept&RID=SZWVP0JX01N&log$=prottop&blast_rank=59) | PREDICTED: similar to LMARRLC1A [Homo sapiens] | [18.5](http://blast.ncbi.nlm.nih.gov/Blast.cgi#239751859%23239751859) | 18.5 | 60% | 482 |
| [NP_001161882.1](http://www.ncbi.nlm.nih.gov/entrez/query.fcgi?cmd=Retrieve&db=Protein&list_uids=270288804&dopt=GenPept&RID=SZWVP0JX01N&log$=prottop&blast_rank=60) | regulating synaptic membrane exocytosis 1 isoform 5 [Homo sapiens] | [18.5](http://blast.ncbi.nlm.nih.gov/Blast.cgi#270288804%23270288804) | 18.5 | 60% | 482 |
| [XP_946127.3](http://www.ncbi.nlm.nih.gov/entrez/query.fcgi?cmd=Retrieve&db=Protein&list_uids=169168128&dopt=GenPept&RID=SZWVP0JX01N&log$=prottop&blast_rank=61) | PREDICTED: chromosome 5 open reading frame 50 [Homo sapiens] >ref|XP_933155.4| PREDICTED: chromosome 5 open reading frame 50 [Homo sapiens] >ref|XP_001715976.2| PREDICTED: chromosome 5 open reading frame 50 [Homo sapiens] | [18.5](http://blast.ncbi.nlm.nih.gov/Blast.cgi#169168128%23169168128) | 18.5 | 60% | 482 |
| [NP_001013049.1](http://www.ncbi.nlm.nih.gov/entrez/query.fcgi?cmd=Retrieve&db=Protein&list_uids=61743975&dopt=GenPept&RID=SZWVP0JX01N&log$=prottop&blast_rank=62) | SORCS receptor 1 isoform b [Homo sapiens] | [18.5](http://blast.ncbi.nlm.nih.gov/Blast.cgi#61743975%2361743975) | 32.2 | 80% | 482 |
| [NP_005459.2](http://www.ncbi.nlm.nih.gov/entrez/query.fcgi?cmd=Retrieve&db=Protein&list_uids=57232740&dopt=GenPept&RID=SZWVP0JX01N&log$=prottop&blast_rank=63) | N-acetylated alpha-linked acidic dipeptidase-like 1 [Homo sapiens] | [18.5](http://blast.ncbi.nlm.nih.gov/Blast.cgi#57232740%2357232740) | 18.5 | 50% | 482 |
| [NP_060217.1](http://www.ncbi.nlm.nih.gov/entrez/query.fcgi?cmd=Retrieve&db=Protein&list_uids=46519147&dopt=GenPept&RID=SZWVP0JX01N&log$=prottop&blast_rank=64) | ankyrin repeat and KH domain containing 1 isoform 1 [Homo sapiens] | [18.5](http://blast.ncbi.nlm.nih.gov/Blast.cgi#46519147%2346519147) | 18.5 | 50% | 482 |
| [NP_065741.3](http://www.ncbi.nlm.nih.gov/entrez/query.fcgi?cmd=Retrieve&db=Protein&list_uids=37620163&dopt=GenPept&RID=SZWVP0JX01N&log$=prottop&blast_rank=65) | ANKHD1-EIF4EBP3 protein [Homo sapiens] | [18.5](http://blast.ncbi.nlm.nih.gov/Blast.cgi#37620163%2337620163) | 18.5 | 50% | 482 |
| [NP_001615.1](http://www.ncbi.nlm.nih.gov/entrez/query.fcgi?cmd=Retrieve&db=Protein&list_uids=62988361&dopt=GenPept&RID=SZWVP0JX01N&log$=prottop&blast_rank=66) | absent in melanoma 1 [Homo sapiens] | [18.5](http://blast.ncbi.nlm.nih.gov/Blast.cgi#62988361%2362988361) | 18.5 | 70% | 482 |
| [NP_848552.1](http://www.ncbi.nlm.nih.gov/entrez/query.fcgi?cmd=Retrieve&db=Protein&list_uids=123701326&dopt=GenPept&RID=SZWVP0JX01N&log$=prottop&blast_rank=67) | zinc finger protein 831 [Homo sapiens] | [18.5](http://blast.ncbi.nlm.nih.gov/Blast.cgi#123701326%23123701326) | 18.5 | 50% | 482 |
| [NP_443150.3](http://www.ncbi.nlm.nih.gov/entrez/query.fcgi?cmd=Retrieve&db=Protein&list_uids=61743973&dopt=GenPept&RID=SZWVP0JX01N&log$=prottop&blast_rank=68) | SORCS receptor 1 isoform a [Homo sapiens] | [18.5](http://blast.ncbi.nlm.nih.gov/Blast.cgi#61743973%2361743973) | 32.2 | 80% | 482 |
| [NP_055804.2](http://www.ncbi.nlm.nih.gov/entrez/query.fcgi?cmd=Retrieve&db=Protein&list_uids=41054864&dopt=GenPept&RID=SZWVP0JX01N&log$=prottop&blast_rank=69) | regulating synaptic membrane exocytosis 1 isoform 1 [Homo sapiens] | [18.5](http://blast.ncbi.nlm.nih.gov/Blast.cgi#41054864%2341054864) | 18.5 | 60% | 482 |
| [NP_937790.2](http://www.ncbi.nlm.nih.gov/entrez/query.fcgi?cmd=Retrieve&db=Protein&list_uids=269315865&dopt=GenPept&RID=SZWVP0JX01N&log$=prottop&blast_rank=70) | abhydrolase domain containing 15 precursor [Homo sapiens] | [18.0](http://blast.ncbi.nlm.nih.gov/Blast.cgi#269315865%23269315865) | 18.0 | 60% | 647 |
| [XP_001716270.2](http://www.ncbi.nlm.nih.gov/entrez/query.fcgi?cmd=Retrieve&db=Protein&list_uids=239755585&dopt=GenPept&RID=SZWVP0JX01N&log$=prottop&blast_rank=71) | PREDICTED: hypothetical protein [Homo sapiens] | [18.0](http://blast.ncbi.nlm.nih.gov/Blast.cgi#239755585%23239755585) | 33.9 | 60% | 647 |
| [XP_002346244.1](http://www.ncbi.nlm.nih.gov/entrez/query.fcgi?cmd=Retrieve&db=Protein&list_uids=239755187&dopt=GenPept&RID=SZWVP0JX01N&log$=prottop&blast_rank=72) | PREDICTED: similar to hemicentin 2 [Homo sapiens] | [18.0](http://blast.ncbi.nlm.nih.gov/Blast.cgi#239755187%23239755187) | 18.0 | 60% | 647 |
| [XP_001716763.2](http://www.ncbi.nlm.nih.gov/entrez/query.fcgi?cmd=Retrieve&db=Protein&list_uids=239750101&dopt=GenPept&RID=SZWVP0JX01N&log$=prottop&blast_rank=73) | PREDICTED: hypothetical protein [Homo sapiens] | [18.0](http://blast.ncbi.nlm.nih.gov/Blast.cgi#239750101%23239750101) | 33.9 | 60% | 647 |
| [XP_002343120.1](http://www.ncbi.nlm.nih.gov/entrez/query.fcgi?cmd=Retrieve&db=Protein&list_uids=239744334&dopt=GenPept&RID=SZWVP0JX01N&log$=prottop&blast_rank=74) | PREDICTED: hypothetical protein XP_002343120 [Homo sapiens] >ref|XP_002347255.1| PREDICTED: hypothetical protein [Homo sapiens] >ref|XP_002344610.1| PREDICTED: hypothetical protein [Homo sapiens] | [18.0](http://blast.ncbi.nlm.nih.gov/Blast.cgi#239744334%23239744334) | 18.0 | 60% | 647 |
| [NP_001120843.1](http://www.ncbi.nlm.nih.gov/entrez/query.fcgi?cmd=Retrieve&db=Protein&list_uids=188497637&dopt=GenPept&RID=SZWVP0JX01N&log$=prottop&blast_rank=75) | cell division cycle associated 7-like isoform 3 [Homo sapiens] | [18.0](http://blast.ncbi.nlm.nih.gov/Blast.cgi#188497637%23188497637) | 18.0 | 50% | 647 |
| [XP_001716273.1](http://www.ncbi.nlm.nih.gov/entrez/query.fcgi?cmd=Retrieve&db=Protein&list_uids=169202233&dopt=GenPept&RID=SZWVP0JX01N&log$=prottop&blast_rank=76) | PREDICTED: hypothetical protein [Homo sapiens] | [18.0](http://blast.ncbi.nlm.nih.gov/Blast.cgi#169202233%23169202233) | 33.9 | 60% | 647 |
| [XP_001716079.1](http://www.ncbi.nlm.nih.gov/entrez/query.fcgi?cmd=Retrieve&db=Protein&list_uids=169192119&dopt=GenPept&RID=SZWVP0JX01N&log$=prottop&blast_rank=77) | PREDICTED: hypothetical protein [Homo sapiens] >ref|XP_001716818.1| PREDICTED: hypothetical protein [Homo sapiens] >ref|XP_001718753.1| PREDICTED: hypothetical protein [Homo sapiens] | [18.0](http://blast.ncbi.nlm.nih.gov/Blast.cgi#169192119%23169192119) | 18.0 | 50% | 647 |
| [NP_001032.2](http://www.ncbi.nlm.nih.gov/entrez/query.fcgi?cmd=Retrieve&db=Protein&list_uids=157364974&dopt=GenPept&RID=SZWVP0JX01N&log$=prottop&blast_rank=78) | sucrase-isomaltase [Homo sapiens] | [18.0](http://blast.ncbi.nlm.nih.gov/Blast.cgi#157364974%23157364974) | 28.8 | 60% | 647 |
| [NP_037450.2](http://www.ncbi.nlm.nih.gov/entrez/query.fcgi?cmd=Retrieve&db=Protein&list_uids=149192855&dopt=GenPept&RID=SZWVP0JX01N&log$=prottop&blast_rank=79) | HLA-B associated transcript 2-like [Homo sapiens] | [18.0](http://blast.ncbi.nlm.nih.gov/Blast.cgi#149192855%23149192855) | 31.4 | 60% | 647 |
| [NP_001128214.1](http://www.ncbi.nlm.nih.gov/entrez/query.fcgi?cmd=Retrieve&db=Protein&list_uids=197927160&dopt=GenPept&RID=SZWVP0JX01N&log$=prottop&blast_rank=80) | solute carrier family 4, sodium bicarbonate cotransporter, member 4 isoform 3 [Homo sapiens] | [18.0](http://blast.ncbi.nlm.nih.gov/Blast.cgi#197927160%23197927160) | 18.0 | 70% | 647 |
| [NP_067019.3](http://www.ncbi.nlm.nih.gov/entrez/query.fcgi?cmd=Retrieve&db=Protein&list_uids=125987596&dopt=GenPept&RID=SZWVP0JX01N&log$=prottop&blast_rank=81) | sodium bicarbonate transporter 4 isoform a [Homo sapiens] | [18.0](http://blast.ncbi.nlm.nih.gov/Blast.cgi#125987596%23125987596) | 18.0 | 70% | 647 |
| [NP_689811.2](http://www.ncbi.nlm.nih.gov/entrez/query.fcgi?cmd=Retrieve&db=Protein&list_uids=154937340&dopt=GenPept&RID=SZWVP0JX01N&log$=prottop&blast_rank=82) | ring finger protein 190 [Homo sapiens] >ref|NP_001094345.1| ring finger protein 190 [Homo sapiens] | [18.0](http://blast.ncbi.nlm.nih.gov/Blast.cgi#154937340%23154937340) | 18.0 | 60% | 647 |
| [XP_943795.2](http://www.ncbi.nlm.nih.gov/entrez/query.fcgi?cmd=Retrieve&db=Protein&list_uids=169216272&dopt=GenPept&RID=SZWVP0JX01N&log$=prottop&blast_rank=83) | PREDICTED: hypothetical protein [Homo sapiens] >ref|XP_933071.2| PREDICTED: hypothetical protein [Homo sapiens] | [18.0](http://blast.ncbi.nlm.nih.gov/Blast.cgi#169216272%23169216272) | 18.0 | 70% | 647 |
| [NP_001120842.1](http://www.ncbi.nlm.nih.gov/entrez/query.fcgi?cmd=Retrieve&db=Protein&list_uids=188497635&dopt=GenPept&RID=SZWVP0JX01N&log$=prottop&blast_rank=84) | cell division cycle associated 7-like isoform 2 [Homo sapiens] | [18.0](http://blast.ncbi.nlm.nih.gov/Blast.cgi#188497635%23188497635) | 18.0 | 50% | 647 |
| [NP_001124295.1](http://www.ncbi.nlm.nih.gov/entrez/query.fcgi?cmd=Retrieve&db=Protein&list_uids=195927037&dopt=GenPept&RID=SZWVP0JX01N&log$=prottop&blast_rank=85) | DNA (cytosine-5-)-methyltransferase 1 isoform a [Homo sapiens] | [18.0](http://blast.ncbi.nlm.nih.gov/Blast.cgi#195927037%23195927037) | 18.0 | 70% | 647 |
| [XP_001126968.1](http://www.ncbi.nlm.nih.gov/entrez/query.fcgi?cmd=Retrieve&db=Protein&list_uids=113414226&dopt=GenPept&RID=SZWVP0JX01N&log$=prottop&blast_rank=86) | PREDICTED: hypothetical protein [Homo sapiens] >ref|XP_001717105.1| PREDICTED: hypothetical protein [Homo sapiens] | [18.0](http://blast.ncbi.nlm.nih.gov/Blast.cgi#113414226%23113414226) | 18.0 | 50% | 647 |
| [XP_001130675.1](http://www.ncbi.nlm.nih.gov/entrez/query.fcgi?cmd=Retrieve&db=Protein&list_uids=113413437&dopt=GenPept&RID=SZWVP0JX01N&log$=prottop&blast_rank=87) | PREDICTED: hypothetical protein [Homo sapiens] | [18.0](http://blast.ncbi.nlm.nih.gov/Blast.cgi#113413437%23113413437) | 18.0 | 50% | 647 |
| [NP_001034461.1](http://www.ncbi.nlm.nih.gov/entrez/query.fcgi?cmd=Retrieve&db=Protein&list_uids=86439957&dopt=GenPept&RID=SZWVP0JX01N&log$=prottop&blast_rank=88) | HEPACAM family member 2 isoform 1 [Homo sapiens] | [18.0](http://blast.ncbi.nlm.nih.gov/Blast.cgi#86439957%2386439957) | 18.0 | 50% | 647 |
| [NP_112733.2](http://www.ncbi.nlm.nih.gov/entrez/query.fcgi?cmd=Retrieve&db=Protein&list_uids=89142735&dopt=GenPept&RID=SZWVP0JX01N&log$=prottop&blast_rank=89) | alpha 3 type IV collagen isoform 4 precursor [Homo sapiens] | [18.0](http://blast.ncbi.nlm.nih.gov/Blast.cgi#89142735%2389142735) | 59.8 | 90% | 647 |
| [NP_112730.2](http://www.ncbi.nlm.nih.gov/entrez/query.fcgi?cmd=Retrieve&db=Protein&list_uids=89142733&dopt=GenPept&RID=SZWVP0JX01N&log$=prottop&blast_rank=90) | alpha 3 type IV collagen isoform 2 precursor [Homo sapiens] | [18.0](http://blast.ncbi.nlm.nih.gov/Blast.cgi#89142733%2389142733) | 59.8 | 90% | 647 |
| [NP_112734.2](http://www.ncbi.nlm.nih.gov/entrez/query.fcgi?cmd=Retrieve&db=Protein&list_uids=89142737&dopt=GenPept&RID=SZWVP0JX01N&log$=prottop&blast_rank=91) | alpha 3 type IV collagen isoform 5 precursor [Homo sapiens] | [18.0](http://blast.ncbi.nlm.nih.gov/Blast.cgi#89142737%2389142737) | 59.8 | 90% | 647 |
| [NP_955389.1](http://www.ncbi.nlm.nih.gov/entrez/query.fcgi?cmd=Retrieve&db=Protein&list_uids=40788018&dopt=GenPept&RID=SZWVP0JX01N&log$=prottop&blast_rank=92) | Rho GTPase activating protein 11A isoform 2 [Homo sapiens] | [18.0](http://blast.ncbi.nlm.nih.gov/Blast.cgi#40788018%2340788018) | 18.0 | 50% | 647 |
| [NP_000082.2](http://www.ncbi.nlm.nih.gov/entrez/query.fcgi?cmd=Retrieve&db=Protein&list_uids=89142730&dopt=GenPept&RID=SZWVP0JX01N&log$=prottop&blast_rank=93) | alpha 3 type IV collagen isoform 1 precursor [Homo sapiens] | [18.0](http://blast.ncbi.nlm.nih.gov/Blast.cgi#89142730%2389142730) | 59.8 | 90% | 647 |
| [NP_001034930.1](http://www.ncbi.nlm.nih.gov/entrez/query.fcgi?cmd=Retrieve&db=Protein&list_uids=89886350&dopt=GenPept&RID=SZWVP0JX01N&log$=prottop&blast_rank=94) | Rho GTPase activating protein 11B [Homo sapiens] | [18.0](http://blast.ncbi.nlm.nih.gov/Blast.cgi#89886350%2389886350) | 18.0 | 50% | 647 |
| [NP_937794.1](http://www.ncbi.nlm.nih.gov/entrez/query.fcgi?cmd=Retrieve&db=Protein&list_uids=37700249&dopt=GenPept&RID=SZWVP0JX01N&log$=prottop&blast_rank=95) | HEPACAM family member 2 isoform 2 [Homo sapiens] | [18.0](http://blast.ncbi.nlm.nih.gov/Blast.cgi#37700249%2337700249) | 18.0 | 50% | 647 |
| [NP_653277.2](http://www.ncbi.nlm.nih.gov/entrez/query.fcgi?cmd=Retrieve&db=Protein&list_uids=269914167&dopt=GenPept&RID=SZWVP0JX01N&log$=prottop&blast_rank=96) | transmembrane emp24 protein transport domain containing 6 precursor [Homo sapiens] | [18.0](http://blast.ncbi.nlm.nih.gov/Blast.cgi#269914167%23269914167) | 18.0 | 50% | 647 |
| [NP_055598.1](http://www.ncbi.nlm.nih.gov/entrez/query.fcgi?cmd=Retrieve&db=Protein&list_uids=7661858&dopt=GenPept&RID=SZWVP0JX01N&log$=prottop&blast_rank=97) | Rho GTPase activating protein 11A isoform 1 [Homo sapiens] | [18.0](http://blast.ncbi.nlm.nih.gov/Blast.cgi#7661858%237661858) | 18.0 | 50% | 647 |
| [NP_114148.3](http://www.ncbi.nlm.nih.gov/entrez/query.fcgi?cmd=Retrieve&db=Protein&list_uids=88999586&dopt=GenPept&RID=SZWVP0JX01N&log$=prottop&blast_rank=98) | cell division cycle associated 7 isoform 1 [Homo sapiens] | [18.0](http://blast.ncbi.nlm.nih.gov/Blast.cgi#88999586%2388999586) | 18.0 | 50% | 647 |
| [NP_665809.1](http://www.ncbi.nlm.nih.gov/entrez/query.fcgi?cmd=Retrieve&db=Protein&list_uids=22027514&dopt=GenPept&RID=SZWVP0JX01N&log$=prottop&blast_rank=99) | cell division cycle associated 7 isoform 2 [Homo sapiens] | [18.0](http://blast.ncbi.nlm.nih.gov/Blast.cgi#22027514%2322027514) | 18.0 | 50% | 647 |
| [NP_001091954.1](http://www.ncbi.nlm.nih.gov/entrez/query.fcgi?cmd=Retrieve&db=Protein&list_uids=148596928&dopt=GenPept&RID=SZWVP0JX01N&log$=prottop&blast_rank=100) | solute carrier family 4, sodium bicarbonate cotransporter, member 4 isoform 1 [Homo sapiens] | [18.0](http://blast.ncbi.nlm.nih.gov/Blast.cgi#148596928%23148596928) | 18.0 | 70% | 647 |

| **Accession** | **Proteins with a match to QNPGETSKMN peptide** | **[Max score](http://blast.ncbi.nlm.nih.gov/Blast.cgi?CMD=Get&ALIGNMENTS=100&ALIGNMENT_VIEW=Pairwise&CDD_SEARCH_STATE=1&DATABASE_SORT=0&DESCRIPTIONS=100&ENTREZ_QUERY=txid9606 %5BORGN%5D&FIRST_QUERY_NUM=0&FORMAT_OBJECT=Alignment&FORMAT_PAGE_TARGET=&FORMAT_TYPE=HTML&GET_SEQUENCE=yes&I_THRESH=&MASK_CHAR=2&MASK_COLOR=1&NEW_DESIGN=on&NEW_VIEW=yes&NUM_OVERVIEW=100&OLD_BLAST=false&PAGE=Proteins&QUERY_INDEX=0&QUERY_NUMBER=0&RESULTS_PAGE_TARGET=&RID=SZXS4X4401N&SHOW_LINKOUT=yes&SHOW_OVERVIEW=yes&STEP_NUMBER=&WORD_SIZE=2&DISPLAY_SORT=1&HSP_SORT=1" \l "sort_mark)** | **[Total score](http://blast.ncbi.nlm.nih.gov/Blast.cgi?CMD=Get&ALIGNMENTS=100&ALIGNMENT_VIEW=Pairwise&CDD_SEARCH_STATE=1&DATABASE_SORT=0&DESCRIPTIONS=100&ENTREZ_QUERY=txid9606 %5BORGN%5D&FIRST_QUERY_NUM=0&FORMAT_OBJECT=Alignment&FORMAT_PAGE_TARGET=&FORMAT_TYPE=HTML&GET_SEQUENCE=yes&I_THRESH=&MASK_CHAR=2&MASK_COLOR=1&NEW_DESIGN=on&NEW_VIEW=yes&NUM_OVERVIEW=100&OLD_BLAST=false&PAGE=Proteins&QUERY_INDEX=0&QUERY_NUMBER=0&RESULTS_PAGE_TARGET=&RID=SZXS4X4401N&SHOW_LINKOUT=yes&SHOW_OVERVIEW=yes&STEP_NUMBER=&WORD_SIZE=2&DISPLAY_SORT=2&HSP_SORT=1" \l "sort_mark)** | **[Query coverage](http://blast.ncbi.nlm.nih.gov/Blast.cgi?CMD=Get&ALIGNMENTS=100&ALIGNMENT_VIEW=Pairwise&CDD_SEARCH_STATE=1&DATABASE_SORT=0&DESCRIPTIONS=100&ENTREZ_QUERY=txid9606 %5BORGN%5D&FIRST_QUERY_NUM=0&FORMAT_OBJECT=Alignment&FORMAT_PAGE_TARGET=&FORMAT_TYPE=HTML&GET_SEQUENCE=yes&I_THRESH=&MASK_CHAR=2&MASK_COLOR=1&NEW_DESIGN=on&NEW_VIEW=yes&NUM_OVERVIEW=100&OLD_BLAST=false&PAGE=Proteins&QUERY_INDEX=0&QUERY_NUMBER=0&RESULTS_PAGE_TARGET=&RID=SZXS4X4401N&SHOW_LINKOUT=yes&SHOW_OVERVIEW=yes&STEP_NUMBER=&WORD_SIZE=2&DISPLAY_SORT=4&HSP_SORT=0" \l "sort_mark)** | **[E value](http://blast.ncbi.nlm.nih.gov/Blast.cgi?CMD=Get&ALIGNMENTS=100&ALIGNMENT_VIEW=Pairwise&CDD_SEARCH_STATE=1&DATABASE_SORT=0&DESCRIPTIONS=100&ENTREZ_QUERY=txid9606 %5BORGN%5D&FIRST_QUERY_NUM=0&FORMAT_OBJECT=Alignment&FORMAT_PAGE_TARGET=&FORMAT_TYPE=HTML&GET_SEQUENCE=yes&I_THRESH=&MASK_CHAR=2&MASK_COLOR=1&NEW_DESIGN=on&NEW_VIEW=yes&NUM_OVERVIEW=100&OLD_BLAST=false&PAGE=Proteins&QUERY_INDEX=0&QUERY_NUMBER=0&RESULTS_PAGE_TARGET=&RID=SZXS4X4401N&SHOW_LINKOUT=yes&SHOW_OVERVIEW=yes&STEP_NUMBER=&WORD_SIZE=2&DISPLAY_SORT=0&HSP_SORT=0" \l "sort_mark)** |
| --- | --- | --- | --- | --- | --- |
| [NP_849196.2](http://www.ncbi.nlm.nih.gov/entrez/query.fcgi?cmd=Retrieve&db=Protein&list_uids=71834872&dopt=GenPept&RID=SZXS4X4401N&log$=prottop&blast_rank=1) | tumor differentially expressed 2-like [Homo sapiens] | [21.8](http://blast.ncbi.nlm.nih.gov/Blast.cgi#71834872%2371834872) | 21.8 | 70% | 46 |
| [NP_060876.5](http://www.ncbi.nlm.nih.gov/entrez/query.fcgi?cmd=Retrieve&db=Protein&list_uids=257471027&dopt=GenPept&RID=SZXS4X4401N&log$=prottop&blast_rank=2) | mucin 4 isoform a [Homo sapiens] | [21.4](http://blast.ncbi.nlm.nih.gov/Blast.cgi#257471027%23257471027) | 21.4 | 60% | 62 |
| [NP_000095.2](http://www.ncbi.nlm.nih.gov/entrez/query.fcgi?cmd=Retrieve&db=Protein&list_uids=189491763&dopt=GenPept&RID=SZXS4X4401N&log$=prottop&blast_rank=3) | cytochrome P450, family 1, subfamily B, polypeptide 1 [Homo sapiens] | [21.4](http://blast.ncbi.nlm.nih.gov/Blast.cgi#189491763%23189491763) | 21.4 | 90% | 62 |
| [NP_872289.1](http://www.ncbi.nlm.nih.gov/entrez/query.fcgi?cmd=Retrieve&db=Protein&list_uids=33286434&dopt=GenPept&RID=SZXS4X4401N&log$=prottop&blast_rank=4) | p47 protein isoform c [Homo sapiens] | [21.0](http://blast.ncbi.nlm.nih.gov/Blast.cgi#33286434%2333286434) | 21.0 | 60% | 83 |
| [NP_003875.3](http://www.ncbi.nlm.nih.gov/entrez/query.fcgi?cmd=Retrieve&db=Protein&list_uids=40805843&dopt=GenPept&RID=SZXS4X4401N&log$=prottop&blast_rank=5) | K(lysine) acetyltransferase 2B [Homo sapiens] | [21.0](http://blast.ncbi.nlm.nih.gov/Blast.cgi#40805843%2340805843) | 21.0 | 80% | 83 |
| [NP_057227.2](http://www.ncbi.nlm.nih.gov/entrez/query.fcgi?cmd=Retrieve&db=Protein&list_uids=20149635&dopt=GenPept&RID=SZXS4X4401N&log$=prottop&blast_rank=6) | p47 protein isoform a [Homo sapiens] | [21.0](http://blast.ncbi.nlm.nih.gov/Blast.cgi#20149635%2320149635) | 21.0 | 60% | 83 |
| [NP_061327.2](http://www.ncbi.nlm.nih.gov/entrez/query.fcgi?cmd=Retrieve&db=Protein&list_uids=29568099&dopt=GenPept&RID=SZXS4X4401N&log$=prottop&blast_rank=7) | p47 protein isoform b [Homo sapiens] | [21.0](http://blast.ncbi.nlm.nih.gov/Blast.cgi#29568099%2329568099) | 21.0 | 60% | 83 |
| [NP_002688.2](http://www.ncbi.nlm.nih.gov/entrez/query.fcgi?cmd=Retrieve&db=Protein&list_uids=42476164&dopt=GenPept&RID=SZXS4X4401N&log$=prottop&blast_rank=8) | POU class 2 homeobox 1 [Homo sapiens] | [21.0](http://blast.ncbi.nlm.nih.gov/Blast.cgi#42476164%2342476164) | 21.0 | 70% | 83 |
| [NP_005246.2](http://www.ncbi.nlm.nih.gov/entrez/query.fcgi?cmd=Retrieve&db=Protein&list_uids=157384971&dopt=GenPept&RID=SZXS4X4401N&log$=prottop&blast_rank=9) | cyclin G associated kinase [Homo sapiens] | [21.0](http://blast.ncbi.nlm.nih.gov/Blast.cgi#157384971%23157384971) | 21.0 | 70% | 83 |
| [NP_055561.2](http://www.ncbi.nlm.nih.gov/entrez/query.fcgi?cmd=Retrieve&db=Protein&list_uids=38045938&dopt=GenPept&RID=SZXS4X4401N&log$=prottop&blast_rank=10) | ring finger protein 144 [Homo sapiens] | [20.6](http://blast.ncbi.nlm.nih.gov/Blast.cgi#38045938%2338045938) | 20.6 | 70% | 111 |
| [NP_055719.1](http://www.ncbi.nlm.nih.gov/entrez/query.fcgi?cmd=Retrieve&db=Protein&list_uids=7662394&dopt=GenPept&RID=SZXS4X4401N&log$=prottop&blast_rank=11) | RAB11 family interacting protein 2 [Homo sapiens] | [20.6](http://blast.ncbi.nlm.nih.gov/Blast.cgi#7662394%237662394) | 20.6 | 60% | 111 |
| [NP_065071.1](http://www.ncbi.nlm.nih.gov/entrez/query.fcgi?cmd=Retrieve&db=Protein&list_uids=31543543&dopt=GenPept&RID=SZXS4X4401N&log$=prottop&blast_rank=12) | retinoic acid induced 17 [Homo sapiens] | [20.2](http://blast.ncbi.nlm.nih.gov/Blast.cgi#31543543%2331543543) | 20.2 | 90% | 149 |
| [NP_061939.3](http://www.ncbi.nlm.nih.gov/entrez/query.fcgi?cmd=Retrieve&db=Protein&list_uids=257900508&dopt=GenPept&RID=SZXS4X4401N&log$=prottop&blast_rank=13) | MAGE-like protein 2 [Homo sapiens] | [19.7](http://blast.ncbi.nlm.nih.gov/Blast.cgi#257900508%23257900508) | 19.7 | 50% | 200 |
| [NP_004500.3](http://www.ncbi.nlm.nih.gov/entrez/query.fcgi?cmd=Retrieve&db=Protein&list_uids=190343010&dopt=GenPept&RID=SZXS4X4401N&log$=prottop&blast_rank=14) | SP110 nuclear body protein isoform a [Homo sapiens] | [19.7](http://blast.ncbi.nlm.nih.gov/Blast.cgi#190343010%23190343010) | 34.8 | 50% | 200 |
| [NP_004501.3](http://www.ncbi.nlm.nih.gov/entrez/query.fcgi?cmd=Retrieve&db=Protein&list_uids=190343008&dopt=GenPept&RID=SZXS4X4401N&log$=prottop&blast_rank=15) | SP110 nuclear body protein isoform b [Homo sapiens] | [19.7](http://blast.ncbi.nlm.nih.gov/Blast.cgi#190343008%23190343008) | 34.8 | 50% | 200 |
| [NP_536349.2](http://www.ncbi.nlm.nih.gov/entrez/query.fcgi?cmd=Retrieve&db=Protein&list_uids=190343006&dopt=GenPept&RID=SZXS4X4401N&log$=prottop&blast_rank=16) | SP110 nuclear body protein isoform c [Homo sapiens] | [19.7](http://blast.ncbi.nlm.nih.gov/Blast.cgi#190343006%23190343006) | 34.8 | 50% | 200 |
| [XP_947707.3](http://www.ncbi.nlm.nih.gov/entrez/query.fcgi?cmd=Retrieve&db=Protein&list_uids=169216202&dopt=GenPept&RID=SZXS4X4401N&log$=prottop&blast_rank=17) | PREDICTED: melanoma antigen family B, 5 [Homo sapiens] >ref|XP_293407.6| PREDICTED: melanoma antigen family B, 5 [Homo sapiens] >ref|XP_001715207.2| PREDICTED: melanoma antigen family B, 5 [Homo sapiens] | [19.7](http://blast.ncbi.nlm.nih.gov/Blast.cgi#169216202%23169216202) | 19.7 | 50% | 200 |
| [XP_001130425.2](http://www.ncbi.nlm.nih.gov/entrez/query.fcgi?cmd=Retrieve&db=Protein&list_uids=169216802&dopt=GenPept&RID=SZXS4X4401N&log$=prottop&blast_rank=18) | PREDICTED: melanoma antigen family B, 17 [Homo sapiens] | [19.7](http://blast.ncbi.nlm.nih.gov/Blast.cgi#169216802%23169216802) | 19.7 | 50% | 200 |
| [NP_001093631.1](http://www.ncbi.nlm.nih.gov/entrez/query.fcgi?cmd=Retrieve&db=Protein&list_uids=154448894&dopt=GenPept&RID=SZXS4X4401N&log$=prottop&blast_rank=19) | exportin 7 isoform a [Homo sapiens] | [19.7](http://blast.ncbi.nlm.nih.gov/Blast.cgi#154448894%23154448894) | 19.7 | 50% | 200 |
| [XP_947659.2](http://www.ncbi.nlm.nih.gov/entrez/query.fcgi?cmd=Retrieve&db=Protein&list_uids=169216191&dopt=GenPept&RID=SZXS4X4401N&log$=prottop&blast_rank=20) | PREDICTED: melanoma antigen family B, 17 [Homo sapiens] >ref|XP_001719446.1| PREDICTED: melanoma antigen family B, 17 [Homo sapiens] | [19.7](http://blast.ncbi.nlm.nih.gov/Blast.cgi#169216191%23169216191) | 19.7 | 50% | 200 |
| [NP_001034794.1](http://www.ncbi.nlm.nih.gov/entrez/query.fcgi?cmd=Retrieve&db=Protein&list_uids=89276766&dopt=GenPept&RID=SZXS4X4401N&log$=prottop&blast_rank=21) | trophinin isoform 5 [Homo sapiens] | [19.7](http://blast.ncbi.nlm.nih.gov/Blast.cgi#89276766%2389276766) | 19.7 | 50% | 200 |
| [NP_004691.2](http://www.ncbi.nlm.nih.gov/entrez/query.fcgi?cmd=Retrieve&db=Protein&list_uids=26638653&dopt=GenPept&RID=SZXS4X4401N&log$=prottop&blast_rank=22) | potassium voltage-gated channel KQT-like protein 4 isoform a [Homo sapiens] | [19.7](http://blast.ncbi.nlm.nih.gov/Blast.cgi#26638653%2326638653) | 32.7 | 70% | 200 |
| [NP_001093391.1](http://www.ncbi.nlm.nih.gov/entrez/query.fcgi?cmd=Retrieve&db=Protein&list_uids=153791873&dopt=GenPept&RID=SZXS4X4401N&log$=prottop&blast_rank=23) | melanoma antigen family B, 16 [Homo sapiens] | [19.7](http://blast.ncbi.nlm.nih.gov/Blast.cgi#153791873%23153791873) | 19.7 | 50% | 200 |
| [NP_002355.2](http://www.ncbi.nlm.nih.gov/entrez/query.fcgi?cmd=Retrieve&db=Protein&list_uids=222418639&dopt=GenPept&RID=SZXS4X4401N&log$=prottop&blast_rank=24) | melanoma antigen family B, 2 [Homo sapiens] | [19.7](http://blast.ncbi.nlm.nih.gov/Blast.cgi#222418639%23222418639) | 19.7 | 50% | 200 |
| [NP_872312.2](http://www.ncbi.nlm.nih.gov/entrez/query.fcgi?cmd=Retrieve&db=Protein&list_uids=73623441&dopt=GenPept&RID=SZXS4X4401N&log$=prottop&blast_rank=25) | melanoma antigen family B, 10 [Homo sapiens] | [19.7](http://blast.ncbi.nlm.nih.gov/Blast.cgi#73623441%2373623441) | 19.7 | 50% | 200 |
| [NP_694546.1](http://www.ncbi.nlm.nih.gov/entrez/query.fcgi?cmd=Retrieve&db=Protein&list_uids=24430155&dopt=GenPept&RID=SZXS4X4401N&log$=prottop&blast_rank=26) | proteasome 26S ATPase subunit 4 isoform 2 [Homo sapiens] | [19.7](http://blast.ncbi.nlm.nih.gov/Blast.cgi#24430155%2324430155) | 30.1 | 70% | 200 |
| [XP_947602.1](http://www.ncbi.nlm.nih.gov/entrez/query.fcgi?cmd=Retrieve&db=Protein&list_uids=89064750&dopt=GenPept&RID=SZXS4X4401N&log$=prottop&blast_rank=27) | PREDICTED: similar to proteasome 26S ATPase subunit 4 [Homo sapiens] | [19.7](http://blast.ncbi.nlm.nih.gov/Blast.cgi#89064750%2389064750) | 30.1 | 70% | 200 |
| [NP_004636.1](http://www.ncbi.nlm.nih.gov/entrez/query.fcgi?cmd=Retrieve&db=Protein&list_uids=4758024&dopt=GenPept&RID=SZXS4X4401N&log$=prottop&blast_rank=28) | coilin [Homo sapiens] | [19.7](http://blast.ncbi.nlm.nih.gov/Blast.cgi#4758024%234758024) | 19.7 | 80% | 200 |
| [NP_835260.2](http://www.ncbi.nlm.nih.gov/entrez/query.fcgi?cmd=Retrieve&db=Protein&list_uids=87196343&dopt=GenPept&RID=SZXS4X4401N&log$=prottop&blast_rank=29) | PDZ domain containing 2 [Homo sapiens] | [19.7](http://blast.ncbi.nlm.nih.gov/Blast.cgi#87196343%2387196343) | 59.8 | 90% | 200 |
| [NP_001093632.1](http://www.ncbi.nlm.nih.gov/entrez/query.fcgi?cmd=Retrieve&db=Protein&list_uids=154448896&dopt=GenPept&RID=SZXS4X4401N&log$=prottop&blast_rank=30) | exportin 7 isoform c [Homo sapiens] | [19.7](http://blast.ncbi.nlm.nih.gov/Blast.cgi#154448896%23154448896) | 19.7 | 50% | 200 |
| [NP_001011544.1](http://www.ncbi.nlm.nih.gov/entrez/query.fcgi?cmd=Retrieve&db=Protein&list_uids=61744473&dopt=GenPept&RID=SZXS4X4401N&log$=prottop&blast_rank=31) | melanoma antigen family A, 11 isoform b [Homo sapiens] | [19.7](http://blast.ncbi.nlm.nih.gov/Blast.cgi#61744473%2361744473) | 19.7 | 50% | 200 |
| [NP_005357.2](http://www.ncbi.nlm.nih.gov/entrez/query.fcgi?cmd=Retrieve&db=Protein&list_uids=65507078&dopt=GenPept&RID=SZXS4X4401N&log$=prottop&blast_rank=32) | melanoma antigen family A, 11 isoform a [Homo sapiens] | [19.7](http://blast.ncbi.nlm.nih.gov/Blast.cgi#65507078%2365507078) | 19.7 | 50% | 200 |
| [NP_002356.2](http://www.ncbi.nlm.nih.gov/entrez/query.fcgi?cmd=Retrieve&db=Protein&list_uids=29171714&dopt=GenPept&RID=SZXS4X4401N&log$=prottop&blast_rank=33) | melanoma antigen family B, 3 [Homo sapiens] | [19.7](http://blast.ncbi.nlm.nih.gov/Blast.cgi#29171714%2329171714) | 33.9 | 60% | 200 |
| [NP_597677.2](http://www.ncbi.nlm.nih.gov/entrez/query.fcgi?cmd=Retrieve&db=Protein&list_uids=47578105&dopt=GenPept&RID=SZXS4X4401N&log$=prottop&blast_rank=34) | delangin isoform A [Homo sapiens] | [19.7](http://blast.ncbi.nlm.nih.gov/Blast.cgi#47578105%2347578105) | 52.4 | 100% | 200 |
| [NP_056199.2](http://www.ncbi.nlm.nih.gov/entrez/query.fcgi?cmd=Retrieve&db=Protein&list_uids=47578107&dopt=GenPept&RID=SZXS4X4401N&log$=prottop&blast_rank=35) | delangin isoform B [Homo sapiens] | [19.7](http://blast.ncbi.nlm.nih.gov/Blast.cgi#47578107%2347578107) | 52.4 | 100% | 200 |
| [NP_055315.2](http://www.ncbi.nlm.nih.gov/entrez/query.fcgi?cmd=Retrieve&db=Protein&list_uids=21361437&dopt=GenPept&RID=SZXS4X4401N&log$=prottop&blast_rank=36) | HIV-1 Tat specific factor 1 [Homo sapiens] >ref|NP_001156752.1| HIV-1 Tat specific factor 1 [Homo sapiens] | [19.7](http://blast.ncbi.nlm.nih.gov/Blast.cgi#21361437%2321361437) | 19.7 | 50% | 200 |
| [NP_055839.3](http://www.ncbi.nlm.nih.gov/entrez/query.fcgi?cmd=Retrieve&db=Protein&list_uids=154448892&dopt=GenPept&RID=SZXS4X4401N&log$=prottop&blast_rank=37) | exportin 7 isoform b [Homo sapiens] | [19.7](http://blast.ncbi.nlm.nih.gov/Blast.cgi#154448892%23154448892) | 19.7 | 50% | 200 |
| [NP_055414.2](http://www.ncbi.nlm.nih.gov/entrez/query.fcgi?cmd=Retrieve&db=Protein&list_uids=19387846&dopt=GenPept&RID=SZXS4X4401N&log$=prottop&blast_rank=38) | melanoma antigen family D, 2 [Homo sapiens] >ref|NP_803182.1| melanoma antigen family D, 2 [Homo sapiens] >ref|NP_957516.1| melanoma antigen family D, 2 [Homo sapiens] | [19.7](http://blast.ncbi.nlm.nih.gov/Blast.cgi#19387846%2319387846) | 19.7 | 50% | 200 |
| [NP_808224.1](http://www.ncbi.nlm.nih.gov/entrez/query.fcgi?cmd=Retrieve&db=Protein&list_uids=29540541&dopt=GenPept&RID=SZXS4X4401N&log$=prottop&blast_rank=39) | trophinin isoform 2 [Homo sapiens] >ref|NP_057241.2| trophinin isoform 2 [Homo sapiens] | [19.7](http://blast.ncbi.nlm.nih.gov/Blast.cgi#29540541%2329540541) | 19.7 | 50% | 200 |
| [NP_110428.2](http://www.ncbi.nlm.nih.gov/entrez/query.fcgi?cmd=Retrieve&db=Protein&list_uids=29337292&dopt=GenPept&RID=SZXS4X4401N&log$=prottop&blast_rank=40) | melanoma antigen family D, 4B isoform 1 [Homo sapiens] >ref|NP_803879.1| melanoma antigen family D, 4B isoform 1 [Homo sapiens] | [19.7](http://blast.ncbi.nlm.nih.gov/Blast.cgi#29337292%2329337292) | 36.1 | 50% | 200 |
| [NP_008917.3](http://www.ncbi.nlm.nih.gov/entrez/query.fcgi?cmd=Retrieve&db=Protein&list_uids=52632377&dopt=GenPept&RID=SZXS4X4401N&log$=prottop&blast_rank=41) | melanoma antigen family D, 1 isoform b [Homo sapiens] >ref|NP_001005332.1| melanoma antigen family D, 1 isoform b [Homo sapiens] | [19.7](http://blast.ncbi.nlm.nih.gov/Blast.cgi#52632377%2352632377) | 35.6 | 90% | 200 |
| [NP_006494.1](http://www.ncbi.nlm.nih.gov/entrez/query.fcgi?cmd=Retrieve&db=Protein&list_uids=5729991&dopt=GenPept&RID=SZXS4X4401N&log$=prottop&blast_rank=42) | proteasome 26S ATPase subunit 4 isoform 1 [Homo sapiens] | [19.7](http://blast.ncbi.nlm.nih.gov/Blast.cgi#5729991%235729991) | 30.1 | 70% | 200 |
| [NP_001005333.1](http://www.ncbi.nlm.nih.gov/entrez/query.fcgi?cmd=Retrieve&db=Protein&list_uids=52632381&dopt=GenPept&RID=SZXS4X4401N&log$=prottop&blast_rank=43) | melanoma antigen family D, 1 isoform a [Homo sapiens] | [19.7](http://blast.ncbi.nlm.nih.gov/Blast.cgi#52632381%2352632381) | 35.6 | 90% | 200 |
| [NP_065983.1](http://www.ncbi.nlm.nih.gov/entrez/query.fcgi?cmd=Retrieve&db=Protein&list_uids=20143482&dopt=GenPept&RID=SZXS4X4401N&log$=prottop&blast_rank=44) | melanoma antigen family E, 1 [Homo sapiens] | [19.7](http://blast.ncbi.nlm.nih.gov/Blast.cgi#20143482%2320143482) | 52.8 | 80% | 200 |
| [NP_619649.1](http://www.ncbi.nlm.nih.gov/entrez/query.fcgi?cmd=Retrieve&db=Protein&list_uids=20162572&dopt=GenPept&RID=SZXS4X4401N&log$=prottop&blast_rank=45) | necdin-like 2 [Homo sapiens] | [19.7](http://blast.ncbi.nlm.nih.gov/Blast.cgi#20162572%2320162572) | 19.7 | 50% | 200 |
| [NP_689597.1](http://www.ncbi.nlm.nih.gov/entrez/query.fcgi?cmd=Retrieve&db=Protein&list_uids=22748823&dopt=GenPept&RID=SZXS4X4401N&log$=prottop&blast_rank=46) | Bardet-Biedl syndrome 5 [Homo sapiens] | [19.7](http://blast.ncbi.nlm.nih.gov/Blast.cgi#22748823%2322748823) | 19.7 | 50% | 200 |
| [NP_775970.1](http://www.ncbi.nlm.nih.gov/entrez/query.fcgi?cmd=Retrieve&db=Protein&list_uids=27734781&dopt=GenPept&RID=SZXS4X4401N&log$=prottop&blast_rank=47) | melanoma antigen family B, 18 [Homo sapiens] | [19.7](http://blast.ncbi.nlm.nih.gov/Blast.cgi#27734781%2327734781) | 19.7 | 50% | 200 |
| [NP_002358.1](http://www.ncbi.nlm.nih.gov/entrez/query.fcgi?cmd=Retrieve&db=Protein&list_uids=4505085&dopt=GenPept&RID=SZXS4X4401N&log$=prottop&blast_rank=48) | melanoma antigen family B, 4 [Homo sapiens] | [19.7](http://blast.ncbi.nlm.nih.gov/Blast.cgi#4505085%234505085) | 19.7 | 50% | 200 |
| [NP_803881.1](http://www.ncbi.nlm.nih.gov/entrez/query.fcgi?cmd=Retrieve&db=Protein&list_uids=29337296&dopt=GenPept&RID=SZXS4X4401N&log$=prottop&blast_rank=49) | melanoma antigen family D, 4B isoform 2 [Homo sapiens] >ref|NP_001092270.1| melanoma antigen family D, 4 [Homo sapiens] | [19.7](http://blast.ncbi.nlm.nih.gov/Blast.cgi#29337296%2329337296) | 36.1 | 50% | 200 |
| [NP_001094889.1](http://www.ncbi.nlm.nih.gov/entrez/query.fcgi?cmd=Retrieve&db=Protein&list_uids=210031154&dopt=GenPept&RID=SZXS4X4401N&log$=prottop&blast_rank=50) | zinc finger protein 541 [Homo sapiens] | [19.3](http://blast.ncbi.nlm.nih.gov/Blast.cgi#210031154%23210031154) | 19.3 | 50% | 268 |
| [NP_001009555.3](http://www.ncbi.nlm.nih.gov/entrez/query.fcgi?cmd=Retrieve&db=Protein&list_uids=193083141&dopt=GenPept&RID=SZXS4X4401N&log$=prottop&blast_rank=51) | SH3 domain containing 19 isoform a [Homo sapiens] | [19.3](http://blast.ncbi.nlm.nih.gov/Blast.cgi#193083141%23193083141) | 19.3 | 50% | 268 |
| [NP_036466.2](http://www.ncbi.nlm.nih.gov/entrez/query.fcgi?cmd=Retrieve&db=Protein&list_uids=154354979&dopt=GenPept&RID=SZXS4X4401N&log$=prottop&blast_rank=52) | myosin X [Homo sapiens] | [19.3](http://blast.ncbi.nlm.nih.gov/Blast.cgi#154354979%23154354979) | 19.3 | 50% | 268 |
| [NP_001129626.1](http://www.ncbi.nlm.nih.gov/entrez/query.fcgi?cmd=Retrieve&db=Protein&list_uids=209954797&dopt=GenPept&RID=SZXS4X4401N&log$=prottop&blast_rank=53) | ets-related isoform 3 [Homo sapiens] | [19.3](http://blast.ncbi.nlm.nih.gov/Blast.cgi#209954797%23209954797) | 19.3 | 60% | 268 |
| [NP_659458.2](http://www.ncbi.nlm.nih.gov/entrez/query.fcgi?cmd=Retrieve&db=Protein&list_uids=50539410&dopt=GenPept&RID=SZXS4X4401N&log$=prottop&blast_rank=54) | cellular modulator of immune recognition isoform 8 [Homo sapiens] >ref|NP_001002265.1| cellular modulator of immune recognition isoform 6 [Homo sapiens] >ref|NP_001002266.1| cellular modulator of immune recognition isoform 7 [Homo sapiens] | [19.3](http://blast.ncbi.nlm.nih.gov/Blast.cgi#50539410%2350539410) | 19.3 | 100% | 268 |
| [NP_060109.2](http://www.ncbi.nlm.nih.gov/entrez/query.fcgi?cmd=Retrieve&db=Protein&list_uids=47059046&dopt=GenPept&RID=SZXS4X4401N&log$=prottop&blast_rank=55) | dachsous 2 isoform 1 [Homo sapiens] | [19.3](http://blast.ncbi.nlm.nih.gov/Blast.cgi#47059046%2347059046) | 19.3 | 50% | 268 |
| [NP_056988.3](http://www.ncbi.nlm.nih.gov/entrez/query.fcgi?cmd=Retrieve&db=Protein&list_uids=84043963&dopt=GenPept&RID=SZXS4X4401N&log$=prottop&blast_rank=56) | eukaryotic translation initiation factor 5B [Homo sapiens] | [19.3](http://blast.ncbi.nlm.nih.gov/Blast.cgi#84043963%2384043963) | 19.3 | 70% | 268 |
| [NP_891548.1](http://www.ncbi.nlm.nih.gov/entrez/query.fcgi?cmd=Retrieve&db=Protein&list_uids=33667107&dopt=GenPept&RID=SZXS4X4401N&log$=prottop&blast_rank=57) | ets-related isoform 1 [Homo sapiens] | [19.3](http://blast.ncbi.nlm.nih.gov/Blast.cgi#33667107%2333667107) | 19.3 | 60% | 268 |
| [NP_596870.2](http://www.ncbi.nlm.nih.gov/entrez/query.fcgi?cmd=Retrieve&db=Protein&list_uids=110349721&dopt=GenPept&RID=SZXS4X4401N&log$=prottop&blast_rank=58) | titin isoform novex-3 [Homo sapiens] | [19.3](http://blast.ncbi.nlm.nih.gov/Blast.cgi#110349721%23110349721) | 125 | 70% | 268 |
| [NP_054889.2](http://www.ncbi.nlm.nih.gov/entrez/query.fcgi?cmd=Retrieve&db=Protein&list_uids=56549685&dopt=GenPept&RID=SZXS4X4401N&log$=prottop&blast_rank=59) | GTP-binding protein 8 isoform 1 [Homo sapiens] | [19.3](http://blast.ncbi.nlm.nih.gov/Blast.cgi#56549685%2356549685) | 19.3 | 80% | 268 |
| [NP_006020.4](http://www.ncbi.nlm.nih.gov/entrez/query.fcgi?cmd=Retrieve&db=Protein&list_uids=52486265&dopt=GenPept&RID=SZXS4X4401N&log$=prottop&blast_rank=60) | paraneoplastic antigen MA1 [Homo sapiens] | [19.3](http://blast.ncbi.nlm.nih.gov/Blast.cgi#52486265%2352486265) | 19.3 | 50% | 268 |
| [NP_003317.1](http://www.ncbi.nlm.nih.gov/entrez/query.fcgi?cmd=Retrieve&db=Protein&list_uids=4507603&dopt=GenPept&RID=SZXS4X4401N&log$=prottop&blast_rank=61) | tumor necrosis factor (ligand) superfamily, member 4 [Homo sapiens] | [19.3](http://blast.ncbi.nlm.nih.gov/Blast.cgi#4507603%234507603) | 19.3 | 50% | 268 |
| [NP_000087.1](http://www.ncbi.nlm.nih.gov/entrez/query.fcgi?cmd=Retrieve&db=Protein&list_uids=4557485&dopt=GenPept&RID=SZXS4X4401N&log$=prottop&blast_rank=62) | ceruloplasmin precursor [Homo sapiens] | [19.3](http://blast.ncbi.nlm.nih.gov/Blast.cgi#4557485%234557485) | 34.8 | 60% | 268 |
| [NP_004440.1](http://www.ncbi.nlm.nih.gov/entrez/query.fcgi?cmd=Retrieve&db=Protein&list_uids=4758300&dopt=GenPept&RID=SZXS4X4401N&log$=prottop&blast_rank=63) | ets-related isoform 2 [Homo sapiens] | [19.3](http://blast.ncbi.nlm.nih.gov/Blast.cgi#4758300%234758300) | 19.3 | 60% | 268 |
| [NP_001120.3](http://www.ncbi.nlm.nih.gov/entrez/query.fcgi?cmd=Retrieve&db=Protein&list_uids=53692189&dopt=GenPept&RID=SZXS4X4401N&log$=prottop&blast_rank=64) | adipocyte enhancer binding protein 1 precursor [Homo sapiens] | [19.3](http://blast.ncbi.nlm.nih.gov/Blast.cgi#53692189%2353692189) | 51.1 | 50% | 268 |
| [NP_065817.2](http://www.ncbi.nlm.nih.gov/entrez/query.fcgi?cmd=Retrieve&db=Protein&list_uids=157426845&dopt=GenPept&RID=SZXS4X4401N&log$=prottop&blast_rank=65) | protocadherin 19 isoform b [Homo sapiens] | [18.9](http://blast.ncbi.nlm.nih.gov/Blast.cgi#157426845%23157426845) | 33.5 | 70% | 359 |
| [NP_001098713.1](http://www.ncbi.nlm.nih.gov/entrez/query.fcgi?cmd=Retrieve&db=Protein&list_uids=157426847&dopt=GenPept&RID=SZXS4X4401N&log$=prottop&blast_rank=66) | protocadherin 19 isoform a [Homo sapiens] | [18.9](http://blast.ncbi.nlm.nih.gov/Blast.cgi#157426847%23157426847) | 33.5 | 70% | 359 |
| [NP_073600.3](http://www.ncbi.nlm.nih.gov/entrez/query.fcgi?cmd=Retrieve&db=Protein&list_uids=205360932&dopt=GenPept&RID=SZXS4X4401N&log$=prottop&blast_rank=67) | fibronectin type III domain containing 3B [Homo sapiens] >ref|NP_001128567.1| fibronectin type III domain containing 3B [Homo sapiens] | [18.9](http://blast.ncbi.nlm.nih.gov/Blast.cgi#205360932%23205360932) | 18.9 | 50% | 359 |
| [NP_001138497.1](http://www.ncbi.nlm.nih.gov/entrez/query.fcgi?cmd=Retrieve&db=Protein&list_uids=222537739&dopt=GenPept&RID=SZXS4X4401N&log$=prottop&blast_rank=68) | ER degradation enhancer, mannosidase alpha-like 2 isoform 2 [Homo sapiens] | [18.9](http://blast.ncbi.nlm.nih.gov/Blast.cgi#222537739%23222537739) | 18.9 | 50% | 359 |
| [NP_006506.3](http://www.ncbi.nlm.nih.gov/entrez/query.fcgi?cmd=Retrieve&db=Protein&list_uids=194306650&dopt=GenPept&RID=SZXS4X4401N&log$=prottop&blast_rank=69) | SET domain and mariner transposase fusion [Homo sapiens] | [18.9](http://blast.ncbi.nlm.nih.gov/Blast.cgi#194306650%23194306650) | 33.9 | 80% | 359 |
| [NP_001139118.1](http://www.ncbi.nlm.nih.gov/entrez/query.fcgi?cmd=Retrieve&db=Protein&list_uids=224548987&dopt=GenPept&RID=SZXS4X4401N&log$=prottop&blast_rank=70) | presenilin stabilization factor-like isoform 2 [Homo sapiens] | [18.9](http://blast.ncbi.nlm.nih.gov/Blast.cgi#224548987%23224548987) | 18.9 | 50% | 359 |
| [NP_079532.5](http://www.ncbi.nlm.nih.gov/entrez/query.fcgi?cmd=Retrieve&db=Protein&list_uids=156142199&dopt=GenPept&RID=SZXS4X4401N&log$=prottop&blast_rank=71) | euchromatic histone-lysine N-methyltransferase 2 isoform b [Homo sapiens] | [18.9](http://blast.ncbi.nlm.nih.gov/Blast.cgi#156142199%23156142199) | 18.9 | 60% | 359 |
| [NP_006700.3](http://www.ncbi.nlm.nih.gov/entrez/query.fcgi?cmd=Retrieve&db=Protein&list_uids=156142197&dopt=GenPept&RID=SZXS4X4401N&log$=prottop&blast_rank=72) | euchromatic histone-lysine N-methyltransferase 2 isoform a [Homo sapiens] | [18.9](http://blast.ncbi.nlm.nih.gov/Blast.cgi#156142197%23156142197) | 18.9 | 60% | 359 |
| [NP_055526.3](http://www.ncbi.nlm.nih.gov/entrez/query.fcgi?cmd=Retrieve&db=Protein&list_uids=190341065&dopt=GenPept&RID=SZXS4X4401N&log$=prottop&blast_rank=73) | CP110 protein [Homo sapiens] | [18.9](http://blast.ncbi.nlm.nih.gov/Blast.cgi#190341065%23190341065) | 18.9 | 60% | 359 |
| [NP_061855.1](http://www.ncbi.nlm.nih.gov/entrez/query.fcgi?cmd=Retrieve&db=Protein&list_uids=10092643&dopt=GenPept&RID=SZXS4X4401N&log$=prottop&blast_rank=74) | Yip1 domain family, member 1 [Homo sapiens] | [18.9](http://blast.ncbi.nlm.nih.gov/Blast.cgi#10092643%2310092643) | 18.9 | 80% | 359 |
| [NP_065847.1](http://www.ncbi.nlm.nih.gov/entrez/query.fcgi?cmd=Retrieve&db=Protein&list_uids=11991660&dopt=GenPept&RID=SZXS4X4401N&log$=prottop&blast_rank=75) | sema domain, transmembrane domain (TM), and cytoplasmic domain, (semaphorin) 6A precursor [Homo sapiens] | [18.9](http://blast.ncbi.nlm.nih.gov/Blast.cgi#11991660%2311991660) | 18.9 | 70% | 359 |
| [NP_000364.1](http://www.ncbi.nlm.nih.gov/entrez/query.fcgi?cmd=Retrieve&db=Protein&list_uids=4507835&dopt=GenPept&RID=SZXS4X4401N&log$=prottop&blast_rank=76) | uridine monophosphate synthase [Homo sapiens] | [18.9](http://blast.ncbi.nlm.nih.gov/Blast.cgi#4507835%234507835) | 18.9 | 50% | 359 |
| [NP_060687.2](http://www.ncbi.nlm.nih.gov/entrez/query.fcgi?cmd=Retrieve&db=Protein&list_uids=222537737&dopt=GenPept&RID=SZXS4X4401N&log$=prottop&blast_rank=77) | ER degradation enhancer, mannosidase alpha-like 2 isoform 1 [Homo sapiens] | [18.9](http://blast.ncbi.nlm.nih.gov/Blast.cgi#222537737%23222537737) | 18.9 | 50% | 359 |
| [NP_112591.2](http://www.ncbi.nlm.nih.gov/entrez/query.fcgi?cmd=Retrieve&db=Protein&list_uids=50726954&dopt=GenPept&RID=SZXS4X4401N&log$=prottop&blast_rank=78) | presenilin stabilization factor-like isoform 1 [Homo sapiens] | [18.9](http://blast.ncbi.nlm.nih.gov/Blast.cgi#50726954%2350726954) | 18.9 | 50% | 359 |
| [NP_848511.1](http://www.ncbi.nlm.nih.gov/entrez/query.fcgi?cmd=Retrieve&db=Protein&list_uids=30581117&dopt=GenPept&RID=SZXS4X4401N&log$=prottop&blast_rank=79) | SRY (sex determining region Y)-box 30 isoform a [Homo sapiens] | [18.9](http://blast.ncbi.nlm.nih.gov/Blast.cgi#30581117%2330581117) | 18.9 | 70% | 359 |
| [NP_002067.1](http://www.ncbi.nlm.nih.gov/entrez/query.fcgi?cmd=Retrieve&db=Protein&list_uids=4504061&dopt=GenPept&RID=SZXS4X4401N&log$=prottop&blast_rank=80) | glucosamine (N-acetyl)-6-sulfatase precursor [Homo sapiens] | [18.9](http://blast.ncbi.nlm.nih.gov/Blast.cgi#4504061%234504061) | 18.9 | 70% | 359 |
| [NP_001120680.1](http://www.ncbi.nlm.nih.gov/entrez/query.fcgi?cmd=Retrieve&db=Protein&list_uids=187761317&dopt=GenPept&RID=SZXS4X4401N&log$=prottop&blast_rank=81) | tet oncogene family member 2 isoform a [Homo sapiens] | [18.5](http://blast.ncbi.nlm.nih.gov/Blast.cgi#187761317%23187761317) | 33.5 | 90% | 482 |
| [NP_060098.3](http://www.ncbi.nlm.nih.gov/entrez/query.fcgi?cmd=Retrieve&db=Protein&list_uids=116063558&dopt=GenPept&RID=SZXS4X4401N&log$=prottop&blast_rank=82) | tet oncogene family member 2 isoform b [Homo sapiens] | [18.5](http://blast.ncbi.nlm.nih.gov/Blast.cgi#116063558%23116063558) | 18.5 | 60% | 482 |
| [NP_001074930.1](http://www.ncbi.nlm.nih.gov/entrez/query.fcgi?cmd=Retrieve&db=Protein&list_uids=125988395&dopt=GenPept&RID=SZXS4X4401N&log$=prottop&blast_rank=83) | jumonji domain containing 6 isoform 1 [Homo sapiens] | [18.5](http://blast.ncbi.nlm.nih.gov/Blast.cgi#125988395%23125988395) | 18.5 | 60% | 482 |
| [NP_002509.2](http://www.ncbi.nlm.nih.gov/entrez/query.fcgi?cmd=Retrieve&db=Protein&list_uids=22027471&dopt=GenPept&RID=SZXS4X4401N&log$=prottop&blast_rank=84) | neuronal PAS domain protein 2 [Homo sapiens] | [18.5](http://blast.ncbi.nlm.nih.gov/Blast.cgi#22027471%2322027471) | 18.5 | 80% | 482 |
| [NP_001030337.1](http://www.ncbi.nlm.nih.gov/entrez/query.fcgi?cmd=Retrieve&db=Protein&list_uids=78482614&dopt=GenPept&RID=SZXS4X4401N&log$=prottop&blast_rank=85) | vacuolar protein sorting 26 A isoform 2 [Homo sapiens] | [18.5](http://blast.ncbi.nlm.nih.gov/Blast.cgi#78482614%2378482614) | 18.5 | 60% | 482 |
| [NP_071767.3](http://www.ncbi.nlm.nih.gov/entrez/query.fcgi?cmd=Retrieve&db=Protein&list_uids=84626578&dopt=GenPept&RID=SZXS4X4401N&log$=prottop&blast_rank=86) | G protein beta subunit-like [Homo sapiens] | [18.5](http://blast.ncbi.nlm.nih.gov/Blast.cgi#84626578%2384626578) | 18.5 | 60% | 482 |
| [NP_001914.3](http://www.ncbi.nlm.nih.gov/entrez/query.fcgi?cmd=Retrieve&db=Protein&list_uids=148529014&dopt=GenPept&RID=SZXS4X4401N&log$=prottop&blast_rank=87) | damage-specific DNA binding protein 1 [Homo sapiens] >ref|XP_002347285.1| PREDICTED: hypothetical protein XP_002347285 [Homo sapiens] | [18.5](http://blast.ncbi.nlm.nih.gov/Blast.cgi#148529014%23148529014) | 33.1 | 70% | 482 |
| [NP_694571.2](http://www.ncbi.nlm.nih.gov/entrez/query.fcgi?cmd=Retrieve&db=Protein&list_uids=222136678&dopt=GenPept&RID=SZXS4X4401N&log$=prottop&blast_rank=88) | prickle homolog 1 [Homo sapiens] >ref|NP_001138353.1| prickle homolog 1 [Homo sapiens] >ref|NP_001138354.1| prickle homolog 1 [Homo sapiens] >ref|NP_001138355.1| prickle homolog 1 [Homo sapiens] | [18.5](http://blast.ncbi.nlm.nih.gov/Blast.cgi#222136678%23222136678) | 18.5 | 100% | 482 |
| [NP_004887.2](http://www.ncbi.nlm.nih.gov/entrez/query.fcgi?cmd=Retrieve&db=Protein&list_uids=17978519&dopt=GenPept&RID=SZXS4X4401N&log$=prottop&blast_rank=89) | vacuolar protein sorting 26 A isoform 1 [Homo sapiens] | [18.5](http://blast.ncbi.nlm.nih.gov/Blast.cgi#17978519%2317978519) | 18.5 | 60% | 482 |
| [NP_060579.3](http://www.ncbi.nlm.nih.gov/entrez/query.fcgi?cmd=Retrieve&db=Protein&list_uids=190194365&dopt=GenPept&RID=SZXS4X4401N&log$=prottop&blast_rank=90) | PAP associated domain containing 1 precursor [Homo sapiens] | [18.5](http://blast.ncbi.nlm.nih.gov/Blast.cgi#190194365%23190194365) | 18.5 | 70% | 482 |
| [NP_000718.1](http://www.ncbi.nlm.nih.gov/entrez/query.fcgi?cmd=Retrieve&db=Protein&list_uids=4502539&dopt=GenPept&RID=SZXS4X4401N&log$=prottop&blast_rank=91) | voltage-dependent calcium channel gamma-1 subunit [Homo sapiens] | [18.5](http://blast.ncbi.nlm.nih.gov/Blast.cgi#4502539%234502539) | 18.5 | 60% | 482 |
| [NP_055982.2](http://www.ncbi.nlm.nih.gov/entrez/query.fcgi?cmd=Retrieve&db=Protein&list_uids=125988389&dopt=GenPept&RID=SZXS4X4401N&log$=prottop&blast_rank=92) | jumonji domain containing 6 isoform 2 [Homo sapiens] | [18.5](http://blast.ncbi.nlm.nih.gov/Blast.cgi#125988389%23125988389) | 18.5 | 60% | 482 |
| [NP_060494.1](http://www.ncbi.nlm.nih.gov/entrez/query.fcgi?cmd=Retrieve&db=Protein&list_uids=8922281&dopt=GenPept&RID=SZXS4X4401N&log$=prottop&blast_rank=93) | WDYHV motif containing 1 [Homo sapiens] | [18.5](http://blast.ncbi.nlm.nih.gov/Blast.cgi#8922281%238922281) | 18.5 | 60% | 482 |
| [NP_115518.3](http://www.ncbi.nlm.nih.gov/entrez/query.fcgi?cmd=Retrieve&db=Protein&list_uids=255003833&dopt=GenPept&RID=SZXS4X4401N&log$=prottop&blast_rank=94) | centrosomal protein 192kDa [Homo sapiens] | [18.0](http://blast.ncbi.nlm.nih.gov/Blast.cgi#255003833%23255003833) | 28.4 | 60% | 647 |
| [XP_002344507.1](http://www.ncbi.nlm.nih.gov/entrez/query.fcgi?cmd=Retrieve&db=Protein&list_uids=239755232&dopt=GenPept&RID=SZXS4X4401N&log$=prottop&blast_rank=95) | PREDICTED: hypothetical protein [Homo sapiens] | [18.0](http://blast.ncbi.nlm.nih.gov/Blast.cgi#239755232%23239755232) | 18.0 | 50% | 647 |
| [XP_001715861.2](http://www.ncbi.nlm.nih.gov/entrez/query.fcgi?cmd=Retrieve&db=Protein&list_uids=239753784&dopt=GenPept&RID=SZXS4X4401N&log$=prottop&blast_rank=96) | PREDICTED: NK1 homeobox 1 [Homo sapiens] | [18.0](http://blast.ncbi.nlm.nih.gov/Blast.cgi#239753784%23239753784) | 18.0 | 70% | 647 |
| [XP_942752.4](http://www.ncbi.nlm.nih.gov/entrez/query.fcgi?cmd=Retrieve&db=Protein&list_uids=239748344&dopt=GenPept&RID=SZXS4X4401N&log$=prottop&blast_rank=97) | PREDICTED: NK1 homeobox 1 [Homo sapiens] | [18.0](http://blast.ncbi.nlm.nih.gov/Blast.cgi#239748344%23239748344) | 18.0 | 70% | 647 |
| [XP_002343007.1](http://www.ncbi.nlm.nih.gov/entrez/query.fcgi?cmd=Retrieve&db=Protein&list_uids=239744027&dopt=GenPept&RID=SZXS4X4401N&log$=prottop&blast_rank=98) | PREDICTED: hypothetical protein XP_002343007 [Homo sapiens] >ref|XP_002347159.1| PREDICTED: hypothetical protein [Homo sapiens] | [18.0](http://blast.ncbi.nlm.nih.gov/Blast.cgi#239744027%23239744027) | 18.0 | 50% | 647 |
| [NP_055919.2](http://www.ncbi.nlm.nih.gov/entrez/query.fcgi?cmd=Retrieve&db=Protein&list_uids=239047271&dopt=GenPept&RID=SZXS4X4401N&log$=prottop&blast_rank=99) | autophagy related 2A [Homo sapiens] | [18.0](http://blast.ncbi.nlm.nih.gov/Blast.cgi#239047271%23239047271) | 18.0 | 50% | 647 |
| [XP_931434.4](http://www.ncbi.nlm.nih.gov/entrez/query.fcgi?cmd=Retrieve&db=Protein&list_uids=239742243&dopt=GenPept&RID=SZXS4X4401N&log$=prottop&blast_rank=100) | PREDICTED: HPX-153 homeobox [Homo sapiens] |  |  |  |  |

| **Accession** | **Proteins with a match to KYRWYK peptide** | **[Max score](http://blast.ncbi.nlm.nih.gov/Blast.cgi?CMD=Get&ALIGNMENTS=100&ALIGNMENT_VIEW=Pairwise&CDD_SEARCH_STATE=1&DATABASE_SORT=0&DESCRIPTIONS=100&ENTREZ_QUERY=txid9606 %5BORGN%5D&FIRST_QUERY_NUM=0&FORMAT_OBJECT=Alignment&FORMAT_PAGE_TARGET=&FORMAT_TYPE=HTML&GET_SEQUENCE=yes&I_THRESH=&MASK_CHAR=2&MASK_COLOR=1&NEW_DESIGN=on&NEW_VIEW=yes&NUM_OVERVIEW=100&OLD_BLAST=false&PAGE=Proteins&QUERY_INDEX=0&QUERY_NUMBER=0&RESULTS_PAGE_TARGET=&RID=T03Z2ZXM01S&SHOW_LINKOUT=yes&SHOW_OVERVIEW=yes&STEP_NUMBER=&WORD_SIZE=2&DISPLAY_SORT=1&HSP_SORT=1" \l "sort_mark)** | **[Total score](http://blast.ncbi.nlm.nih.gov/Blast.cgi?CMD=Get&ALIGNMENTS=100&ALIGNMENT_VIEW=Pairwise&CDD_SEARCH_STATE=1&DATABASE_SORT=0&DESCRIPTIONS=100&ENTREZ_QUERY=txid9606 %5BORGN%5D&FIRST_QUERY_NUM=0&FORMAT_OBJECT=Alignment&FORMAT_PAGE_TARGET=&FORMAT_TYPE=HTML&GET_SEQUENCE=yes&I_THRESH=&MASK_CHAR=2&MASK_COLOR=1&NEW_DESIGN=on&NEW_VIEW=yes&NUM_OVERVIEW=100&OLD_BLAST=false&PAGE=Proteins&QUERY_INDEX=0&QUERY_NUMBER=0&RESULTS_PAGE_TARGET=&RID=T03Z2ZXM01S&SHOW_LINKOUT=yes&SHOW_OVERVIEW=yes&STEP_NUMBER=&WORD_SIZE=2&DISPLAY_SORT=2&HSP_SORT=1" \l "sort_mark)** | **[Query coverage](http://blast.ncbi.nlm.nih.gov/Blast.cgi?CMD=Get&ALIGNMENTS=100&ALIGNMENT_VIEW=Pairwise&CDD_SEARCH_STATE=1&DATABASE_SORT=0&DESCRIPTIONS=100&ENTREZ_QUERY=txid9606 %5BORGN%5D&FIRST_QUERY_NUM=0&FORMAT_OBJECT=Alignment&FORMAT_PAGE_TARGET=&FORMAT_TYPE=HTML&GET_SEQUENCE=yes&I_THRESH=&MASK_CHAR=2&MASK_COLOR=1&NEW_DESIGN=on&NEW_VIEW=yes&NUM_OVERVIEW=100&OLD_BLAST=false&PAGE=Proteins&QUERY_INDEX=0&QUERY_NUMBER=0&RESULTS_PAGE_TARGET=&RID=T03Z2ZXM01S&SHOW_LINKOUT=yes&SHOW_OVERVIEW=yes&STEP_NUMBER=&WORD_SIZE=2&DISPLAY_SORT=4&HSP_SORT=0" \l "sort_mark)** | **[E value](http://blast.ncbi.nlm.nih.gov/Blast.cgi?CMD=Get&ALIGNMENTS=100&ALIGNMENT_VIEW=Pairwise&CDD_SEARCH_STATE=1&DATABASE_SORT=0&DESCRIPTIONS=100&ENTREZ_QUERY=txid9606 %5BORGN%5D&FIRST_QUERY_NUM=0&FORMAT_OBJECT=Alignment&FORMAT_PAGE_TARGET=&FORMAT_TYPE=HTML&GET_SEQUENCE=yes&I_THRESH=&MASK_CHAR=2&MASK_COLOR=1&NEW_DESIGN=on&NEW_VIEW=yes&NUM_OVERVIEW=100&OLD_BLAST=false&PAGE=Proteins&QUERY_INDEX=0&QUERY_NUMBER=0&RESULTS_PAGE_TARGET=&RID=T03Z2ZXM01S&SHOW_LINKOUT=yes&SHOW_OVERVIEW=yes&STEP_NUMBER=&WORD_SIZE=2&DISPLAY_SORT=0&HSP_SORT=0" \l "sort_mark)** |
| --- | --- | --- | --- | --- | --- |
| [XP_002346284.1](http://www.ncbi.nlm.nih.gov/entrez/query.fcgi?cmd=Retrieve&db=Protein&list_uids=239758065&dopt=GenPept&RID=T03Z2ZXM01S&log$=prottop&blast_rank=1) | PREDICTED: similar to BMS1-like, ribosome assembly protein [Homo sapiens] | [21.0](http://blast.ncbi.nlm.nih.gov/Blast.cgi#239758065%23239758065) | 21.0 | 100% | 50 |
| [NP_055568.3](http://www.ncbi.nlm.nih.gov/entrez/query.fcgi?cmd=Retrieve&db=Protein&list_uids=224589071&dopt=GenPept&RID=T03Z2ZXM01S&log$=prottop&blast_rank=2) | BMS1-like, ribosome assembly protein [Homo sapiens] | [21.0](http://blast.ncbi.nlm.nih.gov/Blast.cgi#224589071%23224589071) | 21.0 | 100% | 50 |
| [XP_001715032.1](http://www.ncbi.nlm.nih.gov/entrez/query.fcgi?cmd=Retrieve&db=Protein&list_uids=169207217&dopt=GenPept&RID=T03Z2ZXM01S&log$=prottop&blast_rank=3) | PREDICTED: zinc finger homeobox 2 [Homo sapiens] | [20.6](http://blast.ncbi.nlm.nih.gov/Blast.cgi#169207217%23169207217) | 20.6 | 66% | 67 |
| [XP_001720147.1](http://www.ncbi.nlm.nih.gov/entrez/query.fcgi?cmd=Retrieve&db=Protein&list_uids=169207014&dopt=GenPept&RID=T03Z2ZXM01S&log$=prottop&blast_rank=4) | PREDICTED: zinc finger homeobox 2 [Homo sapiens] >ref|XP_001722771.1| PREDICTED: zinc finger homeobox 2 [Homo sapiens] | [20.6](http://blast.ncbi.nlm.nih.gov/Blast.cgi#169207014%23169207014) | 20.6 | 66% | 67 |
| [NP_078843.2](http://www.ncbi.nlm.nih.gov/entrez/query.fcgi?cmd=Retrieve&db=Protein&list_uids=47059040&dopt=GenPept&RID=T03Z2ZXM01S&log$=prottop&blast_rank=5) | homeobox containing 1 [Homo sapiens] >ref|NP_001129198.1| homeobox containing 1 [Homo sapiens] | [20.6](http://blast.ncbi.nlm.nih.gov/Blast.cgi#47059040%2347059040) | 20.6 | 66% | 67 |
| [NP_000077.1](http://www.ncbi.nlm.nih.gov/entrez/query.fcgi?cmd=Retrieve&db=Protein&list_uids=4502889&dopt=GenPept&RID=T03Z2ZXM01S&log$=prottop&blast_rank=6) | ceroid-lipofuscinosis, neuronal 3 [Homo sapiens] >ref|NP_001035897.1| ceroid-lipofuscinosis, neuronal 3 [Homo sapiens] | [20.6](http://blast.ncbi.nlm.nih.gov/Blast.cgi#4502889%234502889) | 20.6 | 66% | 67 |
| [NP_075556.1](http://www.ncbi.nlm.nih.gov/entrez/query.fcgi?cmd=Retrieve&db=Protein&list_uids=13489095&dopt=GenPept&RID=T03Z2ZXM01S&log$=prottop&blast_rank=7) | sialoadhesin precursor [Homo sapiens] | [20.6](http://blast.ncbi.nlm.nih.gov/Blast.cgi#13489095%2313489095) | 20.6 | 66% | 67 |
| [NP_808881.3](http://www.ncbi.nlm.nih.gov/entrez/query.fcgi?cmd=Retrieve&db=Protein&list_uids=189027129&dopt=GenPept&RID=T03Z2ZXM01S&log$=prottop&blast_rank=8) | phosphodiesterase 12 [Homo sapiens] | [20.2](http://blast.ncbi.nlm.nih.gov/Blast.cgi#189027129%23189027129) | 20.2 | 83% | 89 |
| [NP_004002.2](http://www.ncbi.nlm.nih.gov/entrez/query.fcgi?cmd=Retrieve&db=Protein&list_uids=150036268&dopt=GenPept&RID=T03Z2ZXM01S&log$=prottop&blast_rank=9) | dystrophin Dp260-1 isoform [Homo sapiens] | [20.2](http://blast.ncbi.nlm.nih.gov/Blast.cgi#150036268%23150036268) | 20.2 | 83% | 89 |
| [NP_000100.2](http://www.ncbi.nlm.nih.gov/entrez/query.fcgi?cmd=Retrieve&db=Protein&list_uids=5032281&dopt=GenPept&RID=T03Z2ZXM01S&log$=prottop&blast_rank=10) | dystrophin Dp427c isoform [Homo sapiens] | [20.2](http://blast.ncbi.nlm.nih.gov/Blast.cgi#5032281%235032281) | 20.2 | 83% | 89 |
| [NP_003997.1](http://www.ncbi.nlm.nih.gov/entrez/query.fcgi?cmd=Retrieve&db=Protein&list_uids=5032283&dopt=GenPept&RID=T03Z2ZXM01S&log$=prottop&blast_rank=11) | dystrophin Dp427m isoform [Homo sapiens] | [20.2](http://blast.ncbi.nlm.nih.gov/Blast.cgi#5032283%235032283) | 20.2 | 83% | 89 |
| [NP_004003.1](http://www.ncbi.nlm.nih.gov/entrez/query.fcgi?cmd=Retrieve&db=Protein&list_uids=5032291&dopt=GenPept&RID=T03Z2ZXM01S&log$=prottop&blast_rank=12) | dystrophin Dp260-2 isoform [Homo sapiens] | [20.2](http://blast.ncbi.nlm.nih.gov/Blast.cgi#5032291%235032291) | 20.2 | 83% | 89 |
| [NP_003998.1](http://www.ncbi.nlm.nih.gov/entrez/query.fcgi?cmd=Retrieve&db=Protein&list_uids=5032285&dopt=GenPept&RID=T03Z2ZXM01S&log$=prottop&blast_rank=13) | dystrophin Dp427l isoform [Homo sapiens] >ref|NP_004001.1| dystrophin Dp427p2 isoform [Homo sapiens] | [20.2](http://blast.ncbi.nlm.nih.gov/Blast.cgi#5032285%235032285) | 20.2 | 83% | 89 |
| [NP_004000.1](http://www.ncbi.nlm.nih.gov/entrez/query.fcgi?cmd=Retrieve&db=Protein&list_uids=5032287&dopt=GenPept&RID=T03Z2ZXM01S&log$=prottop&blast_rank=14) | dystrophin Dp427p1 isoform [Homo sapiens] | [20.2](http://blast.ncbi.nlm.nih.gov/Blast.cgi#5032287%235032287) | 20.2 | 83% | 89 |
| [NP_003424.3](http://www.ncbi.nlm.nih.gov/entrez/query.fcgi?cmd=Retrieve&db=Protein&list_uids=154800449&dopt=GenPept&RID=T03Z2ZXM01S&log$=prottop&blast_rank=15) | zinc finger protein 132 [Homo sapiens] | [20.2](http://blast.ncbi.nlm.nih.gov/Blast.cgi#154800449%23154800449) | 20.2 | 83% | 89 |
| [NP_053586.1](http://www.ncbi.nlm.nih.gov/entrez/query.fcgi?cmd=Retrieve&db=Protein&list_uids=7669532&dopt=GenPept&RID=T03Z2ZXM01S&log$=prottop&blast_rank=16) | neuregulin 2 isoform 4 [Homo sapiens] | [20.2](http://blast.ncbi.nlm.nih.gov/Blast.cgi#7669532%237669532) | 20.2 | 83% | 89 |
| [NP_053585.1](http://www.ncbi.nlm.nih.gov/entrez/query.fcgi?cmd=Retrieve&db=Protein&list_uids=7669530&dopt=GenPept&RID=T03Z2ZXM01S&log$=prottop&blast_rank=17) | neuregulin 2 isoform 3 [Homo sapiens] | [20.2](http://blast.ncbi.nlm.nih.gov/Blast.cgi#7669530%237669530) | 20.2 | 83% | 89 |
| [NP_053584.1](http://www.ncbi.nlm.nih.gov/entrez/query.fcgi?cmd=Retrieve&db=Protein&list_uids=7669528&dopt=GenPept&RID=T03Z2ZXM01S&log$=prottop&blast_rank=18) | neuregulin 2 isoform 2 [Homo sapiens] | [20.2](http://blast.ncbi.nlm.nih.gov/Blast.cgi#7669528%237669528) | 20.2 | 83% | 89 |
| [NP_683695.1](http://www.ncbi.nlm.nih.gov/entrez/query.fcgi?cmd=Retrieve&db=Protein&list_uids=22507405&dopt=GenPept&RID=T03Z2ZXM01S&log$=prottop&blast_rank=19) | short chain dehydrogenase/reductase family 9C, member 7 [Homo sapiens] | [20.2](http://blast.ncbi.nlm.nih.gov/Blast.cgi#22507405%2322507405) | 20.2 | 83% | 89 |
| [NP_006415.2](http://www.ncbi.nlm.nih.gov/entrez/query.fcgi?cmd=Retrieve&db=Protein&list_uids=110611906&dopt=GenPept&RID=T03Z2ZXM01S&log$=prottop&blast_rank=20) | solute carrier family 34 (sodium phosphate), member 2 [Homo sapiens] | [20.2](http://blast.ncbi.nlm.nih.gov/Blast.cgi#110611906%23110611906) | 20.2 | 83% | 89 |
| [NP_003043.3](http://www.ncbi.nlm.nih.gov/entrez/query.fcgi?cmd=Retrieve&db=Protein&list_uids=156627569&dopt=GenPept&RID=T03Z2ZXM01S&log$=prottop&blast_rank=21) | solute carrier family 34 (sodium phosphate), member 1 isoform 1 [Homo sapiens] | [20.2](http://blast.ncbi.nlm.nih.gov/Blast.cgi#156627569%23156627569) | 20.2 | 83% | 89 |
| [NP_065699.1](http://www.ncbi.nlm.nih.gov/entrez/query.fcgi?cmd=Retrieve&db=Protein&list_uids=10190664&dopt=GenPept&RID=T03Z2ZXM01S&log$=prottop&blast_rank=22) | twisted gastrulation precursor [Homo sapiens] | [20.2](http://blast.ncbi.nlm.nih.gov/Blast.cgi#10190664%2310190664) | 20.2 | 83% | 89 |
| [NP_004874.1](http://www.ncbi.nlm.nih.gov/entrez/query.fcgi?cmd=Retrieve&db=Protein&list_uids=4758832&dopt=GenPept&RID=T03Z2ZXM01S&log$=prottop&blast_rank=23) | neuregulin 2 isoform 1 [Homo sapiens] | [20.2](http://blast.ncbi.nlm.nih.gov/Blast.cgi#4758832%234758832) | 20.2 | 83% | 89 |
| [NP_689551.1](http://www.ncbi.nlm.nih.gov/entrez/query.fcgi?cmd=Retrieve&db=Protein&list_uids=22748737&dopt=GenPept&RID=T03Z2ZXM01S&log$=prottop&blast_rank=24) | zymogen granule protein 16 homolog precursor [Homo sapiens] | [20.2](http://blast.ncbi.nlm.nih.gov/Blast.cgi#22748737%2322748737) | 20.2 | 83% | 89 |
| [NP_001161086.1](http://www.ncbi.nlm.nih.gov/entrez/query.fcgi?cmd=Retrieve&db=Protein&list_uids=264681410&dopt=GenPept&RID=T03Z2ZXM01S&log$=prottop&blast_rank=25) | LEM domain containing 3 isoform 2 [Homo sapiens] | [19.7](http://blast.ncbi.nlm.nih.gov/Blast.cgi#264681410%23264681410) | 19.7 | 100% | 120 |
| [NP_055134.2](http://www.ncbi.nlm.nih.gov/entrez/query.fcgi?cmd=Retrieve&db=Protein&list_uids=7706607&dopt=GenPept&RID=T03Z2ZXM01S&log$=prottop&blast_rank=26) | LEM domain containing 3 isoform 1 [Homo sapiens] | [19.7](http://blast.ncbi.nlm.nih.gov/Blast.cgi#7706607%237706607) | 19.7 | 100% | 120 |
| [NP_001164058.1](http://www.ncbi.nlm.nih.gov/entrez/query.fcgi?cmd=Retrieve&db=Protein&list_uids=282398133&dopt=GenPept&RID=T03Z2ZXM01S&log$=prottop&blast_rank=27) | hedgehog acyltransferase isoform 4 [Homo sapiens] | [19.3](http://blast.ncbi.nlm.nih.gov/Blast.cgi#282398133%23282398133) | 19.3 | 66% | 161 |
| [XP_002347119.1](http://www.ncbi.nlm.nih.gov/entrez/query.fcgi?cmd=Retrieve&db=Protein&list_uids=239749684&dopt=GenPept&RID=T03Z2ZXM01S&log$=prottop&blast_rank=28) | PREDICTED: hemicentin 2 [Homo sapiens] | [19.3](http://blast.ncbi.nlm.nih.gov/Blast.cgi#239749684%23239749684) | 19.3 | 66% | 161 |
| [NP_001137416.1](http://www.ncbi.nlm.nih.gov/entrez/query.fcgi?cmd=Retrieve&db=Protein&list_uids=221139815&dopt=GenPept&RID=T03Z2ZXM01S&log$=prottop&blast_rank=29) | LEM domain containing 2 isoform 2 [Homo sapiens] | [19.3](http://blast.ncbi.nlm.nih.gov/Blast.cgi#221139815%23221139815) | 19.3 | 66% | 161 |
| [NP_001164059.1](http://www.ncbi.nlm.nih.gov/entrez/query.fcgi?cmd=Retrieve&db=Protein&list_uids=282398139&dopt=GenPept&RID=T03Z2ZXM01S&log$=prottop&blast_rank=30) | hedgehog acyltransferase isoform 3 [Homo sapiens] | [19.3](http://blast.ncbi.nlm.nih.gov/Blast.cgi#282398139%23282398139) | 19.3 | 66% | 161 |
| [NP_001153749.1](http://www.ncbi.nlm.nih.gov/entrez/query.fcgi?cmd=Retrieve&db=Protein&list_uids=237757361&dopt=GenPept&RID=T03Z2ZXM01S&log$=prottop&blast_rank=31) | ceramide kinase-like isoform g [Homo sapiens] | [19.3](http://blast.ncbi.nlm.nih.gov/Blast.cgi#237757361%23237757361) | 19.3 | 66% | 161 |
| [XP_001715206.1](http://www.ncbi.nlm.nih.gov/entrez/query.fcgi?cmd=Retrieve&db=Protein&list_uids=169178458&dopt=GenPept&RID=T03Z2ZXM01S&log$=prottop&blast_rank=32) | PREDICTED: hemicentin 2 [Homo sapiens] | [19.3](http://blast.ncbi.nlm.nih.gov/Blast.cgi#169178458%23169178458) | 19.3 | 66% | 161 |
| [XP_001726994.1](http://www.ncbi.nlm.nih.gov/entrez/query.fcgi?cmd=Retrieve&db=Protein&list_uids=169177000&dopt=GenPept&RID=T03Z2ZXM01S&log$=prottop&blast_rank=33) | PREDICTED: hemicentin 2 [Homo sapiens] | [19.3](http://blast.ncbi.nlm.nih.gov/Blast.cgi#169177000%23169177000) | 19.3 | 66% | 161 |
| [NP_005916.2](http://www.ncbi.nlm.nih.gov/entrez/query.fcgi?cmd=Retrieve&db=Protein&list_uids=153070264&dopt=GenPept&RID=T03Z2ZXM01S&log$=prottop&blast_rank=34) | meprin A beta precursor [Homo sapiens] | [19.3](http://blast.ncbi.nlm.nih.gov/Blast.cgi#153070264%23153070264) | 19.3 | 66% | 161 |
| [NP_001092093.1](http://www.ncbi.nlm.nih.gov/entrez/query.fcgi?cmd=Retrieve&db=Protein&list_uids=148833506&dopt=GenPept&RID=T03Z2ZXM01S&log$=prottop&blast_rank=35) | obscurin, cytoskeletal calmodulin and titin-interacting RhoGEF isoform b [Homo sapiens] | [19.3](http://blast.ncbi.nlm.nih.gov/Blast.cgi#148833506%23148833506) | 51.1 | 66% | 161 |
| [NP_001034679.2](http://www.ncbi.nlm.nih.gov/entrez/query.fcgi?cmd=Retrieve&db=Protein&list_uids=145309309&dopt=GenPept&RID=T03Z2ZXM01S&log$=prottop&blast_rank=36) | ubiquitin specific protease 9, X-linked isoform 3 [Homo sapiens] | [19.3](http://blast.ncbi.nlm.nih.gov/Blast.cgi#145309309%23145309309) | 19.3 | 66% | 161 |
| [NP_056126.1](http://www.ncbi.nlm.nih.gov/entrez/query.fcgi?cmd=Retrieve&db=Protein&list_uids=144226847&dopt=GenPept&RID=T03Z2ZXM01S&log$=prottop&blast_rank=37) | obscurin-like 1 [Homo sapiens] | [19.3](http://blast.ncbi.nlm.nih.gov/Blast.cgi#144226847%23144226847) | 77.2 | 66% | 161 |
| [NP_001034680.2](http://www.ncbi.nlm.nih.gov/entrez/query.fcgi?cmd=Retrieve&db=Protein&list_uids=145309311&dopt=GenPept&RID=T03Z2ZXM01S&log$=prottop&blast_rank=38) | ubiquitin specific protease 9, X-linked isoform 4 [Homo sapiens] | [19.3](http://blast.ncbi.nlm.nih.gov/Blast.cgi#145309311%23145309311) | 19.3 | 66% | 161 |
| [NP_963842.1](http://www.ncbi.nlm.nih.gov/entrez/query.fcgi?cmd=Retrieve&db=Protein&list_uids=41680688&dopt=GenPept&RID=T03Z2ZXM01S&log$=prottop&blast_rank=39) | ceramide kinase-like isoform a [Homo sapiens] | [19.3](http://blast.ncbi.nlm.nih.gov/Blast.cgi#41680688%2341680688) | 19.3 | 66% | 161 |
| [NP_443075.2](http://www.ncbi.nlm.nih.gov/entrez/query.fcgi?cmd=Retrieve&db=Protein&list_uids=58331253&dopt=GenPept&RID=T03Z2ZXM01S&log$=prottop&blast_rank=40) | obscurin, cytoskeletal calmodulin and titin-interacting RhoGEF isoform a [Homo sapiens] | [19.3](http://blast.ncbi.nlm.nih.gov/Blast.cgi#58331253%2358331253) | 51.1 | 66% | 161 |
| [NP_001025483.1](http://www.ncbi.nlm.nih.gov/entrez/query.fcgi?cmd=Retrieve&db=Protein&list_uids=71896725&dopt=GenPept&RID=T03Z2ZXM01S&log$=prottop&blast_rank=41) | ceramide kinase-like isoform c [Homo sapiens] | [19.3](http://blast.ncbi.nlm.nih.gov/Blast.cgi#71896725%2371896725) | 19.3 | 66% | 161 |
| [NP_001025484.1](http://www.ncbi.nlm.nih.gov/entrez/query.fcgi?cmd=Retrieve&db=Protein&list_uids=71896711&dopt=GenPept&RID=T03Z2ZXM01S&log$=prottop&blast_rank=42) | ceramide kinase-like isoform d [Homo sapiens] | [19.3](http://blast.ncbi.nlm.nih.gov/Blast.cgi#71896711%2371896711) | 19.3 | 66% | 161 |
| [NP_003377.1](http://www.ncbi.nlm.nih.gov/entrez/query.fcgi?cmd=Retrieve&db=Protein&list_uids=16554449&dopt=GenPept&RID=T03Z2ZXM01S&log$=prottop&blast_rank=43) | zonadhesin isoform 3 [Homo sapiens] | [19.3](http://blast.ncbi.nlm.nih.gov/Blast.cgi#16554449%2316554449) | 19.3 | 66% | 161 |
| [NP_775082.1](http://www.ncbi.nlm.nih.gov/entrez/query.fcgi?cmd=Retrieve&db=Protein&list_uids=27881494&dopt=GenPept&RID=T03Z2ZXM01S&log$=prottop&blast_rank=44) | zonadhesin isoform 6 [Homo sapiens] | [19.3](http://blast.ncbi.nlm.nih.gov/Blast.cgi#27881494%2327881494) | 19.3 | 66% | 161 |
| [NP_775834.1](http://www.ncbi.nlm.nih.gov/entrez/query.fcgi?cmd=Retrieve&db=Protein&list_uids=27734861&dopt=GenPept&RID=T03Z2ZXM01S&log$=prottop&blast_rank=45) | hypothetical protein LOC222826 [Homo sapiens] | [19.3](http://blast.ncbi.nlm.nih.gov/Blast.cgi#27734861%2327734861) | 19.3 | 66% | 161 |
| [NP_001025482.1](http://www.ncbi.nlm.nih.gov/entrez/query.fcgi?cmd=Retrieve&db=Protein&list_uids=71896723&dopt=GenPept&RID=T03Z2ZXM01S&log$=prottop&blast_rank=46) | ceramide kinase-like isoform b [Homo sapiens] | [19.3](http://blast.ncbi.nlm.nih.gov/Blast.cgi#71896723%2371896723) | 19.3 | 66% | 161 |
| [NP_009044.2](http://www.ncbi.nlm.nih.gov/entrez/query.fcgi?cmd=Retrieve&db=Protein&list_uids=148746195&dopt=GenPept&RID=T03Z2ZXM01S&log$=prottop&blast_rank=47) | trichohyalin [Homo sapiens] | [19.3](http://blast.ncbi.nlm.nih.gov/Blast.cgi#148746195%23148746195) | 19.3 | 66% | 161 |
| [NP_060302.1](http://www.ncbi.nlm.nih.gov/entrez/query.fcgi?cmd=Retrieve&db=Protein&list_uids=8923431&dopt=GenPept&RID=T03Z2ZXM01S&log$=prottop&blast_rank=48) | hypothetical protein LOC54942 [Homo sapiens] | [19.3](http://blast.ncbi.nlm.nih.gov/Blast.cgi#8923431%238923431) | 19.3 | 66% | 161 |
| [NP_002962.1](http://www.ncbi.nlm.nih.gov/entrez/query.fcgi?cmd=Retrieve&db=Protein&list_uids=4506791&dopt=GenPept&RID=T03Z2ZXM01S&log$=prottop&blast_rank=49) | special AT-rich sequence binding protein 1 [Homo sapiens] >ref|NP_001124482.1| special AT-rich sequence binding protein 1 [Homo sapiens] | [19.3](http://blast.ncbi.nlm.nih.gov/Blast.cgi#4506791%234506791) | 19.3 | 66% | 161 |
| [NP_689896.1](http://www.ncbi.nlm.nih.gov/entrez/query.fcgi?cmd=Retrieve&db=Protein&list_uids=22749373&dopt=GenPept&RID=T03Z2ZXM01S&log$=prottop&blast_rank=50) | coiled-coil domain containing 111 [Homo sapiens] | [19.3](http://blast.ncbi.nlm.nih.gov/Blast.cgi#22749373%2322749373) | 19.3 | 66% | 161 |
| [NP_060664.2](http://www.ncbi.nlm.nih.gov/entrez/query.fcgi?cmd=Retrieve&db=Protein&list_uids=170784861&dopt=GenPept&RID=T03Z2ZXM01S&log$=prottop&blast_rank=51) | hedgehog acyltransferase isoform 1 [Homo sapiens] >ref|NP_001116306.1| hedgehog acyltransferase isoform 1 [Homo sapiens] >ref|NP_001164051.1| hedgehog acyltransferase isoform 1 [Homo sapiens] | [19.3](http://blast.ncbi.nlm.nih.gov/Blast.cgi#170784861%23170784861) | 19.3 | 66% | 161 |
| [NP_055410.1](http://www.ncbi.nlm.nih.gov/entrez/query.fcgi?cmd=Retrieve&db=Protein&list_uids=7657033&dopt=GenPept&RID=T03Z2ZXM01S&log$=prottop&blast_rank=52) | 5',3'-nucleotidase, cytosolic [Homo sapiens] | [19.3](http://blast.ncbi.nlm.nih.gov/Blast.cgi#7657033%237657033) | 19.3 | 66% | 161 |
| [NP_056080.1](http://www.ncbi.nlm.nih.gov/entrez/query.fcgi?cmd=Retrieve&db=Protein&list_uids=38016202&dopt=GenPept&RID=T03Z2ZXM01S&log$=prottop&blast_rank=53) | SATB homeobox 2 [Homo sapiens] >ref|NP_001165980.1| SATB homeobox 2 [Homo sapiens] >ref|NP_001165988.1| SATB homeobox 2 [Homo sapiens] | [19.3](http://blast.ncbi.nlm.nih.gov/Blast.cgi#38016202%2338016202) | 19.3 | 66% | 161 |
| [NP_851853.1](http://www.ncbi.nlm.nih.gov/entrez/query.fcgi?cmd=Retrieve&db=Protein&list_uids=31044432&dopt=GenPept&RID=T03Z2ZXM01S&log$=prottop&blast_rank=54) | LEM domain containing 2 isoform 1 [Homo sapiens] | [19.3](http://blast.ncbi.nlm.nih.gov/Blast.cgi#31044432%2331044432) | 19.3 | 66% | 161 |
| [NP_003365.1](http://www.ncbi.nlm.nih.gov/entrez/query.fcgi?cmd=Retrieve&db=Protein&list_uids=4507879&dopt=GenPept&RID=T03Z2ZXM01S&log$=prottop&blast_rank=55) | voltage-dependent anion channel 1 [Homo sapiens] | [19.3](http://blast.ncbi.nlm.nih.gov/Blast.cgi#4507879%234507879) | 19.3 | 66% | 161 |
| [XP_002343922.1](http://www.ncbi.nlm.nih.gov/entrez/query.fcgi?cmd=Retrieve&db=Protein&list_uids=239747139&dopt=GenPept&RID=T03Z2ZXM01S&log$=prottop&blast_rank=56) | PREDICTED: hypothetical protein XP_002343922 [Homo sapiens] | [17.2](http://blast.ncbi.nlm.nih.gov/Blast.cgi#239747139%23239747139) | 17.2 | 83% | 699 |
| [XP_001727028.2](http://www.ncbi.nlm.nih.gov/entrez/query.fcgi?cmd=Retrieve&db=Protein&list_uids=239745068&dopt=GenPept&RID=T03Z2ZXM01S&log$=prottop&blast_rank=57) | PREDICTED: similar to hCG2042704 [Homo sapiens] | [17.2](http://blast.ncbi.nlm.nih.gov/Blast.cgi#239745068%23239745068) | 17.2 | 66% | 699 |
| [XP_001716256.1](http://www.ncbi.nlm.nih.gov/entrez/query.fcgi?cmd=Retrieve&db=Protein&list_uids=169209217&dopt=GenPept&RID=T03Z2ZXM01S&log$=prottop&blast_rank=58) | PREDICTED: similar to hCG2042704 [Homo sapiens] | [17.2](http://blast.ncbi.nlm.nih.gov/Blast.cgi#169209217%23169209217) | 17.2 | 66% | 699 |
| [NP_001164376.1](http://www.ncbi.nlm.nih.gov/entrez/query.fcgi?cmd=Retrieve&db=Protein&list_uids=283135246&dopt=GenPept&RID=T03Z2ZXM01S&log$=prottop&blast_rank=59) | KRAB-containing zinc-finger repressor protein [Homo sapiens] | [17.2](http://blast.ncbi.nlm.nih.gov/Blast.cgi#283135246%23283135246) | 17.2 | 66% | 699 |
| [NP_001091968.1](http://www.ncbi.nlm.nih.gov/entrez/query.fcgi?cmd=Retrieve&db=Protein&list_uids=148612875&dopt=GenPept&RID=T03Z2ZXM01S&log$=prottop&blast_rank=60) | RUN and TBC1 domain containing 2 isoform 4 [Homo sapiens] | [17.2](http://blast.ncbi.nlm.nih.gov/Blast.cgi#148612875%23148612875) | 17.2 | 66% | 699 |
| [NP_055668.2](http://www.ncbi.nlm.nih.gov/entrez/query.fcgi?cmd=Retrieve&db=Protein&list_uids=148612795&dopt=GenPept&RID=T03Z2ZXM01S&log$=prottop&blast_rank=61) | RUN and TBC1 domain containing 1 isoform 1 [Homo sapiens] | [17.2](http://blast.ncbi.nlm.nih.gov/Blast.cgi#148612795%23148612795) | 17.2 | 66% | 699 |
| [NP_001091967.1](http://www.ncbi.nlm.nih.gov/entrez/query.fcgi?cmd=Retrieve&db=Protein&list_uids=148612840&dopt=GenPept&RID=T03Z2ZXM01S&log$=prottop&blast_rank=62) | RUN and TBC1 domain containing 2 isoform 3 [Homo sapiens] | [17.2](http://blast.ncbi.nlm.nih.gov/Blast.cgi#148612840%23148612840) | 17.2 | 66% | 699 |
| [NP_001091979.1](http://www.ncbi.nlm.nih.gov/entrez/query.fcgi?cmd=Retrieve&db=Protein&list_uids=148612829&dopt=GenPept&RID=T03Z2ZXM01S&log$=prottop&blast_rank=63) | RUN and TBC1 domain containing 1 isoform 2 [Homo sapiens] | [17.2](http://blast.ncbi.nlm.nih.gov/Blast.cgi#148612829%23148612829) | 17.2 | 66% | 699 |
| [NP_689805.3](http://www.ncbi.nlm.nih.gov/entrez/query.fcgi?cmd=Retrieve&db=Protein&list_uids=145580592&dopt=GenPept&RID=T03Z2ZXM01S&log$=prottop&blast_rank=64) | nesprin-3 [Homo sapiens] | [17.2](http://blast.ncbi.nlm.nih.gov/Blast.cgi#145580592%23145580592) | 17.2 | 66% | 699 |
| [NP_001120837.1](http://www.ncbi.nlm.nih.gov/entrez/query.fcgi?cmd=Retrieve&db=Protein&list_uids=188219622&dopt=GenPept&RID=T03Z2ZXM01S&log$=prottop&blast_rank=65) | hypothetical protein LOC340277 isoform 3 [Homo sapiens] | [17.2](http://blast.ncbi.nlm.nih.gov/Blast.cgi#188219622%23188219622) | 17.2 | 83% | 699 |
| [NP_001007278.1](http://www.ncbi.nlm.nih.gov/entrez/query.fcgi?cmd=Retrieve&db=Protein&list_uids=55956768&dopt=GenPept&RID=T03Z2ZXM01S&log$=prottop&blast_rank=66) | etoposide induced 2.4 isoform 2 [Homo sapiens] | [17.2](http://blast.ncbi.nlm.nih.gov/Blast.cgi#55956768%2355956768) | 17.2 | 66% | 699 |
| [NP_001074930.1](http://www.ncbi.nlm.nih.gov/entrez/query.fcgi?cmd=Retrieve&db=Protein&list_uids=125988395&dopt=GenPept&RID=T03Z2ZXM01S&log$=prottop&blast_rank=67) | jumonji domain containing 6 isoform 1 [Homo sapiens] | [17.2](http://blast.ncbi.nlm.nih.gov/Blast.cgi#125988395%23125988395) | 17.2 | 66% | 699 |
| [NP_597711.1](http://www.ncbi.nlm.nih.gov/entrez/query.fcgi?cmd=Retrieve&db=Protein&list_uids=90577164&dopt=GenPept&RID=T03Z2ZXM01S&log$=prottop&blast_rank=68) | RUN and TBC1 domain containing 2 isoform 2 [Homo sapiens] | [17.2](http://blast.ncbi.nlm.nih.gov/Blast.cgi#90577164%2390577164) | 17.2 | 66% | 699 |
| [NP_001035037.1](http://www.ncbi.nlm.nih.gov/entrez/query.fcgi?cmd=Retrieve&db=Protein&list_uids=90577167&dopt=GenPept&RID=T03Z2ZXM01S&log$=prottop&blast_rank=69) | RUN and TBC1 domain containing 2 isoform 1 [Homo sapiens] | [17.2](http://blast.ncbi.nlm.nih.gov/Blast.cgi#90577167%2390577167) | 17.2 | 66% | 699 |
| [XP_001719523.1](http://www.ncbi.nlm.nih.gov/entrez/query.fcgi?cmd=Retrieve&db=Protein&list_uids=169218206&dopt=GenPept&RID=T03Z2ZXM01S&log$=prottop&blast_rank=70) | PREDICTED: similar to hCG2042704 [Homo sapiens] | [17.2](http://blast.ncbi.nlm.nih.gov/Blast.cgi#169218206%23169218206) | 17.2 | 66% | 699 |
| [NP_004870.3](http://www.ncbi.nlm.nih.gov/entrez/query.fcgi?cmd=Retrieve&db=Protein&list_uids=55956766&dopt=GenPept&RID=T03Z2ZXM01S&log$=prottop&blast_rank=71) | etoposide induced 2.4 isoform 1 [Homo sapiens] | [17.2](http://blast.ncbi.nlm.nih.gov/Blast.cgi#55956766%2355956766) | 17.2 | 66% | 699 |
| [NP_000086.2](http://www.ncbi.nlm.nih.gov/entrez/query.fcgi?cmd=Retrieve&db=Protein&list_uids=40217843&dopt=GenPept&RID=T03Z2ZXM01S&log$=prottop&blast_rank=72) | cartilage oligomeric matrix protein precursor [Homo sapiens] | [17.2](http://blast.ncbi.nlm.nih.gov/Blast.cgi#40217843%2340217843) | 17.2 | 66% | 699 |
| [NP_683707.1](http://www.ncbi.nlm.nih.gov/entrez/query.fcgi?cmd=Retrieve&db=Protein&list_uids=22547176&dopt=GenPept&RID=T03Z2ZXM01S&log$=prottop&blast_rank=73) | oxysterol binding protein-like 9 isoform f [Homo sapiens] | [17.2](http://blast.ncbi.nlm.nih.gov/Blast.cgi#22547176%2322547176) | 17.2 | 66% | 699 |
| [NP_065988.1](http://www.ncbi.nlm.nih.gov/entrez/query.fcgi?cmd=Retrieve&db=Protein&list_uids=74959747&dopt=GenPept&RID=T03Z2ZXM01S&log$=prottop&blast_rank=74) | Fanconi anemia, complementation group M [Homo sapiens] | [17.2](http://blast.ncbi.nlm.nih.gov/Blast.cgi#74959747%2374959747) | 17.2 | 66% | 699 |
| [NP_003239.2](http://www.ncbi.nlm.nih.gov/entrez/query.fcgi?cmd=Retrieve&db=Protein&list_uids=31543806&dopt=GenPept&RID=T03Z2ZXM01S&log$=prottop&blast_rank=75) | thrombospondin 4 precursor [Homo sapiens] | [17.2](http://blast.ncbi.nlm.nih.gov/Blast.cgi#31543806%2331543806) | 17.2 | 66% | 699 |
| [NP_683706.1](http://www.ncbi.nlm.nih.gov/entrez/query.fcgi?cmd=Retrieve&db=Protein&list_uids=22547173&dopt=GenPept&RID=T03Z2ZXM01S&log$=prottop&blast_rank=76) | oxysterol binding protein-like 9 isoform d [Homo sapiens] | [17.2](http://blast.ncbi.nlm.nih.gov/Blast.cgi#22547173%2322547173) | 17.2 | 66% | 699 |
| [NP_004981.2](http://www.ncbi.nlm.nih.gov/entrez/query.fcgi?cmd=Retrieve&db=Protein&list_uids=14043022&dopt=GenPept&RID=T03Z2ZXM01S&log$=prottop&blast_rank=77) | methionyl-tRNA synthetase [Homo sapiens] | [17.2](http://blast.ncbi.nlm.nih.gov/Blast.cgi#14043022%2314043022) | 17.2 | 66% | 699 |
| [NP_056009.1](http://www.ncbi.nlm.nih.gov/entrez/query.fcgi?cmd=Retrieve&db=Protein&list_uids=51100974&dopt=GenPept&RID=T03Z2ZXM01S&log$=prottop&blast_rank=78) | myosin ID [Homo sapiens] | [17.2](http://blast.ncbi.nlm.nih.gov/Blast.cgi#51100974%2351100974) | 17.2 | 83% | 699 |
| [NP_055982.2](http://www.ncbi.nlm.nih.gov/entrez/query.fcgi?cmd=Retrieve&db=Protein&list_uids=125988389&dopt=GenPept&RID=T03Z2ZXM01S&log$=prottop&blast_rank=79) | jumonji domain containing 6 isoform 2 [Homo sapiens] | [17.2](http://blast.ncbi.nlm.nih.gov/Blast.cgi#125988389%23125988389) | 17.2 | 66% | 699 |
| [NP_078862.2](http://www.ncbi.nlm.nih.gov/entrez/query.fcgi?cmd=Retrieve&db=Protein&list_uids=20070331&dopt=GenPept&RID=T03Z2ZXM01S&log$=prottop&blast_rank=80) | oxysterol binding protein-like 9 isoform e [Homo sapiens] | [17.2](http://blast.ncbi.nlm.nih.gov/Blast.cgi#20070331%2320070331) | 17.2 | 66% | 699 |
| [NP_695003.1](http://www.ncbi.nlm.nih.gov/entrez/query.fcgi?cmd=Retrieve&db=Protein&list_uids=23397574&dopt=GenPept&RID=T03Z2ZXM01S&log$=prottop&blast_rank=81) | sorting nexin 33 [Homo sapiens] | [17.2](http://blast.ncbi.nlm.nih.gov/Blast.cgi#23397574%2323397574) | 17.2 | 83% | 699 |
| [NP_056210.1](http://www.ncbi.nlm.nih.gov/entrez/query.fcgi?cmd=Retrieve&db=Protein&list_uids=32698704&dopt=GenPept&RID=T03Z2ZXM01S&log$=prottop&blast_rank=82) | tectonin beta-propeller repeat containing 1 [Homo sapiens] | [17.2](http://blast.ncbi.nlm.nih.gov/Blast.cgi#32698704%2332698704) | 17.2 | 83% | 699 |
| [NP_003604.3](http://www.ncbi.nlm.nih.gov/entrez/query.fcgi?cmd=Retrieve&db=Protein&list_uids=192449445&dopt=GenPept&RID=T03Z2ZXM01S&log$=prottop&blast_rank=83) | cartilage intermediate layer protein [Homo sapiens] | [16.3](http://blast.ncbi.nlm.nih.gov/Blast.cgi#192449445%23192449445) | 16.3 | 83% | 1259 |
| [NP_001038187.1](http://www.ncbi.nlm.nih.gov/entrez/query.fcgi?cmd=Retrieve&db=Protein&list_uids=113462008&dopt=GenPept&RID=T03Z2ZXM01S&log$=prottop&blast_rank=84) | casein kinase 1, gamma 3 isoform 3 [Homo sapiens] | [16.3](http://blast.ncbi.nlm.nih.gov/Blast.cgi#113462008%23113462008) | 16.3 | 83% | 1259 |
| [NP_001038188.1](http://www.ncbi.nlm.nih.gov/entrez/query.fcgi?cmd=Retrieve&db=Protein&list_uids=113462010&dopt=GenPept&RID=T03Z2ZXM01S&log$=prottop&blast_rank=85) | casein kinase 1, gamma 3 isoform 4 [Homo sapiens] | [16.3](http://blast.ncbi.nlm.nih.gov/Blast.cgi#113462010%23113462010) | 16.3 | 83% | 1259 |
| [NP_001026982.1](http://www.ncbi.nlm.nih.gov/entrez/query.fcgi?cmd=Retrieve&db=Protein&list_uids=73532780&dopt=GenPept&RID=T03Z2ZXM01S&log$=prottop&blast_rank=86) | casein kinase 1, gamma 3 isoform 2 [Homo sapiens] | [16.3](http://blast.ncbi.nlm.nih.gov/Blast.cgi#73532780%2373532780) | 16.3 | 83% | 1259 |
| [NP_001310.3](http://www.ncbi.nlm.nih.gov/entrez/query.fcgi?cmd=Retrieve&db=Protein&list_uids=153791733&dopt=GenPept&RID=T03Z2ZXM01S&log$=prottop&blast_rank=87) | casein kinase 1, gamma 2 [Homo sapiens] | [16.3](http://blast.ncbi.nlm.nih.gov/Blast.cgi#153791733%23153791733) | 16.3 | 83% | 1259 |
| [NP_004375.2](http://www.ncbi.nlm.nih.gov/entrez/query.fcgi?cmd=Retrieve&db=Protein&list_uids=73532778&dopt=GenPept&RID=T03Z2ZXM01S&log$=prottop&blast_rank=88) | casein kinase 1, gamma 3 isoform 1 [Homo sapiens] | [16.3](http://blast.ncbi.nlm.nih.gov/Blast.cgi#73532778%2373532778) | 16.3 | 83% | 1259 |
| [NP_071331.2](http://www.ncbi.nlm.nih.gov/entrez/query.fcgi?cmd=Retrieve&db=Protein&list_uids=98986450&dopt=GenPept&RID=T03Z2ZXM01S&log$=prottop&blast_rank=89) | casein kinase 1, gamma 1 [Homo sapiens] | [16.3](http://blast.ncbi.nlm.nih.gov/Blast.cgi#98986450%2398986450) | 16.3 | 83% | 1259 |
| [NP_001002814.2](http://www.ncbi.nlm.nih.gov/entrez/query.fcgi?cmd=Retrieve&db=Protein&list_uids=289547524&dopt=GenPept&RID=T03Z2ZXM01S&log$=prottop&blast_rank=90) | RAB11 family interacting protein 1 isoform 3 [Homo sapiens] | [15.9](http://blast.ncbi.nlm.nih.gov/Blast.cgi#289547524%23289547524) | 15.9 | 50% | 1689 |
| [NP_001161881.1](http://www.ncbi.nlm.nih.gov/entrez/query.fcgi?cmd=Retrieve&db=Protein&list_uids=270288802&dopt=GenPept&RID=T03Z2ZXM01S&log$=prottop&blast_rank=91) | regulating synaptic membrane exocytosis 1 isoform 4 [Homo sapiens] | [15.9](http://blast.ncbi.nlm.nih.gov/Blast.cgi#270288802%23270288802) | 15.9 | 50% | 1689 |
| [NP_001161880.1](http://www.ncbi.nlm.nih.gov/entrez/query.fcgi?cmd=Retrieve&db=Protein&list_uids=270288800&dopt=GenPept&RID=T03Z2ZXM01S&log$=prottop&blast_rank=92) | regulating synaptic membrane exocytosis 1 isoform 3 [Homo sapiens] | [15.9](http://blast.ncbi.nlm.nih.gov/Blast.cgi#270288800%23270288800) | 15.9 | 50% | 1689 |
| [NP_001161879.1](http://www.ncbi.nlm.nih.gov/entrez/query.fcgi?cmd=Retrieve&db=Protein&list_uids=270288798&dopt=GenPept&RID=T03Z2ZXM01S&log$=prottop&blast_rank=93) | regulating synaptic membrane exocytosis 1 isoform 2 [Homo sapiens] | [15.9](http://blast.ncbi.nlm.nih.gov/Blast.cgi#270288798%23270288798) | 15.9 | 50% | 1689 |
| [XP_002346285.1](http://www.ncbi.nlm.nih.gov/entrez/query.fcgi?cmd=Retrieve&db=Protein&list_uids=239758075&dopt=GenPept&RID=T03Z2ZXM01S&log$=prottop&blast_rank=94) | PREDICTED: similar to deubiquitinating enzyme DUB1 [Homo sapiens] | [15.9](http://blast.ncbi.nlm.nih.gov/Blast.cgi#239758075%23239758075) | 15.9 | 50% | 1689 |
| [XP_001727080.2](http://www.ncbi.nlm.nih.gov/entrez/query.fcgi?cmd=Retrieve&db=Protein&list_uids=239758053&dopt=GenPept&RID=T03Z2ZXM01S&log$=prottop&blast_rank=95) | PREDICTED: Inactive ubiquitin carboxyl-terminal hydrolase 17-like protein 8, partial [Homo sapiens] | [15.9](http://blast.ncbi.nlm.nih.gov/Blast.cgi#239758053%23239758053) | 15.9 | 50% | 1689 |
| [XP_002344831.1](http://www.ncbi.nlm.nih.gov/entrez/query.fcgi?cmd=Retrieve&db=Protein&list_uids=239756165&dopt=GenPept&RID=T03Z2ZXM01S&log$=prottop&blast_rank=96) | PREDICTED: hypothetical protein [Homo sapiens] | [15.9](http://blast.ncbi.nlm.nih.gov/Blast.cgi#239756165%23239756165) | 15.9 | 50% | 1689 |
| [XP_002347504.1](http://www.ncbi.nlm.nih.gov/entrez/query.fcgi?cmd=Retrieve&db=Protein&list_uids=239750681&dopt=GenPept&RID=T03Z2ZXM01S&log$=prottop&blast_rank=97) | PREDICTED: hypothetical protein XP_002347504 [Homo sapiens] | [15.9](http://blast.ncbi.nlm.nih.gov/Blast.cgi#239750681%23239750681) | 15.9 | 50% | 1689 |
| [XP_002343323.1](http://www.ncbi.nlm.nih.gov/entrez/query.fcgi?cmd=Retrieve&db=Protein&list_uids=239744991&dopt=GenPept&RID=T03Z2ZXM01S&log$=prottop&blast_rank=98) | PREDICTED: hypothetical protein XP_002343323 [Homo sapiens] | [15.9](http://blast.ncbi.nlm.nih.gov/Blast.cgi#239744991%23239744991) | 15.9 | 50% | 1689 |
| [XP_002342479.1](http://www.ncbi.nlm.nih.gov/entrez/query.fcgi?cmd=Retrieve&db=Protein&list_uids=239742295&dopt=GenPept&RID=T03Z2ZXM01S&log$=prottop&blast_rank=99) | PREDICTED: hypothetical protein XP_002342479 [Homo sapiens] | [15.9](http://blast.ncbi.nlm.nih.gov/Blast.cgi#239742295%23239742295) | 15.9 | 50% | 1689 |
| [NP_001161882.1](http://www.ncbi.nlm.nih.gov/entrez/query.fcgi?cmd=Retrieve&db=Protein&list_uids=270288804&dopt=GenPept&RID=T03Z2ZXM01S&log$=prottop&blast_rank=100) | regulating synaptic membrane exocytosis 1 isoform 5 [Homo sapiens] | [15.9](http://blast.ncbi.nlm.nih.gov/Blast.cgi#270288804%23270288804) | 15.9 | 50% | 1689 |
