# Supplementary material for: A high throughput method for identifying personalized tumor-associated antigens
Source: Oncotarget. 2010 Jun 27;1(2):148–55. doi: 10.18632/oncotarget.118 (PMC2920534; doi:10.18632/oncotarget.118)
Supplement: Supplemental Table 2 [file oncotarget-01-148-s002.doc]

**IgM Patient A**

| **Accession** | **Proteins with a match to AVHFPD peptide** | **[Max score](http://blast.ncbi.nlm.nih.gov/Blast.cgi?CMD=Get&ALIGNMENTS=100&ALIGNMENT_VIEW=Pairwise&CDD_SEARCH_STATE=1&DATABASE_SORT=0&DESCRIPTIONS=100&ENTREZ_QUERY=txid9606 %5BORGN%5D&FIRST_QUERY_NUM=0&FORMAT_OBJECT=Alignment&FORMAT_PAGE_TARGET=&FORMAT_TYPE=HTML&GET_SEQUENCE=yes&I_THRESH=&MASK_CHAR=2&MASK_COLOR=1&NEW_DESIGN=on&NEW_VIEW=yes&NUM_OVERVIEW=100&OLD_BLAST=false&PAGE=Proteins&QUERY_INDEX=0&QUERY_NUMBER=0&RESULTS_PAGE_TARGET=&RID=T04SGPYZ01N&SHOW_LINKOUT=yes&SHOW_OVERVIEW=yes&STEP_NUMBER=&WORD_SIZE=2&DISPLAY_SORT=1&HSP_SORT=1" \l "sort_mark)** | **[Total score](http://blast.ncbi.nlm.nih.gov/Blast.cgi?CMD=Get&ALIGNMENTS=100&ALIGNMENT_VIEW=Pairwise&CDD_SEARCH_STATE=1&DATABASE_SORT=0&DESCRIPTIONS=100&ENTREZ_QUERY=txid9606 %5BORGN%5D&FIRST_QUERY_NUM=0&FORMAT_OBJECT=Alignment&FORMAT_PAGE_TARGET=&FORMAT_TYPE=HTML&GET_SEQUENCE=yes&I_THRESH=&MASK_CHAR=2&MASK_COLOR=1&NEW_DESIGN=on&NEW_VIEW=yes&NUM_OVERVIEW=100&OLD_BLAST=false&PAGE=Proteins&QUERY_INDEX=0&QUERY_NUMBER=0&RESULTS_PAGE_TARGET=&RID=T04SGPYZ01N&SHOW_LINKOUT=yes&SHOW_OVERVIEW=yes&STEP_NUMBER=&WORD_SIZE=2&DISPLAY_SORT=2&HSP_SORT=1" \l "sort_mark)** | **[Query coverage](http://blast.ncbi.nlm.nih.gov/Blast.cgi?CMD=Get&ALIGNMENTS=100&ALIGNMENT_VIEW=Pairwise&CDD_SEARCH_STATE=1&DATABASE_SORT=0&DESCRIPTIONS=100&ENTREZ_QUERY=txid9606 %5BORGN%5D&FIRST_QUERY_NUM=0&FORMAT_OBJECT=Alignment&FORMAT_PAGE_TARGET=&FORMAT_TYPE=HTML&GET_SEQUENCE=yes&I_THRESH=&MASK_CHAR=2&MASK_COLOR=1&NEW_DESIGN=on&NEW_VIEW=yes&NUM_OVERVIEW=100&OLD_BLAST=false&PAGE=Proteins&QUERY_INDEX=0&QUERY_NUMBER=0&RESULTS_PAGE_TARGET=&RID=T04SGPYZ01N&SHOW_LINKOUT=yes&SHOW_OVERVIEW=yes&STEP_NUMBER=&WORD_SIZE=2&DISPLAY_SORT=4&HSP_SORT=0" \l "sort_mark)** | **[E value](http://blast.ncbi.nlm.nih.gov/Blast.cgi?CMD=Get&ALIGNMENTS=100&ALIGNMENT_VIEW=Pairwise&CDD_SEARCH_STATE=1&DATABASE_SORT=0&DESCRIPTIONS=100&ENTREZ_QUERY=txid9606 %5BORGN%5D&FIRST_QUERY_NUM=0&FORMAT_OBJECT=Alignment&FORMAT_PAGE_TARGET=&FORMAT_TYPE=HTML&GET_SEQUENCE=yes&I_THRESH=&MASK_CHAR=2&MASK_COLOR=1&NEW_DESIGN=on&NEW_VIEW=yes&NUM_OVERVIEW=100&OLD_BLAST=false&PAGE=Proteins&QUERY_INDEX=0&QUERY_NUMBER=0&RESULTS_PAGE_TARGET=&RID=T04SGPYZ01N&SHOW_LINKOUT=yes&SHOW_OVERVIEW=yes&STEP_NUMBER=&WORD_SIZE=2&DISPLAY_SORT=0&HSP_SORT=0" \l "sort_mark)** |
| --- | --- | --- | --- | --- | --- |
| [NP_940980.3](http://www.ncbi.nlm.nih.gov/entrez/query.fcgi?cmd=Retrieve&db=Protein&list_uids=171846278&dopt=GenPept&RID=T04SGPYZ01N&log$=prottop&blast_rank=1) | leucine-rich repeat kinase 2 [Homo sapiens] | [20.6](http://blast.ncbi.nlm.nih.gov/Blast.cgi#171846278%23171846278) | 36.9 | 100% | 67 |
| [NP_542409.1](http://www.ncbi.nlm.nih.gov/entrez/query.fcgi?cmd=Retrieve&db=Protein&list_uids=18087857&dopt=GenPept&RID=T04SGPYZ01N&log$=prottop&blast_rank=2) | NEDD8-conjugating enzyme [Homo sapiens] | [20.6](http://blast.ncbi.nlm.nih.gov/Blast.cgi#18087857%2318087857) | 20.6 | 83% | 67 |
| [NP_065779.1](http://www.ncbi.nlm.nih.gov/entrez/query.fcgi?cmd=Retrieve&db=Protein&list_uids=45387945&dopt=GenPept&RID=T04SGPYZ01N&log$=prottop&blast_rank=3) | family with sequence similarity 62 (C2 domain containing) member B [Homo sapiens] | [20.6](http://blast.ncbi.nlm.nih.gov/Blast.cgi#45387945%2345387945) | 20.6 | 83% | 67 |
| [NP_055907.3](http://www.ncbi.nlm.nih.gov/entrez/query.fcgi?cmd=Retrieve&db=Protein&list_uids=62243658&dopt=GenPept&RID=T04SGPYZ01N&log$=prottop&blast_rank=4) | PI-3-kinase-related kinase SMG-1 [Homo sapiens] | [19.7](http://blast.ncbi.nlm.nih.gov/Blast.cgi#62243658%2362243658) | 19.7 | 83% | 120 |
| [NP_848552.1](http://www.ncbi.nlm.nih.gov/entrez/query.fcgi?cmd=Retrieve&db=Protein&list_uids=123701326&dopt=GenPept&RID=T04SGPYZ01N&log$=prottop&blast_rank=5) | zinc finger protein 831 [Homo sapiens] | [19.7](http://blast.ncbi.nlm.nih.gov/Blast.cgi#123701326%23123701326) | 19.7 | 83% | 120 |
| [NP_001058.2](http://www.ncbi.nlm.nih.gov/entrez/query.fcgi?cmd=Retrieve&db=Protein&list_uids=19913406&dopt=GenPept&RID=T04SGPYZ01N&log$=prottop&blast_rank=6) | DNA topoisomerase II, alpha isozyme [Homo sapiens] | [18.9](http://blast.ncbi.nlm.nih.gov/Blast.cgi#19913406%2319913406) | 18.9 | 100% | 216 |
| [NP_001093882.1](http://www.ncbi.nlm.nih.gov/entrez/query.fcgi?cmd=Retrieve&db=Protein&list_uids=155030236&dopt=GenPept&RID=T04SGPYZ01N&log$=prottop&blast_rank=7) | ribonuclease III, nuclear isoform 2 [Homo sapiens] | [18.5](http://blast.ncbi.nlm.nih.gov/Blast.cgi#155030236%23155030236) | 18.5 | 83% | 289 |
| [NP_001073981.1](http://www.ncbi.nlm.nih.gov/entrez/query.fcgi?cmd=Retrieve&db=Protein&list_uids=122937472&dopt=GenPept&RID=T04SGPYZ01N&log$=prottop&blast_rank=8) | bicaudal C homolog 1 [Homo sapiens] | [18.5](http://blast.ncbi.nlm.nih.gov/Blast.cgi#122937472%23122937472) | 36.9 | 83% | 289 |
| [NP_114119.2](http://www.ncbi.nlm.nih.gov/entrez/query.fcgi?cmd=Retrieve&db=Protein&list_uids=103472031&dopt=GenPept&RID=T04SGPYZ01N&log$=prottop&blast_rank=9) | family with sequence similarity 62 (C2 domain containing), member C [Homo sapiens] | [18.5](http://blast.ncbi.nlm.nih.gov/Blast.cgi#103472031%23103472031) | 18.5 | 83% | 289 |
| [NP_071348.3](http://www.ncbi.nlm.nih.gov/entrez/query.fcgi?cmd=Retrieve&db=Protein&list_uids=38348727&dopt=GenPept&RID=T04SGPYZ01N&log$=prottop&blast_rank=10) | thyroid adenoma associated [Homo sapiens] >ref|NP_001077422.1| thyroid adenoma associated [Homo sapiens] | [18.5](http://blast.ncbi.nlm.nih.gov/Blast.cgi#38348727%2338348727) | 18.5 | 100% | 289 |
| [NP_694536.1](http://www.ncbi.nlm.nih.gov/entrez/query.fcgi?cmd=Retrieve&db=Protein&list_uids=24041023&dopt=GenPept&RID=T04SGPYZ01N&log$=prottop&blast_rank=11) | embryonic ectoderm development isoform b [Homo sapiens] | [18.5](http://blast.ncbi.nlm.nih.gov/Blast.cgi#24041023%2324041023) | 18.5 | 83% | 289 |
| [NP_008932.2](http://www.ncbi.nlm.nih.gov/entrez/query.fcgi?cmd=Retrieve&db=Protein&list_uids=223029426&dopt=GenPept&RID=T04SGPYZ01N&log$=prottop&blast_rank=12) | solute carrier family 35, member D2 [Homo sapiens] | [18.5](http://blast.ncbi.nlm.nih.gov/Blast.cgi#223029426%23223029426) | 18.5 | 83% | 289 |
| [NP_037367.3](http://www.ncbi.nlm.nih.gov/entrez/query.fcgi?cmd=Retrieve&db=Protein&list_uids=155030234&dopt=GenPept&RID=T04SGPYZ01N&log$=prottop&blast_rank=13) | ribonuclease III, nuclear isoform 1 [Homo sapiens] | [18.5](http://blast.ncbi.nlm.nih.gov/Blast.cgi#155030234%23155030234) | 18.5 | 83% | 289 |
| [NP_003788.2](http://www.ncbi.nlm.nih.gov/entrez/query.fcgi?cmd=Retrieve&db=Protein&list_uids=24041020&dopt=GenPept&RID=T04SGPYZ01N&log$=prottop&blast_rank=14) | embryonic ectoderm development isoform a [Homo sapiens] | [18.5](http://blast.ncbi.nlm.nih.gov/Blast.cgi#24041020%2324041020) | 18.5 | 83% | 289 |
| [NP_476518.4](http://www.ncbi.nlm.nih.gov/entrez/query.fcgi?cmd=Retrieve&db=Protein&list_uids=119120903&dopt=GenPept&RID=T04SGPYZ01N&log$=prottop&blast_rank=15) | par-3 partitioning defective 3 homolog B isoform c [Homo sapiens] | [18.0](http://blast.ncbi.nlm.nih.gov/Blast.cgi#119120903%23119120903) | 18.0 | 100% | 388 |
| [NP_995585.2](http://www.ncbi.nlm.nih.gov/entrez/query.fcgi?cmd=Retrieve&db=Protein&list_uids=119120907&dopt=GenPept&RID=T04SGPYZ01N&log$=prottop&blast_rank=16) | par-3 partitioning defective 3 homolog B isoform a [Homo sapiens] | [18.0](http://blast.ncbi.nlm.nih.gov/Blast.cgi#119120907%23119120907) | 18.0 | 100% | 388 |
| [NP_055520.3](http://www.ncbi.nlm.nih.gov/entrez/query.fcgi?cmd=Retrieve&db=Protein&list_uids=92091572&dopt=GenPept&RID=T04SGPYZ01N&log$=prottop&blast_rank=17) | dedicator of cytokinesis 4 [Homo sapiens] | [18.0](http://blast.ncbi.nlm.nih.gov/Blast.cgi#92091572%2392091572) | 18.0 | 83% | 388 |
| [NP_689739.4](http://www.ncbi.nlm.nih.gov/entrez/query.fcgi?cmd=Retrieve&db=Protein&list_uids=119120897&dopt=GenPept&RID=T04SGPYZ01N&log$=prottop&blast_rank=18) | par-3 partitioning defective 3 homolog B isoform b [Homo sapiens] | [18.0](http://blast.ncbi.nlm.nih.gov/Blast.cgi#119120897%23119120897) | 18.0 | 100% | 388 |
| [NP_001157934.1](http://www.ncbi.nlm.nih.gov/entrez/query.fcgi?cmd=Retrieve&db=Protein&list_uids=256600257&dopt=GenPept&RID=T04SGPYZ01N&log$=prottop&blast_rank=19) | mucin 12 [Homo sapiens] | [17.6](http://blast.ncbi.nlm.nih.gov/Blast.cgi#256600257%23256600257) | 70.4 | 66% | 521 |
| [NP_001126.3](http://www.ncbi.nlm.nih.gov/entrez/query.fcgi?cmd=Retrieve&db=Protein&list_uids=256017259&dopt=GenPept&RID=T04SGPYZ01N&log$=prottop&blast_rank=20) | aggrecan isoform 1 precursor [Homo sapiens] | [17.6](http://blast.ncbi.nlm.nih.gov/Blast.cgi#256017259%23256017259) | 17.6 | 66% | 521 |
| [NP_037359.3](http://www.ncbi.nlm.nih.gov/entrez/query.fcgi?cmd=Retrieve&db=Protein&list_uids=256017257&dopt=GenPept&RID=T04SGPYZ01N&log$=prottop&blast_rank=21) | aggrecan isoform 2 precursor [Homo sapiens] | [17.6](http://blast.ncbi.nlm.nih.gov/Blast.cgi#256017257%23256017257) | 17.6 | 66% | 521 |
| [NP_075561.3](http://www.ncbi.nlm.nih.gov/entrez/query.fcgi?cmd=Retrieve&db=Protein&list_uids=242332527&dopt=GenPept&RID=T04SGPYZ01N&log$=prottop&blast_rank=22) | hypothetical protein LOC65250 [Homo sapiens] | [17.6](http://blast.ncbi.nlm.nih.gov/Blast.cgi#242332527%23242332527) | 17.6 | 66% | 521 |
| [XP_001720603.2](http://www.ncbi.nlm.nih.gov/entrez/query.fcgi?cmd=Retrieve&db=Protein&list_uids=239758148&dopt=GenPept&RID=T04SGPYZ01N&log$=prottop&blast_rank=23) | PREDICTED: similar to mucin 11 [Homo sapiens] | [17.6](http://blast.ncbi.nlm.nih.gov/Blast.cgi#239758148%23239758148) | 17.6 | 66% | 521 |
| [XP_002345265.1](http://www.ncbi.nlm.nih.gov/entrez/query.fcgi?cmd=Retrieve&db=Protein&list_uids=239757409&dopt=GenPept&RID=T04SGPYZ01N&log$=prottop&blast_rank=24) | PREDICTED: similar to zinc finger protein 208 [Homo sapiens] | [17.6](http://blast.ncbi.nlm.nih.gov/Blast.cgi#239757409%23239757409) | 17.6 | 66% | 521 |
| [XP_001713947.2](http://www.ncbi.nlm.nih.gov/entrez/query.fcgi?cmd=Retrieve&db=Protein&list_uids=239749217&dopt=GenPept&RID=T04SGPYZ01N&log$=prottop&blast_rank=25) | PREDICTED: similar to Mucin-12, partial [Homo sapiens] | [17.6](http://blast.ncbi.nlm.nih.gov/Blast.cgi#239749217%23239749217) | 17.6 | 66% | 521 |
| [XP_002346533.1](http://www.ncbi.nlm.nih.gov/entrez/query.fcgi?cmd=Retrieve&db=Protein&list_uids=239748112&dopt=GenPept&RID=T04SGPYZ01N&log$=prottop&blast_rank=26) | PREDICTED: hypothetical protein [Homo sapiens] >ref|XP_002345694.1| PREDICTED: hypothetical protein XP_002345694 [Homo sapiens] | [17.6](http://blast.ncbi.nlm.nih.gov/Blast.cgi#239748112%23239748112) | 17.6 | 66% | 521 |
| [XP_001718452.2](http://www.ncbi.nlm.nih.gov/entrez/query.fcgi?cmd=Retrieve&db=Protein&list_uids=239746371&dopt=GenPept&RID=T04SGPYZ01N&log$=prottop&blast_rank=27) | PREDICTED: similar to zinc finger protein 208 [Homo sapiens] | [17.6](http://blast.ncbi.nlm.nih.gov/Blast.cgi#239746371%23239746371) | 17.6 | 66% | 521 |
| [XP_002346719.1](http://www.ncbi.nlm.nih.gov/entrez/query.fcgi?cmd=Retrieve&db=Protein&list_uids=239748533&dopt=GenPept&RID=T04SGPYZ01N&log$=prottop&blast_rank=28) | PREDICTED: hypothetical protein XP_002346719 [Homo sapiens] >ref|XP_002345848.1| PREDICTED: similar to Transmembrane protein ENSP00000382582 [Homo sapiens] | [17.6](http://blast.ncbi.nlm.nih.gov/Blast.cgi#239748533%23239748533) | 17.6 | 66% | 521 |
| [XP_001721664.2](http://www.ncbi.nlm.nih.gov/entrez/query.fcgi?cmd=Retrieve&db=Protein&list_uids=239744890&dopt=GenPept&RID=T04SGPYZ01N&log$=prottop&blast_rank=29) | PREDICTED: hypothetical protein [Homo sapiens] | [17.6](http://blast.ncbi.nlm.nih.gov/Blast.cgi#239744890%23239744890) | 33.1 | 83% | 521 |
| [NP_001137310.1](http://www.ncbi.nlm.nih.gov/entrez/query.fcgi?cmd=Retrieve&db=Protein&list_uids=219802232&dopt=GenPept&RID=T04SGPYZ01N&log$=prottop&blast_rank=30) | solute carrier family 13, member 5 isoform b [Homo sapiens] | [17.6](http://blast.ncbi.nlm.nih.gov/Blast.cgi#219802232%23219802232) | 17.6 | 66% | 521 |
| [XP_001719199.1](http://www.ncbi.nlm.nih.gov/entrez/query.fcgi?cmd=Retrieve&db=Protein&list_uids=169213518&dopt=GenPept&RID=T04SGPYZ01N&log$=prottop&blast_rank=31) | PREDICTED: hypothetical protein [Homo sapiens] >ref|XP_001717478.2| PREDICTED: hypothetical protein [Homo sapiens] >ref|XP_001719153.2| PREDICTED: hypothetical protein [Homo sapiens] | [17.6](http://blast.ncbi.nlm.nih.gov/Blast.cgi#169213518%23169213518) | 17.6 | 66% | 521 |
| [NP_001099007.1](http://www.ncbi.nlm.nih.gov/entrez/query.fcgi?cmd=Retrieve&db=Protein&list_uids=157676340&dopt=GenPept&RID=T04SGPYZ01N&log$=prottop&blast_rank=32) | zinc finger protein 142 [Homo sapiens] | [17.6](http://blast.ncbi.nlm.nih.gov/Blast.cgi#157676340%23157676340) | 17.6 | 66% | 521 |
| [NP_001129577.1](http://www.ncbi.nlm.nih.gov/entrez/query.fcgi?cmd=Retrieve&db=Protein&list_uids=209870059&dopt=GenPept&RID=T04SGPYZ01N&log$=prottop&blast_rank=33) | hypothetical protein LOC120376 [Homo sapiens] | [17.6](http://blast.ncbi.nlm.nih.gov/Blast.cgi#209870059%23209870059) | 17.6 | 66% | 521 |
| [NP_775907.4](http://www.ncbi.nlm.nih.gov/entrez/query.fcgi?cmd=Retrieve&db=Protein&list_uids=145580610&dopt=GenPept&RID=T04SGPYZ01N&log$=prottop&blast_rank=34) | WD repeat domain 62 isoform 2 [Homo sapiens] | [17.6](http://blast.ncbi.nlm.nih.gov/Blast.cgi#145580610%23145580610) | 31.0 | 100% | 521 |
| [NP_001077430.1](http://www.ncbi.nlm.nih.gov/entrez/query.fcgi?cmd=Retrieve&db=Protein&list_uids=145580608&dopt=GenPept&RID=T04SGPYZ01N&log$=prottop&blast_rank=35) | WD repeat domain 62 isoform 1 [Homo sapiens] | [17.6](http://blast.ncbi.nlm.nih.gov/Blast.cgi#145580608%23145580608) | 31.0 | 100% | 521 |
| [NP_848649.3](http://www.ncbi.nlm.nih.gov/entrez/query.fcgi?cmd=Retrieve&db=Protein&list_uids=148806932&dopt=GenPept&RID=T04SGPYZ01N&log$=prottop&blast_rank=36) | kyphoscoliosis peptidase [Homo sapiens] | [17.6](http://blast.ncbi.nlm.nih.gov/Blast.cgi#148806932%23148806932) | 17.6 | 66% | 521 |
| [NP_115826.2](http://www.ncbi.nlm.nih.gov/entrez/query.fcgi?cmd=Retrieve&db=Protein&list_uids=150010654&dopt=GenPept&RID=T04SGPYZ01N&log$=prottop&blast_rank=37) | HEAT repeat containing 7A isoform 1 [Homo sapiens] | [17.6](http://blast.ncbi.nlm.nih.gov/Blast.cgi#150010654%23150010654) | 17.6 | 66% | 521 |
| [NP_659455.3](http://www.ncbi.nlm.nih.gov/entrez/query.fcgi?cmd=Retrieve&db=Protein&list_uids=194239694&dopt=GenPept&RID=T04SGPYZ01N&log$=prottop&blast_rank=38) | nitric oxide-inducible gene protein [Homo sapiens] | [17.6](http://blast.ncbi.nlm.nih.gov/Blast.cgi#194239694%23194239694) | 17.6 | 66% | 521 |
| [NP_061971.3](http://www.ncbi.nlm.nih.gov/entrez/query.fcgi?cmd=Retrieve&db=Protein&list_uids=116642889&dopt=GenPept&RID=T04SGPYZ01N&log$=prottop&blast_rank=39) | cyclic nucleotide gated channel beta 3 [Homo sapiens] | [17.6](http://blast.ncbi.nlm.nih.gov/Blast.cgi#116642889%23116642889) | 17.6 | 83% | 521 |
| [NP_078928.3](http://www.ncbi.nlm.nih.gov/entrez/query.fcgi?cmd=Retrieve&db=Protein&list_uids=90991702&dopt=GenPept&RID=T04SGPYZ01N&log$=prottop&blast_rank=40) | leucine-rich repeat kinase 1 [Homo sapiens] | [17.6](http://blast.ncbi.nlm.nih.gov/Blast.cgi#90991702%2390991702) | 17.6 | 66% | 521 |
| [NP_001120936.1](http://www.ncbi.nlm.nih.gov/entrez/query.fcgi?cmd=Retrieve&db=Protein&list_uids=188536004&dopt=GenPept&RID=T04SGPYZ01N&log$=prottop&blast_rank=41) | zinc finger protein 469 [Homo sapiens] | [17.6](http://blast.ncbi.nlm.nih.gov/Blast.cgi#188536004%23188536004) | 17.6 | 66% | 521 |
| [XP_937130.2](http://www.ncbi.nlm.nih.gov/entrez/query.fcgi?cmd=Retrieve&db=Protein&list_uids=113420243&dopt=GenPept&RID=T04SGPYZ01N&log$=prottop&blast_rank=42) | PREDICTED: hypothetical protein LOC377711 isoform 5 [Homo sapiens] | [17.6](http://blast.ncbi.nlm.nih.gov/Blast.cgi#113420243%23113420243) | 17.6 | 66% | 521 |
| [NP_085911.2](http://www.ncbi.nlm.nih.gov/entrez/query.fcgi?cmd=Retrieve&db=Protein&list_uids=100913202&dopt=GenPept&RID=T04SGPYZ01N&log$=prottop&blast_rank=43) | DEAD/H (Asp-Glu-Ala-Asp/His) box polypeptide 11 isoform 1 [Homo sapiens] | [17.6](http://blast.ncbi.nlm.nih.gov/Blast.cgi#100913202%23100913202) | 33.9 | 83% | 521 |
| [NP_004390.3](http://www.ncbi.nlm.nih.gov/entrez/query.fcgi?cmd=Retrieve&db=Protein&list_uids=100913204&dopt=GenPept&RID=T04SGPYZ01N&log$=prottop&blast_rank=44) | DEAD/H (Asp-Glu-Ala-Asp/His) box polypeptide 11 isoform 2 [Homo sapiens] | [17.6](http://blast.ncbi.nlm.nih.gov/Blast.cgi#100913204%23100913204) | 33.9 | 83% | 521 |
| [NP_001136040.1](http://www.ncbi.nlm.nih.gov/entrez/query.fcgi?cmd=Retrieve&db=Protein&list_uids=217035121&dopt=GenPept&RID=T04SGPYZ01N&log$=prottop&blast_rank=45) | HMG-BOX transcription factor BBX isoform 1 [Homo sapiens] | [17.6](http://blast.ncbi.nlm.nih.gov/Blast.cgi#217035121%23217035121) | 17.6 | 66% | 521 |
| [NP_064620.2](http://www.ncbi.nlm.nih.gov/entrez/query.fcgi?cmd=Retrieve&db=Protein&list_uids=18378731&dopt=GenPept&RID=T04SGPYZ01N&log$=prottop&blast_rank=46) | HMG-BOX transcription factor BBX isoform 2 [Homo sapiens] | [17.6](http://blast.ncbi.nlm.nih.gov/Blast.cgi#18378731%2318378731) | 17.6 | 66% | 521 |
| [NP_001295.2](http://www.ncbi.nlm.nih.gov/entrez/query.fcgi?cmd=Retrieve&db=Protein&list_uids=22202611&dopt=GenPept&RID=T04SGPYZ01N&log$=prottop&blast_rank=47) | carboxypeptidase D precursor [Homo sapiens] | [17.6](http://blast.ncbi.nlm.nih.gov/Blast.cgi#22202611%2322202611) | 17.6 | 66% | 521 |
| [NP_004391.1](http://www.ncbi.nlm.nih.gov/entrez/query.fcgi?cmd=Retrieve&db=Protein&list_uids=212549538&dopt=GenPept&RID=T04SGPYZ01N&log$=prottop&blast_rank=48) | DEAD/H (Asp-Glu-Ala-Asp/His) box polypeptide 12 [Homo sapiens] | [17.6](http://blast.ncbi.nlm.nih.gov/Blast.cgi#212549538%23212549538) | 33.9 | 83% | 521 |
| [NP_001477.2](http://www.ncbi.nlm.nih.gov/entrez/query.fcgi?cmd=Retrieve&db=Protein&list_uids=221625433&dopt=GenPept&RID=T04SGPYZ01N&log$=prottop&blast_rank=49) | glucokinase regulatory protein [Homo sapiens] | [17.6](http://blast.ncbi.nlm.nih.gov/Blast.cgi#221625433%23221625433) | 17.6 | 83% | 521 |
| [NP_056160.2](http://www.ncbi.nlm.nih.gov/entrez/query.fcgi?cmd=Retrieve&db=Protein&list_uids=40548415&dopt=GenPept&RID=T04SGPYZ01N&log$=prottop&blast_rank=50) | dishevelled associated activator of morphogenesis 2 [Homo sapiens] | [17.6](http://blast.ncbi.nlm.nih.gov/Blast.cgi#40548415%2340548415) | 17.6 | 66% | 521 |
| [NP_006639.3](http://www.ncbi.nlm.nih.gov/entrez/query.fcgi?cmd=Retrieve&db=Protein&list_uids=32455273&dopt=GenPept&RID=T04SGPYZ01N&log$=prottop&blast_rank=51) | WNK lysine deficient protein kinase 2 [Homo sapiens] | [17.6](http://blast.ncbi.nlm.nih.gov/Blast.cgi#32455273%2332455273) | 17.6 | 66% | 521 |
| [NP_001019772.1](http://www.ncbi.nlm.nih.gov/entrez/query.fcgi?cmd=Retrieve&db=Protein&list_uids=66912168&dopt=GenPept&RID=T04SGPYZ01N&log$=prottop&blast_rank=52) | hypothetical protein LOC149643 [Homo sapiens] | [17.6](http://blast.ncbi.nlm.nih.gov/Blast.cgi#66912168%2366912168) | 17.6 | 66% | 521 |
| [NP_808218.1](http://www.ncbi.nlm.nih.gov/entrez/query.fcgi?cmd=Retrieve&db=Protein&list_uids=29171306&dopt=GenPept&RID=T04SGPYZ01N&log$=prottop&blast_rank=53) | solute carrier family 13, member 5 isoform a [Homo sapiens] | [17.6](http://blast.ncbi.nlm.nih.gov/Blast.cgi#29171306%2329171306) | 17.6 | 66% | 521 |
| [NP_778203.1](http://www.ncbi.nlm.nih.gov/entrez/query.fcgi?cmd=Retrieve&db=Protein&list_uids=28373119&dopt=GenPept&RID=T04SGPYZ01N&log$=prottop&blast_rank=54) | contactin 1 isoform 2 precursor [Homo sapiens] | [17.6](http://blast.ncbi.nlm.nih.gov/Blast.cgi#28373119%2328373119) | 17.6 | 66% | 521 |
| [NP_005355.2](http://www.ncbi.nlm.nih.gov/entrez/query.fcgi?cmd=Retrieve&db=Protein&list_uids=19923308&dopt=GenPept&RID=T04SGPYZ01N&log$=prottop&blast_rank=55) | melanoma antigen family A, 8 [Homo sapiens] >ref|NP_001159872.1| melanoma antigen family A, 8 [Homo sapiens] >ref|NP_001159873.1| melanoma antigen family A, 8 [Homo sapiens] | [17.6](http://blast.ncbi.nlm.nih.gov/Blast.cgi#19923308%2319923308) | 17.6 | 66% | 521 |
| [NP_078991.3](http://www.ncbi.nlm.nih.gov/entrez/query.fcgi?cmd=Retrieve&db=Protein&list_uids=94421469&dopt=GenPept&RID=T04SGPYZ01N&log$=prottop&blast_rank=56) | disulfide isomerase precursor [Homo sapiens] | [17.6](http://blast.ncbi.nlm.nih.gov/Blast.cgi#94421469%2394421469) | 17.6 | 66% | 521 |
| [NP_689671.2](http://www.ncbi.nlm.nih.gov/entrez/query.fcgi?cmd=Retrieve&db=Protein&list_uids=31377579&dopt=GenPept&RID=T04SGPYZ01N&log$=prottop&blast_rank=57) | zinc finger protein 785 [Homo sapiens] | [17.6](http://blast.ncbi.nlm.nih.gov/Blast.cgi#31377579%2331377579) | 17.6 | 66% | 521 |
| [NP_945345.2](http://www.ncbi.nlm.nih.gov/entrez/query.fcgi?cmd=Retrieve&db=Protein&list_uids=148664232&dopt=GenPept&RID=T04SGPYZ01N&log$=prottop&blast_rank=58) | transglutaminase 6 [Homo sapiens] | [17.6](http://blast.ncbi.nlm.nih.gov/Blast.cgi#148664232%23148664232) | 17.6 | 66% | 521 |
| [NP_000361.1](http://www.ncbi.nlm.nih.gov/entrez/query.fcgi?cmd=Retrieve&db=Protein&list_uids=4507723&dopt=GenPept&RID=T04SGPYZ01N&log$=prottop&blast_rank=59) | tocopherol (alpha) transfer protein [Homo sapiens] | [17.6](http://blast.ncbi.nlm.nih.gov/Blast.cgi#4507723%234507723) | 17.6 | 66% | 521 |
| [NP_002418.1](http://www.ncbi.nlm.nih.gov/entrez/query.fcgi?cmd=Retrieve&db=Protein&list_uids=4505209&dopt=GenPept&RID=T04SGPYZ01N&log$=prottop&blast_rank=60) | matrix metalloproteinase 13 preproprotein [Homo sapiens] | [17.6](http://blast.ncbi.nlm.nih.gov/Blast.cgi#4505209%234505209) | 17.6 | 100% | 521 |
| [NP_036370.2](http://www.ncbi.nlm.nih.gov/entrez/query.fcgi?cmd=Retrieve&db=Protein&list_uids=7657575&dopt=GenPept&RID=T04SGPYZ01N&log$=prottop&blast_rank=61) | sirtuin 1 isoform a [Homo sapiens] | [17.6](http://blast.ncbi.nlm.nih.gov/Blast.cgi#7657575%237657575) | 17.6 | 100% | 521 |
| [NP_003184.1](http://www.ncbi.nlm.nih.gov/entrez/query.fcgi?cmd=Retrieve&db=Protein&list_uids=4507375&dopt=GenPept&RID=T04SGPYZ01N&log$=prottop&blast_rank=62) | beta-tubulin cofactor E [Homo sapiens] >ref|NP_001072983.1| beta-tubulin cofactor E [Homo sapiens] | [17.6](http://blast.ncbi.nlm.nih.gov/Blast.cgi#4507375%234507375) | 17.6 | 66% | 521 |
| [NP_858059.1](http://www.ncbi.nlm.nih.gov/entrez/query.fcgi?cmd=Retrieve&db=Protein&list_uids=32307150&dopt=GenPept&RID=T04SGPYZ01N&log$=prottop&blast_rank=63) | O-linked GlcNAc transferase isoform 2 [Homo sapiens] | [17.6](http://blast.ncbi.nlm.nih.gov/Blast.cgi#32307150%2332307150) | 17.6 | 66% | 521 |
| [NP_149078.1](http://www.ncbi.nlm.nih.gov/entrez/query.fcgi?cmd=Retrieve&db=Protein&list_uids=14861836&dopt=GenPept&RID=T04SGPYZ01N&log$=prottop&blast_rank=64) | alpha-1,3-mannosyltransferase ALG2 [Homo sapiens] | [17.6](http://blast.ncbi.nlm.nih.gov/Blast.cgi#14861836%2314861836) | 17.6 | 66% | 521 |
| [NP_085127.2](http://www.ncbi.nlm.nih.gov/entrez/query.fcgi?cmd=Retrieve&db=Protein&list_uids=226442729&dopt=GenPept&RID=T04SGPYZ01N&log$=prottop&blast_rank=65) | kelch-like 15 [Homo sapiens] | [17.6](http://blast.ncbi.nlm.nih.gov/Blast.cgi#226442729%23226442729) | 17.6 | 66% | 521 |
| [NP_443198.1](http://www.ncbi.nlm.nih.gov/entrez/query.fcgi?cmd=Retrieve&db=Protein&list_uids=16757970&dopt=GenPept&RID=T04SGPYZ01N&log$=prottop&blast_rank=66) | niban protein isoform 2 [Homo sapiens] | [17.6](http://blast.ncbi.nlm.nih.gov/Blast.cgi#16757970%2316757970) | 17.6 | 66% | 521 |
| [NP_005182.1](http://www.ncbi.nlm.nih.gov/entrez/query.fcgi?cmd=Retrieve&db=Protein&list_uids=4885123&dopt=GenPept&RID=T04SGPYZ01N&log$=prottop&blast_rank=67) | CD80 antigen precursor [Homo sapiens] | [17.6](http://blast.ncbi.nlm.nih.gov/Blast.cgi#4885123%234885123) | 17.6 | 66% | 521 |
| [NP_060829.2](http://www.ncbi.nlm.nih.gov/entrez/query.fcgi?cmd=Retrieve&db=Protein&list_uids=227498137&dopt=GenPept&RID=T04SGPYZ01N&log$=prottop&blast_rank=68) | UFM1-specific peptidase 2 [Homo sapiens] | [17.6](http://blast.ncbi.nlm.nih.gov/Blast.cgi#227498137%23227498137) | 17.6 | 66% | 521 |
| [NP_036356.1](http://www.ncbi.nlm.nih.gov/entrez/query.fcgi?cmd=Retrieve&db=Protein&list_uids=41872673&dopt=GenPept&RID=T04SGPYZ01N&log$=prottop&blast_rank=69) | NIMA-related kinase 1 [Homo sapiens] | [17.6](http://blast.ncbi.nlm.nih.gov/Blast.cgi#41872673%2341872673) | 17.6 | 66% | 521 |
| [NP_001130.1](http://www.ncbi.nlm.nih.gov/entrez/query.fcgi?cmd=Retrieve&db=Protein&list_uids=4502053&dopt=GenPept&RID=T04SGPYZ01N&log$=prottop&blast_rank=70) | arachidonate 12-lipoxygenase, 12R type [Homo sapiens] | [17.6](http://blast.ncbi.nlm.nih.gov/Blast.cgi#4502053%234502053) | 17.6 | 66% | 521 |
| [NP_005503.1](http://www.ncbi.nlm.nih.gov/entrez/query.fcgi?cmd=Retrieve&db=Protein&list_uids=5031707&dopt=GenPept&RID=T04SGPYZ01N&log$=prottop&blast_rank=71) | leucine rich repeat containing 32 precursor [Homo sapiens] >ref|NP_001122394.1| leucine rich repeat containing 32 precursor [Homo sapiens] | [17.6](http://blast.ncbi.nlm.nih.gov/Blast.cgi#5031707%235031707) | 17.6 | 66% | 521 |
| [NP_001834.2](http://www.ncbi.nlm.nih.gov/entrez/query.fcgi?cmd=Retrieve&db=Protein&list_uids=28373117&dopt=GenPept&RID=T04SGPYZ01N&log$=prottop&blast_rank=72) | contactin 1 isoform 1 precursor [Homo sapiens] | [17.6](http://blast.ncbi.nlm.nih.gov/Blast.cgi#28373117%2328373117) | 17.6 | 66% | 521 |
| [NP_858058.1](http://www.ncbi.nlm.nih.gov/entrez/query.fcgi?cmd=Retrieve&db=Protein&list_uids=32307148&dopt=GenPept&RID=T04SGPYZ01N&log$=prottop&blast_rank=73) | O-linked GlcNAc transferase isoform 1 [Homo sapiens] | [17.6](http://blast.ncbi.nlm.nih.gov/Blast.cgi#32307148%2332307148) | 17.6 | 66% | 521 |
| [NP_689651.1](http://www.ncbi.nlm.nih.gov/entrez/query.fcgi?cmd=Retrieve&db=Protein&list_uids=132566527&dopt=GenPept&RID=T04SGPYZ01N&log$=prottop&blast_rank=74) | DEAD/H (Asp-Glu-Ala-Asp/His) box polypeptide 11 isoform 3 [Homo sapiens] | [17.6](http://blast.ncbi.nlm.nih.gov/Blast.cgi#132566527%23132566527) | 33.9 | 83% | 521 |
| [NP_031379.2](http://www.ncbi.nlm.nih.gov/entrez/query.fcgi?cmd=Retrieve&db=Protein&list_uids=42476111&dopt=GenPept&RID=T04SGPYZ01N&log$=prottop&blast_rank=75) | guanine nucleotide binding protein (G protein) alpha 12 [Homo sapiens] | [17.6](http://blast.ncbi.nlm.nih.gov/Blast.cgi#42476111%2342476111) | 17.6 | 66% | 521 |
| [NP_001157871.1](http://www.ncbi.nlm.nih.gov/entrez/query.fcgi?cmd=Retrieve&db=Protein&list_uids=256542279&dopt=GenPept&RID=T04SGPYZ01N&log$=prottop&blast_rank=76) | hypothetical protein LOC729665 [Homo sapiens] | [17.2](http://blast.ncbi.nlm.nih.gov/Blast.cgi#256542279%23256542279) | 17.2 | 66% | 699 |
| [XP_001727082.2](http://www.ncbi.nlm.nih.gov/entrez/query.fcgi?cmd=Retrieve&db=Protein&list_uids=239757081&dopt=GenPept&RID=T04SGPYZ01N&log$=prottop&blast_rank=77) | PREDICTED: hypothetical protein LOC100127987 [Homo sapiens] | [17.2](http://blast.ncbi.nlm.nih.gov/Blast.cgi#239757081%23239757081) | 17.2 | 66% | 699 |
| [XP_291816.7](http://www.ncbi.nlm.nih.gov/entrez/query.fcgi?cmd=Retrieve&db=Protein&list_uids=239744300&dopt=GenPept&RID=T04SGPYZ01N&log$=prottop&blast_rank=78) | PREDICTED: otogelin isoform 2 [Homo sapiens] >ref|XP_001717583.2| PREDICTED: otogelin isoform 1 [Homo sapiens] | [17.2](http://blast.ncbi.nlm.nih.gov/Blast.cgi#239744300%23239744300) | 27.2 | 83% | 699 |
| [XP_037557.5](http://www.ncbi.nlm.nih.gov/entrez/query.fcgi?cmd=Retrieve&db=Protein&list_uids=239744669&dopt=GenPept&RID=T04SGPYZ01N&log$=prottop&blast_rank=79) | PREDICTED: TBC1 domain family, member 30 [Homo sapiens] >ref|XP_001716885.2| PREDICTED: TBC1 domain family, member 30 [Homo sapiens] | [17.2](http://blast.ncbi.nlm.nih.gov/Blast.cgi#239744669%23239744669) | 17.2 | 66% | 699 |
| [XP_944569.2](http://www.ncbi.nlm.nih.gov/entrez/query.fcgi?cmd=Retrieve&db=Protein&list_uids=239750365&dopt=GenPept&RID=T04SGPYZ01N&log$=prottop&blast_rank=80) | PREDICTED: TBC1 domain family, member 30 [Homo sapiens] | [17.2](http://blast.ncbi.nlm.nih.gov/Blast.cgi#239750365%23239750365) | 17.2 | 66% | 699 |
| [NP_872425.2](http://www.ncbi.nlm.nih.gov/entrez/query.fcgi?cmd=Retrieve&db=Protein&list_uids=209977023&dopt=GenPept&RID=T04SGPYZ01N&log$=prottop&blast_rank=81) | secretory protein LOC348174 precursor [Homo sapiens] >ref|NP_001129686.1| secretory protein LOC348174 precursor [Homo sapiens] | [17.2](http://blast.ncbi.nlm.nih.gov/Blast.cgi#209977023%23209977023) | 17.2 | 66% | 699 |
| [NP_001011880.2](http://www.ncbi.nlm.nih.gov/entrez/query.fcgi?cmd=Retrieve&db=Protein&list_uids=194018451&dopt=GenPept&RID=T04SGPYZ01N&log$=prottop&blast_rank=82) | C-type lectin domain family 18, member B precursor [Homo sapiens] | [17.2](http://blast.ncbi.nlm.nih.gov/Blast.cgi#194018451%23194018451) | 17.2 | 66% | 699 |
| [NP_694996.5](http://www.ncbi.nlm.nih.gov/entrez/query.fcgi?cmd=Retrieve&db=Protein&list_uids=183583553&dopt=GenPept&RID=T04SGPYZ01N&log$=prottop&blast_rank=83) | collagen, type XXIX, alpha 1 [Homo sapiens] | [17.2](http://blast.ncbi.nlm.nih.gov/Blast.cgi#183583553%23183583553) | 27.2 | 83% | 699 |
| [XP_001717814.1](http://www.ncbi.nlm.nih.gov/entrez/query.fcgi?cmd=Retrieve&db=Protein&list_uids=169212748&dopt=GenPept&RID=T04SGPYZ01N&log$=prottop&blast_rank=84) | PREDICTED: hypothetical protein LOC100127987 [Homo sapiens] | [17.2](http://blast.ncbi.nlm.nih.gov/Blast.cgi#169212748%23169212748) | 17.2 | 66% | 699 |
| [NP_001017969.2](http://www.ncbi.nlm.nih.gov/entrez/query.fcgi?cmd=Retrieve&db=Protein&list_uids=148612838&dopt=GenPept&RID=T04SGPYZ01N&log$=prottop&blast_rank=85) | hypothetical protein LOC158358 [Homo sapiens] | [17.2](http://blast.ncbi.nlm.nih.gov/Blast.cgi#148612838%23148612838) | 17.2 | 66% | 699 |
| [NP_001028225.1](http://www.ncbi.nlm.nih.gov/entrez/query.fcgi?cmd=Retrieve&db=Protein&list_uids=74271814&dopt=GenPept&RID=T04SGPYZ01N&log$=prottop&blast_rank=86) | NLR family, pyrin domain containing 1 isoform 5 [Homo sapiens] | [17.2](http://blast.ncbi.nlm.nih.gov/Blast.cgi#74271814%2374271814) | 17.2 | 66% | 699 |
| [NP_001611.1](http://www.ncbi.nlm.nih.gov/entrez/query.fcgi?cmd=Retrieve&db=Protein&list_uids=61743954&dopt=GenPept&RID=T04SGPYZ01N&log$=prottop&blast_rank=87) | AHNAK nucleoprotein isoform 1 [Homo sapiens] | [17.2](http://blast.ncbi.nlm.nih.gov/Blast.cgi#61743954%2361743954) | 180 | 83% | 699 |
| [NP_079209.3](http://www.ncbi.nlm.nih.gov/entrez/query.fcgi?cmd=Retrieve&db=Protein&list_uids=169403957&dopt=GenPept&RID=T04SGPYZ01N&log$=prottop&blast_rank=88) | ankyrin repeat domain 53 isoform b [Homo sapiens] | [17.2](http://blast.ncbi.nlm.nih.gov/Blast.cgi#169403957%23169403957) | 17.2 | 66% | 699 |
| [NP_689812.3](http://www.ncbi.nlm.nih.gov/entrez/query.fcgi?cmd=Retrieve&db=Protein&list_uids=269846998&dopt=GenPept&RID=T04SGPYZ01N&log$=prottop&blast_rank=89) | major facilitator superfamily domain containing 6-like [Homo sapiens] | [17.2](http://blast.ncbi.nlm.nih.gov/Blast.cgi#269846998%23269846998) | 17.2 | 66% | 699 |
| [XP_946095.3](http://www.ncbi.nlm.nih.gov/entrez/query.fcgi?cmd=Retrieve&db=Protein&list_uids=239749989&dopt=GenPept&RID=T04SGPYZ01N&log$=prottop&blast_rank=90) | PREDICTED: otogelin isoform 2 [Homo sapiens] | [17.2](http://blast.ncbi.nlm.nih.gov/Blast.cgi#239749989%23239749989) | 27.2 | 83% | 699 |
| [XP_001721774.1](http://www.ncbi.nlm.nih.gov/entrez/query.fcgi?cmd=Retrieve&db=Protein&list_uids=169213239&dopt=GenPept&RID=T04SGPYZ01N&log$=prottop&blast_rank=91) | PREDICTED: hypothetical protein LOC100127987 [Homo sapiens] | [17.2](http://blast.ncbi.nlm.nih.gov/Blast.cgi#169213239%23169213239) | 17.2 | 66% | 699 |
| [NP_003234.2](http://www.ncbi.nlm.nih.gov/entrez/query.fcgi?cmd=Retrieve&db=Protein&list_uids=56682966&dopt=GenPept&RID=T04SGPYZ01N&log$=prottop&blast_rank=92) | transforming growth factor, beta receptor III [Homo sapiens] | [17.2](http://blast.ncbi.nlm.nih.gov/Blast.cgi#56682966%2356682966) | 17.2 | 66% | 699 |
| [NP_203748.1](http://www.ncbi.nlm.nih.gov/entrez/query.fcgi?cmd=Retrieve&db=Protein&list_uids=148886668&dopt=GenPept&RID=T04SGPYZ01N&log$=prottop&blast_rank=93) | zinc finger CCCH-type containing 12C [Homo sapiens] | [17.2](http://blast.ncbi.nlm.nih.gov/Blast.cgi#148886668%23148886668) | 17.2 | 66% | 699 |
| [NP_056127.2](http://www.ncbi.nlm.nih.gov/entrez/query.fcgi?cmd=Retrieve&db=Protein&list_uids=150378498&dopt=GenPept&RID=T04SGPYZ01N&log$=prottop&blast_rank=94) | fragile site-associated protein [Homo sapiens] | [17.2](http://blast.ncbi.nlm.nih.gov/Blast.cgi#150378498%23150378498) | 17.2 | 66% | 699 |
| [NP_003619.2](http://www.ncbi.nlm.nih.gov/entrez/query.fcgi?cmd=Retrieve&db=Protein&list_uids=53829374&dopt=GenPept&RID=T04SGPYZ01N&log$=prottop&blast_rank=95) | plakophilin 4 isoform a [Homo sapiens] | [17.2](http://blast.ncbi.nlm.nih.gov/Blast.cgi#53829374%2353829374) | 17.2 | 66% | 699 |
| [NP_001005476.1](http://www.ncbi.nlm.nih.gov/entrez/query.fcgi?cmd=Retrieve&db=Protein&list_uids=53829376&dopt=GenPept&RID=T04SGPYZ01N&log$=prottop&blast_rank=96) | plakophilin 4 isoform b [Homo sapiens] | [17.2](http://blast.ncbi.nlm.nih.gov/Blast.cgi#53829376%2353829376) | 17.2 | 66% | 699 |
| [NP_112235.2](http://www.ncbi.nlm.nih.gov/entrez/query.fcgi?cmd=Retrieve&db=Protein&list_uids=50428940&dopt=GenPept&RID=T04SGPYZ01N&log$=prottop&blast_rank=97) | mediator complex subunit 25 [Homo sapiens] | [17.2](http://blast.ncbi.nlm.nih.gov/Blast.cgi#50428940%2350428940) | 17.2 | 66% | 699 |
| [NP_001019626.1](http://www.ncbi.nlm.nih.gov/entrez/query.fcgi?cmd=Retrieve&db=Protein&list_uids=66792814&dopt=GenPept&RID=T04SGPYZ01N&log$=prottop&blast_rank=98) | retrotransposon gag domain containing 4 [Homo sapiens] | [17.2](http://blast.ncbi.nlm.nih.gov/Blast.cgi#66792814%2366792814) | 17.2 | 66% | 699 |
| [NP_115536.1](http://www.ncbi.nlm.nih.gov/entrez/query.fcgi?cmd=Retrieve&db=Protein&list_uids=23943787&dopt=GenPept&RID=T04SGPYZ01N&log$=prottop&blast_rank=99) | dermatan sulfate epimerase-like [Homo sapiens] | [17.2](http://blast.ncbi.nlm.nih.gov/Blast.cgi#23943787%2323943787) | 17.2 | 66% | 699 |
| [NP_000453.2](http://www.ncbi.nlm.nih.gov/entrez/query.fcgi?cmd=Retrieve&db=Protein&list_uids=19718766&dopt=GenPept&RID=T04SGPYZ01N&log$=prottop&blast_rank=100) | ubiquitin protein ligase E3A isoform 2 [Homo sapiens] | [17.2](http://blast.ncbi.nlm.nih.gov/Blast.cgi#19718766%2319718766) | 17.2 | 66% | 699 |

| **Accession** | **Proteins with a match to DLITPGD peptide** | **[Max score](http://blast.ncbi.nlm.nih.gov/Blast.cgi?CMD=Get&ALIGNMENTS=100&ALIGNMENT_VIEW=Pairwise&CDD_SEARCH_STATE=1&DATABASE_SORT=0&DESCRIPTIONS=100&ENTREZ_QUERY=txid9606 %5BORGN%5D&FIRST_QUERY_NUM=0&FORMAT_OBJECT=Alignment&FORMAT_PAGE_TARGET=&FORMAT_TYPE=HTML&GET_SEQUENCE=yes&I_THRESH=&MASK_CHAR=2&MASK_COLOR=1&NEW_DESIGN=on&NEW_VIEW=yes&NUM_OVERVIEW=100&OLD_BLAST=false&PAGE=Proteins&QUERY_INDEX=0&QUERY_NUMBER=0&RESULTS_PAGE_TARGET=&RID=T0ACP8AC01N&SHOW_LINKOUT=yes&SHOW_OVERVIEW=yes&STEP_NUMBER=&WORD_SIZE=2&DISPLAY_SORT=1&HSP_SORT=1" \l "sort_mark)** | **[Total score](http://blast.ncbi.nlm.nih.gov/Blast.cgi?CMD=Get&ALIGNMENTS=100&ALIGNMENT_VIEW=Pairwise&CDD_SEARCH_STATE=1&DATABASE_SORT=0&DESCRIPTIONS=100&ENTREZ_QUERY=txid9606 %5BORGN%5D&FIRST_QUERY_NUM=0&FORMAT_OBJECT=Alignment&FORMAT_PAGE_TARGET=&FORMAT_TYPE=HTML&GET_SEQUENCE=yes&I_THRESH=&MASK_CHAR=2&MASK_COLOR=1&NEW_DESIGN=on&NEW_VIEW=yes&NUM_OVERVIEW=100&OLD_BLAST=false&PAGE=Proteins&QUERY_INDEX=0&QUERY_NUMBER=0&RESULTS_PAGE_TARGET=&RID=T0ACP8AC01N&SHOW_LINKOUT=yes&SHOW_OVERVIEW=yes&STEP_NUMBER=&WORD_SIZE=2&DISPLAY_SORT=2&HSP_SORT=1" \l "sort_mark)** | **[Query coverage](http://blast.ncbi.nlm.nih.gov/Blast.cgi?CMD=Get&ALIGNMENTS=100&ALIGNMENT_VIEW=Pairwise&CDD_SEARCH_STATE=1&DATABASE_SORT=0&DESCRIPTIONS=100&ENTREZ_QUERY=txid9606 %5BORGN%5D&FIRST_QUERY_NUM=0&FORMAT_OBJECT=Alignment&FORMAT_PAGE_TARGET=&FORMAT_TYPE=HTML&GET_SEQUENCE=yes&I_THRESH=&MASK_CHAR=2&MASK_COLOR=1&NEW_DESIGN=on&NEW_VIEW=yes&NUM_OVERVIEW=100&OLD_BLAST=false&PAGE=Proteins&QUERY_INDEX=0&QUERY_NUMBER=0&RESULTS_PAGE_TARGET=&RID=T0ACP8AC01N&SHOW_LINKOUT=yes&SHOW_OVERVIEW=yes&STEP_NUMBER=&WORD_SIZE=2&DISPLAY_SORT=4&HSP_SORT=0" \l "sort_mark)** | **[E value](http://blast.ncbi.nlm.nih.gov/Blast.cgi?CMD=Get&ALIGNMENTS=100&ALIGNMENT_VIEW=Pairwise&CDD_SEARCH_STATE=1&DATABASE_SORT=0&DESCRIPTIONS=100&ENTREZ_QUERY=txid9606 %5BORGN%5D&FIRST_QUERY_NUM=0&FORMAT_OBJECT=Alignment&FORMAT_PAGE_TARGET=&FORMAT_TYPE=HTML&GET_SEQUENCE=yes&I_THRESH=&MASK_CHAR=2&MASK_COLOR=1&NEW_DESIGN=on&NEW_VIEW=yes&NUM_OVERVIEW=100&OLD_BLAST=false&PAGE=Proteins&QUERY_INDEX=0&QUERY_NUMBER=0&RESULTS_PAGE_TARGET=&RID=T0ACP8AC01N&SHOW_LINKOUT=yes&SHOW_OVERVIEW=yes&STEP_NUMBER=&WORD_SIZE=2&DISPLAY_SORT=0&HSP_SORT=0" \l "sort_mark)** |
| --- | --- | --- | --- | --- | --- |
| [NP_060774.2](http://www.ncbi.nlm.nih.gov/entrez/query.fcgi?cmd=Retrieve&db=Protein&list_uids=88703045&dopt=GenPept&RID=T0ACP8AC01N&log$=prottop&blast_rank=1) | proline rich 11 [Homo sapiens] | [21.8](http://blast.ncbi.nlm.nih.gov/Blast.cgi#88703045%2388703045) | 56.2 | 85% | 32 |
| [NP_055877.3](http://www.ncbi.nlm.nih.gov/entrez/query.fcgi?cmd=Retrieve&db=Protein&list_uids=40807452&dopt=GenPept&RID=T0ACP8AC01N&log$=prottop&blast_rank=2) | peroxisome proliferator-activated receptor gamma, coactivator-related 1 [Homo sapiens] | [20.6](http://blast.ncbi.nlm.nih.gov/Blast.cgi#40807452%2340807452) | 20.6 | 100% | 78 |
| [NP_001505.1](http://www.ncbi.nlm.nih.gov/entrez/query.fcgi?cmd=Retrieve&db=Protein&list_uids=4504193&dopt=GenPept&RID=T0ACP8AC01N&log$=prottop&blast_rank=3) | general transcription factor IIB [Homo sapiens] | [20.2](http://blast.ncbi.nlm.nih.gov/Blast.cgi#4504193%234504193) | 20.2 | 100% | 104 |
| [NP_001011709.2](http://www.ncbi.nlm.nih.gov/entrez/query.fcgi?cmd=Retrieve&db=Protein&list_uids=190341077&dopt=GenPept&RID=T0ACP8AC01N&log$=prottop&blast_rank=4) | pancreatic lipase-related protein 3 precursor [Homo sapiens] | [19.3](http://blast.ncbi.nlm.nih.gov/Blast.cgi#190341077%23190341077) | 19.3 | 71% | 188 |
| [NP_001108105.1](http://www.ncbi.nlm.nih.gov/entrez/query.fcgi?cmd=Retrieve&db=Protein&list_uids=167860120&dopt=GenPept&RID=T0ACP8AC01N&log$=prottop&blast_rank=5) | phospholipase A2, group IVB [Homo sapiens] | [19.3](http://blast.ncbi.nlm.nih.gov/Blast.cgi#167860120%23167860120) | 19.3 | 100% | 188 |
| [NP_001157260.1](http://www.ncbi.nlm.nih.gov/entrez/query.fcgi?cmd=Retrieve&db=Protein&list_uids=255003738&dopt=GenPept&RID=T0ACP8AC01N&log$=prottop&blast_rank=6) | ROD1 regulator of differentiation 1 isoform 2 [Homo sapiens] | [19.3](http://blast.ncbi.nlm.nih.gov/Blast.cgi#255003738%23255003738) | 19.3 | 71% | 188 |
| [NP_997400.2](http://www.ncbi.nlm.nih.gov/entrez/query.fcgi?cmd=Retrieve&db=Protein&list_uids=145275198&dopt=GenPept&RID=T0ACP8AC01N&log$=prottop&blast_rank=7) | ADAMTS-like 3 precursor [Homo sapiens] | [19.3](http://blast.ncbi.nlm.nih.gov/Blast.cgi#145275198%23145275198) | 19.3 | 71% | 188 |
| [NP_006408.3](http://www.ncbi.nlm.nih.gov/entrez/query.fcgi?cmd=Retrieve&db=Protein&list_uids=166706911&dopt=GenPept&RID=T0ACP8AC01N&log$=prottop&blast_rank=8) | interferon-induced, hepatitis C-associated microtubular aggregate protein [Homo sapiens] | [19.3](http://blast.ncbi.nlm.nih.gov/Blast.cgi#166706911%23166706911) | 19.3 | 100% | 188 |
| [NP_060250.2](http://www.ncbi.nlm.nih.gov/entrez/query.fcgi?cmd=Retrieve&db=Protein&list_uids=54112403&dopt=GenPept&RID=T0ACP8AC01N&log$=prottop&blast_rank=9) | chromodomain helicase DNA binding protein 7 [Homo sapiens] | [19.3](http://blast.ncbi.nlm.nih.gov/Blast.cgi#54112403%2354112403) | 19.3 | 71% | 188 |
| [NP_079410.4](http://www.ncbi.nlm.nih.gov/entrez/query.fcgi?cmd=Retrieve&db=Protein&list_uids=95147342&dopt=GenPept&RID=T0ACP8AC01N&log$=prottop&blast_rank=10) | chromodomain helicase DNA binding protein 9 [Homo sapiens] | [19.3](http://blast.ncbi.nlm.nih.gov/Blast.cgi#95147342%2395147342) | 19.3 | 71% | 188 |
| [NP_001157262.1](http://www.ncbi.nlm.nih.gov/entrez/query.fcgi?cmd=Retrieve&db=Protein&list_uids=255003744&dopt=GenPept&RID=T0ACP8AC01N&log$=prottop&blast_rank=11) | ROD1 regulator of differentiation 1 isoform 3 [Homo sapiens] | [19.3](http://blast.ncbi.nlm.nih.gov/Blast.cgi#255003744%23255003744) | 35.2 | 71% | 188 |
| [NP_005147.3](http://www.ncbi.nlm.nih.gov/entrez/query.fcgi?cmd=Retrieve&db=Protein&list_uids=38569466&dopt=GenPept&RID=T0ACP8AC01N&log$=prottop&blast_rank=12) | ROD1 regulator of differentiation 1 isoform 1 [Homo sapiens] | [19.3](http://blast.ncbi.nlm.nih.gov/Blast.cgi#38569466%2338569466) | 35.2 | 71% | 188 |
| [NP_005081.1](http://www.ncbi.nlm.nih.gov/entrez/query.fcgi?cmd=Retrieve&db=Protein&list_uids=4826914&dopt=GenPept&RID=T0ACP8AC01N&log$=prottop&blast_rank=13) | JMJD7-PLA2G4B protein [Homo sapiens] | [19.3](http://blast.ncbi.nlm.nih.gov/Blast.cgi#4826914%234826914) | 19.3 | 100% | 188 |
| [NP_060495.2](http://www.ncbi.nlm.nih.gov/entrez/query.fcgi?cmd=Retrieve&db=Protein&list_uids=21361684&dopt=GenPept&RID=T0ACP8AC01N&log$=prottop&blast_rank=14) | G patch domain containing 1 [Homo sapiens] | [19.3](http://blast.ncbi.nlm.nih.gov/Blast.cgi#21361684%2321361684) | 19.3 | 71% | 188 |
| [NP_055756.1](http://www.ncbi.nlm.nih.gov/entrez/query.fcgi?cmd=Retrieve&db=Protein&list_uids=7662340&dopt=GenPept&RID=T0ACP8AC01N&log$=prottop&blast_rank=15) | MORC family CW-type zinc finger 2 [Homo sapiens] | [19.3](http://blast.ncbi.nlm.nih.gov/Blast.cgi#7662340%237662340) | 19.3 | 100% | 188 |
| [NP_001091988.1](http://www.ncbi.nlm.nih.gov/entrez/query.fcgi?cmd=Retrieve&db=Protein&list_uids=148719671&dopt=GenPept&RID=T0ACP8AC01N&log$=prottop&blast_rank=16) | G-protein coupled receptor 116 precursor [Homo sapiens] >ref|NP_056049.4| G-protein coupled receptor 116 precursor [Homo sapiens] | [18.9](http://blast.ncbi.nlm.nih.gov/Blast.cgi#148719671%23148719671) | 18.9 | 71% | 252 |
| [NP_116028.1](http://www.ncbi.nlm.nih.gov/entrez/query.fcgi?cmd=Retrieve&db=Protein&list_uids=14249174&dopt=GenPept&RID=T0ACP8AC01N&log$=prottop&blast_rank=17) | pleckstrin homology domain containing, family A (phosphoinositide binding specific) member 8 [Homo sapiens] | [18.9](http://blast.ncbi.nlm.nih.gov/Blast.cgi#14249174%2314249174) | 18.9 | 100% | 252 |
| [NP_839943.2](http://www.ncbi.nlm.nih.gov/entrez/query.fcgi?cmd=Retrieve&db=Protein&list_uids=39753961&dopt=GenPept&RID=T0ACP8AC01N&log$=prottop&blast_rank=18) | IQ motif containing GTPase activating protein 3 [Homo sapiens] | [18.9](http://blast.ncbi.nlm.nih.gov/Blast.cgi#39753961%2339753961) | 18.9 | 71% | 252 |
| [NP_001431.1](http://www.ncbi.nlm.nih.gov/entrez/query.fcgi?cmd=Retrieve&db=Protein&list_uids=4503617&dopt=GenPept&RID=T0ACP8AC01N&log$=prottop&blast_rank=19) | exostoses-like 3 [Homo sapiens] | [18.9](http://blast.ncbi.nlm.nih.gov/Blast.cgi#4503617%234503617) | 18.9 | 71% | 252 |
| [NP_001129479.1](http://www.ncbi.nlm.nih.gov/entrez/query.fcgi?cmd=Retrieve&db=Protein&list_uids=209862809&dopt=GenPept&RID=T0ACP8AC01N&log$=prottop&blast_rank=20) | FXYD domain containing ion transport regulator 3 isoform 3 [Homo sapiens] | [18.5](http://blast.ncbi.nlm.nih.gov/Blast.cgi#209862809%23209862809) | 18.5 | 71% | 338 |
| [NP_001128471.1](http://www.ncbi.nlm.nih.gov/entrez/query.fcgi?cmd=Retrieve&db=Protein&list_uids=201861813&dopt=GenPept&RID=T0ACP8AC01N&log$=prottop&blast_rank=21) | fermitin family homolog 2 isoform 2 [Homo sapiens] | [18.5](http://blast.ncbi.nlm.nih.gov/Blast.cgi#201861813%23201861813) | 34.4 | 85% | 338 |
| [NP_055874.2](http://www.ncbi.nlm.nih.gov/entrez/query.fcgi?cmd=Retrieve&db=Protein&list_uids=156938343&dopt=GenPept&RID=T0ACP8AC01N&log$=prottop&blast_rank=22) | talin 2 [Homo sapiens] | [18.5](http://blast.ncbi.nlm.nih.gov/Blast.cgi#156938343%23156938343) | 18.5 | 85% | 338 |
| [NP_997290.2](http://www.ncbi.nlm.nih.gov/entrez/query.fcgi?cmd=Retrieve&db=Protein&list_uids=194018492&dopt=GenPept&RID=T0ACP8AC01N&log$=prottop&blast_rank=23) | transmembrane protease, serine 11F [Homo sapiens] | [18.5](http://blast.ncbi.nlm.nih.gov/Blast.cgi#194018492%23194018492) | 18.5 | 71% | 338 |
| [NP_001136254.1](http://www.ncbi.nlm.nih.gov/entrez/query.fcgi?cmd=Retrieve&db=Protein&list_uids=218505835&dopt=GenPept&RID=T0ACP8AC01N&log$=prottop&blast_rank=24) | membrane-associated guanylate kinase-related 3 isoform 1 [Homo sapiens] | [18.5](http://blast.ncbi.nlm.nih.gov/Blast.cgi#218505835%23218505835) | 18.5 | 71% | 338 |
| [NP_001120703.1](http://www.ncbi.nlm.nih.gov/entrez/query.fcgi?cmd=Retrieve&db=Protein&list_uids=187829452&dopt=GenPept&RID=T0ACP8AC01N&log$=prottop&blast_rank=25) | autism susceptibility candidate 2 isoform 2 [Homo sapiens] | [18.5](http://blast.ncbi.nlm.nih.gov/Blast.cgi#187829452%23187829452) | 18.5 | 71% | 338 |
| [NP_065960.2](http://www.ncbi.nlm.nih.gov/entrez/query.fcgi?cmd=Retrieve&db=Protein&list_uids=40254949&dopt=GenPept&RID=T0ACP8AC01N&log$=prottop&blast_rank=26) | erythrocyte membrane protein band 4.1 like 5 [Homo sapiens] | [18.5](http://blast.ncbi.nlm.nih.gov/Blast.cgi#40254949%2340254949) | 18.5 | 85% | 338 |
| [NP_068710.1](http://www.ncbi.nlm.nih.gov/entrez/query.fcgi?cmd=Retrieve&db=Protein&list_uids=11612674&dopt=GenPept&RID=T0ACP8AC01N&log$=prottop&blast_rank=27) | FXYD domain containing ion transport regulator 3 isoform 2 precursor [Homo sapiens] >ref|NP_001129484.1| FXYD domain containing ion transport regulator 3 isoform 2 precursor [Homo sapiens] | [18.5](http://blast.ncbi.nlm.nih.gov/Blast.cgi#11612674%2311612674) | 18.5 | 71% | 338 |
| [NP_000323.2](http://www.ncbi.nlm.nih.gov/entrez/query.fcgi?cmd=Retrieve&db=Protein&list_uids=51479158&dopt=GenPept&RID=T0ACP8AC01N&log$=prottop&blast_rank=28) | ataxin 1 [Homo sapiens] >ref|NP_001121636.1| ataxin 1 [Homo sapiens] | [18.5](http://blast.ncbi.nlm.nih.gov/Blast.cgi#51479158%2351479158) | 18.5 | 71% | 338 |
| [NP_006833.2](http://www.ncbi.nlm.nih.gov/entrez/query.fcgi?cmd=Retrieve&db=Protein&list_uids=55749531&dopt=GenPept&RID=T0ACP8AC01N&log$=prottop&blast_rank=29) | splicing factor 3B subunit 2 [Homo sapiens] | [18.5](http://blast.ncbi.nlm.nih.gov/Blast.cgi#55749531%2355749531) | 18.5 | 71% | 338 |
| [NP_775183.1](http://www.ncbi.nlm.nih.gov/entrez/query.fcgi?cmd=Retrieve&db=Protein&list_uids=27764904&dopt=GenPept&RID=T0ACP8AC01N&log$=prottop&blast_rank=30) | FXYD domain containing ion transport regulator 4 precursor [Homo sapiens] | [18.5](http://blast.ncbi.nlm.nih.gov/Blast.cgi#27764904%2327764904) | 18.5 | 71% | 338 |
| [NP_683708.1](http://www.ncbi.nlm.nih.gov/entrez/query.fcgi?cmd=Retrieve&db=Protein&list_uids=22547219&dopt=GenPept&RID=T0ACP8AC01N&log$=prottop&blast_rank=31) | Toll-interleukin 1 receptor domain-containing adaptor protein isoform b [Homo sapiens] | [18.5](http://blast.ncbi.nlm.nih.gov/Blast.cgi#22547219%2322547219) | 18.5 | 71% | 338 |
| [NP_064522.3](http://www.ncbi.nlm.nih.gov/entrez/query.fcgi?cmd=Retrieve&db=Protein&list_uids=46592991&dopt=GenPept&RID=T0ACP8AC01N&log$=prottop&blast_rank=32) | GRIP1 associated protein 1 isoform 1 [Homo sapiens] | [18.5](http://blast.ncbi.nlm.nih.gov/Blast.cgi#46592991%2346592991) | 18.5 | 100% | 338 |
| [NP_060736.1](http://www.ncbi.nlm.nih.gov/entrez/query.fcgi?cmd=Retrieve&db=Protein&list_uids=8922756&dopt=GenPept&RID=T0ACP8AC01N&log$=prottop&blast_rank=33) | transmembrane protein 39A [Homo sapiens] | [18.5](http://blast.ncbi.nlm.nih.gov/Blast.cgi#8922756%238922756) | 18.5 | 71% | 338 |
| [NP_005962.1](http://www.ncbi.nlm.nih.gov/entrez/query.fcgi?cmd=Retrieve&db=Protein&list_uids=5174635&dopt=GenPept&RID=T0ACP8AC01N&log$=prottop&blast_rank=34) | FXYD domain containing ion transport regulator 3 isoform 1 precursor [Homo sapiens] >ref|NP_001129483.1| FXYD domain containing ion transport regulator 3 isoform 1 precursor [Homo sapiens] | [18.5](http://blast.ncbi.nlm.nih.gov/Blast.cgi#5174635%235174635) | 18.5 | 71% | 338 |
| [NP_001034750.1](http://www.ncbi.nlm.nih.gov/entrez/query.fcgi?cmd=Retrieve&db=Protein&list_uids=89111122&dopt=GenPept&RID=T0ACP8AC01N&log$=prottop&blast_rank=35) | Toll-interleukin 1 receptor domain-containing adaptor protein isoform a [Homo sapiens] | [18.5](http://blast.ncbi.nlm.nih.gov/Blast.cgi#89111122%2389111122) | 18.5 | 71% | 338 |
| [NP_006823.1](http://www.ncbi.nlm.nih.gov/entrez/query.fcgi?cmd=Retrieve&db=Protein&list_uids=29789006&dopt=GenPept&RID=T0ACP8AC01N&log$=prottop&blast_rank=36) | fermitin family homolog 2 isoform 1 [Homo sapiens] | [18.5](http://blast.ncbi.nlm.nih.gov/Blast.cgi#29789006%2329789006) | 18.5 | 71% | 338 |
| [NP_001128472.1](http://www.ncbi.nlm.nih.gov/entrez/query.fcgi?cmd=Retrieve&db=Protein&list_uids=201861823&dopt=GenPept&RID=T0ACP8AC01N&log$=prottop&blast_rank=37) | fermitin family homolog 2 isoform 3 [Homo sapiens] | [18.5](http://blast.ncbi.nlm.nih.gov/Blast.cgi#201861823%23201861823) | 34.4 | 85% | 338 |
| [NP_001005158.1](http://www.ncbi.nlm.nih.gov/entrez/query.fcgi?cmd=Retrieve&db=Protein&list_uids=53692191&dopt=GenPept&RID=T0ACP8AC01N&log$=prottop&blast_rank=38) | Scm-like with four mbt domains 1 [Homo sapiens] >ref|NP_057413.2| Scm-like with four mbt domains 1 [Homo sapiens] >ref|NP_001005159.1| Scm-like with four mbt domains 1 [Homo sapiens] | [18.5](http://blast.ncbi.nlm.nih.gov/Blast.cgi#53692191%2353692191) | 18.5 | 85% | 338 |
| [NP_056385.1](http://www.ncbi.nlm.nih.gov/entrez/query.fcgi?cmd=Retrieve&db=Protein&list_uids=17864090&dopt=GenPept&RID=T0ACP8AC01N&log$=prottop&blast_rank=39) | autism susceptibility candidate 2 isoform 1 [Homo sapiens] | [18.5](http://blast.ncbi.nlm.nih.gov/Blast.cgi#17864090%2317864090) | 18.5 | 71% | 338 |
| [XP_002347129.1](http://www.ncbi.nlm.nih.gov/entrez/query.fcgi?cmd=Retrieve&db=Protein&list_uids=239749671&dopt=GenPept&RID=T0ACP8AC01N&log$=prottop&blast_rank=40) | PREDICTED: hypothetical protein XP_002347129 [Homo sapiens] >ref|XP_002346239.1| PREDICTED: hypothetical protein [Homo sapiens] | [18.0](http://blast.ncbi.nlm.nih.gov/Blast.cgi#239749671%23239749671) | 18.0 | 85% | 453 |
| [XP_002342640.1](http://www.ncbi.nlm.nih.gov/entrez/query.fcgi?cmd=Retrieve&db=Protein&list_uids=239742802&dopt=GenPept&RID=T0ACP8AC01N&log$=prottop&blast_rank=41) | PREDICTED: hypothetical protein XP_002342640 [Homo sapiens] >ref|XP_002346798.1| PREDICTED: hypothetical protein XP_002346798 [Homo sapiens] >ref|XP_002345915.1| PREDICTED: hypothetical protein [Homo sapiens] | [18.0](http://blast.ncbi.nlm.nih.gov/Blast.cgi#239742802%23239742802) | 18.0 | 100% | 453 |
| [NP_055798.2](http://www.ncbi.nlm.nih.gov/entrez/query.fcgi?cmd=Retrieve&db=Protein&list_uids=241982802&dopt=GenPept&RID=T0ACP8AC01N&log$=prottop&blast_rank=42) | HMG box domain containing 3 [Homo sapiens] | [18.0](http://blast.ncbi.nlm.nih.gov/Blast.cgi#241982802%23241982802) | 18.0 | 85% | 453 |
| [NP_002926.2](http://www.ncbi.nlm.nih.gov/entrez/query.fcgi?cmd=Retrieve&db=Protein&list_uids=45243507&dopt=GenPept&RID=T0ACP8AC01N&log$=prottop&blast_rank=43) | ribonuclease, RNase A family, 3 (eosinophil cationic protein) precursor [Homo sapiens] | [18.0](http://blast.ncbi.nlm.nih.gov/Blast.cgi#45243507%2345243507) | 18.0 | 85% | 453 |
| [NP_062555.1](http://www.ncbi.nlm.nih.gov/entrez/query.fcgi?cmd=Retrieve&db=Protein&list_uids=9994201&dopt=GenPept&RID=T0ACP8AC01N&log$=prottop&blast_rank=44) | carboxypeptidase X, member 1 precursor [Homo sapiens] | [18.0](http://blast.ncbi.nlm.nih.gov/Blast.cgi#9994201%239994201) | 31.8 | 100% | 453 |
| [NP_001159887.1](http://www.ncbi.nlm.nih.gov/entrez/query.fcgi?cmd=Retrieve&db=Protein&list_uids=261878539&dopt=GenPept&RID=T0ACP8AC01N&log$=prottop&blast_rank=45) | enoyl-Coenzyme A, hydratase/3-hydroxyacyl Coenzyme A dehydrogenase isoform 2 [Homo sapiens] | [17.6](http://blast.ncbi.nlm.nih.gov/Blast.cgi#261878539%23261878539) | 17.6 | 85% | 608 |
| [XP_941694.3](http://www.ncbi.nlm.nih.gov/entrez/query.fcgi?cmd=Retrieve&db=Protein&list_uids=169205253&dopt=GenPept&RID=T0ACP8AC01N&log$=prottop&blast_rank=46) | PREDICTED: hypothetical protein [Homo sapiens] | [17.6](http://blast.ncbi.nlm.nih.gov/Blast.cgi#169205253%23169205253) | 116 | 100% | 608 |
| [NP_149102.3](http://www.ncbi.nlm.nih.gov/entrez/query.fcgi?cmd=Retrieve&db=Protein&list_uids=157278100&dopt=GenPept&RID=T0ACP8AC01N&log$=prottop&blast_rank=47) | phosphonoformate immuno-associated protein 5 isoform 1 [Homo sapiens] | [17.6](http://blast.ncbi.nlm.nih.gov/Blast.cgi#157278100%23157278100) | 17.6 | 100% | 608 |
| [NP_003669.4](http://www.ncbi.nlm.nih.gov/entrez/query.fcgi?cmd=Retrieve&db=Protein&list_uids=50959085&dopt=GenPept&RID=T0ACP8AC01N&log$=prottop&blast_rank=48) | THO complex 5 [Homo sapiens] >ref|NP_001002877.1| THO complex 5 [Homo sapiens] >ref|NP_001002878.1| THO complex 5 [Homo sapiens] >ref|NP_001002879.1| THO complex 5 [Homo sapiens] | [17.6](http://blast.ncbi.nlm.nih.gov/Blast.cgi#50959085%2350959085) | 28.8 | 100% | 608 |
| [NP_079421.5](http://www.ncbi.nlm.nih.gov/entrez/query.fcgi?cmd=Retrieve&db=Protein&list_uids=94681049&dopt=GenPept&RID=T0ACP8AC01N&log$=prottop&blast_rank=49) | hypothetical protein LOC80217 [Homo sapiens] | [17.6](http://blast.ncbi.nlm.nih.gov/Blast.cgi#94681049%2394681049) | 17.6 | 85% | 608 |
| [NP_079011.3](http://www.ncbi.nlm.nih.gov/entrez/query.fcgi?cmd=Retrieve&db=Protein&list_uids=83035136&dopt=GenPept&RID=T0ACP8AC01N&log$=prottop&blast_rank=50) | F-box protein 31 [Homo sapiens] | [17.6](http://blast.ncbi.nlm.nih.gov/Blast.cgi#83035136%2383035136) | 17.6 | 85% | 608 |
| [NP_076973.1](http://www.ncbi.nlm.nih.gov/entrez/query.fcgi?cmd=Retrieve&db=Protein&list_uids=13129048&dopt=GenPept&RID=T0ACP8AC01N&log$=prottop&blast_rank=51) | oligonucleotide/oligosaccharide-binding fold containing 2B [Homo sapiens] | [17.6](http://blast.ncbi.nlm.nih.gov/Blast.cgi#13129048%2313129048) | 17.6 | 100% | 608 |
| [NP_115791.3](http://www.ncbi.nlm.nih.gov/entrez/query.fcgi?cmd=Retrieve&db=Protein&list_uids=157743265&dopt=GenPept&RID=T0ACP8AC01N&log$=prottop&blast_rank=52) | caspase recruitment domain family, member 11 [Homo sapiens] | [17.6](http://blast.ncbi.nlm.nih.gov/Blast.cgi#157743265%23157743265) | 17.6 | 85% | 608 |
| [NP_060903.2](http://www.ncbi.nlm.nih.gov/entrez/query.fcgi?cmd=Retrieve&db=Protein&list_uids=20357522&dopt=GenPept&RID=T0ACP8AC01N&log$=prottop&blast_rank=53) | jumonji domain containing 1A [Homo sapiens] >ref|NP_001140160.1| jumonji domain containing 1A [Homo sapiens] | [17.6](http://blast.ncbi.nlm.nih.gov/Blast.cgi#20357522%2320357522) | 17.6 | 85% | 608 |
| [NP_112241.2](http://www.ncbi.nlm.nih.gov/entrez/query.fcgi?cmd=Retrieve&db=Protein&list_uids=45238849&dopt=GenPept&RID=T0ACP8AC01N&log$=prottop&blast_rank=54) | poly(A) binding protein, cytoplasmic 3 [Homo sapiens] | [17.6](http://blast.ncbi.nlm.nih.gov/Blast.cgi#45238849%2345238849) | 17.6 | 85% | 608 |
| [NP_001957.2](http://www.ncbi.nlm.nih.gov/entrez/query.fcgi?cmd=Retrieve&db=Protein&list_uids=68989263&dopt=GenPept&RID=T0ACP8AC01N&log$=prottop&blast_rank=55) | enoyl-Coenzyme A, hydratase/3-hydroxyacyl Coenzyme A dehydrogenase isoform 1 [Homo sapiens] | [17.6](http://blast.ncbi.nlm.nih.gov/Blast.cgi#68989263%2368989263) | 17.6 | 85% | 608 |
| [NP_057336.3](http://www.ncbi.nlm.nih.gov/entrez/query.fcgi?cmd=Retrieve&db=Protein&list_uids=153792694&dopt=GenPept&RID=T0ACP8AC01N&log$=prottop&blast_rank=56) | baculoviral IAP repeat-containing 6 [Homo sapiens] | [17.2](http://blast.ncbi.nlm.nih.gov/Blast.cgi#153792694%23153792694) | 44.3 | 100% | 816 |
| [NP_705838.3](http://www.ncbi.nlm.nih.gov/entrez/query.fcgi?cmd=Retrieve&db=Protein&list_uids=62241003&dopt=GenPept&RID=T0ACP8AC01N&log$=prottop&blast_rank=57) | cardiomyopathy associated 5 [Homo sapiens] | [17.2](http://blast.ncbi.nlm.nih.gov/Blast.cgi#62241003%2362241003) | 17.2 | 85% | 816 |
| [NP_919269.2](http://www.ncbi.nlm.nih.gov/entrez/query.fcgi?cmd=Retrieve&db=Protein&list_uids=40255272&dopt=GenPept&RID=T0ACP8AC01N&log$=prottop&blast_rank=58) | xin actin-binding repeat containing 1 [Homo sapiens] | [17.2](http://blast.ncbi.nlm.nih.gov/Blast.cgi#40255272%2340255272) | 17.2 | 85% | 816 |
| [NP_001128571.1](http://www.ncbi.nlm.nih.gov/entrez/query.fcgi?cmd=Retrieve&db=Protein&list_uids=227499990&dopt=GenPept&RID=T0ACP8AC01N&log$=prottop&blast_rank=59) | transmembrane protease, serine 2 isoform 1 [Homo sapiens] | [16.8](http://blast.ncbi.nlm.nih.gov/Blast.cgi#227499990%23227499990) | 16.8 | 71% | 1094 |
| [NP_004561.3](http://www.ncbi.nlm.nih.gov/entrez/query.fcgi?cmd=Retrieve&db=Protein&list_uids=194353959&dopt=GenPept&RID=T0ACP8AC01N&log$=prottop&blast_rank=60) | phosphoinositide-3-kinase, class 2 gamma polypeptide [Homo sapiens] | [16.8](http://blast.ncbi.nlm.nih.gov/Blast.cgi#194353959%23194353959) | 16.8 | 85% | 1094 |
| [NP_078858.4](http://www.ncbi.nlm.nih.gov/entrez/query.fcgi?cmd=Retrieve&db=Protein&list_uids=165932370&dopt=GenPept&RID=T0ACP8AC01N&log$=prottop&blast_rank=61) | FAT tumor suppressor homolog 4 precursor [Homo sapiens] | [16.8](http://blast.ncbi.nlm.nih.gov/Blast.cgi#165932370%23165932370) | 16.8 | 85% | 1094 |
| [NP_001103984.1](http://www.ncbi.nlm.nih.gov/entrez/query.fcgi?cmd=Retrieve&db=Protein&list_uids=160333411&dopt=GenPept&RID=T0ACP8AC01N&log$=prottop&blast_rank=62) | early B-cell factor 4 [Homo sapiens] | [16.8](http://blast.ncbi.nlm.nih.gov/Blast.cgi#160333411%23160333411) | 16.8 | 71% | 1094 |
| [NP_056280.2](http://www.ncbi.nlm.nih.gov/entrez/query.fcgi?cmd=Retrieve&db=Protein&list_uids=157739942&dopt=GenPept&RID=T0ACP8AC01N&log$=prottop&blast_rank=63) | gemin 5 [Homo sapiens] | [16.8](http://blast.ncbi.nlm.nih.gov/Blast.cgi#157739942%23157739942) | 16.8 | 100% | 1094 |
| [NP_001075019.1](http://www.ncbi.nlm.nih.gov/entrez/query.fcgi?cmd=Retrieve&db=Protein&list_uids=125656165&dopt=GenPept&RID=T0ACP8AC01N&log$=prottop&blast_rank=64) | THO complex 2 [Homo sapiens] | [16.8](http://blast.ncbi.nlm.nih.gov/Blast.cgi#125656165%23125656165) | 16.8 | 71% | 1094 |
| [NP_003234.2](http://www.ncbi.nlm.nih.gov/entrez/query.fcgi?cmd=Retrieve&db=Protein&list_uids=56682966&dopt=GenPept&RID=T0ACP8AC01N&log$=prottop&blast_rank=65) | transforming growth factor, beta receptor III [Homo sapiens] | [16.8](http://blast.ncbi.nlm.nih.gov/Blast.cgi#56682966%2356682966) | 16.8 | 71% | 1094 |
| [NP_073150.2](http://www.ncbi.nlm.nih.gov/entrez/query.fcgi?cmd=Retrieve&db=Protein&list_uids=113930703&dopt=GenPept&RID=T0ACP8AC01N&log$=prottop&blast_rank=66) | early B-cell factor 2 [Homo sapiens] | [16.8](http://blast.ncbi.nlm.nih.gov/Blast.cgi#113930703%23113930703) | 16.8 | 71% | 1094 |
| [NP_055602.1](http://www.ncbi.nlm.nih.gov/entrez/query.fcgi?cmd=Retrieve&db=Protein&list_uids=7662146&dopt=GenPept&RID=T0ACP8AC01N&log$=prottop&blast_rank=67) | DnaJ (Hsp40) homolog, subfamily C, member 6 [Homo sapiens] | [16.8](http://blast.ncbi.nlm.nih.gov/Blast.cgi#7662146%237662146) | 16.8 | 71% | 1094 |
| [NP_115597.3](http://www.ncbi.nlm.nih.gov/entrez/query.fcgi?cmd=Retrieve&db=Protein&list_uids=29244924&dopt=GenPept&RID=T0ACP8AC01N&log$=prottop&blast_rank=68) | chromodomain helicase DNA binding protein 6 [Homo sapiens] | [16.8](http://blast.ncbi.nlm.nih.gov/Blast.cgi#29244924%2329244924) | 16.8 | 71% | 1094 |
| [NP_006629.2](http://www.ncbi.nlm.nih.gov/entrez/query.fcgi?cmd=Retrieve&db=Protein&list_uids=55743132&dopt=GenPept&RID=T0ACP8AC01N&log$=prottop&blast_rank=69) | ribonuclease P 40kDa subunit [Homo sapiens] | [16.8](http://blast.ncbi.nlm.nih.gov/Blast.cgi#55743132%2355743132) | 16.8 | 71% | 1094 |
| [NP_001026886.1](http://www.ncbi.nlm.nih.gov/entrez/query.fcgi?cmd=Retrieve&db=Protein&list_uids=72534722&dopt=GenPept&RID=T0ACP8AC01N&log$=prottop&blast_rank=70) | oligonucleotide/oligosaccharide-binding fold containing 2A [Homo sapiens] | [16.8](http://blast.ncbi.nlm.nih.gov/Blast.cgi#72534722%2372534722) | 16.8 | 85% | 1094 |
| [NP_001005463.1](http://www.ncbi.nlm.nih.gov/entrez/query.fcgi?cmd=Retrieve&db=Protein&list_uids=53828926&dopt=GenPept&RID=T0ACP8AC01N&log$=prottop&blast_rank=71) | early B-cell factor 3 [Homo sapiens] | [16.8](http://blast.ncbi.nlm.nih.gov/Blast.cgi#53828926%2353828926) | 16.8 | 71% | 1094 |
| [NP_060242.2](http://www.ncbi.nlm.nih.gov/entrez/query.fcgi?cmd=Retrieve&db=Protein&list_uids=40068063&dopt=GenPept&RID=T0ACP8AC01N&log$=prottop&blast_rank=72) | TBC1 domain family, member 22B [Homo sapiens] | [16.8](http://blast.ncbi.nlm.nih.gov/Blast.cgi#40068063%2340068063) | 16.8 | 71% | 1094 |
| [NP_057125.2](http://www.ncbi.nlm.nih.gov/entrez/query.fcgi?cmd=Retrieve&db=Protein&list_uids=31455614&dopt=GenPept&RID=T0ACP8AC01N&log$=prottop&blast_rank=73) | Der1-like domain family, member 2 [Homo sapiens] | [16.8](http://blast.ncbi.nlm.nih.gov/Blast.cgi#31455614%2331455614) | 16.8 | 71% | 1094 |
| [NP_060616.1](http://www.ncbi.nlm.nih.gov/entrez/query.fcgi?cmd=Retrieve&db=Protein&list_uids=8922534&dopt=GenPept&RID=T0ACP8AC01N&log$=prottop&blast_rank=74) | RNA methyltransferase like 1 [Homo sapiens] | [16.8](http://blast.ncbi.nlm.nih.gov/Blast.cgi#8922534%238922534) | 16.8 | 71% | 1094 |
| [NP_077287.1](http://www.ncbi.nlm.nih.gov/entrez/query.fcgi?cmd=Retrieve&db=Protein&list_uids=13236549&dopt=GenPept&RID=T0ACP8AC01N&log$=prottop&blast_rank=75) | major facilitator superfamily domain containing 11 [Homo sapiens] | [16.8](http://blast.ncbi.nlm.nih.gov/Blast.cgi#13236549%2313236549) | 16.8 | 71% | 1094 |
| [NP_612808.1](http://www.ncbi.nlm.nih.gov/entrez/query.fcgi?cmd=Retrieve&db=Protein&list_uids=21040334&dopt=GenPept&RID=T0ACP8AC01N&log$=prottop&blast_rank=76) | B-cell CLL/lymphoma 11B isoform 1 [Homo sapiens] | [16.8](http://blast.ncbi.nlm.nih.gov/Blast.cgi#21040334%2321040334) | 16.8 | 71% | 1094 |
| [NP_115909.1](http://www.ncbi.nlm.nih.gov/entrez/query.fcgi?cmd=Retrieve&db=Protein&list_uids=14249738&dopt=GenPept&RID=T0ACP8AC01N&log$=prottop&blast_rank=77) | N-acetylglucosamine-1-phosphotransferase, gamma subunit precursor [Homo sapiens] | [16.8](http://blast.ncbi.nlm.nih.gov/Blast.cgi#14249738%2314249738) | 16.8 | 71% | 1094 |
| [NP_055161.1](http://www.ncbi.nlm.nih.gov/entrez/query.fcgi?cmd=Retrieve&db=Protein&list_uids=22507409&dopt=GenPept&RID=T0ACP8AC01N&log$=prottop&blast_rank=78) | TBC1 domain family, member 22A [Homo sapiens] | [16.8](http://blast.ncbi.nlm.nih.gov/Blast.cgi#22507409%2322507409) | 16.8 | 71% | 1094 |
| [NP_004138.1](http://www.ncbi.nlm.nih.gov/entrez/query.fcgi?cmd=Retrieve&db=Protein&list_uids=4758796&dopt=GenPept&RID=T0ACP8AC01N&log$=prottop&blast_rank=79) | developmentally regulated GTP binding protein 1 [Homo sapiens] | [16.8](http://blast.ncbi.nlm.nih.gov/Blast.cgi#4758796%234758796) | 16.8 | 71% | 1094 |
| [NP_001630.1](http://www.ncbi.nlm.nih.gov/entrez/query.fcgi?cmd=Retrieve&db=Protein&list_uids=4502133&dopt=GenPept&RID=T0ACP8AC01N&log$=prottop&blast_rank=80) | serum amyloid P component precursor [Homo sapiens] | [16.8](http://blast.ncbi.nlm.nih.gov/Blast.cgi#4502133%234502133) | 16.8 | 71% | 1094 |
| [NP_005647.3](http://www.ncbi.nlm.nih.gov/entrez/query.fcgi?cmd=Retrieve&db=Protein&list_uids=205360943&dopt=GenPept&RID=T0ACP8AC01N&log$=prottop&blast_rank=81) | transmembrane protease, serine 2 isoform 2 [Homo sapiens] | [16.8](http://blast.ncbi.nlm.nih.gov/Blast.cgi#205360943%23205360943) | 16.8 | 71% | 1094 |
| [NP_075049.1](http://www.ncbi.nlm.nih.gov/entrez/query.fcgi?cmd=Retrieve&db=Protein&list_uids=12597635&dopt=GenPept&RID=T0ACP8AC01N&log$=prottop&blast_rank=82) | B-cell CLL/lymphoma 11B isoform 2 [Homo sapiens] | [16.8](http://blast.ncbi.nlm.nih.gov/Blast.cgi#12597635%2312597635) | 16.8 | 71% | 1094 |
| [NP_000187.3](http://www.ncbi.nlm.nih.gov/entrez/query.fcgi?cmd=Retrieve&db=Protein&list_uids=119392083&dopt=GenPept&RID=T0ACP8AC01N&log$=prottop&blast_rank=83) | corticosteroid 11-beta-dehydrogenase isozyme 2 [Homo sapiens] | [16.8](http://blast.ncbi.nlm.nih.gov/Blast.cgi#119392083%23119392083) | 16.8 | 100% | 1094 |
| [NP_037513.1](http://www.ncbi.nlm.nih.gov/entrez/query.fcgi?cmd=Retrieve&db=Protein&list_uids=7019561&dopt=GenPept&RID=T0ACP8AC01N&log$=prottop&blast_rank=84) | thyrotropin-releasing hormone degrading enzyme [Homo sapiens] | [16.8](http://blast.ncbi.nlm.nih.gov/Blast.cgi#7019561%237019561) | 16.8 | 71% | 1094 |
| [NP_076870.1](http://www.ncbi.nlm.nih.gov/entrez/query.fcgi?cmd=Retrieve&db=Protein&list_uids=31415878&dopt=GenPept&RID=T0ACP8AC01N&log$=prottop&blast_rank=85) | early B-cell factor [Homo sapiens] | [16.8](http://blast.ncbi.nlm.nih.gov/Blast.cgi#31415878%2331415878) | 16.8 | 71% | 1094 |
| [NP_001159755.1](http://www.ncbi.nlm.nih.gov/entrez/query.fcgi?cmd=Retrieve&db=Protein&list_uids=261878463&dopt=GenPept&RID=T0ACP8AC01N&log$=prottop&blast_rank=86) | RGM domain family, member A isoform 1 [Homo sapiens] | [16.3](http://blast.ncbi.nlm.nih.gov/Blast.cgi#261878463%23261878463) | 16.3 | 100% | 1468 |
| [NP_079044.2](http://www.ncbi.nlm.nih.gov/entrez/query.fcgi?cmd=Retrieve&db=Protein&list_uids=228008351&dopt=GenPept&RID=T0ACP8AC01N&log$=prottop&blast_rank=87) | coiled-coil domain containing 48 [Homo sapiens] | [16.3](http://blast.ncbi.nlm.nih.gov/Blast.cgi#228008351%23228008351) | 16.3 | 71% | 1468 |
| [NP_001012779.2](http://www.ncbi.nlm.nih.gov/entrez/query.fcgi?cmd=Retrieve&db=Protein&list_uids=224589135&dopt=GenPept&RID=T0ACP8AC01N&log$=prottop&blast_rank=88) | RGM domain family, member B [Homo sapiens] | [16.3](http://blast.ncbi.nlm.nih.gov/Blast.cgi#224589135%23224589135) | 16.3 | 100% | 1468 |
| [NP_001129602.1](http://www.ncbi.nlm.nih.gov/entrez/query.fcgi?cmd=Retrieve&db=Protein&list_uids=209915573&dopt=GenPept&RID=T0ACP8AC01N&log$=prottop&blast_rank=89) | amyloid beta A4 protein isoform f precursor [Homo sapiens] | [16.3](http://blast.ncbi.nlm.nih.gov/Blast.cgi#209915573%23209915573) | 16.3 | 85% | 1468 |
| [NP_001009999.1](http://www.ncbi.nlm.nih.gov/entrez/query.fcgi?cmd=Retrieve&db=Protein&list_uids=58761544&dopt=GenPept&RID=T0ACP8AC01N&log$=prottop&blast_rank=90) | lysine-specific histone demethylase 1 isoform a [Homo sapiens] | [16.3](http://blast.ncbi.nlm.nih.gov/Blast.cgi#58761544%2358761544) | 16.3 | 85% | 1468 |
| [NP_056078.2](http://www.ncbi.nlm.nih.gov/entrez/query.fcgi?cmd=Retrieve&db=Protein&list_uids=119120894&dopt=GenPept&RID=T0ACP8AC01N&log$=prottop&blast_rank=91) | Dmx-like 2 [Homo sapiens] | [16.3](http://blast.ncbi.nlm.nih.gov/Blast.cgi#119120894%23119120894) | 16.3 | 85% | 1468 |
| [NP_001036188.1](http://www.ncbi.nlm.nih.gov/entrez/query.fcgi?cmd=Retrieve&db=Protein&list_uids=113204617&dopt=GenPept&RID=T0ACP8AC01N&log$=prottop&blast_rank=92) | skeletal muscle ryanodine receptor isoform 2 [Homo sapiens] | [16.3](http://blast.ncbi.nlm.nih.gov/Blast.cgi#113204617%23113204617) | 32.2 | 85% | 1468 |
| [NP_000531.2](http://www.ncbi.nlm.nih.gov/entrez/query.fcgi?cmd=Retrieve&db=Protein&list_uids=113204615&dopt=GenPept&RID=T0ACP8AC01N&log$=prottop&blast_rank=93) | skeletal muscle ryanodine receptor isoform 1 [Homo sapiens] | [16.3](http://blast.ncbi.nlm.nih.gov/Blast.cgi#113204615%23113204615) | 32.2 | 85% | 1468 |
| [NP_733751.2](http://www.ncbi.nlm.nih.gov/entrez/query.fcgi?cmd=Retrieve&db=Protein&list_uids=91718902&dopt=GenPept&RID=T0ACP8AC01N&log$=prottop&blast_rank=94) | myeloid/lymphoid or mixed-lineage leukemia 3 [Homo sapiens] | [16.3](http://blast.ncbi.nlm.nih.gov/Blast.cgi#91718902%2391718902) | 16.3 | 85% | 1468 |
| [NP_001093250.1](http://www.ncbi.nlm.nih.gov/entrez/query.fcgi?cmd=Retrieve&db=Protein&list_uids=153791216&dopt=GenPept&RID=T0ACP8AC01N&log$=prottop&blast_rank=95) | proteasome beta 11 subunit precursor [Homo sapiens] | [16.3](http://blast.ncbi.nlm.nih.gov/Blast.cgi#153791216%23153791216) | 16.3 | 71% | 1468 |
| [NP_060034.9](http://www.ncbi.nlm.nih.gov/entrez/query.fcgi?cmd=Retrieve&db=Protein&list_uids=61743980&dopt=GenPept&RID=T0ACP8AC01N&log$=prottop&blast_rank=96) | stabilin 2 precursor [Homo sapiens] | [16.3](http://blast.ncbi.nlm.nih.gov/Blast.cgi#61743980%2361743980) | 30.1 | 100% | 1468 |
| [NP_775896.3](http://www.ncbi.nlm.nih.gov/entrez/query.fcgi?cmd=Retrieve&db=Protein&list_uids=45580704&dopt=GenPept&RID=T0ACP8AC01N&log$=prottop&blast_rank=97) | hypothetical protein LOC284099 [Homo sapiens] | [16.3](http://blast.ncbi.nlm.nih.gov/Blast.cgi#45580704%2345580704) | 16.3 | 71% | 1468 |
| [NP_001129601.1](http://www.ncbi.nlm.nih.gov/entrez/query.fcgi?cmd=Retrieve&db=Protein&list_uids=209915570&dopt=GenPept&RID=T0ACP8AC01N&log$=prottop&blast_rank=98) | amyloid beta A4 protein isoform e precursor [Homo sapiens] | [16.3](http://blast.ncbi.nlm.nih.gov/Blast.cgi#209915570%23209915570) | 16.3 | 85% | 1468 |
| [NP_060084.2](http://www.ncbi.nlm.nih.gov/entrez/query.fcgi?cmd=Retrieve&db=Protein&list_uids=13162290&dopt=GenPept&RID=T0ACP8AC01N&log$=prottop&blast_rank=99) | betaine-homocysteine methyltransferase 2 [Homo sapiens] | [16.3](http://blast.ncbi.nlm.nih.gov/Blast.cgi#13162290%2313162290) | 16.3 | 71% | 1468 |
| [NP_078893.2](http://www.ncbi.nlm.nih.gov/entrez/query.fcgi?cmd=Retrieve&db=Protein&list_uids=58331272&dopt=GenPept&RID=T0ACP8AC01N&log$=prottop&blast_rank=100) | zinc finger, CCHC domain containing 6 [Homo sapiens] | [16.3](http://blast.ncbi.nlm.nih.gov/Blast.cgi#58331272%2358331272) | 16.3 | 71% | 1468 |

| **Accession** | **Proteins with a match to AEPPFEF peptide** | **[Max score](http://blast.ncbi.nlm.nih.gov/Blast.cgi?CMD=Get&ALIGNMENTS=100&ALIGNMENT_VIEW=Pairwise&CDD_SEARCH_STATE=1&DATABASE_SORT=0&DESCRIPTIONS=100&ENTREZ_QUERY=txid9606 %5BORGN%5D&FIRST_QUERY_NUM=0&FORMAT_OBJECT=Alignment&FORMAT_PAGE_TARGET=&FORMAT_TYPE=HTML&GET_SEQUENCE=yes&I_THRESH=&MASK_CHAR=2&MASK_COLOR=1&NEW_DESIGN=on&NEW_VIEW=yes&NUM_OVERVIEW=100&OLD_BLAST=false&PAGE=Proteins&QUERY_INDEX=0&QUERY_NUMBER=0&RESULTS_PAGE_TARGET=&RID=T0AP12RA01N&SHOW_LINKOUT=yes&SHOW_OVERVIEW=yes&STEP_NUMBER=&WORD_SIZE=2&DISPLAY_SORT=1&HSP_SORT=1" \l "sort_mark)** | **[Total score](http://blast.ncbi.nlm.nih.gov/Blast.cgi?CMD=Get&ALIGNMENTS=100&ALIGNMENT_VIEW=Pairwise&CDD_SEARCH_STATE=1&DATABASE_SORT=0&DESCRIPTIONS=100&ENTREZ_QUERY=txid9606 %5BORGN%5D&FIRST_QUERY_NUM=0&FORMAT_OBJECT=Alignment&FORMAT_PAGE_TARGET=&FORMAT_TYPE=HTML&GET_SEQUENCE=yes&I_THRESH=&MASK_CHAR=2&MASK_COLOR=1&NEW_DESIGN=on&NEW_VIEW=yes&NUM_OVERVIEW=100&OLD_BLAST=false&PAGE=Proteins&QUERY_INDEX=0&QUERY_NUMBER=0&RESULTS_PAGE_TARGET=&RID=T0AP12RA01N&SHOW_LINKOUT=yes&SHOW_OVERVIEW=yes&STEP_NUMBER=&WORD_SIZE=2&DISPLAY_SORT=2&HSP_SORT=1" \l "sort_mark)** | **[Query coverage](http://blast.ncbi.nlm.nih.gov/Blast.cgi?CMD=Get&ALIGNMENTS=100&ALIGNMENT_VIEW=Pairwise&CDD_SEARCH_STATE=1&DATABASE_SORT=0&DESCRIPTIONS=100&ENTREZ_QUERY=txid9606 %5BORGN%5D&FIRST_QUERY_NUM=0&FORMAT_OBJECT=Alignment&FORMAT_PAGE_TARGET=&FORMAT_TYPE=HTML&GET_SEQUENCE=yes&I_THRESH=&MASK_CHAR=2&MASK_COLOR=1&NEW_DESIGN=on&NEW_VIEW=yes&NUM_OVERVIEW=100&OLD_BLAST=false&PAGE=Proteins&QUERY_INDEX=0&QUERY_NUMBER=0&RESULTS_PAGE_TARGET=&RID=T0AP12RA01N&SHOW_LINKOUT=yes&SHOW_OVERVIEW=yes&STEP_NUMBER=&WORD_SIZE=2&DISPLAY_SORT=4&HSP_SORT=0" \l "sort_mark)** | **[E value](http://blast.ncbi.nlm.nih.gov/Blast.cgi?CMD=Get&ALIGNMENTS=100&ALIGNMENT_VIEW=Pairwise&CDD_SEARCH_STATE=1&DATABASE_SORT=0&DESCRIPTIONS=100&ENTREZ_QUERY=txid9606 %5BORGN%5D&FIRST_QUERY_NUM=0&FORMAT_OBJECT=Alignment&FORMAT_PAGE_TARGET=&FORMAT_TYPE=HTML&GET_SEQUENCE=yes&I_THRESH=&MASK_CHAR=2&MASK_COLOR=1&NEW_DESIGN=on&NEW_VIEW=yes&NUM_OVERVIEW=100&OLD_BLAST=false&PAGE=Proteins&QUERY_INDEX=0&QUERY_NUMBER=0&RESULTS_PAGE_TARGET=&RID=T0AP12RA01N&SHOW_LINKOUT=yes&SHOW_OVERVIEW=yes&STEP_NUMBER=&WORD_SIZE=2&DISPLAY_SORT=0&HSP_SORT=0" \l "sort_mark)** |
| --- | --- | --- | --- | --- | --- |
| [NP_835455.1](http://www.ncbi.nlm.nih.gov/entrez/query.fcgi?cmd=Retrieve&db=Protein&list_uids=30039710&dopt=GenPept&RID=T0AP12RA01N&log$=prottop&blast_rank=1) | pancreas specific transcription factor, 1a [Homo sapiens] | [24.4](http://blast.ncbi.nlm.nih.gov/Blast.cgi#30039710%2330039710) | 24.4 | 85% | 5.5 |
| [NP_061887.2](http://www.ncbi.nlm.nih.gov/entrez/query.fcgi?cmd=Retrieve&db=Protein&list_uids=33469941&dopt=GenPept&RID=T0AP12RA01N&log$=prottop&blast_rank=2) | RNA polymerase I polypeptide B isoform 1 [Homo sapiens] | [21.4](http://blast.ncbi.nlm.nih.gov/Blast.cgi#33469941%2333469941) | 21.4 | 100% | 43 |
| [NP_940857.2](http://www.ncbi.nlm.nih.gov/entrez/query.fcgi?cmd=Retrieve&db=Protein&list_uids=134031945&dopt=GenPept&RID=T0AP12RA01N&log$=prottop&blast_rank=3) | SCO-spondin precursor [Homo sapiens] | [21.0](http://blast.ncbi.nlm.nih.gov/Blast.cgi#134031945%23134031945) | 38.6 | 71% | 58 |
| [NP_878918.2](http://www.ncbi.nlm.nih.gov/entrez/query.fcgi?cmd=Retrieve&db=Protein&list_uids=118918407&dopt=GenPept&RID=T0AP12RA01N&log$=prottop&blast_rank=4) | spectrin repeat containing, nuclear envelope 2 isoform 5 [Homo sapiens] | [20.6](http://blast.ncbi.nlm.nih.gov/Blast.cgi#118918407%23118918407) | 20.6 | 71% | 78 |
| [NP_055995.4](http://www.ncbi.nlm.nih.gov/entrez/query.fcgi?cmd=Retrieve&db=Protein&list_uids=118918403&dopt=GenPept&RID=T0AP12RA01N&log$=prottop&blast_rank=5) | spectrin repeat containing, nuclear envelope 2 isoform 1 [Homo sapiens] | [20.6](http://blast.ncbi.nlm.nih.gov/Blast.cgi#118918403%23118918403) | 20.6 | 71% | 78 |
| [NP_997724.2](http://www.ncbi.nlm.nih.gov/entrez/query.fcgi?cmd=Retrieve&db=Protein&list_uids=237874249&dopt=GenPept&RID=T0AP12RA01N&log$=prottop&blast_rank=6) | XK, Kell blood group complex subunit-related, X-linked [Homo sapiens] | [20.6](http://blast.ncbi.nlm.nih.gov/Blast.cgi#237874249%23237874249) | 20.6 | 71% | 78 |
| [NP_001138466.1](http://www.ncbi.nlm.nih.gov/entrez/query.fcgi?cmd=Retrieve&db=Protein&list_uids=222418604&dopt=GenPept&RID=T0AP12RA01N&log$=prottop&blast_rank=7) | hypothetical protein LOC257407 [Homo sapiens] | [20.2](http://blast.ncbi.nlm.nih.gov/Blast.cgi#222418604%23222418604) | 20.2 | 85% | 104 |
| [NP_114129.1](http://www.ncbi.nlm.nih.gov/entrez/query.fcgi?cmd=Retrieve&db=Protein&list_uids=151301171&dopt=GenPept&RID=T0AP12RA01N&log$=prottop&blast_rank=8) | RNA polymerase II transcription factor TAFII140 [Homo sapiens] | [20.2](http://blast.ncbi.nlm.nih.gov/Blast.cgi#151301171%23151301171) | 20.2 | 85% | 104 |
| [NP_653296.2](http://www.ncbi.nlm.nih.gov/entrez/query.fcgi?cmd=Retrieve&db=Protein&list_uids=124244088&dopt=GenPept&RID=T0AP12RA01N&log$=prottop&blast_rank=9) | Bro1-domain-containing protein [Homo sapiens] | [20.2](http://blast.ncbi.nlm.nih.gov/Blast.cgi#124244088%23124244088) | 20.2 | 85% | 104 |
| [NP_061756.1](http://www.ncbi.nlm.nih.gov/entrez/query.fcgi?cmd=Retrieve&db=Protein&list_uids=11036654&dopt=GenPept&RID=T0AP12RA01N&log$=prottop&blast_rank=10) | protocadherin beta 13 precursor [Homo sapiens] | [20.2](http://blast.ncbi.nlm.nih.gov/Blast.cgi#11036654%2311036654) | 20.2 | 85% | 104 |
| [NP_061993.1](http://www.ncbi.nlm.nih.gov/entrez/query.fcgi?cmd=Retrieve&db=Protein&list_uids=11276081&dopt=GenPept&RID=T0AP12RA01N&log$=prottop&blast_rank=11) | protocadherin beta 8 precursor [Homo sapiens] | [20.2](http://blast.ncbi.nlm.nih.gov/Blast.cgi#11276081%2311276081) | 20.2 | 85% | 104 |
| [NP_001138995.1](http://www.ncbi.nlm.nih.gov/entrez/query.fcgi?cmd=Retrieve&db=Protein&list_uids=224809476&dopt=GenPept&RID=T0AP12RA01N&log$=prottop&blast_rank=12) | retinoic acid induced 14 isoform c [Homo sapiens] | [19.7](http://blast.ncbi.nlm.nih.gov/Blast.cgi#224809476%23224809476) | 19.7 | 71% | 140 |
| [NP_001138994.1](http://www.ncbi.nlm.nih.gov/entrez/query.fcgi?cmd=Retrieve&db=Protein&list_uids=224809474&dopt=GenPept&RID=T0AP12RA01N&log$=prottop&blast_rank=13) | retinoic acid induced 14 isoform b [Homo sapiens] | [19.7](http://blast.ncbi.nlm.nih.gov/Blast.cgi#224809474%23224809474) | 19.7 | 71% | 140 |
| [NP_149123.2](http://www.ncbi.nlm.nih.gov/entrez/query.fcgi?cmd=Retrieve&db=Protein&list_uids=118766337&dopt=GenPept&RID=T0AP12RA01N&log$=prottop&blast_rank=14) | zinc finger protein of the cerebellum 5 [Homo sapiens] | [19.7](http://blast.ncbi.nlm.nih.gov/Blast.cgi#118766337%23118766337) | 19.7 | 71% | 140 |
| [NP_056392.2](http://www.ncbi.nlm.nih.gov/entrez/query.fcgi?cmd=Retrieve&db=Protein&list_uids=224809468&dopt=GenPept&RID=T0AP12RA01N&log$=prottop&blast_rank=15) | retinoic acid induced 14 isoform a [Homo sapiens] >ref|NP_001138992.1| retinoic acid induced 14 isoform a [Homo sapiens] >ref|NP_001138993.1| retinoic acid induced 14 isoform a [Homo sapiens] | [19.7](http://blast.ncbi.nlm.nih.gov/Blast.cgi#224809468%23224809468) | 19.7 | 71% | 140 |
| [NP_001138997.1](http://www.ncbi.nlm.nih.gov/entrez/query.fcgi?cmd=Retrieve&db=Protein&list_uids=224809478&dopt=GenPept&RID=T0AP12RA01N&log$=prottop&blast_rank=16) | retinoic acid induced 14 isoform d [Homo sapiens] | [19.7](http://blast.ncbi.nlm.nih.gov/Blast.cgi#224809478%23224809478) | 19.7 | 71% | 140 |
| [NP_056649.1](http://www.ncbi.nlm.nih.gov/entrez/query.fcgi?cmd=Retrieve&db=Protein&list_uids=7669479&dopt=GenPept&RID=T0AP12RA01N&log$=prottop&blast_rank=17) | RNA-specific adenosine deaminase B1 isoform 3 [Homo sapiens] | [19.7](http://blast.ncbi.nlm.nih.gov/Blast.cgi#7669479%237669479) | 19.7 | 71% | 140 |
| [NP_001153702.1](http://www.ncbi.nlm.nih.gov/entrez/query.fcgi?cmd=Retrieve&db=Protein&list_uids=237681091&dopt=GenPept&RID=T0AP12RA01N&log$=prottop&blast_rank=18) | RNA-specific adenosine deaminase B1 isoform 7 [Homo sapiens] | [19.7](http://blast.ncbi.nlm.nih.gov/Blast.cgi#237681091%23237681091) | 19.7 | 71% | 140 |
| [NP_001070965.1](http://www.ncbi.nlm.nih.gov/entrez/query.fcgi?cmd=Retrieve&db=Protein&list_uids=117320543&dopt=GenPept&RID=T0AP12RA01N&log$=prottop&blast_rank=19) | proline-rich protein 3 isoform b [Homo sapiens] | [19.7](http://blast.ncbi.nlm.nih.gov/Blast.cgi#117320543%23117320543) | 19.7 | 71% | 140 |
| [NP_079539.2](http://www.ncbi.nlm.nih.gov/entrez/query.fcgi?cmd=Retrieve&db=Protein&list_uids=117320535&dopt=GenPept&RID=T0AP12RA01N&log$=prottop&blast_rank=20) | proline-rich protein 3 isoform a [Homo sapiens] | [19.7](http://blast.ncbi.nlm.nih.gov/Blast.cgi#117320535%23117320535) | 19.7 | 71% | 140 |
| [NP_056648.1](http://www.ncbi.nlm.nih.gov/entrez/query.fcgi?cmd=Retrieve&db=Protein&list_uids=7669477&dopt=GenPept&RID=T0AP12RA01N&log$=prottop&blast_rank=21) | RNA-specific adenosine deaminase B1 isoform 2 [Homo sapiens] | [19.7](http://blast.ncbi.nlm.nih.gov/Blast.cgi#7669477%237669477) | 19.7 | 71% | 140 |
| [NP_001103.1](http://www.ncbi.nlm.nih.gov/entrez/query.fcgi?cmd=Retrieve&db=Protein&list_uids=4501919&dopt=GenPept&RID=T0AP12RA01N&log$=prottop&blast_rank=22) | RNA-specific adenosine deaminase B1 isoform 1 [Homo sapiens] | [19.7](http://blast.ncbi.nlm.nih.gov/Blast.cgi#4501919%234501919) | 19.7 | 71% | 140 |
| [NP_003595.1](http://www.ncbi.nlm.nih.gov/entrez/query.fcgi?cmd=Retrieve&db=Protein&list_uids=4504733&dopt=GenPept&RID=T0AP12RA01N&log$=prottop&blast_rank=23) | insulin receptor substrate 4 [Homo sapiens] | [19.7](http://blast.ncbi.nlm.nih.gov/Blast.cgi#4504733%234504733) | 19.7 | 71% | 140 |
| [NP_005900.2](http://www.ncbi.nlm.nih.gov/entrez/query.fcgi?cmd=Retrieve&db=Protein&list_uids=153945728&dopt=GenPept&RID=T0AP12RA01N&log$=prottop&blast_rank=24) | microtubule-associated protein 1B [Homo sapiens] | [18.9](http://blast.ncbi.nlm.nih.gov/Blast.cgi#153945728%23153945728) | 18.9 | 85% | 252 |
| [NP_009297.2](http://www.ncbi.nlm.nih.gov/entrez/query.fcgi?cmd=Retrieve&db=Protein&list_uids=62362412&dopt=GenPept&RID=T0AP12RA01N&log$=prottop&blast_rank=25) | c-abl oncogene 1, receptor tyrosine kinase isoform b [Homo sapiens] | [18.9](http://blast.ncbi.nlm.nih.gov/Blast.cgi#62362412%2362362412) | 18.9 | 85% | 252 |
| [NP_742068.1](http://www.ncbi.nlm.nih.gov/entrez/query.fcgi?cmd=Retrieve&db=Protein&list_uids=73695473&dopt=GenPept&RID=T0AP12RA01N&log$=prottop&blast_rank=26) | roquin [Homo sapiens] | [18.9](http://blast.ncbi.nlm.nih.gov/Blast.cgi#73695473%2373695473) | 18.9 | 85% | 252 |
| [NP_005148.2](http://www.ncbi.nlm.nih.gov/entrez/query.fcgi?cmd=Retrieve&db=Protein&list_uids=62362414&dopt=GenPept&RID=T0AP12RA01N&log$=prottop&blast_rank=27) | c-abl oncogene 1, receptor tyrosine kinase isoform a [Homo sapiens] | [18.9](http://blast.ncbi.nlm.nih.gov/Blast.cgi#62362414%2362362414) | 18.9 | 85% | 252 |
| [NP_001165948.1](http://www.ncbi.nlm.nih.gov/entrez/query.fcgi?cmd=Retrieve&db=Protein&list_uids=289177074&dopt=GenPept&RID=T0AP12RA01N&log$=prottop&blast_rank=28) | ribonucleotide reductase M2 B (TP53 inducible) isoform 2 [Homo sapiens] | [18.5](http://blast.ncbi.nlm.nih.gov/Blast.cgi#289177074%23289177074) | 18.5 | 100% | 338 |
| [NP_001165291.1](http://www.ncbi.nlm.nih.gov/entrez/query.fcgi?cmd=Retrieve&db=Protein&list_uids=284807159&dopt=GenPept&RID=T0AP12RA01N&log$=prottop&blast_rank=29) | peroxisome proliferative activated receptor, delta isoform 4 [Homo sapiens] | [18.5](http://blast.ncbi.nlm.nih.gov/Blast.cgi#284807159%23284807159) | 18.5 | 85% | 338 |
| [NP_079033.4](http://www.ncbi.nlm.nih.gov/entrez/query.fcgi?cmd=Retrieve&db=Protein&list_uids=224465233&dopt=GenPept&RID=T0AP12RA01N&log$=prottop&blast_rank=30) | euchromatic histone-lysine N-methyltransferase 1 isoform 1 [Homo sapiens] | [18.5](http://blast.ncbi.nlm.nih.gov/Blast.cgi#224465233%23224465233) | 18.5 | 85% | 338 |
| [NP_001165290.1](http://www.ncbi.nlm.nih.gov/entrez/query.fcgi?cmd=Retrieve&db=Protein&list_uids=284807157&dopt=GenPept&RID=T0AP12RA01N&log$=prottop&blast_rank=31) | peroxisome proliferative activated receptor, delta isoform 3 [Homo sapiens] | [18.5](http://blast.ncbi.nlm.nih.gov/Blast.cgi#284807157%23284807157) | 18.5 | 85% | 338 |
| [NP_115867.2](http://www.ncbi.nlm.nih.gov/entrez/query.fcgi?cmd=Retrieve&db=Protein&list_uids=169636418&dopt=GenPept&RID=T0AP12RA01N&log$=prottop&blast_rank=32) | mitochondrial ribosomal protein L38 precursor [Homo sapiens] | [18.5](http://blast.ncbi.nlm.nih.gov/Blast.cgi#169636418%23169636418) | 18.5 | 85% | 338 |
| [NP_004940.1](http://www.ncbi.nlm.nih.gov/entrez/query.fcgi?cmd=Retrieve&db=Protein&list_uids=13435366&dopt=GenPept&RID=T0AP12RA01N&log$=prottop&blast_rank=33) | desmocollin 2 isoform Dsc2b preproprotein [Homo sapiens] | [18.5](http://blast.ncbi.nlm.nih.gov/Blast.cgi#13435366%2313435366) | 18.5 | 71% | 338 |
| [NP_620601.1](http://www.ncbi.nlm.nih.gov/entrez/query.fcgi?cmd=Retrieve&db=Protein&list_uids=20986499&dopt=GenPept&RID=T0AP12RA01N&log$=prottop&blast_rank=34) | mitogen-activated protein kinase 7 isoform 2 [Homo sapiens] | [18.5](http://blast.ncbi.nlm.nih.gov/Blast.cgi#20986499%2320986499) | 18.5 | 71% | 338 |
| [NP_002740.2](http://www.ncbi.nlm.nih.gov/entrez/query.fcgi?cmd=Retrieve&db=Protein&list_uids=20986497&dopt=GenPept&RID=T0AP12RA01N&log$=prottop&blast_rank=35) | mitogen-activated protein kinase 7 isoform 1 [Homo sapiens] >ref|NP_620602.2| mitogen-activated protein kinase 7 isoform 1 [Homo sapiens] >ref|NP_620603.2| mitogen-activated protein kinase 7 isoform 1 [Homo sapiens] | [18.5](http://blast.ncbi.nlm.nih.gov/Blast.cgi#20986497%2320986497) | 34.4 | 100% | 338 |
| [NP_077740.1](http://www.ncbi.nlm.nih.gov/entrez/query.fcgi?cmd=Retrieve&db=Protein&list_uids=13435364&dopt=GenPept&RID=T0AP12RA01N&log$=prottop&blast_rank=36) | desmocollin 2 isoform Dsc2a preproprotein [Homo sapiens] | [18.5](http://blast.ncbi.nlm.nih.gov/Blast.cgi#13435364%2313435364) | 18.5 | 71% | 338 |
| [NP_001165949.1](http://www.ncbi.nlm.nih.gov/entrez/query.fcgi?cmd=Retrieve&db=Protein&list_uids=289177076&dopt=GenPept&RID=T0AP12RA01N&log$=prottop&blast_rank=37) | ribonucleotide reductase M2 B (TP53 inducible) isoform 3 [Homo sapiens] | [18.5](http://blast.ncbi.nlm.nih.gov/Blast.cgi#289177076%23289177076) | 18.5 | 100% | 338 |
| [NP_803184.1](http://www.ncbi.nlm.nih.gov/entrez/query.fcgi?cmd=Retrieve&db=Protein&list_uids=29171750&dopt=GenPept&RID=T0AP12RA01N&log$=prottop&blast_rank=38) | peroxisome proliferative activated receptor, delta isoform 2 [Homo sapiens] | [18.5](http://blast.ncbi.nlm.nih.gov/Blast.cgi#29171750%2329171750) | 18.5 | 85% | 338 |
| [NP_006229.1](http://www.ncbi.nlm.nih.gov/entrez/query.fcgi?cmd=Retrieve&db=Protein&list_uids=5453940&dopt=GenPept&RID=T0AP12RA01N&log$=prottop&blast_rank=39) | peroxisome proliferative activated receptor, delta isoform 1 [Homo sapiens] >ref|NP_001165289.1| peroxisome proliferative activated receptor, delta isoform 1 [Homo sapiens] | [18.5](http://blast.ncbi.nlm.nih.gov/Blast.cgi#5453940%235453940) | 18.5 | 85% | 338 |
| [NP_056953.2](http://www.ncbi.nlm.nih.gov/entrez/query.fcgi?cmd=Retrieve&db=Protein&list_uids=20336229&dopt=GenPept&RID=T0AP12RA01N&log$=prottop&blast_rank=40) | peroxisome proliferative activated receptor gamma isoform 2 [Homo sapiens] | [18.5](http://blast.ncbi.nlm.nih.gov/Blast.cgi#20336229%2320336229) | 18.5 | 85% | 338 |
| [NP_005028.4](http://www.ncbi.nlm.nih.gov/entrez/query.fcgi?cmd=Retrieve&db=Protein&list_uids=116284368&dopt=GenPept&RID=T0AP12RA01N&log$=prottop&blast_rank=41) | peroxisome proliferative activated receptor gamma isoform 1 [Homo sapiens] >ref|NP_619726.2| peroxisome proliferative activated receptor gamma isoform 1 [Homo sapiens] >ref|NP_619725.2| peroxisome proliferative activated receptor gamma isoform 1 [Homo sapiens] | [18.5](http://blast.ncbi.nlm.nih.gov/Blast.cgi#116284368%23116284368) | 18.5 | 85% | 338 |
| [NP_056528.2](http://www.ncbi.nlm.nih.gov/entrez/query.fcgi?cmd=Retrieve&db=Protein&list_uids=42544136&dopt=GenPept&RID=T0AP12RA01N&log$=prottop&blast_rank=42) | ribonucleotide reductase M2 B (TP53 inducible) isoform 1 [Homo sapiens] | [18.5](http://blast.ncbi.nlm.nih.gov/Blast.cgi#42544136%2342544136) | 18.5 | 100% | 338 |
| [NP_001154825.1](http://www.ncbi.nlm.nih.gov/entrez/query.fcgi?cmd=Retrieve&db=Protein&list_uids=238624132&dopt=GenPept&RID=T0AP12RA01N&log$=prottop&blast_rank=43) | large conductance calcium-activated potassium channel subfamily M alpha member 1 isoform d [Homo sapiens] | [18.0](http://blast.ncbi.nlm.nih.gov/Blast.cgi#238624132%23238624132) | 18.0 | 71% | 453 |
| [NP_001120694.1](http://www.ncbi.nlm.nih.gov/entrez/query.fcgi?cmd=Retrieve&db=Protein&list_uids=187828892&dopt=GenPept&RID=T0AP12RA01N&log$=prottop&blast_rank=44) | calcium channel, alpha 1A subunit isoform 4 [Homo sapiens] | [18.0](http://blast.ncbi.nlm.nih.gov/Blast.cgi#187828892%23187828892) | 36.1 | 71% | 453 |
| [NP_001120693.1](http://www.ncbi.nlm.nih.gov/entrez/query.fcgi?cmd=Retrieve&db=Protein&list_uids=187828880&dopt=GenPept&RID=T0AP12RA01N&log$=prottop&blast_rank=45) | calcium channel, alpha 1A subunit isoform 3 [Homo sapiens] | [18.0](http://blast.ncbi.nlm.nih.gov/Blast.cgi#187828880%23187828880) | 36.1 | 71% | 453 |
| [NP_001014797.1](http://www.ncbi.nlm.nih.gov/entrez/query.fcgi?cmd=Retrieve&db=Protein&list_uids=62388890&dopt=GenPept&RID=T0AP12RA01N&log$=prottop&blast_rank=46) | large conductance calcium-activated potassium channel subfamily M alpha member 1 isoform a [Homo sapiens] | [18.0](http://blast.ncbi.nlm.nih.gov/Blast.cgi#62388890%2362388890) | 18.0 | 71% | 453 |
| [NP_000712.2](http://www.ncbi.nlm.nih.gov/entrez/query.fcgi?cmd=Retrieve&db=Protein&list_uids=53832005&dopt=GenPept&RID=T0AP12RA01N&log$=prottop&blast_rank=47) | calcium channel, voltage-dependent, R type, alpha 1E subunit [Homo sapiens] | [18.0](http://blast.ncbi.nlm.nih.gov/Blast.cgi#53832005%2353832005) | 18.0 | 71% | 453 |
| [NP_065902.1](http://www.ncbi.nlm.nih.gov/entrez/query.fcgi?cmd=Retrieve&db=Protein&list_uids=39930401&dopt=GenPept&RID=T0AP12RA01N&log$=prottop&blast_rank=48) | immunoglobulin superfamily containing leucine-rich repeat 2 precursor [Homo sapiens] >ref|NP_001123608.1| immunoglobulin superfamily containing leucine-rich repeat 2 precursor [Homo sapiens] >ref|NP_001123609.1| immunoglobulin superfamily containing leucine-rich repeat 2 precursor [Homo sapiens] >ref|NP_001123610.1| immunoglobulin superfamily containing leucine-rich repeat 2 precursor [Homo sapiens] | [18.0](http://blast.ncbi.nlm.nih.gov/Blast.cgi#39930401%2339930401) | 18.0 | 85% | 453 |
| [NP_055926.1](http://www.ncbi.nlm.nih.gov/entrez/query.fcgi?cmd=Retrieve&db=Protein&list_uids=62177129&dopt=GenPept&RID=T0AP12RA01N&log$=prottop&blast_rank=49) | Nedd4 binding protein 3 [Homo sapiens] | [18.0](http://blast.ncbi.nlm.nih.gov/Blast.cgi#62177129%2362177129) | 18.0 | 71% | 453 |
| [NP_115597.3](http://www.ncbi.nlm.nih.gov/entrez/query.fcgi?cmd=Retrieve&db=Protein&list_uids=29244924&dopt=GenPept&RID=T0AP12RA01N&log$=prottop&blast_rank=50) | chromodomain helicase DNA binding protein 6 [Homo sapiens] | [18.0](http://blast.ncbi.nlm.nih.gov/Blast.cgi#29244924%2329244924) | 31.0 | 85% | 453 |
| [NP_037523.2](http://www.ncbi.nlm.nih.gov/entrez/query.fcgi?cmd=Retrieve&db=Protein&list_uids=24797151&dopt=GenPept&RID=T0AP12RA01N&log$=prottop&blast_rank=51) | dimethylglycine dehydrogenase precursor [Homo sapiens] | [18.0](http://blast.ncbi.nlm.nih.gov/Blast.cgi#24797151%2324797151) | 18.0 | 71% | 453 |
| [NP_001154824.1](http://www.ncbi.nlm.nih.gov/entrez/query.fcgi?cmd=Retrieve&db=Protein&list_uids=238624130&dopt=GenPept&RID=T0AP12RA01N&log$=prottop&blast_rank=52) | large conductance calcium-activated potassium channel subfamily M alpha member 1 isoform c [Homo sapiens] | [18.0](http://blast.ncbi.nlm.nih.gov/Blast.cgi#238624130%23238624130) | 18.0 | 71% | 453 |
| [NP_852660.1](http://www.ncbi.nlm.nih.gov/entrez/query.fcgi?cmd=Retrieve&db=Protein&list_uids=31881667&dopt=GenPept&RID=T0AP12RA01N&log$=prottop&blast_rank=53) | synaptotagmin XV isoform b [Homo sapiens] | [18.0](http://blast.ncbi.nlm.nih.gov/Blast.cgi#31881667%2331881667) | 18.0 | 85% | 453 |
| [NP_114118.2](http://www.ncbi.nlm.nih.gov/entrez/query.fcgi?cmd=Retrieve&db=Protein&list_uids=31881675&dopt=GenPept&RID=T0AP12RA01N&log$=prottop&blast_rank=54) | synaptotagmin XV isoform a [Homo sapiens] | [18.0](http://blast.ncbi.nlm.nih.gov/Blast.cgi#31881675%2331881675) | 18.0 | 85% | 453 |
| [NP_659422.2](http://www.ncbi.nlm.nih.gov/entrez/query.fcgi?cmd=Retrieve&db=Protein&list_uids=32880203&dopt=GenPept&RID=T0AP12RA01N&log$=prottop&blast_rank=55) | cadherin-like 24 isoform 2 [Homo sapiens] | [18.0](http://blast.ncbi.nlm.nih.gov/Blast.cgi#32880203%2332880203) | 18.0 | 85% | 453 |
| [NP_001788.2](http://www.ncbi.nlm.nih.gov/entrez/query.fcgi?cmd=Retrieve&db=Protein&list_uids=16306532&dopt=GenPept&RID=T0AP12RA01N&log$=prottop&blast_rank=56) | cadherin 11, type 2 preproprotein [Homo sapiens] | [18.0](http://blast.ncbi.nlm.nih.gov/Blast.cgi#16306532%2316306532) | 18.0 | 85% | 453 |
| [NP_071923.2](http://www.ncbi.nlm.nih.gov/entrez/query.fcgi?cmd=Retrieve&db=Protein&list_uids=32880206&dopt=GenPept&RID=T0AP12RA01N&log$=prottop&blast_rank=57) | cadherin-like 24 isoform 1 [Homo sapiens] | [18.0](http://blast.ncbi.nlm.nih.gov/Blast.cgi#32880206%2332880206) | 18.0 | 85% | 453 |
| [NP_115886.1](http://www.ncbi.nlm.nih.gov/entrez/query.fcgi?cmd=Retrieve&db=Protein&list_uids=23618926&dopt=GenPept&RID=T0AP12RA01N&log$=prottop&blast_rank=58) | zinc finger protein 559 [Homo sapiens] | [18.0](http://blast.ncbi.nlm.nih.gov/Blast.cgi#23618926%2323618926) | 18.0 | 85% | 453 |
| [NP_775921.1](http://www.ncbi.nlm.nih.gov/entrez/query.fcgi?cmd=Retrieve&db=Protein&list_uids=27735107&dopt=GenPept&RID=T0AP12RA01N&log$=prottop&blast_rank=59) | DnaJ (Hsp40) homolog, subfamily C, member 5 gamma [Homo sapiens] | [18.0](http://blast.ncbi.nlm.nih.gov/Blast.cgi#27735107%2327735107) | 18.0 | 71% | 453 |
| [NP_002238.2](http://www.ncbi.nlm.nih.gov/entrez/query.fcgi?cmd=Retrieve&db=Protein&list_uids=26638650&dopt=GenPept&RID=T0AP12RA01N&log$=prottop&blast_rank=60) | large conductance calcium-activated potassium channel subfamily M alpha member 1 isoform b [Homo sapiens] | [18.0](http://blast.ncbi.nlm.nih.gov/Blast.cgi#26638650%2326638650) | 18.0 | 71% | 453 |
| [NP_777570.1](http://www.ncbi.nlm.nih.gov/entrez/query.fcgi?cmd=Retrieve&db=Protein&list_uids=28372535&dopt=GenPept&RID=T0AP12RA01N&log$=prottop&blast_rank=61) | t-complex-associated-testis-expressed 3 [Homo sapiens] | [18.0](http://blast.ncbi.nlm.nih.gov/Blast.cgi#28372535%2328372535) | 18.0 | 71% | 453 |
| [NP_006359.3](http://www.ncbi.nlm.nih.gov/entrez/query.fcgi?cmd=Retrieve&db=Protein&list_uids=22219462&dopt=GenPept&RID=T0AP12RA01N&log$=prottop&blast_rank=62) | cAMP responsive element binding protein 3 [Homo sapiens] | [18.0](http://blast.ncbi.nlm.nih.gov/Blast.cgi#22219462%2322219462) | 18.0 | 85% | 453 |
| [NP_000709.1](http://www.ncbi.nlm.nih.gov/entrez/query.fcgi?cmd=Retrieve&db=Protein&list_uids=4502523&dopt=GenPept&RID=T0AP12RA01N&log$=prottop&blast_rank=63) | calcium channel, voltage-dependent, N type, alpha 1B subunit [Homo sapiens] | [18.0](http://blast.ncbi.nlm.nih.gov/Blast.cgi#4502523%234502523) | 36.1 | 71% | 453 |
| [NP_000275.1](http://www.ncbi.nlm.nih.gov/entrez/query.fcgi?cmd=Retrieve&db=Protein&list_uids=4505685&dopt=GenPept&RID=T0AP12RA01N&log$=prottop&blast_rank=64) | pyruvate dehydrogenase E1 alpha 1 precursor [Homo sapiens] | [18.0](http://blast.ncbi.nlm.nih.gov/Blast.cgi#4505685%234505685) | 18.0 | 71% | 453 |
| [NP_002692.2](http://www.ncbi.nlm.nih.gov/entrez/query.fcgi?cmd=Retrieve&db=Protein&list_uids=42560248&dopt=GenPept&RID=T0AP12RA01N&log$=prottop&blast_rank=65) | POU domain, class 5, transcription factor 1 isoform 1 [Homo sapiens] | [18.0](http://blast.ncbi.nlm.nih.gov/Blast.cgi#42560248%2342560248) | 18.0 | 71% | 453 |
| [NP_001787.2](http://www.ncbi.nlm.nih.gov/entrez/query.fcgi?cmd=Retrieve&db=Protein&list_uids=16306539&dopt=GenPept&RID=T0AP12RA01N&log$=prottop&blast_rank=66) | cadherin 8, type 2 preproprotein [Homo sapiens] | [18.0](http://blast.ncbi.nlm.nih.gov/Blast.cgi#16306539%2316306539) | 18.0 | 85% | 453 |
| [XP_002346530.1](http://www.ncbi.nlm.nih.gov/entrez/query.fcgi?cmd=Retrieve&db=Protein&list_uids=239748101&dopt=GenPept&RID=T0AP12RA01N&log$=prottop&blast_rank=67) | PREDICTED: hypothetical protein [Homo sapiens] >ref|XP_002345697.1| PREDICTED: hypothetical protein XP_002345697 [Homo sapiens] | [17.6](http://blast.ncbi.nlm.nih.gov/Blast.cgi#239748101%23239748101) | 17.6 | 57% | 608 |
| [XP_002343599.1](http://www.ncbi.nlm.nih.gov/entrez/query.fcgi?cmd=Retrieve&db=Protein&list_uids=239745821&dopt=GenPept&RID=T0AP12RA01N&log$=prottop&blast_rank=68) | PREDICTED: zinc finger protein 385C [Homo sapiens] >ref|XP_002347806.1| PREDICTED: zinc finger protein 385C [Homo sapiens] >ref|XP_002345070.1| PREDICTED: zinc finger protein 385C [Homo sapiens] | [17.6](http://blast.ncbi.nlm.nih.gov/Blast.cgi#239745821%23239745821) | 31.4 | 85% | 608 |
| [XP_002342952.1](http://www.ncbi.nlm.nih.gov/entrez/query.fcgi?cmd=Retrieve&db=Protein&list_uids=239743756&dopt=GenPept&RID=T0AP12RA01N&log$=prottop&blast_rank=69) | PREDICTED: hypothetical protein LOC389722 isoform 2 [Homo sapiens] | [17.6](http://blast.ncbi.nlm.nih.gov/Blast.cgi#239743756%23239743756) | 17.6 | 71% | 608 |
| [XP_002344294.1](http://www.ncbi.nlm.nih.gov/entrez/query.fcgi?cmd=Retrieve&db=Protein&list_uids=239743729&dopt=GenPept&RID=T0AP12RA01N&log$=prottop&blast_rank=70) | PREDICTED: similar to contactin associated protein-like 3B [Homo sapiens] | [17.6](http://blast.ncbi.nlm.nih.gov/Blast.cgi#239743729%23239743729) | 17.6 | 71% | 608 |
| [XP_002342369.1](http://www.ncbi.nlm.nih.gov/entrez/query.fcgi?cmd=Retrieve&db=Protein&list_uids=239741997&dopt=GenPept&RID=T0AP12RA01N&log$=prottop&blast_rank=71) | PREDICTED: hypothetical protein XP_002342369 [Homo sapiens] | [17.6](http://blast.ncbi.nlm.nih.gov/Blast.cgi#239741997%23239741997) | 17.6 | 57% | 608 |
| [NP_001139287.1](http://www.ncbi.nlm.nih.gov/entrez/query.fcgi?cmd=Retrieve&db=Protein&list_uids=224922791&dopt=GenPept&RID=T0AP12RA01N&log$=prottop&blast_rank=72) | amidohydrolase domain containing 2 isoform 2 [Homo sapiens] | [17.6](http://blast.ncbi.nlm.nih.gov/Blast.cgi#224922791%23224922791) | 17.6 | 57% | 608 |
| [NP_085095.1](http://www.ncbi.nlm.nih.gov/entrez/query.fcgi?cmd=Retrieve&db=Protein&list_uids=13699836&dopt=GenPept&RID=T0AP12RA01N&log$=prottop&blast_rank=73) | matrilin 4 isoform 3 precursor [Homo sapiens] | [17.6](http://blast.ncbi.nlm.nih.gov/Blast.cgi#13699836%2313699836) | 17.6 | 57% | 608 |
| [NP_001007541.2](http://www.ncbi.nlm.nih.gov/entrez/query.fcgi?cmd=Retrieve&db=Protein&list_uids=203097724&dopt=GenPept&RID=T0AP12RA01N&log$=prottop&blast_rank=74) | cadherin-related family member 4 precursor [Homo sapiens] | [17.6](http://blast.ncbi.nlm.nih.gov/Blast.cgi#203097724%23203097724) | 17.6 | 71% | 608 |
| [NP_001092093.1](http://www.ncbi.nlm.nih.gov/entrez/query.fcgi?cmd=Retrieve&db=Protein&list_uids=148833506&dopt=GenPept&RID=T0AP12RA01N&log$=prottop&blast_rank=75) | obscurin, cytoskeletal calmodulin and titin-interacting RhoGEF isoform b [Homo sapiens] | [17.6](http://blast.ncbi.nlm.nih.gov/Blast.cgi#148833506%23148833506) | 33.5 | 100% | 608 |
| [NP_056483.3](http://www.ncbi.nlm.nih.gov/entrez/query.fcgi?cmd=Retrieve&db=Protein&list_uids=158534077&dopt=GenPept&RID=T0AP12RA01N&log$=prottop&blast_rank=76) | regulator of G-protein signaling 22 [Homo sapiens] | [17.6](http://blast.ncbi.nlm.nih.gov/Blast.cgi#158534077%23158534077) | 34.4 | 85% | 608 |
| [NP_061198.2](http://www.ncbi.nlm.nih.gov/entrez/query.fcgi?cmd=Retrieve&db=Protein&list_uids=153945715&dopt=GenPept&RID=T0AP12RA01N&log$=prottop&blast_rank=77) | myosin VC [Homo sapiens] | [17.6](http://blast.ncbi.nlm.nih.gov/Blast.cgi#153945715%23153945715) | 17.6 | 57% | 608 |
| [NP_536350.2](http://www.ncbi.nlm.nih.gov/entrez/query.fcgi?cmd=Retrieve&db=Protein&list_uids=117938759&dopt=GenPept&RID=T0AP12RA01N&log$=prottop&blast_rank=78) | GNAS complex locus XLas [Homo sapiens] | [17.6](http://blast.ncbi.nlm.nih.gov/Blast.cgi#117938759%23117938759) | 34.8 | 71% | 608 |
| [NP_596869.3](http://www.ncbi.nlm.nih.gov/entrez/query.fcgi?cmd=Retrieve&db=Protein&list_uids=110349719&dopt=GenPept&RID=T0AP12RA01N&log$=prottop&blast_rank=79) | titin isoform N2-A [Homo sapiens] | [17.6](http://blast.ncbi.nlm.nih.gov/Blast.cgi#110349719%23110349719) | 182 | 100% | 608 |
| [NP_114095.2](http://www.ncbi.nlm.nih.gov/entrez/query.fcgi?cmd=Retrieve&db=Protein&list_uids=189491628&dopt=GenPept&RID=T0AP12RA01N&log$=prottop&blast_rank=80) | enamelin precursor [Homo sapiens] | [17.6](http://blast.ncbi.nlm.nih.gov/Blast.cgi#189491628%23189491628) | 17.6 | 57% | 608 |
| [XP_002342951.1](http://www.ncbi.nlm.nih.gov/entrez/query.fcgi?cmd=Retrieve&db=Protein&list_uids=239743754&dopt=GenPept&RID=T0AP12RA01N&log$=prottop&blast_rank=81) | PREDICTED: hypothetical protein LOC389722 isoform 1 [Homo sapiens] | [17.6](http://blast.ncbi.nlm.nih.gov/Blast.cgi#239743754%23239743754) | 17.6 | 71% | 608 |
| [NP_387504.2](http://www.ncbi.nlm.nih.gov/entrez/query.fcgi?cmd=Retrieve&db=Protein&list_uids=47519929&dopt=GenPept&RID=T0AP12RA01N&log$=prottop&blast_rank=82) | cell recognition molecule CASPR3 precursor [Homo sapiens] | [17.6](http://blast.ncbi.nlm.nih.gov/Blast.cgi#47519929%2347519929) | 17.6 | 71% | 608 |
| [NP_002284.3](http://www.ncbi.nlm.nih.gov/entrez/query.fcgi?cmd=Retrieve&db=Protein&list_uids=145309326&dopt=GenPept&RID=T0AP12RA01N&log$=prottop&blast_rank=83) | laminin, gamma 1 precursor [Homo sapiens] | [17.6](http://blast.ncbi.nlm.nih.gov/Blast.cgi#145309326%23145309326) | 17.6 | 57% | 608 |
| [NP_115689.1](http://www.ncbi.nlm.nih.gov/entrez/query.fcgi?cmd=Retrieve&db=Protein&list_uids=14150078&dopt=GenPept&RID=T0AP12RA01N&log$=prottop&blast_rank=84) | hypothetical protein LOC84273 [Homo sapiens] | [17.6](http://blast.ncbi.nlm.nih.gov/Blast.cgi#14150078%2314150078) | 17.6 | 57% | 608 |
| [NP_055613.1](http://www.ncbi.nlm.nih.gov/entrez/query.fcgi?cmd=Retrieve&db=Protein&list_uids=40538728&dopt=GenPept&RID=T0AP12RA01N&log$=prottop&blast_rank=85) | pleckstrin homology domain containing, family M (with RUN domain) member 1 [Homo sapiens] | [17.6](http://blast.ncbi.nlm.nih.gov/Blast.cgi#40538728%2340538728) | 17.6 | 57% | 608 |
| [NP_003824.2](http://www.ncbi.nlm.nih.gov/entrez/query.fcgi?cmd=Retrieve&db=Protein&list_uids=13699830&dopt=GenPept&RID=T0AP12RA01N&log$=prottop&blast_rank=86) | matrilin 4 isoform 1 precursor [Homo sapiens] | [17.6](http://blast.ncbi.nlm.nih.gov/Blast.cgi#13699830%2313699830) | 17.6 | 57% | 608 |
| [NP_036477.2](http://www.ncbi.nlm.nih.gov/entrez/query.fcgi?cmd=Retrieve&db=Protein&list_uids=166235163&dopt=GenPept&RID=T0AP12RA01N&log$=prottop&blast_rank=87) | nuclear fragile X mental retardation protein interacting protein 1 [Homo sapiens] | [17.6](http://blast.ncbi.nlm.nih.gov/Blast.cgi#166235163%23166235163) | 46.0 | 85% | 608 |
| [NP_085080.1](http://www.ncbi.nlm.nih.gov/entrez/query.fcgi?cmd=Retrieve&db=Protein&list_uids=13699834&dopt=GenPept&RID=T0AP12RA01N&log$=prottop&blast_rank=88) | matrilin 4 isoform 2 precursor [Homo sapiens] | [17.6](http://blast.ncbi.nlm.nih.gov/Blast.cgi#13699834%2313699834) | 17.6 | 57% | 608 |
| [NP_001012331.1](http://www.ncbi.nlm.nih.gov/entrez/query.fcgi?cmd=Retrieve&db=Protein&list_uids=59889558&dopt=GenPept&RID=T0AP12RA01N&log$=prottop&blast_rank=89) | neurotrophic tyrosine kinase, receptor, type 1 isoform 1 [Homo sapiens] | [17.6](http://blast.ncbi.nlm.nih.gov/Blast.cgi#59889558%2359889558) | 17.6 | 57% | 608 |
| [NP_065744.2](http://www.ncbi.nlm.nih.gov/entrez/query.fcgi?cmd=Retrieve&db=Protein&list_uids=21359935&dopt=GenPept&RID=T0AP12RA01N&log$=prottop&blast_rank=90) | Down syndrome cell adhesion molecule like 1 [Homo sapiens] | [17.6](http://blast.ncbi.nlm.nih.gov/Blast.cgi#21359935%2321359935) | 33.5 | 100% | 608 |
| [NP_001380.2](http://www.ncbi.nlm.nih.gov/entrez/query.fcgi?cmd=Retrieve&db=Protein&list_uids=20127422&dopt=GenPept&RID=T0AP12RA01N&log$=prottop&blast_rank=91) | Down syndrome cell adhesion molecule isoform CHD2-42 precursor [Homo sapiens] | [17.6](http://blast.ncbi.nlm.nih.gov/Blast.cgi#20127422%2320127422) | 17.6 | 57% | 608 |
| [NP_060082.2](http://www.ncbi.nlm.nih.gov/entrez/query.fcgi?cmd=Retrieve&db=Protein&list_uids=38044290&dopt=GenPept&RID=T0AP12RA01N&log$=prottop&blast_rank=92) | zinc finger, CCHC domain containing 8 [Homo sapiens] | [17.6](http://blast.ncbi.nlm.nih.gov/Blast.cgi#38044290%2338044290) | 17.6 | 57% | 608 |
| [NP_006138.1](http://www.ncbi.nlm.nih.gov/entrez/query.fcgi?cmd=Retrieve&db=Protein&list_uids=5453700&dopt=GenPept&RID=T0AP12RA01N&log$=prottop&blast_rank=93) | interferon regulatory factor 6 [Homo sapiens] | [17.6](http://blast.ncbi.nlm.nih.gov/Blast.cgi#5453700%235453700) | 17.6 | 71% | 608 |
| [NP_005774.2](http://www.ncbi.nlm.nih.gov/entrez/query.fcgi?cmd=Retrieve&db=Protein&list_uids=18104959&dopt=GenPept&RID=T0AP12RA01N&log$=prottop&blast_rank=94) | thioredoxin domain containing 9 [Homo sapiens] | [17.6](http://blast.ncbi.nlm.nih.gov/Blast.cgi#18104959%2318104959) | 17.6 | 71% | 608 |
| [NP_060163.2](http://www.ncbi.nlm.nih.gov/entrez/query.fcgi?cmd=Retrieve&db=Protein&list_uids=22095355&dopt=GenPept&RID=T0AP12RA01N&log$=prottop&blast_rank=95) | basic, immunoglobulin-like variable motif containing isoform a [Homo sapiens] | [17.6](http://blast.ncbi.nlm.nih.gov/Blast.cgi#22095355%2322095355) | 17.6 | 71% | 608 |
| [NP_000916.2](http://www.ncbi.nlm.nih.gov/entrez/query.fcgi?cmd=Retrieve&db=Protein&list_uids=156564403&dopt=GenPept&RID=T0AP12RA01N&log$=prottop&blast_rank=96) | pyruvate dehydrogenase (lipoamide) beta precursor [Homo sapiens] | [17.6](http://blast.ncbi.nlm.nih.gov/Blast.cgi#156564403%23156564403) | 17.6 | 57% | 608 |
| [NP_006043.1](http://www.ncbi.nlm.nih.gov/entrez/query.fcgi?cmd=Retrieve&db=Protein&list_uids=5174425&dopt=GenPept&RID=T0AP12RA01N&log$=prottop&blast_rank=97) | Down syndrome critical region protein 3 [Homo sapiens] | [17.6](http://blast.ncbi.nlm.nih.gov/Blast.cgi#5174425%235174425) | 17.6 | 57% | 608 |
| [NP_620407.1](http://www.ncbi.nlm.nih.gov/entrez/query.fcgi?cmd=Retrieve&db=Protein&list_uids=20986531&dopt=GenPept&RID=T0AP12RA01N&log$=prottop&blast_rank=98) | mitogen-activated protein kinase 1 [Homo sapiens] >ref|NP_002736.3| mitogen-activated protein kinase 1 [Homo sapiens] | [17.6](http://blast.ncbi.nlm.nih.gov/Blast.cgi#20986531%2320986531) | 17.6 | 100% | 608 |
| [NP_005391.1](http://www.ncbi.nlm.nih.gov/entrez/query.fcgi?cmd=Retrieve&db=Protein&list_uids=4885563&dopt=GenPept&RID=T0AP12RA01N&log$=prottop&blast_rank=99) | protein kinase C, epsilon [Homo sapiens] | [17.6](http://blast.ncbi.nlm.nih.gov/Blast.cgi#4885563%234885563) | 17.6 | 71% | 608 |
| [NP_057525.1](http://www.ncbi.nlm.nih.gov/entrez/query.fcgi?cmd=Retrieve&db=Protein&list_uids=10092639&dopt=GenPept&RID=T0AP12RA01N&log$=prottop&blast_rank=100) | cysteine-rich motor neuron 1 precursor [Homo sapiens] | [17.6](http://blast.ncbi.nlm.nih.gov/Blast.cgi#10092639%2310092639) | 17.6 | 57% | 608 |

| **Accession** | **Proteins with a match to PSKAAYVV peptide** | **[Max score](http://blast.ncbi.nlm.nih.gov/Blast.cgi?CMD=Get&ALIGNMENTS=100&ALIGNMENT_VIEW=Pairwise&CDD_SEARCH_STATE=1&DATABASE_SORT=0&DESCRIPTIONS=100&ENTREZ_QUERY=txid9606 %5BORGN%5D&FIRST_QUERY_NUM=0&FORMAT_OBJECT=Alignment&FORMAT_PAGE_TARGET=&FORMAT_TYPE=HTML&GET_SEQUENCE=yes&I_THRESH=&MASK_CHAR=2&MASK_COLOR=1&NEW_DESIGN=on&NEW_VIEW=yes&NUM_OVERVIEW=100&OLD_BLAST=false&PAGE=Proteins&QUERY_INDEX=0&QUERY_NUMBER=0&RESULTS_PAGE_TARGET=&RID=T0BFYNHX01S&SHOW_LINKOUT=yes&SHOW_OVERVIEW=yes&STEP_NUMBER=&WORD_SIZE=2&DISPLAY_SORT=1&HSP_SORT=1" \l "sort_mark)** | **[Total score](http://blast.ncbi.nlm.nih.gov/Blast.cgi?CMD=Get&ALIGNMENTS=100&ALIGNMENT_VIEW=Pairwise&CDD_SEARCH_STATE=1&DATABASE_SORT=0&DESCRIPTIONS=100&ENTREZ_QUERY=txid9606 %5BORGN%5D&FIRST_QUERY_NUM=0&FORMAT_OBJECT=Alignment&FORMAT_PAGE_TARGET=&FORMAT_TYPE=HTML&GET_SEQUENCE=yes&I_THRESH=&MASK_CHAR=2&MASK_COLOR=1&NEW_DESIGN=on&NEW_VIEW=yes&NUM_OVERVIEW=100&OLD_BLAST=false&PAGE=Proteins&QUERY_INDEX=0&QUERY_NUMBER=0&RESULTS_PAGE_TARGET=&RID=T0BFYNHX01S&SHOW_LINKOUT=yes&SHOW_OVERVIEW=yes&STEP_NUMBER=&WORD_SIZE=2&DISPLAY_SORT=2&HSP_SORT=1" \l "sort_mark)** | **[Query coverage](http://blast.ncbi.nlm.nih.gov/Blast.cgi?CMD=Get&ALIGNMENTS=100&ALIGNMENT_VIEW=Pairwise&CDD_SEARCH_STATE=1&DATABASE_SORT=0&DESCRIPTIONS=100&ENTREZ_QUERY=txid9606 %5BORGN%5D&FIRST_QUERY_NUM=0&FORMAT_OBJECT=Alignment&FORMAT_PAGE_TARGET=&FORMAT_TYPE=HTML&GET_SEQUENCE=yes&I_THRESH=&MASK_CHAR=2&MASK_COLOR=1&NEW_DESIGN=on&NEW_VIEW=yes&NUM_OVERVIEW=100&OLD_BLAST=false&PAGE=Proteins&QUERY_INDEX=0&QUERY_NUMBER=0&RESULTS_PAGE_TARGET=&RID=T0BFYNHX01S&SHOW_LINKOUT=yes&SHOW_OVERVIEW=yes&STEP_NUMBER=&WORD_SIZE=2&DISPLAY_SORT=4&HSP_SORT=0" \l "sort_mark)** | **[E value](http://blast.ncbi.nlm.nih.gov/Blast.cgi?CMD=Get&ALIGNMENTS=100&ALIGNMENT_VIEW=Pairwise&CDD_SEARCH_STATE=1&DATABASE_SORT=0&DESCRIPTIONS=100&ENTREZ_QUERY=txid9606 %5BORGN%5D&FIRST_QUERY_NUM=0&FORMAT_OBJECT=Alignment&FORMAT_PAGE_TARGET=&FORMAT_TYPE=HTML&GET_SEQUENCE=yes&I_THRESH=&MASK_CHAR=2&MASK_COLOR=1&NEW_DESIGN=on&NEW_VIEW=yes&NUM_OVERVIEW=100&OLD_BLAST=false&PAGE=Proteins&QUERY_INDEX=0&QUERY_NUMBER=0&RESULTS_PAGE_TARGET=&RID=T0BFYNHX01S&SHOW_LINKOUT=yes&SHOW_OVERVIEW=yes&STEP_NUMBER=&WORD_SIZE=2&DISPLAY_SORT=0&HSP_SORT=0" \l "sort_mark)** |
| --- | --- | --- | --- | --- | --- |
| [NP_001094891.3](http://www.ncbi.nlm.nih.gov/entrez/query.fcgi?cmd=Retrieve&db=Protein&list_uids=254028267&dopt=GenPept&RID=T0BFYNHX01S&log$=prottop&blast_rank=1) | myosin 1H [Homo sapiens] | [21.4](http://blast.ncbi.nlm.nih.gov/Blast.cgi#254028267%23254028267) | 21.4 | 75% | 49 |
| [NP_056655.2](http://www.ncbi.nlm.nih.gov/entrez/query.fcgi?cmd=Retrieve&db=Protein&list_uids=70166944&dopt=GenPept&RID=T0BFYNHX01S&log$=prottop&blast_rank=2) | adenosine deaminase, RNA-specific isoform b [Homo sapiens] | [21.0](http://blast.ncbi.nlm.nih.gov/Blast.cgi#70166944%2370166944) | 21.0 | 87% | 66 |
| [NP_056656.2](http://www.ncbi.nlm.nih.gov/entrez/query.fcgi?cmd=Retrieve&db=Protein&list_uids=70167032&dopt=GenPept&RID=T0BFYNHX01S&log$=prottop&blast_rank=3) | adenosine deaminase, RNA-specific isoform c [Homo sapiens] | [21.0](http://blast.ncbi.nlm.nih.gov/Blast.cgi#70167032%2370167032) | 21.0 | 87% | 66 |
| [NP_001020278.1](http://www.ncbi.nlm.nih.gov/entrez/query.fcgi?cmd=Retrieve&db=Protein&list_uids=70167113&dopt=GenPept&RID=T0BFYNHX01S&log$=prottop&blast_rank=4) | adenosine deaminase, RNA-specific isoform d [Homo sapiens] | [21.0](http://blast.ncbi.nlm.nih.gov/Blast.cgi#70167113%2370167113) | 21.0 | 87% | 66 |
| [NP_079146.2](http://www.ncbi.nlm.nih.gov/entrez/query.fcgi?cmd=Retrieve&db=Protein&list_uids=47578115&dopt=GenPept&RID=T0BFYNHX01S&log$=prottop&blast_rank=5) | DEP domain containing 2 isoform a [Homo sapiens] | [21.0](http://blast.ncbi.nlm.nih.gov/Blast.cgi#47578115%2347578115) | 21.0 | 75% | 66 |
| [NP_004642.2](http://www.ncbi.nlm.nih.gov/entrez/query.fcgi?cmd=Retrieve&db=Protein&list_uids=24234683&dopt=GenPept&RID=T0BFYNHX01S&log$=prottop&blast_rank=6) | ubiquitin specific peptidase 11 [Homo sapiens] | [21.0](http://blast.ncbi.nlm.nih.gov/Blast.cgi#24234683%2324234683) | 21.0 | 75% | 66 |
| [NP_001102.2](http://www.ncbi.nlm.nih.gov/entrez/query.fcgi?cmd=Retrieve&db=Protein&list_uids=70166852&dopt=GenPept&RID=T0BFYNHX01S&log$=prottop&blast_rank=7) | adenosine deaminase, RNA-specific isoform a [Homo sapiens] | [21.0](http://blast.ncbi.nlm.nih.gov/Blast.cgi#70166852%2370166852) | 21.0 | 87% | 66 |
| [NP_006304.1](http://www.ncbi.nlm.nih.gov/entrez/query.fcgi?cmd=Retrieve&db=Protein&list_uids=14149627&dopt=GenPept&RID=T0BFYNHX01S&log$=prottop&blast_rank=8) | ubiquitin specific peptidase 15 [Homo sapiens] | [21.0](http://blast.ncbi.nlm.nih.gov/Blast.cgi#14149627%2314149627) | 32.7 | 87% | 66 |
| [NP_055059.2](http://www.ncbi.nlm.nih.gov/entrez/query.fcgi?cmd=Retrieve&db=Protein&list_uids=110825974&dopt=GenPept&RID=T0BFYNHX01S&log$=prottop&blast_rank=9) | ADAM metallopeptidase with thrombospondin type 1 motif, 2 isoform 1 preproprotein [Homo sapiens] | [19.3](http://blast.ncbi.nlm.nih.gov/Blast.cgi#110825974%23110825974) | 19.3 | 100% | 214 |
| [NP_001136022.1](http://www.ncbi.nlm.nih.gov/entrez/query.fcgi?cmd=Retrieve&db=Protein&list_uids=216548378&dopt=GenPept&RID=T0BFYNHX01S&log$=prottop&blast_rank=10) | WD repeat domain 47 isoform 1 [Homo sapiens] | [18.9](http://blast.ncbi.nlm.nih.gov/Blast.cgi#216548378%23216548378) | 18.9 | 75% | 288 |
| [NP_078966.2](http://www.ncbi.nlm.nih.gov/entrez/query.fcgi?cmd=Retrieve&db=Protein&list_uids=83367077&dopt=GenPept&RID=T0BFYNHX01S&log$=prottop&blast_rank=11) | mucin 16 [Homo sapiens] | [18.9](http://blast.ncbi.nlm.nih.gov/Blast.cgi#83367077%2383367077) | 62.8 | 100% | 288 |
| [NP_055784.3](http://www.ncbi.nlm.nih.gov/entrez/query.fcgi?cmd=Retrieve&db=Protein&list_uids=216548347&dopt=GenPept&RID=T0BFYNHX01S&log$=prottop&blast_rank=12) | WD repeat domain 47 isoform 2 [Homo sapiens] | [18.9](http://blast.ncbi.nlm.nih.gov/Blast.cgi#216548347%23216548347) | 18.9 | 75% | 288 |
| [NP_001136023.1](http://www.ncbi.nlm.nih.gov/entrez/query.fcgi?cmd=Retrieve&db=Protein&list_uids=216548382&dopt=GenPept&RID=T0BFYNHX01S&log$=prottop&blast_rank=13) | WD repeat domain 47 isoform 3 [Homo sapiens] | [18.9](http://blast.ncbi.nlm.nih.gov/Blast.cgi#216548382%23216548382) | 18.9 | 75% | 288 |
| [NP_001157980.1](http://www.ncbi.nlm.nih.gov/entrez/query.fcgi?cmd=Retrieve&db=Protein&list_uids=257743025&dopt=GenPept&RID=T0BFYNHX01S&log$=prottop&blast_rank=14) | nebulin isoform 2 [Homo sapiens] | [18.5](http://blast.ncbi.nlm.nih.gov/Blast.cgi#257743025%23257743025) | 18.5 | 62% | 386 |
| [NP_001157979.1](http://www.ncbi.nlm.nih.gov/entrez/query.fcgi?cmd=Retrieve&db=Protein&list_uids=257743023&dopt=GenPept&RID=T0BFYNHX01S&log$=prottop&blast_rank=15) | nebulin isoform 1 [Homo sapiens] | [18.5](http://blast.ncbi.nlm.nih.gov/Blast.cgi#257743023%23257743023) | 18.5 | 62% | 386 |
| [NP_974487.1](http://www.ncbi.nlm.nih.gov/entrez/query.fcgi?cmd=Retrieve&db=Protein&list_uids=42544117&dopt=GenPept&RID=T0BFYNHX01S&log$=prottop&blast_rank=16) | rTS beta protein isoform rTS gamma [Homo sapiens] | [18.5](http://blast.ncbi.nlm.nih.gov/Blast.cgi#42544117%2342544117) | 18.5 | 62% | 386 |
| [NP_689540.2](http://www.ncbi.nlm.nih.gov/entrez/query.fcgi?cmd=Retrieve&db=Protein&list_uids=148727333&dopt=GenPept&RID=T0BFYNHX01S&log$=prottop&blast_rank=17) | adenylate kinase 7 [Homo sapiens] | [18.5](http://blast.ncbi.nlm.nih.gov/Blast.cgi#148727333%23148727333) | 18.5 | 62% | 386 |
| [NP_004534.2](http://www.ncbi.nlm.nih.gov/entrez/query.fcgi?cmd=Retrieve&db=Protein&list_uids=115527120&dopt=GenPept&RID=T0BFYNHX01S&log$=prottop&blast_rank=18) | nebulin isoform 3 [Homo sapiens] | [18.5](http://blast.ncbi.nlm.nih.gov/Blast.cgi#115527120%23115527120) | 18.5 | 62% | 386 |
| [NP_059982.2](http://www.ncbi.nlm.nih.gov/entrez/query.fcgi?cmd=Retrieve&db=Protein&list_uids=42544119&dopt=GenPept&RID=T0BFYNHX01S&log$=prottop&blast_rank=19) | enolase superfamily 1 isoform rTS beta [Homo sapiens] | [18.5](http://blast.ncbi.nlm.nih.gov/Blast.cgi#42544119%2342544119) | 18.5 | 62% | 386 |
| [NP_955475.1](http://www.ncbi.nlm.nih.gov/entrez/query.fcgi?cmd=Retrieve&db=Protein&list_uids=40795667&dopt=GenPept&RID=T0BFYNHX01S&log$=prottop&blast_rank=20) | ubiquitin specific protease 4 isoform b [Homo sapiens] | [18.5](http://blast.ncbi.nlm.nih.gov/Blast.cgi#40795667%2340795667) | 18.5 | 62% | 386 |
| [NP_003354.2](http://www.ncbi.nlm.nih.gov/entrez/query.fcgi?cmd=Retrieve&db=Protein&list_uids=40795665&dopt=GenPept&RID=T0BFYNHX01S&log$=prottop&blast_rank=21) | ubiquitin specific protease 4 isoform a [Homo sapiens] | [18.5](http://blast.ncbi.nlm.nih.gov/Blast.cgi#40795665%2340795665) | 18.5 | 62% | 386 |
| [NP_000483.3](http://www.ncbi.nlm.nih.gov/entrez/query.fcgi?cmd=Retrieve&db=Protein&list_uids=90421313&dopt=GenPept&RID=T0BFYNHX01S&log$=prottop&blast_rank=22) | cystic fibrosis transmembrane conductance regulator [Homo sapiens] | [18.5](http://blast.ncbi.nlm.nih.gov/Blast.cgi#90421313%2390421313) | 18.5 | 62% | 386 |
| [NP_001161814.1](http://www.ncbi.nlm.nih.gov/entrez/query.fcgi?cmd=Retrieve&db=Protein&list_uids=270265893&dopt=GenPept&RID=T0BFYNHX01S&log$=prottop&blast_rank=23) | tetratricopeptide repeat domain 39B isoform 5 [Homo sapiens] | [18.0](http://blast.ncbi.nlm.nih.gov/Blast.cgi#270265893%23270265893) | 18.0 | 75% | 518 |
| [NP_001161813.1](http://www.ncbi.nlm.nih.gov/entrez/query.fcgi?cmd=Retrieve&db=Protein&list_uids=270265891&dopt=GenPept&RID=T0BFYNHX01S&log$=prottop&blast_rank=24) | tetratricopeptide repeat domain 39B isoform 4 precursor [Homo sapiens] | [18.0](http://blast.ncbi.nlm.nih.gov/Blast.cgi#270265891%23270265891) | 18.0 | 75% | 518 |
| [NP_001161812.1](http://www.ncbi.nlm.nih.gov/entrez/query.fcgi?cmd=Retrieve&db=Protein&list_uids=270265889&dopt=GenPept&RID=T0BFYNHX01S&log$=prottop&blast_rank=25) | tetratricopeptide repeat domain 39B isoform 3 precursor [Homo sapiens] | [18.0](http://blast.ncbi.nlm.nih.gov/Blast.cgi#270265889%23270265889) | 18.0 | 75% | 518 |
| [NP_689787.2](http://www.ncbi.nlm.nih.gov/entrez/query.fcgi?cmd=Retrieve&db=Protein&list_uids=270265887&dopt=GenPept&RID=T0BFYNHX01S&log$=prottop&blast_rank=26) | tetratricopeptide repeat domain 39B isoform 1 precursor [Homo sapiens] | [18.0](http://blast.ncbi.nlm.nih.gov/Blast.cgi#270265887%23270265887) | 18.0 | 75% | 518 |
| [NP_001161811.1](http://www.ncbi.nlm.nih.gov/entrez/query.fcgi?cmd=Retrieve&db=Protein&list_uids=270265883&dopt=GenPept&RID=T0BFYNHX01S&log$=prottop&blast_rank=27) | tetratricopeptide repeat domain 39B isoform 2 precursor [Homo sapiens] | [18.0](http://blast.ncbi.nlm.nih.gov/Blast.cgi#270265883%23270265883) | 18.0 | 75% | 518 |
| [NP_001139447.1](http://www.ncbi.nlm.nih.gov/entrez/query.fcgi?cmd=Retrieve&db=Protein&list_uids=225637549&dopt=GenPept&RID=T0BFYNHX01S&log$=prottop&blast_rank=28) | solute carrier family 13, member 2 isoform a [Homo sapiens] | [18.0](http://blast.ncbi.nlm.nih.gov/Blast.cgi#225637549%23225637549) | 18.0 | 75% | 518 |
| [NP_001136438.1](http://www.ncbi.nlm.nih.gov/entrez/query.fcgi?cmd=Retrieve&db=Protein&list_uids=219282747&dopt=GenPept&RID=T0BFYNHX01S&log$=prottop&blast_rank=29) | growth regulation by estrogen in breast cancer-like [Homo sapiens] | [18.0](http://blast.ncbi.nlm.nih.gov/Blast.cgi#219282747%23219282747) | 18.0 | 62% | 518 |
| [NP_004232.2](http://www.ncbi.nlm.nih.gov/entrez/query.fcgi?cmd=Retrieve&db=Protein&list_uids=68342036&dopt=GenPept&RID=T0BFYNHX01S&log$=prottop&blast_rank=30) | jumonji domain containing 1C isoform b [Homo sapiens] | [18.0](http://blast.ncbi.nlm.nih.gov/Blast.cgi#68342036%2368342036) | 28.4 | 87% | 518 |
| [NP_742108.2](http://www.ncbi.nlm.nih.gov/entrez/query.fcgi?cmd=Retrieve&db=Protein&list_uids=254911007&dopt=GenPept&RID=T0BFYNHX01S&log$=prottop&blast_rank=31) | eyes absent 2 isoform c [Homo sapiens] | [18.0](http://blast.ncbi.nlm.nih.gov/Blast.cgi#254911007%23254911007) | 18.0 | 75% | 518 |
| [NP_597681.2](http://www.ncbi.nlm.nih.gov/entrez/query.fcgi?cmd=Retrieve&db=Protein&list_uids=110349717&dopt=GenPept&RID=T0BFYNHX01S&log$=prottop&blast_rank=32) | titin isoform novex-2 [Homo sapiens] | [18.0](http://blast.ncbi.nlm.nih.gov/Blast.cgi#110349717%23110349717) | 55.2 | 87% | 518 |
| [NP_596869.3](http://www.ncbi.nlm.nih.gov/entrez/query.fcgi?cmd=Retrieve&db=Protein&list_uids=110349719&dopt=GenPept&RID=T0BFYNHX01S&log$=prottop&blast_rank=33) | titin isoform N2-A [Homo sapiens] | [18.0](http://blast.ncbi.nlm.nih.gov/Blast.cgi#110349719%23110349719) | 65.5 | 100% | 518 |
| [NP_597676.2](http://www.ncbi.nlm.nih.gov/entrez/query.fcgi?cmd=Retrieve&db=Protein&list_uids=110349713&dopt=GenPept&RID=T0BFYNHX01S&log$=prottop&blast_rank=34) | titin isoform novex-1 [Homo sapiens] | [18.0](http://blast.ncbi.nlm.nih.gov/Blast.cgi#110349713%23110349713) | 55.2 | 87% | 518 |
| [NP_003310.3](http://www.ncbi.nlm.nih.gov/entrez/query.fcgi?cmd=Retrieve&db=Protein&list_uids=110349715&dopt=GenPept&RID=T0BFYNHX01S&log$=prottop&blast_rank=35) | titin isoform N2-B [Homo sapiens] | [18.0](http://blast.ncbi.nlm.nih.gov/Blast.cgi#110349715%23110349715) | 55.2 | 87% | 518 |
| [NP_000712.2](http://www.ncbi.nlm.nih.gov/entrez/query.fcgi?cmd=Retrieve&db=Protein&list_uids=53832005&dopt=GenPept&RID=T0BFYNHX01S&log$=prottop&blast_rank=36) | calcium channel, voltage-dependent, R type, alpha 1E subunit [Homo sapiens] | [18.0](http://blast.ncbi.nlm.nih.gov/Blast.cgi#53832005%2353832005) | 18.0 | 75% | 518 |
| [NP_059129.3](http://www.ncbi.nlm.nih.gov/entrez/query.fcgi?cmd=Retrieve&db=Protein&list_uids=145275208&dopt=GenPept&RID=T0BFYNHX01S&log$=prottop&blast_rank=37) | myosin IIIA [Homo sapiens] | [18.0](http://blast.ncbi.nlm.nih.gov/Blast.cgi#145275208%23145275208) | 18.0 | 62% | 518 |
| [NP_116165.1](http://www.ncbi.nlm.nih.gov/entrez/query.fcgi?cmd=Retrieve&db=Protein&list_uids=118600981&dopt=GenPept&RID=T0BFYNHX01S&log$=prottop&blast_rank=38) | jumonji domain containing 1C isoform a [Homo sapiens] | [18.0](http://blast.ncbi.nlm.nih.gov/Blast.cgi#118600981%23118600981) | 28.4 | 87% | 518 |
| [NP_065143.2](http://www.ncbi.nlm.nih.gov/entrez/query.fcgi?cmd=Retrieve&db=Protein&list_uids=170016077&dopt=GenPept&RID=T0BFYNHX01S&log$=prottop&blast_rank=39) | ATPase type 13A1 [Homo sapiens] | [18.0](http://blast.ncbi.nlm.nih.gov/Blast.cgi#170016077%23170016077) | 33.5 | 87% | 518 |
| [NP_005235.3](http://www.ncbi.nlm.nih.gov/entrez/query.fcgi?cmd=Retrieve&db=Protein&list_uids=26667227&dopt=GenPept&RID=T0BFYNHX01S&log$=prottop&blast_rank=40) | eyes absent 2 isoform a [Homo sapiens] | [18.0](http://blast.ncbi.nlm.nih.gov/Blast.cgi#26667227%2326667227) | 18.0 | 75% | 518 |
| [NP_003975.1](http://www.ncbi.nlm.nih.gov/entrez/query.fcgi?cmd=Retrieve&db=Protein&list_uids=4506979&dopt=GenPept&RID=T0BFYNHX01S&log$=prottop&blast_rank=41) | solute carrier family 13, member 2 isoform b [Homo sapiens] | [18.0](http://blast.ncbi.nlm.nih.gov/Blast.cgi#4506979%234506979) | 18.0 | 75% | 518 |
| [NP_063950.2](http://www.ncbi.nlm.nih.gov/entrez/query.fcgi?cmd=Retrieve&db=Protein&list_uids=167830488&dopt=GenPept&RID=T0BFYNHX01S&log$=prottop&blast_rank=42) | olfactory receptor, family 2, subfamily S, member 2 [Homo sapiens] | [18.0](http://blast.ncbi.nlm.nih.gov/Blast.cgi#167830488%23167830488) | 18.0 | 62% | 518 |
| [NP_573573.2](http://www.ncbi.nlm.nih.gov/entrez/query.fcgi?cmd=Retrieve&db=Protein&list_uids=254911082&dopt=GenPept&RID=T0BFYNHX01S&log$=prottop&blast_rank=43) | SH3 and multiple ankyrin repeat domains 2 isoform 2 [Homo sapiens] | [17.6](http://blast.ncbi.nlm.nih.gov/Blast.cgi#254911082%23254911082) | 31.8 | 87% | 695 |
| [NP_036441.2](http://www.ncbi.nlm.nih.gov/entrez/query.fcgi?cmd=Retrieve&db=Protein&list_uids=226817313&dopt=GenPept&RID=T0BFYNHX01S&log$=prottop&blast_rank=44) | SH3 and multiple ankyrin repeat domains 2 isoform 1 [Homo sapiens] | [17.6](http://blast.ncbi.nlm.nih.gov/Blast.cgi#226817313%23226817313) | 31.8 | 87% | 695 |
| [NP_001073906.1](http://www.ncbi.nlm.nih.gov/entrez/query.fcgi?cmd=Retrieve&db=Protein&list_uids=122937283&dopt=GenPept&RID=T0BFYNHX01S&log$=prottop&blast_rank=45) | 6720455I24Rik homolog precursor [Homo sapiens] | [17.6](http://blast.ncbi.nlm.nih.gov/Blast.cgi#122937283%23122937283) | 17.6 | 87% | 695 |
| [NP_115532.2](http://www.ncbi.nlm.nih.gov/entrez/query.fcgi?cmd=Retrieve&db=Protein&list_uids=50428931&dopt=GenPept&RID=T0BFYNHX01S&log$=prottop&blast_rank=46) | C1q domain containing 1 isoform 3 [Homo sapiens] | [17.6](http://blast.ncbi.nlm.nih.gov/Blast.cgi#50428931%2350428931) | 17.6 | 75% | 695 |
| [NP_001002259.1](http://www.ncbi.nlm.nih.gov/entrez/query.fcgi?cmd=Retrieve&db=Protein&list_uids=50428933&dopt=GenPept&RID=T0BFYNHX01S&log$=prottop&blast_rank=47) | C1q domain containing 1 isoform 1 [Homo sapiens] | [17.6](http://blast.ncbi.nlm.nih.gov/Blast.cgi#50428933%2350428933) | 17.6 | 75% | 695 |
| [NP_001001961.1](http://www.ncbi.nlm.nih.gov/entrez/query.fcgi?cmd=Retrieve&db=Protein&list_uids=50233846&dopt=GenPept&RID=T0BFYNHX01S&log$=prottop&blast_rank=48) | olfactory receptor, family 13, subfamily C, member 3 [Homo sapiens] | [17.6](http://blast.ncbi.nlm.nih.gov/Blast.cgi#50233846%2350233846) | 17.6 | 75% | 695 |
| [NP_076414.2](http://www.ncbi.nlm.nih.gov/entrez/query.fcgi?cmd=Retrieve&db=Protein&list_uids=23503235&dopt=GenPept&RID=T0BFYNHX01S&log$=prottop&blast_rank=49) | C1q domain containing 1 isoform 2 [Homo sapiens] | [17.6](http://blast.ncbi.nlm.nih.gov/Blast.cgi#23503235%2323503235) | 17.6 | 75% | 695 |
| [NP_001447.2](http://www.ncbi.nlm.nih.gov/entrez/query.fcgi?cmd=Retrieve&db=Protein&list_uids=116063573&dopt=GenPept&RID=T0BFYNHX01S&log$=prottop&blast_rank=50) | filamin A, alpha isoform 1 [Homo sapiens] | [17.6](http://blast.ncbi.nlm.nih.gov/Blast.cgi#116063573%23116063573) | 43.5 | 100% | 695 |
| [NP_443078.1](http://www.ncbi.nlm.nih.gov/entrez/query.fcgi?cmd=Retrieve&db=Protein&list_uids=45433501&dopt=GenPept&RID=T0BFYNHX01S&log$=prottop&blast_rank=51) | elastin microfibril interfacer 3 [Homo sapiens] | [17.6](http://blast.ncbi.nlm.nih.gov/Blast.cgi#45433501%2345433501) | 17.6 | 100% | 695 |
| [NP_001104026.1](http://www.ncbi.nlm.nih.gov/entrez/query.fcgi?cmd=Retrieve&db=Protein&list_uids=160420317&dopt=GenPept&RID=T0BFYNHX01S&log$=prottop&blast_rank=52) | filamin A, alpha isoform 2 [Homo sapiens] | [17.6](http://blast.ncbi.nlm.nih.gov/Blast.cgi#160420317%23160420317) | 43.5 | 100% | 695 |
| [NP_001004483.1](http://www.ncbi.nlm.nih.gov/entrez/query.fcgi?cmd=Retrieve&db=Protein&list_uids=52218864&dopt=GenPept&RID=T0BFYNHX01S&log$=prottop&blast_rank=53) | olfactory receptor, family 13, subfamily C, member 8 [Homo sapiens] | [17.6](http://blast.ncbi.nlm.nih.gov/Blast.cgi#52218864%2352218864) | 17.6 | 75% | 695 |
| [XP_002343563.1](http://www.ncbi.nlm.nih.gov/entrez/query.fcgi?cmd=Retrieve&db=Protein&list_uids=239745844&dopt=GenPept&RID=T0BFYNHX01S&log$=prottop&blast_rank=54) | PREDICTED: hypothetical protein XP_002343563 [Homo sapiens] >ref|XP_002347810.1| PREDICTED: hypothetical protein XP_002347810 [Homo sapiens] >ref|XP_002345075.1| PREDICTED: hypothetical protein [Homo sapiens] | [17.2](http://blast.ncbi.nlm.nih.gov/Blast.cgi#239745844%23239745844) | 17.2 | 87% | 932 |
| [XP_002342981.1](http://www.ncbi.nlm.nih.gov/entrez/query.fcgi?cmd=Retrieve&db=Protein&list_uids=239743861&dopt=GenPept&RID=T0BFYNHX01S&log$=prottop&blast_rank=55) | PREDICTED: hypothetical protein XP_002342981 [Homo sapiens] | [17.2](http://blast.ncbi.nlm.nih.gov/Blast.cgi#239743861%23239743861) | 17.2 | 62% | 932 |
| [XP_001716863.1](http://www.ncbi.nlm.nih.gov/entrez/query.fcgi?cmd=Retrieve&db=Protein&list_uids=169205942&dopt=GenPept&RID=T0BFYNHX01S&log$=prottop&blast_rank=56) | PREDICTED: hypothetical protein [Homo sapiens] >ref|XP_001718483.1| PREDICTED: hypothetical protein [Homo sapiens] | [17.2](http://blast.ncbi.nlm.nih.gov/Blast.cgi#169205942%23169205942) | 28.0 | 62% | 932 |
| [XP_001716867.1](http://www.ncbi.nlm.nih.gov/entrez/query.fcgi?cmd=Retrieve&db=Protein&list_uids=169163744&dopt=GenPept&RID=T0BFYNHX01S&log$=prottop&blast_rank=57) | PREDICTED: similar to ACR [Homo sapiens] | [17.2](http://blast.ncbi.nlm.nih.gov/Blast.cgi#169163744%23169163744) | 17.2 | 87% | 932 |
| [XP_002347096.1](http://www.ncbi.nlm.nih.gov/entrez/query.fcgi?cmd=Retrieve&db=Protein&list_uids=239749594&dopt=GenPept&RID=T0BFYNHX01S&log$=prottop&blast_rank=58) | PREDICTED: hypothetical protein XP_002347096 [Homo sapiens] | [17.2](http://blast.ncbi.nlm.nih.gov/Blast.cgi#239749594%23239749594) | 17.2 | 62% | 932 |
| [NP_061985.2](http://www.ncbi.nlm.nih.gov/entrez/query.fcgi?cmd=Retrieve&db=Protein&list_uids=150417984&dopt=GenPept&RID=T0BFYNHX01S&log$=prottop&blast_rank=59) | ATP-binding cassette, sub-family A, member 7 [Homo sapiens] | [17.2](http://blast.ncbi.nlm.nih.gov/Blast.cgi#150417984%23150417984) | 29.7 | 100% | 932 |
| [NP_068506.2](http://www.ncbi.nlm.nih.gov/entrez/query.fcgi?cmd=Retrieve&db=Protein&list_uids=150417973&dopt=GenPept&RID=T0BFYNHX01S&log$=prottop&blast_rank=60) | supervillin isoform 2 [Homo sapiens] | [17.2](http://blast.ncbi.nlm.nih.gov/Blast.cgi#150417973%23150417973) | 33.1 | 100% | 932 |
| [NP_003676.2](http://www.ncbi.nlm.nih.gov/entrez/query.fcgi?cmd=Retrieve&db=Protein&list_uids=154355000&dopt=GenPept&RID=T0BFYNHX01S&log$=prottop&blast_rank=61) | KH-type splicing regulatory protein [Homo sapiens] | [17.2](http://blast.ncbi.nlm.nih.gov/Blast.cgi#154355000%23154355000) | 17.2 | 62% | 932 |
| [NP_001088.2](http://www.ncbi.nlm.nih.gov/entrez/query.fcgi?cmd=Retrieve&db=Protein&list_uids=148613878&dopt=GenPept&RID=T0BFYNHX01S&log$=prottop&blast_rank=62) | acrosin precursor [Homo sapiens] | [17.2](http://blast.ncbi.nlm.nih.gov/Blast.cgi#148613878%23148613878) | 17.2 | 87% | 932 |
| [NP_003475.1](http://www.ncbi.nlm.nih.gov/entrez/query.fcgi?cmd=Retrieve&db=Protein&list_uids=62912482&dopt=GenPept&RID=T0BFYNHX01S&log$=prottop&blast_rank=63) | high mobility group AT-hook 2 isoform b [Homo sapiens] | [17.2](http://blast.ncbi.nlm.nih.gov/Blast.cgi#62912482%2362912482) | 17.2 | 62% | 932 |
| [NP_001035723.1](http://www.ncbi.nlm.nih.gov/entrez/query.fcgi?cmd=Retrieve&db=Protein&list_uids=100913192&dopt=GenPept&RID=T0BFYNHX01S&log$=prottop&blast_rank=64) | AMP-activated protein kinase gamma2 subunit isoform c [Homo sapiens] | [17.2](http://blast.ncbi.nlm.nih.gov/Blast.cgi#100913192%23100913192) | 17.2 | 62% | 932 |
| [NP_976063.1](http://www.ncbi.nlm.nih.gov/entrez/query.fcgi?cmd=Retrieve&db=Protein&list_uids=42794779&dopt=GenPept&RID=T0BFYNHX01S&log$=prottop&blast_rank=65) | myosin 18A isoform b [Homo sapiens] | [17.2](http://blast.ncbi.nlm.nih.gov/Blast.cgi#42794779%2342794779) | 32.7 | 75% | 932 |
| [NP_004367.2](http://www.ncbi.nlm.nih.gov/entrez/query.fcgi?cmd=Retrieve&db=Protein&list_uids=17921987&dopt=GenPept&RID=T0BFYNHX01S&log$=prottop&blast_rank=66) | COX15 homolog isoform 2 [Homo sapiens] | [17.2](http://blast.ncbi.nlm.nih.gov/Blast.cgi#17921987%2317921987) | 17.2 | 62% | 932 |
| [NP_002474.3](http://www.ncbi.nlm.nih.gov/entrez/query.fcgi?cmd=Retrieve&db=Protein&list_uids=40255013&dopt=GenPept&RID=T0BFYNHX01S&log$=prottop&blast_rank=67) | carcinoembryonic antigen-related cell adhesion molecule 6 precursor [Homo sapiens] | [17.2](http://blast.ncbi.nlm.nih.gov/Blast.cgi#40255013%2340255013) | 17.2 | 75% | 932 |
| [NP_002400.3](http://www.ncbi.nlm.nih.gov/entrez/query.fcgi?cmd=Retrieve&db=Protein&list_uids=148539888&dopt=GenPept&RID=T0BFYNHX01S&log$=prottop&blast_rank=68) | mannosyl (beta-1,4-)-glycoprotein beta-1,4-N-acetylglucosaminyltransferase [Homo sapiens] >ref|NP_001091740.1| mannosyl (beta-1,4-)-glycoprotein beta-1,4-N-acetylglucosaminyltransferase [Homo sapiens] | [17.2](http://blast.ncbi.nlm.nih.gov/Blast.cgi#148539888%23148539888) | 17.2 | 62% | 932 |
| [NP_060542.4](http://www.ncbi.nlm.nih.gov/entrez/query.fcgi?cmd=Retrieve&db=Protein&list_uids=73695475&dopt=GenPept&RID=T0BFYNHX01S&log$=prottop&blast_rank=69) | protein BAP28 [Homo sapiens] | [17.2](http://blast.ncbi.nlm.nih.gov/Blast.cgi#73695475%2373695475) | 17.2 | 87% | 932 |
| [NP_005493.2](http://www.ncbi.nlm.nih.gov/entrez/query.fcgi?cmd=Retrieve&db=Protein&list_uids=21536376&dopt=GenPept&RID=T0BFYNHX01S&log$=prottop&blast_rank=70) | ATP-binding cassette, sub-family A member 1 [Homo sapiens] | [17.2](http://blast.ncbi.nlm.nih.gov/Blast.cgi#21536376%2321536376) | 17.2 | 100% | 932 |
| [NP_001004481.1](http://www.ncbi.nlm.nih.gov/entrez/query.fcgi?cmd=Retrieve&db=Protein&list_uids=52317180&dopt=GenPept&RID=T0BFYNHX01S&log$=prottop&blast_rank=71) | olfactory receptor, family 13, subfamily C, member 2 [Homo sapiens] | [17.2](http://blast.ncbi.nlm.nih.gov/Blast.cgi#52317180%2352317180) | 17.2 | 75% | 932 |
| [NP_005874.1](http://www.ncbi.nlm.nih.gov/entrez/query.fcgi?cmd=Retrieve&db=Protein&list_uids=5031587&dopt=GenPept&RID=T0BFYNHX01S&log$=prottop&blast_rank=72) | adenomatosis polyposis coli 2 [Homo sapiens] | [17.2](http://blast.ncbi.nlm.nih.gov/Blast.cgi#5031587%235031587) | 17.2 | 62% | 932 |
| [NP_002617.3](http://www.ncbi.nlm.nih.gov/entrez/query.fcgi?cmd=Retrieve&db=Protein&list_uids=48762920&dopt=GenPept&RID=T0BFYNHX01S&log$=prottop&blast_rank=73) | liver phosphofructokinase [Homo sapiens] | [17.2](http://blast.ncbi.nlm.nih.gov/Blast.cgi#48762920%2348762920) | 32.7 | 75% | 932 |
| [NP_510880.2](http://www.ncbi.nlm.nih.gov/entrez/query.fcgi?cmd=Retrieve&db=Protein&list_uids=28416946&dopt=GenPept&RID=T0BFYNHX01S&log$=prottop&blast_rank=74) | myosin 18A isoform a [Homo sapiens] | [17.2](http://blast.ncbi.nlm.nih.gov/Blast.cgi#28416946%2328416946) | 32.7 | 75% | 932 |
| [NP_510870.1](http://www.ncbi.nlm.nih.gov/entrez/query.fcgi?cmd=Retrieve&db=Protein&list_uids=17921985&dopt=GenPept&RID=T0BFYNHX01S&log$=prottop&blast_rank=75) | COX15 homolog isoform 1 [Homo sapiens] | [17.2](http://blast.ncbi.nlm.nih.gov/Blast.cgi#17921985%2317921985) | 17.2 | 62% | 932 |
| [NP_055458.1](http://www.ncbi.nlm.nih.gov/entrez/query.fcgi?cmd=Retrieve&db=Protein&list_uids=7662010&dopt=GenPept&RID=T0BFYNHX01S&log$=prottop&blast_rank=76) | zinc finger protein 516 [Homo sapiens] | [17.2](http://blast.ncbi.nlm.nih.gov/Blast.cgi#7662010%237662010) | 28.4 | 100% | 932 |
| [NP_001004482.1](http://www.ncbi.nlm.nih.gov/entrez/query.fcgi?cmd=Retrieve&db=Protein&list_uids=52218854&dopt=GenPept&RID=T0BFYNHX01S&log$=prottop&blast_rank=77) | olfactory receptor, family 13, subfamily C, member 5 [Homo sapiens] | [17.2](http://blast.ncbi.nlm.nih.gov/Blast.cgi#52218854%2352218854) | 17.2 | 75% | 932 |
| [NP_005981.3](http://www.ncbi.nlm.nih.gov/entrez/query.fcgi?cmd=Retrieve&db=Protein&list_uids=126362971&dopt=GenPept&RID=T0BFYNHX01S&log$=prottop&blast_rank=78) | serine/threonine kinase 10 [Homo sapiens] | [17.2](http://blast.ncbi.nlm.nih.gov/Blast.cgi#126362971%23126362971) | 17.2 | 62% | 932 |
| [NP_003474.1](http://www.ncbi.nlm.nih.gov/entrez/query.fcgi?cmd=Retrieve&db=Protein&list_uids=4504431&dopt=GenPept&RID=T0BFYNHX01S&log$=prottop&blast_rank=79) | high mobility group AT-hook 2 isoform a [Homo sapiens] | [17.2](http://blast.ncbi.nlm.nih.gov/Blast.cgi#4504431%234504431) | 17.2 | 62% | 932 |
| [NP_057287.2](http://www.ncbi.nlm.nih.gov/entrez/query.fcgi?cmd=Retrieve&db=Protein&list_uids=33186925&dopt=GenPept&RID=T0BFYNHX01S&log$=prottop&blast_rank=80) | AMP-activated protein kinase gamma2 subunit isoform a [Homo sapiens] | [17.2](http://blast.ncbi.nlm.nih.gov/Blast.cgi#33186925%2333186925) | 17.2 | 62% | 932 |
| [NP_001139400.1](http://www.ncbi.nlm.nih.gov/entrez/query.fcgi?cmd=Retrieve&db=Protein&list_uids=225579127&dopt=GenPept&RID=T0BFYNHX01S&log$=prottop&blast_rank=81) | Sin3A-associated protein, 130kDa isoform a [Homo sapiens] | [16.8](http://blast.ncbi.nlm.nih.gov/Blast.cgi#225579127%23225579127) | 16.8 | 75% | 1251 |
| [NP_078964.2](http://www.ncbi.nlm.nih.gov/entrez/query.fcgi?cmd=Retrieve&db=Protein&list_uids=157739936&dopt=GenPept&RID=T0BFYNHX01S&log$=prottop&blast_rank=82) | chromosome 10 open reading frame 68 [Homo sapiens] | [16.8](http://blast.ncbi.nlm.nih.gov/Blast.cgi#157739936%23157739936) | 16.8 | 75% | 1251 |
| [NP_004652.2](http://www.ncbi.nlm.nih.gov/entrez/query.fcgi?cmd=Retrieve&db=Protein&list_uids=118402596&dopt=GenPept&RID=T0BFYNHX01S&log$=prottop&blast_rank=83) | cell division cycle protein 23 [Homo sapiens] | [16.8](http://blast.ncbi.nlm.nih.gov/Blast.cgi#118402596%23118402596) | 29.3 | 87% | 1251 |
| [NP_073713.2](http://www.ncbi.nlm.nih.gov/entrez/query.fcgi?cmd=Retrieve&db=Protein&list_uids=145580575&dopt=GenPept&RID=T0BFYNHX01S&log$=prottop&blast_rank=84) | C-terminal binding protein 2 isoform 2 [Homo sapiens] | [16.8](http://blast.ncbi.nlm.nih.gov/Blast.cgi#145580575%23145580575) | 40.5 | 75% | 1251 |
| [NP_002989.2](http://www.ncbi.nlm.nih.gov/entrez/query.fcgi?cmd=Retrieve&db=Protein&list_uids=40548378&dopt=GenPept&RID=T0BFYNHX01S&log$=prottop&blast_rank=85) | syndecan 2 precursor [Homo sapiens] | [16.8](http://blast.ncbi.nlm.nih.gov/Blast.cgi#40548378%2340548378) | 16.8 | 75% | 1251 |
| [NP_060568.3](http://www.ncbi.nlm.nih.gov/entrez/query.fcgi?cmd=Retrieve&db=Protein&list_uids=21735572&dopt=GenPept&RID=T0BFYNHX01S&log$=prottop&blast_rank=86) | epithelial cell transforming sequence 2 oncogene protein [Homo sapiens] | [16.8](http://blast.ncbi.nlm.nih.gov/Blast.cgi#21735572%2321735572) | 16.8 | 100% | 1251 |
| [NP_001157329.1](http://www.ncbi.nlm.nih.gov/entrez/query.fcgi?cmd=Retrieve&db=Protein&list_uids=255759947&dopt=GenPept&RID=T0BFYNHX01S&log$=prottop&blast_rank=87) | hypothetical protein LOC339669 isoform 1 [Homo sapiens] | [16.8](http://blast.ncbi.nlm.nih.gov/Blast.cgi#255759947%23255759947) | 16.8 | 75% | 1251 |
| [NP_078821.2](http://www.ncbi.nlm.nih.gov/entrez/query.fcgi?cmd=Retrieve&db=Protein&list_uids=19923597&dopt=GenPept&RID=T0BFYNHX01S&log$=prottop&blast_rank=88) | Sin3A-associated protein, 130kDa isoform b [Homo sapiens] | [16.8](http://blast.ncbi.nlm.nih.gov/Blast.cgi#19923597%2319923597) | 16.8 | 75% | 1251 |
| [NP_001001956.1](http://www.ncbi.nlm.nih.gov/entrez/query.fcgi?cmd=Retrieve&db=Protein&list_uids=50080197&dopt=GenPept&RID=T0BFYNHX01S&log$=prottop&blast_rank=89) | olfactory receptor, family 13, subfamily C, member 9 [Homo sapiens] | [16.8](http://blast.ncbi.nlm.nih.gov/Blast.cgi#50080197%2350080197) | 16.8 | 75% | 1251 |
| [XP_002347644.1](http://www.ncbi.nlm.nih.gov/entrez/query.fcgi?cmd=Retrieve&db=Protein&list_uids=239747524&dopt=GenPept&RID=T0BFYNHX01S&log$=prottop&blast_rank=90) | PREDICTED: similar to serum response factor-related protein C4 [Homo sapiens] | [16.3](http://blast.ncbi.nlm.nih.gov/Blast.cgi#239747524%23239747524) | 27.2 | 100% | 1678 |
| [NP_851851.2](http://www.ncbi.nlm.nih.gov/entrez/query.fcgi?cmd=Retrieve&db=Protein&list_uids=226423858&dopt=GenPept&RID=T0BFYNHX01S&log$=prottop&blast_rank=91) | PRR5-ARHGAP8 fusion protein [Homo sapiens] | [16.3](http://blast.ncbi.nlm.nih.gov/Blast.cgi#226423858%23226423858) | 16.3 | 75% | 1678 |
| [NP_001165907.1](http://www.ncbi.nlm.nih.gov/entrez/query.fcgi?cmd=Retrieve&db=Protein&list_uids=289176994&dopt=GenPept&RID=T0BFYNHX01S&log$=prottop&blast_rank=92) | phosphorylase b kinase regulatory subunit alpha skeletal muscle isoform isoform 3 [Homo sapiens] | [16.3](http://blast.ncbi.nlm.nih.gov/Blast.cgi#289176994%23289176994) | 16.3 | 75% | 1678 |
| [XP_001716209.1](http://www.ncbi.nlm.nih.gov/entrez/query.fcgi?cmd=Retrieve&db=Protein&list_uids=169163718&dopt=GenPept&RID=T0BFYNHX01S&log$=prottop&blast_rank=93) | PREDICTED: hypothetical protein [Homo sapiens] | [16.3](http://blast.ncbi.nlm.nih.gov/Blast.cgi#169163718%23169163718) | 16.3 | 75% | 1678 |
| [XP_001715564.1](http://www.ncbi.nlm.nih.gov/entrez/query.fcgi?cmd=Retrieve&db=Protein&list_uids=169163704&dopt=GenPept&RID=T0BFYNHX01S&log$=prottop&blast_rank=94) | PREDICTED: hypothetical protein [Homo sapiens] | [16.3](http://blast.ncbi.nlm.nih.gov/Blast.cgi#169163704%23169163704) | 16.3 | 75% | 1678 |
| [NP_996830.3](http://www.ncbi.nlm.nih.gov/entrez/query.fcgi?cmd=Retrieve&db=Protein&list_uids=154091310&dopt=GenPept&RID=T0BFYNHX01S&log$=prottop&blast_rank=95) | transient receptor potential cation channel, subfamily M, member 3 isoform g [Homo sapiens] | [16.3](http://blast.ncbi.nlm.nih.gov/Blast.cgi#154091310%23154091310) | 30.5 | 100% | 1678 |
| [XP_001724436.1](http://www.ncbi.nlm.nih.gov/entrez/query.fcgi?cmd=Retrieve&db=Protein&list_uids=169170962&dopt=GenPept&RID=T0BFYNHX01S&log$=prottop&blast_rank=96) | PREDICTED: similar to hCG1804306 [Homo sapiens] >ref|XP_001723071.1| PREDICTED: similar to hCG1804306 [Homo sapiens] >ref|XP_001724468.1| PREDICTED: similar to hCG1804306 [Homo sapiens] >ref|XP_002344310.1| PREDICTED: similar to hCG1804306 [Homo sapiens] | [16.3](http://blast.ncbi.nlm.nih.gov/Blast.cgi#169170962%23169170962) | 16.3 | 62% | 1678 |
| [NP_079247.5](http://www.ncbi.nlm.nih.gov/entrez/query.fcgi?cmd=Retrieve&db=Protein&list_uids=154091312&dopt=GenPept&RID=T0BFYNHX01S&log$=prottop&blast_rank=97) | transient receptor potential cation channel, subfamily M, member 3 isoform b [Homo sapiens] | [16.3](http://blast.ncbi.nlm.nih.gov/Blast.cgi#154091312%23154091312) | 30.5 | 100% | 1678 |
| [NP_066003.3](http://www.ncbi.nlm.nih.gov/entrez/query.fcgi?cmd=Retrieve&db=Protein&list_uids=154091320&dopt=GenPept&RID=T0BFYNHX01S&log$=prottop&blast_rank=98) | transient receptor potential cation channel, subfamily M, member 3 isoform a [Homo sapiens] | [16.3](http://blast.ncbi.nlm.nih.gov/Blast.cgi#154091320%23154091320) | 30.5 | 100% | 1678 |
| [NP_996827.3](http://www.ncbi.nlm.nih.gov/entrez/query.fcgi?cmd=Retrieve&db=Protein&list_uids=154091318&dopt=GenPept&RID=T0BFYNHX01S&log$=prottop&blast_rank=99) | transient receptor potential cation channel, subfamily M, member 3 isoform d [Homo sapiens] | [16.3](http://blast.ncbi.nlm.nih.gov/Blast.cgi#154091318%23154091318) | 30.5 | 100% | 1678 |
| [NP_996828.3](http://www.ncbi.nlm.nih.gov/entrez/query.fcgi?cmd=Retrieve&db=Protein&list_uids=154091316&dopt=GenPept&RID=T0BFYNHX01S&log$=prottop&blast_rank=100) | transient receptor potential cation channel, subfamily M, member 3 isoform e [Homo sapiens] | [16.3](http://blast.ncbi.nlm.nih.gov/Blast.cgi#154091316%23154091316) | 30.5 | 100% | 1678 |

| **Accession** | **Proteins with a match to QDLYSSA peptide** | **[Max score](http://blast.ncbi.nlm.nih.gov/Blast.cgi?CMD=Get&ALIGNMENTS=100&ALIGNMENT_VIEW=Pairwise&CDD_SEARCH_STATE=1&DATABASE_SORT=0&DESCRIPTIONS=100&ENTREZ_QUERY=txid9606 %5BORGN%5D&FIRST_QUERY_NUM=0&FORMAT_OBJECT=Alignment&FORMAT_PAGE_TARGET=&FORMAT_TYPE=HTML&GET_SEQUENCE=yes&I_THRESH=&MASK_CHAR=2&MASK_COLOR=1&NEW_DESIGN=on&NEW_VIEW=yes&NUM_OVERVIEW=100&OLD_BLAST=false&PAGE=Proteins&QUERY_INDEX=0&QUERY_NUMBER=0&RESULTS_PAGE_TARGET=&RID=T0C9V6B301S&SHOW_LINKOUT=yes&SHOW_OVERVIEW=yes&STEP_NUMBER=&WORD_SIZE=2&DISPLAY_SORT=1&HSP_SORT=1" \l "sort_mark)** | **[Total score](http://blast.ncbi.nlm.nih.gov/Blast.cgi?CMD=Get&ALIGNMENTS=100&ALIGNMENT_VIEW=Pairwise&CDD_SEARCH_STATE=1&DATABASE_SORT=0&DESCRIPTIONS=100&ENTREZ_QUERY=txid9606 %5BORGN%5D&FIRST_QUERY_NUM=0&FORMAT_OBJECT=Alignment&FORMAT_PAGE_TARGET=&FORMAT_TYPE=HTML&GET_SEQUENCE=yes&I_THRESH=&MASK_CHAR=2&MASK_COLOR=1&NEW_DESIGN=on&NEW_VIEW=yes&NUM_OVERVIEW=100&OLD_BLAST=false&PAGE=Proteins&QUERY_INDEX=0&QUERY_NUMBER=0&RESULTS_PAGE_TARGET=&RID=T0C9V6B301S&SHOW_LINKOUT=yes&SHOW_OVERVIEW=yes&STEP_NUMBER=&WORD_SIZE=2&DISPLAY_SORT=2&HSP_SORT=1" \l "sort_mark)** | **[Query coverage](http://blast.ncbi.nlm.nih.gov/Blast.cgi?CMD=Get&ALIGNMENTS=100&ALIGNMENT_VIEW=Pairwise&CDD_SEARCH_STATE=1&DATABASE_SORT=0&DESCRIPTIONS=100&ENTREZ_QUERY=txid9606 %5BORGN%5D&FIRST_QUERY_NUM=0&FORMAT_OBJECT=Alignment&FORMAT_PAGE_TARGET=&FORMAT_TYPE=HTML&GET_SEQUENCE=yes&I_THRESH=&MASK_CHAR=2&MASK_COLOR=1&NEW_DESIGN=on&NEW_VIEW=yes&NUM_OVERVIEW=100&OLD_BLAST=false&PAGE=Proteins&QUERY_INDEX=0&QUERY_NUMBER=0&RESULTS_PAGE_TARGET=&RID=T0C9V6B301S&SHOW_LINKOUT=yes&SHOW_OVERVIEW=yes&STEP_NUMBER=&WORD_SIZE=2&DISPLAY_SORT=4&HSP_SORT=0" \l "sort_mark)** | **[E value](http://blast.ncbi.nlm.nih.gov/Blast.cgi?CMD=Get&ALIGNMENTS=100&ALIGNMENT_VIEW=Pairwise&CDD_SEARCH_STATE=1&DATABASE_SORT=0&DESCRIPTIONS=100&ENTREZ_QUERY=txid9606 %5BORGN%5D&FIRST_QUERY_NUM=0&FORMAT_OBJECT=Alignment&FORMAT_PAGE_TARGET=&FORMAT_TYPE=HTML&GET_SEQUENCE=yes&I_THRESH=&MASK_CHAR=2&MASK_COLOR=1&NEW_DESIGN=on&NEW_VIEW=yes&NUM_OVERVIEW=100&OLD_BLAST=false&PAGE=Proteins&QUERY_INDEX=0&QUERY_NUMBER=0&RESULTS_PAGE_TARGET=&RID=T0C9V6B301S&SHOW_LINKOUT=yes&SHOW_OVERVIEW=yes&STEP_NUMBER=&WORD_SIZE=2&DISPLAY_SORT=0&HSP_SORT=0" \l "sort_mark)** |
| --- | --- | --- | --- | --- | --- |
| [NP_065789.1](http://www.ncbi.nlm.nih.gov/entrez/query.fcgi?cmd=Retrieve&db=Protein&list_uids=55741641&dopt=GenPept&RID=T0C9V6B301S&log$=prottop&blast_rank=1) | kinase D-interacting substrate of 220 kDa [Homo sapiens] | [21.4](http://blast.ncbi.nlm.nih.gov/Blast.cgi#55741641%2355741641) | 21.4 | 85% | 43 |
| [NP_001555.1](http://www.ncbi.nlm.nih.gov/entrez/query.fcgi?cmd=Retrieve&db=Protein&list_uids=4504695&dopt=GenPept&RID=T0C9V6B301S&log$=prottop&blast_rank=2) | inhibitor of growth family, member 2 [Homo sapiens] | [20.6](http://blast.ncbi.nlm.nih.gov/Blast.cgi#4504695%234504695) | 20.6 | 100% | 78 |
| [NP_001013049.1](http://www.ncbi.nlm.nih.gov/entrez/query.fcgi?cmd=Retrieve&db=Protein&list_uids=61743975&dopt=GenPept&RID=T0C9V6B301S&log$=prottop&blast_rank=3) | SORCS receptor 1 isoform b [Homo sapiens] | [20.2](http://blast.ncbi.nlm.nih.gov/Blast.cgi#61743975%2361743975) | 20.2 | 100% | 104 |
| [NP_443150.3](http://www.ncbi.nlm.nih.gov/entrez/query.fcgi?cmd=Retrieve&db=Protein&list_uids=61743973&dopt=GenPept&RID=T0C9V6B301S&log$=prottop&blast_rank=4) | SORCS receptor 1 isoform a [Homo sapiens] | [20.2](http://blast.ncbi.nlm.nih.gov/Blast.cgi#61743973%2361743973) | 20.2 | 100% | 104 |
| [NP_004539.1](http://www.ncbi.nlm.nih.gov/entrez/query.fcgi?cmd=Retrieve&db=Protein&list_uids=4758774&dopt=GenPept&RID=T0C9V6B301S&log$=prottop&blast_rank=5) | NADH dehydrogenase (ubiquinone) 1 beta subcomplex, 10, 22kDa [Homo sapiens] | [20.2](http://blast.ncbi.nlm.nih.gov/Blast.cgi#4758774%234758774) | 20.2 | 100% | 104 |
| [NP_569711.2](http://www.ncbi.nlm.nih.gov/entrez/query.fcgi?cmd=Retrieve&db=Protein&list_uids=206597445&dopt=GenPept&RID=T0C9V6B301S&log$=prottop&blast_rank=6) | alpha 1 type XVIII collagen isoform 3 precursor [Homo sapiens] | [19.7](http://blast.ncbi.nlm.nih.gov/Blast.cgi#206597445%23206597445) | 19.7 | 71% | 140 |
| [NP_659429.4](http://www.ncbi.nlm.nih.gov/entrez/query.fcgi?cmd=Retrieve&db=Protein&list_uids=118918435&dopt=GenPept&RID=T0C9V6B301S&log$=prottop&blast_rank=7) | von Willebrand factor A domain containing 3B [Homo sapiens] | [19.7](http://blast.ncbi.nlm.nih.gov/Blast.cgi#118918435%23118918435) | 19.7 | 71% | 140 |
| [NP_085059.2](http://www.ncbi.nlm.nih.gov/entrez/query.fcgi?cmd=Retrieve&db=Protein&list_uids=110611235&dopt=GenPept&RID=T0C9V6B301S&log$=prottop&blast_rank=8) | alpha 1 type XVIII collagen isoform 1 precursor [Homo sapiens] | [19.7](http://blast.ncbi.nlm.nih.gov/Blast.cgi#110611235%23110611235) | 19.7 | 71% | 140 |
| [NP_569712.2](http://www.ncbi.nlm.nih.gov/entrez/query.fcgi?cmd=Retrieve&db=Protein&list_uids=110611233&dopt=GenPept&RID=T0C9V6B301S&log$=prottop&blast_rank=9) | alpha 1 type XVIII collagen isoform 2 precursor [Homo sapiens] | [19.7](http://blast.ncbi.nlm.nih.gov/Blast.cgi#110611233%23110611233) | 19.7 | 71% | 140 |
| [NP_005137.1](http://www.ncbi.nlm.nih.gov/entrez/query.fcgi?cmd=Retrieve&db=Protein&list_uids=10863889&dopt=GenPept&RID=T0C9V6B301S&log$=prottop&blast_rank=10) | squamous cell carcinoma antigen recognized by T cells 1 [Homo sapiens] | [19.7](http://blast.ncbi.nlm.nih.gov/Blast.cgi#10863889%2310863889) | 19.7 | 71% | 140 |
| [NP_000535.3](http://www.ncbi.nlm.nih.gov/entrez/query.fcgi?cmd=Retrieve&db=Protein&list_uids=73747915&dopt=GenPept&RID=T0C9V6B301S&log$=prottop&blast_rank=11) | transporter 2, ATP-binding cassette, sub-family B isoform 1 [Homo sapiens] | [19.7](http://blast.ncbi.nlm.nih.gov/Blast.cgi#73747915%2373747915) | 19.7 | 71% | 140 |
| [NP_055405.3](http://www.ncbi.nlm.nih.gov/entrez/query.fcgi?cmd=Retrieve&db=Protein&list_uids=48949851&dopt=GenPept&RID=T0C9V6B301S&log$=prottop&blast_rank=12) | syncytin precursor [Homo sapiens] >ref|NP_001124397.1| syncytin precursor [Homo sapiens] | [19.7](http://blast.ncbi.nlm.nih.gov/Blast.cgi#48949851%2348949851) | 19.7 | 71% | 140 |
| [NP_001073138.1](http://www.ncbi.nlm.nih.gov/entrez/query.fcgi?cmd=Retrieve&db=Protein&list_uids=118766335&dopt=GenPept&RID=T0C9V6B301S&log$=prottop&blast_rank=13) | calcium binding protein 39-like [Homo sapiens] >ref|NP_112187.2| calcium binding protein 39-like [Homo sapiens] | [19.7](http://blast.ncbi.nlm.nih.gov/Blast.cgi#118766335%23118766335) | 19.7 | 85% | 140 |
| [NP_001162023.1](http://www.ncbi.nlm.nih.gov/entrez/query.fcgi?cmd=Retrieve&db=Protein&list_uids=271398379&dopt=GenPept&RID=T0C9V6B301S&log$=prottop&blast_rank=14) | transmembrane protein 48 isoform 2 [Homo sapiens] | [18.9](http://blast.ncbi.nlm.nih.gov/Blast.cgi#271398379%23271398379) | 18.9 | 71% | 252 |
| [XP_002346350.1](http://www.ncbi.nlm.nih.gov/entrez/query.fcgi?cmd=Retrieve&db=Protein&list_uids=239757825&dopt=GenPept&RID=T0C9V6B301S&log$=prottop&blast_rank=15) | PREDICTED: hypothetical protein XP_002346350 [Homo sapiens] | [18.9](http://blast.ncbi.nlm.nih.gov/Blast.cgi#239757825%23239757825) | 18.9 | 71% | 252 |
| [XP_002347226.1](http://www.ncbi.nlm.nih.gov/entrez/query.fcgi?cmd=Retrieve&db=Protein&list_uids=239749951&dopt=GenPept&RID=T0C9V6B301S&log$=prottop&blast_rank=16) | PREDICTED: similar to mucin 2 [Homo sapiens] | [18.9](http://blast.ncbi.nlm.nih.gov/Blast.cgi#239749951%23239749951) | 18.9 | 71% | 252 |
| [NP_001501.2](http://www.ncbi.nlm.nih.gov/entrez/query.fcgi?cmd=Retrieve&db=Protein&list_uids=157384977&dopt=GenPept&RID=T0C9V6B301S&log$=prottop&blast_rank=17) | glutamate receptor, ionotropic, delta 2 precursor [Homo sapiens] | [18.9](http://blast.ncbi.nlm.nih.gov/Blast.cgi#157384977%23157384977) | 18.9 | 71% | 252 |
| [NP_002448.2](http://www.ncbi.nlm.nih.gov/entrez/query.fcgi?cmd=Retrieve&db=Protein&list_uids=116284392&dopt=GenPept&RID=T0C9V6B301S&log$=prottop&blast_rank=18) | mucin 2 precursor [Homo sapiens] | [18.9](http://blast.ncbi.nlm.nih.gov/Blast.cgi#116284392%23116284392) | 18.9 | 71% | 252 |
| [NP_055753.3](http://www.ncbi.nlm.nih.gov/entrez/query.fcgi?cmd=Retrieve&db=Protein&list_uids=111955326&dopt=GenPept&RID=T0C9V6B301S&log$=prottop&blast_rank=19) | MLX interacting protein [Homo sapiens] | [18.9](http://blast.ncbi.nlm.nih.gov/Blast.cgi#111955326%23111955326) | 18.9 | 85% | 252 |
| [NP_062556.2](http://www.ncbi.nlm.nih.gov/entrez/query.fcgi?cmd=Retrieve&db=Protein&list_uids=21361809&dopt=GenPept&RID=T0C9V6B301S&log$=prottop&blast_rank=20) | RNA binding motif protein, X-linked-like 1 [Homo sapiens] >ref|NP_001156008.1| RNA binding motif protein, X-linked-like 1 [Homo sapiens] | [18.9](http://blast.ncbi.nlm.nih.gov/Blast.cgi#21361809%2321361809) | 18.9 | 71% | 252 |
| [NP_001161434.1](http://www.ncbi.nlm.nih.gov/entrez/query.fcgi?cmd=Retrieve&db=Protein&list_uids=269784651&dopt=GenPept&RID=T0C9V6B301S&log$=prottop&blast_rank=21) | prestin isoform e [Homo sapiens] | [18.9](http://blast.ncbi.nlm.nih.gov/Blast.cgi#269784651%23269784651) | 34.4 | 85% | 252 |
| [NP_079229.2](http://www.ncbi.nlm.nih.gov/entrez/query.fcgi?cmd=Retrieve&db=Protein&list_uids=38679914&dopt=GenPept&RID=T0C9V6B301S&log$=prottop&blast_rank=22) | mitochondrial distribution and morphology 20 [Homo sapiens] | [18.9](http://blast.ncbi.nlm.nih.gov/Blast.cgi#38679914%2338679914) | 18.9 | 71% | 252 |
| [NP_996766.1](http://www.ncbi.nlm.nih.gov/entrez/query.fcgi?cmd=Retrieve&db=Protein&list_uids=45827800&dopt=GenPept&RID=T0C9V6B301S&log$=prottop&blast_rank=23) | prestin isoform b [Homo sapiens] | [18.9](http://blast.ncbi.nlm.nih.gov/Blast.cgi#45827800%2345827800) | 34.4 | 85% | 252 |
| [NP_003965.2](http://www.ncbi.nlm.nih.gov/entrez/query.fcgi?cmd=Retrieve&db=Protein&list_uids=41406050&dopt=GenPept&RID=T0C9V6B301S&log$=prottop&blast_rank=24) | docking protein 2 [Homo sapiens] | [18.9](http://blast.ncbi.nlm.nih.gov/Blast.cgi#41406050%2341406050) | 18.9 | 85% | 252 |
| [NP_005404.1](http://www.ncbi.nlm.nih.gov/entrez/query.fcgi?cmd=Retrieve&db=Protein&list_uids=4885597&dopt=GenPept&RID=T0C9V6B301S&log$=prottop&blast_rank=25) | SIX homeobox 3 [Homo sapiens] | [18.9](http://blast.ncbi.nlm.nih.gov/Blast.cgi#4885597%234885597) | 18.9 | 71% | 252 |
| [NP_001599.1](http://www.ncbi.nlm.nih.gov/entrez/query.fcgi?cmd=Retrieve&db=Protein&list_uids=4501857&dopt=GenPept&RID=T0C9V6B301S&log$=prottop&blast_rank=26) | long-chain acyl-CoA dehydrogenase precursor [Homo sapiens] | [18.9](http://blast.ncbi.nlm.nih.gov/Blast.cgi#4501857%234501857) | 18.9 | 85% | 252 |
| [NP_945350.1](http://www.ncbi.nlm.nih.gov/entrez/query.fcgi?cmd=Retrieve&db=Protein&list_uids=39752683&dopt=GenPept&RID=T0C9V6B301S&log$=prottop&blast_rank=27) | prestin isoform a [Homo sapiens] | [18.9](http://blast.ncbi.nlm.nih.gov/Blast.cgi#39752683%2339752683) | 34.4 | 85% | 252 |
| [NP_056273.2](http://www.ncbi.nlm.nih.gov/entrez/query.fcgi?cmd=Retrieve&db=Protein&list_uids=19923424&dopt=GenPept&RID=T0C9V6B301S&log$=prottop&blast_rank=28) | myotubularin related protein 9 [Homo sapiens] | [18.9](http://blast.ncbi.nlm.nih.gov/Blast.cgi#19923424%2319923424) | 18.9 | 85% | 252 |
| [NP_872577.1](http://www.ncbi.nlm.nih.gov/entrez/query.fcgi?cmd=Retrieve&db=Protein&list_uids=33286414&dopt=GenPept&RID=T0C9V6B301S&log$=prottop&blast_rank=29) | Hermansky-Pudlak syndrome 1 protein isoform c [Homo sapiens] | [18.9](http://blast.ncbi.nlm.nih.gov/Blast.cgi#33286414%2333286414) | 18.9 | 85% | 252 |
| [NP_000186.2](http://www.ncbi.nlm.nih.gov/entrez/query.fcgi?cmd=Retrieve&db=Protein&list_uids=33286416&dopt=GenPept&RID=T0C9V6B301S&log$=prottop&blast_rank=30) | Hermansky-Pudlak syndrome 1 protein isoform a [Homo sapiens] | [18.9](http://blast.ncbi.nlm.nih.gov/Blast.cgi#33286416%2333286416) | 18.9 | 85% | 252 |
| [NP_002130.2](http://www.ncbi.nlm.nih.gov/entrez/query.fcgi?cmd=Retrieve&db=Protein&list_uids=56699409&dopt=GenPept&RID=T0C9V6B301S&log$=prottop&blast_rank=31) | RNA binding motif protein, X-linked isoform 1 [Homo sapiens] | [18.9](http://blast.ncbi.nlm.nih.gov/Blast.cgi#56699409%2356699409) | 18.9 | 71% | 252 |
| [NP_060557.3](http://www.ncbi.nlm.nih.gov/entrez/query.fcgi?cmd=Retrieve&db=Protein&list_uids=271398350&dopt=GenPept&RID=T0C9V6B301S&log$=prottop&blast_rank=32) | transmembrane protein 48 isoform 1 [Homo sapiens] | [18.9](http://blast.ncbi.nlm.nih.gov/Blast.cgi#271398350%23271398350) | 18.9 | 71% | 252 |
| [NP_057329.2](http://www.ncbi.nlm.nih.gov/entrez/query.fcgi?cmd=Retrieve&db=Protein&list_uids=142976729&dopt=GenPept&RID=T0C9V6B301S&log$=prottop&blast_rank=33) | estradiol 17-beta-dehydrogenase 11 [Homo sapiens] | [18.5](http://blast.ncbi.nlm.nih.gov/Blast.cgi#142976729%23142976729) | 18.5 | 100% | 338 |
| [NP_065112.1](http://www.ncbi.nlm.nih.gov/entrez/query.fcgi?cmd=Retrieve&db=Protein&list_uids=9966903&dopt=GenPept&RID=T0C9V6B301S&log$=prottop&blast_rank=34) | mannosidase, alpha, class 1C, member 1 [Homo sapiens] | [18.5](http://blast.ncbi.nlm.nih.gov/Blast.cgi#9966903%239966903) | 18.5 | 85% | 338 |
| [NP_001162578.1](http://www.ncbi.nlm.nih.gov/entrez/query.fcgi?cmd=Retrieve&db=Protein&list_uids=281182700&dopt=GenPept&RID=T0C9V6B301S&log$=prottop&blast_rank=35) | vaccinia virus penetration factor isoform 3 [Homo sapiens] | [18.0](http://blast.ncbi.nlm.nih.gov/Blast.cgi#281182700%23281182700) | 62.8 | 100% | 453 |
| [NP_001162577.1](http://www.ncbi.nlm.nih.gov/entrez/query.fcgi?cmd=Retrieve&db=Protein&list_uids=281182694&dopt=GenPept&RID=T0C9V6B301S&log$=prottop&blast_rank=36) | vaccinia virus penetration factor isoform 2 [Homo sapiens] | [18.0](http://blast.ncbi.nlm.nih.gov/Blast.cgi#281182694%23281182694) | 62.8 | 100% | 453 |
| [NP_005867.3](http://www.ncbi.nlm.nih.gov/entrez/query.fcgi?cmd=Retrieve&db=Protein&list_uids=157785645&dopt=GenPept&RID=T0C9V6B301S&log$=prottop&blast_rank=37) | SPEG complex locus [Homo sapiens] | [18.0](http://blast.ncbi.nlm.nih.gov/Blast.cgi#157785645%23157785645) | 18.0 | 85% | 453 |
| [NP_001087199.1](http://www.ncbi.nlm.nih.gov/entrez/query.fcgi?cmd=Retrieve&db=Protein&list_uids=157364926&dopt=GenPept&RID=T0C9V6B301S&log$=prottop&blast_rank=38) | dystrotelin [Homo sapiens] | [18.0](http://blast.ncbi.nlm.nih.gov/Blast.cgi#157364926%23157364926) | 18.0 | 85% | 453 |
| [NP_060702.1](http://www.ncbi.nlm.nih.gov/entrez/query.fcgi?cmd=Retrieve&db=Protein&list_uids=154090959&dopt=GenPept&RID=T0C9V6B301S&log$=prottop&blast_rank=39) | hypothetical protein LOC55747 [Homo sapiens] | [18.0](http://blast.ncbi.nlm.nih.gov/Blast.cgi#154090959%23154090959) | 62.8 | 100% | 453 |
| [NP_001128131.1](http://www.ncbi.nlm.nih.gov/entrez/query.fcgi?cmd=Retrieve&db=Protein&list_uids=197927236&dopt=GenPept&RID=T0C9V6B301S&log$=prottop&blast_rank=40) | proline rich 23A [Homo sapiens] | [18.0](http://blast.ncbi.nlm.nih.gov/Blast.cgi#197927236%23197927236) | 18.0 | 71% | 453 |
| [NP_005112.2](http://www.ncbi.nlm.nih.gov/entrez/query.fcgi?cmd=Retrieve&db=Protein&list_uids=102468717&dopt=GenPept&RID=T0C9V6B301S&log$=prottop&blast_rank=41) | mediator complex subunit 13 [Homo sapiens] | [18.0](http://blast.ncbi.nlm.nih.gov/Blast.cgi#102468717%23102468717) | 18.0 | 85% | 453 |
| [NP_443728.3](http://www.ncbi.nlm.nih.gov/entrez/query.fcgi?cmd=Retrieve&db=Protein&list_uids=93277088&dopt=GenPept&RID=T0C9V6B301S&log$=prottop&blast_rank=42) | mediator of RNA polymerase II transcription, subunit 12 homolog (S. cerevisiae)-like [Homo sapiens] | [18.0](http://blast.ncbi.nlm.nih.gov/Blast.cgi#93277088%2393277088) | 18.0 | 85% | 453 |
| [NP_068746.2](http://www.ncbi.nlm.nih.gov/entrez/query.fcgi?cmd=Retrieve&db=Protein&list_uids=157426893&dopt=GenPept&RID=T0C9V6B301S&log$=prottop&blast_rank=43) | GUF1 GTPase homolog [Homo sapiens] | [18.0](http://blast.ncbi.nlm.nih.gov/Blast.cgi#157426893%23157426893) | 18.0 | 71% | 453 |
| [NP_001005361.1](http://www.ncbi.nlm.nih.gov/entrez/query.fcgi?cmd=Retrieve&db=Protein&list_uids=56549123&dopt=GenPept&RID=T0C9V6B301S&log$=prottop&blast_rank=44) | dynamin 2 isoform 2 [Homo sapiens] | [18.0](http://blast.ncbi.nlm.nih.gov/Blast.cgi#56549123%2356549123) | 31.4 | 100% | 453 |
| [NP_001005362.1](http://www.ncbi.nlm.nih.gov/entrez/query.fcgi?cmd=Retrieve&db=Protein&list_uids=56549125&dopt=GenPept&RID=T0C9V6B301S&log$=prottop&blast_rank=45) | dynamin 2 isoform 4 [Homo sapiens] | [18.0](http://blast.ncbi.nlm.nih.gov/Blast.cgi#56549125%2356549125) | 31.4 | 100% | 453 |
| [NP_076985.4](http://www.ncbi.nlm.nih.gov/entrez/query.fcgi?cmd=Retrieve&db=Protein&list_uids=109689695&dopt=GenPept&RID=T0C9V6B301S&log$=prottop&blast_rank=46) | transient receptor potential cation channel, subfamily M, member 8 [Homo sapiens] | [18.0](http://blast.ncbi.nlm.nih.gov/Blast.cgi#109689695%23109689695) | 18.0 | 71% | 453 |
| [NP_001005751.1](http://www.ncbi.nlm.nih.gov/entrez/query.fcgi?cmd=Retrieve&db=Protein&list_uids=54124343&dopt=GenPept&RID=T0C9V6B301S&log$=prottop&blast_rank=47) | hypothetical protein LOC387680 [Homo sapiens] | [18.0](http://blast.ncbi.nlm.nih.gov/Blast.cgi#54124343%2354124343) | 62.8 | 100% | 453 |
| [NP_004936.2](http://www.ncbi.nlm.nih.gov/entrez/query.fcgi?cmd=Retrieve&db=Protein&list_uids=56549119&dopt=GenPept&RID=T0C9V6B301S&log$=prottop&blast_rank=48) | dynamin 2 isoform 3 [Homo sapiens] | [18.0](http://blast.ncbi.nlm.nih.gov/Blast.cgi#56549119%2356549119) | 31.4 | 100% | 453 |
| [NP_056077.2](http://www.ncbi.nlm.nih.gov/entrez/query.fcgi?cmd=Retrieve&db=Protein&list_uids=281182690&dopt=GenPept&RID=T0C9V6B301S&log$=prottop&blast_rank=49) | vaccinia virus penetration factor isoform 1 [Homo sapiens] | [18.0](http://blast.ncbi.nlm.nih.gov/Blast.cgi#281182690%23281182690) | 62.8 | 100% | 453 |
| [NP_055220.1](http://www.ncbi.nlm.nih.gov/entrez/query.fcgi?cmd=Retrieve&db=Protein&list_uids=7656948&dopt=GenPept&RID=T0C9V6B301S&log$=prottop&blast_rank=50) | voltage-dependent calcium channel gamma-4 subunit [Homo sapiens] | [18.0](http://blast.ncbi.nlm.nih.gov/Blast.cgi#7656948%237656948) | 18.0 | 71% | 453 |
| [NP_056533.1](http://www.ncbi.nlm.nih.gov/entrez/query.fcgi?cmd=Retrieve&db=Protein&list_uids=11136626&dopt=GenPept&RID=T0C9V6B301S&log$=prottop&blast_rank=51) | NADPH oxidase 3 [Homo sapiens] | [18.0](http://blast.ncbi.nlm.nih.gov/Blast.cgi#11136626%2311136626) | 18.0 | 71% | 453 |
| [NP_001005360.1](http://www.ncbi.nlm.nih.gov/entrez/query.fcgi?cmd=Retrieve&db=Protein&list_uids=56549121&dopt=GenPept&RID=T0C9V6B301S&log$=prottop&blast_rank=52) | dynamin 2 isoform 1 [Homo sapiens] | [18.0](http://blast.ncbi.nlm.nih.gov/Blast.cgi#56549121%2356549121) | 31.4 | 100% | 453 |
| [NP_001977.1](http://www.ncbi.nlm.nih.gov/entrez/query.fcgi?cmd=Retrieve&db=Protein&list_uids=24307883&dopt=GenPept&RID=T0C9V6B301S&log$=prottop&blast_rank=53) | ets variant gene 4 (E1A enhancer binding protein, E1AF) [Homo sapiens] >ref|NP_001073143.1| ets variant gene 4 (E1A enhancer binding protein, E1AF) [Homo sapiens] | [18.0](http://blast.ncbi.nlm.nih.gov/Blast.cgi#24307883%2324307883) | 18.0 | 71% | 453 |
| [NP_116045.2](http://www.ncbi.nlm.nih.gov/entrez/query.fcgi?cmd=Retrieve&db=Protein&list_uids=29029601&dopt=GenPept&RID=T0C9V6B301S&log$=prottop&blast_rank=54) | DEAH (Asp-Glu-Ala-His) box polypeptide 37 [Homo sapiens] | [18.0](http://blast.ncbi.nlm.nih.gov/Blast.cgi#29029601%2329029601) | 18.0 | 71% | 453 |
| [NP_008864.3](http://www.ncbi.nlm.nih.gov/entrez/query.fcgi?cmd=Retrieve&db=Protein&list_uids=110835708&dopt=GenPept&RID=T0C9V6B301S&log$=prottop&blast_rank=55) | solute carrier family 5 (inositol transporters), member 3 [Homo sapiens] | [18.0](http://blast.ncbi.nlm.nih.gov/Blast.cgi#110835708%23110835708) | 18.0 | 85% | 453 |
| [XP_002347048.1](http://www.ncbi.nlm.nih.gov/entrez/query.fcgi?cmd=Retrieve&db=Protein&list_uids=239749462&dopt=GenPept&RID=T0C9V6B301S&log$=prottop&blast_rank=56) | PREDICTED: similar to ubiquitin protein ligase E3 component n-recognin 5 isoform 2 [Homo sapiens] | [17.6](http://blast.ncbi.nlm.nih.gov/Blast.cgi#239749462%23239749462) | 33.1 | 85% | 608 |
| [XP_001125699.1](http://www.ncbi.nlm.nih.gov/entrez/query.fcgi?cmd=Retrieve&db=Protein&list_uids=113420453&dopt=GenPept&RID=T0C9V6B301S&log$=prottop&blast_rank=57) | PREDICTED: similar to ubiquitin protein ligase E3 component n-recognin 5 isoform 1 [Homo sapiens] | [17.6](http://blast.ncbi.nlm.nih.gov/Blast.cgi#113420453%23113420453) | 33.1 | 85% | 608 |
| [NP_061486.2](http://www.ncbi.nlm.nih.gov/entrez/query.fcgi?cmd=Retrieve&db=Protein&list_uids=157419140&dopt=GenPept&RID=T0C9V6B301S&log$=prottop&blast_rank=58) | laminin, gamma 2 isoform b precursor [Homo sapiens] | [17.6](http://blast.ncbi.nlm.nih.gov/Blast.cgi#157419140%23157419140) | 17.6 | 100% | 608 |
| [NP_079408.3](http://www.ncbi.nlm.nih.gov/entrez/query.fcgi?cmd=Retrieve&db=Protein&list_uids=55743151&dopt=GenPept&RID=T0C9V6B301S&log$=prottop&blast_rank=59) | WD repeat domain 19 [Homo sapiens] | [17.6](http://blast.ncbi.nlm.nih.gov/Blast.cgi#55743151%2355743151) | 17.6 | 85% | 608 |
| [NP_005553.2](http://www.ncbi.nlm.nih.gov/entrez/query.fcgi?cmd=Retrieve&db=Protein&list_uids=157419138&dopt=GenPept&RID=T0C9V6B301S&log$=prottop&blast_rank=60) | laminin, gamma 2 isoform a precursor [Homo sapiens] | [17.6](http://blast.ncbi.nlm.nih.gov/Blast.cgi#157419138%23157419138) | 17.6 | 100% | 608 |
| [NP_056986.2](http://www.ncbi.nlm.nih.gov/entrez/query.fcgi?cmd=Retrieve&db=Protein&list_uids=15147337&dopt=GenPept&RID=T0C9V6B301S&log$=prottop&blast_rank=61) | ubiquitin protein ligase E3 component n-recognin 5 [Homo sapiens] | [17.6](http://blast.ncbi.nlm.nih.gov/Blast.cgi#15147337%2315147337) | 33.1 | 85% | 608 |
| [NP_411241.1](http://www.ncbi.nlm.nih.gov/entrez/query.fcgi?cmd=Retrieve&db=Protein&list_uids=16554568&dopt=GenPept&RID=T0C9V6B301S&log$=prottop&blast_rank=62) | cyclin D-type binding-protein 1 isoform 2 [Homo sapiens] | [17.6](http://blast.ncbi.nlm.nih.gov/Blast.cgi#16554568%2316554568) | 17.6 | 85% | 608 |
| [NP_065803.2](http://www.ncbi.nlm.nih.gov/entrez/query.fcgi?cmd=Retrieve&db=Protein&list_uids=93204867&dopt=GenPept&RID=T0C9V6B301S&log$=prottop&blast_rank=63) | G protein-coupled receptor 158 precursor [Homo sapiens] | [17.6](http://blast.ncbi.nlm.nih.gov/Blast.cgi#93204867%2393204867) | 17.6 | 100% | 608 |
| [NP_000454.1](http://www.ncbi.nlm.nih.gov/entrez/query.fcgi?cmd=Retrieve&db=Protein&list_uids=8850236&dopt=GenPept&RID=T0C9V6B301S&log$=prottop&blast_rank=64) | UDP glycosyltransferase 1 family, polypeptide A1 precursor [Homo sapiens] | [17.6](http://blast.ncbi.nlm.nih.gov/Blast.cgi#8850236%238850236) | 17.6 | 100% | 608 |
| [NP_036274.3](http://www.ncbi.nlm.nih.gov/entrez/query.fcgi?cmd=Retrieve&db=Protein&list_uids=227500367&dopt=GenPept&RID=T0C9V6B301S&log$=prottop&blast_rank=65) | cyclin D-type binding-protein 1 isoform 1 [Homo sapiens] | [17.6](http://blast.ncbi.nlm.nih.gov/Blast.cgi#227500367%23227500367) | 17.6 | 85% | 608 |
| [NP_001161288.1](http://www.ncbi.nlm.nih.gov/entrez/query.fcgi?cmd=Retrieve&db=Protein&list_uids=269847465&dopt=GenPept&RID=T0C9V6B301S&log$=prottop&blast_rank=66) | hypothetical protein LOC26074 isoform 2 [Homo sapiens] | [17.2](http://blast.ncbi.nlm.nih.gov/Blast.cgi#269847465%23269847465) | 17.2 | 57% | 816 |
| [NP_001156981.1](http://www.ncbi.nlm.nih.gov/entrez/query.fcgi?cmd=Retrieve&db=Protein&list_uids=254553452&dopt=GenPept&RID=T0C9V6B301S&log$=prottop&blast_rank=67) | DDB1 and CUL4 associated factor 4 isoform 5 [Homo sapiens] | [17.2](http://blast.ncbi.nlm.nih.gov/Blast.cgi#254553452%23254553452) | 17.2 | 57% | 816 |
| [NP_001156980.1](http://www.ncbi.nlm.nih.gov/entrez/query.fcgi?cmd=Retrieve&db=Protein&list_uids=254553450&dopt=GenPept&RID=T0C9V6B301S&log$=prottop&blast_rank=68) | DDB1 and CUL4 associated factor 4 isoform 4 [Homo sapiens] | [17.2](http://blast.ncbi.nlm.nih.gov/Blast.cgi#254553450%23254553450) | 17.2 | 57% | 816 |
| [XP_001716413.2](http://www.ncbi.nlm.nih.gov/entrez/query.fcgi?cmd=Retrieve&db=Protein&list_uids=239756486&dopt=GenPept&RID=T0C9V6B301S&log$=prottop&blast_rank=69) | PREDICTED: similar to GM17625 [Homo sapiens] | [17.2](http://blast.ncbi.nlm.nih.gov/Blast.cgi#239756486%23239756486) | 17.2 | 85% | 816 |
| [XP_002344507.1](http://www.ncbi.nlm.nih.gov/entrez/query.fcgi?cmd=Retrieve&db=Protein&list_uids=239755232&dopt=GenPept&RID=T0C9V6B301S&log$=prottop&blast_rank=70) | PREDICTED: hypothetical protein [Homo sapiens] | [17.2](http://blast.ncbi.nlm.nih.gov/Blast.cgi#239755232%23239755232) | 17.2 | 57% | 816 |
| [XP_002343007.1](http://www.ncbi.nlm.nih.gov/entrez/query.fcgi?cmd=Retrieve&db=Protein&list_uids=239744027&dopt=GenPept&RID=T0C9V6B301S&log$=prottop&blast_rank=71) | PREDICTED: hypothetical protein XP_002343007 [Homo sapiens] >ref|XP_002347159.1| PREDICTED: hypothetical protein [Homo sapiens] | [17.2](http://blast.ncbi.nlm.nih.gov/Blast.cgi#239744027%23239744027) | 17.2 | 57% | 816 |
| [NP_683684.2](http://www.ncbi.nlm.nih.gov/entrez/query.fcgi?cmd=Retrieve&db=Protein&list_uids=222080058&dopt=GenPept&RID=T0C9V6B301S&log$=prottop&blast_rank=72) | Smith-Magenis syndrome chromosome region, candidate 7 isoform 2 [Homo sapiens] | [17.2](http://blast.ncbi.nlm.nih.gov/Blast.cgi#222080058%23222080058) | 17.2 | 57% | 816 |
| [NP_001162118.1](http://www.ncbi.nlm.nih.gov/entrez/query.fcgi?cmd=Retrieve&db=Protein&list_uids=281306725&dopt=GenPept&RID=T0C9V6B301S&log$=prottop&blast_rank=73) | connector enhancer of kinase suppressor of Ras 2 isoform 2 [Homo sapiens] | [17.2](http://blast.ncbi.nlm.nih.gov/Blast.cgi#281306725%23281306725) | 34.4 | 57% | 816 |
| [NP_112598.2](http://www.ncbi.nlm.nih.gov/entrez/query.fcgi?cmd=Retrieve&db=Protein&list_uids=207452735&dopt=GenPept&RID=T0C9V6B301S&log$=prottop&blast_rank=74) | epiplakin 1 [Homo sapiens] | [17.2](http://blast.ncbi.nlm.nih.gov/Blast.cgi#207452735%23207452735) | 40.5 | 85% | 816 |
| [NP_001162120.1](http://www.ncbi.nlm.nih.gov/entrez/query.fcgi?cmd=Retrieve&db=Protein&list_uids=281306729&dopt=GenPept&RID=T0C9V6B301S&log$=prottop&blast_rank=75) | connector enhancer of kinase suppressor of Ras 2 isoform 4 [Homo sapiens] | [17.2](http://blast.ncbi.nlm.nih.gov/Blast.cgi#281306729%23281306729) | 17.2 | 57% | 816 |
| [NP_851938.2](http://www.ncbi.nlm.nih.gov/entrez/query.fcgi?cmd=Retrieve&db=Protein&list_uids=254553448&dopt=GenPept&RID=T0C9V6B301S&log$=prottop&blast_rank=76) | DDB1 and CUL4 associated factor 4 isoform 3 [Homo sapiens] | [17.2](http://blast.ncbi.nlm.nih.gov/Blast.cgi#254553448%23254553448) | 17.2 | 57% | 816 |
| [NP_001129120.1](http://www.ncbi.nlm.nih.gov/entrez/query.fcgi?cmd=Retrieve&db=Protein&list_uids=208431815&dopt=GenPept&RID=T0C9V6B301S&log$=prottop&blast_rank=77) | protein tyrosine phosphatase, receptor type, K isoform a [Homo sapiens] | [17.2](http://blast.ncbi.nlm.nih.gov/Blast.cgi#208431815%23208431815) | 34.4 | 57% | 816 |
| [NP_001073988.2](http://www.ncbi.nlm.nih.gov/entrez/query.fcgi?cmd=Retrieve&db=Protein&list_uids=169658367&dopt=GenPept&RID=T0C9V6B301S&log$=prottop&blast_rank=78) | BAH domain and coiled-coil containing 1 [Homo sapiens] | [17.2](http://blast.ncbi.nlm.nih.gov/Blast.cgi#169658367%23169658367) | 17.2 | 57% | 816 |
| [XP_945670.2](http://www.ncbi.nlm.nih.gov/entrez/query.fcgi?cmd=Retrieve&db=Protein&list_uids=169210665&dopt=GenPept&RID=T0C9V6B301S&log$=prottop&blast_rank=79) | PREDICTED: similar to GM17625 [Homo sapiens] >ref|XP_370934.6| PREDICTED: similar to GM17625 [Homo sapiens] | [17.2](http://blast.ncbi.nlm.nih.gov/Blast.cgi#169210665%23169210665) | 17.2 | 85% | 816 |
| [NP_056342.3](http://www.ncbi.nlm.nih.gov/entrez/query.fcgi?cmd=Retrieve&db=Protein&list_uids=167614488&dopt=GenPept&RID=T0C9V6B301S&log$=prottop&blast_rank=80) | TBC1 domain family, member 10B [Homo sapiens] | [17.2](http://blast.ncbi.nlm.nih.gov/Blast.cgi#167614488%23167614488) | 17.2 | 57% | 816 |
| [NP_001103984.1](http://www.ncbi.nlm.nih.gov/entrez/query.fcgi?cmd=Retrieve&db=Protein&list_uids=160333411&dopt=GenPept&RID=T0C9V6B301S&log$=prottop&blast_rank=81) | early B-cell factor 4 [Homo sapiens] | [17.2](http://blast.ncbi.nlm.nih.gov/Blast.cgi#160333411%23160333411) | 17.2 | 57% | 816 |
| [NP_060272.3](http://www.ncbi.nlm.nih.gov/entrez/query.fcgi?cmd=Retrieve&db=Protein&list_uids=157388904&dopt=GenPept&RID=T0C9V6B301S&log$=prottop&blast_rank=82) | HEAT repeat containing 2 [Homo sapiens] | [17.2](http://blast.ncbi.nlm.nih.gov/Blast.cgi#157388904%23157388904) | 17.2 | 57% | 816 |
| [NP_001096080.1](http://www.ncbi.nlm.nih.gov/entrez/query.fcgi?cmd=Retrieve&db=Protein&list_uids=156616299&dopt=GenPept&RID=T0C9V6B301S&log$=prottop&blast_rank=83) | tubulin, gamma complex associated protein 5 isoform b [Homo sapiens] | [17.2](http://blast.ncbi.nlm.nih.gov/Blast.cgi#156616299%23156616299) | 17.2 | 85% | 816 |
| [NP_060420.2](http://www.ncbi.nlm.nih.gov/entrez/query.fcgi?cmd=Retrieve&db=Protein&list_uids=148664197&dopt=GenPept&RID=T0C9V6B301S&log$=prottop&blast_rank=84) | coiled-coil domain containing 40 [Homo sapiens] | [17.2](http://blast.ncbi.nlm.nih.gov/Blast.cgi#148664197%23148664197) | 17.2 | 57% | 816 |
| [NP_663299.2](http://www.ncbi.nlm.nih.gov/entrez/query.fcgi?cmd=Retrieve&db=Protein&list_uids=222418654&dopt=GenPept&RID=T0C9V6B301S&log$=prottop&blast_rank=85) | zinc finger protein 493 isoform 2 [Homo sapiens] | [17.2](http://blast.ncbi.nlm.nih.gov/Blast.cgi#222418654%23222418654) | 17.2 | 57% | 816 |
| [NP_079465.3](http://www.ncbi.nlm.nih.gov/entrez/query.fcgi?cmd=Retrieve&db=Protein&list_uids=289547690&dopt=GenPept&RID=T0C9V6B301S&log$=prottop&blast_rank=86) | zinc finger protein 430 isoform 1 [Homo sapiens] | [17.2](http://blast.ncbi.nlm.nih.gov/Blast.cgi#289547690%23289547690) | 17.2 | 57% | 816 |
| [NP_056400.3](http://www.ncbi.nlm.nih.gov/entrez/query.fcgi?cmd=Retrieve&db=Protein&list_uids=269847414&dopt=GenPept&RID=T0C9V6B301S&log$=prottop&blast_rank=87) | hypothetical protein LOC26074 isoform 1 [Homo sapiens] | [17.2](http://blast.ncbi.nlm.nih.gov/Blast.cgi#269847414%23269847414) | 33.5 | 71% | 816 |
| [NP_001479.3](http://www.ncbi.nlm.nih.gov/entrez/query.fcgi?cmd=Retrieve&db=Protein&list_uids=68509270&dopt=GenPept&RID=T0C9V6B301S&log$=prottop&blast_rank=88) | transcriptional adaptor 2A isoform a [Homo sapiens] >ref|NP_001159577.1| transcriptional adaptor 2A isoform a [Homo sapiens] | [17.2](http://blast.ncbi.nlm.nih.gov/Blast.cgi#68509270%2368509270) | 17.2 | 57% | 816 |
| [NP_597683.2](http://www.ncbi.nlm.nih.gov/entrez/query.fcgi?cmd=Retrieve&db=Protein&list_uids=68509268&dopt=GenPept&RID=T0C9V6B301S&log$=prottop&blast_rank=89) | transcriptional adaptor 2A isoform b [Homo sapiens] | [17.2](http://blast.ncbi.nlm.nih.gov/Blast.cgi#68509268%2368509268) | 17.2 | 57% | 816 |
| [NP_055858.2](http://www.ncbi.nlm.nih.gov/entrez/query.fcgi?cmd=Retrieve&db=Protein&list_uids=45597177&dopt=GenPept&RID=T0C9V6B301S&log$=prottop&blast_rank=90) | TBC1 domain family, member 9B (with GRAM domain) isoform b [Homo sapiens] | [17.2](http://blast.ncbi.nlm.nih.gov/Blast.cgi#45597177%2345597177) | 17.2 | 57% | 816 |
| [NP_942568.2](http://www.ncbi.nlm.nih.gov/entrez/query.fcgi?cmd=Retrieve&db=Protein&list_uids=45597175&dopt=GenPept&RID=T0C9V6B301S&log$=prottop&blast_rank=91) | TBC1 domain family, member 9B (with GRAM domain) isoform a [Homo sapiens] | [17.2](http://blast.ncbi.nlm.nih.gov/Blast.cgi#45597175%2345597175) | 17.2 | 57% | 816 |
| [NP_001070146.1](http://www.ncbi.nlm.nih.gov/entrez/query.fcgi?cmd=Retrieve&db=Protein&list_uids=115511044&dopt=GenPept&RID=T0C9V6B301S&log$=prottop&blast_rank=92) | zinc finger protein 493 isoform 3 [Homo sapiens] | [17.2](http://blast.ncbi.nlm.nih.gov/Blast.cgi#115511044%23115511044) | 17.2 | 57% | 816 |
| [NP_073150.2](http://www.ncbi.nlm.nih.gov/entrez/query.fcgi?cmd=Retrieve&db=Protein&list_uids=113930703&dopt=GenPept&RID=T0C9V6B301S&log$=prottop&blast_rank=93) | early B-cell factor 2 [Homo sapiens] | [17.2](http://blast.ncbi.nlm.nih.gov/Blast.cgi#113930703%23113930703) | 17.2 | 57% | 816 |
| [NP_573438.2](http://www.ncbi.nlm.nih.gov/entrez/query.fcgi?cmd=Retrieve&db=Protein&list_uids=110735406&dopt=GenPept&RID=T0C9V6B301S&log$=prottop&blast_rank=94) | protein tyrosine phosphatase, receptor type, U isoform 2 precursor [Homo sapiens] | [17.2](http://blast.ncbi.nlm.nih.gov/Blast.cgi#110735406%23110735406) | 17.2 | 57% | 816 |
| [NP_004024.2](http://www.ncbi.nlm.nih.gov/entrez/query.fcgi?cmd=Retrieve&db=Protein&list_uids=71773415&dopt=GenPept&RID=T0C9V6B301S&log$=prottop&blast_rank=95) | annexin VI isoform 2 [Homo sapiens] | [17.2](http://blast.ncbi.nlm.nih.gov/Blast.cgi#71773415%2371773415) | 17.2 | 57% | 816 |
| [NP_060049.2](http://www.ncbi.nlm.nih.gov/entrez/query.fcgi?cmd=Retrieve&db=Protein&list_uids=148539844&dopt=GenPept&RID=T0C9V6B301S&log$=prottop&blast_rank=96) | deleted in malignant brain tumors 1 isoform c precursor [Homo sapiens] | [17.2](http://blast.ncbi.nlm.nih.gov/Blast.cgi#148539844%23148539844) | 17.2 | 57% | 816 |
| [NP_004397.2](http://www.ncbi.nlm.nih.gov/entrez/query.fcgi?cmd=Retrieve&db=Protein&list_uids=148539840&dopt=GenPept&RID=T0C9V6B301S&log$=prottop&blast_rank=97) | deleted in malignant brain tumors 1 isoform a precursor [Homo sapiens] | [17.2](http://blast.ncbi.nlm.nih.gov/Blast.cgi#148539840%23148539840) | 17.2 | 57% | 816 |
| [NP_001621.2](http://www.ncbi.nlm.nih.gov/entrez/query.fcgi?cmd=Retrieve&db=Protein&list_uids=91807122&dopt=GenPept&RID=T0C9V6B301S&log$=prottop&blast_rank=98) | annexin A8L2 [Homo sapiens] | [17.2](http://blast.ncbi.nlm.nih.gov/Blast.cgi#91807122%2391807122) | 17.2 | 57% | 816 |
| [NP_061903.2](http://www.ncbi.nlm.nih.gov/entrez/query.fcgi?cmd=Retrieve&db=Protein&list_uids=67782362&dopt=GenPept&RID=T0C9V6B301S&log$=prottop&blast_rank=99) | DEAH (Asp-Glu-Ala-His) box polypeptide 29 [Homo sapiens] | [17.2](http://blast.ncbi.nlm.nih.gov/Blast.cgi#67782362%2367782362) | 26.7 | 100% | 816 |
| [NP_001092097.1](http://www.ncbi.nlm.nih.gov/entrez/query.fcgi?cmd=Retrieve&db=Protein&list_uids=148833488&dopt=GenPept&RID=T0C9V6B301S&log$=prottop&blast_rank=100) | interferon regulatory factor 5 isoform c [Homo sapiens] >ref|NP_001092098.1| interferon regulatory factor 5 isoform c [Homo sapiens] >ref|NP_001092101.1| interferon regulatory factor 5 isoform c [Homo sapiens] | [17.2](http://blast.ncbi.nlm.nih.gov/Blast.cgi#148833488%23148833488) | 17.2 | 57% | 816 |

| **Accession** | **Proteins with a match to QDIFSSA peptide** | **[Max score](http://blast.ncbi.nlm.nih.gov/Blast.cgi?CMD=Get&ALIGNMENTS=100&ALIGNMENT_VIEW=Pairwise&CDD_SEARCH_STATE=1&DATABASE_SORT=0&DESCRIPTIONS=100&ENTREZ_QUERY=txid9606 %5BORGN%5D&FIRST_QUERY_NUM=0&FORMAT_OBJECT=Alignment&FORMAT_PAGE_TARGET=&FORMAT_TYPE=HTML&GET_SEQUENCE=yes&I_THRESH=&MASK_CHAR=2&MASK_COLOR=1&NEW_DESIGN=on&NEW_VIEW=yes&NUM_OVERVIEW=100&OLD_BLAST=false&PAGE=Proteins&QUERY_INDEX=0&QUERY_NUMBER=0&RESULTS_PAGE_TARGET=&RID=T0CMMW9W01N&SHOW_LINKOUT=yes&SHOW_OVERVIEW=yes&STEP_NUMBER=&WORD_SIZE=2&DISPLAY_SORT=1&HSP_SORT=1" \l "sort_mark)** | **[Total score](http://blast.ncbi.nlm.nih.gov/Blast.cgi?CMD=Get&ALIGNMENTS=100&ALIGNMENT_VIEW=Pairwise&CDD_SEARCH_STATE=1&DATABASE_SORT=0&DESCRIPTIONS=100&ENTREZ_QUERY=txid9606 %5BORGN%5D&FIRST_QUERY_NUM=0&FORMAT_OBJECT=Alignment&FORMAT_PAGE_TARGET=&FORMAT_TYPE=HTML&GET_SEQUENCE=yes&I_THRESH=&MASK_CHAR=2&MASK_COLOR=1&NEW_DESIGN=on&NEW_VIEW=yes&NUM_OVERVIEW=100&OLD_BLAST=false&PAGE=Proteins&QUERY_INDEX=0&QUERY_NUMBER=0&RESULTS_PAGE_TARGET=&RID=T0CMMW9W01N&SHOW_LINKOUT=yes&SHOW_OVERVIEW=yes&STEP_NUMBER=&WORD_SIZE=2&DISPLAY_SORT=2&HSP_SORT=1" \l "sort_mark)** | **[Query coverage](http://blast.ncbi.nlm.nih.gov/Blast.cgi?CMD=Get&ALIGNMENTS=100&ALIGNMENT_VIEW=Pairwise&CDD_SEARCH_STATE=1&DATABASE_SORT=0&DESCRIPTIONS=100&ENTREZ_QUERY=txid9606 %5BORGN%5D&FIRST_QUERY_NUM=0&FORMAT_OBJECT=Alignment&FORMAT_PAGE_TARGET=&FORMAT_TYPE=HTML&GET_SEQUENCE=yes&I_THRESH=&MASK_CHAR=2&MASK_COLOR=1&NEW_DESIGN=on&NEW_VIEW=yes&NUM_OVERVIEW=100&OLD_BLAST=false&PAGE=Proteins&QUERY_INDEX=0&QUERY_NUMBER=0&RESULTS_PAGE_TARGET=&RID=T0CMMW9W01N&SHOW_LINKOUT=yes&SHOW_OVERVIEW=yes&STEP_NUMBER=&WORD_SIZE=2&DISPLAY_SORT=4&HSP_SORT=0" \l "sort_mark)** | **[E value](http://blast.ncbi.nlm.nih.gov/Blast.cgi?CMD=Get&ALIGNMENTS=100&ALIGNMENT_VIEW=Pairwise&CDD_SEARCH_STATE=1&DATABASE_SORT=0&DESCRIPTIONS=100&ENTREZ_QUERY=txid9606 %5BORGN%5D&FIRST_QUERY_NUM=0&FORMAT_OBJECT=Alignment&FORMAT_PAGE_TARGET=&FORMAT_TYPE=HTML&GET_SEQUENCE=yes&I_THRESH=&MASK_CHAR=2&MASK_COLOR=1&NEW_DESIGN=on&NEW_VIEW=yes&NUM_OVERVIEW=100&OLD_BLAST=false&PAGE=Proteins&QUERY_INDEX=0&QUERY_NUMBER=0&RESULTS_PAGE_TARGET=&RID=T0CMMW9W01N&SHOW_LINKOUT=yes&SHOW_OVERVIEW=yes&STEP_NUMBER=&WORD_SIZE=2&DISPLAY_SORT=0&HSP_SORT=0" \l "sort_mark)** |
| --- | --- | --- | --- | --- | --- |
| [NP_872358.3](http://www.ncbi.nlm.nih.gov/entrez/query.fcgi?cmd=Retrieve&db=Protein&list_uids=134133292&dopt=GenPept&RID=T0CMMW9W01N&log$=prottop&blast_rank=1) | WD repeat domain 27 [Homo sapiens] | [22.3](http://blast.ncbi.nlm.nih.gov/Blast.cgi#134133292%23134133292) | 22.3 | 85% | 24 |
| [NP_061486.2](http://www.ncbi.nlm.nih.gov/entrez/query.fcgi?cmd=Retrieve&db=Protein&list_uids=157419140&dopt=GenPept&RID=T0CMMW9W01N&log$=prottop&blast_rank=2) | laminin, gamma 2 isoform b precursor [Homo sapiens] | [22.3](http://blast.ncbi.nlm.nih.gov/Blast.cgi#157419140%23157419140) | 22.3 | 100% | 24 |
| [NP_005553.2](http://www.ncbi.nlm.nih.gov/entrez/query.fcgi?cmd=Retrieve&db=Protein&list_uids=157419138&dopt=GenPept&RID=T0CMMW9W01N&log$=prottop&blast_rank=3) | laminin, gamma 2 isoform a precursor [Homo sapiens] | [22.3](http://blast.ncbi.nlm.nih.gov/Blast.cgi#157419138%23157419138) | 22.3 | 100% | 24 |
| [NP_705873.2](http://www.ncbi.nlm.nih.gov/entrez/query.fcgi?cmd=Retrieve&db=Protein&list_uids=84697022&dopt=GenPept&RID=T0CMMW9W01N&log$=prottop&blast_rank=4) | homeobox A1 isoform b [Homo sapiens] | [21.4](http://blast.ncbi.nlm.nih.gov/Blast.cgi#84697022%2384697022) | 21.4 | 85% | 43 |
| [NP_001161939.1](http://www.ncbi.nlm.nih.gov/entrez/query.fcgi?cmd=Retrieve&db=Protein&list_uids=270483747&dopt=GenPept&RID=T0CMMW9W01N&log$=prottop&blast_rank=5) | MAP7 domain containing 2 isoform 4 [Homo sapiens] | [19.7](http://blast.ncbi.nlm.nih.gov/Blast.cgi#270483747%23270483747) | 19.7 | 71% | 140 |
| [NP_001161937.1](http://www.ncbi.nlm.nih.gov/entrez/query.fcgi?cmd=Retrieve&db=Protein&list_uids=270483742&dopt=GenPept&RID=T0CMMW9W01N&log$=prottop&blast_rank=6) | MAP7 domain containing 2 isoform 1 [Homo sapiens] | [19.7](http://blast.ncbi.nlm.nih.gov/Blast.cgi#270483742%23270483742) | 19.7 | 71% | 140 |
| [NP_001161938.1](http://www.ncbi.nlm.nih.gov/entrez/query.fcgi?cmd=Retrieve&db=Protein&list_uids=270483745&dopt=GenPept&RID=T0CMMW9W01N&log$=prottop&blast_rank=7) | MAP7 domain containing 2 isoform 3 [Homo sapiens] | [19.7](http://blast.ncbi.nlm.nih.gov/Blast.cgi#270483745%23270483745) | 19.7 | 71% | 140 |
| [NP_689993.2](http://www.ncbi.nlm.nih.gov/entrez/query.fcgi?cmd=Retrieve&db=Protein&list_uids=270483740&dopt=GenPept&RID=T0CMMW9W01N&log$=prottop&blast_rank=8) | MAP7 domain containing 2 isoform 2 [Homo sapiens] | [19.7](http://blast.ncbi.nlm.nih.gov/Blast.cgi#270483740%23270483740) | 19.7 | 71% | 140 |
| [NP_037450.2](http://www.ncbi.nlm.nih.gov/entrez/query.fcgi?cmd=Retrieve&db=Protein&list_uids=149192855&dopt=GenPept&RID=T0CMMW9W01N&log$=prottop&blast_rank=9) | HLA-B associated transcript 2-like [Homo sapiens] | [19.7](http://blast.ncbi.nlm.nih.gov/Blast.cgi#149192855%23149192855) | 19.7 | 85% | 140 |
| [NP_001131031.1](http://www.ncbi.nlm.nih.gov/entrez/query.fcgi?cmd=Retrieve&db=Protein&list_uids=213385323&dopt=GenPept&RID=T0CMMW9W01N&log$=prottop&blast_rank=10) | anaphase-promoting complex subunit 5 isoform b [Homo sapiens] | [19.7](http://blast.ncbi.nlm.nih.gov/Blast.cgi#213385323%23213385323) | 19.7 | 85% | 140 |
| [NP_001121548.1](http://www.ncbi.nlm.nih.gov/entrez/query.fcgi?cmd=Retrieve&db=Protein&list_uids=189303549&dopt=GenPept&RID=T0CMMW9W01N&log$=prottop&blast_rank=11) | speedy homolog E4 [Homo sapiens] | [19.7](http://blast.ncbi.nlm.nih.gov/Blast.cgi#189303549%23189303549) | 19.7 | 71% | 140 |
| [NP_057407.2](http://www.ncbi.nlm.nih.gov/entrez/query.fcgi?cmd=Retrieve&db=Protein&list_uids=110825982&dopt=GenPept&RID=T0CMMW9W01N&log$=prottop&blast_rank=12) | hect domain and RLD 5 [Homo sapiens] | [19.7](http://blast.ncbi.nlm.nih.gov/Blast.cgi#110825982%23110825982) | 19.7 | 85% | 140 |
| [NP_078930.3](http://www.ncbi.nlm.nih.gov/entrez/query.fcgi?cmd=Retrieve&db=Protein&list_uids=40217805&dopt=GenPept&RID=T0CMMW9W01N&log$=prottop&blast_rank=13) | nucleolar protein 9 [Homo sapiens] | [19.7](http://blast.ncbi.nlm.nih.gov/Blast.cgi#40217805%2340217805) | 19.7 | 71% | 140 |
| [NP_647479.2](http://www.ncbi.nlm.nih.gov/entrez/query.fcgi?cmd=Retrieve&db=Protein&list_uids=22024390&dopt=GenPept&RID=T0CMMW9W01N&log$=prottop&blast_rank=14) | potassium voltage-gated channel, subfamily H, member 5 isoform 1 [Homo sapiens] | [19.7](http://blast.ncbi.nlm.nih.gov/Blast.cgi#22024390%2322024390) | 19.7 | 71% | 140 |
| [NP_064505.1](http://www.ncbi.nlm.nih.gov/entrez/query.fcgi?cmd=Retrieve&db=Protein&list_uids=9910280&dopt=GenPept&RID=T0CMMW9W01N&log$=prottop&blast_rank=15) | UDP-glucose ceramide glucosyltransferase-like 1 precursor [Homo sapiens] | [19.7](http://blast.ncbi.nlm.nih.gov/Blast.cgi#9910280%239910280) | 19.7 | 71% | 140 |
| [NP_073588.1](http://www.ncbi.nlm.nih.gov/entrez/query.fcgi?cmd=Retrieve&db=Protein&list_uids=12232415&dopt=GenPept&RID=T0CMMW9W01N&log$=prottop&blast_rank=16) | family with sequence similarity 59, member A [Homo sapiens] | [19.7](http://blast.ncbi.nlm.nih.gov/Blast.cgi#12232415%2312232415) | 19.7 | 71% | 140 |
| [NP_057321.2](http://www.ncbi.nlm.nih.gov/entrez/query.fcgi?cmd=Retrieve&db=Protein&list_uids=20127553&dopt=GenPept&RID=T0CMMW9W01N&log$=prottop&blast_rank=17) | anaphase-promoting complex subunit 5 isoform a [Homo sapiens] | [19.7](http://blast.ncbi.nlm.nih.gov/Blast.cgi#20127553%2320127553) | 19.7 | 85% | 140 |
| [NP_001135775.1](http://www.ncbi.nlm.nih.gov/entrez/query.fcgi?cmd=Retrieve&db=Protein&list_uids=214831008&dopt=GenPept&RID=T0CMMW9W01N&log$=prottop&blast_rank=18) | membrane-spanning 4-domains, subfamily A, member 2 isoform 2 [Homo sapiens] | [18.9](http://blast.ncbi.nlm.nih.gov/Blast.cgi#214831008%23214831008) | 18.9 | 71% | 252 |
| [NP_003820.2](http://www.ncbi.nlm.nih.gov/entrez/query.fcgi?cmd=Retrieve&db=Protein&list_uids=148746189&dopt=GenPept&RID=T0CMMW9W01N&log$=prottop&blast_rank=19) | multiple PDZ domain protein [Homo sapiens] | [18.9](http://blast.ncbi.nlm.nih.gov/Blast.cgi#148746189%23148746189) | 18.9 | 71% | 252 |
| [NP_057329.2](http://www.ncbi.nlm.nih.gov/entrez/query.fcgi?cmd=Retrieve&db=Protein&list_uids=142976729&dopt=GenPept&RID=T0CMMW9W01N&log$=prottop&blast_rank=20) | estradiol 17-beta-dehydrogenase 11 [Homo sapiens] | [18.9](http://blast.ncbi.nlm.nih.gov/Blast.cgi#142976729%23142976729) | 18.9 | 100% | 252 |
| [NP_060702.1](http://www.ncbi.nlm.nih.gov/entrez/query.fcgi?cmd=Retrieve&db=Protein&list_uids=154090959&dopt=GenPept&RID=T0CMMW9W01N&log$=prottop&blast_rank=21) | hypothetical protein LOC55747 [Homo sapiens] | [18.9](http://blast.ncbi.nlm.nih.gov/Blast.cgi#154090959%23154090959) | 70.0 | 100% | 252 |
| [NP_001007594.1](http://www.ncbi.nlm.nih.gov/entrez/query.fcgi?cmd=Retrieve&db=Protein&list_uids=56117842&dopt=GenPept&RID=T0CMMW9W01N&log$=prottop&blast_rank=22) | sphingomyelin phosphodiesterase 1, acid lysosomal isoform 2 precursor [Homo sapiens] | [18.9](http://blast.ncbi.nlm.nih.gov/Blast.cgi#56117842%2356117842) | 18.9 | 71% | 252 |
| [NP_000534.3](http://www.ncbi.nlm.nih.gov/entrez/query.fcgi?cmd=Retrieve&db=Protein&list_uids=56117840&dopt=GenPept&RID=T0CMMW9W01N&log$=prottop&blast_rank=23) | sphingomyelin phosphodiesterase 1, acid lysosomal isoform 1 precursor [Homo sapiens] | [18.9](http://blast.ncbi.nlm.nih.gov/Blast.cgi#56117840%2356117840) | 18.9 | 71% | 252 |
| [NP_055144.3](http://www.ncbi.nlm.nih.gov/entrez/query.fcgi?cmd=Retrieve&db=Protein&list_uids=45827771&dopt=GenPept&RID=T0CMMW9W01N&log$=prottop&blast_rank=24) | autoantigen RCD8 [Homo sapiens] | [18.9](http://blast.ncbi.nlm.nih.gov/Blast.cgi#45827771%2345827771) | 18.9 | 71% | 252 |
| [NP_000410.2](http://www.ncbi.nlm.nih.gov/entrez/query.fcgi?cmd=Retrieve&db=Protein&list_uids=88758615&dopt=GenPept&RID=T0CMMW9W01N&log$=prottop&blast_rank=25) | integrin alpha 2b preproprotein [Homo sapiens] | [18.9](http://blast.ncbi.nlm.nih.gov/Blast.cgi#88758615%2388758615) | 18.9 | 71% | 252 |
| [NP_001005751.1](http://www.ncbi.nlm.nih.gov/entrez/query.fcgi?cmd=Retrieve&db=Protein&list_uids=54124343&dopt=GenPept&RID=T0CMMW9W01N&log$=prottop&blast_rank=26) | hypothetical protein LOC387680 [Homo sapiens] | [18.9](http://blast.ncbi.nlm.nih.gov/Blast.cgi#54124343%2354124343) | 70.0 | 100% | 252 |
| [NP_005192.1](http://www.ncbi.nlm.nih.gov/entrez/query.fcgi?cmd=Retrieve&db=Protein&list_uids=4885121&dopt=GenPept&RID=T0CMMW9W01N&log$=prottop&blast_rank=27) | chemokine (C-C motif) receptor 8 [Homo sapiens] | [18.9](http://blast.ncbi.nlm.nih.gov/Blast.cgi#4885121%234885121) | 18.9 | 71% | 252 |
| [NP_000130.1](http://www.ncbi.nlm.nih.gov/entrez/query.fcgi?cmd=Retrieve&db=Protein&list_uids=4503677&dopt=GenPept&RID=T0CMMW9W01N&log$=prottop&blast_rank=28) | membrane-spanning 4-domains, subfamily A, member 2 isoform 1 [Homo sapiens] | [18.9](http://blast.ncbi.nlm.nih.gov/Blast.cgi#4503677%234503677) | 18.9 | 71% | 252 |
| [NP_055927.2](http://www.ncbi.nlm.nih.gov/entrez/query.fcgi?cmd=Retrieve&db=Protein&list_uids=112363080&dopt=GenPept&RID=T0CMMW9W01N&log$=prottop&blast_rank=29) | microtubule associated serine/threonine kinase 2 [Homo sapiens] | [18.9](http://blast.ncbi.nlm.nih.gov/Blast.cgi#112363080%23112363080) | 18.9 | 71% | 252 |
| [NP_570115.1](http://www.ncbi.nlm.nih.gov/entrez/query.fcgi?cmd=Retrieve&db=Protein&list_uids=18594498&dopt=GenPept&RID=T0CMMW9W01N&log$=prottop&blast_rank=30) | GTPase, IMAP family member 1 [Homo sapiens] | [18.9](http://blast.ncbi.nlm.nih.gov/Blast.cgi#18594498%2318594498) | 18.9 | 71% | 252 |
| [NP_055753.3](http://www.ncbi.nlm.nih.gov/entrez/query.fcgi?cmd=Retrieve&db=Protein&list_uids=111955326&dopt=GenPept&RID=T0CMMW9W01N&log$=prottop&blast_rank=31) | MLX interacting protein [Homo sapiens] | [18.5](http://blast.ncbi.nlm.nih.gov/Blast.cgi#111955326%23111955326) | 18.5 | 85% | 338 |
| [NP_067032.3](http://www.ncbi.nlm.nih.gov/entrez/query.fcgi?cmd=Retrieve&db=Protein&list_uids=40788015&dopt=GenPept&RID=T0CMMW9W01N&log$=prottop&blast_rank=32) | caspase recruitment domain protein 12 [Homo sapiens] | [18.5](http://blast.ncbi.nlm.nih.gov/Blast.cgi#40788015%2340788015) | 18.5 | 100% | 338 |
| [NP_001128653.1](http://www.ncbi.nlm.nih.gov/entrez/query.fcgi?cmd=Retrieve&db=Protein&list_uids=206597487&dopt=GenPept&RID=T0CMMW9W01N&log$=prottop&blast_rank=33) | solute carrier family 5 (sodium/glucose cotransporter), member 9 isoform 1 [Homo sapiens] | [18.0](http://blast.ncbi.nlm.nih.gov/Blast.cgi#206597487%23206597487) | 18.0 | 85% | 453 |
| [NP_001139509.1](http://www.ncbi.nlm.nih.gov/entrez/query.fcgi?cmd=Retrieve&db=Protein&list_uids=225690519&dopt=GenPept&RID=T0CMMW9W01N&log$=prottop&blast_rank=34) | solute carrier family 14 (urea transporter), member 1 isoform 1 [Homo sapiens] >ref|NP_001122060.3| solute carrier family 14 (urea transporter), member 1 isoform 1 [Homo sapiens] | [18.0](http://blast.ncbi.nlm.nih.gov/Blast.cgi#225690519%23225690519) | 18.0 | 71% | 453 |
| [NP_001011547.2](http://www.ncbi.nlm.nih.gov/entrez/query.fcgi?cmd=Retrieve&db=Protein&list_uids=206597483&dopt=GenPept&RID=T0CMMW9W01N&log$=prottop&blast_rank=35) | solute carrier family 5 (sodium/glucose cotransporter), member 9 isoform 2 [Homo sapiens] | [18.0](http://blast.ncbi.nlm.nih.gov/Blast.cgi#206597483%23206597483) | 18.0 | 85% | 453 |
| [NP_114157.3](http://www.ncbi.nlm.nih.gov/entrez/query.fcgi?cmd=Retrieve&db=Protein&list_uids=221219020&dopt=GenPept&RID=T0CMMW9W01N&log$=prottop&blast_rank=36) | NYD-SP11 protein [Homo sapiens] | [18.0](http://blast.ncbi.nlm.nih.gov/Blast.cgi#221219020%23221219020) | 18.0 | 85% | 453 |
| [NP_853530.2](http://www.ncbi.nlm.nih.gov/entrez/query.fcgi?cmd=Retrieve&db=Protein&list_uids=148277064&dopt=GenPept&RID=T0CMMW9W01N&log$=prottop&blast_rank=37) | cut-like homeobox 1 isoform a [Homo sapiens] | [18.0](http://blast.ncbi.nlm.nih.gov/Blast.cgi#148277064%23148277064) | 18.0 | 85% | 453 |
| [NP_078961.3](http://www.ncbi.nlm.nih.gov/entrez/query.fcgi?cmd=Retrieve&db=Protein&list_uids=100816407&dopt=GenPept&RID=T0CMMW9W01N&log$=prottop&blast_rank=38) | Bardet-Biedl syndrome 10 [Homo sapiens] | [18.0](http://blast.ncbi.nlm.nih.gov/Blast.cgi#100816407%23100816407) | 18.0 | 71% | 453 |
| [NP_054790.2](http://www.ncbi.nlm.nih.gov/entrez/query.fcgi?cmd=Retrieve&db=Protein&list_uids=32307128&dopt=GenPept&RID=T0CMMW9W01N&log$=prottop&blast_rank=39) | nuclear receptor coactivator 6 [Homo sapiens] | [18.0](http://blast.ncbi.nlm.nih.gov/Blast.cgi#32307128%2332307128) | 30.1 | 100% | 453 |
| [XP_002342079.1](http://www.ncbi.nlm.nih.gov/entrez/query.fcgi?cmd=Retrieve&db=Protein&list_uids=239741053&dopt=GenPept&RID=T0CMMW9W01N&log$=prottop&blast_rank=40) | PREDICTED: similar to Putative PRAME family member 24 [Homo sapiens] | [18.0](http://blast.ncbi.nlm.nih.gov/Blast.cgi#239741053%23239741053) | 18.0 | 85% | 453 |
| [NP_057217.2](http://www.ncbi.nlm.nih.gov/entrez/query.fcgi?cmd=Retrieve&db=Protein&list_uids=38327533&dopt=GenPept&RID=T0CMMW9W01N&log$=prottop&blast_rank=41) | insulin induced protein 2 [Homo sapiens] | [18.0](http://blast.ncbi.nlm.nih.gov/Blast.cgi#38327533%2338327533) | 18.0 | 71% | 453 |
| [NP_001012277.1](http://www.ncbi.nlm.nih.gov/entrez/query.fcgi?cmd=Retrieve&db=Protein&list_uids=59676593&dopt=GenPept&RID=T0CMMW9W01N&log$=prottop&blast_rank=42) | PRAME family member 7 [Homo sapiens] | [18.0](http://blast.ncbi.nlm.nih.gov/Blast.cgi#59676593%2359676593) | 18.0 | 85% | 453 |
| [NP_056150.1](http://www.ncbi.nlm.nih.gov/entrez/query.fcgi?cmd=Retrieve&db=Protein&list_uids=44771211&dopt=GenPept&RID=T0CMMW9W01N&log$=prottop&blast_rank=43) | mediator complex subunit 13-like [Homo sapiens] | [18.0](http://blast.ncbi.nlm.nih.gov/Blast.cgi#44771211%2344771211) | 18.0 | 71% | 453 |
| [NP_001001663.1](http://www.ncbi.nlm.nih.gov/entrez/query.fcgi?cmd=Retrieve&db=Protein&list_uids=48717249&dopt=GenPept&RID=T0CMMW9W01N&log$=prottop&blast_rank=44) | transmembrane protein 211 [Homo sapiens] | [18.0](http://blast.ncbi.nlm.nih.gov/Blast.cgi#48717249%2348717249) | 18.0 | 71% | 453 |
| [NP_001012276.1](http://www.ncbi.nlm.nih.gov/entrez/query.fcgi?cmd=Retrieve&db=Protein&list_uids=59676587&dopt=GenPept&RID=T0CMMW9W01N&log$=prottop&blast_rank=45) | PRAME family member 8 [Homo sapiens] | [18.0](http://blast.ncbi.nlm.nih.gov/Blast.cgi#59676587%2359676587) | 18.0 | 85% | 453 |
| [NP_006736.1](http://www.ncbi.nlm.nih.gov/entrez/query.fcgi?cmd=Retrieve&db=Protein&list_uids=5803157&dopt=GenPept&RID=T0CMMW9W01N&log$=prottop&blast_rank=46) | sterol-C4-methyl oxidase-like isoform 1 [Homo sapiens] | [18.0](http://blast.ncbi.nlm.nih.gov/Blast.cgi#5803157%235803157) | 18.0 | 71% | 453 |
| [NP_056949.2](http://www.ncbi.nlm.nih.gov/entrez/query.fcgi?cmd=Retrieve&db=Protein&list_uids=190570158&dopt=GenPept&RID=T0CMMW9W01N&log$=prottop&blast_rank=47) | solute carrier family 14 (urea transporter), member 1 isoform 2 [Homo sapiens] >ref|NP_001139508.2| solute carrier family 14 (urea transporter), member 1 isoform 2 [Homo sapiens] | [18.0](http://blast.ncbi.nlm.nih.gov/Blast.cgi#190570158%23190570158) | 18.0 | 71% | 453 |
| [NP_000334.1](http://www.ncbi.nlm.nih.gov/entrez/query.fcgi?cmd=Retrieve&db=Protein&list_uids=4507031&dopt=GenPept&RID=T0CMMW9W01N&log$=prottop&blast_rank=48) | solute carrier family 5, member 1 [Homo sapiens] | [18.0](http://blast.ncbi.nlm.nih.gov/Blast.cgi#4507031%234507031) | 33.5 | 85% | 453 |
| [NP_938151.1](http://www.ncbi.nlm.nih.gov/entrez/query.fcgi?cmd=Retrieve&db=Protein&list_uids=38327531&dopt=GenPept&RID=T0CMMW9W01N&log$=prottop&blast_rank=49) | insulin induced gene 1 isoform 3 [Homo sapiens] | [18.0](http://blast.ncbi.nlm.nih.gov/Blast.cgi#38327531%2338327531) | 18.0 | 71% | 453 |
| [NP_005533.2](http://www.ncbi.nlm.nih.gov/entrez/query.fcgi?cmd=Retrieve&db=Protein&list_uids=28882053&dopt=GenPept&RID=T0CMMW9W01N&log$=prottop&blast_rank=50) | insulin induced gene 1 isoform 1 [Homo sapiens] | [18.0](http://blast.ncbi.nlm.nih.gov/Blast.cgi#28882053%2328882053) | 18.0 | 71% | 453 |
| [NP_001162578.1](http://www.ncbi.nlm.nih.gov/entrez/query.fcgi?cmd=Retrieve&db=Protein&list_uids=281182700&dopt=GenPept&RID=T0CMMW9W01N&log$=prottop&blast_rank=51) | vaccinia virus penetration factor isoform 3 [Homo sapiens] | [17.6](http://blast.ncbi.nlm.nih.gov/Blast.cgi#281182700%23281182700) | 67.5 | 100% | 608 |
| [NP_001162577.1](http://www.ncbi.nlm.nih.gov/entrez/query.fcgi?cmd=Retrieve&db=Protein&list_uids=281182694&dopt=GenPept&RID=T0CMMW9W01N&log$=prottop&blast_rank=52) | vaccinia virus penetration factor isoform 2 [Homo sapiens] | [17.6](http://blast.ncbi.nlm.nih.gov/Blast.cgi#281182694%23281182694) | 67.5 | 100% | 608 |
| [NP_443728.3](http://www.ncbi.nlm.nih.gov/entrez/query.fcgi?cmd=Retrieve&db=Protein&list_uids=93277088&dopt=GenPept&RID=T0CMMW9W01N&log$=prottop&blast_rank=53) | mediator of RNA polymerase II transcription, subunit 12 homolog (S. cerevisiae)-like [Homo sapiens] | [17.6](http://blast.ncbi.nlm.nih.gov/Blast.cgi#93277088%2393277088) | 17.6 | 85% | 608 |
| [NP_056077.2](http://www.ncbi.nlm.nih.gov/entrez/query.fcgi?cmd=Retrieve&db=Protein&list_uids=281182690&dopt=GenPept&RID=T0CMMW9W01N&log$=prottop&blast_rank=54) | vaccinia virus penetration factor isoform 1 [Homo sapiens] | [17.6](http://blast.ncbi.nlm.nih.gov/Blast.cgi#281182690%23281182690) | 67.5 | 100% | 608 |
| [NP_005688.2](http://www.ncbi.nlm.nih.gov/entrez/query.fcgi?cmd=Retrieve&db=Protein&list_uids=5730031&dopt=GenPept&RID=T0CMMW9W01N&log$=prottop&blast_rank=55) | secretory carrier membrane protein 2 [Homo sapiens] | [17.6](http://blast.ncbi.nlm.nih.gov/Blast.cgi#5730031%235730031) | 17.6 | 85% | 608 |
| [NP_068708.1](http://www.ncbi.nlm.nih.gov/entrez/query.fcgi?cmd=Retrieve&db=Protein&list_uids=11761626&dopt=GenPept&RID=T0CMMW9W01N&log$=prottop&blast_rank=56) | suppression of tumorigenicity 7 isoform b [Homo sapiens] | [17.6](http://blast.ncbi.nlm.nih.gov/Blast.cgi#11761626%2311761626) | 17.6 | 85% | 608 |
| [NP_001159609.1](http://www.ncbi.nlm.nih.gov/entrez/query.fcgi?cmd=Retrieve&db=Protein&list_uids=260898770&dopt=GenPept&RID=T0CMMW9W01N&log$=prottop&blast_rank=57) | hypothetical protein LOC645961 [Homo sapiens] | [17.2](http://blast.ncbi.nlm.nih.gov/Blast.cgi#260898770%23260898770) | 17.2 | 71% | 816 |
| [NP_001159436.1](http://www.ncbi.nlm.nih.gov/entrez/query.fcgi?cmd=Retrieve&db=Protein&list_uids=260166635&dopt=GenPept&RID=T0CMMW9W01N&log$=prottop&blast_rank=58) | sodium channel, voltage-gated, type I, alpha isoform 3 [Homo sapiens] | [17.2](http://blast.ncbi.nlm.nih.gov/Blast.cgi#260166635%23260166635) | 17.2 | 57% | 816 |
| [NP_001136336.2](http://www.ncbi.nlm.nih.gov/entrez/query.fcgi?cmd=Retrieve&db=Protein&list_uids=257196142&dopt=GenPept&RID=T0CMMW9W01N&log$=prottop&blast_rank=59) | family with sequence similarity 38, member A [Homo sapiens] | [17.2](http://blast.ncbi.nlm.nih.gov/Blast.cgi#257196142%23257196142) | 28.4 | 85% | 816 |
| [NP_057424.3](http://www.ncbi.nlm.nih.gov/entrez/query.fcgi?cmd=Retrieve&db=Protein&list_uids=256600194&dopt=GenPept&RID=T0CMMW9W01N&log$=prottop&blast_rank=60) | PDZ domain-containing guanine nucleotide exchange factor I isoform 2 [Homo sapiens] | [17.2](http://blast.ncbi.nlm.nih.gov/Blast.cgi#256600194%23256600194) | 17.2 | 57% | 816 |
| [NP_001157859.1](http://www.ncbi.nlm.nih.gov/entrez/query.fcgi?cmd=Retrieve&db=Protein&list_uids=256600198&dopt=GenPept&RID=T0CMMW9W01N&log$=prottop&blast_rank=61) | PDZ domain-containing guanine nucleotide exchange factor I isoform 3 [Homo sapiens] | [17.2](http://blast.ncbi.nlm.nih.gov/Blast.cgi#256600198%23256600198) | 17.2 | 57% | 816 |
| [XP_002345140.1](http://www.ncbi.nlm.nih.gov/entrez/query.fcgi?cmd=Retrieve&db=Protein&list_uids=239757014&dopt=GenPept&RID=T0CMMW9W01N&log$=prottop&blast_rank=62) | PREDICTED: hypothetical protein [Homo sapiens] | [17.2](http://blast.ncbi.nlm.nih.gov/Blast.cgi#239757014%23239757014) | 17.2 | 57% | 816 |
| [XP_002347048.1](http://www.ncbi.nlm.nih.gov/entrez/query.fcgi?cmd=Retrieve&db=Protein&list_uids=239749462&dopt=GenPept&RID=T0CMMW9W01N&log$=prottop&blast_rank=63) | PREDICTED: similar to ubiquitin protein ligase E3 component n-recognin 5 isoform 2 [Homo sapiens] | [17.2](http://blast.ncbi.nlm.nih.gov/Blast.cgi#239749462%23239749462) | 17.2 | 57% | 816 |
| [XP_002346528.1](http://www.ncbi.nlm.nih.gov/entrez/query.fcgi?cmd=Retrieve&db=Protein&list_uids=239748090&dopt=GenPept&RID=T0CMMW9W01N&log$=prottop&blast_rank=64) | PREDICTED: similar to sodium channel, voltage-gated, type V, alpha (long QT syndrome 3) [Homo sapiens] | [17.2](http://blast.ncbi.nlm.nih.gov/Blast.cgi#239748090%23239748090) | 17.2 | 57% | 816 |
| [XP_002343916.1](http://www.ncbi.nlm.nih.gov/entrez/query.fcgi?cmd=Retrieve&db=Protein&list_uids=239747122&dopt=GenPept&RID=T0CMMW9W01N&log$=prottop&blast_rank=65) | PREDICTED: hypothetical protein XP_002343916 [Homo sapiens] | [17.2](http://blast.ncbi.nlm.nih.gov/Blast.cgi#239747122%23239747122) | 17.2 | 57% | 816 |
| [XP_002343788.1](http://www.ncbi.nlm.nih.gov/entrez/query.fcgi?cmd=Retrieve&db=Protein&list_uids=239746558&dopt=GenPept&RID=T0CMMW9W01N&log$=prottop&blast_rank=66) | PREDICTED: hypothetical protein XP_002343788 [Homo sapiens] | [17.2](http://blast.ncbi.nlm.nih.gov/Blast.cgi#239746558%23239746558) | 17.2 | 57% | 816 |
| [XP_001714976.2](http://www.ncbi.nlm.nih.gov/entrez/query.fcgi?cmd=Retrieve&db=Protein&list_uids=239743732&dopt=GenPept&RID=T0CMMW9W01N&log$=prottop&blast_rank=67) | PREDICTED: family with sequence similarity 75, member A4 isoform 1 [Homo sapiens] | [17.2](http://blast.ncbi.nlm.nih.gov/Blast.cgi#239743732%23239743732) | 17.2 | 71% | 816 |
| [NP_001138600.2](http://www.ncbi.nlm.nih.gov/entrez/query.fcgi?cmd=Retrieve&db=Protein&list_uids=237858799&dopt=GenPept&RID=T0CMMW9W01N&log$=prottop&blast_rank=68) | adenylate kinase domain containing 1 isoform 1 [Homo sapiens] | [17.2](http://blast.ncbi.nlm.nih.gov/Blast.cgi#237858799%23237858799) | 31.8 | 100% | 816 |
| [NP_001153633.1](http://www.ncbi.nlm.nih.gov/entrez/query.fcgi?cmd=Retrieve&db=Protein&list_uids=237512982&dopt=GenPept&RID=T0CMMW9W01N&log$=prottop&blast_rank=69) | voltage-gated sodium channel type V alpha isoform f [Homo sapiens] | [17.2](http://blast.ncbi.nlm.nih.gov/Blast.cgi#237512982%23237512982) | 17.2 | 57% | 816 |
| [NP_001153632.1](http://www.ncbi.nlm.nih.gov/entrez/query.fcgi?cmd=Retrieve&db=Protein&list_uids=237512980&dopt=GenPept&RID=T0CMMW9W01N&log$=prottop&blast_rank=70) | voltage-gated sodium channel type V alpha isoform e [Homo sapiens] | [17.2](http://blast.ncbi.nlm.nih.gov/Blast.cgi#237512980%23237512980) | 17.2 | 57% | 816 |
| [NP_001138596.1](http://www.ncbi.nlm.nih.gov/entrez/query.fcgi?cmd=Retrieve&db=Protein&list_uids=223468617&dopt=GenPept&RID=T0CMMW9W01N&log$=prottop&blast_rank=71) | hypothetical protein LOC441452 [Homo sapiens] | [17.2](http://blast.ncbi.nlm.nih.gov/Blast.cgi#223468617%23223468617) | 17.2 | 71% | 816 |
| [NP_001139405.1](http://www.ncbi.nlm.nih.gov/entrez/query.fcgi?cmd=Retrieve&db=Protein&list_uids=225637461&dopt=GenPept&RID=T0CMMW9W01N&log$=prottop&blast_rank=72) | transketolase-like 1 isoform b [Homo sapiens] | [17.2](http://blast.ncbi.nlm.nih.gov/Blast.cgi#225637461%23225637461) | 17.2 | 57% | 816 |
| [NP_001157858.1](http://www.ncbi.nlm.nih.gov/entrez/query.fcgi?cmd=Retrieve&db=Protein&list_uids=256600196&dopt=GenPept&RID=T0CMMW9W01N&log$=prottop&blast_rank=73) | PDZ domain-containing guanine nucleotide exchange factor I isoform 1 [Homo sapiens] | [17.2](http://blast.ncbi.nlm.nih.gov/Blast.cgi#256600196%23256600196) | 17.2 | 57% | 816 |
| [NP_002757.2](http://www.ncbi.nlm.nih.gov/entrez/query.fcgi?cmd=Retrieve&db=Protein&list_uids=194018537&dopt=GenPept&RID=T0CMMW9W01N&log$=prottop&blast_rank=74) | phosphoribosyl pyrophosphate synthetase-associated protein 1 [Homo sapiens] | [17.2](http://blast.ncbi.nlm.nih.gov/Blast.cgi#194018537%23194018537) | 17.2 | 57% | 816 |
| [XP_001716760.1](http://www.ncbi.nlm.nih.gov/entrez/query.fcgi?cmd=Retrieve&db=Protein&list_uids=169214611&dopt=GenPept&RID=T0CMMW9W01N&log$=prottop&blast_rank=75) | PREDICTED: similar to hCG1790590 [Homo sapiens] >ref|XP_001717265.1| PREDICTED: similar to hCG1790590 [Homo sapiens] >ref|XP_001717070.1| PREDICTED: hypothetical protein [Homo sapiens] | [17.2](http://blast.ncbi.nlm.nih.gov/Blast.cgi#169214611%23169214611) | 17.2 | 57% | 816 |
| [XP_001714979.1](http://www.ncbi.nlm.nih.gov/entrez/query.fcgi?cmd=Retrieve&db=Protein&list_uids=169174618&dopt=GenPept&RID=T0CMMW9W01N&log$=prottop&blast_rank=76) | PREDICTED: family with sequence similarity 75, member A4 isoform 2 [Homo sapiens] | [17.2](http://blast.ncbi.nlm.nih.gov/Blast.cgi#169174618%23169174618) | 17.2 | 71% | 816 |
| [XP_370557.2](http://www.ncbi.nlm.nih.gov/entrez/query.fcgi?cmd=Retrieve&db=Protein&list_uids=169190542&dopt=GenPept&RID=T0CMMW9W01N&log$=prottop&blast_rank=77) | PREDICTED: hypothetical protein [Homo sapiens] | [17.2](http://blast.ncbi.nlm.nih.gov/Blast.cgi#169190542%23169190542) | 49.8 | 71% | 816 |
| [NP_001107590.1](http://www.ncbi.nlm.nih.gov/entrez/query.fcgi?cmd=Retrieve&db=Protein&list_uids=166295179&dopt=GenPept&RID=T0CMMW9W01N&log$=prottop&blast_rank=78) | ELG protein isoform a [Homo sapiens] | [17.2](http://blast.ncbi.nlm.nih.gov/Blast.cgi#166295179%23166295179) | 17.2 | 71% | 816 |
| [NP_079461.2](http://www.ncbi.nlm.nih.gov/entrez/query.fcgi?cmd=Retrieve&db=Protein&list_uids=157739945&dopt=GenPept&RID=T0CMMW9W01N&log$=prottop&blast_rank=79) | tetratricopeptide repeat, ankyrin repeat and coiled-coil containing 2 [Homo sapiens] | [17.2](http://blast.ncbi.nlm.nih.gov/Blast.cgi#157739945%23157739945) | 17.2 | 57% | 816 |
| [NP_005916.2](http://www.ncbi.nlm.nih.gov/entrez/query.fcgi?cmd=Retrieve&db=Protein&list_uids=153070264&dopt=GenPept&RID=T0CMMW9W01N&log$=prottop&blast_rank=80) | meprin A beta precursor [Homo sapiens] | [17.2](http://blast.ncbi.nlm.nih.gov/Blast.cgi#153070264%23153070264) | 17.2 | 57% | 816 |
| [NP_001092875.1](http://www.ncbi.nlm.nih.gov/entrez/query.fcgi?cmd=Retrieve&db=Protein&list_uids=150417969&dopt=GenPept&RID=T0CMMW9W01N&log$=prottop&blast_rank=81) | voltage-gated sodium channel type V alpha isoform d [Homo sapiens] | [17.2](http://blast.ncbi.nlm.nih.gov/Blast.cgi#150417969%23150417969) | 17.2 | 57% | 816 |
| [NP_001092874.1](http://www.ncbi.nlm.nih.gov/entrez/query.fcgi?cmd=Retrieve&db=Protein&list_uids=150417967&dopt=GenPept&RID=T0CMMW9W01N&log$=prottop&blast_rank=82) | voltage-gated sodium channel type V alpha isoform c [Homo sapiens] | [17.2](http://blast.ncbi.nlm.nih.gov/Blast.cgi#150417967%23150417967) | 17.2 | 57% | 816 |
| [NP_001138668.1](http://www.ncbi.nlm.nih.gov/entrez/query.fcgi?cmd=Retrieve&db=Protein&list_uids=223633941&dopt=GenPept&RID=T0CMMW9W01N&log$=prottop&blast_rank=83) | hypothetical protein LOC389730 [Homo sapiens] | [17.2](http://blast.ncbi.nlm.nih.gov/Blast.cgi#223633941%23223633941) | 17.2 | 71% | 816 |
| [NP_001157860.1](http://www.ncbi.nlm.nih.gov/entrez/query.fcgi?cmd=Retrieve&db=Protein&list_uids=256600200&dopt=GenPept&RID=T0CMMW9W01N&log$=prottop&blast_rank=84) | PDZ domain-containing guanine nucleotide exchange factor I isoform 4 [Homo sapiens] | [17.2](http://blast.ncbi.nlm.nih.gov/Blast.cgi#256600200%23256600200) | 17.2 | 57% | 816 |
| [NP_001073860.1](http://www.ncbi.nlm.nih.gov/entrez/query.fcgi?cmd=Retrieve&db=Protein&list_uids=122939208&dopt=GenPept&RID=T0CMMW9W01N&log$=prottop&blast_rank=85) | nuclear antigen Sp100 isoform 1 [Homo sapiens] | [17.2](http://blast.ncbi.nlm.nih.gov/Blast.cgi#122939208%23122939208) | 28.0 | 100% | 816 |
| [NP_660281.2](http://www.ncbi.nlm.nih.gov/entrez/query.fcgi?cmd=Retrieve&db=Protein&list_uids=148596977&dopt=GenPept&RID=T0CMMW9W01N&log$=prottop&blast_rank=86) | zinc finger protein 31 [Homo sapiens] | [17.2](http://blast.ncbi.nlm.nih.gov/Blast.cgi#148596977%23148596977) | 17.2 | 57% | 816 |
| [NP_001154992.1](http://www.ncbi.nlm.nih.gov/entrez/query.fcgi?cmd=Retrieve&db=Protein&list_uids=240849567&dopt=GenPept&RID=T0CMMW9W01N&log$=prottop&blast_rank=87) | component of oligomeric golgi complex 5 isoform 3 [Homo sapiens] | [17.2](http://blast.ncbi.nlm.nih.gov/Blast.cgi#240849567%23240849567) | 17.2 | 57% | 816 |
| [NP_001157861.1](http://www.ncbi.nlm.nih.gov/entrez/query.fcgi?cmd=Retrieve&db=Protein&list_uids=256600202&dopt=GenPept&RID=T0CMMW9W01N&log$=prottop&blast_rank=88) | PDZ domain-containing guanine nucleotide exchange factor I isoform 5 [Homo sapiens] | [17.2](http://blast.ncbi.nlm.nih.gov/Blast.cgi#256600202%23256600202) | 17.2 | 57% | 816 |
| [NP_689525.3](http://www.ncbi.nlm.nih.gov/entrez/query.fcgi?cmd=Retrieve&db=Protein&list_uids=116235482&dopt=GenPept&RID=T0CMMW9W01N&log$=prottop&blast_rank=89) | glycosyltransferase-like 1B [Homo sapiens] | [17.2](http://blast.ncbi.nlm.nih.gov/Blast.cgi#116235482%23116235482) | 17.2 | 57% | 816 |
| [XP_001125699.1](http://www.ncbi.nlm.nih.gov/entrez/query.fcgi?cmd=Retrieve&db=Protein&list_uids=113420453&dopt=GenPept&RID=T0CMMW9W01N&log$=prottop&blast_rank=90) | PREDICTED: similar to ubiquitin protein ligase E3 component n-recognin 5 isoform 1 [Homo sapiens] | [17.2](http://blast.ncbi.nlm.nih.gov/Blast.cgi#113420453%23113420453) | 17.2 | 57% | 816 |
| [NP_006505.2](http://www.ncbi.nlm.nih.gov/entrez/query.fcgi?cmd=Retrieve&db=Protein&list_uids=110835710&dopt=GenPept&RID=T0CMMW9W01N&log$=prottop&blast_rank=91) | sodium channel, voltage-gated, type X, alpha [Homo sapiens] | [17.2](http://blast.ncbi.nlm.nih.gov/Blast.cgi#110835710%23110835710) | 17.2 | 57% | 816 |
| [NP_060899.2](http://www.ncbi.nlm.nih.gov/entrez/query.fcgi?cmd=Retrieve&db=Protein&list_uids=109637791&dopt=GenPept&RID=T0CMMW9W01N&log$=prottop&blast_rank=92) | transcription factor-like nuclear regulator [Homo sapiens] | [17.2](http://blast.ncbi.nlm.nih.gov/Blast.cgi#109637791%23109637791) | 17.2 | 57% | 816 |
| [NP_149115.2](http://www.ncbi.nlm.nih.gov/entrez/query.fcgi?cmd=Retrieve&db=Protein&list_uids=224586875&dopt=GenPept&RID=T0CMMW9W01N&log$=prottop&blast_rank=93) | coiled-coil domain containing 65 [Homo sapiens] | [17.2](http://blast.ncbi.nlm.nih.gov/Blast.cgi#224586875%23224586875) | 17.2 | 57% | 816 |
| [NP_001035154.1](http://www.ncbi.nlm.nih.gov/entrez/query.fcgi?cmd=Retrieve&db=Protein&list_uids=91206406&dopt=GenPept&RID=T0CMMW9W01N&log$=prottop&blast_rank=94) | hypothetical protein LOC642265 [Homo sapiens] | [17.2](http://blast.ncbi.nlm.nih.gov/Blast.cgi#91206406%2391206406) | 17.2 | 71% | 816 |
| [NP_056482.2](http://www.ncbi.nlm.nih.gov/entrez/query.fcgi?cmd=Retrieve&db=Protein&list_uids=153791826&dopt=GenPept&RID=T0CMMW9W01N&log$=prottop&blast_rank=95) | hypothetical protein LOC26165 [Homo sapiens] | [17.2](http://blast.ncbi.nlm.nih.gov/Blast.cgi#153791826%23153791826) | 17.2 | 71% | 816 |
| [NP_001078921.1](http://www.ncbi.nlm.nih.gov/entrez/query.fcgi?cmd=Retrieve&db=Protein&list_uids=146231958&dopt=GenPept&RID=T0CMMW9W01N&log$=prottop&blast_rank=96) | hypothetical protein LOC647060 [Homo sapiens] | [17.2](http://blast.ncbi.nlm.nih.gov/Blast.cgi#146231958%23146231958) | 17.2 | 71% | 816 |
| [NP_001107013.1](http://www.ncbi.nlm.nih.gov/entrez/query.fcgi?cmd=Retrieve&db=Protein&list_uids=165377334&dopt=GenPept&RID=T0CMMW9W01N&log$=prottop&blast_rank=97) | hypothetical protein LOC727905 [Homo sapiens] | [17.2](http://blast.ncbi.nlm.nih.gov/Blast.cgi#165377334%23165377334) | 17.2 | 71% | 816 |
| [NP_001076593.1](http://www.ncbi.nlm.nih.gov/entrez/query.fcgi?cmd=Retrieve&db=Protein&list_uids=133892686&dopt=GenPept&RID=T0CMMW9W01N&log$=prottop&blast_rank=98) | hypothetical protein LOC727830 [Homo sapiens] | [17.2](http://blast.ncbi.nlm.nih.gov/Blast.cgi#133892686%23133892686) | 17.2 | 71% | 816 |
| [NP_056083.3](http://www.ncbi.nlm.nih.gov/entrez/query.fcgi?cmd=Retrieve&db=Protein&list_uids=112421122&dopt=GenPept&RID=T0CMMW9W01N&log$=prottop&blast_rank=99) | DnaJ (Hsp40) homolog, subfamily C, member 13 [Homo sapiens] | [17.2](http://blast.ncbi.nlm.nih.gov/Blast.cgi#112421122%23112421122) | 17.2 | 57% | 816 |
| [NP_001124323.1](http://www.ncbi.nlm.nih.gov/entrez/query.fcgi?cmd=Retrieve&db=Protein&list_uids=195927025&dopt=GenPept&RID=T0CMMW9W01N&log$=prottop&blast_rank=100) | cyclin-dependent kinase inhibitor 3 isoform 2 [Homo sapiens] | [17.2](http://blast.ncbi.nlm.nih.gov/Blast.cgi#195927025%23195927025) | 17.2 | 57% | 816 |

| **Accession** | **Proteins with a match to MSSVMTY peptide** | **[Max score](http://blast.ncbi.nlm.nih.gov/Blast.cgi?CMD=Get&ALIGNMENTS=100&ALIGNMENT_VIEW=Pairwise&CDD_SEARCH_STATE=1&DATABASE_SORT=0&DESCRIPTIONS=100&ENTREZ_QUERY=txid9606 %5BORGN%5D&FIRST_QUERY_NUM=0&FORMAT_OBJECT=Alignment&FORMAT_PAGE_TARGET=&FORMAT_TYPE=HTML&GET_SEQUENCE=yes&I_THRESH=&MASK_CHAR=2&MASK_COLOR=1&NEW_DESIGN=on&NEW_VIEW=yes&NUM_OVERVIEW=100&OLD_BLAST=false&PAGE=Proteins&QUERY_INDEX=0&QUERY_NUMBER=0&RESULTS_PAGE_TARGET=&RID=T0CXSAHZ01N&SHOW_LINKOUT=yes&SHOW_OVERVIEW=yes&STEP_NUMBER=&WORD_SIZE=2&DISPLAY_SORT=1&HSP_SORT=1" \l "sort_mark)** | **[Total score](http://blast.ncbi.nlm.nih.gov/Blast.cgi?CMD=Get&ALIGNMENTS=100&ALIGNMENT_VIEW=Pairwise&CDD_SEARCH_STATE=1&DATABASE_SORT=0&DESCRIPTIONS=100&ENTREZ_QUERY=txid9606 %5BORGN%5D&FIRST_QUERY_NUM=0&FORMAT_OBJECT=Alignment&FORMAT_PAGE_TARGET=&FORMAT_TYPE=HTML&GET_SEQUENCE=yes&I_THRESH=&MASK_CHAR=2&MASK_COLOR=1&NEW_DESIGN=on&NEW_VIEW=yes&NUM_OVERVIEW=100&OLD_BLAST=false&PAGE=Proteins&QUERY_INDEX=0&QUERY_NUMBER=0&RESULTS_PAGE_TARGET=&RID=T0CXSAHZ01N&SHOW_LINKOUT=yes&SHOW_OVERVIEW=yes&STEP_NUMBER=&WORD_SIZE=2&DISPLAY_SORT=2&HSP_SORT=1" \l "sort_mark)** | **[Query coverage](http://blast.ncbi.nlm.nih.gov/Blast.cgi?CMD=Get&ALIGNMENTS=100&ALIGNMENT_VIEW=Pairwise&CDD_SEARCH_STATE=1&DATABASE_SORT=0&DESCRIPTIONS=100&ENTREZ_QUERY=txid9606 %5BORGN%5D&FIRST_QUERY_NUM=0&FORMAT_OBJECT=Alignment&FORMAT_PAGE_TARGET=&FORMAT_TYPE=HTML&GET_SEQUENCE=yes&I_THRESH=&MASK_CHAR=2&MASK_COLOR=1&NEW_DESIGN=on&NEW_VIEW=yes&NUM_OVERVIEW=100&OLD_BLAST=false&PAGE=Proteins&QUERY_INDEX=0&QUERY_NUMBER=0&RESULTS_PAGE_TARGET=&RID=T0CXSAHZ01N&SHOW_LINKOUT=yes&SHOW_OVERVIEW=yes&STEP_NUMBER=&WORD_SIZE=2&DISPLAY_SORT=4&HSP_SORT=0" \l "sort_mark)** | **[E value](http://blast.ncbi.nlm.nih.gov/Blast.cgi?CMD=Get&ALIGNMENTS=100&ALIGNMENT_VIEW=Pairwise&CDD_SEARCH_STATE=1&DATABASE_SORT=0&DESCRIPTIONS=100&ENTREZ_QUERY=txid9606 %5BORGN%5D&FIRST_QUERY_NUM=0&FORMAT_OBJECT=Alignment&FORMAT_PAGE_TARGET=&FORMAT_TYPE=HTML&GET_SEQUENCE=yes&I_THRESH=&MASK_CHAR=2&MASK_COLOR=1&NEW_DESIGN=on&NEW_VIEW=yes&NUM_OVERVIEW=100&OLD_BLAST=false&PAGE=Proteins&QUERY_INDEX=0&QUERY_NUMBER=0&RESULTS_PAGE_TARGET=&RID=T0CXSAHZ01N&SHOW_LINKOUT=yes&SHOW_OVERVIEW=yes&STEP_NUMBER=&WORD_SIZE=2&DISPLAY_SORT=0&HSP_SORT=0" \l "sort_mark)** |
| --- | --- | --- | --- | --- | --- |
| [NP_001123628.1](http://www.ncbi.nlm.nih.gov/entrez/query.fcgi?cmd=Retrieve&db=Protein&list_uids=194328678&dopt=GenPept&RID=T0CXSAHZ01N&log$=prottop&blast_rank=1) | myeloid leukemia factor 1 isoform 2 [Homo sapiens] >ref|NP_001123629.1| myeloid leukemia factor 1 isoform 2 [Homo sapiens] | [23.1](http://blast.ncbi.nlm.nih.gov/Blast.cgi#194328678%23194328678) | 23.1 | 85% | 13 |
| [NP_071888.1](http://www.ncbi.nlm.nih.gov/entrez/query.fcgi?cmd=Retrieve&db=Protein&list_uids=11967975&dopt=GenPept&RID=T0CXSAHZ01N&log$=prottop&blast_rank=2) | myeloid leukemia factor 1 isoform 1 [Homo sapiens] | [23.1](http://blast.ncbi.nlm.nih.gov/Blast.cgi#11967975%2311967975) | 23.1 | 85% | 13 |
| [NP_001157791.1](http://www.ncbi.nlm.nih.gov/entrez/query.fcgi?cmd=Retrieve&db=Protein&list_uids=256222415&dopt=GenPept&RID=T0CXSAHZ01N&log$=prottop&blast_rank=3) | filamin B isoform 4 [Homo sapiens] | [20.6](http://blast.ncbi.nlm.nih.gov/Blast.cgi#256222415%23256222415) | 30.1 | 85% | 78 |
| [NP_001157789.1](http://www.ncbi.nlm.nih.gov/entrez/query.fcgi?cmd=Retrieve&db=Protein&list_uids=256222411&dopt=GenPept&RID=T0CXSAHZ01N&log$=prottop&blast_rank=4) | filamin B isoform 1 [Homo sapiens] | [20.6](http://blast.ncbi.nlm.nih.gov/Blast.cgi#256222411%23256222411) | 30.1 | 85% | 78 |
| [NP_060362.3](http://www.ncbi.nlm.nih.gov/entrez/query.fcgi?cmd=Retrieve&db=Protein&list_uids=151301228&dopt=GenPept&RID=T0CXSAHZ01N&log$=prottop&blast_rank=5) | formin binding protein 3 [Homo sapiens] | [20.6](http://blast.ncbi.nlm.nih.gov/Blast.cgi#151301228%23151301228) | 20.6 | 71% | 78 |
| [NP_001120959.1](http://www.ncbi.nlm.nih.gov/entrez/query.fcgi?cmd=Retrieve&db=Protein&list_uids=188595687&dopt=GenPept&RID=T0CXSAHZ01N&log$=prottop&blast_rank=6) | gamma filamin isoform b [Homo sapiens] | [20.6](http://blast.ncbi.nlm.nih.gov/Blast.cgi#188595687%23188595687) | 31.4 | 100% | 78 |
| [XP_001721353.1](http://www.ncbi.nlm.nih.gov/entrez/query.fcgi?cmd=Retrieve&db=Protein&list_uids=169167518&dopt=GenPept&RID=T0CXSAHZ01N&log$=prottop&blast_rank=7) | PREDICTED: hypothetical protein LOC100127206 [Homo sapiens] >ref|XP_001721185.1| PREDICTED: hypothetical protein LOC100127206 [Homo sapiens] >ref|XP_001722822.1| PREDICTED: hypothetical protein LOC100127206 [Homo sapiens] | [20.6](http://blast.ncbi.nlm.nih.gov/Blast.cgi#169167518%23169167518) | 20.6 | 71% | 78 |
| [NP_001448.2](http://www.ncbi.nlm.nih.gov/entrez/query.fcgi?cmd=Retrieve&db=Protein&list_uids=105990514&dopt=GenPept&RID=T0CXSAHZ01N&log$=prottop&blast_rank=8) | filamin B isoform 2 [Homo sapiens] | [20.6](http://blast.ncbi.nlm.nih.gov/Blast.cgi#105990514%23105990514) | 30.1 | 85% | 78 |
| [NP_001447.2](http://www.ncbi.nlm.nih.gov/entrez/query.fcgi?cmd=Retrieve&db=Protein&list_uids=116063573&dopt=GenPept&RID=T0CXSAHZ01N&log$=prottop&blast_rank=9) | filamin A, alpha isoform 1 [Homo sapiens] | [20.6](http://blast.ncbi.nlm.nih.gov/Blast.cgi#116063573%23116063573) | 20.6 | 71% | 78 |
| [NP_001449.3](http://www.ncbi.nlm.nih.gov/entrez/query.fcgi?cmd=Retrieve&db=Protein&list_uids=116805322&dopt=GenPept&RID=T0CXSAHZ01N&log$=prottop&blast_rank=10) | gamma filamin isoform a [Homo sapiens] | [20.6](http://blast.ncbi.nlm.nih.gov/Blast.cgi#116805322%23116805322) | 31.4 | 100% | 78 |
| [NP_001157790.1](http://www.ncbi.nlm.nih.gov/entrez/query.fcgi?cmd=Retrieve&db=Protein&list_uids=256222413&dopt=GenPept&RID=T0CXSAHZ01N&log$=prottop&blast_rank=11) | filamin B isoform 3 [Homo sapiens] | [20.6](http://blast.ncbi.nlm.nih.gov/Blast.cgi#256222413%23256222413) | 30.1 | 85% | 78 |
| [NP_001104026.1](http://www.ncbi.nlm.nih.gov/entrez/query.fcgi?cmd=Retrieve&db=Protein&list_uids=160420317&dopt=GenPept&RID=T0CXSAHZ01N&log$=prottop&blast_rank=12) | filamin A, alpha isoform 2 [Homo sapiens] | [20.6](http://blast.ncbi.nlm.nih.gov/Blast.cgi#160420317%23160420317) | 20.6 | 71% | 78 |
| [NP_057318.2](http://www.ncbi.nlm.nih.gov/entrez/query.fcgi?cmd=Retrieve&db=Protein&list_uids=42794756&dopt=GenPept&RID=T0CXSAHZ01N&log$=prottop&blast_rank=13) | acyl-CoA synthetase long-chain family member 5 isoform a [Homo sapiens] | [20.2](http://blast.ncbi.nlm.nih.gov/Blast.cgi#42794756%2342794756) | 20.2 | 100% | 104 |
| [NP_976313.1](http://www.ncbi.nlm.nih.gov/entrez/query.fcgi?cmd=Retrieve&db=Protein&list_uids=42794758&dopt=GenPept&RID=T0CXSAHZ01N&log$=prottop&blast_rank=14) | acyl-CoA synthetase long-chain family member 5 isoform b [Homo sapiens] >ref|NP_976314.1| acyl-CoA synthetase long-chain family member 5 isoform b [Homo sapiens] | [20.2](http://blast.ncbi.nlm.nih.gov/Blast.cgi#42794758%2342794758) | 20.2 | 100% | 104 |
| [NP_203744.1](http://www.ncbi.nlm.nih.gov/entrez/query.fcgi?cmd=Retrieve&db=Protein&list_uids=31324577&dopt=GenPept&RID=T0CXSAHZ01N&log$=prottop&blast_rank=15) | molecule interacting with Rab13 [Homo sapiens] | [19.7](http://blast.ncbi.nlm.nih.gov/Blast.cgi#31324577%2331324577) | 19.7 | 100% | 140 |
| [NP_065831.1](http://www.ncbi.nlm.nih.gov/entrez/query.fcgi?cmd=Retrieve&db=Protein&list_uids=149274653&dopt=GenPept&RID=T0CXSAHZ01N&log$=prottop&blast_rank=16) | patched domain containing 2 [Homo sapiens] | [19.3](http://blast.ncbi.nlm.nih.gov/Blast.cgi#149274653%23149274653) | 29.7 | 85% | 188 |
| [NP_078966.2](http://www.ncbi.nlm.nih.gov/entrez/query.fcgi?cmd=Retrieve&db=Protein&list_uids=83367077&dopt=GenPept&RID=T0CXSAHZ01N&log$=prottop&blast_rank=17) | mucin 16 [Homo sapiens] | [19.3](http://blast.ncbi.nlm.nih.gov/Blast.cgi#83367077%2383367077) | 308 | 85% | 188 |
| [NP_852002.1](http://www.ncbi.nlm.nih.gov/entrez/query.fcgi?cmd=Retrieve&db=Protein&list_uids=31657101&dopt=GenPept&RID=T0CXSAHZ01N&log$=prottop&blast_rank=18) | DDB1 and CUL4 associated factor 11 isoform 2 [Homo sapiens] | [19.3](http://blast.ncbi.nlm.nih.gov/Blast.cgi#31657101%2331657101) | 19.3 | 85% | 188 |
| [NP_055078.1](http://www.ncbi.nlm.nih.gov/entrez/query.fcgi?cmd=Retrieve&db=Protein&list_uids=7657689&dopt=GenPept&RID=T0CXSAHZ01N&log$=prottop&blast_rank=19) | YME1-like 1 isoform 3 [Homo sapiens] | [19.3](http://blast.ncbi.nlm.nih.gov/Blast.cgi#7657689%237657689) | 19.3 | 100% | 188 |
| [NP_060616.1](http://www.ncbi.nlm.nih.gov/entrez/query.fcgi?cmd=Retrieve&db=Protein&list_uids=8922534&dopt=GenPept&RID=T0CXSAHZ01N&log$=prottop&blast_rank=20) | RNA methyltransferase like 1 [Homo sapiens] | [19.3](http://blast.ncbi.nlm.nih.gov/Blast.cgi#8922534%238922534) | 19.3 | 85% | 188 |
| [NP_647473.1](http://www.ncbi.nlm.nih.gov/entrez/query.fcgi?cmd=Retrieve&db=Protein&list_uids=21327685&dopt=GenPept&RID=T0CXSAHZ01N&log$=prottop&blast_rank=21) | YME1-like 1 isoform 1 [Homo sapiens] | [19.3](http://blast.ncbi.nlm.nih.gov/Blast.cgi#21327685%2321327685) | 19.3 | 100% | 188 |
| [NP_036478.2](http://www.ncbi.nlm.nih.gov/entrez/query.fcgi?cmd=Retrieve&db=Protein&list_uids=24497603&dopt=GenPept&RID=T0CXSAHZ01N&log$=prottop&blast_rank=22) | nucleoporin 62kDa [Homo sapiens] >ref|NP_057637.2| nucleoporin 62kDa [Homo sapiens] >ref|NP_714940.1| nucleoporin 62kDa [Homo sapiens] >ref|NP_714941.1| nucleoporin 62kDa [Homo sapiens] | [19.3](http://blast.ncbi.nlm.nih.gov/Blast.cgi#24497603%2324497603) | 19.3 | 85% | 188 |
| [NP_079506.3](http://www.ncbi.nlm.nih.gov/entrez/query.fcgi?cmd=Retrieve&db=Protein&list_uids=31657103&dopt=GenPept&RID=T0CXSAHZ01N&log$=prottop&blast_rank=23) | DDB1 and CUL4 associated factor 11 isoform 1 [Homo sapiens] >ref|NP_001156956.1| DDB1 and CUL4 associated factor 11 isoform 1 [Homo sapiens] | [19.3](http://blast.ncbi.nlm.nih.gov/Blast.cgi#31657103%2331657103) | 19.3 | 85% | 188 |
| [NP_149015.2](http://www.ncbi.nlm.nih.gov/entrez/query.fcgi?cmd=Retrieve&db=Protein&list_uids=150378539&dopt=GenPept&RID=T0CXSAHZ01N&log$=prottop&blast_rank=24) | piccolo isoform 1 [Homo sapiens] | [18.9](http://blast.ncbi.nlm.nih.gov/Blast.cgi#150378539%23150378539) | 31.8 | 71% | 252 |
| [NP_055325.2](http://www.ncbi.nlm.nih.gov/entrez/query.fcgi?cmd=Retrieve&db=Protein&list_uids=150170670&dopt=GenPept&RID=T0CXSAHZ01N&log$=prottop&blast_rank=25) | piccolo isoform 2 [Homo sapiens] | [18.9](http://blast.ncbi.nlm.nih.gov/Blast.cgi#150170670%23150170670) | 31.8 | 71% | 252 |
| [NP_115609.2](http://www.ncbi.nlm.nih.gov/entrez/query.fcgi?cmd=Retrieve&db=Protein&list_uids=40068481&dopt=GenPept&RID=T0CXSAHZ01N&log$=prottop&blast_rank=26) | SET domain containing 3 isoform a [Homo sapiens] | [18.9](http://blast.ncbi.nlm.nih.gov/Blast.cgi#40068481%2340068481) | 18.9 | 71% | 252 |
| [NP_078973.1](http://www.ncbi.nlm.nih.gov/entrez/query.fcgi?cmd=Retrieve&db=Protein&list_uids=13375981&dopt=GenPept&RID=T0CXSAHZ01N&log$=prottop&blast_rank=27) | zinc finger protein 385D [Homo sapiens] | [18.9](http://blast.ncbi.nlm.nih.gov/Blast.cgi#13375981%2313375981) | 18.9 | 71% | 252 |
| [NP_954574.1](http://www.ncbi.nlm.nih.gov/entrez/query.fcgi?cmd=Retrieve&db=Protein&list_uids=40068483&dopt=GenPept&RID=T0CXSAHZ01N&log$=prottop&blast_rank=28) | SET domain containing 3 isoform b [Homo sapiens] | [18.9](http://blast.ncbi.nlm.nih.gov/Blast.cgi#40068483%2340068483) | 18.9 | 71% | 252 |
| [NP_002676.1](http://www.ncbi.nlm.nih.gov/entrez/query.fcgi?cmd=Retrieve&db=Protein&list_uids=4505917&dopt=GenPept&RID=T0CXSAHZ01N&log$=prottop&blast_rank=29) | exosome component 10 isoform 2 [Homo sapiens] | [18.9](http://blast.ncbi.nlm.nih.gov/Blast.cgi#4505917%234505917) | 18.9 | 100% | 252 |
| [NP_006226.2](http://www.ncbi.nlm.nih.gov/entrez/query.fcgi?cmd=Retrieve&db=Protein&list_uids=167900478&dopt=GenPept&RID=T0CXSAHZ01N&log$=prottop&blast_rank=30) | POU class 2 associating factor 1 [Homo sapiens] | [18.9](http://blast.ncbi.nlm.nih.gov/Blast.cgi#167900478%23167900478) | 18.9 | 85% | 252 |
| [NP_001001998.1](http://www.ncbi.nlm.nih.gov/entrez/query.fcgi?cmd=Retrieve&db=Protein&list_uids=50301240&dopt=GenPept&RID=T0CXSAHZ01N&log$=prottop&blast_rank=31) | exosome component 10 isoform 1 [Homo sapiens] | [18.9](http://blast.ncbi.nlm.nih.gov/Blast.cgi#50301240%2350301240) | 18.9 | 100% | 252 |
| [NP_004422.2](http://www.ncbi.nlm.nih.gov/entrez/query.fcgi?cmd=Retrieve&db=Protein&list_uids=32967311&dopt=GenPept&RID=T0CXSAHZ01N&log$=prottop&blast_rank=32) | ephrin receptor EphA2 precursor [Homo sapiens] | [18.9](http://blast.ncbi.nlm.nih.gov/Blast.cgi#32967311%2332967311) | 18.9 | 100% | 252 |
| [NP_073728.1](http://www.ncbi.nlm.nih.gov/entrez/query.fcgi?cmd=Retrieve&db=Protein&list_uids=12707562&dopt=GenPept&RID=T0CXSAHZ01N&log$=prottop&blast_rank=33) | period 2 [Homo sapiens] | [18.9](http://blast.ncbi.nlm.nih.gov/Blast.cgi#12707562%2312707562) | 18.9 | 85% | 252 |
| [NP_057726.3](http://www.ncbi.nlm.nih.gov/entrez/query.fcgi?cmd=Retrieve&db=Protein&list_uids=170016061&dopt=GenPept&RID=T0CXSAHZ01N&log$=prottop&blast_rank=34) | spectrin, beta, non-erythrocytic 5 [Homo sapiens] | [18.5](http://blast.ncbi.nlm.nih.gov/Blast.cgi#170016061%23170016061) | 31.8 | 100% | 338 |
| [NP_892006.2](http://www.ncbi.nlm.nih.gov/entrez/query.fcgi?cmd=Retrieve&db=Protein&list_uids=154277116&dopt=GenPept&RID=T0CXSAHZ01N&log$=prottop&blast_rank=35) | spectrin repeat containing, nuclear envelope 1 isoform 1 [Homo sapiens] | [18.5](http://blast.ncbi.nlm.nih.gov/Blast.cgi#154277116%23154277116) | 18.5 | 71% | 338 |
| [NP_878918.2](http://www.ncbi.nlm.nih.gov/entrez/query.fcgi?cmd=Retrieve&db=Protein&list_uids=118918407&dopt=GenPept&RID=T0CXSAHZ01N&log$=prottop&blast_rank=36) | spectrin repeat containing, nuclear envelope 2 isoform 5 [Homo sapiens] | [18.5](http://blast.ncbi.nlm.nih.gov/Blast.cgi#118918407%23118918407) | 18.5 | 71% | 338 |
| [NP_055995.4](http://www.ncbi.nlm.nih.gov/entrez/query.fcgi?cmd=Retrieve&db=Protein&list_uids=118918403&dopt=GenPept&RID=T0CXSAHZ01N&log$=prottop&blast_rank=37) | spectrin repeat containing, nuclear envelope 2 isoform 1 [Homo sapiens] | [18.5](http://blast.ncbi.nlm.nih.gov/Blast.cgi#118918403%23118918403) | 18.5 | 71% | 338 |
| [NP_776297.2](http://www.ncbi.nlm.nih.gov/entrez/query.fcgi?cmd=Retrieve&db=Protein&list_uids=55770840&dopt=GenPept&RID=T0CXSAHZ01N&log$=prottop&blast_rank=38) | chloride channel 3 isoform e [Homo sapiens] | [18.5](http://blast.ncbi.nlm.nih.gov/Blast.cgi#55770840%2355770840) | 28.8 | 85% | 338 |
| [NP_001820.2](http://www.ncbi.nlm.nih.gov/entrez/query.fcgi?cmd=Retrieve&db=Protein&list_uids=55770838&dopt=GenPept&RID=T0CXSAHZ01N&log$=prottop&blast_rank=39) | chloride channel 3 isoform b [Homo sapiens] | [18.5](http://blast.ncbi.nlm.nih.gov/Blast.cgi#55770838%2355770838) | 28.8 | 85% | 338 |
| [NP_149062.1](http://www.ncbi.nlm.nih.gov/entrez/query.fcgi?cmd=Retrieve&db=Protein&list_uids=23097308&dopt=GenPept&RID=T0CXSAHZ01N&log$=prottop&blast_rank=40) | spectrin repeat containing, nuclear envelope 1 isoform 2 [Homo sapiens] | [18.5](http://blast.ncbi.nlm.nih.gov/Blast.cgi#23097308%2323097308) | 18.5 | 71% | 338 |
| [NP_690874.2](http://www.ncbi.nlm.nih.gov/entrez/query.fcgi?cmd=Retrieve&db=Protein&list_uids=29788760&dopt=GenPept&RID=T0CXSAHZ01N&log$=prottop&blast_rank=41) | diacylglycerol kinase, eta isoform 1 [Homo sapiens] | [18.5](http://blast.ncbi.nlm.nih.gov/Blast.cgi#29788760%2329788760) | 18.5 | 71% | 338 |
| [NP_079010.2](http://www.ncbi.nlm.nih.gov/entrez/query.fcgi?cmd=Retrieve&db=Protein&list_uids=19923599&dopt=GenPept&RID=T0CXSAHZ01N&log$=prottop&blast_rank=42) | calmin [Homo sapiens] | [18.5](http://blast.ncbi.nlm.nih.gov/Blast.cgi#19923599%2319923599) | 18.5 | 71% | 338 |
| [NP_112221.1](http://www.ncbi.nlm.nih.gov/entrez/query.fcgi?cmd=Retrieve&db=Protein&list_uids=13624335&dopt=GenPept&RID=T0CXSAHZ01N&log$=prottop&blast_rank=43) | olfactory receptor, family 12, subfamily D, member 3 [Homo sapiens] | [18.5](http://blast.ncbi.nlm.nih.gov/Blast.cgi#13624335%2313624335) | 18.5 | 85% | 338 |
| [NP_004891.3](http://www.ncbi.nlm.nih.gov/entrez/query.fcgi?cmd=Retrieve&db=Protein&list_uids=22907025&dopt=GenPept&RID=T0CXSAHZ01N&log$=prottop&blast_rank=44) | apolipoprotein B mRNA editing enzyme, catalytic polypeptide-like 3B [Homo sapiens] | [18.5](http://blast.ncbi.nlm.nih.gov/Blast.cgi#22907025%2322907025) | 18.5 | 71% | 338 |
| [NP_058634.3](http://www.ncbi.nlm.nih.gov/entrez/query.fcgi?cmd=Retrieve&db=Protein&list_uids=190343004&dopt=GenPept&RID=T0CXSAHZ01N&log$=prottop&blast_rank=45) | EGF-containing fibulin-like extracellular matrix protein 2 precursor [Homo sapiens] | [18.5](http://blast.ncbi.nlm.nih.gov/Blast.cgi#190343004%23190343004) | 18.5 | 100% | 338 |
| [NP_821077.1](http://www.ncbi.nlm.nih.gov/entrez/query.fcgi?cmd=Retrieve&db=Protein&list_uids=29788751&dopt=GenPept&RID=T0CXSAHZ01N&log$=prottop&blast_rank=46) | diacylglycerol kinase, eta isoform 2 [Homo sapiens] | [18.5](http://blast.ncbi.nlm.nih.gov/Blast.cgi#29788751%2329788751) | 18.5 | 71% | 338 |
| [NP_001267.2](http://www.ncbi.nlm.nih.gov/entrez/query.fcgi?cmd=Retrieve&db=Protein&list_uids=144226251&dopt=GenPept&RID=T0CXSAHZ01N&log$=prottop&blast_rank=47) | chitinase 3-like 1 precursor [Homo sapiens] | [18.5](http://blast.ncbi.nlm.nih.gov/Blast.cgi#144226251%23144226251) | 18.5 | 71% | 338 |
| [NP_068594.1](http://www.ncbi.nlm.nih.gov/entrez/query.fcgi?cmd=Retrieve&db=Protein&list_uids=13399304&dopt=GenPept&RID=T0CXSAHZ01N&log$=prottop&blast_rank=48) | apolipoprotein B mRNA editing enzyme, catalytic polypeptide-like 3G [Homo sapiens] | [18.5](http://blast.ncbi.nlm.nih.gov/Blast.cgi#13399304%2313399304) | 18.5 | 71% | 338 |
| [NP_663745.1](http://www.ncbi.nlm.nih.gov/entrez/query.fcgi?cmd=Retrieve&db=Protein&list_uids=21955158&dopt=GenPept&RID=T0CXSAHZ01N&log$=prottop&blast_rank=49) | phorbolin 1 [Homo sapiens] | [18.5](http://blast.ncbi.nlm.nih.gov/Blast.cgi#21955158%2321955158) | 18.5 | 71% | 338 |
| [NP_001003700.1](http://www.ncbi.nlm.nih.gov/entrez/query.fcgi?cmd=Retrieve&db=Protein&list_uids=270132935&dopt=GenPept&RID=T0CXSAHZ01N&log$=prottop&blast_rank=50) | ras responsive element binding protein 1 isoform 3 [Homo sapiens] | [18.0](http://blast.ncbi.nlm.nih.gov/Blast.cgi#270132935%23270132935) | 18.0 | 71% | 453 |
| [NP_001156506.1](http://www.ncbi.nlm.nih.gov/entrez/query.fcgi?cmd=Retrieve&db=Protein&list_uids=251823858&dopt=GenPept&RID=T0CXSAHZ01N&log$=prottop&blast_rank=51) | raptor isoform 2 [Homo sapiens] | [18.0](http://blast.ncbi.nlm.nih.gov/Blast.cgi#251823858%23251823858) | 18.0 | 57% | 453 |
| [XP_001726752.2](http://www.ncbi.nlm.nih.gov/entrez/query.fcgi?cmd=Retrieve&db=Protein&list_uids=239753208&dopt=GenPept&RID=T0CXSAHZ01N&log$=prottop&blast_rank=52) | PREDICTED: similar to anaphase promoting complex subunit 1 [Homo sapiens] | [18.0](http://blast.ncbi.nlm.nih.gov/Blast.cgi#239753208%23239753208) | 18.0 | 57% | 453 |
| [XP_002344148.1](http://www.ncbi.nlm.nih.gov/entrez/query.fcgi?cmd=Retrieve&db=Protein&list_uids=239745633&dopt=GenPept&RID=T0CXSAHZ01N&log$=prottop&blast_rank=53) | PREDICTED: hypothetical protein XP_002344148 [Homo sapiens] >ref|XP_002347723.1| PREDICTED: hypothetical protein XP_002347723 [Homo sapiens] >ref|XP_002344969.1| PREDICTED: hypothetical protein XP_002344969 [Homo sapiens] | [18.0](http://blast.ncbi.nlm.nih.gov/Blast.cgi#239745633%23239745633) | 18.0 | 85% | 453 |
| [NP_001135798.1](http://www.ncbi.nlm.nih.gov/entrez/query.fcgi?cmd=Retrieve&db=Protein&list_uids=215599981&dopt=GenPept&RID=T0CXSAHZ01N&log$=prottop&blast_rank=54) | cyclin D binding myb-like transcription factor 1 isoform b [Homo sapiens] | [18.0](http://blast.ncbi.nlm.nih.gov/Blast.cgi#215599981%23215599981) | 18.0 | 71% | 453 |
| [NP_848599.3](http://www.ncbi.nlm.nih.gov/entrez/query.fcgi?cmd=Retrieve&db=Protein&list_uids=194440727&dopt=GenPept&RID=T0CXSAHZ01N&log$=prottop&blast_rank=55) | dynein heavy chain domain 2 isoform 1 [Homo sapiens] | [18.0](http://blast.ncbi.nlm.nih.gov/Blast.cgi#194440727%23194440727) | 29.3 | 100% | 453 |
| [NP_001135774.1](http://www.ncbi.nlm.nih.gov/entrez/query.fcgi?cmd=Retrieve&db=Protein&list_uids=214830928&dopt=GenPept&RID=T0CXSAHZ01N&log$=prottop&blast_rank=56) | coiled-coil domain containing 113 isoform 2 [Homo sapiens] | [18.0](http://blast.ncbi.nlm.nih.gov/Blast.cgi#214830928%23214830928) | 18.0 | 57% | 453 |
| [NP_079255.3](http://www.ncbi.nlm.nih.gov/entrez/query.fcgi?cmd=Retrieve&db=Protein&list_uids=163644323&dopt=GenPept&RID=T0CXSAHZ01N&log$=prottop&blast_rank=57) | MCF.2 cell line derived transforming sequence-like isoform b [Homo sapiens] | [18.0](http://blast.ncbi.nlm.nih.gov/Blast.cgi#163644323%23163644323) | 18.0 | 57% | 453 |
| [NP_997320.2](http://www.ncbi.nlm.nih.gov/entrez/query.fcgi?cmd=Retrieve&db=Protein&list_uids=198442844&dopt=GenPept&RID=T0CXSAHZ01N&log$=prottop&blast_rank=58) | dynein, axonemal, heavy chain 10 [Homo sapiens] | [18.0](http://blast.ncbi.nlm.nih.gov/Blast.cgi#198442844%23198442844) | 18.0 | 71% | 453 |
| [NP_001106203.1](http://www.ncbi.nlm.nih.gov/entrez/query.fcgi?cmd=Retrieve&db=Protein&list_uids=163644325&dopt=GenPept&RID=T0CXSAHZ01N&log$=prottop&blast_rank=59) | MCF.2 cell line derived transforming sequence-like isoform a [Homo sapiens] | [18.0](http://blast.ncbi.nlm.nih.gov/Blast.cgi#163644325%23163644325) | 18.0 | 57% | 453 |
| [NP_001160132.1](http://www.ncbi.nlm.nih.gov/entrez/query.fcgi?cmd=Retrieve&db=Protein&list_uids=262359974&dopt=GenPept&RID=T0CXSAHZ01N&log$=prottop&blast_rank=60) | neuroligin 3 isoform 3 [Homo sapiens] | [18.0](http://blast.ncbi.nlm.nih.gov/Blast.cgi#262359974%23262359974) | 18.0 | 57% | 453 |
| [NP_056067.2](http://www.ncbi.nlm.nih.gov/entrez/query.fcgi?cmd=Retrieve&db=Protein&list_uids=217330568&dopt=GenPept&RID=T0CXSAHZ01N&log$=prottop&blast_rank=61) | EH domain binding protein 1 isoform 1 [Homo sapiens] | [18.0](http://blast.ncbi.nlm.nih.gov/Blast.cgi#217330568%23217330568) | 41.8 | 100% | 453 |
| [NP_001096034.1](http://www.ncbi.nlm.nih.gov/entrez/query.fcgi?cmd=Retrieve&db=Protein&list_uids=156523264&dopt=GenPept&RID=T0CXSAHZ01N&log$=prottop&blast_rank=62) | hypothetical protein LOC112752 isoform 2 [Homo sapiens] | [18.0](http://blast.ncbi.nlm.nih.gov/Blast.cgi#156523264%23156523264) | 18.0 | 57% | 453 |
| [NP_443105.2](http://www.ncbi.nlm.nih.gov/entrez/query.fcgi?cmd=Retrieve&db=Protein&list_uids=156523262&dopt=GenPept&RID=T0CXSAHZ01N&log$=prottop&blast_rank=63) | hypothetical protein LOC112752 isoform 1 [Homo sapiens] | [18.0](http://blast.ncbi.nlm.nih.gov/Blast.cgi#156523262%23156523262) | 18.0 | 57% | 453 |
| [NP_478054.2](http://www.ncbi.nlm.nih.gov/entrez/query.fcgi?cmd=Retrieve&db=Protein&list_uids=115527066&dopt=GenPept&RID=T0CXSAHZ01N&log$=prottop&blast_rank=64) | alpha 2 type VI collagen isoform 2C2a precursor [Homo sapiens] | [18.0](http://blast.ncbi.nlm.nih.gov/Blast.cgi#115527066%23115527066) | 18.0 | 57% | 453 |
| [NP_478055.2](http://www.ncbi.nlm.nih.gov/entrez/query.fcgi?cmd=Retrieve&db=Protein&list_uids=115527070&dopt=GenPept&RID=T0CXSAHZ01N&log$=prottop&blast_rank=65) | alpha 2 type VI collagen isoform 2C2a' precursor [Homo sapiens] | [18.0](http://blast.ncbi.nlm.nih.gov/Blast.cgi#115527070%23115527070) | 18.0 | 57% | 453 |
| [NP_001840.3](http://www.ncbi.nlm.nih.gov/entrez/query.fcgi?cmd=Retrieve&db=Protein&list_uids=115527062&dopt=GenPept&RID=T0CXSAHZ01N&log$=prottop&blast_rank=66) | alpha 2 type VI collagen isoform 2C2 precursor [Homo sapiens] | [18.0](http://blast.ncbi.nlm.nih.gov/Blast.cgi#115527062%23115527062) | 18.0 | 57% | 453 |
| [NP_056127.2](http://www.ncbi.nlm.nih.gov/entrez/query.fcgi?cmd=Retrieve&db=Protein&list_uids=150378498&dopt=GenPept&RID=T0CXSAHZ01N&log$=prottop&blast_rank=67) | fragile site-associated protein [Homo sapiens] | [18.0](http://blast.ncbi.nlm.nih.gov/Blast.cgi#150378498%23150378498) | 30.5 | 100% | 453 |
| [NP_005682.2](http://www.ncbi.nlm.nih.gov/entrez/query.fcgi?cmd=Retrieve&db=Protein&list_uids=110832835&dopt=GenPept&RID=T0CXSAHZ01N&log$=prottop&blast_rank=68) | ATP-binding cassette, sub-family C, member 9 isoform SUR2A [Homo sapiens] | [18.0](http://blast.ncbi.nlm.nih.gov/Blast.cgi#110832835%23110832835) | 31.8 | 71% | 453 |
| [NP_064694.2](http://www.ncbi.nlm.nih.gov/entrez/query.fcgi?cmd=Retrieve&db=Protein&list_uids=110832839&dopt=GenPept&RID=T0CXSAHZ01N&log$=prottop&blast_rank=69) | ATP-binding cassette, sub-family C, member 9 isoform SUR2A-delta-14 [Homo sapiens] | [18.0](http://blast.ncbi.nlm.nih.gov/Blast.cgi#110832839%23110832839) | 31.8 | 71% | 453 |
| [NP_064693.2](http://www.ncbi.nlm.nih.gov/entrez/query.fcgi?cmd=Retrieve&db=Protein&list_uids=110832837&dopt=GenPept&RID=T0CXSAHZ01N&log$=prottop&blast_rank=70) | ATP-binding cassette, sub-family C, member 9 isoform SUR2B [Homo sapiens] | [18.0](http://blast.ncbi.nlm.nih.gov/Blast.cgi#110832837%23110832837) | 18.0 | 71% | 453 |
| [NP_060896.1](http://www.ncbi.nlm.nih.gov/entrez/query.fcgi?cmd=Retrieve&db=Protein&list_uids=55769589&dopt=GenPept&RID=T0CXSAHZ01N&log$=prottop&blast_rank=71) | transmembrane protein 63B [Homo sapiens] | [18.0](http://blast.ncbi.nlm.nih.gov/Blast.cgi#55769589%2355769589) | 18.0 | 57% | 453 |
| [NP_001136087.1](http://www.ncbi.nlm.nih.gov/entrez/query.fcgi?cmd=Retrieve&db=Protein&list_uids=217330572&dopt=GenPept&RID=T0CXSAHZ01N&log$=prottop&blast_rank=72) | EH domain binding protein 1 isoform 3 [Homo sapiens] >ref|NP_001136088.1| EH domain binding protein 1 isoform 3 [Homo sapiens] | [18.0](http://blast.ncbi.nlm.nih.gov/Blast.cgi#217330572%23217330572) | 41.8 | 100% | 453 |
| [NP_001136086.1](http://www.ncbi.nlm.nih.gov/entrez/query.fcgi?cmd=Retrieve&db=Protein&list_uids=217330570&dopt=GenPept&RID=T0CXSAHZ01N&log$=prottop&blast_rank=73) | EH domain binding protein 1 isoform 2 [Homo sapiens] | [18.0](http://blast.ncbi.nlm.nih.gov/Blast.cgi#217330570%23217330570) | 41.8 | 100% | 453 |
| [NP_849144.2](http://www.ncbi.nlm.nih.gov/entrez/query.fcgi?cmd=Retrieve&db=Protein&list_uids=38490688&dopt=GenPept&RID=T0CXSAHZ01N&log$=prottop&blast_rank=74) | immunoglobulin superfamily, member 10 precursor [Homo sapiens] | [18.0](http://blast.ncbi.nlm.nih.gov/Blast.cgi#38490688%2338490688) | 33.5 | 71% | 453 |
| [NP_006413.2](http://www.ncbi.nlm.nih.gov/entrez/query.fcgi?cmd=Retrieve&db=Protein&list_uids=21493041&dopt=GenPept&RID=T0CXSAHZ01N&log$=prottop&blast_rank=75) | A-kinase anchor protein 3 [Homo sapiens] | [18.0](http://blast.ncbi.nlm.nih.gov/Blast.cgi#21493041%2321493041) | 33.9 | 100% | 453 |
| [NP_001129505.1](http://www.ncbi.nlm.nih.gov/entrez/query.fcgi?cmd=Retrieve&db=Protein&list_uids=209862867&dopt=GenPept&RID=T0CXSAHZ01N&log$=prottop&blast_rank=76) | poly-U binding splicing factor 60KDa isoform c [Homo sapiens] | [18.0](http://blast.ncbi.nlm.nih.gov/Blast.cgi#209862867%23209862867) | 18.0 | 71% | 453 |
| [NP_055096.2](http://www.ncbi.nlm.nih.gov/entrez/query.fcgi?cmd=Retrieve&db=Protein&list_uids=17298690&dopt=GenPept&RID=T0CXSAHZ01N&log$=prottop&blast_rank=77) | poly-U binding splicing factor 60KDa isoform b [Homo sapiens] | [18.0](http://blast.ncbi.nlm.nih.gov/Blast.cgi#17298690%2317298690) | 18.0 | 71% | 453 |
| [NP_004758.3](http://www.ncbi.nlm.nih.gov/entrez/query.fcgi?cmd=Retrieve&db=Protein&list_uids=134244291&dopt=GenPept&RID=T0CXSAHZ01N&log$=prottop&blast_rank=78) | G-protein coupled receptor 37 like 1 precursor [Homo sapiens] | [18.0](http://blast.ncbi.nlm.nih.gov/Blast.cgi#134244291%23134244291) | 18.0 | 57% | 453 |
| [NP_001003698.1](http://www.ncbi.nlm.nih.gov/entrez/query.fcgi?cmd=Retrieve&db=Protein&list_uids=51173735&dopt=GenPept&RID=T0CXSAHZ01N&log$=prottop&blast_rank=79) | ras responsive element binding protein 1 isoform 2 [Homo sapiens] >ref|NP_001161816.1| ras responsive element binding protein 1 isoform 2 [Homo sapiens] | [18.0](http://blast.ncbi.nlm.nih.gov/Blast.cgi#51173735%2351173735) | 18.0 | 71% | 453 |
| [NP_006833.2](http://www.ncbi.nlm.nih.gov/entrez/query.fcgi?cmd=Retrieve&db=Protein&list_uids=55749531&dopt=GenPept&RID=T0CXSAHZ01N&log$=prottop&blast_rank=80) | splicing factor 3B subunit 2 [Homo sapiens] | [18.0](http://blast.ncbi.nlm.nih.gov/Blast.cgi#55749531%2355749531) | 18.0 | 71% | 453 |
| [NP_055893.2](http://www.ncbi.nlm.nih.gov/entrez/query.fcgi?cmd=Retrieve&db=Protein&list_uids=31742505&dopt=GenPept&RID=T0CXSAHZ01N&log$=prottop&blast_rank=81) | Rho family guanine-nucleotide exchange factor [Homo sapiens] | [18.0](http://blast.ncbi.nlm.nih.gov/Blast.cgi#31742505%2331742505) | 18.0 | 57% | 453 |
| [NP_060151.2](http://www.ncbi.nlm.nih.gov/entrez/query.fcgi?cmd=Retrieve&db=Protein&list_uids=222144277&dopt=GenPept&RID=T0CXSAHZ01N&log$=prottop&blast_rank=82) | nucleoporin 62kDa C-terminal like [Homo sapiens] | [18.0](http://blast.ncbi.nlm.nih.gov/Blast.cgi#222144277%23222144277) | 18.0 | 57% | 453 |
| [NP_065928.2](http://www.ncbi.nlm.nih.gov/entrez/query.fcgi?cmd=Retrieve&db=Protein&list_uids=75677365&dopt=GenPept&RID=T0CXSAHZ01N&log$=prottop&blast_rank=83) | dynein heavy chain domain 3 [Homo sapiens] | [18.0](http://blast.ncbi.nlm.nih.gov/Blast.cgi#75677365%2375677365) | 59.8 | 100% | 453 |
| [NP_056128.1](http://www.ncbi.nlm.nih.gov/entrez/query.fcgi?cmd=Retrieve&db=Protein&list_uids=7662088&dopt=GenPept&RID=T0CXSAHZ01N&log$=prottop&blast_rank=84) | Rho guanine nucleotide exchange factor (GEF) 12 [Homo sapiens] | [18.0](http://blast.ncbi.nlm.nih.gov/Blast.cgi#7662088%237662088) | 33.9 | 85% | 453 |
| [NP_001092879.1](http://www.ncbi.nlm.nih.gov/entrez/query.fcgi?cmd=Retrieve&db=Protein&list_uids=150378549&dopt=GenPept&RID=T0CXSAHZ01N&log$=prottop&blast_rank=85) | tangerin [Homo sapiens] | [18.0](http://blast.ncbi.nlm.nih.gov/Blast.cgi#150378549%23150378549) | 31.4 | 100% | 453 |
| [NP_001073946.1](http://www.ncbi.nlm.nih.gov/entrez/query.fcgi?cmd=Retrieve&db=Protein&list_uids=122937400&dopt=GenPept&RID=T0CXSAHZ01N&log$=prottop&blast_rank=86) | odz, odd Oz/ten-m homolog 3 [Homo sapiens] | [18.0](http://blast.ncbi.nlm.nih.gov/Blast.cgi#122937400%23122937400) | 42.2 | 85% | 453 |
| [NP_001003699.1](http://www.ncbi.nlm.nih.gov/entrez/query.fcgi?cmd=Retrieve&db=Protein&list_uids=51173737&dopt=GenPept&RID=T0CXSAHZ01N&log$=prottop&blast_rank=87) | ras responsive element binding protein 1 isoform 1 [Homo sapiens] | [18.0](http://blast.ncbi.nlm.nih.gov/Blast.cgi#51173737%2351173737) | 18.0 | 71% | 453 |
| [NP_009007.2](http://www.ncbi.nlm.nih.gov/entrez/query.fcgi?cmd=Retrieve&db=Protein&list_uids=42794620&dopt=GenPept&RID=T0CXSAHZ01N&log$=prottop&blast_rank=88) | Huntingtin interacting protein E [Homo sapiens] | [18.0](http://blast.ncbi.nlm.nih.gov/Blast.cgi#42794620%2342794620) | 18.0 | 71% | 453 |
| [NP_061850.2](http://www.ncbi.nlm.nih.gov/entrez/query.fcgi?cmd=Retrieve&db=Protein&list_uids=51593088&dopt=GenPept&RID=T0CXSAHZ01N&log$=prottop&blast_rank=89) | neuroligin 3 isoform 2 [Homo sapiens] | [18.0](http://blast.ncbi.nlm.nih.gov/Blast.cgi#51593088%2351593088) | 18.0 | 57% | 453 |
| [NP_699185.1](http://www.ncbi.nlm.nih.gov/entrez/query.fcgi?cmd=Retrieve&db=Protein&list_uids=23503279&dopt=GenPept&RID=T0CXSAHZ01N&log$=prottop&blast_rank=90) | transmembrane protein 161B [Homo sapiens] | [18.0](http://blast.ncbi.nlm.nih.gov/Blast.cgi#23503279%2323503279) | 18.0 | 71% | 453 |
| [NP_066968.3](http://www.ncbi.nlm.nih.gov/entrez/query.fcgi?cmd=Retrieve&db=Protein&list_uids=215599967&dopt=GenPept&RID=T0CXSAHZ01N&log$=prottop&blast_rank=91) | cyclin D binding myb-like transcription factor 1 isoform a [Homo sapiens] >ref|NP_001135799.1| cyclin D binding myb-like transcription factor 1 isoform a [Homo sapiens] | [18.0](http://blast.ncbi.nlm.nih.gov/Blast.cgi#215599967%23215599967) | 18.0 | 71% | 453 |
| [NP_068569.2](http://www.ncbi.nlm.nih.gov/entrez/query.fcgi?cmd=Retrieve&db=Protein&list_uids=42542398&dopt=GenPept&RID=T0CXSAHZ01N&log$=prottop&blast_rank=92) | acidic chitinase isoform a [Homo sapiens] | [18.0](http://blast.ncbi.nlm.nih.gov/Blast.cgi#42542398%2342542398) | 18.0 | 57% | 453 |
| [NP_055747.1](http://www.ncbi.nlm.nih.gov/entrez/query.fcgi?cmd=Retrieve&db=Protein&list_uids=7662470&dopt=GenPept&RID=T0CXSAHZ01N&log$=prottop&blast_rank=93) | neuroligin 1 [Homo sapiens] | [18.0](http://blast.ncbi.nlm.nih.gov/Blast.cgi#7662470%237662470) | 18.0 | 57% | 453 |
| [NP_055456.2](http://www.ncbi.nlm.nih.gov/entrez/query.fcgi?cmd=Retrieve&db=Protein&list_uids=132626688&dopt=GenPept&RID=T0CXSAHZ01N&log$=prottop&blast_rank=94) | mediator of DNA-damage checkpoint 1 [Homo sapiens] | [18.0](http://blast.ncbi.nlm.nih.gov/Blast.cgi#132626688%23132626688) | 46.5 | 85% | 453 |
| [NP_851820.1](http://www.ncbi.nlm.nih.gov/entrez/query.fcgi?cmd=Retrieve&db=Protein&list_uids=262359971&dopt=GenPept&RID=T0CXSAHZ01N&log$=prottop&blast_rank=95) | neuroligin 3 isoform 1 [Homo sapiens] | [18.0](http://blast.ncbi.nlm.nih.gov/Blast.cgi#262359971%23262359971) | 18.0 | 57% | 453 |
| [NP_073153.1](http://www.ncbi.nlm.nih.gov/entrez/query.fcgi?cmd=Retrieve&db=Protein&list_uids=12056971&dopt=GenPept&RID=T0CXSAHZ01N&log$=prottop&blast_rank=96) | anaphase promoting complex subunit 1 [Homo sapiens] | [18.0](http://blast.ncbi.nlm.nih.gov/Blast.cgi#12056971%2312056971) | 18.0 | 57% | 453 |
| [NP_065793.1](http://www.ncbi.nlm.nih.gov/entrez/query.fcgi?cmd=Retrieve&db=Protein&list_uids=24308209&dopt=GenPept&RID=T0CXSAHZ01N&log$=prottop&blast_rank=97) | X-linked neuroligin 4 precursor [Homo sapiens] >ref|NP_851849.1| X-linked neuroligin 4 precursor [Homo sapiens] | [18.0](http://blast.ncbi.nlm.nih.gov/Blast.cgi#24308209%2324308209) | 18.0 | 57% | 453 |
| [NP_002538.1](http://www.ncbi.nlm.nih.gov/entrez/query.fcgi?cmd=Retrieve&db=Protein&list_uids=4505507&dopt=GenPept&RID=T0CXSAHZ01N&log$=prottop&blast_rank=98) | oligophrenin 1 [Homo sapiens] | [18.0](http://blast.ncbi.nlm.nih.gov/Blast.cgi#4505507%234505507) | 18.0 | 57% | 453 |
| [NP_055708.3](http://www.ncbi.nlm.nih.gov/entrez/query.fcgi?cmd=Retrieve&db=Protein&list_uids=256222771&dopt=GenPept&RID=T0CXSAHZ01N&log$=prottop&blast_rank=99) | neuroligin 4, Y-linked isoform 1 [Homo sapiens] | [18.0](http://blast.ncbi.nlm.nih.gov/Blast.cgi#256222771%23256222771) | 18.0 | 57% | 453 |
| [NP_689846.1](http://www.ncbi.nlm.nih.gov/entrez/query.fcgi?cmd=Retrieve&db=Protein&list_uids=22749293&dopt=GenPept&RID=T0CXSAHZ01N&log$=prottop&blast_rank=100) | Fanconi anemia complementation group B [Homo sapiens] >ref|NP_001018123.1| Fanconi anemia complementation group B [Homo sapiens] | [18.0](http://blast.ncbi.nlm.nih.gov/Blast.cgi#22749293%2322749293) | 18.0 | 85% | 453 |

| **Accession** | **Proteins with a match to FQSPK peptide** | **[Max score](http://blast.ncbi.nlm.nih.gov/Blast.cgi?CMD=Get&ALIGNMENTS=100&ALIGNMENT_VIEW=Pairwise&CDD_SEARCH_STATE=1&DATABASE_SORT=0&DESCRIPTIONS=100&ENTREZ_QUERY=txid9606 %5BORGN%5D&FIRST_QUERY_NUM=0&FORMAT_OBJECT=Alignment&FORMAT_PAGE_TARGET=&FORMAT_TYPE=HTML&GET_SEQUENCE=yes&I_THRESH=&MASK_CHAR=2&MASK_COLOR=1&NEW_DESIGN=on&NEW_VIEW=yes&NUM_OVERVIEW=100&OLD_BLAST=false&PAGE=Proteins&QUERY_INDEX=0&QUERY_NUMBER=0&RESULTS_PAGE_TARGET=&RID=T2B1WG1R014&SHOW_LINKOUT=yes&SHOW_OVERVIEW=yes&STEP_NUMBER=&WORD_SIZE=2&DISPLAY_SORT=1&HSP_SORT=1" \l "sort_mark)** | **[Total score](http://blast.ncbi.nlm.nih.gov/Blast.cgi?CMD=Get&ALIGNMENTS=100&ALIGNMENT_VIEW=Pairwise&CDD_SEARCH_STATE=1&DATABASE_SORT=0&DESCRIPTIONS=100&ENTREZ_QUERY=txid9606 %5BORGN%5D&FIRST_QUERY_NUM=0&FORMAT_OBJECT=Alignment&FORMAT_PAGE_TARGET=&FORMAT_TYPE=HTML&GET_SEQUENCE=yes&I_THRESH=&MASK_CHAR=2&MASK_COLOR=1&NEW_DESIGN=on&NEW_VIEW=yes&NUM_OVERVIEW=100&OLD_BLAST=false&PAGE=Proteins&QUERY_INDEX=0&QUERY_NUMBER=0&RESULTS_PAGE_TARGET=&RID=T2B1WG1R014&SHOW_LINKOUT=yes&SHOW_OVERVIEW=yes&STEP_NUMBER=&WORD_SIZE=2&DISPLAY_SORT=2&HSP_SORT=1" \l "sort_mark)** | **[Query coverage](http://blast.ncbi.nlm.nih.gov/Blast.cgi?CMD=Get&ALIGNMENTS=100&ALIGNMENT_VIEW=Pairwise&CDD_SEARCH_STATE=1&DATABASE_SORT=0&DESCRIPTIONS=100&ENTREZ_QUERY=txid9606 %5BORGN%5D&FIRST_QUERY_NUM=0&FORMAT_OBJECT=Alignment&FORMAT_PAGE_TARGET=&FORMAT_TYPE=HTML&GET_SEQUENCE=yes&I_THRESH=&MASK_CHAR=2&MASK_COLOR=1&NEW_DESIGN=on&NEW_VIEW=yes&NUM_OVERVIEW=100&OLD_BLAST=false&PAGE=Proteins&QUERY_INDEX=0&QUERY_NUMBER=0&RESULTS_PAGE_TARGET=&RID=T2B1WG1R014&SHOW_LINKOUT=yes&SHOW_OVERVIEW=yes&STEP_NUMBER=&WORD_SIZE=2&DISPLAY_SORT=4&HSP_SORT=0" \l "sort_mark)** | **[E value](http://blast.ncbi.nlm.nih.gov/Blast.cgi?CMD=Get&ALIGNMENTS=100&ALIGNMENT_VIEW=Pairwise&CDD_SEARCH_STATE=1&DATABASE_SORT=0&DESCRIPTIONS=100&ENTREZ_QUERY=txid9606 %5BORGN%5D&FIRST_QUERY_NUM=0&FORMAT_OBJECT=Alignment&FORMAT_PAGE_TARGET=&FORMAT_TYPE=HTML&GET_SEQUENCE=yes&I_THRESH=&MASK_CHAR=2&MASK_COLOR=1&NEW_DESIGN=on&NEW_VIEW=yes&NUM_OVERVIEW=100&OLD_BLAST=false&PAGE=Proteins&QUERY_INDEX=0&QUERY_NUMBER=0&RESULTS_PAGE_TARGET=&RID=T2B1WG1R014&SHOW_LINKOUT=yes&SHOW_OVERVIEW=yes&STEP_NUMBER=&WORD_SIZE=2&DISPLAY_SORT=0&HSP_SORT=0" \l "sort_mark)** |
| --- | --- | --- | --- | --- | --- |
| [NP_001159921.1](http://www.ncbi.nlm.nih.gov/entrez/query.fcgi?cmd=Retrieve&db=Protein&list_uids=262050538&dopt=GenPept&RID=T2B1WG1R014&log$=prottop&blast_rank=1) | inter-alpha (globulin) inhibitor H4 isoform 2 precursor [Homo sapiens] | [19.3](http://blast.ncbi.nlm.nih.gov/Blast.cgi#262050538%23262050538) | 19.3 | 100% | 134 |
| [NP_060024.2](http://www.ncbi.nlm.nih.gov/entrez/query.fcgi?cmd=Retrieve&db=Protein&list_uids=154813199&dopt=GenPept&RID=T2B1WG1R014&log$=prottop&blast_rank=2) | poly (ADP-ribose) polymerase family, member 14 [Homo sapiens] | [19.3](http://blast.ncbi.nlm.nih.gov/Blast.cgi#154813199%23154813199) | 19.3 | 100% | 134 |
| [NP_002025.2](http://www.ncbi.nlm.nih.gov/entrez/query.fcgi?cmd=Retrieve&db=Protein&list_uids=52485799&dopt=GenPept&RID=T2B1WG1R014&log$=prottop&blast_rank=3) | fucosyltransferase 5 [Homo sapiens] | [19.3](http://blast.ncbi.nlm.nih.gov/Blast.cgi#52485799%2352485799) | 19.3 | 100% | 134 |
| [NP_000140.1](http://www.ncbi.nlm.nih.gov/entrez/query.fcgi?cmd=Retrieve&db=Protein&list_uids=4503809&dopt=GenPept&RID=T2B1WG1R014&log$=prottop&blast_rank=4) | fucosyltransferase 3 [Homo sapiens] >ref|NP_001091108.1| fucosyltransferase 3 [Homo sapiens] >ref|NP_001091109.1| fucosyltransferase 3 [Homo sapiens] >ref|NP_001091110.1| fucosyltransferase 3 [Homo sapiens] | [19.3](http://blast.ncbi.nlm.nih.gov/Blast.cgi#4503809%234503809) | 19.3 | 100% | 134 |
| [NP_000141.1](http://www.ncbi.nlm.nih.gov/entrez/query.fcgi?cmd=Retrieve&db=Protein&list_uids=4503815&dopt=GenPept&RID=T2B1WG1R014&log$=prottop&blast_rank=5) | fucosyltransferase 6 [Homo sapiens] >ref|NP_001035791.1| fucosyltransferase 6 [Homo sapiens] | [19.3](http://blast.ncbi.nlm.nih.gov/Blast.cgi#4503815%234503815) | 19.3 | 100% | 134 |
| [NP_002209.2](http://www.ncbi.nlm.nih.gov/entrez/query.fcgi?cmd=Retrieve&db=Protein&list_uids=31542984&dopt=GenPept&RID=T2B1WG1R014&log$=prottop&blast_rank=6) | inter-alpha (globulin) inhibitor H4 isoform 1 precursor [Homo sapiens] | [19.3](http://blast.ncbi.nlm.nih.gov/Blast.cgi#31542984%2331542984) | 19.3 | 100% | 134 |
| [NP_001162023.1](http://www.ncbi.nlm.nih.gov/entrez/query.fcgi?cmd=Retrieve&db=Protein&list_uids=271398379&dopt=GenPept&RID=T2B1WG1R014&log$=prottop&blast_rank=7) | transmembrane protein 48 isoform 2 [Homo sapiens] | [16.8](http://blast.ncbi.nlm.nih.gov/Blast.cgi#271398379%23271398379) | 16.8 | 100% | 782 |
| [NP_653267.2](http://www.ncbi.nlm.nih.gov/entrez/query.fcgi?cmd=Retrieve&db=Protein&list_uids=222144249&dopt=GenPept&RID=T2B1WG1R014&log$=prottop&blast_rank=8) | dynein heavy chain domain 1 isoform 1 [Homo sapiens] | [16.8](http://blast.ncbi.nlm.nih.gov/Blast.cgi#222144249%23222144249) | 16.8 | 100% | 782 |
| [XP_001719624.1](http://www.ncbi.nlm.nih.gov/entrez/query.fcgi?cmd=Retrieve&db=Protein&list_uids=169177315&dopt=GenPept&RID=T2B1WG1R014&log$=prottop&blast_rank=9) | PREDICTED: similar to Protein FAM27D1 [Homo sapiens] | [16.8](http://blast.ncbi.nlm.nih.gov/Blast.cgi#169177315%23169177315) | 16.8 | 100% | 782 |
| [XP_001720518.1](http://www.ncbi.nlm.nih.gov/entrez/query.fcgi?cmd=Retrieve&db=Protein&list_uids=169184225&dopt=GenPept&RID=T2B1WG1R014&log$=prottop&blast_rank=10) | PREDICTED: similar to Protein FAM27D1 [Homo sapiens] | [16.8](http://blast.ncbi.nlm.nih.gov/Blast.cgi#169184225%23169184225) | 16.8 | 100% | 782 |
| [NP_065770.1](http://www.ncbi.nlm.nih.gov/entrez/query.fcgi?cmd=Retrieve&db=Protein&list_uids=153792074&dopt=GenPept&RID=T2B1WG1R014&log$=prottop&blast_rank=11) | proline rich 12 [Homo sapiens] | [16.8](http://blast.ncbi.nlm.nih.gov/Blast.cgi#153792074%23153792074) | 16.8 | 100% | 782 |
| [NP_005116.1](http://www.ncbi.nlm.nih.gov/entrez/query.fcgi?cmd=Retrieve&db=Protein&list_uids=4826665&dopt=GenPept&RID=T2B1WG1R014&log$=prottop&blast_rank=12) | copper chaperone for superoxide dismutase [Homo sapiens] | [16.8](http://blast.ncbi.nlm.nih.gov/Blast.cgi#4826665%234826665) | 16.8 | 100% | 782 |
| [NP_057031.2](http://www.ncbi.nlm.nih.gov/entrez/query.fcgi?cmd=Retrieve&db=Protein&list_uids=56676391&dopt=GenPept&RID=T2B1WG1R014&log$=prottop&blast_rank=13) | activating signal cointegrator 1 complex subunit 1 [Homo sapiens] | [16.8](http://blast.ncbi.nlm.nih.gov/Blast.cgi#56676391%2356676391) | 16.8 | 100% | 782 |
| [NP_060557.3](http://www.ncbi.nlm.nih.gov/entrez/query.fcgi?cmd=Retrieve&db=Protein&list_uids=271398350&dopt=GenPept&RID=T2B1WG1R014&log$=prottop&blast_rank=14) | transmembrane protein 48 isoform 1 [Homo sapiens] | [16.8](http://blast.ncbi.nlm.nih.gov/Blast.cgi#271398350%23271398350) | 16.8 | 100% | 782 |
| [NP_000457.1](http://www.ncbi.nlm.nih.gov/entrez/query.fcgi?cmd=Retrieve&db=Protein&list_uids=4505725&dopt=GenPept&RID=T2B1WG1R014&log$=prottop&blast_rank=15) | peroxin1 [Homo sapiens] | [16.8](http://blast.ncbi.nlm.nih.gov/Blast.cgi#4505725%234505725) | 16.8 | 100% | 782 |
| [NP_055649.4](http://www.ncbi.nlm.nih.gov/entrez/query.fcgi?cmd=Retrieve&db=Protein&list_uids=289547512&dopt=GenPept&RID=T2B1WG1R014&log$=prottop&blast_rank=16) | leucine rich repeat containing 37A precursor [Homo sapiens] | [16.3](http://blast.ncbi.nlm.nih.gov/Blast.cgi#289547512%23289547512) | 16.3 | 80% | 1049 |
| [NP_001164190.1](http://www.ncbi.nlm.nih.gov/entrez/query.fcgi?cmd=Retrieve&db=Protein&list_uids=282398123&dopt=GenPept&RID=T2B1WG1R014&log$=prottop&blast_rank=17) | breast cancer anti-estrogen resistance 1 isoform 7 [Homo sapiens] | [16.3](http://blast.ncbi.nlm.nih.gov/Blast.cgi#282398123%23282398123) | 16.3 | 80% | 1049 |
| [NP_001164189.1](http://www.ncbi.nlm.nih.gov/entrez/query.fcgi?cmd=Retrieve&db=Protein&list_uids=282398120&dopt=GenPept&RID=T2B1WG1R014&log$=prottop&blast_rank=18) | breast cancer anti-estrogen resistance 1 isoform 5 [Homo sapiens] | [16.3](http://blast.ncbi.nlm.nih.gov/Blast.cgi#282398120%23282398120) | 16.3 | 80% | 1049 |
| [NP_001164188.1](http://www.ncbi.nlm.nih.gov/entrez/query.fcgi?cmd=Retrieve&db=Protein&list_uids=282398118&dopt=GenPept&RID=T2B1WG1R014&log$=prottop&blast_rank=19) | breast cancer anti-estrogen resistance 1 isoform 4 [Homo sapiens] | [16.3](http://blast.ncbi.nlm.nih.gov/Blast.cgi#282398118%23282398118) | 16.3 | 80% | 1049 |
| [NP_001164186.1](http://www.ncbi.nlm.nih.gov/entrez/query.fcgi?cmd=Retrieve&db=Protein&list_uids=282398114&dopt=GenPept&RID=T2B1WG1R014&log$=prottop&blast_rank=20) | breast cancer anti-estrogen resistance 1 isoform 2 [Homo sapiens] | [16.3](http://blast.ncbi.nlm.nih.gov/Blast.cgi#282398114%23282398114) | 16.3 | 80% | 1049 |
| [NP_001164185.1](http://www.ncbi.nlm.nih.gov/entrez/query.fcgi?cmd=Retrieve&db=Protein&list_uids=282398112&dopt=GenPept&RID=T2B1WG1R014&log$=prottop&blast_rank=21) | breast cancer anti-estrogen resistance 1 isoform 1 [Homo sapiens] | [16.3](http://blast.ncbi.nlm.nih.gov/Blast.cgi#282398112%23282398112) | 16.3 | 80% | 1049 |
| [NP_065810.2](http://www.ncbi.nlm.nih.gov/entrez/query.fcgi?cmd=Retrieve&db=Protein&list_uids=270265793&dopt=GenPept&RID=T2B1WG1R014&log$=prottop&blast_rank=22) | StAR-related lipid transfer (START) domain containing 9 [Homo sapiens] | [16.3](http://blast.ncbi.nlm.nih.gov/Blast.cgi#270265793%23270265793) | 31.8 | 100% | 1049 |
| [NP_001073882.2](http://www.ncbi.nlm.nih.gov/entrez/query.fcgi?cmd=Retrieve&db=Protein&list_uids=269784635&dopt=GenPept&RID=T2B1WG1R014&log$=prottop&blast_rank=23) | NOBOX oogenesis homeobox [Homo sapiens] | [16.3](http://blast.ncbi.nlm.nih.gov/Blast.cgi#269784635%23269784635) | 16.3 | 80% | 1049 |
| [NP_001161081.1](http://www.ncbi.nlm.nih.gov/entrez/query.fcgi?cmd=Retrieve&db=Protein&list_uids=263190680&dopt=GenPept&RID=T2B1WG1R014&log$=prottop&blast_rank=24) | GLI family zinc finger 1 isoform 3 [Homo sapiens] | [16.3](http://blast.ncbi.nlm.nih.gov/Blast.cgi#263190680%23263190680) | 16.3 | 100% | 1049 |
| [XP_001717557.2](http://www.ncbi.nlm.nih.gov/entrez/query.fcgi?cmd=Retrieve&db=Protein&list_uids=239756270&dopt=GenPept&RID=T2B1WG1R014&log$=prottop&blast_rank=25) | PREDICTED: StAR-related lipid transfer (START) domain containing 9 [Homo sapiens] | [16.3](http://blast.ncbi.nlm.nih.gov/Blast.cgi#239756270%23239756270) | 31.8 | 100% | 1049 |
| [XP_002347637.1](http://www.ncbi.nlm.nih.gov/entrez/query.fcgi?cmd=Retrieve&db=Protein&list_uids=239750986&dopt=GenPept&RID=T2B1WG1R014&log$=prottop&blast_rank=26) | PREDICTED: hypothetical protein XP_002347637 [Homo sapiens] >ref|XP_002344993.1| PREDICTED: similar to ZNF668 protein [Homo sapiens] | [16.3](http://blast.ncbi.nlm.nih.gov/Blast.cgi#239750986%23239750986) | 16.3 | 80% | 1049 |
| [XP_001717819.2](http://www.ncbi.nlm.nih.gov/entrez/query.fcgi?cmd=Retrieve&db=Protein&list_uids=239750776&dopt=GenPept&RID=T2B1WG1R014&log$=prottop&blast_rank=27) | PREDICTED: similar to breast cancer anti-estrogen resistance 1 [Homo sapiens] | [16.3](http://blast.ncbi.nlm.nih.gov/Blast.cgi#239750776%23239750776) | 16.3 | 80% | 1049 |
| [XP_002346883.1](http://www.ncbi.nlm.nih.gov/entrez/query.fcgi?cmd=Retrieve&db=Protein&list_uids=239749021&dopt=GenPept&RID=T2B1WG1R014&log$=prottop&blast_rank=28) | PREDICTED: hypothetical protein LOC401287 [Homo sapiens] >ref|XP_002345999.1| PREDICTED: hypothetical protein LOC401287 [Homo sapiens] | [16.3](http://blast.ncbi.nlm.nih.gov/Blast.cgi#239749021%23239749021) | 16.3 | 80% | 1049 |
| [XP_002343487.1](http://www.ncbi.nlm.nih.gov/entrez/query.fcgi?cmd=Retrieve&db=Protein&list_uids=239745391&dopt=GenPept&RID=T2B1WG1R014&log$=prottop&blast_rank=29) | PREDICTED: hypothetical protein XP_002343487 [Homo sapiens] | [16.3](http://blast.ncbi.nlm.nih.gov/Blast.cgi#239745391%23239745391) | 16.3 | 80% | 1049 |
| [XP_001129482.3](http://www.ncbi.nlm.nih.gov/entrez/query.fcgi?cmd=Retrieve&db=Protein&list_uids=239745175&dopt=GenPept&RID=T2B1WG1R014&log$=prottop&blast_rank=30) | PREDICTED: StAR-related lipid transfer (START) domain containing 9 [Homo sapiens] >ref|XP_001129290.3| PREDICTED: StAR-related lipid transfer (START) domain containing 9 [Homo sapiens] | [16.3](http://blast.ncbi.nlm.nih.gov/Blast.cgi#239745175%23239745175) | 31.8 | 100% | 1049 |
| [XP_001716809.2](http://www.ncbi.nlm.nih.gov/entrez/query.fcgi?cmd=Retrieve&db=Protein&list_uids=239745060&dopt=GenPept&RID=T2B1WG1R014&log$=prottop&blast_rank=31) | PREDICTED: similar to breast cancer anti-estrogen resistance 1 [Homo sapiens] | [16.3](http://blast.ncbi.nlm.nih.gov/Blast.cgi#239745060%23239745060) | 16.3 | 80% | 1049 |
| [XP_934132.2](http://www.ncbi.nlm.nih.gov/entrez/query.fcgi?cmd=Retrieve&db=Protein&list_uids=239745044&dopt=GenPept&RID=T2B1WG1R014&log$=prottop&blast_rank=32) | PREDICTED: similar to breast cancer anti-estrogen resistance 1 [Homo sapiens] | [16.3](http://blast.ncbi.nlm.nih.gov/Blast.cgi#239745044%23239745044) | 16.3 | 80% | 1049 |
| [XP_002342724.1](http://www.ncbi.nlm.nih.gov/entrez/query.fcgi?cmd=Retrieve&db=Protein&list_uids=239743040&dopt=GenPept&RID=T2B1WG1R014&log$=prottop&blast_rank=33) | PREDICTED: hypothetical protein LOC401287 [Homo sapiens] | [16.3](http://blast.ncbi.nlm.nih.gov/Blast.cgi#239743040%23239743040) | 16.3 | 80% | 1049 |
| [XP_002342170.1](http://www.ncbi.nlm.nih.gov/entrez/query.fcgi?cmd=Retrieve&db=Protein&list_uids=239741440&dopt=GenPept&RID=T2B1WG1R014&log$=prottop&blast_rank=34) | PREDICTED: hypothetical protein XP_002342170 [Homo sapiens] >ref|XP_002347661.1| PREDICTED: hypothetical protein XP_002347661 [Homo sapiens] | [16.3](http://blast.ncbi.nlm.nih.gov/Blast.cgi#239741440%23239741440) | 16.3 | 80% | 1049 |
| [XP_002344102.1](http://www.ncbi.nlm.nih.gov/entrez/query.fcgi?cmd=Retrieve&db=Protein&list_uids=239740955&dopt=GenPept&RID=T2B1WG1R014&log$=prottop&blast_rank=35) | PREDICTED: similar to c114 SLIT-like testicular protein isoform 1 [Homo sapiens] | [16.3](http://blast.ncbi.nlm.nih.gov/Blast.cgi#239740955%23239740955) | 16.3 | 80% | 1049 |
| [XP_002344103.1](http://www.ncbi.nlm.nih.gov/entrez/query.fcgi?cmd=Retrieve&db=Protein&list_uids=239740953&dopt=GenPept&RID=T2B1WG1R014&log$=prottop&blast_rank=36) | PREDICTED: similar to c114 SLIT-like testicular protein isoform 2 [Homo sapiens] | [16.3](http://blast.ncbi.nlm.nih.gov/Blast.cgi#239740953%23239740953) | 16.3 | 80% | 1049 |
| [XP_001716006.2](http://www.ncbi.nlm.nih.gov/entrez/query.fcgi?cmd=Retrieve&db=Protein&list_uids=239756225&dopt=GenPept&RID=T2B1WG1R014&log$=prottop&blast_rank=37) | PREDICTED: similar to breast cancer anti-estrogen resistance 1 [Homo sapiens] | [16.3](http://blast.ncbi.nlm.nih.gov/Blast.cgi#239756225%23239756225) | 16.3 | 80% | 1049 |
| [XP_002343606.1](http://www.ncbi.nlm.nih.gov/entrez/query.fcgi?cmd=Retrieve&db=Protein&list_uids=239745855&dopt=GenPept&RID=T2B1WG1R014&log$=prottop&blast_rank=38) | PREDICTED: hypothetical protein XP_002343606 [Homo sapiens] | [16.3](http://blast.ncbi.nlm.nih.gov/Blast.cgi#239745855%23239745855) | 16.3 | 80% | 1049 |
| [NP_001153517.1](http://www.ncbi.nlm.nih.gov/entrez/query.fcgi?cmd=Retrieve&db=Protein&list_uids=229892345&dopt=GenPept&RID=T2B1WG1R014&log$=prottop&blast_rank=39) | GLI family zinc finger 1 isoform 2 [Homo sapiens] | [16.3](http://blast.ncbi.nlm.nih.gov/Blast.cgi#229892345%23229892345) | 16.3 | 100% | 1049 |
| [NP_073741.2](http://www.ncbi.nlm.nih.gov/entrez/query.fcgi?cmd=Retrieve&db=Protein&list_uids=226371750&dopt=GenPept&RID=T2B1WG1R014&log$=prottop&blast_rank=40) | terminal uridylyl transferase 1, U6 snRNA-specific [Homo sapiens] | [16.3](http://blast.ncbi.nlm.nih.gov/Blast.cgi#226371750%23226371750) | 16.3 | 80% | 1049 |
| [NP_055633.1](http://www.ncbi.nlm.nih.gov/entrez/query.fcgi?cmd=Retrieve&db=Protein&list_uids=209977097&dopt=GenPept&RID=T2B1WG1R014&log$=prottop&blast_rank=41) | tripartite motif-containing 66 [Homo sapiens] | [16.3](http://blast.ncbi.nlm.nih.gov/Blast.cgi#209977097%23209977097) | 16.3 | 80% | 1049 |
| [NP_115511.3](http://www.ncbi.nlm.nih.gov/entrez/query.fcgi?cmd=Retrieve&db=Protein&list_uids=209977046&dopt=GenPept&RID=T2B1WG1R014&log$=prottop&blast_rank=42) | fibrous sheath CABYR binding protein [Homo sapiens] | [16.3](http://blast.ncbi.nlm.nih.gov/Blast.cgi#209977046%23209977046) | 32.7 | 80% | 1049 |
| [NP_001129206.1](http://www.ncbi.nlm.nih.gov/entrez/query.fcgi?cmd=Retrieve&db=Protein&list_uids=209180475&dopt=GenPept&RID=T2B1WG1R014&log$=prottop&blast_rank=43) | nuclear matrix transcription factor 4 isoform d [Homo sapiens] | [16.3](http://blast.ncbi.nlm.nih.gov/Blast.cgi#209180475%23209180475) | 16.3 | 80% | 1049 |
| [NP_001164187.1](http://www.ncbi.nlm.nih.gov/entrez/query.fcgi?cmd=Retrieve&db=Protein&list_uids=282398116&dopt=GenPept&RID=T2B1WG1R014&log$=prottop&blast_rank=44) | breast cancer anti-estrogen resistance 1 isoform 3 [Homo sapiens] | [16.3](http://blast.ncbi.nlm.nih.gov/Blast.cgi#282398116%23282398116) | 16.3 | 80% | 1049 |
| [NP_001129292.1](http://www.ncbi.nlm.nih.gov/entrez/query.fcgi?cmd=Retrieve&db=Protein&list_uids=209447096&dopt=GenPept&RID=T2B1WG1R014&log$=prottop&blast_rank=45) | transmembrane protein 2 isoform b [Homo sapiens] | [16.3](http://blast.ncbi.nlm.nih.gov/Blast.cgi#209447096%23209447096) | 16.3 | 80% | 1049 |
| [NP_067008.3](http://www.ncbi.nlm.nih.gov/entrez/query.fcgi?cmd=Retrieve&db=Protein&list_uids=187761334&dopt=GenPept&RID=T2B1WG1R014&log$=prottop&blast_rank=46) | cation channel, sperm-associated, gamma precursor [Homo sapiens] | [16.3](http://blast.ncbi.nlm.nih.gov/Blast.cgi#187761334%23187761334) | 16.3 | 80% | 1049 |
| [NP_001136438.1](http://www.ncbi.nlm.nih.gov/entrez/query.fcgi?cmd=Retrieve&db=Protein&list_uids=219282747&dopt=GenPept&RID=T2B1WG1R014&log$=prottop&blast_rank=47) | growth regulation by estrogen in breast cancer-like [Homo sapiens] | [16.3](http://blast.ncbi.nlm.nih.gov/Blast.cgi#219282747%23219282747) | 16.3 | 80% | 1049 |
| [NP_997192.2](http://www.ncbi.nlm.nih.gov/entrez/query.fcgi?cmd=Retrieve&db=Protein&list_uids=187936958&dopt=GenPept&RID=T2B1WG1R014&log$=prottop&blast_rank=48) | UDP-N-acteylglucosamine pyrophosphorylase 1-like 1 [Homo sapiens] | [16.3](http://blast.ncbi.nlm.nih.gov/Blast.cgi#187936958%23187936958) | 16.3 | 80% | 1049 |
| [NP_963869.2](http://www.ncbi.nlm.nih.gov/entrez/query.fcgi?cmd=Retrieve&db=Protein&list_uids=166235136&dopt=GenPept&RID=T2B1WG1R014&log$=prottop&blast_rank=49) | seizure related 6 homolog (mouse)-like 2 isoform 2 [Homo sapiens] | [16.3](http://blast.ncbi.nlm.nih.gov/Blast.cgi#166235136%23166235136) | 16.3 | 80% | 1049 |
| [NP_056040.2](http://www.ncbi.nlm.nih.gov/entrez/query.fcgi?cmd=Retrieve&db=Protein&list_uids=162287219&dopt=GenPept&RID=T2B1WG1R014&log$=prottop&blast_rank=50) | prune homolog 2 [Homo sapiens] | [16.3](http://blast.ncbi.nlm.nih.gov/Blast.cgi#162287219%23162287219) | 16.3 | 80% | 1049 |
| [NP_001164722.1](http://www.ncbi.nlm.nih.gov/entrez/query.fcgi?cmd=Retrieve&db=Protein&list_uids=284005249&dopt=GenPept&RID=T2B1WG1R014&log$=prottop&blast_rank=51) | hypothetical protein LOC78995 isoform 2 [Homo sapiens] | [16.3](http://blast.ncbi.nlm.nih.gov/Blast.cgi#284005249%23284005249) | 16.3 | 80% | 1049 |
| [NP_065870.3](http://www.ncbi.nlm.nih.gov/entrez/query.fcgi?cmd=Retrieve&db=Protein&list_uids=157671939&dopt=GenPept&RID=T2B1WG1R014&log$=prottop&blast_rank=52) | hypothetical protein LOC57579 isoform b [Homo sapiens] | [16.3](http://blast.ncbi.nlm.nih.gov/Blast.cgi#157671939%23157671939) | 16.3 | 80% | 1049 |
| [NP_001099001.1](http://www.ncbi.nlm.nih.gov/entrez/query.fcgi?cmd=Retrieve&db=Protein&list_uids=157671937&dopt=GenPept&RID=T2B1WG1R014&log$=prottop&blast_rank=53) | hypothetical protein LOC57579 isoform a [Homo sapiens] | [16.3](http://blast.ncbi.nlm.nih.gov/Blast.cgi#157671937%23157671937) | 16.3 | 80% | 1049 |
| [NP_002438.2](http://www.ncbi.nlm.nih.gov/entrez/query.fcgi?cmd=Retrieve&db=Protein&list_uids=153946393&dopt=GenPept&RID=T2B1WG1R014&log$=prottop&blast_rank=54) | macrophage stimulating 1 receptor precursor [Homo sapiens] | [16.3](http://blast.ncbi.nlm.nih.gov/Blast.cgi#153946393%23153946393) | 16.3 | 80% | 1049 |
| [NP_065869.3](http://www.ncbi.nlm.nih.gov/entrez/query.fcgi?cmd=Retrieve&db=Protein&list_uids=150456444&dopt=GenPept&RID=T2B1WG1R014&log$=prottop&blast_rank=55) | hypothetical protein LOC57578 [Homo sapiens] | [16.3](http://blast.ncbi.nlm.nih.gov/Blast.cgi#150456444%23150456444) | 16.3 | 80% | 1049 |
| [NP_061985.2](http://www.ncbi.nlm.nih.gov/entrez/query.fcgi?cmd=Retrieve&db=Protein&list_uids=150417984&dopt=GenPept&RID=T2B1WG1R014&log$=prottop&blast_rank=56) | ATP-binding cassette, sub-family A, member 7 [Homo sapiens] | [16.3](http://blast.ncbi.nlm.nih.gov/Blast.cgi#150417984%23150417984) | 16.3 | 80% | 1049 |
| [NP_055671.2](http://www.ncbi.nlm.nih.gov/entrez/query.fcgi?cmd=Retrieve&db=Protein&list_uids=148922920&dopt=GenPept&RID=T2B1WG1R014&log$=prottop&blast_rank=57) | DENN/MADD domain containing 4B [Homo sapiens] | [16.3](http://blast.ncbi.nlm.nih.gov/Blast.cgi#148922920%23148922920) | 16.3 | 80% | 1049 |
| [NP_060862.3](http://www.ncbi.nlm.nih.gov/entrez/query.fcgi?cmd=Retrieve&db=Protein&list_uids=254826809&dopt=GenPept&RID=T2B1WG1R014&log$=prottop&blast_rank=58) | prematurely terminated mRNA decay factor-like [Homo sapiens] | [16.3](http://blast.ncbi.nlm.nih.gov/Blast.cgi#254826809%23254826809) | 16.3 | 80% | 1049 |
| [NP_001130010.1](http://www.ncbi.nlm.nih.gov/entrez/query.fcgi?cmd=Retrieve&db=Protein&list_uids=211971008&dopt=GenPept&RID=T2B1WG1R014&log$=prottop&blast_rank=59) | acyl-Coenzyme A dehydrogenase family, member 10 isoform a [Homo sapiens] | [16.3](http://blast.ncbi.nlm.nih.gov/Blast.cgi#211971008%23211971008) | 16.3 | 80% | 1049 |
| [NP_001107869.1](http://www.ncbi.nlm.nih.gov/entrez/query.fcgi?cmd=Retrieve&db=Protein&list_uids=167830436&dopt=GenPept&RID=T2B1WG1R014&log$=prottop&blast_rank=60) | DEAH (Asp-Glu-Ala-His) box polypeptide 36 isoform 2 [Homo sapiens] | [16.3](http://blast.ncbi.nlm.nih.gov/Blast.cgi#167830436%23167830436) | 16.3 | 80% | 1049 |
| [NP_065916.2](http://www.ncbi.nlm.nih.gov/entrez/query.fcgi?cmd=Retrieve&db=Protein&list_uids=167830433&dopt=GenPept&RID=T2B1WG1R014&log$=prottop&blast_rank=61) | DEAH (Asp-Glu-Ala-His) box polypeptide 36 isoform 1 [Homo sapiens] | [16.3](http://blast.ncbi.nlm.nih.gov/Blast.cgi#167830433%23167830433) | 16.3 | 80% | 1049 |
| [NP_003473.3](http://www.ncbi.nlm.nih.gov/entrez/query.fcgi?cmd=Retrieve&db=Protein&list_uids=148762969&dopt=GenPept&RID=T2B1WG1R014&log$=prottop&blast_rank=62) | myeloid/lymphoid or mixed-lineage leukemia 2 [Homo sapiens] | [16.3](http://blast.ncbi.nlm.nih.gov/Blast.cgi#148762969%23148762969) | 16.3 | 80% | 1049 |
| [NP_001073881.2](http://www.ncbi.nlm.nih.gov/entrez/query.fcgi?cmd=Retrieve&db=Protein&list_uids=148276990&dopt=GenPept&RID=T2B1WG1R014&log$=prottop&blast_rank=63) | zinc finger and BTB domain containing 38 [Homo sapiens] | [16.3](http://blast.ncbi.nlm.nih.gov/Blast.cgi#148276990%23148276990) | 31.8 | 100% | 1049 |
| [NP_001006608.2](http://www.ncbi.nlm.nih.gov/entrez/query.fcgi?cmd=Retrieve&db=Protein&list_uids=116325993&dopt=GenPept&RID=T2B1WG1R014&log$=prottop&blast_rank=64) | c114 SLIT-like testicular protein precursor [Homo sapiens] | [16.3](http://blast.ncbi.nlm.nih.gov/Blast.cgi#116325993%23116325993) | 16.3 | 80% | 1049 |
| [NP_001002243.1](http://www.ncbi.nlm.nih.gov/entrez/query.fcgi?cmd=Retrieve&db=Protein&list_uids=50409856&dopt=GenPept&RID=T2B1WG1R014&log$=prottop&blast_rank=65) | aftiphilin protein isoform c [Homo sapiens] | [16.3](http://blast.ncbi.nlm.nih.gov/Blast.cgi#50409856%2350409856) | 16.3 | 80% | 1049 |
| [NP_982261.2](http://www.ncbi.nlm.nih.gov/entrez/query.fcgi?cmd=Retrieve&db=Protein&list_uids=50409939&dopt=GenPept&RID=T2B1WG1R014&log$=prottop&blast_rank=66) | aftiphilin protein isoform a [Homo sapiens] | [16.3](http://blast.ncbi.nlm.nih.gov/Blast.cgi#50409939%2350409939) | 16.3 | 80% | 1049 |
| [NP_689668.3](http://www.ncbi.nlm.nih.gov/entrez/query.fcgi?cmd=Retrieve&db=Protein&list_uids=109715825&dopt=GenPept&RID=T2B1WG1R014&log$=prottop&blast_rank=67) | zinc finger protein 690 [Homo sapiens] | [16.3](http://blast.ncbi.nlm.nih.gov/Blast.cgi#109715825%23109715825) | 16.3 | 80% | 1049 |
| [NP_001121682.1](http://www.ncbi.nlm.nih.gov/entrez/query.fcgi?cmd=Retrieve&db=Protein&list_uids=189571671&dopt=GenPept&RID=T2B1WG1R014&log$=prottop&blast_rank=68) | sprouty-related protein with EVH-1 domain 2 isoform b [Homo sapiens] | [16.3](http://blast.ncbi.nlm.nih.gov/Blast.cgi#189571671%23189571671) | 16.3 | 80% | 1049 |
| [NP_861449.2](http://www.ncbi.nlm.nih.gov/entrez/query.fcgi?cmd=Retrieve&db=Protein&list_uids=189571669&dopt=GenPept&RID=T2B1WG1R014&log$=prottop&blast_rank=69) | sprouty-related protein with EVH-1 domain 2 isoform a [Homo sapiens] | [16.3](http://blast.ncbi.nlm.nih.gov/Blast.cgi#189571669%23189571669) | 16.3 | 80% | 1049 |
| [NP_038286.2](http://www.ncbi.nlm.nih.gov/entrez/query.fcgi?cmd=Retrieve&db=Protein&list_uids=95091875&dopt=GenPept&RID=T2B1WG1R014&log$=prottop&blast_rank=70) | Rho GTPase activating protein 6 isoform 1 [Homo sapiens] | [16.3](http://blast.ncbi.nlm.nih.gov/Blast.cgi#95091875%2395091875) | 16.3 | 80% | 1049 |
| [NP_006116.2](http://www.ncbi.nlm.nih.gov/entrez/query.fcgi?cmd=Retrieve&db=Protein&list_uids=95091988&dopt=GenPept&RID=T2B1WG1R014&log$=prottop&blast_rank=71) | Rho GTPase activating protein 6 isoform 3 [Homo sapiens] | [16.3](http://blast.ncbi.nlm.nih.gov/Blast.cgi#95091988%2395091988) | 16.3 | 80% | 1049 |
| [NP_001035500.1](http://www.ncbi.nlm.nih.gov/entrez/query.fcgi?cmd=Retrieve&db=Protein&list_uids=94536860&dopt=GenPept&RID=T2B1WG1R014&log$=prottop&blast_rank=72) | hypothetical protein LOC138724 isoform B [Homo sapiens] | [16.3](http://blast.ncbi.nlm.nih.gov/Blast.cgi#94536860%2394536860) | 16.3 | 80% | 1049 |
| [NP_066015.2](http://www.ncbi.nlm.nih.gov/entrez/query.fcgi?cmd=Retrieve&db=Protein&list_uids=93204865&dopt=GenPept&RID=T2B1WG1R014&log$=prottop&blast_rank=73) | hypothetical protein LOC57724 [Homo sapiens] | [16.3](http://blast.ncbi.nlm.nih.gov/Blast.cgi#93204865%2393204865) | 16.3 | 80% | 1049 |
| [NP_001438.1](http://www.ncbi.nlm.nih.gov/entrez/query.fcgi?cmd=Retrieve&db=Protein&list_uids=13787217&dopt=GenPept&RID=T2B1WG1R014&log$=prottop&blast_rank=74) | FAT tumor suppressor 2 precursor [Homo sapiens] | [16.3](http://blast.ncbi.nlm.nih.gov/Blast.cgi#13787217%2313787217) | 16.3 | 80% | 1049 |
| [NP_001035007.1](http://www.ncbi.nlm.nih.gov/entrez/query.fcgi?cmd=Retrieve&db=Protein&list_uids=90193615&dopt=GenPept&RID=T2B1WG1R014&log$=prottop&blast_rank=75) | nuclear matrix transcription factor 4 isoform b [Homo sapiens] >ref|NP_001035008.1| nuclear matrix transcription factor 4 isoform b [Homo sapiens] | [16.3](http://blast.ncbi.nlm.nih.gov/Blast.cgi#90193615%2390193615) | 16.3 | 80% | 1049 |
| [NP_001035009.1](http://www.ncbi.nlm.nih.gov/entrez/query.fcgi?cmd=Retrieve&db=Protein&list_uids=90193619&dopt=GenPept&RID=T2B1WG1R014&log$=prottop&blast_rank=76) | nuclear matrix transcription factor 4 isoform c [Homo sapiens] | [16.3](http://blast.ncbi.nlm.nih.gov/Blast.cgi#90193619%2390193619) | 16.3 | 80% | 1049 |
| [NP_056099.2](http://www.ncbi.nlm.nih.gov/entrez/query.fcgi?cmd=Retrieve&db=Protein&list_uids=71999153&dopt=GenPept&RID=T2B1WG1R014&log$=prottop&blast_rank=77) | hypothetical protein LOC23334 [Homo sapiens] | [16.3](http://blast.ncbi.nlm.nih.gov/Blast.cgi#71999153%2371999153) | 16.3 | 80% | 1049 |
| [NP_116127.3](http://www.ncbi.nlm.nih.gov/entrez/query.fcgi?cmd=Retrieve&db=Protein&list_uids=69885149&dopt=GenPept&RID=T2B1WG1R014&log$=prottop&blast_rank=78) | Fc receptor-like and mucin-like 1 [Homo sapiens] | [16.3](http://blast.ncbi.nlm.nih.gov/Blast.cgi#69885149%2369885149) | 16.3 | 80% | 1049 |
| [NP_115532.2](http://www.ncbi.nlm.nih.gov/entrez/query.fcgi?cmd=Retrieve&db=Protein&list_uids=50428931&dopt=GenPept&RID=T2B1WG1R014&log$=prottop&blast_rank=79) | C1q domain containing 1 isoform 3 [Homo sapiens] | [16.3](http://blast.ncbi.nlm.nih.gov/Blast.cgi#50428931%2350428931) | 16.3 | 80% | 1049 |
| [NP_001035723.1](http://www.ncbi.nlm.nih.gov/entrez/query.fcgi?cmd=Retrieve&db=Protein&list_uids=100913192&dopt=GenPept&RID=T2B1WG1R014&log$=prottop&blast_rank=80) | AMP-activated protein kinase gamma2 subunit isoform c [Homo sapiens] | [16.3](http://blast.ncbi.nlm.nih.gov/Blast.cgi#100913192%23100913192) | 16.3 | 80% | 1049 |
| [NP_919262.2](http://www.ncbi.nlm.nih.gov/entrez/query.fcgi?cmd=Retrieve&db=Protein&list_uids=50083281&dopt=GenPept&RID=T2B1WG1R014&log$=prottop&blast_rank=81) | KIAA1853 protein [Homo sapiens] | [16.3](http://blast.ncbi.nlm.nih.gov/Blast.cgi#50083281%2350083281) | 16.3 | 80% | 1049 |
| [NP_001002259.1](http://www.ncbi.nlm.nih.gov/entrez/query.fcgi?cmd=Retrieve&db=Protein&list_uids=50428933&dopt=GenPept&RID=T2B1WG1R014&log$=prottop&blast_rank=82) | C1q domain containing 1 isoform 1 [Homo sapiens] | [16.3](http://blast.ncbi.nlm.nih.gov/Blast.cgi#50428933%2350428933) | 16.3 | 80% | 1049 |
| [NP_597733.2](http://www.ncbi.nlm.nih.gov/entrez/query.fcgi?cmd=Retrieve&db=Protein&list_uids=36054194&dopt=GenPept&RID=T2B1WG1R014&log$=prottop&blast_rank=83) | nuclear matrix transcription factor 4 isoform a [Homo sapiens] >ref|NP_001035005.1| nuclear matrix transcription factor 4 isoform a [Homo sapiens] >ref|NP_001035006.1| nuclear matrix transcription factor 4 isoform a [Homo sapiens] | [16.3](http://blast.ncbi.nlm.nih.gov/Blast.cgi#36054194%2336054194) | 16.3 | 80% | 1049 |
| [NP_060594.3](http://www.ncbi.nlm.nih.gov/entrez/query.fcgi?cmd=Retrieve&db=Protein&list_uids=71143112&dopt=GenPept&RID=T2B1WG1R014&log$=prottop&blast_rank=84) | ring finger and WD repeat domain 3 [Homo sapiens] | [16.3](http://blast.ncbi.nlm.nih.gov/Blast.cgi#71143112%2371143112) | 16.3 | 80% | 1049 |
| [NP_061903.2](http://www.ncbi.nlm.nih.gov/entrez/query.fcgi?cmd=Retrieve&db=Protein&list_uids=67782362&dopt=GenPept&RID=T2B1WG1R014&log$=prottop&blast_rank=85) | DEAH (Asp-Glu-Ala-His) box polypeptide 29 [Homo sapiens] | [16.3](http://blast.ncbi.nlm.nih.gov/Blast.cgi#67782362%2367782362) | 16.3 | 80% | 1049 |
| [NP_079523.3](http://www.ncbi.nlm.nih.gov/entrez/query.fcgi?cmd=Retrieve&db=Protein&list_uids=48976061&dopt=GenPept&RID=T2B1WG1R014&log$=prottop&blast_rank=86) | acyl-Coenzyme A dehydrogenase family, member 10 isoform b [Homo sapiens] | [16.3](http://blast.ncbi.nlm.nih.gov/Blast.cgi#48976061%2348976061) | 16.3 | 80% | 1049 |
| [NP_699179.2](http://www.ncbi.nlm.nih.gov/entrez/query.fcgi?cmd=Retrieve&db=Protein&list_uids=30795123&dopt=GenPept&RID=T2B1WG1R014&log$=prottop&blast_rank=87) | F-box and WD repeat domain containing 8 isoform 1 [Homo sapiens] | [16.3](http://blast.ncbi.nlm.nih.gov/Blast.cgi#30795123%2330795123) | 16.3 | 80% | 1049 |
| [NP_036306.1](http://www.ncbi.nlm.nih.gov/entrez/query.fcgi?cmd=Retrieve&db=Protein&list_uids=30795121&dopt=GenPept&RID=T2B1WG1R014&log$=prottop&blast_rank=88) | F-box and WD repeat domain containing 8 isoform 2 [Homo sapiens] | [16.3](http://blast.ncbi.nlm.nih.gov/Blast.cgi#30795121%2330795121) | 16.3 | 80% | 1049 |
| [NP_079439.2](http://www.ncbi.nlm.nih.gov/entrez/query.fcgi?cmd=Retrieve&db=Protein&list_uids=30581137&dopt=GenPept&RID=T2B1WG1R014&log$=prottop&blast_rank=89) | phosphatidylinositol glycan anchor biosynthesis, class Z [Homo sapiens] | [16.3](http://blast.ncbi.nlm.nih.gov/Blast.cgi#30581137%2330581137) | 16.3 | 80% | 1049 |
| [NP_055313.1](http://www.ncbi.nlm.nih.gov/entrez/query.fcgi?cmd=Retrieve&db=Protein&list_uids=7657138&dopt=GenPept&RID=T2B1WG1R014&log$=prottop&blast_rank=90) | golgi integral membrane protein 4 [Homo sapiens] | [16.3](http://blast.ncbi.nlm.nih.gov/Blast.cgi#7657138%237657138) | 16.3 | 80% | 1049 |
| [NP_612373.2](http://www.ncbi.nlm.nih.gov/entrez/query.fcgi?cmd=Retrieve&db=Protein&list_uids=40538772&dopt=GenPept&RID=T2B1WG1R014&log$=prottop&blast_rank=91) | protein arginine methyltransferase 10 (putative) [Homo sapiens] | [16.3](http://blast.ncbi.nlm.nih.gov/Blast.cgi#40538772%2340538772) | 16.3 | 80% | 1049 |
| [NP_002294.2](http://www.ncbi.nlm.nih.gov/entrez/query.fcgi?cmd=Retrieve&db=Protein&list_uids=40254464&dopt=GenPept&RID=T2B1WG1R014&log$=prottop&blast_rank=92) | leptin receptor isoform 1 precursor [Homo sapiens] | [16.3](http://blast.ncbi.nlm.nih.gov/Blast.cgi#40254464%2340254464) | 16.3 | 80% | 1049 |
| [NP_001003680.1](http://www.ncbi.nlm.nih.gov/entrez/query.fcgi?cmd=Retrieve&db=Protein&list_uids=51093381&dopt=GenPept&RID=T2B1WG1R014&log$=prottop&blast_rank=93) | leptin receptor isoform 2 precursor [Homo sapiens] | [16.3](http://blast.ncbi.nlm.nih.gov/Blast.cgi#51093381%2351093381) | 16.3 | 80% | 1049 |
| [NP_001003679.1](http://www.ncbi.nlm.nih.gov/entrez/query.fcgi?cmd=Retrieve&db=Protein&list_uids=51093379&dopt=GenPept&RID=T2B1WG1R014&log$=prottop&blast_rank=94) | leptin receptor isoform 3 precursor [Homo sapiens] | [16.3](http://blast.ncbi.nlm.nih.gov/Blast.cgi#51093379%2351093379) | 16.3 | 80% | 1049 |
| [NP_955372.2](http://www.ncbi.nlm.nih.gov/entrez/query.fcgi?cmd=Retrieve&db=Protein&list_uids=75677612&dopt=GenPept&RID=T2B1WG1R014&log$=prottop&blast_rank=95) | leucine rich repeat containing 37, member A3 precursor [Homo sapiens] | [16.3](http://blast.ncbi.nlm.nih.gov/Blast.cgi#75677612%2375677612) | 16.3 | 80% | 1049 |
| [NP_671726.2](http://www.ncbi.nlm.nih.gov/entrez/query.fcgi?cmd=Retrieve&db=Protein&list_uids=166706915&dopt=GenPept&RID=T2B1WG1R014&log$=prottop&blast_rank=96) | GLIS family zinc finger 1 [Homo sapiens] | [16.3](http://blast.ncbi.nlm.nih.gov/Blast.cgi#166706915%23166706915) | 16.3 | 80% | 1049 |
| [NP_055456.2](http://www.ncbi.nlm.nih.gov/entrez/query.fcgi?cmd=Retrieve&db=Protein&list_uids=132626688&dopt=GenPept&RID=T2B1WG1R014&log$=prottop&blast_rank=97) | mediator of DNA-damage checkpoint 1 [Homo sapiens] | [16.3](http://blast.ncbi.nlm.nih.gov/Blast.cgi#132626688%23132626688) | 16.3 | 80% | 1049 |
| [NP_998820.1](http://www.ncbi.nlm.nih.gov/entrez/query.fcgi?cmd=Retrieve&db=Protein&list_uids=47458036&dopt=GenPept&RID=T2B1WG1R014&log$=prottop&blast_rank=98) | hereditary sensory neuropathy, type II precursor [Homo sapiens] | [16.3](http://blast.ncbi.nlm.nih.gov/Blast.cgi#47458036%2347458036) | 16.3 | 80% | 1049 |
| [NP_001207.2](http://www.ncbi.nlm.nih.gov/entrez/query.fcgi?cmd=Retrieve&db=Protein&list_uids=169636420&dopt=GenPept&RID=T2B1WG1R014&log$=prottop&blast_rank=99) | carbonic anhydrase IX precursor [Homo sapiens] | [16.3](http://blast.ncbi.nlm.nih.gov/Blast.cgi#169636420%23169636420) | 16.3 | 80% | 1049 |
| [NP_942122.2](http://www.ncbi.nlm.nih.gov/entrez/query.fcgi?cmd=Retrieve&db=Protein&list_uids=45433552&dopt=GenPept&RID=T2B1WG1R014&log$=prottop&blast_rank=100) | G protein-coupled receptor 133 precursor [Homo sapiens] | [16.3](http://blast.ncbi.nlm.nih.gov/Blast.cgi#45433552%2345433552) | 16.3 | 80% | 1049 |

| **Accession** | **Proteins with a match to ASHQNRP peptide** | **[Max score](http://blast.ncbi.nlm.nih.gov/Blast.cgi?CMD=Get&ALIGNMENTS=100&ALIGNMENT_VIEW=Pairwise&CDD_SEARCH_STATE=1&DATABASE_SORT=0&DESCRIPTIONS=100&ENTREZ_QUERY=txid9606 %5BORGN%5D&FIRST_QUERY_NUM=0&FORMAT_OBJECT=Alignment&FORMAT_PAGE_TARGET=&FORMAT_TYPE=HTML&GET_SEQUENCE=yes&I_THRESH=&MASK_CHAR=2&MASK_COLOR=1&NEW_DESIGN=on&NEW_VIEW=yes&NUM_OVERVIEW=100&OLD_BLAST=false&PAGE=Proteins&QUERY_INDEX=0&QUERY_NUMBER=0&RESULTS_PAGE_TARGET=&RID=T0DBANU301N&SHOW_LINKOUT=yes&SHOW_OVERVIEW=yes&STEP_NUMBER=&WORD_SIZE=2&DISPLAY_SORT=1&HSP_SORT=1" \l "sort_mark)** | **[Total score](http://blast.ncbi.nlm.nih.gov/Blast.cgi?CMD=Get&ALIGNMENTS=100&ALIGNMENT_VIEW=Pairwise&CDD_SEARCH_STATE=1&DATABASE_SORT=0&DESCRIPTIONS=100&ENTREZ_QUERY=txid9606 %5BORGN%5D&FIRST_QUERY_NUM=0&FORMAT_OBJECT=Alignment&FORMAT_PAGE_TARGET=&FORMAT_TYPE=HTML&GET_SEQUENCE=yes&I_THRESH=&MASK_CHAR=2&MASK_COLOR=1&NEW_DESIGN=on&NEW_VIEW=yes&NUM_OVERVIEW=100&OLD_BLAST=false&PAGE=Proteins&QUERY_INDEX=0&QUERY_NUMBER=0&RESULTS_PAGE_TARGET=&RID=T0DBANU301N&SHOW_LINKOUT=yes&SHOW_OVERVIEW=yes&STEP_NUMBER=&WORD_SIZE=2&DISPLAY_SORT=2&HSP_SORT=1" \l "sort_mark)** | **[Query coverage](http://blast.ncbi.nlm.nih.gov/Blast.cgi?CMD=Get&ALIGNMENTS=100&ALIGNMENT_VIEW=Pairwise&CDD_SEARCH_STATE=1&DATABASE_SORT=0&DESCRIPTIONS=100&ENTREZ_QUERY=txid9606 %5BORGN%5D&FIRST_QUERY_NUM=0&FORMAT_OBJECT=Alignment&FORMAT_PAGE_TARGET=&FORMAT_TYPE=HTML&GET_SEQUENCE=yes&I_THRESH=&MASK_CHAR=2&MASK_COLOR=1&NEW_DESIGN=on&NEW_VIEW=yes&NUM_OVERVIEW=100&OLD_BLAST=false&PAGE=Proteins&QUERY_INDEX=0&QUERY_NUMBER=0&RESULTS_PAGE_TARGET=&RID=T0DBANU301N&SHOW_LINKOUT=yes&SHOW_OVERVIEW=yes&STEP_NUMBER=&WORD_SIZE=2&DISPLAY_SORT=4&HSP_SORT=0" \l "sort_mark)** | **[E value](http://blast.ncbi.nlm.nih.gov/Blast.cgi?CMD=Get&ALIGNMENTS=100&ALIGNMENT_VIEW=Pairwise&CDD_SEARCH_STATE=1&DATABASE_SORT=0&DESCRIPTIONS=100&ENTREZ_QUERY=txid9606 %5BORGN%5D&FIRST_QUERY_NUM=0&FORMAT_OBJECT=Alignment&FORMAT_PAGE_TARGET=&FORMAT_TYPE=HTML&GET_SEQUENCE=yes&I_THRESH=&MASK_CHAR=2&MASK_COLOR=1&NEW_DESIGN=on&NEW_VIEW=yes&NUM_OVERVIEW=100&OLD_BLAST=false&PAGE=Proteins&QUERY_INDEX=0&QUERY_NUMBER=0&RESULTS_PAGE_TARGET=&RID=T0DBANU301N&SHOW_LINKOUT=yes&SHOW_OVERVIEW=yes&STEP_NUMBER=&WORD_SIZE=2&DISPLAY_SORT=0&HSP_SORT=0" \l "sort_mark)** |
| --- | --- | --- | --- | --- | --- |
| [NP_653170.3](http://www.ncbi.nlm.nih.gov/entrez/query.fcgi?cmd=Retrieve&db=Protein&list_uids=47271475&dopt=GenPept&RID=T0DBANU301N&log$=prottop&blast_rank=1) | SPOC domain containing 1 [Homo sapiens] | [20.6](http://blast.ncbi.nlm.nih.gov/Blast.cgi#47271475%2347271475) | 20.6 | 85% | 78 |
| [NP_113584.3](http://www.ncbi.nlm.nih.gov/entrez/query.fcgi?cmd=Retrieve&db=Protein&list_uids=61676188&dopt=GenPept&RID=T0DBANU301N&log$=prottop&blast_rank=2) | HECT, UBA and WWE domain containing 1 [Homo sapiens] | [20.2](http://blast.ncbi.nlm.nih.gov/Blast.cgi#61676188%2361676188) | 20.2 | 85% | 104 |
| [NP_001077059.1](http://www.ncbi.nlm.nih.gov/entrez/query.fcgi?cmd=Retrieve&db=Protein&list_uids=134152683&dopt=GenPept&RID=T0DBANU301N&log$=prottop&blast_rank=3) | transmembrane protein 214 isoform 2 [Homo sapiens] | [19.7](http://blast.ncbi.nlm.nih.gov/Blast.cgi#134152683%23134152683) | 19.7 | 85% | 140 |
| [NP_060197.4](http://www.ncbi.nlm.nih.gov/entrez/query.fcgi?cmd=Retrieve&db=Protein&list_uids=134152721&dopt=GenPept&RID=T0DBANU301N&log$=prottop&blast_rank=4) | transmembrane protein 214 isoform 1 [Homo sapiens] | [19.7](http://blast.ncbi.nlm.nih.gov/Blast.cgi#134152721%23134152721) | 19.7 | 85% | 140 |
| [NP_689769.2](http://www.ncbi.nlm.nih.gov/entrez/query.fcgi?cmd=Retrieve&db=Protein&list_uids=116812632&dopt=GenPept&RID=T0DBANU301N&log$=prottop&blast_rank=5) | hypothetical protein LOC154743 [Homo sapiens] | [19.7](http://blast.ncbi.nlm.nih.gov/Blast.cgi#116812632%23116812632) | 19.7 | 71% | 140 |
| [NP_003325.2](http://www.ncbi.nlm.nih.gov/entrez/query.fcgi?cmd=Retrieve&db=Protein&list_uids=23510338&dopt=GenPept&RID=T0DBANU301N&log$=prottop&blast_rank=6) | ubiquitin-activating enzyme E1 [Homo sapiens] >ref|NP_695012.1| ubiquitin-activating enzyme E1 [Homo sapiens] | [19.7](http://blast.ncbi.nlm.nih.gov/Blast.cgi#23510338%2323510338) | 19.7 | 71% | 140 |
| [NP_775837.2](http://www.ncbi.nlm.nih.gov/entrez/query.fcgi?cmd=Retrieve&db=Protein&list_uids=209969819&dopt=GenPept&RID=T0DBANU301N&log$=prottop&blast_rank=7) | hypothetical protein LOC253143 [Homo sapiens] | [19.3](http://blast.ncbi.nlm.nih.gov/Blast.cgi#209969819%23209969819) | 19.3 | 85% | 188 |
| [NP_057511.2](http://www.ncbi.nlm.nih.gov/entrez/query.fcgi?cmd=Retrieve&db=Protein&list_uids=45439357&dopt=GenPept&RID=T0DBANU301N&log$=prottop&blast_rank=8) | elongin A2 [Homo sapiens] | [19.3](http://blast.ncbi.nlm.nih.gov/Blast.cgi#45439357%2345439357) | 19.3 | 85% | 188 |
| [NP_848658.3](http://www.ncbi.nlm.nih.gov/entrez/query.fcgi?cmd=Retrieve&db=Protein&list_uids=222352158&dopt=GenPept&RID=T0DBANU301N&log$=prottop&blast_rank=9) | carboxypeptidase 3, cytosolic [Homo sapiens] | [18.9](http://blast.ncbi.nlm.nih.gov/Blast.cgi#222352158%23222352158) | 18.9 | 71% | 252 |
| [NP_001033729.2](http://www.ncbi.nlm.nih.gov/entrez/query.fcgi?cmd=Retrieve&db=Protein&list_uids=146133848&dopt=GenPept&RID=T0DBANU301N&log$=prottop&blast_rank=10) | golgi autoantigen, golgin subfamily a, 6 [Homo sapiens] | [18.9](http://blast.ncbi.nlm.nih.gov/Blast.cgi#146133848%23146133848) | 18.9 | 71% | 252 |
| [NP_055524.3](http://www.ncbi.nlm.nih.gov/entrez/query.fcgi?cmd=Retrieve&db=Protein&list_uids=110347427&dopt=GenPept&RID=T0DBANU301N&log$=prottop&blast_rank=11) | ubiquitin specific protease 34 [Homo sapiens] | [18.9](http://blast.ncbi.nlm.nih.gov/Blast.cgi#110347427%23110347427) | 18.9 | 71% | 252 |
| [NP_059115.1](http://www.ncbi.nlm.nih.gov/entrez/query.fcgi?cmd=Retrieve&db=Protein&list_uids=31442761&dopt=GenPept&RID=T0DBANU301N&log$=prottop&blast_rank=12) | amiloride-sensitive cation channel 5, intestinal [Homo sapiens] | [18.9](http://blast.ncbi.nlm.nih.gov/Blast.cgi#31442761%2331442761) | 18.9 | 71% | 252 |
| [NP_631938.1](http://www.ncbi.nlm.nih.gov/entrez/query.fcgi?cmd=Retrieve&db=Protein&list_uids=34452707&dopt=GenPept&RID=T0DBANU301N&log$=prottop&blast_rank=13) | bromodomain containing 8 isoform 2 [Homo sapiens] | [18.9](http://blast.ncbi.nlm.nih.gov/Blast.cgi#34452707%2334452707) | 18.9 | 71% | 252 |
| [NP_001154825.1](http://www.ncbi.nlm.nih.gov/entrez/query.fcgi?cmd=Retrieve&db=Protein&list_uids=238624132&dopt=GenPept&RID=T0DBANU301N&log$=prottop&blast_rank=14) | large conductance calcium-activated potassium channel subfamily M alpha member 1 isoform d [Homo sapiens] | [18.5](http://blast.ncbi.nlm.nih.gov/Blast.cgi#238624132%23238624132) | 18.5 | 100% | 338 |
| [NP_001014797.1](http://www.ncbi.nlm.nih.gov/entrez/query.fcgi?cmd=Retrieve&db=Protein&list_uids=62388890&dopt=GenPept&RID=T0DBANU301N&log$=prottop&blast_rank=15) | large conductance calcium-activated potassium channel subfamily M alpha member 1 isoform a [Homo sapiens] | [18.5](http://blast.ncbi.nlm.nih.gov/Blast.cgi#62388890%2362388890) | 18.5 | 100% | 338 |
| [NP_001013049.1](http://www.ncbi.nlm.nih.gov/entrez/query.fcgi?cmd=Retrieve&db=Protein&list_uids=61743975&dopt=GenPept&RID=T0DBANU301N&log$=prottop&blast_rank=16) | SORCS receptor 1 isoform b [Homo sapiens] | [18.5](http://blast.ncbi.nlm.nih.gov/Blast.cgi#61743975%2361743975) | 18.5 | 85% | 338 |
| [NP_001154824.1](http://www.ncbi.nlm.nih.gov/entrez/query.fcgi?cmd=Retrieve&db=Protein&list_uids=238624130&dopt=GenPept&RID=T0DBANU301N&log$=prottop&blast_rank=17) | large conductance calcium-activated potassium channel subfamily M alpha member 1 isoform c [Homo sapiens] | [18.5](http://blast.ncbi.nlm.nih.gov/Blast.cgi#238624130%23238624130) | 18.5 | 100% | 338 |
| [NP_443150.3](http://www.ncbi.nlm.nih.gov/entrez/query.fcgi?cmd=Retrieve&db=Protein&list_uids=61743973&dopt=GenPept&RID=T0DBANU301N&log$=prottop&blast_rank=18) | SORCS receptor 1 isoform a [Homo sapiens] | [18.5](http://blast.ncbi.nlm.nih.gov/Blast.cgi#61743973%2361743973) | 18.5 | 85% | 338 |
| [NP_005602.3](http://www.ncbi.nlm.nih.gov/entrez/query.fcgi?cmd=Retrieve&db=Protein&list_uids=172072597&dopt=GenPept&RID=T0DBANU301N&log$=prottop&blast_rank=19) | retinoblastoma-like 2 (p130) [Homo sapiens] | [18.5](http://blast.ncbi.nlm.nih.gov/Blast.cgi#172072597%23172072597) | 18.5 | 85% | 338 |
| [NP_006525.2](http://www.ncbi.nlm.nih.gov/entrez/query.fcgi?cmd=Retrieve&db=Protein&list_uids=32307124&dopt=GenPept&RID=T0DBANU301N&log$=prottop&blast_rank=20) | nuclear receptor coactivator 3 isoform b [Homo sapiens] | [18.5](http://blast.ncbi.nlm.nih.gov/Blast.cgi#32307124%2332307124) | 33.9 | 100% | 338 |
| [NP_002238.2](http://www.ncbi.nlm.nih.gov/entrez/query.fcgi?cmd=Retrieve&db=Protein&list_uids=26638650&dopt=GenPept&RID=T0DBANU301N&log$=prottop&blast_rank=21) | large conductance calcium-activated potassium channel subfamily M alpha member 1 isoform b [Homo sapiens] | [18.5](http://blast.ncbi.nlm.nih.gov/Blast.cgi#26638650%2326638650) | 18.5 | 100% | 338 |
| [NP_858045.1](http://www.ncbi.nlm.nih.gov/entrez/query.fcgi?cmd=Retrieve&db=Protein&list_uids=32307126&dopt=GenPept&RID=T0DBANU301N&log$=prottop&blast_rank=22) | nuclear receptor coactivator 3 isoform a [Homo sapiens] | [18.5](http://blast.ncbi.nlm.nih.gov/Blast.cgi#32307126%2332307126) | 33.9 | 100% | 338 |
| [NP_001161081.1](http://www.ncbi.nlm.nih.gov/entrez/query.fcgi?cmd=Retrieve&db=Protein&list_uids=263190680&dopt=GenPept&RID=T0DBANU301N&log$=prottop&blast_rank=23) | GLI family zinc finger 1 isoform 3 [Homo sapiens] | [18.0](http://blast.ncbi.nlm.nih.gov/Blast.cgi#263190680%23263190680) | 32.2 | 100% | 453 |
| [NP_001159438.1](http://www.ncbi.nlm.nih.gov/entrez/query.fcgi?cmd=Retrieve&db=Protein&list_uids=260166647&dopt=GenPept&RID=T0DBANU301N&log$=prottop&blast_rank=24) | PITPNM family member 3 isoform 2 [Homo sapiens] | [18.0](http://blast.ncbi.nlm.nih.gov/Blast.cgi#260166647%23260166647) | 28.0 | 100% | 453 |
| [NP_001153517.1](http://www.ncbi.nlm.nih.gov/entrez/query.fcgi?cmd=Retrieve&db=Protein&list_uids=229892345&dopt=GenPept&RID=T0DBANU301N&log$=prottop&blast_rank=25) | GLI family zinc finger 1 isoform 2 [Homo sapiens] | [18.0](http://blast.ncbi.nlm.nih.gov/Blast.cgi#229892345%23229892345) | 32.2 | 100% | 453 |
| [NP_112497.2](http://www.ncbi.nlm.nih.gov/entrez/query.fcgi?cmd=Retrieve&db=Protein&list_uids=190358515&dopt=GenPept&RID=T0DBANU301N&log$=prottop&blast_rank=26) | PITPNM family member 3 isoform 1 [Homo sapiens] | [18.0](http://blast.ncbi.nlm.nih.gov/Blast.cgi#190358515%23190358515) | 28.0 | 100% | 453 |
| [NP_001157164.1](http://www.ncbi.nlm.nih.gov/entrez/query.fcgi?cmd=Retrieve&db=Protein&list_uids=254939613&dopt=GenPept&RID=T0DBANU301N&log$=prottop&blast_rank=27) | hypothetical protein LOC390595 [Homo sapiens] | [18.0](http://blast.ncbi.nlm.nih.gov/Blast.cgi#254939613%23254939613) | 18.0 | 100% | 453 |
| [NP_005261.2](http://www.ncbi.nlm.nih.gov/entrez/query.fcgi?cmd=Retrieve&db=Protein&list_uids=86991432&dopt=GenPept&RID=T0DBANU301N&log$=prottop&blast_rank=28) | GLI-Kruppel family member GLI2 [Homo sapiens] | [18.0](http://blast.ncbi.nlm.nih.gov/Blast.cgi#86991432%2386991432) | 18.0 | 85% | 453 |
| [NP_115820.2](http://www.ncbi.nlm.nih.gov/entrez/query.fcgi?cmd=Retrieve&db=Protein&list_uids=63252863&dopt=GenPept&RID=T0DBANU301N&log$=prottop&blast_rank=29) | BTB (POZ) domain containing 12 [Homo sapiens] | [18.0](http://blast.ncbi.nlm.nih.gov/Blast.cgi#63252863%2363252863) | 28.0 | 85% | 453 |
| [NP_115927.1](http://www.ncbi.nlm.nih.gov/entrez/query.fcgi?cmd=Retrieve&db=Protein&list_uids=58761548&dopt=GenPept&RID=T0DBANU301N&log$=prottop&blast_rank=30) | tau tubulin kinase 1 [Homo sapiens] | [18.0](http://blast.ncbi.nlm.nih.gov/Blast.cgi#58761548%2358761548) | 18.0 | 85% | 453 |
| [NP_005260.1](http://www.ncbi.nlm.nih.gov/entrez/query.fcgi?cmd=Retrieve&db=Protein&list_uids=4885279&dopt=GenPept&RID=T0DBANU301N&log$=prottop&blast_rank=31) | GLI family zinc finger 1 isoform 1 [Homo sapiens] | [18.0](http://blast.ncbi.nlm.nih.gov/Blast.cgi#4885279%234885279) | 32.2 | 100% | 453 |
| [NP_001005237.1](http://www.ncbi.nlm.nih.gov/entrez/query.fcgi?cmd=Retrieve&db=Protein&list_uids=52546693&dopt=GenPept&RID=T0DBANU301N&log$=prottop&blast_rank=32) | olfactory receptor, family 51, subfamily G, member 1 [Homo sapiens] | [18.0](http://blast.ncbi.nlm.nih.gov/Blast.cgi#52546693%2352546693) | 18.0 | 85% | 453 |
| [NP_000159.3](http://www.ncbi.nlm.nih.gov/entrez/query.fcgi?cmd=Retrieve&db=Protein&list_uids=119393899&dopt=GenPept&RID=T0DBANU301N&log$=prottop&blast_rank=33) | GLI-Kruppel family member GLI3 [Homo sapiens] | [18.0](http://blast.ncbi.nlm.nih.gov/Blast.cgi#119393899%23119393899) | 18.0 | 85% | 453 |
| [NP_001123541.1](http://www.ncbi.nlm.nih.gov/entrez/query.fcgi?cmd=Retrieve&db=Protein&list_uids=194248090&dopt=GenPept&RID=T0DBANU301N&log$=prottop&blast_rank=34) | sulfatase modifying factor 2 isoform e precursor [Homo sapiens] | [17.6](http://blast.ncbi.nlm.nih.gov/Blast.cgi#194248090%23194248090) | 17.6 | 85% | 608 |
| [NP_055602.1](http://www.ncbi.nlm.nih.gov/entrez/query.fcgi?cmd=Retrieve&db=Protein&list_uids=7662146&dopt=GenPept&RID=T0DBANU301N&log$=prottop&blast_rank=35) | DnaJ (Hsp40) homolog, subfamily C, member 6 [Homo sapiens] | [17.6](http://blast.ncbi.nlm.nih.gov/Blast.cgi#7662146%237662146) | 17.6 | 85% | 608 |
| [NP_775893.2](http://www.ncbi.nlm.nih.gov/entrez/query.fcgi?cmd=Retrieve&db=Protein&list_uids=82617663&dopt=GenPept&RID=T0DBANU301N&log$=prottop&blast_rank=36) | CMT1A duplicated region transcript 4 [Homo sapiens] | [17.6](http://blast.ncbi.nlm.nih.gov/Blast.cgi#82617663%2382617663) | 17.6 | 100% | 608 |
| [NP_060413.2](http://www.ncbi.nlm.nih.gov/entrez/query.fcgi?cmd=Retrieve&db=Protein&list_uids=40807493&dopt=GenPept&RID=T0DBANU301N&log$=prottop&blast_rank=37) | F-box only protein 34 [Homo sapiens] >ref|NP_689417.1| F-box only protein 34 [Homo sapiens] | [17.6](http://blast.ncbi.nlm.nih.gov/Blast.cgi#40807493%2340807493) | 17.6 | 85% | 608 |
| [XP_002346012.1](http://www.ncbi.nlm.nih.gov/entrez/query.fcgi?cmd=Retrieve&db=Protein&list_uids=239754516&dopt=GenPept&RID=T0DBANU301N&log$=prottop&blast_rank=38) | PREDICTED: hypothetical protein [Homo sapiens] | [17.2](http://blast.ncbi.nlm.nih.gov/Blast.cgi#239754516%23239754516) | 17.2 | 100% | 816 |
| [XP_001721879.2](http://www.ncbi.nlm.nih.gov/entrez/query.fcgi?cmd=Retrieve&db=Protein&list_uids=239753362&dopt=GenPept&RID=T0DBANU301N&log$=prottop&blast_rank=39) | PREDICTED: hypothetical protein [Homo sapiens] | [17.2](http://blast.ncbi.nlm.nih.gov/Blast.cgi#239753362%23239753362) | 17.2 | 57% | 816 |
| [XP_002346898.1](http://www.ncbi.nlm.nih.gov/entrez/query.fcgi?cmd=Retrieve&db=Protein&list_uids=239749077&dopt=GenPept&RID=T0DBANU301N&log$=prottop&blast_rank=40) | PREDICTED: hypothetical protein XP_002346898 [Homo sapiens] | [17.2](http://blast.ncbi.nlm.nih.gov/Blast.cgi#239749077%23239749077) | 17.2 | 100% | 816 |
| [XP_001721897.2](http://www.ncbi.nlm.nih.gov/entrez/query.fcgi?cmd=Retrieve&db=Protein&list_uids=239741824&dopt=GenPept&RID=T0DBANU301N&log$=prottop&blast_rank=41) | PREDICTED: hypothetical protein [Homo sapiens] >ref|XP_001725806.2| PREDICTED: hypothetical protein [Homo sapiens] | [17.2](http://blast.ncbi.nlm.nih.gov/Blast.cgi#239741824%23239741824) | 17.2 | 57% | 816 |
| [XP_002344340.1](http://www.ncbi.nlm.nih.gov/entrez/query.fcgi?cmd=Retrieve&db=Protein&list_uids=239508758&dopt=GenPept&RID=T0DBANU301N&log$=prottop&blast_rank=42) | PREDICTED: hypothetical protein [Homo sapiens] >ref|XP_002342755.1| PREDICTED: hypothetical protein XP_002342755 [Homo sapiens] | [17.2](http://blast.ncbi.nlm.nih.gov/Blast.cgi#239508758%23239508758) | 17.2 | 100% | 816 |
| [NP_001165911.1](http://www.ncbi.nlm.nih.gov/entrez/query.fcgi?cmd=Retrieve&db=Protein&list_uids=289063435&dopt=GenPept&RID=T0DBANU301N&log$=prottop&blast_rank=43) | endonuclease, polyU-specific isoform 3 precursor [Homo sapiens] | [17.2](http://blast.ncbi.nlm.nih.gov/Blast.cgi#289063435%23289063435) | 17.2 | 57% | 816 |
| [XP_001714312.1](http://www.ncbi.nlm.nih.gov/entrez/query.fcgi?cmd=Retrieve&db=Protein&list_uids=169177031&dopt=GenPept&RID=T0DBANU301N&log$=prottop&blast_rank=44) | PREDICTED: similar to hCG30082, partial [Homo sapiens] | [17.2](http://blast.ncbi.nlm.nih.gov/Blast.cgi#169177031%23169177031) | 17.2 | 71% | 816 |
| [NP_001129610.1](http://www.ncbi.nlm.nih.gov/entrez/query.fcgi?cmd=Retrieve&db=Protein&list_uids=209915591&dopt=GenPept&RID=T0DBANU301N&log$=prottop&blast_rank=45) | ribosomal protein S6 kinase, 52kDa, polypeptide 1 isoform b [Homo sapiens] | [17.2](http://blast.ncbi.nlm.nih.gov/Blast.cgi#209915591%23209915591) | 17.2 | 100% | 816 |
| [NP_001124396.2](http://www.ncbi.nlm.nih.gov/entrez/query.fcgi?cmd=Retrieve&db=Protein&list_uids=262399371&dopt=GenPept&RID=T0DBANU301N&log$=prottop&blast_rank=46) | transmembrane protein 201 isoform 1 [Homo sapiens] | [17.2](http://blast.ncbi.nlm.nih.gov/Blast.cgi#262399371%23262399371) | 17.2 | 57% | 816 |
| [NP_001004441.2](http://www.ncbi.nlm.nih.gov/entrez/query.fcgi?cmd=Retrieve&db=Protein&list_uids=257467559&dopt=GenPept&RID=T0DBANU301N&log$=prottop&blast_rank=47) | ankyrin repeat domain 34B [Homo sapiens] | [17.2](http://blast.ncbi.nlm.nih.gov/Blast.cgi#257467559%23257467559) | 17.2 | 57% | 816 |
| [NP_079185.2](http://www.ncbi.nlm.nih.gov/entrez/query.fcgi?cmd=Retrieve&db=Protein&list_uids=186910323&dopt=GenPept&RID=T0DBANU301N&log$=prottop&blast_rank=48) | hypothetical protein LOC79969 isoform 2 [Homo sapiens] | [17.2](http://blast.ncbi.nlm.nih.gov/Blast.cgi#186910323%23186910323) | 17.2 | 71% | 816 |
| [NP_660281.2](http://www.ncbi.nlm.nih.gov/entrez/query.fcgi?cmd=Retrieve&db=Protein&list_uids=148596977&dopt=GenPept&RID=T0DBANU301N&log$=prottop&blast_rank=49) | zinc finger protein 31 [Homo sapiens] | [17.2](http://blast.ncbi.nlm.nih.gov/Blast.cgi#148596977%23148596977) | 17.2 | 57% | 816 |
| [NP_003913.3](http://www.ncbi.nlm.nih.gov/entrez/query.fcgi?cmd=Retrieve&db=Protein&list_uids=126131099&dopt=GenPept&RID=T0DBANU301N&log$=prottop&blast_rank=50) | hect domain and RCC1-like domain 1 [Homo sapiens] | [17.2](http://blast.ncbi.nlm.nih.gov/Blast.cgi#126131099%23126131099) | 17.2 | 57% | 816 |
| [NP_065986.2](http://www.ncbi.nlm.nih.gov/entrez/query.fcgi?cmd=Retrieve&db=Protein&list_uids=188497648&dopt=GenPept&RID=T0DBANU301N&log$=prottop&blast_rank=51) | ubiquitin specific peptidase 37 [Homo sapiens] | [17.2](http://blast.ncbi.nlm.nih.gov/Blast.cgi#188497648%23188497648) | 17.2 | 57% | 816 |
| [NP_001106853.1](http://www.ncbi.nlm.nih.gov/entrez/query.fcgi?cmd=Retrieve&db=Protein&list_uids=164607133&dopt=GenPept&RID=T0DBANU301N&log$=prottop&blast_rank=52) | fer-1-like 5 isoform 2 [Homo sapiens] | [17.2](http://blast.ncbi.nlm.nih.gov/Blast.cgi#164607133%23164607133) | 17.2 | 57% | 816 |
| [NP_001121693.1](http://www.ncbi.nlm.nih.gov/entrez/query.fcgi?cmd=Retrieve&db=Protein&list_uids=190014597&dopt=GenPept&RID=T0DBANU301N&log$=prottop&blast_rank=53) | vestigial like 4 isoform d [Homo sapiens] | [17.2](http://blast.ncbi.nlm.nih.gov/Blast.cgi#190014597%23190014597) | 17.2 | 71% | 816 |
| [NP_001121692.1](http://www.ncbi.nlm.nih.gov/entrez/query.fcgi?cmd=Retrieve&db=Protein&list_uids=190014595&dopt=GenPept&RID=T0DBANU301N&log$=prottop&blast_rank=54) | vestigial like 4 isoform c [Homo sapiens] | [17.2](http://blast.ncbi.nlm.nih.gov/Blast.cgi#190014595%23190014595) | 17.2 | 71% | 816 |
| [NP_001165910.1](http://www.ncbi.nlm.nih.gov/entrez/query.fcgi?cmd=Retrieve&db=Protein&list_uids=289063433&dopt=GenPept&RID=T0DBANU301N&log$=prottop&blast_rank=55) | endonuclease, polyU-specific isoform 1 precursor [Homo sapiens] | [17.2](http://blast.ncbi.nlm.nih.gov/Blast.cgi#289063433%23289063433) | 17.2 | 57% | 816 |
| [NP_055482.2](http://www.ncbi.nlm.nih.gov/entrez/query.fcgi?cmd=Retrieve&db=Protein&list_uids=190014591&dopt=GenPept&RID=T0DBANU301N&log$=prottop&blast_rank=56) | vestigial like 4 isoform b [Homo sapiens] | [17.2](http://blast.ncbi.nlm.nih.gov/Blast.cgi#190014591%23190014591) | 17.2 | 71% | 816 |
| [NP_001121691.1](http://www.ncbi.nlm.nih.gov/entrez/query.fcgi?cmd=Retrieve&db=Protein&list_uids=190014593&dopt=GenPept&RID=T0DBANU301N&log$=prottop&blast_rank=57) | vestigial like 4 isoform a [Homo sapiens] | [17.2](http://blast.ncbi.nlm.nih.gov/Blast.cgi#190014593%23190014593) | 17.2 | 71% | 816 |
| [NP_001073278.1](http://www.ncbi.nlm.nih.gov/entrez/query.fcgi?cmd=Retrieve&db=Protein&list_uids=119372315&dopt=GenPept&RID=T0DBANU301N&log$=prottop&blast_rank=58) | xin actin-binding repeat containing 2 isoform 2 [Homo sapiens] | [17.2](http://blast.ncbi.nlm.nih.gov/Blast.cgi#119372315%23119372315) | 17.2 | 57% | 816 |
| [NP_689594.4](http://www.ncbi.nlm.nih.gov/entrez/query.fcgi?cmd=Retrieve&db=Protein&list_uids=119372317&dopt=GenPept&RID=T0DBANU301N&log$=prottop&blast_rank=59) | xin actin-binding repeat containing 2 isoform 1 [Homo sapiens] | [17.2](http://blast.ncbi.nlm.nih.gov/Blast.cgi#119372317%23119372317) | 17.2 | 57% | 816 |
| [NP_001074011.1](http://www.ncbi.nlm.nih.gov/entrez/query.fcgi?cmd=Retrieve&db=Protein&list_uids=122937382&dopt=GenPept&RID=T0DBANU301N&log$=prottop&blast_rank=60) | Fas (TNFRSF6) binding factor 1 [Homo sapiens] | [17.2](http://blast.ncbi.nlm.nih.gov/Blast.cgi#122937382%23122937382) | 17.2 | 57% | 816 |
| [NP_055935.4](http://www.ncbi.nlm.nih.gov/entrez/query.fcgi?cmd=Retrieve&db=Protein&list_uids=110349786&dopt=GenPept&RID=T0DBANU301N&log$=prottop&blast_rank=61) | Alstrom syndrome 1 [Homo sapiens] | [17.2](http://blast.ncbi.nlm.nih.gov/Blast.cgi#110349786%23110349786) | 17.2 | 57% | 816 |
| [NP_056493.3](http://www.ncbi.nlm.nih.gov/entrez/query.fcgi?cmd=Retrieve&db=Protein&list_uids=62422577&dopt=GenPept&RID=T0DBANU301N&log$=prottop&blast_rank=62) | neurobeachin [Homo sapiens] | [17.2](http://blast.ncbi.nlm.nih.gov/Blast.cgi#62422577%2362422577) | 17.2 | 57% | 816 |
| [NP_005581.2](http://www.ncbi.nlm.nih.gov/entrez/query.fcgi?cmd=Retrieve&db=Protein&list_uids=24234690&dopt=GenPept&RID=T0DBANU301N&log$=prottop&blast_rank=63) | meiotic recombination 11 homolog A isoform 2 [Homo sapiens] | [17.2](http://blast.ncbi.nlm.nih.gov/Blast.cgi#24234690%2324234690) | 17.2 | 57% | 816 |
| [NP_005488.2](http://www.ncbi.nlm.nih.gov/entrez/query.fcgi?cmd=Retrieve&db=Protein&list_uids=69122473&dopt=GenPept&RID=T0DBANU301N&log$=prottop&blast_rank=64) | connexin 45 [Homo sapiens] >ref|NP_001073852.1| connexin 45 [Homo sapiens] | [17.2](http://blast.ncbi.nlm.nih.gov/Blast.cgi#69122473%2369122473) | 17.2 | 85% | 816 |
| [NP_001018126.1](http://www.ncbi.nlm.nih.gov/entrez/query.fcgi?cmd=Retrieve&db=Protein&list_uids=66472922&dopt=GenPept&RID=T0DBANU301N&log$=prottop&blast_rank=65) | muscle-related coiled-coil protein [Homo sapiens] | [17.2](http://blast.ncbi.nlm.nih.gov/Blast.cgi#66472922%2366472922) | 17.2 | 57% | 816 |
| [NP_821075.1](http://www.ncbi.nlm.nih.gov/entrez/query.fcgi?cmd=Retrieve&db=Protein&list_uids=41281907&dopt=GenPept&RID=T0DBANU301N&log$=prottop&blast_rank=66) | StAR-related lipid transfer (START) domain containing 13 isoform beta [Homo sapiens] | [17.2](http://blast.ncbi.nlm.nih.gov/Blast.cgi#41281907%2341281907) | 17.2 | 85% | 816 |
| [NP_116162.1](http://www.ncbi.nlm.nih.gov/entrez/query.fcgi?cmd=Retrieve&db=Protein&list_uids=14249428&dopt=GenPept&RID=T0DBANU301N&log$=prottop&blast_rank=67) | leucine-rich repeats and calponin homology (CH) domain containing 3 [Homo sapiens] | [17.2](http://blast.ncbi.nlm.nih.gov/Blast.cgi#14249428%2314249428) | 17.2 | 57% | 816 |
| [NP_821074.1](http://www.ncbi.nlm.nih.gov/entrez/query.fcgi?cmd=Retrieve&db=Protein&list_uids=41281898&dopt=GenPept&RID=T0DBANU301N&log$=prottop&blast_rank=68) | StAR-related lipid transfer (START) domain containing 13 isoform alpha [Homo sapiens] | [17.2](http://blast.ncbi.nlm.nih.gov/Blast.cgi#41281898%2341281898) | 17.2 | 85% | 816 |
| [NP_055387.2](http://www.ncbi.nlm.nih.gov/entrez/query.fcgi?cmd=Retrieve&db=Protein&list_uids=126507091&dopt=GenPept&RID=T0DBANU301N&log$=prottop&blast_rank=69) | LATS, large tumor suppressor, homolog 2 [Homo sapiens] | [17.2](http://blast.ncbi.nlm.nih.gov/Blast.cgi#126507091%23126507091) | 17.2 | 85% | 816 |
| [NP_001010866.1](http://www.ncbi.nlm.nih.gov/entrez/query.fcgi?cmd=Retrieve&db=Protein&list_uids=58197554&dopt=GenPept&RID=T0DBANU301N&log$=prottop&blast_rank=70) | transmembrane protein 201 isoform 2 [Homo sapiens] | [17.2](http://blast.ncbi.nlm.nih.gov/Blast.cgi#58197554%2358197554) | 17.2 | 57% | 816 |
| [NP_938014.1](http://www.ncbi.nlm.nih.gov/entrez/query.fcgi?cmd=Retrieve&db=Protein&list_uids=84370276&dopt=GenPept&RID=T0DBANU301N&log$=prottop&blast_rank=71) | LysM, putative peptidoglycan-binding, domain containing 3 [Homo sapiens] | [17.2](http://blast.ncbi.nlm.nih.gov/Blast.cgi#84370276%2384370276) | 17.2 | 57% | 816 |
| [NP_053586.1](http://www.ncbi.nlm.nih.gov/entrez/query.fcgi?cmd=Retrieve&db=Protein&list_uids=7669532&dopt=GenPept&RID=T0DBANU301N&log$=prottop&blast_rank=72) | neuregulin 2 isoform 4 [Homo sapiens] | [17.2](http://blast.ncbi.nlm.nih.gov/Blast.cgi#7669532%237669532) | 17.2 | 57% | 816 |
| [NP_671766.1](http://www.ncbi.nlm.nih.gov/entrez/query.fcgi?cmd=Retrieve&db=Protein&list_uids=22538459&dopt=GenPept&RID=T0DBANU301N&log$=prottop&blast_rank=73) | nuclear receptor coactivator 1 isoform 3 [Homo sapiens] | [17.2](http://blast.ncbi.nlm.nih.gov/Blast.cgi#22538459%2322538459) | 28.0 | 85% | 816 |
| [NP_053585.1](http://www.ncbi.nlm.nih.gov/entrez/query.fcgi?cmd=Retrieve&db=Protein&list_uids=7669530&dopt=GenPept&RID=T0DBANU301N&log$=prottop&blast_rank=74) | neuregulin 2 isoform 3 [Homo sapiens] | [17.2](http://blast.ncbi.nlm.nih.gov/Blast.cgi#7669530%237669530) | 17.2 | 57% | 816 |
| [NP_053584.1](http://www.ncbi.nlm.nih.gov/entrez/query.fcgi?cmd=Retrieve&db=Protein&list_uids=7669528&dopt=GenPept&RID=T0DBANU301N&log$=prottop&blast_rank=75) | neuregulin 2 isoform 2 [Homo sapiens] | [17.2](http://blast.ncbi.nlm.nih.gov/Blast.cgi#7669528%237669528) | 17.2 | 57% | 816 |
| [NP_001122387.1](http://www.ncbi.nlm.nih.gov/entrez/query.fcgi?cmd=Retrieve&db=Protein&list_uids=193083117&dopt=GenPept&RID=T0DBANU301N&log$=prottop&blast_rank=76) | cytochrome P450 family 24 subfamily A polypeptide 1 isoform 2 precursor [Homo sapiens] | [17.2](http://blast.ncbi.nlm.nih.gov/Blast.cgi#193083117%23193083117) | 17.2 | 57% | 816 |
| [NP_932076.1](http://www.ncbi.nlm.nih.gov/entrez/query.fcgi?cmd=Retrieve&db=Protein&list_uids=37574614&dopt=GenPept&RID=T0DBANU301N&log$=prottop&blast_rank=77) | nucleoside diphosphate kinase 7 isoform b [Homo sapiens] | [17.2](http://blast.ncbi.nlm.nih.gov/Blast.cgi#37574614%2337574614) | 17.2 | 71% | 816 |
| [NP_689808.2](http://www.ncbi.nlm.nih.gov/entrez/query.fcgi?cmd=Retrieve&db=Protein&list_uids=25777746&dopt=GenPept&RID=T0DBANU301N&log$=prottop&blast_rank=78) | piggyBac transposable element derived 4 [Homo sapiens] | [17.2](http://blast.ncbi.nlm.nih.gov/Blast.cgi#25777746%2325777746) | 17.2 | 57% | 816 |
| [NP_055790.1](http://www.ncbi.nlm.nih.gov/entrez/query.fcgi?cmd=Retrieve&db=Protein&list_uids=45120119&dopt=GenPept&RID=T0DBANU301N&log$=prottop&blast_rank=79) | microtubule associated serine/threonine kinase 1 [Homo sapiens] | [17.2](http://blast.ncbi.nlm.nih.gov/Blast.cgi#45120119%2345120119) | 17.2 | 100% | 816 |
| [NP_036411.1](http://www.ncbi.nlm.nih.gov/entrez/query.fcgi?cmd=Retrieve&db=Protein&list_uids=6912440&dopt=GenPept&RID=T0DBANU301N&log$=prottop&blast_rank=80) | zinc finger protein 346 [Homo sapiens] | [17.2](http://blast.ncbi.nlm.nih.gov/Blast.cgi#6912440%236912440) | 17.2 | 57% | 816 |
| [NP_689670.1](http://www.ncbi.nlm.nih.gov/entrez/query.fcgi?cmd=Retrieve&db=Protein&list_uids=22748967&dopt=GenPept&RID=T0DBANU301N&log$=prottop&blast_rank=81) | zinc finger protein 597 [Homo sapiens] | [17.2](http://blast.ncbi.nlm.nih.gov/Blast.cgi#22748967%2322748967) | 17.2 | 85% | 816 |
| [NP_036556.2](http://www.ncbi.nlm.nih.gov/entrez/query.fcgi?cmd=Retrieve&db=Protein&list_uids=19923723&dopt=GenPept&RID=T0DBANU301N&log$=prottop&blast_rank=82) | ribosomal protein S6 kinase, 52kDa, polypeptide 1 isoform a [Homo sapiens] | [17.2](http://blast.ncbi.nlm.nih.gov/Blast.cgi#19923723%2319923723) | 17.2 | 100% | 816 |
| [NP_004874.1](http://www.ncbi.nlm.nih.gov/entrez/query.fcgi?cmd=Retrieve&db=Protein&list_uids=4758832&dopt=GenPept&RID=T0DBANU301N&log$=prottop&blast_rank=83) | neuregulin 2 isoform 1 [Homo sapiens] | [17.2](http://blast.ncbi.nlm.nih.gov/Blast.cgi#4758832%234758832) | 17.2 | 57% | 816 |
| [NP_037462.1](http://www.ncbi.nlm.nih.gov/entrez/query.fcgi?cmd=Retrieve&db=Protein&list_uids=7019465&dopt=GenPept&RID=T0DBANU301N&log$=prottop&blast_rank=84) | nucleoside diphosphate kinase 7 isoform a [Homo sapiens] | [17.2](http://blast.ncbi.nlm.nih.gov/Blast.cgi#7019465%237019465) | 17.2 | 71% | 816 |
| [NP_000244.2](http://www.ncbi.nlm.nih.gov/entrez/query.fcgi?cmd=Retrieve&db=Protein&list_uids=153285408&dopt=GenPept&RID=T0DBANU301N&log$=prottop&blast_rank=85) | microsomal triglyceride transfer protein large subunit precursor [Homo sapiens] | [17.2](http://blast.ncbi.nlm.nih.gov/Blast.cgi#153285408%23153285408) | 17.2 | 57% | 816 |
| [NP_443083.1](http://www.ncbi.nlm.nih.gov/entrez/query.fcgi?cmd=Retrieve&db=Protein&list_uids=16445031&dopt=GenPept&RID=T0DBANU301N&log$=prottop&blast_rank=86) | StAR-related lipid transfer (START) domain containing 13 isoform gamma [Homo sapiens] | [17.2](http://blast.ncbi.nlm.nih.gov/Blast.cgi#16445031%2316445031) | 17.2 | 85% | 816 |
| [NP_005582.1](http://www.ncbi.nlm.nih.gov/entrez/query.fcgi?cmd=Retrieve&db=Protein&list_uids=5031923&dopt=GenPept&RID=T0DBANU301N&log$=prottop&blast_rank=87) | meiotic recombination 11 homolog A isoform 1 [Homo sapiens] | [17.2](http://blast.ncbi.nlm.nih.gov/Blast.cgi#5031923%235031923) | 17.2 | 57% | 816 |
| [NP_056481.1](http://www.ncbi.nlm.nih.gov/entrez/query.fcgi?cmd=Retrieve&db=Protein&list_uids=24308117&dopt=GenPept&RID=T0DBANU301N&log$=prottop&blast_rank=88) | GTP binding protein 5 [Homo sapiens] | [17.2](http://blast.ncbi.nlm.nih.gov/Blast.cgi#24308117%2324308117) | 17.2 | 57% | 816 |
| [NP_003734.3](http://www.ncbi.nlm.nih.gov/entrez/query.fcgi?cmd=Retrieve&db=Protein&list_uids=22538455&dopt=GenPept&RID=T0DBANU301N&log$=prottop&blast_rank=89) | nuclear receptor coactivator 1 isoform 1 [Homo sapiens] | [17.2](http://blast.ncbi.nlm.nih.gov/Blast.cgi#22538455%2322538455) | 28.0 | 85% | 816 |
| [NP_000773.2](http://www.ncbi.nlm.nih.gov/entrez/query.fcgi?cmd=Retrieve&db=Protein&list_uids=55770850&dopt=GenPept&RID=T0DBANU301N&log$=prottop&blast_rank=90) | cytochrome P450 family 24 subfamily A polypeptide 1 isoform 1 precursor [Homo sapiens] | [17.2](http://blast.ncbi.nlm.nih.gov/Blast.cgi#55770850%2355770850) | 17.2 | 57% | 816 |
| [NP_757384.1](http://www.ncbi.nlm.nih.gov/entrez/query.fcgi?cmd=Retrieve&db=Protein&list_uids=27437008&dopt=GenPept&RID=T0DBANU301N&log$=prottop&blast_rank=91) | kringle-containing transmembrane protein 2 isoform d precursor [Homo sapiens] | [17.2](http://blast.ncbi.nlm.nih.gov/Blast.cgi#27437008%2327437008) | 17.2 | 57% | 816 |
| [NP_075380.1](http://www.ncbi.nlm.nih.gov/entrez/query.fcgi?cmd=Retrieve&db=Protein&list_uids=13194201&dopt=GenPept&RID=T0DBANU301N&log$=prottop&blast_rank=92) | reticulon 4 receptor precursor [Homo sapiens] | [17.2](http://blast.ncbi.nlm.nih.gov/Blast.cgi#13194201%2313194201) | 17.2 | 57% | 816 |
| [NP_671756.1](http://www.ncbi.nlm.nih.gov/entrez/query.fcgi?cmd=Retrieve&db=Protein&list_uids=22538457&dopt=GenPept&RID=T0DBANU301N&log$=prottop&blast_rank=93) | nuclear receptor coactivator 1 isoform 2 [Homo sapiens] | [17.2](http://blast.ncbi.nlm.nih.gov/Blast.cgi#22538457%2322538457) | 28.0 | 85% | 816 |
| [NP_597728.1](http://www.ncbi.nlm.nih.gov/entrez/query.fcgi?cmd=Retrieve&db=Protein&list_uids=145386517&dopt=GenPept&RID=T0DBANU301N&log$=prottop&blast_rank=94) | phostensin [Homo sapiens] >ref|NP_001128342.1| phostensin [Homo sapiens] | [17.2](http://blast.ncbi.nlm.nih.gov/Blast.cgi#145386517%23145386517) | 17.2 | 57% | 816 |
| [NP_078783.1](http://www.ncbi.nlm.nih.gov/entrez/query.fcgi?cmd=Retrieve&db=Protein&list_uids=13375642&dopt=GenPept&RID=T0DBANU301N&log$=prottop&blast_rank=95) | kringle-containing transmembrane protein 2 isoform b precursor [Homo sapiens] | [17.2](http://blast.ncbi.nlm.nih.gov/Blast.cgi#13375642%2313375642) | 17.2 | 57% | 816 |
| [NP_065881.1](http://www.ncbi.nlm.nih.gov/entrez/query.fcgi?cmd=Retrieve&db=Protein&list_uids=18482373&dopt=GenPept&RID=T0DBANU301N&log$=prottop&blast_rank=96) | WD repeat and FYVE domain containing 1 [Homo sapiens] | [17.2](http://blast.ncbi.nlm.nih.gov/Blast.cgi#18482373%2318482373) | 17.2 | 57% | 816 |
| [NP_006016.1](http://www.ncbi.nlm.nih.gov/entrez/query.fcgi?cmd=Retrieve&db=Protein&list_uids=5174623&dopt=GenPept&RID=T0DBANU301N&log$=prottop&blast_rank=97) | endonuclease, polyU-specific isoform 2 precursor [Homo sapiens] | [17.2](http://blast.ncbi.nlm.nih.gov/Blast.cgi#5174623%235174623) | 17.2 | 57% | 816 |
| [NP_112214.1](http://www.ncbi.nlm.nih.gov/entrez/query.fcgi?cmd=Retrieve&db=Protein&list_uids=13569922&dopt=GenPept&RID=T0DBANU301N&log$=prottop&blast_rank=98) | NUAK family, SNF1-like kinase, 2 [Homo sapiens] | [17.2](http://blast.ncbi.nlm.nih.gov/Blast.cgi#13569922%2313569922) | 17.2 | 57% | 816 |
| [NP_001158508.1](http://www.ncbi.nlm.nih.gov/entrez/query.fcgi?cmd=Retrieve&db=Protein&list_uids=259013553&dopt=GenPept&RID=T0DBANU301N&log$=prottop&blast_rank=99) | oxoglutarate dehydrogenase isoform 3 precursor [Homo sapiens] | [16.8](http://blast.ncbi.nlm.nih.gov/Blast.cgi#259013553%23259013553) | 16.8 | 57% | 1094 |
| [XP_002345162.1](http://www.ncbi.nlm.nih.gov/entrez/query.fcgi?cmd=Retrieve&db=Protein&list_uids=239757085&dopt=GenPept&RID=T0DBANU301N&log$=prottop&blast_rank=100) | PREDICTED: hypothetical protein [Homo sapiens] | [16.8](http://blast.ncbi.nlm.nih.gov/Blast.cgi#239757085%23239757085) | 16.8 | 57% | 1094 |

| **Accession** | **Proteins with a match to FRQAAS peptide** | **[Max score](http://blast.ncbi.nlm.nih.gov/Blast.cgi?CMD=Get&ALIGNMENTS=100&ALIGNMENT_VIEW=Pairwise&CDD_SEARCH_STATE=1&DATABASE_SORT=0&DESCRIPTIONS=100&ENTREZ_QUERY=txid9606 %5BORGN%5D&FIRST_QUERY_NUM=0&FORMAT_OBJECT=Alignment&FORMAT_PAGE_TARGET=&FORMAT_TYPE=HTML&GET_SEQUENCE=yes&I_THRESH=&MASK_CHAR=2&MASK_COLOR=1&NEW_DESIGN=on&NEW_VIEW=yes&NUM_OVERVIEW=100&OLD_BLAST=false&PAGE=Proteins&QUERY_INDEX=0&QUERY_NUMBER=0&RESULTS_PAGE_TARGET=&RID=T0DFMR3T01N&SHOW_LINKOUT=yes&SHOW_OVERVIEW=yes&STEP_NUMBER=&WORD_SIZE=2&DISPLAY_SORT=1&HSP_SORT=1" \l "sort_mark)** | **[Total score](http://blast.ncbi.nlm.nih.gov/Blast.cgi?CMD=Get&ALIGNMENTS=100&ALIGNMENT_VIEW=Pairwise&CDD_SEARCH_STATE=1&DATABASE_SORT=0&DESCRIPTIONS=100&ENTREZ_QUERY=txid9606 %5BORGN%5D&FIRST_QUERY_NUM=0&FORMAT_OBJECT=Alignment&FORMAT_PAGE_TARGET=&FORMAT_TYPE=HTML&GET_SEQUENCE=yes&I_THRESH=&MASK_CHAR=2&MASK_COLOR=1&NEW_DESIGN=on&NEW_VIEW=yes&NUM_OVERVIEW=100&OLD_BLAST=false&PAGE=Proteins&QUERY_INDEX=0&QUERY_NUMBER=0&RESULTS_PAGE_TARGET=&RID=T0DFMR3T01N&SHOW_LINKOUT=yes&SHOW_OVERVIEW=yes&STEP_NUMBER=&WORD_SIZE=2&DISPLAY_SORT=2&HSP_SORT=1" \l "sort_mark)** | **[Query coverage](http://blast.ncbi.nlm.nih.gov/Blast.cgi?CMD=Get&ALIGNMENTS=100&ALIGNMENT_VIEW=Pairwise&CDD_SEARCH_STATE=1&DATABASE_SORT=0&DESCRIPTIONS=100&ENTREZ_QUERY=txid9606 %5BORGN%5D&FIRST_QUERY_NUM=0&FORMAT_OBJECT=Alignment&FORMAT_PAGE_TARGET=&FORMAT_TYPE=HTML&GET_SEQUENCE=yes&I_THRESH=&MASK_CHAR=2&MASK_COLOR=1&NEW_DESIGN=on&NEW_VIEW=yes&NUM_OVERVIEW=100&OLD_BLAST=false&PAGE=Proteins&QUERY_INDEX=0&QUERY_NUMBER=0&RESULTS_PAGE_TARGET=&RID=T0DFMR3T01N&SHOW_LINKOUT=yes&SHOW_OVERVIEW=yes&STEP_NUMBER=&WORD_SIZE=2&DISPLAY_SORT=4&HSP_SORT=0" \l "sort_mark)** | **[E value](http://blast.ncbi.nlm.nih.gov/Blast.cgi?CMD=Get&ALIGNMENTS=100&ALIGNMENT_VIEW=Pairwise&CDD_SEARCH_STATE=1&DATABASE_SORT=0&DESCRIPTIONS=100&ENTREZ_QUERY=txid9606 %5BORGN%5D&FIRST_QUERY_NUM=0&FORMAT_OBJECT=Alignment&FORMAT_PAGE_TARGET=&FORMAT_TYPE=HTML&GET_SEQUENCE=yes&I_THRESH=&MASK_CHAR=2&MASK_COLOR=1&NEW_DESIGN=on&NEW_VIEW=yes&NUM_OVERVIEW=100&OLD_BLAST=false&PAGE=Proteins&QUERY_INDEX=0&QUERY_NUMBER=0&RESULTS_PAGE_TARGET=&RID=T0DFMR3T01N&SHOW_LINKOUT=yes&SHOW_OVERVIEW=yes&STEP_NUMBER=&WORD_SIZE=2&DISPLAY_SORT=0&HSP_SORT=0" \l "sort_mark)** |
| --- | --- | --- | --- | --- | --- |
| [NP_002138.1](http://www.ncbi.nlm.nih.gov/entrez/query.fcgi?cmd=Retrieve&db=Protein&list_uids=4504469&dopt=GenPept&RID=T0DFMR3T01N&log$=prottop&blast_rank=1) | homeobox B5 [Homo sapiens] | [21.4](http://blast.ncbi.nlm.nih.gov/Blast.cgi#4504469%234504469) | 21.4 | 100% | 37 |
| [NP_000567.1](http://www.ncbi.nlm.nih.gov/entrez/query.fcgi?cmd=Retrieve&db=Protein&list_uids=10835145&dopt=GenPept&RID=T0DFMR3T01N&log$=prottop&blast_rank=2) | interleukin 1, beta proprotein [Homo sapiens] | [21.4](http://blast.ncbi.nlm.nih.gov/Blast.cgi#10835145%2310835145) | 21.4 | 100% | 37 |
| [NP_037493.3](http://www.ncbi.nlm.nih.gov/entrez/query.fcgi?cmd=Retrieve&db=Protein&list_uids=156547039&dopt=GenPept&RID=T0DFMR3T01N&log$=prottop&blast_rank=3) | zinc finger protein 223 [Homo sapiens] | [18.9](http://blast.ncbi.nlm.nih.gov/Blast.cgi#156547039%23156547039) | 18.9 | 100% | 216 |
| [NP_061198.2](http://www.ncbi.nlm.nih.gov/entrez/query.fcgi?cmd=Retrieve&db=Protein&list_uids=153945715&dopt=GenPept&RID=T0DFMR3T01N&log$=prottop&blast_rank=4) | myosin VC [Homo sapiens] | [18.0](http://blast.ncbi.nlm.nih.gov/Blast.cgi#153945715%23153945715) | 18.0 | 100% | 388 |
| [NP_078928.3](http://www.ncbi.nlm.nih.gov/entrez/query.fcgi?cmd=Retrieve&db=Protein&list_uids=90991702&dopt=GenPept&RID=T0DFMR3T01N&log$=prottop&blast_rank=5) | leucine-rich repeat kinase 1 [Homo sapiens] | [18.0](http://blast.ncbi.nlm.nih.gov/Blast.cgi#90991702%2390991702) | 33.1 | 100% | 388 |
| [NP_714964.2](http://www.ncbi.nlm.nih.gov/entrez/query.fcgi?cmd=Retrieve&db=Protein&list_uids=25453487&dopt=GenPept&RID=T0DFMR3T01N&log$=prottop&blast_rank=6) | cystathionase isoform 2 [Homo sapiens] | [18.0](http://blast.ncbi.nlm.nih.gov/Blast.cgi#25453487%2325453487) | 18.0 | 100% | 388 |
| [NP_001001557.1](http://www.ncbi.nlm.nih.gov/entrez/query.fcgi?cmd=Retrieve&db=Protein&list_uids=48475062&dopt=GenPept&RID=T0DFMR3T01N&log$=prottop&blast_rank=7) | growth differentiation factor 6 precursor [Homo sapiens] | [18.0](http://blast.ncbi.nlm.nih.gov/Blast.cgi#48475062%2348475062) | 18.0 | 100% | 388 |
| [NP_001893.2](http://www.ncbi.nlm.nih.gov/entrez/query.fcgi?cmd=Retrieve&db=Protein&list_uids=21361334&dopt=GenPept&RID=T0DFMR3T01N&log$=prottop&blast_rank=8) | cystathionase isoform 1 [Homo sapiens] | [18.0](http://blast.ncbi.nlm.nih.gov/Blast.cgi#21361334%2321361334) | 18.0 | 100% | 388 |
| [NP_079133.3](http://www.ncbi.nlm.nih.gov/entrez/query.fcgi?cmd=Retrieve&db=Protein&list_uids=26080431&dopt=GenPept&RID=T0DFMR3T01N&log$=prottop&blast_rank=9) | ATPase family, AAA domain containing 5 [Homo sapiens] | [18.0](http://blast.ncbi.nlm.nih.gov/Blast.cgi#26080431%2326080431) | 18.0 | 100% | 388 |
| [NP_001157980.1](http://www.ncbi.nlm.nih.gov/entrez/query.fcgi?cmd=Retrieve&db=Protein&list_uids=257743025&dopt=GenPept&RID=T0DFMR3T01N&log$=prottop&blast_rank=10) | nebulin isoform 2 [Homo sapiens] | [17.6](http://blast.ncbi.nlm.nih.gov/Blast.cgi#257743025%23257743025) | 17.6 | 83% | 521 |
| [NP_001157979.1](http://www.ncbi.nlm.nih.gov/entrez/query.fcgi?cmd=Retrieve&db=Protein&list_uids=257743023&dopt=GenPept&RID=T0DFMR3T01N&log$=prottop&blast_rank=11) | nebulin isoform 1 [Homo sapiens] | [17.6](http://blast.ncbi.nlm.nih.gov/Blast.cgi#257743023%23257743023) | 17.6 | 83% | 521 |
| [XP_002348149.1](http://www.ncbi.nlm.nih.gov/entrez/query.fcgi?cmd=Retrieve&db=Protein&list_uids=239752220&dopt=GenPept&RID=T0DFMR3T01N&log$=prottop&blast_rank=12) | PREDICTED: hypothetical protein XP_002348149 [Homo sapiens] | [17.6](http://blast.ncbi.nlm.nih.gov/Blast.cgi#239752220%23239752220) | 17.6 | 83% | 521 |
| [XP_002343822.1](http://www.ncbi.nlm.nih.gov/entrez/query.fcgi?cmd=Retrieve&db=Protein&list_uids=239746741&dopt=GenPept&RID=T0DFMR3T01N&log$=prottop&blast_rank=13) | PREDICTED: hypothetical protein XP_002343822 [Homo sapiens] | [17.6](http://blast.ncbi.nlm.nih.gov/Blast.cgi#239746741%23239746741) | 17.6 | 83% | 521 |
| [NP_001070995.1](http://www.ncbi.nlm.nih.gov/entrez/query.fcgi?cmd=Retrieve&db=Protein&list_uids=117938295&dopt=GenPept&RID=T0DFMR3T01N&log$=prottop&blast_rank=14) | jerky isoform b [Homo sapiens] | [17.6](http://blast.ncbi.nlm.nih.gov/Blast.cgi#117938295%23117938295) | 17.6 | 83% | 521 |
| [NP_003715.2](http://www.ncbi.nlm.nih.gov/entrez/query.fcgi?cmd=Retrieve&db=Protein&list_uids=117938293&dopt=GenPept&RID=T0DFMR3T01N&log$=prottop&blast_rank=15) | jerky isoform a [Homo sapiens] | [17.6](http://blast.ncbi.nlm.nih.gov/Blast.cgi#117938293%23117938293) | 17.6 | 83% | 521 |
| [NP_004534.2](http://www.ncbi.nlm.nih.gov/entrez/query.fcgi?cmd=Retrieve&db=Protein&list_uids=115527120&dopt=GenPept&RID=T0DFMR3T01N&log$=prottop&blast_rank=16) | nebulin isoform 3 [Homo sapiens] | [17.6](http://blast.ncbi.nlm.nih.gov/Blast.cgi#115527120%23115527120) | 17.6 | 83% | 521 |
| [NP_056127.2](http://www.ncbi.nlm.nih.gov/entrez/query.fcgi?cmd=Retrieve&db=Protein&list_uids=150378498&dopt=GenPept&RID=T0DFMR3T01N&log$=prottop&blast_rank=17) | fragile site-associated protein [Homo sapiens] | [17.6](http://blast.ncbi.nlm.nih.gov/Blast.cgi#150378498%23150378498) | 17.6 | 83% | 521 |
| [NP_056308.3](http://www.ncbi.nlm.nih.gov/entrez/query.fcgi?cmd=Retrieve&db=Protein&list_uids=258613875&dopt=GenPept&RID=T0DFMR3T01N&log$=prottop&blast_rank=18) | ankyrin repeat domain 25 isoform 1 [Homo sapiens] | [17.6](http://blast.ncbi.nlm.nih.gov/Blast.cgi#258613875%23258613875) | 17.6 | 83% | 521 |
| [NP_001093326.2](http://www.ncbi.nlm.nih.gov/entrez/query.fcgi?cmd=Retrieve&db=Protein&list_uids=223671870&dopt=GenPept&RID=T0DFMR3T01N&log$=prottop&blast_rank=19) | inhibitor of kappa light polypeptide gene enhancer in B-cells, kinase gamma isoform b [Homo sapiens] | [17.6](http://blast.ncbi.nlm.nih.gov/Blast.cgi#223671870%23223671870) | 17.6 | 83% | 521 |
| [NP_001159574.1](http://www.ncbi.nlm.nih.gov/entrez/query.fcgi?cmd=Retrieve&db=Protein&list_uids=260656005&dopt=GenPept&RID=T0DFMR3T01N&log$=prottop&blast_rank=20) | NADH dehydrogenase ubiquinone flavoprotein 1 isoform 2 precursor [Homo sapiens] | [17.6](http://blast.ncbi.nlm.nih.gov/Blast.cgi#260656005%23260656005) | 17.6 | 83% | 521 |
| [NP_001003811.1](http://www.ncbi.nlm.nih.gov/entrez/query.fcgi?cmd=Retrieve&db=Protein&list_uids=51477721&dopt=GenPept&RID=T0DFMR3T01N&log$=prottop&blast_rank=21) | testis expressed sequence 11 isoform 1 [Homo sapiens] | [17.6](http://blast.ncbi.nlm.nih.gov/Blast.cgi#51477721%2351477721) | 17.6 | 83% | 521 |
| [NP_112566.2](http://www.ncbi.nlm.nih.gov/entrez/query.fcgi?cmd=Retrieve&db=Protein&list_uids=51477723&dopt=GenPept&RID=T0DFMR3T01N&log$=prottop&blast_rank=22) | testis expressed sequence 11 isoform 2 [Homo sapiens] | [17.6](http://blast.ncbi.nlm.nih.gov/Blast.cgi#51477723%2351477723) | 17.6 | 83% | 521 |
| [NP_001032221.1](http://www.ncbi.nlm.nih.gov/entrez/query.fcgi?cmd=Retrieve&db=Protein&list_uids=80861396&dopt=GenPept&RID=T0DFMR3T01N&log$=prottop&blast_rank=23) | centrobin, centrosomal BRCA2 interacting protein isoform beta [Homo sapiens] | [17.6](http://blast.ncbi.nlm.nih.gov/Blast.cgi#80861396%2380861396) | 17.6 | 83% | 521 |
| [NP_001129663.1](http://www.ncbi.nlm.nih.gov/entrez/query.fcgi?cmd=Retrieve&db=Protein&list_uids=209969812&dopt=GenPept&RID=T0DFMR3T01N&log$=prottop&blast_rank=24) | ankyrin repeat domain 25 isoform 2 [Homo sapiens] | [17.6](http://blast.ncbi.nlm.nih.gov/Blast.cgi#209969812%23209969812) | 17.6 | 83% | 521 |
| [NP_003630.1](http://www.ncbi.nlm.nih.gov/entrez/query.fcgi?cmd=Retrieve&db=Protein&list_uids=4504631&dopt=GenPept&RID=T0DFMR3T01N&log$=prottop&blast_rank=25) | inhibitor of kappa light polypeptide gene enhancer in B-cells, kinase gamma isoform a [Homo sapiens] >ref|NP_001093327.1| inhibitor of kappa light polypeptide gene enhancer in B-cells, kinase gamma isoform a [Homo sapiens] | [17.6](http://blast.ncbi.nlm.nih.gov/Blast.cgi#4504631%234504631) | 17.6 | 83% | 521 |
| [NP_073568.2](http://www.ncbi.nlm.nih.gov/entrez/query.fcgi?cmd=Retrieve&db=Protein&list_uids=56118310&dopt=GenPept&RID=T0DFMR3T01N&log$=prottop&blast_rank=26) | nuclear casein kinase and cyclin-dependent kinase substrate 1 [Homo sapiens] | [17.6](http://blast.ncbi.nlm.nih.gov/Blast.cgi#56118310%2356118310) | 17.6 | 83% | 521 |
| [NP_444279.2](http://www.ncbi.nlm.nih.gov/entrez/query.fcgi?cmd=Retrieve&db=Protein&list_uids=191252785&dopt=GenPept&RID=T0DFMR3T01N&log$=prottop&blast_rank=27) | centrobin, centrosomal BRCA2 interacting protein isoform alpha [Homo sapiens] | [17.6](http://blast.ncbi.nlm.nih.gov/Blast.cgi#191252785%23191252785) | 17.6 | 83% | 521 |
| [NP_004125.3](http://www.ncbi.nlm.nih.gov/entrez/query.fcgi?cmd=Retrieve&db=Protein&list_uids=24234688&dopt=GenPept&RID=T0DFMR3T01N&log$=prottop&blast_rank=28) | heat shock 70kDa protein 9 precursor [Homo sapiens] | [17.6](http://blast.ncbi.nlm.nih.gov/Blast.cgi#24234688%2324234688) | 17.6 | 83% | 521 |
| [NP_078812.2](http://www.ncbi.nlm.nih.gov/entrez/query.fcgi?cmd=Retrieve&db=Protein&list_uids=27545323&dopt=GenPept&RID=T0DFMR3T01N&log$=prottop&blast_rank=29) | chondroitin polymerizing factor [Homo sapiens] | [17.6](http://blast.ncbi.nlm.nih.gov/Blast.cgi#27545323%2327545323) | 17.6 | 83% | 521 |
| [NP_009034.2](http://www.ncbi.nlm.nih.gov/entrez/query.fcgi?cmd=Retrieve&db=Protein&list_uids=20149568&dopt=GenPept&RID=T0DFMR3T01N&log$=prottop&blast_rank=30) | NADH dehydrogenase ubiquinone flavoprotein 1 isoform 1 precursor [Homo sapiens] | [17.6](http://blast.ncbi.nlm.nih.gov/Blast.cgi#20149568%2320149568) | 17.6 | 83% | 521 |
| [NP_078884.2](http://www.ncbi.nlm.nih.gov/entrez/query.fcgi?cmd=Retrieve&db=Protein&list_uids=205360991&dopt=GenPept&RID=T0DFMR3T01N&log$=prottop&blast_rank=31) | nei endonuclease VIII-like 1 [Homo sapiens] | [17.6](http://blast.ncbi.nlm.nih.gov/Blast.cgi#205360991%23205360991) | 17.6 | 83% | 521 |
| [NP_071436.1](http://www.ncbi.nlm.nih.gov/entrez/query.fcgi?cmd=Retrieve&db=Protein&list_uids=62339432&dopt=GenPept&RID=T0DFMR3T01N&log$=prottop&blast_rank=32) | platelet receptor Gi24 precursor [Homo sapiens] | [17.6](http://blast.ncbi.nlm.nih.gov/Blast.cgi#62339432%2362339432) | 17.6 | 83% | 521 |
| [NP_001157962.1](http://www.ncbi.nlm.nih.gov/entrez/query.fcgi?cmd=Retrieve&db=Protein&list_uids=256773272&dopt=GenPept&RID=T0DFMR3T01N&log$=prottop&blast_rank=33) | ADAM metallopeptidase domain 8 isoform 3 precursor [Homo sapiens] | [17.2](http://blast.ncbi.nlm.nih.gov/Blast.cgi#256773272%23256773272) | 17.2 | 100% | 699 |
| [NP_001100.3](http://www.ncbi.nlm.nih.gov/entrez/query.fcgi?cmd=Retrieve&db=Protein&list_uids=256773264&dopt=GenPept&RID=T0DFMR3T01N&log$=prottop&blast_rank=34) | ADAM metallopeptidase domain 8 isoform 1 precursor [Homo sapiens] | [17.2](http://blast.ncbi.nlm.nih.gov/Blast.cgi#256773264%23256773264) | 17.2 | 100% | 699 |
| [NP_001001668.3](http://www.ncbi.nlm.nih.gov/entrez/query.fcgi?cmd=Retrieve&db=Protein&list_uids=154091003&dopt=GenPept&RID=T0DFMR3T01N&log$=prottop&blast_rank=35) | zinc finger protein 470 [Homo sapiens] | [17.2](http://blast.ncbi.nlm.nih.gov/Blast.cgi#154091003%23154091003) | 33.1 | 100% | 699 |
| [NP_997297.2](http://www.ncbi.nlm.nih.gov/entrez/query.fcgi?cmd=Retrieve&db=Protein&list_uids=148727311&dopt=GenPept&RID=T0DFMR3T01N&log$=prottop&blast_rank=36) | hypothetical protein LOC389690 [Homo sapiens] | [17.2](http://blast.ncbi.nlm.nih.gov/Blast.cgi#148727311%23148727311) | 17.2 | 100% | 699 |
| [NP_073600.3](http://www.ncbi.nlm.nih.gov/entrez/query.fcgi?cmd=Retrieve&db=Protein&list_uids=205360932&dopt=GenPept&RID=T0DFMR3T01N&log$=prottop&blast_rank=37) | fibronectin type III domain containing 3B [Homo sapiens] >ref|NP_001128567.1| fibronectin type III domain containing 3B [Homo sapiens] | [17.2](http://blast.ncbi.nlm.nih.gov/Blast.cgi#205360932%23205360932) | 43.5 | 100% | 699 |
| [NP_076968.2](http://www.ncbi.nlm.nih.gov/entrez/query.fcgi?cmd=Retrieve&db=Protein&list_uids=229577436&dopt=GenPept&RID=T0DFMR3T01N&log$=prottop&blast_rank=38) | spermatogenesis associated 5-like 1 [Homo sapiens] | [17.2](http://blast.ncbi.nlm.nih.gov/Blast.cgi#229577436%23229577436) | 17.2 | 100% | 699 |
| [NP_006411.2](http://www.ncbi.nlm.nih.gov/entrez/query.fcgi?cmd=Retrieve&db=Protein&list_uids=150417986&dopt=GenPept&RID=T0DFMR3T01N&log$=prottop&blast_rank=39) | ADP-ribosylation factor guanine nucleotide-exchange factor 2 [Homo sapiens] | [17.2](http://blast.ncbi.nlm.nih.gov/Blast.cgi#150417986%23150417986) | 30.1 | 100% | 699 |
| [NP_068587.1](http://www.ncbi.nlm.nih.gov/entrez/query.fcgi?cmd=Retrieve&db=Protein&list_uids=11141885&dopt=GenPept&RID=T0DFMR3T01N&log$=prottop&blast_rank=40) | solute carrier family 5 (choline transporter), member 7 [Homo sapiens] | [17.2](http://blast.ncbi.nlm.nih.gov/Blast.cgi#11141885%2311141885) | 17.2 | 100% | 699 |
| [NP_001159529.1](http://www.ncbi.nlm.nih.gov/entrez/query.fcgi?cmd=Retrieve&db=Protein&list_uids=260593665&dopt=GenPept&RID=T0DFMR3T01N&log$=prottop&blast_rank=41) | prolidase isoform 3 [Homo sapiens] | [16.8](http://blast.ncbi.nlm.nih.gov/Blast.cgi#260593665%23260593665) | 16.8 | 100% | 938 |
| [NP_001020115.1](http://www.ncbi.nlm.nih.gov/entrez/query.fcgi?cmd=Retrieve&db=Protein&list_uids=68303547&dopt=GenPept&RID=T0DFMR3T01N&log$=prottop&blast_rank=42) | argininosuccinate lyase isoform 2 [Homo sapiens] | [16.8](http://blast.ncbi.nlm.nih.gov/Blast.cgi#68303547%2368303547) | 16.8 | 100% | 938 |
| [NP_001020117.1](http://www.ncbi.nlm.nih.gov/entrez/query.fcgi?cmd=Retrieve&db=Protein&list_uids=68303549&dopt=GenPept&RID=T0DFMR3T01N&log$=prottop&blast_rank=43) | argininosuccinate lyase isoform 3 [Homo sapiens] | [16.8](http://blast.ncbi.nlm.nih.gov/Blast.cgi#68303549%2368303549) | 16.8 | 100% | 938 |
| [NP_443087.1](http://www.ncbi.nlm.nih.gov/entrez/query.fcgi?cmd=Retrieve&db=Protein&list_uids=16418357&dopt=GenPept&RID=T0DFMR3T01N&log$=prottop&blast_rank=44) | ankyrin repeat domain 40 [Homo sapiens] | [16.8](http://blast.ncbi.nlm.nih.gov/Blast.cgi#16418357%2316418357) | 16.8 | 100% | 938 |
| [NP_079384.2](http://www.ncbi.nlm.nih.gov/entrez/query.fcgi?cmd=Retrieve&db=Protein&list_uids=84626580&dopt=GenPept&RID=T0DFMR3T01N&log$=prottop&blast_rank=45) | hypothetical protein LOC80178 [Homo sapiens] | [16.8](http://blast.ncbi.nlm.nih.gov/Blast.cgi#84626580%2384626580) | 16.8 | 100% | 938 |
| [NP_000039.2](http://www.ncbi.nlm.nih.gov/entrez/query.fcgi?cmd=Retrieve&db=Protein&list_uids=31541964&dopt=GenPept&RID=T0DFMR3T01N&log$=prottop&blast_rank=46) | argininosuccinate lyase isoform 1 [Homo sapiens] >ref|NP_001020114.1| argininosuccinate lyase isoform 1 [Homo sapiens] | [16.8](http://blast.ncbi.nlm.nih.gov/Blast.cgi#31541964%2331541964) | 16.8 | 100% | 938 |
| [NP_079334.3](http://www.ncbi.nlm.nih.gov/entrez/query.fcgi?cmd=Retrieve&db=Protein&list_uids=47174859&dopt=GenPept&RID=T0DFMR3T01N&log$=prottop&blast_rank=47) | tripartite motif-containing 46 [Homo sapiens] | [16.8](http://blast.ncbi.nlm.nih.gov/Blast.cgi#47174859%2347174859) | 16.8 | 100% | 938 |
| [NP_001113.2](http://www.ncbi.nlm.nih.gov/entrez/query.fcgi?cmd=Retrieve&db=Protein&list_uids=34577059&dopt=GenPept&RID=T0DFMR3T01N&log$=prottop&blast_rank=48) | adipose differentiation-related protein [Homo sapiens] | [16.8](http://blast.ncbi.nlm.nih.gov/Blast.cgi#34577059%2334577059) | 16.8 | 100% | 938 |
| [NP_060273.1](http://www.ncbi.nlm.nih.gov/entrez/query.fcgi?cmd=Retrieve&db=Protein&list_uids=8923374&dopt=GenPept&RID=T0DFMR3T01N&log$=prottop&blast_rank=49) | dihydrouridine synthase 2-like, SMM1 homolog [Homo sapiens] | [16.8](http://blast.ncbi.nlm.nih.gov/Blast.cgi#8923374%238923374) | 16.8 | 100% | 938 |
| [NP_008849.2](http://www.ncbi.nlm.nih.gov/entrez/query.fcgi?cmd=Retrieve&db=Protein&list_uids=68160941&dopt=GenPept&RID=T0DFMR3T01N&log$=prottop&blast_rank=50) | sterol-C5-desaturase [Homo sapiens] >ref|NP_001020127.1| sterol-C5-desaturase [Homo sapiens] | [16.8](http://blast.ncbi.nlm.nih.gov/Blast.cgi#68160941%2368160941) | 16.8 | 100% | 938 |
| [NP_000483.3](http://www.ncbi.nlm.nih.gov/entrez/query.fcgi?cmd=Retrieve&db=Protein&list_uids=90421313&dopt=GenPept&RID=T0DFMR3T01N&log$=prottop&blast_rank=51) | cystic fibrosis transmembrane conductance regulator [Homo sapiens] | [16.8](http://blast.ncbi.nlm.nih.gov/Blast.cgi#90421313%2390421313) | 16.8 | 100% | 938 |
| [NP_612642.2](http://www.ncbi.nlm.nih.gov/entrez/query.fcgi?cmd=Retrieve&db=Protein&list_uids=281604151&dopt=GenPept&RID=T0DFMR3T01N&log$=prottop&blast_rank=52) | phosphoglycerate mutase family member 5 isoform 3 [Homo sapiens] | [16.3](http://blast.ncbi.nlm.nih.gov/Blast.cgi#281604151%23281604151) | 16.3 | 66% | 1259 |
| [NP_001164015.1](http://www.ncbi.nlm.nih.gov/entrez/query.fcgi?cmd=Retrieve&db=Protein&list_uids=281604138&dopt=GenPept&RID=T0DFMR3T01N&log$=prottop&blast_rank=53) | phosphoglycerate mutase family member 5 isoform 2 [Homo sapiens] | [16.3](http://blast.ncbi.nlm.nih.gov/Blast.cgi#281604138%23281604138) | 16.3 | 66% | 1259 |
| [XP_002345019.1](http://www.ncbi.nlm.nih.gov/entrez/query.fcgi?cmd=Retrieve&db=Protein&list_uids=239756699&dopt=GenPept&RID=T0DFMR3T01N&log$=prottop&blast_rank=54) | PREDICTED: hypothetical protein [Homo sapiens] | [16.3](http://blast.ncbi.nlm.nih.gov/Blast.cgi#239756699%23239756699) | 16.3 | 66% | 1259 |
| [XP_002347119.1](http://www.ncbi.nlm.nih.gov/entrez/query.fcgi?cmd=Retrieve&db=Protein&list_uids=239749684&dopt=GenPept&RID=T0DFMR3T01N&log$=prottop&blast_rank=55) | PREDICTED: hemicentin 2 [Homo sapiens] | [16.3](http://blast.ncbi.nlm.nih.gov/Blast.cgi#239749684%23239749684) | 16.3 | 66% | 1259 |
| [XP_002347059.1](http://www.ncbi.nlm.nih.gov/entrez/query.fcgi?cmd=Retrieve&db=Protein&list_uids=239749502&dopt=GenPept&RID=T0DFMR3T01N&log$=prottop&blast_rank=56) | PREDICTED: hypothetical protein [Homo sapiens] | [16.3](http://blast.ncbi.nlm.nih.gov/Blast.cgi#239749502%23239749502) | 16.3 | 66% | 1259 |
| [XP_002343543.1](http://www.ncbi.nlm.nih.gov/entrez/query.fcgi?cmd=Retrieve&db=Protein&list_uids=239745682&dopt=GenPept&RID=T0DFMR3T01N&log$=prottop&blast_rank=57) | PREDICTED: hypothetical protein XP_002343543 [Homo sapiens] | [16.3](http://blast.ncbi.nlm.nih.gov/Blast.cgi#239745682%23239745682) | 16.3 | 66% | 1259 |
| [XP_002342441.1](http://www.ncbi.nlm.nih.gov/entrez/query.fcgi?cmd=Retrieve&db=Protein&list_uids=239742138&dopt=GenPept&RID=T0DFMR3T01N&log$=prottop&blast_rank=58) | PREDICTED: hypothetical protein XP_002342441 [Homo sapiens] >ref|XP_002346599.1| PREDICTED: hypothetical protein XP_002346599 [Homo sapiens] >ref|XP_002345759.1| PREDICTED: hypothetical protein [Homo sapiens] | [16.3](http://blast.ncbi.nlm.nih.gov/Blast.cgi#239742138%23239742138) | 16.3 | 66% | 1259 |
| [NP_001124495.1](http://www.ncbi.nlm.nih.gov/entrez/query.fcgi?cmd=Retrieve&db=Protein&list_uids=196259770&dopt=GenPept&RID=T0DFMR3T01N&log$=prottop&blast_rank=59) | peroxisomal biogenesis factor 5 isoform a [Homo sapiens] | [16.3](http://blast.ncbi.nlm.nih.gov/Blast.cgi#196259770%23196259770) | 16.3 | 66% | 1259 |
| [NP_057726.3](http://www.ncbi.nlm.nih.gov/entrez/query.fcgi?cmd=Retrieve&db=Protein&list_uids=170016061&dopt=GenPept&RID=T0DFMR3T01N&log$=prottop&blast_rank=60) | spectrin, beta, non-erythrocytic 5 [Homo sapiens] | [16.3](http://blast.ncbi.nlm.nih.gov/Blast.cgi#170016061%23170016061) | 16.3 | 66% | 1259 |
| [XP_001715206.1](http://www.ncbi.nlm.nih.gov/entrez/query.fcgi?cmd=Retrieve&db=Protein&list_uids=169178458&dopt=GenPept&RID=T0DFMR3T01N&log$=prottop&blast_rank=61) | PREDICTED: hemicentin 2 [Homo sapiens] | [16.3](http://blast.ncbi.nlm.nih.gov/Blast.cgi#169178458%23169178458) | 16.3 | 66% | 1259 |
| [XP_001726994.1](http://www.ncbi.nlm.nih.gov/entrez/query.fcgi?cmd=Retrieve&db=Protein&list_uids=169177000&dopt=GenPept&RID=T0DFMR3T01N&log$=prottop&blast_rank=62) | PREDICTED: hemicentin 2 [Homo sapiens] | [16.3](http://blast.ncbi.nlm.nih.gov/Blast.cgi#169177000%23169177000) | 16.3 | 66% | 1259 |
| [NP_775840.3](http://www.ncbi.nlm.nih.gov/entrez/query.fcgi?cmd=Retrieve&db=Protein&list_uids=236459851&dopt=GenPept&RID=T0DFMR3T01N&log$=prottop&blast_rank=63) | ubinuclein 2 [Homo sapiens] | [16.3](http://blast.ncbi.nlm.nih.gov/Blast.cgi#236459851%23236459851) | 16.3 | 66% | 1259 |
| [NP_001164014.1](http://www.ncbi.nlm.nih.gov/entrez/query.fcgi?cmd=Retrieve&db=Protein&list_uids=281604136&dopt=GenPept&RID=T0DFMR3T01N&log$=prottop&blast_rank=64) | phosphoglycerate mutase family member 5 isoform 1 [Homo sapiens] | [16.3](http://blast.ncbi.nlm.nih.gov/Blast.cgi#281604136%23281604136) | 16.3 | 66% | 1259 |
| [NP_001092007.1](http://www.ncbi.nlm.nih.gov/entrez/query.fcgi?cmd=Retrieve&db=Protein&list_uids=148727335&dopt=GenPept&RID=T0DFMR3T01N&log$=prottop&blast_rank=65) | patatin-like phospholipase domain containing 7 isoform a [Homo sapiens] | [16.3](http://blast.ncbi.nlm.nih.gov/Blast.cgi#148727335%23148727335) | 16.3 | 66% | 1259 |
| [NP_775901.3](http://www.ncbi.nlm.nih.gov/entrez/query.fcgi?cmd=Retrieve&db=Protein&list_uids=145046269&dopt=GenPept&RID=T0DFMR3T01N&log$=prottop&blast_rank=66) | rotatin [Homo sapiens] | [16.3](http://blast.ncbi.nlm.nih.gov/Blast.cgi#145046269%23145046269) | 16.3 | 66% | 1259 |
| [NP_001084.3](http://www.ncbi.nlm.nih.gov/entrez/query.fcgi?cmd=Retrieve&db=Protein&list_uids=134142062&dopt=GenPept&RID=T0DFMR3T01N&log$=prottop&blast_rank=67) | acetyl-Coenzyme A carboxylase beta [Homo sapiens] | [16.3](http://blast.ncbi.nlm.nih.gov/Blast.cgi#134142062%23134142062) | 16.3 | 66% | 1259 |
| [NP_689499.3](http://www.ncbi.nlm.nih.gov/entrez/query.fcgi?cmd=Retrieve&db=Protein&list_uids=148727290&dopt=GenPept&RID=T0DFMR3T01N&log$=prottop&blast_rank=68) | patatin-like phospholipase domain containing 7 isoform b [Homo sapiens] | [16.3](http://blast.ncbi.nlm.nih.gov/Blast.cgi#148727290%23148727290) | 16.3 | 66% | 1259 |
| [NP_476518.4](http://www.ncbi.nlm.nih.gov/entrez/query.fcgi?cmd=Retrieve&db=Protein&list_uids=119120903&dopt=GenPept&RID=T0DFMR3T01N&log$=prottop&blast_rank=69) | par-3 partitioning defective 3 homolog B isoform c [Homo sapiens] | [16.3](http://blast.ncbi.nlm.nih.gov/Blast.cgi#119120903%23119120903) | 28.0 | 100% | 1259 |
| [NP_056078.2](http://www.ncbi.nlm.nih.gov/entrez/query.fcgi?cmd=Retrieve&db=Protein&list_uids=119120894&dopt=GenPept&RID=T0DFMR3T01N&log$=prottop&blast_rank=70) | Dmx-like 2 [Homo sapiens] | [16.3](http://blast.ncbi.nlm.nih.gov/Blast.cgi#119120894%23119120894) | 29.7 | 83% | 1259 |
| [NP_995585.2](http://www.ncbi.nlm.nih.gov/entrez/query.fcgi?cmd=Retrieve&db=Protein&list_uids=119120907&dopt=GenPept&RID=T0DFMR3T01N&log$=prottop&blast_rank=71) | par-3 partitioning defective 3 homolog B isoform a [Homo sapiens] | [16.3](http://blast.ncbi.nlm.nih.gov/Blast.cgi#119120907%23119120907) | 28.0 | 100% | 1259 |
| [NP_001153736.1](http://www.ncbi.nlm.nih.gov/entrez/query.fcgi?cmd=Retrieve&db=Protein&list_uids=237681196&dopt=GenPept&RID=T0DFMR3T01N&log$=prottop&blast_rank=72) | FEZ family zinc finger 1 isoform 2 [Homo sapiens] | [16.3](http://blast.ncbi.nlm.nih.gov/Blast.cgi#237681196%23237681196) | 16.3 | 66% | 1259 |
| [NP_001019784.2](http://www.ncbi.nlm.nih.gov/entrez/query.fcgi?cmd=Retrieve&db=Protein&list_uids=237681194&dopt=GenPept&RID=T0DFMR3T01N&log$=prottop&blast_rank=73) | FEZ family zinc finger 1 isoform 1 [Homo sapiens] | [16.3](http://blast.ncbi.nlm.nih.gov/Blast.cgi#237681194%23237681194) | 16.3 | 66% | 1259 |
| [NP_997397.1](http://www.ncbi.nlm.nih.gov/entrez/query.fcgi?cmd=Retrieve&db=Protein&list_uids=46447820&dopt=GenPept&RID=T0DFMR3T01N&log$=prottop&blast_rank=74) | differentially expressed in FDCP 8 isoform 1 [Homo sapiens] | [16.3](http://blast.ncbi.nlm.nih.gov/Blast.cgi#46447820%2346447820) | 16.3 | 66% | 1259 |
| [NP_001034976.1](http://www.ncbi.nlm.nih.gov/entrez/query.fcgi?cmd=Retrieve&db=Protein&list_uids=89941479&dopt=GenPept&RID=T0DFMR3T01N&log$=prottop&blast_rank=75) | hypothetical protein LOC148137 [Homo sapiens] | [16.3](http://blast.ncbi.nlm.nih.gov/Blast.cgi#89941479%2389941479) | 16.3 | 66% | 1259 |
| [NP_001072.2](http://www.ncbi.nlm.nih.gov/entrez/query.fcgi?cmd=Retrieve&db=Protein&list_uids=126091152&dopt=GenPept&RID=T0DFMR3T01N&log$=prottop&blast_rank=76) | cubilin precursor [Homo sapiens] | [16.3](http://blast.ncbi.nlm.nih.gov/Blast.cgi#126091152%23126091152) | 16.3 | 66% | 1259 |
| [NP_005179.2](http://www.ncbi.nlm.nih.gov/entrez/query.fcgi?cmd=Retrieve&db=Protein&list_uids=52426745&dopt=GenPept&RID=T0DFMR3T01N&log$=prottop&blast_rank=77) | Cas-Br-M (murine) ecotropic retroviral transforming sequence [Homo sapiens] | [16.3](http://blast.ncbi.nlm.nih.gov/Blast.cgi#52426745%2352426745) | 16.3 | 66% | 1259 |
| [NP_001071062.1](http://www.ncbi.nlm.nih.gov/entrez/query.fcgi?cmd=Retrieve&db=Protein&list_uids=117938266&dopt=GenPept&RID=T0DFMR3T01N&log$=prottop&blast_rank=78) | hypothetical protein LOC91828 [Homo sapiens] | [16.3](http://blast.ncbi.nlm.nih.gov/Blast.cgi#117938266%23117938266) | 16.3 | 66% | 1259 |
| [NP_036355.2](http://www.ncbi.nlm.nih.gov/entrez/query.fcgi?cmd=Retrieve&db=Protein&list_uids=44889481&dopt=GenPept&RID=T0DFMR3T01N&log$=prottop&blast_rank=79) | myosin IB isoform 2 [Homo sapiens] | [16.3](http://blast.ncbi.nlm.nih.gov/Blast.cgi#44889481%2344889481) | 16.3 | 66% | 1259 |
| [NP_690621.1](http://www.ncbi.nlm.nih.gov/entrez/query.fcgi?cmd=Retrieve&db=Protein&list_uids=22902130&dopt=GenPept&RID=T0DFMR3T01N&log$=prottop&blast_rank=80) | PTK7 protein tyrosine kinase 7 isoform d precursor [Homo sapiens] | [16.3](http://blast.ncbi.nlm.nih.gov/Blast.cgi#22902130%2322902130) | 16.3 | 66% | 1259 |
| [NP_000310.2](http://www.ncbi.nlm.nih.gov/entrez/query.fcgi?cmd=Retrieve&db=Protein&list_uids=21361204&dopt=GenPept&RID=T0DFMR3T01N&log$=prottop&blast_rank=81) | peroxisomal biogenesis factor 5 isoform b [Homo sapiens] | [16.3](http://blast.ncbi.nlm.nih.gov/Blast.cgi#21361204%2321361204) | 16.3 | 66% | 1259 |
| [NP_060478.3](http://www.ncbi.nlm.nih.gov/entrez/query.fcgi?cmd=Retrieve&db=Protein&list_uids=157388917&dopt=GenPept&RID=T0DFMR3T01N&log$=prottop&blast_rank=82) | FEZ family zinc finger 2 [Homo sapiens] | [16.3](http://blast.ncbi.nlm.nih.gov/Blast.cgi#157388917%23157388917) | 16.3 | 66% | 1259 |
| [NP_690619.1](http://www.ncbi.nlm.nih.gov/entrez/query.fcgi?cmd=Retrieve&db=Protein&list_uids=22902126&dopt=GenPept&RID=T0DFMR3T01N&log$=prottop&blast_rank=83) | PTK7 protein tyrosine kinase 7 isoform b precursor [Homo sapiens] | [16.3](http://blast.ncbi.nlm.nih.gov/Blast.cgi#22902126%2322902126) | 16.3 | 66% | 1259 |
| [NP_689739.4](http://www.ncbi.nlm.nih.gov/entrez/query.fcgi?cmd=Retrieve&db=Protein&list_uids=119120897&dopt=GenPept&RID=T0DFMR3T01N&log$=prottop&blast_rank=84) | par-3 partitioning defective 3 homolog B isoform b [Homo sapiens] | [16.3](http://blast.ncbi.nlm.nih.gov/Blast.cgi#119120897%23119120897) | 28.0 | 100% | 1259 |
| [NP_112169.2](http://www.ncbi.nlm.nih.gov/entrez/query.fcgi?cmd=Retrieve&db=Protein&list_uids=241666479&dopt=GenPept&RID=T0DFMR3T01N&log$=prottop&blast_rank=85) | hypothetical protein LOC79363 [Homo sapiens] | [16.3](http://blast.ncbi.nlm.nih.gov/Blast.cgi#241666479%23241666479) | 16.3 | 66% | 1259 |
| [NP_690620.1](http://www.ncbi.nlm.nih.gov/entrez/query.fcgi?cmd=Retrieve&db=Protein&list_uids=22902128&dopt=GenPept&RID=T0DFMR3T01N&log$=prottop&blast_rank=86) | PTK7 protein tyrosine kinase 7 isoform c precursor [Homo sapiens] | [16.3](http://blast.ncbi.nlm.nih.gov/Blast.cgi#22902128%2322902128) | 16.3 | 66% | 1259 |
| [NP_620168.1](http://www.ncbi.nlm.nih.gov/entrez/query.fcgi?cmd=Retrieve&db=Protein&list_uids=44888835&dopt=GenPept&RID=T0DFMR3T01N&log$=prottop&blast_rank=87) | ATPase, class I, type 8B, member 3 [Homo sapiens] | [16.3](http://blast.ncbi.nlm.nih.gov/Blast.cgi#44888835%2344888835) | 30.5 | 100% | 1259 |
| [NP_115940.2](http://www.ncbi.nlm.nih.gov/entrez/query.fcgi?cmd=Retrieve&db=Protein&list_uids=62865887&dopt=GenPept&RID=T0DFMR3T01N&log$=prottop&blast_rank=88) | G protein-coupled receptor 54 [Homo sapiens] | [16.3](http://blast.ncbi.nlm.nih.gov/Blast.cgi#62865887%2362865887) | 16.3 | 66% | 1259 |
| [NP_054825.2](http://www.ncbi.nlm.nih.gov/entrez/query.fcgi?cmd=Retrieve&db=Protein&list_uids=46094072&dopt=GenPept&RID=T0DFMR3T01N&log$=prottop&blast_rank=89) | zinc finger protein 770 [Homo sapiens] | [16.3](http://blast.ncbi.nlm.nih.gov/Blast.cgi#46094072%2346094072) | 16.3 | 66% | 1259 |
| [NP_775754.2](http://www.ncbi.nlm.nih.gov/entrez/query.fcgi?cmd=Retrieve&db=Protein&list_uids=158138530&dopt=GenPept&RID=T0DFMR3T01N&log$=prottop&blast_rank=90) | cytochrome P450, family 4, subfamily F, polypeptide 22 [Homo sapiens] | [16.3](http://blast.ncbi.nlm.nih.gov/Blast.cgi#158138530%23158138530) | 16.3 | 66% | 1259 |
| [NP_680477.1](http://www.ncbi.nlm.nih.gov/entrez/query.fcgi?cmd=Retrieve&db=Protein&list_uids=22538478&dopt=GenPept&RID=T0DFMR3T01N&log$=prottop&blast_rank=91) | phosphatidylethanolamine N-methyltransferase isoform 1 [Homo sapiens] | [16.3](http://blast.ncbi.nlm.nih.gov/Blast.cgi#22538478%2322538478) | 29.3 | 100% | 1259 |
| [NP_597841.1](http://www.ncbi.nlm.nih.gov/entrez/query.fcgi?cmd=Retrieve&db=Protein&list_uids=19743571&dopt=GenPept&RID=T0DFMR3T01N&log$=prottop&blast_rank=92) | TRAF interacting protein TANK isoform b [Homo sapiens] | [16.3](http://blast.ncbi.nlm.nih.gov/Blast.cgi#19743571%2319743571) | 16.3 | 66% | 1259 |
| [NP_060729.2](http://www.ncbi.nlm.nih.gov/entrez/query.fcgi?cmd=Retrieve&db=Protein&list_uids=21361787&dopt=GenPept&RID=T0DFMR3T01N&log$=prottop&blast_rank=93) | tetratricopeptide repeat domain 17 [Homo sapiens] | [16.3](http://blast.ncbi.nlm.nih.gov/Blast.cgi#21361787%2321361787) | 16.3 | 66% | 1259 |
| [NP_001018088.1](http://www.ncbi.nlm.nih.gov/entrez/query.fcgi?cmd=Retrieve&db=Protein&list_uids=66932990&dopt=GenPept&RID=T0DFMR3T01N&log$=prottop&blast_rank=94) | folylpolyglutamate synthase isoform b [Homo sapiens] | [16.3](http://blast.ncbi.nlm.nih.gov/Blast.cgi#66932990%2366932990) | 16.3 | 66% | 1259 |
| [NP_055685.3](http://www.ncbi.nlm.nih.gov/entrez/query.fcgi?cmd=Retrieve&db=Protein&list_uids=139394556&dopt=GenPept&RID=T0DFMR3T01N&log$=prottop&blast_rank=95) | zinc finger and BTB domain containing 40 [Homo sapiens] >ref|NP_001077090.1| zinc finger and BTB domain containing 40 [Homo sapiens] | [16.3](http://blast.ncbi.nlm.nih.gov/Blast.cgi#139394556%23139394556) | 16.3 | 66% | 1259 |
| [NP_659496.2](http://www.ncbi.nlm.nih.gov/entrez/query.fcgi?cmd=Retrieve&db=Protein&list_uids=63175654&dopt=GenPept&RID=T0DFMR3T01N&log$=prottop&blast_rank=96) | fucokinase [Homo sapiens] | [16.3](http://blast.ncbi.nlm.nih.gov/Blast.cgi#63175654%2363175654) | 16.3 | 66% | 1259 |
| [NP_055805.1](http://www.ncbi.nlm.nih.gov/entrez/query.fcgi?cmd=Retrieve&db=Protein&list_uids=51226124&dopt=GenPept&RID=T0DFMR3T01N&log$=prottop&blast_rank=97) | Ral GTPase activating protein, alpha subunit 1 (catalytic) isoform 1 [Homo sapiens] | [16.3](http://blast.ncbi.nlm.nih.gov/Blast.cgi#51226124%2351226124) | 16.3 | 66% | 1259 |
| [NP_004660.2](http://www.ncbi.nlm.nih.gov/entrez/query.fcgi?cmd=Retrieve&db=Protein&list_uids=40288290&dopt=GenPept&RID=T0DFMR3T01N&log$=prottop&blast_rank=98) | chloride intracellular channel 3 [Homo sapiens] | [16.3](http://blast.ncbi.nlm.nih.gov/Blast.cgi#40288290%2340288290) | 16.3 | 66% | 1259 |
| [NP_861455.1](http://www.ncbi.nlm.nih.gov/entrez/query.fcgi?cmd=Retrieve&db=Protein&list_uids=32401433&dopt=GenPept&RID=T0DFMR3T01N&log$=prottop&blast_rank=99) | G protein-coupled receptor 142 [Homo sapiens] | [16.3](http://blast.ncbi.nlm.nih.gov/Blast.cgi#32401433%2332401433) | 16.3 | 66% | 1259 |
| [NP_001123630.1](http://www.ncbi.nlm.nih.gov/entrez/query.fcgi?cmd=Retrieve&db=Protein&list_uids=194328685&dopt=GenPept&RID=T0DFMR3T01N&log$=prottop&blast_rank=100) | myosin IB isoform 1 [Homo sapiens] >ref|NP_001155291.1| myosin IB isoform 1 [Homo sapiens] | [16.3](http://blast.ncbi.nlm.nih.gov/Blast.cgi#194328685%23194328685) | 16.3 | 66% | 1259 |
