# Supplementary material for: A high throughput method for identifying personalized tumor-associated antigens
Source: Oncotarget. 2010 Jun 27;1(2):148–55. doi: 10.18632/oncotarget.118 (PMC2920534; doi:10.18632/oncotarget.118)
Supplement: Supplemental Table 3 [file oncotarget-01-148-s003.doc]

**IgG Patient B**

| **Accession** | **Proteins with a match to FSRRAQQVGAK peptide** | **[Max score](http://blast.ncbi.nlm.nih.gov/Blast.cgi?CMD=Get&ALIGNMENTS=100&ALIGNMENT_VIEW=Pairwise&CDD_SEARCH_STATE=1&DATABASE_SORT=0&DESCRIPTIONS=100&ENTREZ_QUERY=txid9606 %5BORGN%5D&FIRST_QUERY_NUM=0&FORMAT_OBJECT=Alignment&FORMAT_PAGE_TARGET=&FORMAT_TYPE=HTML&GET_SEQUENCE=yes&I_THRESH=&MASK_CHAR=2&MASK_COLOR=1&NEW_DESIGN=on&NEW_VIEW=yes&NUM_OVERVIEW=100&OLD_BLAST=false&PAGE=Proteins&QUERY_INDEX=0&QUERY_NUMBER=0&RESULTS_PAGE_TARGET=&RID=T24PVJKM01S&SHOW_LINKOUT=yes&SHOW_OVERVIEW=yes&STEP_NUMBER=&WORD_SIZE=2&DISPLAY_SORT=1&HSP_SORT=1" \l "sort_mark)** | **[Total score](http://blast.ncbi.nlm.nih.gov/Blast.cgi?CMD=Get&ALIGNMENTS=100&ALIGNMENT_VIEW=Pairwise&CDD_SEARCH_STATE=1&DATABASE_SORT=0&DESCRIPTIONS=100&ENTREZ_QUERY=txid9606 %5BORGN%5D&FIRST_QUERY_NUM=0&FORMAT_OBJECT=Alignment&FORMAT_PAGE_TARGET=&FORMAT_TYPE=HTML&GET_SEQUENCE=yes&I_THRESH=&MASK_CHAR=2&MASK_COLOR=1&NEW_DESIGN=on&NEW_VIEW=yes&NUM_OVERVIEW=100&OLD_BLAST=false&PAGE=Proteins&QUERY_INDEX=0&QUERY_NUMBER=0&RESULTS_PAGE_TARGET=&RID=T24PVJKM01S&SHOW_LINKOUT=yes&SHOW_OVERVIEW=yes&STEP_NUMBER=&WORD_SIZE=2&DISPLAY_SORT=2&HSP_SORT=1" \l "sort_mark)** | **[Query coverage](http://blast.ncbi.nlm.nih.gov/Blast.cgi?CMD=Get&ALIGNMENTS=100&ALIGNMENT_VIEW=Pairwise&CDD_SEARCH_STATE=1&DATABASE_SORT=0&DESCRIPTIONS=100&ENTREZ_QUERY=txid9606 %5BORGN%5D&FIRST_QUERY_NUM=0&FORMAT_OBJECT=Alignment&FORMAT_PAGE_TARGET=&FORMAT_TYPE=HTML&GET_SEQUENCE=yes&I_THRESH=&MASK_CHAR=2&MASK_COLOR=1&NEW_DESIGN=on&NEW_VIEW=yes&NUM_OVERVIEW=100&OLD_BLAST=false&PAGE=Proteins&QUERY_INDEX=0&QUERY_NUMBER=0&RESULTS_PAGE_TARGET=&RID=T24PVJKM01S&SHOW_LINKOUT=yes&SHOW_OVERVIEW=yes&STEP_NUMBER=&WORD_SIZE=2&DISPLAY_SORT=4&HSP_SORT=0" \l "sort_mark)** | **[E value](http://blast.ncbi.nlm.nih.gov/Blast.cgi?CMD=Get&ALIGNMENTS=100&ALIGNMENT_VIEW=Pairwise&CDD_SEARCH_STATE=1&DATABASE_SORT=0&DESCRIPTIONS=100&ENTREZ_QUERY=txid9606 %5BORGN%5D&FIRST_QUERY_NUM=0&FORMAT_OBJECT=Alignment&FORMAT_PAGE_TARGET=&FORMAT_TYPE=HTML&GET_SEQUENCE=yes&I_THRESH=&MASK_CHAR=2&MASK_COLOR=1&NEW_DESIGN=on&NEW_VIEW=yes&NUM_OVERVIEW=100&OLD_BLAST=false&PAGE=Proteins&QUERY_INDEX=0&QUERY_NUMBER=0&RESULTS_PAGE_TARGET=&RID=T24PVJKM01S&SHOW_LINKOUT=yes&SHOW_OVERVIEW=yes&STEP_NUMBER=&WORD_SIZE=2&DISPLAY_SORT=0&HSP_SORT=0" \l "sort_mark)** |
| --- | --- | --- | --- | --- | --- |
| [XP_002347229.1](http://www.ncbi.nlm.nih.gov/entrez/query.fcgi?cmd=Retrieve&db=Protein&list_uids=239749957&dopt=GenPept&RID=T24PVJKM01S&log$=prottop&blast_rank=1) | PREDICTED: hypothetical protein [Homo sapiens] | [24.8](http://blast.ncbi.nlm.nih.gov/Blast.cgi#239749957%23239749957) | 39.9 | 63% | 5.9 |
| [NP_116291.1](http://www.ncbi.nlm.nih.gov/entrez/query.fcgi?cmd=Retrieve&db=Protein&list_uids=14249672&dopt=GenPept&RID=T24PVJKM01S&log$=prottop&blast_rank=2) | protein phosphatase 1, regulatory (inhibitor) subunit 16A [Homo sapiens] | [22.3](http://blast.ncbi.nlm.nih.gov/Blast.cgi#14249672%2314249672) | 22.3 | 54% | 34 |
| [NP_055847.1](http://www.ncbi.nlm.nih.gov/entrez/query.fcgi?cmd=Retrieve&db=Protein&list_uids=7657269&dopt=GenPept&RID=T24PVJKM01S&log$=prottop&blast_rank=3) | PDS5, regulator of cohesion maintenance, homolog B [Homo sapiens] | [21.8](http://blast.ncbi.nlm.nih.gov/Blast.cgi#7657269%237657269) | 21.8 | 54% | 46 |
| [NP_005430.1](http://www.ncbi.nlm.nih.gov/entrez/query.fcgi?cmd=Retrieve&db=Protein&list_uids=4885487&dopt=GenPept&RID=T24PVJKM01S&log$=prottop&blast_rank=4) | myeloid leukemia factor 2 [Homo sapiens] | [21.4](http://blast.ncbi.nlm.nih.gov/Blast.cgi#4885487%234885487) | 32.2 | 81% | 61 |
| [NP_056410.3](http://www.ncbi.nlm.nih.gov/entrez/query.fcgi?cmd=Retrieve&db=Protein&list_uids=194473700&dopt=GenPept&RID=T24PVJKM01S&log$=prottop&blast_rank=5) | Src homology 3 domain-containing guanine nucleotide exchange factor [Homo sapiens] | [21.0](http://blast.ncbi.nlm.nih.gov/Blast.cgi#194473700%23194473700) | 21.0 | 72% | 82 |
| [NP_005468.1](http://www.ncbi.nlm.nih.gov/entrez/query.fcgi?cmd=Retrieve&db=Protein&list_uids=4885407&dopt=GenPept&RID=T24PVJKM01S&log$=prottop&blast_rank=6) | hyperpolarization activated cyclic nucleotide-gated potassium channel 4 [Homo sapiens] | [21.0](http://blast.ncbi.nlm.nih.gov/Blast.cgi#4885407%234885407) | 21.0 | 54% | 82 |
| [XP_002342939.1](http://www.ncbi.nlm.nih.gov/entrez/query.fcgi?cmd=Retrieve&db=Protein&list_uids=239743664&dopt=GenPept&RID=T24PVJKM01S&log$=prottop&blast_rank=7) | PREDICTED: hypothetical protein XP_002342939 isoform 1 [Homo sapiens] | [20.6](http://blast.ncbi.nlm.nih.gov/Blast.cgi#239743664%23239743664) | 20.6 | 72% | 111 |
| [NP_878908.4](http://www.ncbi.nlm.nih.gov/entrez/query.fcgi?cmd=Retrieve&db=Protein&list_uids=261337183&dopt=GenPept&RID=T24PVJKM01S&log$=prottop&blast_rank=8) | WAS protein family homolog 1 [Homo sapiens] >ref|XP_002342940.1| PREDICTED: hypothetical protein XP_002342940 isoform 2 [Homo sapiens] | [20.6](http://blast.ncbi.nlm.nih.gov/Blast.cgi#261337183%23261337183) | 20.6 | 72% | 111 |
| [NP_060368.2](http://www.ncbi.nlm.nih.gov/entrez/query.fcgi?cmd=Retrieve&db=Protein&list_uids=31542713&dopt=GenPept&RID=T24PVJKM01S&log$=prottop&blast_rank=9) | MOCO sulphurase C-terminal domain containing 2 precursor [Homo sapiens] | [20.6](http://blast.ncbi.nlm.nih.gov/Blast.cgi#31542713%2331542713) | 20.6 | 81% | 111 |
| [NP_066300.1](http://www.ncbi.nlm.nih.gov/entrez/query.fcgi?cmd=Retrieve&db=Protein&list_uids=10440566&dopt=GenPept&RID=T24PVJKM01S&log$=prottop&blast_rank=10) | leucine zipper, putative tumor suppressor 1 [Homo sapiens] | [20.6](http://blast.ncbi.nlm.nih.gov/Blast.cgi#10440566%2310440566) | 20.6 | 63% | 111 |
| [NP_000227.2](http://www.ncbi.nlm.nih.gov/entrez/query.fcgi?cmd=Retrieve&db=Protein&list_uids=194097335&dopt=GenPept&RID=T24PVJKM01S&log$=prottop&blast_rank=11) | lipase C precursor [Homo sapiens] | [20.2](http://blast.ncbi.nlm.nih.gov/Blast.cgi#194097335%23194097335) | 20.2 | 72% | 148 |
| [NP_064536.2](http://www.ncbi.nlm.nih.gov/entrez/query.fcgi?cmd=Retrieve&db=Protein&list_uids=151301035&dopt=GenPept&RID=T24PVJKM01S&log$=prottop&blast_rank=12) | START domain containing 7 precursor [Homo sapiens] | [20.2](http://blast.ncbi.nlm.nih.gov/Blast.cgi#151301035%23151301035) | 20.2 | 54% | 148 |
| [NP_001160072.1](http://www.ncbi.nlm.nih.gov/entrez/query.fcgi?cmd=Retrieve&db=Protein&list_uids=262205380&dopt=GenPept&RID=T24PVJKM01S&log$=prottop&blast_rank=13) | family with sequence similarity 122B isoform 3 [Homo sapiens] | [19.7](http://blast.ncbi.nlm.nih.gov/Blast.cgi#262205380%23262205380) | 19.7 | 54% | 199 |
| [NP_001164227.1](http://www.ncbi.nlm.nih.gov/entrez/query.fcgi?cmd=Retrieve&db=Protein&list_uids=282847483&dopt=GenPept&RID=T24PVJKM01S&log$=prottop&blast_rank=14) | family with sequence similarity 122B isoform 4 [Homo sapiens] | [19.7](http://blast.ncbi.nlm.nih.gov/Blast.cgi#282847483%23282847483) | 19.7 | 54% | 199 |
| [NP_115676.1](http://www.ncbi.nlm.nih.gov/entrez/query.fcgi?cmd=Retrieve&db=Protein&list_uids=14150056&dopt=GenPept&RID=T24PVJKM01S&log$=prottop&blast_rank=15) | trichoplein [Homo sapiens] >ref|NP_001137324.1| trichoplein [Homo sapiens] | [19.7](http://blast.ncbi.nlm.nih.gov/Blast.cgi#14150056%2314150056) | 36.1 | 63% | 199 |
| [NP_055563.1](http://www.ncbi.nlm.nih.gov/entrez/query.fcgi?cmd=Retrieve&db=Protein&list_uids=7661890&dopt=GenPept&RID=T24PVJKM01S&log$=prottop&blast_rank=16) | sorting nexin 17 [Homo sapiens] | [19.7](http://blast.ncbi.nlm.nih.gov/Blast.cgi#7661890%237661890) | 19.7 | 63% | 199 |
| [NP_000981.1](http://www.ncbi.nlm.nih.gov/entrez/query.fcgi?cmd=Retrieve&db=Protein&list_uids=4506625&dopt=GenPept&RID=T24PVJKM01S&log$=prottop&blast_rank=17) | ribosomal protein L27a [Homo sapiens] | [19.7](http://blast.ncbi.nlm.nih.gov/Blast.cgi#4506625%234506625) | 19.7 | 63% | 199 |
| [NP_065099.3](http://www.ncbi.nlm.nih.gov/entrez/query.fcgi?cmd=Retrieve&db=Protein&list_uids=112734867&dopt=GenPept&RID=T24PVJKM01S&log$=prottop&blast_rank=18) | retinitis pigmentosa GTPase regulator interacting protein 1 [Homo sapiens] | [19.7](http://blast.ncbi.nlm.nih.gov/Blast.cgi#112734867%23112734867) | 19.7 | 63% | 199 |
| [NP_001160071.1](http://www.ncbi.nlm.nih.gov/entrez/query.fcgi?cmd=Retrieve&db=Protein&list_uids=262205374&dopt=GenPept&RID=T24PVJKM01S&log$=prottop&blast_rank=19) | family with sequence similarity 122B isoform 2 [Homo sapiens] | [19.7](http://blast.ncbi.nlm.nih.gov/Blast.cgi#262205374%23262205374) | 19.7 | 54% | 199 |
| [NP_001166206.1](http://www.ncbi.nlm.nih.gov/entrez/query.fcgi?cmd=Retrieve&db=Protein&list_uids=289629249&dopt=GenPept&RID=T24PVJKM01S&log$=prottop&blast_rank=20) | protein phosphatase 1 regulatory inhibitor subunit 16B isoform 2 [Homo sapiens] | [19.3](http://blast.ncbi.nlm.nih.gov/Blast.cgi#289629249%23289629249) | 19.3 | 45% | 267 |
| [XP_002343463.1](http://www.ncbi.nlm.nih.gov/entrez/query.fcgi?cmd=Retrieve&db=Protein&list_uids=239745424&dopt=GenPept&RID=T24PVJKM01S&log$=prottop&blast_rank=21) | PREDICTED: hypothetical protein XP_002343463 [Homo sapiens] | [19.3](http://blast.ncbi.nlm.nih.gov/Blast.cgi#239745424%23239745424) | 19.3 | 54% | 267 |
| [XP_002344383.1](http://www.ncbi.nlm.nih.gov/entrez/query.fcgi?cmd=Retrieve&db=Protein&list_uids=239508894&dopt=GenPept&RID=T24PVJKM01S&log$=prottop&blast_rank=22) | PREDICTED: hypothetical protein [Homo sapiens] >ref|XP_002342807.1| PREDICTED: hypothetical protein XP_002342807 [Homo sapiens] >ref|XP_002346929.1| PREDICTED: hypothetical protein XP_002346929 [Homo sapiens] >ref|XP_002346046.1| PREDICTED: hypothetical protein [Homo sapiens] | [19.3](http://blast.ncbi.nlm.nih.gov/Blast.cgi#239508894%23239508894) | 19.3 | 45% | 267 |
| [NP_001137311.1](http://www.ncbi.nlm.nih.gov/entrez/query.fcgi?cmd=Retrieve&db=Protein&list_uids=219802706&dopt=GenPept&RID=T24PVJKM01S&log$=prottop&blast_rank=23) | phosphodiesterase 2A isoform 2 [Homo sapiens] | [19.3](http://blast.ncbi.nlm.nih.gov/Blast.cgi#219802706%23219802706) | 31.0 | 90% | 267 |
| [NP_001130034.1](http://www.ncbi.nlm.nih.gov/entrez/query.fcgi?cmd=Retrieve&db=Protein&list_uids=211971074&dopt=GenPept&RID=T24PVJKM01S&log$=prottop&blast_rank=24) | A kinase (PRKA) anchor protein 2 isoform 2 [Homo sapiens] | [19.3](http://blast.ncbi.nlm.nih.gov/Blast.cgi#211971074%23211971074) | 19.3 | 45% | 267 |
| [NP_892006.2](http://www.ncbi.nlm.nih.gov/entrez/query.fcgi?cmd=Retrieve&db=Protein&list_uids=154277116&dopt=GenPept&RID=T24PVJKM01S&log$=prottop&blast_rank=25) | spectrin repeat containing, nuclear envelope 1 isoform 1 [Homo sapiens] | [19.3](http://blast.ncbi.nlm.nih.gov/Blast.cgi#154277116%23154277116) | 35.2 | 45% | 267 |
| [NP_444271.2](http://www.ncbi.nlm.nih.gov/entrez/query.fcgi?cmd=Retrieve&db=Protein&list_uids=151301053&dopt=GenPept&RID=T24PVJKM01S&log$=prottop&blast_rank=26) | RNA binding motif protein 33 [Homo sapiens] | [19.3](http://blast.ncbi.nlm.nih.gov/Blast.cgi#151301053%23151301053) | 19.3 | 72% | 267 |
| [NP_055366.3](http://www.ncbi.nlm.nih.gov/entrez/query.fcgi?cmd=Retrieve&db=Protein&list_uids=148368987&dopt=GenPept&RID=T24PVJKM01S&log$=prottop&blast_rank=27) | kleisin beta isoform 1 [Homo sapiens] | [19.3](http://blast.ncbi.nlm.nih.gov/Blast.cgi#148368987%23148368987) | 19.3 | 72% | 267 |
| [NP_001096034.1](http://www.ncbi.nlm.nih.gov/entrez/query.fcgi?cmd=Retrieve&db=Protein&list_uids=156523264&dopt=GenPept&RID=T24PVJKM01S&log$=prottop&blast_rank=28) | hypothetical protein LOC112752 isoform 2 [Homo sapiens] | [19.3](http://blast.ncbi.nlm.nih.gov/Blast.cgi#156523264%23156523264) | 19.3 | 45% | 267 |
| [NP_443105.2](http://www.ncbi.nlm.nih.gov/entrez/query.fcgi?cmd=Retrieve&db=Protein&list_uids=156523262&dopt=GenPept&RID=T24PVJKM01S&log$=prottop&blast_rank=29) | hypothetical protein LOC112752 isoform 1 [Homo sapiens] | [19.3](http://blast.ncbi.nlm.nih.gov/Blast.cgi#156523262%23156523262) | 19.3 | 45% | 267 |
| [NP_036232.2](http://www.ncbi.nlm.nih.gov/entrez/query.fcgi?cmd=Retrieve&db=Protein&list_uids=156416028&dopt=GenPept&RID=T24PVJKM01S&log$=prottop&blast_rank=30) | aspartyl aminopeptidase [Homo sapiens] | [19.3](http://blast.ncbi.nlm.nih.gov/Blast.cgi#156416028%23156416028) | 19.3 | 72% | 267 |
| [NP_057323.3](http://www.ncbi.nlm.nih.gov/entrez/query.fcgi?cmd=Retrieve&db=Protein&list_uids=118402590&dopt=GenPept&RID=T24PVJKM01S&log$=prottop&blast_rank=31) | myosin XV [Homo sapiens] | [19.3](http://blast.ncbi.nlm.nih.gov/Blast.cgi#118402590%23118402590) | 81.2 | 81% | 267 |
| [NP_001070946.1](http://www.ncbi.nlm.nih.gov/entrez/query.fcgi?cmd=Retrieve&db=Protein&list_uids=117938746&dopt=GenPept&RID=T24PVJKM01S&log$=prottop&blast_rank=32) | constitutive androstane receptor isoform 7 [Homo sapiens] | [19.3](http://blast.ncbi.nlm.nih.gov/Blast.cgi#117938746%23117938746) | 19.3 | 45% | 267 |
| [NP_001070945.1](http://www.ncbi.nlm.nih.gov/entrez/query.fcgi?cmd=Retrieve&db=Protein&list_uids=117938748&dopt=GenPept&RID=T24PVJKM01S&log$=prottop&blast_rank=33) | constitutive androstane receptor isoform 14 [Homo sapiens] | [19.3](http://blast.ncbi.nlm.nih.gov/Blast.cgi#117938748%23117938748) | 19.3 | 45% | 267 |
| [NP_001070942.1](http://www.ncbi.nlm.nih.gov/entrez/query.fcgi?cmd=Retrieve&db=Protein&list_uids=117938740&dopt=GenPept&RID=T24PVJKM01S&log$=prottop&blast_rank=34) | constitutive androstane receptor isoform 8 [Homo sapiens] | [19.3](http://blast.ncbi.nlm.nih.gov/Blast.cgi#117938740%23117938740) | 19.3 | 45% | 267 |
| [NP_001070941.1](http://www.ncbi.nlm.nih.gov/entrez/query.fcgi?cmd=Retrieve&db=Protein&list_uids=117938738&dopt=GenPept&RID=T24PVJKM01S&log$=prottop&blast_rank=35) | constitutive androstane receptor isoform 12 [Homo sapiens] | [19.3](http://blast.ncbi.nlm.nih.gov/Blast.cgi#117938738%23117938738) | 19.3 | 45% | 267 |
| [NP_001070944.1](http://www.ncbi.nlm.nih.gov/entrez/query.fcgi?cmd=Retrieve&db=Protein&list_uids=117938744&dopt=GenPept&RID=T24PVJKM01S&log$=prottop&blast_rank=36) | constitutive androstane receptor isoform 13 [Homo sapiens] | [19.3](http://blast.ncbi.nlm.nih.gov/Blast.cgi#117938744%23117938744) | 19.3 | 45% | 267 |
| [NP_001070943.1](http://www.ncbi.nlm.nih.gov/entrez/query.fcgi?cmd=Retrieve&db=Protein&list_uids=117938742&dopt=GenPept&RID=T24PVJKM01S&log$=prottop&blast_rank=37) | constitutive androstane receptor isoform 15 [Homo sapiens] | [19.3](http://blast.ncbi.nlm.nih.gov/Blast.cgi#117938742%23117938742) | 19.3 | 45% | 267 |
| [NP_001070937.1](http://www.ncbi.nlm.nih.gov/entrez/query.fcgi?cmd=Retrieve&db=Protein&list_uids=117938729&dopt=GenPept&RID=T24PVJKM01S&log$=prottop&blast_rank=38) | constitutive androstane receptor isoform 6 [Homo sapiens] | [19.3](http://blast.ncbi.nlm.nih.gov/Blast.cgi#117938729%23117938729) | 19.3 | 45% | 267 |
| [NP_001070949.1](http://www.ncbi.nlm.nih.gov/entrez/query.fcgi?cmd=Retrieve&db=Protein&list_uids=117938754&dopt=GenPept&RID=T24PVJKM01S&log$=prottop&blast_rank=39) | constitutive androstane receptor isoform 4 [Homo sapiens] | [19.3](http://blast.ncbi.nlm.nih.gov/Blast.cgi#117938754%23117938754) | 19.3 | 45% | 267 |
| [NP_001070950.1](http://www.ncbi.nlm.nih.gov/entrez/query.fcgi?cmd=Retrieve&db=Protein&list_uids=117938756&dopt=GenPept&RID=T24PVJKM01S&log$=prottop&blast_rank=40) | constitutive androstane receptor isoform 1 [Homo sapiens] | [19.3](http://blast.ncbi.nlm.nih.gov/Blast.cgi#117938756%23117938756) | 19.3 | 45% | 267 |
| [NP_001004065.2](http://www.ncbi.nlm.nih.gov/entrez/query.fcgi?cmd=Retrieve&db=Protein&list_uids=51873058&dopt=GenPept&RID=T24PVJKM01S&log$=prottop&blast_rank=41) | A kinase (PRKA) anchor protein 2 isoform 1 [Homo sapiens] | [19.3](http://blast.ncbi.nlm.nih.gov/Blast.cgi#51873058%2351873058) | 19.3 | 45% | 267 |
| [NP_001070940.1](http://www.ncbi.nlm.nih.gov/entrez/query.fcgi?cmd=Retrieve&db=Protein&list_uids=117938736&dopt=GenPept&RID=T24PVJKM01S&log$=prottop&blast_rank=42) | constitutive androstane receptor isoform 9 [Homo sapiens] | [19.3](http://blast.ncbi.nlm.nih.gov/Blast.cgi#117938736%23117938736) | 19.3 | 45% | 267 |
| [NP_001070939.1](http://www.ncbi.nlm.nih.gov/entrez/query.fcgi?cmd=Retrieve&db=Protein&list_uids=117938734&dopt=GenPept&RID=T24PVJKM01S&log$=prottop&blast_rank=43) | constitutive androstane receptor isoform 5 [Homo sapiens] | [19.3](http://blast.ncbi.nlm.nih.gov/Blast.cgi#117938734%23117938734) | 19.3 | 45% | 267 |
| [NP_001070938.1](http://www.ncbi.nlm.nih.gov/entrez/query.fcgi?cmd=Retrieve&db=Protein&list_uids=117938732&dopt=GenPept&RID=T24PVJKM01S&log$=prottop&blast_rank=44) | constitutive androstane receptor isoform 11 [Homo sapiens] | [19.3](http://blast.ncbi.nlm.nih.gov/Blast.cgi#117938732%23117938732) | 19.3 | 45% | 267 |
| [NP_001070947.1](http://www.ncbi.nlm.nih.gov/entrez/query.fcgi?cmd=Retrieve&db=Protein&list_uids=117938750&dopt=GenPept&RID=T24PVJKM01S&log$=prottop&blast_rank=45) | constitutive androstane receptor isoform 10 [Homo sapiens] | [19.3](http://blast.ncbi.nlm.nih.gov/Blast.cgi#117938750%23117938750) | 19.3 | 45% | 267 |
| [NP_671492.1](http://www.ncbi.nlm.nih.gov/entrez/query.fcgi?cmd=Retrieve&db=Protein&list_uids=22325356&dopt=GenPept&RID=T24PVJKM01S&log$=prottop&blast_rank=46) | PALM2-AKAP2 protein isoform 2 [Homo sapiens] | [19.3](http://blast.ncbi.nlm.nih.gov/Blast.cgi#22325356%2322325356) | 29.3 | 63% | 267 |
| [NP_006474.1](http://www.ncbi.nlm.nih.gov/entrez/query.fcgi?cmd=Retrieve&db=Protein&list_uids=5921999&dopt=GenPept&RID=T24PVJKM01S&log$=prottop&blast_rank=47) | dual-specificity tyrosine-(Y)-phosphorylation regulated kinase 1B isoform b [Homo sapiens] | [19.3](http://blast.ncbi.nlm.nih.gov/Blast.cgi#5921999%235921999) | 19.3 | 45% | 267 |
| [NP_006475.1](http://www.ncbi.nlm.nih.gov/entrez/query.fcgi?cmd=Retrieve&db=Protein&list_uids=5922001&dopt=GenPept&RID=T24PVJKM01S&log$=prottop&blast_rank=48) | dual-specificity tyrosine-(Y)-phosphorylation regulated kinase 1B isoform c [Homo sapiens] | [19.3](http://blast.ncbi.nlm.nih.gov/Blast.cgi#5922001%235922001) | 19.3 | 45% | 267 |
| [NP_009111.2](http://www.ncbi.nlm.nih.gov/entrez/query.fcgi?cmd=Retrieve&db=Protein&list_uids=116284412&dopt=GenPept&RID=T24PVJKM01S&log$=prottop&blast_rank=49) | trehalase precursor [Homo sapiens] | [19.3](http://blast.ncbi.nlm.nih.gov/Blast.cgi#116284412%23116284412) | 19.3 | 54% | 267 |
| [NP_001005407.1](http://www.ncbi.nlm.nih.gov/entrez/query.fcgi?cmd=Retrieve&db=Protein&list_uids=53832011&dopt=GenPept&RID=T24PVJKM01S&log$=prottop&blast_rank=50) | calcium channel, voltage-dependent, T type, alpha 1H subunit isoform b [Homo sapiens] | [19.3](http://blast.ncbi.nlm.nih.gov/Blast.cgi#53832011%2353832011) | 19.3 | 45% | 267 |
| [NP_958780.1](http://www.ncbi.nlm.nih.gov/entrez/query.fcgi?cmd=Retrieve&db=Protein&list_uids=41322912&dopt=GenPept&RID=T24PVJKM01S&log$=prottop&blast_rank=51) | plectin isoform 1f [Homo sapiens] | [19.3](http://blast.ncbi.nlm.nih.gov/Blast.cgi#41322912%2341322912) | 64.9 | 63% | 267 |
| [NP_958786.1](http://www.ncbi.nlm.nih.gov/entrez/query.fcgi?cmd=Retrieve&db=Protein&list_uids=41322923&dopt=GenPept&RID=T24PVJKM01S&log$=prottop&blast_rank=52) | plectin isoform 1a [Homo sapiens] | [19.3](http://blast.ncbi.nlm.nih.gov/Blast.cgi#41322923%2341322923) | 64.9 | 63% | 267 |
| [NP_000436.2](http://www.ncbi.nlm.nih.gov/entrez/query.fcgi?cmd=Retrieve&db=Protein&list_uids=47607492&dopt=GenPept&RID=T24PVJKM01S&log$=prottop&blast_rank=53) | plectin isoform 1c [Homo sapiens] | [19.3](http://blast.ncbi.nlm.nih.gov/Blast.cgi#47607492%2347607492) | 64.9 | 63% | 267 |
| [NP_958783.1](http://www.ncbi.nlm.nih.gov/entrez/query.fcgi?cmd=Retrieve&db=Protein&list_uids=41322910&dopt=GenPept&RID=T24PVJKM01S&log$=prottop&blast_rank=54) | plectin isoform 1d [Homo sapiens] | [19.3](http://blast.ncbi.nlm.nih.gov/Blast.cgi#41322910%2341322910) | 64.9 | 63% | 267 |
| [NP_958785.1](http://www.ncbi.nlm.nih.gov/entrez/query.fcgi?cmd=Retrieve&db=Protein&list_uids=41322914&dopt=GenPept&RID=T24PVJKM01S&log$=prottop&blast_rank=55) | plectin isoform 1g [Homo sapiens] | [19.3](http://blast.ncbi.nlm.nih.gov/Blast.cgi#41322914%2341322914) | 64.9 | 63% | 267 |
| [NP_958781.1](http://www.ncbi.nlm.nih.gov/entrez/query.fcgi?cmd=Retrieve&db=Protein&list_uids=41322908&dopt=GenPept&RID=T24PVJKM01S&log$=prottop&blast_rank=56) | plectin isoform 1e [Homo sapiens] | [19.3](http://blast.ncbi.nlm.nih.gov/Blast.cgi#41322908%2341322908) | 64.9 | 63% | 267 |
| [NP_958782.1](http://www.ncbi.nlm.nih.gov/entrez/query.fcgi?cmd=Retrieve&db=Protein&list_uids=41322916&dopt=GenPept&RID=T24PVJKM01S&log$=prottop&blast_rank=57) | plectin isoform 1 [Homo sapiens] | [19.3](http://blast.ncbi.nlm.nih.gov/Blast.cgi#41322916%2341322916) | 64.9 | 63% | 267 |
| [NP_958784.1](http://www.ncbi.nlm.nih.gov/entrez/query.fcgi?cmd=Retrieve&db=Protein&list_uids=41322919&dopt=GenPept&RID=T24PVJKM01S&log$=prottop&blast_rank=58) | plectin isoform 1b [Homo sapiens] | [19.3](http://blast.ncbi.nlm.nih.gov/Blast.cgi#41322919%2341322919) | 64.9 | 63% | 267 |
| [NP_116208.3](http://www.ncbi.nlm.nih.gov/entrez/query.fcgi?cmd=Retrieve&db=Protein&list_uids=40807465&dopt=GenPept&RID=T24PVJKM01S&log$=prottop&blast_rank=59) | zinc finger protein 341 [Homo sapiens] | [19.3](http://blast.ncbi.nlm.nih.gov/Blast.cgi#40807465%2340807465) | 19.3 | 54% | 267 |
| [NP_149062.1](http://www.ncbi.nlm.nih.gov/entrez/query.fcgi?cmd=Retrieve&db=Protein&list_uids=23097308&dopt=GenPept&RID=T24PVJKM01S&log$=prottop&blast_rank=60) | spectrin repeat containing, nuclear envelope 1 isoform 2 [Homo sapiens] | [19.3](http://blast.ncbi.nlm.nih.gov/Blast.cgi#23097308%2323097308) | 35.2 | 45% | 267 |
| [NP_056108.2](http://www.ncbi.nlm.nih.gov/entrez/query.fcgi?cmd=Retrieve&db=Protein&list_uids=154277118&dopt=GenPept&RID=T24PVJKM01S&log$=prottop&blast_rank=61) | spectrin repeat containing, nuclear envelope 1 isoform 4 [Homo sapiens] | [19.3](http://blast.ncbi.nlm.nih.gov/Blast.cgi#154277118%23154277118) | 19.3 | 45% | 267 |
| [NP_001070948.1](http://www.ncbi.nlm.nih.gov/entrez/query.fcgi?cmd=Retrieve&db=Protein&list_uids=117938752&dopt=GenPept&RID=T24PVJKM01S&log$=prottop&blast_rank=62) | constitutive androstane receptor isoform 2 [Homo sapiens] | [19.3](http://blast.ncbi.nlm.nih.gov/Blast.cgi#117938752%23117938752) | 19.3 | 45% | 267 |
| [NP_004705.1](http://www.ncbi.nlm.nih.gov/entrez/query.fcgi?cmd=Retrieve&db=Protein&list_uids=4758222&dopt=GenPept&RID=T24PVJKM01S&log$=prottop&blast_rank=63) | dual-specificity tyrosine-(Y)-phosphorylation regulated kinase 1B isoform a [Homo sapiens] | [19.3](http://blast.ncbi.nlm.nih.gov/Blast.cgi#4758222%234758222) | 19.3 | 45% | 267 |
| [NP_009134.1](http://www.ncbi.nlm.nih.gov/entrez/query.fcgi?cmd=Retrieve&db=Protein&list_uids=6005709&dopt=GenPept&RID=T24PVJKM01S&log$=prottop&blast_rank=64) | PALM2-AKAP2 protein isoform 1 [Homo sapiens] | [19.3](http://blast.ncbi.nlm.nih.gov/Blast.cgi#6005709%236005709) | 29.3 | 63% | 267 |
| [NP_005113.1](http://www.ncbi.nlm.nih.gov/entrez/query.fcgi?cmd=Retrieve&db=Protein&list_uids=4826661&dopt=GenPept&RID=T24PVJKM01S&log$=prottop&blast_rank=65) | constitutive androstane receptor isoform 3 [Homo sapiens] | [19.3](http://blast.ncbi.nlm.nih.gov/Blast.cgi#4826661%234826661) | 19.3 | 45% | 267 |
| [NP_056383.1](http://www.ncbi.nlm.nih.gov/entrez/query.fcgi?cmd=Retrieve&db=Protein&list_uids=28626517&dopt=GenPept&RID=T24PVJKM01S&log$=prottop&blast_rank=66) | protein phosphatase 1 regulatory inhibitor subunit 16B isoform 1 [Homo sapiens] | [19.3](http://blast.ncbi.nlm.nih.gov/Blast.cgi#28626517%2328626517) | 19.3 | 45% | 267 |
| [NP_004231.1](http://www.ncbi.nlm.nih.gov/entrez/query.fcgi?cmd=Retrieve&db=Protein&list_uids=11342676&dopt=GenPept&RID=T24PVJKM01S&log$=prottop&blast_rank=67) | thyroid hormone receptor interactor 10 [Homo sapiens] | [19.3](http://blast.ncbi.nlm.nih.gov/Blast.cgi#11342676%2311342676) | 33.1 | 72% | 267 |
| [NP_511042.1](http://www.ncbi.nlm.nih.gov/entrez/query.fcgi?cmd=Retrieve&db=Protein&list_uids=17981696&dopt=GenPept&RID=T24PVJKM01S&log$=prottop&blast_rank=68) | cyclin-dependent kinase inhibitor 2B isoform 2 [Homo sapiens] | [19.3](http://blast.ncbi.nlm.nih.gov/Blast.cgi#17981696%2317981696) | 19.3 | 90% | 267 |
| [NP_066921.2](http://www.ncbi.nlm.nih.gov/entrez/query.fcgi?cmd=Retrieve&db=Protein&list_uids=53832009&dopt=GenPept&RID=T24PVJKM01S&log$=prottop&blast_rank=69) | calcium channel, voltage-dependent, T type, alpha 1H subunit isoform a [Homo sapiens] | [19.3](http://blast.ncbi.nlm.nih.gov/Blast.cgi#53832009%2353832009) | 19.3 | 45% | 267 |
| [NP_057105.2](http://www.ncbi.nlm.nih.gov/entrez/query.fcgi?cmd=Retrieve&db=Protein&list_uids=37577122&dopt=GenPept&RID=T24PVJKM01S&log$=prottop&blast_rank=70) | ubiquitin-conjugating enzyme E2, J1 [Homo sapiens] | [19.3](http://blast.ncbi.nlm.nih.gov/Blast.cgi#37577122%2337577122) | 19.3 | 45% | 267 |
| [NP_004970.3](http://www.ncbi.nlm.nih.gov/entrez/query.fcgi?cmd=Retrieve&db=Protein&list_uids=27436981&dopt=GenPept&RID=T24PVJKM01S&log$=prottop&blast_rank=71) | potassium voltage-gated channel, Shal-related subfamily, member 1 [Homo sapiens] | [19.3](http://blast.ncbi.nlm.nih.gov/Blast.cgi#27436981%2327436981) | 30.5 | 63% | 267 |
| [NP_001166101.1](http://www.ncbi.nlm.nih.gov/entrez/query.fcgi?cmd=Retrieve&db=Protein&list_uids=289547507&dopt=GenPept&RID=T24PVJKM01S&log$=prottop&blast_rank=72) | TC10/CDC42 GTPase-activating protein isoform 2 [Homo sapiens] | [18.9](http://blast.ncbi.nlm.nih.gov/Blast.cgi#289547507%23289547507) | 18.9 | 45% | 359 |
| [NP_001159586.1](http://www.ncbi.nlm.nih.gov/entrez/query.fcgi?cmd=Retrieve&db=Protein&list_uids=260656043&dopt=GenPept&RID=T24PVJKM01S&log$=prottop&blast_rank=73) | neuropathy target esterase isoform d [Homo sapiens] | [18.9](http://blast.ncbi.nlm.nih.gov/Blast.cgi#260656043%23260656043) | 61.9 | 72% | 359 |
| [NP_001159584.1](http://www.ncbi.nlm.nih.gov/entrez/query.fcgi?cmd=Retrieve&db=Protein&list_uids=260656039&dopt=GenPept&RID=T24PVJKM01S&log$=prottop&blast_rank=74) | neuropathy target esterase isoform c [Homo sapiens] | [18.9](http://blast.ncbi.nlm.nih.gov/Blast.cgi#260656039%23260656039) | 61.9 | 72% | 359 |
| [NP_001159583.1](http://www.ncbi.nlm.nih.gov/entrez/query.fcgi?cmd=Retrieve&db=Protein&list_uids=260656037&dopt=GenPept&RID=T24PVJKM01S&log$=prottop&blast_rank=75) | neuropathy target esterase isoform a [Homo sapiens] | [18.9](http://blast.ncbi.nlm.nih.gov/Blast.cgi#260656037%23260656037) | 61.9 | 72% | 359 |
| [XP_002344233.1](http://www.ncbi.nlm.nih.gov/entrez/query.fcgi?cmd=Retrieve&db=Protein&list_uids=239746618&dopt=GenPept&RID=T24PVJKM01S&log$=prottop&blast_rank=76) | PREDICTED: hypothetical protein XP_002344233 [Homo sapiens] >ref|XP_002348117.1| PREDICTED: hypothetical protein XP_002348117 [Homo sapiens] >ref|XP_002345501.1| PREDICTED: hypothetical protein [Homo sapiens] | [18.9](http://blast.ncbi.nlm.nih.gov/Blast.cgi#239746618%23239746618) | 31.4 | 81% | 359 |
| [XP_002342915.1](http://www.ncbi.nlm.nih.gov/entrez/query.fcgi?cmd=Retrieve&db=Protein&list_uids=239743615&dopt=GenPept&RID=T24PVJKM01S&log$=prottop&blast_rank=77) | PREDICTED: hypothetical protein XP_002342915 [Homo sapiens] >ref|XP_002347062.1| PREDICTED: hypothetical protein XP_002347062 [Homo sapiens] >ref|XP_002346162.1| PREDICTED: hypothetical protein [Homo sapiens] | [18.9](http://blast.ncbi.nlm.nih.gov/Blast.cgi#239743615%23239743615) | 18.9 | 45% | 359 |
| [NP_001139681.1](http://www.ncbi.nlm.nih.gov/entrez/query.fcgi?cmd=Retrieve&db=Protein&list_uids=226246591&dopt=GenPept&RID=T24PVJKM01S&log$=prottop&blast_rank=78) | phosphodiesterase 2A isoform 3 [Homo sapiens] | [18.9](http://blast.ncbi.nlm.nih.gov/Blast.cgi#226246591%23226246591) | 30.5 | 81% | 359 |
| [NP_001129108.1](http://www.ncbi.nlm.nih.gov/entrez/query.fcgi?cmd=Retrieve&db=Protein&list_uids=208431774&dopt=GenPept&RID=T24PVJKM01S&log$=prottop&blast_rank=79) | phosphatidylinositol-4-phosphate 5-kinase, type I, alpha isoform 3 [Homo sapiens] | [18.9](http://blast.ncbi.nlm.nih.gov/Blast.cgi#208431774%23208431774) | 18.9 | 45% | 359 |
| [NP_110434.3](http://www.ncbi.nlm.nih.gov/entrez/query.fcgi?cmd=Retrieve&db=Protein&list_uids=190684655&dopt=GenPept&RID=T24PVJKM01S&log$=prottop&blast_rank=80) | glucose transporter protein 10 isoform a [Homo sapiens] | [18.9](http://blast.ncbi.nlm.nih.gov/Blast.cgi#190684655%23190684655) | 18.9 | 54% | 359 |
| [XP_943402.3](http://www.ncbi.nlm.nih.gov/entrez/query.fcgi?cmd=Retrieve&db=Protein&list_uids=169216732&dopt=GenPept&RID=T24PVJKM01S&log$=prottop&blast_rank=81) | PREDICTED: similar to hCG1645335 [Homo sapiens] | [18.9](http://blast.ncbi.nlm.nih.gov/Blast.cgi#169216732%23169216732) | 18.9 | 54% | 359 |
| [XP_001715277.1](http://www.ncbi.nlm.nih.gov/entrez/query.fcgi?cmd=Retrieve&db=Protein&list_uids=169171483&dopt=GenPept&RID=T24PVJKM01S&log$=prottop&blast_rank=82) | PREDICTED: hypothetical protein [Homo sapiens] | [18.9](http://blast.ncbi.nlm.nih.gov/Blast.cgi#169171483%23169171483) | 18.9 | 54% | 359 |
| [NP_663327.2](http://www.ncbi.nlm.nih.gov/entrez/query.fcgi?cmd=Retrieve&db=Protein&list_uids=145309306&dopt=GenPept&RID=T24PVJKM01S&log$=prottop&blast_rank=83) | scavenger receptor class F, member 1 isoform 5 precursor [Homo sapiens] | [18.9](http://blast.ncbi.nlm.nih.gov/Blast.cgi#145309306%23145309306) | 30.1 | 54% | 359 |
| [NP_116068.2](http://www.ncbi.nlm.nih.gov/entrez/query.fcgi?cmd=Retrieve&db=Protein&list_uids=207450723&dopt=GenPept&RID=T24PVJKM01S&log$=prottop&blast_rank=84) | zinc finger protein 577 isoform a [Homo sapiens] | [18.9](http://blast.ncbi.nlm.nih.gov/Blast.cgi#207450723%23207450723) | 18.9 | 54% | 359 |
| [NP_001129062.1](http://www.ncbi.nlm.nih.gov/entrez/query.fcgi?cmd=Retrieve&db=Protein&list_uids=207450725&dopt=GenPept&RID=T24PVJKM01S&log$=prottop&blast_rank=85) | zinc finger protein 577 isoform b [Homo sapiens] | [18.9](http://blast.ncbi.nlm.nih.gov/Blast.cgi#207450725%23207450725) | 18.9 | 54% | 359 |
| [XP_001717830.1](http://www.ncbi.nlm.nih.gov/entrez/query.fcgi?cmd=Retrieve&db=Protein&list_uids=169216507&dopt=GenPept&RID=T24PVJKM01S&log$=prottop&blast_rank=86) | PREDICTED: similar to hCG1645335 [Homo sapiens] | [18.9](http://blast.ncbi.nlm.nih.gov/Blast.cgi#169216507%23169216507) | 18.9 | 54% | 359 |
| [NP_055455.3](http://www.ncbi.nlm.nih.gov/entrez/query.fcgi?cmd=Retrieve&db=Protein&list_uids=217330594&dopt=GenPept&RID=T24PVJKM01S&log$=prottop&blast_rank=87) | tubulin tyrosine ligase-like family, member 4 [Homo sapiens] | [18.9](http://blast.ncbi.nlm.nih.gov/Blast.cgi#217330594%23217330594) | 18.9 | 54% | 359 |
| [NP_821078.1](http://www.ncbi.nlm.nih.gov/entrez/query.fcgi?cmd=Retrieve&db=Protein&list_uids=30061558&dopt=GenPept&RID=T24PVJKM01S&log$=prottop&blast_rank=88) | SRY (sex determining region Y)-box 5 isoform c [Homo sapiens] | [18.9](http://blast.ncbi.nlm.nih.gov/Blast.cgi#30061558%2330061558) | 18.9 | 54% | 359 |
| [NP_002327.2](http://www.ncbi.nlm.nih.gov/entrez/query.fcgi?cmd=Retrieve&db=Protein&list_uids=148727288&dopt=GenPept&RID=T24PVJKM01S&log$=prottop&blast_rank=89) | low density lipoprotein receptor-related protein 6 precursor [Homo sapiens] | [18.9](http://blast.ncbi.nlm.nih.gov/Blast.cgi#148727288%23148727288) | 29.3 | 90% | 359 |
| [NP_001438.1](http://www.ncbi.nlm.nih.gov/entrez/query.fcgi?cmd=Retrieve&db=Protein&list_uids=13787217&dopt=GenPept&RID=T24PVJKM01S&log$=prottop&blast_rank=90) | FAT tumor suppressor 2 precursor [Homo sapiens] | [18.9](http://blast.ncbi.nlm.nih.gov/Blast.cgi#13787217%2313787217) | 18.9 | 54% | 359 |
| [NP_001020110.1](http://www.ncbi.nlm.nih.gov/entrez/query.fcgi?cmd=Retrieve&db=Protein&list_uids=68226418&dopt=GenPept&RID=T24PVJKM01S&log$=prottop&blast_rank=91) | glucose transporter protein 10 isoform b [Homo sapiens] | [18.9](http://blast.ncbi.nlm.nih.gov/Blast.cgi#68226418%2368226418) | 18.9 | 54% | 359 |
| [NP_001165697.1](http://www.ncbi.nlm.nih.gov/entrez/query.fcgi?cmd=Retrieve&db=Protein&list_uids=288557353&dopt=GenPept&RID=T24PVJKM01S&log$=prottop&blast_rank=92) | zinc finger protein 540 isoform b [Homo sapiens] | [18.9](http://blast.ncbi.nlm.nih.gov/Blast.cgi#288557353%23288557353) | 18.9 | 54% | 359 |
| [NP_612444.2](http://www.ncbi.nlm.nih.gov/entrez/query.fcgi?cmd=Retrieve&db=Protein&list_uids=156564372&dopt=GenPept&RID=T24PVJKM01S&log$=prottop&blast_rank=93) | hypothetical protein LOC113828 [Homo sapiens] | [18.9](http://blast.ncbi.nlm.nih.gov/Blast.cgi#156564372%23156564372) | 18.9 | 45% | 359 |
| [NP_443180.2](http://www.ncbi.nlm.nih.gov/entrez/query.fcgi?cmd=Retrieve&db=Protein&list_uids=23510413&dopt=GenPept&RID=T24PVJKM01S&log$=prottop&blast_rank=94) | TC10/CDC42 GTPase-activating protein isoform 1 [Homo sapiens] | [18.9](http://blast.ncbi.nlm.nih.gov/Blast.cgi#23510413%2323510413) | 18.9 | 45% | 359 |
| [NP_003684.2](http://www.ncbi.nlm.nih.gov/entrez/query.fcgi?cmd=Retrieve&db=Protein&list_uids=33598929&dopt=GenPept&RID=T24PVJKM01S&log$=prottop&blast_rank=95) | scavenger receptor class F, member 1 isoform 1 precursor [Homo sapiens] | [18.9](http://blast.ncbi.nlm.nih.gov/Blast.cgi#33598929%2333598929) | 30.1 | 54% | 359 |
| [NP_078795.2](http://www.ncbi.nlm.nih.gov/entrez/query.fcgi?cmd=Retrieve&db=Protein&list_uids=39995080&dopt=GenPept&RID=T24PVJKM01S&log$=prottop&blast_rank=96) | hypothetical protein LOC79567 [Homo sapiens] | [18.9](http://blast.ncbi.nlm.nih.gov/Blast.cgi#39995080%2339995080) | 18.9 | 72% | 359 |
| [NP_665806.1](http://www.ncbi.nlm.nih.gov/entrez/query.fcgi?cmd=Retrieve&db=Protein&list_uids=24308065&dopt=GenPept&RID=T24PVJKM01S&log$=prottop&blast_rank=97) | netrin 5 precursor [Homo sapiens] | [18.9](http://blast.ncbi.nlm.nih.gov/Blast.cgi#24308065%2324308065) | 18.9 | 45% | 359 |
| [NP_689819.1](http://www.ncbi.nlm.nih.gov/entrez/query.fcgi?cmd=Retrieve&db=Protein&list_uids=22749241&dopt=GenPept&RID=T24PVJKM01S&log$=prottop&blast_rank=98) | zinc finger protein 540 isoform a [Homo sapiens] >ref|NP_001165696.1| zinc finger protein 540 isoform a [Homo sapiens] | [18.9](http://blast.ncbi.nlm.nih.gov/Blast.cgi#22749241%2322749241) | 18.9 | 54% | 359 |
| [NP_001027544.1](http://www.ncbi.nlm.nih.gov/entrez/query.fcgi?cmd=Retrieve&db=Protein&list_uids=74027274&dopt=GenPept&RID=T24PVJKM01S&log$=prottop&blast_rank=99) | zinc finger protein 226 isoform a [Homo sapiens] >ref|NP_001027545.1| zinc finger protein 226 isoform a [Homo sapiens] | [18.9](http://blast.ncbi.nlm.nih.gov/Blast.cgi#74027274%2374027274) | 35.2 | 45% | 359 |
| [NP_000266.2](http://www.ncbi.nlm.nih.gov/entrez/query.fcgi?cmd=Retrieve&db=Protein&list_uids=157266326&dopt=GenPept&RID=T24PVJKM01S&log$=prottop&blast_rank=100) | oculocutaneous albinism II [Homo sapiens] | [18.9](http://blast.ncbi.nlm.nih.gov/Blast.cgi#157266326%23157266326) | 18.9 | 54% | 359 |

| **Accession** | **Proteins with a match to DHNRSM peptide** | **[Max score](http://blast.ncbi.nlm.nih.gov/Blast.cgi?CMD=Get&ALIGNMENTS=100&ALIGNMENT_VIEW=Pairwise&CDD_SEARCH_STATE=1&DATABASE_SORT=0&DESCRIPTIONS=100&ENTREZ_QUERY=txid9606 %5BORGN%5D&FIRST_QUERY_NUM=0&FORMAT_OBJECT=Alignment&FORMAT_PAGE_TARGET=&FORMAT_TYPE=HTML&GET_SEQUENCE=yes&I_THRESH=&MASK_CHAR=2&MASK_COLOR=1&NEW_DESIGN=on&NEW_VIEW=yes&NUM_OVERVIEW=100&OLD_BLAST=false&PAGE=Proteins&QUERY_INDEX=0&QUERY_NUMBER=0&RESULTS_PAGE_TARGET=&RID=T250N4CW01N&SHOW_LINKOUT=yes&SHOW_OVERVIEW=yes&STEP_NUMBER=&WORD_SIZE=2&DISPLAY_SORT=1&HSP_SORT=1" \l "sort_mark)** | **[Total score](http://blast.ncbi.nlm.nih.gov/Blast.cgi?CMD=Get&ALIGNMENTS=100&ALIGNMENT_VIEW=Pairwise&CDD_SEARCH_STATE=1&DATABASE_SORT=0&DESCRIPTIONS=100&ENTREZ_QUERY=txid9606 %5BORGN%5D&FIRST_QUERY_NUM=0&FORMAT_OBJECT=Alignment&FORMAT_PAGE_TARGET=&FORMAT_TYPE=HTML&GET_SEQUENCE=yes&I_THRESH=&MASK_CHAR=2&MASK_COLOR=1&NEW_DESIGN=on&NEW_VIEW=yes&NUM_OVERVIEW=100&OLD_BLAST=false&PAGE=Proteins&QUERY_INDEX=0&QUERY_NUMBER=0&RESULTS_PAGE_TARGET=&RID=T250N4CW01N&SHOW_LINKOUT=yes&SHOW_OVERVIEW=yes&STEP_NUMBER=&WORD_SIZE=2&DISPLAY_SORT=2&HSP_SORT=1" \l "sort_mark)** | **[Query coverage](http://blast.ncbi.nlm.nih.gov/Blast.cgi?CMD=Get&ALIGNMENTS=100&ALIGNMENT_VIEW=Pairwise&CDD_SEARCH_STATE=1&DATABASE_SORT=0&DESCRIPTIONS=100&ENTREZ_QUERY=txid9606 %5BORGN%5D&FIRST_QUERY_NUM=0&FORMAT_OBJECT=Alignment&FORMAT_PAGE_TARGET=&FORMAT_TYPE=HTML&GET_SEQUENCE=yes&I_THRESH=&MASK_CHAR=2&MASK_COLOR=1&NEW_DESIGN=on&NEW_VIEW=yes&NUM_OVERVIEW=100&OLD_BLAST=false&PAGE=Proteins&QUERY_INDEX=0&QUERY_NUMBER=0&RESULTS_PAGE_TARGET=&RID=T250N4CW01N&SHOW_LINKOUT=yes&SHOW_OVERVIEW=yes&STEP_NUMBER=&WORD_SIZE=2&DISPLAY_SORT=4&HSP_SORT=0" \l "sort_mark)** | **[E value](http://blast.ncbi.nlm.nih.gov/Blast.cgi?CMD=Get&ALIGNMENTS=100&ALIGNMENT_VIEW=Pairwise&CDD_SEARCH_STATE=1&DATABASE_SORT=0&DESCRIPTIONS=100&ENTREZ_QUERY=txid9606 %5BORGN%5D&FIRST_QUERY_NUM=0&FORMAT_OBJECT=Alignment&FORMAT_PAGE_TARGET=&FORMAT_TYPE=HTML&GET_SEQUENCE=yes&I_THRESH=&MASK_CHAR=2&MASK_COLOR=1&NEW_DESIGN=on&NEW_VIEW=yes&NUM_OVERVIEW=100&OLD_BLAST=false&PAGE=Proteins&QUERY_INDEX=0&QUERY_NUMBER=0&RESULTS_PAGE_TARGET=&RID=T250N4CW01N&SHOW_LINKOUT=yes&SHOW_OVERVIEW=yes&STEP_NUMBER=&WORD_SIZE=2&DISPLAY_SORT=0&HSP_SORT=0" \l "sort_mark)** |
| --- | --- | --- | --- | --- | --- |
| [XP_001130201.3](http://www.ncbi.nlm.nih.gov/entrez/query.fcgi?cmd=Retrieve&db=Protein&list_uids=239745902&dopt=GenPept&RID=T250N4CW01N&log$=prottop&blast_rank=1) | PREDICTED: hypothetical protein [Homo sapiens] | [21.0](http://blast.ncbi.nlm.nih.gov/Blast.cgi#239745902%23239745902) | 21.0 | 100% | 50 |
| [XP_001130175.1](http://www.ncbi.nlm.nih.gov/entrez/query.fcgi?cmd=Retrieve&db=Protein&list_uids=113427491&dopt=GenPept&RID=T250N4CW01N&log$=prottop&blast_rank=2) | PREDICTED: hypothetical protein [Homo sapiens] >ref|XP_001717001.2| PREDICTED: hypothetical protein [Homo sapiens] | [21.0](http://blast.ncbi.nlm.nih.gov/Blast.cgi#113427491%23113427491) | 21.0 | 100% | 50 |
| [NP_061867.1](http://www.ncbi.nlm.nih.gov/entrez/query.fcgi?cmd=Retrieve&db=Protein&list_uids=62955044&dopt=GenPept&RID=T250N4CW01N&log$=prottop&blast_rank=3) | F-box protein 42 [Homo sapiens] | [21.0](http://blast.ncbi.nlm.nih.gov/Blast.cgi#62955044%2362955044) | 21.0 | 83% | 50 |
| [NP_689525.3](http://www.ncbi.nlm.nih.gov/entrez/query.fcgi?cmd=Retrieve&db=Protein&list_uids=116235482&dopt=GenPept&RID=T250N4CW01N&log$=prottop&blast_rank=4) | glycosyltransferase-like 1B [Homo sapiens] | [19.7](http://blast.ncbi.nlm.nih.gov/Blast.cgi#116235482%23116235482) | 19.7 | 83% | 120 |
| [NP_001348.2](http://www.ncbi.nlm.nih.gov/entrez/query.fcgi?cmd=Retrieve&db=Protein&list_uids=100913206&dopt=GenPept&RID=T250N4CW01N&log$=prottop&blast_rank=5) | ATP-dependent RNA helicase A [Homo sapiens] | [19.7](http://blast.ncbi.nlm.nih.gov/Blast.cgi#100913206%23100913206) | 19.7 | 83% | 120 |
| [NP_037534.3](http://www.ncbi.nlm.nih.gov/entrez/query.fcgi?cmd=Retrieve&db=Protein&list_uids=214831730&dopt=GenPept&RID=T250N4CW01N&log$=prottop&blast_rank=6) | fatty acid desaturase 1 [Homo sapiens] | [19.3](http://blast.ncbi.nlm.nih.gov/Blast.cgi#214831730%23214831730) | 19.3 | 100% | 161 |
| [XP_002347299.1](http://www.ncbi.nlm.nih.gov/entrez/query.fcgi?cmd=Retrieve&db=Protein&list_uids=239750143&dopt=GenPept&RID=T250N4CW01N&log$=prottop&blast_rank=7) | PREDICTED: tripartite motif-containing 77 [Homo sapiens] >ref|XP_002344647.1| PREDICTED: tripartite motif-containing 77 [Homo sapiens] | [18.5](http://blast.ncbi.nlm.nih.gov/Blast.cgi#239750143%23239750143) | 18.5 | 83% | 289 |
| [NP_001015049.1](http://www.ncbi.nlm.nih.gov/entrez/query.fcgi?cmd=Retrieve&db=Protein&list_uids=62548856&dopt=GenPept&RID=T250N4CW01N&log$=prottop&blast_rank=8) | BCL2-associated athanogene 5 isoform a [Homo sapiens] | [18.5](http://blast.ncbi.nlm.nih.gov/Blast.cgi#62548856%2362548856) | 18.5 | 100% | 289 |
| [NP_054907.1](http://www.ncbi.nlm.nih.gov/entrez/query.fcgi?cmd=Retrieve&db=Protein&list_uids=7661832&dopt=GenPept&RID=T250N4CW01N&log$=prottop&blast_rank=9) | Ssu72 RNA polymerase II CTD phosphatase homolog [Homo sapiens] | [18.5](http://blast.ncbi.nlm.nih.gov/Blast.cgi#7661832%237661832) | 18.5 | 100% | 289 |
| [XP_496693.4](http://www.ncbi.nlm.nih.gov/entrez/query.fcgi?cmd=Retrieve&db=Protein&list_uids=169166418&dopt=GenPept&RID=T250N4CW01N&log$=prottop&blast_rank=10) | PREDICTED: similar to COMM domain containing 5 [Homo sapiens] >ref|XP_941557.3| PREDICTED: similar to COMM domain containing 5 [Homo sapiens] >ref|XP_001714919.1| PREDICTED: similar to COMM domain containing 5 [Homo sapiens] | [18.0](http://blast.ncbi.nlm.nih.gov/Blast.cgi#169166418%23169166418) | 18.0 | 100% | 388 |
| [NP_573400.3](http://www.ncbi.nlm.nih.gov/entrez/query.fcgi?cmd=Retrieve&db=Protein&list_uids=148539858&dopt=GenPept&RID=T250N4CW01N&log$=prottop&blast_rank=11) | protein tyrosine phosphatase, receptor type, T isoform 1 precursor [Homo sapiens] | [18.0](http://blast.ncbi.nlm.nih.gov/Blast.cgi#148539858%23148539858) | 18.0 | 100% | 388 |
| [NP_573438.2](http://www.ncbi.nlm.nih.gov/entrez/query.fcgi?cmd=Retrieve&db=Protein&list_uids=110735406&dopt=GenPept&RID=T250N4CW01N&log$=prottop&blast_rank=12) | protein tyrosine phosphatase, receptor type, U isoform 2 precursor [Homo sapiens] | [18.0](http://blast.ncbi.nlm.nih.gov/Blast.cgi#110735406%23110735406) | 18.0 | 100% | 388 |
| [NP_008981.4](http://www.ncbi.nlm.nih.gov/entrez/query.fcgi?cmd=Retrieve&db=Protein&list_uids=148539860&dopt=GenPept&RID=T250N4CW01N&log$=prottop&blast_rank=13) | protein tyrosine phosphatase, receptor type, T isoform 2 precursor [Homo sapiens] | [18.0](http://blast.ncbi.nlm.nih.gov/Blast.cgi#148539860%23148539860) | 18.0 | 100% | 388 |
| [NP_573439.2](http://www.ncbi.nlm.nih.gov/entrez/query.fcgi?cmd=Retrieve&db=Protein&list_uids=110735404&dopt=GenPept&RID=T250N4CW01N&log$=prottop&blast_rank=14) | protein tyrosine phosphatase, receptor type, U isoform 1 precursor [Homo sapiens] | [18.0](http://blast.ncbi.nlm.nih.gov/Blast.cgi#110735404%23110735404) | 18.0 | 100% | 388 |
| [NP_055525.3](http://www.ncbi.nlm.nih.gov/entrez/query.fcgi?cmd=Retrieve&db=Protein&list_uids=103472136&dopt=GenPept&RID=T250N4CW01N&log$=prottop&blast_rank=15) | G protein-coupled receptor associated sorting protein 1 [Homo sapiens] >ref|NP_001092880.1| G protein-coupled receptor associated sorting protein 1 [Homo sapiens] >ref|NP_001092881.1| G protein-coupled receptor associated sorting protein 1 [Homo sapiens] | [18.0](http://blast.ncbi.nlm.nih.gov/Blast.cgi#103472136%23103472136) | 18.0 | 100% | 388 |
| [NP_005695.3](http://www.ncbi.nlm.nih.gov/entrez/query.fcgi?cmd=Retrieve&db=Protein&list_uids=110735402&dopt=GenPept&RID=T250N4CW01N&log$=prottop&blast_rank=16) | protein tyrosine phosphatase, receptor type, U isoform 3 precursor [Homo sapiens] | [18.0](http://blast.ncbi.nlm.nih.gov/Blast.cgi#110735402%23110735402) | 18.0 | 100% | 388 |
| [NP_056343.1](http://www.ncbi.nlm.nih.gov/entrez/query.fcgi?cmd=Retrieve&db=Protein&list_uids=14149702&dopt=GenPept&RID=T250N4CW01N&log$=prottop&blast_rank=17) | ring finger protein 167 precursor [Homo sapiens] | [18.0](http://blast.ncbi.nlm.nih.gov/Blast.cgi#14149702%2314149702) | 18.0 | 100% | 388 |
| [NP_742067.3](http://www.ncbi.nlm.nih.gov/entrez/query.fcgi?cmd=Retrieve&db=Protein&list_uids=160948610&dopt=GenPept&RID=T250N4CW01N&log$=prottop&blast_rank=18) | E3 ubiquitin-protein ligase UBR3 [Homo sapiens] | [17.6](http://blast.ncbi.nlm.nih.gov/Blast.cgi#160948610%23160948610) | 17.6 | 100% | 521 |
| [NP_001098989.1](http://www.ncbi.nlm.nih.gov/entrez/query.fcgi?cmd=Retrieve&db=Protein&list_uids=157502167&dopt=GenPept&RID=T250N4CW01N&log$=prottop&blast_rank=19) | hypothetical protein LOC339778 [Homo sapiens] | [17.6](http://blast.ncbi.nlm.nih.gov/Blast.cgi#157502167%23157502167) | 17.6 | 100% | 521 |
| [NP_001073883.2](http://www.ncbi.nlm.nih.gov/entrez/query.fcgi?cmd=Retrieve&db=Protein&list_uids=148762940&dopt=GenPept&RID=T250N4CW01N&log$=prottop&blast_rank=20) | DVL-binding protein DAPLE [Homo sapiens] | [17.6](http://blast.ncbi.nlm.nih.gov/Blast.cgi#148762940%23148762940) | 17.6 | 100% | 521 |
| [NP_001078933.1](http://www.ncbi.nlm.nih.gov/entrez/query.fcgi?cmd=Retrieve&db=Protein&list_uids=146231991&dopt=GenPept&RID=T250N4CW01N&log$=prottop&blast_rank=21) | catenin, delta 1 isoform 3AB [Homo sapiens] | [17.6](http://blast.ncbi.nlm.nih.gov/Blast.cgi#146231991%23146231991) | 17.6 | 100% | 521 |
| [NP_001078937.1](http://www.ncbi.nlm.nih.gov/entrez/query.fcgi?cmd=Retrieve&db=Protein&list_uids=146231948&dopt=GenPept&RID=T250N4CW01N&log$=prottop&blast_rank=22) | catenin, delta 1 isoform 3A [Homo sapiens] >ref|NP_001078938.1| catenin, delta 1 isoform 3A [Homo sapiens] >ref|NP_001078936.1| catenin, delta 1 isoform 3A [Homo sapiens] | [17.6](http://blast.ncbi.nlm.nih.gov/Blast.cgi#146231948%23146231948) | 17.6 | 100% | 521 |
| [NP_001078929.1](http://www.ncbi.nlm.nih.gov/entrez/query.fcgi?cmd=Retrieve&db=Protein&list_uids=146231938&dopt=GenPept&RID=T250N4CW01N&log$=prottop&blast_rank=23) | catenin, delta 1 isoform 1A [Homo sapiens] >ref|NP_001078931.1| catenin, delta 1 isoform 1A [Homo sapiens] >ref|NP_001078930.1| catenin, delta 1 isoform 1A [Homo sapiens] | [17.6](http://blast.ncbi.nlm.nih.gov/Blast.cgi#146231938%23146231938) | 17.6 | 100% | 521 |
| [NP_001322.1](http://www.ncbi.nlm.nih.gov/entrez/query.fcgi?cmd=Retrieve&db=Protein&list_uids=10835010&dopt=GenPept&RID=T250N4CW01N&log$=prottop&blast_rank=24) | catenin, delta 1 isoform 1B [Homo sapiens] | [17.6](http://blast.ncbi.nlm.nih.gov/Blast.cgi#10835010%2310835010) | 17.6 | 100% | 521 |
| [NP_001078934.1](http://www.ncbi.nlm.nih.gov/entrez/query.fcgi?cmd=Retrieve&db=Protein&list_uids=146231946&dopt=GenPept&RID=T250N4CW01N&log$=prottop&blast_rank=25) | catenin, delta 1 isoform 3B [Homo sapiens] | [17.6](http://blast.ncbi.nlm.nih.gov/Blast.cgi#146231946%23146231946) | 17.6 | 100% | 521 |
| [NP_001078932.1](http://www.ncbi.nlm.nih.gov/entrez/query.fcgi?cmd=Retrieve&db=Protein&list_uids=146231970&dopt=GenPept&RID=T250N4CW01N&log$=prottop&blast_rank=26) | catenin, delta 1 isoform 3ABC [Homo sapiens] | [17.6](http://blast.ncbi.nlm.nih.gov/Blast.cgi#146231970%23146231970) | 17.6 | 100% | 521 |
| [NP_001078935.1](http://www.ncbi.nlm.nih.gov/entrez/query.fcgi?cmd=Retrieve&db=Protein&list_uids=146231977&dopt=GenPept&RID=T250N4CW01N&log$=prottop&blast_rank=27) | catenin, delta 1 isoform 3AC [Homo sapiens] | [17.6](http://blast.ncbi.nlm.nih.gov/Blast.cgi#146231977%23146231977) | 17.6 | 100% | 521 |
| [NP_001078928.1](http://www.ncbi.nlm.nih.gov/entrez/query.fcgi?cmd=Retrieve&db=Protein&list_uids=146231962&dopt=GenPept&RID=T250N4CW01N&log$=prottop&blast_rank=28) | catenin, delta 1 isoform 1AB [Homo sapiens] | [17.6](http://blast.ncbi.nlm.nih.gov/Blast.cgi#146231962%23146231962) | 17.6 | 100% | 521 |
| [NP_001078927.1](http://www.ncbi.nlm.nih.gov/entrez/query.fcgi?cmd=Retrieve&db=Protein&list_uids=146231940&dopt=GenPept&RID=T250N4CW01N&log$=prottop&blast_rank=29) | catenin, delta 1 isoform 1ABC [Homo sapiens] | [17.6](http://blast.ncbi.nlm.nih.gov/Blast.cgi#146231940%23146231940) | 17.6 | 100% | 521 |
| [NP_149044.2](http://www.ncbi.nlm.nih.gov/entrez/query.fcgi?cmd=Retrieve&db=Protein&list_uids=39753965&dopt=GenPept&RID=T250N4CW01N&log$=prottop&blast_rank=30) | hippocampus abundant transcript 1 [Homo sapiens] | [17.6](http://blast.ncbi.nlm.nih.gov/Blast.cgi#39753965%2339753965) | 17.6 | 100% | 521 |
| [NP_065810.2](http://www.ncbi.nlm.nih.gov/entrez/query.fcgi?cmd=Retrieve&db=Protein&list_uids=270265793&dopt=GenPept&RID=T250N4CW01N&log$=prottop&blast_rank=31) | StAR-related lipid transfer (START) domain containing 9 [Homo sapiens] | [17.2](http://blast.ncbi.nlm.nih.gov/Blast.cgi#270265793%23270265793) | 17.2 | 66% | 699 |
| [XP_001717557.2](http://www.ncbi.nlm.nih.gov/entrez/query.fcgi?cmd=Retrieve&db=Protein&list_uids=239756270&dopt=GenPept&RID=T250N4CW01N&log$=prottop&blast_rank=32) | PREDICTED: StAR-related lipid transfer (START) domain containing 9 [Homo sapiens] | [17.2](http://blast.ncbi.nlm.nih.gov/Blast.cgi#239756270%23239756270) | 17.2 | 66% | 699 |
| [XP_001724145.2](http://www.ncbi.nlm.nih.gov/entrez/query.fcgi?cmd=Retrieve&db=Protein&list_uids=239754216&dopt=GenPept&RID=T250N4CW01N&log$=prottop&blast_rank=33) | PREDICTED: hypothetical protein MGC39372 [Homo sapiens] | [17.2](http://blast.ncbi.nlm.nih.gov/Blast.cgi#239754216%23239754216) | 17.2 | 66% | 699 |
| [XP_001129482.3](http://www.ncbi.nlm.nih.gov/entrez/query.fcgi?cmd=Retrieve&db=Protein&list_uids=239745175&dopt=GenPept&RID=T250N4CW01N&log$=prottop&blast_rank=34) | PREDICTED: StAR-related lipid transfer (START) domain containing 9 [Homo sapiens] >ref|XP_001129290.3| PREDICTED: StAR-related lipid transfer (START) domain containing 9 [Homo sapiens] | [17.2](http://blast.ncbi.nlm.nih.gov/Blast.cgi#239745175%23239745175) | 17.2 | 66% | 699 |
| [XP_002344345.1](http://www.ncbi.nlm.nih.gov/entrez/query.fcgi?cmd=Retrieve&db=Protein&list_uids=239508786&dopt=GenPept&RID=T250N4CW01N&log$=prottop&blast_rank=35) | PREDICTED: similar to HSPC047 protein [Homo sapiens] >ref|XP_002342758.1| PREDICTED: hypothetical protein XP_002342758 [Homo sapiens] | [17.2](http://blast.ncbi.nlm.nih.gov/Blast.cgi#239508786%23239508786) | 17.2 | 66% | 699 |
| [NP_001154178.1](http://www.ncbi.nlm.nih.gov/entrez/query.fcgi?cmd=Retrieve&db=Protein&list_uids=238908501&dopt=GenPept&RID=T250N4CW01N&log$=prottop&blast_rank=36) | sciellin isoform 3 [Homo sapiens] | [17.2](http://blast.ncbi.nlm.nih.gov/Blast.cgi#238908501%23238908501) | 17.2 | 66% | 699 |
| [NP_001153836.1](http://www.ncbi.nlm.nih.gov/entrez/query.fcgi?cmd=Retrieve&db=Protein&list_uids=238550100&dopt=GenPept&RID=T250N4CW01N&log$=prottop&blast_rank=37) | transmembrane channel-like 7 isoform b [Homo sapiens] | [17.2](http://blast.ncbi.nlm.nih.gov/Blast.cgi#238550100%23238550100) | 17.2 | 66% | 699 |
| [NP_001138818.1](http://www.ncbi.nlm.nih.gov/entrez/query.fcgi?cmd=Retrieve&db=Protein&list_uids=223890219&dopt=GenPept&RID=T250N4CW01N&log$=prottop&blast_rank=38) | RNA binding motif protein, X-linked-like 3 [Homo sapiens] | [17.2](http://blast.ncbi.nlm.nih.gov/Blast.cgi#223890219%23223890219) | 17.2 | 83% | 699 |
| [NP_001138424.1](http://www.ncbi.nlm.nih.gov/entrez/query.fcgi?cmd=Retrieve&db=Protein&list_uids=222352127&dopt=GenPept&RID=T250N4CW01N&log$=prottop&blast_rank=39) | sidekick 2 [Homo sapiens] | [17.2](http://blast.ncbi.nlm.nih.gov/Blast.cgi#222352127%23222352127) | 17.2 | 66% | 699 |
| [NP_004954.2](http://www.ncbi.nlm.nih.gov/entrez/query.fcgi?cmd=Retrieve&db=Protein&list_uids=222080083&dopt=GenPept&RID=T250N4CW01N&log$=prottop&blast_rank=40) | guanylate cyclase 2C precursor [Homo sapiens] | [17.2](http://blast.ncbi.nlm.nih.gov/Blast.cgi#222080083%23222080083) | 17.2 | 66% | 699 |
| [NP_001128210.1](http://www.ncbi.nlm.nih.gov/entrez/query.fcgi?cmd=Retrieve&db=Protein&list_uids=197927147&dopt=GenPept&RID=T250N4CW01N&log$=prottop&blast_rank=41) | B-cell lymphoma 6 protein isoform 2 [Homo sapiens] | [17.2](http://blast.ncbi.nlm.nih.gov/Blast.cgi#197927147%23197927147) | 17.2 | 66% | 699 |
| [XP_001720583.1](http://www.ncbi.nlm.nih.gov/entrez/query.fcgi?cmd=Retrieve&db=Protein&list_uids=169217528&dopt=GenPept&RID=T250N4CW01N&log$=prottop&blast_rank=42) | PREDICTED: similar to sidekick homolog 2 (chicken) [Homo sapiens] | [17.2](http://blast.ncbi.nlm.nih.gov/Blast.cgi#169217528%23169217528) | 17.2 | 66% | 699 |
| [NP_056025.2](http://www.ncbi.nlm.nih.gov/entrez/query.fcgi?cmd=Retrieve&db=Protein&list_uids=163644316&dopt=GenPept&RID=T250N4CW01N&log$=prottop&blast_rank=43) | hypothetical protein LOC23255 [Homo sapiens] | [17.2](http://blast.ncbi.nlm.nih.gov/Blast.cgi#163644316%23163644316) | 17.2 | 66% | 699 |
| [NP_001128570.1](http://www.ncbi.nlm.nih.gov/entrez/query.fcgi?cmd=Retrieve&db=Protein&list_uids=205360941&dopt=GenPept&RID=T250N4CW01N&log$=prottop&blast_rank=44) | uridine phosphorylase 2 isoform b [Homo sapiens] | [17.2](http://blast.ncbi.nlm.nih.gov/Blast.cgi#205360941%23205360941) | 17.2 | 66% | 699 |
| [NP_001156758.1](http://www.ncbi.nlm.nih.gov/entrez/query.fcgi?cmd=Retrieve&db=Protein&list_uids=253970504&dopt=GenPept&RID=T250N4CW01N&log$=prottop&blast_rank=45) | Ewing sarcoma breakpoint region 1 isoform 4 [Homo sapiens] | [17.2](http://blast.ncbi.nlm.nih.gov/Blast.cgi#253970504%23253970504) | 31.0 | 100% | 699 |
| [NP_997246.2](http://www.ncbi.nlm.nih.gov/entrez/query.fcgi?cmd=Retrieve&db=Protein&list_uids=126362967&dopt=GenPept&RID=T250N4CW01N&log$=prottop&blast_rank=46) | Nck-associated protein 5 isoform 1 [Homo sapiens] | [17.2](http://blast.ncbi.nlm.nih.gov/Blast.cgi#126362967%23126362967) | 29.3 | 100% | 699 |
| [NP_775771.3](http://www.ncbi.nlm.nih.gov/entrez/query.fcgi?cmd=Retrieve&db=Protein&list_uids=116812624&dopt=GenPept&RID=T250N4CW01N&log$=prottop&blast_rank=47) | tau tubulin kinase 2 [Homo sapiens] | [17.2](http://blast.ncbi.nlm.nih.gov/Blast.cgi#116812624%23116812624) | 17.2 | 66% | 699 |
| [NP_060933.3](http://www.ncbi.nlm.nih.gov/entrez/query.fcgi?cmd=Retrieve&db=Protein&list_uids=271397437&dopt=GenPept&RID=T250N4CW01N&log$=prottop&blast_rank=48) | integrin alpha FG-GAP repeat containing 2 [Homo sapiens] | [17.2](http://blast.ncbi.nlm.nih.gov/Blast.cgi#271397437%23271397437) | 17.2 | 66% | 699 |
| [NP_001008844.1](http://www.ncbi.nlm.nih.gov/entrez/query.fcgi?cmd=Retrieve&db=Protein&list_uids=58530842&dopt=GenPept&RID=T250N4CW01N&log$=prottop&blast_rank=49) | desmoplakin isoform II [Homo sapiens] | [17.2](http://blast.ncbi.nlm.nih.gov/Blast.cgi#58530842%2358530842) | 17.2 | 66% | 699 |
| [NP_001138890.1](http://www.ncbi.nlm.nih.gov/entrez/query.fcgi?cmd=Retrieve&db=Protein&list_uids=224028289&dopt=GenPept&RID=T250N4CW01N&log$=prottop&blast_rank=50) | tetratricopeptide repeat domain 28 [Homo sapiens] | [17.2](http://blast.ncbi.nlm.nih.gov/Blast.cgi#224028289%23224028289) | 17.2 | 83% | 699 |
| [NP_003834.3](http://www.ncbi.nlm.nih.gov/entrez/query.fcgi?cmd=Retrieve&db=Protein&list_uids=238908497&dopt=GenPept&RID=T250N4CW01N&log$=prottop&blast_rank=51) | sciellin isoform 2 [Homo sapiens] | [17.2](http://blast.ncbi.nlm.nih.gov/Blast.cgi#238908497%23238908497) | 17.2 | 66% | 699 |
| [NP_001007247.1](http://www.ncbi.nlm.nih.gov/entrez/query.fcgi?cmd=Retrieve&db=Protein&list_uids=55956930&dopt=GenPept&RID=T250N4CW01N&log$=prottop&blast_rank=52) | bromodomain and WD repeat domain containing 1 isoform C [Homo sapiens] | [17.2](http://blast.ncbi.nlm.nih.gov/Blast.cgi#55956930%2355956930) | 17.2 | 83% | 699 |
| [NP_001013675.1](http://www.ncbi.nlm.nih.gov/entrez/query.fcgi?cmd=Retrieve&db=Protein&list_uids=61966761&dopt=GenPept&RID=T250N4CW01N&log$=prottop&blast_rank=53) | leucine rich repeat containing 26 precursor [Homo sapiens] | [17.2](http://blast.ncbi.nlm.nih.gov/Blast.cgi#61966761%2361966761) | 17.2 | 66% | 699 |
| [NP_079123.3](http://www.ncbi.nlm.nih.gov/entrez/query.fcgi?cmd=Retrieve&db=Protein&list_uids=238550098&dopt=GenPept&RID=T250N4CW01N&log$=prottop&blast_rank=54) | transmembrane channel-like 7 isoform a [Homo sapiens] | [17.2](http://blast.ncbi.nlm.nih.gov/Blast.cgi#238550098%23238550098) | 17.2 | 66% | 699 |
| [NP_066267.2](http://www.ncbi.nlm.nih.gov/entrez/query.fcgi?cmd=Retrieve&db=Protein&list_uids=32967601&dopt=GenPept&RID=T250N4CW01N&log$=prottop&blast_rank=55) | ankyrin 3 isoform 1 [Homo sapiens] | [17.2](http://blast.ncbi.nlm.nih.gov/Blast.cgi#32967601%2332967601) | 17.2 | 66% | 699 |
| [NP_001156759.1](http://www.ncbi.nlm.nih.gov/entrez/query.fcgi?cmd=Retrieve&db=Protein&list_uids=253970506&dopt=GenPept&RID=T250N4CW01N&log$=prottop&blast_rank=56) | Ewing sarcoma breakpoint region 1 isoform 5 [Homo sapiens] | [17.2](http://blast.ncbi.nlm.nih.gov/Blast.cgi#253970506%23253970506) | 31.0 | 100% | 699 |
| [NP_002531.3](http://www.ncbi.nlm.nih.gov/entrez/query.fcgi?cmd=Retrieve&db=Protein&list_uids=24430181&dopt=GenPept&RID=T250N4CW01N&log$=prottop&blast_rank=57) | outer dense fiber of sperm tails 2 isoform 1 [Homo sapiens] | [17.2](http://blast.ncbi.nlm.nih.gov/Blast.cgi#24430181%2324430181) | 17.2 | 66% | 699 |
| [NP_061836.2](http://www.ncbi.nlm.nih.gov/entrez/query.fcgi?cmd=Retrieve&db=Protein&list_uids=16445436&dopt=GenPept&RID=T250N4CW01N&log$=prottop&blast_rank=58) | bromodomain and WD repeat domain containing 1 isoform A [Homo sapiens] | [17.2](http://blast.ncbi.nlm.nih.gov/Blast.cgi#16445436%2316445436) | 17.2 | 83% | 699 |
| [NP_387505.1](http://www.ncbi.nlm.nih.gov/entrez/query.fcgi?cmd=Retrieve&db=Protein&list_uids=16445438&dopt=GenPept&RID=T250N4CW01N&log$=prottop&blast_rank=59) | bromodomain and WD repeat domain containing 1 isoform B [Homo sapiens] | [17.2](http://blast.ncbi.nlm.nih.gov/Blast.cgi#16445438%2316445438) | 17.2 | 83% | 699 |
| [NP_004406.2](http://www.ncbi.nlm.nih.gov/entrez/query.fcgi?cmd=Retrieve&db=Protein&list_uids=58530840&dopt=GenPept&RID=T250N4CW01N&log$=prottop&blast_rank=60) | desmoplakin isoform I [Homo sapiens] | [17.2](http://blast.ncbi.nlm.nih.gov/Blast.cgi#58530840%2358530840) | 17.2 | 66% | 699 |
| [NP_659001.2](http://www.ncbi.nlm.nih.gov/entrez/query.fcgi?cmd=Retrieve&db=Protein&list_uids=238908499&dopt=GenPept&RID=T250N4CW01N&log$=prottop&blast_rank=61) | sciellin isoform 1 [Homo sapiens] | [17.2](http://blast.ncbi.nlm.nih.gov/Blast.cgi#238908499%23238908499) | 17.2 | 66% | 699 |
| [NP_053733.2](http://www.ncbi.nlm.nih.gov/entrez/query.fcgi?cmd=Retrieve&db=Protein&list_uids=253970500&dopt=GenPept&RID=T250N4CW01N&log$=prottop&blast_rank=62) | Ewing sarcoma breakpoint region 1 isoform 1 [Homo sapiens] | [17.2](http://blast.ncbi.nlm.nih.gov/Blast.cgi#253970500%23253970500) | 31.0 | 100% | 699 |
| [NP_958434.1](http://www.ncbi.nlm.nih.gov/entrez/query.fcgi?cmd=Retrieve&db=Protein&list_uids=41327730&dopt=GenPept&RID=T250N4CW01N&log$=prottop&blast_rank=63) | calponin 2 isoform b [Homo sapiens] | [17.2](http://blast.ncbi.nlm.nih.gov/Blast.cgi#41327730%2341327730) | 17.2 | 66% | 699 |
| [NP_055869.1](http://www.ncbi.nlm.nih.gov/entrez/query.fcgi?cmd=Retrieve&db=Protein&list_uids=55749644&dopt=GenPept&RID=T250N4CW01N&log$=prottop&blast_rank=64) | UHRF1 binding protein 1-like isoform a [Homo sapiens] | [17.2](http://blast.ncbi.nlm.nih.gov/Blast.cgi#55749644%2355749644) | 17.2 | 66% | 699 |
| [NP_789786.2](http://www.ncbi.nlm.nih.gov/entrez/query.fcgi?cmd=Retrieve&db=Protein&list_uids=88014609&dopt=GenPept&RID=T250N4CW01N&log$=prottop&blast_rank=65) | coiled-coil domain containing 125 [Homo sapiens] | [17.2](http://blast.ncbi.nlm.nih.gov/Blast.cgi#88014609%2388014609) | 17.2 | 66% | 699 |
| [NP_115548.1](http://www.ncbi.nlm.nih.gov/entrez/query.fcgi?cmd=Retrieve&db=Protein&list_uids=79750944&dopt=GenPept&RID=T250N4CW01N&log$=prottop&blast_rank=66) | ubiquitin specific peptidase 42 [Homo sapiens] | [17.2](http://blast.ncbi.nlm.nih.gov/Blast.cgi#79750944%2379750944) | 17.2 | 66% | 699 |
| [NP_006222.2](http://www.ncbi.nlm.nih.gov/entrez/query.fcgi?cmd=Retrieve&db=Protein&list_uids=62198237&dopt=GenPept&RID=T250N4CW01N&log$=prottop&blast_rank=67) | DNA-directed DNA polymerase epsilon [Homo sapiens] | [17.2](http://blast.ncbi.nlm.nih.gov/Blast.cgi#62198237%2362198237) | 17.2 | 66% | 699 |
| [NP_954672.1](http://www.ncbi.nlm.nih.gov/entrez/query.fcgi?cmd=Retrieve&db=Protein&list_uids=40549422&dopt=GenPept&RID=T250N4CW01N&log$=prottop&blast_rank=68) | Theg homolog isoform 2 [Homo sapiens] | [17.2](http://blast.ncbi.nlm.nih.gov/Blast.cgi#40549422%2340549422) | 17.2 | 66% | 699 |
| [NP_631874.1](http://www.ncbi.nlm.nih.gov/entrez/query.fcgi?cmd=Retrieve&db=Protein&list_uids=21217561&dopt=GenPept&RID=T250N4CW01N&log$=prottop&blast_rank=69) | Shaw-related voltage-gated potassium channel protein 2 isoform KV3.2a [Homo sapiens] | [17.2](http://blast.ncbi.nlm.nih.gov/Blast.cgi#21217561%2321217561) | 17.2 | 83% | 699 |
| [NP_631875.1](http://www.ncbi.nlm.nih.gov/entrez/query.fcgi?cmd=Retrieve&db=Protein&list_uids=21217563&dopt=GenPept&RID=T250N4CW01N&log$=prottop&blast_rank=70) | Shaw-related voltage-gated potassium channel protein 2 isoform KV3.2b [Homo sapiens] | [17.2](http://blast.ncbi.nlm.nih.gov/Blast.cgi#21217563%2321217563) | 17.2 | 83% | 699 |
| [NP_057416.1](http://www.ncbi.nlm.nih.gov/entrez/query.fcgi?cmd=Retrieve&db=Protein&list_uids=7706511&dopt=GenPept&RID=T250N4CW01N&log$=prottop&blast_rank=71) | selenoprotein X, 1 [Homo sapiens] | [17.2](http://blast.ncbi.nlm.nih.gov/Blast.cgi#7706511%237706511) | 17.2 | 83% | 699 |
| [NP_061027.2](http://www.ncbi.nlm.nih.gov/entrez/query.fcgi?cmd=Retrieve&db=Protein&list_uids=93102379&dopt=GenPept&RID=T250N4CW01N&log$=prottop&blast_rank=72) | low density lipoprotein-related protein 1B precursor [Homo sapiens] | [17.2](http://blast.ncbi.nlm.nih.gov/Blast.cgi#93102379%2393102379) | 17.2 | 83% | 699 |
| [NP_060310.1](http://www.ncbi.nlm.nih.gov/entrez/query.fcgi?cmd=Retrieve&db=Protein&list_uids=8923448&dopt=GenPept&RID=T250N4CW01N&log$=prottop&blast_rank=73) | mitochondrial ribosomal protein L16 precursor [Homo sapiens] | [17.2](http://blast.ncbi.nlm.nih.gov/Blast.cgi#8923448%238923448) | 17.2 | 66% | 699 |
| [NP_715624.1](http://www.ncbi.nlm.nih.gov/entrez/query.fcgi?cmd=Retrieve&db=Protein&list_uids=24497458&dopt=GenPept&RID=T250N4CW01N&log$=prottop&blast_rank=74) | Shaw-related voltage-gated potassium channel protein 2 isoform KV3.2c [Homo sapiens] | [17.2](http://blast.ncbi.nlm.nih.gov/Blast.cgi#24497458%2324497458) | 17.2 | 83% | 699 |
| [NP_005908.1](http://www.ncbi.nlm.nih.gov/entrez/query.fcgi?cmd=Retrieve&db=Protein&list_uids=5174539&dopt=GenPept&RID=T250N4CW01N&log$=prottop&blast_rank=75) | cytosolic malate dehydrogenase [Homo sapiens] | [17.2](http://blast.ncbi.nlm.nih.gov/Blast.cgi#5174539%235174539) | 17.2 | 66% | 699 |
| [NP_055186.2](http://www.ncbi.nlm.nih.gov/entrez/query.fcgi?cmd=Retrieve&db=Protein&list_uids=49472841&dopt=GenPept&RID=T250N4CW01N&log$=prottop&blast_rank=76) | A kinase (PRKA) anchor protein 8-like [Homo sapiens] | [17.2](http://blast.ncbi.nlm.nih.gov/Blast.cgi#49472841%2349472841) | 17.2 | 66% | 699 |
| [NP_002421.3](http://www.ncbi.nlm.nih.gov/entrez/query.fcgi?cmd=Retrieve&db=Protein&list_uids=55956910&dopt=GenPept&RID=T250N4CW01N&log$=prottop&blast_rank=77) | meningioma 1 [Homo sapiens] | [17.2](http://blast.ncbi.nlm.nih.gov/Blast.cgi#55956910%2355956910) | 17.2 | 66% | 699 |
| [NP_001736.1](http://www.ncbi.nlm.nih.gov/entrez/query.fcgi?cmd=Retrieve&db=Protein&list_uids=4502559&dopt=GenPept&RID=T250N4CW01N&log$=prottop&blast_rank=78) | calcium modulating ligand [Homo sapiens] | [17.2](http://blast.ncbi.nlm.nih.gov/Blast.cgi#4502559%234502559) | 17.2 | 66% | 699 |
| [NP_775491.1](http://www.ncbi.nlm.nih.gov/entrez/query.fcgi?cmd=Retrieve&db=Protein&list_uids=27597096&dopt=GenPept&RID=T250N4CW01N&log$=prottop&blast_rank=79) | uridine phosphorylase 2 isoform a [Homo sapiens] | [17.2](http://blast.ncbi.nlm.nih.gov/Blast.cgi#27597096%2327597096) | 17.2 | 66% | 699 |
| [NP_057669.1](http://www.ncbi.nlm.nih.gov/entrez/query.fcgi?cmd=Retrieve&db=Protein&list_uids=7706121&dopt=GenPept&RID=T250N4CW01N&log$=prottop&blast_rank=80) | Theg homolog isoform 1 [Homo sapiens] | [17.2](http://blast.ncbi.nlm.nih.gov/Blast.cgi#7706121%237706121) | 17.2 | 66% | 699 |
| [NP_005234.1](http://www.ncbi.nlm.nih.gov/entrez/query.fcgi?cmd=Retrieve&db=Protein&list_uids=4885225&dopt=GenPept&RID=T250N4CW01N&log$=prottop&blast_rank=81) | Ewing sarcoma breakpoint region 1 isoform 2 [Homo sapiens] | [17.2](http://blast.ncbi.nlm.nih.gov/Blast.cgi#4885225%234885225) | 31.0 | 100% | 699 |
| [NP_113671.3](http://www.ncbi.nlm.nih.gov/entrez/query.fcgi?cmd=Retrieve&db=Protein&list_uids=27477109&dopt=GenPept&RID=T250N4CW01N&log$=prottop&blast_rank=82) | itchy homolog E3 ubiquitin protein ligase [Homo sapiens] | [17.2](http://blast.ncbi.nlm.nih.gov/Blast.cgi#27477109%2327477109) | 17.2 | 66% | 699 |
| [NP_001697.2](http://www.ncbi.nlm.nih.gov/entrez/query.fcgi?cmd=Retrieve&db=Protein&list_uids=21040324&dopt=GenPept&RID=T250N4CW01N&log$=prottop&blast_rank=83) | B-cell lymphoma 6 protein isoform 1 [Homo sapiens] >ref|NP_001124317.1| B-cell lymphoma 6 protein isoform 1 [Homo sapiens] | [17.2](http://blast.ncbi.nlm.nih.gov/Blast.cgi#21040324%2321040324) | 17.2 | 66% | 699 |
| [NP_777576.1](http://www.ncbi.nlm.nih.gov/entrez/query.fcgi?cmd=Retrieve&db=Protein&list_uids=28372497&dopt=GenPept&RID=T250N4CW01N&log$=prottop&blast_rank=84) | ubiquitin protein ligase E3 component n-recognin 1 [Homo sapiens] | [17.2](http://blast.ncbi.nlm.nih.gov/Blast.cgi#28372497%2328372497) | 17.2 | 83% | 699 |
| [NP_055400.1](http://www.ncbi.nlm.nih.gov/entrez/query.fcgi?cmd=Retrieve&db=Protein&list_uids=7657100&dopt=GenPept&RID=T250N4CW01N&log$=prottop&blast_rank=85) | solute carrier family 40 (iron-regulated transporter), member 1 [Homo sapiens] | [17.2](http://blast.ncbi.nlm.nih.gov/Blast.cgi#7657100%237657100) | 17.2 | 66% | 699 |
| [NP_001002800.1](http://www.ncbi.nlm.nih.gov/entrez/query.fcgi?cmd=Retrieve&db=Protein&list_uids=50658063&dopt=GenPept&RID=T250N4CW01N&log$=prottop&blast_rank=86) | SMC4 structural maintenance of chromosomes 4-like 1 [Homo sapiens] >ref|NP_005487.3| SMC4 structural maintenance of chromosomes 4-like 1 [Homo sapiens] | [17.2](http://blast.ncbi.nlm.nih.gov/Blast.cgi#50658063%2350658063) | 17.2 | 66% | 699 |
| [NP_037406.1](http://www.ncbi.nlm.nih.gov/entrez/query.fcgi?cmd=Retrieve&db=Protein&list_uids=7019491&dopt=GenPept&RID=T250N4CW01N&log$=prottop&blast_rank=87) | DNA-directed DNA polymerase lambda [Homo sapiens] | [17.2](http://blast.ncbi.nlm.nih.gov/Blast.cgi#7019491%237019491) | 17.2 | 66% | 699 |
| [NP_001156757.1](http://www.ncbi.nlm.nih.gov/entrez/query.fcgi?cmd=Retrieve&db=Protein&list_uids=253970502&dopt=GenPept&RID=T250N4CW01N&log$=prottop&blast_rank=88) | Ewing sarcoma breakpoint region 1 isoform 3 [Homo sapiens] | [17.2](http://blast.ncbi.nlm.nih.gov/Blast.cgi#253970502%23253970502) | 31.0 | 100% | 699 |
| [NP_004359.1](http://www.ncbi.nlm.nih.gov/entrez/query.fcgi?cmd=Retrieve&db=Protein&list_uids=4758018&dopt=GenPept&RID=T250N4CW01N&log$=prottop&blast_rank=89) | calponin 2 isoform a [Homo sapiens] | [17.2](http://blast.ncbi.nlm.nih.gov/Blast.cgi#4758018%234758018) | 17.2 | 66% | 699 |
| [NP_073737.1](http://www.ncbi.nlm.nih.gov/entrez/query.fcgi?cmd=Retrieve&db=Protein&list_uids=12383066&dopt=GenPept&RID=T250N4CW01N&log$=prottop&blast_rank=90) | axotrophin [Homo sapiens] | [17.2](http://blast.ncbi.nlm.nih.gov/Blast.cgi#12383066%2312383066) | 17.2 | 66% | 699 |
| [NP_005626.1](http://www.ncbi.nlm.nih.gov/entrez/query.fcgi?cmd=Retrieve&db=Protein&list_uids=5032121&dopt=GenPept&RID=T250N4CW01N&log$=prottop&blast_rank=91) | synovial sarcoma, X breakpoint 1 [Homo sapiens] | [17.2](http://blast.ncbi.nlm.nih.gov/Blast.cgi#5032121%235032121) | 17.2 | 66% | 699 |
| [NP_001166251.1](http://www.ncbi.nlm.nih.gov/entrez/query.fcgi?cmd=Retrieve&db=Protein&list_uids=289666782&dopt=GenPept&RID=T250N4CW01N&log$=prottop&blast_rank=92) | leucine rich repeat containing 34 isoform 1 [Homo sapiens] | [16.8](http://blast.ncbi.nlm.nih.gov/Blast.cgi#289666782%23289666782) | 16.8 | 83% | 938 |
| [NP_699184.2](http://www.ncbi.nlm.nih.gov/entrez/query.fcgi?cmd=Retrieve&db=Protein&list_uids=289666778&dopt=GenPept&RID=T250N4CW01N&log$=prottop&blast_rank=93) | leucine rich repeat containing 34 isoform 3 [Homo sapiens] | [16.8](http://blast.ncbi.nlm.nih.gov/Blast.cgi#289666778%23289666778) | 16.8 | 83% | 938 |
| [XP_002347089.1](http://www.ncbi.nlm.nih.gov/entrez/query.fcgi?cmd=Retrieve&db=Protein&list_uids=239749576&dopt=GenPept&RID=T250N4CW01N&log$=prottop&blast_rank=94) | PREDICTED: hypothetical protein XP_002347089 [Homo sapiens] | [16.8](http://blast.ncbi.nlm.nih.gov/Blast.cgi#239749576%23239749576) | 16.8 | 83% | 938 |
| [NP_001166250.1](http://www.ncbi.nlm.nih.gov/entrez/query.fcgi?cmd=Retrieve&db=Protein&list_uids=289666780&dopt=GenPept&RID=T250N4CW01N&log$=prottop&blast_rank=95) | leucine rich repeat containing 34 isoform 2 [Homo sapiens] | [16.8](http://blast.ncbi.nlm.nih.gov/Blast.cgi#289666780%23289666780) | 16.8 | 83% | 938 |
| [NP_066554.2](http://www.ncbi.nlm.nih.gov/entrez/query.fcgi?cmd=Retrieve&db=Protein&list_uids=32483416&dopt=GenPept&RID=T250N4CW01N&log$=prottop&blast_rank=96) | neurofilament, heavy polypeptide 200kDa [Homo sapiens] | [16.8](http://blast.ncbi.nlm.nih.gov/Blast.cgi#32483416%2332483416) | 16.8 | 83% | 938 |
| [NP_787048.1](http://www.ncbi.nlm.nih.gov/entrez/query.fcgi?cmd=Retrieve&db=Protein&list_uids=28460688&dopt=GenPept&RID=T250N4CW01N&log$=prottop&blast_rank=97) | taxilin [Homo sapiens] | [16.8](http://blast.ncbi.nlm.nih.gov/Blast.cgi#28460688%2328460688) | 16.8 | 83% | 938 |
| [NP_066272.1](http://www.ncbi.nlm.nih.gov/entrez/query.fcgi?cmd=Retrieve&db=Protein&list_uids=13994151&dopt=GenPept&RID=T250N4CW01N&log$=prottop&blast_rank=98) | PDZ and LIM domain 1 [Homo sapiens] | [16.8](http://blast.ncbi.nlm.nih.gov/Blast.cgi#13994151%2313994151) | 16.8 | 83% | 938 |
| [NP_877423.1](http://www.ncbi.nlm.nih.gov/entrez/query.fcgi?cmd=Retrieve&db=Protein&list_uids=33469917&dopt=GenPept&RID=T250N4CW01N&log$=prottop&blast_rank=99) | minichromosome maintenance complex component 4 [Homo sapiens] >ref|NP_005905.2| minichromosome maintenance complex component 4 [Homo sapiens] | [16.8](http://blast.ncbi.nlm.nih.gov/Blast.cgi#33469917%2333469917) | 16.8 | 83% | 938 |
| [NP_001165124.1](http://www.ncbi.nlm.nih.gov/entrez/query.fcgi?cmd=Retrieve&db=Protein&list_uids=284413746&dopt=GenPept&RID=T250N4CW01N&log$=prottop&blast_rank=100) | zinc finger homeobox 1b isoform 2 [Homo sapiens] | [16.3](http://blast.ncbi.nlm.nih.gov/Blast.cgi#284413746%23284413746) | 16.3 | 83% | 1258 |

| **Accession** | **Proteins with a match to SHNRVSN peptide** | **[Max score](http://blast.ncbi.nlm.nih.gov/Blast.cgi?CMD=Get&ALIGNMENTS=100&ALIGNMENT_VIEW=Pairwise&CDD_SEARCH_STATE=1&DATABASE_SORT=0&DESCRIPTIONS=100&ENTREZ_QUERY=txid9606 %5BORGN%5D&FIRST_QUERY_NUM=0&FORMAT_OBJECT=Alignment&FORMAT_PAGE_TARGET=&FORMAT_TYPE=HTML&GET_SEQUENCE=yes&I_THRESH=&MASK_CHAR=2&MASK_COLOR=1&NEW_DESIGN=on&NEW_VIEW=yes&NUM_OVERVIEW=100&OLD_BLAST=false&PAGE=Proteins&QUERY_INDEX=0&QUERY_NUMBER=0&RESULTS_PAGE_TARGET=&RID=T25AT6S2014&SHOW_LINKOUT=yes&SHOW_OVERVIEW=yes&STEP_NUMBER=&WORD_SIZE=2&DISPLAY_SORT=1&HSP_SORT=1" \l "sort_mark)** | **[Total score](http://blast.ncbi.nlm.nih.gov/Blast.cgi?CMD=Get&ALIGNMENTS=100&ALIGNMENT_VIEW=Pairwise&CDD_SEARCH_STATE=1&DATABASE_SORT=0&DESCRIPTIONS=100&ENTREZ_QUERY=txid9606 %5BORGN%5D&FIRST_QUERY_NUM=0&FORMAT_OBJECT=Alignment&FORMAT_PAGE_TARGET=&FORMAT_TYPE=HTML&GET_SEQUENCE=yes&I_THRESH=&MASK_CHAR=2&MASK_COLOR=1&NEW_DESIGN=on&NEW_VIEW=yes&NUM_OVERVIEW=100&OLD_BLAST=false&PAGE=Proteins&QUERY_INDEX=0&QUERY_NUMBER=0&RESULTS_PAGE_TARGET=&RID=T25AT6S2014&SHOW_LINKOUT=yes&SHOW_OVERVIEW=yes&STEP_NUMBER=&WORD_SIZE=2&DISPLAY_SORT=2&HSP_SORT=1" \l "sort_mark)** | **[Query coverage](http://blast.ncbi.nlm.nih.gov/Blast.cgi?CMD=Get&ALIGNMENTS=100&ALIGNMENT_VIEW=Pairwise&CDD_SEARCH_STATE=1&DATABASE_SORT=0&DESCRIPTIONS=100&ENTREZ_QUERY=txid9606 %5BORGN%5D&FIRST_QUERY_NUM=0&FORMAT_OBJECT=Alignment&FORMAT_PAGE_TARGET=&FORMAT_TYPE=HTML&GET_SEQUENCE=yes&I_THRESH=&MASK_CHAR=2&MASK_COLOR=1&NEW_DESIGN=on&NEW_VIEW=yes&NUM_OVERVIEW=100&OLD_BLAST=false&PAGE=Proteins&QUERY_INDEX=0&QUERY_NUMBER=0&RESULTS_PAGE_TARGET=&RID=T25AT6S2014&SHOW_LINKOUT=yes&SHOW_OVERVIEW=yes&STEP_NUMBER=&WORD_SIZE=2&DISPLAY_SORT=4&HSP_SORT=0" \l "sort_mark)** | **[E value](http://blast.ncbi.nlm.nih.gov/Blast.cgi?CMD=Get&ALIGNMENTS=100&ALIGNMENT_VIEW=Pairwise&CDD_SEARCH_STATE=1&DATABASE_SORT=0&DESCRIPTIONS=100&ENTREZ_QUERY=txid9606 %5BORGN%5D&FIRST_QUERY_NUM=0&FORMAT_OBJECT=Alignment&FORMAT_PAGE_TARGET=&FORMAT_TYPE=HTML&GET_SEQUENCE=yes&I_THRESH=&MASK_CHAR=2&MASK_COLOR=1&NEW_DESIGN=on&NEW_VIEW=yes&NUM_OVERVIEW=100&OLD_BLAST=false&PAGE=Proteins&QUERY_INDEX=0&QUERY_NUMBER=0&RESULTS_PAGE_TARGET=&RID=T25AT6S2014&SHOW_LINKOUT=yes&SHOW_OVERVIEW=yes&STEP_NUMBER=&WORD_SIZE=2&DISPLAY_SORT=0&HSP_SORT=0" \l "sort_mark)** |
| --- | --- | --- | --- | --- | --- |
| [NP_055628.1](http://www.ncbi.nlm.nih.gov/entrez/query.fcgi?cmd=Retrieve&db=Protein&list_uids=7662320&dopt=GenPept&RID=T25AT6S2014&log$=prottop&blast_rank=1) | leucine-rich repeats and immunoglobulin-like domains 2 precursor [Homo sapiens] | [21.4](http://blast.ncbi.nlm.nih.gov/Blast.cgi#7662320%237662320) | 21.4 | 100% | 43 |
| [NP_060606.3](http://www.ncbi.nlm.nih.gov/entrez/query.fcgi?cmd=Retrieve&db=Protein&list_uids=126116596&dopt=GenPept&RID=T25AT6S2014&log$=prottop&blast_rank=2) | asp (abnormal spindle)-like, microcephaly associated [Homo sapiens] | [21.0](http://blast.ncbi.nlm.nih.gov/Blast.cgi#126116596%23126116596) | 21.0 | 100% | 58 |
| [NP_002716.1](http://www.ncbi.nlm.nih.gov/entrez/query.fcgi?cmd=Retrieve&db=Protein&list_uids=4506041&dopt=GenPept&RID=T25AT6S2014&log$=prottop&blast_rank=3) | proline arginine-rich end leucine-rich repeat protein precursor [Homo sapiens] >ref|NP_958505.1| proline arginine-rich end leucine-rich repeat protein precursor [Homo sapiens] | [19.7](http://blast.ncbi.nlm.nih.gov/Blast.cgi#4506041%234506041) | 19.7 | 85% | 140 |
| [NP_619542.1](http://www.ncbi.nlm.nih.gov/entrez/query.fcgi?cmd=Retrieve&db=Protein&list_uids=20302168&dopt=GenPept&RID=T25AT6S2014&log$=prottop&blast_rank=4) | toll-like receptor 8 precursor [Homo sapiens] | [19.7](http://blast.ncbi.nlm.nih.gov/Blast.cgi#20302168%2320302168) | 19.7 | 85% | 140 |
| [NP_653309.3](http://www.ncbi.nlm.nih.gov/entrez/query.fcgi?cmd=Retrieve&db=Protein&list_uids=284413734&dopt=GenPept&RID=T25AT6S2014&log$=prottop&blast_rank=5) | ankyrin and armadillo repeat containing [Homo sapiens] | [19.3](http://blast.ncbi.nlm.nih.gov/Blast.cgi#284413734%23284413734) | 19.3 | 71% | 188 |
| [XP_002344774.1](http://www.ncbi.nlm.nih.gov/entrez/query.fcgi?cmd=Retrieve&db=Protein&list_uids=239756009&dopt=GenPept&RID=T25AT6S2014&log$=prottop&blast_rank=6) | PREDICTED: similar to transmembrane phosphoinositide 3-phosphatase and tensin homolog 2, partial [Homo sapiens] | [19.3](http://blast.ncbi.nlm.nih.gov/Blast.cgi#239756009%23239756009) | 19.3 | 71% | 188 |
| [XP_001726752.2](http://www.ncbi.nlm.nih.gov/entrez/query.fcgi?cmd=Retrieve&db=Protein&list_uids=239753208&dopt=GenPept&RID=T25AT6S2014&log$=prottop&blast_rank=7) | PREDICTED: similar to anaphase promoting complex subunit 1 [Homo sapiens] | [19.3](http://blast.ncbi.nlm.nih.gov/Blast.cgi#239753208%23239753208) | 19.3 | 71% | 188 |
| [NP_001139478.1](http://www.ncbi.nlm.nih.gov/entrez/query.fcgi?cmd=Retrieve&db=Protein&list_uids=225579152&dopt=GenPept&RID=T25AT6S2014&log$=prottop&blast_rank=8) | insulin-like growth factor binding protein, acid labile subunit isoform 1 precursor [Homo sapiens] | [19.3](http://blast.ncbi.nlm.nih.gov/Blast.cgi#225579152%23225579152) | 35.6 | 71% | 188 |
| [NP_996816.2](http://www.ncbi.nlm.nih.gov/entrez/query.fcgi?cmd=Retrieve&db=Protein&list_uids=219842266&dopt=GenPept&RID=T25AT6S2014&log$=prottop&blast_rank=9) | usherin isoform B [Homo sapiens] | [19.3](http://blast.ncbi.nlm.nih.gov/Blast.cgi#219842266%23219842266) | 62.4 | 85% | 188 |
| [NP_001135440.1](http://www.ncbi.nlm.nih.gov/entrez/query.fcgi?cmd=Retrieve&db=Protein&list_uids=213972593&dopt=GenPept&RID=T25AT6S2014&log$=prottop&blast_rank=10) | TPTE and PTEN homologous inositol lipid phosphatase isoform delta [Homo sapiens] | [19.3](http://blast.ncbi.nlm.nih.gov/Blast.cgi#213972593%23213972593) | 19.3 | 71% | 188 |
| [NP_954863.2](http://www.ncbi.nlm.nih.gov/entrez/query.fcgi?cmd=Retrieve&db=Protein&list_uids=213972591&dopt=GenPept&RID=T25AT6S2014&log$=prottop&blast_rank=11) | TPTE and PTEN homologous inositol lipid phosphatase isoform gamma [Homo sapiens] | [19.3](http://blast.ncbi.nlm.nih.gov/Blast.cgi#213972591%23213972591) | 19.3 | 71% | 188 |
| [NP_570141.3](http://www.ncbi.nlm.nih.gov/entrez/query.fcgi?cmd=Retrieve&db=Protein&list_uids=213972589&dopt=GenPept&RID=T25AT6S2014&log$=prottop&blast_rank=12) | TPTE and PTEN homologous inositol lipid phosphatase isoform alpha [Homo sapiens] | [19.3](http://blast.ncbi.nlm.nih.gov/Blast.cgi#213972589%23213972589) | 19.3 | 71% | 188 |
| [NP_001003676.1](http://www.ncbi.nlm.nih.gov/entrez/query.fcgi?cmd=Retrieve&db=Protein&list_uids=51558745&dopt=GenPept&RID=T25AT6S2014&log$=prottop&blast_rank=13) | hypothetical protein LOC79096 isoform 1 [Homo sapiens] | [19.3](http://blast.ncbi.nlm.nih.gov/Blast.cgi#51558745%2351558745) | 19.3 | 71% | 188 |
| [NP_001003678.1](http://www.ncbi.nlm.nih.gov/entrez/query.fcgi?cmd=Retrieve&db=Protein&list_uids=51558751&dopt=GenPept&RID=T25AT6S2014&log$=prottop&blast_rank=14) | hypothetical protein LOC79096 isoform 4 [Homo sapiens] | [19.3](http://blast.ncbi.nlm.nih.gov/Blast.cgi#51558751%2351558751) | 19.3 | 71% | 188 |
| [NP_006613.2](http://www.ncbi.nlm.nih.gov/entrez/query.fcgi?cmd=Retrieve&db=Protein&list_uids=93004081&dopt=GenPept&RID=T25AT6S2014&log$=prottop&blast_rank=15) | polo-like kinase 2 [Homo sapiens] | [19.3](http://blast.ncbi.nlm.nih.gov/Blast.cgi#93004081%2393004081) | 19.3 | 71% | 188 |
| [NP_077018.1](http://www.ncbi.nlm.nih.gov/entrez/query.fcgi?cmd=Retrieve&db=Protein&list_uids=13129130&dopt=GenPept&RID=T25AT6S2014&log$=prottop&blast_rank=16) | hypothetical protein LOC79096 isoform 3 [Homo sapiens] | [19.3](http://blast.ncbi.nlm.nih.gov/Blast.cgi#13129130%2313129130) | 19.3 | 71% | 188 |
| [NP_002817.2](http://www.ncbi.nlm.nih.gov/entrez/query.fcgi?cmd=Retrieve&db=Protein&list_uids=13325075&dopt=GenPept&RID=T25AT6S2014&log$=prottop&blast_rank=17) | quiescin Q6 sulfhydryl oxidase 1 isoform a [Homo sapiens] | [19.3](http://blast.ncbi.nlm.nih.gov/Blast.cgi#13325075%2313325075) | 19.3 | 71% | 188 |
| [NP_004961.1](http://www.ncbi.nlm.nih.gov/entrez/query.fcgi?cmd=Retrieve&db=Protein&list_uids=4826772&dopt=GenPept&RID=T25AT6S2014&log$=prottop&blast_rank=18) | insulin-like growth factor binding protein, acid labile subunit isoform 2 precursor [Homo sapiens] | [19.3](http://blast.ncbi.nlm.nih.gov/Blast.cgi#4826772%234826772) | 35.6 | 71% | 188 |
| [NP_001003677.1](http://www.ncbi.nlm.nih.gov/entrez/query.fcgi?cmd=Retrieve&db=Protein&list_uids=51558748&dopt=GenPept&RID=T25AT6S2014&log$=prottop&blast_rank=19) | hypothetical protein LOC79096 isoform 2 [Homo sapiens] | [19.3](http://blast.ncbi.nlm.nih.gov/Blast.cgi#51558748%2351558748) | 19.3 | 71% | 188 |
| [NP_005246.2](http://www.ncbi.nlm.nih.gov/entrez/query.fcgi?cmd=Retrieve&db=Protein&list_uids=157384971&dopt=GenPept&RID=T25AT6S2014&log$=prottop&blast_rank=20) | cyclin G associated kinase [Homo sapiens] | [19.3](http://blast.ncbi.nlm.nih.gov/Blast.cgi#157384971%23157384971) | 19.3 | 71% | 188 |
| [NP_055702.1](http://www.ncbi.nlm.nih.gov/entrez/query.fcgi?cmd=Retrieve&db=Protein&list_uids=7656971&dopt=GenPept&RID=T25AT6S2014&log$=prottop&blast_rank=21) | phosphonoformate immuno-associated protein 5 isoform 2 [Homo sapiens] | [19.3](http://blast.ncbi.nlm.nih.gov/Blast.cgi#7656971%237656971) | 19.3 | 71% | 188 |
| [NP_001004128.1](http://www.ncbi.nlm.nih.gov/entrez/query.fcgi?cmd=Retrieve&db=Protein&list_uids=51873067&dopt=GenPept&RID=T25AT6S2014&log$=prottop&blast_rank=22) | quiescin Q6 sulfhydryl oxidase 1 isoform b [Homo sapiens] | [19.3](http://blast.ncbi.nlm.nih.gov/Blast.cgi#51873067%2351873067) | 19.3 | 71% | 188 |
| [NP_001005851.1](http://www.ncbi.nlm.nih.gov/entrez/query.fcgi?cmd=Retrieve&db=Protein&list_uids=54291708&dopt=GenPept&RID=T25AT6S2014&log$=prottop&blast_rank=23) | zinc finger protein 780B [Homo sapiens] | [18.9](http://blast.ncbi.nlm.nih.gov/Blast.cgi#54291708%2354291708) | 102 | 71% | 252 |
| [NP_060493.3](http://www.ncbi.nlm.nih.gov/entrez/query.fcgi?cmd=Retrieve&db=Protein&list_uids=33620755&dopt=GenPept&RID=T25AT6S2014&log$=prottop&blast_rank=24) | YEATS domain containing 2 [Homo sapiens] | [18.9](http://blast.ncbi.nlm.nih.gov/Blast.cgi#33620755%2333620755) | 18.9 | 100% | 252 |
| [NP_699184.2](http://www.ncbi.nlm.nih.gov/entrez/query.fcgi?cmd=Retrieve&db=Protein&list_uids=289666778&dopt=GenPept&RID=T25AT6S2014&log$=prottop&blast_rank=25) | leucine rich repeat containing 34 isoform 3 [Homo sapiens] | [18.5](http://blast.ncbi.nlm.nih.gov/Blast.cgi#289666778%23289666778) | 18.5 | 100% | 338 |
| [XP_002342600.1](http://www.ncbi.nlm.nih.gov/entrez/query.fcgi?cmd=Retrieve&db=Protein&list_uids=239742631&dopt=GenPept&RID=T25AT6S2014&log$=prottop&blast_rank=26) | PREDICTED: hypothetical protein XP_002342600 [Homo sapiens] >ref|XP_002345889.1| PREDICTED: hypothetical protein [Homo sapiens] | [18.5](http://blast.ncbi.nlm.nih.gov/Blast.cgi#239742631%23239742631) | 18.5 | 85% | 338 |
| [NP_001166250.1](http://www.ncbi.nlm.nih.gov/entrez/query.fcgi?cmd=Retrieve&db=Protein&list_uids=289666780&dopt=GenPept&RID=T25AT6S2014&log$=prottop&blast_rank=27) | leucine rich repeat containing 34 isoform 2 [Homo sapiens] | [18.5](http://blast.ncbi.nlm.nih.gov/Blast.cgi#289666780%23289666780) | 18.5 | 100% | 338 |
| [NP_064713.3](http://www.ncbi.nlm.nih.gov/entrez/query.fcgi?cmd=Retrieve&db=Protein&list_uids=46309849&dopt=GenPept&RID=T25AT6S2014&log$=prottop&blast_rank=28) | hypothetical protein LOC57035 [Homo sapiens] | [18.5](http://blast.ncbi.nlm.nih.gov/Blast.cgi#46309849%2346309849) | 18.5 | 85% | 338 |
| [NP_001129523.1](http://www.ncbi.nlm.nih.gov/entrez/query.fcgi?cmd=Retrieve&db=Protein&list_uids=209862903&dopt=GenPept&RID=T25AT6S2014&log$=prottop&blast_rank=29) | leucine-rich repeats and immunoglobulin-like domains 3 isoform 1 [Homo sapiens] | [18.0](http://blast.ncbi.nlm.nih.gov/Blast.cgi#209862903%23209862903) | 33.5 | 85% | 453 |
| [NP_055165.2](http://www.ncbi.nlm.nih.gov/entrez/query.fcgi?cmd=Retrieve&db=Protein&list_uids=117676365&dopt=GenPept&RID=T25AT6S2014&log$=prottop&blast_rank=30) | tumor necrosis factor, alpha-induced protein 8 isoform a [Homo sapiens] | [18.0](http://blast.ncbi.nlm.nih.gov/Blast.cgi#117676365%23117676365) | 18.0 | 100% | 453 |
| [NP_001071122.1](http://www.ncbi.nlm.nih.gov/entrez/query.fcgi?cmd=Retrieve&db=Protein&list_uids=117676370&dopt=GenPept&RID=T25AT6S2014&log$=prottop&blast_rank=31) | tumor necrosis factor, alpha-induced protein 8 isoform b [Homo sapiens] | [18.0](http://blast.ncbi.nlm.nih.gov/Blast.cgi#117676370%23117676370) | 18.0 | 100% | 453 |
| [NP_065706.2](http://www.ncbi.nlm.nih.gov/entrez/query.fcgi?cmd=Retrieve&db=Protein&list_uids=21704283&dopt=GenPept&RID=T25AT6S2014&log$=prottop&blast_rank=32) | junctophilin 3 [Homo sapiens] | [18.0](http://blast.ncbi.nlm.nih.gov/Blast.cgi#21704283%2321704283) | 18.0 | 100% | 453 |
| [NP_722582.2](http://www.ncbi.nlm.nih.gov/entrez/query.fcgi?cmd=Retrieve&db=Protein&list_uids=61743940&dopt=GenPept&RID=T25AT6S2014&log$=prottop&blast_rank=33) | G-protein coupled receptor 110 isoform 1 [Homo sapiens] | [18.0](http://blast.ncbi.nlm.nih.gov/Blast.cgi#61743940%2361743940) | 18.0 | 100% | 453 |
| [NP_700356.2](http://www.ncbi.nlm.nih.gov/entrez/query.fcgi?cmd=Retrieve&db=Protein&list_uids=40255157&dopt=GenPept&RID=T25AT6S2014&log$=prottop&blast_rank=34) | leucine-rich repeats and immunoglobulin-like domains 3 isoform 2 [Homo sapiens] | [18.0](http://blast.ncbi.nlm.nih.gov/Blast.cgi#40255157%2340255157) | 33.5 | 85% | 453 |
| [NP_775836.3](http://www.ncbi.nlm.nih.gov/entrez/query.fcgi?cmd=Retrieve&db=Protein&list_uids=157502187&dopt=GenPept&RID=T25AT6S2014&log$=prottop&blast_rank=35) | radial spoke head 10 homolog B [Homo sapiens] | [17.6](http://blast.ncbi.nlm.nih.gov/Blast.cgi#157502187%23157502187) | 17.6 | 85% | 608 |
| [NP_001093167.1](http://www.ncbi.nlm.nih.gov/entrez/query.fcgi?cmd=Retrieve&db=Protein&list_uids=153792461&dopt=GenPept&RID=T25AT6S2014&log$=prottop&blast_rank=36) | radial spoke head 10 homolog B2 [Homo sapiens] | [17.6](http://blast.ncbi.nlm.nih.gov/Blast.cgi#153792461%23153792461) | 17.6 | 85% | 608 |
| [NP_056292.1](http://www.ncbi.nlm.nih.gov/entrez/query.fcgi?cmd=Retrieve&db=Protein&list_uids=23397666&dopt=GenPept&RID=T25AT6S2014&log$=prottop&blast_rank=37) | transcriptional co-repressor Sin3A [Homo sapiens] >ref|NP_001138829.1| transcriptional co-repressor Sin3A [Homo sapiens] >ref|NP_001138830.1| transcriptional co-repressor Sin3A [Homo sapiens] | [17.6](http://blast.ncbi.nlm.nih.gov/Blast.cgi#23397666%2323397666) | 17.6 | 85% | 608 |
| [NP_001120866.1](http://www.ncbi.nlm.nih.gov/entrez/query.fcgi?cmd=Retrieve&db=Protein&list_uids=188528677&dopt=GenPept&RID=T25AT6S2014&log$=prottop&blast_rank=38) | tRNA splicing endonuclease 15 isoform 2 [Homo sapiens] | [17.2](http://blast.ncbi.nlm.nih.gov/Blast.cgi#188528677%23188528677) | 17.2 | 71% | 816 |
| [NP_001092688.1](http://www.ncbi.nlm.nih.gov/entrez/query.fcgi?cmd=Retrieve&db=Protein&list_uids=149773468&dopt=GenPept&RID=T25AT6S2014&log$=prottop&blast_rank=39) | RAD51 associated protein 2 [Homo sapiens] | [17.2](http://blast.ncbi.nlm.nih.gov/Blast.cgi#149773468%23149773468) | 17.2 | 85% | 816 |
| [NP_003254.2](http://www.ncbi.nlm.nih.gov/entrez/query.fcgi?cmd=Retrieve&db=Protein&list_uids=41350337&dopt=GenPept&RID=T25AT6S2014&log$=prottop&blast_rank=40) | toll-like receptor 1 precursor [Homo sapiens] | [17.2](http://blast.ncbi.nlm.nih.gov/Blast.cgi#41350337%2341350337) | 31.8 | 85% | 816 |
| [NP_067647.2](http://www.ncbi.nlm.nih.gov/entrez/query.fcgi?cmd=Retrieve&db=Protein&list_uids=85986601&dopt=GenPept&RID=T25AT6S2014&log$=prottop&blast_rank=41) | relaxin/insulin-like family peptide receptor 1 [Homo sapiens] | [17.2](http://blast.ncbi.nlm.nih.gov/Blast.cgi#85986601%2385986601) | 17.2 | 71% | 816 |
| [NP_006059.2](http://www.ncbi.nlm.nih.gov/entrez/query.fcgi?cmd=Retrieve&db=Protein&list_uids=20143971&dopt=GenPept&RID=T25AT6S2014&log$=prottop&blast_rank=42) | toll-like receptor 6 precursor [Homo sapiens] | [17.2](http://blast.ncbi.nlm.nih.gov/Blast.cgi#20143971%2320143971) | 31.8 | 85% | 816 |
| [NP_443197.1](http://www.ncbi.nlm.nih.gov/entrez/query.fcgi?cmd=Retrieve&db=Protein&list_uids=24308390&dopt=GenPept&RID=T25AT6S2014&log$=prottop&blast_rank=43) | tRNA splicing endonuclease 15 isoform 1 [Homo sapiens] | [17.2](http://blast.ncbi.nlm.nih.gov/Blast.cgi#24308390%2324308390) | 17.2 | 71% | 816 |
| [NP_073153.1](http://www.ncbi.nlm.nih.gov/entrez/query.fcgi?cmd=Retrieve&db=Protein&list_uids=12056971&dopt=GenPept&RID=T25AT6S2014&log$=prottop&blast_rank=44) | anaphase promoting complex subunit 1 [Homo sapiens] | [17.2](http://blast.ncbi.nlm.nih.gov/Blast.cgi#12056971%2312056971) | 27.6 | 100% | 816 |
| [NP_940968.1](http://www.ncbi.nlm.nih.gov/entrez/query.fcgi?cmd=Retrieve&db=Protein&list_uids=38348408&dopt=GenPept&RID=T25AT6S2014&log$=prottop&blast_rank=45) | hypothetical protein LOC375444 [Homo sapiens] | [17.2](http://blast.ncbi.nlm.nih.gov/Blast.cgi#38348408%2338348408) | 32.7 | 85% | 816 |
| [NP_878185.1](http://www.ncbi.nlm.nih.gov/entrez/query.fcgi?cmd=Retrieve&db=Protein&list_uids=33598922&dopt=GenPept&RID=T25AT6S2014&log$=prottop&blast_rank=46) | scavenger receptor class A, member 3 isoform 2 [Homo sapiens] | [17.2](http://blast.ncbi.nlm.nih.gov/Blast.cgi#33598922%2333598922) | 17.2 | 71% | 816 |
| [NP_065854.3](http://www.ncbi.nlm.nih.gov/entrez/query.fcgi?cmd=Retrieve&db=Protein&list_uids=34101268&dopt=GenPept&RID=T25AT6S2014&log$=prottop&blast_rank=47) | kelch-like 8 [Homo sapiens] | [17.2](http://blast.ncbi.nlm.nih.gov/Blast.cgi#34101268%2334101268) | 17.2 | 71% | 816 |
| [XP_001133746.2](http://www.ncbi.nlm.nih.gov/entrez/query.fcgi?cmd=Retrieve&db=Protein&list_uids=239752549&dopt=GenPept&RID=T25AT6S2014&log$=prottop&blast_rank=48) | PREDICTED: hypothetical protein [Homo sapiens] | [16.8](http://blast.ncbi.nlm.nih.gov/Blast.cgi#239752549%23239752549) | 16.8 | 57% | 1095 |
| [NP_001152994.1](http://www.ncbi.nlm.nih.gov/entrez/query.fcgi?cmd=Retrieve&db=Protein&list_uids=226958478&dopt=GenPept&RID=T25AT6S2014&log$=prottop&blast_rank=49) | zinc finger protein 727 [Homo sapiens] | [16.8](http://blast.ncbi.nlm.nih.gov/Blast.cgi#226958478%23226958478) | 16.8 | 57% | 1095 |
| [NP_001138638.1](http://www.ncbi.nlm.nih.gov/entrez/query.fcgi?cmd=Retrieve&db=Protein&list_uids=223556008&dopt=GenPept&RID=T25AT6S2014&log$=prottop&blast_rank=50) | protein arginine methyltransferase 3 isoform 2 [Homo sapiens] | [16.8](http://blast.ncbi.nlm.nih.gov/Blast.cgi#223556008%23223556008) | 16.8 | 57% | 1095 |
| [NP_974487.1](http://www.ncbi.nlm.nih.gov/entrez/query.fcgi?cmd=Retrieve&db=Protein&list_uids=42544117&dopt=GenPept&RID=T25AT6S2014&log$=prottop&blast_rank=51) | rTS beta protein isoform rTS gamma [Homo sapiens] | [16.8](http://blast.ncbi.nlm.nih.gov/Blast.cgi#42544117%2342544117) | 16.8 | 57% | 1095 |
| [NP_060565.4](http://www.ncbi.nlm.nih.gov/entrez/query.fcgi?cmd=Retrieve&db=Protein&list_uids=209571547&dopt=GenPept&RID=T25AT6S2014&log$=prottop&blast_rank=52) | kelch repeat and BTB (POZ) domain containing 4 isoform a [Homo sapiens] | [16.8](http://blast.ncbi.nlm.nih.gov/Blast.cgi#209571547%23209571547) | 16.8 | 57% | 1095 |
| [NP_065875.3](http://www.ncbi.nlm.nih.gov/entrez/query.fcgi?cmd=Retrieve&db=Protein&list_uids=203097003&dopt=GenPept&RID=T25AT6S2014&log$=prottop&blast_rank=53) | Rho GTPase activating protein 21 [Homo sapiens] | [16.8](http://blast.ncbi.nlm.nih.gov/Blast.cgi#203097003%23203097003) | 16.8 | 71% | 1095 |
| [NP_940927.2](http://www.ncbi.nlm.nih.gov/entrez/query.fcgi?cmd=Retrieve&db=Protein&list_uids=203096856&dopt=GenPept&RID=T25AT6S2014&log$=prottop&blast_rank=54) | kinesin family member 7 [Homo sapiens] | [16.8](http://blast.ncbi.nlm.nih.gov/Blast.cgi#203096856%23203096856) | 16.8 | 71% | 1095 |
| [XP_002344379.1](http://www.ncbi.nlm.nih.gov/entrez/query.fcgi?cmd=Retrieve&db=Protein&list_uids=239508879&dopt=GenPept&RID=T25AT6S2014&log$=prottop&blast_rank=55) | PREDICTED: similar to zinc finger protein 208 [Homo sapiens] | [16.8](http://blast.ncbi.nlm.nih.gov/Blast.cgi#239508879%23239508879) | 16.8 | 57% | 1095 |
| [NP_061963.2](http://www.ncbi.nlm.nih.gov/entrez/query.fcgi?cmd=Retrieve&db=Protein&list_uids=149192843&dopt=GenPept&RID=T25AT6S2014&log$=prottop&blast_rank=56) | hypothetical protein LOC54627 [Homo sapiens] | [16.8](http://blast.ncbi.nlm.nih.gov/Blast.cgi#149192843%23149192843) | 16.8 | 57% | 1095 |
| [NP_653252.3](http://www.ncbi.nlm.nih.gov/entrez/query.fcgi?cmd=Retrieve&db=Protein&list_uids=126517478&dopt=GenPept&RID=T25AT6S2014&log$=prottop&blast_rank=57) | peroxidasin homolog-like precursor [Homo sapiens] | [16.8](http://blast.ncbi.nlm.nih.gov/Blast.cgi#126517478%23126517478) | 16.8 | 57% | 1095 |
| [NP_001138639.1](http://www.ncbi.nlm.nih.gov/entrez/query.fcgi?cmd=Retrieve&db=Protein&list_uids=223556010&dopt=GenPept&RID=T25AT6S2014&log$=prottop&blast_rank=58) | protein arginine methyltransferase 3 isoform 3 [Homo sapiens] | [16.8](http://blast.ncbi.nlm.nih.gov/Blast.cgi#223556010%23223556010) | 16.8 | 57% | 1095 |
| [NP_004524.3](http://www.ncbi.nlm.nih.gov/entrez/query.fcgi?cmd=Retrieve&db=Protein&list_uids=133908641&dopt=GenPept&RID=T25AT6S2014&log$=prottop&blast_rank=59) | myosin binding protein C, fast type [Homo sapiens] | [16.8](http://blast.ncbi.nlm.nih.gov/Blast.cgi#133908641%23133908641) | 16.8 | 100% | 1095 |
| [NP_055771.4](http://www.ncbi.nlm.nih.gov/entrez/query.fcgi?cmd=Retrieve&db=Protein&list_uids=115648142&dopt=GenPept&RID=T25AT6S2014&log$=prottop&blast_rank=60) | centrosomal protein 164kDa [Homo sapiens] | [16.8](http://blast.ncbi.nlm.nih.gov/Blast.cgi#115648142%23115648142) | 16.8 | 57% | 1095 |
| [NP_851996.2](http://www.ncbi.nlm.nih.gov/entrez/query.fcgi?cmd=Retrieve&db=Protein&list_uids=115529482&dopt=GenPept&RID=T25AT6S2014&log$=prottop&blast_rank=61) | neural cell adhesion molecule 1 isoform 2 [Homo sapiens] | [16.8](http://blast.ncbi.nlm.nih.gov/Blast.cgi#115529482%23115529482) | 16.8 | 85% | 1095 |
| [NP_689419.2](http://www.ncbi.nlm.nih.gov/entrez/query.fcgi?cmd=Retrieve&db=Protein&list_uids=88703041&dopt=GenPept&RID=T25AT6S2014&log$=prottop&blast_rank=62) | sorting nexin 6 isoform b [Homo sapiens] | [16.8](http://blast.ncbi.nlm.nih.gov/Blast.cgi#88703041%2388703041) | 16.8 | 57% | 1095 |
| [NP_005913.2](http://www.ncbi.nlm.nih.gov/entrez/query.fcgi?cmd=Retrieve&db=Protein&list_uids=55956904&dopt=GenPept&RID=T25AT6S2014&log$=prottop&blast_rank=63) | mitogen-activated protein kinase kinase kinase 4 isoform a [Homo sapiens] | [16.8](http://blast.ncbi.nlm.nih.gov/Blast.cgi#55956904%2355956904) | 16.8 | 71% | 1095 |
| [NP_006715.2](http://www.ncbi.nlm.nih.gov/entrez/query.fcgi?cmd=Retrieve&db=Protein&list_uids=55956902&dopt=GenPept&RID=T25AT6S2014&log$=prottop&blast_rank=64) | mitogen-activated protein kinase kinase kinase 4 isoform b [Homo sapiens] | [16.8](http://blast.ncbi.nlm.nih.gov/Blast.cgi#55956902%2355956902) | 16.8 | 71% | 1095 |
| [NP_001013675.1](http://www.ncbi.nlm.nih.gov/entrez/query.fcgi?cmd=Retrieve&db=Protein&list_uids=61966761&dopt=GenPept&RID=T25AT6S2014&log$=prottop&blast_rank=65) | leucine rich repeat containing 26 precursor [Homo sapiens] | [16.8](http://blast.ncbi.nlm.nih.gov/Blast.cgi#61966761%2361966761) | 16.8 | 57% | 1095 |
| [NP_954868.1](http://www.ncbi.nlm.nih.gov/entrez/query.fcgi?cmd=Retrieve&db=Protein&list_uids=40549433&dopt=GenPept&RID=T25AT6S2014&log$=prottop&blast_rank=66) | transmembrane phosphatase with tensin homology isoform beta [Homo sapiens] | [16.8](http://blast.ncbi.nlm.nih.gov/Blast.cgi#40549433%2340549433) | 16.8 | 57% | 1095 |
| [NP_066267.2](http://www.ncbi.nlm.nih.gov/entrez/query.fcgi?cmd=Retrieve&db=Protein&list_uids=32967601&dopt=GenPept&RID=T25AT6S2014&log$=prottop&blast_rank=67) | ankyrin 3 isoform 1 [Homo sapiens] | [16.8](http://blast.ncbi.nlm.nih.gov/Blast.cgi#32967601%2332967601) | 16.8 | 57% | 1095 |
| [NP_001036038.1](http://www.ncbi.nlm.nih.gov/entrez/query.fcgi?cmd=Retrieve&db=Protein&list_uids=110431350&dopt=GenPept&RID=T25AT6S2014&log$=prottop&blast_rank=68) | endo-beta-N-acetylglucosaminidase [Homo sapiens] | [16.8](http://blast.ncbi.nlm.nih.gov/Blast.cgi#110431350%23110431350) | 16.8 | 57% | 1095 |
| [NP_001019553.1](http://www.ncbi.nlm.nih.gov/entrez/query.fcgi?cmd=Retrieve&db=Protein&list_uids=66933009&dopt=GenPept&RID=T25AT6S2014&log$=prottop&blast_rank=69) | hydroxymethylbilane synthase isoform 2 [Homo sapiens] | [16.8](http://blast.ncbi.nlm.nih.gov/Blast.cgi#66933009%2366933009) | 16.8 | 57% | 1095 |
| [NP_115609.2](http://www.ncbi.nlm.nih.gov/entrez/query.fcgi?cmd=Retrieve&db=Protein&list_uids=40068481&dopt=GenPept&RID=T25AT6S2014&log$=prottop&blast_rank=70) | SET domain containing 3 isoform a [Homo sapiens] | [16.8](http://blast.ncbi.nlm.nih.gov/Blast.cgi#40068481%2340068481) | 16.8 | 71% | 1095 |
| [NP_116164.2](http://www.ncbi.nlm.nih.gov/entrez/query.fcgi?cmd=Retrieve&db=Protein&list_uids=24432026&dopt=GenPept&RID=T25AT6S2014&log$=prottop&blast_rank=71) | kelch-like [Homo sapiens] | [16.8](http://blast.ncbi.nlm.nih.gov/Blast.cgi#24432026%2324432026) | 16.8 | 85% | 1095 |
| [NP_705834.2](http://www.ncbi.nlm.nih.gov/entrez/query.fcgi?cmd=Retrieve&db=Protein&list_uids=282721094&dopt=GenPept&RID=T25AT6S2014&log$=prottop&blast_rank=72) | hypothetical protein LOC149647 [Homo sapiens] | [16.8](http://blast.ncbi.nlm.nih.gov/Blast.cgi#282721094%23282721094) | 27.6 | 85% | 1095 |
| [NP_067072.3](http://www.ncbi.nlm.nih.gov/entrez/query.fcgi?cmd=Retrieve&db=Protein&list_uids=88703043&dopt=GenPept&RID=T25AT6S2014&log$=prottop&blast_rank=73) | sorting nexin 6 isoform a [Homo sapiens] | [16.8](http://blast.ncbi.nlm.nih.gov/Blast.cgi#88703043%2388703043) | 16.8 | 57% | 1095 |
| [NP_570969.2](http://www.ncbi.nlm.nih.gov/entrez/query.fcgi?cmd=Retrieve&db=Protein&list_uids=222418633&dopt=GenPept&RID=T25AT6S2014&log$=prottop&blast_rank=74) | family with sequence similarity 71, member B [Homo sapiens] | [16.8](http://blast.ncbi.nlm.nih.gov/Blast.cgi#222418633%23222418633) | 16.8 | 57% | 1095 |
| [NP_059982.2](http://www.ncbi.nlm.nih.gov/entrez/query.fcgi?cmd=Retrieve&db=Protein&list_uids=42544119&dopt=GenPept&RID=T25AT6S2014&log$=prottop&blast_rank=75) | enolase superfamily 1 isoform rTS beta [Homo sapiens] | [16.8](http://blast.ncbi.nlm.nih.gov/Blast.cgi#42544119%2342544119) | 16.8 | 57% | 1095 |
| [NP_004095.4](http://www.ncbi.nlm.nih.gov/entrez/query.fcgi?cmd=Retrieve&db=Protein&list_uids=41872631&dopt=GenPept&RID=T25AT6S2014&log$=prottop&blast_rank=76) | fatty acid synthase [Homo sapiens] | [16.8](http://blast.ncbi.nlm.nih.gov/Blast.cgi#41872631%2341872631) | 16.8 | 57% | 1095 |
| [NP_659419.3](http://www.ncbi.nlm.nih.gov/entrez/query.fcgi?cmd=Retrieve&db=Protein&list_uids=87116683&dopt=GenPept&RID=T25AT6S2014&log$=prottop&blast_rank=77) | proline/serine-rich coiled-coil 2 [Homo sapiens] | [16.8](http://blast.ncbi.nlm.nih.gov/Blast.cgi#87116683%2387116683) | 16.8 | 57% | 1095 |
| [NP_954869.1](http://www.ncbi.nlm.nih.gov/entrez/query.fcgi?cmd=Retrieve&db=Protein&list_uids=40549435&dopt=GenPept&RID=T25AT6S2014&log$=prottop&blast_rank=78) | transmembrane phosphatase with tensin homology isoform gamma [Homo sapiens] | [16.8](http://blast.ncbi.nlm.nih.gov/Blast.cgi#40549435%2340549435) | 16.8 | 57% | 1095 |
| [NP_954870.2](http://www.ncbi.nlm.nih.gov/entrez/query.fcgi?cmd=Retrieve&db=Protein&list_uids=109689707&dopt=GenPept&RID=T25AT6S2014&log$=prottop&blast_rank=79) | transmembrane phosphatase with tensin homology isoform alpha [Homo sapiens] | [16.8](http://blast.ncbi.nlm.nih.gov/Blast.cgi#109689707%23109689707) | 16.8 | 57% | 1095 |
| [NP_065075.1](http://www.ncbi.nlm.nih.gov/entrez/query.fcgi?cmd=Retrieve&db=Protein&list_uids=55741750&dopt=GenPept&RID=T25AT6S2014&log$=prottop&blast_rank=80) | solute carrier family 39 (zinc transporter), member 10 precursor [Homo sapiens] >ref|NP_001120729.1| solute carrier family 39 (zinc transporter), member 10 precursor [Homo sapiens] | [16.8](http://blast.ncbi.nlm.nih.gov/Blast.cgi#55741750%2355741750) | 40.1 | 100% | 1095 |
| [NP_005779.1](http://www.ncbi.nlm.nih.gov/entrez/query.fcgi?cmd=Retrieve&db=Protein&list_uids=44771198&dopt=GenPept&RID=T25AT6S2014&log$=prottop&blast_rank=81) | protein arginine methyltransferase 3 isoform 1 [Homo sapiens] | [16.8](http://blast.ncbi.nlm.nih.gov/Blast.cgi#44771198%2344771198) | 16.8 | 57% | 1095 |
| [NP_006436.3](http://www.ncbi.nlm.nih.gov/entrez/query.fcgi?cmd=Retrieve&db=Protein&list_uids=91208426&dopt=GenPept&RID=T25AT6S2014&log$=prottop&blast_rank=82) | U5 snRNP-specific protein [Homo sapiens] | [16.8](http://blast.ncbi.nlm.nih.gov/Blast.cgi#91208426%2391208426) | 16.8 | 57% | 1095 |
| [NP_005958.1](http://www.ncbi.nlm.nih.gov/entrez/query.fcgi?cmd=Retrieve&db=Protein&list_uids=5174607&dopt=GenPept&RID=T25AT6S2014&log$=prottop&blast_rank=83) | NGFI-A binding protein 2 [Homo sapiens] | [16.8](http://blast.ncbi.nlm.nih.gov/Blast.cgi#5174607%235174607) | 16.8 | 71% | 1095 |
| [NP_001914.3](http://www.ncbi.nlm.nih.gov/entrez/query.fcgi?cmd=Retrieve&db=Protein&list_uids=148529014&dopt=GenPept&RID=T25AT6S2014&log$=prottop&blast_rank=84) | damage-specific DNA binding protein 1 [Homo sapiens] >ref|XP_002347285.1| PREDICTED: hypothetical protein XP_002347285 [Homo sapiens] | [16.8](http://blast.ncbi.nlm.nih.gov/Blast.cgi#148529014%23148529014) | 16.8 | 57% | 1095 |
| [NP_000181.2](http://www.ncbi.nlm.nih.gov/entrez/query.fcgi?cmd=Retrieve&db=Protein&list_uids=20149500&dopt=GenPept&RID=T25AT6S2014&log$=prottop&blast_rank=85) | hydroxymethylbilane synthase isoform 1 [Homo sapiens] | [16.8](http://blast.ncbi.nlm.nih.gov/Blast.cgi#20149500%2320149500) | 16.8 | 57% | 1095 |
| [NP_000606.3](http://www.ncbi.nlm.nih.gov/entrez/query.fcgi?cmd=Retrieve&db=Protein&list_uids=94420689&dopt=GenPept&RID=T25AT6S2014&log$=prottop&blast_rank=86) | neural cell adhesion molecule 1 isoform 1 [Homo sapiens] | [16.8](http://blast.ncbi.nlm.nih.gov/Blast.cgi#94420689%2394420689) | 16.8 | 85% | 1095 |
| [NP_001070150.1](http://www.ncbi.nlm.nih.gov/entrez/query.fcgi?cmd=Retrieve&db=Protein&list_uids=115529478&dopt=GenPept&RID=T25AT6S2014&log$=prottop&blast_rank=87) | neural cell adhesion molecule 1 isoform 3 [Homo sapiens] | [16.8](http://blast.ncbi.nlm.nih.gov/Blast.cgi#115529478%23115529478) | 16.8 | 85% | 1095 |
| [NP_001119595.1](http://www.ncbi.nlm.nih.gov/entrez/query.fcgi?cmd=Retrieve&db=Protein&list_uids=186972148&dopt=GenPept&RID=T25AT6S2014&log$=prottop&blast_rank=88) | enolase superfamily 1 isoform rTSalpha [Homo sapiens] | [16.8](http://blast.ncbi.nlm.nih.gov/Blast.cgi#186972148%23186972148) | 16.8 | 57% | 1095 |
| [NP_002419.1](http://www.ncbi.nlm.nih.gov/entrez/query.fcgi?cmd=Retrieve&db=Protein&list_uids=4505211&dopt=GenPept&RID=T25AT6S2014&log$=prottop&blast_rank=89) | matrix metalloproteinase 15 preproprotein [Homo sapiens] | [16.8](http://blast.ncbi.nlm.nih.gov/Blast.cgi#4505211%234505211) | 16.8 | 57% | 1095 |
| [NP_542158.1](http://www.ncbi.nlm.nih.gov/entrez/query.fcgi?cmd=Retrieve&db=Protein&list_uids=18104969&dopt=GenPept&RID=T25AT6S2014&log$=prottop&blast_rank=90) | prostaglandin-endoperoxide synthase 1 isoform 2 precursor [Homo sapiens] | [16.8](http://blast.ncbi.nlm.nih.gov/Blast.cgi#18104969%2318104969) | 16.8 | 57% | 1095 |
| [NP_000954.1](http://www.ncbi.nlm.nih.gov/entrez/query.fcgi?cmd=Retrieve&db=Protein&list_uids=4506265&dopt=GenPept&RID=T25AT6S2014&log$=prottop&blast_rank=91) | prostaglandin-endoperoxide synthase 2 precursor [Homo sapiens] | [16.8](http://blast.ncbi.nlm.nih.gov/Blast.cgi#4506265%234506265) | 16.8 | 57% | 1095 |
| [NP_000953.2](http://www.ncbi.nlm.nih.gov/entrez/query.fcgi?cmd=Retrieve&db=Protein&list_uids=18104967&dopt=GenPept&RID=T25AT6S2014&log$=prottop&blast_rank=92) | prostaglandin-endoperoxide synthase 1 isoform 1 precursor [Homo sapiens] | [16.8](http://blast.ncbi.nlm.nih.gov/Blast.cgi#18104967%2318104967) | 16.8 | 57% | 1095 |
| [NP_542787.1](http://www.ncbi.nlm.nih.gov/entrez/query.fcgi?cmd=Retrieve&db=Protein&list_uids=18152787&dopt=GenPept&RID=T25AT6S2014&log$=prottop&blast_rank=93) | neuralized-like protein 2 [Homo sapiens] | [16.8](http://blast.ncbi.nlm.nih.gov/Blast.cgi#18152787%2318152787) | 16.8 | 57% | 1095 |
| [NP_002537.3](http://www.ncbi.nlm.nih.gov/entrez/query.fcgi?cmd=Retrieve&db=Protein&list_uids=148743793&dopt=GenPept&RID=T25AT6S2014&log$=prottop&blast_rank=94) | osteoprotegerin precursor [Homo sapiens] | [16.8](http://blast.ncbi.nlm.nih.gov/Blast.cgi#148743793%23148743793) | 16.8 | 57% | 1095 |
| [NP_066997.3](http://www.ncbi.nlm.nih.gov/entrez/query.fcgi?cmd=Retrieve&db=Protein&list_uids=24432106&dopt=GenPept&RID=T25AT6S2014&log$=prottop&blast_rank=95) | p30 DBC protein [Homo sapiens] >ref|NP_954675.1| p30 DBC protein [Homo sapiens] | [16.8](http://blast.ncbi.nlm.nih.gov/Blast.cgi#24432106%2324432106) | 16.8 | 100% | 1095 |
| [NP_057590.3](http://www.ncbi.nlm.nih.gov/entrez/query.fcgi?cmd=Retrieve&db=Protein&list_uids=209693446&dopt=GenPept&RID=T25AT6S2014&log$=prottop&blast_rank=96) | kelch repeat and BTB (POZ) domain containing 4 isoform b [Homo sapiens] | [16.8](http://blast.ncbi.nlm.nih.gov/Blast.cgi#209693446%23209693446) | 16.8 | 57% | 1095 |
| [NP_001166251.1](http://www.ncbi.nlm.nih.gov/entrez/query.fcgi?cmd=Retrieve&db=Protein&list_uids=289666782&dopt=GenPept&RID=T25AT6S2014&log$=prottop&blast_rank=97) | leucine rich repeat containing 34 isoform 1 [Homo sapiens] | [16.3](http://blast.ncbi.nlm.nih.gov/Blast.cgi#289666782%23289666782) | 16.3 | 57% | 1469 |
| [XP_002343437.1](http://www.ncbi.nlm.nih.gov/entrez/query.fcgi?cmd=Retrieve&db=Protein&list_uids=239745320&dopt=GenPept&RID=T25AT6S2014&log$=prottop&blast_rank=98) | PREDICTED: hypothetical protein XP_002343437 [Homo sapiens] >ref|XP_002347596.1| PREDICTED: hypothetical protein [Homo sapiens] | [16.3](http://blast.ncbi.nlm.nih.gov/Blast.cgi#239745320%23239745320) | 16.3 | 57% | 1469 |
| [NP_001138548.1](http://www.ncbi.nlm.nih.gov/entrez/query.fcgi?cmd=Retrieve&db=Protein&list_uids=223029510&dopt=GenPept&RID=T25AT6S2014&log$=prottop&blast_rank=99) | echinoderm microtubule associated protein like 4 isoform b [Homo sapiens] | [16.3](http://blast.ncbi.nlm.nih.gov/Blast.cgi#223029510%23223029510) | 16.3 | 57% | 1469 |
| [NP_694996.5](http://www.ncbi.nlm.nih.gov/entrez/query.fcgi?cmd=Retrieve&db=Protein&list_uids=183583553&dopt=GenPept&RID=T25AT6S2014&log$=prottop&blast_rank=100) | collagen, type XXIX, alpha 1 [Homo sapiens] | [16.3](http://blast.ncbi.nlm.nih.gov/Blast.cgi#183583553%23183583553) | 16.3 | 71% | 1469 |

| **Accession** | **Proteins with a match to KKGMGHHGNG peptide** | **[Max score](http://blast.ncbi.nlm.nih.gov/Blast.cgi?CMD=Get&ALIGNMENTS=100&ALIGNMENT_VIEW=Pairwise&CDD_SEARCH_STATE=1&DATABASE_SORT=0&DESCRIPTIONS=100&ENTREZ_QUERY=txid9606 %5BORGN%5D&FIRST_QUERY_NUM=0&FORMAT_OBJECT=Alignment&FORMAT_PAGE_TARGET=&FORMAT_TYPE=HTML&GET_SEQUENCE=yes&I_THRESH=&MASK_CHAR=2&MASK_COLOR=1&NEW_DESIGN=on&NEW_VIEW=yes&NUM_OVERVIEW=100&OLD_BLAST=false&PAGE=Proteins&QUERY_INDEX=0&QUERY_NUMBER=0&RESULTS_PAGE_TARGET=&RID=T25GAK6Z016&SHOW_LINKOUT=yes&SHOW_OVERVIEW=yes&STEP_NUMBER=&WORD_SIZE=2&DISPLAY_SORT=1&HSP_SORT=1" \l "sort_mark)** | **[Total score](http://blast.ncbi.nlm.nih.gov/Blast.cgi?CMD=Get&ALIGNMENTS=100&ALIGNMENT_VIEW=Pairwise&CDD_SEARCH_STATE=1&DATABASE_SORT=0&DESCRIPTIONS=100&ENTREZ_QUERY=txid9606 %5BORGN%5D&FIRST_QUERY_NUM=0&FORMAT_OBJECT=Alignment&FORMAT_PAGE_TARGET=&FORMAT_TYPE=HTML&GET_SEQUENCE=yes&I_THRESH=&MASK_CHAR=2&MASK_COLOR=1&NEW_DESIGN=on&NEW_VIEW=yes&NUM_OVERVIEW=100&OLD_BLAST=false&PAGE=Proteins&QUERY_INDEX=0&QUERY_NUMBER=0&RESULTS_PAGE_TARGET=&RID=T25GAK6Z016&SHOW_LINKOUT=yes&SHOW_OVERVIEW=yes&STEP_NUMBER=&WORD_SIZE=2&DISPLAY_SORT=2&HSP_SORT=1" \l "sort_mark)** | **[Query coverage](http://blast.ncbi.nlm.nih.gov/Blast.cgi?CMD=Get&ALIGNMENTS=100&ALIGNMENT_VIEW=Pairwise&CDD_SEARCH_STATE=1&DATABASE_SORT=0&DESCRIPTIONS=100&ENTREZ_QUERY=txid9606 %5BORGN%5D&FIRST_QUERY_NUM=0&FORMAT_OBJECT=Alignment&FORMAT_PAGE_TARGET=&FORMAT_TYPE=HTML&GET_SEQUENCE=yes&I_THRESH=&MASK_CHAR=2&MASK_COLOR=1&NEW_DESIGN=on&NEW_VIEW=yes&NUM_OVERVIEW=100&OLD_BLAST=false&PAGE=Proteins&QUERY_INDEX=0&QUERY_NUMBER=0&RESULTS_PAGE_TARGET=&RID=T25GAK6Z016&SHOW_LINKOUT=yes&SHOW_OVERVIEW=yes&STEP_NUMBER=&WORD_SIZE=2&DISPLAY_SORT=4&HSP_SORT=0" \l "sort_mark)** | **[E value](http://blast.ncbi.nlm.nih.gov/Blast.cgi?CMD=Get&ALIGNMENTS=100&ALIGNMENT_VIEW=Pairwise&CDD_SEARCH_STATE=1&DATABASE_SORT=0&DESCRIPTIONS=100&ENTREZ_QUERY=txid9606 %5BORGN%5D&FIRST_QUERY_NUM=0&FORMAT_OBJECT=Alignment&FORMAT_PAGE_TARGET=&FORMAT_TYPE=HTML&GET_SEQUENCE=yes&I_THRESH=&MASK_CHAR=2&MASK_COLOR=1&NEW_DESIGN=on&NEW_VIEW=yes&NUM_OVERVIEW=100&OLD_BLAST=false&PAGE=Proteins&QUERY_INDEX=0&QUERY_NUMBER=0&RESULTS_PAGE_TARGET=&RID=T25GAK6Z016&SHOW_LINKOUT=yes&SHOW_OVERVIEW=yes&STEP_NUMBER=&WORD_SIZE=2&DISPLAY_SORT=0&HSP_SORT=0" \l "sort_mark)** |
| --- | --- | --- | --- | --- | --- |
| [NP_057736.4](http://www.ncbi.nlm.nih.gov/entrez/query.fcgi?cmd=Retrieve&db=Protein&list_uids=124256489&dopt=GenPept&RID=T25GAK6Z016&log$=prottop&blast_rank=1) | crooked neck-like 1 protein [Homo sapiens] | [21.8](http://blast.ncbi.nlm.nih.gov/Blast.cgi#124256489%23124256489) | 21.8 | 60% | 46 |
| [NP_078813.1](http://www.ncbi.nlm.nih.gov/entrez/query.fcgi?cmd=Retrieve&db=Protein&list_uids=13375695&dopt=GenPept&RID=T25GAK6Z016&log$=prottop&blast_rank=2) | cysteinyl-tRNA synthetase 2, mitochondrial precursor [Homo sapiens] | [21.4](http://blast.ncbi.nlm.nih.gov/Blast.cgi#13375695%2313375695) | 21.4 | 80% | 62 |
| [NP_001019628.3](http://www.ncbi.nlm.nih.gov/entrez/query.fcgi?cmd=Retrieve&db=Protein&list_uids=262118265&dopt=GenPept&RID=T25GAK6Z016&log$=prottop&blast_rank=3) | RANBP2-like and GRIP domain containing 1 [Homo sapiens] | [20.6](http://blast.ncbi.nlm.nih.gov/Blast.cgi#262118265%23262118265) | 20.6 | 60% | 111 |
| [NP_872394.2](http://www.ncbi.nlm.nih.gov/entrez/query.fcgi?cmd=Retrieve&db=Protein&list_uids=211059431&dopt=GenPept&RID=T25GAK6Z016&log$=prottop&blast_rank=4) | RANBP2-like and GRIP domain containing 4 [Homo sapiens] | [20.6](http://blast.ncbi.nlm.nih.gov/Blast.cgi#211059431%23211059431) | 20.6 | 60% | 111 |
| [NP_001157935.1](http://www.ncbi.nlm.nih.gov/entrez/query.fcgi?cmd=Retrieve&db=Protein&list_uids=256600210&dopt=GenPept&RID=T25GAK6Z016&log$=prottop&blast_rank=5) | RANBP2-like and GRIP domain containing 8 [Homo sapiens] | [20.6](http://blast.ncbi.nlm.nih.gov/Blast.cgi#256600210%23256600210) | 20.6 | 60% | 111 |
| [NP_004418.2](http://www.ncbi.nlm.nih.gov/entrez/query.fcgi?cmd=Retrieve&db=Protein&list_uids=37595530&dopt=GenPept&RID=T25GAK6Z016&log$=prottop&blast_rank=6) | polyhomeotic-like 2 isoform b [Homo sapiens] | [20.6](http://blast.ncbi.nlm.nih.gov/Blast.cgi#37595530%2337595530) | 20.6 | 50% | 111 |
| [NP_001071638.2](http://www.ncbi.nlm.nih.gov/entrez/query.fcgi?cmd=Retrieve&db=Protein&list_uids=262118271&dopt=GenPept&RID=T25GAK6Z016&log$=prottop&blast_rank=7) | RANBP2-like and GRIP domain containing 2 [Homo sapiens] | [20.6](http://blast.ncbi.nlm.nih.gov/Blast.cgi#262118271%23262118271) | 20.6 | 60% | 111 |
| [NP_001137485.1](http://www.ncbi.nlm.nih.gov/entrez/query.fcgi?cmd=Retrieve&db=Protein&list_uids=221307607&dopt=GenPept&RID=T25GAK6Z016&log$=prottop&blast_rank=8) | RANBP2-like and GRIP domain containing 3 [Homo sapiens] | [20.6](http://blast.ncbi.nlm.nih.gov/Blast.cgi#221307607%23221307607) | 20.6 | 60% | 111 |
| [NP_932157.1](http://www.ncbi.nlm.nih.gov/entrez/query.fcgi?cmd=Retrieve&db=Protein&list_uids=37595528&dopt=GenPept&RID=T25GAK6Z016&log$=prottop&blast_rank=9) | polyhomeotic-like 2 isoform a [Homo sapiens] | [20.6](http://blast.ncbi.nlm.nih.gov/Blast.cgi#37595528%2337595528) | 20.6 | 50% | 111 |
| [NP_115636.1](http://www.ncbi.nlm.nih.gov/entrez/query.fcgi?cmd=Retrieve&db=Protein&list_uids=14149991&dopt=GenPept&RID=T25GAK6Z016&log$=prottop&blast_rank=10) | RANBP2-like and GRIP domain containing 5 isoform 2 [Homo sapiens] >ref|NP_001032955.1| RANBP2-like and GRIP domain containing 6 isoform 2 [Homo sapiens] | [20.6](http://blast.ncbi.nlm.nih.gov/Blast.cgi#14149991%2314149991) | 20.6 | 60% | 111 |
| [NP_005045.2](http://www.ncbi.nlm.nih.gov/entrez/query.fcgi?cmd=Retrieve&db=Protein&list_uids=83267877&dopt=GenPept&RID=T25GAK6Z016&log$=prottop&blast_rank=11) | RANBP2-like and GRIP domain containing 5 isoform 1 [Homo sapiens] >ref|NP_001116835.1| RANBP2-like and GRIP domain containing 6 isoform 1 [Homo sapiens] | [20.6](http://blast.ncbi.nlm.nih.gov/Blast.cgi#83267877%2383267877) | 20.6 | 60% | 111 |
| [NP_599028.1](http://www.ncbi.nlm.nih.gov/entrez/query.fcgi?cmd=Retrieve&db=Protein&list_uids=20336282&dopt=GenPept&RID=T25GAK6Z016&log$=prottop&blast_rank=12) | solute carrier family 26, member 7 isoform b [Homo sapiens] | [20.6](http://blast.ncbi.nlm.nih.gov/Blast.cgi#20336282%2320336282) | 20.6 | 50% | 111 |
| [NP_439897.1](http://www.ncbi.nlm.nih.gov/entrez/query.fcgi?cmd=Retrieve&db=Protein&list_uids=16306483&dopt=GenPept&RID=T25GAK6Z016&log$=prottop&blast_rank=13) | solute carrier family 26, member 7 isoform a [Homo sapiens] | [20.6](http://blast.ncbi.nlm.nih.gov/Blast.cgi#16306483%2316306483) | 20.6 | 50% | 111 |
| [NP_001036182.1](http://www.ncbi.nlm.nih.gov/entrez/query.fcgi?cmd=Retrieve&db=Protein&list_uids=111955063&dopt=GenPept&RID=T25GAK6Z016&log$=prottop&blast_rank=14) | GTP-binding protein 10 isoform 1 [Homo sapiens] | [19.7](http://blast.ncbi.nlm.nih.gov/Blast.cgi#111955063%23111955063) | 19.7 | 50% | 200 |
| [NP_149098.2](http://www.ncbi.nlm.nih.gov/entrez/query.fcgi?cmd=Retrieve&db=Protein&list_uids=111955139&dopt=GenPept&RID=T25GAK6Z016&log$=prottop&blast_rank=15) | GTP-binding protein 10 isoform 2 [Homo sapiens] | [19.7](http://blast.ncbi.nlm.nih.gov/Blast.cgi#111955139%23111955139) | 19.7 | 50% | 200 |
| [NP_694941.2](http://www.ncbi.nlm.nih.gov/entrez/query.fcgi?cmd=Retrieve&db=Protein&list_uids=156616271&dopt=GenPept&RID=T25GAK6Z016&log$=prottop&blast_rank=16) | kinesin family member 19 [Homo sapiens] | [19.3](http://blast.ncbi.nlm.nih.gov/Blast.cgi#156616271%23156616271) | 19.3 | 60% | 268 |
| [NP_006028.2](http://www.ncbi.nlm.nih.gov/entrez/query.fcgi?cmd=Retrieve&db=Protein&list_uids=153085395&dopt=GenPept&RID=T25GAK6Z016&log$=prottop&blast_rank=17) | histone deacetylase 4 [Homo sapiens] | [19.3](http://blast.ncbi.nlm.nih.gov/Blast.cgi#153085395%23153085395) | 19.3 | 50% | 268 |
| [NP_006035.2](http://www.ncbi.nlm.nih.gov/entrez/query.fcgi?cmd=Retrieve&db=Protein&list_uids=13128864&dopt=GenPept&RID=T25GAK6Z016&log$=prottop&blast_rank=18) | histone deacetylase 6 [Homo sapiens] | [19.3](http://blast.ncbi.nlm.nih.gov/Blast.cgi#13128864%2313128864) | 36.1 | 50% | 268 |
| [NP_001015053.1](http://www.ncbi.nlm.nih.gov/entrez/query.fcgi?cmd=Retrieve&db=Protein&list_uids=62750349&dopt=GenPept&RID=T25GAK6Z016&log$=prottop&blast_rank=19) | histone deacetylase 5 isoform 3 [Homo sapiens] | [19.3](http://blast.ncbi.nlm.nih.gov/Blast.cgi#62750349%2362750349) | 19.3 | 50% | 268 |
| [NP_005465.2](http://www.ncbi.nlm.nih.gov/entrez/query.fcgi?cmd=Retrieve&db=Protein&list_uids=62750347&dopt=GenPept&RID=T25GAK6Z016&log$=prottop&blast_rank=20) | histone deacetylase 5 isoform 1 [Homo sapiens] | [19.3](http://blast.ncbi.nlm.nih.gov/Blast.cgi#62750347%2362750347) | 19.3 | 50% | 268 |
| [NP_001091886.1](http://www.ncbi.nlm.nih.gov/entrez/query.fcgi?cmd=Retrieve&db=Protein&list_uids=148539870&dopt=GenPept&RID=T25GAK6Z016&log$=prottop&blast_rank=21) | histone deacetylase 7 isoform d [Homo sapiens] | [19.3](http://blast.ncbi.nlm.nih.gov/Blast.cgi#148539870%23148539870) | 19.3 | 50% | 268 |
| [NP_056216.2](http://www.ncbi.nlm.nih.gov/entrez/query.fcgi?cmd=Retrieve&db=Protein&list_uids=169234807&dopt=GenPept&RID=T25GAK6Z016&log$=prottop&blast_rank=22) | histone deacetylase 7 isoform a [Homo sapiens] | [19.3](http://blast.ncbi.nlm.nih.gov/Blast.cgi#169234807%23169234807) | 19.3 | 50% | 268 |
| [NP_848512.1](http://www.ncbi.nlm.nih.gov/entrez/query.fcgi?cmd=Retrieve&db=Protein&list_uids=30795204&dopt=GenPept&RID=T25GAK6Z016&log$=prottop&blast_rank=23) | histone deacetylase 9 isoform 5 [Homo sapiens] | [19.3](http://blast.ncbi.nlm.nih.gov/Blast.cgi#30795204%2330795204) | 19.3 | 50% | 268 |
| [NP_478057.1](http://www.ncbi.nlm.nih.gov/entrez/query.fcgi?cmd=Retrieve&db=Protein&list_uids=17158041&dopt=GenPept&RID=T25GAK6Z016&log$=prottop&blast_rank=24) | histone deacetylase 9 isoform 2 [Homo sapiens] | [19.3](http://blast.ncbi.nlm.nih.gov/Blast.cgi#17158041%2317158041) | 19.3 | 50% | 268 |
| [NP_001018082.1](http://www.ncbi.nlm.nih.gov/entrez/query.fcgi?cmd=Retrieve&db=Protein&list_uids=65786661&dopt=GenPept&RID=T25GAK6Z016&log$=prottop&blast_rank=25) | BTB (POZ) domain containing 11 isoform a [Homo sapiens] | [19.3](http://blast.ncbi.nlm.nih.gov/Blast.cgi#65786661%2365786661) | 19.3 | 50% | 268 |
| [NP_036549.2](http://www.ncbi.nlm.nih.gov/entrez/query.fcgi?cmd=Retrieve&db=Protein&list_uids=32307140&dopt=GenPept&RID=T25GAK6Z016&log$=prottop&blast_rank=26) | phosphatidylinositol transfer protein, cytoplasmic 1 isoform a [Homo sapiens] | [19.3](http://blast.ncbi.nlm.nih.gov/Blast.cgi#32307140%2332307140) | 19.3 | 50% | 268 |
| [NP_000879.2](http://www.ncbi.nlm.nih.gov/entrez/query.fcgi?cmd=Retrieve&db=Protein&list_uids=9625002&dopt=GenPept&RID=T25GAK6Z016&log$=prottop&blast_rank=27) | integrin, beta 6 precursor [Homo sapiens] | [19.3](http://blast.ncbi.nlm.nih.gov/Blast.cgi#9625002%239625002) | 19.3 | 50% | 268 |
| [NP_858057.1](http://www.ncbi.nlm.nih.gov/entrez/query.fcgi?cmd=Retrieve&db=Protein&list_uids=32307142&dopt=GenPept&RID=T25GAK6Z016&log$=prottop&blast_rank=28) | phosphatidylinositol transfer protein, cytoplasmic 1 isoform b [Homo sapiens] | [19.3](http://blast.ncbi.nlm.nih.gov/Blast.cgi#32307142%2332307142) | 19.3 | 50% | 268 |
| [NP_848510.1](http://www.ncbi.nlm.nih.gov/entrez/query.fcgi?cmd=Retrieve&db=Protein&list_uids=30795202&dopt=GenPept&RID=T25GAK6Z016&log$=prottop&blast_rank=29) | histone deacetylase 9 isoform 4 [Homo sapiens] | [19.3](http://blast.ncbi.nlm.nih.gov/Blast.cgi#30795202%2330795202) | 19.3 | 50% | 268 |
| [NP_478056.1](http://www.ncbi.nlm.nih.gov/entrez/query.fcgi?cmd=Retrieve&db=Protein&list_uids=17158039&dopt=GenPept&RID=T25GAK6Z016&log$=prottop&blast_rank=30) | histone deacetylase 9 isoform 1 [Homo sapiens] | [19.3](http://blast.ncbi.nlm.nih.gov/Blast.cgi#17158039%2317158039) | 19.3 | 50% | 268 |
| [NP_002684.1](http://www.ncbi.nlm.nih.gov/entrez/query.fcgi?cmd=Retrieve&db=Protein&list_uids=4505937&dopt=GenPept&RID=T25GAK6Z016&log$=prottop&blast_rank=31) | DNA-directed DNA polymerase gamma [Homo sapiens] >ref|NP_001119603.1| DNA-directed DNA polymerase gamma [Homo sapiens] | [19.3](http://blast.ncbi.nlm.nih.gov/Blast.cgi#4505937%234505937) | 19.3 | 50% | 268 |
| [NP_061027.2](http://www.ncbi.nlm.nih.gov/entrez/query.fcgi?cmd=Retrieve&db=Protein&list_uids=93102379&dopt=GenPept&RID=T25GAK6Z016&log$=prottop&blast_rank=32) | low density lipoprotein-related protein 1B precursor [Homo sapiens] | [18.9](http://blast.ncbi.nlm.nih.gov/Blast.cgi#93102379%2393102379) | 18.9 | 70% | 360 |
| [NP_003926.1](http://www.ncbi.nlm.nih.gov/entrez/query.fcgi?cmd=Retrieve&db=Protein&list_uids=4507635&dopt=GenPept&RID=T25GAK6Z016&log$=prottop&blast_rank=33) | topoisomerase (DNA) III beta [Homo sapiens] | [18.9](http://blast.ncbi.nlm.nih.gov/Blast.cgi#4507635%234507635) | 18.9 | 50% | 360 |
| [XP_002343816.1](http://www.ncbi.nlm.nih.gov/entrez/query.fcgi?cmd=Retrieve&db=Protein&list_uids=239746709&dopt=GenPept&RID=T25GAK6Z016&log$=prottop&blast_rank=34) | PREDICTED: hypothetical protein XP_002343816 [Homo sapiens] >ref|XP_002348142.1| PREDICTED: hypothetical protein [Homo sapiens] >ref|XP_002345525.1| PREDICTED: hypothetical protein XP_002345525 [Homo sapiens] | [18.5](http://blast.ncbi.nlm.nih.gov/Blast.cgi#239746709%23239746709) | 18.5 | 60% | 482 |
| [NP_037502.3](http://www.ncbi.nlm.nih.gov/entrez/query.fcgi?cmd=Retrieve&db=Protein&list_uids=118572590&dopt=GenPept&RID=T25GAK6Z016&log$=prottop&blast_rank=35) | oxidative stress induced growth inhibitor 1 isoform 1 [Homo sapiens] | [18.5](http://blast.ncbi.nlm.nih.gov/Blast.cgi#118572590%23118572590) | 18.5 | 60% | 482 |
| [NP_001122401.1](http://www.ncbi.nlm.nih.gov/entrez/query.fcgi?cmd=Retrieve&db=Protein&list_uids=193083163&dopt=GenPept&RID=T25GAK6Z016&log$=prottop&blast_rank=36) | roundabout, axon guidance receptor, homolog 2 isoform ROBO2a [Homo sapiens] | [18.5](http://blast.ncbi.nlm.nih.gov/Blast.cgi#193083163%23193083163) | 18.5 | 60% | 482 |
| [NP_892025.1](http://www.ncbi.nlm.nih.gov/entrez/query.fcgi?cmd=Retrieve&db=Protein&list_uids=33695099&dopt=GenPept&RID=T25GAK6Z016&log$=prottop&blast_rank=37) | oxidative stress induced growth inhibitor 1 isoform 2 [Homo sapiens] >ref|NP_892026.1| oxidative stress induced growth inhibitor 1 isoform 3 [Homo sapiens] | [18.5](http://blast.ncbi.nlm.nih.gov/Blast.cgi#33695099%2333695099) | 18.5 | 60% | 482 |
| [XP_002346428.1](http://www.ncbi.nlm.nih.gov/entrez/query.fcgi?cmd=Retrieve&db=Protein&list_uids=239747348&dopt=GenPept&RID=T25GAK6Z016&log$=prottop&blast_rank=38) | PREDICTED: hypothetical protein XP_002346428 [Homo sapiens] | [18.0](http://blast.ncbi.nlm.nih.gov/Blast.cgi#239747348%23239747348) | 18.0 | 90% | 647 |
| [XP_002343815.1](http://www.ncbi.nlm.nih.gov/entrez/query.fcgi?cmd=Retrieve&db=Protein&list_uids=239746705&dopt=GenPept&RID=T25GAK6Z016&log$=prottop&blast_rank=39) | PREDICTED: hypothetical protein XP_002343815 [Homo sapiens] | [18.0](http://blast.ncbi.nlm.nih.gov/Blast.cgi#239746705%23239746705) | 18.0 | 100% | 647 |
| [NP_001611.1](http://www.ncbi.nlm.nih.gov/entrez/query.fcgi?cmd=Retrieve&db=Protein&list_uids=61743954&dopt=GenPept&RID=T25GAK6Z016&log$=prottop&blast_rank=40) | AHNAK nucleoprotein isoform 1 [Homo sapiens] | [18.0](http://blast.ncbi.nlm.nih.gov/Blast.cgi#61743954%2361743954) | 18.0 | 40% | 647 |
| [NP_001121699.1](http://www.ncbi.nlm.nih.gov/entrez/query.fcgi?cmd=Retrieve&db=Protein&list_uids=190014632&dopt=GenPept&RID=T25GAK6Z016&log$=prottop&blast_rank=41) | UDP-N-acetylglucosamine-2-epimerase/N-acetylmannosamine kinase isoform 1 [Homo sapiens] | [18.0](http://blast.ncbi.nlm.nih.gov/Blast.cgi#190014632%23190014632) | 18.0 | 90% | 647 |
| [NP_060681.2](http://www.ncbi.nlm.nih.gov/entrez/query.fcgi?cmd=Retrieve&db=Protein&list_uids=51948366&dopt=GenPept&RID=T25GAK6Z016&log$=prottop&blast_rank=42) | ribonucleoprotein, PTB-binding 2 [Homo sapiens] | [18.0](http://blast.ncbi.nlm.nih.gov/Blast.cgi#51948366%2351948366) | 18.0 | 70% | 647 |
| [NP_001003674.1](http://www.ncbi.nlm.nih.gov/entrez/query.fcgi?cmd=Retrieve&db=Protein&list_uids=51093708&dopt=GenPept&RID=T25GAK6Z016&log$=prottop&blast_rank=43) | hypothetical protein LOC753 isoform gamma 1 [Homo sapiens] | [18.0](http://blast.ncbi.nlm.nih.gov/Blast.cgi#51093708%2351093708) | 18.0 | 40% | 647 |
| [NP_001003675.1](http://www.ncbi.nlm.nih.gov/entrez/query.fcgi?cmd=Retrieve&db=Protein&list_uids=51093710&dopt=GenPept&RID=T25GAK6Z016&log$=prottop&blast_rank=44) | hypothetical protein LOC753 isoform gamma 2 [Homo sapiens] | [18.0](http://blast.ncbi.nlm.nih.gov/Blast.cgi#51093710%2351093710) | 18.0 | 40% | 647 |
| [NP_076965.2](http://www.ncbi.nlm.nih.gov/entrez/query.fcgi?cmd=Retrieve&db=Protein&list_uids=55769541&dopt=GenPept&RID=T25GAK6Z016&log$=prottop&blast_rank=45) | AHNAK nucleoprotein isoform 2 [Homo sapiens] | [18.0](http://blast.ncbi.nlm.nih.gov/Blast.cgi#55769541%2355769541) | 18.0 | 40% | 647 |
| [NP_852148.1](http://www.ncbi.nlm.nih.gov/entrez/query.fcgi?cmd=Retrieve&db=Protein&list_uids=41281948&dopt=GenPept&RID=T25GAK6Z016&log$=prottop&blast_rank=46) | hypothetical protein LOC753 isoform beta 2 [Homo sapiens] | [18.0](http://blast.ncbi.nlm.nih.gov/Blast.cgi#41281948%2341281948) | 18.0 | 40% | 647 |
| [NP_852147.1](http://www.ncbi.nlm.nih.gov/entrez/query.fcgi?cmd=Retrieve&db=Protein&list_uids=41281957&dopt=GenPept&RID=T25GAK6Z016&log$=prottop&blast_rank=47) | hypothetical protein LOC753 isoform alpha 2 [Homo sapiens] | [18.0](http://blast.ncbi.nlm.nih.gov/Blast.cgi#41281957%2341281957) | 18.0 | 40% | 647 |
| [NP_004329.1](http://www.ncbi.nlm.nih.gov/entrez/query.fcgi?cmd=Retrieve&db=Protein&list_uids=4757884&dopt=GenPept&RID=T25GAK6Z016&log$=prottop&blast_rank=48) | hypothetical protein LOC753 isoform beta 1 [Homo sapiens] | [18.0](http://blast.ncbi.nlm.nih.gov/Blast.cgi#4757884%234757884) | 18.0 | 40% | 647 |
| [NP_056957.3](http://www.ncbi.nlm.nih.gov/entrez/query.fcgi?cmd=Retrieve&db=Protein&list_uids=94721268&dopt=GenPept&RID=T25GAK6Z016&log$=prottop&blast_rank=49) | villin-like protein [Homo sapiens] | [18.0](http://blast.ncbi.nlm.nih.gov/Blast.cgi#94721268%2394721268) | 18.0 | 60% | 647 |
| [NP_002143.1](http://www.ncbi.nlm.nih.gov/entrez/query.fcgi?cmd=Retrieve&db=Protein&list_uids=4504487&dopt=GenPept&RID=T25GAK6Z016&log$=prottop&blast_rank=50) | histidine rich calcium binding protein precursor [Homo sapiens] | [18.0](http://blast.ncbi.nlm.nih.gov/Blast.cgi#4504487%234504487) | 18.0 | 40% | 647 |
| [NP_005760.1](http://www.ncbi.nlm.nih.gov/entrez/query.fcgi?cmd=Retrieve&db=Protein&list_uids=5031735&dopt=GenPept&RID=T25GAK6Z016&log$=prottop&blast_rank=51) | carbohydrate (N-acetylglucosamine 6-O) sulfotransferase 4 [Homo sapiens] >ref|NP_001159867.1| carbohydrate (N-acetylglucosamine 6-O) sulfotransferase 4 [Homo sapiens] | [18.0](http://blast.ncbi.nlm.nih.gov/Blast.cgi#5031735%235031735) | 18.0 | 60% | 647 |
| [NP_852146.1](http://www.ncbi.nlm.nih.gov/entrez/query.fcgi?cmd=Retrieve&db=Protein&list_uids=41281943&dopt=GenPept&RID=T25GAK6Z016&log$=prottop&blast_rank=52) | hypothetical protein LOC753 isoform alpha 1 [Homo sapiens] | [18.0](http://blast.ncbi.nlm.nih.gov/Blast.cgi#41281943%2341281943) | 18.0 | 40% | 647 |
| [NP_000480.2](http://www.ncbi.nlm.nih.gov/entrez/query.fcgi?cmd=Retrieve&db=Protein&list_uids=20336209&dopt=GenPept&RID=T25GAK6Z016&log$=prottop&blast_rank=53) | transcriptional regulator ATRX isoform 1 [Homo sapiens] | [18.0](http://blast.ncbi.nlm.nih.gov/Blast.cgi#20336209%2320336209) | 18.0 | 80% | 647 |
| [NP_612114.1](http://www.ncbi.nlm.nih.gov/entrez/query.fcgi?cmd=Retrieve&db=Protein&list_uids=20336205&dopt=GenPept&RID=T25GAK6Z016&log$=prottop&blast_rank=54) | transcriptional regulator ATRX isoform 2 [Homo sapiens] | [18.0](http://blast.ncbi.nlm.nih.gov/Blast.cgi#20336205%2320336205) | 18.0 | 80% | 647 |
| [NP_071447.1](http://www.ncbi.nlm.nih.gov/entrez/query.fcgi?cmd=Retrieve&db=Protein&list_uids=11545918&dopt=GenPept&RID=T25GAK6Z016&log$=prottop&blast_rank=55) | tubulointerstitial nephritis antigen-like 1 [Homo sapiens] | [18.0](http://blast.ncbi.nlm.nih.gov/Blast.cgi#11545918%2311545918) | 18.0 | 40% | 647 |
| [NP_005080.1](http://www.ncbi.nlm.nih.gov/entrez/query.fcgi?cmd=Retrieve&db=Protein&list_uids=4827046&dopt=GenPept&RID=T25GAK6Z016&log$=prottop&blast_rank=56) | U2 small nuclear RNA auxiliary factor 1-like 2 [Homo sapiens] | [18.0](http://blast.ncbi.nlm.nih.gov/Blast.cgi#4827046%234827046) | 18.0 | 40% | 647 |
| [NP_067547.1](http://www.ncbi.nlm.nih.gov/entrez/query.fcgi?cmd=Retrieve&db=Protein&list_uids=11034849&dopt=GenPept&RID=T25GAK6Z016&log$=prottop&blast_rank=57) | ectonucleotide pyrophosphatase/phosphodiesterase 5 (putative function) precursor [Homo sapiens] | [18.0](http://blast.ncbi.nlm.nih.gov/Blast.cgi#11034849%2311034849) | 18.0 | 40% | 647 |
| [NP_005467.1](http://www.ncbi.nlm.nih.gov/entrez/query.fcgi?cmd=Retrieve&db=Protein&list_uids=4885285&dopt=GenPept&RID=T25GAK6Z016&log$=prottop&blast_rank=58) | UDP-N-acetylglucosamine-2-epimerase/N-acetylmannosamine kinase isoform 2 [Homo sapiens] | [18.0](http://blast.ncbi.nlm.nih.gov/Blast.cgi#4885285%234885285) | 18.0 | 90% | 647 |
| [NP_060540.2](http://www.ncbi.nlm.nih.gov/entrez/query.fcgi?cmd=Retrieve&db=Protein&list_uids=21314684&dopt=GenPept&RID=T25GAK6Z016&log$=prottop&blast_rank=59) | single stranded DNA binding protein 3 isoform b [Homo sapiens] | [17.6](http://blast.ncbi.nlm.nih.gov/Blast.cgi#21314684%2321314684) | 17.6 | 80% | 868 |
| [NP_001009955.1](http://www.ncbi.nlm.nih.gov/entrez/query.fcgi?cmd=Retrieve&db=Protein&list_uids=58218981&dopt=GenPept&RID=T25GAK6Z016&log$=prottop&blast_rank=60) | single stranded DNA binding protein 3 isoform c [Homo sapiens] | [17.6](http://blast.ncbi.nlm.nih.gov/Blast.cgi#58218981%2358218981) | 17.6 | 80% | 868 |
| [NP_000015.1](http://www.ncbi.nlm.nih.gov/entrez/query.fcgi?cmd=Retrieve&db=Protein&list_uids=4501969&dopt=GenPept&RID=T25GAK6Z016&log$=prottop&blast_rank=61) | adrenergic, beta-2-, receptor, surface [Homo sapiens] | [17.6](http://blast.ncbi.nlm.nih.gov/Blast.cgi#4501969%234501969) | 17.6 | 70% | 868 |
| [NP_663768.1](http://www.ncbi.nlm.nih.gov/entrez/query.fcgi?cmd=Retrieve&db=Protein&list_uids=21955170&dopt=GenPept&RID=T25GAK6Z016&log$=prottop&blast_rank=62) | single stranded DNA binding protein 3 isoform a [Homo sapiens] | [17.6](http://blast.ncbi.nlm.nih.gov/Blast.cgi#21955170%2321955170) | 17.6 | 80% | 868 |
| [NP_003835.3](http://www.ncbi.nlm.nih.gov/entrez/query.fcgi?cmd=Retrieve&db=Protein&list_uids=259906438&dopt=GenPept&RID=T25GAK6Z016&log$=prottop&blast_rank=63) | tumor necrosis factor receptor superfamily, member 10a precursor [Homo sapiens] | [17.2](http://blast.ncbi.nlm.nih.gov/Blast.cgi#259906438%23259906438) | 17.2 | 50% | 1165 |
| [NP_064450.3](http://www.ncbi.nlm.nih.gov/entrez/query.fcgi?cmd=Retrieve&db=Protein&list_uids=160707881&dopt=GenPept&RID=T25GAK6Z016&log$=prottop&blast_rank=64) | formin 2 [Homo sapiens] | [17.2](http://blast.ncbi.nlm.nih.gov/Blast.cgi#160707881%23160707881) | 17.2 | 100% | 1165 |
| [NP_056123.2](http://www.ncbi.nlm.nih.gov/entrez/query.fcgi?cmd=Retrieve&db=Protein&list_uids=158534059&dopt=GenPept&RID=T25GAK6Z016&log$=prottop&blast_rank=65) | formin binding protein 4 [Homo sapiens] | [17.2](http://blast.ncbi.nlm.nih.gov/Blast.cgi#158534059%23158534059) | 17.2 | 50% | 1165 |
| [NP_705835.2](http://www.ncbi.nlm.nih.gov/entrez/query.fcgi?cmd=Retrieve&db=Protein&list_uids=270309176&dopt=GenPept&RID=T25GAK6Z016&log$=prottop&blast_rank=66) | luman-recruiting factor isoform 1 [Homo sapiens] | [17.2](http://blast.ncbi.nlm.nih.gov/Blast.cgi#270309176%23270309176) | 17.2 | 60% | 1165 |
| [NP_004968.2](http://www.ncbi.nlm.nih.gov/entrez/query.fcgi?cmd=Retrieve&db=Protein&list_uids=24497460&dopt=GenPept&RID=T25GAK6Z016&log$=prottop&blast_rank=67) | Shaw-related voltage-gated potassium channel protein 3 [Homo sapiens] | [17.2](http://blast.ncbi.nlm.nih.gov/Blast.cgi#24497460%2324497460) | 17.2 | 70% | 1165 |
| [NP_001161865.1](http://www.ncbi.nlm.nih.gov/entrez/query.fcgi?cmd=Retrieve&db=Protein&list_uids=270309178&dopt=GenPept&RID=T25GAK6Z016&log$=prottop&blast_rank=68) | luman-recruiting factor isoform 2 [Homo sapiens] >ref|NP_001161866.1| luman-recruiting factor isoform 2 [Homo sapiens] | [17.2](http://blast.ncbi.nlm.nih.gov/Blast.cgi#270309178%23270309178) | 17.2 | 60% | 1165 |
| [NP_001165413.1](http://www.ncbi.nlm.nih.gov/entrez/query.fcgi?cmd=Retrieve&db=Protein&list_uids=284925161&dopt=GenPept&RID=T25GAK6Z016&log$=prottop&blast_rank=69) | glycine receptor, alpha 2 isoform C [Homo sapiens] | [16.8](http://blast.ncbi.nlm.nih.gov/Blast.cgi#284925161%23284925161) | 16.8 | 40% | 1564 |
| [NP_001164190.1](http://www.ncbi.nlm.nih.gov/entrez/query.fcgi?cmd=Retrieve&db=Protein&list_uids=282398123&dopt=GenPept&RID=T25GAK6Z016&log$=prottop&blast_rank=70) | breast cancer anti-estrogen resistance 1 isoform 7 [Homo sapiens] | [16.8](http://blast.ncbi.nlm.nih.gov/Blast.cgi#282398123%23282398123) | 16.8 | 40% | 1564 |
| [NP_001164189.1](http://www.ncbi.nlm.nih.gov/entrez/query.fcgi?cmd=Retrieve&db=Protein&list_uids=282398120&dopt=GenPept&RID=T25GAK6Z016&log$=prottop&blast_rank=71) | breast cancer anti-estrogen resistance 1 isoform 5 [Homo sapiens] | [16.8](http://blast.ncbi.nlm.nih.gov/Blast.cgi#282398120%23282398120) | 16.8 | 40% | 1564 |
| [NP_001164188.1](http://www.ncbi.nlm.nih.gov/entrez/query.fcgi?cmd=Retrieve&db=Protein&list_uids=282398118&dopt=GenPept&RID=T25GAK6Z016&log$=prottop&blast_rank=72) | breast cancer anti-estrogen resistance 1 isoform 4 [Homo sapiens] | [16.8](http://blast.ncbi.nlm.nih.gov/Blast.cgi#282398118%23282398118) | 16.8 | 40% | 1564 |
| [NP_001164186.1](http://www.ncbi.nlm.nih.gov/entrez/query.fcgi?cmd=Retrieve&db=Protein&list_uids=282398114&dopt=GenPept&RID=T25GAK6Z016&log$=prottop&blast_rank=73) | breast cancer anti-estrogen resistance 1 isoform 2 [Homo sapiens] | [16.8](http://blast.ncbi.nlm.nih.gov/Blast.cgi#282398114%23282398114) | 16.8 | 40% | 1564 |
| [NP_001164185.1](http://www.ncbi.nlm.nih.gov/entrez/query.fcgi?cmd=Retrieve&db=Protein&list_uids=282398112&dopt=GenPept&RID=T25GAK6Z016&log$=prottop&blast_rank=74) | breast cancer anti-estrogen resistance 1 isoform 1 [Homo sapiens] | [16.8](http://blast.ncbi.nlm.nih.gov/Blast.cgi#282398112%23282398112) | 16.8 | 40% | 1564 |
| [XP_001717873.2](http://www.ncbi.nlm.nih.gov/entrez/query.fcgi?cmd=Retrieve&db=Protein&list_uids=239757419&dopt=GenPept&RID=T25GAK6Z016&log$=prottop&blast_rank=75) | PREDICTED: hypothetical protein [Homo sapiens] | [16.8](http://blast.ncbi.nlm.nih.gov/Blast.cgi#239757419%23239757419) | 16.8 | 40% | 1564 |
| [XP_001717028.2](http://www.ncbi.nlm.nih.gov/entrez/query.fcgi?cmd=Retrieve&db=Protein&list_uids=239756681&dopt=GenPept&RID=T25GAK6Z016&log$=prottop&blast_rank=76) | PREDICTED: basic helix-loop-helix family, member a9 [Homo sapiens] | [16.8](http://blast.ncbi.nlm.nih.gov/Blast.cgi#239756681%23239756681) | 16.8 | 40% | 1564 |
| [XP_949003.3](http://www.ncbi.nlm.nih.gov/entrez/query.fcgi?cmd=Retrieve&db=Protein&list_uids=239751936&dopt=GenPept&RID=T25GAK6Z016&log$=prottop&blast_rank=77) | PREDICTED: hypothetical protein [Homo sapiens] | [16.8](http://blast.ncbi.nlm.nih.gov/Blast.cgi#239751936%23239751936) | 16.8 | 40% | 1564 |
| [XP_001717819.2](http://www.ncbi.nlm.nih.gov/entrez/query.fcgi?cmd=Retrieve&db=Protein&list_uids=239750776&dopt=GenPept&RID=T25GAK6Z016&log$=prottop&blast_rank=78) | PREDICTED: similar to breast cancer anti-estrogen resistance 1 [Homo sapiens] | [16.8](http://blast.ncbi.nlm.nih.gov/Blast.cgi#239750776%23239750776) | 16.8 | 40% | 1564 |
| [XP_001716809.2](http://www.ncbi.nlm.nih.gov/entrez/query.fcgi?cmd=Retrieve&db=Protein&list_uids=239745060&dopt=GenPept&RID=T25GAK6Z016&log$=prottop&blast_rank=79) | PREDICTED: similar to breast cancer anti-estrogen resistance 1 [Homo sapiens] | [16.8](http://blast.ncbi.nlm.nih.gov/Blast.cgi#239745060%23239745060) | 16.8 | 40% | 1564 |
| [XP_934132.2](http://www.ncbi.nlm.nih.gov/entrez/query.fcgi?cmd=Retrieve&db=Protein&list_uids=239745044&dopt=GenPept&RID=T25GAK6Z016&log$=prottop&blast_rank=80) | PREDICTED: similar to breast cancer anti-estrogen resistance 1 [Homo sapiens] | [16.8](http://blast.ncbi.nlm.nih.gov/Blast.cgi#239745044%23239745044) | 16.8 | 40% | 1564 |
| [XP_001716006.2](http://www.ncbi.nlm.nih.gov/entrez/query.fcgi?cmd=Retrieve&db=Protein&list_uids=239756225&dopt=GenPept&RID=T25GAK6Z016&log$=prottop&blast_rank=81) | PREDICTED: similar to breast cancer anti-estrogen resistance 1 [Homo sapiens] | [16.8](http://blast.ncbi.nlm.nih.gov/Blast.cgi#239756225%23239756225) | 16.8 | 40% | 1564 |
| [NP_001137246.1](http://www.ncbi.nlm.nih.gov/entrez/query.fcgi?cmd=Retrieve&db=Protein&list_uids=219555646&dopt=GenPept&RID=T25GAK6Z016&log$=prottop&blast_rank=82) | phytanoyl-CoA 2-hydroxylase interacting protein-like isoform 2 [Homo sapiens] | [16.8](http://blast.ncbi.nlm.nih.gov/Blast.cgi#219555646%23219555646) | 16.8 | 40% | 1564 |
| [NP_001159395.1](http://www.ncbi.nlm.nih.gov/entrez/query.fcgi?cmd=Retrieve&db=Protein&list_uids=260064065&dopt=GenPept&RID=T25GAK6Z016&log$=prottop&blast_rank=83) | hepatocyte nuclear factor 1-beta isoform 2 [Homo sapiens] | [16.8](http://blast.ncbi.nlm.nih.gov/Blast.cgi#260064065%23260064065) | 16.8 | 40% | 1564 |
| [NP_001164187.1](http://www.ncbi.nlm.nih.gov/entrez/query.fcgi?cmd=Retrieve&db=Protein&list_uids=282398116&dopt=GenPept&RID=T25GAK6Z016&log$=prottop&blast_rank=84) | breast cancer anti-estrogen resistance 1 isoform 3 [Homo sapiens] | [16.8](http://blast.ncbi.nlm.nih.gov/Blast.cgi#282398116%23282398116) | 16.8 | 40% | 1564 |
| [NP_001157877.1](http://www.ncbi.nlm.nih.gov/entrez/query.fcgi?cmd=Retrieve&db=Protein&list_uids=256542291&dopt=GenPept&RID=T25GAK6Z016&log$=prottop&blast_rank=85) | basic helix-loop-helix family, member a9 [Homo sapiens] >ref|XP_001126841.3| PREDICTED: basic helix-loop-helix family, member a9 [Homo sapiens] | [16.8](http://blast.ncbi.nlm.nih.gov/Blast.cgi#256542291%23256542291) | 16.8 | 40% | 1564 |
| [NP_115815.2](http://www.ncbi.nlm.nih.gov/entrez/query.fcgi?cmd=Retrieve&db=Protein&list_uids=190341060&dopt=GenPept&RID=T25GAK6Z016&log$=prottop&blast_rank=86) | phytanoyl-CoA 2-hydroxylase interacting protein-like isoform 1 [Homo sapiens] | [16.8](http://blast.ncbi.nlm.nih.gov/Blast.cgi#190341060%23190341060) | 16.8 | 40% | 1564 |
| [NP_001073988.2](http://www.ncbi.nlm.nih.gov/entrez/query.fcgi?cmd=Retrieve&db=Protein&list_uids=169658367&dopt=GenPept&RID=T25GAK6Z016&log$=prottop&blast_rank=87) | BAH domain and coiled-coil containing 1 [Homo sapiens] | [16.8](http://blast.ncbi.nlm.nih.gov/Blast.cgi#169658367%23169658367) | 16.8 | 40% | 1564 |
| [XP_001722349.1](http://www.ncbi.nlm.nih.gov/entrez/query.fcgi?cmd=Retrieve&db=Protein&list_uids=169160876&dopt=GenPept&RID=T25GAK6Z016&log$=prottop&blast_rank=88) | PREDICTED: similar to hCG1648021 [Homo sapiens] | [16.8](http://blast.ncbi.nlm.nih.gov/Blast.cgi#169160876%23169160876) | 16.8 | 40% | 1564 |
| [NP_694942.3](http://www.ncbi.nlm.nih.gov/entrez/query.fcgi?cmd=Retrieve&db=Protein&list_uids=148612811&dopt=GenPept&RID=T25GAK6Z016&log$=prottop&blast_rank=89) | ubiquitin specific protease 43 [Homo sapiens] | [16.8](http://blast.ncbi.nlm.nih.gov/Blast.cgi#148612811%23148612811) | 28.0 | 60% | 1564 |
| [NP_891989.2](http://www.ncbi.nlm.nih.gov/entrez/query.fcgi?cmd=Retrieve&db=Protein&list_uids=148536871&dopt=GenPept&RID=T25GAK6Z016&log$=prottop&blast_rank=90) | ninein isoform 1 [Homo sapiens] | [16.8](http://blast.ncbi.nlm.nih.gov/Blast.cgi#148536871%23148536871) | 16.8 | 40% | 1564 |
| [NP_065972.3](http://www.ncbi.nlm.nih.gov/entrez/query.fcgi?cmd=Retrieve&db=Protein&list_uids=148536869&dopt=GenPept&RID=T25GAK6Z016&log$=prottop&blast_rank=91) | ninein isoform 2 [Homo sapiens] | [16.8](http://blast.ncbi.nlm.nih.gov/Blast.cgi#148536869%23148536869) | 16.8 | 40% | 1564 |
| [NP_002323.2](http://www.ncbi.nlm.nih.gov/entrez/query.fcgi?cmd=Retrieve&db=Protein&list_uids=126012562&dopt=GenPept&RID=T25GAK6Z016&log$=prottop&blast_rank=92) | low density lipoprotein receptor-related protein 1 precursor [Homo sapiens] | [16.8](http://blast.ncbi.nlm.nih.gov/Blast.cgi#126012562%23126012562) | 41.4 | 70% | 1564 |
| [NP_001137435.1](http://www.ncbi.nlm.nih.gov/entrez/query.fcgi?cmd=Retrieve&db=Protein&list_uids=221316760&dopt=GenPept&RID=T25GAK6Z016&log$=prottop&blast_rank=93) | L1 cell adhesion molecule isoform 3 precursor [Homo sapiens] | [16.8](http://blast.ncbi.nlm.nih.gov/Blast.cgi#221316760%23221316760) | 16.8 | 60% | 1564 |
| [XP_002342100.1](http://www.ncbi.nlm.nih.gov/entrez/query.fcgi?cmd=Retrieve&db=Protein&list_uids=239741224&dopt=GenPept&RID=T25GAK6Z016&log$=prottop&blast_rank=94) | PREDICTED: similar to hCG1648021 [Homo sapiens] >ref|XP_002346642.1| PREDICTED: similar to hCG1648021 [Homo sapiens] | [16.8](http://blast.ncbi.nlm.nih.gov/Blast.cgi#239741224%23239741224) | 16.8 | 40% | 1564 |
| [NP_005985.3](http://www.ncbi.nlm.nih.gov/entrez/query.fcgi?cmd=Retrieve&db=Protein&list_uids=197333868&dopt=GenPept&RID=T25GAK6Z016&log$=prottop&blast_rank=95) | T-box 2 [Homo sapiens] | [16.8](http://blast.ncbi.nlm.nih.gov/Blast.cgi#197333868%23197333868) | 16.8 | 40% | 1564 |
| [NP_001262.3](http://www.ncbi.nlm.nih.gov/entrez/query.fcgi?cmd=Retrieve&db=Protein&list_uids=118421089&dopt=GenPept&RID=T25GAK6Z016&log$=prottop&blast_rank=96) | chromodomain helicase DNA binding protein 2 isoform 1 [Homo sapiens] | [16.8](http://blast.ncbi.nlm.nih.gov/Blast.cgi#118421089%23118421089) | 16.8 | 70% | 1564 |
| [NP_001009899.2](http://www.ncbi.nlm.nih.gov/entrez/query.fcgi?cmd=Retrieve&db=Protein&list_uids=114431248&dopt=GenPept&RID=T25GAK6Z016&log$=prottop&blast_rank=97) | hypothetical protein LOC205717 [Homo sapiens] | [16.8](http://blast.ncbi.nlm.nih.gov/Blast.cgi#114431248%23114431248) | 32.2 | 50% | 1564 |
| [NP_001011649.1](http://www.ncbi.nlm.nih.gov/entrez/query.fcgi?cmd=Retrieve&db=Protein&list_uids=58535453&dopt=GenPept&RID=T25GAK6Z016&log$=prottop&blast_rank=98) | CDK5 regulatory subunit associated protein 2 isoform b [Homo sapiens] | [16.8](http://blast.ncbi.nlm.nih.gov/Blast.cgi#58535453%2358535453) | 16.8 | 70% | 1564 |
| [NP_056290.3](http://www.ncbi.nlm.nih.gov/entrez/query.fcgi?cmd=Retrieve&db=Protein&list_uids=56699482&dopt=GenPept&RID=T25GAK6Z016&log$=prottop&blast_rank=99) | hypothetical protein LOC25940 [Homo sapiens] | [16.8](http://blast.ncbi.nlm.nih.gov/Blast.cgi#56699482%2356699482) | 30.5 | 90% | 1564 |
| [NP_001003694.1](http://www.ncbi.nlm.nih.gov/entrez/query.fcgi?cmd=Retrieve&db=Protein&list_uids=51173720&dopt=GenPept&RID=T25GAK6Z016&log$=prottop&blast_rank=100) | bromodomain and PHD finger-containing protein 1 isoform 1 [Homo sapiens] | [16.8](http://blast.ncbi.nlm.nih.gov/Blast.cgi#51173720%2351173720) | 16.8 | 60% | 1564 |

| **Accession** | **Proteins with a match to KAYGHHLSAE peptide** | **[Max score](http://blast.ncbi.nlm.nih.gov/Blast.cgi?CMD=Get&ALIGNMENTS=100&ALIGNMENT_VIEW=Pairwise&CDD_SEARCH_STATE=1&DATABASE_SORT=0&DESCRIPTIONS=100&ENTREZ_QUERY=txid9606 %5BORGN%5D&FIRST_QUERY_NUM=0&FORMAT_OBJECT=Alignment&FORMAT_PAGE_TARGET=&FORMAT_TYPE=HTML&GET_SEQUENCE=yes&I_THRESH=&MASK_CHAR=2&MASK_COLOR=1&NEW_DESIGN=on&NEW_VIEW=yes&NUM_OVERVIEW=100&OLD_BLAST=false&PAGE=Proteins&QUERY_INDEX=0&QUERY_NUMBER=0&RESULTS_PAGE_TARGET=&RID=T26J5VN701S&SHOW_LINKOUT=yes&SHOW_OVERVIEW=yes&STEP_NUMBER=&WORD_SIZE=2&DISPLAY_SORT=1&HSP_SORT=1" \l "sort_mark)** | **[Total score](http://blast.ncbi.nlm.nih.gov/Blast.cgi?CMD=Get&ALIGNMENTS=100&ALIGNMENT_VIEW=Pairwise&CDD_SEARCH_STATE=1&DATABASE_SORT=0&DESCRIPTIONS=100&ENTREZ_QUERY=txid9606 %5BORGN%5D&FIRST_QUERY_NUM=0&FORMAT_OBJECT=Alignment&FORMAT_PAGE_TARGET=&FORMAT_TYPE=HTML&GET_SEQUENCE=yes&I_THRESH=&MASK_CHAR=2&MASK_COLOR=1&NEW_DESIGN=on&NEW_VIEW=yes&NUM_OVERVIEW=100&OLD_BLAST=false&PAGE=Proteins&QUERY_INDEX=0&QUERY_NUMBER=0&RESULTS_PAGE_TARGET=&RID=T26J5VN701S&SHOW_LINKOUT=yes&SHOW_OVERVIEW=yes&STEP_NUMBER=&WORD_SIZE=2&DISPLAY_SORT=2&HSP_SORT=1" \l "sort_mark)** | **[Query coverage](http://blast.ncbi.nlm.nih.gov/Blast.cgi?CMD=Get&ALIGNMENTS=100&ALIGNMENT_VIEW=Pairwise&CDD_SEARCH_STATE=1&DATABASE_SORT=0&DESCRIPTIONS=100&ENTREZ_QUERY=txid9606 %5BORGN%5D&FIRST_QUERY_NUM=0&FORMAT_OBJECT=Alignment&FORMAT_PAGE_TARGET=&FORMAT_TYPE=HTML&GET_SEQUENCE=yes&I_THRESH=&MASK_CHAR=2&MASK_COLOR=1&NEW_DESIGN=on&NEW_VIEW=yes&NUM_OVERVIEW=100&OLD_BLAST=false&PAGE=Proteins&QUERY_INDEX=0&QUERY_NUMBER=0&RESULTS_PAGE_TARGET=&RID=T26J5VN701S&SHOW_LINKOUT=yes&SHOW_OVERVIEW=yes&STEP_NUMBER=&WORD_SIZE=2&DISPLAY_SORT=4&HSP_SORT=0" \l "sort_mark)** | **[E value](http://blast.ncbi.nlm.nih.gov/Blast.cgi?CMD=Get&ALIGNMENTS=100&ALIGNMENT_VIEW=Pairwise&CDD_SEARCH_STATE=1&DATABASE_SORT=0&DESCRIPTIONS=100&ENTREZ_QUERY=txid9606 %5BORGN%5D&FIRST_QUERY_NUM=0&FORMAT_OBJECT=Alignment&FORMAT_PAGE_TARGET=&FORMAT_TYPE=HTML&GET_SEQUENCE=yes&I_THRESH=&MASK_CHAR=2&MASK_COLOR=1&NEW_DESIGN=on&NEW_VIEW=yes&NUM_OVERVIEW=100&OLD_BLAST=false&PAGE=Proteins&QUERY_INDEX=0&QUERY_NUMBER=0&RESULTS_PAGE_TARGET=&RID=T26J5VN701S&SHOW_LINKOUT=yes&SHOW_OVERVIEW=yes&STEP_NUMBER=&WORD_SIZE=2&DISPLAY_SORT=0&HSP_SORT=0" \l "sort_mark)** |
| --- | --- | --- | --- | --- | --- |
| [NP_003307.3](http://www.ncbi.nlm.nih.gov/entrez/query.fcgi?cmd=Retrieve&db=Protein&list_uids=49640009&dopt=GenPept&RID=T26J5VN701S&log$=prottop&blast_rank=1) | tetratricopeptide repeat domain 3 [Homo sapiens] >ref|NP_001001894.1| tetratricopeptide repeat domain 3 [Homo sapiens] | [24.4](http://blast.ncbi.nlm.nih.gov/Blast.cgi#49640009%2349640009) | 24.4 | 90% | 7.9 |
| [NP_001106946.1](http://www.ncbi.nlm.nih.gov/entrez/query.fcgi?cmd=Retrieve&db=Protein&list_uids=164698426&dopt=GenPept&RID=T26J5VN701S&log$=prottop&blast_rank=2) | hypothetical protein LOC122945 isoform 1 [Homo sapiens] | [20.2](http://blast.ncbi.nlm.nih.gov/Blast.cgi#164698426%23164698426) | 20.2 | 100% | 149 |
| [NP_078995.2](http://www.ncbi.nlm.nih.gov/entrez/query.fcgi?cmd=Retrieve&db=Protein&list_uids=118722351&dopt=GenPept&RID=T26J5VN701S&log$=prottop&blast_rank=3) | growth hormone regulated TBC protein 1 [Homo sapiens] | [20.2](http://blast.ncbi.nlm.nih.gov/Blast.cgi#118722351%23118722351) | 20.2 | 50% | 149 |
| [NP_055816.2](http://www.ncbi.nlm.nih.gov/entrez/query.fcgi?cmd=Retrieve&db=Protein&list_uids=14790190&dopt=GenPept&RID=T26J5VN701S&log$=prottop&blast_rank=4) | spen homolog, transcriptional regulator [Homo sapiens] | [20.2](http://blast.ncbi.nlm.nih.gov/Blast.cgi#14790190%2314790190) | 20.2 | 50% | 149 |
| [NP_919277.2](http://www.ncbi.nlm.nih.gov/entrez/query.fcgi?cmd=Retrieve&db=Protein&list_uids=51230412&dopt=GenPept&RID=T26J5VN701S&log$=prottop&blast_rank=5) | Ral GTPase activating protein, alpha subunit 1 (catalytic) isoform 2 [Homo sapiens] | [19.7](http://blast.ncbi.nlm.nih.gov/Blast.cgi#51230412%2351230412) | 19.7 | 70% | 200 |
| [NP_055805.1](http://www.ncbi.nlm.nih.gov/entrez/query.fcgi?cmd=Retrieve&db=Protein&list_uids=51226124&dopt=GenPept&RID=T26J5VN701S&log$=prottop&blast_rank=6) | Ral GTPase activating protein, alpha subunit 1 (catalytic) isoform 1 [Homo sapiens] | [19.7](http://blast.ncbi.nlm.nih.gov/Blast.cgi#51226124%2351226124) | 19.7 | 70% | 200 |
| [NP_065908.1](http://www.ncbi.nlm.nih.gov/entrez/query.fcgi?cmd=Retrieve&db=Protein&list_uids=17978485&dopt=GenPept&RID=T26J5VN701S&log$=prottop&blast_rank=7) | vacuolar protein sorting 18 [Homo sapiens] | [19.3](http://blast.ncbi.nlm.nih.gov/Blast.cgi#17978485%2317978485) | 19.3 | 60% | 268 |
| [NP_001290.2](http://www.ncbi.nlm.nih.gov/entrez/query.fcgi?cmd=Retrieve&db=Protein&list_uids=21361120&dopt=GenPept&RID=T26J5VN701S&log$=prottop&blast_rank=8) | calponin 1, basic, smooth muscle [Homo sapiens] | [19.3](http://blast.ncbi.nlm.nih.gov/Blast.cgi#21361120%2321361120) | 19.3 | 90% | 268 |
| [NP_065855.1](http://www.ncbi.nlm.nih.gov/entrez/query.fcgi?cmd=Retrieve&db=Protein&list_uids=24308229&dopt=GenPept&RID=T26J5VN701S&log$=prottop&blast_rank=9) | protein kinase C and casein kinase substrate in neurons 1 [Homo sapiens] | [19.3](http://blast.ncbi.nlm.nih.gov/Blast.cgi#24308229%2324308229) | 19.3 | 70% | 268 |
| [NP_005559.2](http://www.ncbi.nlm.nih.gov/entrez/query.fcgi?cmd=Retrieve&db=Protein&list_uids=30410785&dopt=GenPept&RID=T26J5VN701S&log$=prottop&blast_rank=10) | LIM homeobox protein 1 [Homo sapiens] | [19.3](http://blast.ncbi.nlm.nih.gov/Blast.cgi#30410785%2330410785) | 19.3 | 60% | 268 |
| [NP_115652.2](http://www.ncbi.nlm.nih.gov/entrez/query.fcgi?cmd=Retrieve&db=Protein&list_uids=33300639&dopt=GenPept&RID=T26J5VN701S&log$=prottop&blast_rank=11) | rhomboid domain containing 1 [Homo sapiens] >ref|NP_001161080.1| rhomboid domain containing 1 [Homo sapiens] | [19.3](http://blast.ncbi.nlm.nih.gov/Blast.cgi#33300639%2333300639) | 19.3 | 80% | 268 |
| [XP_002343107.1](http://www.ncbi.nlm.nih.gov/entrez/query.fcgi?cmd=Retrieve&db=Protein&list_uids=239744265&dopt=GenPept&RID=T26J5VN701S&log$=prottop&blast_rank=12) | PREDICTED: hypothetical protein XP_002343107 [Homo sapiens] | [18.9](http://blast.ncbi.nlm.nih.gov/Blast.cgi#239744265%23239744265) | 18.9 | 50% | 359 |
| [NP_009231.2](http://www.ncbi.nlm.nih.gov/entrez/query.fcgi?cmd=Retrieve&db=Protein&list_uids=237681119&dopt=GenPept&RID=T26J5VN701S&log$=prottop&blast_rank=13) | breast cancer 1, early onset isoform 2 [Homo sapiens] | [18.9](http://blast.ncbi.nlm.nih.gov/Blast.cgi#237681119%23237681119) | 18.9 | 60% | 359 |
| [NP_009228.2](http://www.ncbi.nlm.nih.gov/entrez/query.fcgi?cmd=Retrieve&db=Protein&list_uids=237681121&dopt=GenPept&RID=T26J5VN701S&log$=prottop&blast_rank=14) | breast cancer 1, early onset isoform 3 [Homo sapiens] | [18.9](http://blast.ncbi.nlm.nih.gov/Blast.cgi#237681121%23237681121) | 18.9 | 60% | 359 |
| [XP_001715398.1](http://www.ncbi.nlm.nih.gov/entrez/query.fcgi?cmd=Retrieve&db=Protein&list_uids=169167512&dopt=GenPept&RID=T26J5VN701S&log$=prottop&blast_rank=15) | PREDICTED: hypothetical protein [Homo sapiens] | [18.9](http://blast.ncbi.nlm.nih.gov/Blast.cgi#169167512%23169167512) | 28.8 | 50% | 359 |
| [NP_742066.2](http://www.ncbi.nlm.nih.gov/entrez/query.fcgi?cmd=Retrieve&db=Protein&list_uids=217416392&dopt=GenPept&RID=T26J5VN701S&log$=prottop&blast_rank=16) | pleckstrin homology domain containing, family H (with MyTH4 domain) member 2 [Homo sapiens] | [18.9](http://blast.ncbi.nlm.nih.gov/Blast.cgi#217416392%23217416392) | 18.9 | 50% | 359 |
| [NP_001091971.1](http://www.ncbi.nlm.nih.gov/entrez/query.fcgi?cmd=Retrieve&db=Protein&list_uids=148612857&dopt=GenPept&RID=T26J5VN701S&log$=prottop&blast_rank=17) | sickle tail isoform 3 [Homo sapiens] | [18.9](http://blast.ncbi.nlm.nih.gov/Blast.cgi#148612857%23148612857) | 33.9 | 70% | 359 |
| [NP_060229.3](http://www.ncbi.nlm.nih.gov/entrez/query.fcgi?cmd=Retrieve&db=Protein&list_uids=148539591&dopt=GenPept&RID=T26J5VN701S&log$=prottop&blast_rank=18) | INO80 complex subunit D [Homo sapiens] | [18.9](http://blast.ncbi.nlm.nih.gov/Blast.cgi#148539591%23148539591) | 31.4 | 70% | 359 |
| [NP_001091970.1](http://www.ncbi.nlm.nih.gov/entrez/query.fcgi?cmd=Retrieve&db=Protein&list_uids=148612813&dopt=GenPept&RID=T26J5VN701S&log$=prottop&blast_rank=19) | sickle tail isoform 2 [Homo sapiens] | [18.9](http://blast.ncbi.nlm.nih.gov/Blast.cgi#148612813%23148612813) | 33.9 | 70% | 359 |
| [NP_001005731.1](http://www.ncbi.nlm.nih.gov/entrez/query.fcgi?cmd=Retrieve&db=Protein&list_uids=54607033&dopt=GenPept&RID=T26J5VN701S&log$=prottop&blast_rank=20) | integrin beta 4 isoform 3 precursor [Homo sapiens] | [18.9](http://blast.ncbi.nlm.nih.gov/Blast.cgi#54607033%2354607033) | 18.9 | 70% | 359 |
| [NP_000204.3](http://www.ncbi.nlm.nih.gov/entrez/query.fcgi?cmd=Retrieve&db=Protein&list_uids=54607035&dopt=GenPept&RID=T26J5VN701S&log$=prottop&blast_rank=21) | integrin beta 4 isoform 1 precursor [Homo sapiens] | [18.9](http://blast.ncbi.nlm.nih.gov/Blast.cgi#54607035%2354607035) | 18.9 | 70% | 359 |
| [NP_001005619.1](http://www.ncbi.nlm.nih.gov/entrez/query.fcgi?cmd=Retrieve&db=Protein&list_uids=54607027&dopt=GenPept&RID=T26J5VN701S&log$=prottop&blast_rank=22) | integrin beta 4 isoform 2 precursor [Homo sapiens] | [18.9](http://blast.ncbi.nlm.nih.gov/Blast.cgi#54607027%2354607027) | 18.9 | 70% | 359 |
| [NP_062536.2](http://www.ncbi.nlm.nih.gov/entrez/query.fcgi?cmd=Retrieve&db=Protein&list_uids=50843820&dopt=GenPept&RID=T26J5VN701S&log$=prottop&blast_rank=23) | sickle tail isoform 1 [Homo sapiens] | [18.9](http://blast.ncbi.nlm.nih.gov/Blast.cgi#50843820%2350843820) | 33.9 | 70% | 359 |
| [NP_005011.1](http://www.ncbi.nlm.nih.gov/entrez/query.fcgi?cmd=Retrieve&db=Protein&list_uids=4826894&dopt=GenPept&RID=T26J5VN701S&log$=prottop&blast_rank=24) | phosphodiesterase 1C [Homo sapiens] | [18.9](http://blast.ncbi.nlm.nih.gov/Blast.cgi#4826894%234826894) | 18.9 | 50% | 359 |
| [NP_065826.2](http://www.ncbi.nlm.nih.gov/entrez/query.fcgi?cmd=Retrieve&db=Protein&list_uids=38569482&dopt=GenPept&RID=T26J5VN701S&log$=prottop&blast_rank=25) | hypothetical protein LOC57535 precursor [Homo sapiens] | [18.9](http://blast.ncbi.nlm.nih.gov/Blast.cgi#38569482%2338569482) | 18.9 | 50% | 359 |
| [NP_009225.1](http://www.ncbi.nlm.nih.gov/entrez/query.fcgi?cmd=Retrieve&db=Protein&list_uids=6552299&dopt=GenPept&RID=T26J5VN701S&log$=prottop&blast_rank=26) | breast cancer 1, early onset isoform 1 [Homo sapiens] | [18.9](http://blast.ncbi.nlm.nih.gov/Blast.cgi#6552299%236552299) | 18.9 | 60% | 359 |
| [NP_659471.1](http://www.ncbi.nlm.nih.gov/entrez/query.fcgi?cmd=Retrieve&db=Protein&list_uids=21450775&dopt=GenPept&RID=T26J5VN701S&log$=prottop&blast_rank=27) | torsin A interacting protein 2 [Homo sapiens] | [18.9](http://blast.ncbi.nlm.nih.gov/Blast.cgi#21450775%2321450775) | 18.9 | 70% | 359 |
| [NP_115674.1](http://www.ncbi.nlm.nih.gov/entrez/query.fcgi?cmd=Retrieve&db=Protein&list_uids=14150054&dopt=GenPept&RID=T26J5VN701S&log$=prottop&blast_rank=28) | synaptotagmin III [Homo sapiens] >ref|NP_001153800.1| synaptotagmin III [Homo sapiens] >ref|NP_001153801.1| synaptotagmin III [Homo sapiens] | [18.9](http://blast.ncbi.nlm.nih.gov/Blast.cgi#14150054%2314150054) | 18.9 | 60% | 359 |
| [NP_009032.2](http://www.ncbi.nlm.nih.gov/entrez/query.fcgi?cmd=Retrieve&db=Protein&list_uids=21361378&dopt=GenPept&RID=T26J5VN701S&log$=prottop&blast_rank=29) | sarcosine dehydrogenase precursor [Homo sapiens] >ref|NP_001128179.1| sarcosine dehydrogenase precursor [Homo sapiens] | [18.9](http://blast.ncbi.nlm.nih.gov/Blast.cgi#21361378%2321361378) | 18.9 | 100% | 359 |
| [NP_036393.1](http://www.ncbi.nlm.nih.gov/entrez/query.fcgi?cmd=Retrieve&db=Protein&list_uids=7110633&dopt=GenPept&RID=T26J5VN701S&log$=prottop&blast_rank=30) | chromosome 20 open reading frame 103 precursor [Homo sapiens] | [18.9](http://blast.ncbi.nlm.nih.gov/Blast.cgi#7110633%237110633) | 18.9 | 50% | 359 |
| [NP_056243.1](http://www.ncbi.nlm.nih.gov/entrez/query.fcgi?cmd=Retrieve&db=Protein&list_uids=54792148&dopt=GenPept&RID=T26J5VN701S&log$=prottop&blast_rank=31) | zinc finger protein 473 [Homo sapiens] >ref|NP_001006657.1| zinc finger protein 473 [Homo sapiens] | [18.9](http://blast.ncbi.nlm.nih.gov/Blast.cgi#54792148%2354792148) | 18.9 | 100% | 359 |
| [NP_658988.2](http://www.ncbi.nlm.nih.gov/entrez/query.fcgi?cmd=Retrieve&db=Protein&list_uids=146260268&dopt=GenPept&RID=T26J5VN701S&log$=prottop&blast_rank=32) | Smith-Magenis syndrome chromosome region, candidate 8 [Homo sapiens] | [18.5](http://blast.ncbi.nlm.nih.gov/Blast.cgi#146260268%23146260268) | 33.1 | 90% | 482 |
| [NP_001078956.1](http://www.ncbi.nlm.nih.gov/entrez/query.fcgi?cmd=Retrieve&db=Protein&list_uids=148227842&dopt=GenPept&RID=T26J5VN701S&log$=prottop&blast_rank=33) | Myb-like, SWIRM and MPN domains 1 [Homo sapiens] | [18.5](http://blast.ncbi.nlm.nih.gov/Blast.cgi#148227842%23148227842) | 18.5 | 50% | 482 |
| [NP_065902.1](http://www.ncbi.nlm.nih.gov/entrez/query.fcgi?cmd=Retrieve&db=Protein&list_uids=39930401&dopt=GenPept&RID=T26J5VN701S&log$=prottop&blast_rank=34) | immunoglobulin superfamily containing leucine-rich repeat 2 precursor [Homo sapiens] >ref|NP_001123608.1| immunoglobulin superfamily containing leucine-rich repeat 2 precursor [Homo sapiens] >ref|NP_001123609.1| immunoglobulin superfamily containing leucine-rich repeat 2 precursor [Homo sapiens] >ref|NP_001123610.1| immunoglobulin superfamily containing leucine-rich repeat 2 precursor [Homo sapiens] | [18.5](http://blast.ncbi.nlm.nih.gov/Blast.cgi#39930401%2339930401) | 18.5 | 50% | 482 |
| [NP_001136118.1](http://www.ncbi.nlm.nih.gov/entrez/query.fcgi?cmd=Retrieve&db=Protein&list_uids=217416360&dopt=GenPept&RID=T26J5VN701S&log$=prottop&blast_rank=35) | G protein-coupled receptor 175 isoform 2 [Homo sapiens] | [18.5](http://blast.ncbi.nlm.nih.gov/Blast.cgi#217416360%23217416360) | 18.5 | 50% | 482 |
| [NP_056014.2](http://www.ncbi.nlm.nih.gov/entrez/query.fcgi?cmd=Retrieve&db=Protein&list_uids=68131557&dopt=GenPept&RID=T26J5VN701S&log$=prottop&blast_rank=36) | ankyrin repeat domain 28 [Homo sapiens] | [18.5](http://blast.ncbi.nlm.nih.gov/Blast.cgi#68131557%2368131557) | 33.5 | 80% | 482 |
| [NP_001107609.1](http://www.ncbi.nlm.nih.gov/entrez/query.fcgi?cmd=Retrieve&db=Protein&list_uids=166706881&dopt=GenPept&RID=T26J5VN701S&log$=prottop&blast_rank=37) | erythrocyte membrane protein band 4.9 isoform 2 [Homo sapiens] >ref|NP_001107610.1| erythrocyte membrane protein band 4.9 isoform 2 [Homo sapiens] | [18.5](http://blast.ncbi.nlm.nih.gov/Blast.cgi#166706881%23166706881) | 18.5 | 50% | 482 |
| [NP_005325.2](http://www.ncbi.nlm.nih.gov/entrez/query.fcgi?cmd=Retrieve&db=Protein&list_uids=98986457&dopt=GenPept&RID=T26J5VN701S&log$=prottop&blast_rank=38) | host cell factor 1 [Homo sapiens] | [18.5](http://blast.ncbi.nlm.nih.gov/Blast.cgi#98986457%2398986457) | 18.5 | 50% | 482 |
| [NP_001107611.1](http://www.ncbi.nlm.nih.gov/entrez/query.fcgi?cmd=Retrieve&db=Protein&list_uids=166706885&dopt=GenPept&RID=T26J5VN701S&log$=prottop&blast_rank=39) | erythrocyte membrane protein band 4.9 isoform 3 [Homo sapiens] | [18.5](http://blast.ncbi.nlm.nih.gov/Blast.cgi#166706885%23166706885) | 18.5 | 50% | 482 |
| [NP_061940.1](http://www.ncbi.nlm.nih.gov/entrez/query.fcgi?cmd=Retrieve&db=Protein&list_uids=9506611&dopt=GenPept&RID=T26J5VN701S&log$=prottop&blast_rank=40) | guanine nucleotide binding protein-like 3 (nucleolar)-like [Homo sapiens] | [18.5](http://blast.ncbi.nlm.nih.gov/Blast.cgi#9506611%239506611) | 18.5 | 50% | 482 |
| [NP_001969.2](http://www.ncbi.nlm.nih.gov/entrez/query.fcgi?cmd=Retrieve&db=Protein&list_uids=166706875&dopt=GenPept&RID=T26J5VN701S&log$=prottop&blast_rank=41) | erythrocyte membrane protein band 4.9 isoform 1 [Homo sapiens] >ref|NP_001107607.1| erythrocyte membrane protein band 4.9 isoform 1 [Homo sapiens] >ref|NP_001107608.1| erythrocyte membrane protein band 4.9 isoform 1 [Homo sapiens] | [18.5](http://blast.ncbi.nlm.nih.gov/Blast.cgi#166706875%23166706875) | 18.5 | 50% | 482 |
| [NP_000272.1](http://www.ncbi.nlm.nih.gov/entrez/query.fcgi?cmd=Retrieve&db=Protein&list_uids=4557831&dopt=GenPept&RID=T26J5VN701S&log$=prottop&blast_rank=42) | pterin-4 alpha-carbinolamine dehydratase/dimerization cofactor of hepatocyte nuclear factor 1 alpha precursor [Homo sapiens] | [18.5](http://blast.ncbi.nlm.nih.gov/Blast.cgi#4557831%234557831) | 18.5 | 100% | 482 |
| [NP_001129525.1](http://www.ncbi.nlm.nih.gov/entrez/query.fcgi?cmd=Retrieve&db=Protein&list_uids=209862909&dopt=GenPept&RID=T26J5VN701S&log$=prottop&blast_rank=43) | G protein-coupled receptor 175 isoform 1 [Homo sapiens] >ref|NP_057456.2| G protein-coupled receptor 175 isoform 1 [Homo sapiens] | [18.5](http://blast.ncbi.nlm.nih.gov/Blast.cgi#209862909%23209862909) | 18.5 | 50% | 482 |
| [XP_001133863.2](http://www.ncbi.nlm.nih.gov/entrez/query.fcgi?cmd=Retrieve&db=Protein&list_uids=239740972&dopt=GenPept&RID=T26J5VN701S&log$=prottop&blast_rank=44) | PREDICTED: hypothetical protein [Homo sapiens] | [18.0](http://blast.ncbi.nlm.nih.gov/Blast.cgi#239740972%23239740972) | 18.0 | 70% | 647 |
| [NP_006549.1](http://www.ncbi.nlm.nih.gov/entrez/query.fcgi?cmd=Retrieve&db=Protein&list_uids=5730073&dopt=GenPept&RID=T26J5VN701S&log$=prottop&blast_rank=45) | KH domain containing, RNA binding, signal transduction associated 3 [Homo sapiens] | [18.0](http://blast.ncbi.nlm.nih.gov/Blast.cgi#5730073%235730073) | 18.0 | 80% | 647 |
| [NP_085131.1](http://www.ncbi.nlm.nih.gov/entrez/query.fcgi?cmd=Retrieve&db=Protein&list_uids=44771201&dopt=GenPept&RID=T26J5VN701S&log$=prottop&blast_rank=46) | integrator complex subunit 5 [Homo sapiens] | [18.0](http://blast.ncbi.nlm.nih.gov/Blast.cgi#44771201%2344771201) | 30.5 | 60% | 647 |
| [NP_955805.1](http://www.ncbi.nlm.nih.gov/entrez/query.fcgi?cmd=Retrieve&db=Protein&list_uids=41152074&dopt=GenPept&RID=T26J5VN701S&log$=prottop&blast_rank=47) | steroid-sensitive protein 1 precursor [Homo sapiens] >ref|NP_955806.1| steroid-sensitive protein 1 precursor [Homo sapiens] | [18.0](http://blast.ncbi.nlm.nih.gov/Blast.cgi#41152074%2341152074) | 18.0 | 80% | 647 |
| [NP_006633.1](http://www.ncbi.nlm.nih.gov/entrez/query.fcgi?cmd=Retrieve&db=Protein&list_uids=28269672&dopt=GenPept&RID=T26J5VN701S&log$=prottop&blast_rank=48) | serologically defined colon cancer antigen 8 [Homo sapiens] | [18.0](http://blast.ncbi.nlm.nih.gov/Blast.cgi#28269672%2328269672) | 18.0 | 60% | 647 |
| [NP_001166150.1](http://www.ncbi.nlm.nih.gov/entrez/query.fcgi?cmd=Retrieve&db=Protein&list_uids=289547721&dopt=GenPept&RID=T26J5VN701S&log$=prottop&blast_rank=49) | zinc finger protein 764 isoform 2 [Homo sapiens] | [17.6](http://blast.ncbi.nlm.nih.gov/Blast.cgi#289547721%23289547721) | 17.6 | 70% | 868 |
| [XP_002343409.1](http://www.ncbi.nlm.nih.gov/entrez/query.fcgi?cmd=Retrieve&db=Protein&list_uids=239745211&dopt=GenPept&RID=T26J5VN701S&log$=prottop&blast_rank=50) | PREDICTED: hypothetical protein XP_002343409 [Homo sapiens] | [17.6](http://blast.ncbi.nlm.nih.gov/Blast.cgi#239745211%23239745211) | 17.6 | 40% | 868 |
| [NP_001123990.1](http://www.ncbi.nlm.nih.gov/entrez/query.fcgi?cmd=Retrieve&db=Protein&list_uids=194473683&dopt=GenPept&RID=T26J5VN701S&log$=prottop&blast_rank=51) | chondroitin sulfate N-acetylgalactosaminyltransferase 1 [Homo sapiens] >ref|NP_060841.5| chondroitin sulfate N-acetylgalactosaminyltransferase 1 [Homo sapiens] | [17.6](http://blast.ncbi.nlm.nih.gov/Blast.cgi#194473683%23194473683) | 17.6 | 40% | 868 |
| [NP_055284.3](http://www.ncbi.nlm.nih.gov/entrez/query.fcgi?cmd=Retrieve&db=Protein&list_uids=153252068&dopt=GenPept&RID=T26J5VN701S&log$=prottop&blast_rank=52) | testes-specific heterogenous nuclear ribonucleoprotein G-T [Homo sapiens] | [17.6](http://blast.ncbi.nlm.nih.gov/Blast.cgi#153252068%23153252068) | 17.6 | 60% | 868 |
| [NP_004215.2](http://www.ncbi.nlm.nih.gov/entrez/query.fcgi?cmd=Retrieve&db=Protein&list_uids=150170722&dopt=GenPept&RID=T26J5VN701S&log$=prottop&blast_rank=53) | G protein-coupled receptor 50 [Homo sapiens] | [17.6](http://blast.ncbi.nlm.nih.gov/Blast.cgi#150170722%23150170722) | 27.6 | 60% | 868 |
| [NP_004230.2](http://www.ncbi.nlm.nih.gov/entrez/query.fcgi?cmd=Retrieve&db=Protein&list_uids=190194412&dopt=GenPept&RID=T26J5VN701S&log$=prottop&blast_rank=54) | thyroid hormone receptor interactor 11 [Homo sapiens] | [17.6](http://blast.ncbi.nlm.nih.gov/Blast.cgi#190194412%23190194412) | 17.6 | 80% | 868 |
| [NP_219363.2](http://www.ncbi.nlm.nih.gov/entrez/query.fcgi?cmd=Retrieve&db=Protein&list_uids=289547718&dopt=GenPept&RID=T26J5VN701S&log$=prottop&blast_rank=55) | zinc finger protein 764 isoform 1 [Homo sapiens] | [17.6](http://blast.ncbi.nlm.nih.gov/Blast.cgi#289547718%23289547718) | 17.6 | 70% | 868 |
| [NP_996885.1](http://www.ncbi.nlm.nih.gov/entrez/query.fcgi?cmd=Retrieve&db=Protein&list_uids=46276880&dopt=GenPept&RID=T26J5VN701S&log$=prottop&blast_rank=56) | BCL2-like 11 isoform 9 [Homo sapiens] | [17.6](http://blast.ncbi.nlm.nih.gov/Blast.cgi#46276880%2346276880) | 17.6 | 40% | 868 |
| [NP_955471.1](http://www.ncbi.nlm.nih.gov/entrez/query.fcgi?cmd=Retrieve&db=Protein&list_uids=41349464&dopt=GenPept&RID=T26J5VN701S&log$=prottop&blast_rank=57) | PR domain containing 10 isoform 4 [Homo sapiens] | [17.6](http://blast.ncbi.nlm.nih.gov/Blast.cgi#41349464%2341349464) | 17.6 | 40% | 868 |
| [NP_689920.1](http://www.ncbi.nlm.nih.gov/entrez/query.fcgi?cmd=Retrieve&db=Protein&list_uids=27544933&dopt=GenPept&RID=T26J5VN701S&log$=prottop&blast_rank=58) | solute carrier family 25, member 16 [Homo sapiens] | [17.6](http://blast.ncbi.nlm.nih.gov/Blast.cgi#27544933%2327544933) | 17.6 | 40% | 868 |
| [NP_057095.2](http://www.ncbi.nlm.nih.gov/entrez/query.fcgi?cmd=Retrieve&db=Protein&list_uids=67078404&dopt=GenPept&RID=T26J5VN701S&log$=prottop&blast_rank=59) | trans-2-enoyl-CoA reductase, mitochondrial isoform a [Homo sapiens] | [17.6](http://blast.ncbi.nlm.nih.gov/Blast.cgi#67078404%2367078404) | 17.6 | 40% | 868 |
| [NP_955469.1](http://www.ncbi.nlm.nih.gov/entrez/query.fcgi?cmd=Retrieve&db=Protein&list_uids=41349460&dopt=GenPept&RID=T26J5VN701S&log$=prottop&blast_rank=60) | PR domain containing 10 isoform 2 [Homo sapiens] | [17.6](http://blast.ncbi.nlm.nih.gov/Blast.cgi#41349460%2341349460) | 17.6 | 40% | 868 |
| [NP_064613.2](http://www.ncbi.nlm.nih.gov/entrez/query.fcgi?cmd=Retrieve&db=Protein&list_uids=41349458&dopt=GenPept&RID=T26J5VN701S&log$=prottop&blast_rank=61) | PR domain containing 10 isoform 1 [Homo sapiens] | [17.6](http://blast.ncbi.nlm.nih.gov/Blast.cgi#41349458%2341349458) | 17.6 | 40% | 868 |
| [NP_005103.2](http://www.ncbi.nlm.nih.gov/entrez/query.fcgi?cmd=Retrieve&db=Protein&list_uids=53729352&dopt=GenPept&RID=T26J5VN701S&log$=prottop&blast_rank=62) | WD repeat-containing protein 1 isoform 2 [Homo sapiens] | [17.6](http://blast.ncbi.nlm.nih.gov/Blast.cgi#53729352%2353729352) | 17.6 | 40% | 868 |
| [NP_000074.2](http://www.ncbi.nlm.nih.gov/entrez/query.fcgi?cmd=Retrieve&db=Protein&list_uids=119433677&dopt=GenPept&RID=T26J5VN701S&log$=prottop&blast_rank=63) | chloride channel 1, skeletal muscle [Homo sapiens] | [17.6](http://blast.ncbi.nlm.nih.gov/Blast.cgi#119433677%23119433677) | 17.6 | 40% | 868 |
| [NP_005261.2](http://www.ncbi.nlm.nih.gov/entrez/query.fcgi?cmd=Retrieve&db=Protein&list_uids=86991432&dopt=GenPept&RID=T26J5VN701S&log$=prottop&blast_rank=64) | GLI-Kruppel family member GLI2 [Homo sapiens] | [17.6](http://blast.ncbi.nlm.nih.gov/Blast.cgi#86991432%2386991432) | 17.6 | 70% | 868 |
| [NP_958780.1](http://www.ncbi.nlm.nih.gov/entrez/query.fcgi?cmd=Retrieve&db=Protein&list_uids=41322912&dopt=GenPept&RID=T26J5VN701S&log$=prottop&blast_rank=65) | plectin isoform 1f [Homo sapiens] | [17.6](http://blast.ncbi.nlm.nih.gov/Blast.cgi#41322912%2341322912) | 120 | 60% | 868 |
| [NP_958786.1](http://www.ncbi.nlm.nih.gov/entrez/query.fcgi?cmd=Retrieve&db=Protein&list_uids=41322923&dopt=GenPept&RID=T26J5VN701S&log$=prottop&blast_rank=66) | plectin isoform 1a [Homo sapiens] | [17.6](http://blast.ncbi.nlm.nih.gov/Blast.cgi#41322923%2341322923) | 120 | 60% | 868 |
| [NP_000436.2](http://www.ncbi.nlm.nih.gov/entrez/query.fcgi?cmd=Retrieve&db=Protein&list_uids=47607492&dopt=GenPept&RID=T26J5VN701S&log$=prottop&blast_rank=67) | plectin isoform 1c [Homo sapiens] | [17.6](http://blast.ncbi.nlm.nih.gov/Blast.cgi#47607492%2347607492) | 120 | 60% | 868 |
| [NP_958783.1](http://www.ncbi.nlm.nih.gov/entrez/query.fcgi?cmd=Retrieve&db=Protein&list_uids=41322910&dopt=GenPept&RID=T26J5VN701S&log$=prottop&blast_rank=68) | plectin isoform 1d [Homo sapiens] | [17.6](http://blast.ncbi.nlm.nih.gov/Blast.cgi#41322910%2341322910) | 120 | 60% | 868 |
| [NP_958785.1](http://www.ncbi.nlm.nih.gov/entrez/query.fcgi?cmd=Retrieve&db=Protein&list_uids=41322914&dopt=GenPept&RID=T26J5VN701S&log$=prottop&blast_rank=69) | plectin isoform 1g [Homo sapiens] | [17.6](http://blast.ncbi.nlm.nih.gov/Blast.cgi#41322914%2341322914) | 120 | 60% | 868 |
| [NP_958781.1](http://www.ncbi.nlm.nih.gov/entrez/query.fcgi?cmd=Retrieve&db=Protein&list_uids=41322908&dopt=GenPept&RID=T26J5VN701S&log$=prottop&blast_rank=70) | plectin isoform 1e [Homo sapiens] | [17.6](http://blast.ncbi.nlm.nih.gov/Blast.cgi#41322908%2341322908) | 120 | 60% | 868 |
| [NP_958782.1](http://www.ncbi.nlm.nih.gov/entrez/query.fcgi?cmd=Retrieve&db=Protein&list_uids=41322916&dopt=GenPept&RID=T26J5VN701S&log$=prottop&blast_rank=71) | plectin isoform 1 [Homo sapiens] | [17.6](http://blast.ncbi.nlm.nih.gov/Blast.cgi#41322916%2341322916) | 120 | 60% | 868 |
| [NP_958784.1](http://www.ncbi.nlm.nih.gov/entrez/query.fcgi?cmd=Retrieve&db=Protein&list_uids=41322919&dopt=GenPept&RID=T26J5VN701S&log$=prottop&blast_rank=72) | plectin isoform 1b [Homo sapiens] | [17.6](http://blast.ncbi.nlm.nih.gov/Blast.cgi#41322919%2341322919) | 120 | 60% | 868 |
| [NP_076982.3](http://www.ncbi.nlm.nih.gov/entrez/query.fcgi?cmd=Retrieve&db=Protein&list_uids=83779010&dopt=GenPept&RID=T26J5VN701S&log$=prottop&blast_rank=73) | SECIS binding protein 2 [Homo sapiens] | [17.6](http://blast.ncbi.nlm.nih.gov/Blast.cgi#83779010%2383779010) | 17.6 | 60% | 868 |
| [NP_055868.1](http://www.ncbi.nlm.nih.gov/entrez/query.fcgi?cmd=Retrieve&db=Protein&list_uids=55769554&dopt=GenPept&RID=T26J5VN701S&log$=prottop&blast_rank=74) | protein tyrosine phosphatase, receptor type, f polypeptide (PTPRF), interacting protein (liprin), alpha 4 [Homo sapiens] | [17.6](http://blast.ncbi.nlm.nih.gov/Blast.cgi#55769554%2355769554) | 17.6 | 70% | 868 |
| [NP_955470.1](http://www.ncbi.nlm.nih.gov/entrez/query.fcgi?cmd=Retrieve&db=Protein&list_uids=41349462&dopt=GenPept&RID=T26J5VN701S&log$=prottop&blast_rank=75) | PR domain containing 10 isoform 3 [Homo sapiens] | [17.6](http://blast.ncbi.nlm.nih.gov/Blast.cgi#41349462%2341349462) | 17.6 | 40% | 868 |
| [NP_060350.1](http://www.ncbi.nlm.nih.gov/entrez/query.fcgi?cmd=Retrieve&db=Protein&list_uids=8923528&dopt=GenPept&RID=T26J5VN701S&log$=prottop&blast_rank=76) | hypothetical protein LOC54980 [Homo sapiens] | [17.6](http://blast.ncbi.nlm.nih.gov/Blast.cgi#8923528%238923528) | 17.6 | 40% | 868 |
| [NP_060352.1](http://www.ncbi.nlm.nih.gov/entrez/query.fcgi?cmd=Retrieve&db=Protein&list_uids=8923532&dopt=GenPept&RID=T26J5VN701S&log$=prottop&blast_rank=77) | CLN6 protein [Homo sapiens] | [17.6](http://blast.ncbi.nlm.nih.gov/Blast.cgi#8923532%238923532) | 17.6 | 60% | 868 |
| [NP_003400.2](http://www.ncbi.nlm.nih.gov/entrez/query.fcgi?cmd=Retrieve&db=Protein&list_uids=19923242&dopt=GenPept&RID=T26J5VN701S&log$=prottop&blast_rank=78) | zinc finger protein 161 homolog [Homo sapiens] >ref|NP_001137295.1| zinc finger protein 161 homolog [Homo sapiens] | [17.6](http://blast.ncbi.nlm.nih.gov/Blast.cgi#19923242%2319923242) | 17.6 | 40% | 868 |
| [NP_612808.1](http://www.ncbi.nlm.nih.gov/entrez/query.fcgi?cmd=Retrieve&db=Protein&list_uids=21040334&dopt=GenPept&RID=T26J5VN701S&log$=prottop&blast_rank=79) | B-cell CLL/lymphoma 11B isoform 1 [Homo sapiens] | [17.6](http://blast.ncbi.nlm.nih.gov/Blast.cgi#21040334%2321040334) | 17.6 | 60% | 868 |
| [NP_065980.1](http://www.ncbi.nlm.nih.gov/entrez/query.fcgi?cmd=Retrieve&db=Protein&list_uids=51317373&dopt=GenPept&RID=T26J5VN701S&log$=prottop&blast_rank=80) | netrin-G1 ligand precursor [Homo sapiens] | [17.6](http://blast.ncbi.nlm.nih.gov/Blast.cgi#51317373%2351317373) | 17.6 | 60% | 868 |
| [NP_075049.1](http://www.ncbi.nlm.nih.gov/entrez/query.fcgi?cmd=Retrieve&db=Protein&list_uids=12597635&dopt=GenPept&RID=T26J5VN701S&log$=prottop&blast_rank=81) | B-cell CLL/lymphoma 11B isoform 2 [Homo sapiens] | [17.6](http://blast.ncbi.nlm.nih.gov/Blast.cgi#12597635%2312597635) | 17.6 | 60% | 868 |
| [NP_003038.2](http://www.ncbi.nlm.nih.gov/entrez/query.fcgi?cmd=Retrieve&db=Protein&list_uids=27777632&dopt=GenPept&RID=T26J5VN701S&log$=prottop&blast_rank=82) | solute carrier family 9, isoform A1 [Homo sapiens] | [17.6](http://blast.ncbi.nlm.nih.gov/Blast.cgi#27777632%2327777632) | 33.9 | 90% | 868 |
| [NP_059830.1](http://www.ncbi.nlm.nih.gov/entrez/query.fcgi?cmd=Retrieve&db=Protein&list_uids=9257257&dopt=GenPept&RID=T26J5VN701S&log$=prottop&blast_rank=83) | WD repeat-containing protein 1 isoform 1 [Homo sapiens] | [17.6](http://blast.ncbi.nlm.nih.gov/Blast.cgi#9257257%239257257) | 17.6 | 40% | 868 |
| [NP_000669.1](http://www.ncbi.nlm.nih.gov/entrez/query.fcgi?cmd=Retrieve&db=Protein&list_uids=4501957&dopt=GenPept&RID=T26J5VN701S&log$=prottop&blast_rank=84) | alpha-1D-adrenergic receptor [Homo sapiens] | [17.6](http://blast.ncbi.nlm.nih.gov/Blast.cgi#4501957%234501957) | 17.6 | 40% | 868 |
| [NP_000157.1](http://www.ncbi.nlm.nih.gov/entrez/query.fcgi?cmd=Retrieve&db=Protein&list_uids=4504005&dopt=GenPept&RID=T26J5VN701S&log$=prottop&blast_rank=85) | gap junction protein, beta 1, 32kDa [Homo sapiens] >ref|NP_001091111.1| gap junction protein, beta 1, 32kDa [Homo sapiens] | [17.6](http://blast.ncbi.nlm.nih.gov/Blast.cgi#4504005%234504005) | 17.6 | 80% | 868 |
| [NP_000159.3](http://www.ncbi.nlm.nih.gov/entrez/query.fcgi?cmd=Retrieve&db=Protein&list_uids=119393899&dopt=GenPept&RID=T26J5VN701S&log$=prottop&blast_rank=86) | GLI-Kruppel family member GLI3 [Homo sapiens] | [17.6](http://blast.ncbi.nlm.nih.gov/Blast.cgi#119393899%23119393899) | 17.6 | 70% | 868 |
| [NP_001161744.1](http://www.ncbi.nlm.nih.gov/entrez/query.fcgi?cmd=Retrieve&db=Protein&list_uids=269954694&dopt=GenPept&RID=T26J5VN701S&log$=prottop&blast_rank=87) | inositol 1,4,5-triphosphate receptor, type 1 isoform 3 [Homo sapiens] | [17.2](http://blast.ncbi.nlm.nih.gov/Blast.cgi#269954694%23269954694) | 17.2 | 70% | 1165 |
| [NP_001093422.2](http://www.ncbi.nlm.nih.gov/entrez/query.fcgi?cmd=Retrieve&db=Protein&list_uids=269954690&dopt=GenPept&RID=T26J5VN701S&log$=prottop&blast_rank=88) | inositol 1,4,5-triphosphate receptor, type 1 isoform 1 [Homo sapiens] | [17.2](http://blast.ncbi.nlm.nih.gov/Blast.cgi#269954690%23269954690) | 17.2 | 70% | 1165 |
| [NP_079199.2](http://www.ncbi.nlm.nih.gov/entrez/query.fcgi?cmd=Retrieve&db=Protein&list_uids=27477134&dopt=GenPept&RID=T26J5VN701S&log$=prottop&blast_rank=89) | nucleoporin 210 precursor [Homo sapiens] | [17.2](http://blast.ncbi.nlm.nih.gov/Blast.cgi#27477134%2327477134) | 17.2 | 60% | 1165 |
| [NP_001073878.1](http://www.ncbi.nlm.nih.gov/entrez/query.fcgi?cmd=Retrieve&db=Protein&list_uids=122937213&dopt=GenPept&RID=T26J5VN701S&log$=prottop&blast_rank=90) | zinc finger protein 99 [Homo sapiens] | [17.2](http://blast.ncbi.nlm.nih.gov/Blast.cgi#122937213%23122937213) | 17.2 | 90% | 1165 |
| [NP_775810.2](http://www.ncbi.nlm.nih.gov/entrez/query.fcgi?cmd=Retrieve&db=Protein&list_uids=109240540&dopt=GenPept&RID=T26J5VN701S&log$=prottop&blast_rank=91) | zinc finger protein 596 [Homo sapiens] >ref|NP_001035880.1| zinc finger protein 596 [Homo sapiens] >ref|NP_001035881.1| zinc finger protein 596 [Homo sapiens] | [17.2](http://blast.ncbi.nlm.nih.gov/Blast.cgi#109240540%23109240540) | 43.1 | 70% | 1165 |
| [NP_005931.2](http://www.ncbi.nlm.nih.gov/entrez/query.fcgi?cmd=Retrieve&db=Protein&list_uids=58331148&dopt=GenPept&RID=T26J5VN701S&log$=prottop&blast_rank=92) | matrix metalloproteinase 11 preproprotein [Homo sapiens] | [17.2](http://blast.ncbi.nlm.nih.gov/Blast.cgi#58331148%2358331148) | 17.2 | 60% | 1165 |
| [NP_003631.2](http://www.ncbi.nlm.nih.gov/entrez/query.fcgi?cmd=Retrieve&db=Protein&list_uids=38569394&dopt=GenPept&RID=T26J5VN701S&log$=prottop&blast_rank=93) | inhibitor of kappa light polypeptide gene enhancer in B-cells, kinase complex-associated protein [Homo sapiens] | [17.2](http://blast.ncbi.nlm.nih.gov/Blast.cgi#38569394%2338569394) | 31.0 | 80% | 1165 |
| [NP_006589.2](http://www.ncbi.nlm.nih.gov/entrez/query.fcgi?cmd=Retrieve&db=Protein&list_uids=123701900&dopt=GenPept&RID=T26J5VN701S&log$=prottop&blast_rank=94) | solute carrier family 12 (potassium/chloride transporters), member 7 [Homo sapiens] | [17.2](http://blast.ncbi.nlm.nih.gov/Blast.cgi#123701900%23123701900) | 17.2 | 90% | 1165 |
| [NP_115803.1](http://www.ncbi.nlm.nih.gov/entrez/query.fcgi?cmd=Retrieve&db=Protein&list_uids=33286444&dopt=GenPept&RID=T26J5VN701S&log$=prottop&blast_rank=95) | mastermind-like 2 [Homo sapiens] | [17.2](http://blast.ncbi.nlm.nih.gov/Blast.cgi#33286444%2333286444) | 17.2 | 60% | 1165 |
| [NP_055831.1](http://www.ncbi.nlm.nih.gov/entrez/query.fcgi?cmd=Retrieve&db=Protein&list_uids=150170729&dopt=GenPept&RID=T26J5VN701S&log$=prottop&blast_rank=96) | microtubule associated serine/threonine kinase 3 [Homo sapiens] | [17.2](http://blast.ncbi.nlm.nih.gov/Blast.cgi#150170729%23150170729) | 17.2 | 80% | 1165 |
| [NP_065740.2](http://www.ncbi.nlm.nih.gov/entrez/query.fcgi?cmd=Retrieve&db=Protein&list_uids=31563526&dopt=GenPept&RID=T26J5VN701S&log$=prottop&blast_rank=97) | solute carrier family 24 (sodium/potassium/calcium exchanger), member 3 precursor [Homo sapiens] | [17.2](http://blast.ncbi.nlm.nih.gov/Blast.cgi#31563526%2331563526) | 17.2 | 70% | 1165 |
| [NP_002213.5](http://www.ncbi.nlm.nih.gov/entrez/query.fcgi?cmd=Retrieve&db=Protein&list_uids=269954692&dopt=GenPept&RID=T26J5VN701S&log$=prottop&blast_rank=98) | inositol 1,4,5-triphosphate receptor, type 1 isoform 2 [Homo sapiens] | [17.2](http://blast.ncbi.nlm.nih.gov/Blast.cgi#269954692%23269954692) | 17.2 | 70% | 1165 |
| [NP_060176.2](http://www.ncbi.nlm.nih.gov/entrez/query.fcgi?cmd=Retrieve&db=Protein&list_uids=38327642&dopt=GenPept&RID=T26J5VN701S&log$=prottop&blast_rank=99) | WD repeat domain 55 [Homo sapiens] | [17.2](http://blast.ncbi.nlm.nih.gov/Blast.cgi#38327642%2338327642) | 17.2 | 60% | 1165 |

| **Accession** | **Proteins with a match to GLGVGHK peptide** | **[Max score](http://blast.ncbi.nlm.nih.gov/Blast.cgi?CMD=Get&ALIGNMENTS=100&ALIGNMENT_VIEW=Pairwise&CDD_SEARCH_STATE=1&DATABASE_SORT=0&DESCRIPTIONS=100&ENTREZ_QUERY=txid9606 %5BORGN%5D&FIRST_QUERY_NUM=0&FORMAT_OBJECT=Alignment&FORMAT_PAGE_TARGET=&FORMAT_TYPE=HTML&GET_SEQUENCE=yes&I_THRESH=&MASK_CHAR=2&MASK_COLOR=1&NEW_DESIGN=on&NEW_VIEW=yes&NUM_OVERVIEW=100&OLD_BLAST=false&PAGE=Proteins&QUERY_INDEX=0&QUERY_NUMBER=0&RESULTS_PAGE_TARGET=&RID=T270DUCN016&SHOW_LINKOUT=yes&SHOW_OVERVIEW=yes&STEP_NUMBER=&WORD_SIZE=2&DISPLAY_SORT=1&HSP_SORT=1" \l "sort_mark)** | **[Total score](http://blast.ncbi.nlm.nih.gov/Blast.cgi?CMD=Get&ALIGNMENTS=100&ALIGNMENT_VIEW=Pairwise&CDD_SEARCH_STATE=1&DATABASE_SORT=0&DESCRIPTIONS=100&ENTREZ_QUERY=txid9606 %5BORGN%5D&FIRST_QUERY_NUM=0&FORMAT_OBJECT=Alignment&FORMAT_PAGE_TARGET=&FORMAT_TYPE=HTML&GET_SEQUENCE=yes&I_THRESH=&MASK_CHAR=2&MASK_COLOR=1&NEW_DESIGN=on&NEW_VIEW=yes&NUM_OVERVIEW=100&OLD_BLAST=false&PAGE=Proteins&QUERY_INDEX=0&QUERY_NUMBER=0&RESULTS_PAGE_TARGET=&RID=T270DUCN016&SHOW_LINKOUT=yes&SHOW_OVERVIEW=yes&STEP_NUMBER=&WORD_SIZE=2&DISPLAY_SORT=2&HSP_SORT=1" \l "sort_mark)** | **[Query coverage](http://blast.ncbi.nlm.nih.gov/Blast.cgi?CMD=Get&ALIGNMENTS=100&ALIGNMENT_VIEW=Pairwise&CDD_SEARCH_STATE=1&DATABASE_SORT=0&DESCRIPTIONS=100&ENTREZ_QUERY=txid9606 %5BORGN%5D&FIRST_QUERY_NUM=0&FORMAT_OBJECT=Alignment&FORMAT_PAGE_TARGET=&FORMAT_TYPE=HTML&GET_SEQUENCE=yes&I_THRESH=&MASK_CHAR=2&MASK_COLOR=1&NEW_DESIGN=on&NEW_VIEW=yes&NUM_OVERVIEW=100&OLD_BLAST=false&PAGE=Proteins&QUERY_INDEX=0&QUERY_NUMBER=0&RESULTS_PAGE_TARGET=&RID=T270DUCN016&SHOW_LINKOUT=yes&SHOW_OVERVIEW=yes&STEP_NUMBER=&WORD_SIZE=2&DISPLAY_SORT=4&HSP_SORT=0" \l "sort_mark)** | **[E value](http://blast.ncbi.nlm.nih.gov/Blast.cgi?CMD=Get&ALIGNMENTS=100&ALIGNMENT_VIEW=Pairwise&CDD_SEARCH_STATE=1&DATABASE_SORT=0&DESCRIPTIONS=100&ENTREZ_QUERY=txid9606 %5BORGN%5D&FIRST_QUERY_NUM=0&FORMAT_OBJECT=Alignment&FORMAT_PAGE_TARGET=&FORMAT_TYPE=HTML&GET_SEQUENCE=yes&I_THRESH=&MASK_CHAR=2&MASK_COLOR=1&NEW_DESIGN=on&NEW_VIEW=yes&NUM_OVERVIEW=100&OLD_BLAST=false&PAGE=Proteins&QUERY_INDEX=0&QUERY_NUMBER=0&RESULTS_PAGE_TARGET=&RID=T270DUCN016&SHOW_LINKOUT=yes&SHOW_OVERVIEW=yes&STEP_NUMBER=&WORD_SIZE=2&DISPLAY_SORT=0&HSP_SORT=0" \l "sort_mark)** |
| --- | --- | --- | --- | --- | --- |
| [NP_001003700.1](http://www.ncbi.nlm.nih.gov/entrez/query.fcgi?cmd=Retrieve&db=Protein&list_uids=270132935&dopt=GenPept&RID=T270DUCN016&log$=prottop&blast_rank=1) | ras responsive element binding protein 1 isoform 3 [Homo sapiens] | [18.5](http://blast.ncbi.nlm.nih.gov/Blast.cgi#270132935%23270132935) | 18.5 | 100% | 338 |
| [NP_001003698.1](http://www.ncbi.nlm.nih.gov/entrez/query.fcgi?cmd=Retrieve&db=Protein&list_uids=51173735&dopt=GenPept&RID=T270DUCN016&log$=prottop&blast_rank=2) | ras responsive element binding protein 1 isoform 2 [Homo sapiens] >ref|NP_001161816.1| ras responsive element binding protein 1 isoform 2 [Homo sapiens] | [18.5](http://blast.ncbi.nlm.nih.gov/Blast.cgi#51173735%2351173735) | 18.5 | 100% | 338 |
| [NP_001003699.1](http://www.ncbi.nlm.nih.gov/entrez/query.fcgi?cmd=Retrieve&db=Protein&list_uids=51173737&dopt=GenPept&RID=T270DUCN016&log$=prottop&blast_rank=3) | ras responsive element binding protein 1 isoform 1 [Homo sapiens] | [18.5](http://blast.ncbi.nlm.nih.gov/Blast.cgi#51173737%2351173737) | 18.5 | 100% | 338 |
| [NP_001157739.1](http://www.ncbi.nlm.nih.gov/entrez/query.fcgi?cmd=Retrieve&db=Protein&list_uids=256773178&dopt=GenPept&RID=T270DUCN016&log$=prottop&blast_rank=4) | WD repeat domain 46 isoform 2 [Homo sapiens] | [18.0](http://blast.ncbi.nlm.nih.gov/Blast.cgi#256773178%23256773178) | 18.0 | 71% | 453 |
| [NP_005443.3](http://www.ncbi.nlm.nih.gov/entrez/query.fcgi?cmd=Retrieve&db=Protein&list_uids=256773176&dopt=GenPept&RID=T270DUCN016&log$=prottop&blast_rank=5) | WD repeat domain 46 isoform 1 [Homo sapiens] | [18.0](http://blast.ncbi.nlm.nih.gov/Blast.cgi#256773176%23256773176) | 18.0 | 71% | 453 |
| [NP_001095886.1](http://www.ncbi.nlm.nih.gov/entrez/query.fcgi?cmd=Retrieve&db=Protein&list_uids=156231037&dopt=GenPept&RID=T270DUCN016&log$=prottop&blast_rank=6) | kininogen 1 isoform 1 [Homo sapiens] | [18.0](http://blast.ncbi.nlm.nih.gov/Blast.cgi#156231037%23156231037) | 48.6 | 100% | 453 |
| [NP_056494.1](http://www.ncbi.nlm.nih.gov/entrez/query.fcgi?cmd=Retrieve&db=Protein&list_uids=7661556&dopt=GenPept&RID=T270DUCN016&log$=prottop&blast_rank=7) | TruB pseudouridine (psi) synthase homolog 2 [Homo sapiens] | [18.0](http://blast.ncbi.nlm.nih.gov/Blast.cgi#7661556%237661556) | 33.1 | 71% | 453 |
| [NP_000422.1](http://www.ncbi.nlm.nih.gov/entrez/query.fcgi?cmd=Retrieve&db=Protein&list_uids=4557769&dopt=GenPept&RID=T270DUCN016&log$=prottop&blast_rank=8) | mevalonate kinase [Homo sapiens] >ref|NP_001107657.1| mevalonate kinase [Homo sapiens] | [18.0](http://blast.ncbi.nlm.nih.gov/Blast.cgi#4557769%234557769) | 18.0 | 71% | 453 |
| [NP_057272.1](http://www.ncbi.nlm.nih.gov/entrez/query.fcgi?cmd=Retrieve&db=Protein&list_uids=7705294&dopt=GenPept&RID=T270DUCN016&log$=prottop&blast_rank=9) | actin-like 6B [Homo sapiens] | [18.0](http://blast.ncbi.nlm.nih.gov/Blast.cgi#7705294%237705294) | 18.0 | 71% | 453 |
| [NP_857595.3](http://www.ncbi.nlm.nih.gov/entrez/query.fcgi?cmd=Retrieve&db=Protein&list_uids=170932532&dopt=GenPept&RID=T270DUCN016&log$=prottop&blast_rank=10) | major facilitator superfamily domain containing 4 [Homo sapiens] | [17.6](http://blast.ncbi.nlm.nih.gov/Blast.cgi#170932532%23170932532) | 17.6 | 85% | 608 |
| [XP_002344230.1](http://www.ncbi.nlm.nih.gov/entrez/query.fcgi?cmd=Retrieve&db=Protein&list_uids=239746628&dopt=GenPept&RID=T270DUCN016&log$=prottop&blast_rank=11) | PREDICTED: hypothetical protein XP_002344230 [Homo sapiens] | [17.2](http://blast.ncbi.nlm.nih.gov/Blast.cgi#239746628%23239746628) | 17.2 | 85% | 816 |
| [NP_001020604.1](http://www.ncbi.nlm.nih.gov/entrez/query.fcgi?cmd=Retrieve&db=Protein&list_uids=70995396&dopt=GenPept&RID=T270DUCN016&log$=prottop&blast_rank=12) | NAD(P)H dehydrogenase, quinone 1 isoform b [Homo sapiens] | [17.2](http://blast.ncbi.nlm.nih.gov/Blast.cgi#70995396%2370995396) | 17.2 | 85% | 816 |
| [NP_001020605.1](http://www.ncbi.nlm.nih.gov/entrez/query.fcgi?cmd=Retrieve&db=Protein&list_uids=70995422&dopt=GenPept&RID=T270DUCN016&log$=prottop&blast_rank=13) | NAD(P)H dehydrogenase, quinone 1 isoform c [Homo sapiens] | [17.2](http://blast.ncbi.nlm.nih.gov/Blast.cgi#70995422%2370995422) | 17.2 | 85% | 816 |
| [NP_001092099.1](http://www.ncbi.nlm.nih.gov/entrez/query.fcgi?cmd=Retrieve&db=Protein&list_uids=148833494&dopt=GenPept&RID=T270DUCN016&log$=prottop&blast_rank=14) | interferon regulatory factor 5 isoform d [Homo sapiens] | [17.2](http://blast.ncbi.nlm.nih.gov/Blast.cgi#148833494%23148833494) | 17.2 | 85% | 816 |
| [NP_001092097.1](http://www.ncbi.nlm.nih.gov/entrez/query.fcgi?cmd=Retrieve&db=Protein&list_uids=148833488&dopt=GenPept&RID=T270DUCN016&log$=prottop&blast_rank=15) | interferon regulatory factor 5 isoform c [Homo sapiens] >ref|NP_001092098.1| interferon regulatory factor 5 isoform c [Homo sapiens] >ref|NP_001092101.1| interferon regulatory factor 5 isoform c [Homo sapiens] | [17.2](http://blast.ncbi.nlm.nih.gov/Blast.cgi#148833488%23148833488) | 17.2 | 85% | 816 |
| [NP_113612.1](http://www.ncbi.nlm.nih.gov/entrez/query.fcgi?cmd=Retrieve&db=Protein&list_uids=13937401&dopt=GenPept&RID=T270DUCN016&log$=prottop&blast_rank=16) | hypothetical protein LOC83541 [Homo sapiens] >ref|NP_997004.1| hypothetical protein LOC83541 [Homo sapiens] >ref|NP_001035812.1| hypothetical protein LOC83541 [Homo sapiens] | [17.2](http://blast.ncbi.nlm.nih.gov/Blast.cgi#13937401%2313937401) | 17.2 | 85% | 816 |
| [NP_000894.1](http://www.ncbi.nlm.nih.gov/entrez/query.fcgi?cmd=Retrieve&db=Protein&list_uids=4505415&dopt=GenPept&RID=T270DUCN016&log$=prottop&blast_rank=17) | NAD(P)H dehydrogenase, quinone 1 isoform a [Homo sapiens] | [17.2](http://blast.ncbi.nlm.nih.gov/Blast.cgi#4505415%234505415) | 17.2 | 85% | 816 |
| [NP_036271.1](http://www.ncbi.nlm.nih.gov/entrez/query.fcgi?cmd=Retrieve&db=Protein&list_uids=6912330&dopt=GenPept&RID=T270DUCN016&log$=prottop&blast_rank=18) | deafness locus associated putative guanine nucleotide exchange factor [Homo sapiens] | [17.2](http://blast.ncbi.nlm.nih.gov/Blast.cgi#6912330%236912330) | 41.4 | 100% | 816 |
| [NP_003357.2](http://www.ncbi.nlm.nih.gov/entrez/query.fcgi?cmd=Retrieve&db=Protein&list_uids=50592988&dopt=GenPept&RID=T270DUCN016&log$=prottop&blast_rank=19) | ubiquinol-cytochrome c reductase core protein II precursor [Homo sapiens] | [17.2](http://blast.ncbi.nlm.nih.gov/Blast.cgi#50592988%2350592988) | 17.2 | 85% | 816 |
| [NP_116032.1](http://www.ncbi.nlm.nih.gov/entrez/query.fcgi?cmd=Retrieve&db=Protein&list_uids=14249182&dopt=GenPept&RID=T270DUCN016&log$=prottop&blast_rank=20) | interferon regulatory factor 5 isoform b [Homo sapiens] >ref|NP_001092100.1| interferon regulatory factor 5 isoform b [Homo sapiens] | [17.2](http://blast.ncbi.nlm.nih.gov/Blast.cgi#14249182%2314249182) | 17.2 | 85% | 816 |
| [NP_002191.1](http://www.ncbi.nlm.nih.gov/entrez/query.fcgi?cmd=Retrieve&db=Protein&list_uids=4504727&dopt=GenPept&RID=T270DUCN016&log$=prottop&blast_rank=21) | interferon regulatory factor 5 isoform a [Homo sapiens] | [17.2](http://blast.ncbi.nlm.nih.gov/Blast.cgi#4504727%234504727) | 17.2 | 85% | 816 |
| [NP_940880.3](http://www.ncbi.nlm.nih.gov/entrez/query.fcgi?cmd=Retrieve&db=Protein&list_uids=254553319&dopt=GenPept&RID=T270DUCN016&log$=prottop&blast_rank=22) | NTPase, KAP family P-loop domain containing 1 isoform 2 [Homo sapiens] | [16.8](http://blast.ncbi.nlm.nih.gov/Blast.cgi#254553319%23254553319) | 16.8 | 71% | 1095 |
| [XP_002345603.1](http://www.ncbi.nlm.nih.gov/entrez/query.fcgi?cmd=Retrieve&db=Protein&list_uids=239753264&dopt=GenPept&RID=T270DUCN016&log$=prottop&blast_rank=23) | PREDICTED: hypothetical protein [Homo sapiens] | [16.8](http://blast.ncbi.nlm.nih.gov/Blast.cgi#239753264%23239753264) | 16.8 | 71% | 1095 |
| [XP_002348141.1](http://www.ncbi.nlm.nih.gov/entrez/query.fcgi?cmd=Retrieve&db=Protein&list_uids=239752182&dopt=GenPept&RID=T270DUCN016&log$=prottop&blast_rank=24) | PREDICTED: hypothetical protein [Homo sapiens] >ref|XP_002345524.1| PREDICTED: hypothetical protein XP_002345524 [Homo sapiens] | [16.8](http://blast.ncbi.nlm.nih.gov/Blast.cgi#239752182%23239752182) | 16.8 | 71% | 1095 |
| [XP_002347748.1](http://www.ncbi.nlm.nih.gov/entrez/query.fcgi?cmd=Retrieve&db=Protein&list_uids=239751211&dopt=GenPept&RID=T270DUCN016&log$=prottop&blast_rank=25) | PREDICTED: hypothetical protein XP_002347748 [Homo sapiens] >ref|XP_002345022.1| PREDICTED: hypothetical protein XP_002345022 [Homo sapiens] | [16.8](http://blast.ncbi.nlm.nih.gov/Blast.cgi#239751211%23239751211) | 16.8 | 71% | 1095 |
| [XP_002348330.1](http://www.ncbi.nlm.nih.gov/entrez/query.fcgi?cmd=Retrieve&db=Protein&list_uids=239747823&dopt=GenPept&RID=T270DUCN016&log$=prottop&blast_rank=26) | PREDICTED: hypothetical protein XP_002348330 [Homo sapiens] | [16.8](http://blast.ncbi.nlm.nih.gov/Blast.cgi#239747823%23239747823) | 16.8 | 71% | 1095 |
| [XP_002343815.1](http://www.ncbi.nlm.nih.gov/entrez/query.fcgi?cmd=Retrieve&db=Protein&list_uids=239746705&dopt=GenPept&RID=T270DUCN016&log$=prottop&blast_rank=27) | PREDICTED: hypothetical protein XP_002343815 [Homo sapiens] | [16.8](http://blast.ncbi.nlm.nih.gov/Blast.cgi#239746705%23239746705) | 16.8 | 71% | 1095 |
| [XP_002343519.1](http://www.ncbi.nlm.nih.gov/entrez/query.fcgi?cmd=Retrieve&db=Protein&list_uids=239745701&dopt=GenPept&RID=T270DUCN016&log$=prottop&blast_rank=28) | PREDICTED: hypothetical protein XP_002343519 [Homo sapiens] | [16.8](http://blast.ncbi.nlm.nih.gov/Blast.cgi#239745701%23239745701) | 16.8 | 71% | 1095 |
| [XP_002342282.1](http://www.ncbi.nlm.nih.gov/entrez/query.fcgi?cmd=Retrieve&db=Protein&list_uids=239741693&dopt=GenPept&RID=T270DUCN016&log$=prottop&blast_rank=29) | PREDICTED: hypothetical protein XP_002342282 [Homo sapiens] | [16.8](http://blast.ncbi.nlm.nih.gov/Blast.cgi#239741693%23239741693) | 16.8 | 71% | 1095 |
| [NP_001128653.1](http://www.ncbi.nlm.nih.gov/entrez/query.fcgi?cmd=Retrieve&db=Protein&list_uids=206597487&dopt=GenPept&RID=T270DUCN016&log$=prottop&blast_rank=30) | solute carrier family 5 (sodium/glucose cotransporter), member 9 isoform 1 [Homo sapiens] | [16.8](http://blast.ncbi.nlm.nih.gov/Blast.cgi#206597487%23206597487) | 30.1 | 71% | 1095 |
| [NP_001011547.2](http://www.ncbi.nlm.nih.gov/entrez/query.fcgi?cmd=Retrieve&db=Protein&list_uids=206597483&dopt=GenPept&RID=T270DUCN016&log$=prottop&blast_rank=31) | solute carrier family 5 (sodium/glucose cotransporter), member 9 isoform 2 [Homo sapiens] | [16.8](http://blast.ncbi.nlm.nih.gov/Blast.cgi#206597483%23206597483) | 30.1 | 71% | 1095 |
| [XP_001719908.1](http://www.ncbi.nlm.nih.gov/entrez/query.fcgi?cmd=Retrieve&db=Protein&list_uids=169209574&dopt=GenPept&RID=T270DUCN016&log$=prottop&blast_rank=32) | PREDICTED: hypothetical protein LOC400558 [Homo sapiens] | [16.8](http://blast.ncbi.nlm.nih.gov/Blast.cgi#169209574%23169209574) | 16.8 | 71% | 1095 |
| [NP_001075224.1](http://www.ncbi.nlm.nih.gov/entrez/query.fcgi?cmd=Retrieve&db=Protein&list_uids=126352608&dopt=GenPept&RID=T270DUCN016&log$=prottop&blast_rank=33) | elastin isoform e precursor [Homo sapiens] | [16.8](http://blast.ncbi.nlm.nih.gov/Blast.cgi#126352608%23126352608) | 95.5 | 71% | 1095 |
| [NP_001099008.1](http://www.ncbi.nlm.nih.gov/entrez/query.fcgi?cmd=Retrieve&db=Protein&list_uids=157694494&dopt=GenPept&RID=T270DUCN016&log$=prottop&blast_rank=34) | MYB binding protein 1a isoform 1 [Homo sapiens] | [16.8](http://blast.ncbi.nlm.nih.gov/Blast.cgi#157694494%23157694494) | 16.8 | 71% | 1095 |
| [NP_000492.2](http://www.ncbi.nlm.nih.gov/entrez/query.fcgi?cmd=Retrieve&db=Protein&list_uids=126352440&dopt=GenPept&RID=T270DUCN016&log$=prottop&blast_rank=35) | elastin isoform a precursor [Homo sapiens] | [16.8](http://blast.ncbi.nlm.nih.gov/Blast.cgi#126352440%23126352440) | 95.5 | 71% | 1095 |
| [NP_001075221.1](http://www.ncbi.nlm.nih.gov/entrez/query.fcgi?cmd=Retrieve&db=Protein&list_uids=126352700&dopt=GenPept&RID=T270DUCN016&log$=prottop&blast_rank=36) | elastin isoform b precursor [Homo sapiens] | [16.8](http://blast.ncbi.nlm.nih.gov/Blast.cgi#126352700%23126352700) | 95.5 | 71% | 1095 |
| [NP_001075222.1](http://www.ncbi.nlm.nih.gov/entrez/query.fcgi?cmd=Retrieve&db=Protein&list_uids=126352322&dopt=GenPept&RID=T270DUCN016&log$=prottop&blast_rank=37) | elastin isoform c precursor [Homo sapiens] | [16.8](http://blast.ncbi.nlm.nih.gov/Blast.cgi#126352322%23126352322) | 95.5 | 71% | 1095 |
| [NP_001075223.1](http://www.ncbi.nlm.nih.gov/entrez/query.fcgi?cmd=Retrieve&db=Protein&list_uids=126352446&dopt=GenPept&RID=T270DUCN016&log$=prottop&blast_rank=38) | elastin isoform d precursor [Homo sapiens] | [16.8](http://blast.ncbi.nlm.nih.gov/Blast.cgi#126352446%23126352446) | 95.5 | 71% | 1095 |
| [NP_000415.2](http://www.ncbi.nlm.nih.gov/entrez/query.fcgi?cmd=Retrieve&db=Protein&list_uids=119395754&dopt=GenPept&RID=T270DUCN016&log$=prottop&blast_rank=39) | keratin 5 [Homo sapiens] | [16.8](http://blast.ncbi.nlm.nih.gov/Blast.cgi#119395754%23119395754) | 30.1 | 71% | 1095 |
| [NP_006805.2](http://www.ncbi.nlm.nih.gov/entrez/query.fcgi?cmd=Retrieve&db=Protein&list_uids=145611426&dopt=GenPept&RID=T270DUCN016&log$=prottop&blast_rank=40) | proteasome inhibitor subunit 1 [Homo sapiens] >ref|NP_848693.2| proteasome inhibitor subunit 1 [Homo sapiens] | [16.8](http://blast.ncbi.nlm.nih.gov/Blast.cgi#145611426%23145611426) | 16.8 | 71% | 1095 |
| [NP_005735.2](http://www.ncbi.nlm.nih.gov/entrez/query.fcgi?cmd=Retrieve&db=Protein&list_uids=187761373&dopt=GenPept&RID=T270DUCN016&log$=prottop&blast_rank=41) | ariadne ubiquitin-conjugating enzyme E2 binding protein homolog 1 [Homo sapiens] | [16.8](http://blast.ncbi.nlm.nih.gov/Blast.cgi#187761373%23187761373) | 30.5 | 100% | 1095 |
| [NP_005546.2](http://www.ncbi.nlm.nih.gov/entrez/query.fcgi?cmd=Retrieve&db=Protein&list_uids=119703753&dopt=GenPept&RID=T270DUCN016&log$=prottop&blast_rank=42) | keratin 6B [Homo sapiens] | [16.8](http://blast.ncbi.nlm.nih.gov/Blast.cgi#119703753%23119703753) | 16.8 | 71% | 1095 |
| [NP_061763.1](http://www.ncbi.nlm.nih.gov/entrez/query.fcgi?cmd=Retrieve&db=Protein&list_uids=11036656&dopt=GenPept&RID=T270DUCN016&log$=prottop&blast_rank=43) | protocadherin beta 7 precursor [Homo sapiens] | [16.8](http://blast.ncbi.nlm.nih.gov/Blast.cgi#11036656%2311036656) | 16.8 | 71% | 1095 |
| [NP_991404.1](http://www.ncbi.nlm.nih.gov/entrez/query.fcgi?cmd=Retrieve&db=Protein&list_uids=45593136&dopt=GenPept&RID=T270DUCN016&log$=prottop&blast_rank=44) | lipolysis stimulated lipoprotein receptor isoform 3 [Homo sapiens] | [16.8](http://blast.ncbi.nlm.nih.gov/Blast.cgi#45593136%2345593136) | 16.8 | 71% | 1095 |
| [NP_005545.1](http://www.ncbi.nlm.nih.gov/entrez/query.fcgi?cmd=Retrieve&db=Protein&list_uids=5031839&dopt=GenPept&RID=T270DUCN016&log$=prottop&blast_rank=45) | keratin 6A [Homo sapiens] | [16.8](http://blast.ncbi.nlm.nih.gov/Blast.cgi#5031839%235031839) | 16.8 | 71% | 1095 |
| [NP_057217.2](http://www.ncbi.nlm.nih.gov/entrez/query.fcgi?cmd=Retrieve&db=Protein&list_uids=38327533&dopt=GenPept&RID=T270DUCN016&log$=prottop&blast_rank=46) | insulin induced protein 2 [Homo sapiens] | [16.8](http://blast.ncbi.nlm.nih.gov/Blast.cgi#38327533%2338327533) | 27.2 | 100% | 1095 |
| [NP_057009.3](http://www.ncbi.nlm.nih.gov/entrez/query.fcgi?cmd=Retrieve&db=Protein&list_uids=45505163&dopt=GenPept&RID=T270DUCN016&log$=prottop&blast_rank=47) | lipolysis stimulated lipoprotein receptor isoform 1 [Homo sapiens] | [16.8](http://blast.ncbi.nlm.nih.gov/Blast.cgi#45505163%2345505163) | 16.8 | 71% | 1095 |
| [NP_061758.1](http://www.ncbi.nlm.nih.gov/entrez/query.fcgi?cmd=Retrieve&db=Protein&list_uids=9256610&dopt=GenPept&RID=T270DUCN016&log$=prottop&blast_rank=48) | protocadherin beta 15 precursor [Homo sapiens] | [16.8](http://blast.ncbi.nlm.nih.gov/Blast.cgi#9256610%239256610) | 16.8 | 71% | 1095 |
| [NP_991403.1](http://www.ncbi.nlm.nih.gov/entrez/query.fcgi?cmd=Retrieve&db=Protein&list_uids=45545424&dopt=GenPept&RID=T270DUCN016&log$=prottop&blast_rank=49) | lipolysis stimulated lipoprotein receptor isoform 2 [Homo sapiens] | [16.8](http://blast.ncbi.nlm.nih.gov/Blast.cgi#45545424%2345545424) | 16.8 | 71% | 1095 |
| [NP_056484.1](http://www.ncbi.nlm.nih.gov/entrez/query.fcgi?cmd=Retrieve&db=Protein&list_uids=7661662&dopt=GenPept&RID=T270DUCN016&log$=prottop&blast_rank=50) | protocadherin beta 5 precursor [Homo sapiens] | [16.8](http://blast.ncbi.nlm.nih.gov/Blast.cgi#7661662%237661662) | 16.8 | 71% | 1095 |
| [NP_000912.3](http://www.ncbi.nlm.nih.gov/entrez/query.fcgi?cmd=Retrieve&db=Protein&list_uids=70608155&dopt=GenPept&RID=T270DUCN016&log$=prottop&blast_rank=51) | phosphodiesterase 3A [Homo sapiens] | [16.8](http://blast.ncbi.nlm.nih.gov/Blast.cgi#70608155%2370608155) | 16.8 | 71% | 1095 |
| [XP_001715701.1](http://www.ncbi.nlm.nih.gov/entrez/query.fcgi?cmd=Retrieve&db=Protein&list_uids=169210101&dopt=GenPept&RID=T270DUCN016&log$=prottop&blast_rank=52) | PREDICTED: hypothetical protein LOC400558 [Homo sapiens] >ref|XP_001717951.1| PREDICTED: hypothetical protein LOC400558 [Homo sapiens] | [16.8](http://blast.ncbi.nlm.nih.gov/Blast.cgi#169210101%23169210101) | 16.8 | 71% | 1095 |
| [NP_001155280.1](http://www.ncbi.nlm.nih.gov/entrez/query.fcgi?cmd=Retrieve&db=Protein&list_uids=240120161&dopt=GenPept&RID=T270DUCN016&log$=prottop&blast_rank=53) | G protein-coupled receptor 144 [Homo sapiens] | [16.8](http://blast.ncbi.nlm.nih.gov/Blast.cgi#240120161%23240120161) | 16.8 | 85% | 1095 |
| [NP_009155.1](http://www.ncbi.nlm.nih.gov/entrez/query.fcgi?cmd=Retrieve&db=Protein&list_uids=39930315&dopt=GenPept&RID=T270DUCN016&log$=prottop&blast_rank=54) | neurexophilin 4 precursor [Homo sapiens] | [16.8](http://blast.ncbi.nlm.nih.gov/Blast.cgi#39930315%2339930315) | 16.8 | 71% | 1095 |
| [NP_005707.1](http://www.ncbi.nlm.nih.gov/entrez/query.fcgi?cmd=Retrieve&db=Protein&list_uids=5031715&dopt=GenPept&RID=T270DUCN016&log$=prottop&blast_rank=55) | regulator of G-protein signalling 19 interacting protein 1 isoform 1 [Homo sapiens] >ref|NP_974199.1| regulator of G-protein signalling 19 interacting protein 1 isoform 1 [Homo sapiens] >ref|NP_974197.1| regulator of G-protein signalling 19 interacting protein 1 isoform 1 [Homo sapiens] | [16.8](http://blast.ncbi.nlm.nih.gov/Blast.cgi#5031715%235031715) | 16.8 | 71% | 1095 |
| [NP_002664.2](http://www.ncbi.nlm.nih.gov/entrez/query.fcgi?cmd=Retrieve&db=Protein&list_uids=40254442&dopt=GenPept&RID=T270DUCN016&log$=prottop&blast_rank=56) | plexin B1 precursor [Homo sapiens] >ref|NP_001123554.1| plexin B1 precursor [Homo sapiens] | [16.8](http://blast.ncbi.nlm.nih.gov/Blast.cgi#40254442%2340254442) | 16.8 | 71% | 1095 |
| [NP_003404.1](http://www.ncbi.nlm.nih.gov/entrez/query.fcgi?cmd=Retrieve&db=Protein&list_uids=4507973&dopt=GenPept&RID=T270DUCN016&log$=prottop&blast_rank=57) | zinc finger protein of the cerebellum 3 [Homo sapiens] | [16.8](http://blast.ncbi.nlm.nih.gov/Blast.cgi#4507973%234507973) | 16.8 | 71% | 1095 |
| [NP_066363.1](http://www.ncbi.nlm.nih.gov/entrez/query.fcgi?cmd=Retrieve&db=Protein&list_uids=28626521&dopt=GenPept&RID=T270DUCN016&log$=prottop&blast_rank=58) | zinc finger, NFX1-type containing 1 [Homo sapiens] | [16.8](http://blast.ncbi.nlm.nih.gov/Blast.cgi#28626521%2328626521) | 29.3 | 85% | 1095 |
| [NP_071378.1](http://www.ncbi.nlm.nih.gov/entrez/query.fcgi?cmd=Retrieve&db=Protein&list_uids=11560152&dopt=GenPept&RID=T270DUCN016&log$=prottop&blast_rank=59) | zinc finger protein 335 [Homo sapiens] | [16.8](http://blast.ncbi.nlm.nih.gov/Blast.cgi#11560152%2311560152) | 16.8 | 71% | 1095 |
| [NP_001138939.1](http://www.ncbi.nlm.nih.gov/entrez/query.fcgi?cmd=Retrieve&db=Protein&list_uids=224586865&dopt=GenPept&RID=T270DUCN016&log$=prottop&blast_rank=60) | natural cytotoxicity triggering receptor 3 isoform c [Homo sapiens] | [16.8](http://blast.ncbi.nlm.nih.gov/Blast.cgi#224586865%23224586865) | 16.8 | 71% | 1095 |
| [NP_002939.2](http://www.ncbi.nlm.nih.gov/entrez/query.fcgi?cmd=Retrieve&db=Protein&list_uids=15431293&dopt=GenPept&RID=T270DUCN016&log$=prottop&blast_rank=61) | ribosomal protein L15 [Homo sapiens] | [16.8](http://blast.ncbi.nlm.nih.gov/Blast.cgi#15431293%2315431293) | 16.8 | 100% | 1095 |
| [NP_001138938.1](http://www.ncbi.nlm.nih.gov/entrez/query.fcgi?cmd=Retrieve&db=Protein&list_uids=224586860&dopt=GenPept&RID=T270DUCN016&log$=prottop&blast_rank=62) | natural cytotoxicity triggering receptor 3 isoform b [Homo sapiens] | [16.8](http://blast.ncbi.nlm.nih.gov/Blast.cgi#224586860%23224586860) | 16.8 | 71% | 1095 |
| [NP_667341.1](http://www.ncbi.nlm.nih.gov/entrez/query.fcgi?cmd=Retrieve&db=Protein&list_uids=24475832&dopt=GenPept&RID=T270DUCN016&log$=prottop&blast_rank=63) | natural cytotoxicity triggering receptor 3 isoform a [Homo sapiens] | [16.8](http://blast.ncbi.nlm.nih.gov/Blast.cgi#24475832%2324475832) | 16.8 | 71% | 1095 |
| [NP_071445.1](http://www.ncbi.nlm.nih.gov/entrez/query.fcgi?cmd=Retrieve&db=Protein&list_uids=11545912&dopt=GenPept&RID=T270DUCN016&log$=prottop&blast_rank=64) | nucleotide-binding oligomerization domain containing 2 [Homo sapiens] | [16.8](http://blast.ncbi.nlm.nih.gov/Blast.cgi#11545912%2311545912) | 16.8 | 85% | 1095 |
| [NP_085155.1](http://www.ncbi.nlm.nih.gov/entrez/query.fcgi?cmd=Retrieve&db=Protein&list_uids=13449287&dopt=GenPept&RID=T270DUCN016&log$=prottop&blast_rank=65) | NG3 protein precursor [Homo sapiens] | [16.8](http://blast.ncbi.nlm.nih.gov/Blast.cgi#13449287%2313449287) | 16.8 | 85% | 1095 |
| [NP_055335.2](http://www.ncbi.nlm.nih.gov/entrez/query.fcgi?cmd=Retrieve&db=Protein&list_uids=157694492&dopt=GenPept&RID=T270DUCN016&log$=prottop&blast_rank=66) | MYB binding protein 1a isoform 2 [Homo sapiens] | [16.8](http://blast.ncbi.nlm.nih.gov/Blast.cgi#157694492%23157694492) | 16.8 | 71% | 1095 |
| [NP_057407.2](http://www.ncbi.nlm.nih.gov/entrez/query.fcgi?cmd=Retrieve&db=Protein&list_uids=110825982&dopt=GenPept&RID=T270DUCN016&log$=prottop&blast_rank=67) | hect domain and RLD 5 [Homo sapiens] | [16.3](http://blast.ncbi.nlm.nih.gov/Blast.cgi#110825982%23110825982) | 30.5 | 85% | 1469 |
| [XP_002344346.1](http://www.ncbi.nlm.nih.gov/entrez/query.fcgi?cmd=Retrieve&db=Protein&list_uids=239508794&dopt=GenPept&RID=T270DUCN016&log$=prottop&blast_rank=68) | PREDICTED: hypothetical protein [Homo sapiens] >ref|XP_002342759.1| PREDICTED: hypothetical protein XP_002342759 [Homo sapiens] >ref|XP_002346912.1| PREDICTED: hypothetical protein XP_002346912 [Homo sapiens] >ref|XP_002346028.1| PREDICTED: hypothetical protein [Homo sapiens] | [15.9](http://blast.ncbi.nlm.nih.gov/Blast.cgi#239508794%23239508794) | 15.9 | 71% | 1971 |
| [XP_001715774.1](http://www.ncbi.nlm.nih.gov/entrez/query.fcgi?cmd=Retrieve&db=Protein&list_uids=169210247&dopt=GenPept&RID=T270DUCN016&log$=prottop&blast_rank=69) | PREDICTED: similar to hCG2041551 [Homo sapiens] | [15.9](http://blast.ncbi.nlm.nih.gov/Blast.cgi#169210247%23169210247) | 15.9 | 71% | 1971 |
| [XP_001718298.1](http://www.ncbi.nlm.nih.gov/entrez/query.fcgi?cmd=Retrieve&db=Protein&list_uids=169210132&dopt=GenPept&RID=T270DUCN016&log$=prottop&blast_rank=70) | PREDICTED: similar to hCG2041551 [Homo sapiens] | [15.9](http://blast.ncbi.nlm.nih.gov/Blast.cgi#169210132%23169210132) | 15.9 | 71% | 1971 |
| [NP_060087.3](http://www.ncbi.nlm.nih.gov/entrez/query.fcgi?cmd=Retrieve&db=Protein&list_uids=148833508&dopt=GenPept&RID=T270DUCN016&log$=prottop&blast_rank=71) | notch1 preproprotein [Homo sapiens] | [15.9](http://blast.ncbi.nlm.nih.gov/Blast.cgi#148833508%23148833508) | 15.9 | 71% | 1971 |
| [NP_872374.3](http://www.ncbi.nlm.nih.gov/entrez/query.fcgi?cmd=Retrieve&db=Protein&list_uids=116517224&dopt=GenPept&RID=T270DUCN016&log$=prottop&blast_rank=72) | coiled-coil domain containing 144B [Homo sapiens] | [15.9](http://blast.ncbi.nlm.nih.gov/Blast.cgi#116517224%23116517224) | 15.9 | 71% | 1971 |
| [NP_002694.3](http://www.ncbi.nlm.nih.gov/entrez/query.fcgi?cmd=Retrieve&db=Protein&list_uids=29570798&dopt=GenPept&RID=T270DUCN016&log$=prottop&blast_rank=73) | phosphoribosyl pyrophosphate amidotransferase proprotein [Homo sapiens] | [15.9](http://blast.ncbi.nlm.nih.gov/Blast.cgi#29570798%2329570798) | 15.9 | 71% | 1971 |
| [NP_001166148.1](http://www.ncbi.nlm.nih.gov/entrez/query.fcgi?cmd=Retrieve&db=Protein&list_uids=289547712&dopt=GenPept&RID=T270DUCN016&log$=prottop&blast_rank=74) | zinc finger protein 607 isoform 2 [Homo sapiens] | [15.5](http://blast.ncbi.nlm.nih.gov/Blast.cgi#289547712%23289547712) | 15.5 | 57% | 2644 |
| [NP_001164158.1](http://www.ncbi.nlm.nih.gov/entrez/query.fcgi?cmd=Retrieve&db=Protein&list_uids=282394034&dopt=GenPept&RID=T270DUCN016&log$=prottop&blast_rank=75) | mindbomb homolog 2 isoform 3 [Homo sapiens] | [15.5](http://blast.ncbi.nlm.nih.gov/Blast.cgi#282394034%23282394034) | 15.5 | 57% | 2644 |
| [NP_001164157.1](http://www.ncbi.nlm.nih.gov/entrez/query.fcgi?cmd=Retrieve&db=Protein&list_uids=282394032&dopt=GenPept&RID=T270DUCN016&log$=prottop&blast_rank=76) | mindbomb homolog 2 isoform 2 [Homo sapiens] | [15.5](http://blast.ncbi.nlm.nih.gov/Blast.cgi#282394032%23282394032) | 15.5 | 57% | 2644 |
| [NP_543151.2](http://www.ncbi.nlm.nih.gov/entrez/query.fcgi?cmd=Retrieve&db=Protein&list_uids=282394030&dopt=GenPept&RID=T270DUCN016&log$=prottop&blast_rank=77) | mindbomb homolog 2 isoform 1 [Homo sapiens] | [15.5](http://blast.ncbi.nlm.nih.gov/Blast.cgi#282394030%23282394030) | 15.5 | 57% | 2644 |
| [NP_001157745.1](http://www.ncbi.nlm.nih.gov/entrez/query.fcgi?cmd=Retrieve&db=Protein&list_uids=256017163&dopt=GenPept&RID=T270DUCN016&log$=prottop&blast_rank=78) | MAX-interacting protein isoform 1 [Homo sapiens] | [15.5](http://blast.ncbi.nlm.nih.gov/Blast.cgi#256017163%23256017163) | 15.5 | 57% | 2644 |
| [NP_001074010.2](http://www.ncbi.nlm.nih.gov/entrez/query.fcgi?cmd=Retrieve&db=Protein&list_uids=256017159&dopt=GenPept&RID=T270DUCN016&log$=prottop&blast_rank=79) | MAX-interacting protein isoform 2 [Homo sapiens] | [15.5](http://blast.ncbi.nlm.nih.gov/Blast.cgi#256017159%23256017159) | 15.5 | 57% | 2644 |
| [NP_116078.4](http://www.ncbi.nlm.nih.gov/entrez/query.fcgi?cmd=Retrieve&db=Protein&list_uids=289547710&dopt=GenPept&RID=T270DUCN016&log$=prottop&blast_rank=80) | zinc finger protein 607 isoform 1 [Homo sapiens] | [15.5](http://blast.ncbi.nlm.nih.gov/Blast.cgi#289547710%23289547710) | 15.5 | 57% | 2644 |
| [XP_002345221.1](http://www.ncbi.nlm.nih.gov/entrez/query.fcgi?cmd=Retrieve&db=Protein&list_uids=239757261&dopt=GenPept&RID=T270DUCN016&log$=prottop&blast_rank=81) | PREDICTED: hypothetical protein isoform 1 [Homo sapiens] | [15.5](http://blast.ncbi.nlm.nih.gov/Blast.cgi#239757261%23239757261) | 15.5 | 85% | 2644 |
| [XP_002345069.1](http://www.ncbi.nlm.nih.gov/entrez/query.fcgi?cmd=Retrieve&db=Protein&list_uids=239756827&dopt=GenPept&RID=T270DUCN016&log$=prottop&blast_rank=82) | PREDICTED: hypothetical protein [Homo sapiens] | [15.5](http://blast.ncbi.nlm.nih.gov/Blast.cgi#239756827%23239756827) | 15.5 | 57% | 2644 |
| [XP_002344877.1](http://www.ncbi.nlm.nih.gov/entrez/query.fcgi?cmd=Retrieve&db=Protein&list_uids=239756268&dopt=GenPept&RID=T270DUCN016&log$=prottop&blast_rank=83) | PREDICTED: hypothetical protein XP_002344877 [Homo sapiens] | [15.5](http://blast.ncbi.nlm.nih.gov/Blast.cgi#239756268%23239756268) | 15.5 | 57% | 2644 |
| [XP_002343600.1](http://www.ncbi.nlm.nih.gov/entrez/query.fcgi?cmd=Retrieve&db=Protein&list_uids=239745823&dopt=GenPept&RID=T270DUCN016&log$=prottop&blast_rank=84) | PREDICTED: hypothetical protein XP_002343600 [Homo sapiens] >ref|XP_002347807.1| PREDICTED: hypothetical protein XP_002347807 [Homo sapiens] | [15.5](http://blast.ncbi.nlm.nih.gov/Blast.cgi#239745823%23239745823) | 15.5 | 57% | 2644 |
| [XP_002343381.1](http://www.ncbi.nlm.nih.gov/entrez/query.fcgi?cmd=Retrieve&db=Protein&list_uids=239745177&dopt=GenPept&RID=T270DUCN016&log$=prottop&blast_rank=85) | PREDICTED: hypothetical protein XP_002343381 [Homo sapiens] | [15.5](http://blast.ncbi.nlm.nih.gov/Blast.cgi#239745177%23239745177) | 15.5 | 57% | 2644 |
| [XP_001716116.2](http://www.ncbi.nlm.nih.gov/entrez/query.fcgi?cmd=Retrieve&db=Protein&list_uids=239744401&dopt=GenPept&RID=T270DUCN016&log$=prottop&blast_rank=86) | PREDICTED: hypothetical protein [Homo sapiens] >ref|XP_001716583.2| PREDICTED: hypothetical protein [Homo sapiens] | [15.5](http://blast.ncbi.nlm.nih.gov/Blast.cgi#239744401%23239744401) | 15.5 | 57% | 2644 |
| [NP_001138586.1](http://www.ncbi.nlm.nih.gov/entrez/query.fcgi?cmd=Retrieve&db=Protein&list_uids=223278381&dopt=GenPept&RID=T270DUCN016&log$=prottop&blast_rank=87) | ribosomal RNA processing 12 homolog isoform 2 [Homo sapiens] | [15.5](http://blast.ncbi.nlm.nih.gov/Blast.cgi#223278381%23223278381) | 29.7 | 85% | 2644 |
| [XP_001716427.1](http://www.ncbi.nlm.nih.gov/entrez/query.fcgi?cmd=Retrieve&db=Protein&list_uids=169202942&dopt=GenPept&RID=T270DUCN016&log$=prottop&blast_rank=88) | PREDICTED: hypothetical protein [Homo sapiens] | [15.5](http://blast.ncbi.nlm.nih.gov/Blast.cgi#169202942%23169202942) | 15.5 | 57% | 2644 |
| [NP_002281.2](http://www.ncbi.nlm.nih.gov/entrez/query.fcgi?cmd=Retrieve&db=Protein&list_uids=157419122&dopt=GenPept&RID=T270DUCN016&log$=prottop&blast_rank=89) | laminin, alpha 4 isoform 2 precursor [Homo sapiens] >ref|NP_001098677.1| laminin, alpha 4 isoform 2 precursor [Homo sapiens] | [15.5](http://blast.ncbi.nlm.nih.gov/Blast.cgi#157419122%23157419122) | 29.7 | 85% | 2644 |
| [NP_057594.3](http://www.ncbi.nlm.nih.gov/entrez/query.fcgi?cmd=Retrieve&db=Protein&list_uids=156713448&dopt=GenPept&RID=T270DUCN016&log$=prottop&blast_rank=90) | selenocysteine lyase [Homo sapiens] | [15.5](http://blast.ncbi.nlm.nih.gov/Blast.cgi#156713448%23156713448) | 15.5 | 57% | 2644 |
| [NP_001098676.1](http://www.ncbi.nlm.nih.gov/entrez/query.fcgi?cmd=Retrieve&db=Protein&list_uids=157419124&dopt=GenPept&RID=T270DUCN016&log$=prottop&blast_rank=91) | laminin, alpha 4 isoform 1 precursor [Homo sapiens] | [15.5](http://blast.ncbi.nlm.nih.gov/Blast.cgi#157419124%23157419124) | 29.7 | 85% | 2644 |
| [NP_001092272.1](http://www.ncbi.nlm.nih.gov/entrez/query.fcgi?cmd=Retrieve&db=Protein&list_uids=149274621&dopt=GenPept&RID=T270DUCN016&log$=prottop&blast_rank=92) | centrosomal protein 78kDa isoform a [Homo sapiens] | [15.5](http://blast.ncbi.nlm.nih.gov/Blast.cgi#149274621%23149274621) | 15.5 | 85% | 2644 |
| [NP_001611.1](http://www.ncbi.nlm.nih.gov/entrez/query.fcgi?cmd=Retrieve&db=Protein&list_uids=61743954&dopt=GenPept&RID=T270DUCN016&log$=prottop&blast_rank=93) | AHNAK nucleoprotein isoform 1 [Homo sapiens] | [15.5](http://blast.ncbi.nlm.nih.gov/Blast.cgi#61743954%2361743954) | 28.0 | 85% | 2644 |
| [NP_775771.3](http://www.ncbi.nlm.nih.gov/entrez/query.fcgi?cmd=Retrieve&db=Protein&list_uids=116812624&dopt=GenPept&RID=T270DUCN016&log$=prottop&blast_rank=94) | tau tubulin kinase 2 [Homo sapiens] | [15.5](http://blast.ncbi.nlm.nih.gov/Blast.cgi#116812624%23116812624) | 15.5 | 57% | 2644 |
| [NP_001073889.1](http://www.ncbi.nlm.nih.gov/entrez/query.fcgi?cmd=Retrieve&db=Protein&list_uids=122937241&dopt=GenPept&RID=T270DUCN016&log$=prottop&blast_rank=95) | SH3 and multiple ankyrin repeat domains 3 [Homo sapiens] | [15.5](http://blast.ncbi.nlm.nih.gov/Blast.cgi#122937241%23122937241) | 15.5 | 57% | 2644 |
| [NP_115547.1](http://www.ncbi.nlm.nih.gov/entrez/query.fcgi?cmd=Retrieve&db=Protein&list_uids=149274651&dopt=GenPept&RID=T270DUCN016&log$=prottop&blast_rank=96) | centrosomal protein 78kDa isoform b [Homo sapiens] | [15.5](http://blast.ncbi.nlm.nih.gov/Blast.cgi#149274651%23149274651) | 15.5 | 85% | 2644 |
| [NP_055906.2](http://www.ncbi.nlm.nih.gov/entrez/query.fcgi?cmd=Retrieve&db=Protein&list_uids=93102424&dopt=GenPept&RID=T270DUCN016&log$=prottop&blast_rank=97) | hypothetical protein LOC23116 [Homo sapiens] | [15.5](http://blast.ncbi.nlm.nih.gov/Blast.cgi#93102424%2393102424) | 15.5 | 57% | 2644 |
| [NP_006819.2](http://www.ncbi.nlm.nih.gov/entrez/query.fcgi?cmd=Retrieve&db=Protein&list_uids=76880486&dopt=GenPept&RID=T270DUCN016&log$=prottop&blast_rank=98) | activating signal cointegrator 1 complex subunit 3 isoform a [Homo sapiens] | [15.5](http://blast.ncbi.nlm.nih.gov/Blast.cgi#76880486%2376880486) | 15.5 | 57% | 2644 |
| [NP_009178.3](http://www.ncbi.nlm.nih.gov/entrez/query.fcgi?cmd=Retrieve&db=Protein&list_uids=38569409&dopt=GenPept&RID=T270DUCN016&log$=prottop&blast_rank=99) | synergin, gamma isoform 1 [Homo sapiens] | [15.5](http://blast.ncbi.nlm.nih.gov/Blast.cgi#38569409%2338569409) | 29.7 | 100% | 2644 |
| [NP_005398.1](http://www.ncbi.nlm.nih.gov/entrez/query.fcgi?cmd=Retrieve&db=Protein&list_uids=49087132&dopt=GenPept&RID=T270DUCN016&log$=prottop&blast_rank=100) | sal-like 2 [Homo sapiens] | [15.5](http://blast.ncbi.nlm.nih.gov/Blast.cgi#49087132%2349087132) | 15.5 | 57% | 2644 |

| **Accession** | **Proteins with a match to SYSGYWHSWI peptide** | **[Max score](http://blast.ncbi.nlm.nih.gov/Blast.cgi?CMD=Get&ALIGNMENTS=100&ALIGNMENT_VIEW=Pairwise&CDD_SEARCH_STATE=1&DATABASE_SORT=0&DESCRIPTIONS=100&ENTREZ_QUERY=txid9606 %5BORGN%5D&FIRST_QUERY_NUM=0&FORMAT_OBJECT=Alignment&FORMAT_PAGE_TARGET=&FORMAT_TYPE=HTML&GET_SEQUENCE=yes&I_THRESH=&MASK_CHAR=2&MASK_COLOR=1&NEW_DESIGN=on&NEW_VIEW=yes&NUM_OVERVIEW=100&OLD_BLAST=false&PAGE=Proteins&QUERY_INDEX=0&QUERY_NUMBER=0&RESULTS_PAGE_TARGET=&RID=T277CMJ2014&SHOW_LINKOUT=yes&SHOW_OVERVIEW=yes&STEP_NUMBER=&WORD_SIZE=2&DISPLAY_SORT=1&HSP_SORT=1" \l "sort_mark)** | **[Total score](http://blast.ncbi.nlm.nih.gov/Blast.cgi?CMD=Get&ALIGNMENTS=100&ALIGNMENT_VIEW=Pairwise&CDD_SEARCH_STATE=1&DATABASE_SORT=0&DESCRIPTIONS=100&ENTREZ_QUERY=txid9606 %5BORGN%5D&FIRST_QUERY_NUM=0&FORMAT_OBJECT=Alignment&FORMAT_PAGE_TARGET=&FORMAT_TYPE=HTML&GET_SEQUENCE=yes&I_THRESH=&MASK_CHAR=2&MASK_COLOR=1&NEW_DESIGN=on&NEW_VIEW=yes&NUM_OVERVIEW=100&OLD_BLAST=false&PAGE=Proteins&QUERY_INDEX=0&QUERY_NUMBER=0&RESULTS_PAGE_TARGET=&RID=T277CMJ2014&SHOW_LINKOUT=yes&SHOW_OVERVIEW=yes&STEP_NUMBER=&WORD_SIZE=2&DISPLAY_SORT=2&HSP_SORT=1" \l "sort_mark)** | **[Query coverage](http://blast.ncbi.nlm.nih.gov/Blast.cgi?CMD=Get&ALIGNMENTS=100&ALIGNMENT_VIEW=Pairwise&CDD_SEARCH_STATE=1&DATABASE_SORT=0&DESCRIPTIONS=100&ENTREZ_QUERY=txid9606 %5BORGN%5D&FIRST_QUERY_NUM=0&FORMAT_OBJECT=Alignment&FORMAT_PAGE_TARGET=&FORMAT_TYPE=HTML&GET_SEQUENCE=yes&I_THRESH=&MASK_CHAR=2&MASK_COLOR=1&NEW_DESIGN=on&NEW_VIEW=yes&NUM_OVERVIEW=100&OLD_BLAST=false&PAGE=Proteins&QUERY_INDEX=0&QUERY_NUMBER=0&RESULTS_PAGE_TARGET=&RID=T277CMJ2014&SHOW_LINKOUT=yes&SHOW_OVERVIEW=yes&STEP_NUMBER=&WORD_SIZE=2&DISPLAY_SORT=4&HSP_SORT=0" \l "sort_mark)** | **[E value](http://blast.ncbi.nlm.nih.gov/Blast.cgi?CMD=Get&ALIGNMENTS=100&ALIGNMENT_VIEW=Pairwise&CDD_SEARCH_STATE=1&DATABASE_SORT=0&DESCRIPTIONS=100&ENTREZ_QUERY=txid9606 %5BORGN%5D&FIRST_QUERY_NUM=0&FORMAT_OBJECT=Alignment&FORMAT_PAGE_TARGET=&FORMAT_TYPE=HTML&GET_SEQUENCE=yes&I_THRESH=&MASK_CHAR=2&MASK_COLOR=1&NEW_DESIGN=on&NEW_VIEW=yes&NUM_OVERVIEW=100&OLD_BLAST=false&PAGE=Proteins&QUERY_INDEX=0&QUERY_NUMBER=0&RESULTS_PAGE_TARGET=&RID=T277CMJ2014&SHOW_LINKOUT=yes&SHOW_OVERVIEW=yes&STEP_NUMBER=&WORD_SIZE=2&DISPLAY_SORT=0&HSP_SORT=0" \l "sort_mark)** |
| --- | --- | --- | --- | --- | --- |
| [NP_001154912.1](http://www.ncbi.nlm.nih.gov/entrez/query.fcgi?cmd=Retrieve&db=Protein&list_uids=241896926&dopt=GenPept&RID=T277CMJ2014&log$=prottop&blast_rank=1) | protein tyrosine phosphatase, receptor type, H isoform 2 precursor [Homo sapiens] | [24.4](http://blast.ncbi.nlm.nih.gov/Blast.cgi#241896926%23241896926) | 24.4 | 100% | 7.9 |
| [NP_002833.3](http://www.ncbi.nlm.nih.gov/entrez/query.fcgi?cmd=Retrieve&db=Protein&list_uids=241896924&dopt=GenPept&RID=T277CMJ2014&log$=prottop&blast_rank=2) | protein tyrosine phosphatase, receptor type, H isoform 1 precursor [Homo sapiens] | [24.4](http://blast.ncbi.nlm.nih.gov/Blast.cgi#241896924%23241896924) | 24.4 | 100% | 7.9 |
| [NP_059117.3](http://www.ncbi.nlm.nih.gov/entrez/query.fcgi?cmd=Retrieve&db=Protein&list_uids=118600965&dopt=GenPept&RID=T277CMJ2014&log$=prottop&blast_rank=3) | hexaprenyldihydroxybenzoate methyltransferase [Homo sapiens] | [24.0](http://blast.ncbi.nlm.nih.gov/Blast.cgi#118600965%23118600965) | 24.0 | 80% | 11 |
| [NP_060213.2](http://www.ncbi.nlm.nih.gov/entrez/query.fcgi?cmd=Retrieve&db=Protein&list_uids=18450278&dopt=GenPept&RID=T277CMJ2014&log$=prottop&blast_rank=4) | dipeptidyl peptidase 8 isoform 2 [Homo sapiens] | [23.1](http://blast.ncbi.nlm.nih.gov/Blast.cgi#18450278%2318450278) | 37.3 | 80% | 19 |
| [NP_932065.1](http://www.ncbi.nlm.nih.gov/entrez/query.fcgi?cmd=Retrieve&db=Protein&list_uids=37577091&dopt=GenPept&RID=T277CMJ2014&log$=prottop&blast_rank=5) | dipeptidyl peptidase 8 isoform 4 [Homo sapiens] | [23.1](http://blast.ncbi.nlm.nih.gov/Blast.cgi#37577091%2337577091) | 37.3 | 80% | 19 |
| [NP_569118.1](http://www.ncbi.nlm.nih.gov/entrez/query.fcgi?cmd=Retrieve&db=Protein&list_uids=18450280&dopt=GenPept&RID=T277CMJ2014&log$=prottop&blast_rank=6) | dipeptidyl peptidase 8 isoform 1 [Homo sapiens] | [23.1](http://blast.ncbi.nlm.nih.gov/Blast.cgi#18450280%2318450280) | 53.2 | 90% | 19 |
| [NP_932064.1](http://www.ncbi.nlm.nih.gov/entrez/query.fcgi?cmd=Retrieve&db=Protein&list_uids=37577089&dopt=GenPept&RID=T277CMJ2014&log$=prottop&blast_rank=7) | dipeptidyl peptidase 8 isoform 3 [Homo sapiens] | [23.1](http://blast.ncbi.nlm.nih.gov/Blast.cgi#37577089%2337577089) | 53.2 | 90% | 19 |
| [NP_057339.1](http://www.ncbi.nlm.nih.gov/entrez/query.fcgi?cmd=Retrieve&db=Protein&list_uids=7705268&dopt=GenPept&RID=T277CMJ2014&log$=prottop&blast_rank=8) | family with sequence similarity 8, member A1 [Homo sapiens] | [22.7](http://blast.ncbi.nlm.nih.gov/Blast.cgi#7705268%237705268) | 33.5 | 90% | 26 |
| [NP_446464.1](http://www.ncbi.nlm.nih.gov/entrez/query.fcgi?cmd=Retrieve&db=Protein&list_uids=16950633&dopt=GenPept&RID=T277CMJ2014&log$=prottop&blast_rank=9) | argininosuccinate synthetase 1 [Homo sapiens] >ref|NP_000041.2| argininosuccinate synthetase 1 [Homo sapiens] | [22.7](http://blast.ncbi.nlm.nih.gov/Blast.cgi#16950633%2316950633) | 22.7 | 70% | 26 |
| [NP_892003.2](http://www.ncbi.nlm.nih.gov/entrez/query.fcgi?cmd=Retrieve&db=Protein&list_uids=226371638&dopt=GenPept&RID=T277CMJ2014&log$=prottop&blast_rank=10) | MYST histone acetyltransferase 1 isoform 2 [Homo sapiens] | [22.3](http://blast.ncbi.nlm.nih.gov/Blast.cgi#226371638%23226371638) | 22.3 | 100% | 34 |
| [NP_115683.3](http://www.ncbi.nlm.nih.gov/entrez/query.fcgi?cmd=Retrieve&db=Protein&list_uids=110815802&dopt=GenPept&RID=T277CMJ2014&log$=prottop&blast_rank=11) | hypothetical protein LOC84267 [Homo sapiens] | [22.3](http://blast.ncbi.nlm.nih.gov/Blast.cgi#110815802%23110815802) | 22.3 | 50% | 34 |
| [NP_115564.2](http://www.ncbi.nlm.nih.gov/entrez/query.fcgi?cmd=Retrieve&db=Protein&list_uids=226371636&dopt=GenPept&RID=T277CMJ2014&log$=prottop&blast_rank=12) | MYST histone acetyltransferase 1 isoform 1 [Homo sapiens] | [22.3](http://blast.ncbi.nlm.nih.gov/Blast.cgi#226371636%23226371636) | 22.3 | 100% | 34 |
| [XP_002342991.1](http://www.ncbi.nlm.nih.gov/entrez/query.fcgi?cmd=Retrieve&db=Protein&list_uids=239743922&dopt=GenPept&RID=T277CMJ2014&log$=prottop&blast_rank=13) | PREDICTED: hypothetical protein XP_002342991 [Homo sapiens] >ref|XP_002347137.1| PREDICTED: hypothetical protein [Homo sapiens] >ref|XP_002346229.1| PREDICTED: hypothetical protein [Homo sapiens] | [21.8](http://blast.ncbi.nlm.nih.gov/Blast.cgi#239743922%23239743922) | 21.8 | 70% | 46 |
| [NP_001028895.1](http://www.ncbi.nlm.nih.gov/entrez/query.fcgi?cmd=Retrieve&db=Protein&list_uids=76253910&dopt=GenPept&RID=T277CMJ2014&log$=prottop&blast_rank=14) | zinc finger protein 704 [Homo sapiens] | [21.8](http://blast.ncbi.nlm.nih.gov/Blast.cgi#76253910%2376253910) | 21.8 | 70% | 46 |
| [NP_004056.2](http://www.ncbi.nlm.nih.gov/entrez/query.fcgi?cmd=Retrieve&db=Protein&list_uids=44889483&dopt=GenPept&RID=T277CMJ2014&log$=prottop&blast_rank=15) | cerebellar degeneration-related protein 1 [Homo sapiens] | [21.8](http://blast.ncbi.nlm.nih.gov/Blast.cgi#44889483%2344889483) | 53.7 | 70% | 46 |
| [NP_001127874.1](http://www.ncbi.nlm.nih.gov/entrez/query.fcgi?cmd=Retrieve&db=Protein&list_uids=197304729&dopt=GenPept&RID=T277CMJ2014&log$=prottop&blast_rank=16) | cytochrome b-561 domain containing 1 isoform 3 [Homo sapiens] | [21.4](http://blast.ncbi.nlm.nih.gov/Blast.cgi#197304729%23197304729) | 21.4 | 50% | 62 |
| [NP_001127872.1](http://www.ncbi.nlm.nih.gov/entrez/query.fcgi?cmd=Retrieve&db=Protein&list_uids=197304721&dopt=GenPept&RID=T277CMJ2014&log$=prottop&blast_rank=17) | cytochrome b-561 domain containing 1 isoform 1 [Homo sapiens] | [21.4](http://blast.ncbi.nlm.nih.gov/Blast.cgi#197304721%23197304721) | 21.4 | 50% | 62 |
| [NP_872386.1](http://www.ncbi.nlm.nih.gov/entrez/query.fcgi?cmd=Retrieve&db=Protein&list_uids=32698982&dopt=GenPept&RID=T277CMJ2014&log$=prottop&blast_rank=18) | cytochrome b-561 domain containing 1 isoform 2 [Homo sapiens] | [21.4](http://blast.ncbi.nlm.nih.gov/Blast.cgi#32698982%2332698982) | 21.4 | 50% | 62 |
| [NP_114096.1](http://www.ncbi.nlm.nih.gov/entrez/query.fcgi?cmd=Retrieve&db=Protein&list_uids=13994236&dopt=GenPept&RID=T277CMJ2014&log$=prottop&blast_rank=19) | cat eye syndrome chromosome region, candidate 6 isoform a [Homo sapiens] | [21.4](http://blast.ncbi.nlm.nih.gov/Blast.cgi#13994236%2313994236) | 21.4 | 60% | 62 |
| [NP_076431.1](http://www.ncbi.nlm.nih.gov/entrez/query.fcgi?cmd=Retrieve&db=Protein&list_uids=13027616&dopt=GenPept&RID=T277CMJ2014&log$=prottop&blast_rank=20) | leucine rich repeat containing 61 [Homo sapiens] >ref|NP_001136400.1| leucine rich repeat containing 61 [Homo sapiens] | [21.4](http://blast.ncbi.nlm.nih.gov/Blast.cgi#13027616%2313027616) | 21.4 | 60% | 62 |
| [NP_001009994.1](http://www.ncbi.nlm.nih.gov/entrez/query.fcgi?cmd=Retrieve&db=Protein&list_uids=58000465&dopt=GenPept&RID=T277CMJ2014&log$=prottop&blast_rank=21) | ripply2 protein [Homo sapiens] | [21.0](http://blast.ncbi.nlm.nih.gov/Blast.cgi#58000465%2358000465) | 21.0 | 90% | 83 |
| [NP_000104.1](http://www.ncbi.nlm.nih.gov/entrez/query.fcgi?cmd=Retrieve&db=Protein&list_uids=4557541&dopt=GenPept&RID=T277CMJ2014&log$=prottop&blast_rank=22) | torsin A precursor [Homo sapiens] | [21.0](http://blast.ncbi.nlm.nih.gov/Blast.cgi#4557541%234557541) | 21.0 | 60% | 83 |
| [XP_002343693.1](http://www.ncbi.nlm.nih.gov/entrez/query.fcgi?cmd=Retrieve&db=Protein&list_uids=239746315&dopt=GenPept&RID=T277CMJ2014&log$=prottop&blast_rank=23) | PREDICTED: hypothetical protein XP_002343693 [Homo sapiens] >ref|XP_002347994.1| PREDICTED: hypothetical protein [Homo sapiens] | [20.6](http://blast.ncbi.nlm.nih.gov/Blast.cgi#239746315%23239746315) | 20.6 | 40% | 111 |
| [NP_077729.1](http://www.ncbi.nlm.nih.gov/entrez/query.fcgi?cmd=Retrieve&db=Protein&list_uids=171906567&dopt=GenPept&RID=T277CMJ2014&log$=prottop&blast_rank=24) | peripheral benzodiazepine receptor-associated protein 1 isoform b [Homo sapiens] | [20.6](http://blast.ncbi.nlm.nih.gov/Blast.cgi#171906567%23171906567) | 20.6 | 40% | 111 |
| [NP_004749.2](http://www.ncbi.nlm.nih.gov/entrez/query.fcgi?cmd=Retrieve&db=Protein&list_uids=171906559&dopt=GenPept&RID=T277CMJ2014&log$=prottop&blast_rank=25) | peripheral benzodiazepine receptor-associated protein 1 isoform a [Homo sapiens] | [20.6](http://blast.ncbi.nlm.nih.gov/Blast.cgi#171906559%23171906559) | 20.6 | 40% | 111 |
| [NP_001012331.1](http://www.ncbi.nlm.nih.gov/entrez/query.fcgi?cmd=Retrieve&db=Protein&list_uids=59889558&dopt=GenPept&RID=T277CMJ2014&log$=prottop&blast_rank=26) | neurotrophic tyrosine kinase, receptor, type 1 isoform 1 [Homo sapiens] | [20.6](http://blast.ncbi.nlm.nih.gov/Blast.cgi#59889558%2359889558) | 20.6 | 40% | 111 |
| [NP_002520.2](http://www.ncbi.nlm.nih.gov/entrez/query.fcgi?cmd=Retrieve&db=Protein&list_uids=4585712&dopt=GenPept&RID=T277CMJ2014&log$=prottop&blast_rank=27) | neurotrophic tyrosine kinase, receptor, type 1 isoform 2 [Homo sapiens] | [20.6](http://blast.ncbi.nlm.nih.gov/Blast.cgi#4585712%234585712) | 20.6 | 40% | 111 |
| [NP_114141.2](http://www.ncbi.nlm.nih.gov/entrez/query.fcgi?cmd=Retrieve&db=Protein&list_uids=118572606&dopt=GenPept&RID=T277CMJ2014&log$=prottop&blast_rank=28) | hemicentin 1 precursor [Homo sapiens] | [20.6](http://blast.ncbi.nlm.nih.gov/Blast.cgi#118572606%23118572606) | 34.4 | 40% | 111 |
| [NP_000604.1](http://www.ncbi.nlm.nih.gov/entrez/query.fcgi?cmd=Retrieve&db=Protein&list_uids=11321561&dopt=GenPept&RID=T277CMJ2014&log$=prottop&blast_rank=29) | hemopexin precursor [Homo sapiens] | [20.6](http://blast.ncbi.nlm.nih.gov/Blast.cgi#11321561%2311321561) | 20.6 | 40% | 111 |
| [NP_065683.2](http://www.ncbi.nlm.nih.gov/entrez/query.fcgi?cmd=Retrieve&db=Protein&list_uids=85386053&dopt=GenPept&RID=T277CMJ2014&log$=prottop&blast_rank=30) | ATPase, H+ transporting, lysosomal V0 subunit a4 [Homo sapiens] >ref|NP_570855.2| ATPase, H+ transporting, lysosomal V0 subunit a4 [Homo sapiens] >ref|NP_570856.2| ATPase, H+ transporting, lysosomal V0 subunit a4 [Homo sapiens] | [20.6](http://blast.ncbi.nlm.nih.gov/Blast.cgi#85386053%2385386053) | 20.6 | 40% | 111 |
| [NP_001164408.1](http://www.ncbi.nlm.nih.gov/entrez/query.fcgi?cmd=Retrieve&db=Protein&list_uids=283135173&dopt=GenPept&RID=T277CMJ2014&log$=prottop&blast_rank=31) | fused in sarcoma isoform 3 [Homo sapiens] | [20.2](http://blast.ncbi.nlm.nih.gov/Blast.cgi#283135173%23283135173) | 36.1 | 80% | 149 |
| [XP_002347522.1](http://www.ncbi.nlm.nih.gov/entrez/query.fcgi?cmd=Retrieve&db=Protein&list_uids=239750743&dopt=GenPept&RID=T277CMJ2014&log$=prottop&blast_rank=32) | PREDICTED: hypothetical protein XP_002347522 [Homo sapiens] | [20.2](http://blast.ncbi.nlm.nih.gov/Blast.cgi#239750743%23239750743) | 20.2 | 90% | 149 |
| [NP_001093392.1](http://www.ncbi.nlm.nih.gov/entrez/query.fcgi?cmd=Retrieve&db=Protein&list_uids=153791910&dopt=GenPept&RID=T277CMJ2014&log$=prottop&blast_rank=33) | asparagine-linked glycosylation 13 homolog isoform 1 [Homo sapiens] | [20.2](http://blast.ncbi.nlm.nih.gov/Blast.cgi#153791910%23153791910) | 20.2 | 70% | 149 |
| [NP_005375.2](http://www.ncbi.nlm.nih.gov/entrez/query.fcgi?cmd=Retrieve&db=Protein&list_uids=52630429&dopt=GenPept&RID=T277CMJ2014&log$=prottop&blast_rank=34) | nuclear factor, interleukin 3 regulated [Homo sapiens] | [20.2](http://blast.ncbi.nlm.nih.gov/Blast.cgi#52630429%2352630429) | 20.2 | 80% | 149 |
| [NP_001164105.1](http://www.ncbi.nlm.nih.gov/entrez/query.fcgi?cmd=Retrieve&db=Protein&list_uids=283135201&dopt=GenPept&RID=T277CMJ2014&log$=prottop&blast_rank=35) | fused in sarcoma isoform 2 [Homo sapiens] | [20.2](http://blast.ncbi.nlm.nih.gov/Blast.cgi#283135201%23283135201) | 36.1 | 80% | 149 |
| [NP_004951.1](http://www.ncbi.nlm.nih.gov/entrez/query.fcgi?cmd=Retrieve&db=Protein&list_uids=4826734&dopt=GenPept&RID=T277CMJ2014&log$=prottop&blast_rank=36) | fused in sarcoma isoform 1 [Homo sapiens] | [20.2](http://blast.ncbi.nlm.nih.gov/Blast.cgi#4826734%234826734) | 36.1 | 80% | 149 |
| [NP_000780.1](http://www.ncbi.nlm.nih.gov/entrez/query.fcgi?cmd=Retrieve&db=Protein&list_uids=4503273&dopt=GenPept&RID=T277CMJ2014&log$=prottop&blast_rank=37) | angiotensin I converting enzyme 1 isoform 1 precursor [Homo sapiens] | [20.2](http://blast.ncbi.nlm.nih.gov/Blast.cgi#4503273%234503273) | 20.2 | 50% | 149 |
| [NP_640335.2](http://www.ncbi.nlm.nih.gov/entrez/query.fcgi?cmd=Retrieve&db=Protein&list_uids=164663775&dopt=GenPept&RID=T277CMJ2014&log$=prottop&blast_rank=38) | mitochondrial methionyl-tRNA formyltransferase precursor [Homo sapiens] | [20.2](http://blast.ncbi.nlm.nih.gov/Blast.cgi#164663775%23164663775) | 20.2 | 80% | 149 |
| [XP_001716109.2](http://www.ncbi.nlm.nih.gov/entrez/query.fcgi?cmd=Retrieve&db=Protein&list_uids=239744397&dopt=GenPept&RID=T277CMJ2014&log$=prottop&blast_rank=39) | PREDICTED: hypothetical protein LOC100130348 [Homo sapiens] | [19.7](http://blast.ncbi.nlm.nih.gov/Blast.cgi#239744397%23239744397) | 19.7 | 60% | 200 |
| [NP_001098714.1](http://www.ncbi.nlm.nih.gov/entrez/query.fcgi?cmd=Retrieve&db=Protein&list_uids=157419152&dopt=GenPept&RID=T277CMJ2014&log$=prottop&blast_rank=40) | protein tyrosine phosphatase, receptor type, M isoform 1 precursor [Homo sapiens] | [19.7](http://blast.ncbi.nlm.nih.gov/Blast.cgi#157419152%23157419152) | 30.5 | 50% | 200 |
| [NP_573400.3](http://www.ncbi.nlm.nih.gov/entrez/query.fcgi?cmd=Retrieve&db=Protein&list_uids=148539858&dopt=GenPept&RID=T277CMJ2014&log$=prottop&blast_rank=41) | protein tyrosine phosphatase, receptor type, T isoform 1 precursor [Homo sapiens] | [19.7](http://blast.ncbi.nlm.nih.gov/Blast.cgi#148539858%23148539858) | 19.7 | 50% | 200 |
| [NP_002836.3](http://www.ncbi.nlm.nih.gov/entrez/query.fcgi?cmd=Retrieve&db=Protein&list_uids=157419150&dopt=GenPept&RID=T277CMJ2014&log$=prottop&blast_rank=42) | protein tyrosine phosphatase, receptor type, M isoform 2 precursor [Homo sapiens] | [19.7](http://blast.ncbi.nlm.nih.gov/Blast.cgi#157419150%23157419150) | 30.5 | 50% | 200 |
| [NP_061161.2](http://www.ncbi.nlm.nih.gov/entrez/query.fcgi?cmd=Retrieve&db=Protein&list_uids=93102375&dopt=GenPept&RID=T277CMJ2014&log$=prottop&blast_rank=43) | hypothetical protein LOC10827 [Homo sapiens] | [19.7](http://blast.ncbi.nlm.nih.gov/Blast.cgi#93102375%2393102375) | 19.7 | 60% | 200 |
| [NP_008981.4](http://www.ncbi.nlm.nih.gov/entrez/query.fcgi?cmd=Retrieve&db=Protein&list_uids=148539860&dopt=GenPept&RID=T277CMJ2014&log$=prottop&blast_rank=44) | protein tyrosine phosphatase, receptor type, T isoform 2 precursor [Homo sapiens] | [19.7](http://blast.ncbi.nlm.nih.gov/Blast.cgi#148539860%23148539860) | 19.7 | 50% | 200 |
| [NP_002420.1](http://www.ncbi.nlm.nih.gov/entrez/query.fcgi?cmd=Retrieve&db=Protein&list_uids=4505213&dopt=GenPept&RID=T277CMJ2014&log$=prottop&blast_rank=45) | matrix metalloproteinase 19 isoform rasi-1 preproprotein [Homo sapiens] | [19.7](http://blast.ncbi.nlm.nih.gov/Blast.cgi#4505213%234505213) | 19.7 | 70% | 200 |
| [NP_001457.1](http://www.ncbi.nlm.nih.gov/entrez/query.fcgi?cmd=Retrieve&db=Protein&list_uids=4503827&dopt=GenPept&RID=T277CMJ2014&log$=prottop&blast_rank=46) | frizzled 2 precursor [Homo sapiens] | [19.7](http://blast.ncbi.nlm.nih.gov/Blast.cgi#4503827%234503827) | 19.7 | 70% | 200 |
| [NP_065685.1](http://www.ncbi.nlm.nih.gov/entrez/query.fcgi?cmd=Retrieve&db=Protein&list_uids=10190670&dopt=GenPept&RID=T277CMJ2014&log$=prottop&blast_rank=47) | growth differentiation factor 3 precursor [Homo sapiens] | [19.7](http://blast.ncbi.nlm.nih.gov/Blast.cgi#10190670%2310190670) | 19.7 | 50% | 200 |
| [NP_005529.1](http://www.ncbi.nlm.nih.gov/entrez/query.fcgi?cmd=Retrieve&db=Protein&list_uids=5031795&dopt=GenPept&RID=T277CMJ2014&log$=prottop&blast_rank=48) | inhibin beta C chain preproprotein [Homo sapiens] | [19.7](http://blast.ncbi.nlm.nih.gov/Blast.cgi#5031795%235031795) | 19.7 | 50% | 200 |
| [NP_001159591.1](http://www.ncbi.nlm.nih.gov/entrez/query.fcgi?cmd=Retrieve&db=Protein&list_uids=260656057&dopt=GenPept&RID=T277CMJ2014&log$=prottop&blast_rank=49) | lymphoid enhancer-binding factor 1 isoform 4 [Homo sapiens] | [19.3](http://blast.ncbi.nlm.nih.gov/Blast.cgi#260656057%23260656057) | 19.3 | 50% | 268 |
| [XP_002347556.1](http://www.ncbi.nlm.nih.gov/entrez/query.fcgi?cmd=Retrieve&db=Protein&list_uids=239747244&dopt=GenPept&RID=T277CMJ2014&log$=prottop&blast_rank=50) | PREDICTED: hypothetical protein [Homo sapiens] | [19.3](http://blast.ncbi.nlm.nih.gov/Blast.cgi#239747244%23239747244) | 19.3 | 40% | 268 |
| [XP_002342074.1](http://www.ncbi.nlm.nih.gov/entrez/query.fcgi?cmd=Retrieve&db=Protein&list_uids=239741065&dopt=GenPept&RID=T277CMJ2014&log$=prottop&blast_rank=51) | PREDICTED: hypothetical protein [Homo sapiens] | [19.3](http://blast.ncbi.nlm.nih.gov/Blast.cgi#239741065%23239741065) | 19.3 | 40% | 268 |
| [NP_001129175.1](http://www.ncbi.nlm.nih.gov/entrez/query.fcgi?cmd=Retrieve&db=Protein&list_uids=208973262&dopt=GenPept&RID=T277CMJ2014&log$=prottop&blast_rank=52) | low density lipoprotein-related protein 12 isoform b precursor [Homo sapiens] | [19.3](http://blast.ncbi.nlm.nih.gov/Blast.cgi#208973262%23208973262) | 19.3 | 40% | 268 |
| [NP_079389.2](http://www.ncbi.nlm.nih.gov/entrez/query.fcgi?cmd=Retrieve&db=Protein&list_uids=156151402&dopt=GenPept&RID=T277CMJ2014&log$=prottop&blast_rank=53) | hypothetical protein LOC80183 [Homo sapiens] | [19.3](http://blast.ncbi.nlm.nih.gov/Blast.cgi#156151402%23156151402) | 19.3 | 50% | 268 |
| [NP_997198.2](http://www.ncbi.nlm.nih.gov/entrez/query.fcgi?cmd=Retrieve&db=Protein&list_uids=117606370&dopt=GenPept&RID=T277CMJ2014&log$=prottop&blast_rank=54) | UMP-CMP kinase 2 precursor [Homo sapiens] | [19.3](http://blast.ncbi.nlm.nih.gov/Blast.cgi#117606370%23117606370) | 19.3 | 40% | 268 |
| [NP_055201.2](http://www.ncbi.nlm.nih.gov/entrez/query.fcgi?cmd=Retrieve&db=Protein&list_uids=116812588&dopt=GenPept&RID=T277CMJ2014&log$=prottop&blast_rank=55) | polycystic kidney disease 2-like 2 [Homo sapiens] | [19.3](http://blast.ncbi.nlm.nih.gov/Blast.cgi#116812588%23116812588) | 19.3 | 50% | 268 |
| [NP_001154180.1](http://www.ncbi.nlm.nih.gov/entrez/query.fcgi?cmd=Retrieve&db=Protein&list_uids=238908505&dopt=GenPept&RID=T277CMJ2014&log$=prottop&blast_rank=56) | DNA-directed RNA polymerase III B isoform 2 [Homo sapiens] | [19.3](http://blast.ncbi.nlm.nih.gov/Blast.cgi#238908505%23238908505) | 19.3 | 50% | 268 |
| [NP_079191.2](http://www.ncbi.nlm.nih.gov/entrez/query.fcgi?cmd=Retrieve&db=Protein&list_uids=170784817&dopt=GenPept&RID=T277CMJ2014&log$=prottop&blast_rank=57) | transcription factor CP2-like 3 [Homo sapiens] | [19.3](http://blast.ncbi.nlm.nih.gov/Blast.cgi#170784817%23170784817) | 19.3 | 40% | 268 |
| [NP_001157940.1](http://www.ncbi.nlm.nih.gov/entrez/query.fcgi?cmd=Retrieve&db=Protein&list_uids=256985100&dopt=GenPept&RID=T277CMJ2014&log$=prottop&blast_rank=58) | TRAM adaptor with GOLD domain isoform 1 [Homo sapiens] | [19.3](http://blast.ncbi.nlm.nih.gov/Blast.cgi#256985100%23256985100) | 19.3 | 40% | 268 |
| [NP_060879.2](http://www.ncbi.nlm.nih.gov/entrez/query.fcgi?cmd=Retrieve&db=Protein&list_uids=33620759&dopt=GenPept&RID=T277CMJ2014&log$=prottop&blast_rank=59) | LRP2 binding protein [Homo sapiens] | [19.3](http://blast.ncbi.nlm.nih.gov/Blast.cgi#33620759%2333620759) | 19.3 | 40% | 268 |
| [NP_150279.1](http://www.ncbi.nlm.nih.gov/entrez/query.fcgi?cmd=Retrieve&db=Protein&list_uids=15147240&dopt=GenPept&RID=T277CMJ2014&log$=prottop&blast_rank=60) | brother of CDO precursor [Homo sapiens] | [19.3](http://blast.ncbi.nlm.nih.gov/Blast.cgi#15147240%2315147240) | 32.7 | 50% | 268 |
| [NP_001007532.1](http://www.ncbi.nlm.nih.gov/entrez/query.fcgi?cmd=Retrieve&db=Protein&list_uids=56090620&dopt=GenPept&RID=T277CMJ2014&log$=prottop&blast_rank=61) | NFKB activating protein-like [Homo sapiens] | [19.3](http://blast.ncbi.nlm.nih.gov/Blast.cgi#56090620%2356090620) | 19.3 | 50% | 268 |
| [NP_001027563.1](http://www.ncbi.nlm.nih.gov/entrez/query.fcgi?cmd=Retrieve&db=Protein&list_uids=74048545&dopt=GenPept&RID=T277CMJ2014&log$=prottop&blast_rank=62) | leucine carboxyl methyltransferase 1 isoform b [Homo sapiens] | [19.3](http://blast.ncbi.nlm.nih.gov/Blast.cgi#74048545%2374048545) | 19.3 | 40% | 268 |
| [NP_001124186.1](http://www.ncbi.nlm.nih.gov/entrez/query.fcgi?cmd=Retrieve&db=Protein&list_uids=195222734&dopt=GenPept&RID=T277CMJ2014&log$=prottop&blast_rank=63) | lymphoid enhancer-binding factor 1 isoform 3 [Homo sapiens] | [19.3](http://blast.ncbi.nlm.nih.gov/Blast.cgi#195222734%23195222734) | 19.3 | 50% | 268 |
| [NP_001014364.1](http://www.ncbi.nlm.nih.gov/entrez/query.fcgi?cmd=Retrieve&db=Protein&list_uids=62122917&dopt=GenPept&RID=T277CMJ2014&log$=prottop&blast_rank=64) | filaggrin family member 2 [Homo sapiens] | [19.3](http://blast.ncbi.nlm.nih.gov/Blast.cgi#62122917%2362122917) | 34.4 | 90% | 268 |
| [NP_689547.2](http://www.ncbi.nlm.nih.gov/entrez/query.fcgi?cmd=Retrieve&db=Protein&list_uids=38570111&dopt=GenPept&RID=T277CMJ2014&log$=prottop&blast_rank=65) | threonyl-tRNA synthetase-like 2 [Homo sapiens] | [19.3](http://blast.ncbi.nlm.nih.gov/Blast.cgi#38570111%2338570111) | 32.2 | 60% | 268 |
| [NP_057191.2](http://www.ncbi.nlm.nih.gov/entrez/query.fcgi?cmd=Retrieve&db=Protein&list_uids=34101286&dopt=GenPept&RID=T277CMJ2014&log$=prottop&blast_rank=66) | zinc finger RNA binding protein [Homo sapiens] | [19.3](http://blast.ncbi.nlm.nih.gov/Blast.cgi#34101286%2334101286) | 19.3 | 50% | 268 |
| [NP_775099.2](http://www.ncbi.nlm.nih.gov/entrez/query.fcgi?cmd=Retrieve&db=Protein&list_uids=30795238&dopt=GenPept&RID=T277CMJ2014&log$=prottop&blast_rank=67) | ATP-binding cassette, sub-family A, member 12 isoform a [Homo sapiens] | [19.3](http://blast.ncbi.nlm.nih.gov/Blast.cgi#30795238%2330795238) | 19.3 | 50% | 268 |
| [NP_060823.3](http://www.ncbi.nlm.nih.gov/entrez/query.fcgi?cmd=Retrieve&db=Protein&list_uids=42415492&dopt=GenPept&RID=T277CMJ2014&log$=prottop&blast_rank=68) | chromosome 14 open reading frame 106 [Homo sapiens] | [19.3](http://blast.ncbi.nlm.nih.gov/Blast.cgi#42415492%2342415492) | 19.3 | 40% | 268 |
| [NP_060280.2](http://www.ncbi.nlm.nih.gov/entrez/query.fcgi?cmd=Retrieve&db=Protein&list_uids=32129212&dopt=GenPept&RID=T277CMJ2014&log$=prottop&blast_rank=69) | zinc finger protein 434 [Homo sapiens] | [19.3](http://blast.ncbi.nlm.nih.gov/Blast.cgi#32129212%2332129212) | 19.3 | 40% | 268 |
| [NP_060552.4](http://www.ncbi.nlm.nih.gov/entrez/query.fcgi?cmd=Retrieve&db=Protein&list_uids=238908503&dopt=GenPept&RID=T277CMJ2014&log$=prottop&blast_rank=70) | DNA-directed RNA polymerase III B isoform 1 [Homo sapiens] | [19.3](http://blast.ncbi.nlm.nih.gov/Blast.cgi#238908503%23238908503) | 19.3 | 50% | 268 |
| [NP_038465.1](http://www.ncbi.nlm.nih.gov/entrez/query.fcgi?cmd=Retrieve&db=Protein&list_uids=7305525&dopt=GenPept&RID=T277CMJ2014&log$=prottop&blast_rank=71) | low density lipoprotein-related protein 12 isoform a precursor [Homo sapiens] | [19.3](http://blast.ncbi.nlm.nih.gov/Blast.cgi#7305525%237305525) | 19.3 | 40% | 268 |
| [NP_003478.1](http://www.ncbi.nlm.nih.gov/entrez/query.fcgi?cmd=Retrieve&db=Protein&list_uids=4507353&dopt=GenPept&RID=T277CMJ2014&log$=prottop&blast_rank=72) | TBP-associated factor 15 isoform 2 [Homo sapiens] | [19.3](http://blast.ncbi.nlm.nih.gov/Blast.cgi#4507353%234507353) | 52.0 | 50% | 268 |
| [NP_057393.2](http://www.ncbi.nlm.nih.gov/entrez/query.fcgi?cmd=Retrieve&db=Protein&list_uids=74048434&dopt=GenPept&RID=T277CMJ2014&log$=prottop&blast_rank=73) | leucine carboxyl methyltransferase 1 isoform a [Homo sapiens] | [19.3](http://blast.ncbi.nlm.nih.gov/Blast.cgi#74048434%2374048434) | 19.3 | 40% | 268 |
| [NP_001973.2](http://www.ncbi.nlm.nih.gov/entrez/query.fcgi?cmd=Retrieve&db=Protein&list_uids=54792100&dopt=GenPept&RID=T277CMJ2014&log$=prottop&blast_rank=74) | erbB-3 isoform 1 precursor [Homo sapiens] | [19.3](http://blast.ncbi.nlm.nih.gov/Blast.cgi#54792100%2354792100) | 19.3 | 40% | 268 |
| [NP_006760.1](http://www.ncbi.nlm.nih.gov/entrez/query.fcgi?cmd=Retrieve&db=Protein&list_uids=5803072&dopt=GenPept&RID=T277CMJ2014&log$=prottop&blast_rank=75) | LIM domain only 4 [Homo sapiens] | [19.3](http://blast.ncbi.nlm.nih.gov/Blast.cgi#5803072%235803072) | 19.3 | 40% | 268 |
| [NP_689508.3](http://www.ncbi.nlm.nih.gov/entrez/query.fcgi?cmd=Retrieve&db=Protein&list_uids=38202255&dopt=GenPept&RID=T277CMJ2014&log$=prottop&blast_rank=76) | threonyl-tRNA synthetase [Homo sapiens] | [19.3](http://blast.ncbi.nlm.nih.gov/Blast.cgi#38202255%2338202255) | 32.2 | 60% | 268 |
| [NP_055006.1](http://www.ncbi.nlm.nih.gov/entrez/query.fcgi?cmd=Retrieve&db=Protein&list_uids=7657544&dopt=GenPept&RID=T277CMJ2014&log$=prottop&blast_rank=77) | sodium channel, voltage gated, type VIII, alpha [Homo sapiens] | [19.3](http://blast.ncbi.nlm.nih.gov/Blast.cgi#7657544%237657544) | 36.1 | 50% | 268 |
| [NP_631961.1](http://www.ncbi.nlm.nih.gov/entrez/query.fcgi?cmd=Retrieve&db=Protein&list_uids=21327701&dopt=GenPept&RID=T277CMJ2014&log$=prottop&blast_rank=78) | TBP-associated factor 15 isoform 1 [Homo sapiens] | [19.3](http://blast.ncbi.nlm.nih.gov/Blast.cgi#21327701%2321327701) | 52.0 | 50% | 268 |
| [NP_057353.1](http://www.ncbi.nlm.nih.gov/entrez/query.fcgi?cmd=Retrieve&db=Protein&list_uids=7705917&dopt=GenPept&RID=T277CMJ2014&log$=prottop&blast_rank=79) | lymphoid enhancer-binding factor 1 isoform 1 [Homo sapiens] | [19.3](http://blast.ncbi.nlm.nih.gov/Blast.cgi#7705917%237705917) | 19.3 | 50% | 268 |
| [NP_001124185.1](http://www.ncbi.nlm.nih.gov/entrez/query.fcgi?cmd=Retrieve&db=Protein&list_uids=195222732&dopt=GenPept&RID=T277CMJ2014&log$=prottop&blast_rank=80) | lymphoid enhancer-binding factor 1 isoform 2 [Homo sapiens] | [19.3](http://blast.ncbi.nlm.nih.gov/Blast.cgi#195222732%23195222732) | 19.3 | 50% | 268 |
| [NP_937825.2](http://www.ncbi.nlm.nih.gov/entrez/query.fcgi?cmd=Retrieve&db=Protein&list_uids=239735569&dopt=GenPept&RID=T277CMJ2014&log$=prottop&blast_rank=81) | grainyhead-like 1 [Homo sapiens] | [19.3](http://blast.ncbi.nlm.nih.gov/Blast.cgi#239735569%23239735569) | 19.3 | 40% | 268 |
| [NP_055794.1](http://www.ncbi.nlm.nih.gov/entrez/query.fcgi?cmd=Retrieve&db=Protein&list_uids=73695465&dopt=GenPept&RID=T277CMJ2014&log$=prottop&blast_rank=82) | synaptic vesicle glycoprotein 2C [Homo sapiens] | [18.9](http://blast.ncbi.nlm.nih.gov/Blast.cgi#73695465%2373695465) | 18.9 | 50% | 360 |
| [NP_776246.1](http://www.ncbi.nlm.nih.gov/entrez/query.fcgi?cmd=Retrieve&db=Protein&list_uids=27777653&dopt=GenPept&RID=T277CMJ2014&log$=prottop&blast_rank=83) | sperm acrosome associated 3 [Homo sapiens] | [18.9](http://blast.ncbi.nlm.nih.gov/Blast.cgi#27777653%2327777653) | 18.9 | 60% | 360 |
| [NP_006062.1](http://www.ncbi.nlm.nih.gov/entrez/query.fcgi?cmd=Retrieve&db=Protein&list_uids=5174633&dopt=GenPept&RID=T277CMJ2014&log$=prottop&blast_rank=84) | receptor for egg jelly-like protein precursor [Homo sapiens] | [18.9](http://blast.ncbi.nlm.nih.gov/Blast.cgi#5174633%235174633) | 18.9 | 50% | 360 |
| [NP_079435.1](http://www.ncbi.nlm.nih.gov/entrez/query.fcgi?cmd=Retrieve&db=Protein&list_uids=13376759&dopt=GenPept&RID=T277CMJ2014&log$=prottop&blast_rank=85) | hypothetical protein LOC80231 [Homo sapiens] | [18.9](http://blast.ncbi.nlm.nih.gov/Blast.cgi#13376759%2313376759) | 18.9 | 50% | 360 |
| [XP_002345236.1](http://www.ncbi.nlm.nih.gov/entrez/query.fcgi?cmd=Retrieve&db=Protein&list_uids=239757289&dopt=GenPept&RID=T277CMJ2014&log$=prottop&blast_rank=86) | PREDICTED: hypothetical protein [Homo sapiens] | [18.5](http://blast.ncbi.nlm.nih.gov/Blast.cgi#239757289%23239757289) | 18.5 | 50% | 482 |
| [XP_001721426.2](http://www.ncbi.nlm.nih.gov/entrez/query.fcgi?cmd=Retrieve&db=Protein&list_uids=239755577&dopt=GenPept&RID=T277CMJ2014&log$=prottop&blast_rank=87) | PREDICTED: similar to unc-93 homolog B1 (C. elegans) [Homo sapiens] | [18.5](http://blast.ncbi.nlm.nih.gov/Blast.cgi#239755577%23239755577) | 18.5 | 40% | 482 |
| [XP_001722389.2](http://www.ncbi.nlm.nih.gov/entrez/query.fcgi?cmd=Retrieve&db=Protein&list_uids=239750109&dopt=GenPept&RID=T277CMJ2014&log$=prottop&blast_rank=88) | PREDICTED: similar to unc-93 homolog B1 (C. elegans) [Homo sapiens] | [18.5](http://blast.ncbi.nlm.nih.gov/Blast.cgi#239750109%23239750109) | 18.5 | 40% | 482 |
| [XP_002343924.1](http://www.ncbi.nlm.nih.gov/entrez/query.fcgi?cmd=Retrieve&db=Protein&list_uids=239747143&dopt=GenPept&RID=T277CMJ2014&log$=prottop&blast_rank=89) | PREDICTED: hypothetical protein XP_002343924 [Homo sapiens] | [18.5](http://blast.ncbi.nlm.nih.gov/Blast.cgi#239747143%23239747143) | 18.5 | 40% | 482 |
| [NP_055919.2](http://www.ncbi.nlm.nih.gov/entrez/query.fcgi?cmd=Retrieve&db=Protein&list_uids=239047271&dopt=GenPept&RID=T277CMJ2014&log$=prottop&blast_rank=90) | autophagy related 2A [Homo sapiens] | [18.5](http://blast.ncbi.nlm.nih.gov/Blast.cgi#239047271%23239047271) | 18.5 | 50% | 482 |
| [NP_000251.3](http://www.ncbi.nlm.nih.gov/entrez/query.fcgi?cmd=Retrieve&db=Protein&list_uids=189083798&dopt=GenPept&RID=T277CMJ2014&log$=prottop&blast_rank=91) | myosin VIIA isoform 1 [Homo sapiens] | [18.5](http://blast.ncbi.nlm.nih.gov/Blast.cgi#189083798%23189083798) | 18.5 | 40% | 482 |
| [XP_001717326.1](http://www.ncbi.nlm.nih.gov/entrez/query.fcgi?cmd=Retrieve&db=Protein&list_uids=169173013&dopt=GenPept&RID=T277CMJ2014&log$=prottop&blast_rank=92) | PREDICTED: similar to hCG2008575 [Homo sapiens] | [18.5](http://blast.ncbi.nlm.nih.gov/Blast.cgi#169173013%23169173013) | 18.5 | 50% | 482 |
| [XP_001717243.1](http://www.ncbi.nlm.nih.gov/entrez/query.fcgi?cmd=Retrieve&db=Protein&list_uids=169172616&dopt=GenPept&RID=T277CMJ2014&log$=prottop&blast_rank=93) | PREDICTED: similar to hCG2008575 [Homo sapiens] | [18.5](http://blast.ncbi.nlm.nih.gov/Blast.cgi#169172616%23169172616) | 18.5 | 50% | 482 |
| [NP_001138998.1](http://www.ncbi.nlm.nih.gov/entrez/query.fcgi?cmd=Retrieve&db=Protein&list_uids=224465228&dopt=GenPept&RID=T277CMJ2014&log$=prottop&blast_rank=94) | arylformamidase isoform 2 [Homo sapiens] | [18.5](http://blast.ncbi.nlm.nih.gov/Blast.cgi#224465228%23224465228) | 18.5 | 50% | 482 |
| [NP_001120651.2](http://www.ncbi.nlm.nih.gov/entrez/query.fcgi?cmd=Retrieve&db=Protein&list_uids=256355179&dopt=GenPept&RID=T277CMJ2014&log$=prottop&blast_rank=95) | myosin VIIA isoform 3 [Homo sapiens] | [18.5](http://blast.ncbi.nlm.nih.gov/Blast.cgi#256355179%23256355179) | 18.5 | 40% | 482 |
| [NP_877435.2](http://www.ncbi.nlm.nih.gov/entrez/query.fcgi?cmd=Retrieve&db=Protein&list_uids=157674354&dopt=GenPept&RID=T277CMJ2014&log$=prottop&blast_rank=96) | WD repeat domain 72 [Homo sapiens] | [18.5](http://blast.ncbi.nlm.nih.gov/Blast.cgi#157674354%23157674354) | 18.5 | 40% | 482 |
| [NP_001120652.1](http://www.ncbi.nlm.nih.gov/entrez/query.fcgi?cmd=Retrieve&db=Protein&list_uids=189083802&dopt=GenPept&RID=T277CMJ2014&log$=prottop&blast_rank=97) | myosin VIIA isoform 2 [Homo sapiens] | [18.5](http://blast.ncbi.nlm.nih.gov/Blast.cgi#189083802%23189083802) | 18.5 | 40% | 482 |
| [NP_001010915.2](http://www.ncbi.nlm.nih.gov/entrez/query.fcgi?cmd=Retrieve&db=Protein&list_uids=148226324&dopt=GenPept&RID=T277CMJ2014&log$=prottop&blast_rank=98) | protein tyrosine phosphatase-like A domain containing 2 [Homo sapiens] | [18.5](http://blast.ncbi.nlm.nih.gov/Blast.cgi#148226324%23148226324) | 18.5 | 40% | 482 |
| [NP_001010875.1](http://www.ncbi.nlm.nih.gov/entrez/query.fcgi?cmd=Retrieve&db=Protein&list_uids=58197562&dopt=GenPept&RID=T277CMJ2014&log$=prottop&blast_rank=99) | solute carrier family 25, member 30 [Homo sapiens] | [18.5](http://blast.ncbi.nlm.nih.gov/Blast.cgi#58197562%2358197562) | 18.5 | 80% | 482 |
| [NP_056269.1](http://www.ncbi.nlm.nih.gov/entrez/query.fcgi?cmd=Retrieve&db=Protein&list_uids=109809739&dopt=GenPept&RID=T277CMJ2014&log$=prottop&blast_rank=100) | La ribonucleoprotein domain family, member 7 [Homo sapiens] >ref|NP_057732.2| La ribonucleoprotein domain family, member 7 [Homo sapiens] | [18.5](http://blast.ncbi.nlm.nih.gov/Blast.cgi#109809739%23109809739) | 18.5 | 40% | 482 |

| **Accession** | **Proteins with a match to FGAKSHGHHR peptide** | **[Max score](http://blast.ncbi.nlm.nih.gov/Blast.cgi?CMD=Get&ALIGNMENTS=100&ALIGNMENT_VIEW=Pairwise&CDD_SEARCH_STATE=1&DATABASE_SORT=0&DESCRIPTIONS=100&ENTREZ_QUERY=txid9606 %5BORGN%5D&FIRST_QUERY_NUM=0&FORMAT_OBJECT=Alignment&FORMAT_PAGE_TARGET=&FORMAT_TYPE=HTML&GET_SEQUENCE=yes&I_THRESH=&MASK_CHAR=2&MASK_COLOR=1&NEW_DESIGN=on&NEW_VIEW=yes&NUM_OVERVIEW=100&OLD_BLAST=false&PAGE=Proteins&QUERY_INDEX=0&QUERY_NUMBER=0&RESULTS_PAGE_TARGET=&RID=T27S4NHB012&SHOW_LINKOUT=yes&SHOW_OVERVIEW=yes&STEP_NUMBER=&WORD_SIZE=2&DISPLAY_SORT=1&HSP_SORT=1" \l "sort_mark)** | **[Total score](http://blast.ncbi.nlm.nih.gov/Blast.cgi?CMD=Get&ALIGNMENTS=100&ALIGNMENT_VIEW=Pairwise&CDD_SEARCH_STATE=1&DATABASE_SORT=0&DESCRIPTIONS=100&ENTREZ_QUERY=txid9606 %5BORGN%5D&FIRST_QUERY_NUM=0&FORMAT_OBJECT=Alignment&FORMAT_PAGE_TARGET=&FORMAT_TYPE=HTML&GET_SEQUENCE=yes&I_THRESH=&MASK_CHAR=2&MASK_COLOR=1&NEW_DESIGN=on&NEW_VIEW=yes&NUM_OVERVIEW=100&OLD_BLAST=false&PAGE=Proteins&QUERY_INDEX=0&QUERY_NUMBER=0&RESULTS_PAGE_TARGET=&RID=T27S4NHB012&SHOW_LINKOUT=yes&SHOW_OVERVIEW=yes&STEP_NUMBER=&WORD_SIZE=2&DISPLAY_SORT=2&HSP_SORT=1" \l "sort_mark)** | **[Query coverage](http://blast.ncbi.nlm.nih.gov/Blast.cgi?CMD=Get&ALIGNMENTS=100&ALIGNMENT_VIEW=Pairwise&CDD_SEARCH_STATE=1&DATABASE_SORT=0&DESCRIPTIONS=100&ENTREZ_QUERY=txid9606 %5BORGN%5D&FIRST_QUERY_NUM=0&FORMAT_OBJECT=Alignment&FORMAT_PAGE_TARGET=&FORMAT_TYPE=HTML&GET_SEQUENCE=yes&I_THRESH=&MASK_CHAR=2&MASK_COLOR=1&NEW_DESIGN=on&NEW_VIEW=yes&NUM_OVERVIEW=100&OLD_BLAST=false&PAGE=Proteins&QUERY_INDEX=0&QUERY_NUMBER=0&RESULTS_PAGE_TARGET=&RID=T27S4NHB012&SHOW_LINKOUT=yes&SHOW_OVERVIEW=yes&STEP_NUMBER=&WORD_SIZE=2&DISPLAY_SORT=4&HSP_SORT=0" \l "sort_mark)** | **[E value](http://blast.ncbi.nlm.nih.gov/Blast.cgi?CMD=Get&ALIGNMENTS=100&ALIGNMENT_VIEW=Pairwise&CDD_SEARCH_STATE=1&DATABASE_SORT=0&DESCRIPTIONS=100&ENTREZ_QUERY=txid9606 %5BORGN%5D&FIRST_QUERY_NUM=0&FORMAT_OBJECT=Alignment&FORMAT_PAGE_TARGET=&FORMAT_TYPE=HTML&GET_SEQUENCE=yes&I_THRESH=&MASK_CHAR=2&MASK_COLOR=1&NEW_DESIGN=on&NEW_VIEW=yes&NUM_OVERVIEW=100&OLD_BLAST=false&PAGE=Proteins&QUERY_INDEX=0&QUERY_NUMBER=0&RESULTS_PAGE_TARGET=&RID=T27S4NHB012&SHOW_LINKOUT=yes&SHOW_OVERVIEW=yes&STEP_NUMBER=&WORD_SIZE=2&DISPLAY_SORT=0&HSP_SORT=0" \l "sort_mark)** |
| --- | --- | --- | --- | --- | --- |
| [NP_001001662.1](http://www.ncbi.nlm.nih.gov/entrez/query.fcgi?cmd=Retrieve&db=Protein&list_uids=48717244&dopt=GenPept&RID=T27S4NHB012&log$=prottop&blast_rank=1) | zinc finger protein 782 [Homo sapiens] | [22.7](http://blast.ncbi.nlm.nih.gov/Blast.cgi#48717244%2348717244) | 22.7 | 100% | 26 |
| [NP_060699.3](http://www.ncbi.nlm.nih.gov/entrez/query.fcgi?cmd=Retrieve&db=Protein&list_uids=224451116&dopt=GenPept&RID=T27S4NHB012&log$=prottop&blast_rank=2) | Mu-2 related death-inducing protein [Homo sapiens] | [21.4](http://blast.ncbi.nlm.nih.gov/Blast.cgi#224451116%23224451116) | 21.4 | 60% | 62 |
| [NP_065908.1](http://www.ncbi.nlm.nih.gov/entrez/query.fcgi?cmd=Retrieve&db=Protein&list_uids=17978485&dopt=GenPept&RID=T27S4NHB012&log$=prottop&blast_rank=3) | vacuolar protein sorting 18 [Homo sapiens] | [20.6](http://blast.ncbi.nlm.nih.gov/Blast.cgi#17978485%2317978485) | 20.6 | 50% | 111 |
| [NP_116253.2](http://www.ncbi.nlm.nih.gov/entrez/query.fcgi?cmd=Retrieve&db=Protein&list_uids=24762236&dopt=GenPept&RID=T27S4NHB012&log$=prottop&blast_rank=4) | PRP38 pre-mRNA processing factor 38 (yeast) domain containing A [Homo sapiens] | [20.6](http://blast.ncbi.nlm.nih.gov/Blast.cgi#24762236%2324762236) | 31.0 | 70% | 111 |
| [NP_065798.2](http://www.ncbi.nlm.nih.gov/entrez/query.fcgi?cmd=Retrieve&db=Protein&list_uids=153945755&dopt=GenPept&RID=T27S4NHB012&log$=prottop&blast_rank=5) | zinc finger protein 608 [Homo sapiens] | [20.2](http://blast.ncbi.nlm.nih.gov/Blast.cgi#153945755%23153945755) | 20.2 | 70% | 149 |
| [NP_054790.2](http://www.ncbi.nlm.nih.gov/entrez/query.fcgi?cmd=Retrieve&db=Protein&list_uids=32307128&dopt=GenPept&RID=T27S4NHB012&log$=prottop&blast_rank=6) | nuclear receptor coactivator 6 [Homo sapiens] | [19.7](http://blast.ncbi.nlm.nih.gov/Blast.cgi#32307128%2332307128) | 19.7 | 50% | 200 |
| [NP_005240.3](http://www.ncbi.nlm.nih.gov/entrez/query.fcgi?cmd=Retrieve&db=Protein&list_uids=32307177&dopt=GenPept&RID=T27S4NHB012&log$=prottop&blast_rank=7) | forkhead box G1 [Homo sapiens] | [19.7](http://blast.ncbi.nlm.nih.gov/Blast.cgi#32307177%2332307177) | 19.7 | 50% | 200 |
| [NP_001001788.2](http://www.ncbi.nlm.nih.gov/entrez/query.fcgi?cmd=Retrieve&db=Protein&list_uids=130484306&dopt=GenPept&RID=T27S4NHB012&log$=prottop&blast_rank=8) | retinoic acid early transcript 1G precursor [Homo sapiens] | [19.7](http://blast.ncbi.nlm.nih.gov/Blast.cgi#130484306%23130484306) | 19.7 | 50% | 200 |
| [NP_001129479.1](http://www.ncbi.nlm.nih.gov/entrez/query.fcgi?cmd=Retrieve&db=Protein&list_uids=209862809&dopt=GenPept&RID=T27S4NHB012&log$=prottop&blast_rank=9) | FXYD domain containing ion transport regulator 3 isoform 3 [Homo sapiens] | [19.3](http://blast.ncbi.nlm.nih.gov/Blast.cgi#209862809%23209862809) | 19.3 | 90% | 268 |
| [NP_068710.1](http://www.ncbi.nlm.nih.gov/entrez/query.fcgi?cmd=Retrieve&db=Protein&list_uids=11612674&dopt=GenPept&RID=T27S4NHB012&log$=prottop&blast_rank=10) | FXYD domain containing ion transport regulator 3 isoform 2 precursor [Homo sapiens] >ref|NP_001129484.1| FXYD domain containing ion transport regulator 3 isoform 2 precursor [Homo sapiens] | [19.3](http://blast.ncbi.nlm.nih.gov/Blast.cgi#11612674%2311612674) | 19.3 | 90% | 268 |
| [NP_060647.2](http://www.ncbi.nlm.nih.gov/entrez/query.fcgi?cmd=Retrieve&db=Protein&list_uids=31742492&dopt=GenPept&RID=T27S4NHB012&log$=prottop&blast_rank=11) | Nedd4 binding protein 2 [Homo sapiens] | [19.3](http://blast.ncbi.nlm.nih.gov/Blast.cgi#31742492%2331742492) | 19.3 | 60% | 268 |
| [NP_005962.1](http://www.ncbi.nlm.nih.gov/entrez/query.fcgi?cmd=Retrieve&db=Protein&list_uids=5174635&dopt=GenPept&RID=T27S4NHB012&log$=prottop&blast_rank=12) | FXYD domain containing ion transport regulator 3 isoform 1 precursor [Homo sapiens] >ref|NP_001129483.1| FXYD domain containing ion transport regulator 3 isoform 1 precursor [Homo sapiens] | [19.3](http://blast.ncbi.nlm.nih.gov/Blast.cgi#5174635%235174635) | 19.3 | 90% | 268 |
| [XP_002344829.1](http://www.ncbi.nlm.nih.gov/entrez/query.fcgi?cmd=Retrieve&db=Protein&list_uids=239756161&dopt=GenPept&RID=T27S4NHB012&log$=prottop&blast_rank=13) | PREDICTED: hypothetical protein [Homo sapiens] | [18.9](http://blast.ncbi.nlm.nih.gov/Blast.cgi#239756161%23239756161) | 18.9 | 50% | 360 |
| [XP_002347506.1](http://www.ncbi.nlm.nih.gov/entrez/query.fcgi?cmd=Retrieve&db=Protein&list_uids=239750685&dopt=GenPept&RID=T27S4NHB012&log$=prottop&blast_rank=14) | PREDICTED: hypothetical protein XP_002347506 [Homo sapiens] | [18.9](http://blast.ncbi.nlm.nih.gov/Blast.cgi#239750685%23239750685) | 18.9 | 50% | 360 |
| [XP_002343325.1](http://www.ncbi.nlm.nih.gov/entrez/query.fcgi?cmd=Retrieve&db=Protein&list_uids=239744995&dopt=GenPept&RID=T27S4NHB012&log$=prottop&blast_rank=15) | PREDICTED: hypothetical protein XP_002343325 [Homo sapiens] | [18.9](http://blast.ncbi.nlm.nih.gov/Blast.cgi#239744995%23239744995) | 18.9 | 50% | 360 |
| [NP_955452.3](http://www.ncbi.nlm.nih.gov/entrez/query.fcgi?cmd=Retrieve&db=Protein&list_uids=139394648&dopt=GenPept&RID=T27S4NHB012&log$=prottop&blast_rank=16) | DNA polymerase theta [Homo sapiens] | [18.9](http://blast.ncbi.nlm.nih.gov/Blast.cgi#139394648%23139394648) | 32.7 | 70% | 360 |
| [NP_064703.1](http://www.ncbi.nlm.nih.gov/entrez/query.fcgi?cmd=Retrieve&db=Protein&list_uids=9945320&dopt=GenPept&RID=T27S4NHB012&log$=prottop&blast_rank=17) | cyclin L1 [Homo sapiens] | [18.9](http://blast.ncbi.nlm.nih.gov/Blast.cgi#9945320%239945320) | 35.6 | 90% | 360 |
| [XP_002345467.1](http://www.ncbi.nlm.nih.gov/entrez/query.fcgi?cmd=Retrieve&db=Protein&list_uids=239757500&dopt=GenPept&RID=T27S4NHB012&log$=prottop&blast_rank=18) | PREDICTED: hypothetical protein [Homo sapiens] | [18.5](http://blast.ncbi.nlm.nih.gov/Blast.cgi#239757500%23239757500) | 18.5 | 80% | 482 |
| [XP_002348069.1](http://www.ncbi.nlm.nih.gov/entrez/query.fcgi?cmd=Retrieve&db=Protein&list_uids=239752017&dopt=GenPept&RID=T27S4NHB012&log$=prottop&blast_rank=19) | PREDICTED: hypothetical protein XP_002348069 [Homo sapiens] | [18.5](http://blast.ncbi.nlm.nih.gov/Blast.cgi#239752017%23239752017) | 18.5 | 80% | 482 |
| [NP_002083.3](http://www.ncbi.nlm.nih.gov/entrez/query.fcgi?cmd=Retrieve&db=Protein&list_uids=149193321&dopt=GenPept&RID=T27S4NHB012&log$=prottop&blast_rank=20) | G-rich RNA sequence binding factor 1 isoform 1 [Homo sapiens] | [18.5](http://blast.ncbi.nlm.nih.gov/Blast.cgi#149193321%23149193321) | 18.5 | 60% | 482 |
| [NP_001091947.1](http://www.ncbi.nlm.nih.gov/entrez/query.fcgi?cmd=Retrieve&db=Protein&list_uids=149193319&dopt=GenPept&RID=T27S4NHB012&log$=prottop&blast_rank=21) | G-rich RNA sequence binding factor 1 isoform 2 [Homo sapiens] | [18.5](http://blast.ncbi.nlm.nih.gov/Blast.cgi#149193319%23149193319) | 18.5 | 60% | 482 |
| [NP_940905.2](http://www.ncbi.nlm.nih.gov/entrez/query.fcgi?cmd=Retrieve&db=Protein&list_uids=41349443&dopt=GenPept&RID=T27S4NHB012&log$=prottop&blast_rank=22) | potassium channel, subfamily T, member 2 [Homo sapiens] | [18.5](http://blast.ncbi.nlm.nih.gov/Blast.cgi#41349443%2341349443) | 18.5 | 70% | 482 |
| [NP_079507.1](http://www.ncbi.nlm.nih.gov/entrez/query.fcgi?cmd=Retrieve&db=Protein&list_uids=13376834&dopt=GenPept&RID=T27S4NHB012&log$=prottop&blast_rank=23) | zinc finger and SCAN domain containing 16 [Homo sapiens] | [18.5](http://blast.ncbi.nlm.nih.gov/Blast.cgi#13376834%2313376834) | 18.5 | 60% | 482 |
| [NP_065995.1](http://www.ncbi.nlm.nih.gov/entrez/query.fcgi?cmd=Retrieve&db=Protein&list_uids=24308251&dopt=GenPept&RID=T27S4NHB012&log$=prottop&blast_rank=24) | bile acid beta-glucosidase [Homo sapiens] | [18.5](http://blast.ncbi.nlm.nih.gov/Blast.cgi#24308251%2324308251) | 18.5 | 100% | 482 |
| [NP_005439.2](http://www.ncbi.nlm.nih.gov/entrez/query.fcgi?cmd=Retrieve&db=Protein&list_uids=257743454&dopt=GenPept&RID=T27S4NHB012&log$=prottop&blast_rank=25) | bone morphogenetic protein 15 precursor [Homo sapiens] | [18.5](http://blast.ncbi.nlm.nih.gov/Blast.cgi#257743454%23257743454) | 18.5 | 80% | 482 |
| [NP_060469.4](http://www.ncbi.nlm.nih.gov/entrez/query.fcgi?cmd=Retrieve&db=Protein&list_uids=109150431&dopt=GenPept&RID=T27S4NHB012&log$=prottop&blast_rank=26) | ring finger protein 31 [Homo sapiens] | [18.5](http://blast.ncbi.nlm.nih.gov/Blast.cgi#109150431%23109150431) | 18.5 | 60% | 482 |
| [NP_002281.2](http://www.ncbi.nlm.nih.gov/entrez/query.fcgi?cmd=Retrieve&db=Protein&list_uids=157419122&dopt=GenPept&RID=T27S4NHB012&log$=prottop&blast_rank=27) | laminin, alpha 4 isoform 2 precursor [Homo sapiens] >ref|NP_001098677.1| laminin, alpha 4 isoform 2 precursor [Homo sapiens] | [18.0](http://blast.ncbi.nlm.nih.gov/Blast.cgi#157419122%23157419122) | 31.8 | 60% | 647 |
| [NP_001098676.1](http://www.ncbi.nlm.nih.gov/entrez/query.fcgi?cmd=Retrieve&db=Protein&list_uids=157419124&dopt=GenPept&RID=T27S4NHB012&log$=prottop&blast_rank=28) | laminin, alpha 4 isoform 1 precursor [Homo sapiens] | [18.0](http://blast.ncbi.nlm.nih.gov/Blast.cgi#157419124%23157419124) | 31.8 | 60% | 647 |
| [NP_031370.2](http://www.ncbi.nlm.nih.gov/entrez/query.fcgi?cmd=Retrieve&db=Protein&list_uids=33356179&dopt=GenPept&RID=T27S4NHB012&log$=prottop&blast_rank=29) | transcription termination factor, RNA polymerase I [Homo sapiens] | [18.0](http://blast.ncbi.nlm.nih.gov/Blast.cgi#33356179%2333356179) | 18.0 | 60% | 647 |
| [NP_891550.1](http://www.ncbi.nlm.nih.gov/entrez/query.fcgi?cmd=Retrieve&db=Protein&list_uids=33624896&dopt=GenPept&RID=T27S4NHB012&log$=prottop&blast_rank=30) | ADAM metallopeptidase with thrombospondin type 1 motif, 9 preproprotein [Homo sapiens] | [18.0](http://blast.ncbi.nlm.nih.gov/Blast.cgi#33624896%2333624896) | 18.0 | 60% | 647 |
| [NP_008841.2](http://www.ncbi.nlm.nih.gov/entrez/query.fcgi?cmd=Retrieve&db=Protein&list_uids=33620769&dopt=GenPept&RID=T27S4NHB012&log$=prottop&blast_rank=31) | retinoblastoma-binding protein 6 isoform 1 [Homo sapiens] | [18.0](http://blast.ncbi.nlm.nih.gov/Blast.cgi#33620769%2333620769) | 18.0 | 60% | 647 |
| [NP_061173.1](http://www.ncbi.nlm.nih.gov/entrez/query.fcgi?cmd=Retrieve&db=Protein&list_uids=33620716&dopt=GenPept&RID=T27S4NHB012&log$=prottop&blast_rank=32) | retinoblastoma-binding protein 6 isoform 2 [Homo sapiens] | [18.0](http://blast.ncbi.nlm.nih.gov/Blast.cgi#33620716%2333620716) | 18.0 | 60% | 647 |
| [NP_006271.1](http://www.ncbi.nlm.nih.gov/entrez/query.fcgi?cmd=Retrieve&db=Protein&list_uids=5454090&dopt=GenPept&RID=T27S4NHB012&log$=prottop&blast_rank=33) | signal sequence receptor, delta precursor [Homo sapiens] | [18.0](http://blast.ncbi.nlm.nih.gov/Blast.cgi#5454090%235454090) | 18.0 | 60% | 647 |
| [NP_001155134.1](http://www.ncbi.nlm.nih.gov/entrez/query.fcgi?cmd=Retrieve&db=Protein&list_uids=242247257&dopt=GenPept&RID=T27S4NHB012&log$=prottop&blast_rank=34) | WW and C2 domain containing 1 isoform 2 [Homo sapiens] | [17.6](http://blast.ncbi.nlm.nih.gov/Blast.cgi#242247257%23242247257) | 17.6 | 50% | 868 |
| [XP_002346266.1](http://www.ncbi.nlm.nih.gov/entrez/query.fcgi?cmd=Retrieve&db=Protein&list_uids=239758008&dopt=GenPept&RID=T27S4NHB012&log$=prottop&blast_rank=35) | PREDICTED: hypothetical protein XP_002346266 [Homo sapiens] | [17.6](http://blast.ncbi.nlm.nih.gov/Blast.cgi#239758008%23239758008) | 62.4 | 60% | 868 |
| [XP_002344886.1](http://www.ncbi.nlm.nih.gov/entrez/query.fcgi?cmd=Retrieve&db=Protein&list_uids=239756344&dopt=GenPept&RID=T27S4NHB012&log$=prottop&blast_rank=36) | PREDICTED: hypothetical protein [Homo sapiens] | [17.6](http://blast.ncbi.nlm.nih.gov/Blast.cgi#239756344%23239756344) | 17.6 | 90% | 868 |
| [NP_079032.2](http://www.ncbi.nlm.nih.gov/entrez/query.fcgi?cmd=Retrieve&db=Protein&list_uids=221316695&dopt=GenPept&RID=T27S4NHB012&log$=prottop&blast_rank=37) | multimerin 2 precursor [Homo sapiens] | [17.6](http://blast.ncbi.nlm.nih.gov/Blast.cgi#221316695%23221316695) | 17.6 | 100% | 868 |
| [NP_835467.2](http://www.ncbi.nlm.nih.gov/entrez/query.fcgi?cmd=Retrieve&db=Protein&list_uids=206597547&dopt=GenPept&RID=T27S4NHB012&log$=prottop&blast_rank=38) | coiled-coil domain containing 36 [Homo sapiens] >ref|NP_001128669.1| coiled-coil domain containing 36 [Homo sapiens] | [17.6](http://blast.ncbi.nlm.nih.gov/Blast.cgi#206597547%23206597547) | 17.6 | 60% | 868 |
| [NP_001155133.1](http://www.ncbi.nlm.nih.gov/entrez/query.fcgi?cmd=Retrieve&db=Protein&list_uids=242247251&dopt=GenPept&RID=T27S4NHB012&log$=prottop&blast_rank=39) | WW and C2 domain containing 1 isoform 1 [Homo sapiens] | [17.6](http://blast.ncbi.nlm.nih.gov/Blast.cgi#242247251%23242247251) | 17.6 | 50% | 868 |
| [NP_115921.2](http://www.ncbi.nlm.nih.gov/entrez/query.fcgi?cmd=Retrieve&db=Protein&list_uids=148806908&dopt=GenPept&RID=T27S4NHB012&log$=prottop&blast_rank=40) | fibronectin type III domain containing 1 [Homo sapiens] | [17.6](http://blast.ncbi.nlm.nih.gov/Blast.cgi#148806908%23148806908) | 42.6 | 90% | 868 |
| [NP_001611.1](http://www.ncbi.nlm.nih.gov/entrez/query.fcgi?cmd=Retrieve&db=Protein&list_uids=61743954&dopt=GenPept&RID=T27S4NHB012&log$=prottop&blast_rank=41) | AHNAK nucleoprotein isoform 1 [Homo sapiens] | [17.6](http://blast.ncbi.nlm.nih.gov/Blast.cgi#61743954%2361743954) | 49.8 | 80% | 868 |
| [NP_001161414.1](http://www.ncbi.nlm.nih.gov/entrez/query.fcgi?cmd=Retrieve&db=Protein&list_uids=269846912&dopt=GenPept&RID=T27S4NHB012&log$=prottop&blast_rank=42) | tumor necrosis factor, alpha-induced protein 8-like 1 [Homo sapiens] >ref|NP_689575.2| tumor necrosis factor, alpha-induced protein 8-like 1 [Homo sapiens] | [17.6](http://blast.ncbi.nlm.nih.gov/Blast.cgi#269846912%23269846912) | 17.6 | 50% | 868 |
| [NP_002355.2](http://www.ncbi.nlm.nih.gov/entrez/query.fcgi?cmd=Retrieve&db=Protein&list_uids=222418639&dopt=GenPept&RID=T27S4NHB012&log$=prottop&blast_rank=43) | melanoma antigen family B, 2 [Homo sapiens] | [17.6](http://blast.ncbi.nlm.nih.gov/Blast.cgi#222418639%23222418639) | 17.6 | 50% | 868 |
| [NP_055165.2](http://www.ncbi.nlm.nih.gov/entrez/query.fcgi?cmd=Retrieve&db=Protein&list_uids=117676365&dopt=GenPept&RID=T27S4NHB012&log$=prottop&blast_rank=44) | tumor necrosis factor, alpha-induced protein 8 isoform a [Homo sapiens] | [17.6](http://blast.ncbi.nlm.nih.gov/Blast.cgi#117676365%23117676365) | 17.6 | 50% | 868 |
| [NP_056053.1](http://www.ncbi.nlm.nih.gov/entrez/query.fcgi?cmd=Retrieve&db=Protein&list_uids=29789058&dopt=GenPept&RID=T27S4NHB012&log$=prottop&blast_rank=45) | WW and C2 domain containing 1 isoform 3 [Homo sapiens] | [17.6](http://blast.ncbi.nlm.nih.gov/Blast.cgi#29789058%2329789058) | 17.6 | 50% | 868 |
| [NP_001071122.1](http://www.ncbi.nlm.nih.gov/entrez/query.fcgi?cmd=Retrieve&db=Protein&list_uids=117676370&dopt=GenPept&RID=T27S4NHB012&log$=prottop&blast_rank=46) | tumor necrosis factor, alpha-induced protein 8 isoform b [Homo sapiens] | [17.6](http://blast.ncbi.nlm.nih.gov/Blast.cgi#117676370%23117676370) | 17.6 | 50% | 868 |
| [NP_055087.2](http://www.ncbi.nlm.nih.gov/entrez/query.fcgi?cmd=Retrieve&db=Protein&list_uids=38683827&dopt=GenPept&RID=T27S4NHB012&log$=prottop&blast_rank=47) | ADAM metallopeptidase with thrombospondin type 1 motif, 7 preproprotein [Homo sapiens] | [17.6](http://blast.ncbi.nlm.nih.gov/Blast.cgi#38683827%2338683827) | 17.6 | 90% | 868 |
| [NP_003646.2](http://www.ncbi.nlm.nih.gov/entrez/query.fcgi?cmd=Retrieve&db=Protein&list_uids=55770830&dopt=GenPept&RID=T27S4NHB012&log$=prottop&blast_rank=48) | chromobox homolog 4 [Homo sapiens] | [17.6](http://blast.ncbi.nlm.nih.gov/Blast.cgi#55770830%2355770830) | 17.6 | 50% | 868 |
| [NP_065121.2](http://www.ncbi.nlm.nih.gov/entrez/query.fcgi?cmd=Retrieve&db=Protein&list_uids=20357504&dopt=GenPept&RID=T27S4NHB012&log$=prottop&blast_rank=49) | dystonin isoform 1eB precursor [Homo sapiens] | [17.6](http://blast.ncbi.nlm.nih.gov/Blast.cgi#20357504%2320357504) | 28.0 | 70% | 868 |
| [NP_640338.2](http://www.ncbi.nlm.nih.gov/entrez/query.fcgi?cmd=Retrieve&db=Protein&list_uids=63003905&dopt=GenPept&RID=T27S4NHB012&log$=prottop&blast_rank=50) | protein phosphatase 1 (formerly 2C)-like [Homo sapiens] | [17.6](http://blast.ncbi.nlm.nih.gov/Blast.cgi#63003905%2363003905) | 17.6 | 50% | 868 |
| [NP_877398.1](http://www.ncbi.nlm.nih.gov/entrez/query.fcgi?cmd=Retrieve&db=Protein&list_uids=33356544&dopt=GenPept&RID=T27S4NHB012&log$=prottop&blast_rank=51) | phosphoinositide-specific phospholipase C beta 1 isoform b [Homo sapiens] | [17.6](http://blast.ncbi.nlm.nih.gov/Blast.cgi#33356544%2333356544) | 17.6 | 50% | 868 |
| [NP_056007.1](http://www.ncbi.nlm.nih.gov/entrez/query.fcgi?cmd=Retrieve&db=Protein&list_uids=12083581&dopt=GenPept&RID=T27S4NHB012&log$=prottop&blast_rank=52) | phosphoinositide-specific phospholipase C beta 1 isoform a [Homo sapiens] | [17.6](http://blast.ncbi.nlm.nih.gov/Blast.cgi#12083581%2312083581) | 17.6 | 50% | 868 |
| [NP_003150.1](http://www.ncbi.nlm.nih.gov/entrez/query.fcgi?cmd=Retrieve&db=Protein&list_uids=4507281&dopt=GenPept&RID=T27S4NHB012&log$=prottop&blast_rank=53) | cyclin-dependent kinase-like 5 [Homo sapiens] >ref|NP_001032420.1| cyclin-dependent kinase-like 5 [Homo sapiens] | [17.6](http://blast.ncbi.nlm.nih.gov/Blast.cgi#4507281%234507281) | 17.6 | 50% | 868 |
| [NP_005580.1](http://www.ncbi.nlm.nih.gov/entrez/query.fcgi?cmd=Retrieve&db=Protein&list_uids=11095441&dopt=GenPept&RID=T27S4NHB012&log$=prottop&blast_rank=54) | aldehyde dehydrogenase 6A1 precursor [Homo sapiens] | [17.6](http://blast.ncbi.nlm.nih.gov/Blast.cgi#11095441%2311095441) | 17.6 | 60% | 868 |
| [NP_000370.2](http://www.ncbi.nlm.nih.gov/entrez/query.fcgi?cmd=Retrieve&db=Protein&list_uids=91823271&dopt=GenPept&RID=T27S4NHB012&log$=prottop&blast_rank=55) | xanthine dehydrogenase [Homo sapiens] | [17.6](http://blast.ncbi.nlm.nih.gov/Blast.cgi#91823271%2391823271) | 17.6 | 50% | 868 |
| [NP_001157945.1](http://www.ncbi.nlm.nih.gov/entrez/query.fcgi?cmd=Retrieve&db=Protein&list_uids=256600192&dopt=GenPept&RID=T27S4NHB012&log$=prottop&blast_rank=56) | formin binding protein 1-like isoform 3 [Homo sapiens] | [17.2](http://blast.ncbi.nlm.nih.gov/Blast.cgi#256600192%23256600192) | 17.2 | 40% | 1165 |
| [XP_002347245.1](http://www.ncbi.nlm.nih.gov/entrez/query.fcgi?cmd=Retrieve&db=Protein&list_uids=239749968&dopt=GenPept&RID=T27S4NHB012&log$=prottop&blast_rank=57) | PREDICTED: hypothetical protein XP_002347245 [Homo sapiens] >ref|XP_002344583.1| PREDICTED: hypothetical protein [Homo sapiens] | [17.2](http://blast.ncbi.nlm.nih.gov/Blast.cgi#239749968%23239749968) | 17.2 | 50% | 1165 |
| [XP_002343752.1](http://www.ncbi.nlm.nih.gov/entrez/query.fcgi?cmd=Retrieve&db=Protein&list_uids=239746528&dopt=GenPept&RID=T27S4NHB012&log$=prottop&blast_rank=58) | PREDICTED: hypothetical protein XP_002343752 [Homo sapiens] >ref|XP_002348074.1| PREDICTED: hypothetical protein XP_002348074 [Homo sapiens] >ref|XP_002345471.1| PREDICTED: hypothetical protein [Homo sapiens] | [17.2](http://blast.ncbi.nlm.nih.gov/Blast.cgi#239746528%23239746528) | 27.6 | 50% | 1165 |
| [XP_002343110.1](http://www.ncbi.nlm.nih.gov/entrez/query.fcgi?cmd=Retrieve&db=Protein&list_uids=239744278&dopt=GenPept&RID=T27S4NHB012&log$=prottop&blast_rank=59) | PREDICTED: hypothetical protein XP_002343110 [Homo sapiens] | [17.2](http://blast.ncbi.nlm.nih.gov/Blast.cgi#239744278%23239744278) | 17.2 | 50% | 1165 |
| [NP_003189.2](http://www.ncbi.nlm.nih.gov/entrez/query.fcgi?cmd=Retrieve&db=Protein&list_uids=208022641&dopt=GenPept&RID=T27S4NHB012&log$=prottop&blast_rank=60) | elongin A [Homo sapiens] | [17.2](http://blast.ncbi.nlm.nih.gov/Blast.cgi#208022641%23208022641) | 17.2 | 60% | 1165 |
| [NP_001127854.1](http://www.ncbi.nlm.nih.gov/entrez/query.fcgi?cmd=Retrieve&db=Protein&list_uids=197304786&dopt=GenPept&RID=T27S4NHB012&log$=prottop&blast_rank=61) | IQ motif and Sec7 domain 1 isoform a [Homo sapiens] | [17.2](http://blast.ncbi.nlm.nih.gov/Blast.cgi#197304786%23197304786) | 33.9 | 90% | 1165 |
| [NP_904358.2](http://www.ncbi.nlm.nih.gov/entrez/query.fcgi?cmd=Retrieve&db=Protein&list_uids=171460948&dopt=GenPept&RID=T27S4NHB012&log$=prottop&blast_rank=62) | TSC22 domain family, member 1 isoform 1 [Homo sapiens] | [17.2](http://blast.ncbi.nlm.nih.gov/Blast.cgi#171460948%23171460948) | 34.4 | 40% | 1165 |
| [NP_005867.3](http://www.ncbi.nlm.nih.gov/entrez/query.fcgi?cmd=Retrieve&db=Protein&list_uids=157785645&dopt=GenPept&RID=T27S4NHB012&log$=prottop&blast_rank=63) | SPEG complex locus [Homo sapiens] | [17.2](http://blast.ncbi.nlm.nih.gov/Blast.cgi#157785645%23157785645) | 17.2 | 40% | 1165 |
| [NP_036466.2](http://www.ncbi.nlm.nih.gov/entrez/query.fcgi?cmd=Retrieve&db=Protein&list_uids=154354979&dopt=GenPept&RID=T27S4NHB012&log$=prottop&blast_rank=64) | myosin X [Homo sapiens] | [17.2](http://blast.ncbi.nlm.nih.gov/Blast.cgi#154354979%23154354979) | 17.2 | 40% | 1165 |
| [NP_940857.2](http://www.ncbi.nlm.nih.gov/entrez/query.fcgi?cmd=Retrieve&db=Protein&list_uids=134031945&dopt=GenPept&RID=T27S4NHB012&log$=prottop&blast_rank=65) | SCO-spondin precursor [Homo sapiens] | [17.2](http://blast.ncbi.nlm.nih.gov/Blast.cgi#134031945%23134031945) | 17.2 | 40% | 1165 |
| [NP_067047.4](http://www.ncbi.nlm.nih.gov/entrez/query.fcgi?cmd=Retrieve&db=Protein&list_uids=114431236&dopt=GenPept&RID=T27S4NHB012&log$=prottop&blast_rank=66) | zinc finger protein 462 [Homo sapiens] | [17.2](http://blast.ncbi.nlm.nih.gov/Blast.cgi#114431236%23114431236) | 33.5 | 50% | 1165 |
| [NP_001073916.1](http://www.ncbi.nlm.nih.gov/entrez/query.fcgi?cmd=Retrieve&db=Protein&list_uids=122937293&dopt=GenPept&RID=T27S4NHB012&log$=prottop&blast_rank=67) | tubulin tyrosine ligase-like family, member 8 [Homo sapiens] | [17.2](http://blast.ncbi.nlm.nih.gov/Blast.cgi#122937293%23122937293) | 17.2 | 40% | 1165 |
| [NP_001020119.1](http://www.ncbi.nlm.nih.gov/entrez/query.fcgi?cmd=Retrieve&db=Protein&list_uids=68348709&dopt=GenPept&RID=T27S4NHB012&log$=prottop&blast_rank=68) | formin binding protein 1-like isoform 1 [Homo sapiens] | [17.2](http://blast.ncbi.nlm.nih.gov/Blast.cgi#68348709%2368348709) | 17.2 | 40% | 1165 |
| [NP_775837.2](http://www.ncbi.nlm.nih.gov/entrez/query.fcgi?cmd=Retrieve&db=Protein&list_uids=209969819&dopt=GenPept&RID=T27S4NHB012&log$=prottop&blast_rank=69) | hypothetical protein LOC253143 [Homo sapiens] | [17.2](http://blast.ncbi.nlm.nih.gov/Blast.cgi#209969819%23209969819) | 17.2 | 40% | 1165 |
| [NP_060207.2](http://www.ncbi.nlm.nih.gov/entrez/query.fcgi?cmd=Retrieve&db=Protein&list_uids=41581463&dopt=GenPept&RID=T27S4NHB012&log$=prottop&blast_rank=70) | formin binding protein 1-like isoform 2 [Homo sapiens] | [17.2](http://blast.ncbi.nlm.nih.gov/Blast.cgi#41581463%2341581463) | 17.2 | 40% | 1165 |
| [NP_060080.6](http://www.ncbi.nlm.nih.gov/entrez/query.fcgi?cmd=Retrieve&db=Protein&list_uids=37595553&dopt=GenPept&RID=T27S4NHB012&log$=prottop&blast_rank=71) | ring finger protein 111 [Homo sapiens] | [17.2](http://blast.ncbi.nlm.nih.gov/Blast.cgi#37595553%2337595553) | 34.4 | 40% | 1165 |
| [NP_004645.2](http://www.ncbi.nlm.nih.gov/entrez/query.fcgi?cmd=Retrieve&db=Protein&list_uids=74319833&dopt=GenPept&RID=T27S4NHB012&log$=prottop&blast_rank=72) | ubiquitin specific protease 9, Y-linked [Homo sapiens] | [17.2](http://blast.ncbi.nlm.nih.gov/Blast.cgi#74319833%2374319833) | 17.2 | 40% | 1165 |
| [NP_296373.1](http://www.ncbi.nlm.nih.gov/entrez/query.fcgi?cmd=Retrieve&db=Protein&list_uids=16306502&dopt=GenPept&RID=T27S4NHB012&log$=prottop&blast_rank=73) | F-box only protein 21 isoform 1 [Homo sapiens] | [17.2](http://blast.ncbi.nlm.nih.gov/Blast.cgi#16306502%2316306502) | 17.2 | 40% | 1165 |
| [XP_001720822.1](http://www.ncbi.nlm.nih.gov/entrez/query.fcgi?cmd=Retrieve&db=Protein&list_uids=169209613&dopt=GenPept&RID=T27S4NHB012&log$=prottop&blast_rank=74) | PREDICTED: hypothetical protein LOC100128785 [Homo sapiens] >ref|XP_001720440.1| PREDICTED: hypothetical protein LOC100128785 [Homo sapiens] >ref|XP_001723673.1| PREDICTED: hypothetical protein LOC100128785 [Homo sapiens] | [17.2](http://blast.ncbi.nlm.nih.gov/Blast.cgi#169209613%23169209613) | 17.2 | 40% | 1165 |
| [NP_055623.1](http://www.ncbi.nlm.nih.gov/entrez/query.fcgi?cmd=Retrieve&db=Protein&list_uids=7662310&dopt=GenPept&RID=T27S4NHB012&log$=prottop&blast_rank=75) | FERM, RhoGEF and pleckstrin domain protein 2 [Homo sapiens] | [17.2](http://blast.ncbi.nlm.nih.gov/Blast.cgi#7662310%237662310) | 17.2 | 50% | 1165 |
| [NP_060798.2](http://www.ncbi.nlm.nih.gov/entrez/query.fcgi?cmd=Retrieve&db=Protein&list_uids=38202209&dopt=GenPept&RID=T27S4NHB012&log$=prottop&blast_rank=76) | methyl-CpG binding domain protein 5 [Homo sapiens] | [17.2](http://blast.ncbi.nlm.nih.gov/Blast.cgi#38202209%2338202209) | 17.2 | 60% | 1165 |
| [NP_219483.1](http://www.ncbi.nlm.nih.gov/entrez/query.fcgi?cmd=Retrieve&db=Protein&list_uids=15529984&dopt=GenPept&RID=T27S4NHB012&log$=prottop&blast_rank=77) | armadillo repeat containing 6 [Homo sapiens] | [17.2](http://blast.ncbi.nlm.nih.gov/Blast.cgi#15529984%2315529984) | 17.2 | 40% | 1165 |
| [NP_056014.2](http://www.ncbi.nlm.nih.gov/entrez/query.fcgi?cmd=Retrieve&db=Protein&list_uids=68131557&dopt=GenPept&RID=T27S4NHB012&log$=prottop&blast_rank=78) | ankyrin repeat domain 28 [Homo sapiens] | [17.2](http://blast.ncbi.nlm.nih.gov/Blast.cgi#68131557%2368131557) | 17.2 | 40% | 1165 |
| [NP_005481.2](http://www.ncbi.nlm.nih.gov/entrez/query.fcgi?cmd=Retrieve&db=Protein&list_uids=208609985&dopt=GenPept&RID=T27S4NHB012&log$=prottop&blast_rank=79) | SH2 domain containing 3A [Homo sapiens] | [17.2](http://blast.ncbi.nlm.nih.gov/Blast.cgi#208609985%23208609985) | 17.2 | 40% | 1165 |
| [NP_976227.1](http://www.ncbi.nlm.nih.gov/entrez/query.fcgi?cmd=Retrieve&db=Protein&list_uids=42741675&dopt=GenPept&RID=T27S4NHB012&log$=prottop&blast_rank=80) | PDZ and LIM domain 7 isoform 2 [Homo sapiens] | [17.2](http://blast.ncbi.nlm.nih.gov/Blast.cgi#42741675%2342741675) | 17.2 | 70% | 1165 |
| [NP_849193.1](http://www.ncbi.nlm.nih.gov/entrez/query.fcgi?cmd=Retrieve&db=Protein&list_uids=30578410&dopt=GenPept&RID=T27S4NHB012&log$=prottop&blast_rank=81) | STT3, subunit of the oligosaccharyltransferase complex, homolog B [Homo sapiens] | [17.2](http://blast.ncbi.nlm.nih.gov/Blast.cgi#30578410%2330578410) | 17.2 | 40% | 1165 |
| [NP_694691.1](http://www.ncbi.nlm.nih.gov/entrez/query.fcgi?cmd=Retrieve&db=Protein&list_uids=23510389&dopt=GenPept&RID=T27S4NHB012&log$=prottop&blast_rank=82) | myotubularin-related protein 3 isoform b [Homo sapiens] | [17.2](http://blast.ncbi.nlm.nih.gov/Blast.cgi#23510389%2323510389) | 17.2 | 50% | 1165 |
| [NP_001788.2](http://www.ncbi.nlm.nih.gov/entrez/query.fcgi?cmd=Retrieve&db=Protein&list_uids=16306532&dopt=GenPept&RID=T27S4NHB012&log$=prottop&blast_rank=83) | cadherin 11, type 2 preproprotein [Homo sapiens] | [17.2](http://blast.ncbi.nlm.nih.gov/Blast.cgi#16306532%2316306532) | 17.2 | 40% | 1165 |
| [NP_694690.1](http://www.ncbi.nlm.nih.gov/entrez/query.fcgi?cmd=Retrieve&db=Protein&list_uids=23510387&dopt=GenPept&RID=T27S4NHB012&log$=prottop&blast_rank=84) | myotubularin-related protein 3 isoform a [Homo sapiens] | [17.2](http://blast.ncbi.nlm.nih.gov/Blast.cgi#23510387%2323510387) | 17.2 | 50% | 1165 |
| [NP_112603.2](http://www.ncbi.nlm.nih.gov/entrez/query.fcgi?cmd=Retrieve&db=Protein&list_uids=157266296&dopt=GenPept&RID=T27S4NHB012&log$=prottop&blast_rank=85) | placental-like alkaline phosphatase preproprotein [Homo sapiens] | [17.2](http://blast.ncbi.nlm.nih.gov/Blast.cgi#157266296%23157266296) | 17.2 | 40% | 1165 |
| [NP_002007.1](http://www.ncbi.nlm.nih.gov/entrez/query.fcgi?cmd=Retrieve&db=Protein&list_uids=60097902&dopt=GenPept&RID=T27S4NHB012&log$=prottop&blast_rank=86) | filaggrin [Homo sapiens] | [17.2](http://blast.ncbi.nlm.nih.gov/Blast.cgi#60097902%2360097902) | 80.8 | 60% | 1165 |
| [NP_997278.2](http://www.ncbi.nlm.nih.gov/entrez/query.fcgi?cmd=Retrieve&db=Protein&list_uids=194018495&dopt=GenPept&RID=T27S4NHB012&log$=prottop&blast_rank=87) | zinc finger protein 324B [Homo sapiens] | [17.2](http://blast.ncbi.nlm.nih.gov/Blast.cgi#194018495%23194018495) | 27.6 | 50% | 1165 |
| [NP_937879.1](http://www.ncbi.nlm.nih.gov/entrez/query.fcgi?cmd=Retrieve&db=Protein&list_uids=38026934&dopt=GenPept&RID=T27S4NHB012&log$=prottop&blast_rank=88) | Rho guanine nucleotide exchange factor (GEF) 11 isoform 2 [Homo sapiens] | [17.2](http://blast.ncbi.nlm.nih.gov/Blast.cgi#38026934%2338026934) | 17.2 | 70% | 1165 |
| [NP_857593.1](http://www.ncbi.nlm.nih.gov/entrez/query.fcgi?cmd=Retrieve&db=Protein&list_uids=32313599&dopt=GenPept&RID=T27S4NHB012&log$=prottop&blast_rank=89) | serine peptidase inhibitor, Kunitz type 1 isoform 1 precursor [Homo sapiens] | [17.2](http://blast.ncbi.nlm.nih.gov/Blast.cgi#32313599%2332313599) | 17.2 | 40% | 1165 |
| [NP_036474.1](http://www.ncbi.nlm.nih.gov/entrez/query.fcgi?cmd=Retrieve&db=Protein&list_uids=6912534&dopt=GenPept&RID=T27S4NHB012&log$=prottop&blast_rank=90) | BMP and activin membrane-bound inhibitor precursor [Homo sapiens] | [17.2](http://blast.ncbi.nlm.nih.gov/Blast.cgi#6912534%236912534) | 17.2 | 40% | 1165 |
| [NP_066576.1](http://www.ncbi.nlm.nih.gov/entrez/query.fcgi?cmd=Retrieve&db=Protein&list_uids=10835109&dopt=GenPept&RID=T27S4NHB012&log$=prottop&blast_rank=91) | myotubularin-related protein 3 isoform c [Homo sapiens] | [17.2](http://blast.ncbi.nlm.nih.gov/Blast.cgi#10835109%2310835109) | 17.2 | 50% | 1165 |
| [NP_001623.3](http://www.ncbi.nlm.nih.gov/entrez/query.fcgi?cmd=Retrieve&db=Protein&list_uids=94721246&dopt=GenPept&RID=T27S4NHB012&log$=prottop&blast_rank=92) | placental alkaline phosphatase preproprotein [Homo sapiens] | [17.2](http://blast.ncbi.nlm.nih.gov/Blast.cgi#94721246%2394721246) | 17.2 | 40% | 1165 |
| [NP_000981.1](http://www.ncbi.nlm.nih.gov/entrez/query.fcgi?cmd=Retrieve&db=Protein&list_uids=4506625&dopt=GenPept&RID=T27S4NHB012&log$=prottop&blast_rank=93) | ribosomal protein L27a [Homo sapiens] | [17.2](http://blast.ncbi.nlm.nih.gov/Blast.cgi#4506625%234506625) | 17.2 | 60% | 1165 |
| [NP_000469.3](http://www.ncbi.nlm.nih.gov/entrez/query.fcgi?cmd=Retrieve&db=Protein&list_uids=116734717&dopt=GenPept&RID=T27S4NHB012&log$=prottop&blast_rank=94) | tissue-nonspecific alkaline phosphatase precursor [Homo sapiens] >ref|NP_001120973.1| tissue-nonspecific alkaline phosphatase precursor [Homo sapiens] | [17.2](http://blast.ncbi.nlm.nih.gov/Blast.cgi#116734717%23116734717) | 17.2 | 40% | 1165 |
| [NP_003066.2](http://www.ncbi.nlm.nih.gov/entrez/query.fcgi?cmd=Retrieve&db=Protein&list_uids=21237805&dopt=GenPept&RID=T27S4NHB012&log$=prottop&blast_rank=95) | SWI/SNF-related matrix-associated actin-dependent regulator of chromatin c2 isoform a [Homo sapiens] | [17.2](http://blast.ncbi.nlm.nih.gov/Blast.cgi#21237805%2321237805) | 27.6 | 50% | 1165 |
| [NP_060695.2](http://www.ncbi.nlm.nih.gov/entrez/query.fcgi?cmd=Retrieve&db=Protein&list_uids=109948304&dopt=GenPept&RID=T27S4NHB012&log$=prottop&blast_rank=96) | smu-1 suppressor of mec-8 and unc-52 homolog [Homo sapiens] | [17.2](http://blast.ncbi.nlm.nih.gov/Blast.cgi#109948304%23109948304) | 17.2 | 60% | 1165 |
| [NP_005242.1](http://www.ncbi.nlm.nih.gov/entrez/query.fcgi?cmd=Retrieve&db=Protein&list_uids=4885237&dopt=GenPept&RID=T27S4NHB012&log$=prottop&blast_rank=97) | forkhead box C2 [Homo sapiens] | [17.2](http://blast.ncbi.nlm.nih.gov/Blast.cgi#4885237%234885237) | 17.2 | 40% | 1165 |
| [NP_003701.1](http://www.ncbi.nlm.nih.gov/entrez/query.fcgi?cmd=Retrieve&db=Protein&list_uids=4504329&dopt=GenPept&RID=T27S4NHB012&log$=prottop&blast_rank=98) | serine peptidase inhibitor, Kunitz type 1 isoform 2 precursor [Homo sapiens] >ref|NP_001027539.1| serine peptidase inhibitor, Kunitz type 1 isoform 2 precursor [Homo sapiens] | [17.2](http://blast.ncbi.nlm.nih.gov/Blast.cgi#4504329%234504329) | 17.2 | 40% | 1165 |
| [NP_079456.2](http://www.ncbi.nlm.nih.gov/entrez/query.fcgi?cmd=Retrieve&db=Protein&list_uids=22095367&dopt=GenPept&RID=T27S4NHB012&log$=prottop&blast_rank=99) | centrosomal protein 63 isoform a [Homo sapiens] | [17.2](http://blast.ncbi.nlm.nih.gov/Blast.cgi#22095367%2322095367) | 17.2 | 50% | 1165 |
| [NP_055817.1](http://www.ncbi.nlm.nih.gov/entrez/query.fcgi?cmd=Retrieve&db=Protein&list_uids=16306500&dopt=GenPept&RID=T27S4NHB012&log$=prottop&blast_rank=100) | F-box only protein 21 isoform 2 [Homo sapiens] | [17.2](http://blast.ncbi.nlm.nih.gov/Blast.cgi#16306500%2316306500) | 17.2 | 40% | 1165 |

| **Accession** | **Proteins with a match to KSNKCF peptide** | **[Max score](http://blast.ncbi.nlm.nih.gov/Blast.cgi?CMD=Get&ALIGNMENTS=100&ALIGNMENT_VIEW=Pairwise&CDD_SEARCH_STATE=1&DATABASE_SORT=0&DESCRIPTIONS=100&ENTREZ_QUERY=txid9606 %5BORGN%5D&FIRST_QUERY_NUM=0&FORMAT_OBJECT=Alignment&FORMAT_PAGE_TARGET=&FORMAT_TYPE=HTML&GET_SEQUENCE=yes&I_THRESH=&MASK_CHAR=2&MASK_COLOR=1&NEW_DESIGN=on&NEW_VIEW=yes&NUM_OVERVIEW=100&OLD_BLAST=false&PAGE=Proteins&QUERY_INDEX=0&QUERY_NUMBER=0&RESULTS_PAGE_TARGET=&RID=T27VS6KG01S&SHOW_LINKOUT=yes&SHOW_OVERVIEW=yes&STEP_NUMBER=&WORD_SIZE=2&DISPLAY_SORT=1&HSP_SORT=1" \l "sort_mark)** | **[Total score](http://blast.ncbi.nlm.nih.gov/Blast.cgi?CMD=Get&ALIGNMENTS=100&ALIGNMENT_VIEW=Pairwise&CDD_SEARCH_STATE=1&DATABASE_SORT=0&DESCRIPTIONS=100&ENTREZ_QUERY=txid9606 %5BORGN%5D&FIRST_QUERY_NUM=0&FORMAT_OBJECT=Alignment&FORMAT_PAGE_TARGET=&FORMAT_TYPE=HTML&GET_SEQUENCE=yes&I_THRESH=&MASK_CHAR=2&MASK_COLOR=1&NEW_DESIGN=on&NEW_VIEW=yes&NUM_OVERVIEW=100&OLD_BLAST=false&PAGE=Proteins&QUERY_INDEX=0&QUERY_NUMBER=0&RESULTS_PAGE_TARGET=&RID=T27VS6KG01S&SHOW_LINKOUT=yes&SHOW_OVERVIEW=yes&STEP_NUMBER=&WORD_SIZE=2&DISPLAY_SORT=2&HSP_SORT=1" \l "sort_mark)** | **[Query coverage](http://blast.ncbi.nlm.nih.gov/Blast.cgi?CMD=Get&ALIGNMENTS=100&ALIGNMENT_VIEW=Pairwise&CDD_SEARCH_STATE=1&DATABASE_SORT=0&DESCRIPTIONS=100&ENTREZ_QUERY=txid9606 %5BORGN%5D&FIRST_QUERY_NUM=0&FORMAT_OBJECT=Alignment&FORMAT_PAGE_TARGET=&FORMAT_TYPE=HTML&GET_SEQUENCE=yes&I_THRESH=&MASK_CHAR=2&MASK_COLOR=1&NEW_DESIGN=on&NEW_VIEW=yes&NUM_OVERVIEW=100&OLD_BLAST=false&PAGE=Proteins&QUERY_INDEX=0&QUERY_NUMBER=0&RESULTS_PAGE_TARGET=&RID=T27VS6KG01S&SHOW_LINKOUT=yes&SHOW_OVERVIEW=yes&STEP_NUMBER=&WORD_SIZE=2&DISPLAY_SORT=4&HSP_SORT=0" \l "sort_mark)** | **[E value](http://blast.ncbi.nlm.nih.gov/Blast.cgi?CMD=Get&ALIGNMENTS=100&ALIGNMENT_VIEW=Pairwise&CDD_SEARCH_STATE=1&DATABASE_SORT=0&DESCRIPTIONS=100&ENTREZ_QUERY=txid9606 %5BORGN%5D&FIRST_QUERY_NUM=0&FORMAT_OBJECT=Alignment&FORMAT_PAGE_TARGET=&FORMAT_TYPE=HTML&GET_SEQUENCE=yes&I_THRESH=&MASK_CHAR=2&MASK_COLOR=1&NEW_DESIGN=on&NEW_VIEW=yes&NUM_OVERVIEW=100&OLD_BLAST=false&PAGE=Proteins&QUERY_INDEX=0&QUERY_NUMBER=0&RESULTS_PAGE_TARGET=&RID=T27VS6KG01S&SHOW_LINKOUT=yes&SHOW_OVERVIEW=yes&STEP_NUMBER=&WORD_SIZE=2&DISPLAY_SORT=0&HSP_SORT=0" \l "sort_mark)** |
| --- | --- | --- | --- | --- | --- |
| [NP_001009567.1](http://www.ncbi.nlm.nih.gov/entrez/query.fcgi?cmd=Retrieve&db=Protein&list_uids=57546917&dopt=GenPept&RID=T27VS6KG01S&log$=prottop&blast_rank=1) | mannose receptor, C type 1-like 1 precursor [Homo sapiens] | [20.2](http://blast.ncbi.nlm.nih.gov/Blast.cgi#57546917%2357546917) | 20.2 | 83% | 89 |
| [NP_002429.1](http://www.ncbi.nlm.nih.gov/entrez/query.fcgi?cmd=Retrieve&db=Protein&list_uids=4505245&dopt=GenPept&RID=T27VS6KG01S&log$=prottop&blast_rank=2) | mannose receptor C type 1 precursor [Homo sapiens] | [20.2](http://blast.ncbi.nlm.nih.gov/Blast.cgi#4505245%234505245) | 20.2 | 83% | 89 |
| [NP_001975.1](http://www.ncbi.nlm.nih.gov/entrez/query.fcgi?cmd=Retrieve&db=Protein&list_uids=33413400&dopt=GenPept&RID=T27VS6KG01S&log$=prottop&blast_rank=3) | esterase D/formylglutathione hydrolase [Homo sapiens] | [20.2](http://blast.ncbi.nlm.nih.gov/Blast.cgi#33413400%2333413400) | 20.2 | 83% | 89 |
| [NP_060821.3](http://www.ncbi.nlm.nih.gov/entrez/query.fcgi?cmd=Retrieve&db=Protein&list_uids=154240686&dopt=GenPept&RID=T27VS6KG01S&log$=prottop&blast_rank=4) | FYVE, RhoGEF and PH domain containing 6 [Homo sapiens] | [19.7](http://blast.ncbi.nlm.nih.gov/Blast.cgi#154240686%23154240686) | 19.7 | 100% | 120 |
| [NP_001007526.2](http://www.ncbi.nlm.nih.gov/entrez/query.fcgi?cmd=Retrieve&db=Protein&list_uids=169234742&dopt=GenPept&RID=T27VS6KG01S&log$=prottop&blast_rank=5) | NACHT and WD repeat domain containing 1 [Homo sapiens] | [19.3](http://blast.ncbi.nlm.nih.gov/Blast.cgi#169234742%23169234742) | 19.3 | 83% | 161 |
| [NP_060385.2](http://www.ncbi.nlm.nih.gov/entrez/query.fcgi?cmd=Retrieve&db=Protein&list_uids=90819239&dopt=GenPept&RID=T27VS6KG01S&log$=prottop&blast_rank=6) | hypothetical protein LOC55010 [Homo sapiens] | [19.3](http://blast.ncbi.nlm.nih.gov/Blast.cgi#90819239%2390819239) | 19.3 | 83% | 161 |
| [NP_000923.1](http://www.ncbi.nlm.nih.gov/entrez/query.fcgi?cmd=Retrieve&db=Protein&list_uids=11386139&dopt=GenPept&RID=T27VS6KG01S&log$=prottop&blast_rank=7) | phospholipase C beta 3 [Homo sapiens] | [19.3](http://blast.ncbi.nlm.nih.gov/Blast.cgi#11386139%2311386139) | 19.3 | 100% | 161 |
| [NP_658988.2](http://www.ncbi.nlm.nih.gov/entrez/query.fcgi?cmd=Retrieve&db=Protein&list_uids=146260268&dopt=GenPept&RID=T27VS6KG01S&log$=prottop&blast_rank=8) | Smith-Magenis syndrome chromosome region, candidate 8 [Homo sapiens] | [18.9](http://blast.ncbi.nlm.nih.gov/Blast.cgi#146260268%23146260268) | 18.9 | 100% | 216 |
| [NP_015628.2](http://www.ncbi.nlm.nih.gov/entrez/query.fcgi?cmd=Retrieve&db=Protein&list_uids=116534990&dopt=GenPept&RID=T27VS6KG01S&log$=prottop&blast_rank=9) | ankyrin-like protein 1 [Homo sapiens] | [18.5](http://blast.ncbi.nlm.nih.gov/Blast.cgi#116534990%23116534990) | 18.5 | 100% | 289 |
| [XP_001726826.2](http://www.ncbi.nlm.nih.gov/entrez/query.fcgi?cmd=Retrieve&db=Protein&list_uids=239751469&dopt=GenPept&RID=T27VS6KG01S&log$=prottop&blast_rank=10) | PREDICTED: hypothetical protein [Homo sapiens] | [17.6](http://blast.ncbi.nlm.nih.gov/Blast.cgi#239751469%23239751469) | 17.6 | 66% | 521 |
| [NP_660334.3](http://www.ncbi.nlm.nih.gov/entrez/query.fcgi?cmd=Retrieve&db=Protein&list_uids=223972645&dopt=GenPept&RID=T27VS6KG01S&log$=prottop&blast_rank=11) | zinc finger protein 509 [Homo sapiens] | [17.6](http://blast.ncbi.nlm.nih.gov/Blast.cgi#223972645%23223972645) | 17.6 | 66% | 521 |
| [XP_001716605.1](http://www.ncbi.nlm.nih.gov/entrez/query.fcgi?cmd=Retrieve&db=Protein&list_uids=169167589&dopt=GenPept&RID=T27VS6KG01S&log$=prottop&blast_rank=12) | PREDICTED: hypothetical protein [Homo sapiens] >ref|XP_001715532.1| PREDICTED: hypothetical protein [Homo sapiens] >ref|XP_001717732.2| PREDICTED: hypothetical protein [Homo sapiens] | [17.6](http://blast.ncbi.nlm.nih.gov/Blast.cgi#169167589%23169167589) | 17.6 | 66% | 521 |
| [NP_062546.2](http://www.ncbi.nlm.nih.gov/entrez/query.fcgi?cmd=Retrieve&db=Protein&list_uids=154350236&dopt=GenPept&RID=T27VS6KG01S&log$=prottop&blast_rank=13) | hypothetical protein LOC56204 [Homo sapiens] | [17.6](http://blast.ncbi.nlm.nih.gov/Blast.cgi#154350236%23154350236) | 17.6 | 66% | 521 |
| [NP_002340.2](http://www.ncbi.nlm.nih.gov/entrez/query.fcgi?cmd=Retrieve&db=Protein&list_uids=144446030&dopt=GenPept&RID=T27VS6KG01S&log$=prottop&blast_rank=14) | lymphocyte antigen 75 precursor [Homo sapiens] | [17.6](http://blast.ncbi.nlm.nih.gov/Blast.cgi#144446030%23144446030) | 17.6 | 66% | 521 |
| [NP_056262.3](http://www.ncbi.nlm.nih.gov/entrez/query.fcgi?cmd=Retrieve&db=Protein&list_uids=186659512&dopt=GenPept&RID=T27VS6KG01S&log$=prottop&blast_rank=15) | calmodulin regulated spectrin-associated protein 1 [Homo sapiens] | [17.6](http://blast.ncbi.nlm.nih.gov/Blast.cgi#186659512%23186659512) | 32.7 | 100% | 521 |
| [NP_006759.3](http://www.ncbi.nlm.nih.gov/entrez/query.fcgi?cmd=Retrieve&db=Protein&list_uids=188497705&dopt=GenPept&RID=T27VS6KG01S&log$=prottop&blast_rank=16) | BRCA1 associated protein [Homo sapiens] | [17.6](http://blast.ncbi.nlm.nih.gov/Blast.cgi#188497705%23188497705) | 17.6 | 66% | 521 |
| [NP_006030.2](http://www.ncbi.nlm.nih.gov/entrez/query.fcgi?cmd=Retrieve&db=Protein&list_uids=110624774&dopt=GenPept&RID=T27VS6KG01S&log$=prottop&blast_rank=17) | mannose receptor, C type 2 [Homo sapiens] | [17.6](http://blast.ncbi.nlm.nih.gov/Blast.cgi#110624774%23110624774) | 17.6 | 66% | 521 |
| [NP_079191.2](http://www.ncbi.nlm.nih.gov/entrez/query.fcgi?cmd=Retrieve&db=Protein&list_uids=170784817&dopt=GenPept&RID=T27VS6KG01S&log$=prottop&blast_rank=18) | transcription factor CP2-like 3 [Homo sapiens] | [17.6](http://blast.ncbi.nlm.nih.gov/Blast.cgi#170784817%23170784817) | 17.6 | 66% | 521 |
| [NP_002149.2](http://www.ncbi.nlm.nih.gov/entrez/query.fcgi?cmd=Retrieve&db=Protein&list_uids=40549454&dopt=GenPept&RID=T27VS6KG01S&log$=prottop&blast_rank=19) | T-cell leukemia virus enhancer factor [Homo sapiens] | [17.6](http://blast.ncbi.nlm.nih.gov/Blast.cgi#40549454%2340549454) | 17.6 | 66% | 521 |
| [NP_079163.2](http://www.ncbi.nlm.nih.gov/entrez/query.fcgi?cmd=Retrieve&db=Protein&list_uids=45580742&dopt=GenPept&RID=T27VS6KG01S&log$=prottop&blast_rank=20) | dehydrodolichyl diphosphate synthase isoform a [Homo sapiens] | [17.6](http://blast.ncbi.nlm.nih.gov/Blast.cgi#45580742%2345580742) | 17.6 | 66% | 521 |
| [NP_005188.2](http://www.ncbi.nlm.nih.gov/entrez/query.fcgi?cmd=Retrieve&db=Protein&list_uids=51093865&dopt=GenPept&RID=T27VS6KG01S&log$=prottop&blast_rank=21) | checkpoint suppressor 1 isoform 2 [Homo sapiens] | [17.6](http://blast.ncbi.nlm.nih.gov/Blast.cgi#51093865%2351093865) | 17.6 | 66% | 521 |
| [NP_058197.2](http://www.ncbi.nlm.nih.gov/entrez/query.fcgi?cmd=Retrieve&db=Protein&list_uids=74229019&dopt=GenPept&RID=T27VS6KG01S&log$=prottop&blast_rank=22) | 2'-5'-oligoadenylate synthetase 2 isoform 1 [Homo sapiens] | [17.6](http://blast.ncbi.nlm.nih.gov/Blast.cgi#74229019%2374229019) | 17.6 | 66% | 521 |
| [NP_055762.3](http://www.ncbi.nlm.nih.gov/entrez/query.fcgi?cmd=Retrieve&db=Protein&list_uids=50345831&dopt=GenPept&RID=T27VS6KG01S&log$=prottop&blast_rank=23) | forkhead box J3 [Homo sapiens] | [17.6](http://blast.ncbi.nlm.nih.gov/Blast.cgi#50345831%2350345831) | 17.6 | 66% | 521 |
| [NP_005240.3](http://www.ncbi.nlm.nih.gov/entrez/query.fcgi?cmd=Retrieve&db=Protein&list_uids=32307177&dopt=GenPept&RID=T27VS6KG01S&log$=prottop&blast_rank=24) | forkhead box G1 [Homo sapiens] | [17.6](http://blast.ncbi.nlm.nih.gov/Blast.cgi#32307177%2332307177) | 17.6 | 66% | 521 |
| [NP_775864.3](http://www.ncbi.nlm.nih.gov/entrez/query.fcgi?cmd=Retrieve&db=Protein&list_uids=71043500&dopt=GenPept&RID=T27VS6KG01S&log$=prottop&blast_rank=25) | beta 1,4-N-acetylgalactosaminyltransferase-transferase 3 [Homo sapiens] | [17.6](http://blast.ncbi.nlm.nih.gov/Blast.cgi#71043500%2371043500) | 17.6 | 66% | 521 |
| [NP_002526.2](http://www.ncbi.nlm.nih.gov/entrez/query.fcgi?cmd=Retrieve&db=Protein&list_uids=74229017&dopt=GenPept&RID=T27VS6KG01S&log$=prottop&blast_rank=26) | 2'-5'-oligoadenylate synthetase 2 isoform 2 [Homo sapiens] | [17.6](http://blast.ncbi.nlm.nih.gov/Blast.cgi#74229017%2374229017) | 17.6 | 66% | 521 |
| [NP_998761.2](http://www.ncbi.nlm.nih.gov/entrez/query.fcgi?cmd=Retrieve&db=Protein&list_uids=166795280&dopt=GenPept&RID=T27VS6KG01S&log$=prottop&blast_rank=27) | forkhead box N4 [Homo sapiens] | [17.6](http://blast.ncbi.nlm.nih.gov/Blast.cgi#166795280%23166795280) | 17.6 | 66% | 521 |
| [NP_848632.2](http://www.ncbi.nlm.nih.gov/entrez/query.fcgi?cmd=Retrieve&db=Protein&list_uids=40789265&dopt=GenPept&RID=T27VS6KG01S&log$=prottop&blast_rank=28) | beta 1,4-N-acetylgalactosaminyltransferase-transferase 4 [Homo sapiens] | [17.6](http://blast.ncbi.nlm.nih.gov/Blast.cgi#40789265%2340789265) | 17.6 | 66% | 521 |
| [NP_940947.3](http://www.ncbi.nlm.nih.gov/entrez/query.fcgi?cmd=Retrieve&db=Protein&list_uids=86990448&dopt=GenPept&RID=T27VS6KG01S&log$=prottop&blast_rank=29) | chromosome 1 open reading frame 187 precursor [Homo sapiens] | [17.6](http://blast.ncbi.nlm.nih.gov/Blast.cgi#86990448%2386990448) | 17.6 | 66% | 521 |
| [NP_060886.1](http://www.ncbi.nlm.nih.gov/entrez/query.fcgi?cmd=Retrieve&db=Protein&list_uids=8923842&dopt=GenPept&RID=T27VS6KG01S&log$=prottop&blast_rank=30) | forkhead box J2 [Homo sapiens] | [17.6](http://blast.ncbi.nlm.nih.gov/Blast.cgi#8923842%238923842) | 17.6 | 66% | 521 |
| [NP_003584.2](http://www.ncbi.nlm.nih.gov/entrez/query.fcgi?cmd=Retrieve&db=Protein&list_uids=18201913&dopt=GenPept&RID=T27VS6KG01S&log$=prottop&blast_rank=31) | forkhead box N1 [Homo sapiens] | [17.6](http://blast.ncbi.nlm.nih.gov/Blast.cgi#18201913%2318201913) | 17.6 | 66% | 521 |
| [NP_000927.1](http://www.ncbi.nlm.nih.gov/entrez/query.fcgi?cmd=Retrieve&db=Protein&list_uids=10835000&dopt=GenPept&RID=T27VS6KG01S&log$=prottop&blast_rank=32) | pancreatic lipase precursor [Homo sapiens] | [17.6](http://blast.ncbi.nlm.nih.gov/Blast.cgi#10835000%2310835000) | 17.6 | 66% | 521 |
| [NP_690048.1](http://www.ncbi.nlm.nih.gov/entrez/query.fcgi?cmd=Retrieve&db=Protein&list_uids=22779870&dopt=GenPept&RID=T27VS6KG01S&log$=prottop&blast_rank=33) | PDLIM1 interacting kinase 1 like [Homo sapiens] | [17.6](http://blast.ncbi.nlm.nih.gov/Blast.cgi#22779870%2322779870) | 17.6 | 66% | 521 |
| [NP_995583.1](http://www.ncbi.nlm.nih.gov/entrez/query.fcgi?cmd=Retrieve&db=Protein&list_uids=45580738&dopt=GenPept&RID=T27VS6KG01S&log$=prottop&blast_rank=34) | dehydrodolichyl diphosphate synthase isoform b [Homo sapiens] | [17.6](http://blast.ncbi.nlm.nih.gov/Blast.cgi#45580738%2345580738) | 17.6 | 66% | 521 |
| [NP_001078940.1](http://www.ncbi.nlm.nih.gov/entrez/query.fcgi?cmd=Retrieve&db=Protein&list_uids=146232000&dopt=GenPept&RID=T27VS6KG01S&log$=prottop&blast_rank=35) | checkpoint suppressor 1 isoform 1 [Homo sapiens] | [17.6](http://blast.ncbi.nlm.nih.gov/Blast.cgi#146232000%23146232000) | 17.6 | 66% | 521 |
| [NP_001445.2](http://www.ncbi.nlm.nih.gov/entrez/query.fcgi?cmd=Retrieve&db=Protein&list_uids=50301236&dopt=GenPept&RID=T27VS6KG01S&log$=prottop&blast_rank=36) | forkhead box J1 [Homo sapiens] | [17.6](http://blast.ncbi.nlm.nih.gov/Blast.cgi#50301236%2350301236) | 17.6 | 66% | 521 |
| [NP_542185.2](http://www.ncbi.nlm.nih.gov/entrez/query.fcgi?cmd=Retrieve&db=Protein&list_uids=29570785&dopt=GenPept&RID=T27VS6KG01S&log$=prottop&blast_rank=37) | CCCTC-binding factor-like protein [Homo sapiens] | [17.6](http://blast.ncbi.nlm.nih.gov/Blast.cgi#29570785%2329570785) | 17.6 | 66% | 521 |
| [NP_653252.3](http://www.ncbi.nlm.nih.gov/entrez/query.fcgi?cmd=Retrieve&db=Protein&list_uids=126517478&dopt=GenPept&RID=T27VS6KG01S&log$=prottop&blast_rank=38) | peroxidasin homolog-like precursor [Homo sapiens] | [17.2](http://blast.ncbi.nlm.nih.gov/Blast.cgi#126517478%23126517478) | 17.2 | 83% | 699 |
| [NP_003292.1](http://www.ncbi.nlm.nih.gov/entrez/query.fcgi?cmd=Retrieve&db=Protein&list_uids=4507681&dopt=GenPept&RID=T27VS6KG01S&log$=prottop&blast_rank=39) | thyrotropin-releasing hormone receptor [Homo sapiens] | [17.2](http://blast.ncbi.nlm.nih.gov/Blast.cgi#4507681%234507681) | 17.2 | 83% | 699 |
| [NP_003914.1](http://www.ncbi.nlm.nih.gov/entrez/query.fcgi?cmd=Retrieve&db=Protein&list_uids=4503657&dopt=GenPept&RID=T27VS6KG01S&log$=prottop&blast_rank=40) | forkhead box H1 [Homo sapiens] | [17.2](http://blast.ncbi.nlm.nih.gov/Blast.cgi#4503657%234503657) | 17.2 | 83% | 699 |
| [NP_004715.1](http://www.ncbi.nlm.nih.gov/entrez/query.fcgi?cmd=Retrieve&db=Protein&list_uids=4759344&dopt=GenPept&RID=T27VS6KG01S&log$=prottop&blast_rank=41) | centromere/kinetochore protein zw10 [Homo sapiens] | [17.2](http://blast.ncbi.nlm.nih.gov/Blast.cgi#4759344%234759344) | 17.2 | 100% | 699 |
| [NP_055990.1](http://www.ncbi.nlm.nih.gov/entrez/query.fcgi?cmd=Retrieve&db=Protein&list_uids=149944548&dopt=GenPept&RID=T27VS6KG01S&log$=prottop&blast_rank=42) | neurobeachin-like 2 [Homo sapiens] | [16.8](http://blast.ncbi.nlm.nih.gov/Blast.cgi#149944548%23149944548) | 16.8 | 83% | 938 |
| [NP_733751.2](http://www.ncbi.nlm.nih.gov/entrez/query.fcgi?cmd=Retrieve&db=Protein&list_uids=91718902&dopt=GenPept&RID=T27VS6KG01S&log$=prottop&blast_rank=43) | myeloid/lymphoid or mixed-lineage leukemia 3 [Homo sapiens] | [16.8](http://blast.ncbi.nlm.nih.gov/Blast.cgi#91718902%2391718902) | 16.8 | 83% | 938 |
| [NP_036247.1](http://www.ncbi.nlm.nih.gov/entrez/query.fcgi?cmd=Retrieve&db=Protein&list_uids=6912288&dopt=GenPept&RID=T27VS6KG01S&log$=prottop&blast_rank=44) | caspase 8 associated protein 2 [Homo sapiens] >ref|NP_001131140.1| caspase 8 associated protein 2 [Homo sapiens] >ref|NP_001131139.1| caspase 8 associated protein 2 [Homo sapiens] | [16.8](http://blast.ncbi.nlm.nih.gov/Blast.cgi#6912288%236912288) | 16.8 | 83% | 938 |
| [XP_002346601.1](http://www.ncbi.nlm.nih.gov/entrez/query.fcgi?cmd=Retrieve&db=Protein&list_uids=239748256&dopt=GenPept&RID=T27VS6KG01S&log$=prottop&blast_rank=45) | PREDICTED: hypothetical protein XP_002346601 [Homo sapiens] | [16.3](http://blast.ncbi.nlm.nih.gov/Blast.cgi#239748256%23239748256) | 16.3 | 66% | 1258 |
| [XP_001718573.2](http://www.ncbi.nlm.nih.gov/entrez/query.fcgi?cmd=Retrieve&db=Protein&list_uids=239746385&dopt=GenPept&RID=T27VS6KG01S&log$=prottop&blast_rank=46) | PREDICTED: hypothetical protein [Homo sapiens] >ref|XP_001718621.2| PREDICTED: hypothetical protein [Homo sapiens] >ref|XP_001719190.2| PREDICTED: hypothetical protein [Homo sapiens] | [16.3](http://blast.ncbi.nlm.nih.gov/Blast.cgi#239746385%23239746385) | 16.3 | 66% | 1258 |
| [XP_002342443.1](http://www.ncbi.nlm.nih.gov/entrez/query.fcgi?cmd=Retrieve&db=Protein&list_uids=239742157&dopt=GenPept&RID=T27VS6KG01S&log$=prottop&blast_rank=47) | PREDICTED: hypothetical protein XP_002342443 [Homo sapiens] >ref|XP_002345757.1| PREDICTED: hypothetical protein [Homo sapiens] | [16.3](http://blast.ncbi.nlm.nih.gov/Blast.cgi#239742157%23239742157) | 16.3 | 66% | 1258 |
| [XP_932420.2](http://www.ncbi.nlm.nih.gov/entrez/query.fcgi?cmd=Retrieve&db=Protein&list_uids=169167503&dopt=GenPept&RID=T27VS6KG01S&log$=prottop&blast_rank=48) | PREDICTED: hypothetical protein [Homo sapiens] >ref|XP_942481.2| PREDICTED: hypothetical protein [Homo sapiens] >ref|XP_001716958.1| PREDICTED: hypothetical protein [Homo sapiens] | [16.3](http://blast.ncbi.nlm.nih.gov/Blast.cgi#169167503%23169167503) | 16.3 | 66% | 1258 |
| [NP_004678.3](http://www.ncbi.nlm.nih.gov/entrez/query.fcgi?cmd=Retrieve&db=Protein&list_uids=217272865&dopt=GenPept&RID=T27VS6KG01S&log$=prottop&blast_rank=49) | myotubularin related protein 4 [Homo sapiens] | [16.3](http://blast.ncbi.nlm.nih.gov/Blast.cgi#217272865%23217272865) | 16.3 | 66% | 1258 |
| [NP_078997.3](http://www.ncbi.nlm.nih.gov/entrez/query.fcgi?cmd=Retrieve&db=Protein&list_uids=126090663&dopt=GenPept&RID=T27VS6KG01S&log$=prottop&blast_rank=50) | zinc finger homeodomain 4 [Homo sapiens] | [16.3](http://blast.ncbi.nlm.nih.gov/Blast.cgi#126090663%23126090663) | 16.3 | 66% | 1258 |
| [NP_653316.3](http://www.ncbi.nlm.nih.gov/entrez/query.fcgi?cmd=Retrieve&db=Protein&list_uids=148613876&dopt=GenPept&RID=T27VS6KG01S&log$=prottop&blast_rank=51) | EF hand domain family, member B [Homo sapiens] | [16.3](http://blast.ncbi.nlm.nih.gov/Blast.cgi#148613876%23148613876) | 16.3 | 66% | 1258 |
| [NP_001123475.1](http://www.ncbi.nlm.nih.gov/entrez/query.fcgi?cmd=Retrieve&db=Protein&list_uids=194018518&dopt=GenPept&RID=T27VS6KG01S&log$=prottop&blast_rank=52) | synaptoporin isoform 1 [Homo sapiens] | [16.3](http://blast.ncbi.nlm.nih.gov/Blast.cgi#194018518%23194018518) | 16.3 | 66% | 1258 |
| [NP_391988.1](http://www.ncbi.nlm.nih.gov/entrez/query.fcgi?cmd=Retrieve&db=Protein&list_uids=19743819&dopt=GenPept&RID=T27VS6KG01S&log$=prottop&blast_rank=53) | integrin beta 1 isoform 1D precursor [Homo sapiens] | [16.3](http://blast.ncbi.nlm.nih.gov/Blast.cgi#19743819%2319743819) | 16.3 | 66% | 1258 |
| [NP_037382.2](http://www.ncbi.nlm.nih.gov/entrez/query.fcgi?cmd=Retrieve&db=Protein&list_uids=93004074&dopt=GenPept&RID=T27VS6KG01S&log$=prottop&blast_rank=54) | zinc finger protein 215 [Homo sapiens] | [16.3](http://blast.ncbi.nlm.nih.gov/Blast.cgi#93004074%2393004074) | 16.3 | 66% | 1258 |
| [NP_389647.1](http://www.ncbi.nlm.nih.gov/entrez/query.fcgi?cmd=Retrieve&db=Protein&list_uids=19743815&dopt=GenPept&RID=T27VS6KG01S&log$=prottop&blast_rank=55) | integrin beta 1 isoform 1B precursor [Homo sapiens] | [16.3](http://blast.ncbi.nlm.nih.gov/Blast.cgi#19743815%2319743815) | 16.3 | 66% | 1258 |
| [NP_008820.2](http://www.ncbi.nlm.nih.gov/entrez/query.fcgi?cmd=Retrieve&db=Protein&list_uids=91208433&dopt=GenPept&RID=T27VS6KG01S&log$=prottop&blast_rank=56) | CD86 antigen isoform 2 precursor [Homo sapiens] | [16.3](http://blast.ncbi.nlm.nih.gov/Blast.cgi#91208433%2391208433) | 16.3 | 100% | 1258 |
| [NP_391987.1](http://www.ncbi.nlm.nih.gov/entrez/query.fcgi?cmd=Retrieve&db=Protein&list_uids=19743817&dopt=GenPept&RID=T27VS6KG01S&log$=prottop&blast_rank=57) | integrin beta 1 isoform 1C-1 precursor [Homo sapiens] | [16.3](http://blast.ncbi.nlm.nih.gov/Blast.cgi#19743817%2319743817) | 16.3 | 66% | 1258 |
| [NP_391989.1](http://www.ncbi.nlm.nih.gov/entrez/query.fcgi?cmd=Retrieve&db=Protein&list_uids=19743821&dopt=GenPept&RID=T27VS6KG01S&log$=prottop&blast_rank=58) | integrin beta 1 isoform 1C-2 precursor [Homo sapiens] | [16.3](http://blast.ncbi.nlm.nih.gov/Blast.cgi#19743821%2319743821) | 16.3 | 66% | 1258 |
| [NP_787058.3](http://www.ncbi.nlm.nih.gov/entrez/query.fcgi?cmd=Retrieve&db=Protein&list_uids=91208430&dopt=GenPept&RID=T27VS6KG01S&log$=prottop&blast_rank=59) | CD86 antigen isoform 1 [Homo sapiens] | [16.3](http://blast.ncbi.nlm.nih.gov/Blast.cgi#91208430%2391208430) | 16.3 | 100% | 1258 |
| [NP_001804.2](http://www.ncbi.nlm.nih.gov/entrez/query.fcgi?cmd=Retrieve&db=Protein&list_uids=71061468&dopt=GenPept&RID=T27VS6KG01S&log$=prottop&blast_rank=60) | centromere protein E [Homo sapiens] | [16.3](http://blast.ncbi.nlm.nih.gov/Blast.cgi#71061468%2371061468) | 16.3 | 66% | 1258 |
| [NP_689976.2](http://www.ncbi.nlm.nih.gov/entrez/query.fcgi?cmd=Retrieve&db=Protein&list_uids=91754185&dopt=GenPept&RID=T27VS6KG01S&log$=prottop&blast_rank=61) | AKNA domain containing 1 [Homo sapiens] | [16.3](http://blast.ncbi.nlm.nih.gov/Blast.cgi#91754185%2391754185) | 16.3 | 66% | 1258 |
| [NP_942152.1](http://www.ncbi.nlm.nih.gov/entrez/query.fcgi?cmd=Retrieve&db=Protein&list_uids=38569447&dopt=GenPept&RID=T27VS6KG01S&log$=prottop&blast_rank=62) | zinc finger protein 211 isoform 2 [Homo sapiens] | [16.3](http://blast.ncbi.nlm.nih.gov/Blast.cgi#38569447%2338569447) | 16.3 | 66% | 1258 |
| [NP_006376.2](http://www.ncbi.nlm.nih.gov/entrez/query.fcgi?cmd=Retrieve&db=Protein&list_uids=38569445&dopt=GenPept&RID=T27VS6KG01S&log$=prottop&blast_rank=63) | zinc finger protein 211 isoform 1 [Homo sapiens] | [16.3](http://blast.ncbi.nlm.nih.gov/Blast.cgi#38569445%2338569445) | 16.3 | 66% | 1258 |
| [NP_733829.1](http://www.ncbi.nlm.nih.gov/entrez/query.fcgi?cmd=Retrieve&db=Protein&list_uids=25470890&dopt=GenPept&RID=T27VS6KG01S&log$=prottop&blast_rank=64) | DAZ associated protein 1 isoform a [Homo sapiens] | [16.3](http://blast.ncbi.nlm.nih.gov/Blast.cgi#25470890%2325470890) | 16.3 | 100% | 1258 |
| [NP_002202.2](http://www.ncbi.nlm.nih.gov/entrez/query.fcgi?cmd=Retrieve&db=Protein&list_uids=19743813&dopt=GenPept&RID=T27VS6KG01S&log$=prottop&blast_rank=65) | integrin beta 1 isoform 1A precursor [Homo sapiens] >ref|NP_596867.1| integrin beta 1 isoform 1A precursor [Homo sapiens] | [16.3](http://blast.ncbi.nlm.nih.gov/Blast.cgi#19743813%2319743813) | 16.3 | 66% | 1258 |
| [NP_848586.1](http://www.ncbi.nlm.nih.gov/entrez/query.fcgi?cmd=Retrieve&db=Protein&list_uids=30425410&dopt=GenPept&RID=T27VS6KG01S&log$=prottop&blast_rank=66) | R3H domain containing-like precursor [Homo sapiens] | [16.3](http://blast.ncbi.nlm.nih.gov/Blast.cgi#30425410%2330425410) | 31.4 | 100% | 1258 |
| [NP_149350.3](http://www.ncbi.nlm.nih.gov/entrez/query.fcgi?cmd=Retrieve&db=Protein&list_uids=55769537&dopt=GenPept&RID=T27VS6KG01S&log$=prottop&blast_rank=67) | zinc finger protein 658 [Homo sapiens] | [16.3](http://blast.ncbi.nlm.nih.gov/Blast.cgi#55769537%2355769537) | 16.3 | 66% | 1258 |
| [NP_006528.2](http://www.ncbi.nlm.nih.gov/entrez/query.fcgi?cmd=Retrieve&db=Protein&list_uids=55770886&dopt=GenPept&RID=T27VS6KG01S&log$=prottop&blast_rank=68) | ubiquitin thiolesterase 3 [Homo sapiens] | [16.3](http://blast.ncbi.nlm.nih.gov/Blast.cgi#55770886%2355770886) | 16.3 | 66% | 1258 |
| [NP_998784.1](http://www.ncbi.nlm.nih.gov/entrez/query.fcgi?cmd=Retrieve&db=Protein&list_uids=47717100&dopt=GenPept&RID=T27VS6KG01S&log$=prottop&blast_rank=69) | ATPase, H+ transporting, lysosomal 50/57kDa, V1 subunit H isoform 2 [Homo sapiens] | [16.3](http://blast.ncbi.nlm.nih.gov/Blast.cgi#47717100%2347717100) | 16.3 | 66% | 1258 |
| [NP_001019782.1](http://www.ncbi.nlm.nih.gov/entrez/query.fcgi?cmd=Retrieve&db=Protein&list_uids=66912176&dopt=GenPept&RID=T27VS6KG01S&log$=prottop&blast_rank=70) | leucine rich repeat containing 66 [Homo sapiens] | [16.3](http://blast.ncbi.nlm.nih.gov/Blast.cgi#66912176%2366912176) | 16.3 | 100% | 1258 |
| [NP_997228.1](http://www.ncbi.nlm.nih.gov/entrez/query.fcgi?cmd=Retrieve&db=Protein&list_uids=46409326&dopt=GenPept&RID=T27VS6KG01S&log$=prottop&blast_rank=71) | C-type lectin domain family 9, member A [Homo sapiens] | [16.3](http://blast.ncbi.nlm.nih.gov/Blast.cgi#46409326%2346409326) | 16.3 | 66% | 1258 |
| [NP_000889.3](http://www.ncbi.nlm.nih.gov/entrez/query.fcgi?cmd=Retrieve&db=Protein&list_uids=38202207&dopt=GenPept&RID=T27VS6KG01S&log$=prottop&blast_rank=72) | monoamine oxidase B [Homo sapiens] | [16.3](http://blast.ncbi.nlm.nih.gov/Blast.cgi#38202207%2338202207) | 16.3 | 66% | 1258 |
| [NP_700359.1](http://www.ncbi.nlm.nih.gov/entrez/query.fcgi?cmd=Retrieve&db=Protein&list_uids=23510455&dopt=GenPept&RID=T27VS6KG01S&log$=prottop&blast_rank=73) | zinc finger protein 41 [Homo sapiens] >ref|NP_009061.1| zinc finger protein 41 [Homo sapiens] | [16.3](http://blast.ncbi.nlm.nih.gov/Blast.cgi#23510455%2323510455) | 16.3 | 100% | 1258 |
| [NP_061832.2](http://www.ncbi.nlm.nih.gov/entrez/query.fcgi?cmd=Retrieve&db=Protein&list_uids=25470886&dopt=GenPept&RID=T27VS6KG01S&log$=prottop&blast_rank=74) | DAZ associated protein 1 isoform b [Homo sapiens] | [16.3](http://blast.ncbi.nlm.nih.gov/Blast.cgi#25470886%2325470886) | 16.3 | 100% | 1258 |
| [NP_002524.2](http://www.ncbi.nlm.nih.gov/entrez/query.fcgi?cmd=Retrieve&db=Protein&list_uids=45643123&dopt=GenPept&RID=T27VS6KG01S&log$=prottop&blast_rank=75) | nuclear VCP-like isoform 1 [Homo sapiens] | [16.3](http://blast.ncbi.nlm.nih.gov/Blast.cgi#45643123%2345643123) | 16.3 | 66% | 1258 |
| [NP_000123.1](http://www.ncbi.nlm.nih.gov/entrez/query.fcgi?cmd=Retrieve&db=Protein&list_uids=4503647&dopt=GenPept&RID=T27VS6KG01S&log$=prottop&blast_rank=76) | coagulation factor VIII isoform a precursor [Homo sapiens] | [16.3](http://blast.ncbi.nlm.nih.gov/Blast.cgi#4503647%234503647) | 16.3 | 66% | 1258 |
| [NP_004460.1](http://www.ncbi.nlm.nih.gov/entrez/query.fcgi?cmd=Retrieve&db=Protein&list_uids=4758378&dopt=GenPept&RID=T27VS6KG01S&log$=prottop&blast_rank=77) | vascular endothelial growth factor D preproprotein [Homo sapiens] | [16.3](http://blast.ncbi.nlm.nih.gov/Blast.cgi#4758378%234758378) | 16.3 | 66% | 1258 |
| [NP_006707.1](http://www.ncbi.nlm.nih.gov/entrez/query.fcgi?cmd=Retrieve&db=Protein&list_uids=5729734&dopt=GenPept&RID=T27VS6KG01S&log$=prottop&blast_rank=78) | activator of S phase kinase [Homo sapiens] | [16.3](http://blast.ncbi.nlm.nih.gov/Blast.cgi#5729734%235729734) | 16.3 | 66% | 1258 |
| [NP_543152.1](http://www.ncbi.nlm.nih.gov/entrez/query.fcgi?cmd=Retrieve&db=Protein&list_uids=18254478&dopt=GenPept&RID=T27VS6KG01S&log$=prottop&blast_rank=79) | dual specificity phosphatase 19 isoform 1 [Homo sapiens] | [16.3](http://blast.ncbi.nlm.nih.gov/Blast.cgi#18254478%2318254478) | 16.3 | 66% | 1258 |
| [NP_653243.1](http://www.ncbi.nlm.nih.gov/entrez/query.fcgi?cmd=Retrieve&db=Protein&list_uids=21389481&dopt=GenPept&RID=T27VS6KG01S&log$=prottop&blast_rank=80) | synaptoporin isoform 2 [Homo sapiens] | [16.3](http://blast.ncbi.nlm.nih.gov/Blast.cgi#21389481%2321389481) | 16.3 | 66% | 1258 |
| [NP_001135786.1](http://www.ncbi.nlm.nih.gov/entrez/query.fcgi?cmd=Retrieve&db=Protein&list_uids=214832050&dopt=GenPept&RID=T27VS6KG01S&log$=prottop&blast_rank=81) | dual specificity phosphatase 19 isoform 2 [Homo sapiens] | [16.3](http://blast.ncbi.nlm.nih.gov/Blast.cgi#214832050%23214832050) | 16.3 | 66% | 1258 |
| [NP_998785.1](http://www.ncbi.nlm.nih.gov/entrez/query.fcgi?cmd=Retrieve&db=Protein&list_uids=47717102&dopt=GenPept&RID=T27VS6KG01S&log$=prottop&blast_rank=82) | ATPase, H+ transporting, lysosomal 50/57kDa, V1 subunit H isoform 1 [Homo sapiens] >ref|NP_057025.2| ATPase, H+ transporting, lysosomal 50/57kDa, V1 subunit H isoform 1 [Homo sapiens] | [16.3](http://blast.ncbi.nlm.nih.gov/Blast.cgi#47717102%2347717102) | 16.3 | 66% | 1258 |
| [NP_004051.1](http://www.ncbi.nlm.nih.gov/entrez/query.fcgi?cmd=Retrieve&db=Protein&list_uids=4757934&dopt=GenPept&RID=T27VS6KG01S&log$=prottop&blast_rank=83) | cyclin G1 [Homo sapiens] >ref|NP_954854.1| cyclin G1 [Homo sapiens] | [16.3](http://blast.ncbi.nlm.nih.gov/Blast.cgi#4757934%234757934) | 16.3 | 66% | 1258 |
| [XP_002348289.1](http://www.ncbi.nlm.nih.gov/entrez/query.fcgi?cmd=Retrieve&db=Protein&list_uids=239752477&dopt=GenPept&RID=T27VS6KG01S&log$=prottop&blast_rank=84) | PREDICTED: similar to zinc finger protein 208 [Homo sapiens] | [15.9](http://blast.ncbi.nlm.nih.gov/Blast.cgi#239752477%23239752477) | 30.1 | 100% | 1689 |
| [XP_002343284.1](http://www.ncbi.nlm.nih.gov/entrez/query.fcgi?cmd=Retrieve&db=Protein&list_uids=239744840&dopt=GenPept&RID=T27VS6KG01S&log$=prottop&blast_rank=85) | PREDICTED: hypothetical protein XP_002343284 [Homo sapiens] >ref|XP_002347436.1| PREDICTED: hypothetical protein XP_002347436 [Homo sapiens] >ref|XP_002344777.1| PREDICTED: hypothetical protein [Homo sapiens] | [15.9](http://blast.ncbi.nlm.nih.gov/Blast.cgi#239744840%23239744840) | 15.9 | 100% | 1689 |
| [XP_002343641.1](http://www.ncbi.nlm.nih.gov/entrez/query.fcgi?cmd=Retrieve&db=Protein&list_uids=239746079&dopt=GenPept&RID=T27VS6KG01S&log$=prottop&blast_rank=86) | PREDICTED: hypothetical protein XP_002343641 [Homo sapiens] >ref|XP_002347907.1| PREDICTED: hypothetical protein [Homo sapiens] >ref|XP_002345155.1| PREDICTED: hypothetical protein [Homo sapiens] | [15.9](http://blast.ncbi.nlm.nih.gov/Blast.cgi#239746079%23239746079) | 15.9 | 83% | 1689 |
| [NP_940859.2](http://www.ncbi.nlm.nih.gov/entrez/query.fcgi?cmd=Retrieve&db=Protein&list_uids=156766086&dopt=GenPept&RID=T27VS6KG01S&log$=prottop&blast_rank=87) | zinc finger protein 600 [Homo sapiens] | [15.9](http://blast.ncbi.nlm.nih.gov/Blast.cgi#156766086%23156766086) | 44.3 | 83% | 1689 |
| [NP_073579.3](http://www.ncbi.nlm.nih.gov/entrez/query.fcgi?cmd=Retrieve&db=Protein&list_uids=115511012&dopt=GenPept&RID=T27VS6KG01S&log$=prottop&blast_rank=88) | coiled-coil domain containing 136 [Homo sapiens] | [15.9](http://blast.ncbi.nlm.nih.gov/Blast.cgi#115511012%23115511012) | 15.9 | 83% | 1689 |
| [NP_001028725.1](http://www.ncbi.nlm.nih.gov/entrez/query.fcgi?cmd=Retrieve&db=Protein&list_uids=75750472&dopt=GenPept&RID=T27VS6KG01S&log$=prottop&blast_rank=89) | spectrin domain with coiled-coils 1 NSP5b3b [Homo sapiens] | [15.9](http://blast.ncbi.nlm.nih.gov/Blast.cgi#75750472%2375750472) | 15.9 | 83% | 1689 |
| [NP_001028726.1](http://www.ncbi.nlm.nih.gov/entrez/query.fcgi?cmd=Retrieve&db=Protein&list_uids=75750468&dopt=GenPept&RID=T27VS6KG01S&log$=prottop&blast_rank=90) | spectrin domain with coiled-coils 1 NSP5a3a [Homo sapiens] | [15.9](http://blast.ncbi.nlm.nih.gov/Blast.cgi#75750468%2375750468) | 15.9 | 83% | 1689 |
| [NP_690868.3](http://www.ncbi.nlm.nih.gov/entrez/query.fcgi?cmd=Retrieve&db=Protein&list_uids=75750466&dopt=GenPept&RID=T27VS6KG01S&log$=prottop&blast_rank=91) | spectrin domain with coiled-coils 1 NSP5b3a [Homo sapiens] | [15.9](http://blast.ncbi.nlm.nih.gov/Blast.cgi#75750466%2375750466) | 15.9 | 83% | 1689 |
| [NP_001028727.1](http://www.ncbi.nlm.nih.gov/entrez/query.fcgi?cmd=Retrieve&db=Protein&list_uids=75750474&dopt=GenPept&RID=T27VS6KG01S&log$=prottop&blast_rank=92) | spectrin domain with coiled-coils 1 NSP5a3b [Homo sapiens] | [15.9](http://blast.ncbi.nlm.nih.gov/Blast.cgi#75750474%2375750474) | 15.9 | 83% | 1689 |
| [NP_001726.2](http://www.ncbi.nlm.nih.gov/entrez/query.fcgi?cmd=Retrieve&db=Protein&list_uids=38016947&dopt=GenPept&RID=T27VS6KG01S&log$=prottop&blast_rank=93) | complement component 5 preproprotein [Homo sapiens] | [15.9](http://blast.ncbi.nlm.nih.gov/Blast.cgi#38016947%2338016947) | 15.9 | 83% | 1689 |
| [NP_112494.3](http://www.ncbi.nlm.nih.gov/entrez/query.fcgi?cmd=Retrieve&db=Protein&list_uids=148612831&dopt=GenPept&RID=T27VS6KG01S&log$=prottop&blast_rank=94) | kinesin family member 18A [Homo sapiens] | [15.9](http://blast.ncbi.nlm.nih.gov/Blast.cgi#148612831%23148612831) | 15.9 | 83% | 1689 |
| [NP_008894.2](http://www.ncbi.nlm.nih.gov/entrez/query.fcgi?cmd=Retrieve&db=Protein&list_uids=42714596&dopt=GenPept&RID=T27VS6KG01S&log$=prottop&blast_rank=95) | zinc finger protein 22 (KOX 15) [Homo sapiens] | [15.9](http://blast.ncbi.nlm.nih.gov/Blast.cgi#42714596%2342714596) | 15.9 | 83% | 1689 |
| [NP_663161.1](http://www.ncbi.nlm.nih.gov/entrez/query.fcgi?cmd=Retrieve&db=Protein&list_uids=21735582&dopt=GenPept&RID=T27VS6KG01S&log$=prottop&blast_rank=96) | oxysterol-binding protein-like protein 3 isoform c [Homo sapiens] | [15.9](http://blast.ncbi.nlm.nih.gov/Blast.cgi#21735582%2321735582) | 15.9 | 83% | 1689 |
| [NP_056365.1](http://www.ncbi.nlm.nih.gov/entrez/query.fcgi?cmd=Retrieve&db=Protein&list_uids=14149704&dopt=GenPept&RID=T27VS6KG01S&log$=prottop&blast_rank=97) | oxysterol-binding protein-like protein 3 isoform a [Homo sapiens] | [15.9](http://blast.ncbi.nlm.nih.gov/Blast.cgi#14149704%2314149704) | 15.9 | 83% | 1689 |
| [NP_663160.1](http://www.ncbi.nlm.nih.gov/entrez/query.fcgi?cmd=Retrieve&db=Protein&list_uids=21735580&dopt=GenPept&RID=T27VS6KG01S&log$=prottop&blast_rank=98) | oxysterol-binding protein-like protein 3 isoform b [Homo sapiens] | [15.9](http://blast.ncbi.nlm.nih.gov/Blast.cgi#21735580%2321735580) | 15.9 | 83% | 1689 |
| [NP_663162.1](http://www.ncbi.nlm.nih.gov/entrez/query.fcgi?cmd=Retrieve&db=Protein&list_uids=21735584&dopt=GenPept&RID=T27VS6KG01S&log$=prottop&blast_rank=99) | oxysterol-binding protein-like protein 3 isoform d [Homo sapiens] | [15.9](http://blast.ncbi.nlm.nih.gov/Blast.cgi#21735584%2321735584) | 15.9 | 83% | 1689 |
| [XP_002342520.1](http://www.ncbi.nlm.nih.gov/entrez/query.fcgi?cmd=Retrieve&db=Protein&list_uids=239742456&dopt=GenPept&RID=T27VS6KG01S&log$=prottop&blast_rank=100) | PREDICTED: hypothetical protein XP_002342520 [Homo sapiens] >ref|XP_002346675.1| PREDICTED: hypothetical protein XP_002346675 [Homo sapiens] >ref|XP_002345822.1| PREDICTED: hypothetical protein [Homo sapiens] | [15.5](http://blast.ncbi.nlm.nih.gov/Blast.cgi#239742456%23239742456) | 15.5 | 83% | 2266 |

| **Accession** | **Proteins with a match to MKQSGHHRSE peptide** | **[Max score](http://blast.ncbi.nlm.nih.gov/Blast.cgi?CMD=Get&ALIGNMENTS=100&ALIGNMENT_VIEW=Pairwise&CDD_SEARCH_STATE=1&DATABASE_SORT=0&DESCRIPTIONS=100&ENTREZ_QUERY=txid9606 %5BORGN%5D&FIRST_QUERY_NUM=0&FORMAT_OBJECT=Alignment&FORMAT_PAGE_TARGET=&FORMAT_TYPE=HTML&GET_SEQUENCE=yes&I_THRESH=&MASK_CHAR=2&MASK_COLOR=1&NEW_DESIGN=on&NEW_VIEW=yes&NUM_OVERVIEW=100&OLD_BLAST=false&PAGE=Proteins&QUERY_INDEX=0&QUERY_NUMBER=0&RESULTS_PAGE_TARGET=&RID=T280CBCZ01N&SHOW_LINKOUT=yes&SHOW_OVERVIEW=yes&STEP_NUMBER=&WORD_SIZE=2&DISPLAY_SORT=1&HSP_SORT=1" \l "sort_mark)** | **[Total score](http://blast.ncbi.nlm.nih.gov/Blast.cgi?CMD=Get&ALIGNMENTS=100&ALIGNMENT_VIEW=Pairwise&CDD_SEARCH_STATE=1&DATABASE_SORT=0&DESCRIPTIONS=100&ENTREZ_QUERY=txid9606 %5BORGN%5D&FIRST_QUERY_NUM=0&FORMAT_OBJECT=Alignment&FORMAT_PAGE_TARGET=&FORMAT_TYPE=HTML&GET_SEQUENCE=yes&I_THRESH=&MASK_CHAR=2&MASK_COLOR=1&NEW_DESIGN=on&NEW_VIEW=yes&NUM_OVERVIEW=100&OLD_BLAST=false&PAGE=Proteins&QUERY_INDEX=0&QUERY_NUMBER=0&RESULTS_PAGE_TARGET=&RID=T280CBCZ01N&SHOW_LINKOUT=yes&SHOW_OVERVIEW=yes&STEP_NUMBER=&WORD_SIZE=2&DISPLAY_SORT=2&HSP_SORT=1" \l "sort_mark)** | **[Query coverage](http://blast.ncbi.nlm.nih.gov/Blast.cgi?CMD=Get&ALIGNMENTS=100&ALIGNMENT_VIEW=Pairwise&CDD_SEARCH_STATE=1&DATABASE_SORT=0&DESCRIPTIONS=100&ENTREZ_QUERY=txid9606 %5BORGN%5D&FIRST_QUERY_NUM=0&FORMAT_OBJECT=Alignment&FORMAT_PAGE_TARGET=&FORMAT_TYPE=HTML&GET_SEQUENCE=yes&I_THRESH=&MASK_CHAR=2&MASK_COLOR=1&NEW_DESIGN=on&NEW_VIEW=yes&NUM_OVERVIEW=100&OLD_BLAST=false&PAGE=Proteins&QUERY_INDEX=0&QUERY_NUMBER=0&RESULTS_PAGE_TARGET=&RID=T280CBCZ01N&SHOW_LINKOUT=yes&SHOW_OVERVIEW=yes&STEP_NUMBER=&WORD_SIZE=2&DISPLAY_SORT=4&HSP_SORT=0" \l "sort_mark)** | **[E value](http://blast.ncbi.nlm.nih.gov/Blast.cgi?CMD=Get&ALIGNMENTS=100&ALIGNMENT_VIEW=Pairwise&CDD_SEARCH_STATE=1&DATABASE_SORT=0&DESCRIPTIONS=100&ENTREZ_QUERY=txid9606 %5BORGN%5D&FIRST_QUERY_NUM=0&FORMAT_OBJECT=Alignment&FORMAT_PAGE_TARGET=&FORMAT_TYPE=HTML&GET_SEQUENCE=yes&I_THRESH=&MASK_CHAR=2&MASK_COLOR=1&NEW_DESIGN=on&NEW_VIEW=yes&NUM_OVERVIEW=100&OLD_BLAST=false&PAGE=Proteins&QUERY_INDEX=0&QUERY_NUMBER=0&RESULTS_PAGE_TARGET=&RID=T280CBCZ01N&SHOW_LINKOUT=yes&SHOW_OVERVIEW=yes&STEP_NUMBER=&WORD_SIZE=2&DISPLAY_SORT=0&HSP_SORT=0" \l "sort_mark)** |
| --- | --- | --- | --- | --- | --- |
| [NP_004642.2](http://www.ncbi.nlm.nih.gov/entrez/query.fcgi?cmd=Retrieve&db=Protein&list_uids=24234683&dopt=GenPept&RID=T280CBCZ01N&log$=prottop&blast_rank=1) | ubiquitin specific peptidase 11 [Homo sapiens] | [23.5](http://blast.ncbi.nlm.nih.gov/Blast.cgi#24234683%2324234683) | 23.5 | 80% | 14 |
| [NP_060154.3](http://www.ncbi.nlm.nih.gov/entrez/query.fcgi?cmd=Retrieve&db=Protein&list_uids=42544121&dopt=GenPept&RID=T280CBCZ01N&log$=prottop&blast_rank=2) | vacuolar protein sorting 13C protein isoform 1A [Homo sapiens] | [21.4](http://blast.ncbi.nlm.nih.gov/Blast.cgi#42544121%2342544121) | 52.8 | 90% | 62 |
| [NP_060550.2](http://www.ncbi.nlm.nih.gov/entrez/query.fcgi?cmd=Retrieve&db=Protein&list_uids=66348091&dopt=GenPept&RID=T280CBCZ01N&log$=prottop&blast_rank=3) | vacuolar protein sorting 13C protein isoform 1B [Homo sapiens] | [21.4](http://blast.ncbi.nlm.nih.gov/Blast.cgi#66348091%2366348091) | 36.1 | 80% | 62 |
| [NP_001018098.1](http://www.ncbi.nlm.nih.gov/entrez/query.fcgi?cmd=Retrieve&db=Protein&list_uids=66347845&dopt=GenPept&RID=T280CBCZ01N&log$=prottop&blast_rank=4) | vacuolar protein sorting 13C protein isoform 2B [Homo sapiens] | [21.4](http://blast.ncbi.nlm.nih.gov/Blast.cgi#66347845%2366347845) | 36.1 | 80% | 62 |
| [NP_065872.1](http://www.ncbi.nlm.nih.gov/entrez/query.fcgi?cmd=Retrieve&db=Protein&list_uids=66347828&dopt=GenPept&RID=T280CBCZ01N&log$=prottop&blast_rank=5) | vacuolar protein sorting 13C protein isoform 2A [Homo sapiens] | [21.4](http://blast.ncbi.nlm.nih.gov/Blast.cgi#66347828%2366347828) | 52.8 | 90% | 62 |
| [NP_940905.2](http://www.ncbi.nlm.nih.gov/entrez/query.fcgi?cmd=Retrieve&db=Protein&list_uids=41349443&dopt=GenPept&RID=T280CBCZ01N&log$=prottop&blast_rank=6) | potassium channel, subfamily T, member 2 [Homo sapiens] | [20.6](http://blast.ncbi.nlm.nih.gov/Blast.cgi#41349443%2341349443) | 20.6 | 80% | 111 |
| [NP_002940.2](http://www.ncbi.nlm.nih.gov/entrez/query.fcgi?cmd=Retrieve&db=Protein&list_uids=27436901&dopt=GenPept&RID=T280CBCZ01N&log$=prottop&blast_rank=7) | mitochondrial ribosomal protein L12 precursor [Homo sapiens] | [20.6](http://blast.ncbi.nlm.nih.gov/Blast.cgi#27436901%2327436901) | 20.6 | 100% | 111 |
| [NP_001137459.1](http://www.ncbi.nlm.nih.gov/entrez/query.fcgi?cmd=Retrieve&db=Protein&list_uids=221136844&dopt=GenPept&RID=T280CBCZ01N&log$=prottop&blast_rank=8) | neuroblastoma breakpoint family, member 6 isoform 1 [Homo sapiens] | [20.2](http://blast.ncbi.nlm.nih.gov/Blast.cgi#221136844%23221136844) | 20.2 | 50% | 149 |
| [XP_001715524.1](http://www.ncbi.nlm.nih.gov/entrez/query.fcgi?cmd=Retrieve&db=Protein&list_uids=169217327&dopt=GenPept&RID=T280CBCZ01N&log$=prottop&blast_rank=9) | PREDICTED: hypothetical protein [Homo sapiens] >ref|XP_001714950.1| PREDICTED: similar to CYorf16 protein [Homo sapiens] | [20.2](http://blast.ncbi.nlm.nih.gov/Blast.cgi#169217327%23169217327) | 20.2 | 80% | 149 |
| [NP_001093640.1](http://www.ncbi.nlm.nih.gov/entrez/query.fcgi?cmd=Retrieve&db=Protein&list_uids=154354966&dopt=GenPept&RID=T280CBCZ01N&log$=prottop&blast_rank=10) | inner membrane protein, mitochondrial isoform 3 [Homo sapiens] | [20.2](http://blast.ncbi.nlm.nih.gov/Blast.cgi#154354966%23154354966) | 20.2 | 50% | 149 |
| [NP_001137460.1](http://www.ncbi.nlm.nih.gov/entrez/query.fcgi?cmd=Retrieve&db=Protein&list_uids=221136848&dopt=GenPept&RID=T280CBCZ01N&log$=prottop&blast_rank=11) | neuroblastoma breakpoint family, member 6 isoform 2 [Homo sapiens] | [20.2](http://blast.ncbi.nlm.nih.gov/Blast.cgi#221136848%23221136848) | 20.2 | 50% | 149 |
| [NP_060301.2](http://www.ncbi.nlm.nih.gov/entrez/query.fcgi?cmd=Retrieve&db=Protein&list_uids=37595555&dopt=GenPept&RID=T280CBCZ01N&log$=prottop&blast_rank=12) | ring finger protein 125 [Homo sapiens] | [20.2](http://blast.ncbi.nlm.nih.gov/Blast.cgi#37595555%2337595555) | 20.2 | 50% | 149 |
| [NP_001093639.1](http://www.ncbi.nlm.nih.gov/entrez/query.fcgi?cmd=Retrieve&db=Protein&list_uids=154354962&dopt=GenPept&RID=T280CBCZ01N&log$=prottop&blast_rank=13) | inner membrane protein, mitochondrial isoform 2 [Homo sapiens] | [20.2](http://blast.ncbi.nlm.nih.gov/Blast.cgi#154354962%23154354962) | 20.2 | 50% | 149 |
| [NP_060286.1](http://www.ncbi.nlm.nih.gov/entrez/query.fcgi?cmd=Retrieve&db=Protein&list_uids=8923398&dopt=GenPept&RID=T280CBCZ01N&log$=prottop&blast_rank=14) | Ly1 antibody reactive homolog [Homo sapiens] >ref|NP_001139197.1| Ly1 antibody reactive homolog [Homo sapiens] | [20.2](http://blast.ncbi.nlm.nih.gov/Blast.cgi#8923398%238923398) | 20.2 | 50% | 149 |
| [NP_006830.2](http://www.ncbi.nlm.nih.gov/entrez/query.fcgi?cmd=Retrieve&db=Protein&list_uids=154354964&dopt=GenPept&RID=T280CBCZ01N&log$=prottop&blast_rank=15) | inner membrane protein, mitochondrial isoform 1 [Homo sapiens] | [20.2](http://blast.ncbi.nlm.nih.gov/Blast.cgi#154354964%23154354964) | 20.2 | 50% | 149 |
| [XP_002346152.1](http://www.ncbi.nlm.nih.gov/entrez/query.fcgi?cmd=Retrieve&db=Protein&list_uids=239754887&dopt=GenPept&RID=T280CBCZ01N&log$=prottop&blast_rank=16) | PREDICTED: hypothetical protein XP_002346152 [Homo sapiens] | [19.3](http://blast.ncbi.nlm.nih.gov/Blast.cgi#239754887%23239754887) | 36.1 | 60% | 268 |
| [XP_002345671.1](http://www.ncbi.nlm.nih.gov/entrez/query.fcgi?cmd=Retrieve&db=Protein&list_uids=239753480&dopt=GenPept&RID=T280CBCZ01N&log$=prottop&blast_rank=17) | PREDICTED: hypothetical protein [Homo sapiens] | [19.3](http://blast.ncbi.nlm.nih.gov/Blast.cgi#239753480%23239753480) | 99.3 | 50% | 268 |
| [XP_002342893.1](http://www.ncbi.nlm.nih.gov/entrez/query.fcgi?cmd=Retrieve&db=Protein&list_uids=239743560&dopt=GenPept&RID=T280CBCZ01N&log$=prottop&blast_rank=18) | PREDICTED: hypothetical protein XP_002342893 [Homo sapiens] >ref|XP_002347042.1| PREDICTED: hypothetical protein XP_002347042 [Homo sapiens] | [19.3](http://blast.ncbi.nlm.nih.gov/Blast.cgi#239743560%23239743560) | 36.1 | 60% | 268 |
| [NP_001138908.1](http://www.ncbi.nlm.nih.gov/entrez/query.fcgi?cmd=Retrieve&db=Protein&list_uids=224177556&dopt=GenPept&RID=T280CBCZ01N&log$=prottop&blast_rank=19) | lanosterol synthase isoform 2 [Homo sapiens] | [19.3](http://blast.ncbi.nlm.nih.gov/Blast.cgi#224177556%23224177556) | 19.3 | 60% | 268 |
| [NP_001138909.1](http://www.ncbi.nlm.nih.gov/entrez/query.fcgi?cmd=Retrieve&db=Protein&list_uids=224177558&dopt=GenPept&RID=T280CBCZ01N&log$=prottop&blast_rank=20) | lanosterol synthase isoform 3 [Homo sapiens] | [19.3](http://blast.ncbi.nlm.nih.gov/Blast.cgi#224177558%23224177558) | 19.3 | 60% | 268 |
| [NP_001019634.1](http://www.ncbi.nlm.nih.gov/entrez/query.fcgi?cmd=Retrieve&db=Protein&list_uids=66932994&dopt=GenPept&RID=T280CBCZ01N&log$=prottop&blast_rank=21) | histidine acid phosphatase domain containing 2A isoform 4 [Homo sapiens] | [19.3](http://blast.ncbi.nlm.nih.gov/Blast.cgi#66932994%2366932994) | 19.3 | 50% | 268 |
| [NP_945324.1](http://www.ncbi.nlm.nih.gov/entrez/query.fcgi?cmd=Retrieve&db=Protein&list_uids=39752689&dopt=GenPept&RID=T280CBCZ01N&log$=prottop&blast_rank=22) | MAP kinase-interacting serine/threonine kinase 1 isoform 2 [Homo sapiens] | [19.3](http://blast.ncbi.nlm.nih.gov/Blast.cgi#39752689%2339752689) | 19.3 | 80% | 268 |
| [NP_955468.1](http://www.ncbi.nlm.nih.gov/entrez/query.fcgi?cmd=Retrieve&db=Protein&list_uids=40806170&dopt=GenPept&RID=T280CBCZ01N&log$=prottop&blast_rank=23) | spastin isoform 2 [Homo sapiens] | [19.3](http://blast.ncbi.nlm.nih.gov/Blast.cgi#40806170%2340806170) | 19.3 | 50% | 268 |
| [NP_055474.3](http://www.ncbi.nlm.nih.gov/entrez/query.fcgi?cmd=Retrieve&db=Protein&list_uids=66932992&dopt=GenPept&RID=T280CBCZ01N&log$=prottop&blast_rank=24) | histidine acid phosphatase domain containing 2A isoform 2 [Homo sapiens] >ref|NP_001124331.1| histidine acid phosphatase domain containing 2A isoform 2 [Homo sapiens] | [19.3](http://blast.ncbi.nlm.nih.gov/Blast.cgi#66932992%2366932992) | 19.3 | 50% | 268 |
| [NP_001034221.1](http://www.ncbi.nlm.nih.gov/entrez/query.fcgi?cmd=Retrieve&db=Protein&list_uids=85068578&dopt=GenPept&RID=T280CBCZ01N&log$=prottop&blast_rank=25) | intercellular adhesion molecule 4 isoform 3 precursor [Homo sapiens] | [19.3](http://blast.ncbi.nlm.nih.gov/Blast.cgi#85068578%2385068578) | 19.3 | 50% | 268 |
| [NP_001124330.1](http://www.ncbi.nlm.nih.gov/entrez/query.fcgi?cmd=Retrieve&db=Protein&list_uids=195947359&dopt=GenPept&RID=T280CBCZ01N&log$=prottop&blast_rank=26) | histidine acid phosphatase domain containing 2A isoform 5 [Homo sapiens] | [19.3](http://blast.ncbi.nlm.nih.gov/Blast.cgi#195947359%23195947359) | 19.3 | 50% | 268 |
| [NP_066999.1](http://www.ncbi.nlm.nih.gov/entrez/query.fcgi?cmd=Retrieve&db=Protein&list_uids=10863975&dopt=GenPept&RID=T280CBCZ01N&log$=prottop&blast_rank=27) | islet-specific glucose-6-phosphatase-related protein isoform 1 [Homo sapiens] | [19.3](http://blast.ncbi.nlm.nih.gov/Blast.cgi#10863975%2310863975) | 19.3 | 50% | 268 |
| [NP_694691.1](http://www.ncbi.nlm.nih.gov/entrez/query.fcgi?cmd=Retrieve&db=Protein&list_uids=23510389&dopt=GenPept&RID=T280CBCZ01N&log$=prottop&blast_rank=28) | myotubularin-related protein 3 isoform b [Homo sapiens] | [19.3](http://blast.ncbi.nlm.nih.gov/Blast.cgi#23510389%2323510389) | 19.3 | 50% | 268 |
| [NP_694690.1](http://www.ncbi.nlm.nih.gov/entrez/query.fcgi?cmd=Retrieve&db=Protein&list_uids=23510387&dopt=GenPept&RID=T280CBCZ01N&log$=prottop&blast_rank=29) | myotubularin-related protein 3 isoform a [Homo sapiens] | [19.3](http://blast.ncbi.nlm.nih.gov/Blast.cgi#23510387%2323510387) | 19.3 | 50% | 268 |
| [NP_001868.2](http://www.ncbi.nlm.nih.gov/entrez/query.fcgi?cmd=Retrieve&db=Protein&list_uids=42544177&dopt=GenPept&RID=T280CBCZ01N&log$=prottop&blast_rank=30) | complement component (3d/Epstein Barr virus) receptor 2 isoform 2 precursor [Homo sapiens] | [19.3](http://blast.ncbi.nlm.nih.gov/Blast.cgi#42544177%2342544177) | 19.3 | 100% | 268 |
| [NP_066576.1](http://www.ncbi.nlm.nih.gov/entrez/query.fcgi?cmd=Retrieve&db=Protein&list_uids=10835109&dopt=GenPept&RID=T280CBCZ01N&log$=prottop&blast_rank=31) | myotubularin-related protein 3 isoform c [Homo sapiens] | [19.3](http://blast.ncbi.nlm.nih.gov/Blast.cgi#10835109%2310835109) | 19.3 | 50% | 268 |
| [NP_116253.2](http://www.ncbi.nlm.nih.gov/entrez/query.fcgi?cmd=Retrieve&db=Protein&list_uids=24762236&dopt=GenPept&RID=T280CBCZ01N&log$=prottop&blast_rank=32) | PRP38 pre-mRNA processing factor 38 (yeast) domain containing A [Homo sapiens] | [19.3](http://blast.ncbi.nlm.nih.gov/Blast.cgi#24762236%2324762236) | 19.3 | 50% | 268 |
| [NP_001006659.1](http://www.ncbi.nlm.nih.gov/entrez/query.fcgi?cmd=Retrieve&db=Protein&list_uids=54792123&dopt=GenPept&RID=T280CBCZ01N&log$=prottop&blast_rank=33) | complement component (3d/Epstein Barr virus) receptor 2 isoform 1 precursor [Homo sapiens] | [19.3](http://blast.ncbi.nlm.nih.gov/Blast.cgi#54792123%2354792123) | 19.3 | 100% | 268 |
| [NP_001129025.1](http://www.ncbi.nlm.nih.gov/entrez/query.fcgi?cmd=Retrieve&db=Protein&list_uids=207113186&dopt=GenPept&RID=T280CBCZ01N&log$=prottop&blast_rank=34) | MAP kinase-interacting serine/threonine kinase 1 isoform 3 [Homo sapiens] | [19.3](http://blast.ncbi.nlm.nih.gov/Blast.cgi#207113186%23207113186) | 19.3 | 80% | 268 |
| [NP_076942.1](http://www.ncbi.nlm.nih.gov/entrez/query.fcgi?cmd=Retrieve&db=Protein&list_uids=13128990&dopt=GenPept&RID=T280CBCZ01N&log$=prottop&blast_rank=35) | aurora A-binding protein [Homo sapiens] | [19.3](http://blast.ncbi.nlm.nih.gov/Blast.cgi#13128990%2313128990) | 19.3 | 50% | 268 |
| [NP_003675.2](http://www.ncbi.nlm.nih.gov/entrez/query.fcgi?cmd=Retrieve&db=Protein&list_uids=21361101&dopt=GenPept&RID=T280CBCZ01N&log$=prottop&blast_rank=36) | MAP kinase-interacting serine/threonine kinase 1 isoform 1 [Homo sapiens] | [19.3](http://blast.ncbi.nlm.nih.gov/Blast.cgi#21361101%2321361101) | 19.3 | 80% | 268 |
| [NP_002331.3](http://www.ncbi.nlm.nih.gov/entrez/query.fcgi?cmd=Retrieve&db=Protein&list_uids=47933395&dopt=GenPept&RID=T280CBCZ01N&log$=prottop&blast_rank=37) | lanosterol synthase isoform 1 [Homo sapiens] >ref|NP_001001438.1| lanosterol synthase isoform 1 [Homo sapiens] | [19.3](http://blast.ncbi.nlm.nih.gov/Blast.cgi#47933395%2347933395) | 19.3 | 60% | 268 |
| [NP_055761.2](http://www.ncbi.nlm.nih.gov/entrez/query.fcgi?cmd=Retrieve&db=Protein&list_uids=11875211&dopt=GenPept&RID=T280CBCZ01N&log$=prottop&blast_rank=38) | spastin isoform 1 [Homo sapiens] | [19.3](http://blast.ncbi.nlm.nih.gov/Blast.cgi#11875211%2311875211) | 31.4 | 70% | 268 |
| [NP_001159887.1](http://www.ncbi.nlm.nih.gov/entrez/query.fcgi?cmd=Retrieve&db=Protein&list_uids=261878539&dopt=GenPept&RID=T280CBCZ01N&log$=prottop&blast_rank=39) | enoyl-Coenzyme A, hydratase/3-hydroxyacyl Coenzyme A dehydrogenase isoform 2 [Homo sapiens] | [18.9](http://blast.ncbi.nlm.nih.gov/Blast.cgi#261878539%23261878539) | 18.9 | 60% | 359 |
| [NP_001129476.1](http://www.ncbi.nlm.nih.gov/entrez/query.fcgi?cmd=Retrieve&db=Protein&list_uids=209862793&dopt=GenPept&RID=T280CBCZ01N&log$=prottop&blast_rank=40) | microtubule associated monoxygenase, calponin and LIM domain containing 3 isoform 2 [Homo sapiens] | [18.9](http://blast.ncbi.nlm.nih.gov/Blast.cgi#209862793%23209862793) | 18.9 | 90% | 359 |
| [NP_001957.2](http://www.ncbi.nlm.nih.gov/entrez/query.fcgi?cmd=Retrieve&db=Protein&list_uids=68989263&dopt=GenPept&RID=T280CBCZ01N&log$=prottop&blast_rank=41) | enoyl-Coenzyme A, hydratase/3-hydroxyacyl Coenzyme A dehydrogenase isoform 1 [Homo sapiens] | [18.9](http://blast.ncbi.nlm.nih.gov/Blast.cgi#68989263%2368989263) | 18.9 | 60% | 359 |
| [NP_005155.1](http://www.ncbi.nlm.nih.gov/entrez/query.fcgi?cmd=Retrieve&db=Protein&list_uids=9945308&dopt=GenPept&RID=T280CBCZ01N&log$=prottop&blast_rank=42) | ATP-binding cassette, sub-family D, member 2 [Homo sapiens] | [18.9](http://blast.ncbi.nlm.nih.gov/Blast.cgi#9945308%239945308) | 18.9 | 60% | 359 |
| [NP_114143.1](http://www.ncbi.nlm.nih.gov/entrez/query.fcgi?cmd=Retrieve&db=Protein&list_uids=13994322&dopt=GenPept&RID=T280CBCZ01N&log$=prottop&blast_rank=43) | TBC1 domain family, member 10A [Homo sapiens] | [18.9](http://blast.ncbi.nlm.nih.gov/Blast.cgi#13994322%2313994322) | 18.9 | 70% | 359 |
| [NP_001073972.2](http://www.ncbi.nlm.nih.gov/entrez/query.fcgi?cmd=Retrieve&db=Protein&list_uids=209969682&dopt=GenPept&RID=T280CBCZ01N&log$=prottop&blast_rank=44) | coiled-coil domain-containing-like [Homo sapiens] | [18.5](http://blast.ncbi.nlm.nih.gov/Blast.cgi#209969682%23209969682) | 18.5 | 60% | 482 |
| [NP_001123553.1](http://www.ncbi.nlm.nih.gov/entrez/query.fcgi?cmd=Retrieve&db=Protein&list_uids=194272176&dopt=GenPept&RID=T280CBCZ01N&log$=prottop&blast_rank=45) | phospholipase D1 isoform b [Homo sapiens] | [18.5](http://blast.ncbi.nlm.nih.gov/Blast.cgi#194272176%23194272176) | 18.5 | 80% | 482 |
| [NP_055681.1](http://www.ncbi.nlm.nih.gov/entrez/query.fcgi?cmd=Retrieve&db=Protein&list_uids=124378039&dopt=GenPept&RID=T280CBCZ01N&log$=prottop&blast_rank=46) | SEC16 homolog A [Homo sapiens] | [18.5](http://blast.ncbi.nlm.nih.gov/Blast.cgi#124378039%23124378039) | 33.5 | 70% | 482 |
| [NP_055885.3](http://www.ncbi.nlm.nih.gov/entrez/query.fcgi?cmd=Retrieve&db=Protein&list_uids=116008442&dopt=GenPept&RID=T280CBCZ01N&log$=prottop&blast_rank=47) | zinc finger CCCH-type containing 13 [Homo sapiens] | [18.5](http://blast.ncbi.nlm.nih.gov/Blast.cgi#116008442%23116008442) | 29.3 | 70% | 482 |
| [NP_663696.1](http://www.ncbi.nlm.nih.gov/entrez/query.fcgi?cmd=Retrieve&db=Protein&list_uids=22035682&dopt=GenPept&RID=T280CBCZ01N&log$=prottop&blast_rank=48) | DBF4 homolog B isoform 1 [Homo sapiens] | [18.5](http://blast.ncbi.nlm.nih.gov/Blast.cgi#22035682%2322035682) | 18.5 | 70% | 482 |
| [NP_000121.2](http://www.ncbi.nlm.nih.gov/entrez/query.fcgi?cmd=Retrieve&db=Protein&list_uids=105990535&dopt=GenPept&RID=T280CBCZ01N&log$=prottop&blast_rank=49) | coagulation factor V precursor [Homo sapiens] | [18.5](http://blast.ncbi.nlm.nih.gov/Blast.cgi#105990535%23105990535) | 18.5 | 50% | 482 |
| [NP_001013864.1](http://www.ncbi.nlm.nih.gov/entrez/query.fcgi?cmd=Retrieve&db=Protein&list_uids=62241036&dopt=GenPept&RID=T280CBCZ01N&log$=prottop&blast_rank=50) | hypothetical protein LOC541565 [Homo sapiens] | [18.5](http://blast.ncbi.nlm.nih.gov/Blast.cgi#62241036%2362241036) | 18.5 | 60% | 482 |
| [NP_008841.2](http://www.ncbi.nlm.nih.gov/entrez/query.fcgi?cmd=Retrieve&db=Protein&list_uids=33620769&dopt=GenPept&RID=T280CBCZ01N&log$=prottop&blast_rank=51) | retinoblastoma-binding protein 6 isoform 1 [Homo sapiens] | [18.5](http://blast.ncbi.nlm.nih.gov/Blast.cgi#33620769%2333620769) | 18.5 | 60% | 482 |
| [NP_061173.1](http://www.ncbi.nlm.nih.gov/entrez/query.fcgi?cmd=Retrieve&db=Protein&list_uids=33620716&dopt=GenPept&RID=T280CBCZ01N&log$=prottop&blast_rank=52) | retinoblastoma-binding protein 6 isoform 2 [Homo sapiens] | [18.5](http://blast.ncbi.nlm.nih.gov/Blast.cgi#33620716%2333620716) | 18.5 | 60% | 482 |
| [NP_004074.2](http://www.ncbi.nlm.nih.gov/entrez/query.fcgi?cmd=Retrieve&db=Protein&list_uids=21361118&dopt=GenPept&RID=T280CBCZ01N&log$=prottop&blast_rank=53) | DNA-damage-inducible transcript 3 [Homo sapiens] | [18.5](http://blast.ncbi.nlm.nih.gov/Blast.cgi#21361118%2321361118) | 18.5 | 50% | 482 |
| [NP_003150.1](http://www.ncbi.nlm.nih.gov/entrez/query.fcgi?cmd=Retrieve&db=Protein&list_uids=4507281&dopt=GenPept&RID=T280CBCZ01N&log$=prottop&blast_rank=54) | cyclin-dependent kinase-like 5 [Homo sapiens] >ref|NP_001032420.1| cyclin-dependent kinase-like 5 [Homo sapiens] | [18.5](http://blast.ncbi.nlm.nih.gov/Blast.cgi#4507281%234507281) | 29.3 | 80% | 482 |
| [NP_758872.1](http://www.ncbi.nlm.nih.gov/entrez/query.fcgi?cmd=Retrieve&db=Protein&list_uids=27437001&dopt=GenPept&RID=T280CBCZ01N&log$=prottop&blast_rank=55) | potassium voltage-gated channel, subfamily H, member 1 isoform 1 [Homo sapiens] | [18.5](http://blast.ncbi.nlm.nih.gov/Blast.cgi#27437001%2327437001) | 18.5 | 90% | 482 |
| [NP_002229.1](http://www.ncbi.nlm.nih.gov/entrez/query.fcgi?cmd=Retrieve&db=Protein&list_uids=4504831&dopt=GenPept&RID=T280CBCZ01N&log$=prottop&blast_rank=56) | potassium voltage-gated channel, subfamily H, member 1 isoform 2 [Homo sapiens] | [18.5](http://blast.ncbi.nlm.nih.gov/Blast.cgi#4504831%234504831) | 18.5 | 90% | 482 |
| [NP_002653.1](http://www.ncbi.nlm.nih.gov/entrez/query.fcgi?cmd=Retrieve&db=Protein&list_uids=4505873&dopt=GenPept&RID=T280CBCZ01N&log$=prottop&blast_rank=57) | phospholipase D1 isoform a [Homo sapiens] | [18.5](http://blast.ncbi.nlm.nih.gov/Blast.cgi#4505873%234505873) | 18.5 | 80% | 482 |
| [NP_001157980.1](http://www.ncbi.nlm.nih.gov/entrez/query.fcgi?cmd=Retrieve&db=Protein&list_uids=257743025&dopt=GenPept&RID=T280CBCZ01N&log$=prottop&blast_rank=58) | nebulin isoform 2 [Homo sapiens] | [18.0](http://blast.ncbi.nlm.nih.gov/Blast.cgi#257743025%23257743025) | 305 | 80% | 647 |
| [NP_001157979.1](http://www.ncbi.nlm.nih.gov/entrez/query.fcgi?cmd=Retrieve&db=Protein&list_uids=257743023&dopt=GenPept&RID=T280CBCZ01N&log$=prottop&blast_rank=59) | nebulin isoform 1 [Homo sapiens] | [18.0](http://blast.ncbi.nlm.nih.gov/Blast.cgi#257743023%23257743023) | 305 | 80% | 647 |
| [XP_002344782.1](http://www.ncbi.nlm.nih.gov/entrez/query.fcgi?cmd=Retrieve&db=Protein&list_uids=239756039&dopt=GenPept&RID=T280CBCZ01N&log$=prottop&blast_rank=60) | PREDICTED: hypothetical protein LOC643677 [Homo sapiens] | [18.0](http://blast.ncbi.nlm.nih.gov/Blast.cgi#239756039%23239756039) | 18.0 | 60% | 647 |
| [XP_001725026.2](http://www.ncbi.nlm.nih.gov/entrez/query.fcgi?cmd=Retrieve&db=Protein&list_uids=239755877&dopt=GenPept&RID=T280CBCZ01N&log$=prottop&blast_rank=61) | PREDICTED: hypothetical protein [Homo sapiens] | [18.0](http://blast.ncbi.nlm.nih.gov/Blast.cgi#239755877%23239755877) | 18.0 | 100% | 647 |
| [XP_002347245.1](http://www.ncbi.nlm.nih.gov/entrez/query.fcgi?cmd=Retrieve&db=Protein&list_uids=239749968&dopt=GenPept&RID=T280CBCZ01N&log$=prottop&blast_rank=62) | PREDICTED: hypothetical protein XP_002347245 [Homo sapiens] >ref|XP_002344583.1| PREDICTED: hypothetical protein [Homo sapiens] | [18.0](http://blast.ncbi.nlm.nih.gov/Blast.cgi#239749968%23239749968) | 18.0 | 60% | 647 |
| [XP_002343110.1](http://www.ncbi.nlm.nih.gov/entrez/query.fcgi?cmd=Retrieve&db=Protein&list_uids=239744278&dopt=GenPept&RID=T280CBCZ01N&log$=prottop&blast_rank=63) | PREDICTED: hypothetical protein XP_002343110 [Homo sapiens] | [18.0](http://blast.ncbi.nlm.nih.gov/Blast.cgi#239744278%23239744278) | 18.0 | 60% | 647 |
| [XP_002342255.1](http://www.ncbi.nlm.nih.gov/entrez/query.fcgi?cmd=Retrieve&db=Protein&list_uids=239741576&dopt=GenPept&RID=T280CBCZ01N&log$=prottop&blast_rank=64) | PREDICTED: hypothetical protein XP_002342255 [Homo sapiens] >ref|XP_002348232.1| PREDICTED: hypothetical protein XP_002348232 [Homo sapiens] >ref|XP_002345559.1| PREDICTED: hypothetical protein [Homo sapiens] | [18.0](http://blast.ncbi.nlm.nih.gov/Blast.cgi#239741576%23239741576) | 18.0 | 100% | 647 |
| [XP_002344126.1](http://www.ncbi.nlm.nih.gov/entrez/query.fcgi?cmd=Retrieve&db=Protein&list_uids=239744870&dopt=GenPept&RID=T280CBCZ01N&log$=prottop&blast_rank=65) | PREDICTED: hypothetical protein LOC643677 [Homo sapiens] | [18.0](http://blast.ncbi.nlm.nih.gov/Blast.cgi#239744870%23239744870) | 18.0 | 60% | 647 |
| [NP_001139669.1](http://www.ncbi.nlm.nih.gov/entrez/query.fcgi?cmd=Retrieve&db=Protein&list_uids=226246554&dopt=GenPept&RID=T280CBCZ01N&log$=prottop&blast_rank=66) | hypothetical protein LOC643677 [Homo sapiens] | [18.0](http://blast.ncbi.nlm.nih.gov/Blast.cgi#226246554%23226246554) | 18.0 | 60% | 647 |
| [NP_001138863.1](http://www.ncbi.nlm.nih.gov/entrez/query.fcgi?cmd=Retrieve&db=Protein&list_uids=223972627&dopt=GenPept&RID=T280CBCZ01N&log$=prottop&blast_rank=67) | 3-oxoacyl-ACP synthase, mitochondrial isoform 2 precursor [Homo sapiens] | [18.0](http://blast.ncbi.nlm.nih.gov/Blast.cgi#223972627%23223972627) | 18.0 | 100% | 647 |
| [NP_001138590.1](http://www.ncbi.nlm.nih.gov/entrez/query.fcgi?cmd=Retrieve&db=Protein&list_uids=223278403&dopt=GenPept&RID=T280CBCZ01N&log$=prottop&blast_rank=68) | glutamate receptor, ionotropic, delta 2 (Grid2) interacting protein [Homo sapiens] | [18.0](http://blast.ncbi.nlm.nih.gov/Blast.cgi#223278403%23223278403) | 18.0 | 60% | 647 |
| [XP_001718215.1](http://www.ncbi.nlm.nih.gov/entrez/query.fcgi?cmd=Retrieve&db=Protein&list_uids=169214758&dopt=GenPept&RID=T280CBCZ01N&log$=prottop&blast_rank=69) | PREDICTED: hypothetical protein [Homo sapiens] | [18.0](http://blast.ncbi.nlm.nih.gov/Blast.cgi#169214758%23169214758) | 18.0 | 90% | 647 |
| [XP_001718505.1](http://www.ncbi.nlm.nih.gov/entrez/query.fcgi?cmd=Retrieve&db=Protein&list_uids=169214665&dopt=GenPept&RID=T280CBCZ01N&log$=prottop&blast_rank=70) | PREDICTED: hypothetical protein [Homo sapiens] >ref|XP_001717907.1| PREDICTED: hypothetical protein [Homo sapiens] | [18.0](http://blast.ncbi.nlm.nih.gov/Blast.cgi#169214665%23169214665) | 18.0 | 90% | 647 |
| [NP_001073860.1](http://www.ncbi.nlm.nih.gov/entrez/query.fcgi?cmd=Retrieve&db=Protein&list_uids=122939208&dopt=GenPept&RID=T280CBCZ01N&log$=prottop&blast_rank=71) | nuclear antigen Sp100 isoform 1 [Homo sapiens] | [18.0](http://blast.ncbi.nlm.nih.gov/Blast.cgi#122939208%23122939208) | 18.0 | 80% | 647 |
| [XP_002347449.1](http://www.ncbi.nlm.nih.gov/entrez/query.fcgi?cmd=Retrieve&db=Protein&list_uids=239750569&dopt=GenPept&RID=T280CBCZ01N&log$=prottop&blast_rank=72) | PREDICTED: hypothetical protein LOC643677 [Homo sapiens] | [18.0](http://blast.ncbi.nlm.nih.gov/Blast.cgi#239750569%23239750569) | 18.0 | 60% | 647 |
| [NP_004534.2](http://www.ncbi.nlm.nih.gov/entrez/query.fcgi?cmd=Retrieve&db=Protein&list_uids=115527120&dopt=GenPept&RID=T280CBCZ01N&log$=prottop&blast_rank=73) | nebulin isoform 3 [Homo sapiens] | [18.0](http://blast.ncbi.nlm.nih.gov/Blast.cgi#115527120%23115527120) | 197 | 80% | 647 |
| [NP_001013049.1](http://www.ncbi.nlm.nih.gov/entrez/query.fcgi?cmd=Retrieve&db=Protein&list_uids=61743975&dopt=GenPept&RID=T280CBCZ01N&log$=prottop&blast_rank=74) | SORCS receptor 1 isoform b [Homo sapiens] | [18.0](http://blast.ncbi.nlm.nih.gov/Blast.cgi#61743975%2361743975) | 18.0 | 60% | 647 |
| [NP_443150.3](http://www.ncbi.nlm.nih.gov/entrez/query.fcgi?cmd=Retrieve&db=Protein&list_uids=61743973&dopt=GenPept&RID=T280CBCZ01N&log$=prottop&blast_rank=75) | SORCS receptor 1 isoform a [Homo sapiens] | [18.0](http://blast.ncbi.nlm.nih.gov/Blast.cgi#61743973%2361743973) | 18.0 | 60% | 647 |
| [NP_060367.1](http://www.ncbi.nlm.nih.gov/entrez/query.fcgi?cmd=Retrieve&db=Protein&list_uids=8923559&dopt=GenPept&RID=T280CBCZ01N&log$=prottop&blast_rank=76) | 3-oxoacyl-ACP synthase, mitochondrial isoform 1 precursor [Homo sapiens] | [18.0](http://blast.ncbi.nlm.nih.gov/Blast.cgi#8923559%238923559) | 18.0 | 100% | 647 |
| [XP_002345272.1](http://www.ncbi.nlm.nih.gov/entrez/query.fcgi?cmd=Retrieve&db=Protein&list_uids=239757412&dopt=GenPept&RID=T280CBCZ01N&log$=prottop&blast_rank=77) | PREDICTED: hypothetical protein XP_002345272 [Homo sapiens] | [17.6](http://blast.ncbi.nlm.nih.gov/Blast.cgi#239757412%23239757412) | 17.6 | 80% | 868 |
| [XP_002348035.1](http://www.ncbi.nlm.nih.gov/entrez/query.fcgi?cmd=Retrieve&db=Protein&list_uids=239751877&dopt=GenPept&RID=T280CBCZ01N&log$=prottop&blast_rank=78) | PREDICTED: hypothetical protein XP_002348035 [Homo sapiens] | [17.6](http://blast.ncbi.nlm.nih.gov/Blast.cgi#239751877%23239751877) | 30.5 | 80% | 868 |
| [NP_444282.3](http://www.ncbi.nlm.nih.gov/entrez/query.fcgi?cmd=Retrieve&db=Protein&list_uids=221316609&dopt=GenPept&RID=T280CBCZ01N&log$=prottop&blast_rank=79) | sperm-associated cation channel 1 [Homo sapiens] | [17.6](http://blast.ncbi.nlm.nih.gov/Blast.cgi#221316609%23221316609) | 33.9 | 80% | 868 |
| [XP_001715486.1](http://www.ncbi.nlm.nih.gov/entrez/query.fcgi?cmd=Retrieve&db=Protein&list_uids=169207417&dopt=GenPept&RID=T280CBCZ01N&log$=prottop&blast_rank=80) | PREDICTED: similar to hCG2042489 [Homo sapiens] | [17.6](http://blast.ncbi.nlm.nih.gov/Blast.cgi#169207417%23169207417) | 17.6 | 60% | 868 |
| [NP_001092008.1](http://www.ncbi.nlm.nih.gov/entrez/query.fcgi?cmd=Retrieve&db=Protein&list_uids=154448882&dopt=GenPept&RID=T280CBCZ01N&log$=prottop&blast_rank=81) | proline rich 4 (lacrimal) isoform 1 [Homo sapiens] | [17.6](http://blast.ncbi.nlm.nih.gov/Blast.cgi#154448882%23154448882) | 17.6 | 70% | 868 |
| [NP_005900.2](http://www.ncbi.nlm.nih.gov/entrez/query.fcgi?cmd=Retrieve&db=Protein&list_uids=153945728&dopt=GenPept&RID=T280CBCZ01N&log$=prottop&blast_rank=82) | microtubule-associated protein 1B [Homo sapiens] | [17.6](http://blast.ncbi.nlm.nih.gov/Blast.cgi#153945728%23153945728) | 33.9 | 50% | 868 |
| [NP_009175.2](http://www.ncbi.nlm.nih.gov/entrez/query.fcgi?cmd=Retrieve&db=Protein&list_uids=154448886&dopt=GenPept&RID=T280CBCZ01N&log$=prottop&blast_rank=83) | proline rich 4 (lacrimal) isoform 2 [Homo sapiens] | [17.6](http://blast.ncbi.nlm.nih.gov/Blast.cgi#154448886%23154448886) | 17.6 | 70% | 868 |
| [NP_919277.2](http://www.ncbi.nlm.nih.gov/entrez/query.fcgi?cmd=Retrieve&db=Protein&list_uids=51230412&dopt=GenPept&RID=T280CBCZ01N&log$=prottop&blast_rank=84) | Ral GTPase activating protein, alpha subunit 1 (catalytic) isoform 2 [Homo sapiens] | [17.6](http://blast.ncbi.nlm.nih.gov/Blast.cgi#51230412%2351230412) | 33.1 | 100% | 868 |
| [NP_671722.1](http://www.ncbi.nlm.nih.gov/entrez/query.fcgi?cmd=Retrieve&db=Protein&list_uids=22218339&dopt=GenPept&RID=T280CBCZ01N&log$=prottop&blast_rank=85) | hypothetical protein LOC90362 [Homo sapiens] | [17.6](http://blast.ncbi.nlm.nih.gov/Blast.cgi#22218339%2322218339) | 17.6 | 70% | 868 |
| [NP_055805.1](http://www.ncbi.nlm.nih.gov/entrez/query.fcgi?cmd=Retrieve&db=Protein&list_uids=51226124&dopt=GenPept&RID=T280CBCZ01N&log$=prottop&blast_rank=86) | Ral GTPase activating protein, alpha subunit 1 (catalytic) isoform 1 [Homo sapiens] | [17.6](http://blast.ncbi.nlm.nih.gov/Blast.cgi#51226124%2351226124) | 33.1 | 100% | 868 |
| [NP_001164218.1](http://www.ncbi.nlm.nih.gov/entrez/query.fcgi?cmd=Retrieve&db=Protein&list_uids=282847476&dopt=GenPept&RID=T280CBCZ01N&log$=prottop&blast_rank=87) | protein (peptidyl-prolyl cis/trans isomerase) NIMA-interacting, 4 (parvulin) isoform 2 [Homo sapiens] | [17.2](http://blast.ncbi.nlm.nih.gov/Blast.cgi#282847476%23282847476) | 17.2 | 60% | 1165 |
| [NP_001157108.1](http://www.ncbi.nlm.nih.gov/entrez/query.fcgi?cmd=Retrieve&db=Protein&list_uids=254692934&dopt=GenPept&RID=T280CBCZ01N&log$=prottop&blast_rank=88) | general transcription factor IIi isoform 5 [Homo sapiens] | [17.2](http://blast.ncbi.nlm.nih.gov/Blast.cgi#254692934%23254692934) | 17.2 | 80% | 1165 |
| [XP_002346011.1](http://www.ncbi.nlm.nih.gov/entrez/query.fcgi?cmd=Retrieve&db=Protein&list_uids=239754512&dopt=GenPept&RID=T280CBCZ01N&log$=prottop&blast_rank=89) | PREDICTED: hypothetical protein [Homo sapiens] | [17.2](http://blast.ncbi.nlm.nih.gov/Blast.cgi#239754512%23239754512) | 17.2 | 60% | 1165 |
| [XP_002346897.1](http://www.ncbi.nlm.nih.gov/entrez/query.fcgi?cmd=Retrieve&db=Protein&list_uids=239749073&dopt=GenPept&RID=T280CBCZ01N&log$=prottop&blast_rank=90) | PREDICTED: hypothetical protein [Homo sapiens] | [17.2](http://blast.ncbi.nlm.nih.gov/Blast.cgi#239749073%23239749073) | 17.2 | 60% | 1165 |
| [XP_002346883.1](http://www.ncbi.nlm.nih.gov/entrez/query.fcgi?cmd=Retrieve&db=Protein&list_uids=239749021&dopt=GenPept&RID=T280CBCZ01N&log$=prottop&blast_rank=91) | PREDICTED: hypothetical protein LOC401287 [Homo sapiens] >ref|XP_002345999.1| PREDICTED: hypothetical protein LOC401287 [Homo sapiens] | [17.2](http://blast.ncbi.nlm.nih.gov/Blast.cgi#239749021%23239749021) | 17.2 | 50% | 1165 |
| [XP_001714126.2](http://www.ncbi.nlm.nih.gov/entrez/query.fcgi?cmd=Retrieve&db=Protein&list_uids=239748788&dopt=GenPept&RID=T280CBCZ01N&log$=prottop&blast_rank=92) | PREDICTED: similar to major histocompatibility complex, class II, DR beta 5 isoform 2 [Homo sapiens] | [17.2](http://blast.ncbi.nlm.nih.gov/Blast.cgi#239748788%23239748788) | 17.2 | 50% | 1165 |
| [XP_001715497.2](http://www.ncbi.nlm.nih.gov/entrez/query.fcgi?cmd=Retrieve&db=Protein&list_uids=239743415&dopt=GenPept&RID=T280CBCZ01N&log$=prottop&blast_rank=93) | PREDICTED: hypothetical protein [Homo sapiens] >ref|XP_002346114.1| PREDICTED: hypothetical protein [Homo sapiens] | [17.2](http://blast.ncbi.nlm.nih.gov/Blast.cgi#239743415%23239743415) | 17.2 | 70% | 1165 |
| [XP_002342724.1](http://www.ncbi.nlm.nih.gov/entrez/query.fcgi?cmd=Retrieve&db=Protein&list_uids=239743040&dopt=GenPept&RID=T280CBCZ01N&log$=prottop&blast_rank=94) | PREDICTED: hypothetical protein LOC401287 [Homo sapiens] | [17.2](http://blast.ncbi.nlm.nih.gov/Blast.cgi#239743040%23239743040) | 17.2 | 50% | 1165 |
| [XP_002344087.1](http://www.ncbi.nlm.nih.gov/entrez/query.fcgi?cmd=Retrieve&db=Protein&list_uids=239740918&dopt=GenPept&RID=T280CBCZ01N&log$=prottop&blast_rank=95) | PREDICTED: similar to major histocompatibility complex, class II, DQ beta 1 isoform 1 [Homo sapiens] | [17.2](http://blast.ncbi.nlm.nih.gov/Blast.cgi#239740918%23239740918) | 17.2 | 50% | 1165 |
| [XP_002344003.1](http://www.ncbi.nlm.nih.gov/entrez/query.fcgi?cmd=Retrieve&db=Protein&list_uids=239740749&dopt=GenPept&RID=T280CBCZ01N&log$=prottop&blast_rank=96) | PREDICTED: similar to major histocompatibility complex, class II, DR beta 5 [Homo sapiens] >ref|XP_002346290.1| PREDICTED: similar to major histocompatibility complex, class II, DR beta 5 [Homo sapiens] | [17.2](http://blast.ncbi.nlm.nih.gov/Blast.cgi#239740749%23239740749) | 17.2 | 50% | 1165 |
| [XP_002344479.1](http://www.ncbi.nlm.nih.gov/entrez/query.fcgi?cmd=Retrieve&db=Protein&list_uids=239509122&dopt=GenPept&RID=T280CBCZ01N&log$=prottop&blast_rank=97) | PREDICTED: hypothetical protein [Homo sapiens] >ref|XP_001714494.2| PREDICTED: hypothetical protein [Homo sapiens] | [17.2](http://blast.ncbi.nlm.nih.gov/Blast.cgi#239509122%23239509122) | 17.2 | 70% | 1165 |
| [XP_002344337.1](http://www.ncbi.nlm.nih.gov/entrez/query.fcgi?cmd=Retrieve&db=Protein&list_uids=239508751&dopt=GenPept&RID=T280CBCZ01N&log$=prottop&blast_rank=98) | PREDICTED: hypothetical protein [Homo sapiens] >ref|XP_002342754.1| PREDICTED: hypothetical protein XP_002342754 [Homo sapiens] | [17.2](http://blast.ncbi.nlm.nih.gov/Blast.cgi#239508751%23239508751) | 17.2 | 60% | 1165 |
| [XP_002343953.1](http://www.ncbi.nlm.nih.gov/entrez/query.fcgi?cmd=Retrieve&db=Protein&list_uids=239513955&dopt=GenPept&RID=T280CBCZ01N&log$=prottop&blast_rank=99) | PREDICTED: similar to major histocompatibility complex, class II, DR beta 5 [Homo sapiens] >ref|XP_002344058.1| PREDICTED: similar to major histocompatibility complex, class II, DR beta 5 [Homo sapiens] | [17.2](http://blast.ncbi.nlm.nih.gov/Blast.cgi#239513955%23239513955) | 17.2 | 50% | 1165 |
| [XP_002344088.1](http://www.ncbi.nlm.nih.gov/entrez/query.fcgi?cmd=Retrieve&db=Protein&list_uids=239740920&dopt=GenPept&RID=T280CBCZ01N&log$=prottop&blast_rank=100) | PREDICTED: similar to major histocompatibility complex, class II, DQ beta 1 isoform 2 [Homo sapiens] | [17.2](http://blast.ncbi.nlm.nih.gov/Blast.cgi#239740920%23239740920) | 17.2 | 50% | 1165 |

| **Accession** | **Proteins with a match to WTRRPYDELIV peptide** | **[Max score](http://blast.ncbi.nlm.nih.gov/Blast.cgi?CMD=Get&ALIGNMENTS=100&ALIGNMENT_VIEW=Pairwise&CDD_SEARCH_STATE=1&DATABASE_SORT=0&DESCRIPTIONS=100&ENTREZ_QUERY=txid9606 %5BORGN%5D&FIRST_QUERY_NUM=0&FORMAT_OBJECT=Alignment&FORMAT_PAGE_TARGET=&FORMAT_TYPE=HTML&GET_SEQUENCE=yes&I_THRESH=&MASK_CHAR=2&MASK_COLOR=1&NEW_DESIGN=on&NEW_VIEW=yes&NUM_OVERVIEW=100&OLD_BLAST=false&PAGE=Proteins&QUERY_INDEX=0&QUERY_NUMBER=0&RESULTS_PAGE_TARGET=&RID=T284T3FC012&SHOW_LINKOUT=yes&SHOW_OVERVIEW=yes&STEP_NUMBER=&WORD_SIZE=2&DISPLAY_SORT=1&HSP_SORT=1" \l "sort_mark)** | **[Total score](http://blast.ncbi.nlm.nih.gov/Blast.cgi?CMD=Get&ALIGNMENTS=100&ALIGNMENT_VIEW=Pairwise&CDD_SEARCH_STATE=1&DATABASE_SORT=0&DESCRIPTIONS=100&ENTREZ_QUERY=txid9606 %5BORGN%5D&FIRST_QUERY_NUM=0&FORMAT_OBJECT=Alignment&FORMAT_PAGE_TARGET=&FORMAT_TYPE=HTML&GET_SEQUENCE=yes&I_THRESH=&MASK_CHAR=2&MASK_COLOR=1&NEW_DESIGN=on&NEW_VIEW=yes&NUM_OVERVIEW=100&OLD_BLAST=false&PAGE=Proteins&QUERY_INDEX=0&QUERY_NUMBER=0&RESULTS_PAGE_TARGET=&RID=T284T3FC012&SHOW_LINKOUT=yes&SHOW_OVERVIEW=yes&STEP_NUMBER=&WORD_SIZE=2&DISPLAY_SORT=2&HSP_SORT=1" \l "sort_mark)** | **[Query coverage](http://blast.ncbi.nlm.nih.gov/Blast.cgi?CMD=Get&ALIGNMENTS=100&ALIGNMENT_VIEW=Pairwise&CDD_SEARCH_STATE=1&DATABASE_SORT=0&DESCRIPTIONS=100&ENTREZ_QUERY=txid9606 %5BORGN%5D&FIRST_QUERY_NUM=0&FORMAT_OBJECT=Alignment&FORMAT_PAGE_TARGET=&FORMAT_TYPE=HTML&GET_SEQUENCE=yes&I_THRESH=&MASK_CHAR=2&MASK_COLOR=1&NEW_DESIGN=on&NEW_VIEW=yes&NUM_OVERVIEW=100&OLD_BLAST=false&PAGE=Proteins&QUERY_INDEX=0&QUERY_NUMBER=0&RESULTS_PAGE_TARGET=&RID=T284T3FC012&SHOW_LINKOUT=yes&SHOW_OVERVIEW=yes&STEP_NUMBER=&WORD_SIZE=2&DISPLAY_SORT=4&HSP_SORT=0" \l "sort_mark)** | **[E value](http://blast.ncbi.nlm.nih.gov/Blast.cgi?CMD=Get&ALIGNMENTS=100&ALIGNMENT_VIEW=Pairwise&CDD_SEARCH_STATE=1&DATABASE_SORT=0&DESCRIPTIONS=100&ENTREZ_QUERY=txid9606 %5BORGN%5D&FIRST_QUERY_NUM=0&FORMAT_OBJECT=Alignment&FORMAT_PAGE_TARGET=&FORMAT_TYPE=HTML&GET_SEQUENCE=yes&I_THRESH=&MASK_CHAR=2&MASK_COLOR=1&NEW_DESIGN=on&NEW_VIEW=yes&NUM_OVERVIEW=100&OLD_BLAST=false&PAGE=Proteins&QUERY_INDEX=0&QUERY_NUMBER=0&RESULTS_PAGE_TARGET=&RID=T284T3FC012&SHOW_LINKOUT=yes&SHOW_OVERVIEW=yes&STEP_NUMBER=&WORD_SIZE=2&DISPLAY_SORT=0&HSP_SORT=0" \l "sort_mark)** |
| --- | --- | --- | --- | --- | --- |
| [NP_000350.1](http://www.ncbi.nlm.nih.gov/entrez/query.fcgi?cmd=Retrieve&db=Protein&list_uids=4507475&dopt=GenPept&RID=T284T3FC012&log$=prottop&blast_rank=1) | transglutaminase 1 [Homo sapiens] | [23.5](http://blast.ncbi.nlm.nih.gov/Blast.cgi#4507475%234507475) | 39.9 | 90% | 14 |
| [NP_079002.3](http://www.ncbi.nlm.nih.gov/entrez/query.fcgi?cmd=Retrieve&db=Protein&list_uids=40255052&dopt=GenPept&RID=T284T3FC012&log$=prottop&blast_rank=2) | IQ motif containing with AAA domain 1 [Homo sapiens] | [22.7](http://blast.ncbi.nlm.nih.gov/Blast.cgi#40255052%2340255052) | 22.7 | 72% | 25 |
| [NP_001123559.1](http://www.ncbi.nlm.nih.gov/entrez/query.fcgi?cmd=Retrieve&db=Protein&list_uids=194272204&dopt=GenPept&RID=T284T3FC012&log$=prottop&blast_rank=3) | actin binding LIM protein family, member 2 isoform 5 [Homo sapiens] | [21.8](http://blast.ncbi.nlm.nih.gov/Blast.cgi#194272204%23194272204) | 21.8 | 63% | 46 |
| [NP_001123555.1](http://www.ncbi.nlm.nih.gov/entrez/query.fcgi?cmd=Retrieve&db=Protein&list_uids=194272196&dopt=GenPept&RID=T284T3FC012&log$=prottop&blast_rank=4) | actin binding LIM protein family, member 2 isoform 1 [Homo sapiens] | [21.8](http://blast.ncbi.nlm.nih.gov/Blast.cgi#194272196%23194272196) | 21.8 | 63% | 46 |
| [NP_115808.3](http://www.ncbi.nlm.nih.gov/entrez/query.fcgi?cmd=Retrieve&db=Protein&list_uids=153792514&dopt=GenPept&RID=T284T3FC012&log$=prottop&blast_rank=5) | actin binding LIM protein family, member 2 isoform 6 [Homo sapiens] | [21.8](http://blast.ncbi.nlm.nih.gov/Blast.cgi#153792514%23153792514) | 21.8 | 63% | 46 |
| [NP_001123558.1](http://www.ncbi.nlm.nih.gov/entrez/query.fcgi?cmd=Retrieve&db=Protein&list_uids=194272202&dopt=GenPept&RID=T284T3FC012&log$=prottop&blast_rank=6) | actin binding LIM protein family, member 2 isoform 4 [Homo sapiens] | [21.8](http://blast.ncbi.nlm.nih.gov/Blast.cgi#194272202%23194272202) | 21.8 | 63% | 46 |
| [NP_001123557.1](http://www.ncbi.nlm.nih.gov/entrez/query.fcgi?cmd=Retrieve&db=Protein&list_uids=194272200&dopt=GenPept&RID=T284T3FC012&log$=prottop&blast_rank=7) | actin binding LIM protein family, member 2 isoform 3 [Homo sapiens] | [21.8](http://blast.ncbi.nlm.nih.gov/Blast.cgi#194272200%23194272200) | 21.8 | 63% | 46 |
| [NP_001123556.1](http://www.ncbi.nlm.nih.gov/entrez/query.fcgi?cmd=Retrieve&db=Protein&list_uids=194272198&dopt=GenPept&RID=T284T3FC012&log$=prottop&blast_rank=8) | actin binding LIM protein family, member 2 isoform 2 [Homo sapiens] | [21.8](http://blast.ncbi.nlm.nih.gov/Blast.cgi#194272198%23194272198) | 21.8 | 63% | 46 |
| [NP_056981.2](http://www.ncbi.nlm.nih.gov/entrez/query.fcgi?cmd=Retrieve&db=Protein&list_uids=24850133&dopt=GenPept&RID=T284T3FC012&log$=prottop&blast_rank=9) | protein inhibitor of activated STAT, 4 [Homo sapiens] | [21.8](http://blast.ncbi.nlm.nih.gov/Blast.cgi#24850133%2324850133) | 21.8 | 63% | 46 |
| [NP_596869.3](http://www.ncbi.nlm.nih.gov/entrez/query.fcgi?cmd=Retrieve&db=Protein&list_uids=110349719&dopt=GenPept&RID=T284T3FC012&log$=prottop&blast_rank=10) | titin isoform N2-A [Homo sapiens] | [21.4](http://blast.ncbi.nlm.nih.gov/Blast.cgi#110349719%23110349719) | 225 | 100% | 61 |
| [NP_689649.1](http://www.ncbi.nlm.nih.gov/entrez/query.fcgi?cmd=Retrieve&db=Protein&list_uids=22748923&dopt=GenPept&RID=T284T3FC012&log$=prottop&blast_rank=11) | GLI pathogenesis-related 1 like 2 [Homo sapiens] | [21.4](http://blast.ncbi.nlm.nih.gov/Blast.cgi#22748923%2322748923) | 21.4 | 54% | 61 |
| [NP_116045.2](http://www.ncbi.nlm.nih.gov/entrez/query.fcgi?cmd=Retrieve&db=Protein&list_uids=29029601&dopt=GenPept&RID=T284T3FC012&log$=prottop&blast_rank=12) | DEAH (Asp-Glu-Ala-His) box polypeptide 37 [Homo sapiens] | [21.4](http://blast.ncbi.nlm.nih.gov/Blast.cgi#29029601%2329029601) | 34.8 | 81% | 61 |
| [XP_002347399.1](http://www.ncbi.nlm.nih.gov/entrez/query.fcgi?cmd=Retrieve&db=Protein&list_uids=239750456&dopt=GenPept&RID=T284T3FC012&log$=prottop&blast_rank=13) | PREDICTED: similar to KIAA0655 protein [Homo sapiens] | [21.0](http://blast.ncbi.nlm.nih.gov/Blast.cgi#239750456%23239750456) | 21.0 | 54% | 83 |
| [NP_009019.1](http://www.ncbi.nlm.nih.gov/entrez/query.fcgi?cmd=Retrieve&db=Protein&list_uids=6031162&dopt=GenPept&RID=T284T3FC012&log$=prottop&blast_rank=14) | calbindin 2 isoform 22k [Homo sapiens] | [21.0](http://blast.ncbi.nlm.nih.gov/Blast.cgi#6031162%236031162) | 21.0 | 45% | 83 |
| [NP_001731.2](http://www.ncbi.nlm.nih.gov/entrez/query.fcgi?cmd=Retrieve&db=Protein&list_uids=153946409&dopt=GenPept&RID=T284T3FC012&log$=prottop&blast_rank=15) | calbindin 2 isoform 1 [Homo sapiens] | [21.0](http://blast.ncbi.nlm.nih.gov/Blast.cgi#153946409%23153946409) | 21.0 | 45% | 83 |
| [NP_000143.2](http://www.ncbi.nlm.nih.gov/entrez/query.fcgi?cmd=Retrieve&db=Protein&list_uids=119393891&dopt=GenPept&RID=T284T3FC012&log$=prottop&blast_rank=16) | acid alpha-glucosidase preproprotein [Homo sapiens] >ref|NP_001073271.1| acid alpha-glucosidase preproprotein [Homo sapiens] >ref|NP_001073272.1| acid alpha-glucosidase preproprotein [Homo sapiens] | [21.0](http://blast.ncbi.nlm.nih.gov/Blast.cgi#119393891%23119393891) | 21.0 | 45% | 83 |
| [NP_002958.2](http://www.ncbi.nlm.nih.gov/entrez/query.fcgi?cmd=Retrieve&db=Protein&list_uids=21264343&dopt=GenPept&RID=T284T3FC012&log$=prottop&blast_rank=17) | scaffold attachment factor B [Homo sapiens] | [21.0](http://blast.ncbi.nlm.nih.gov/Blast.cgi#21264343%2321264343) | 21.0 | 45% | 83 |
| [NP_055464.1](http://www.ncbi.nlm.nih.gov/entrez/query.fcgi?cmd=Retrieve&db=Protein&list_uids=7661936&dopt=GenPept&RID=T284T3FC012&log$=prottop&blast_rank=18) | scaffold attachment factor B2 [Homo sapiens] | [21.0](http://blast.ncbi.nlm.nih.gov/Blast.cgi#7661936%237661936) | 21.0 | 45% | 83 |
| [NP_003950.1](http://www.ncbi.nlm.nih.gov/entrez/query.fcgi?cmd=Retrieve&db=Protein&list_uids=48762942&dopt=GenPept&RID=T284T3FC012&log$=prottop&blast_rank=19) | huntingtin interacting protein-1-related [Homo sapiens] | [21.0](http://blast.ncbi.nlm.nih.gov/Blast.cgi#48762942%2348762942) | 21.0 | 54% | 83 |
| [NP_001165113.1](http://www.ncbi.nlm.nih.gov/entrez/query.fcgi?cmd=Retrieve&db=Protein&list_uids=284172514&dopt=GenPept&RID=T284T3FC012&log$=prottop&blast_rank=20) | myosin IIIB isoform 3 [Homo sapiens] | [20.6](http://blast.ncbi.nlm.nih.gov/Blast.cgi#284172514%23284172514) | 20.6 | 45% | 111 |
| [NP_620482.3](http://www.ncbi.nlm.nih.gov/entrez/query.fcgi?cmd=Retrieve&db=Protein&list_uids=284172512&dopt=GenPept&RID=T284T3FC012&log$=prottop&blast_rank=21) | myosin IIIB isoform 2 [Homo sapiens] | [20.6](http://blast.ncbi.nlm.nih.gov/Blast.cgi#284172512%23284172512) | 20.6 | 45% | 111 |
| [NP_001077084.2](http://www.ncbi.nlm.nih.gov/entrez/query.fcgi?cmd=Retrieve&db=Protein&list_uids=284172510&dopt=GenPept&RID=T284T3FC012&log$=prottop&blast_rank=22) | myosin IIIB isoform 1 [Homo sapiens] | [20.6](http://blast.ncbi.nlm.nih.gov/Blast.cgi#284172510%23284172510) | 20.6 | 45% | 111 |
| [NP_001157738.1](http://www.ncbi.nlm.nih.gov/entrez/query.fcgi?cmd=Retrieve&db=Protein&list_uids=256017124&dopt=GenPept&RID=T284T3FC012&log$=prottop&blast_rank=23) | latent transforming growth factor beta binding protein 3 isoform 3 [Homo sapiens] | [20.6](http://blast.ncbi.nlm.nih.gov/Blast.cgi#256017124%23256017124) | 20.6 | 63% | 111 |
| [NP_001123616.1](http://www.ncbi.nlm.nih.gov/entrez/query.fcgi?cmd=Retrieve&db=Protein&list_uids=194328809&dopt=GenPept&RID=T284T3FC012&log$=prottop&blast_rank=24) | latent transforming growth factor beta binding protein 3 isoform 1 precursor [Homo sapiens] | [20.6](http://blast.ncbi.nlm.nih.gov/Blast.cgi#194328809%23194328809) | 20.6 | 63% | 111 |
| [NP_066548.2](http://www.ncbi.nlm.nih.gov/entrez/query.fcgi?cmd=Retrieve&db=Protein&list_uids=18497288&dopt=GenPept&RID=T284T3FC012&log$=prottop&blast_rank=25) | latent transforming growth factor beta binding protein 3 isoform 2 precursor [Homo sapiens] | [20.6](http://blast.ncbi.nlm.nih.gov/Blast.cgi#18497288%2318497288) | 20.6 | 63% | 111 |
| [NP_579899.1](http://www.ncbi.nlm.nih.gov/entrez/query.fcgi?cmd=Retrieve&db=Protein&list_uids=19718759&dopt=GenPept&RID=T284T3FC012&log$=prottop&blast_rank=26) | myoferlin isoform b [Homo sapiens] | [20.6](http://blast.ncbi.nlm.nih.gov/Blast.cgi#19718759%2319718759) | 20.6 | 45% | 111 |
| [NP_004761.2](http://www.ncbi.nlm.nih.gov/entrez/query.fcgi?cmd=Retrieve&db=Protein&list_uids=27436974&dopt=GenPept&RID=T284T3FC012&log$=prottop&blast_rank=27) | potassium voltage-gated channel, Shab-related subfamily, member 2 [Homo sapiens] | [20.6](http://blast.ncbi.nlm.nih.gov/Blast.cgi#27436974%2327436974) | 20.6 | 63% | 111 |
| [NP_055617.1](http://www.ncbi.nlm.nih.gov/entrez/query.fcgi?cmd=Retrieve&db=Protein&list_uids=29789060&dopt=GenPept&RID=T284T3FC012&log$=prottop&blast_rank=28) | hypothetical protein LOC9847 [Homo sapiens] | [20.6](http://blast.ncbi.nlm.nih.gov/Blast.cgi#29789060%2329789060) | 36.9 | 54% | 111 |
| [NP_038479.1](http://www.ncbi.nlm.nih.gov/entrez/query.fcgi?cmd=Retrieve&db=Protein&list_uids=7305053&dopt=GenPept&RID=T284T3FC012&log$=prottop&blast_rank=29) | myoferlin isoform a [Homo sapiens] | [20.6](http://blast.ncbi.nlm.nih.gov/Blast.cgi#7305053%237305053) | 20.6 | 45% | 111 |
| [NP_004966.1](http://www.ncbi.nlm.nih.gov/entrez/query.fcgi?cmd=Retrieve&db=Protein&list_uids=4826784&dopt=GenPept&RID=T284T3FC012&log$=prottop&blast_rank=30) | potassium voltage-gated channel, Shab-related subfamily, member 1 [Homo sapiens] | [20.6](http://blast.ncbi.nlm.nih.gov/Blast.cgi#4826784%234826784) | 20.6 | 63% | 111 |
| [NP_695001.1](http://www.ncbi.nlm.nih.gov/entrez/query.fcgi?cmd=Retrieve&db=Protein&list_uids=23943928&dopt=GenPept&RID=T284T3FC012&log$=prottop&blast_rank=31) | hypothetical protein LOC140680 [Homo sapiens] | [20.6](http://blast.ncbi.nlm.nih.gov/Blast.cgi#23943928%2323943928) | 20.6 | 63% | 111 |
| [NP_055394.2](http://www.ncbi.nlm.nih.gov/entrez/query.fcgi?cmd=Retrieve&db=Protein&list_uids=156415986&dopt=GenPept&RID=T284T3FC012&log$=prottop&blast_rank=32) | solute carrier family 39 (zinc transporter), member 2 [Homo sapiens] | [20.2](http://blast.ncbi.nlm.nih.gov/Blast.cgi#156415986%23156415986) | 20.2 | 63% | 149 |
| [NP_064545.1](http://www.ncbi.nlm.nih.gov/entrez/query.fcgi?cmd=Retrieve&db=Protein&list_uids=57863281&dopt=GenPept&RID=T284T3FC012&log$=prottop&blast_rank=33) | Meis1, myeloid ecotropic viral integration site 1 homolog 3 isoform 1 [Homo sapiens] | [20.2](http://blast.ncbi.nlm.nih.gov/Blast.cgi#57863281%2357863281) | 20.2 | 81% | 149 |
| [NP_001009813.1](http://www.ncbi.nlm.nih.gov/entrez/query.fcgi?cmd=Retrieve&db=Protein&list_uids=57863279&dopt=GenPept&RID=T284T3FC012&log$=prottop&blast_rank=34) | Meis1, myeloid ecotropic viral integration site 1 homolog 3 isoform 2 [Homo sapiens] | [20.2](http://blast.ncbi.nlm.nih.gov/Blast.cgi#57863279%2357863279) | 20.2 | 81% | 149 |
| [NP_055660.1](http://www.ncbi.nlm.nih.gov/entrez/query.fcgi?cmd=Retrieve&db=Protein&list_uids=7662034&dopt=GenPept&RID=T284T3FC012&log$=prottop&blast_rank=35) | Sac domain-containing inositol phosphatase 3 [Homo sapiens] | [20.2](http://blast.ncbi.nlm.nih.gov/Blast.cgi#7662034%237662034) | 36.5 | 63% | 149 |
| [NP_006250.1](http://www.ncbi.nlm.nih.gov/entrez/query.fcgi?cmd=Retrieve&db=Protein&list_uids=5453978&dopt=GenPept&RID=T284T3FC012&log$=prottop&blast_rank=36) | protein kinase, cGMP-dependent, type II [Homo sapiens] | [20.2](http://blast.ncbi.nlm.nih.gov/Blast.cgi#5453978%235453978) | 20.2 | 81% | 149 |
| [XP_002342076.1](http://www.ncbi.nlm.nih.gov/entrez/query.fcgi?cmd=Retrieve&db=Protein&list_uids=239741085&dopt=GenPept&RID=T284T3FC012&log$=prottop&blast_rank=37) | PREDICTED: beta-lactamase-like protein ENSP00000383859-like [Homo sapiens] >ref|XP_001130038.3| PREDICTED: beta-lactamase-like protein ENSP00000383859-like [Homo sapiens] >ref|XP_001715076.2| PREDICTED: beta-lactamase-like protein ENSP00000383859-like [Homo sapiens] | [19.7](http://blast.ncbi.nlm.nih.gov/Blast.cgi#239741085%23239741085) | 19.7 | 54% | 199 |
| [NP_001139311.1](http://www.ncbi.nlm.nih.gov/entrez/query.fcgi?cmd=Retrieve&db=Protein&list_uids=224994164&dopt=GenPept&RID=T284T3FC012&log$=prottop&blast_rank=38) | mitochondrial fission regulator 1 isoform 3 [Homo sapiens] | [19.7](http://blast.ncbi.nlm.nih.gov/Blast.cgi#224994164%23224994164) | 19.7 | 54% | 199 |
| [NP_001096080.1](http://www.ncbi.nlm.nih.gov/entrez/query.fcgi?cmd=Retrieve&db=Protein&list_uids=156616299&dopt=GenPept&RID=T284T3FC012&log$=prottop&blast_rank=39) | tubulin, gamma complex associated protein 5 isoform b [Homo sapiens] | [19.7](http://blast.ncbi.nlm.nih.gov/Blast.cgi#156616299%23156616299) | 35.2 | 100% | 199 |
| [NP_075463.2](http://www.ncbi.nlm.nih.gov/entrez/query.fcgi?cmd=Retrieve&db=Protein&list_uids=117606355&dopt=GenPept&RID=T284T3FC012&log$=prottop&blast_rank=40) | furry homolog [Homo sapiens] | [19.7](http://blast.ncbi.nlm.nih.gov/Blast.cgi#117606355%23117606355) | 19.7 | 63% | 199 |
| [NP_001059.2](http://www.ncbi.nlm.nih.gov/entrez/query.fcgi?cmd=Retrieve&db=Protein&list_uids=19913408&dopt=GenPept&RID=T284T3FC012&log$=prottop&blast_rank=41) | DNA topoisomerase II, beta isozyme [Homo sapiens] | [19.7](http://blast.ncbi.nlm.nih.gov/Blast.cgi#19913408%2319913408) | 19.7 | 54% | 199 |
| [NP_116220.1](http://www.ncbi.nlm.nih.gov/entrez/query.fcgi?cmd=Retrieve&db=Protein&list_uids=14249538&dopt=GenPept&RID=T284T3FC012&log$=prottop&blast_rank=42) | ORAI calcium release-activated calcium modulator 2 [Homo sapiens] >ref|NP_001119812.1| ORAI calcium release-activated calcium modulator 2 [Homo sapiens] | [19.7](http://blast.ncbi.nlm.nih.gov/Blast.cgi#14249538%2314249538) | 33.9 | 90% | 199 |
| [NP_443099.1](http://www.ncbi.nlm.nih.gov/entrez/query.fcgi?cmd=Retrieve&db=Protein&list_uids=24119274&dopt=GenPept&RID=T284T3FC012&log$=prottop&blast_rank=43) | voltage gated channel like 1 [Homo sapiens] | [19.7](http://blast.ncbi.nlm.nih.gov/Blast.cgi#24119274%2324119274) | 19.7 | 54% | 199 |
| [NP_055452.3](http://www.ncbi.nlm.nih.gov/entrez/query.fcgi?cmd=Retrieve&db=Protein&list_uids=224994160&dopt=GenPept&RID=T284T3FC012&log$=prottop&blast_rank=44) | mitochondrial fission regulator 1 isoform 1 [Homo sapiens] | [19.7](http://blast.ncbi.nlm.nih.gov/Blast.cgi#224994160%23224994160) | 19.7 | 54% | 199 |
| [NP_005473.1](http://www.ncbi.nlm.nih.gov/entrez/query.fcgi?cmd=Retrieve&db=Protein&list_uids=23199983&dopt=GenPept&RID=T284T3FC012&log$=prottop&blast_rank=45) | phosphatidylinositol glycan anchor biosynthesis, class K precursor [Homo sapiens] | [19.7](http://blast.ncbi.nlm.nih.gov/Blast.cgi#23199983%2323199983) | 19.7 | 81% | 199 |
| [NP_443135.3](http://www.ncbi.nlm.nih.gov/entrez/query.fcgi?cmd=Retrieve&db=Protein&list_uids=156616297&dopt=GenPept&RID=T284T3FC012&log$=prottop&blast_rank=46) | tubulin, gamma complex associated protein 5 isoform a [Homo sapiens] | [19.7](http://blast.ncbi.nlm.nih.gov/Blast.cgi#156616297%23156616297) | 35.2 | 100% | 199 |
| [NP_001165780.1](http://www.ncbi.nlm.nih.gov/entrez/query.fcgi?cmd=Retrieve&db=Protein&list_uids=288856248&dopt=GenPept&RID=T284T3FC012&log$=prottop&blast_rank=47) | nexilin (F actin binding protein) isoform 2 [Homo sapiens] | [19.3](http://blast.ncbi.nlm.nih.gov/Blast.cgi#288856248%23288856248) | 19.3 | 63% | 267 |
| [NP_001155155.1](http://www.ncbi.nlm.nih.gov/entrez/query.fcgi?cmd=Retrieve&db=Protein&list_uids=239735575&dopt=GenPept&RID=T284T3FC012&log$=prottop&blast_rank=48) | otoancorin isoform 3 [Homo sapiens] | [19.3](http://blast.ncbi.nlm.nih.gov/Blast.cgi#239735575%23239735575) | 19.3 | 72% | 267 |
| [NP_653174.3](http://www.ncbi.nlm.nih.gov/entrez/query.fcgi?cmd=Retrieve&db=Protein&list_uids=148839339&dopt=GenPept&RID=T284T3FC012&log$=prottop&blast_rank=49) | nexilin (F actin binding protein) isoform 1 [Homo sapiens] | [19.3](http://blast.ncbi.nlm.nih.gov/Blast.cgi#148839339%23148839339) | 45.6 | 90% | 267 |
| [NP_001019764.1](http://www.ncbi.nlm.nih.gov/entrez/query.fcgi?cmd=Retrieve&db=Protein&list_uids=67782303&dopt=GenPept&RID=T284T3FC012&log$=prottop&blast_rank=50) | zinc finger, MYND domain containing 17 [Homo sapiens] | [19.3](http://blast.ncbi.nlm.nih.gov/Blast.cgi#67782303%2367782303) | 30.5 | 63% | 267 |
| [NP_060109.2](http://www.ncbi.nlm.nih.gov/entrez/query.fcgi?cmd=Retrieve&db=Protein&list_uids=47059046&dopt=GenPept&RID=T284T3FC012&log$=prottop&blast_rank=51) | dachsous 2 isoform 1 [Homo sapiens] | [19.3](http://blast.ncbi.nlm.nih.gov/Blast.cgi#47059046%2347059046) | 47.3 | 63% | 267 |
| [NP_653273.3](http://www.ncbi.nlm.nih.gov/entrez/query.fcgi?cmd=Retrieve&db=Protein&list_uids=77404409&dopt=GenPept&RID=T284T3FC012&log$=prottop&blast_rank=52) | otoancorin isoform 1 [Homo sapiens] | [19.3](http://blast.ncbi.nlm.nih.gov/Blast.cgi#77404409%2377404409) | 19.3 | 72% | 267 |
| [NP_001028229.1](http://www.ncbi.nlm.nih.gov/entrez/query.fcgi?cmd=Retrieve&db=Protein&list_uids=74272284&dopt=GenPept&RID=T284T3FC012&log$=prottop&blast_rank=53) | membrane associated guanylate kinase, WW and PDZ domain containing 1 isoform c [Homo sapiens] | [19.3](http://blast.ncbi.nlm.nih.gov/Blast.cgi#74272284%2374272284) | 35.6 | 72% | 267 |
| [NP_001032203.1](http://www.ncbi.nlm.nih.gov/entrez/query.fcgi?cmd=Retrieve&db=Protein&list_uids=82546832&dopt=GenPept&RID=T284T3FC012&log$=prottop&blast_rank=54) | SEC8 protein isoform b [Homo sapiens] | [19.3](http://blast.ncbi.nlm.nih.gov/Blast.cgi#82546832%2382546832) | 19.3 | 45% | 267 |
| [NP_078886.2](http://www.ncbi.nlm.nih.gov/entrez/query.fcgi?cmd=Retrieve&db=Protein&list_uids=21314714&dopt=GenPept&RID=T284T3FC012&log$=prottop&blast_rank=55) | Hspb associated protein 1 [Homo sapiens] | [19.3](http://blast.ncbi.nlm.nih.gov/Blast.cgi#21314714%2321314714) | 19.3 | 63% | 267 |
| [NP_114141.2](http://www.ncbi.nlm.nih.gov/entrez/query.fcgi?cmd=Retrieve&db=Protein&list_uids=118572606&dopt=GenPept&RID=T284T3FC012&log$=prottop&blast_rank=56) | hemicentin 1 precursor [Homo sapiens] | [19.3](http://blast.ncbi.nlm.nih.gov/Blast.cgi#118572606%23118572606) | 19.3 | 54% | 267 |
| [NP_997053.1](http://www.ncbi.nlm.nih.gov/entrez/query.fcgi?cmd=Retrieve&db=Protein&list_uids=46371998&dopt=GenPept&RID=T284T3FC012&log$=prottop&blast_rank=57) | SYF2 homolog, RNA splicing factor isoform 2 [Homo sapiens] | [19.3](http://blast.ncbi.nlm.nih.gov/Blast.cgi#46371998%2346371998) | 19.3 | 54% | 267 |
| [NP_001692.1](http://www.ncbi.nlm.nih.gov/entrez/query.fcgi?cmd=Retrieve&db=Protein&list_uids=4502351&dopt=GenPept&RID=T284T3FC012&log$=prottop&blast_rank=58) | bile acid Coenzyme A: amino acid N-acyltransferase [Homo sapiens] >ref|NP_001121082.1| bile acid Coenzyme A: amino acid N-acyltransferase [Homo sapiens] | [19.3](http://blast.ncbi.nlm.nih.gov/Blast.cgi#4502351%234502351) | 19.3 | 54% | 267 |
| [NP_116262.2](http://www.ncbi.nlm.nih.gov/entrez/query.fcgi?cmd=Retrieve&db=Protein&list_uids=24497612&dopt=GenPept&RID=T284T3FC012&log$=prottop&blast_rank=59) | ubiquitin associated and SH3 domain containing, B [Homo sapiens] | [19.3](http://blast.ncbi.nlm.nih.gov/Blast.cgi#24497612%2324497612) | 19.3 | 54% | 267 |
| [NP_733764.1](http://www.ncbi.nlm.nih.gov/entrez/query.fcgi?cmd=Retrieve&db=Protein&list_uids=24638452&dopt=GenPept&RID=T284T3FC012&log$=prottop&blast_rank=60) | otoancorin isoform 2 [Homo sapiens] | [19.3](http://blast.ncbi.nlm.nih.gov/Blast.cgi#24638452%2324638452) | 19.3 | 72% | 267 |
| [NP_056299.1](http://www.ncbi.nlm.nih.gov/entrez/query.fcgi?cmd=Retrieve&db=Protein&list_uids=7661636&dopt=GenPept&RID=T284T3FC012&log$=prottop&blast_rank=61) | SYF2 homolog, RNA splicing factor isoform 1 [Homo sapiens] | [19.3](http://blast.ncbi.nlm.nih.gov/Blast.cgi#7661636%237661636) | 19.3 | 54% | 267 |
| [NP_149093.1](http://www.ncbi.nlm.nih.gov/entrez/query.fcgi?cmd=Retrieve&db=Protein&list_uids=14916437&dopt=GenPept&RID=T284T3FC012&log$=prottop&blast_rank=62) | prostein [Homo sapiens] | [19.3](http://blast.ncbi.nlm.nih.gov/Blast.cgi#14916437%2314916437) | 19.3 | 54% | 267 |
| [NP_751948.1](http://www.ncbi.nlm.nih.gov/entrez/query.fcgi?cmd=Retrieve&db=Protein&list_uids=27436986&dopt=GenPept&RID=T284T3FC012&log$=prottop&blast_rank=63) | potassium voltage-gated channel, Shal-related subfamily, member 3 isoform 2 [Homo sapiens] | [19.3](http://blast.ncbi.nlm.nih.gov/Blast.cgi#27436986%2327436986) | 19.3 | 45% | 267 |
| [NP_004971.2](http://www.ncbi.nlm.nih.gov/entrez/query.fcgi?cmd=Retrieve&db=Protein&list_uids=27436984&dopt=GenPept&RID=T284T3FC012&log$=prottop&blast_rank=64) | potassium voltage-gated channel, Shal-related subfamily, member 3 isoform 1 [Homo sapiens] | [19.3](http://blast.ncbi.nlm.nih.gov/Blast.cgi#27436984%2327436984) | 19.3 | 45% | 267 |
| [NP_001530.1](http://www.ncbi.nlm.nih.gov/entrez/query.fcgi?cmd=Retrieve&db=Protein&list_uids=4504511&dopt=GenPept&RID=T284T3FC012&log$=prottop&blast_rank=65) | DnaJ (Hsp40) homolog, subfamily A, member 1 [Homo sapiens] | [19.3](http://blast.ncbi.nlm.nih.gov/Blast.cgi#4504511%234504511) | 32.7 | 81% | 267 |
| [NP_068579.3](http://www.ncbi.nlm.nih.gov/entrez/query.fcgi?cmd=Retrieve&db=Protein&list_uids=82546830&dopt=GenPept&RID=T284T3FC012&log$=prottop&blast_rank=66) | SEC8 protein isoform a [Homo sapiens] | [19.3](http://blast.ncbi.nlm.nih.gov/Blast.cgi#82546830%2382546830) | 19.3 | 45% | 267 |
| [NP_612455.1](http://www.ncbi.nlm.nih.gov/entrez/query.fcgi?cmd=Retrieve&db=Protein&list_uids=19923977&dopt=GenPept&RID=T284T3FC012&log$=prottop&blast_rank=67) | hypothetical protein LOC115416 [Homo sapiens] | [19.3](http://blast.ncbi.nlm.nih.gov/Blast.cgi#19923977%2319923977) | 19.3 | 72% | 267 |
| [NP_001139381.1](http://www.ncbi.nlm.nih.gov/entrez/query.fcgi?cmd=Retrieve&db=Protein&list_uids=225543463&dopt=GenPept&RID=T284T3FC012&log$=prottop&blast_rank=68) | tetratricopeptide repeat, ankyrin repeat and coiled-coil containing 1 isoform 2 [Homo sapiens] | [18.9](http://blast.ncbi.nlm.nih.gov/Blast.cgi#225543463%23225543463) | 18.9 | 72% | 359 |
| [NP_203752.2](http://www.ncbi.nlm.nih.gov/entrez/query.fcgi?cmd=Retrieve&db=Protein&list_uids=225543461&dopt=GenPept&RID=T284T3FC012&log$=prottop&blast_rank=69) | tetratricopeptide repeat, ankyrin repeat and coiled-coil containing 1 isoform 1 [Homo sapiens] | [18.9](http://blast.ncbi.nlm.nih.gov/Blast.cgi#225543461%23225543461) | 18.9 | 72% | 359 |
| [NP_573400.3](http://www.ncbi.nlm.nih.gov/entrez/query.fcgi?cmd=Retrieve&db=Protein&list_uids=148539858&dopt=GenPept&RID=T284T3FC012&log$=prottop&blast_rank=70) | protein tyrosine phosphatase, receptor type, T isoform 1 precursor [Homo sapiens] | [18.9](http://blast.ncbi.nlm.nih.gov/Blast.cgi#148539858%23148539858) | 18.9 | 54% | 359 |
| [NP_001078880.1](http://www.ncbi.nlm.nih.gov/entrez/query.fcgi?cmd=Retrieve&db=Protein&list_uids=146134341&dopt=GenPept&RID=T284T3FC012&log$=prottop&blast_rank=71) | hypothetical protein LOC133686 isoform 1 [Homo sapiens] | [18.9](http://blast.ncbi.nlm.nih.gov/Blast.cgi#146134341%23146134341) | 18.9 | 54% | 359 |
| [NP_065769.3](http://www.ncbi.nlm.nih.gov/entrez/query.fcgi?cmd=Retrieve&db=Protein&list_uids=120587027&dopt=GenPept&RID=T284T3FC012&log$=prottop&blast_rank=72) | ubiquitin specific peptidase 31 [Homo sapiens] | [18.9](http://blast.ncbi.nlm.nih.gov/Blast.cgi#120587027%23120587027) | 18.9 | 54% | 359 |
| [NP_003913.3](http://www.ncbi.nlm.nih.gov/entrez/query.fcgi?cmd=Retrieve&db=Protein&list_uids=126131099&dopt=GenPept&RID=T284T3FC012&log$=prottop&blast_rank=73) | hect domain and RCC1-like domain 1 [Homo sapiens] | [18.9](http://blast.ncbi.nlm.nih.gov/Blast.cgi#126131099%23126131099) | 18.9 | 54% | 359 |
| [NP_000637.2](http://www.ncbi.nlm.nih.gov/entrez/query.fcgi?cmd=Retrieve&db=Protein&list_uids=116734853&dopt=GenPept&RID=T284T3FC012&log$=prottop&blast_rank=74) | amylo-1,6-glucosidase, 4-alpha-glucanotransferase isoform 3 [Homo sapiens] | [18.9](http://blast.ncbi.nlm.nih.gov/Blast.cgi#116734853%23116734853) | 30.1 | 72% | 359 |
| [NP_620687.2](http://www.ncbi.nlm.nih.gov/entrez/query.fcgi?cmd=Retrieve&db=Protein&list_uids=110735441&dopt=GenPept&RID=T284T3FC012&log$=prottop&blast_rank=75) | ADAM metallopeptidase with thrombospondin type 1 motif, 16 preproprotein [Homo sapiens] | [18.9](http://blast.ncbi.nlm.nih.gov/Blast.cgi#110735441%23110735441) | 18.9 | 72% | 359 |
| [NP_001155855.1](http://www.ncbi.nlm.nih.gov/entrez/query.fcgi?cmd=Retrieve&db=Protein&list_uids=253735775&dopt=GenPept&RID=T284T3FC012&log$=prottop&blast_rank=76) | Rho/Rac guanine nucleotide exchange factor 2 isoform 1 [Homo sapiens] | [18.9](http://blast.ncbi.nlm.nih.gov/Blast.cgi#253735775%23253735775) | 18.9 | 63% | 359 |
| [NP_008981.4](http://www.ncbi.nlm.nih.gov/entrez/query.fcgi?cmd=Retrieve&db=Protein&list_uids=148539860&dopt=GenPept&RID=T284T3FC012&log$=prottop&blast_rank=77) | protein tyrosine phosphatase, receptor type, T isoform 2 precursor [Homo sapiens] | [18.9](http://blast.ncbi.nlm.nih.gov/Blast.cgi#148539860%23148539860) | 18.9 | 54% | 359 |
| [NP_057605.3](http://www.ncbi.nlm.nih.gov/entrez/query.fcgi?cmd=Retrieve&db=Protein&list_uids=189409125&dopt=GenPept&RID=T284T3FC012&log$=prottop&blast_rank=78) | transcription factor Dp family, member 3 [Homo sapiens] | [18.9](http://blast.ncbi.nlm.nih.gov/Blast.cgi#189409125%23189409125) | 18.9 | 81% | 359 |
| [NP_001073904.1](http://www.ncbi.nlm.nih.gov/entrez/query.fcgi?cmd=Retrieve&db=Protein&list_uids=122937271&dopt=GenPept&RID=T284T3FC012&log$=prottop&blast_rank=79) | WAS protein homolog associated with actin, golgi membranes and microtubules [Homo sapiens] | [18.9](http://blast.ncbi.nlm.nih.gov/Blast.cgi#122937271%23122937271) | 18.9 | 100% | 359 |
| [NP_001155856.1](http://www.ncbi.nlm.nih.gov/entrez/query.fcgi?cmd=Retrieve&db=Protein&list_uids=253735778&dopt=GenPept&RID=T284T3FC012&log$=prottop&blast_rank=80) | Rho/Rac guanine nucleotide exchange factor 2 isoform 2 [Homo sapiens] | [18.9](http://blast.ncbi.nlm.nih.gov/Blast.cgi#253735778%23253735778) | 18.9 | 63% | 359 |
| [NP_000258.1](http://www.ncbi.nlm.nih.gov/entrez/query.fcgi?cmd=Retrieve&db=Protein&list_uids=4557793&dopt=GenPept&RID=T284T3FC012&log$=prottop&blast_rank=81) | neurofibromin isoform 2 [Homo sapiens] | [18.9](http://blast.ncbi.nlm.nih.gov/Blast.cgi#4557793%234557793) | 36.1 | 90% | 359 |
| [NP_991404.1](http://www.ncbi.nlm.nih.gov/entrez/query.fcgi?cmd=Retrieve&db=Protein&list_uids=45593136&dopt=GenPept&RID=T284T3FC012&log$=prottop&blast_rank=82) | lipolysis stimulated lipoprotein receptor isoform 3 [Homo sapiens] | [18.9](http://blast.ncbi.nlm.nih.gov/Blast.cgi#45593136%2345593136) | 18.9 | 54% | 359 |
| [NP_055181.3](http://www.ncbi.nlm.nih.gov/entrez/query.fcgi?cmd=Retrieve&db=Protein&list_uids=45593130&dopt=GenPept&RID=T284T3FC012&log$=prottop&blast_rank=83) | guanine nucleotide binding protein-like 3 isoform 1 [Homo sapiens] | [18.9](http://blast.ncbi.nlm.nih.gov/Blast.cgi#45593130%2345593130) | 18.9 | 100% | 359 |
| [NP_057009.3](http://www.ncbi.nlm.nih.gov/entrez/query.fcgi?cmd=Retrieve&db=Protein&list_uids=45505163&dopt=GenPept&RID=T284T3FC012&log$=prottop&blast_rank=84) | lipolysis stimulated lipoprotein receptor isoform 1 [Homo sapiens] | [18.9](http://blast.ncbi.nlm.nih.gov/Blast.cgi#45505163%2345505163) | 18.9 | 54% | 359 |
| [NP_991403.1](http://www.ncbi.nlm.nih.gov/entrez/query.fcgi?cmd=Retrieve&db=Protein&list_uids=45545424&dopt=GenPept&RID=T284T3FC012&log$=prottop&blast_rank=85) | lipolysis stimulated lipoprotein receptor isoform 2 [Homo sapiens] | [18.9](http://blast.ncbi.nlm.nih.gov/Blast.cgi#45545424%2345545424) | 18.9 | 54% | 359 |
| [NP_061908.1](http://www.ncbi.nlm.nih.gov/entrez/query.fcgi?cmd=Retrieve&db=Protein&list_uids=14589929&dopt=GenPept&RID=T284T3FC012&log$=prottop&blast_rank=86) | protocadherin 18 precursor [Homo sapiens] | [18.9](http://blast.ncbi.nlm.nih.gov/Blast.cgi#14589929%2314589929) | 18.9 | 54% | 359 |
| [NP_001091980.1](http://www.ncbi.nlm.nih.gov/entrez/query.fcgi?cmd=Retrieve&db=Protein&list_uids=148612833&dopt=GenPept&RID=T284T3FC012&log$=prottop&blast_rank=87) | pellino 3 isoform 2 [Homo sapiens] | [18.9](http://blast.ncbi.nlm.nih.gov/Blast.cgi#148612833%23148612833) | 18.9 | 54% | 359 |
| [NP_940894.1](http://www.ncbi.nlm.nih.gov/entrez/query.fcgi?cmd=Retrieve&db=Protein&list_uids=38348296&dopt=GenPept&RID=T284T3FC012&log$=prottop&blast_rank=88) | C-type lectin domain family 4, member G [Homo sapiens] | [18.9](http://blast.ncbi.nlm.nih.gov/Blast.cgi#38348296%2338348296) | 18.9 | 45% | 359 |
| [NP_057125.2](http://www.ncbi.nlm.nih.gov/entrez/query.fcgi?cmd=Retrieve&db=Protein&list_uids=31455614&dopt=GenPept&RID=T284T3FC012&log$=prottop&blast_rank=89) | Der1-like domain family, member 2 [Homo sapiens] | [18.9](http://blast.ncbi.nlm.nih.gov/Blast.cgi#31455614%2331455614) | 18.9 | 54% | 359 |
| [NP_000636.2](http://www.ncbi.nlm.nih.gov/entrez/query.fcgi?cmd=Retrieve&db=Protein&list_uids=116734849&dopt=GenPept&RID=T284T3FC012&log$=prottop&blast_rank=90) | amylo-1,6-glucosidase, 4-alpha-glucanotransferase isoform 2 [Homo sapiens] | [18.9](http://blast.ncbi.nlm.nih.gov/Blast.cgi#116734849%23116734849) | 30.1 | 72% | 359 |
| [NP_004714.2](http://www.ncbi.nlm.nih.gov/entrez/query.fcgi?cmd=Retrieve&db=Protein&list_uids=15011974&dopt=GenPept&RID=T284T3FC012&log$=prottop&blast_rank=91) | Rho/Rac guanine nucleotide exchange factor 2 isooform 3 [Homo sapiens] | [18.9](http://blast.ncbi.nlm.nih.gov/Blast.cgi#15011974%2315011974) | 18.9 | 63% | 359 |
| [NP_000019.2](http://www.ncbi.nlm.nih.gov/entrez/query.fcgi?cmd=Retrieve&db=Protein&list_uids=116734847&dopt=GenPept&RID=T284T3FC012&log$=prottop&blast_rank=92) | amylo-1,6-glucosidase, 4-alpha-glucanotransferase isoform 1 [Homo sapiens] >ref|NP_000634.2| amylo-1,6-glucosidase, 4-alpha-glucanotransferase isoform 1 [Homo sapiens] >ref|NP_000635.2| amylo-1,6-glucosidase, 4-alpha-glucanotransferase isoform 1 [Homo sapiens] >ref|NP_000633.2| amylo-1,6-glucosidase, 4-alpha-glucanotransferase isoform 1 [Homo sapiens] | [18.9](http://blast.ncbi.nlm.nih.gov/Blast.cgi#116734847%23116734847) | 30.1 | 72% | 359 |
| [NP_001876.1](http://www.ncbi.nlm.nih.gov/entrez/query.fcgi?cmd=Retrieve&db=Protein&list_uids=4503057&dopt=GenPept&RID=T284T3FC012&log$=prottop&blast_rank=93) | crystallin, alpha B [Homo sapiens] | [18.9](http://blast.ncbi.nlm.nih.gov/Blast.cgi#4503057%234503057) | 18.9 | 54% | 359 |
| [NP_065702.2](http://www.ncbi.nlm.nih.gov/entrez/query.fcgi?cmd=Retrieve&db=Protein&list_uids=11037063&dopt=GenPept&RID=T284T3FC012&log$=prottop&blast_rank=94) | pellino protein [Homo sapiens] | [18.9](http://blast.ncbi.nlm.nih.gov/Blast.cgi#11037063%2311037063) | 18.9 | 54% | 359 |
| [NP_996561.1](http://www.ncbi.nlm.nih.gov/entrez/query.fcgi?cmd=Retrieve&db=Protein&list_uids=45643127&dopt=GenPept&RID=T284T3FC012&log$=prottop&blast_rank=95) | guanine nucleotide binding protein-like 3 isoform 2 [Homo sapiens] >ref|NP_996562.1| guanine nucleotide binding protein-like 3 isoform 2 [Homo sapiens] | [18.9](http://blast.ncbi.nlm.nih.gov/Blast.cgi#45643127%2345643127) | 18.9 | 100% | 359 |
| [NP_001035957.1](http://www.ncbi.nlm.nih.gov/entrez/query.fcgi?cmd=Retrieve&db=Protein&list_uids=109826564&dopt=GenPept&RID=T284T3FC012&log$=prottop&blast_rank=96) | neurofibromin isoform 1 [Homo sapiens] | [18.9](http://blast.ncbi.nlm.nih.gov/Blast.cgi#109826564%23109826564) | 36.1 | 90% | 359 |
| [NP_003623.1](http://www.ncbi.nlm.nih.gov/entrez/query.fcgi?cmd=Retrieve&db=Protein&list_uids=4505463&dopt=GenPept&RID=T284T3FC012&log$=prottop&blast_rank=97) | contactin associated protein 1 precursor [Homo sapiens] | [18.9](http://blast.ncbi.nlm.nih.gov/Blast.cgi#4505463%234505463) | 18.9 | 54% | 359 |
| [NP_115575.1](http://www.ncbi.nlm.nih.gov/entrez/query.fcgi?cmd=Retrieve&db=Protein&list_uids=74136549&dopt=GenPept&RID=T284T3FC012&log$=prottop&blast_rank=98) | AT rich interactive domain 5B (MRF1-like) [Homo sapiens] | [18.9](http://blast.ncbi.nlm.nih.gov/Blast.cgi#74136549%2374136549) | 36.1 | 81% | 359 |
| [NP_001323.1](http://www.ncbi.nlm.nih.gov/entrez/query.fcgi?cmd=Retrieve&db=Protein&list_uids=11034811&dopt=GenPept&RID=T284T3FC012&log$=prottop&blast_rank=99) | catenin (cadherin-associated protein), delta 2 (neural plakophilin-related arm-repeat protein) [Homo sapiens] | [18.9](http://blast.ncbi.nlm.nih.gov/Blast.cgi#11034811%2311034811) | 18.9 | 54% | 359 |
| [NP_659502.2](http://www.ncbi.nlm.nih.gov/entrez/query.fcgi?cmd=Retrieve&db=Protein&list_uids=148612798&dopt=GenPept&RID=T284T3FC012&log$=prottop&blast_rank=100) | pellino 3 isoform 1 [Homo sapiens] | [18.9](http://blast.ncbi.nlm.nih.gov/Blast.cgi#148612798%23148612798) | 18.9 | 54% | 359 |
