# Supplementary material for: A high throughput method for identifying personalized tumor-associated antigens
Source: Oncotarget. 2010 Jun 27;1(2):148–55. doi: 10.18632/oncotarget.118 (PMC2920534; doi:10.18632/oncotarget.118)
Supplement: Supplemental Table 4 [file oncotarget-01-148-s004.doc]

**IgG Patient C**

| **Accession** | **Proteins with a match to KENGRSPTHS peptide** | **[Max score](http://blast.ncbi.nlm.nih.gov/Blast.cgi?CMD=Get&ALIGNMENTS=100&ALIGNMENT_VIEW=Pairwise&CDD_SEARCH_STATE=1&DATABASE_SORT=0&DESCRIPTIONS=100&ENTREZ_QUERY=txid9606 %5BORGN%5D&FIRST_QUERY_NUM=0&FORMAT_OBJECT=Alignment&FORMAT_PAGE_TARGET=&FORMAT_TYPE=HTML&GET_SEQUENCE=yes&I_THRESH=&MASK_CHAR=2&MASK_COLOR=1&NEW_DESIGN=on&NEW_VIEW=yes&NUM_OVERVIEW=100&OLD_BLAST=false&PAGE=Proteins&QUERY_INDEX=0&QUERY_NUMBER=0&RESULTS_PAGE_TARGET=&RID=T2G464PN01S&SHOW_LINKOUT=yes&SHOW_OVERVIEW=yes&STEP_NUMBER=&WORD_SIZE=2&DISPLAY_SORT=1&HSP_SORT=1" \l "sort_mark)** | **[Total score](http://blast.ncbi.nlm.nih.gov/Blast.cgi?CMD=Get&ALIGNMENTS=100&ALIGNMENT_VIEW=Pairwise&CDD_SEARCH_STATE=1&DATABASE_SORT=0&DESCRIPTIONS=100&ENTREZ_QUERY=txid9606 %5BORGN%5D&FIRST_QUERY_NUM=0&FORMAT_OBJECT=Alignment&FORMAT_PAGE_TARGET=&FORMAT_TYPE=HTML&GET_SEQUENCE=yes&I_THRESH=&MASK_CHAR=2&MASK_COLOR=1&NEW_DESIGN=on&NEW_VIEW=yes&NUM_OVERVIEW=100&OLD_BLAST=false&PAGE=Proteins&QUERY_INDEX=0&QUERY_NUMBER=0&RESULTS_PAGE_TARGET=&RID=T2G464PN01S&SHOW_LINKOUT=yes&SHOW_OVERVIEW=yes&STEP_NUMBER=&WORD_SIZE=2&DISPLAY_SORT=2&HSP_SORT=1" \l "sort_mark)** | **[Query coverage](http://blast.ncbi.nlm.nih.gov/Blast.cgi?CMD=Get&ALIGNMENTS=100&ALIGNMENT_VIEW=Pairwise&CDD_SEARCH_STATE=1&DATABASE_SORT=0&DESCRIPTIONS=100&ENTREZ_QUERY=txid9606 %5BORGN%5D&FIRST_QUERY_NUM=0&FORMAT_OBJECT=Alignment&FORMAT_PAGE_TARGET=&FORMAT_TYPE=HTML&GET_SEQUENCE=yes&I_THRESH=&MASK_CHAR=2&MASK_COLOR=1&NEW_DESIGN=on&NEW_VIEW=yes&NUM_OVERVIEW=100&OLD_BLAST=false&PAGE=Proteins&QUERY_INDEX=0&QUERY_NUMBER=0&RESULTS_PAGE_TARGET=&RID=T2G464PN01S&SHOW_LINKOUT=yes&SHOW_OVERVIEW=yes&STEP_NUMBER=&WORD_SIZE=2&DISPLAY_SORT=4&HSP_SORT=0" \l "sort_mark)** | **[E value](http://blast.ncbi.nlm.nih.gov/Blast.cgi?CMD=Get&ALIGNMENTS=100&ALIGNMENT_VIEW=Pairwise&CDD_SEARCH_STATE=1&DATABASE_SORT=0&DESCRIPTIONS=100&ENTREZ_QUERY=txid9606 %5BORGN%5D&FIRST_QUERY_NUM=0&FORMAT_OBJECT=Alignment&FORMAT_PAGE_TARGET=&FORMAT_TYPE=HTML&GET_SEQUENCE=yes&I_THRESH=&MASK_CHAR=2&MASK_COLOR=1&NEW_DESIGN=on&NEW_VIEW=yes&NUM_OVERVIEW=100&OLD_BLAST=false&PAGE=Proteins&QUERY_INDEX=0&QUERY_NUMBER=0&RESULTS_PAGE_TARGET=&RID=T2G464PN01S&SHOW_LINKOUT=yes&SHOW_OVERVIEW=yes&STEP_NUMBER=&WORD_SIZE=2&DISPLAY_SORT=0&HSP_SORT=0" \l "sort_mark)** |
| --- | --- | --- | --- | --- | --- |
| [NP_036433.2](http://www.ncbi.nlm.nih.gov/entrez/query.fcgi?cmd=Retrieve&db=Protein&list_uids=27436957&dopt=GenPept&RID=T2G464PN01S&log$=prottop&blast_rank=1) | membrane associated guanylate kinase, WW and PDZ domain containing 2 [Homo sapiens] | [23.1](http://blast.ncbi.nlm.nih.gov/Blast.cgi#27436957%2327436957) | 23.1 | 80% | 19 |
| [NP_002904.3](http://www.ncbi.nlm.nih.gov/entrez/query.fcgi?cmd=Retrieve&db=Protein&list_uids=32528306&dopt=GenPept&RID=T2G464PN01S&log$=prottop&blast_rank=2) | replication factor C large subunit [Homo sapiens] | [22.7](http://blast.ncbi.nlm.nih.gov/Blast.cgi#32528306%2332528306) | 22.7 | 100% | 25 |
| [NP_055790.1](http://www.ncbi.nlm.nih.gov/entrez/query.fcgi?cmd=Retrieve&db=Protein&list_uids=45120119&dopt=GenPept&RID=T2G464PN01S&log$=prottop&blast_rank=3) | microtubule associated serine/threonine kinase 1 [Homo sapiens] | [21.8](http://blast.ncbi.nlm.nih.gov/Blast.cgi#45120119%2345120119) | 52.8 | 70% | 46 |
| [NP_057427.3](http://www.ncbi.nlm.nih.gov/entrez/query.fcgi?cmd=Retrieve&db=Protein&list_uids=55770834&dopt=GenPept&RID=T2G464PN01S&log$=prottop&blast_rank=4) | centromere protein F [Homo sapiens] | [21.4](http://blast.ncbi.nlm.nih.gov/Blast.cgi#55770834%2355770834) | 37.8 | 90% | 62 |
| [NP_001073961.1](http://www.ncbi.nlm.nih.gov/entrez/query.fcgi?cmd=Retrieve&db=Protein&list_uids=122937420&dopt=GenPept&RID=T2G464PN01S&log$=prottop&blast_rank=5) | plasma kallikrein-like protein 4 precursor [Homo sapiens] | [21.4](http://blast.ncbi.nlm.nih.gov/Blast.cgi#122937420%23122937420) | 21.4 | 70% | 62 |
| [NP_079010.2](http://www.ncbi.nlm.nih.gov/entrez/query.fcgi?cmd=Retrieve&db=Protein&list_uids=19923599&dopt=GenPept&RID=T2G464PN01S&log$=prottop&blast_rank=6) | calmin [Homo sapiens] | [21.4](http://blast.ncbi.nlm.nih.gov/Blast.cgi#19923599%2319923599) | 21.4 | 60% | 62 |
| [NP_061848.2](http://www.ncbi.nlm.nih.gov/entrez/query.fcgi?cmd=Retrieve&db=Protein&list_uids=52627149&dopt=GenPept&RID=T2G464PN01S&log$=prottop&blast_rank=7) | telomeric repeat binding factor 2, interacting protein [Homo sapiens] | [21.4](http://blast.ncbi.nlm.nih.gov/Blast.cgi#52627149%2352627149) | 38.6 | 100% | 62 |
| [XP_002347412.1](http://www.ncbi.nlm.nih.gov/entrez/query.fcgi?cmd=Retrieve&db=Protein&list_uids=239750388&dopt=GenPept&RID=T2G464PN01S&log$=prottop&blast_rank=8) | PREDICTED: hypothetical protein XP_002347412 [Homo sapiens] >ref|XP_002344733.1| PREDICTED: hypothetical protein [Homo sapiens] | [21.0](http://blast.ncbi.nlm.nih.gov/Blast.cgi#239750388%23239750388) | 21.0 | 90% | 83 |
| [NP_001014975.1](http://www.ncbi.nlm.nih.gov/entrez/query.fcgi?cmd=Retrieve&db=Protein&list_uids=62739188&dopt=GenPept&RID=T2G464PN01S&log$=prottop&blast_rank=9) | complement factor H isoform b precursor [Homo sapiens] | [20.6](http://blast.ncbi.nlm.nih.gov/Blast.cgi#62739188%2362739188) | 20.6 | 70% | 111 |
| [NP_000177.2](http://www.ncbi.nlm.nih.gov/entrez/query.fcgi?cmd=Retrieve&db=Protein&list_uids=62739186&dopt=GenPept&RID=T2G464PN01S&log$=prottop&blast_rank=10) | complement factor H isoform a precursor [Homo sapiens] | [20.6](http://blast.ncbi.nlm.nih.gov/Blast.cgi#62739186%2362739186) | 20.6 | 70% | 111 |
| [XP_002345546.1](http://www.ncbi.nlm.nih.gov/entrez/query.fcgi?cmd=Retrieve&db=Protein&list_uids=239757736&dopt=GenPept&RID=T2G464PN01S&log$=prottop&blast_rank=11) | PREDICTED: hypothetical protein [Homo sapiens] | [20.2](http://blast.ncbi.nlm.nih.gov/Blast.cgi#239757736%23239757736) | 20.2 | 70% | 149 |
| [XP_002348167.1](http://www.ncbi.nlm.nih.gov/entrez/query.fcgi?cmd=Retrieve&db=Protein&list_uids=239752235&dopt=GenPept&RID=T2G464PN01S&log$=prottop&blast_rank=12) | PREDICTED: hypothetical protein XP_002348167 [Homo sapiens] | [20.2](http://blast.ncbi.nlm.nih.gov/Blast.cgi#239752235%23239752235) | 20.2 | 70% | 149 |
| [NP_001138818.1](http://www.ncbi.nlm.nih.gov/entrez/query.fcgi?cmd=Retrieve&db=Protein&list_uids=223890219&dopt=GenPept&RID=T2G464PN01S&log$=prottop&blast_rank=13) | RNA binding motif protein, X-linked-like 3 [Homo sapiens] | [20.2](http://blast.ncbi.nlm.nih.gov/Blast.cgi#223890219%23223890219) | 144 | 70% | 149 |
| [NP_003778.2](http://www.ncbi.nlm.nih.gov/entrez/query.fcgi?cmd=Retrieve&db=Protein&list_uids=148235600&dopt=GenPept&RID=T2G464PN01S&log$=prottop&blast_rank=14) | nucleolar protein 4 [Homo sapiens] | [20.2](http://blast.ncbi.nlm.nih.gov/Blast.cgi#148235600%23148235600) | 38.6 | 100% | 149 |
| [NP_001163935.1](http://www.ncbi.nlm.nih.gov/entrez/query.fcgi?cmd=Retrieve&db=Protein&list_uids=281427168&dopt=GenPept&RID=T2G464PN01S&log$=prottop&blast_rank=15) | PCTAIRE protein kinase 2 isoform 2 [Homo sapiens] | [20.2](http://blast.ncbi.nlm.nih.gov/Blast.cgi#281427168%23281427168) | 20.2 | 80% | 149 |
| [NP_002586.2](http://www.ncbi.nlm.nih.gov/entrez/query.fcgi?cmd=Retrieve&db=Protein&list_uids=37595545&dopt=GenPept&RID=T2G464PN01S&log$=prottop&blast_rank=16) | PCTAIRE protein kinase 2 isoform 1 [Homo sapiens] | [20.2](http://blast.ncbi.nlm.nih.gov/Blast.cgi#37595545%2337595545) | 20.2 | 80% | 149 |
| [NP_036252.1](http://www.ncbi.nlm.nih.gov/entrez/query.fcgi?cmd=Retrieve&db=Protein&list_uids=11321634&dopt=GenPept&RID=T2G464PN01S&log$=prottop&blast_rank=17) | CD2-associated protein [Homo sapiens] | [20.2](http://blast.ncbi.nlm.nih.gov/Blast.cgi#11321634%2311321634) | 20.2 | 70% | 149 |
| [NP_000697.1](http://www.ncbi.nlm.nih.gov/entrez/query.fcgi?cmd=Retrieve&db=Protein&list_uids=4502331&dopt=GenPept&RID=T2G464PN01S&log$=prottop&blast_rank=18) | arginine vasopressin receptor 1A [Homo sapiens] | [20.2](http://blast.ncbi.nlm.nih.gov/Blast.cgi#4502331%234502331) | 20.2 | 80% | 149 |
| [NP_001197.1](http://www.ncbi.nlm.nih.gov/entrez/query.fcgi?cmd=Retrieve&db=Protein&list_uids=4557375&dopt=GenPept&RID=T2G464PN01S&log$=prottop&blast_rank=19) | Kruppel-like factor 9 [Homo sapiens] | [20.2](http://blast.ncbi.nlm.nih.gov/Blast.cgi#4557375%234557375) | 20.2 | 90% | 149 |
| [XP_002346297.1](http://www.ncbi.nlm.nih.gov/entrez/query.fcgi?cmd=Retrieve&db=Protein&list_uids=239758105&dopt=GenPept&RID=T2G464PN01S&log$=prottop&blast_rank=20) | PREDICTED: similar to Protein FAM27D1 [Homo sapiens] | [19.7](http://blast.ncbi.nlm.nih.gov/Blast.cgi#239758105%23239758105) | 19.7 | 90% | 200 |
| [NP_663628.2](http://www.ncbi.nlm.nih.gov/entrez/query.fcgi?cmd=Retrieve&db=Protein&list_uids=156938279&dopt=GenPept&RID=T2G464PN01S&log$=prottop&blast_rank=21) | transcription elongation factor B polypeptide 3C [Homo sapiens] | [19.7](http://blast.ncbi.nlm.nih.gov/Blast.cgi#156938279%23156938279) | 19.7 | 90% | 200 |
| [NP_057511.2](http://www.ncbi.nlm.nih.gov/entrez/query.fcgi?cmd=Retrieve&db=Protein&list_uids=45439357&dopt=GenPept&RID=T2G464PN01S&log$=prottop&blast_rank=22) | elongin A2 [Homo sapiens] | [19.7](http://blast.ncbi.nlm.nih.gov/Blast.cgi#45439357%2345439357) | 33.9 | 100% | 200 |
| [NP_001094287.1](http://www.ncbi.nlm.nih.gov/entrez/query.fcgi?cmd=Retrieve&db=Protein&list_uids=154816198&dopt=GenPept&RID=T2G464PN01S&log$=prottop&blast_rank=23) | transcription elongation factor B polypeptide 3C-like [Homo sapiens] | [19.7](http://blast.ncbi.nlm.nih.gov/Blast.cgi#154816198%23154816198) | 19.7 | 90% | 200 |
| [XP_002344750.1](http://www.ncbi.nlm.nih.gov/entrez/query.fcgi?cmd=Retrieve&db=Protein&list_uids=239755939&dopt=GenPept&RID=T2G464PN01S&log$=prottop&blast_rank=24) | PREDICTED: hypothetical protein [Homo sapiens] | [19.3](http://blast.ncbi.nlm.nih.gov/Blast.cgi#239755939%23239755939) | 19.3 | 50% | 268 |
| [XP_002345976.1](http://www.ncbi.nlm.nih.gov/entrez/query.fcgi?cmd=Retrieve&db=Protein&list_uids=239754405&dopt=GenPept&RID=T2G464PN01S&log$=prottop&blast_rank=25) | PREDICTED: hypothetical protein [Homo sapiens] | [19.3](http://blast.ncbi.nlm.nih.gov/Blast.cgi#239754405%23239754405) | 19.3 | 50% | 268 |
| [XP_002346866.1](http://www.ncbi.nlm.nih.gov/entrez/query.fcgi?cmd=Retrieve&db=Protein&list_uids=239748958&dopt=GenPept&RID=T2G464PN01S&log$=prottop&blast_rank=26) | PREDICTED: hypothetical protein XP_002346866 [Homo sapiens] | [19.3](http://blast.ncbi.nlm.nih.gov/Blast.cgi#239748958%23239748958) | 19.3 | 50% | 268 |
| [NP_653267.2](http://www.ncbi.nlm.nih.gov/entrez/query.fcgi?cmd=Retrieve&db=Protein&list_uids=222144249&dopt=GenPept&RID=T2G464PN01S&log$=prottop&blast_rank=27) | dynein heavy chain domain 1 isoform 1 [Homo sapiens] | [19.3](http://blast.ncbi.nlm.nih.gov/Blast.cgi#222144249%23222144249) | 36.5 | 80% | 268 |
| [NP_001129494.1](http://www.ncbi.nlm.nih.gov/entrez/query.fcgi?cmd=Retrieve&db=Protein&list_uids=209862845&dopt=GenPept&RID=T2G464PN01S&log$=prottop&blast_rank=28) | nuclear factor of activated T-cells, cytoplasmic, calcineurin-dependent 4 isoform 1 [Homo sapiens] | [19.3](http://blast.ncbi.nlm.nih.gov/Blast.cgi#209862845%23209862845) | 19.3 | 80% | 268 |
| [NP_001092105.1](http://www.ncbi.nlm.nih.gov/entrez/query.fcgi?cmd=Retrieve&db=Protein&list_uids=148839346&dopt=GenPept&RID=T2G464PN01S&log$=prottop&blast_rank=29) | seizure related 6 homolog isoform 2 [Homo sapiens] | [19.3](http://blast.ncbi.nlm.nih.gov/Blast.cgi#148839346%23148839346) | 30.1 | 90% | 268 |
| [NP_849191.3](http://www.ncbi.nlm.nih.gov/entrez/query.fcgi?cmd=Retrieve&db=Protein&list_uids=148839280&dopt=GenPept&RID=T2G464PN01S&log$=prottop&blast_rank=30) | seizure related 6 homolog isoform 1 [Homo sapiens] | [19.3](http://blast.ncbi.nlm.nih.gov/Blast.cgi#148839280%23148839280) | 30.1 | 90% | 268 |
| [NP_004232.2](http://www.ncbi.nlm.nih.gov/entrez/query.fcgi?cmd=Retrieve&db=Protein&list_uids=68342036&dopt=GenPept&RID=T2G464PN01S&log$=prottop&blast_rank=31) | jumonji domain containing 1C isoform b [Homo sapiens] | [19.3](http://blast.ncbi.nlm.nih.gov/Blast.cgi#68342036%2368342036) | 19.3 | 50% | 268 |
| [NP_001070729.1](http://www.ncbi.nlm.nih.gov/entrez/query.fcgi?cmd=Retrieve&db=Protein&list_uids=116256445&dopt=GenPept&RID=T2G464PN01S&log$=prottop&blast_rank=32) | nuclear receptor co-repressor 2 isoform 2 [Homo sapiens] | [19.3](http://blast.ncbi.nlm.nih.gov/Blast.cgi#116256445%23116256445) | 35.6 | 90% | 268 |
| [NP_006303.3](http://www.ncbi.nlm.nih.gov/entrez/query.fcgi?cmd=Retrieve&db=Protein&list_uids=116256453&dopt=GenPept&RID=T2G464PN01S&log$=prottop&blast_rank=33) | nuclear receptor co-repressor 2 isoform 1 [Homo sapiens] | [19.3](http://blast.ncbi.nlm.nih.gov/Blast.cgi#116256453%23116256453) | 35.6 | 90% | 268 |
| [NP_001035192.1](http://www.ncbi.nlm.nih.gov/entrez/query.fcgi?cmd=Retrieve&db=Protein&list_uids=91932793&dopt=GenPept&RID=T2G464PN01S&log$=prottop&blast_rank=34) | transmembrane protein 10 isoform c [Homo sapiens] | [19.3](http://blast.ncbi.nlm.nih.gov/Blast.cgi#91932793%2391932793) | 19.3 | 50% | 268 |
| [NP_001035191.1](http://www.ncbi.nlm.nih.gov/entrez/query.fcgi?cmd=Retrieve&db=Protein&list_uids=91932795&dopt=GenPept&RID=T2G464PN01S&log$=prottop&blast_rank=35) | transmembrane protein 10 isoform b [Homo sapiens] | [19.3](http://blast.ncbi.nlm.nih.gov/Blast.cgi#91932795%2391932795) | 19.3 | 50% | 268 |
| [NP_001137231.1](http://www.ncbi.nlm.nih.gov/entrez/query.fcgi?cmd=Retrieve&db=Protein&list_uids=219555704&dopt=GenPept&RID=T2G464PN01S&log$=prottop&blast_rank=36) | leucine rich repeat containing 27 isoform c [Homo sapiens] | [19.3](http://blast.ncbi.nlm.nih.gov/Blast.cgi#219555704%23219555704) | 19.3 | 60% | 268 |
| [NP_116165.1](http://www.ncbi.nlm.nih.gov/entrez/query.fcgi?cmd=Retrieve&db=Protein&list_uids=118600981&dopt=GenPept&RID=T2G464PN01S&log$=prottop&blast_rank=37) | jumonji domain containing 1C isoform a [Homo sapiens] | [19.3](http://blast.ncbi.nlm.nih.gov/Blast.cgi#118600981%23118600981) | 19.3 | 50% | 268 |
| [NP_004545.2](http://www.ncbi.nlm.nih.gov/entrez/query.fcgi?cmd=Retrieve&db=Protein&list_uids=27886563&dopt=GenPept&RID=T2G464PN01S&log$=prottop&blast_rank=38) | nuclear factor of activated T-cells, cytoplasmic, calcineurin-dependent 4 isoform 2 [Homo sapiens] | [19.3](http://blast.ncbi.nlm.nih.gov/Blast.cgi#27886563%2327886563) | 19.3 | 80% | 268 |
| [NP_001017964.1](http://www.ncbi.nlm.nih.gov/entrez/query.fcgi?cmd=Retrieve&db=Protein&list_uids=63054868&dopt=GenPept&RID=T2G464PN01S&log$=prottop&blast_rank=39) | YdjC homolog [Homo sapiens] | [19.3](http://blast.ncbi.nlm.nih.gov/Blast.cgi#63054868%2363054868) | 19.3 | 60% | 268 |
| [NP_005344.2](http://www.ncbi.nlm.nih.gov/entrez/query.fcgi?cmd=Retrieve&db=Protein&list_uids=62548866&dopt=GenPept&RID=T2G464PN01S&log$=prottop&blast_rank=40) | integrin, alpha D precursor [Homo sapiens] | [19.3](http://blast.ncbi.nlm.nih.gov/Blast.cgi#62548866%2362548866) | 19.3 | 90% | 268 |
| [NP_004783.2](http://www.ncbi.nlm.nih.gov/entrez/query.fcgi?cmd=Retrieve&db=Protein&list_uids=42560244&dopt=GenPept&RID=T2G464PN01S&log$=prottop&blast_rank=41) | peptidylprolyl isomerase G [Homo sapiens] | [19.3](http://blast.ncbi.nlm.nih.gov/Blast.cgi#42560244%2342560244) | 33.5 | 90% | 268 |
| [NP_149984.1](http://www.ncbi.nlm.nih.gov/entrez/query.fcgi?cmd=Retrieve&db=Protein&list_uids=15082236&dopt=GenPept&RID=T2G464PN01S&log$=prottop&blast_rank=42) | transmembrane protein 10 isoform a [Homo sapiens] | [19.3](http://blast.ncbi.nlm.nih.gov/Blast.cgi#15082236%2315082236) | 19.3 | 50% | 268 |
| [NP_056411.1](http://www.ncbi.nlm.nih.gov/entrez/query.fcgi?cmd=Retrieve&db=Protein&list_uids=11496281&dopt=GenPept&RID=T2G464PN01S&log$=prottop&blast_rank=43) | kallikrein 13 precursor [Homo sapiens] | [19.3](http://blast.ncbi.nlm.nih.gov/Blast.cgi#11496281%2311496281) | 19.3 | 50% | 268 |
| [NP_001071096.1](http://www.ncbi.nlm.nih.gov/entrez/query.fcgi?cmd=Retrieve&db=Protein&list_uids=117606362&dopt=GenPept&RID=T2G464PN01S&log$=prottop&blast_rank=44) | anterior pharynx defective 1 homolog A isoform 1 [Homo sapiens] | [19.3](http://blast.ncbi.nlm.nih.gov/Blast.cgi#117606362%23117606362) | 19.3 | 60% | 268 |
| [NP_004340.1](http://www.ncbi.nlm.nih.gov/entrez/query.fcgi?cmd=Retrieve&db=Protein&list_uids=4757916&dopt=GenPept&RID=T2G464PN01S&log$=prottop&blast_rank=45) | acute myelogenous leukemia 1 translocation 1 protein isoform MTG8a [Homo sapiens] | [19.3](http://blast.ncbi.nlm.nih.gov/Blast.cgi#4757916%234757916) | 19.3 | 100% | 268 |
| [NP_057106.2](http://www.ncbi.nlm.nih.gov/entrez/query.fcgi?cmd=Retrieve&db=Protein&list_uids=117606357&dopt=GenPept&RID=T2G464PN01S&log$=prottop&blast_rank=46) | anterior pharynx defective 1 homolog A isoform 2 [Homo sapiens] | [19.3](http://blast.ncbi.nlm.nih.gov/Blast.cgi#117606357%23117606357) | 19.3 | 60% | 268 |
| [NP_783553.1](http://www.ncbi.nlm.nih.gov/entrez/query.fcgi?cmd=Retrieve&db=Protein&list_uids=28329419&dopt=GenPept&RID=T2G464PN01S&log$=prottop&blast_rank=47) | acute myelogenous leukemia 1 translocation 1 protein isoform MTG8c [Homo sapiens] >ref|NP_783554.1| acute myelogenous leukemia 1 translocation 1 protein isoform MTG8c [Homo sapiens] | [19.3](http://blast.ncbi.nlm.nih.gov/Blast.cgi#28329419%2328329419) | 19.3 | 100% | 268 |
| [NP_783552.1](http://www.ncbi.nlm.nih.gov/entrez/query.fcgi?cmd=Retrieve&db=Protein&list_uids=28329416&dopt=GenPept&RID=T2G464PN01S&log$=prottop&blast_rank=48) | acute myelogenous leukemia 1 translocation 1 protein isoform MTG8b [Homo sapiens] | [19.3](http://blast.ncbi.nlm.nih.gov/Blast.cgi#28329416%2328329416) | 19.3 | 100% | 268 |
| [XP_002343354.1](http://www.ncbi.nlm.nih.gov/entrez/query.fcgi?cmd=Retrieve&db=Protein&list_uids=239745062&dopt=GenPept&RID=T2G464PN01S&log$=prottop&blast_rank=49) | PREDICTED: hypothetical protein [Homo sapiens] | [18.9](http://blast.ncbi.nlm.nih.gov/Blast.cgi#239745062%23239745062) | 18.9 | 70% | 359 |
| [XP_291816.7](http://www.ncbi.nlm.nih.gov/entrez/query.fcgi?cmd=Retrieve&db=Protein&list_uids=239744300&dopt=GenPept&RID=T2G464PN01S&log$=prottop&blast_rank=50) | PREDICTED: otogelin isoform 2 [Homo sapiens] >ref|XP_001717583.2| PREDICTED: otogelin isoform 1 [Homo sapiens] | [18.9](http://blast.ncbi.nlm.nih.gov/Blast.cgi#239744300%23239744300) | 34.4 | 80% | 359 |
| [NP_001138472.1](http://www.ncbi.nlm.nih.gov/entrez/query.fcgi?cmd=Retrieve&db=Protein&list_uids=223468597&dopt=GenPept&RID=T2G464PN01S&log$=prottop&blast_rank=51) | integrin alpha-V isoform 3 precursor [Homo sapiens] | [18.9](http://blast.ncbi.nlm.nih.gov/Blast.cgi#223468597%23223468597) | 33.9 | 80% | 359 |
| [NP_001138471.1](http://www.ncbi.nlm.nih.gov/entrez/query.fcgi?cmd=Retrieve&db=Protein&list_uids=223468595&dopt=GenPept&RID=T2G464PN01S&log$=prottop&blast_rank=52) | integrin alpha-V isoform 2 [Homo sapiens] | [18.9](http://blast.ncbi.nlm.nih.gov/Blast.cgi#223468595%23223468595) | 33.9 | 80% | 359 |
| [NP_996816.2](http://www.ncbi.nlm.nih.gov/entrez/query.fcgi?cmd=Retrieve&db=Protein&list_uids=219842266&dopt=GenPept&RID=T2G464PN01S&log$=prottop&blast_rank=53) | usherin isoform B [Homo sapiens] | [18.9](http://blast.ncbi.nlm.nih.gov/Blast.cgi#219842266%23219842266) | 32.2 | 70% | 359 |
| [NP_056056.2](http://www.ncbi.nlm.nih.gov/entrez/query.fcgi?cmd=Retrieve&db=Protein&list_uids=209862789&dopt=GenPept&RID=T2G464PN01S&log$=prottop&blast_rank=54) | microtubule associated monoxygenase, calponin and LIM domain containing 3 isoform 1 [Homo sapiens] | [18.9](http://blast.ncbi.nlm.nih.gov/Blast.cgi#209862789%23209862789) | 31.8 | 70% | 359 |
| [NP_001093868.1](http://www.ncbi.nlm.nih.gov/entrez/query.fcgi?cmd=Retrieve&db=Protein&list_uids=156086740&dopt=GenPept&RID=T2G464PN01S&log$=prottop&blast_rank=55) | RAP1 GTPase activating protein 2 isoform 2 [Homo sapiens] | [18.9](http://blast.ncbi.nlm.nih.gov/Blast.cgi#156086740%23156086740) | 34.4 | 80% | 359 |
| [NP_055900.4](http://www.ncbi.nlm.nih.gov/entrez/query.fcgi?cmd=Retrieve&db=Protein&list_uids=156086724&dopt=GenPept&RID=T2G464PN01S&log$=prottop&blast_rank=56) | RAP1 GTPase activating protein 2 isoform 1 [Homo sapiens] | [18.9](http://blast.ncbi.nlm.nih.gov/Blast.cgi#156086724%23156086724) | 34.4 | 80% | 359 |
| [XP_946095.3](http://www.ncbi.nlm.nih.gov/entrez/query.fcgi?cmd=Retrieve&db=Protein&list_uids=239749989&dopt=GenPept&RID=T2G464PN01S&log$=prottop&blast_rank=57) | PREDICTED: otogelin isoform 2 [Homo sapiens] | [18.9](http://blast.ncbi.nlm.nih.gov/Blast.cgi#239749989%23239749989) | 34.4 | 80% | 359 |
| [NP_001009899.2](http://www.ncbi.nlm.nih.gov/entrez/query.fcgi?cmd=Retrieve&db=Protein&list_uids=114431248&dopt=GenPept&RID=T2G464PN01S&log$=prottop&blast_rank=58) | hypothetical protein LOC205717 [Homo sapiens] | [18.9](http://blast.ncbi.nlm.nih.gov/Blast.cgi#114431248%23114431248) | 18.9 | 50% | 359 |
| [NP_001073981.1](http://www.ncbi.nlm.nih.gov/entrez/query.fcgi?cmd=Retrieve&db=Protein&list_uids=122937472&dopt=GenPept&RID=T2G464PN01S&log$=prottop&blast_rank=59) | bicaudal C homolog 1 [Homo sapiens] | [18.9](http://blast.ncbi.nlm.nih.gov/Blast.cgi#122937472%23122937472) | 18.9 | 60% | 359 |
| [NP_703157.2](http://www.ncbi.nlm.nih.gov/entrez/query.fcgi?cmd=Retrieve&db=Protein&list_uids=45580707&dopt=GenPept&RID=T2G464PN01S&log$=prottop&blast_rank=60) | heparan sulfate 6-O-sulfotransferase 3 [Homo sapiens] >ref|XP_002344784.1| PREDICTED: heparan sulfate 6-O-sulfotransferase 3 [Homo sapiens] | [18.9](http://blast.ncbi.nlm.nih.gov/Blast.cgi#45580707%2345580707) | 18.9 | 60% | 359 |
| [NP_689865.1](http://www.ncbi.nlm.nih.gov/entrez/query.fcgi?cmd=Retrieve&db=Protein&list_uids=22749325&dopt=GenPept&RID=T2G464PN01S&log$=prottop&blast_rank=61) | zinc finger protein 48 [Homo sapiens] | [18.9](http://blast.ncbi.nlm.nih.gov/Blast.cgi#22749325%2322749325) | 18.9 | 60% | 359 |
| [NP_000029.2](http://www.ncbi.nlm.nih.gov/entrez/query.fcgi?cmd=Retrieve&db=Protein&list_uids=53759122&dopt=GenPept&RID=T2G464PN01S&log$=prottop&blast_rank=62) | adenomatous polyposis coli [Homo sapiens] >ref|NP_001120982.1| adenomatous polyposis coli [Homo sapiens] >ref|NP_001120983.1| adenomatous polyposis coli [Homo sapiens] | [18.9](http://blast.ncbi.nlm.nih.gov/Blast.cgi#53759122%2353759122) | 49.8 | 100% | 359 |
| [XP_002347263.1](http://www.ncbi.nlm.nih.gov/entrez/query.fcgi?cmd=Retrieve&db=Protein&list_uids=239749987&dopt=GenPept&RID=T2G464PN01S&log$=prottop&blast_rank=63) | PREDICTED: otogelin isoform 1 [Homo sapiens] | [18.9](http://blast.ncbi.nlm.nih.gov/Blast.cgi#239749987%23239749987) | 34.4 | 80% | 359 |
| [XP_002343092.1](http://www.ncbi.nlm.nih.gov/entrez/query.fcgi?cmd=Retrieve&db=Protein&list_uids=239744298&dopt=GenPept&RID=T2G464PN01S&log$=prottop&blast_rank=64) | PREDICTED: otogelin isoform 1 [Homo sapiens] >ref|XP_002344605.1| PREDICTED: otogelin isoform 2 [Homo sapiens] | [18.9](http://blast.ncbi.nlm.nih.gov/Blast.cgi#239744298%23239744298) | 34.4 | 80% | 359 |
| [NP_005924.2](http://www.ncbi.nlm.nih.gov/entrez/query.fcgi?cmd=Retrieve&db=Protein&list_uids=56550039&dopt=GenPept&RID=T2G464PN01S&log$=prottop&blast_rank=65) | myeloid/lymphoid or mixed-lineage leukemia protein [Homo sapiens] | [18.9](http://blast.ncbi.nlm.nih.gov/Blast.cgi#56550039%2356550039) | 94.2 | 100% | 359 |
| [NP_002201.1](http://www.ncbi.nlm.nih.gov/entrez/query.fcgi?cmd=Retrieve&db=Protein&list_uids=4504763&dopt=GenPept&RID=T2G464PN01S&log$=prottop&blast_rank=66) | integrin alpha-V isoform 1 precursor [Homo sapiens] | [18.9](http://blast.ncbi.nlm.nih.gov/Blast.cgi#4504763%234504763) | 33.9 | 80% | 359 |
| [NP_079285.2](http://www.ncbi.nlm.nih.gov/entrez/query.fcgi?cmd=Retrieve&db=Protein&list_uids=50083279&dopt=GenPept&RID=T2G464PN01S&log$=prottop&blast_rank=67) | centrosome protein 4 [Homo sapiens] | [18.9](http://blast.ncbi.nlm.nih.gov/Blast.cgi#50083279%2350083279) | 33.9 | 70% | 359 |
| [NP_057688.2](http://www.ncbi.nlm.nih.gov/entrez/query.fcgi?cmd=Retrieve&db=Protein&list_uids=38372909&dopt=GenPept&RID=T2G464PN01S&log$=prottop&blast_rank=68) | jumonji domain containing 1B [Homo sapiens] | [18.9](http://blast.ncbi.nlm.nih.gov/Blast.cgi#38372909%2338372909) | 35.6 | 80% | 359 |
| [NP_055693.4](http://www.ncbi.nlm.nih.gov/entrez/query.fcgi?cmd=Retrieve&db=Protein&list_uids=109948283&dopt=GenPept&RID=T2G464PN01S&log$=prottop&blast_rank=69) | KIAA0020 protein [Homo sapiens] | [18.9](http://blast.ncbi.nlm.nih.gov/Blast.cgi#109948283%23109948283) | 18.9 | 50% | 359 |
| [NP_004651.2](http://www.ncbi.nlm.nih.gov/entrez/query.fcgi?cmd=Retrieve&db=Protein&list_uids=13514809&dopt=GenPept&RID=T2G464PN01S&log$=prottop&blast_rank=70) | DEAD (Asp-Glu-Ala-Asp) box polypeptide 3, Y-linked [Homo sapiens] >ref|NP_001116137.1| DEAD (Asp-Glu-Ala-Asp) box polypeptide 3, Y-linked [Homo sapiens] | [18.9](http://blast.ncbi.nlm.nih.gov/Blast.cgi#13514809%2313514809) | 18.9 | 50% | 359 |
| [NP_612152.1](http://www.ncbi.nlm.nih.gov/entrez/query.fcgi?cmd=Retrieve&db=Protein&list_uids=19923084&dopt=GenPept&RID=T2G464PN01S&log$=prottop&blast_rank=71) | polycystin-1L1 [Homo sapiens] | [18.9](http://blast.ncbi.nlm.nih.gov/Blast.cgi#19923084%2319923084) | 18.9 | 70% | 359 |
| [NP_001245.1](http://www.ncbi.nlm.nih.gov/entrez/query.fcgi?cmd=Retrieve&db=Protein&list_uids=4502703&dopt=GenPept&RID=T2G464PN01S&log$=prottop&blast_rank=72) | cell division cycle 6 protein [Homo sapiens] | [18.9](http://blast.ncbi.nlm.nih.gov/Blast.cgi#4502703%234502703) | 18.9 | 100% | 359 |
| [NP_005617.2](http://www.ncbi.nlm.nih.gov/entrez/query.fcgi?cmd=Retrieve&db=Protein&list_uids=21361282&dopt=GenPept&RID=T2G464PN01S&log$=prottop&blast_rank=73) | splicing factor, arginine/serine-rich 4 [Homo sapiens] | [18.9](http://blast.ncbi.nlm.nih.gov/Blast.cgi#21361282%2321361282) | 64.1 | 100% | 359 |
| [NP_001973.2](http://www.ncbi.nlm.nih.gov/entrez/query.fcgi?cmd=Retrieve&db=Protein&list_uids=54792100&dopt=GenPept&RID=T2G464PN01S&log$=prottop&blast_rank=74) | erbB-3 isoform 1 precursor [Homo sapiens] | [18.9](http://blast.ncbi.nlm.nih.gov/Blast.cgi#54792100%2354792100) | 18.9 | 60% | 359 |
| [NP_055642.3](http://www.ncbi.nlm.nih.gov/entrez/query.fcgi?cmd=Retrieve&db=Protein&list_uids=114842410&dopt=GenPept&RID=T2G464PN01S&log$=prottop&blast_rank=75) | zinc finger CCCH-type containing 11A [Homo sapiens] | [18.9](http://blast.ncbi.nlm.nih.gov/Blast.cgi#114842410%23114842410) | 18.9 | 80% | 359 |
| [NP_149417.1](http://www.ncbi.nlm.nih.gov/entrez/query.fcgi?cmd=Retrieve&db=Protein&list_uids=15042961&dopt=GenPept&RID=T2G464PN01S&log$=prottop&blast_rank=76) | HLA-B associated transcript 4 [Homo sapiens] | [18.9](http://blast.ncbi.nlm.nih.gov/Blast.cgi#15042961%2315042961) | 18.9 | 90% | 359 |
| [NP_001347.3](http://www.ncbi.nlm.nih.gov/entrez/query.fcgi?cmd=Retrieve&db=Protein&list_uids=87196351&dopt=GenPept&RID=T2G464PN01S&log$=prottop&blast_rank=77) | DEAD/H (Asp-Glu-Ala-Asp/His) box polypeptide 3 [Homo sapiens] | [18.9](http://blast.ncbi.nlm.nih.gov/Blast.cgi#87196351%2387196351) | 18.9 | 50% | 359 |
| [NP_001161881.1](http://www.ncbi.nlm.nih.gov/entrez/query.fcgi?cmd=Retrieve&db=Protein&list_uids=270288802&dopt=GenPept&RID=T2G464PN01S&log$=prottop&blast_rank=78) | regulating synaptic membrane exocytosis 1 isoform 4 [Homo sapiens] | [18.5](http://blast.ncbi.nlm.nih.gov/Blast.cgi#270288802%23270288802) | 33.9 | 60% | 482 |
| [NP_001161880.1](http://www.ncbi.nlm.nih.gov/entrez/query.fcgi?cmd=Retrieve&db=Protein&list_uids=270288800&dopt=GenPept&RID=T2G464PN01S&log$=prottop&blast_rank=79) | regulating synaptic membrane exocytosis 1 isoform 3 [Homo sapiens] | [18.5](http://blast.ncbi.nlm.nih.gov/Blast.cgi#270288800%23270288800) | 33.9 | 60% | 482 |
| [NP_001161879.1](http://www.ncbi.nlm.nih.gov/entrez/query.fcgi?cmd=Retrieve&db=Protein&list_uids=270288798&dopt=GenPept&RID=T2G464PN01S&log$=prottop&blast_rank=80) | regulating synaptic membrane exocytosis 1 isoform 2 [Homo sapiens] | [18.5](http://blast.ncbi.nlm.nih.gov/Blast.cgi#270288798%23270288798) | 33.9 | 60% | 482 |
| [NP_001161081.1](http://www.ncbi.nlm.nih.gov/entrez/query.fcgi?cmd=Retrieve&db=Protein&list_uids=263190680&dopt=GenPept&RID=T2G464PN01S&log$=prottop&blast_rank=81) | GLI family zinc finger 1 isoform 3 [Homo sapiens] | [18.5](http://blast.ncbi.nlm.nih.gov/Blast.cgi#263190680%23263190680) | 18.5 | 50% | 482 |
| [NP_001159907.1](http://www.ncbi.nlm.nih.gov/entrez/query.fcgi?cmd=Retrieve&db=Protein&list_uids=261878616&dopt=GenPept&RID=T2G464PN01S&log$=prottop&blast_rank=82) | inter-alpha (globulin) inhibitor H1 isoform c [Homo sapiens] >ref|NP_001159908.1| inter-alpha (globulin) inhibitor H1 isoform c [Homo sapiens] | [18.5](http://blast.ncbi.nlm.nih.gov/Blast.cgi#261878616%23261878616) | 18.5 | 50% | 482 |
| [NP_001159906.1](http://www.ncbi.nlm.nih.gov/entrez/query.fcgi?cmd=Retrieve&db=Protein&list_uids=261878614&dopt=GenPept&RID=T2G464PN01S&log$=prottop&blast_rank=83) | inter-alpha (globulin) inhibitor H1 isoform b [Homo sapiens] | [18.5](http://blast.ncbi.nlm.nih.gov/Blast.cgi#261878614%23261878614) | 18.5 | 50% | 482 |
| [NP_001158136.1](http://www.ncbi.nlm.nih.gov/entrez/query.fcgi?cmd=Retrieve&db=Protein&list_uids=257467648&dopt=GenPept&RID=T2G464PN01S&log$=prottop&blast_rank=84) | microtubule associated serine/threonine kinase family member 4 isoform c [Homo sapiens] | [18.5](http://blast.ncbi.nlm.nih.gov/Blast.cgi#257467648%23257467648) | 92.1 | 100% | 482 |
| [XP_002346638.1](http://www.ncbi.nlm.nih.gov/entrez/query.fcgi?cmd=Retrieve&db=Protein&list_uids=239748356&dopt=GenPept&RID=T2G464PN01S&log$=prottop&blast_rank=85) | PREDICTED: hypothetical protein XP_002346638 [Homo sapiens] | [18.5](http://blast.ncbi.nlm.nih.gov/Blast.cgi#239748356%23239748356) | 18.5 | 60% | 482 |
| [XP_002342462.1](http://www.ncbi.nlm.nih.gov/entrez/query.fcgi?cmd=Retrieve&db=Protein&list_uids=239742256&dopt=GenPept&RID=T2G464PN01S&log$=prottop&blast_rank=86) | PREDICTED: hypothetical protein XP_002342462 [Homo sapiens] >ref|XP_002345781.1| PREDICTED: hypothetical protein XP_002345781 [Homo sapiens] | [18.5](http://blast.ncbi.nlm.nih.gov/Blast.cgi#239742256%23239742256) | 18.5 | 60% | 482 |
| [NP_001153517.1](http://www.ncbi.nlm.nih.gov/entrez/query.fcgi?cmd=Retrieve&db=Protein&list_uids=229892345&dopt=GenPept&RID=T2G464PN01S&log$=prottop&blast_rank=87) | GLI family zinc finger 1 isoform 2 [Homo sapiens] | [18.5](http://blast.ncbi.nlm.nih.gov/Blast.cgi#229892345%23229892345) | 18.5 | 50% | 482 |
| [NP_001139144.1](http://www.ncbi.nlm.nih.gov/entrez/query.fcgi?cmd=Retrieve&db=Protein&list_uids=224586849&dopt=GenPept&RID=T2G464PN01S&log$=prottop&blast_rank=88) | sorbin and SH3 domain containing 2 isoform 5 [Homo sapiens] | [18.5](http://blast.ncbi.nlm.nih.gov/Blast.cgi#224586849%23224586849) | 18.5 | 60% | 482 |
| [NP_001139142.1](http://www.ncbi.nlm.nih.gov/entrez/query.fcgi?cmd=Retrieve&db=Protein&list_uids=224586844&dopt=GenPept&RID=T2G464PN01S&log$=prottop&blast_rank=89) | sorbin and SH3 domain containing 2 isoform 3 [Homo sapiens] | [18.5](http://blast.ncbi.nlm.nih.gov/Blast.cgi#224586844%23224586844) | 18.5 | 60% | 482 |
| [NP_001161882.1](http://www.ncbi.nlm.nih.gov/entrez/query.fcgi?cmd=Retrieve&db=Protein&list_uids=270288804&dopt=GenPept&RID=T2G464PN01S&log$=prottop&blast_rank=90) | regulating synaptic membrane exocytosis 1 isoform 5 [Homo sapiens] | [18.5](http://blast.ncbi.nlm.nih.gov/Blast.cgi#270288804%23270288804) | 33.9 | 60% | 482 |
| [NP_056121.2](http://www.ncbi.nlm.nih.gov/entrez/query.fcgi?cmd=Retrieve&db=Protein&list_uids=260064009&dopt=GenPept&RID=T2G464PN01S&log$=prottop&blast_rank=91) | ubiquitin specific protease 24 [Homo sapiens] | [18.5](http://blast.ncbi.nlm.nih.gov/Blast.cgi#260064009%23260064009) | 18.5 | 50% | 482 |
| [NP_001139143.1](http://www.ncbi.nlm.nih.gov/entrez/query.fcgi?cmd=Retrieve&db=Protein&list_uids=224586846&dopt=GenPept&RID=T2G464PN01S&log$=prottop&blast_rank=92) | sorbin and SH3 domain containing 2 isoform 4 [Homo sapiens] | [18.5](http://blast.ncbi.nlm.nih.gov/Blast.cgi#224586846%23224586846) | 18.5 | 60% | 482 |
| [NP_073594.4](http://www.ncbi.nlm.nih.gov/entrez/query.fcgi?cmd=Retrieve&db=Protein&list_uids=169646281&dopt=GenPept&RID=T2G464PN01S&log$=prottop&blast_rank=93) | coiled-coil domain containing 14 [Homo sapiens] | [18.5](http://blast.ncbi.nlm.nih.gov/Blast.cgi#169646281%23169646281) | 18.5 | 60% | 482 |
| [XP_001714194.1](http://www.ncbi.nlm.nih.gov/entrez/query.fcgi?cmd=Retrieve&db=Protein&list_uids=169216502&dopt=GenPept&RID=T2G464PN01S&log$=prottop&blast_rank=94) | PREDICTED: similar to hCG1653500 [Homo sapiens] >ref|XP_001716602.1| PREDICTED: similar to hCG1653500 [Homo sapiens] | [18.5](http://blast.ncbi.nlm.nih.gov/Blast.cgi#169216502%23169216502) | 18.5 | 90% | 482 |
| [NP_689576.4](http://www.ncbi.nlm.nih.gov/entrez/query.fcgi?cmd=Retrieve&db=Protein&list_uids=168229256&dopt=GenPept&RID=T2G464PN01S&log$=prottop&blast_rank=95) | ankyrin repeat domain 41 [Homo sapiens] | [18.5](http://blast.ncbi.nlm.nih.gov/Blast.cgi#168229256%23168229256) | 18.5 | 60% | 482 |
| [NP_115921.2](http://www.ncbi.nlm.nih.gov/entrez/query.fcgi?cmd=Retrieve&db=Protein&list_uids=148806908&dopt=GenPept&RID=T2G464PN01S&log$=prottop&blast_rank=96) | fibronectin type III domain containing 1 [Homo sapiens] | [18.5](http://blast.ncbi.nlm.nih.gov/Blast.cgi#148806908%23148806908) | 33.9 | 70% | 482 |
| [NP_055998.1](http://www.ncbi.nlm.nih.gov/entrez/query.fcgi?cmd=Retrieve&db=Protein&list_uids=148727255&dopt=GenPept&RID=T2G464PN01S&log$=prottop&blast_rank=97) | microtubule associated serine/threonine kinase family member 4 isoform a [Homo sapiens] | [18.5](http://blast.ncbi.nlm.nih.gov/Blast.cgi#148727255%23148727255) | 92.1 | 100% | 482 |
| [NP_003594.3](http://www.ncbi.nlm.nih.gov/entrez/query.fcgi?cmd=Retrieve&db=Protein&list_uids=194733749&dopt=GenPept&RID=T2G464PN01S&log$=prottop&blast_rank=98) | sorbin and SH3 domain containing 2 isoform 1 [Homo sapiens] | [18.5](http://blast.ncbi.nlm.nih.gov/Blast.cgi#194733749%23194733749) | 18.5 | 60% | 482 |
| [NP_775831.2](http://www.ncbi.nlm.nih.gov/entrez/query.fcgi?cmd=Retrieve&db=Protein&list_uids=258547126&dopt=GenPept&RID=T2G464PN01S&log$=prottop&blast_rank=99) | regulatory factor X, 6 [Homo sapiens] | [18.5](http://blast.ncbi.nlm.nih.gov/Blast.cgi#258547126%23258547126) | 18.5 | 60% | 482 |
| [NP_060506.5](http://www.ncbi.nlm.nih.gov/entrez/query.fcgi?cmd=Retrieve&db=Protein&list_uids=118197272&dopt=GenPept&RID=T2G464PN01S&log$=prottop&blast_rank=100) | ATG2 autophagy related 2 homolog B [Homo sapiens] | [18.5](http://blast.ncbi.nlm.nih.gov/Blast.cgi#118197272%23118197272) | 18.5 | 60% | 482 |

| **Accession** | **Proteins with a match to GRSNKSG peptide** | **[Max score](http://blast.ncbi.nlm.nih.gov/Blast.cgi?CMD=Get&ALIGNMENTS=100&ALIGNMENT_VIEW=Pairwise&CDD_SEARCH_STATE=1&DATABASE_SORT=0&DESCRIPTIONS=100&ENTREZ_QUERY=txid9606 %5BORGN%5D&FIRST_QUERY_NUM=0&FORMAT_OBJECT=Alignment&FORMAT_PAGE_TARGET=&FORMAT_TYPE=HTML&GET_SEQUENCE=yes&I_THRESH=&MASK_CHAR=2&MASK_COLOR=1&NEW_DESIGN=on&NEW_VIEW=yes&NUM_OVERVIEW=100&OLD_BLAST=false&PAGE=Proteins&QUERY_INDEX=0&QUERY_NUMBER=0&RESULTS_PAGE_TARGET=&RID=T2GBRAZZ016&SHOW_LINKOUT=yes&SHOW_OVERVIEW=yes&STEP_NUMBER=&WORD_SIZE=2&DISPLAY_SORT=1&HSP_SORT=1" \l "sort_mark)** | **[Total score](http://blast.ncbi.nlm.nih.gov/Blast.cgi?CMD=Get&ALIGNMENTS=100&ALIGNMENT_VIEW=Pairwise&CDD_SEARCH_STATE=1&DATABASE_SORT=0&DESCRIPTIONS=100&ENTREZ_QUERY=txid9606 %5BORGN%5D&FIRST_QUERY_NUM=0&FORMAT_OBJECT=Alignment&FORMAT_PAGE_TARGET=&FORMAT_TYPE=HTML&GET_SEQUENCE=yes&I_THRESH=&MASK_CHAR=2&MASK_COLOR=1&NEW_DESIGN=on&NEW_VIEW=yes&NUM_OVERVIEW=100&OLD_BLAST=false&PAGE=Proteins&QUERY_INDEX=0&QUERY_NUMBER=0&RESULTS_PAGE_TARGET=&RID=T2GBRAZZ016&SHOW_LINKOUT=yes&SHOW_OVERVIEW=yes&STEP_NUMBER=&WORD_SIZE=2&DISPLAY_SORT=2&HSP_SORT=1" \l "sort_mark)** | **[Query coverage](http://blast.ncbi.nlm.nih.gov/Blast.cgi?CMD=Get&ALIGNMENTS=100&ALIGNMENT_VIEW=Pairwise&CDD_SEARCH_STATE=1&DATABASE_SORT=0&DESCRIPTIONS=100&ENTREZ_QUERY=txid9606 %5BORGN%5D&FIRST_QUERY_NUM=0&FORMAT_OBJECT=Alignment&FORMAT_PAGE_TARGET=&FORMAT_TYPE=HTML&GET_SEQUENCE=yes&I_THRESH=&MASK_CHAR=2&MASK_COLOR=1&NEW_DESIGN=on&NEW_VIEW=yes&NUM_OVERVIEW=100&OLD_BLAST=false&PAGE=Proteins&QUERY_INDEX=0&QUERY_NUMBER=0&RESULTS_PAGE_TARGET=&RID=T2GBRAZZ016&SHOW_LINKOUT=yes&SHOW_OVERVIEW=yes&STEP_NUMBER=&WORD_SIZE=2&DISPLAY_SORT=4&HSP_SORT=0" \l "sort_mark)** | **[E value](http://blast.ncbi.nlm.nih.gov/Blast.cgi?CMD=Get&ALIGNMENTS=100&ALIGNMENT_VIEW=Pairwise&CDD_SEARCH_STATE=1&DATABASE_SORT=0&DESCRIPTIONS=100&ENTREZ_QUERY=txid9606 %5BORGN%5D&FIRST_QUERY_NUM=0&FORMAT_OBJECT=Alignment&FORMAT_PAGE_TARGET=&FORMAT_TYPE=HTML&GET_SEQUENCE=yes&I_THRESH=&MASK_CHAR=2&MASK_COLOR=1&NEW_DESIGN=on&NEW_VIEW=yes&NUM_OVERVIEW=100&OLD_BLAST=false&PAGE=Proteins&QUERY_INDEX=0&QUERY_NUMBER=0&RESULTS_PAGE_TARGET=&RID=T2GBRAZZ016&SHOW_LINKOUT=yes&SHOW_OVERVIEW=yes&STEP_NUMBER=&WORD_SIZE=2&DISPLAY_SORT=0&HSP_SORT=0" \l "sort_mark)** |
| --- | --- | --- | --- | --- | --- |
| [XP_001722402.1](http://www.ncbi.nlm.nih.gov/entrez/query.fcgi?cmd=Retrieve&db=Protein&list_uids=169212850&dopt=GenPept&RID=T2GBRAZZ016&log$=prottop&blast_rank=1) | PREDICTED: hypothetical protein [Homo sapiens] >ref|XP_001720022.1| PREDICTED: hypothetical protein [Homo sapiens] >ref|XP_001720012.1| PREDICTED: hypothetical protein [Homo sapiens] | [18.9](http://blast.ncbi.nlm.nih.gov/Blast.cgi#169212850%23169212850) | 18.9 | 100% | 252 |
| [NP_060842.3](http://www.ncbi.nlm.nih.gov/entrez/query.fcgi?cmd=Retrieve&db=Protein&list_uids=55743124&dopt=GenPept&RID=T2GBRAZZ016&log$=prottop&blast_rank=2) | receptor-interacting factor 1 isoform 1 [Homo sapiens] | [18.0](http://blast.ncbi.nlm.nih.gov/Blast.cgi#55743124%2355743124) | 18.0 | 71% | 453 |
| [NP_898868.1](http://www.ncbi.nlm.nih.gov/entrez/query.fcgi?cmd=Retrieve&db=Protein&list_uids=34335262&dopt=GenPept&RID=T2GBRAZZ016&log$=prottop&blast_rank=3) | zinc finger, MYND-type containing 8 isoform a [Homo sapiens] | [18.0](http://blast.ncbi.nlm.nih.gov/Blast.cgi#34335262%2334335262) | 31.8 | 85% | 453 |
| [NP_036540.3](http://www.ncbi.nlm.nih.gov/entrez/query.fcgi?cmd=Retrieve&db=Protein&list_uids=34335266&dopt=GenPept&RID=T2GBRAZZ016&log$=prottop&blast_rank=4) | zinc finger, MYND-type containing 8 isoform b [Homo sapiens] | [18.0](http://blast.ncbi.nlm.nih.gov/Blast.cgi#34335266%2334335266) | 31.8 | 85% | 453 |
| [NP_898869.1](http://www.ncbi.nlm.nih.gov/entrez/query.fcgi?cmd=Retrieve&db=Protein&list_uids=34335264&dopt=GenPept&RID=T2GBRAZZ016&log$=prottop&blast_rank=5) | zinc finger, MYND-type containing 8 isoform c [Homo sapiens] | [18.0](http://blast.ncbi.nlm.nih.gov/Blast.cgi#34335264%2334335264) | 31.8 | 85% | 453 |
| [NP_006528.2](http://www.ncbi.nlm.nih.gov/entrez/query.fcgi?cmd=Retrieve&db=Protein&list_uids=55770886&dopt=GenPept&RID=T2GBRAZZ016&log$=prottop&blast_rank=6) | ubiquitin thiolesterase 3 [Homo sapiens] | [18.0](http://blast.ncbi.nlm.nih.gov/Blast.cgi#55770886%2355770886) | 18.0 | 71% | 453 |
| [NP_009293.1](http://www.ncbi.nlm.nih.gov/entrez/query.fcgi?cmd=Retrieve&db=Protein&list_uids=6382071&dopt=GenPept&RID=T2GBRAZZ016&log$=prottop&blast_rank=7) | diaphanous 2 isoform 12C [Homo sapiens] | [18.0](http://blast.ncbi.nlm.nih.gov/Blast.cgi#6382071%236382071) | 18.0 | 71% | 453 |
| [NP_001006946.1](http://www.ncbi.nlm.nih.gov/entrez/query.fcgi?cmd=Retrieve&db=Protein&list_uids=55743126&dopt=GenPept&RID=T2GBRAZZ016&log$=prottop&blast_rank=8) | receptor-interacting factor 1 isoform 2 [Homo sapiens] | [18.0](http://blast.ncbi.nlm.nih.gov/Blast.cgi#55743126%2355743126) | 18.0 | 71% | 453 |
| [NP_005740.1](http://www.ncbi.nlm.nih.gov/entrez/query.fcgi?cmd=Retrieve&db=Protein&list_uids=5032187&dopt=GenPept&RID=T2GBRAZZ016&log$=prottop&blast_rank=9) | transducer of ERBB2, 1 [Homo sapiens] | [18.0](http://blast.ncbi.nlm.nih.gov/Blast.cgi#5032187%235032187) | 18.0 | 71% | 453 |
| [NP_006733.1](http://www.ncbi.nlm.nih.gov/entrez/query.fcgi?cmd=Retrieve&db=Protein&list_uids=27901803&dopt=GenPept&RID=T2GBRAZZ016&log$=prottop&blast_rank=10) | protein serine kinase H1 [Homo sapiens] | [18.0](http://blast.ncbi.nlm.nih.gov/Blast.cgi#27901803%2327901803) | 18.0 | 71% | 453 |
| [NP_006720.1](http://www.ncbi.nlm.nih.gov/entrez/query.fcgi?cmd=Retrieve&db=Protein&list_uids=5803003&dopt=GenPept&RID=T2GBRAZZ016&log$=prottop&blast_rank=11) | diaphanous 2 isoform 156 [Homo sapiens] | [18.0](http://blast.ncbi.nlm.nih.gov/Blast.cgi#5803003%235803003) | 18.0 | 71% | 453 |
| [NP_001139137.1](http://www.ncbi.nlm.nih.gov/entrez/query.fcgi?cmd=Retrieve&db=Protein&list_uids=224586807&dopt=GenPept&RID=T2GBRAZZ016&log$=prottop&blast_rank=12) | zinc finger protein 181 isoform 2 [Homo sapiens] | [17.2](http://blast.ncbi.nlm.nih.gov/Blast.cgi#224586807%23224586807) | 17.2 | 71% | 816 |
| [NP_001127853.1](http://www.ncbi.nlm.nih.gov/entrez/query.fcgi?cmd=Retrieve&db=Protein&list_uids=197304777&dopt=GenPept&RID=T2GBRAZZ016&log$=prottop&blast_rank=13) | TBC1 domain family, member 5 isoform a [Homo sapiens] | [17.2](http://blast.ncbi.nlm.nih.gov/Blast.cgi#197304777%23197304777) | 17.2 | 71% | 816 |
| [NP_001001668.3](http://www.ncbi.nlm.nih.gov/entrez/query.fcgi?cmd=Retrieve&db=Protein&list_uids=154091003&dopt=GenPept&RID=T2GBRAZZ016&log$=prottop&blast_rank=14) | zinc finger protein 470 [Homo sapiens] | [17.2](http://blast.ncbi.nlm.nih.gov/Blast.cgi#154091003%23154091003) | 17.2 | 71% | 816 |
| [NP_443175.2](http://www.ncbi.nlm.nih.gov/entrez/query.fcgi?cmd=Retrieve&db=Protein&list_uids=209862821&dopt=GenPept&RID=T2GBRAZZ016&log$=prottop&blast_rank=15) | hypothetical protein LOC115572 [Homo sapiens] | [17.2](http://blast.ncbi.nlm.nih.gov/Blast.cgi#209862821%23209862821) | 17.2 | 71% | 816 |
| [NP_001139659.1](http://www.ncbi.nlm.nih.gov/entrez/query.fcgi?cmd=Retrieve&db=Protein&list_uids=226053185&dopt=GenPept&RID=T2GBRAZZ016&log$=prottop&blast_rank=16) | paternally expressed 3 isoform 3 [Homo sapiens] | [17.2](http://blast.ncbi.nlm.nih.gov/Blast.cgi#226053185%23226053185) | 17.2 | 85% | 816 |
| [NP_001073867.1](http://www.ncbi.nlm.nih.gov/entrez/query.fcgi?cmd=Retrieve&db=Protein&list_uids=122937211&dopt=GenPept&RID=T2GBRAZZ016&log$=prottop&blast_rank=17) | KIAA0368 protein [Homo sapiens] | [17.2](http://blast.ncbi.nlm.nih.gov/Blast.cgi#122937211%23122937211) | 17.2 | 71% | 816 |
| [NP_006455.2](http://www.ncbi.nlm.nih.gov/entrez/query.fcgi?cmd=Retrieve&db=Protein&list_uids=42518068&dopt=GenPept&RID=T2GBRAZZ016&log$=prottop&blast_rank=18) | trans-golgi network protein 2 [Homo sapiens] | [17.2](http://blast.ncbi.nlm.nih.gov/Blast.cgi#42518068%2342518068) | 78.3 | 71% | 816 |
| [NP_066267.2](http://www.ncbi.nlm.nih.gov/entrez/query.fcgi?cmd=Retrieve&db=Protein&list_uids=32967601&dopt=GenPept&RID=T2GBRAZZ016&log$=prottop&blast_rank=19) | ankyrin 3 isoform 1 [Homo sapiens] | [17.2](http://blast.ncbi.nlm.nih.gov/Blast.cgi#32967601%2332967601) | 28.4 | 71% | 816 |
| [NP_001139657.1](http://www.ncbi.nlm.nih.gov/entrez/query.fcgi?cmd=Retrieve&db=Protein&list_uids=226053148&dopt=GenPept&RID=T2GBRAZZ016&log$=prottop&blast_rank=20) | paternally expressed 3 isoform 2 [Homo sapiens] | [17.2](http://blast.ncbi.nlm.nih.gov/Blast.cgi#226053148%23226053148) | 17.2 | 85% | 816 |
| [NP_001025168.2](http://www.ncbi.nlm.nih.gov/entrez/query.fcgi?cmd=Retrieve&db=Protein&list_uids=224586802&dopt=GenPept&RID=T2GBRAZZ016&log$=prottop&blast_rank=21) | zinc finger protein 181 isoform 1 [Homo sapiens] | [17.2](http://blast.ncbi.nlm.nih.gov/Blast.cgi#224586802%23224586802) | 17.2 | 71% | 816 |
| [NP_001018082.1](http://www.ncbi.nlm.nih.gov/entrez/query.fcgi?cmd=Retrieve&db=Protein&list_uids=65786661&dopt=GenPept&RID=T2GBRAZZ016&log$=prottop&blast_rank=22) | BTB (POZ) domain containing 11 isoform a [Homo sapiens] | [17.2](http://blast.ncbi.nlm.nih.gov/Blast.cgi#65786661%2365786661) | 17.2 | 85% | 816 |
| [NP_006201.1](http://www.ncbi.nlm.nih.gov/entrez/query.fcgi?cmd=Retrieve&db=Protein&list_uids=33354285&dopt=GenPept&RID=T2GBRAZZ016&log$=prottop&blast_rank=23) | paternally expressed 3 isoform 1 [Homo sapiens] >ref|NP_001139656.1| paternally expressed 3 isoform 1 [Homo sapiens] >ref|NP_001139658.1| paternally expressed 3 isoform 1 [Homo sapiens] | [17.2](http://blast.ncbi.nlm.nih.gov/Blast.cgi#33354285%2333354285) | 17.2 | 85% | 816 |
| [NP_065114.3](http://www.ncbi.nlm.nih.gov/entrez/query.fcgi?cmd=Retrieve&db=Protein&list_uids=169808399&dopt=GenPept&RID=T2GBRAZZ016&log$=prottop&blast_rank=24) | prenyl diphosphate synthase, subunit 2 [Homo sapiens] | [17.2](http://blast.ncbi.nlm.nih.gov/Blast.cgi#169808399%23169808399) | 17.2 | 85% | 816 |
| [NP_060913.2](http://www.ncbi.nlm.nih.gov/entrez/query.fcgi?cmd=Retrieve&db=Protein&list_uids=59850649&dopt=GenPept&RID=T2GBRAZZ016&log$=prottop&blast_rank=25) | zinc finger protein 302 [Homo sapiens] >ref|NP_001012320.1| zinc finger protein 302 [Homo sapiens] | [17.2](http://blast.ncbi.nlm.nih.gov/Blast.cgi#59850649%2359850649) | 17.2 | 71% | 816 |
| [NP_055312.2](http://www.ncbi.nlm.nih.gov/entrez/query.fcgi?cmd=Retrieve&db=Protein&list_uids=21626468&dopt=GenPept&RID=T2GBRAZZ016&log$=prottop&blast_rank=26) | zinc finger protein 638 [Homo sapiens] >ref|NP_001014972.1| zinc finger protein 638 [Homo sapiens] | [17.2](http://blast.ncbi.nlm.nih.gov/Blast.cgi#21626468%2321626468) | 17.2 | 85% | 816 |
| [NP_055559.1](http://www.ncbi.nlm.nih.gov/entrez/query.fcgi?cmd=Retrieve&db=Protein&list_uids=7661998&dopt=GenPept&RID=T2GBRAZZ016&log$=prottop&blast_rank=27) | TBC1 domain family, member 5 isoform b [Homo sapiens] >ref|NP_001127852.1| TBC1 domain family, member 5 isoform b [Homo sapiens] | [17.2](http://blast.ncbi.nlm.nih.gov/Blast.cgi#7661998%237661998) | 17.2 | 71% | 816 |
| [NP_004353.1](http://www.ncbi.nlm.nih.gov/entrez/query.fcgi?cmd=Retrieve&db=Protein&list_uids=4758004&dopt=GenPept&RID=T2GBRAZZ016&log$=prottop&blast_rank=28) | calmegin precursor [Homo sapiens] >ref|NP_001124147.1| calmegin precursor [Homo sapiens] | [17.2](http://blast.ncbi.nlm.nih.gov/Blast.cgi#4758004%234758004) | 17.2 | 71% | 816 |
| [NP_064610.1](http://www.ncbi.nlm.nih.gov/entrez/query.fcgi?cmd=Retrieve&db=Protein&list_uids=55742730&dopt=GenPept&RID=T2GBRAZZ016&log$=prottop&blast_rank=29) | storkhead box 2 [Homo sapiens] | [16.8](http://blast.ncbi.nlm.nih.gov/Blast.cgi#55742730%2355742730) | 32.2 | 85% | 1095 |
| [NP_057547.5](http://www.ncbi.nlm.nih.gov/entrez/query.fcgi?cmd=Retrieve&db=Protein&list_uids=158261990&dopt=GenPept&RID=T2GBRAZZ016&log$=prottop&blast_rank=30) | CXXC finger 5 [Homo sapiens] | [16.8](http://blast.ncbi.nlm.nih.gov/Blast.cgi#158261990%23158261990) | 16.8 | 85% | 1095 |
| [NP_006396.2](http://www.ncbi.nlm.nih.gov/entrez/query.fcgi?cmd=Retrieve&db=Protein&list_uids=21361315&dopt=GenPept&RID=T2GBRAZZ016&log$=prottop&blast_rank=31) | transmembrane 9 superfamily member 1 isoform a [Homo sapiens] | [16.8](http://blast.ncbi.nlm.nih.gov/Blast.cgi#21361315%2321361315) | 16.8 | 85% | 1095 |
| [NP_001156386.1](http://www.ncbi.nlm.nih.gov/entrez/query.fcgi?cmd=Retrieve&db=Protein&list_uids=242332545&dopt=GenPept&RID=T2GBRAZZ016&log$=prottop&blast_rank=32) | coiled-coil domain containing 121-like [Homo sapiens] | [16.3](http://blast.ncbi.nlm.nih.gov/Blast.cgi#242332545%23242332545) | 16.3 | 85% | 1469 |
| [XP_001717293.1](http://www.ncbi.nlm.nih.gov/entrez/query.fcgi?cmd=Retrieve&db=Protein&list_uids=169202578&dopt=GenPept&RID=T2GBRAZZ016&log$=prottop&blast_rank=33) | PREDICTED: hypothetical protein [Homo sapiens] >ref|XP_001717790.1| PREDICTED: hypothetical protein [Homo sapiens] | [16.3](http://blast.ncbi.nlm.nih.gov/Blast.cgi#169202578%23169202578) | 16.3 | 100% | 1469 |
| [NP_874369.1](http://www.ncbi.nlm.nih.gov/entrez/query.fcgi?cmd=Retrieve&db=Protein&list_uids=36287069&dopt=GenPept&RID=T2GBRAZZ016&log$=prottop&blast_rank=34) | K(lysine) acetyltransferase 5 isoform 1 [Homo sapiens] | [16.3](http://blast.ncbi.nlm.nih.gov/Blast.cgi#36287069%2336287069) | 26.7 | 85% | 1469 |
| [NP_004662.2](http://www.ncbi.nlm.nih.gov/entrez/query.fcgi?cmd=Retrieve&db=Protein&list_uids=56699458&dopt=GenPept&RID=T2GBRAZZ016&log$=prottop&blast_rank=35) | protein inhibitor of activated STAT X isoform beta [Homo sapiens] | [16.3](http://blast.ncbi.nlm.nih.gov/Blast.cgi#56699458%2356699458) | 16.3 | 100% | 1469 |
| [NP_001073918.1](http://www.ncbi.nlm.nih.gov/entrez/query.fcgi?cmd=Retrieve&db=Protein&list_uids=122937299&dopt=GenPept&RID=T2GBRAZZ016&log$=prottop&blast_rank=36) | DNA replication helicase 2 homolog [Homo sapiens] | [16.3](http://blast.ncbi.nlm.nih.gov/Blast.cgi#122937299%23122937299) | 16.3 | 85% | 1469 |
| [NP_981958.1](http://www.ncbi.nlm.nih.gov/entrez/query.fcgi?cmd=Retrieve&db=Protein&list_uids=44662832&dopt=GenPept&RID=T2GBRAZZ016&log$=prottop&blast_rank=37) | S-phase 2 protein isoform 2 [Homo sapiens] | [16.3](http://blast.ncbi.nlm.nih.gov/Blast.cgi#44662832%2344662832) | 16.3 | 85% | 1469 |
| [NP_001107566.1](http://www.ncbi.nlm.nih.gov/entrez/query.fcgi?cmd=Retrieve&db=Protein&list_uids=166235901&dopt=GenPept&RID=T2GBRAZZ016&log$=prottop&blast_rank=38) | B-cell linker isoform 2 [Homo sapiens] | [16.3](http://blast.ncbi.nlm.nih.gov/Blast.cgi#166235901%23166235901) | 16.3 | 85% | 1469 |
| [NP_775298.1](http://www.ncbi.nlm.nih.gov/entrez/query.fcgi?cmd=Retrieve&db=Protein&list_uids=27532984&dopt=GenPept&RID=T2GBRAZZ016&log$=prottop&blast_rank=39) | protein inhibitor of activated STAT X isoform alpha [Homo sapiens] | [16.3](http://blast.ncbi.nlm.nih.gov/Blast.cgi#27532984%2327532984) | 16.3 | 100% | 1469 |
| [NP_937770.1](http://www.ncbi.nlm.nih.gov/entrez/query.fcgi?cmd=Retrieve&db=Protein&list_uids=62988334&dopt=GenPept&RID=T2GBRAZZ016&log$=prottop&blast_rank=40) | cation channel, sperm associated 4 [Homo sapiens] | [16.3](http://blast.ncbi.nlm.nih.gov/Blast.cgi#62988334%2362988334) | 29.3 | 85% | 1469 |
| [NP_037446.1](http://www.ncbi.nlm.nih.gov/entrez/query.fcgi?cmd=Retrieve&db=Protein&list_uids=7019535&dopt=GenPept&RID=T2GBRAZZ016&log$=prottop&blast_rank=41) | B-cell linker isoform 1 [Homo sapiens] | [16.3](http://blast.ncbi.nlm.nih.gov/Blast.cgi#7019535%237019535) | 16.3 | 85% | 1469 |
| [NP_699202.1](http://www.ncbi.nlm.nih.gov/entrez/query.fcgi?cmd=Retrieve&db=Protein&list_uids=24025688&dopt=GenPept&RID=T2GBRAZZ016&log$=prottop&blast_rank=42) | ligand of numb-protein X 2 [Homo sapiens] | [16.3](http://blast.ncbi.nlm.nih.gov/Blast.cgi#24025688%2324025688) | 16.3 | 100% | 1469 |
| [NP_874368.1](http://www.ncbi.nlm.nih.gov/entrez/query.fcgi?cmd=Retrieve&db=Protein&list_uids=36287060&dopt=GenPept&RID=T2GBRAZZ016&log$=prottop&blast_rank=43) | K(lysine) acetyltransferase 5 isoform 3 [Homo sapiens] | [16.3](http://blast.ncbi.nlm.nih.gov/Blast.cgi#36287060%2336287060) | 26.7 | 85% | 1469 |
| [NP_006090.2](http://www.ncbi.nlm.nih.gov/entrez/query.fcgi?cmd=Retrieve&db=Protein&list_uids=115298686&dopt=GenPept&RID=T2GBRAZZ016&log$=prottop&blast_rank=44) | protein inhibitor of activated STAT, 3 [Homo sapiens] | [16.3](http://blast.ncbi.nlm.nih.gov/Blast.cgi#115298686%23115298686) | 16.3 | 100% | 1469 |
| [NP_006379.2](http://www.ncbi.nlm.nih.gov/entrez/query.fcgi?cmd=Retrieve&db=Protein&list_uids=36287049&dopt=GenPept&RID=T2GBRAZZ016&log$=prottop&blast_rank=45) | K(lysine) acetyltransferase 5 isoform 2 [Homo sapiens] | [16.3](http://blast.ncbi.nlm.nih.gov/Blast.cgi#36287049%2336287049) | 26.7 | 85% | 1469 |
| [NP_001126.3](http://www.ncbi.nlm.nih.gov/entrez/query.fcgi?cmd=Retrieve&db=Protein&list_uids=256017259&dopt=GenPept&RID=T2GBRAZZ016&log$=prottop&blast_rank=46) | aggrecan isoform 1 precursor [Homo sapiens] | [15.9](http://blast.ncbi.nlm.nih.gov/Blast.cgi#256017259%23256017259) | 15.9 | 85% | 1971 |
| [NP_037359.3](http://www.ncbi.nlm.nih.gov/entrez/query.fcgi?cmd=Retrieve&db=Protein&list_uids=256017257&dopt=GenPept&RID=T2GBRAZZ016&log$=prottop&blast_rank=47) | aggrecan isoform 2 precursor [Homo sapiens] | [15.9](http://blast.ncbi.nlm.nih.gov/Blast.cgi#256017257%23256017257) | 15.9 | 85% | 1971 |
| [XP_002345647.1](http://www.ncbi.nlm.nih.gov/entrez/query.fcgi?cmd=Retrieve&db=Protein&list_uids=239753420&dopt=GenPept&RID=T2GBRAZZ016&log$=prottop&blast_rank=48) | PREDICTED: hypothetical protein XP_002345647 [Homo sapiens] | [15.9](http://blast.ncbi.nlm.nih.gov/Blast.cgi#239753420%23239753420) | 15.9 | 85% | 1971 |
| [XP_002343034.1](http://www.ncbi.nlm.nih.gov/entrez/query.fcgi?cmd=Retrieve&db=Protein&list_uids=239744106&dopt=GenPept&RID=T2GBRAZZ016&log$=prottop&blast_rank=49) | PREDICTED: hypothetical protein XP_002343034 [Homo sapiens] >ref|XP_002347178.1| PREDICTED: hypothetical protein [Homo sapiens] >ref|XP_002344527.1| PREDICTED: hypothetical protein XP_002344527 [Homo sapiens] | [15.9](http://blast.ncbi.nlm.nih.gov/Blast.cgi#239744106%23239744106) | 15.9 | 85% | 1971 |
| [NP_001130027.1](http://www.ncbi.nlm.nih.gov/entrez/query.fcgi?cmd=Retrieve&db=Protein&list_uids=211971038&dopt=GenPept&RID=T2GBRAZZ016&log$=prottop&blast_rank=50) | tousled-like kinase 1 isoform 3 [Homo sapiens] | [15.9](http://blast.ncbi.nlm.nih.gov/Blast.cgi#211971038%23211971038) | 15.9 | 85% | 1971 |
| [NP_001130026.1](http://www.ncbi.nlm.nih.gov/entrez/query.fcgi?cmd=Retrieve&db=Protein&list_uids=211971036&dopt=GenPept&RID=T2GBRAZZ016&log$=prottop&blast_rank=51) | tousled-like kinase 1 isoform 2 [Homo sapiens] | [15.9](http://blast.ncbi.nlm.nih.gov/Blast.cgi#211971036%23211971036) | 15.9 | 85% | 1971 |
| [NP_001106197.1](http://www.ncbi.nlm.nih.gov/entrez/query.fcgi?cmd=Retrieve&db=Protein&list_uids=163644261&dopt=GenPept&RID=T2GBRAZZ016&log$=prottop&blast_rank=52) | hypothetical protein LOC283638 isoform 1 [Homo sapiens] | [15.9](http://blast.ncbi.nlm.nih.gov/Blast.cgi#163644261%23163644261) | 15.9 | 85% | 1971 |
| [NP_689558.4](http://www.ncbi.nlm.nih.gov/entrez/query.fcgi?cmd=Retrieve&db=Protein&list_uids=124517699&dopt=GenPept&RID=T2GBRAZZ016&log$=prottop&blast_rank=53) | ankyrin repeat domain 13B [Homo sapiens] | [15.9](http://blast.ncbi.nlm.nih.gov/Blast.cgi#124517699%23124517699) | 15.9 | 100% | 1971 |
| [NP_056297.1](http://www.ncbi.nlm.nih.gov/entrez/query.fcgi?cmd=Retrieve&db=Protein&list_uids=170650643&dopt=GenPept&RID=T2GBRAZZ016&log$=prottop&blast_rank=54) | solute carrier family 22, member 23 isoform a [Homo sapiens] | [15.9](http://blast.ncbi.nlm.nih.gov/Blast.cgi#170650643%23170650643) | 15.9 | 85% | 1971 |
| [NP_001092286.2](http://www.ncbi.nlm.nih.gov/entrez/query.fcgi?cmd=Retrieve&db=Protein&list_uids=169790825&dopt=GenPept&RID=T2GBRAZZ016&log$=prottop&blast_rank=55) | odz, odd Oz/ten-m homolog 4 [Homo sapiens] | [15.9](http://blast.ncbi.nlm.nih.gov/Blast.cgi#169790825%23169790825) | 15.9 | 100% | 1971 |
| [NP_997263.2](http://www.ncbi.nlm.nih.gov/entrez/query.fcgi?cmd=Retrieve&db=Protein&list_uids=155029552&dopt=GenPept&RID=T2GBRAZZ016&log$=prottop&blast_rank=56) | hypothetical protein LOC388115 [Homo sapiens] | [15.9](http://blast.ncbi.nlm.nih.gov/Blast.cgi#155029552%23155029552) | 15.9 | 85% | 1971 |
| [NP_055820.1](http://www.ncbi.nlm.nih.gov/entrez/query.fcgi?cmd=Retrieve&db=Protein&list_uids=61742808&dopt=GenPept&RID=T2GBRAZZ016&log$=prottop&blast_rank=57) | hypothetical protein LOC283638 isoform 2 [Homo sapiens] | [15.9](http://blast.ncbi.nlm.nih.gov/Blast.cgi#61742808%2361742808) | 15.9 | 85% | 1971 |
| [NP_064612.2](http://www.ncbi.nlm.nih.gov/entrez/query.fcgi?cmd=Retrieve&db=Protein&list_uids=147905620&dopt=GenPept&RID=T2GBRAZZ016&log$=prottop&blast_rank=58) | PR domain containing 9 [Homo sapiens] | [15.9](http://blast.ncbi.nlm.nih.gov/Blast.cgi#147905620%23147905620) | 31.8 | 85% | 1971 |
| [NP_149107.4](http://www.ncbi.nlm.nih.gov/entrez/query.fcgi?cmd=Retrieve&db=Protein&list_uids=222136641&dopt=GenPept&RID=T2GBRAZZ016&log$=prottop&blast_rank=59) | NIMA-related kinase 9 [Homo sapiens] | [15.9](http://blast.ncbi.nlm.nih.gov/Blast.cgi#222136641%23222136641) | 15.9 | 85% | 1971 |
| [NP_004809.2](http://www.ncbi.nlm.nih.gov/entrez/query.fcgi?cmd=Retrieve&db=Protein&list_uids=41327771&dopt=GenPept&RID=T2GBRAZZ016&log$=prottop&blast_rank=60) | DEAD (Asp-Glu-Ala-Asp) box polypeptide 23 [Homo sapiens] | [15.9](http://blast.ncbi.nlm.nih.gov/Blast.cgi#41327771%2341327771) | 15.9 | 100% | 1971 |
| [NP_001104251.1](http://www.ncbi.nlm.nih.gov/entrez/query.fcgi?cmd=Retrieve&db=Protein&list_uids=160707905&dopt=GenPept&RID=T2GBRAZZ016&log$=prottop&blast_rank=61) | similar to solute carrier family 35, member E2 [Homo sapiens] | [15.9](http://blast.ncbi.nlm.nih.gov/Blast.cgi#160707905%23160707905) | 15.9 | 85% | 1971 |
| [NP_054889.2](http://www.ncbi.nlm.nih.gov/entrez/query.fcgi?cmd=Retrieve&db=Protein&list_uids=56549685&dopt=GenPept&RID=T2GBRAZZ016&log$=prottop&blast_rank=62) | GTP-binding protein 8 isoform 1 [Homo sapiens] | [15.9](http://blast.ncbi.nlm.nih.gov/Blast.cgi#56549685%2356549685) | 15.9 | 85% | 1971 |
| [NP_036419.3](http://www.ncbi.nlm.nih.gov/entrez/query.fcgi?cmd=Retrieve&db=Protein&list_uids=187608516&dopt=GenPept&RID=T2GBRAZZ016&log$=prottop&blast_rank=63) | centaurin, beta 2 [Homo sapiens] | [15.9](http://blast.ncbi.nlm.nih.gov/Blast.cgi#187608516%23187608516) | 15.9 | 85% | 1971 |
| [NP_060163.2](http://www.ncbi.nlm.nih.gov/entrez/query.fcgi?cmd=Retrieve&db=Protein&list_uids=22095355&dopt=GenPept&RID=T2GBRAZZ016&log$=prottop&blast_rank=64) | basic, immunoglobulin-like variable motif containing isoform a [Homo sapiens] | [15.9](http://blast.ncbi.nlm.nih.gov/Blast.cgi#22095355%2322095355) | 15.9 | 85% | 1971 |
| [NP_003218.2](http://www.ncbi.nlm.nih.gov/entrez/query.fcgi?cmd=Retrieve&db=Protein&list_uids=33589848&dopt=GenPept&RID=T2GBRAZZ016&log$=prottop&blast_rank=65) | transferrin receptor 2 [Homo sapiens] | [15.9](http://blast.ncbi.nlm.nih.gov/Blast.cgi#33589848%2333589848) | 15.9 | 85% | 1971 |
| [NP_036422.3](http://www.ncbi.nlm.nih.gov/entrez/query.fcgi?cmd=Retrieve&db=Protein&list_uids=33636698&dopt=GenPept&RID=T2GBRAZZ016&log$=prottop&blast_rank=66) | tousled-like kinase 1 isoform 1 [Homo sapiens] | [15.9](http://blast.ncbi.nlm.nih.gov/Blast.cgi#33636698%2333636698) | 15.9 | 85% | 1971 |
| [NP_008984.1](http://www.ncbi.nlm.nih.gov/entrez/query.fcgi?cmd=Retrieve&db=Protein&list_uids=5901910&dopt=GenPept&RID=T2GBRAZZ016&log$=prottop&blast_rank=67) | CD160 antigen precursor [Homo sapiens] | [15.9](http://blast.ncbi.nlm.nih.gov/Blast.cgi#5901910%235901910) | 15.9 | 85% | 1971 |
| [NP_006796.1](http://www.ncbi.nlm.nih.gov/entrez/query.fcgi?cmd=Retrieve&db=Protein&list_uids=5803036&dopt=GenPept&RID=T2GBRAZZ016&log$=prottop&blast_rank=68) | heterogeneous nuclear ribonucleoprotein A0 [Homo sapiens] | [15.9](http://blast.ncbi.nlm.nih.gov/Blast.cgi#5803036%235803036) | 15.9 | 100% | 1971 |
| [NP_056470.1](http://www.ncbi.nlm.nih.gov/entrez/query.fcgi?cmd=Retrieve&db=Protein&list_uids=23308733&dopt=GenPept&RID=T2GBRAZZ016&log$=prottop&blast_rank=69) | zinc finger protein 337 [Homo sapiens] | [15.9](http://blast.ncbi.nlm.nih.gov/Blast.cgi#23308733%2323308733) | 31.8 | 85% | 1971 |
| [NP_003027.1](http://www.ncbi.nlm.nih.gov/entrez/query.fcgi?cmd=Retrieve&db=Protein&list_uids=4506967&dopt=GenPept&RID=T2GBRAZZ016&log$=prottop&blast_rank=70) | v-ski sarcoma viral oncogene homolog [Homo sapiens] | [15.9](http://blast.ncbi.nlm.nih.gov/Blast.cgi#4506967%234506967) | 15.9 | 85% | 1971 |
| [NP_000831.2](http://www.ncbi.nlm.nih.gov/entrez/query.fcgi?cmd=Retrieve&db=Protein&list_uids=46358417&dopt=GenPept&RID=T2GBRAZZ016&log$=prottop&blast_rank=71) | glutamate receptor, metabotropic 3 precursor [Homo sapiens] | [15.9](http://blast.ncbi.nlm.nih.gov/Blast.cgi#46358417%2346358417) | 15.9 | 85% | 1971 |
| [NP_001161881.1](http://www.ncbi.nlm.nih.gov/entrez/query.fcgi?cmd=Retrieve&db=Protein&list_uids=270288802&dopt=GenPept&RID=T2GBRAZZ016&log$=prottop&blast_rank=72) | regulating synaptic membrane exocytosis 1 isoform 4 [Homo sapiens] | [15.5](http://blast.ncbi.nlm.nih.gov/Blast.cgi#270288802%23270288802) | 15.5 | 71% | 2644 |
| [NP_001161880.1](http://www.ncbi.nlm.nih.gov/entrez/query.fcgi?cmd=Retrieve&db=Protein&list_uids=270288800&dopt=GenPept&RID=T2GBRAZZ016&log$=prottop&blast_rank=73) | regulating synaptic membrane exocytosis 1 isoform 3 [Homo sapiens] | [15.5](http://blast.ncbi.nlm.nih.gov/Blast.cgi#270288800%23270288800) | 15.5 | 71% | 2644 |
| [NP_001161879.1](http://www.ncbi.nlm.nih.gov/entrez/query.fcgi?cmd=Retrieve&db=Protein&list_uids=270288798&dopt=GenPept&RID=T2GBRAZZ016&log$=prottop&blast_rank=74) | regulating synaptic membrane exocytosis 1 isoform 2 [Homo sapiens] | [15.5](http://blast.ncbi.nlm.nih.gov/Blast.cgi#270288798%23270288798) | 15.5 | 71% | 2644 |
| [NP_001019628.3](http://www.ncbi.nlm.nih.gov/entrez/query.fcgi?cmd=Retrieve&db=Protein&list_uids=262118265&dopt=GenPept&RID=T2GBRAZZ016&log$=prottop&blast_rank=75) | RANBP2-like and GRIP domain containing 1 [Homo sapiens] | [15.5](http://blast.ncbi.nlm.nih.gov/Blast.cgi#262118265%23262118265) | 30.1 | 85% | 2644 |
| [XP_002345200.1](http://www.ncbi.nlm.nih.gov/entrez/query.fcgi?cmd=Retrieve&db=Protein&list_uids=239757169&dopt=GenPept&RID=T2GBRAZZ016&log$=prottop&blast_rank=76) | PREDICTED: hypothetical protein [Homo sapiens] | [15.5](http://blast.ncbi.nlm.nih.gov/Blast.cgi#239757169%23239757169) | 15.5 | 57% | 2644 |
| [XP_002344782.1](http://www.ncbi.nlm.nih.gov/entrez/query.fcgi?cmd=Retrieve&db=Protein&list_uids=239756039&dopt=GenPept&RID=T2GBRAZZ016&log$=prottop&blast_rank=77) | PREDICTED: hypothetical protein LOC643677 [Homo sapiens] | [15.5](http://blast.ncbi.nlm.nih.gov/Blast.cgi#239756039%23239756039) | 15.5 | 57% | 2644 |
| [XP_002347965.1](http://www.ncbi.nlm.nih.gov/entrez/query.fcgi?cmd=Retrieve&db=Protein&list_uids=239751681&dopt=GenPept&RID=T2GBRAZZ016&log$=prottop&blast_rank=78) | PREDICTED: hypothetical protein XP_002347965 [Homo sapiens] | [15.5](http://blast.ncbi.nlm.nih.gov/Blast.cgi#239751681%23239751681) | 15.5 | 57% | 2644 |
| [XP_002343659.1](http://www.ncbi.nlm.nih.gov/entrez/query.fcgi?cmd=Retrieve&db=Protein&list_uids=239746185&dopt=GenPept&RID=T2GBRAZZ016&log$=prottop&blast_rank=79) | PREDICTED: hypothetical protein XP_002343659 [Homo sapiens] | [15.5](http://blast.ncbi.nlm.nih.gov/Blast.cgi#239746185%23239746185) | 15.5 | 57% | 2644 |
| [XP_002344126.1](http://www.ncbi.nlm.nih.gov/entrez/query.fcgi?cmd=Retrieve&db=Protein&list_uids=239744870&dopt=GenPept&RID=T2GBRAZZ016&log$=prottop&blast_rank=80) | PREDICTED: hypothetical protein LOC643677 [Homo sapiens] | [15.5](http://blast.ncbi.nlm.nih.gov/Blast.cgi#239744870%23239744870) | 15.5 | 57% | 2644 |
| [NP_001139669.1](http://www.ncbi.nlm.nih.gov/entrez/query.fcgi?cmd=Retrieve&db=Protein&list_uids=226246554&dopt=GenPept&RID=T2GBRAZZ016&log$=prottop&blast_rank=81) | hypothetical protein LOC643677 [Homo sapiens] | [15.5](http://blast.ncbi.nlm.nih.gov/Blast.cgi#226246554%23226246554) | 15.5 | 57% | 2644 |
| [NP_001161882.1](http://www.ncbi.nlm.nih.gov/entrez/query.fcgi?cmd=Retrieve&db=Protein&list_uids=270288804&dopt=GenPept&RID=T2GBRAZZ016&log$=prottop&blast_rank=82) | regulating synaptic membrane exocytosis 1 isoform 5 [Homo sapiens] | [15.5](http://blast.ncbi.nlm.nih.gov/Blast.cgi#270288804%23270288804) | 15.5 | 71% | 2644 |
| [NP_001137540.1](http://www.ncbi.nlm.nih.gov/entrez/query.fcgi?cmd=Retrieve&db=Protein&list_uids=221136788&dopt=GenPept&RID=T2GBRAZZ016&log$=prottop&blast_rank=83) | zinc finger protein 772 isoform 2 [Homo sapiens] | [15.5](http://blast.ncbi.nlm.nih.gov/Blast.cgi#221136788%23221136788) | 15.5 | 71% | 2644 |
| [NP_001122396.1](http://www.ncbi.nlm.nih.gov/entrez/query.fcgi?cmd=Retrieve&db=Protein&list_uids=193083145&dopt=GenPept&RID=T2GBRAZZ016&log$=prottop&blast_rank=84) | SH3 domain containing 19 isoform c [Homo sapiens] | [15.5](http://blast.ncbi.nlm.nih.gov/Blast.cgi#193083145%23193083145) | 15.5 | 57% | 2644 |
| [NP_001122395.1](http://www.ncbi.nlm.nih.gov/entrez/query.fcgi?cmd=Retrieve&db=Protein&list_uids=193083143&dopt=GenPept&RID=T2GBRAZZ016&log$=prottop&blast_rank=85) | SH3 domain containing 19 isoform b [Homo sapiens] | [15.5](http://blast.ncbi.nlm.nih.gov/Blast.cgi#193083143%23193083143) | 15.5 | 57% | 2644 |
| [NP_001009555.3](http://www.ncbi.nlm.nih.gov/entrez/query.fcgi?cmd=Retrieve&db=Protein&list_uids=193083141&dopt=GenPept&RID=T2GBRAZZ016&log$=prottop&blast_rank=86) | SH3 domain containing 19 isoform a [Homo sapiens] | [15.5](http://blast.ncbi.nlm.nih.gov/Blast.cgi#193083141%23193083141) | 15.5 | 57% | 2644 |
| [XP_001717499.1](http://www.ncbi.nlm.nih.gov/entrez/query.fcgi?cmd=Retrieve&db=Protein&list_uids=169163653&dopt=GenPept&RID=T2GBRAZZ016&log$=prottop&blast_rank=87) | PREDICTED: similar to tudor domain containing 1 [Homo sapiens] >ref|XP_001720041.1| PREDICTED: similar to tudor domain containing 1 [Homo sapiens] | [15.5](http://blast.ncbi.nlm.nih.gov/Blast.cgi#169163653%23169163653) | 15.5 | 71% | 2644 |
| [XP_001717396.1](http://www.ncbi.nlm.nih.gov/entrez/query.fcgi?cmd=Retrieve&db=Protein&list_uids=169163210&dopt=GenPept&RID=T2GBRAZZ016&log$=prottop&blast_rank=88) | PREDICTED: similar to tudor domain containing 1 [Homo sapiens] | [15.5](http://blast.ncbi.nlm.nih.gov/Blast.cgi#169163210%23169163210) | 15.5 | 71% | 2644 |
| [NP_001549.2](http://www.ncbi.nlm.nih.gov/entrez/query.fcgi?cmd=Retrieve&db=Protein&list_uids=222136575&dopt=GenPept&RID=T2GBRAZZ016&log$=prottop&blast_rank=89) | interleukin 10 receptor, alpha precursor [Homo sapiens] | [15.5](http://blast.ncbi.nlm.nih.gov/Blast.cgi#222136575%23222136575) | 15.5 | 57% | 2644 |
| [NP_001137160.1](http://www.ncbi.nlm.nih.gov/entrez/query.fcgi?cmd=Retrieve&db=Protein&list_uids=219521928&dopt=GenPept&RID=T2GBRAZZ016&log$=prottop&blast_rank=90) | DIS3 mitotic control homolog (S. cerevisiae)-like isoform 1 [Homo sapiens] | [15.5](http://blast.ncbi.nlm.nih.gov/Blast.cgi#219521928%23219521928) | 15.5 | 57% | 2644 |
| [NP_001032412.2](http://www.ncbi.nlm.nih.gov/entrez/query.fcgi?cmd=Retrieve&db=Protein&list_uids=156105693&dopt=GenPept&RID=T2GBRAZZ016&log$=prottop&blast_rank=91) | PPAR-alpha interacting complex protein 285 isoform 1 [Homo sapiens] | [15.5](http://blast.ncbi.nlm.nih.gov/Blast.cgi#156105693%23156105693) | 15.5 | 85% | 2644 |
| [NP_001093587.1](http://www.ncbi.nlm.nih.gov/entrez/query.fcgi?cmd=Retrieve&db=Protein&list_uids=154354983&dopt=GenPept&RID=T2GBRAZZ016&log$=prottop&blast_rank=92) | regulating synaptic membrane exocytosis 2 isoform a [Homo sapiens] | [15.5](http://blast.ncbi.nlm.nih.gov/Blast.cgi#154354983%23154354983) | 15.5 | 57% | 2644 |
| [NP_001092272.1](http://www.ncbi.nlm.nih.gov/entrez/query.fcgi?cmd=Retrieve&db=Protein&list_uids=149274621&dopt=GenPept&RID=T2GBRAZZ016&log$=prottop&blast_rank=93) | centrosomal protein 78kDa isoform a [Homo sapiens] | [15.5](http://blast.ncbi.nlm.nih.gov/Blast.cgi#149274621%23149274621) | 15.5 | 85% | 2644 |
| [NP_037450.2](http://www.ncbi.nlm.nih.gov/entrez/query.fcgi?cmd=Retrieve&db=Protein&list_uids=149192855&dopt=GenPept&RID=T2GBRAZZ016&log$=prottop&blast_rank=94) | HLA-B associated transcript 2-like [Homo sapiens] | [15.5](http://blast.ncbi.nlm.nih.gov/Blast.cgi#149192855%23149192855) | 27.2 | 100% | 2644 |
| [NP_001092142.1](http://www.ncbi.nlm.nih.gov/entrez/query.fcgi?cmd=Retrieve&db=Protein&list_uids=148886707&dopt=GenPept&RID=T2GBRAZZ016&log$=prottop&blast_rank=95) | hephaestin-like 1 precursor [Homo sapiens] | [15.5](http://blast.ncbi.nlm.nih.gov/Blast.cgi#148886707%23148886707) | 15.5 | 57% | 2644 |
| [NP_001091968.1](http://www.ncbi.nlm.nih.gov/entrez/query.fcgi?cmd=Retrieve&db=Protein&list_uids=148612875&dopt=GenPept&RID=T2GBRAZZ016&log$=prottop&blast_rank=96) | RUN and TBC1 domain containing 2 isoform 4 [Homo sapiens] | [15.5](http://blast.ncbi.nlm.nih.gov/Blast.cgi#148612875%23148612875) | 15.5 | 57% | 2644 |
| [NP_001091967.1](http://www.ncbi.nlm.nih.gov/entrez/query.fcgi?cmd=Retrieve&db=Protein&list_uids=148612840&dopt=GenPept&RID=T2GBRAZZ016&log$=prottop&blast_rank=97) | RUN and TBC1 domain containing 2 isoform 3 [Homo sapiens] | [15.5](http://blast.ncbi.nlm.nih.gov/Blast.cgi#148612840%23148612840) | 15.5 | 57% | 2644 |
| [NP_001137309.1](http://www.ncbi.nlm.nih.gov/entrez/query.fcgi?cmd=Retrieve&db=Protein&list_uids=219842348&dopt=GenPept&RID=T2GBRAZZ016&log$=prottop&blast_rank=98) | NADPH oxidase 4 isoform c [Homo sapiens] | [15.5](http://blast.ncbi.nlm.nih.gov/Blast.cgi#219842348%23219842348) | 15.5 | 57% | 2644 |
| [NP_689582.2](http://www.ncbi.nlm.nih.gov/entrez/query.fcgi?cmd=Retrieve&db=Protein&list_uids=166235171&dopt=GenPept&RID=T2GBRAZZ016&log$=prottop&blast_rank=99) | solute carrier family 44, member 3 isoform 2 [Homo sapiens] | [15.5](http://blast.ncbi.nlm.nih.gov/Blast.cgi#166235171%23166235171) | 15.5 | 57% | 2644 |
| [NP_005631.1](http://www.ncbi.nlm.nih.gov/entrez/query.fcgi?cmd=Retrieve&db=Protein&list_uids=148792970&dopt=GenPept&RID=T2GBRAZZ016&log$=prottop&blast_rank=100) | TAF4b RNA polymerase II, TATA box binding protein (TBP)-associated factor, 105kDa [Homo sapiens] | [15.5](http://blast.ncbi.nlm.nih.gov/Blast.cgi#148792970%23148792970) | 15.5 | 57% | 2644 |

| **Accession** | **Proteins with a match to SPTHP peptide** | **[Max score](http://blast.ncbi.nlm.nih.gov/Blast.cgi?CMD=Get&ALIGNMENTS=100&ALIGNMENT_VIEW=Pairwise&CDD_SEARCH_STATE=1&DATABASE_SORT=0&DESCRIPTIONS=100&ENTREZ_QUERY=txid9606 %5BORGN%5D&FIRST_QUERY_NUM=0&FORMAT_OBJECT=Alignment&FORMAT_PAGE_TARGET=&FORMAT_TYPE=HTML&GET_SEQUENCE=yes&I_THRESH=&MASK_CHAR=2&MASK_COLOR=1&NEW_DESIGN=on&NEW_VIEW=yes&NUM_OVERVIEW=100&OLD_BLAST=false&PAGE=Proteins&QUERY_INDEX=0&QUERY_NUMBER=0&RESULTS_PAGE_TARGET=&RID=T2GR2G3101S&SHOW_LINKOUT=yes&SHOW_OVERVIEW=yes&STEP_NUMBER=&WORD_SIZE=2&DISPLAY_SORT=1&HSP_SORT=1" \l "sort_mark)** | **[Total score](http://blast.ncbi.nlm.nih.gov/Blast.cgi?CMD=Get&ALIGNMENTS=100&ALIGNMENT_VIEW=Pairwise&CDD_SEARCH_STATE=1&DATABASE_SORT=0&DESCRIPTIONS=100&ENTREZ_QUERY=txid9606 %5BORGN%5D&FIRST_QUERY_NUM=0&FORMAT_OBJECT=Alignment&FORMAT_PAGE_TARGET=&FORMAT_TYPE=HTML&GET_SEQUENCE=yes&I_THRESH=&MASK_CHAR=2&MASK_COLOR=1&NEW_DESIGN=on&NEW_VIEW=yes&NUM_OVERVIEW=100&OLD_BLAST=false&PAGE=Proteins&QUERY_INDEX=0&QUERY_NUMBER=0&RESULTS_PAGE_TARGET=&RID=T2GR2G3101S&SHOW_LINKOUT=yes&SHOW_OVERVIEW=yes&STEP_NUMBER=&WORD_SIZE=2&DISPLAY_SORT=2&HSP_SORT=1" \l "sort_mark)** | **[Query coverage](http://blast.ncbi.nlm.nih.gov/Blast.cgi?CMD=Get&ALIGNMENTS=100&ALIGNMENT_VIEW=Pairwise&CDD_SEARCH_STATE=1&DATABASE_SORT=0&DESCRIPTIONS=100&ENTREZ_QUERY=txid9606 %5BORGN%5D&FIRST_QUERY_NUM=0&FORMAT_OBJECT=Alignment&FORMAT_PAGE_TARGET=&FORMAT_TYPE=HTML&GET_SEQUENCE=yes&I_THRESH=&MASK_CHAR=2&MASK_COLOR=1&NEW_DESIGN=on&NEW_VIEW=yes&NUM_OVERVIEW=100&OLD_BLAST=false&PAGE=Proteins&QUERY_INDEX=0&QUERY_NUMBER=0&RESULTS_PAGE_TARGET=&RID=T2GR2G3101S&SHOW_LINKOUT=yes&SHOW_OVERVIEW=yes&STEP_NUMBER=&WORD_SIZE=2&DISPLAY_SORT=4&HSP_SORT=0" \l "sort_mark)** | **[E value](http://blast.ncbi.nlm.nih.gov/Blast.cgi?CMD=Get&ALIGNMENTS=100&ALIGNMENT_VIEW=Pairwise&CDD_SEARCH_STATE=1&DATABASE_SORT=0&DESCRIPTIONS=100&ENTREZ_QUERY=txid9606 %5BORGN%5D&FIRST_QUERY_NUM=0&FORMAT_OBJECT=Alignment&FORMAT_PAGE_TARGET=&FORMAT_TYPE=HTML&GET_SEQUENCE=yes&I_THRESH=&MASK_CHAR=2&MASK_COLOR=1&NEW_DESIGN=on&NEW_VIEW=yes&NUM_OVERVIEW=100&OLD_BLAST=false&PAGE=Proteins&QUERY_INDEX=0&QUERY_NUMBER=0&RESULTS_PAGE_TARGET=&RID=T2GR2G3101S&SHOW_LINKOUT=yes&SHOW_OVERVIEW=yes&STEP_NUMBER=&WORD_SIZE=2&DISPLAY_SORT=0&HSP_SORT=0" \l "sort_mark)** |
| --- | --- | --- | --- | --- | --- |
| [XP_002346388.1](http://www.ncbi.nlm.nih.gov/entrez/query.fcgi?cmd=Retrieve&db=Protein&list_uids=239757944&dopt=GenPept&RID=T2GR2G3101S&log$=prottop&blast_rank=1) | PREDICTED: hypothetical protein [Homo sapiens] | [19.3](http://blast.ncbi.nlm.nih.gov/Blast.cgi#239757944%23239757944) | 19.3 | 100% | 134 |
| [XP_001715551.2](http://www.ncbi.nlm.nih.gov/entrez/query.fcgi?cmd=Retrieve&db=Protein&list_uids=239755199&dopt=GenPept&RID=T2GR2G3101S&log$=prottop&blast_rank=2) | PREDICTED: hypothetical protein [Homo sapiens] | [19.3](http://blast.ncbi.nlm.nih.gov/Blast.cgi#239755199%23239755199) | 52.8 | 100% | 134 |
| [XP_941653.2](http://www.ncbi.nlm.nih.gov/entrez/query.fcgi?cmd=Retrieve&db=Protein&list_uids=239749931&dopt=GenPept&RID=T2GR2G3101S&log$=prottop&blast_rank=3) | PREDICTED: FLJ43861 protein [Homo sapiens] | [19.3](http://blast.ncbi.nlm.nih.gov/Blast.cgi#239749931%23239749931) | 35.2 | 100% | 134 |
| [XP_002344404.1](http://www.ncbi.nlm.nih.gov/entrez/query.fcgi?cmd=Retrieve&db=Protein&list_uids=239508962&dopt=GenPept&RID=T2GR2G3101S&log$=prottop&blast_rank=4) | PREDICTED: hypothetical protein [Homo sapiens] >ref|XP_002342816.1| PREDICTED: hypothetical protein XP_002342816 [Homo sapiens] >ref|XP_002346062.1| PREDICTED: hypothetical protein XP_002346062 [Homo sapiens] | [19.3](http://blast.ncbi.nlm.nih.gov/Blast.cgi#239508962%23239508962) | 19.3 | 100% | 134 |
| [XP_001129558.1](http://www.ncbi.nlm.nih.gov/entrez/query.fcgi?cmd=Retrieve&db=Protein&list_uids=113415539&dopt=GenPept&RID=T2GR2G3101S&log$=prottop&blast_rank=5) | PREDICTED: hypothetical protein [Homo sapiens] >ref|XP_001131204.1| PREDICTED: hypothetical protein [Homo sapiens] >ref|XP_001717566.1| PREDICTED: hypothetical protein [Homo sapiens] | [19.3](http://blast.ncbi.nlm.nih.gov/Blast.cgi#113415539%23113415539) | 36.1 | 100% | 134 |
| [XP_001714285.1](http://www.ncbi.nlm.nih.gov/entrez/query.fcgi?cmd=Retrieve&db=Protein&list_uids=169162130&dopt=GenPept&RID=T2GR2G3101S&log$=prottop&blast_rank=6) | PREDICTED: tetratricopeptide repeat domain 34 [Homo sapiens] | [19.3](http://blast.ncbi.nlm.nih.gov/Blast.cgi#169162130%23169162130) | 19.3 | 100% | 134 |
| [NP_001032242.1](http://www.ncbi.nlm.nih.gov/entrez/query.fcgi?cmd=Retrieve&db=Protein&list_uids=82546824&dopt=GenPept&RID=T2GR2G3101S&log$=prottop&blast_rank=7) | forkhead box K1 [Homo sapiens] | [19.3](http://blast.ncbi.nlm.nih.gov/Blast.cgi#82546824%2382546824) | 19.3 | 100% | 134 |
| [NP_005667.2](http://www.ncbi.nlm.nih.gov/entrez/query.fcgi?cmd=Retrieve&db=Protein&list_uids=20127479&dopt=GenPept&RID=T2GR2G3101S&log$=prottop&blast_rank=8) | RNA binding motif protein 10 isoform 1 [Homo sapiens] | [19.3](http://blast.ncbi.nlm.nih.gov/Blast.cgi#20127479%2320127479) | 19.3 | 100% | 134 |
| [NP_690595.1](http://www.ncbi.nlm.nih.gov/entrez/query.fcgi?cmd=Retrieve&db=Protein&list_uids=23111018&dopt=GenPept&RID=T2GR2G3101S&log$=prottop&blast_rank=9) | RNA binding motif protein 10 isoform 2 [Homo sapiens] | [19.3](http://blast.ncbi.nlm.nih.gov/Blast.cgi#23111018%2323111018) | 19.3 | 100% | 134 |
| [NP_078795.2](http://www.ncbi.nlm.nih.gov/entrez/query.fcgi?cmd=Retrieve&db=Protein&list_uids=39995080&dopt=GenPept&RID=T2GR2G3101S&log$=prottop&blast_rank=10) | hypothetical protein LOC79567 [Homo sapiens] | [19.3](http://blast.ncbi.nlm.nih.gov/Blast.cgi#39995080%2339995080) | 134 | 100% | 134 |
| [NP_003763.2](http://www.ncbi.nlm.nih.gov/entrez/query.fcgi?cmd=Retrieve&db=Protein&list_uids=22547224&dopt=GenPept&RID=T2GR2G3101S&log$=prottop&blast_rank=11) | jerky homolog-like [Homo sapiens] | [19.3](http://blast.ncbi.nlm.nih.gov/Blast.cgi#22547224%2322547224) | 19.3 | 100% | 134 |
| [NP_004512.1](http://www.ncbi.nlm.nih.gov/entrez/query.fcgi?cmd=Retrieve&db=Protein&list_uids=4758648&dopt=GenPept&RID=T2GR2G3101S&log$=prottop&blast_rank=12) | kinesin family member 5B [Homo sapiens] | [19.3](http://blast.ncbi.nlm.nih.gov/Blast.cgi#4758648%234758648) | 19.3 | 100% | 134 |
| [NP_932332.1](http://www.ncbi.nlm.nih.gov/entrez/query.fcgi?cmd=Retrieve&db=Protein&list_uids=37620194&dopt=GenPept&RID=T2GR2G3101S&log$=prottop&blast_rank=13) | glucosamine-phosphate N-acetyltransferase 1 [Homo sapiens] | [19.3](http://blast.ncbi.nlm.nih.gov/Blast.cgi#37620194%2337620194) | 19.3 | 100% | 134 |
| [NP_000451.1](http://www.ncbi.nlm.nih.gov/entrez/query.fcgi?cmd=Retrieve&db=Protein&list_uids=4507493&dopt=GenPept&RID=T2GR2G3101S&log$=prottop&blast_rank=14) | thrombopoietin precursor [Homo sapiens] | [19.3](http://blast.ncbi.nlm.nih.gov/Blast.cgi#4507493%234507493) | 19.3 | 100% | 134 |
| [NP_065137.1](http://www.ncbi.nlm.nih.gov/entrez/query.fcgi?cmd=Retrieve&db=Protein&list_uids=9966885&dopt=GenPept&RID=T2GR2G3101S&log$=prottop&blast_rank=15) | tumor endothelial marker 1 precursor [Homo sapiens] | [19.3](http://blast.ncbi.nlm.nih.gov/Blast.cgi#9966885%239966885) | 19.3 | 100% | 134 |
| [NP_055368.1](http://www.ncbi.nlm.nih.gov/entrez/query.fcgi?cmd=Retrieve&db=Protein&list_uids=7657299&dopt=GenPept&RID=T2GR2G3101S&log$=prottop&blast_rank=16) | LBP-9 [Homo sapiens] | [19.3](http://blast.ncbi.nlm.nih.gov/Blast.cgi#7657299%237657299) | 19.3 | 100% | 134 |
| [NP_055642.3](http://www.ncbi.nlm.nih.gov/entrez/query.fcgi?cmd=Retrieve&db=Protein&list_uids=114842410&dopt=GenPept&RID=T2GR2G3101S&log$=prottop&blast_rank=17) | zinc finger CCCH-type containing 11A [Homo sapiens] | [19.3](http://blast.ncbi.nlm.nih.gov/Blast.cgi#114842410%23114842410) | 19.3 | 100% | 134 |
| [NP_001369.1](http://www.ncbi.nlm.nih.gov/entrez/query.fcgi?cmd=Retrieve&db=Protein&list_uids=24307879&dopt=GenPept&RID=T2GR2G3101S&log$=prottop&blast_rank=18) | dynein, cytoplasmic 1, intermediate chain 2 [Homo sapiens] | [19.3](http://blast.ncbi.nlm.nih.gov/Blast.cgi#24307879%2324307879) | 19.3 | 100% | 134 |
| [NP_001164225.1](http://www.ncbi.nlm.nih.gov/entrez/query.fcgi?cmd=Retrieve&db=Protein&list_uids=282721090&dopt=GenPept&RID=T2GR2G3101S&log$=prottop&blast_rank=19) | hypothetical protein LOC148345 [Homo sapiens] | [16.8](http://blast.ncbi.nlm.nih.gov/Blast.cgi#282721090%23282721090) | 16.8 | 80% | 782 |
| [XP_001717666.2](http://www.ncbi.nlm.nih.gov/entrez/query.fcgi?cmd=Retrieve&db=Protein&list_uids=239755446&dopt=GenPept&RID=T2GR2G3101S&log$=prottop&blast_rank=20) | PREDICTED: hypothetical protein [Homo sapiens] | [16.8](http://blast.ncbi.nlm.nih.gov/Blast.cgi#239755446%23239755446) | 16.8 | 80% | 782 |
| [XP_002346172.1](http://www.ncbi.nlm.nih.gov/entrez/query.fcgi?cmd=Retrieve&db=Protein&list_uids=239754965&dopt=GenPept&RID=T2GR2G3101S&log$=prottop&blast_rank=21) | PREDICTED: hypothetical protein [Homo sapiens] | [16.8](http://blast.ncbi.nlm.nih.gov/Blast.cgi#239754965%23239754965) | 16.8 | 80% | 782 |
| [XP_002345881.1](http://www.ncbi.nlm.nih.gov/entrez/query.fcgi?cmd=Retrieve&db=Protein&list_uids=239754110&dopt=GenPept&RID=T2GR2G3101S&log$=prottop&blast_rank=22) | PREDICTED: hypothetical protein XP_002345881 [Homo sapiens] | [16.8](http://blast.ncbi.nlm.nih.gov/Blast.cgi#239754110%23239754110) | 16.8 | 80% | 782 |
| [XP_002345835.1](http://www.ncbi.nlm.nih.gov/entrez/query.fcgi?cmd=Retrieve&db=Protein&list_uids=239753972&dopt=GenPept&RID=T2GR2G3101S&log$=prottop&blast_rank=23) | PREDICTED: hypothetical protein [Homo sapiens] | [16.8](http://blast.ncbi.nlm.nih.gov/Blast.cgi#239753972%23239753972) | 82.5 | 100% | 782 |
| [XP_002347816.1](http://www.ncbi.nlm.nih.gov/entrez/query.fcgi?cmd=Retrieve&db=Protein&list_uids=239752628&dopt=GenPept&RID=T2GR2G3101S&log$=prottop&blast_rank=24) | PREDICTED: hypothetical protein XP_002347816 [Homo sapiens] | [16.8](http://blast.ncbi.nlm.nih.gov/Blast.cgi#239752628%23239752628) | 16.8 | 80% | 782 |
| [XP_002347924.1](http://www.ncbi.nlm.nih.gov/entrez/query.fcgi?cmd=Retrieve&db=Protein&list_uids=239751592&dopt=GenPept&RID=T2GR2G3101S&log$=prottop&blast_rank=25) | PREDICTED: hypothetical protein XP_002347924 [Homo sapiens] | [16.8](http://blast.ncbi.nlm.nih.gov/Blast.cgi#239751592%23239751592) | 16.8 | 80% | 782 |
| [XP_002347214.1](http://www.ncbi.nlm.nih.gov/entrez/query.fcgi?cmd=Retrieve&db=Protein&list_uids=239749927&dopt=GenPept&RID=T2GR2G3101S&log$=prottop&blast_rank=26) | PREDICTED: hypothetical protein [Homo sapiens] | [16.8](http://blast.ncbi.nlm.nih.gov/Blast.cgi#239749927%23239749927) | 16.8 | 80% | 782 |
| [XP_002346752.1](http://www.ncbi.nlm.nih.gov/entrez/query.fcgi?cmd=Retrieve&db=Protein&list_uids=239748639&dopt=GenPept&RID=T2GR2G3101S&log$=prottop&blast_rank=27) | PREDICTED: hypothetical protein XP_002346752 [Homo sapiens] | [16.8](http://blast.ncbi.nlm.nih.gov/Blast.cgi#239748639%23239748639) | 16.8 | 80% | 782 |
| [XP_002343446.1](http://www.ncbi.nlm.nih.gov/entrez/query.fcgi?cmd=Retrieve&db=Protein&list_uids=239745360&dopt=GenPept&RID=T2GR2G3101S&log$=prottop&blast_rank=28) | PREDICTED: hypothetical protein XP_002343446 [Homo sapiens] >ref|XP_002347616.1| PREDICTED: hypothetical protein XP_002347616 [Homo sapiens] | [16.8](http://blast.ncbi.nlm.nih.gov/Blast.cgi#239745360%23239745360) | 16.8 | 80% | 782 |
| [XP_001727010.2](http://www.ncbi.nlm.nih.gov/entrez/query.fcgi?cmd=Retrieve&db=Protein&list_uids=239745140&dopt=GenPept&RID=T2GR2G3101S&log$=prottop&blast_rank=29) | PREDICTED: similar to hect domain and RLD 2 [Homo sapiens] | [16.8](http://blast.ncbi.nlm.nih.gov/Blast.cgi#239745140%23239745140) | 16.8 | 80% | 782 |
| [XP_001724205.2](http://www.ncbi.nlm.nih.gov/entrez/query.fcgi?cmd=Retrieve&db=Protein&list_uids=239745095&dopt=GenPept&RID=T2GR2G3101S&log$=prottop&blast_rank=30) | PREDICTED: hypothetical protein LOC645202 [Homo sapiens] | [16.8](http://blast.ncbi.nlm.nih.gov/Blast.cgi#239745095%23239745095) | 83.8 | 80% | 782 |
| [XP_496041.5](http://www.ncbi.nlm.nih.gov/entrez/query.fcgi?cmd=Retrieve&db=Protein&list_uids=239745079&dopt=GenPept&RID=T2GR2G3101S&log$=prottop&blast_rank=31) | PREDICTED: Putative golgin subfamily A member 6-like protein 6 [Homo sapiens] | [16.8](http://blast.ncbi.nlm.nih.gov/Blast.cgi#239745079%23239745079) | 50.3 | 80% | 782 |
| [XP_002343336.1](http://www.ncbi.nlm.nih.gov/entrez/query.fcgi?cmd=Retrieve&db=Protein&list_uids=239745032&dopt=GenPept&RID=T2GR2G3101S&log$=prottop&blast_rank=32) | PREDICTED: hypothetical protein XP_002343336 [Homo sapiens] | [16.8](http://blast.ncbi.nlm.nih.gov/Blast.cgi#239745032%23239745032) | 114 | 100% | 782 |
| [XP_002342304.1](http://www.ncbi.nlm.nih.gov/entrez/query.fcgi?cmd=Retrieve&db=Protein&list_uids=239741712&dopt=GenPept&RID=T2GR2G3101S&log$=prottop&blast_rank=33) | PREDICTED: hypothetical protein XP_002342304 [Homo sapiens] >ref|XP_002346446.1| PREDICTED: hypothetical protein [Homo sapiens] >ref|XP_002345606.1| PREDICTED: hypothetical protein [Homo sapiens] | [16.8](http://blast.ncbi.nlm.nih.gov/Blast.cgi#239741712%23239741712) | 16.8 | 80% | 782 |
| [NP_001001413.3](http://www.ncbi.nlm.nih.gov/entrez/query.fcgi?cmd=Retrieve&db=Protein&list_uids=222446603&dopt=GenPept&RID=T2GR2G3101S&log$=prottop&blast_rank=34) | golgin A6 family-like 1 [Homo sapiens] | [16.8](http://blast.ncbi.nlm.nih.gov/Blast.cgi#222446603%23222446603) | 117 | 80% | 782 |
| [NP_001138476.1](http://www.ncbi.nlm.nih.gov/entrez/query.fcgi?cmd=Retrieve&db=Protein&list_uids=222418637&dopt=GenPept&RID=T2GR2G3101S&log$=prottop&blast_rank=35) | golgin A6 family-like 6 [Homo sapiens] | [16.8](http://blast.ncbi.nlm.nih.gov/Blast.cgi#222418637%23222418637) | 117 | 80% | 782 |
| [NP_996816.2](http://www.ncbi.nlm.nih.gov/entrez/query.fcgi?cmd=Retrieve&db=Protein&list_uids=219842266&dopt=GenPept&RID=T2GR2G3101S&log$=prottop&blast_rank=36) | usherin isoform B [Homo sapiens] | [16.8](http://blast.ncbi.nlm.nih.gov/Blast.cgi#219842266%23219842266) | 16.8 | 80% | 782 |
| [NP_001136202.1](http://www.ncbi.nlm.nih.gov/entrez/query.fcgi?cmd=Retrieve&db=Protein&list_uids=218505694&dopt=GenPept&RID=T2GR2G3101S&log$=prottop&blast_rank=37) | potassium channel tetramerisation domain containing 1 isoform b [Homo sapiens] | [16.8](http://blast.ncbi.nlm.nih.gov/Blast.cgi#218505694%23218505694) | 16.8 | 80% | 782 |
| [NP_001155973.1](http://www.ncbi.nlm.nih.gov/entrez/query.fcgi?cmd=Retrieve&db=Protein&list_uids=241982729&dopt=GenPept&RID=T2GR2G3101S&log$=prottop&blast_rank=38) | trinucleotide repeat containing 6B isoform 1 [Homo sapiens] | [16.8](http://blast.ncbi.nlm.nih.gov/Blast.cgi#241982729%23241982729) | 33.1 | 100% | 782 |
| [NP_060212.4](http://www.ncbi.nlm.nih.gov/entrez/query.fcgi?cmd=Retrieve&db=Protein&list_uids=170016069&dopt=GenPept&RID=T2GR2G3101S&log$=prottop&blast_rank=39) | zinc finger, CCHC domain containing 2 [Homo sapiens] | [16.8](http://blast.ncbi.nlm.nih.gov/Blast.cgi#170016069%23170016069) | 16.8 | 80% | 782 |
| [XP_001726064.1](http://www.ncbi.nlm.nih.gov/entrez/query.fcgi?cmd=Retrieve&db=Protein&list_uids=169216018&dopt=GenPept&RID=T2GR2G3101S&log$=prottop&blast_rank=40) | PREDICTED: similar to hCG40360 [Homo sapiens] | [16.8](http://blast.ncbi.nlm.nih.gov/Blast.cgi#169216018%23169216018) | 16.8 | 80% | 782 |
| [NP_001161707.1](http://www.ncbi.nlm.nih.gov/entrez/query.fcgi?cmd=Retrieve&db=Protein&list_uids=270265871&dopt=GenPept&RID=T2GR2G3101S&log$=prottop&blast_rank=41) | FRAS1 related extracellular matrix 3 precursor [Homo sapiens] | [16.8](http://blast.ncbi.nlm.nih.gov/Blast.cgi#270265871%23270265871) | 16.8 | 80% | 782 |
| [XP_001716716.1](http://www.ncbi.nlm.nih.gov/entrez/query.fcgi?cmd=Retrieve&db=Protein&list_uids=169161159&dopt=GenPept&RID=T2GR2G3101S&log$=prottop&blast_rank=42) | PREDICTED: hypothetical protein [Homo sapiens] >ref|XP_001716051.1| PREDICTED: hypothetical protein [Homo sapiens] >ref|XP_001721177.1| PREDICTED: hypothetical protein [Homo sapiens] | [16.8](http://blast.ncbi.nlm.nih.gov/Blast.cgi#169161159%23169161159) | 16.8 | 80% | 782 |
| [XP_001131286.2](http://www.ncbi.nlm.nih.gov/entrez/query.fcgi?cmd=Retrieve&db=Protein&list_uids=169215613&dopt=GenPept&RID=T2GR2G3101S&log$=prottop&blast_rank=43) | PREDICTED: similar to hCG40360 [Homo sapiens] | [16.8](http://blast.ncbi.nlm.nih.gov/Blast.cgi#169215613%23169215613) | 16.8 | 80% | 782 |
| [NP_694591.2](http://www.ncbi.nlm.nih.gov/entrez/query.fcgi?cmd=Retrieve&db=Protein&list_uids=166851804&dopt=GenPept&RID=T2GR2G3101S&log$=prottop&blast_rank=44) | tudor domain containing 9 [Homo sapiens] | [16.8](http://blast.ncbi.nlm.nih.gov/Blast.cgi#166851804%23166851804) | 16.8 | 80% | 782 |
| [NP_001164722.1](http://www.ncbi.nlm.nih.gov/entrez/query.fcgi?cmd=Retrieve&db=Protein&list_uids=284005249&dopt=GenPept&RID=T2GR2G3101S&log$=prottop&blast_rank=45) | hypothetical protein LOC78995 isoform 2 [Homo sapiens] | [16.8](http://blast.ncbi.nlm.nih.gov/Blast.cgi#284005249%23284005249) | 16.8 | 80% | 782 |
| [NP_001087195.1](http://www.ncbi.nlm.nih.gov/entrez/query.fcgi?cmd=Retrieve&db=Protein&list_uids=148277022&dopt=GenPept&RID=T2GR2G3101S&log$=prottop&blast_rank=46) | selenoprotein P isoform 2 [Homo sapiens] | [16.8](http://blast.ncbi.nlm.nih.gov/Blast.cgi#148277022%23148277022) | 16.8 | 80% | 782 |
| [NP_004516.2](http://www.ncbi.nlm.nih.gov/entrez/query.fcgi?cmd=Retrieve&db=Protein&list_uids=126012573&dopt=GenPept&RID=T2GR2G3101S&log$=prottop&blast_rank=47) | low density lipoprotein-related protein 2 precursor [Homo sapiens] | [16.8](http://blast.ncbi.nlm.nih.gov/Blast.cgi#126012573%23126012573) | 16.8 | 80% | 782 |
| [NP_998770.2](http://www.ncbi.nlm.nih.gov/entrez/query.fcgi?cmd=Retrieve&db=Protein&list_uids=125490356&dopt=GenPept&RID=T2GR2G3101S&log$=prottop&blast_rank=48) | zinc finger protein 517 [Homo sapiens] | [16.8](http://blast.ncbi.nlm.nih.gov/Blast.cgi#125490356%23125490356) | 16.8 | 80% | 782 |
| [NP_001164261.1](http://www.ncbi.nlm.nih.gov/entrez/query.fcgi?cmd=Retrieve&db=Protein&list_uids=283046671&dopt=GenPept&RID=T2GR2G3101S&log$=prottop&blast_rank=49) | major facilitator superfamily domain containing 5 isoform 1 [Homo sapiens] | [16.8](http://blast.ncbi.nlm.nih.gov/Blast.cgi#283046671%23283046671) | 16.8 | 80% | 782 |
| [NP_001119808.1](http://www.ncbi.nlm.nih.gov/entrez/query.fcgi?cmd=Retrieve&db=Protein&list_uids=187607300&dopt=GenPept&RID=T2GR2G3101S&log$=prottop&blast_rank=50) | versican isoform 2 precursor [Homo sapiens] | [16.8](http://blast.ncbi.nlm.nih.gov/Blast.cgi#187607300%23187607300) | 16.8 | 80% | 782 |
| [NP_001120864.1](http://www.ncbi.nlm.nih.gov/entrez/query.fcgi?cmd=Retrieve&db=Protein&list_uids=188528652&dopt=GenPept&RID=T2GR2G3101S&log$=prottop&blast_rank=51) | myelin gene regulatory factor isoform 2 [Homo sapiens] | [16.8](http://blast.ncbi.nlm.nih.gov/Blast.cgi#188528652%23188528652) | 16.8 | 80% | 782 |
| [NP_003473.3](http://www.ncbi.nlm.nih.gov/entrez/query.fcgi?cmd=Retrieve&db=Protein&list_uids=148762969&dopt=GenPept&RID=T2GR2G3101S&log$=prottop&blast_rank=52) | myeloid/lymphoid or mixed-lineage leukemia 2 [Homo sapiens] | [16.8](http://blast.ncbi.nlm.nih.gov/Blast.cgi#148762969%23148762969) | 16.8 | 80% | 782 |
| [NP_003606.3](http://www.ncbi.nlm.nih.gov/entrez/query.fcgi?cmd=Retrieve&db=Protein&list_uids=134288865&dopt=GenPept&RID=T2GR2G3101S&log$=prottop&blast_rank=53) | solute carrier family 4, sodium bicarbonate cotransporter, member 7 [Homo sapiens] | [16.8](http://blast.ncbi.nlm.nih.gov/Blast.cgi#134288865%23134288865) | 16.8 | 80% | 782 |
| [NP_149016.2](http://www.ncbi.nlm.nih.gov/entrez/query.fcgi?cmd=Retrieve&db=Protein&list_uids=224593754&dopt=GenPept&RID=T2GR2G3101S&log$=prottop&blast_rank=54) | AXIN1 up-regulated 1 [Homo sapiens] | [16.8](http://blast.ncbi.nlm.nih.gov/Blast.cgi#224593754%23224593754) | 16.8 | 80% | 782 |
| [NP_001017395.2](http://www.ncbi.nlm.nih.gov/entrez/query.fcgi?cmd=Retrieve&db=Protein&list_uids=190014610&dopt=GenPept&RID=T2GR2G3101S&log$=prottop&blast_rank=55) | transmembrane and coiled-coil domain family 1 isoform a [Homo sapiens] | [16.8](http://blast.ncbi.nlm.nih.gov/Blast.cgi#190014610%23190014610) | 16.8 | 80% | 782 |
| [NP_001120936.1](http://www.ncbi.nlm.nih.gov/entrez/query.fcgi?cmd=Retrieve&db=Protein&list_uids=188536004&dopt=GenPept&RID=T2GR2G3101S&log$=prottop&blast_rank=56) | zinc finger protein 469 [Homo sapiens] | [16.8](http://blast.ncbi.nlm.nih.gov/Blast.cgi#188536004%23188536004) | 30.5 | 100% | 782 |
| [NP_005206.2](http://www.ncbi.nlm.nih.gov/entrez/query.fcgi?cmd=Retrieve&db=Protein&list_uids=110431348&dopt=GenPept&RID=T2GR2G3101S&log$=prottop&blast_rank=57) | netrin receptor DCC precursor [Homo sapiens] | [16.8](http://blast.ncbi.nlm.nih.gov/Blast.cgi#110431348%23110431348) | 16.8 | 80% | 782 |
| [NP_705838.3](http://www.ncbi.nlm.nih.gov/entrez/query.fcgi?cmd=Retrieve&db=Protein&list_uids=62241003&dopt=GenPept&RID=T2GR2G3101S&log$=prottop&blast_rank=58) | cardiomyopathy associated 5 [Homo sapiens] | [16.8](http://blast.ncbi.nlm.nih.gov/Blast.cgi#62241003%2362241003) | 16.8 | 80% | 782 |
| [NP_060252.3](http://www.ncbi.nlm.nih.gov/entrez/query.fcgi?cmd=Retrieve&db=Protein&list_uids=148806891&dopt=GenPept&RID=T2GR2G3101S&log$=prottop&blast_rank=59) | hypothetical protein LOC54906 [Homo sapiens] | [16.8](http://blast.ncbi.nlm.nih.gov/Blast.cgi#148806891%23148806891) | 16.8 | 80% | 782 |
| [NP_055988.2](http://www.ncbi.nlm.nih.gov/entrez/query.fcgi?cmd=Retrieve&db=Protein&list_uids=50658061&dopt=GenPept&RID=T2GR2G3101S&log$=prottop&blast_rank=60) | TBC1 (tre-2/USP6, BUB2, cdc16) domain family, member 1 [Homo sapiens] | [16.8](http://blast.ncbi.nlm.nih.gov/Blast.cgi#50658061%2350658061) | 16.8 | 80% | 782 |
| [NP_982284.1](http://www.ncbi.nlm.nih.gov/entrez/query.fcgi?cmd=Retrieve&db=Protein&list_uids=44955929&dopt=GenPept&RID=T2GR2G3101S&log$=prottop&blast_rank=61) | calmodulin regulated spectrin-associated protein 1-like 1 [Homo sapiens] | [16.8](http://blast.ncbi.nlm.nih.gov/Blast.cgi#44955929%2344955929) | 16.8 | 80% | 782 |
| [NP_954872.1](http://www.ncbi.nlm.nih.gov/entrez/query.fcgi?cmd=Retrieve&db=Protein&list_uids=40805852&dopt=GenPept&RID=T2GR2G3101S&log$=prottop&blast_rank=62) | thrombospondin type I domain-containing 1 isoform 2 [Homo sapiens] | [16.8](http://blast.ncbi.nlm.nih.gov/Blast.cgi#40805852%2340805852) | 16.8 | 80% | 782 |
| [NP_112562.3](http://www.ncbi.nlm.nih.gov/entrez/query.fcgi?cmd=Retrieve&db=Protein&list_uids=57863261&dopt=GenPept&RID=T2GR2G3101S&log$=prottop&blast_rank=63) | testis expressed sequence 14 isoform b [Homo sapiens] | [16.8](http://blast.ncbi.nlm.nih.gov/Blast.cgi#57863261%2357863261) | 16.8 | 80% | 782 |
| [NP_689705.2](http://www.ncbi.nlm.nih.gov/entrez/query.fcgi?cmd=Retrieve&db=Protein&list_uids=217035164&dopt=GenPept&RID=T2GR2G3101S&log$=prottop&blast_rank=64) | coiled-coil domain containing 27 [Homo sapiens] | [16.8](http://blast.ncbi.nlm.nih.gov/Blast.cgi#217035164%23217035164) | 16.8 | 80% | 782 |
| [NP_997054.1](http://www.ncbi.nlm.nih.gov/entrez/query.fcgi?cmd=Retrieve&db=Protein&list_uids=46397394&dopt=GenPept&RID=T2GR2G3101S&log$=prottop&blast_rank=65) | pogo transposable element with ZNF domain isoform 2 [Homo sapiens] | [16.8](http://blast.ncbi.nlm.nih.gov/Blast.cgi#46397394%2346397394) | 16.8 | 80% | 782 |
| [NP_003695.3](http://www.ncbi.nlm.nih.gov/entrez/query.fcgi?cmd=Retrieve&db=Protein&list_uids=51873049&dopt=GenPept&RID=T2GR2G3101S&log$=prottop&blast_rank=66) | hypothetical protein LOC8603 [Homo sapiens] | [16.8](http://blast.ncbi.nlm.nih.gov/Blast.cgi#51873049%2351873049) | 16.8 | 80% | 782 |
| [NP_003822.2](http://www.ncbi.nlm.nih.gov/entrez/query.fcgi?cmd=Retrieve&db=Protein&list_uids=22325377&dopt=GenPept&RID=T2GR2G3101S&log$=prottop&blast_rank=67) | sudD suppressor of bimD6 homolog [Homo sapiens] | [16.8](http://blast.ncbi.nlm.nih.gov/Blast.cgi#22325377%2322325377) | 16.8 | 80% | 782 |
| [NP_001157569.1](http://www.ncbi.nlm.nih.gov/entrez/query.fcgi?cmd=Retrieve&db=Protein&list_uids=255918077&dopt=GenPept&RID=T2GR2G3101S&log$=prottop&blast_rank=68) | versican isoform 3 precursor [Homo sapiens] | [16.8](http://blast.ncbi.nlm.nih.gov/Blast.cgi#255918077%23255918077) | 16.8 | 80% | 782 |
| [NP_001074295.1](http://www.ncbi.nlm.nih.gov/entrez/query.fcgi?cmd=Retrieve&db=Protein&list_uids=157909822&dopt=GenPept&RID=T2GR2G3101S&log$=prottop&blast_rank=69) | pragmin [Homo sapiens] | [16.8](http://blast.ncbi.nlm.nih.gov/Blast.cgi#157909822%23157909822) | 16.8 | 80% | 782 |
| [NP_644671.1](http://www.ncbi.nlm.nih.gov/entrez/query.fcgi?cmd=Retrieve&db=Protein&list_uids=21536301&dopt=GenPept&RID=T2GR2G3101S&log$=prottop&blast_rank=70) | signal transducer and activator of transcription 1 isoform beta [Homo sapiens] | [16.8](http://blast.ncbi.nlm.nih.gov/Blast.cgi#21536301%2321536301) | 16.8 | 80% | 782 |
| [NP_001157570.1](http://www.ncbi.nlm.nih.gov/entrez/query.fcgi?cmd=Retrieve&db=Protein&list_uids=255918079&dopt=GenPept&RID=T2GR2G3101S&log$=prottop&blast_rank=71) | versican isoform 4 precursor [Homo sapiens] | [16.8](http://blast.ncbi.nlm.nih.gov/Blast.cgi#255918079%23255918079) | 16.8 | 80% | 782 |
| [NP_919278.2](http://www.ncbi.nlm.nih.gov/entrez/query.fcgi?cmd=Retrieve&db=Protein&list_uids=83035129&dopt=GenPept&RID=T2GR2G3101S&log$=prottop&blast_rank=72) | coiled-coil domain containing 108 isoform 1 [Homo sapiens] | [16.8](http://blast.ncbi.nlm.nih.gov/Blast.cgi#83035129%2383035129) | 16.8 | 80% | 782 |
| [NP_055928.3](http://www.ncbi.nlm.nih.gov/entrez/query.fcgi?cmd=Retrieve&db=Protein&list_uids=73747881&dopt=GenPept&RID=T2GR2G3101S&log$=prottop&blast_rank=73) | zinc finger, ZZ type with EF hand domain 1 [Homo sapiens] | [16.8](http://blast.ncbi.nlm.nih.gov/Blast.cgi#73747881%2373747881) | 16.8 | 80% | 782 |
| [NP_001158608.1](http://www.ncbi.nlm.nih.gov/entrez/query.fcgi?cmd=Retrieve&db=Protein&list_uids=259089405&dopt=GenPept&RID=T2GR2G3101S&log$=prottop&blast_rank=74) | hect domain and RLD 6 isoform 2 [Homo sapiens] | [16.8](http://blast.ncbi.nlm.nih.gov/Blast.cgi#259089405%23259089405) | 16.8 | 80% | 782 |
| [NP_060382.3](http://www.ncbi.nlm.nih.gov/entrez/query.fcgi?cmd=Retrieve&db=Protein&list_uids=61563742&dopt=GenPept&RID=T2GR2G3101S&log$=prottop&blast_rank=75) | hect domain and RLD 6 isoform 1 [Homo sapiens] | [16.8](http://blast.ncbi.nlm.nih.gov/Blast.cgi#61563742%2361563742) | 16.8 | 80% | 782 |
| [NP_005574.2](http://www.ncbi.nlm.nih.gov/entrez/query.fcgi?cmd=Retrieve&db=Protein&list_uids=34147558&dopt=GenPept&RID=T2GR2G3101S&log$=prottop&blast_rank=76) | lymphoblastic leukemia derived sequence 1 [Homo sapiens] | [16.8](http://blast.ncbi.nlm.nih.gov/Blast.cgi#34147558%2334147558) | 16.8 | 80% | 782 |
| [NP_116161.2](http://www.ncbi.nlm.nih.gov/entrez/query.fcgi?cmd=Retrieve&db=Protein&list_uids=24432032&dopt=GenPept&RID=T2GR2G3101S&log$=prottop&blast_rank=77) | zinc finger protein 503 [Homo sapiens] | [16.8](http://blast.ncbi.nlm.nih.gov/Blast.cgi#24432032%2324432032) | 16.8 | 80% | 782 |
| [NP_076432.1](http://www.ncbi.nlm.nih.gov/entrez/query.fcgi?cmd=Retrieve&db=Protein&list_uids=45387951&dopt=GenPept&RID=T2GR2G3101S&log$=prottop&blast_rank=78) | transmembrane protein 108 precursor [Homo sapiens] >ref|NP_001129941.1| transmembrane protein 108 precursor [Homo sapiens] | [16.8](http://blast.ncbi.nlm.nih.gov/Blast.cgi#45387951%2345387951) | 16.8 | 80% | 782 |
| [NP_003945.2](http://www.ncbi.nlm.nih.gov/entrez/query.fcgi?cmd=Retrieve&db=Protein&list_uids=115298645&dopt=GenPept&RID=T2GR2G3101S&log$=prottop&blast_rank=79) | mitogen-activated protein kinase kinase kinase 14 [Homo sapiens] | [16.8](http://blast.ncbi.nlm.nih.gov/Blast.cgi#115298645%23115298645) | 16.8 | 80% | 782 |
| [NP_037411.1](http://www.ncbi.nlm.nih.gov/entrez/query.fcgi?cmd=Retrieve&db=Protein&list_uids=7019335&dopt=GenPept&RID=T2GR2G3101S&log$=prottop&blast_rank=80) | myelin gene regulatory factor isoform 1 [Homo sapiens] | [16.8](http://blast.ncbi.nlm.nih.gov/Blast.cgi#7019335%237019335) | 16.8 | 80% | 782 |
| [NP_733776.1](http://www.ncbi.nlm.nih.gov/entrez/query.fcgi?cmd=Retrieve&db=Protein&list_uids=24762250&dopt=GenPept&RID=T2GR2G3101S&log$=prottop&blast_rank=81) | Meis homeobox 2 isoform d [Homo sapiens] | [16.8](http://blast.ncbi.nlm.nih.gov/Blast.cgi#24762250%2324762250) | 16.8 | 80% | 782 |
| [NP_071435.2](http://www.ncbi.nlm.nih.gov/entrez/query.fcgi?cmd=Retrieve&db=Protein&list_uids=50593008&dopt=GenPept&RID=T2GR2G3101S&log$=prottop&blast_rank=82) | transmembrane BAX inhibitor motif containing 1 [Homo sapiens] | [16.8](http://blast.ncbi.nlm.nih.gov/Blast.cgi#50593008%2350593008) | 16.8 | 80% | 782 |
| [NP_005401.3](http://www.ncbi.nlm.nih.gov/entrez/query.fcgi?cmd=Retrieve&db=Protein&list_uids=62530391&dopt=GenPept&RID=T2GR2G3101S&log$=prottop&blast_rank=83) | selenoprotein P isoform 1 precursor [Homo sapiens] >ref|NP_001078955.1| selenoprotein P isoform 1 precursor [Homo sapiens] | [16.8](http://blast.ncbi.nlm.nih.gov/Blast.cgi#62530391%2362530391) | 16.8 | 80% | 782 |
| [NP_938207.2](http://www.ncbi.nlm.nih.gov/entrez/query.fcgi?cmd=Retrieve&db=Protein&list_uids=57863263&dopt=GenPept&RID=T2GR2G3101S&log$=prottop&blast_rank=84) | testis expressed sequence 14 isoform a [Homo sapiens] | [16.8](http://blast.ncbi.nlm.nih.gov/Blast.cgi#57863263%2357863263) | 16.8 | 80% | 782 |
| [NP_055647.2](http://www.ncbi.nlm.nih.gov/entrez/query.fcgi?cmd=Retrieve&db=Protein&list_uids=114688046&dopt=GenPept&RID=T2GR2G3101S&log$=prottop&blast_rank=85) | TBC1 domain family, member 4 [Homo sapiens] | [16.8](http://blast.ncbi.nlm.nih.gov/Blast.cgi#114688046%23114688046) | 16.8 | 80% | 782 |
| [NP_055915.2](http://www.ncbi.nlm.nih.gov/entrez/query.fcgi?cmd=Retrieve&db=Protein&list_uids=46397390&dopt=GenPept&RID=T2GR2G3101S&log$=prottop&blast_rank=86) | pogo transposable element with ZNF domain isoform 1 [Homo sapiens] | [16.8](http://blast.ncbi.nlm.nih.gov/Blast.cgi#46397390%2346397390) | 16.8 | 80% | 782 |
| [NP_005086.2](http://www.ncbi.nlm.nih.gov/entrez/query.fcgi?cmd=Retrieve&db=Protein&list_uids=44890068&dopt=GenPept&RID=T2GR2G3101S&log$=prottop&blast_rank=87) | zinc finger protein 262 [Homo sapiens] | [16.8](http://blast.ncbi.nlm.nih.gov/Blast.cgi#44890068%2344890068) | 16.8 | 80% | 782 |
| [NP_954871.1](http://www.ncbi.nlm.nih.gov/entrez/query.fcgi?cmd=Retrieve&db=Protein&list_uids=42263098&dopt=GenPept&RID=T2GR2G3101S&log$=prottop&blast_rank=88) | Sp6 transcription factor [Homo sapiens] | [16.8](http://blast.ncbi.nlm.nih.gov/Blast.cgi#42263098%2342263098) | 30.5 | 100% | 782 |
| [NP_004144.2](http://www.ncbi.nlm.nih.gov/entrez/query.fcgi?cmd=Retrieve&db=Protein&list_uids=31795544&dopt=GenPept&RID=T2GR2G3101S&log$=prottop&blast_rank=89) | origin recognition complex, subunit 1 [Homo sapiens] | [16.8](http://blast.ncbi.nlm.nih.gov/Blast.cgi#31795544%2331795544) | 16.8 | 80% | 782 |
| [NP_000098.1](http://www.ncbi.nlm.nih.gov/entrez/query.fcgi?cmd=Retrieve&db=Protein&list_uids=4557515&dopt=GenPept&RID=T2GR2G3101S&log$=prottop&blast_rank=90) | damage-specific DNA binding protein 2 [Homo sapiens] | [16.8](http://blast.ncbi.nlm.nih.gov/Blast.cgi#4557515%234557515) | 16.8 | 80% | 782 |
| [NP_775780.1](http://www.ncbi.nlm.nih.gov/entrez/query.fcgi?cmd=Retrieve&db=Protein&list_uids=28316806&dopt=GenPept&RID=T2GR2G3101S&log$=prottop&blast_rank=91) | family with sequence similarity 163, member A [Homo sapiens] | [16.8](http://blast.ncbi.nlm.nih.gov/Blast.cgi#28316806%2328316806) | 16.8 | 80% | 782 |
| [NP_003180.1](http://www.ncbi.nlm.nih.gov/entrez/query.fcgi?cmd=Retrieve&db=Protein&list_uids=4507363&dopt=GenPept&RID=T2GR2G3101S&log$=prottop&blast_rank=92) | T-cell acute lymphocytic leukemia 1 [Homo sapiens] | [16.8](http://blast.ncbi.nlm.nih.gov/Blast.cgi#4507363%234507363) | 16.8 | 80% | 782 |
| [NP_004376.2](http://www.ncbi.nlm.nih.gov/entrez/query.fcgi?cmd=Retrieve&db=Protein&list_uids=21361116&dopt=GenPept&RID=T2GR2G3101S&log$=prottop&blast_rank=93) | versican isoform 1 precursor [Homo sapiens] | [16.8](http://blast.ncbi.nlm.nih.gov/Blast.cgi#21361116%2321361116) | 16.8 | 80% | 782 |
| [NP_003396.1](http://www.ncbi.nlm.nih.gov/entrez/query.fcgi?cmd=Retrieve&db=Protein&list_uids=4507951&dopt=GenPept&RID=T2GR2G3101S&log$=prottop&blast_rank=94) | tyrosine 3-monooxygenase/tryptophan 5-monooxygenase activation protein, eta polypeptide [Homo sapiens] | [16.8](http://blast.ncbi.nlm.nih.gov/Blast.cgi#4507951%234507951) | 16.8 | 80% | 782 |
| [NP_055903.2](http://www.ncbi.nlm.nih.gov/entrez/query.fcgi?cmd=Retrieve&db=Protein&list_uids=148491080&dopt=GenPept&RID=T2GR2G3101S&log$=prottop&blast_rank=95) | trinucleotide repeat containing 6B isoform 2 [Homo sapiens] | [16.8](http://blast.ncbi.nlm.nih.gov/Blast.cgi#148491080%23148491080) | 33.1 | 100% | 782 |
| [NP_004349.1](http://www.ncbi.nlm.nih.gov/entrez/query.fcgi?cmd=Retrieve&db=Protein&list_uids=4757950&dopt=GenPept&RID=T2GR2G3101S&log$=prottop&blast_rank=96) | cell division cycle 25B isoform 2 [Homo sapiens] | [16.8](http://blast.ncbi.nlm.nih.gov/Blast.cgi#4757950%234757950) | 16.8 | 80% | 782 |
| [NP_733775.1](http://www.ncbi.nlm.nih.gov/entrez/query.fcgi?cmd=Retrieve&db=Protein&list_uids=24762241&dopt=GenPept&RID=T2GR2G3101S&log$=prottop&blast_rank=97) | Meis homeobox 2 isoform c [Homo sapiens] | [16.8](http://blast.ncbi.nlm.nih.gov/Blast.cgi#24762241%2324762241) | 16.8 | 80% | 782 |
| [NP_009330.1](http://www.ncbi.nlm.nih.gov/entrez/query.fcgi?cmd=Retrieve&db=Protein&list_uids=6274552&dopt=GenPept&RID=T2GR2G3101S&log$=prottop&blast_rank=98) | signal transducer and activator of transcription 1 isoform alpha [Homo sapiens] | [16.8](http://blast.ncbi.nlm.nih.gov/Blast.cgi#6274552%236274552) | 16.8 | 80% | 782 |
| [NP_068659.1](http://www.ncbi.nlm.nih.gov/entrez/query.fcgi?cmd=Retrieve&db=Protein&list_uids=11641413&dopt=GenPept&RID=T2GR2G3101S&log$=prottop&blast_rank=99) | cell division cycle 25B isoform 1 [Homo sapiens] | [16.8](http://blast.ncbi.nlm.nih.gov/Blast.cgi#11641413%2311641413) | 16.8 | 80% | 782 |
| [NP_005412.1](http://www.ncbi.nlm.nih.gov/entrez/query.fcgi?cmd=Retrieve&db=Protein&list_uids=4885619&dopt=GenPept&RID=T2GR2G3101S&log$=prottop&blast_rank=100) | T-cell acute lymphocytic leukemia 2 [Homo sapiens] | [16.8](http://blast.ncbi.nlm.nih.gov/Blast.cgi#4885619%234885619) | 16.8 | 80% | 782 |

| **Accession** | **Proteins with a match to GRRNKSG peptide** | **[Max score](http://blast.ncbi.nlm.nih.gov/Blast.cgi?CMD=Get&ALIGNMENTS=100&ALIGNMENT_VIEW=Pairwise&CDD_SEARCH_STATE=1&DATABASE_SORT=0&DESCRIPTIONS=100&ENTREZ_QUERY=txid9606 %5BORGN%5D&FIRST_QUERY_NUM=0&FORMAT_OBJECT=Alignment&FORMAT_PAGE_TARGET=&FORMAT_TYPE=HTML&GET_SEQUENCE=yes&I_THRESH=&MASK_CHAR=2&MASK_COLOR=1&NEW_DESIGN=on&NEW_VIEW=yes&NUM_OVERVIEW=100&OLD_BLAST=false&PAGE=Proteins&QUERY_INDEX=0&QUERY_NUMBER=0&RESULTS_PAGE_TARGET=&RID=T2GZEE68012&SHOW_LINKOUT=yes&SHOW_OVERVIEW=yes&STEP_NUMBER=&WORD_SIZE=2&DISPLAY_SORT=1&HSP_SORT=1" \l "sort_mark)** | **[Total score](http://blast.ncbi.nlm.nih.gov/Blast.cgi?CMD=Get&ALIGNMENTS=100&ALIGNMENT_VIEW=Pairwise&CDD_SEARCH_STATE=1&DATABASE_SORT=0&DESCRIPTIONS=100&ENTREZ_QUERY=txid9606 %5BORGN%5D&FIRST_QUERY_NUM=0&FORMAT_OBJECT=Alignment&FORMAT_PAGE_TARGET=&FORMAT_TYPE=HTML&GET_SEQUENCE=yes&I_THRESH=&MASK_CHAR=2&MASK_COLOR=1&NEW_DESIGN=on&NEW_VIEW=yes&NUM_OVERVIEW=100&OLD_BLAST=false&PAGE=Proteins&QUERY_INDEX=0&QUERY_NUMBER=0&RESULTS_PAGE_TARGET=&RID=T2GZEE68012&SHOW_LINKOUT=yes&SHOW_OVERVIEW=yes&STEP_NUMBER=&WORD_SIZE=2&DISPLAY_SORT=2&HSP_SORT=1" \l "sort_mark)** | **[Query coverage](http://blast.ncbi.nlm.nih.gov/Blast.cgi?CMD=Get&ALIGNMENTS=100&ALIGNMENT_VIEW=Pairwise&CDD_SEARCH_STATE=1&DATABASE_SORT=0&DESCRIPTIONS=100&ENTREZ_QUERY=txid9606 %5BORGN%5D&FIRST_QUERY_NUM=0&FORMAT_OBJECT=Alignment&FORMAT_PAGE_TARGET=&FORMAT_TYPE=HTML&GET_SEQUENCE=yes&I_THRESH=&MASK_CHAR=2&MASK_COLOR=1&NEW_DESIGN=on&NEW_VIEW=yes&NUM_OVERVIEW=100&OLD_BLAST=false&PAGE=Proteins&QUERY_INDEX=0&QUERY_NUMBER=0&RESULTS_PAGE_TARGET=&RID=T2GZEE68012&SHOW_LINKOUT=yes&SHOW_OVERVIEW=yes&STEP_NUMBER=&WORD_SIZE=2&DISPLAY_SORT=4&HSP_SORT=0" \l "sort_mark)** | **[E value](http://blast.ncbi.nlm.nih.gov/Blast.cgi?CMD=Get&ALIGNMENTS=100&ALIGNMENT_VIEW=Pairwise&CDD_SEARCH_STATE=1&DATABASE_SORT=0&DESCRIPTIONS=100&ENTREZ_QUERY=txid9606 %5BORGN%5D&FIRST_QUERY_NUM=0&FORMAT_OBJECT=Alignment&FORMAT_PAGE_TARGET=&FORMAT_TYPE=HTML&GET_SEQUENCE=yes&I_THRESH=&MASK_CHAR=2&MASK_COLOR=1&NEW_DESIGN=on&NEW_VIEW=yes&NUM_OVERVIEW=100&OLD_BLAST=false&PAGE=Proteins&QUERY_INDEX=0&QUERY_NUMBER=0&RESULTS_PAGE_TARGET=&RID=T2GZEE68012&SHOW_LINKOUT=yes&SHOW_OVERVIEW=yes&STEP_NUMBER=&WORD_SIZE=2&DISPLAY_SORT=0&HSP_SORT=0" \l "sort_mark)** |
| --- | --- | --- | --- | --- | --- |
| [NP_057547.5](http://www.ncbi.nlm.nih.gov/entrez/query.fcgi?cmd=Retrieve&db=Protein&list_uids=158261990&dopt=GenPept&RID=T2GZEE68012&log$=prottop&blast_rank=1) | CXXC finger 5 [Homo sapiens] | [21.4](http://blast.ncbi.nlm.nih.gov/Blast.cgi#158261990%23158261990) | 21.4 | 85% | 43 |
| [XP_001722402.1](http://www.ncbi.nlm.nih.gov/entrez/query.fcgi?cmd=Retrieve&db=Protein&list_uids=169212850&dopt=GenPept&RID=T2GZEE68012&log$=prottop&blast_rank=2) | PREDICTED: hypothetical protein [Homo sapiens] >ref|XP_001720022.1| PREDICTED: hypothetical protein [Homo sapiens] >ref|XP_001720012.1| PREDICTED: hypothetical protein [Homo sapiens] | [20.6](http://blast.ncbi.nlm.nih.gov/Blast.cgi#169212850%23169212850) | 20.6 | 100% | 78 |
| [NP_001157281.1](http://www.ncbi.nlm.nih.gov/entrez/query.fcgi?cmd=Retrieve&db=Protein&list_uids=255759952&dopt=GenPept&RID=T2GZEE68012&log$=prottop&blast_rank=3) | WD repeat domain 81 isoform 1 [Homo sapiens] | [18.9](http://blast.ncbi.nlm.nih.gov/Blast.cgi#255759952%23255759952) | 18.9 | 71% | 252 |
| [NP_001078823.1](http://www.ncbi.nlm.nih.gov/entrez/query.fcgi?cmd=Retrieve&db=Protein&list_uids=145553971&dopt=GenPept&RID=T2GZEE68012&log$=prottop&blast_rank=4) | zinc finger, CW type with coiled-coil domain 2 isoform b [Homo sapiens] | [18.9](http://blast.ncbi.nlm.nih.gov/Blast.cgi#145553971%23145553971) | 18.9 | 85% | 252 |
| [NP_056386.2](http://www.ncbi.nlm.nih.gov/entrez/query.fcgi?cmd=Retrieve&db=Protein&list_uids=156105701&dopt=GenPept&RID=T2GZEE68012&log$=prottop&blast_rank=5) | SUMO1/sentrin specific peptidase 6 isoform 1 [Homo sapiens] | [18.9](http://blast.ncbi.nlm.nih.gov/Blast.cgi#156105701%23156105701) | 18.9 | 71% | 252 |
| [NP_006378.3](http://www.ncbi.nlm.nih.gov/entrez/query.fcgi?cmd=Retrieve&db=Protein&list_uids=119226260&dopt=GenPept&RID=T2GZEE68012&log$=prottop&blast_rank=6) | calcium homeostasis endoplasmic reticulum protein [Homo sapiens] | [18.9](http://blast.ncbi.nlm.nih.gov/Blast.cgi#119226260%23119226260) | 18.9 | 71% | 252 |
| [NP_001025053.1](http://www.ncbi.nlm.nih.gov/entrez/query.fcgi?cmd=Retrieve&db=Protein&list_uids=71274144&dopt=GenPept&RID=T2GZEE68012&log$=prottop&blast_rank=7) | AT hook, DNA binding motif, containing 1 [Homo sapiens] | [18.9](http://blast.ncbi.nlm.nih.gov/Blast.cgi#71274144%2371274144) | 18.9 | 71% | 252 |
| [NP_001012331.1](http://www.ncbi.nlm.nih.gov/entrez/query.fcgi?cmd=Retrieve&db=Protein&list_uids=59889558&dopt=GenPept&RID=T2GZEE68012&log$=prottop&blast_rank=8) | neurotrophic tyrosine kinase, receptor, type 1 isoform 1 [Homo sapiens] | [18.9](http://blast.ncbi.nlm.nih.gov/Blast.cgi#59889558%2359889558) | 18.9 | 71% | 252 |
| [NP_002520.2](http://www.ncbi.nlm.nih.gov/entrez/query.fcgi?cmd=Retrieve&db=Protein&list_uids=4585712&dopt=GenPept&RID=T2GZEE68012&log$=prottop&blast_rank=9) | neurotrophic tyrosine kinase, receptor, type 1 isoform 2 [Homo sapiens] | [18.9](http://blast.ncbi.nlm.nih.gov/Blast.cgi#4585712%234585712) | 18.9 | 71% | 252 |
| [NP_001007793.1](http://www.ncbi.nlm.nih.gov/entrez/query.fcgi?cmd=Retrieve&db=Protein&list_uids=56118210&dopt=GenPept&RID=T2GZEE68012&log$=prottop&blast_rank=10) | neurotrophic tyrosine kinase, receptor, type 1 isoform 3 [Homo sapiens] | [18.9](http://blast.ncbi.nlm.nih.gov/Blast.cgi#56118210%2356118210) | 18.9 | 71% | 252 |
| [NP_065199.1](http://www.ncbi.nlm.nih.gov/entrez/query.fcgi?cmd=Retrieve&db=Protein&list_uids=10092689&dopt=GenPept&RID=T2GZEE68012&log$=prottop&blast_rank=11) | LYR motif containing 2 [Homo sapiens] | [18.9](http://blast.ncbi.nlm.nih.gov/Blast.cgi#10092689%2310092689) | 18.9 | 71% | 252 |
| [NP_001093879.1](http://www.ncbi.nlm.nih.gov/entrez/query.fcgi?cmd=Retrieve&db=Protein&list_uids=156105703&dopt=GenPept&RID=T2GZEE68012&log$=prottop&blast_rank=12) | SUMO1/sentrin specific peptidase 6 isoform 2 [Homo sapiens] | [18.9](http://blast.ncbi.nlm.nih.gov/Blast.cgi#156105703%23156105703) | 18.9 | 71% | 252 |
| [NP_078933.3](http://www.ncbi.nlm.nih.gov/entrez/query.fcgi?cmd=Retrieve&db=Protein&list_uids=145553976&dopt=GenPept&RID=T2GZEE68012&log$=prottop&blast_rank=13) | zinc finger, CW type with coiled-coil domain 2 isoform a [Homo sapiens] | [18.9](http://blast.ncbi.nlm.nih.gov/Blast.cgi#145553976%23145553976) | 18.9 | 85% | 252 |
| [NP_150254.1](http://www.ncbi.nlm.nih.gov/entrez/query.fcgi?cmd=Retrieve&db=Protein&list_uids=15431295&dopt=GenPept&RID=T2GZEE68012&log$=prottop&blast_rank=14) | ribosomal protein L13 [Homo sapiens] >ref|NP_000968.2| ribosomal protein L13 [Homo sapiens] | [18.9](http://blast.ncbi.nlm.nih.gov/Blast.cgi#15431295%2315431295) | 18.9 | 71% | 252 |
| [NP_002348.1](http://www.ncbi.nlm.nih.gov/entrez/query.fcgi?cmd=Retrieve&db=Protein&list_uids=4505069&dopt=GenPept&RID=T2GZEE68012&log$=prottop&blast_rank=15) | MAX dimerization protein 1 [Homo sapiens] | [18.9](http://blast.ncbi.nlm.nih.gov/Blast.cgi#4505069%234505069) | 18.9 | 71% | 252 |
| [NP_056051.2](http://www.ncbi.nlm.nih.gov/entrez/query.fcgi?cmd=Retrieve&db=Protein&list_uids=163792198&dopt=GenPept&RID=T2GZEE68012&log$=prottop&blast_rank=16) | latrophilin 3 precursor [Homo sapiens] | [18.5](http://blast.ncbi.nlm.nih.gov/Blast.cgi#163792198%23163792198) | 18.5 | 85% | 338 |
| [NP_061836.2](http://www.ncbi.nlm.nih.gov/entrez/query.fcgi?cmd=Retrieve&db=Protein&list_uids=16445436&dopt=GenPept&RID=T2GZEE68012&log$=prottop&blast_rank=17) | bromodomain and WD repeat domain containing 1 isoform A [Homo sapiens] | [18.5](http://blast.ncbi.nlm.nih.gov/Blast.cgi#16445436%2316445436) | 18.5 | 85% | 338 |
| [NP_387505.1](http://www.ncbi.nlm.nih.gov/entrez/query.fcgi?cmd=Retrieve&db=Protein&list_uids=16445438&dopt=GenPept&RID=T2GZEE68012&log$=prottop&blast_rank=18) | bromodomain and WD repeat domain containing 1 isoform B [Homo sapiens] | [18.5](http://blast.ncbi.nlm.nih.gov/Blast.cgi#16445438%2316445438) | 18.5 | 85% | 338 |
| [NP_001138995.1](http://www.ncbi.nlm.nih.gov/entrez/query.fcgi?cmd=Retrieve&db=Protein&list_uids=224809476&dopt=GenPept&RID=T2GZEE68012&log$=prottop&blast_rank=19) | retinoic acid induced 14 isoform c [Homo sapiens] | [18.0](http://blast.ncbi.nlm.nih.gov/Blast.cgi#224809476%23224809476) | 18.0 | 71% | 453 |
| [NP_001138994.1](http://www.ncbi.nlm.nih.gov/entrez/query.fcgi?cmd=Retrieve&db=Protein&list_uids=224809474&dopt=GenPept&RID=T2GZEE68012&log$=prottop&blast_rank=20) | retinoic acid induced 14 isoform b [Homo sapiens] | [18.0](http://blast.ncbi.nlm.nih.gov/Blast.cgi#224809474%23224809474) | 18.0 | 71% | 453 |
| [XP_001717293.1](http://www.ncbi.nlm.nih.gov/entrez/query.fcgi?cmd=Retrieve&db=Protein&list_uids=169202578&dopt=GenPept&RID=T2GZEE68012&log$=prottop&blast_rank=21) | PREDICTED: hypothetical protein [Homo sapiens] >ref|XP_001717790.1| PREDICTED: hypothetical protein [Homo sapiens] | [18.0](http://blast.ncbi.nlm.nih.gov/Blast.cgi#169202578%23169202578) | 18.0 | 100% | 453 |
| [NP_078858.4](http://www.ncbi.nlm.nih.gov/entrez/query.fcgi?cmd=Retrieve&db=Protein&list_uids=165932370&dopt=GenPept&RID=T2GZEE68012&log$=prottop&blast_rank=22) | FAT tumor suppressor homolog 4 precursor [Homo sapiens] | [18.0](http://blast.ncbi.nlm.nih.gov/Blast.cgi#165932370%23165932370) | 18.0 | 85% | 453 |
| [NP_001087194.1](http://www.ncbi.nlm.nih.gov/entrez/query.fcgi?cmd=Retrieve&db=Protein&list_uids=147902746&dopt=GenPept&RID=T2GZEE68012&log$=prottop&blast_rank=23) | MEX3A protein [Homo sapiens] | [18.0](http://blast.ncbi.nlm.nih.gov/Blast.cgi#147902746%23147902746) | 18.0 | 71% | 453 |
| [NP_874369.1](http://www.ncbi.nlm.nih.gov/entrez/query.fcgi?cmd=Retrieve&db=Protein&list_uids=36287069&dopt=GenPept&RID=T2GZEE68012&log$=prottop&blast_rank=24) | K(lysine) acetyltransferase 5 isoform 1 [Homo sapiens] | [18.0](http://blast.ncbi.nlm.nih.gov/Blast.cgi#36287069%2336287069) | 30.1 | 85% | 453 |
| [NP_056392.2](http://www.ncbi.nlm.nih.gov/entrez/query.fcgi?cmd=Retrieve&db=Protein&list_uids=224809468&dopt=GenPept&RID=T2GZEE68012&log$=prottop&blast_rank=25) | retinoic acid induced 14 isoform a [Homo sapiens] >ref|NP_001138992.1| retinoic acid induced 14 isoform a [Homo sapiens] >ref|NP_001138993.1| retinoic acid induced 14 isoform a [Homo sapiens] | [18.0](http://blast.ncbi.nlm.nih.gov/Blast.cgi#224809468%23224809468) | 18.0 | 71% | 453 |
| [NP_079410.4](http://www.ncbi.nlm.nih.gov/entrez/query.fcgi?cmd=Retrieve&db=Protein&list_uids=95147342&dopt=GenPept&RID=T2GZEE68012&log$=prottop&blast_rank=26) | chromodomain helicase DNA binding protein 9 [Homo sapiens] | [18.0](http://blast.ncbi.nlm.nih.gov/Blast.cgi#95147342%2395147342) | 32.2 | 100% | 453 |
| [NP_001032209.1](http://www.ncbi.nlm.nih.gov/entrez/query.fcgi?cmd=Retrieve&db=Protein&list_uids=81158226&dopt=GenPept&RID=T2GZEE68012&log$=prottop&blast_rank=27) | neuronal cell adhesion molecule isoform A precursor [Homo sapiens] | [18.0](http://blast.ncbi.nlm.nih.gov/Blast.cgi#81158226%2381158226) | 18.0 | 85% | 453 |
| [NP_005001.3](http://www.ncbi.nlm.nih.gov/entrez/query.fcgi?cmd=Retrieve&db=Protein&list_uids=81158224&dopt=GenPept&RID=T2GZEE68012&log$=prottop&blast_rank=28) | neuronal cell adhesion molecule isoform B precursor [Homo sapiens] | [18.0](http://blast.ncbi.nlm.nih.gov/Blast.cgi#81158224%2381158224) | 18.0 | 85% | 453 |
| [NP_004662.2](http://www.ncbi.nlm.nih.gov/entrez/query.fcgi?cmd=Retrieve&db=Protein&list_uids=56699458&dopt=GenPept&RID=T2GZEE68012&log$=prottop&blast_rank=29) | protein inhibitor of activated STAT X isoform beta [Homo sapiens] | [18.0](http://blast.ncbi.nlm.nih.gov/Blast.cgi#56699458%2356699458) | 18.0 | 71% | 453 |
| [NP_001138997.1](http://www.ncbi.nlm.nih.gov/entrez/query.fcgi?cmd=Retrieve&db=Protein&list_uids=224809478&dopt=GenPept&RID=T2GZEE68012&log$=prottop&blast_rank=30) | retinoic acid induced 14 isoform d [Homo sapiens] | [18.0](http://blast.ncbi.nlm.nih.gov/Blast.cgi#224809478%23224809478) | 18.0 | 71% | 453 |
| [NP_073589.4](http://www.ncbi.nlm.nih.gov/entrez/query.fcgi?cmd=Retrieve&db=Protein&list_uids=154744870&dopt=GenPept&RID=T2GZEE68012&log$=prottop&blast_rank=31) | zinc finger protein 574 [Homo sapiens] | [18.0](http://blast.ncbi.nlm.nih.gov/Blast.cgi#154744870%23154744870) | 18.0 | 85% | 453 |
| [NP_699193.2](http://www.ncbi.nlm.nih.gov/entrez/query.fcgi?cmd=Retrieve&db=Protein&list_uids=281427284&dopt=GenPept&RID=T2GZEE68012&log$=prottop&blast_rank=32) | inactive serine protease 35 precursor [Homo sapiens] >ref|NP_001163894.1| inactive serine protease 35 precursor [Homo sapiens] | [18.0](http://blast.ncbi.nlm.nih.gov/Blast.cgi#281427284%23281427284) | 46.9 | 85% | 453 |
| [NP_775298.1](http://www.ncbi.nlm.nih.gov/entrez/query.fcgi?cmd=Retrieve&db=Protein&list_uids=27532984&dopt=GenPept&RID=T2GZEE68012&log$=prottop&blast_rank=33) | protein inhibitor of activated STAT X isoform alpha [Homo sapiens] | [18.0](http://blast.ncbi.nlm.nih.gov/Blast.cgi#27532984%2327532984) | 18.0 | 71% | 453 |
| [NP_899068.1](http://www.ncbi.nlm.nih.gov/entrez/query.fcgi?cmd=Retrieve&db=Protein&list_uids=34304379&dopt=GenPept&RID=T2GZEE68012&log$=prottop&blast_rank=34) | inversin isoform b [Homo sapiens] | [18.0](http://blast.ncbi.nlm.nih.gov/Blast.cgi#34304379%2334304379) | 18.0 | 71% | 453 |
| [NP_055240.2](http://www.ncbi.nlm.nih.gov/entrez/query.fcgi?cmd=Retrieve&db=Protein&list_uids=34304381&dopt=GenPept&RID=T2GZEE68012&log$=prottop&blast_rank=35) | inversin isoform a [Homo sapiens] | [18.0](http://blast.ncbi.nlm.nih.gov/Blast.cgi#34304381%2334304381) | 18.0 | 71% | 453 |
| [NP_874368.1](http://www.ncbi.nlm.nih.gov/entrez/query.fcgi?cmd=Retrieve&db=Protein&list_uids=36287060&dopt=GenPept&RID=T2GZEE68012&log$=prottop&blast_rank=36) | K(lysine) acetyltransferase 5 isoform 3 [Homo sapiens] | [18.0](http://blast.ncbi.nlm.nih.gov/Blast.cgi#36287060%2336287060) | 30.1 | 85% | 453 |
| [NP_006090.2](http://www.ncbi.nlm.nih.gov/entrez/query.fcgi?cmd=Retrieve&db=Protein&list_uids=115298686&dopt=GenPept&RID=T2GZEE68012&log$=prottop&blast_rank=37) | protein inhibitor of activated STAT, 3 [Homo sapiens] | [18.0](http://blast.ncbi.nlm.nih.gov/Blast.cgi#115298686%23115298686) | 18.0 | 71% | 453 |
| [NP_078976.2](http://www.ncbi.nlm.nih.gov/entrez/query.fcgi?cmd=Retrieve&db=Protein&list_uids=21314720&dopt=GenPept&RID=T2GZEE68012&log$=prottop&blast_rank=38) | Smad nuclear interacting protein [Homo sapiens] | [18.0](http://blast.ncbi.nlm.nih.gov/Blast.cgi#21314720%2321314720) | 18.0 | 85% | 453 |
| [NP_006379.2](http://www.ncbi.nlm.nih.gov/entrez/query.fcgi?cmd=Retrieve&db=Protein&list_uids=36287049&dopt=GenPept&RID=T2GZEE68012&log$=prottop&blast_rank=39) | K(lysine) acetyltransferase 5 isoform 2 [Homo sapiens] | [18.0](http://blast.ncbi.nlm.nih.gov/Blast.cgi#36287049%2336287049) | 30.1 | 85% | 453 |
| [NP_891847.1](http://www.ncbi.nlm.nih.gov/entrez/query.fcgi?cmd=Retrieve&db=Protein&list_uids=33636768&dopt=GenPept&RID=T2GZEE68012&log$=prottop&blast_rank=40) | myeloid/lymphoid or mixed-lineage leukemia 5 [Homo sapiens] >ref|NP_061152.3| myeloid/lymphoid or mixed-lineage leukemia 5 [Homo sapiens] | [17.6](http://blast.ncbi.nlm.nih.gov/Blast.cgi#33636768%2333636768) | 17.6 | 85% | 608 |
| [NP_775773.2](http://www.ncbi.nlm.nih.gov/entrez/query.fcgi?cmd=Retrieve&db=Protein&list_uids=58218970&dopt=GenPept&RID=T2GZEE68012&log$=prottop&blast_rank=41) | protease, serine, 36 precursor [Homo sapiens] | [17.6](http://blast.ncbi.nlm.nih.gov/Blast.cgi#58218970%2358218970) | 17.6 | 100% | 608 |
| [NP_060413.2](http://www.ncbi.nlm.nih.gov/entrez/query.fcgi?cmd=Retrieve&db=Protein&list_uids=40807493&dopt=GenPept&RID=T2GZEE68012&log$=prottop&blast_rank=42) | F-box only protein 34 [Homo sapiens] >ref|NP_689417.1| F-box only protein 34 [Homo sapiens] | [17.6](http://blast.ncbi.nlm.nih.gov/Blast.cgi#40807493%2340807493) | 17.6 | 85% | 608 |
| [XP_002344946.1](http://www.ncbi.nlm.nih.gov/entrez/query.fcgi?cmd=Retrieve&db=Protein&list_uids=239756597&dopt=GenPept&RID=T2GZEE68012&log$=prottop&blast_rank=43) | PREDICTED: hypothetical protein [Homo sapiens] | [17.2](http://blast.ncbi.nlm.nih.gov/Blast.cgi#239756597%23239756597) | 17.2 | 100% | 816 |
| [NP_001129947.1](http://www.ncbi.nlm.nih.gov/entrez/query.fcgi?cmd=Retrieve&db=Protein&list_uids=210147527&dopt=GenPept&RID=T2GZEE68012&log$=prottop&blast_rank=44) | vasohibin 2 isoform 3 [Homo sapiens] | [17.2](http://blast.ncbi.nlm.nih.gov/Blast.cgi#210147527%23210147527) | 17.2 | 85% | 816 |
| [NP_001156806.1](http://www.ncbi.nlm.nih.gov/entrez/query.fcgi?cmd=Retrieve&db=Protein&list_uids=254039648&dopt=GenPept&RID=T2GZEE68012&log$=prottop&blast_rank=45) | synaptotagmin-like 5 isoform 2 [Homo sapiens] | [17.2](http://blast.ncbi.nlm.nih.gov/Blast.cgi#254039648%23254039648) | 17.2 | 85% | 816 |
| [NP_997644.2](http://www.ncbi.nlm.nih.gov/entrez/query.fcgi?cmd=Retrieve&db=Protein&list_uids=238814387&dopt=GenPept&RID=T2GZEE68012&log$=prottop&blast_rank=46) | zinc finger, MYND domain containing 11 isoform b [Homo sapiens] | [17.2](http://blast.ncbi.nlm.nih.gov/Blast.cgi#238814387%23238814387) | 17.2 | 85% | 816 |
| [NP_071396.4](http://www.ncbi.nlm.nih.gov/entrez/query.fcgi?cmd=Retrieve&db=Protein&list_uids=157738621&dopt=GenPept&RID=T2GZEE68012&log$=prottop&blast_rank=47) | kinesin family member 13A isoform a [Homo sapiens] | [17.2](http://blast.ncbi.nlm.nih.gov/Blast.cgi#157738621%23157738621) | 17.2 | 85% | 816 |
| [NP_001099036.1](http://www.ncbi.nlm.nih.gov/entrez/query.fcgi?cmd=Retrieve&db=Protein&list_uids=157738625&dopt=GenPept&RID=T2GZEE68012&log$=prottop&blast_rank=48) | kinesin family member 13A isoform b [Homo sapiens] | [17.2](http://blast.ncbi.nlm.nih.gov/Blast.cgi#157738625%23157738625) | 17.2 | 85% | 816 |
| [NP_919301.2](http://www.ncbi.nlm.nih.gov/entrez/query.fcgi?cmd=Retrieve&db=Protein&list_uids=110815807&dopt=GenPept&RID=T2GZEE68012&log$=prottop&blast_rank=49) | zinc finger protein 169 [Homo sapiens] | [17.2](http://blast.ncbi.nlm.nih.gov/Blast.cgi#110815807%23110815807) | 17.2 | 85% | 816 |
| [NP_006615.2](http://www.ncbi.nlm.nih.gov/entrez/query.fcgi?cmd=Retrieve&db=Protein&list_uids=238814385&dopt=GenPept&RID=T2GZEE68012&log$=prottop&blast_rank=50) | zinc finger, MYND domain containing 11 isoform a [Homo sapiens] >ref|NP_001154954.1| zinc finger, MYND domain containing 11 isoform a [Homo sapiens] | [17.2](http://blast.ncbi.nlm.nih.gov/Blast.cgi#238814385%23238814385) | 17.2 | 85% | 816 |
| [NP_001120870.1](http://www.ncbi.nlm.nih.gov/entrez/query.fcgi?cmd=Retrieve&db=Protein&list_uids=188528698&dopt=GenPept&RID=T2GZEE68012&log$=prottop&blast_rank=51) | erlectin isoform 3 [Homo sapiens] | [17.2](http://blast.ncbi.nlm.nih.gov/Blast.cgi#188528698%23188528698) | 17.2 | 85% | 816 |
| [NP_000453.2](http://www.ncbi.nlm.nih.gov/entrez/query.fcgi?cmd=Retrieve&db=Protein&list_uids=19718766&dopt=GenPept&RID=T2GZEE68012&log$=prottop&blast_rank=52) | ubiquitin protein ligase E3A isoform 2 [Homo sapiens] | [17.2](http://blast.ncbi.nlm.nih.gov/Blast.cgi#19718766%2319718766) | 17.2 | 85% | 816 |
| [NP_001099037.1](http://www.ncbi.nlm.nih.gov/entrez/query.fcgi?cmd=Retrieve&db=Protein&list_uids=157738627&dopt=GenPept&RID=T2GZEE68012&log$=prottop&blast_rank=53) | kinesin family member 13A isoform c [Homo sapiens] | [17.2](http://blast.ncbi.nlm.nih.gov/Blast.cgi#157738627%23157738627) | 17.2 | 85% | 816 |
| [NP_001129946.1](http://www.ncbi.nlm.nih.gov/entrez/query.fcgi?cmd=Retrieve&db=Protein&list_uids=210147525&dopt=GenPept&RID=T2GZEE68012&log$=prottop&blast_rank=54) | vasohibin 2 isoform 2 [Homo sapiens] | [17.2](http://blast.ncbi.nlm.nih.gov/Blast.cgi#210147525%23210147525) | 17.2 | 85% | 816 |
| [NP_570854.1](http://www.ncbi.nlm.nih.gov/entrez/query.fcgi?cmd=Retrieve&db=Protein&list_uids=19718764&dopt=GenPept&RID=T2GZEE68012&log$=prottop&blast_rank=55) | ubiquitin protein ligase E3A isoform 3 [Homo sapiens] | [17.2](http://blast.ncbi.nlm.nih.gov/Blast.cgi#19718764%2319718764) | 17.2 | 85% | 816 |
| [NP_001099038.1](http://www.ncbi.nlm.nih.gov/entrez/query.fcgi?cmd=Retrieve&db=Protein&list_uids=157738629&dopt=GenPept&RID=T2GZEE68012&log$=prottop&blast_rank=56) | kinesin family member 13A isoform d [Homo sapiens] | [17.2](http://blast.ncbi.nlm.nih.gov/Blast.cgi#157738629%23157738629) | 17.2 | 85% | 816 |
| [NP_079025.2](http://www.ncbi.nlm.nih.gov/entrez/query.fcgi?cmd=Retrieve&db=Protein&list_uids=31982921&dopt=GenPept&RID=T2GZEE68012&log$=prottop&blast_rank=57) | vasohibin 2 isoform 1 [Homo sapiens] | [17.2](http://blast.ncbi.nlm.nih.gov/Blast.cgi#31982921%2331982921) | 31.8 | 100% | 816 |
| [NP_570853.1](http://www.ncbi.nlm.nih.gov/entrez/query.fcgi?cmd=Retrieve&db=Protein&list_uids=19718762&dopt=GenPept&RID=T2GZEE68012&log$=prottop&blast_rank=58) | ubiquitin protein ligase E3A isoform 1 [Homo sapiens] | [17.2](http://blast.ncbi.nlm.nih.gov/Blast.cgi#19718762%2319718762) | 17.2 | 85% | 816 |
| [NP_116011.2](http://www.ncbi.nlm.nih.gov/entrez/query.fcgi?cmd=Retrieve&db=Protein&list_uids=187607071&dopt=GenPept&RID=T2GZEE68012&log$=prottop&blast_rank=59) | ligand of numb-protein X 1 isoform b [Homo sapiens] | [17.2](http://blast.ncbi.nlm.nih.gov/Blast.cgi#187607071%23187607071) | 17.2 | 85% | 816 |
| [NP_115901.2](http://www.ncbi.nlm.nih.gov/entrez/query.fcgi?cmd=Retrieve&db=Protein&list_uids=46559761&dopt=GenPept&RID=T2GZEE68012&log$=prottop&blast_rank=60) | PDZ domain containing 4 [Homo sapiens] | [17.2](http://blast.ncbi.nlm.nih.gov/Blast.cgi#46559761%2346559761) | 17.2 | 100% | 816 |
| [NP_001119800.1](http://www.ncbi.nlm.nih.gov/entrez/query.fcgi?cmd=Retrieve&db=Protein&list_uids=187607085&dopt=GenPept&RID=T2GZEE68012&log$=prottop&blast_rank=61) | ligand of numb-protein X 1 isoform a [Homo sapiens] | [17.2](http://blast.ncbi.nlm.nih.gov/Blast.cgi#187607085%23187607085) | 17.2 | 85% | 816 |
| [NP_620135.1](http://www.ncbi.nlm.nih.gov/entrez/query.fcgi?cmd=Retrieve&db=Protein&list_uids=20270305&dopt=GenPept&RID=T2GZEE68012&log$=prottop&blast_rank=62) | synaptotagmin-like 5 isoform 1 [Homo sapiens] >ref|NP_001156807.1| synaptotagmin-like 5 isoform 1 [Homo sapiens] | [17.2](http://blast.ncbi.nlm.nih.gov/Blast.cgi#20270305%2320270305) | 17.2 | 85% | 816 |
| [XP_002348003.1](http://www.ncbi.nlm.nih.gov/entrez/query.fcgi?cmd=Retrieve&db=Protein&list_uids=239751791&dopt=GenPept&RID=T2GZEE68012&log$=prottop&blast_rank=63) | PREDICTED: hypothetical protein [Homo sapiens] | [16.8](http://blast.ncbi.nlm.nih.gov/Blast.cgi#239751791%23239751791) | 27.6 | 100% | 1095 |
| [XP_002343723.1](http://www.ncbi.nlm.nih.gov/entrez/query.fcgi?cmd=Retrieve&db=Protein&list_uids=239746282&dopt=GenPept&RID=T2GZEE68012&log$=prottop&blast_rank=64) | PREDICTED: hypothetical protein XP_002343723 [Homo sapiens] | [16.8](http://blast.ncbi.nlm.nih.gov/Blast.cgi#239746282%23239746282) | 27.6 | 100% | 1095 |
| [NP_065979.1](http://www.ncbi.nlm.nih.gov/entrez/query.fcgi?cmd=Retrieve&db=Protein&list_uids=210147522&dopt=GenPept&RID=T2GZEE68012&log$=prottop&blast_rank=65) | zinc finger, SWIM-type containing 6 [Homo sapiens] | [16.8](http://blast.ncbi.nlm.nih.gov/Blast.cgi#210147522%23210147522) | 16.8 | 100% | 1095 |
| [NP_001130033.2](http://www.ncbi.nlm.nih.gov/entrez/query.fcgi?cmd=Retrieve&db=Protein&list_uids=282396082&dopt=GenPept&RID=T2GZEE68012&log$=prottop&blast_rank=66) | heterogeneous nuclear ribonucleoprotein C-like [Homo sapiens] | [16.8](http://blast.ncbi.nlm.nih.gov/Blast.cgi#282396082%23282396082) | 16.8 | 85% | 1095 |
| [NP_001139653.1](http://www.ncbi.nlm.nih.gov/entrez/query.fcgi?cmd=Retrieve&db=Protein&list_uids=226052117&dopt=GenPept&RID=T2GZEE68012&log$=prottop&blast_rank=67) | heterogeneous nuclear ribonucleoprotein C-like [Homo sapiens] >ref|XP_001719866.2| PREDICTED: similar to heterogeneous nuclear ribonucleoprotein C-like [Homo sapiens] >ref|XP_002346319.1| PREDICTED: heterogeneous nuclear ribonucleoprotein C-like [Homo sapiens] | [16.8](http://blast.ncbi.nlm.nih.gov/Blast.cgi#226052117%23226052117) | 16.8 | 85% | 1095 |
| [NP_112604.2](http://www.ncbi.nlm.nih.gov/entrez/query.fcgi?cmd=Retrieve&db=Protein&list_uids=117189975&dopt=GenPept&RID=T2GZEE68012&log$=prottop&blast_rank=68) | heterogeneous nuclear ribonucleoprotein C isoform a [Homo sapiens] >ref|NP_001070910.1| heterogeneous nuclear ribonucleoprotein C isoform a [Homo sapiens] | [16.8](http://blast.ncbi.nlm.nih.gov/Blast.cgi#117189975%23117189975) | 16.8 | 85% | 1095 |
| [NP_001161054.1](http://www.ncbi.nlm.nih.gov/entrez/query.fcgi?cmd=Retrieve&db=Protein&list_uids=262399389&dopt=GenPept&RID=T2GZEE68012&log$=prottop&blast_rank=69) | RNA binding motif protein 6 isoform 2 [Homo sapiens] | [16.8](http://blast.ncbi.nlm.nih.gov/Blast.cgi#262399389%23262399389) | 16.8 | 85% | 1095 |
| [NP_004491.2](http://www.ncbi.nlm.nih.gov/entrez/query.fcgi?cmd=Retrieve&db=Protein&list_uids=117190174&dopt=GenPept&RID=T2GZEE68012&log$=prottop&blast_rank=70) | heterogeneous nuclear ribonucleoprotein C isoform b [Homo sapiens] >ref|NP_001070911.1| heterogeneous nuclear ribonucleoprotein C isoform b [Homo sapiens] | [16.8](http://blast.ncbi.nlm.nih.gov/Blast.cgi#117190174%23117190174) | 16.8 | 85% | 1095 |
| [NP_005768.1](http://www.ncbi.nlm.nih.gov/entrez/query.fcgi?cmd=Retrieve&db=Protein&list_uids=5032033&dopt=GenPept&RID=T2GZEE68012&log$=prottop&blast_rank=71) | RNA binding motif protein 6 isoform 1 [Homo sapiens] | [16.8](http://blast.ncbi.nlm.nih.gov/Blast.cgi#5032033%235032033) | 16.8 | 85% | 1095 |
| [NP_005009.2](http://www.ncbi.nlm.nih.gov/entrez/query.fcgi?cmd=Retrieve&db=Protein&list_uids=167857792&dopt=GenPept&RID=T2GZEE68012&log$=prottop&blast_rank=72) | programmed cell death 1 precursor [Homo sapiens] | [16.8](http://blast.ncbi.nlm.nih.gov/Blast.cgi#167857792%23167857792) | 16.8 | 85% | 1095 |
| [NP_064612.2](http://www.ncbi.nlm.nih.gov/entrez/query.fcgi?cmd=Retrieve&db=Protein&list_uids=147905620&dopt=GenPept&RID=T2GZEE68012&log$=prottop&blast_rank=73) | PR domain containing 9 [Homo sapiens] | [16.8](http://blast.ncbi.nlm.nih.gov/Blast.cgi#147905620%23147905620) | 16.8 | 85% | 1095 |
| [NP_940967.1](http://www.ncbi.nlm.nih.gov/entrez/query.fcgi?cmd=Retrieve&db=Protein&list_uids=38348406&dopt=GenPept&RID=T2GZEE68012&log$=prottop&blast_rank=74) | leucine rich repeat containing 33 precursor [Homo sapiens] | [16.8](http://blast.ncbi.nlm.nih.gov/Blast.cgi#38348406%2338348406) | 16.8 | 100% | 1095 |
| [NP_000930.1](http://www.ncbi.nlm.nih.gov/entrez/query.fcgi?cmd=Retrieve&db=Protein&list_uids=4505949&dopt=GenPept&RID=T2GZEE68012&log$=prottop&blast_rank=75) | proopiomelanocortin preproprotein [Homo sapiens] >ref|NP_001030333.1| proopiomelanocortin preproprotein [Homo sapiens] | [16.8](http://blast.ncbi.nlm.nih.gov/Blast.cgi#4505949%234505949) | 16.8 | 85% | 1095 |
| [NP_001013653.1](http://www.ncbi.nlm.nih.gov/entrez/query.fcgi?cmd=Retrieve&db=Protein&list_uids=61966711&dopt=GenPept&RID=T2GZEE68012&log$=prottop&blast_rank=76) | heterogeneous nuclear ribonucleoprotein C-like 1 [Homo sapiens] | [16.8](http://blast.ncbi.nlm.nih.gov/Blast.cgi#61966711%2361966711) | 16.8 | 85% | 1095 |
| [NP_079147.1](http://www.ncbi.nlm.nih.gov/entrez/query.fcgi?cmd=Retrieve&db=Protein&list_uids=13376306&dopt=GenPept&RID=T2GZEE68012&log$=prottop&blast_rank=77) | MAP6 domain containing 1 [Homo sapiens] | [16.8](http://blast.ncbi.nlm.nih.gov/Blast.cgi#13376306%2313376306) | 16.8 | 85% | 1095 |
| [NP_542399.1](http://www.ncbi.nlm.nih.gov/entrez/query.fcgi?cmd=Retrieve&db=Protein&list_uids=18087845&dopt=GenPept&RID=T2GZEE68012&log$=prottop&blast_rank=78) | cell division cycle associated 5 [Homo sapiens] | [16.8](http://blast.ncbi.nlm.nih.gov/Blast.cgi#18087845%2318087845) | 16.8 | 100% | 1095 |
| [NP_001157968.1](http://www.ncbi.nlm.nih.gov/entrez/query.fcgi?cmd=Retrieve&db=Protein&list_uids=256818774&dopt=GenPept&RID=T2GZEE68012&log$=prottop&blast_rank=79) | WD repeat domain 52 isoform 1 [Homo sapiens] | [16.3](http://blast.ncbi.nlm.nih.gov/Blast.cgi#256818774%23256818774) | 16.3 | 57% | 1469 |
| [NP_001154912.1](http://www.ncbi.nlm.nih.gov/entrez/query.fcgi?cmd=Retrieve&db=Protein&list_uids=241896926&dopt=GenPept&RID=T2GZEE68012&log$=prottop&blast_rank=80) | protein tyrosine phosphatase, receptor type, H isoform 2 precursor [Homo sapiens] | [16.3](http://blast.ncbi.nlm.nih.gov/Blast.cgi#241896926%23241896926) | 16.3 | 57% | 1469 |
| [XP_002345044.1](http://www.ncbi.nlm.nih.gov/entrez/query.fcgi?cmd=Retrieve&db=Protein&list_uids=239756746&dopt=GenPept&RID=T2GZEE68012&log$=prottop&blast_rank=81) | PREDICTED: TBC1 domain family, member 27 [Homo sapiens] | [16.3](http://blast.ncbi.nlm.nih.gov/Blast.cgi#239756746%23239756746) | 16.3 | 57% | 1469 |
| [XP_002348268.1](http://www.ncbi.nlm.nih.gov/entrez/query.fcgi?cmd=Retrieve&db=Protein&list_uids=239752427&dopt=GenPept&RID=T2GZEE68012&log$=prottop&blast_rank=82) | PREDICTED: SAGE1-like protein-like [Homo sapiens] >ref|XP_002346377.1| PREDICTED: SAGE1-like protein-like [Homo sapiens] | [16.3](http://blast.ncbi.nlm.nih.gov/Blast.cgi#239752427%23239752427) | 16.3 | 57% | 1469 |
| [XP_002347765.1](http://www.ncbi.nlm.nih.gov/entrez/query.fcgi?cmd=Retrieve&db=Protein&list_uids=239751241&dopt=GenPept&RID=T2GZEE68012&log$=prottop&blast_rank=83) | PREDICTED: TBC1 domain family, member 27 [Homo sapiens] | [16.3](http://blast.ncbi.nlm.nih.gov/Blast.cgi#239751241%23239751241) | 16.3 | 57% | 1469 |
| [XP_002343891.1](http://www.ncbi.nlm.nih.gov/entrez/query.fcgi?cmd=Retrieve&db=Protein&list_uids=239746945&dopt=GenPept&RID=T2GZEE68012&log$=prottop&blast_rank=84) | PREDICTED: SAGE1-like protein-like [Homo sapiens] | [16.3](http://blast.ncbi.nlm.nih.gov/Blast.cgi#239746945%23239746945) | 16.3 | 57% | 1469 |
| [XP_002343522.1](http://www.ncbi.nlm.nih.gov/entrez/query.fcgi?cmd=Retrieve&db=Protein&list_uids=239745731&dopt=GenPept&RID=T2GZEE68012&log$=prottop&blast_rank=85) | PREDICTED: TBC1 domain family, member 27 [Homo sapiens] | [16.3](http://blast.ncbi.nlm.nih.gov/Blast.cgi#239745731%23239745731) | 16.3 | 57% | 1469 |
| [XP_002343409.1](http://www.ncbi.nlm.nih.gov/entrez/query.fcgi?cmd=Retrieve&db=Protein&list_uids=239745211&dopt=GenPept&RID=T2GZEE68012&log$=prottop&blast_rank=86) | PREDICTED: hypothetical protein XP_002343409 [Homo sapiens] | [16.3](http://blast.ncbi.nlm.nih.gov/Blast.cgi#239745211%23239745211) | 16.3 | 57% | 1469 |
| [XP_001719373.2](http://www.ncbi.nlm.nih.gov/entrez/query.fcgi?cmd=Retrieve&db=Protein&list_uids=239741422&dopt=GenPept&RID=T2GZEE68012&log$=prottop&blast_rank=87) | PREDICTED: achaete-scute complex homolog 5 [Homo sapiens] >ref|XP_946059.4| PREDICTED: achaete-scute complex homolog 5 (Drosophila) [Homo sapiens] >ref|XP_001718279.2| PREDICTED: achaete-scute complex homolog 5 (Drosophila) [Homo sapiens] | [16.3](http://blast.ncbi.nlm.nih.gov/Blast.cgi#239741422%23239741422) | 16.3 | 57% | 1469 |
| [NP_001158018.1](http://www.ncbi.nlm.nih.gov/entrez/query.fcgi?cmd=Retrieve&db=Protein&list_uids=257153476&dopt=GenPept&RID=T2GZEE68012&log$=prottop&blast_rank=88) | disrupted in schizophrenia 1 isoform i [Homo sapiens] >ref|NP_001158019.1| disrupted in schizophrenia 1 isoform j [Homo sapiens] | [16.3](http://blast.ncbi.nlm.nih.gov/Blast.cgi#257153476%23257153476) | 16.3 | 57% | 1469 |
| [NP_001153891.1](http://www.ncbi.nlm.nih.gov/entrez/query.fcgi?cmd=Retrieve&db=Protein&list_uids=238550212&dopt=GenPept&RID=T2GZEE68012&log$=prottop&blast_rank=89) | Z-DNA binding protein 1 isoform d [Homo sapiens] | [16.3](http://blast.ncbi.nlm.nih.gov/Blast.cgi#238550212%23238550212) | 16.3 | 85% | 1469 |
| [NP_001153889.1](http://www.ncbi.nlm.nih.gov/entrez/query.fcgi?cmd=Retrieve&db=Protein&list_uids=238550208&dopt=GenPept&RID=T2GZEE68012&log$=prottop&blast_rank=90) | Z-DNA binding protein 1 isoform b [Homo sapiens] | [16.3](http://blast.ncbi.nlm.nih.gov/Blast.cgi#238550208%23238550208) | 16.3 | 85% | 1469 |
| [NP_001153476.1](http://www.ncbi.nlm.nih.gov/entrez/query.fcgi?cmd=Retrieve&db=Protein&list_uids=236463556&dopt=GenPept&RID=T2GZEE68012&log$=prottop&blast_rank=91) | neuregulin 1 isoform ndf43b [Homo sapiens] | [16.3](http://blast.ncbi.nlm.nih.gov/Blast.cgi#236463556%23236463556) | 16.3 | 57% | 1469 |
| [NP_001153468.1](http://www.ncbi.nlm.nih.gov/entrez/query.fcgi?cmd=Retrieve&db=Protein&list_uids=236461846&dopt=GenPept&RID=T2GZEE68012&log$=prottop&blast_rank=92) | neuregulin 1 isoform ndf43c [Homo sapiens] | [16.3](http://blast.ncbi.nlm.nih.gov/Blast.cgi#236461846%23236461846) | 16.3 | 57% | 1469 |
| [NP_001135975.1](http://www.ncbi.nlm.nih.gov/entrez/query.fcgi?cmd=Retrieve&db=Protein&list_uids=215820650&dopt=GenPept&RID=T2GZEE68012&log$=prottop&blast_rank=93) | StAR-related lipid transfer (START) domain containing 8 isoform a [Homo sapiens] | [16.3](http://blast.ncbi.nlm.nih.gov/Blast.cgi#215820650%23215820650) | 16.3 | 57% | 1469 |
| [NP_056056.2](http://www.ncbi.nlm.nih.gov/entrez/query.fcgi?cmd=Retrieve&db=Protein&list_uids=209862789&dopt=GenPept&RID=T2GZEE68012&log$=prottop&blast_rank=94) | microtubule associated monoxygenase, calponin and LIM domain containing 3 isoform 1 [Homo sapiens] | [16.3](http://blast.ncbi.nlm.nih.gov/Blast.cgi#209862789%23209862789) | 32.2 | 71% | 1469 |
| [NP_001009877.2](http://www.ncbi.nlm.nih.gov/entrez/query.fcgi?cmd=Retrieve&db=Protein&list_uids=242247075&dopt=GenPept&RID=T2GZEE68012&log$=prottop&blast_rank=95) | bromodomain containing 9 isoform 2 [Homo sapiens] | [16.3](http://blast.ncbi.nlm.nih.gov/Blast.cgi#242247075%23242247075) | 16.3 | 57% | 1469 |
| [NP_076413.3](http://www.ncbi.nlm.nih.gov/entrez/query.fcgi?cmd=Retrieve&db=Protein&list_uids=237649104&dopt=GenPept&RID=T2GZEE68012&log$=prottop&blast_rank=96) | bromodomain containing 9 isoform 1 [Homo sapiens] | [16.3](http://blast.ncbi.nlm.nih.gov/Blast.cgi#237649104%23237649104) | 16.3 | 57% | 1469 |
| [NP_003604.3](http://www.ncbi.nlm.nih.gov/entrez/query.fcgi?cmd=Retrieve&db=Protein&list_uids=192449445&dopt=GenPept&RID=T2GZEE68012&log$=prottop&blast_rank=97) | cartilage intermediate layer protein [Homo sapiens] | [16.3](http://blast.ncbi.nlm.nih.gov/Blast.cgi#192449445%23192449445) | 16.3 | 57% | 1469 |
| [NP_001122089.1](http://www.ncbi.nlm.nih.gov/entrez/query.fcgi?cmd=Retrieve&db=Protein&list_uids=190886437&dopt=GenPept&RID=T2GZEE68012&log$=prottop&blast_rank=98) | RALBP1 associated Eps domain containing 1 isoform b [Homo sapiens] | [16.3](http://blast.ncbi.nlm.nih.gov/Blast.cgi#190886437%23190886437) | 28.8 | 71% | 1469 |
| [NP_001120869.1](http://www.ncbi.nlm.nih.gov/entrez/query.fcgi?cmd=Retrieve&db=Protein&list_uids=188528696&dopt=GenPept&RID=T2GZEE68012&log$=prottop&blast_rank=99) | erlectin isoform 2 [Homo sapiens] | [16.3](http://blast.ncbi.nlm.nih.gov/Blast.cgi#188528696%23188528696) | 16.3 | 57% | 1469 |
| [NP_001153890.1](http://www.ncbi.nlm.nih.gov/entrez/query.fcgi?cmd=Retrieve&db=Protein&list_uids=238550210&dopt=GenPept&RID=T2GZEE68012&log$=prottop&blast_rank=100) | Z-DNA binding protein 1 isoform c [Homo sapiens] | [16.3](http://blast.ncbi.nlm.nih.gov/Blast.cgi#238550210%23238550210) | 16.3 | 85% | 1469 |
