# Supplementary material for: A high throughput method for identifying personalized tumor-associated antigens
Source: Oncotarget. 2010 Jun 27;1(2):148–55. doi: 10.18632/oncotarget.118 (PMC2920534; doi:10.18632/oncotarget.118)
Supplement: Supplemental Table 5 [file oncotarget-01-148-s005.doc]

**IgG Patient D**

| **Accession** | **Proteins with a match to VPWSKPWWTQ peptide** | **[Max score](http://blast.ncbi.nlm.nih.gov/Blast.cgi?CMD=Get&ALIGNMENTS=100&ALIGNMENT_VIEW=Pairwise&CDD_SEARCH_STATE=1&DATABASE_SORT=0&DESCRIPTIONS=100&ENTREZ_QUERY=txid9606 %5BORGN%5D&FIRST_QUERY_NUM=0&FORMAT_OBJECT=Alignment&FORMAT_PAGE_TARGET=&FORMAT_TYPE=HTML&GET_SEQUENCE=yes&I_THRESH=&MASK_CHAR=2&MASK_COLOR=1&NEW_DESIGN=on&NEW_VIEW=yes&NUM_OVERVIEW=100&OLD_BLAST=false&PAGE=Proteins&QUERY_INDEX=0&QUERY_NUMBER=0&RESULTS_PAGE_TARGET=&RID=T2HZ6M4N016&SHOW_LINKOUT=yes&SHOW_OVERVIEW=yes&STEP_NUMBER=&WORD_SIZE=2&DISPLAY_SORT=1&HSP_SORT=1" \l "sort_mark)** | **[Total score](http://blast.ncbi.nlm.nih.gov/Blast.cgi?CMD=Get&ALIGNMENTS=100&ALIGNMENT_VIEW=Pairwise&CDD_SEARCH_STATE=1&DATABASE_SORT=0&DESCRIPTIONS=100&ENTREZ_QUERY=txid9606 %5BORGN%5D&FIRST_QUERY_NUM=0&FORMAT_OBJECT=Alignment&FORMAT_PAGE_TARGET=&FORMAT_TYPE=HTML&GET_SEQUENCE=yes&I_THRESH=&MASK_CHAR=2&MASK_COLOR=1&NEW_DESIGN=on&NEW_VIEW=yes&NUM_OVERVIEW=100&OLD_BLAST=false&PAGE=Proteins&QUERY_INDEX=0&QUERY_NUMBER=0&RESULTS_PAGE_TARGET=&RID=T2HZ6M4N016&SHOW_LINKOUT=yes&SHOW_OVERVIEW=yes&STEP_NUMBER=&WORD_SIZE=2&DISPLAY_SORT=2&HSP_SORT=1" \l "sort_mark)** | **[Query coverage](http://blast.ncbi.nlm.nih.gov/Blast.cgi?CMD=Get&ALIGNMENTS=100&ALIGNMENT_VIEW=Pairwise&CDD_SEARCH_STATE=1&DATABASE_SORT=0&DESCRIPTIONS=100&ENTREZ_QUERY=txid9606 %5BORGN%5D&FIRST_QUERY_NUM=0&FORMAT_OBJECT=Alignment&FORMAT_PAGE_TARGET=&FORMAT_TYPE=HTML&GET_SEQUENCE=yes&I_THRESH=&MASK_CHAR=2&MASK_COLOR=1&NEW_DESIGN=on&NEW_VIEW=yes&NUM_OVERVIEW=100&OLD_BLAST=false&PAGE=Proteins&QUERY_INDEX=0&QUERY_NUMBER=0&RESULTS_PAGE_TARGET=&RID=T2HZ6M4N016&SHOW_LINKOUT=yes&SHOW_OVERVIEW=yes&STEP_NUMBER=&WORD_SIZE=2&DISPLAY_SORT=4&HSP_SORT=0" \l "sort_mark)** | **[E value](http://blast.ncbi.nlm.nih.gov/Blast.cgi?CMD=Get&ALIGNMENTS=100&ALIGNMENT_VIEW=Pairwise&CDD_SEARCH_STATE=1&DATABASE_SORT=0&DESCRIPTIONS=100&ENTREZ_QUERY=txid9606 %5BORGN%5D&FIRST_QUERY_NUM=0&FORMAT_OBJECT=Alignment&FORMAT_PAGE_TARGET=&FORMAT_TYPE=HTML&GET_SEQUENCE=yes&I_THRESH=&MASK_CHAR=2&MASK_COLOR=1&NEW_DESIGN=on&NEW_VIEW=yes&NUM_OVERVIEW=100&OLD_BLAST=false&PAGE=Proteins&QUERY_INDEX=0&QUERY_NUMBER=0&RESULTS_PAGE_TARGET=&RID=T2HZ6M4N016&SHOW_LINKOUT=yes&SHOW_OVERVIEW=yes&STEP_NUMBER=&WORD_SIZE=2&DISPLAY_SORT=0&HSP_SORT=0" \l "sort_mark)** |
| --- | --- | --- | --- | --- | --- |
| [XP_002342301.1](http://www.ncbi.nlm.nih.gov/entrez/query.fcgi?cmd=Retrieve&db=Protein&list_uids=239741790&dopt=GenPept&RID=T2HZ6M4N016&log$=prottop&blast_rank=1) | PREDICTED: hypothetical protein XP_002342301 [Homo sapiens] | [24.0](http://blast.ncbi.nlm.nih.gov/Blast.cgi#239741790%23239741790) | 24.0 | 60% | 11 |
| [NP_085045.3](http://www.ncbi.nlm.nih.gov/entrez/query.fcgi?cmd=Retrieve&db=Protein&list_uids=117956389&dopt=GenPept&RID=T2HZ6M4N016&log$=prottop&blast_rank=2) | KH homology domain containing 1 [Homo sapiens] | [23.5](http://blast.ncbi.nlm.nih.gov/Blast.cgi#117956389%23117956389) | 23.5 | 50% | 14 |
| [NP_001014447.1](http://www.ncbi.nlm.nih.gov/entrez/query.fcgi?cmd=Retrieve&db=Protein&list_uids=62388877&dopt=GenPept&RID=T2HZ6M4N016&log$=prottop&blast_rank=3) | carboxypeptidase Z isoform 1 [Homo sapiens] | [23.1](http://blast.ncbi.nlm.nih.gov/Blast.cgi#62388877%2362388877) | 23.1 | 50% | 19 |
| [NP_003643.2](http://www.ncbi.nlm.nih.gov/entrez/query.fcgi?cmd=Retrieve&db=Protein&list_uids=62388875&dopt=GenPept&RID=T2HZ6M4N016&log$=prottop&blast_rank=4) | carboxypeptidase Z isoform 2 precursor [Homo sapiens] | [23.1](http://blast.ncbi.nlm.nih.gov/Blast.cgi#62388875%2362388875) | 23.1 | 50% | 19 |
| [NP_001014448.1](http://www.ncbi.nlm.nih.gov/entrez/query.fcgi?cmd=Retrieve&db=Protein&list_uids=62388879&dopt=GenPept&RID=T2HZ6M4N016&log$=prottop&blast_rank=5) | carboxypeptidase Z isoform 3 [Homo sapiens] | [23.1](http://blast.ncbi.nlm.nih.gov/Blast.cgi#62388879%2362388879) | 23.1 | 50% | 19 |
| [NP_001348.2](http://www.ncbi.nlm.nih.gov/entrez/query.fcgi?cmd=Retrieve&db=Protein&list_uids=100913206&dopt=GenPept&RID=T2HZ6M4N016&log$=prottop&blast_rank=6) | ATP-dependent RNA helicase A [Homo sapiens] | [22.7](http://blast.ncbi.nlm.nih.gov/Blast.cgi#100913206%23100913206) | 22.7 | 90% | 26 |
| [NP_001157412.1](http://www.ncbi.nlm.nih.gov/entrez/query.fcgi?cmd=Retrieve&db=Protein&list_uids=255653002&dopt=GenPept&RID=T2HZ6M4N016&log$=prottop&blast_rank=7) | liver glycogen phosphorylase isoform 2 [Homo sapiens] | [22.3](http://blast.ncbi.nlm.nih.gov/Blast.cgi#255653002%23255653002) | 22.3 | 60% | 34 |
| [XP_002345476.1](http://www.ncbi.nlm.nih.gov/entrez/query.fcgi?cmd=Retrieve&db=Protein&list_uids=239757548&dopt=GenPept&RID=T2HZ6M4N016&log$=prottop&blast_rank=8) | PREDICTED: hypothetical protein [Homo sapiens] | [22.3](http://blast.ncbi.nlm.nih.gov/Blast.cgi#239757548%23239757548) | 22.3 | 70% | 34 |
| [NP_002854.3](http://www.ncbi.nlm.nih.gov/entrez/query.fcgi?cmd=Retrieve&db=Protein&list_uids=71037379&dopt=GenPept&RID=T2HZ6M4N016&log$=prottop&blast_rank=9) | liver glycogen phosphorylase isoform 1 [Homo sapiens] | [22.3](http://blast.ncbi.nlm.nih.gov/Blast.cgi#71037379%2371037379) | 22.3 | 60% | 34 |
| [NP_001108198.1](http://www.ncbi.nlm.nih.gov/entrez/query.fcgi?cmd=Retrieve&db=Protein&list_uids=168229226&dopt=GenPept&RID=T2HZ6M4N016&log$=prottop&blast_rank=10) | proline-rich transmembrane protein 4 [Homo sapiens] | [21.8](http://blast.ncbi.nlm.nih.gov/Blast.cgi#168229226%23168229226) | 21.8 | 70% | 46 |
| [NP_001119535.1](http://www.ncbi.nlm.nih.gov/entrez/query.fcgi?cmd=Retrieve&db=Protein&list_uids=186910188&dopt=GenPept&RID=T2HZ6M4N016&log$=prottop&blast_rank=11) | KH homology domain containing 1-like [Homo sapiens] | [21.8](http://blast.ncbi.nlm.nih.gov/Blast.cgi#186910188%23186910188) | 21.8 | 60% | 46 |
| [NP_997400.2](http://www.ncbi.nlm.nih.gov/entrez/query.fcgi?cmd=Retrieve&db=Protein&list_uids=145275198&dopt=GenPept&RID=T2HZ6M4N016&log$=prottop&blast_rank=12) | ADAMTS-like 3 precursor [Homo sapiens] | [21.4](http://blast.ncbi.nlm.nih.gov/Blast.cgi#145275198%23145275198) | 21.4 | 60% | 62 |
| [NP_055578.2](http://www.ncbi.nlm.nih.gov/entrez/query.fcgi?cmd=Retrieve&db=Protein&list_uids=94557305&dopt=GenPept&RID=T2HZ6M4N016&log$=prottop&blast_rank=13) | mitochondrial ribosomal protein L19 precursor [Homo sapiens] | [21.4](http://blast.ncbi.nlm.nih.gov/Blast.cgi#94557305%2394557305) | 37.8 | 60% | 62 |
| [NP_001130013.1](http://www.ncbi.nlm.nih.gov/entrez/query.fcgi?cmd=Retrieve&db=Protein&list_uids=211938442&dopt=GenPept&RID=T2HZ6M4N016&log$=prottop&blast_rank=14) | apolipoprotein L1 isoform c precursor [Homo sapiens] | [21.0](http://blast.ncbi.nlm.nih.gov/Blast.cgi#211938442%23211938442) | 21.0 | 60% | 83 |
| [NP_003652.2](http://www.ncbi.nlm.nih.gov/entrez/query.fcgi?cmd=Retrieve&db=Protein&list_uids=21735614&dopt=GenPept&RID=T2HZ6M4N016&log$=prottop&blast_rank=15) | apolipoprotein L1 isoform a precursor [Homo sapiens] >ref|NP_001130012.1| apolipoprotein L1 isoform a precursor [Homo sapiens] | [21.0](http://blast.ncbi.nlm.nih.gov/Blast.cgi#21735614%2321735614) | 21.0 | 60% | 83 |
| [NP_663318.1](http://www.ncbi.nlm.nih.gov/entrez/query.fcgi?cmd=Retrieve&db=Protein&list_uids=21735616&dopt=GenPept&RID=T2HZ6M4N016&log$=prottop&blast_rank=16) | apolipoprotein L1 isoform b precursor [Homo sapiens] | [21.0](http://blast.ncbi.nlm.nih.gov/Blast.cgi#21735616%2321735616) | 21.0 | 60% | 83 |
| [NP_001158222.1](http://www.ncbi.nlm.nih.gov/entrez/query.fcgi?cmd=Retrieve&db=Protein&list_uids=258613943&dopt=GenPept&RID=T2HZ6M4N016&log$=prottop&blast_rank=17) | aspartate beta-hydroxylase isoform f [Homo sapiens] | [20.6](http://blast.ncbi.nlm.nih.gov/Blast.cgi#258613943%23258613943) | 20.6 | 40% | 111 |
| [XP_002344607.1](http://www.ncbi.nlm.nih.gov/entrez/query.fcgi?cmd=Retrieve&db=Protein&list_uids=239755499&dopt=GenPept&RID=T2HZ6M4N016&log$=prottop&blast_rank=18) | PREDICTED: hypothetical protein [Homo sapiens] | [20.6](http://blast.ncbi.nlm.nih.gov/Blast.cgi#239755499%23239755499) | 34.4 | 70% | 111 |
| [XP_002343123.1](http://www.ncbi.nlm.nih.gov/entrez/query.fcgi?cmd=Retrieve&db=Protein&list_uids=239744342&dopt=GenPept&RID=T2HZ6M4N016&log$=prottop&blast_rank=19) | PREDICTED: hypothetical protein XP_002343123 [Homo sapiens] >ref|XP_002347258.1| PREDICTED: hypothetical protein [Homo sapiens] | [20.6](http://blast.ncbi.nlm.nih.gov/Blast.cgi#239744342%23239744342) | 34.4 | 70% | 111 |
| [NP_001124469.1](http://www.ncbi.nlm.nih.gov/entrez/query.fcgi?cmd=Retrieve&db=Protein&list_uids=196049384&dopt=GenPept&RID=T2HZ6M4N016&log$=prottop&blast_rank=20) | family with sequence similarity 58, member A isoform 2 [Homo sapiens] | [20.6](http://blast.ncbi.nlm.nih.gov/Blast.cgi#196049384%23196049384) | 20.6 | 40% | 111 |
| [XP_002342535.1](http://www.ncbi.nlm.nih.gov/entrez/query.fcgi?cmd=Retrieve&db=Protein&list_uids=239742535&dopt=GenPept&RID=T2HZ6M4N016&log$=prottop&blast_rank=21) | PREDICTED: hypothetical protein XP_002342535 [Homo sapiens] >ref|XP_002346721.1| PREDICTED: hypothetical protein XP_002346721 [Homo sapiens] >ref|XP_002345845.1| PREDICTED: hypothetical protein [Homo sapiens] | [20.6](http://blast.ncbi.nlm.nih.gov/Blast.cgi#239742535%23239742535) | 20.6 | 40% | 111 |
| [NP_009094.3](http://www.ncbi.nlm.nih.gov/entrez/query.fcgi?cmd=Retrieve&db=Protein&list_uids=157694503&dopt=GenPept&RID=T2HZ6M4N016&log$=prottop&blast_rank=22) | solute carrier family 14 (urea transporter), member 2 [Homo sapiens] | [20.6](http://blast.ncbi.nlm.nih.gov/Blast.cgi#157694503%23157694503) | 48.6 | 60% | 111 |
| [NP_997320.2](http://www.ncbi.nlm.nih.gov/entrez/query.fcgi?cmd=Retrieve&db=Protein&list_uids=198442844&dopt=GenPept&RID=T2HZ6M4N016&log$=prottop&blast_rank=23) | dynein, axonemal, heavy chain 10 [Homo sapiens] | [20.6](http://blast.ncbi.nlm.nih.gov/Blast.cgi#198442844%23198442844) | 31.8 | 80% | 111 |
| [NP_071341.2](http://www.ncbi.nlm.nih.gov/entrez/query.fcgi?cmd=Retrieve&db=Protein&list_uids=155722998&dopt=GenPept&RID=T2HZ6M4N016&log$=prottop&blast_rank=24) | solute carrier family 4, sodium bicarbonate transporter-like, member 10 [Homo sapiens] | [20.6](http://blast.ncbi.nlm.nih.gov/Blast.cgi#155722998%23155722998) | 38.2 | 70% | 111 |
| [XP_934664.2](http://www.ncbi.nlm.nih.gov/entrez/query.fcgi?cmd=Retrieve&db=Protein&list_uids=169212799&dopt=GenPept&RID=T2HZ6M4N016&log$=prottop&blast_rank=25) | PREDICTED: hypothetical protein LOC646629 [Homo sapiens] >ref|XP_001723184.1| PREDICTED: similar to hCG1643857 [Homo sapiens] >ref|XP_941881.2| PREDICTED: similar to hCG1643857 [Homo sapiens] | [20.6](http://blast.ncbi.nlm.nih.gov/Blast.cgi#169212799%23169212799) | 20.6 | 40% | 111 |
| [NP_003606.3](http://www.ncbi.nlm.nih.gov/entrez/query.fcgi?cmd=Retrieve&db=Protein&list_uids=134288865&dopt=GenPept&RID=T2HZ6M4N016&log$=prottop&blast_rank=26) | solute carrier family 4, sodium bicarbonate cotransporter, member 7 [Homo sapiens] | [20.6](http://blast.ncbi.nlm.nih.gov/Blast.cgi#134288865%23134288865) | 51.5 | 90% | 111 |
| [NP_689487.2](http://www.ncbi.nlm.nih.gov/entrez/query.fcgi?cmd=Retrieve&db=Protein&list_uids=196049382&dopt=GenPept&RID=T2HZ6M4N016&log$=prottop&blast_rank=27) | family with sequence similarity 58, member A isoform 1 [Homo sapiens] | [20.6](http://blast.ncbi.nlm.nih.gov/Blast.cgi#196049382%23196049382) | 20.6 | 40% | 111 |
| [NP_004849.2](http://www.ncbi.nlm.nih.gov/entrez/query.fcgi?cmd=Retrieve&db=Protein&list_uids=90568034&dopt=GenPept&RID=T2HZ6M4N016&log$=prottop&blast_rank=28) | solute carrier family 4, sodium bicarbonate cotransporter, member 8 isoform b [Homo sapiens] | [20.6](http://blast.ncbi.nlm.nih.gov/Blast.cgi#90568034%2390568034) | 38.2 | 70% | 111 |
| [NP_004309.2](http://www.ncbi.nlm.nih.gov/entrez/query.fcgi?cmd=Retrieve&db=Protein&list_uids=14589866&dopt=GenPept&RID=T2HZ6M4N016&log$=prottop&blast_rank=29) | aspartate beta-hydroxylase isoform a [Homo sapiens] | [20.6](http://blast.ncbi.nlm.nih.gov/Blast.cgi#14589866%2314589866) | 20.6 | 40% | 111 |
| [NP_001035049.1](http://www.ncbi.nlm.nih.gov/entrez/query.fcgi?cmd=Retrieve&db=Protein&list_uids=90403614&dopt=GenPept&RID=T2HZ6M4N016&log$=prottop&blast_rank=30) | solute carrier family 4, sodium bicarbonate cotransporter, member 8 isoform a [Homo sapiens] | [20.6](http://blast.ncbi.nlm.nih.gov/Blast.cgi#90403614%2390403614) | 38.2 | 70% | 111 |
| [NP_922932.2](http://www.ncbi.nlm.nih.gov/entrez/query.fcgi?cmd=Retrieve&db=Protein&list_uids=64276808&dopt=GenPept&RID=T2HZ6M4N016&log$=prottop&blast_rank=31) | ADAM metallopeptidase with thrombospondin type 1 motif, 6 preproprotein [Homo sapiens] | [20.6](http://blast.ncbi.nlm.nih.gov/Blast.cgi#64276808%2364276808) | 20.6 | 40% | 111 |
| [NP_079337.2](http://www.ncbi.nlm.nih.gov/entrez/query.fcgi?cmd=Retrieve&db=Protein&list_uids=40217803&dopt=GenPept&RID=T2HZ6M4N016&log$=prottop&blast_rank=32) | leucine rich repeat containing 8 family, member E [Homo sapiens] | [20.6](http://blast.ncbi.nlm.nih.gov/Blast.cgi#40217803%2340217803) | 20.6 | 40% | 111 |
| [NP_060205.3](http://www.ncbi.nlm.nih.gov/entrez/query.fcgi?cmd=Retrieve&db=Protein&list_uids=42476022&dopt=GenPept&RID=T2HZ6M4N016&log$=prottop&blast_rank=33) | tetratricopeptide repeat domain 27 [Homo sapiens] | [20.6](http://blast.ncbi.nlm.nih.gov/Blast.cgi#42476022%2342476022) | 20.6 | 40% | 111 |
| [NP_653208.2](http://www.ncbi.nlm.nih.gov/entrez/query.fcgi?cmd=Retrieve&db=Protein&list_uids=40255060&dopt=GenPept&RID=T2HZ6M4N016&log$=prottop&blast_rank=34) | cytochrome b5 domain containing 1 [Homo sapiens] | [20.6](http://blast.ncbi.nlm.nih.gov/Blast.cgi#40255060%2340255060) | 20.6 | 40% | 111 |
| [NP_060600.2](http://www.ncbi.nlm.nih.gov/entrez/query.fcgi?cmd=Retrieve&db=Protein&list_uids=56550033&dopt=GenPept&RID=T2HZ6M4N016&log$=prottop&blast_rank=35) | SHQ1 homolog [Homo sapiens] | [20.6](http://blast.ncbi.nlm.nih.gov/Blast.cgi#56550033%2356550033) | 20.6 | 40% | 111 |
| [NP_689947.2](http://www.ncbi.nlm.nih.gov/entrez/query.fcgi?cmd=Retrieve&db=Protein&list_uids=47271471&dopt=GenPept&RID=T2HZ6M4N016&log$=prottop&blast_rank=36) | hypothetical protein LOC221477 [Homo sapiens] | [20.6](http://blast.ncbi.nlm.nih.gov/Blast.cgi#47271471%2347271471) | 20.6 | 40% | 111 |
| [NP_060573.2](http://www.ncbi.nlm.nih.gov/entrez/query.fcgi?cmd=Retrieve&db=Protein&list_uids=34222199&dopt=GenPept&RID=T2HZ6M4N016&log$=prottop&blast_rank=37) | leucine rich repeat containing 8 family, member D [Homo sapiens] >ref|NP_001127951.1| leucine rich repeat containing 8 family, member D [Homo sapiens] | [20.6](http://blast.ncbi.nlm.nih.gov/Blast.cgi#34222199%2334222199) | 20.6 | 40% | 111 |
| [NP_115646.2](http://www.ncbi.nlm.nih.gov/entrez/query.fcgi?cmd=Retrieve&db=Protein&list_uids=19923729&dopt=GenPept&RID=T2HZ6M4N016&log$=prottop&blast_rank=38) | leucine rich repeat containing 8 family, member C [Homo sapiens] | [20.6](http://blast.ncbi.nlm.nih.gov/Blast.cgi#19923729%2319923729) | 20.6 | 40% | 111 |
| [NP_009218.2](http://www.ncbi.nlm.nih.gov/entrez/query.fcgi?cmd=Retrieve&db=Protein&list_uids=116256327&dopt=GenPept&RID=T2HZ6M4N016&log$=prottop&blast_rank=39) | membrane metallo-endopeptidase [Homo sapiens] >ref|NP_000893.2| membrane metallo-endopeptidase [Homo sapiens] >ref|NP_009219.2| membrane metallo-endopeptidase [Homo sapiens] >ref|NP_009220.2| membrane metallo-endopeptidase [Homo sapiens] | [20.6](http://blast.ncbi.nlm.nih.gov/Blast.cgi#116256327%23116256327) | 20.6 | 40% | 111 |
| [NP_612152.1](http://www.ncbi.nlm.nih.gov/entrez/query.fcgi?cmd=Retrieve&db=Protein&list_uids=19923084&dopt=GenPept&RID=T2HZ6M4N016&log$=prottop&blast_rank=40) | polycystin-1L1 [Homo sapiens] | [20.6](http://blast.ncbi.nlm.nih.gov/Blast.cgi#19923084%2319923084) | 20.6 | 60% | 111 |
| [NP_005347.3](http://www.ncbi.nlm.nih.gov/entrez/query.fcgi?cmd=Retrieve&db=Protein&list_uids=112789546&dopt=GenPept&RID=T2HZ6M4N016&log$=prottop&blast_rank=41) | lymphocyte-specific protein tyrosine kinase precursor [Homo sapiens] >ref|NP_001036236.1| lymphocyte-specific protein tyrosine kinase precursor [Homo sapiens] | [20.6](http://blast.ncbi.nlm.nih.gov/Blast.cgi#112789546%23112789546) | 20.6 | 40% | 111 |
| [NP_000062.1](http://www.ncbi.nlm.nih.gov/entrez/query.fcgi?cmd=Retrieve&db=Protein&list_uids=4557415&dopt=GenPept&RID=T2HZ6M4N016&log$=prottop&blast_rank=42) | cystathionine-beta-synthase [Homo sapiens] | [20.6](http://blast.ncbi.nlm.nih.gov/Blast.cgi#4557415%234557415) | 20.6 | 40% | 111 |
| [NP_056165.1](http://www.ncbi.nlm.nih.gov/entrez/query.fcgi?cmd=Retrieve&db=Protein&list_uids=21245134&dopt=GenPept&RID=T2HZ6M4N016&log$=prottop&blast_rank=43) | leucine rich repeat containing 8 family, member B [Homo sapiens] >ref|NP_001127948.1| leucine rich repeat containing 8 family, member B [Homo sapiens] | [20.6](http://blast.ncbi.nlm.nih.gov/Blast.cgi#21245134%2321245134) | 20.6 | 40% | 111 |
| [NP_973724.1](http://www.ncbi.nlm.nih.gov/entrez/query.fcgi?cmd=Retrieve&db=Protein&list_uids=42544125&dopt=GenPept&RID=T2HZ6M4N016&log$=prottop&blast_rank=44) | splicing factor 1 isoform 2 [Homo sapiens] | [20.6](http://blast.ncbi.nlm.nih.gov/Blast.cgi#42544125%2342544125) | 20.6 | 40% | 111 |
| [NP_062540.2](http://www.ncbi.nlm.nih.gov/entrez/query.fcgi?cmd=Retrieve&db=Protein&list_uids=62241040&dopt=GenPept&RID=T2HZ6M4N016&log$=prottop&blast_rank=45) | leucine rich repeat containing 8 family, member A [Homo sapiens] >ref|NP_001120716.1| leucine rich repeat containing 8 family, member A [Homo sapiens] >ref|NP_001120717.1| leucine rich repeat containing 8 family, member A [Homo sapiens] | [20.6](http://blast.ncbi.nlm.nih.gov/Blast.cgi#62241040%2362241040) | 20.6 | 40% | 111 |
| [NP_001773.1](http://www.ncbi.nlm.nih.gov/entrez/query.fcgi?cmd=Retrieve&db=Protein&list_uids=4502683&dopt=GenPept&RID=T2HZ6M4N016&log$=prottop&blast_rank=46) | CD72 molecule [Homo sapiens] | [20.6](http://blast.ncbi.nlm.nih.gov/Blast.cgi#4502683%234502683) | 20.6 | 70% | 111 |
| [XP_933414.4](http://www.ncbi.nlm.nih.gov/entrez/query.fcgi?cmd=Retrieve&db=Protein&list_uids=239744776&dopt=GenPept&RID=T2HZ6M4N016&log$=prottop&blast_rank=47) | PREDICTED: hypothetical protein [Homo sapiens] | [20.2](http://blast.ncbi.nlm.nih.gov/Blast.cgi#239744776%23239744776) | 37.3 | 60% | 149 |
| [NP_940857.2](http://www.ncbi.nlm.nih.gov/entrez/query.fcgi?cmd=Retrieve&db=Protein&list_uids=134031945&dopt=GenPept&RID=T2HZ6M4N016&log$=prottop&blast_rank=48) | SCO-spondin precursor [Homo sapiens] | [20.2](http://blast.ncbi.nlm.nih.gov/Blast.cgi#134031945%23134031945) | 20.2 | 70% | 149 |
| [NP_620714.2](http://www.ncbi.nlm.nih.gov/entrez/query.fcgi?cmd=Retrieve&db=Protein&list_uids=157502181&dopt=GenPept&RID=T2HZ6M4N016&log$=prottop&blast_rank=49) | two pore segment channel 2 [Homo sapiens] | [20.2](http://blast.ncbi.nlm.nih.gov/Blast.cgi#157502181%23157502181) | 20.2 | 70% | 149 |
| [NP_006647.3](http://www.ncbi.nlm.nih.gov/entrez/query.fcgi?cmd=Retrieve&db=Protein&list_uids=117190519&dopt=GenPept&RID=T2HZ6M4N016&log$=prottop&blast_rank=50) | neuraminidase 3 [Homo sapiens] | [20.2](http://blast.ncbi.nlm.nih.gov/Blast.cgi#117190519%23117190519) | 20.2 | 50% | 149 |
| [XP_941531.2](http://www.ncbi.nlm.nih.gov/entrez/query.fcgi?cmd=Retrieve&db=Protein&list_uids=113424011&dopt=GenPept&RID=T2HZ6M4N016&log$=prottop&blast_rank=51) | PREDICTED: hypothetical protein [Homo sapiens] >ref|XP_001716086.1| PREDICTED: hypothetical protein [Homo sapiens] | [20.2](http://blast.ncbi.nlm.nih.gov/Blast.cgi#113424011%23113424011) | 37.3 | 60% | 149 |
| [NP_085097.3](http://www.ncbi.nlm.nih.gov/entrez/query.fcgi?cmd=Retrieve&db=Protein&list_uids=119395725&dopt=GenPept&RID=T2HZ6M4N016&log$=prottop&blast_rank=52) | cytoplasmic polyadenylation element binding protein 1 isoform 1 [Homo sapiens] | [20.2](http://blast.ncbi.nlm.nih.gov/Blast.cgi#119395725%23119395725) | 20.2 | 80% | 149 |
| [NP_001073002.1](http://www.ncbi.nlm.nih.gov/entrez/query.fcgi?cmd=Retrieve&db=Protein&list_uids=119395720&dopt=GenPept&RID=T2HZ6M4N016&log$=prottop&blast_rank=53) | cytoplasmic polyadenylation element binding protein 1 isoform 3 [Homo sapiens] >ref|NP_001073003.1| cytoplasmic polyadenylation element binding protein 1 isoform 3 [Homo sapiens] | [20.2](http://blast.ncbi.nlm.nih.gov/Blast.cgi#119395720%23119395720) | 20.2 | 80% | 149 |
| [NP_001073001.1](http://www.ncbi.nlm.nih.gov/entrez/query.fcgi?cmd=Retrieve&db=Protein&list_uids=119395718&dopt=GenPept&RID=T2HZ6M4N016&log$=prottop&blast_rank=54) | cytoplasmic polyadenylation element binding protein 1 isoform 2 [Homo sapiens] | [20.2](http://blast.ncbi.nlm.nih.gov/Blast.cgi#119395718%23119395718) | 20.2 | 80% | 149 |
| [NP_006867.1](http://www.ncbi.nlm.nih.gov/entrez/query.fcgi?cmd=Retrieve&db=Protein&list_uids=5802984&dopt=GenPept&RID=T2HZ6M4N016&log$=prottop&blast_rank=55) | UDP-GlcNAc:betaGal beta-1,3-N-acetylglucosaminyltransferase 1 [Homo sapiens] | [20.2](http://blast.ncbi.nlm.nih.gov/Blast.cgi#5802984%235802984) | 20.2 | 70% | 149 |
| [NP_919276.2](http://www.ncbi.nlm.nih.gov/entrez/query.fcgi?cmd=Retrieve&db=Protein&list_uids=154937346&dopt=GenPept&RID=T2HZ6M4N016&log$=prottop&blast_rank=56) | coiled-coil domain containing 129 [Homo sapiens] | [19.7](http://blast.ncbi.nlm.nih.gov/Blast.cgi#154937346%23154937346) | 19.7 | 70% | 200 |
| [NP_003719.2](http://www.ncbi.nlm.nih.gov/entrez/query.fcgi?cmd=Retrieve&db=Protein&list_uids=16933525&dopt=GenPept&RID=T2HZ6M4N016&log$=prottop&blast_rank=57) | unc5C precursor [Homo sapiens] | [19.7](http://blast.ncbi.nlm.nih.gov/Blast.cgi#16933525%2316933525) | 19.7 | 80% | 200 |
| [NP_001017995.1](http://www.ncbi.nlm.nih.gov/entrez/query.fcgi?cmd=Retrieve&db=Protein&list_uids=63055059&dopt=GenPept&RID=T2HZ6M4N016&log$=prottop&blast_rank=58) | SH3 and PX domains 2B [Homo sapiens] | [19.7](http://blast.ncbi.nlm.nih.gov/Blast.cgi#63055059%2363055059) | 19.7 | 60% | 200 |
| [NP_060685.2](http://www.ncbi.nlm.nih.gov/entrez/query.fcgi?cmd=Retrieve&db=Protein&list_uids=39780597&dopt=GenPept&RID=T2HZ6M4N016&log$=prottop&blast_rank=59) | PNMA-like 1 isoform a [Homo sapiens] | [19.7](http://blast.ncbi.nlm.nih.gov/Blast.cgi#39780597%2339780597) | 19.7 | 60% | 200 |
| [NP_671723.1](http://www.ncbi.nlm.nih.gov/entrez/query.fcgi?cmd=Retrieve&db=Protein&list_uids=22218345&dopt=GenPept&RID=T2HZ6M4N016&log$=prottop&blast_rank=60) | LAG1 homolog, ceramide synthase 5 [Homo sapiens] | [19.7](http://blast.ncbi.nlm.nih.gov/Blast.cgi#22218345%2322218345) | 19.7 | 60% | 200 |
| [NP_001096619.1](http://www.ncbi.nlm.nih.gov/entrez/query.fcgi?cmd=Retrieve&db=Protein&list_uids=156766054&dopt=GenPept&RID=T2HZ6M4N016&log$=prottop&blast_rank=61) | PNMA-like 1 isoform b [Homo sapiens] | [19.7](http://blast.ncbi.nlm.nih.gov/Blast.cgi#156766054%23156766054) | 19.7 | 60% | 200 |
| [NP_000657.1](http://www.ncbi.nlm.nih.gov/entrez/query.fcgi?cmd=Retrieve&db=Protein&list_uids=4501901&dopt=GenPept&RID=T2HZ6M4N016&log$=prottop&blast_rank=62) | aminoacylase 1 [Homo sapiens] | [19.7](http://blast.ncbi.nlm.nih.gov/Blast.cgi#4501901%234501901) | 19.7 | 50% | 200 |
| [NP_001195.2](http://www.ncbi.nlm.nih.gov/entrez/query.fcgi?cmd=Retrieve&db=Protein&list_uids=15451916&dopt=GenPept&RID=T2HZ6M4N016&log$=prottop&blast_rank=63) | bone morphogenetic protein receptor type II precursor [Homo sapiens] | [19.7](http://blast.ncbi.nlm.nih.gov/Blast.cgi#15451916%2315451916) | 19.7 | 80% | 200 |
| [XP_002346265.1](http://www.ncbi.nlm.nih.gov/entrez/query.fcgi?cmd=Retrieve&db=Protein&list_uids=239758005&dopt=GenPept&RID=T2HZ6M4N016&log$=prottop&blast_rank=64) | PREDICTED: hypothetical protein XP_002346265 [Homo sapiens] | [19.3](http://blast.ncbi.nlm.nih.gov/Blast.cgi#239758005%23239758005) | 19.3 | 80% | 268 |
| [XP_002342633.1](http://www.ncbi.nlm.nih.gov/entrez/query.fcgi?cmd=Retrieve&db=Protein&list_uids=239742716&dopt=GenPept&RID=T2HZ6M4N016&log$=prottop&blast_rank=65) | PREDICTED: hypothetical protein XP_002342633 [Homo sapiens] >ref|XP_002346765.1| PREDICTED: hypothetical protein XP_002346765 [Homo sapiens] >ref|XP_002345898.1| PREDICTED: hypothetical protein [Homo sapiens] | [19.3](http://blast.ncbi.nlm.nih.gov/Blast.cgi#239742716%23239742716) | 19.3 | 70% | 268 |
| [NP_775822.3](http://www.ncbi.nlm.nih.gov/entrez/query.fcgi?cmd=Retrieve&db=Protein&list_uids=67906195&dopt=GenPept&RID=T2HZ6M4N016&log$=prottop&blast_rank=66) | ankyrin repeat and sterile alpha motif domain containing 6 [Homo sapiens] | [19.3](http://blast.ncbi.nlm.nih.gov/Blast.cgi#67906195%2367906195) | 19.3 | 70% | 268 |
| [NP_001416.1](http://www.ncbi.nlm.nih.gov/entrez/query.fcgi?cmd=Retrieve&db=Protein&list_uids=4503563&dopt=GenPept&RID=T2HZ6M4N016&log$=prottop&blast_rank=67) | epithelial membrane protein 3 [Homo sapiens] | [19.3](http://blast.ncbi.nlm.nih.gov/Blast.cgi#4503563%234503563) | 19.3 | 50% | 268 |
| [NP_000171.1](http://www.ncbi.nlm.nih.gov/entrez/query.fcgi?cmd=Retrieve&db=Protein&list_uids=4504217&dopt=GenPept&RID=T2HZ6M4N016&log$=prottop&blast_rank=68) | guanylate cyclase 2D, membrane (retina-specific) precursor [Homo sapiens] | [19.3](http://blast.ncbi.nlm.nih.gov/Blast.cgi#4504217%234504217) | 33.5 | 100% | 268 |
| [XP_002347830.1](http://www.ncbi.nlm.nih.gov/entrez/query.fcgi?cmd=Retrieve&db=Protein&list_uids=239751374&dopt=GenPept&RID=T2HZ6M4N016&log$=prottop&blast_rank=69) | PREDICTED: hypothetical protein XP_002347830 [Homo sapiens] >ref|XP_002345094.1| PREDICTED: hypothetical protein [Homo sapiens] | [18.9](http://blast.ncbi.nlm.nih.gov/Blast.cgi#239751374%23239751374) | 18.9 | 80% | 360 |
| [NP_597681.2](http://www.ncbi.nlm.nih.gov/entrez/query.fcgi?cmd=Retrieve&db=Protein&list_uids=110349717&dopt=GenPept&RID=T2HZ6M4N016&log$=prottop&blast_rank=70) | titin isoform novex-2 [Homo sapiens] | [18.9](http://blast.ncbi.nlm.nih.gov/Blast.cgi#110349717%23110349717) | 470 | 90% | 360 |
| [NP_596869.3](http://www.ncbi.nlm.nih.gov/entrez/query.fcgi?cmd=Retrieve&db=Protein&list_uids=110349719&dopt=GenPept&RID=T2HZ6M4N016&log$=prottop&blast_rank=71) | titin isoform N2-A [Homo sapiens] | [18.9](http://blast.ncbi.nlm.nih.gov/Blast.cgi#110349719%23110349719) | 470 | 90% | 360 |
| [NP_597676.2](http://www.ncbi.nlm.nih.gov/entrez/query.fcgi?cmd=Retrieve&db=Protein&list_uids=110349713&dopt=GenPept&RID=T2HZ6M4N016&log$=prottop&blast_rank=72) | titin isoform novex-1 [Homo sapiens] | [18.9](http://blast.ncbi.nlm.nih.gov/Blast.cgi#110349713%23110349713) | 470 | 90% | 360 |
| [NP_003310.3](http://www.ncbi.nlm.nih.gov/entrez/query.fcgi?cmd=Retrieve&db=Protein&list_uids=110349715&dopt=GenPept&RID=T2HZ6M4N016&log$=prottop&blast_rank=73) | titin isoform N2-B [Homo sapiens] | [18.9](http://blast.ncbi.nlm.nih.gov/Blast.cgi#110349715%23110349715) | 470 | 90% | 360 |
| [NP_588610.2](http://www.ncbi.nlm.nih.gov/entrez/query.fcgi?cmd=Retrieve&db=Protein&list_uids=62243567&dopt=GenPept&RID=T2HZ6M4N016&log$=prottop&blast_rank=74) | netrin receptor Unc5h1 precursor [Homo sapiens] | [18.9](http://blast.ncbi.nlm.nih.gov/Blast.cgi#62243567%2362243567) | 18.9 | 60% | 360 |
| [NP_036313.3](http://www.ncbi.nlm.nih.gov/entrez/query.fcgi?cmd=Retrieve&db=Protein&list_uids=52630440&dopt=GenPept&RID=T2HZ6M4N016&log$=prottop&blast_rank=75) | FK506-binding protein 8 [Homo sapiens] | [18.9](http://blast.ncbi.nlm.nih.gov/Blast.cgi#52630440%2352630440) | 18.9 | 60% | 360 |
| [NP_006193.1](http://www.ncbi.nlm.nih.gov/entrez/query.fcgi?cmd=Retrieve&db=Protein&list_uids=5453862&dopt=GenPept&RID=T2HZ6M4N016&log$=prottop&blast_rank=76) | phosphodiesterase 4A isoform 4 [Homo sapiens] | [18.9](http://blast.ncbi.nlm.nih.gov/Blast.cgi#5453862%235453862) | 18.9 | 70% | 360 |
| [NP_000138.2](http://www.ncbi.nlm.nih.gov/entrez/query.fcgi?cmd=Retrieve&db=Protein&list_uids=119360348&dopt=GenPept&RID=T2HZ6M4N016&log$=prottop&blast_rank=77) | fucosidase, alpha-L-1, tissue precursor [Homo sapiens] | [18.9](http://blast.ncbi.nlm.nih.gov/Blast.cgi#119360348%23119360348) | 18.9 | 70% | 360 |
| [NP_001004320.1](http://www.ncbi.nlm.nih.gov/entrez/query.fcgi?cmd=Retrieve&db=Protein&list_uids=51972212&dopt=GenPept&RID=T2HZ6M4N016&log$=prottop&blast_rank=78) | transmembrane protein 195 [Homo sapiens] | [18.9](http://blast.ncbi.nlm.nih.gov/Blast.cgi#51972212%2351972212) | 18.9 | 70% | 360 |
| [NP_067028.1](http://www.ncbi.nlm.nih.gov/entrez/query.fcgi?cmd=Retrieve&db=Protein&list_uids=11034843&dopt=GenPept&RID=T2HZ6M4N016&log$=prottop&blast_rank=79) | ras homolog gene family, member U [Homo sapiens] | [18.9](http://blast.ncbi.nlm.nih.gov/Blast.cgi#11034843%2311034843) | 18.9 | 50% | 360 |
| [NP_006144.1](http://www.ncbi.nlm.nih.gov/entrez/query.fcgi?cmd=Retrieve&db=Protein&list_uids=5453754&dopt=GenPept&RID=T2HZ6M4N016&log$=prottop&blast_rank=80) | NCK adaptor protein 1 [Homo sapiens] | [18.9](http://blast.ncbi.nlm.nih.gov/Blast.cgi#5453754%235453754) | 18.9 | 50% | 360 |
| [NP_037514.2](http://www.ncbi.nlm.nih.gov/entrez/query.fcgi?cmd=Retrieve&db=Protein&list_uids=32455271&dopt=GenPept&RID=T2HZ6M4N016&log$=prottop&blast_rank=81) | protein-O-mannosyltransferase 2 [Homo sapiens] | [18.9](http://blast.ncbi.nlm.nih.gov/Blast.cgi#32455271%2332455271) | 18.9 | 50% | 360 |
| [XP_002345486.1](http://www.ncbi.nlm.nih.gov/entrez/query.fcgi?cmd=Retrieve&db=Protein&list_uids=239757576&dopt=GenPept&RID=T2HZ6M4N016&log$=prottop&blast_rank=82) | PREDICTED: hypothetical protein [Homo sapiens] | [18.5](http://blast.ncbi.nlm.nih.gov/Blast.cgi#239757576%23239757576) | 18.5 | 60% | 482 |
| [XP_002345614.1](http://www.ncbi.nlm.nih.gov/entrez/query.fcgi?cmd=Retrieve&db=Protein&list_uids=239753301&dopt=GenPept&RID=T2HZ6M4N016&log$=prottop&blast_rank=83) | PREDICTED: hypothetical protein [Homo sapiens] | [18.5](http://blast.ncbi.nlm.nih.gov/Blast.cgi#239753301%23239753301) | 18.5 | 70% | 482 |
| [XP_002348162.1](http://www.ncbi.nlm.nih.gov/entrez/query.fcgi?cmd=Retrieve&db=Protein&list_uids=239752232&dopt=GenPept&RID=T2HZ6M4N016&log$=prottop&blast_rank=84) | PREDICTED: hypothetical protein [Homo sapiens] >ref|XP_002345544.1| PREDICTED: hypothetical protein [Homo sapiens] | [18.5](http://blast.ncbi.nlm.nih.gov/Blast.cgi#239752232%23239752232) | 18.5 | 90% | 482 |
| [XP_001719309.2](http://www.ncbi.nlm.nih.gov/entrez/query.fcgi?cmd=Retrieve&db=Protein&list_uids=239751445&dopt=GenPept&RID=T2HZ6M4N016&log$=prottop&blast_rank=85) | PREDICTED: similar to CD300C antigen [Homo sapiens] | [18.5](http://blast.ncbi.nlm.nih.gov/Blast.cgi#239751445%23239751445) | 18.5 | 60% | 482 |
| [XP_001720138.2](http://www.ncbi.nlm.nih.gov/entrez/query.fcgi?cmd=Retrieve&db=Protein&list_uids=239749027&dopt=GenPept&RID=T2HZ6M4N016&log$=prottop&blast_rank=86) | PREDICTED: hypothetical protein LOC285804 [Homo sapiens] | [18.5](http://blast.ncbi.nlm.nih.gov/Blast.cgi#239749027%23239749027) | 73.8 | 50% | 482 |
| [XP_001714704.2](http://www.ncbi.nlm.nih.gov/entrez/query.fcgi?cmd=Retrieve&db=Protein&list_uids=239745950&dopt=GenPept&RID=T2HZ6M4N016&log$=prottop&blast_rank=87) | PREDICTED: similar to CD300C antigen [Homo sapiens] >ref|XP_001718105.2| PREDICTED: similar to CD300C antigen [Homo sapiens] | [18.5](http://blast.ncbi.nlm.nih.gov/Blast.cgi#239745950%23239745950) | 18.5 | 60% | 482 |
| [NP_001138777.1](http://www.ncbi.nlm.nih.gov/entrez/query.fcgi?cmd=Retrieve&db=Protein&list_uids=224451036&dopt=GenPept&RID=T2HZ6M4N016&log$=prottop&blast_rank=88) | KIAA1683 isoform c [Homo sapiens] | [18.5](http://blast.ncbi.nlm.nih.gov/Blast.cgi#224451036%23224451036) | 31.8 | 60% | 482 |
| [NP_001138776.1](http://www.ncbi.nlm.nih.gov/entrez/query.fcgi?cmd=Retrieve&db=Protein&list_uids=224451032&dopt=GenPept&RID=T2HZ6M4N016&log$=prottop&blast_rank=89) | KIAA1683 isoform a [Homo sapiens] | [18.5](http://blast.ncbi.nlm.nih.gov/Blast.cgi#224451032%23224451032) | 31.8 | 60% | 482 |
| [XP_001714340.1](http://www.ncbi.nlm.nih.gov/entrez/query.fcgi?cmd=Retrieve&db=Protein&list_uids=169165926&dopt=GenPept&RID=T2HZ6M4N016&log$=prottop&blast_rank=90) | PREDICTED: similar to ataxia telangiectasia and Rad3 related protein, partial [Homo sapiens] | [18.5](http://blast.ncbi.nlm.nih.gov/Blast.cgi#169165926%23169165926) | 18.5 | 60% | 482 |
| [NP_775907.4](http://www.ncbi.nlm.nih.gov/entrez/query.fcgi?cmd=Retrieve&db=Protein&list_uids=145580610&dopt=GenPept&RID=T2HZ6M4N016&log$=prottop&blast_rank=91) | WD repeat domain 62 isoform 2 [Homo sapiens] | [18.5](http://blast.ncbi.nlm.nih.gov/Blast.cgi#145580610%23145580610) | 18.5 | 50% | 482 |
| [NP_689771.3](http://www.ncbi.nlm.nih.gov/entrez/query.fcgi?cmd=Retrieve&db=Protein&list_uids=154813191&dopt=GenPept&RID=T2HZ6M4N016&log$=prottop&blast_rank=92) | IQ motif containing E isoform 1 [Homo sapiens] | [18.5](http://blast.ncbi.nlm.nih.gov/Blast.cgi#154813191%23154813191) | 18.5 | 60% | 482 |
| [NP_001077430.1](http://www.ncbi.nlm.nih.gov/entrez/query.fcgi?cmd=Retrieve&db=Protein&list_uids=145580608&dopt=GenPept&RID=T2HZ6M4N016&log$=prottop&blast_rank=93) | WD repeat domain 62 isoform 1 [Homo sapiens] | [18.5](http://blast.ncbi.nlm.nih.gov/Blast.cgi#145580608%23145580608) | 18.5 | 50% | 482 |
| [NP_001093860.1](http://www.ncbi.nlm.nih.gov/entrez/query.fcgi?cmd=Retrieve&db=Protein&list_uids=154813193&dopt=GenPept&RID=T2HZ6M4N016&log$=prottop&blast_rank=94) | IQ motif containing E isoform 2 [Homo sapiens] | [18.5](http://blast.ncbi.nlm.nih.gov/Blast.cgi#154813193%23154813193) | 18.5 | 60% | 482 |
| [XP_002343271.1](http://www.ncbi.nlm.nih.gov/entrez/query.fcgi?cmd=Retrieve&db=Protein&list_uids=239744784&dopt=GenPept&RID=T2HZ6M4N016&log$=prottop&blast_rank=95) | PREDICTED: hypothetical protein XP_002343271 [Homo sapiens] >ref|XP_002347420.1| PREDICTED: hypothetical protein XP_002347420 [Homo sapiens] >ref|XP_002344759.1| PREDICTED: similar to hCG2019585 [Homo sapiens] | [18.5](http://blast.ncbi.nlm.nih.gov/Blast.cgi#239744784%23239744784) | 18.5 | 70% | 482 |
| [NP_001160164.1](http://www.ncbi.nlm.nih.gov/entrez/query.fcgi?cmd=Retrieve&db=Protein&list_uids=262359907&dopt=GenPept&RID=T2HZ6M4N016&log$=prottop&blast_rank=96) | hypothetical protein LOC100131378 [Homo sapiens] | [18.5](http://blast.ncbi.nlm.nih.gov/Blast.cgi#262359907%23262359907) | 18.5 | 60% | 482 |
| [NP_001007278.1](http://www.ncbi.nlm.nih.gov/entrez/query.fcgi?cmd=Retrieve&db=Protein&list_uids=55956768&dopt=GenPept&RID=T2HZ6M4N016&log$=prottop&blast_rank=97) | etoposide induced 2.4 isoform 2 [Homo sapiens] | [18.5](http://blast.ncbi.nlm.nih.gov/Blast.cgi#55956768%2355956768) | 18.5 | 50% | 482 |
| [NP_000834.2](http://www.ncbi.nlm.nih.gov/entrez/query.fcgi?cmd=Retrieve&db=Protein&list_uids=110611176&dopt=GenPept&RID=T2HZ6M4N016&log$=prottop&blast_rank=98) | glutamate receptor, metabotropic 6 precursor [Homo sapiens] | [18.5](http://blast.ncbi.nlm.nih.gov/Blast.cgi#110611176%23110611176) | 18.5 | 50% | 482 |
| [NP_001129334.1](http://www.ncbi.nlm.nih.gov/entrez/query.fcgi?cmd=Retrieve&db=Protein&list_uids=209529726&dopt=GenPept&RID=T2HZ6M4N016&log$=prottop&blast_rank=99) | PHD finger protein 21B isoform 2 [Homo sapiens] | [18.5](http://blast.ncbi.nlm.nih.gov/Blast.cgi#209529726%23209529726) | 33.5 | 60% | 482 |
| [NP_612424.1](http://www.ncbi.nlm.nih.gov/entrez/query.fcgi?cmd=Retrieve&db=Protein&list_uids=19923937&dopt=GenPept&RID=T2HZ6M4N016&log$=prottop&blast_rank=100) | PHD finger protein 21B isoform 1 [Homo sapiens] | [18.5](http://blast.ncbi.nlm.nih.gov/Blast.cgi#19923937%2319923937) | 33.5 | 60% | 482 |

| **Accession** | **Proteins with a match to GHNNHNRHHP peptide** | **[Max score](http://blast.ncbi.nlm.nih.gov/Blast.cgi?CMD=Get&ALIGNMENTS=100&ALIGNMENT_VIEW=Pairwise&CDD_SEARCH_STATE=1&DATABASE_SORT=0&DESCRIPTIONS=100&ENTREZ_QUERY=txid9606 %5BORGN%5D&FIRST_QUERY_NUM=0&FORMAT_OBJECT=Alignment&FORMAT_PAGE_TARGET=&FORMAT_TYPE=HTML&GET_SEQUENCE=yes&I_THRESH=&MASK_CHAR=2&MASK_COLOR=1&NEW_DESIGN=on&NEW_VIEW=yes&NUM_OVERVIEW=100&OLD_BLAST=false&PAGE=Proteins&QUERY_INDEX=0&QUERY_NUMBER=0&RESULTS_PAGE_TARGET=&RID=T2J4UBHW012&SHOW_LINKOUT=yes&SHOW_OVERVIEW=yes&STEP_NUMBER=&WORD_SIZE=2&DISPLAY_SORT=1&HSP_SORT=1" \l "sort_mark)** | **[Total score](http://blast.ncbi.nlm.nih.gov/Blast.cgi?CMD=Get&ALIGNMENTS=100&ALIGNMENT_VIEW=Pairwise&CDD_SEARCH_STATE=1&DATABASE_SORT=0&DESCRIPTIONS=100&ENTREZ_QUERY=txid9606 %5BORGN%5D&FIRST_QUERY_NUM=0&FORMAT_OBJECT=Alignment&FORMAT_PAGE_TARGET=&FORMAT_TYPE=HTML&GET_SEQUENCE=yes&I_THRESH=&MASK_CHAR=2&MASK_COLOR=1&NEW_DESIGN=on&NEW_VIEW=yes&NUM_OVERVIEW=100&OLD_BLAST=false&PAGE=Proteins&QUERY_INDEX=0&QUERY_NUMBER=0&RESULTS_PAGE_TARGET=&RID=T2J4UBHW012&SHOW_LINKOUT=yes&SHOW_OVERVIEW=yes&STEP_NUMBER=&WORD_SIZE=2&DISPLAY_SORT=2&HSP_SORT=1" \l "sort_mark)** | **[Query coverage](http://blast.ncbi.nlm.nih.gov/Blast.cgi?CMD=Get&ALIGNMENTS=100&ALIGNMENT_VIEW=Pairwise&CDD_SEARCH_STATE=1&DATABASE_SORT=0&DESCRIPTIONS=100&ENTREZ_QUERY=txid9606 %5BORGN%5D&FIRST_QUERY_NUM=0&FORMAT_OBJECT=Alignment&FORMAT_PAGE_TARGET=&FORMAT_TYPE=HTML&GET_SEQUENCE=yes&I_THRESH=&MASK_CHAR=2&MASK_COLOR=1&NEW_DESIGN=on&NEW_VIEW=yes&NUM_OVERVIEW=100&OLD_BLAST=false&PAGE=Proteins&QUERY_INDEX=0&QUERY_NUMBER=0&RESULTS_PAGE_TARGET=&RID=T2J4UBHW012&SHOW_LINKOUT=yes&SHOW_OVERVIEW=yes&STEP_NUMBER=&WORD_SIZE=2&DISPLAY_SORT=4&HSP_SORT=0" \l "sort_mark)** | **[E value](http://blast.ncbi.nlm.nih.gov/Blast.cgi?CMD=Get&ALIGNMENTS=100&ALIGNMENT_VIEW=Pairwise&CDD_SEARCH_STATE=1&DATABASE_SORT=0&DESCRIPTIONS=100&ENTREZ_QUERY=txid9606 %5BORGN%5D&FIRST_QUERY_NUM=0&FORMAT_OBJECT=Alignment&FORMAT_PAGE_TARGET=&FORMAT_TYPE=HTML&GET_SEQUENCE=yes&I_THRESH=&MASK_CHAR=2&MASK_COLOR=1&NEW_DESIGN=on&NEW_VIEW=yes&NUM_OVERVIEW=100&OLD_BLAST=false&PAGE=Proteins&QUERY_INDEX=0&QUERY_NUMBER=0&RESULTS_PAGE_TARGET=&RID=T2J4UBHW012&SHOW_LINKOUT=yes&SHOW_OVERVIEW=yes&STEP_NUMBER=&WORD_SIZE=2&DISPLAY_SORT=0&HSP_SORT=0" \l "sort_mark)** |
| --- | --- | --- | --- | --- | --- |
| [NP_001034200.1](http://www.ncbi.nlm.nih.gov/entrez/query.fcgi?cmd=Retrieve&db=Protein&list_uids=84993742&dopt=GenPept&RID=T2J4UBHW012&log$=prottop&blast_rank=1) | tripartite motif-containing 71 [Homo sapiens] | [23.5](http://blast.ncbi.nlm.nih.gov/Blast.cgi#84993742%2384993742) | 23.5 | 90% | 14 |
| [NP_005240.3](http://www.ncbi.nlm.nih.gov/entrez/query.fcgi?cmd=Retrieve&db=Protein&list_uids=32307177&dopt=GenPept&RID=T2J4UBHW012&log$=prottop&blast_rank=2) | forkhead box G1 [Homo sapiens] | [22.7](http://blast.ncbi.nlm.nih.gov/Blast.cgi#32307177%2332307177) | 22.7 | 90% | 26 |
| [NP_006141.2](http://www.ncbi.nlm.nih.gov/entrez/query.fcgi?cmd=Retrieve&db=Protein&list_uids=7710129&dopt=GenPept&RID=T2J4UBHW012&log$=prottop&blast_rank=3) | LIM domain only 6 [Homo sapiens] | [21.4](http://blast.ncbi.nlm.nih.gov/Blast.cgi#7710129%237710129) | 63.4 | 80% | 62 |
| [NP_001128529.2](http://www.ncbi.nlm.nih.gov/entrez/query.fcgi?cmd=Retrieve&db=Protein&list_uids=288541295&dopt=GenPept&RID=T2J4UBHW012&log$=prottop&blast_rank=4) | leucine rich repeat containing 15 isoform a [Homo sapiens] | [21.0](http://blast.ncbi.nlm.nih.gov/Blast.cgi#288541295%23288541295) | 21.0 | 50% | 83 |
| [XP_002342064.1](http://www.ncbi.nlm.nih.gov/entrez/query.fcgi?cmd=Retrieve&db=Protein&list_uids=239740984&dopt=GenPept&RID=T2J4UBHW012&log$=prottop&blast_rank=5) | PREDICTED: hypothetical protein XP_002342064 [Homo sapiens] | [21.0](http://blast.ncbi.nlm.nih.gov/Blast.cgi#239740984%23239740984) | 21.0 | 50% | 83 |
| [NP_001074295.1](http://www.ncbi.nlm.nih.gov/entrez/query.fcgi?cmd=Retrieve&db=Protein&list_uids=157909822&dopt=GenPept&RID=T2J4UBHW012&log$=prottop&blast_rank=6) | pragmin [Homo sapiens] | [21.0](http://blast.ncbi.nlm.nih.gov/Blast.cgi#157909822%23157909822) | 21.0 | 50% | 83 |
| [NP_001034881.1](http://www.ncbi.nlm.nih.gov/entrez/query.fcgi?cmd=Retrieve&db=Protein&list_uids=89886261&dopt=GenPept&RID=T2J4UBHW012&log$=prottop&blast_rank=7) | histidine rich carboxyl terminus 1 [Homo sapiens] | [21.0](http://blast.ncbi.nlm.nih.gov/Blast.cgi#89886261%2389886261) | 31.8 | 90% | 83 |
| [NP_570843.2](http://www.ncbi.nlm.nih.gov/entrez/query.fcgi?cmd=Retrieve&db=Protein&list_uids=288541297&dopt=GenPept&RID=T2J4UBHW012&log$=prottop&blast_rank=8) | leucine rich repeat containing 15 isoform b [Homo sapiens] | [21.0](http://blast.ncbi.nlm.nih.gov/Blast.cgi#288541297%23288541297) | 21.0 | 50% | 83 |
| [NP_065823.1](http://www.ncbi.nlm.nih.gov/entrez/query.fcgi?cmd=Retrieve&db=Protein&list_uids=32698730&dopt=GenPept&RID=T2J4UBHW012&log$=prottop&blast_rank=9) | nuclear fragile X mental retardation protein interacting protein 2 [Homo sapiens] | [21.0](http://blast.ncbi.nlm.nih.gov/Blast.cgi#32698730%2332698730) | 21.0 | 80% | 83 |
| [NP_005131.1](http://www.ncbi.nlm.nih.gov/entrez/query.fcgi?cmd=Retrieve&db=Protein&list_uids=42718011&dopt=GenPept&RID=T2J4UBHW012&log$=prottop&blast_rank=10) | cyclic nucleotide gated channel alpha 2 [Homo sapiens] | [20.6](http://blast.ncbi.nlm.nih.gov/Blast.cgi#42718011%2342718011) | 20.6 | 80% | 111 |
| [NP_057315.3](http://www.ncbi.nlm.nih.gov/entrez/query.fcgi?cmd=Retrieve&db=Protein&list_uids=149408126&dopt=GenPept&RID=T2J4UBHW012&log$=prottop&blast_rank=11) | nemo like kinase [Homo sapiens] | [20.2](http://blast.ncbi.nlm.nih.gov/Blast.cgi#149408126%23149408126) | 20.2 | 90% | 149 |
| [NP_001139190.1](http://www.ncbi.nlm.nih.gov/entrez/query.fcgi?cmd=Retrieve&db=Protein&list_uids=224591404&dopt=GenPept&RID=T2J4UBHW012&log$=prottop&blast_rank=12) | cancer/testis antigen family 147, member B1 [Homo sapiens] | [20.2](http://blast.ncbi.nlm.nih.gov/Blast.cgi#224591404%23224591404) | 20.2 | 70% | 149 |
| [NP_005915.2](http://www.ncbi.nlm.nih.gov/entrez/query.fcgi?cmd=Retrieve&db=Protein&list_uids=84105335&dopt=GenPept&RID=T2J4UBHW012&log$=prottop&blast_rank=13) | mesenchyme homeobox 2 [Homo sapiens] | [20.2](http://blast.ncbi.nlm.nih.gov/Blast.cgi#84105335%2384105335) | 20.2 | 90% | 149 |
| [NP_055377.1](http://www.ncbi.nlm.nih.gov/entrez/query.fcgi?cmd=Retrieve&db=Protein&list_uids=20070107&dopt=GenPept&RID=T2J4UBHW012&log$=prottop&blast_rank=14) | orthodenticle homeobox 1 [Homo sapiens] | [20.2](http://blast.ncbi.nlm.nih.gov/Blast.cgi#20070107%2320070107) | 20.2 | 90% | 149 |
| [NP_003404.1](http://www.ncbi.nlm.nih.gov/entrez/query.fcgi?cmd=Retrieve&db=Protein&list_uids=4507973&dopt=GenPept&RID=T2J4UBHW012&log$=prottop&blast_rank=15) | zinc finger protein of the cerebellum 3 [Homo sapiens] | [20.2](http://blast.ncbi.nlm.nih.gov/Blast.cgi#4507973%234507973) | 20.2 | 90% | 149 |
| [NP_001628.1](http://www.ncbi.nlm.nih.gov/entrez/query.fcgi?cmd=Retrieve&db=Protein&list_uids=4502115&dopt=GenPept&RID=T2J4UBHW012&log$=prottop&blast_rank=16) | acyloxyacyl hydrolase precursor [Homo sapiens] | [20.2](http://blast.ncbi.nlm.nih.gov/Blast.cgi#4502115%234502115) | 20.2 | 80% | 149 |
| [NP_115701.2](http://www.ncbi.nlm.nih.gov/entrez/query.fcgi?cmd=Retrieve&db=Protein&list_uids=31377656&dopt=GenPept&RID=T2J4UBHW012&log$=prottop&blast_rank=17) | eukaryotic translation initiation factor 1A domain containing [Homo sapiens] | [20.2](http://blast.ncbi.nlm.nih.gov/Blast.cgi#31377656%2331377656) | 20.2 | 70% | 149 |
| [NP_003394.1](http://www.ncbi.nlm.nih.gov/entrez/query.fcgi?cmd=Retrieve&db=Protein&list_uids=4507955&dopt=GenPept&RID=T2J4UBHW012&log$=prottop&blast_rank=18) | YY1 transcription factor [Homo sapiens] | [20.2](http://blast.ncbi.nlm.nih.gov/Blast.cgi#4507955%234507955) | 37.3 | 90% | 149 |
| [NP_001139542.1](http://www.ncbi.nlm.nih.gov/entrez/query.fcgi?cmd=Retrieve&db=Protein&list_uids=225703106&dopt=GenPept&RID=T2J4UBHW012&log$=prottop&blast_rank=19) | tudor domain containing 3 isoform 1 [Homo sapiens] | [19.7](http://blast.ncbi.nlm.nih.gov/Blast.cgi#225703106%23225703106) | 19.7 | 80% | 200 |
| [NP_660276.1](http://www.ncbi.nlm.nih.gov/entrez/query.fcgi?cmd=Retrieve&db=Protein&list_uids=21687161&dopt=GenPept&RID=T2J4UBHW012&log$=prottop&blast_rank=20) | zinc finger protein 625 [Homo sapiens] | [19.7](http://blast.ncbi.nlm.nih.gov/Blast.cgi#21687161%2321687161) | 19.7 | 90% | 200 |
| [NP_110421.1](http://www.ncbi.nlm.nih.gov/entrez/query.fcgi?cmd=Retrieve&db=Protein&list_uids=13540576&dopt=GenPept&RID=T2J4UBHW012&log$=prottop&blast_rank=21) | tudor domain containing 3 isoform 2 [Homo sapiens] >ref|NP_001139543.1| tudor domain containing 3 isoform 2 [Homo sapiens] | [19.7](http://blast.ncbi.nlm.nih.gov/Blast.cgi#13540576%2313540576) | 19.7 | 80% | 200 |
| [NP_004087.1](http://www.ncbi.nlm.nih.gov/entrez/query.fcgi?cmd=Retrieve&db=Protein&list_uids=4758260&dopt=GenPept&RID=T2J4UBHW012&log$=prottop&blast_rank=22) | eukaryotic translation initiation factor 4E binding protein 2 [Homo sapiens] | [19.3](http://blast.ncbi.nlm.nih.gov/Blast.cgi#4758260%234758260) | 19.3 | 70% | 268 |
| [NP_003434.2](http://www.ncbi.nlm.nih.gov/entrez/query.fcgi?cmd=Retrieve&db=Protein&list_uids=167234429&dopt=GenPept&RID=T2J4UBHW012&log$=prottop&blast_rank=23) | zinc finger and BTB domain containing 17 [Homo sapiens] | [19.3](http://blast.ncbi.nlm.nih.gov/Blast.cgi#167234429%23167234429) | 19.3 | 60% | 268 |
| [NP_002929.1](http://www.ncbi.nlm.nih.gov/entrez/query.fcgi?cmd=Retrieve&db=Protein&list_uids=4506561&dopt=GenPept&RID=T2J4UBHW012&log$=prottop&blast_rank=24) | ring finger protein 4 [Homo sapiens] | [19.3](http://blast.ncbi.nlm.nih.gov/Blast.cgi#4506561%234506561) | 19.3 | 70% | 268 |
| [NP_001164100.1](http://www.ncbi.nlm.nih.gov/entrez/query.fcgi?cmd=Retrieve&db=Protein&list_uids=282165704&dopt=GenPept&RID=T2J4UBHW012&log$=prottop&blast_rank=25) | chromodomain helicase DNA binding protein 8 isoform 1 [Homo sapiens] | [18.9](http://blast.ncbi.nlm.nih.gov/Blast.cgi#282165704%23282165704) | 18.9 | 50% | 360 |
| [NP_001120680.1](http://www.ncbi.nlm.nih.gov/entrez/query.fcgi?cmd=Retrieve&db=Protein&list_uids=187761317&dopt=GenPept&RID=T2J4UBHW012&log$=prottop&blast_rank=26) | tet oncogene family member 2 isoform a [Homo sapiens] | [18.9](http://blast.ncbi.nlm.nih.gov/Blast.cgi#187761317%23187761317) | 18.9 | 70% | 360 |
| [NP_065971.2](http://www.ncbi.nlm.nih.gov/entrez/query.fcgi?cmd=Retrieve&db=Protein&list_uids=114326455&dopt=GenPept&RID=T2J4UBHW012&log$=prottop&blast_rank=27) | chromodomain helicase DNA binding protein 8 isoform 2 [Homo sapiens] | [18.9](http://blast.ncbi.nlm.nih.gov/Blast.cgi#114326455%23114326455) | 18.9 | 50% | 360 |
| [NP_598377.3](http://www.ncbi.nlm.nih.gov/entrez/query.fcgi?cmd=Retrieve&db=Protein&list_uids=112789555&dopt=GenPept&RID=T2J4UBHW012&log$=prottop&blast_rank=28) | ADAM metallopeptidase with thrombospondin type 1 motif, 19 preproprotein [Homo sapiens] | [18.9](http://blast.ncbi.nlm.nih.gov/Blast.cgi#112789555%23112789555) | 18.9 | 100% | 360 |
| [NP_005890.2](http://www.ncbi.nlm.nih.gov/entrez/query.fcgi?cmd=Retrieve&db=Protein&list_uids=14110375&dopt=GenPept&RID=T2J4UBHW012&log$=prottop&blast_rank=29) | neighbor of BRCA1 gene 1 [Homo sapiens] >ref|NP_114064.1| neighbor of BRCA1 gene 1 [Homo sapiens] >ref|NP_114068.1| neighbor of BRCA1 gene 1 [Homo sapiens] | [18.9](http://blast.ncbi.nlm.nih.gov/Blast.cgi#14110375%2314110375) | 18.9 | 60% | 360 |
| [NP_001338.2](http://www.ncbi.nlm.nih.gov/entrez/query.fcgi?cmd=Retrieve&db=Protein&list_uids=40806175&dopt=GenPept&RID=T2J4UBHW012&log$=prottop&blast_rank=30) | diacylglycerol kinase, theta [Homo sapiens] | [18.9](http://blast.ncbi.nlm.nih.gov/Blast.cgi#40806175%2340806175) | 18.9 | 90% | 360 |
| [NP_005626.1](http://www.ncbi.nlm.nih.gov/entrez/query.fcgi?cmd=Retrieve&db=Protein&list_uids=5032121&dopt=GenPept&RID=T2J4UBHW012&log$=prottop&blast_rank=31) | synovial sarcoma, X breakpoint 1 [Homo sapiens] | [18.9](http://blast.ncbi.nlm.nih.gov/Blast.cgi#5032121%235032121) | 18.9 | 80% | 360 |
| [NP_001035375.2](http://www.ncbi.nlm.nih.gov/entrez/query.fcgi?cmd=Retrieve&db=Protein&list_uids=256818782&dopt=GenPept&RID=T2J4UBHW012&log$=prottop&blast_rank=32) | PAP associated domain containing 5 isoform b [Homo sapiens] | [18.5](http://blast.ncbi.nlm.nih.gov/Blast.cgi#256818782%23256818782) | 18.5 | 70% | 482 |
| [XP_002347556.1](http://www.ncbi.nlm.nih.gov/entrez/query.fcgi?cmd=Retrieve&db=Protein&list_uids=239747244&dopt=GenPept&RID=T2J4UBHW012&log$=prottop&blast_rank=33) | PREDICTED: hypothetical protein [Homo sapiens] | [18.5](http://blast.ncbi.nlm.nih.gov/Blast.cgi#239747244%23239747244) | 33.5 | 50% | 482 |
| [XP_002342074.1](http://www.ncbi.nlm.nih.gov/entrez/query.fcgi?cmd=Retrieve&db=Protein&list_uids=239741065&dopt=GenPept&RID=T2J4UBHW012&log$=prottop&blast_rank=34) | PREDICTED: hypothetical protein [Homo sapiens] | [18.5](http://blast.ncbi.nlm.nih.gov/Blast.cgi#239741065%23239741065) | 33.5 | 50% | 482 |
| [NP_001035374.2](http://www.ncbi.nlm.nih.gov/entrez/query.fcgi?cmd=Retrieve&db=Protein&list_uids=256818780&dopt=GenPept&RID=T2J4UBHW012&log$=prottop&blast_rank=35) | PAP associated domain containing 5 isoform a [Homo sapiens] | [18.5](http://blast.ncbi.nlm.nih.gov/Blast.cgi#256818780%23256818780) | 18.5 | 70% | 482 |
| [XP_001714195.1](http://www.ncbi.nlm.nih.gov/entrez/query.fcgi?cmd=Retrieve&db=Protein&list_uids=169161033&dopt=GenPept&RID=T2J4UBHW012&log$=prottop&blast_rank=36) | PREDICTED: hypothetical protein [Homo sapiens] | [18.5](http://blast.ncbi.nlm.nih.gov/Blast.cgi#169161033%23169161033) | 33.5 | 50% | 482 |
| [NP_037381.2](http://www.ncbi.nlm.nih.gov/entrez/query.fcgi?cmd=Retrieve&db=Protein&list_uids=156546992&dopt=GenPept&RID=T2J4UBHW012&log$=prottop&blast_rank=37) | zinc finger protein 214 [Homo sapiens] | [18.5](http://blast.ncbi.nlm.nih.gov/Blast.cgi#156546992%23156546992) | 18.5 | 60% | 482 |
| [NP_008890.2](http://www.ncbi.nlm.nih.gov/entrez/query.fcgi?cmd=Retrieve&db=Protein&list_uids=115495000&dopt=GenPept&RID=T2J4UBHW012&log$=prottop&blast_rank=38) | zinc finger protein 17 [Homo sapiens] | [18.5](http://blast.ncbi.nlm.nih.gov/Blast.cgi#115495000%23115495000) | 28.8 | 90% | 482 |
| [NP_115496.2](http://www.ncbi.nlm.nih.gov/entrez/query.fcgi?cmd=Retrieve&db=Protein&list_uids=119120844&dopt=GenPept&RID=T2J4UBHW012&log$=prottop&blast_rank=39) | hypothetical protein LOC84060 [Homo sapiens] | [18.5](http://blast.ncbi.nlm.nih.gov/Blast.cgi#119120844%23119120844) | 18.5 | 60% | 482 |
| [NP_003175.1](http://www.ncbi.nlm.nih.gov/entrez/query.fcgi?cmd=Retrieve&db=Protein&list_uids=4507347&dopt=GenPept&RID=T2J4UBHW012&log$=prottop&blast_rank=40) | TBP-associated factor 2 [Homo sapiens] | [18.5](http://blast.ncbi.nlm.nih.gov/Blast.cgi#4507347%234507347) | 18.5 | 80% | 482 |
| [NP_005536.1](http://www.ncbi.nlm.nih.gov/entrez/query.fcgi?cmd=Retrieve&db=Protein&list_uids=5031809&dopt=GenPept&RID=T2J4UBHW012&log$=prottop&blast_rank=41) | immunoglobulin superfamily containing leucine-rich repeat precursor [Homo sapiens] >ref|NP_958934.1| immunoglobulin superfamily containing leucine-rich repeat precursor [Homo sapiens] | [18.5](http://blast.ncbi.nlm.nih.gov/Blast.cgi#5031809%235031809) | 18.5 | 60% | 482 |
| [NP_055514.3](http://www.ncbi.nlm.nih.gov/entrez/query.fcgi?cmd=Retrieve&db=Protein&list_uids=215820619&dopt=GenPept&RID=T2J4UBHW012&log$=prottop&blast_rank=42) | zinc finger protein 646 [Homo sapiens] | [18.0](http://blast.ncbi.nlm.nih.gov/Blast.cgi#215820619%23215820619) | 18.0 | 70% | 647 |
| [NP_036451.3](http://www.ncbi.nlm.nih.gov/entrez/query.fcgi?cmd=Retrieve&db=Protein&list_uids=153252201&dopt=GenPept&RID=T2J4UBHW012&log$=prottop&blast_rank=43) | solute carrier family 39 (zinc transporter), member 6 isoform 1 [Homo sapiens] | [18.0](http://blast.ncbi.nlm.nih.gov/Blast.cgi#153252201%23153252201) | 48.2 | 80% | 647 |
| [NP_056078.2](http://www.ncbi.nlm.nih.gov/entrez/query.fcgi?cmd=Retrieve&db=Protein&list_uids=119120894&dopt=GenPept&RID=T2J4UBHW012&log$=prottop&blast_rank=44) | Dmx-like 2 [Homo sapiens] | [18.0](http://blast.ncbi.nlm.nih.gov/Blast.cgi#119120894%23119120894) | 18.0 | 70% | 647 |
| [XP_001132826.1](http://www.ncbi.nlm.nih.gov/entrez/query.fcgi?cmd=Retrieve&db=Protein&list_uids=113412985&dopt=GenPept&RID=T2J4UBHW012&log$=prottop&blast_rank=45) | PREDICTED: hypothetical protein [Homo sapiens] >ref|XP_001132908.1| PREDICTED: hypothetical protein [Homo sapiens] >ref|XP_001719219.1| PREDICTED: hypothetical protein [Homo sapiens] | [18.0](http://blast.ncbi.nlm.nih.gov/Blast.cgi#113412985%23113412985) | 18.0 | 70% | 647 |
| [NP_071328.2](http://www.ncbi.nlm.nih.gov/entrez/query.fcgi?cmd=Retrieve&db=Protein&list_uids=21630257&dopt=GenPept&RID=T2J4UBHW012&log$=prottop&blast_rank=46) | Mdm2, transformed 3T3 cell double minute 2, p53 binding protein binding protein [Homo sapiens] | [18.0](http://blast.ncbi.nlm.nih.gov/Blast.cgi#21630257%2321630257) | 18.0 | 90% | 647 |
| [NP_001073275.1](http://www.ncbi.nlm.nih.gov/entrez/query.fcgi?cmd=Retrieve&db=Protein&list_uids=119372298&dopt=GenPept&RID=T2J4UBHW012&log$=prottop&blast_rank=47) | pepsinogen 3, group I precursor [Homo sapiens] | [18.0](http://blast.ncbi.nlm.nih.gov/Blast.cgi#119372298%23119372298) | 18.0 | 70% | 647 |
| [NP_115548.1](http://www.ncbi.nlm.nih.gov/entrez/query.fcgi?cmd=Retrieve&db=Protein&list_uids=79750944&dopt=GenPept&RID=T2J4UBHW012&log$=prottop&blast_rank=48) | ubiquitin specific peptidase 42 [Homo sapiens] | [18.0](http://blast.ncbi.nlm.nih.gov/Blast.cgi#79750944%2379750944) | 18.0 | 60% | 647 |
| [NP_001788.2](http://www.ncbi.nlm.nih.gov/entrez/query.fcgi?cmd=Retrieve&db=Protein&list_uids=16306532&dopt=GenPept&RID=T2J4UBHW012&log$=prottop&blast_rank=49) | cadherin 11, type 2 preproprotein [Homo sapiens] | [18.0](http://blast.ncbi.nlm.nih.gov/Blast.cgi#16306532%2316306532) | 18.0 | 50% | 647 |
| [NP_055039.1](http://www.ncbi.nlm.nih.gov/entrez/query.fcgi?cmd=Retrieve&db=Protein&list_uids=23943854&dopt=GenPept&RID=T2J4UBHW012&log$=prottop&blast_rank=50) | pepsinogen 5, group I precursor [Homo sapiens] | [18.0](http://blast.ncbi.nlm.nih.gov/Blast.cgi#23943854%2323943854) | 18.0 | 70% | 647 |
| [NP_004045.1](http://www.ncbi.nlm.nih.gov/entrez/query.fcgi?cmd=Retrieve&db=Protein&list_uids=4757888&dopt=GenPept&RID=T2J4UBHW012&log$=prottop&blast_rank=51) | complement component 3a receptor 1 [Homo sapiens] | [18.0](http://blast.ncbi.nlm.nih.gov/Blast.cgi#4757888%234757888) | 18.0 | 50% | 647 |
| [NP_004517.2](http://www.ncbi.nlm.nih.gov/entrez/query.fcgi?cmd=Retrieve&db=Protein&list_uids=33356547&dopt=GenPept&RID=T2J4UBHW012&log$=prottop&blast_rank=52) | minichromosome maintenance complex component 2 [Homo sapiens] | [18.0](http://blast.ncbi.nlm.nih.gov/Blast.cgi#33356547%2333356547) | 18.0 | 60% | 647 |
| [NP_001073276.1](http://www.ncbi.nlm.nih.gov/entrez/query.fcgi?cmd=Retrieve&db=Protein&list_uids=119372302&dopt=GenPept&RID=T2J4UBHW012&log$=prottop&blast_rank=53) | pepsinogen 4, group I precursor [Homo sapiens] | [18.0](http://blast.ncbi.nlm.nih.gov/Blast.cgi#119372302%23119372302) | 18.0 | 70% | 647 |
| [NP_114096.1](http://www.ncbi.nlm.nih.gov/entrez/query.fcgi?cmd=Retrieve&db=Protein&list_uids=13994236&dopt=GenPept&RID=T2J4UBHW012&log$=prottop&blast_rank=54) | cat eye syndrome chromosome region, candidate 6 isoform a [Homo sapiens] | [18.0](http://blast.ncbi.nlm.nih.gov/Blast.cgi#13994236%2313994236) | 18.0 | 80% | 647 |
| [NP_005793.2](http://www.ncbi.nlm.nih.gov/entrez/query.fcgi?cmd=Retrieve&db=Protein&list_uids=40805104&dopt=GenPept&RID=T2J4UBHW012&log$=prottop&blast_rank=55) | topoisomerase I binding, arginine/serine-rich [Homo sapiens] | [18.0](http://blast.ncbi.nlm.nih.gov/Blast.cgi#40805104%2340805104) | 18.0 | 90% | 647 |
| [XP_002345215.1](http://www.ncbi.nlm.nih.gov/entrez/query.fcgi?cmd=Retrieve&db=Protein&list_uids=239757235&dopt=GenPept&RID=T2J4UBHW012&log$=prottop&blast_rank=56) | PREDICTED: hypothetical protein [Homo sapiens] | [17.6](http://blast.ncbi.nlm.nih.gov/Blast.cgi#239757235%23239757235) | 33.9 | 50% | 868 |
| [XP_002345168.1](http://www.ncbi.nlm.nih.gov/entrez/query.fcgi?cmd=Retrieve&db=Protein&list_uids=239757110&dopt=GenPept&RID=T2J4UBHW012&log$=prottop&blast_rank=57) | PREDICTED: hypothetical protein [Homo sapiens] | [17.6](http://blast.ncbi.nlm.nih.gov/Blast.cgi#239757110%23239757110) | 17.6 | 50% | 868 |
| [XP_001713987.2](http://www.ncbi.nlm.nih.gov/entrez/query.fcgi?cmd=Retrieve&db=Protein&list_uids=239757043&dopt=GenPept&RID=T2J4UBHW012&log$=prottop&blast_rank=58) | PREDICTED: functional smad suppressing element 18 [Homo sapiens] | [17.6](http://blast.ncbi.nlm.nih.gov/Blast.cgi#239757043%23239757043) | 17.6 | 40% | 868 |
| [XP_002344829.1](http://www.ncbi.nlm.nih.gov/entrez/query.fcgi?cmd=Retrieve&db=Protein&list_uids=239756161&dopt=GenPept&RID=T2J4UBHW012&log$=prottop&blast_rank=59) | PREDICTED: hypothetical protein [Homo sapiens] | [17.6](http://blast.ncbi.nlm.nih.gov/Blast.cgi#239756161%23239756161) | 17.6 | 40% | 868 |
| [XP_002344649.1](http://www.ncbi.nlm.nih.gov/entrez/query.fcgi?cmd=Retrieve&db=Protein&list_uids=239755665&dopt=GenPept&RID=T2J4UBHW012&log$=prottop&blast_rank=60) | PREDICTED: hypothetical protein [Homo sapiens] | [17.6](http://blast.ncbi.nlm.nih.gov/Blast.cgi#239755665%23239755665) | 17.6 | 40% | 868 |
| [XP_002345619.1](http://www.ncbi.nlm.nih.gov/entrez/query.fcgi?cmd=Retrieve&db=Protein&list_uids=239753314&dopt=GenPept&RID=T2J4UBHW012&log$=prottop&blast_rank=61) | PREDICTED: similar to RIKEN cDNA C230030N03 [Homo sapiens] | [17.6](http://blast.ncbi.nlm.nih.gov/Blast.cgi#239753314%23239753314) | 17.6 | 40% | 868 |
| [XP_002345386.1](http://www.ncbi.nlm.nih.gov/entrez/query.fcgi?cmd=Retrieve&db=Protein&list_uids=239752812&dopt=GenPept&RID=T2J4UBHW012&log$=prottop&blast_rank=62) | PREDICTED: hypothetical protein [Homo sapiens] | [17.6](http://blast.ncbi.nlm.nih.gov/Blast.cgi#239752812%23239752812) | 17.6 | 40% | 868 |
| [XP_002347981.1](http://www.ncbi.nlm.nih.gov/entrez/query.fcgi?cmd=Retrieve&db=Protein&list_uids=239751744&dopt=GenPept&RID=T2J4UBHW012&log$=prottop&blast_rank=63) | PREDICTED: hypothetical protein [Homo sapiens] | [17.6](http://blast.ncbi.nlm.nih.gov/Blast.cgi#239751744%23239751744) | 33.9 | 50% | 868 |
| [XP_002347506.1](http://www.ncbi.nlm.nih.gov/entrez/query.fcgi?cmd=Retrieve&db=Protein&list_uids=239750685&dopt=GenPept&RID=T2J4UBHW012&log$=prottop&blast_rank=64) | PREDICTED: hypothetical protein XP_002347506 [Homo sapiens] | [17.6](http://blast.ncbi.nlm.nih.gov/Blast.cgi#239750685%23239750685) | 17.6 | 40% | 868 |
| [XP_002347243.1](http://www.ncbi.nlm.nih.gov/entrez/query.fcgi?cmd=Retrieve&db=Protein&list_uids=239750040&dopt=GenPept&RID=T2J4UBHW012&log$=prottop&blast_rank=65) | PREDICTED: similar to tripartite motif-containing 53 [Homo sapiens] >ref|XP_002344594.1| PREDICTED: similar to tripartite motif-containing 53 [Homo sapiens] | [17.6](http://blast.ncbi.nlm.nih.gov/Blast.cgi#239750040%23239750040) | 17.6 | 40% | 868 |
| [XP_001715271.2](http://www.ncbi.nlm.nih.gov/entrez/query.fcgi?cmd=Retrieve&db=Protein&list_uids=239746067&dopt=GenPept&RID=T2J4UBHW012&log$=prottop&blast_rank=66) | PREDICTED: functional smad suppressing element 18 [Homo sapiens] >ref|XP_001714189.2| PREDICTED: functional smad suppressing element 18 [Homo sapiens] | [17.6](http://blast.ncbi.nlm.nih.gov/Blast.cgi#239746067%23239746067) | 17.6 | 40% | 868 |
| [XP_002344167.1](http://www.ncbi.nlm.nih.gov/entrez/query.fcgi?cmd=Retrieve&db=Protein&list_uids=239745650&dopt=GenPept&RID=T2J4UBHW012&log$=prottop&blast_rank=67) | PREDICTED: hypothetical protein XP_002344167 [Homo sapiens] >ref|XP_002347739.1| PREDICTED: hypothetical protein XP_002347739 [Homo sapiens] >ref|XP_002344975.1| PREDICTED: hypothetical protein [Homo sapiens] | [17.6](http://blast.ncbi.nlm.nih.gov/Blast.cgi#239745650%23239745650) | 17.6 | 40% | 868 |
| [XP_002343325.1](http://www.ncbi.nlm.nih.gov/entrez/query.fcgi?cmd=Retrieve&db=Protein&list_uids=239744995&dopt=GenPept&RID=T2J4UBHW012&log$=prottop&blast_rank=68) | PREDICTED: hypothetical protein XP_002343325 [Homo sapiens] | [17.6](http://blast.ncbi.nlm.nih.gov/Blast.cgi#239744995%23239744995) | 17.6 | 40% | 868 |
| [XP_002342136.1](http://www.ncbi.nlm.nih.gov/entrez/query.fcgi?cmd=Retrieve&db=Protein&list_uids=239741215&dopt=GenPept&RID=T2J4UBHW012&log$=prottop&blast_rank=69) | PREDICTED: hypothetical protein XP_002342136 [Homo sapiens] >ref|XP_002346575.1| PREDICTED: hypothetical protein XP_002346575 [Homo sapiens] >ref|XP_002345398.1| PREDICTED: similar to NADH dehydrogenase [ubiquinone] iron-sulfur protein 5 [Homo sapiens] | [17.6](http://blast.ncbi.nlm.nih.gov/Blast.cgi#239741215%23239741215) | 17.6 | 40% | 868 |
| [XP_002342125.1](http://www.ncbi.nlm.nih.gov/entrez/query.fcgi?cmd=Retrieve&db=Protein&list_uids=239741171&dopt=GenPept&RID=T2J4UBHW012&log$=prottop&blast_rank=70) | PREDICTED: hypothetical protein XP_002342125 [Homo sapiens] >ref|XP_002346427.1| PREDICTED: hypothetical protein XP_002346427 [Homo sapiens] | [17.6](http://blast.ncbi.nlm.nih.gov/Blast.cgi#239741171%23239741171) | 17.6 | 40% | 868 |
| [NP_001153509.1](http://www.ncbi.nlm.nih.gov/entrez/query.fcgi?cmd=Retrieve&db=Protein&list_uids=237649111&dopt=GenPept&RID=T2J4UBHW012&log$=prottop&blast_rank=71) | Rho-related BTB domain containing 2 isoform 2 [Homo sapiens] | [17.6](http://blast.ncbi.nlm.nih.gov/Blast.cgi#237649111%23237649111) | 17.6 | 90% | 868 |
| [NP_001153508.1](http://www.ncbi.nlm.nih.gov/entrez/query.fcgi?cmd=Retrieve&db=Protein&list_uids=237649109&dopt=GenPept&RID=T2J4UBHW012&log$=prottop&blast_rank=72) | Rho-related BTB domain containing 2 isoform 1 [Homo sapiens] | [17.6](http://blast.ncbi.nlm.nih.gov/Blast.cgi#237649109%23237649109) | 17.6 | 90% | 868 |
| [NP_660334.3](http://www.ncbi.nlm.nih.gov/entrez/query.fcgi?cmd=Retrieve&db=Protein&list_uids=223972645&dopt=GenPept&RID=T2J4UBHW012&log$=prottop&blast_rank=73) | zinc finger protein 509 [Homo sapiens] | [17.6](http://blast.ncbi.nlm.nih.gov/Blast.cgi#223972645%23223972645) | 17.6 | 40% | 868 |
| [NP_001122311.1](http://www.ncbi.nlm.nih.gov/entrez/query.fcgi?cmd=Retrieve&db=Protein&list_uids=192807298&dopt=GenPept&RID=T2J4UBHW012&log$=prottop&blast_rank=74) | calcium channel, voltage-dependent, L type, alpha 1D subunit isoform c [Homo sapiens] | [17.6](http://blast.ncbi.nlm.nih.gov/Blast.cgi#192807298%23192807298) | 17.6 | 50% | 868 |
| [NP_006009.2](http://www.ncbi.nlm.nih.gov/entrez/query.fcgi?cmd=Retrieve&db=Protein&list_uids=157738694&dopt=GenPept&RID=T2J4UBHW012&log$=prottop&blast_rank=75) | G protein-coupled receptor 109B [Homo sapiens] | [17.6](http://blast.ncbi.nlm.nih.gov/Blast.cgi#157738694%23157738694) | 17.6 | 40% | 868 |
| [NP_001099007.1](http://www.ncbi.nlm.nih.gov/entrez/query.fcgi?cmd=Retrieve&db=Protein&list_uids=157676340&dopt=GenPept&RID=T2J4UBHW012&log$=prottop&blast_rank=76) | zinc finger protein 142 [Homo sapiens] | [17.6](http://blast.ncbi.nlm.nih.gov/Blast.cgi#157676340%23157676340) | 17.6 | 40% | 868 |
| [NP_001098983.1](http://www.ncbi.nlm.nih.gov/entrez/query.fcgi?cmd=Retrieve&db=Protein&list_uids=157502179&dopt=GenPept&RID=T2J4UBHW012&log$=prottop&blast_rank=77) | tetratricopeptide repeat domain 21A isoform 1 [Homo sapiens] | [17.6](http://blast.ncbi.nlm.nih.gov/Blast.cgi#157502179%23157502179) | 31.8 | 60% | 868 |
| [NP_665698.2](http://www.ncbi.nlm.nih.gov/entrez/query.fcgi?cmd=Retrieve&db=Protein&list_uids=157502177&dopt=GenPept&RID=T2J4UBHW012&log$=prottop&blast_rank=78) | tetratricopeptide repeat domain 21A isoform 2 [Homo sapiens] | [17.6](http://blast.ncbi.nlm.nih.gov/Blast.cgi#157502177%23157502177) | 31.8 | 60% | 868 |
| [NP_001138599.1](http://www.ncbi.nlm.nih.gov/entrez/query.fcgi?cmd=Retrieve&db=Protein&list_uids=223468634&dopt=GenPept&RID=T2J4UBHW012&log$=prottop&blast_rank=79) | envoplakin-like [Homo sapiens] | [17.6](http://blast.ncbi.nlm.nih.gov/Blast.cgi#223468634%23223468634) | 17.6 | 40% | 868 |
| [NP_001129595.1](http://www.ncbi.nlm.nih.gov/entrez/query.fcgi?cmd=Retrieve&db=Protein&list_uids=209915547&dopt=GenPept&RID=T2J4UBHW012&log$=prottop&blast_rank=80) | hypothetical protein LOC55719 isoform 2 [Homo sapiens] | [17.6](http://blast.ncbi.nlm.nih.gov/Blast.cgi#209915547%23209915547) | 17.6 | 40% | 868 |
| [NP_079363.2](http://www.ncbi.nlm.nih.gov/entrez/query.fcgi?cmd=Retrieve&db=Protein&list_uids=156151386&dopt=GenPept&RID=T2J4UBHW012&log$=prottop&blast_rank=81) | cell wall biogenesis 43 C-terminal homolog [Homo sapiens] | [17.6](http://blast.ncbi.nlm.nih.gov/Blast.cgi#156151386%23156151386) | 17.6 | 90% | 868 |
| [NP_001092093.1](http://www.ncbi.nlm.nih.gov/entrez/query.fcgi?cmd=Retrieve&db=Protein&list_uids=148833506&dopt=GenPept&RID=T2J4UBHW012&log$=prottop&blast_rank=82) | obscurin, cytoskeletal calmodulin and titin-interacting RhoGEF isoform b [Homo sapiens] | [17.6](http://blast.ncbi.nlm.nih.gov/Blast.cgi#148833506%23148833506) | 17.6 | 40% | 868 |
| [NP_000615.3](http://www.ncbi.nlm.nih.gov/entrez/query.fcgi?cmd=Retrieve&db=Protein&list_uids=194018472&dopt=GenPept&RID=T2J4UBHW012&log$=prottop&blast_rank=83) | serine (or cysteine) proteinase inhibitor, clade A (alpha-1 antiproteinase, antitrypsin), member 5 [Homo sapiens] | [17.6](http://blast.ncbi.nlm.nih.gov/Blast.cgi#194018472%23194018472) | 17.6 | 40% | 868 |
| [NP_689558.4](http://www.ncbi.nlm.nih.gov/entrez/query.fcgi?cmd=Retrieve&db=Protein&list_uids=124517699&dopt=GenPept&RID=T2J4UBHW012&log$=prottop&blast_rank=84) | ankyrin repeat domain 13B [Homo sapiens] | [17.6](http://blast.ncbi.nlm.nih.gov/Blast.cgi#124517699%23124517699) | 17.6 | 40% | 868 |
| [NP_115829.1](http://www.ncbi.nlm.nih.gov/entrez/query.fcgi?cmd=Retrieve&db=Protein&list_uids=149192841&dopt=GenPept&RID=T2J4UBHW012&log$=prottop&blast_rank=85) | zinc finger protein 527 [Homo sapiens] | [17.6](http://blast.ncbi.nlm.nih.gov/Blast.cgi#149192841%23149192841) | 17.6 | 40% | 868 |
| [NP_775858.2](http://www.ncbi.nlm.nih.gov/entrez/query.fcgi?cmd=Retrieve&db=Protein&list_uids=269847040&dopt=GenPept&RID=T2J4UBHW012&log$=prottop&blast_rank=86) | REST corepressor 2 [Homo sapiens] | [17.6](http://blast.ncbi.nlm.nih.gov/Blast.cgi#269847040%23269847040) | 17.6 | 40% | 868 |
| [XP_001726377.1](http://www.ncbi.nlm.nih.gov/entrez/query.fcgi?cmd=Retrieve&db=Protein&list_uids=169168879&dopt=GenPept&RID=T2J4UBHW012&log$=prottop&blast_rank=87) | PREDICTED: similar to hCG34808 [Homo sapiens] >ref|XP_001723072.1| PREDICTED: similar to hCG34808 [Homo sapiens] >ref|XP_001725236.1| PREDICTED: similar to hCG34808 [Homo sapiens] | [17.6](http://blast.ncbi.nlm.nih.gov/Blast.cgi#169168879%23169168879) | 17.6 | 40% | 868 |
| [NP_076987.3](http://www.ncbi.nlm.nih.gov/entrez/query.fcgi?cmd=Retrieve&db=Protein&list_uids=284807142&dopt=GenPept&RID=T2J4UBHW012&log$=prottop&blast_rank=88) | proline rich gamma-carboxyglutamic acid protein 3 precursor [Homo sapiens] | [17.6](http://blast.ncbi.nlm.nih.gov/Blast.cgi#284807142%23284807142) | 17.6 | 40% | 868 |
| [NP_803237.3](http://www.ncbi.nlm.nih.gov/entrez/query.fcgi?cmd=Retrieve&db=Protein&list_uids=116875848&dopt=GenPept&RID=T2J4UBHW012&log$=prottop&blast_rank=89) | KIAA1946 [Homo sapiens] | [17.6](http://blast.ncbi.nlm.nih.gov/Blast.cgi#116875848%23116875848) | 17.6 | 40% | 868 |
| [NP_001070729.1](http://www.ncbi.nlm.nih.gov/entrez/query.fcgi?cmd=Retrieve&db=Protein&list_uids=116256445&dopt=GenPept&RID=T2J4UBHW012&log$=prottop&blast_rank=90) | nuclear receptor co-repressor 2 isoform 2 [Homo sapiens] | [17.6](http://blast.ncbi.nlm.nih.gov/Blast.cgi#116256445%23116256445) | 17.6 | 40% | 868 |
| [NP_006303.3](http://www.ncbi.nlm.nih.gov/entrez/query.fcgi?cmd=Retrieve&db=Protein&list_uids=116256453&dopt=GenPept&RID=T2J4UBHW012&log$=prottop&blast_rank=91) | nuclear receptor co-repressor 2 isoform 1 [Homo sapiens] | [17.6](http://blast.ncbi.nlm.nih.gov/Blast.cgi#116256453%23116256453) | 17.6 | 40% | 868 |
| [NP_001122312.1](http://www.ncbi.nlm.nih.gov/entrez/query.fcgi?cmd=Retrieve&db=Protein&list_uids=192807300&dopt=GenPept&RID=T2J4UBHW012&log$=prottop&blast_rank=92) | calcium channel, voltage-dependent, L type, alpha 1D subunit isoform b [Homo sapiens] | [17.6](http://blast.ncbi.nlm.nih.gov/Blast.cgi#192807300%23192807300) | 17.6 | 50% | 868 |
| [NP_078966.2](http://www.ncbi.nlm.nih.gov/entrez/query.fcgi?cmd=Retrieve&db=Protein&list_uids=83367077&dopt=GenPept&RID=T2J4UBHW012&log$=prottop&blast_rank=93) | mucin 16 [Homo sapiens] | [17.6](http://blast.ncbi.nlm.nih.gov/Blast.cgi#83367077%2383367077) | 17.6 | 40% | 868 |
| [NP_001129973.1](http://www.ncbi.nlm.nih.gov/entrez/query.fcgi?cmd=Retrieve&db=Protein&list_uids=211058421&dopt=GenPept&RID=T2J4UBHW012&log$=prottop&blast_rank=94) | zinc finger protein 844 [Homo sapiens] | [17.6](http://blast.ncbi.nlm.nih.gov/Blast.cgi#211058421%23211058421) | 17.6 | 80% | 868 |
| [NP_008816.3](http://www.ncbi.nlm.nih.gov/entrez/query.fcgi?cmd=Retrieve&db=Protein&list_uids=118498345&dopt=GenPept&RID=T2J4UBHW012&log$=prottop&blast_rank=95) | zinc finger homeobox 3 isoform A [Homo sapiens] | [17.6](http://blast.ncbi.nlm.nih.gov/Blast.cgi#118498345%23118498345) | 17.6 | 40% | 868 |
| [NP_003738.2](http://www.ncbi.nlm.nih.gov/entrez/query.fcgi?cmd=Retrieve&db=Protein&list_uids=87239981&dopt=GenPept&RID=T2J4UBHW012&log$=prottop&blast_rank=96) | tankyrase, TRF1-interacting ankyrin-related ADP-ribose polymerase [Homo sapiens] | [17.6](http://blast.ncbi.nlm.nih.gov/Blast.cgi#87239981%2387239981) | 17.6 | 50% | 868 |
| [NP_001028227.1](http://www.ncbi.nlm.nih.gov/entrez/query.fcgi?cmd=Retrieve&db=Protein&list_uids=74272280&dopt=GenPept&RID=T2J4UBHW012&log$=prottop&blast_rank=97) | aryl hydrocarbon receptor interacting protein-like 1 isoform 3 [Homo sapiens] | [17.6](http://blast.ncbi.nlm.nih.gov/Blast.cgi#74272280%2374272280) | 17.6 | 40% | 868 |
| [NP_001527.3](http://www.ncbi.nlm.nih.gov/entrez/query.fcgi?cmd=Retrieve&db=Protein&list_uids=154759421&dopt=GenPept&RID=T2J4UBHW012&log$=prottop&blast_rank=98) | HMT1 hnRNP methyltransferase-like 2 isoform 1 [Homo sapiens] | [17.6](http://blast.ncbi.nlm.nih.gov/Blast.cgi#154759421%23154759421) | 17.6 | 40% | 868 |
| [NP_071414.2](http://www.ncbi.nlm.nih.gov/entrez/query.fcgi?cmd=Retrieve&db=Protein&list_uids=209364544&dopt=GenPept&RID=T2J4UBHW012&log$=prottop&blast_rank=99) | calsyntenin 2 precursor [Homo sapiens] | [17.6](http://blast.ncbi.nlm.nih.gov/Blast.cgi#209364544%23209364544) | 17.6 | 40% | 868 |
| [NP_938074.2](http://www.ncbi.nlm.nih.gov/entrez/query.fcgi?cmd=Retrieve&db=Protein&list_uids=151301219&dopt=GenPept&RID=T2J4UBHW012&log$=prottop&blast_rank=100) | HMT1 hnRNP methyltransferase-like 2 isoform 3 [Homo sapiens] | [17.6](http://blast.ncbi.nlm.nih.gov/Blast.cgi#151301219%23151301219) | 17.6 | 40% | 868 |

| **Accession** | **Proteins with a match to SNVRSFDNPI peptide** | **[Max score](http://blast.ncbi.nlm.nih.gov/Blast.cgi?CMD=Get&ALIGNMENTS=100&ALIGNMENT_VIEW=Pairwise&CDD_SEARCH_STATE=1&DATABASE_SORT=0&DESCRIPTIONS=100&ENTREZ_QUERY=txid9606 %5BORGN%5D&FIRST_QUERY_NUM=0&FORMAT_OBJECT=Alignment&FORMAT_PAGE_TARGET=&FORMAT_TYPE=HTML&GET_SEQUENCE=yes&I_THRESH=&MASK_CHAR=2&MASK_COLOR=1&NEW_DESIGN=on&NEW_VIEW=yes&NUM_OVERVIEW=100&OLD_BLAST=false&PAGE=Proteins&QUERY_INDEX=0&QUERY_NUMBER=0&RESULTS_PAGE_TARGET=&RID=T2JDDAMS01S&SHOW_LINKOUT=yes&SHOW_OVERVIEW=yes&STEP_NUMBER=&WORD_SIZE=2&DISPLAY_SORT=1&HSP_SORT=1" \l "sort_mark)** | **[Total score](http://blast.ncbi.nlm.nih.gov/Blast.cgi?CMD=Get&ALIGNMENTS=100&ALIGNMENT_VIEW=Pairwise&CDD_SEARCH_STATE=1&DATABASE_SORT=0&DESCRIPTIONS=100&ENTREZ_QUERY=txid9606 %5BORGN%5D&FIRST_QUERY_NUM=0&FORMAT_OBJECT=Alignment&FORMAT_PAGE_TARGET=&FORMAT_TYPE=HTML&GET_SEQUENCE=yes&I_THRESH=&MASK_CHAR=2&MASK_COLOR=1&NEW_DESIGN=on&NEW_VIEW=yes&NUM_OVERVIEW=100&OLD_BLAST=false&PAGE=Proteins&QUERY_INDEX=0&QUERY_NUMBER=0&RESULTS_PAGE_TARGET=&RID=T2JDDAMS01S&SHOW_LINKOUT=yes&SHOW_OVERVIEW=yes&STEP_NUMBER=&WORD_SIZE=2&DISPLAY_SORT=2&HSP_SORT=1" \l "sort_mark)** | **[Query coverage](http://blast.ncbi.nlm.nih.gov/Blast.cgi?CMD=Get&ALIGNMENTS=100&ALIGNMENT_VIEW=Pairwise&CDD_SEARCH_STATE=1&DATABASE_SORT=0&DESCRIPTIONS=100&ENTREZ_QUERY=txid9606 %5BORGN%5D&FIRST_QUERY_NUM=0&FORMAT_OBJECT=Alignment&FORMAT_PAGE_TARGET=&FORMAT_TYPE=HTML&GET_SEQUENCE=yes&I_THRESH=&MASK_CHAR=2&MASK_COLOR=1&NEW_DESIGN=on&NEW_VIEW=yes&NUM_OVERVIEW=100&OLD_BLAST=false&PAGE=Proteins&QUERY_INDEX=0&QUERY_NUMBER=0&RESULTS_PAGE_TARGET=&RID=T2JDDAMS01S&SHOW_LINKOUT=yes&SHOW_OVERVIEW=yes&STEP_NUMBER=&WORD_SIZE=2&DISPLAY_SORT=4&HSP_SORT=0" \l "sort_mark)** | **[E value](http://blast.ncbi.nlm.nih.gov/Blast.cgi?CMD=Get&ALIGNMENTS=100&ALIGNMENT_VIEW=Pairwise&CDD_SEARCH_STATE=1&DATABASE_SORT=0&DESCRIPTIONS=100&ENTREZ_QUERY=txid9606 %5BORGN%5D&FIRST_QUERY_NUM=0&FORMAT_OBJECT=Alignment&FORMAT_PAGE_TARGET=&FORMAT_TYPE=HTML&GET_SEQUENCE=yes&I_THRESH=&MASK_CHAR=2&MASK_COLOR=1&NEW_DESIGN=on&NEW_VIEW=yes&NUM_OVERVIEW=100&OLD_BLAST=false&PAGE=Proteins&QUERY_INDEX=0&QUERY_NUMBER=0&RESULTS_PAGE_TARGET=&RID=T2JDDAMS01S&SHOW_LINKOUT=yes&SHOW_OVERVIEW=yes&STEP_NUMBER=&WORD_SIZE=2&DISPLAY_SORT=0&HSP_SORT=0" \l "sort_mark)** |
| --- | --- | --- | --- | --- | --- |
| [NP_055093.2](http://www.ncbi.nlm.nih.gov/entrez/query.fcgi?cmd=Retrieve&db=Protein&list_uids=31541941&dopt=GenPept&RID=T2JDDAMS01S&log$=prottop&blast_rank=1) | heat shock 70kDa protein 4-like [Homo sapiens] | [24.0](http://blast.ncbi.nlm.nih.gov/Blast.cgi#31541941%2331541941) | 24.0 | 70% | 11 |
| [NP_005199.2](http://www.ncbi.nlm.nih.gov/entrez/query.fcgi?cmd=Retrieve&db=Protein&list_uids=12056461&dopt=GenPept&RID=T2JDDAMS01S&log$=prottop&blast_rank=2) | crystallin, beta A3 [Homo sapiens] | [21.4](http://blast.ncbi.nlm.nih.gov/Blast.cgi#12056461%2312056461) | 36.9 | 70% | 62 |
| [NP_001010872.1](http://www.ncbi.nlm.nih.gov/entrez/query.fcgi?cmd=Retrieve&db=Protein&list_uids=61676089&dopt=GenPept&RID=T2JDDAMS01S&log$=prottop&blast_rank=3) | hypothetical protein LOC222584 [Homo sapiens] | [21.0](http://blast.ncbi.nlm.nih.gov/Blast.cgi#61676089%2361676089) | 21.0 | 100% | 83 |
| [XP_002343933.1](http://www.ncbi.nlm.nih.gov/entrez/query.fcgi?cmd=Retrieve&db=Protein&list_uids=239747180&dopt=GenPept&RID=T2JDDAMS01S&log$=prottop&blast_rank=4) | PREDICTED: hypothetical protein XP_002343933 [Homo sapiens] | [20.6](http://blast.ncbi.nlm.nih.gov/Blast.cgi#239747180%23239747180) | 20.6 | 80% | 111 |
| [NP_001138678.1](http://www.ncbi.nlm.nih.gov/entrez/query.fcgi?cmd=Retrieve&db=Protein&list_uids=223633988&dopt=GenPept&RID=T2JDDAMS01S&log$=prottop&blast_rank=5) | hypothetical protein LOC85379 [Homo sapiens] | [20.6](http://blast.ncbi.nlm.nih.gov/Blast.cgi#223633988%23223633988) | 20.6 | 80% | 111 |
| [NP_001035904.1](http://www.ncbi.nlm.nih.gov/entrez/query.fcgi?cmd=Retrieve&db=Protein&list_uids=109627656&dopt=GenPept&RID=T2JDDAMS01S&log$=prottop&blast_rank=6) | short long palate, lung and nasal epithelium carcinoma associated 3 isoform 2 [Homo sapiens] | [20.6](http://blast.ncbi.nlm.nih.gov/Blast.cgi#109627656%23109627656) | 20.6 | 70% | 111 |
| [NP_066938.2](http://www.ncbi.nlm.nih.gov/entrez/query.fcgi?cmd=Retrieve&db=Protein&list_uids=32261332&dopt=GenPept&RID=T2JDDAMS01S&log$=prottop&blast_rank=7) | seizure related 6 homolog (mouse)-like precursor [Homo sapiens] | [20.6](http://blast.ncbi.nlm.nih.gov/Blast.cgi#32261332%2332261332) | 35.2 | 60% | 111 |
| [NP_443128.2](http://www.ncbi.nlm.nih.gov/entrez/query.fcgi?cmd=Retrieve&db=Protein&list_uids=92110053&dopt=GenPept&RID=T2JDDAMS01S&log$=prottop&blast_rank=8) | CUB and Sushi multiple domains 2 [Homo sapiens] | [20.2](http://blast.ncbi.nlm.nih.gov/Blast.cgi#92110053%2392110053) | 30.1 | 90% | 149 |
| [NP_000829.2](http://www.ncbi.nlm.nih.gov/entrez/query.fcgi?cmd=Retrieve&db=Protein&list_uids=166999098&dopt=GenPept&RID=T2JDDAMS01S&log$=prottop&blast_rank=9) | glutamate receptor, metabotropic 1 isoform alpha precursor [Homo sapiens] | [20.2](http://blast.ncbi.nlm.nih.gov/Blast.cgi#166999098%23166999098) | 35.6 | 70% | 149 |
| [NP_065825.1](http://www.ncbi.nlm.nih.gov/entrez/query.fcgi?cmd=Retrieve&db=Protein&list_uids=30348954&dopt=GenPept&RID=T2JDDAMS01S&log$=prottop&blast_rank=10) | mindbomb homolog 1 [Homo sapiens] | [20.2](http://blast.ncbi.nlm.nih.gov/Blast.cgi#30348954%2330348954) | 20.2 | 70% | 149 |
| [NP_001107801.1](http://www.ncbi.nlm.nih.gov/entrez/query.fcgi?cmd=Retrieve&db=Protein&list_uids=166999223&dopt=GenPept&RID=T2JDDAMS01S&log$=prottop&blast_rank=11) | glutamate receptor, metabotropic 1 isoform beta precursor [Homo sapiens] | [20.2](http://blast.ncbi.nlm.nih.gov/Blast.cgi#166999223%23166999223) | 35.6 | 70% | 149 |
| [NP_001166119.1](http://www.ncbi.nlm.nih.gov/entrez/query.fcgi?cmd=Retrieve&db=Protein&list_uids=289547602&dopt=GenPept&RID=T2JDDAMS01S&log$=prottop&blast_rank=12) | CUG-BP- and ETR-3-like factor 3 isoform 2 [Homo sapiens] | [19.7](http://blast.ncbi.nlm.nih.gov/Blast.cgi#289547602%23289547602) | 19.7 | 50% | 200 |
| [NP_001166155.1](http://www.ncbi.nlm.nih.gov/entrez/query.fcgi?cmd=Retrieve&db=Protein&list_uids=289547741&dopt=GenPept&RID=T2JDDAMS01S&log$=prottop&blast_rank=13) | CUG-BP- and ETR-3-like factor 6 isoform 2 [Homo sapiens] | [19.7](http://blast.ncbi.nlm.nih.gov/Blast.cgi#289547741%23289547741) | 19.7 | 50% | 200 |
| [NP_001166156.1](http://www.ncbi.nlm.nih.gov/entrez/query.fcgi?cmd=Retrieve&db=Protein&list_uids=289547743&dopt=GenPept&RID=T2JDDAMS01S&log$=prottop&blast_rank=14) | CUG-BP- and ETR-3-like factor 6 isoform 3 [Homo sapiens] | [19.7](http://blast.ncbi.nlm.nih.gov/Blast.cgi#289547743%23289547743) | 19.7 | 50% | 200 |
| [NP_001129120.1](http://www.ncbi.nlm.nih.gov/entrez/query.fcgi?cmd=Retrieve&db=Protein&list_uids=208431815&dopt=GenPept&RID=T2JDDAMS01S&log$=prottop&blast_rank=15) | protein tyrosine phosphatase, receptor type, K isoform a [Homo sapiens] | [19.7](http://blast.ncbi.nlm.nih.gov/Blast.cgi#208431815%23208431815) | 29.7 | 90% | 200 |
| [NP_001020259.1](http://www.ncbi.nlm.nih.gov/entrez/query.fcgi?cmd=Retrieve&db=Protein&list_uids=187761301&dopt=GenPept&RID=T2JDDAMS01S&log$=prottop&blast_rank=16) | CUG-BP- and ETR-3-like factor 4 isoform 3 [Homo sapiens] | [19.7](http://blast.ncbi.nlm.nih.gov/Blast.cgi#187761301%23187761301) | 19.7 | 50% | 200 |
| [NP_001020258.1](http://www.ncbi.nlm.nih.gov/entrez/query.fcgi?cmd=Retrieve&db=Protein&list_uids=187761299&dopt=GenPept&RID=T2JDDAMS01S&log$=prottop&blast_rank=17) | CUG-BP- and ETR-3-like factor 4 isoform 2 [Homo sapiens] | [19.7](http://blast.ncbi.nlm.nih.gov/Blast.cgi#187761299%23187761299) | 19.7 | 50% | 200 |
| [NP_001166120.1](http://www.ncbi.nlm.nih.gov/entrez/query.fcgi?cmd=Retrieve&db=Protein&list_uids=289547605&dopt=GenPept&RID=T2JDDAMS01S&log$=prottop&blast_rank=18) | CUG-BP- and ETR-3-like factor 3 isoform 3 [Homo sapiens] | [19.7](http://blast.ncbi.nlm.nih.gov/Blast.cgi#289547605%23289547605) | 19.7 | 50% | 200 |
| [NP_002572.2](http://www.ncbi.nlm.nih.gov/entrez/query.fcgi?cmd=Retrieve&db=Protein&list_uids=38045915&dopt=GenPept&RID=T2JDDAMS01S&log$=prottop&blast_rank=19) | pregnancy-associated plasma protein A preproprotein [Homo sapiens] | [19.7](http://blast.ncbi.nlm.nih.gov/Blast.cgi#38045915%2338045915) | 19.7 | 50% | 200 |
| [NP_852469.1](http://www.ncbi.nlm.nih.gov/entrez/query.fcgi?cmd=Retrieve&db=Protein&list_uids=31652242&dopt=GenPept&RID=T2JDDAMS01S&log$=prottop&blast_rank=20) | transcription factor 20 isoform 2 [Homo sapiens] | [19.7](http://blast.ncbi.nlm.nih.gov/Blast.cgi#31652242%2331652242) | 19.7 | 80% | 200 |
| [NP_005641.1](http://www.ncbi.nlm.nih.gov/entrez/query.fcgi?cmd=Retrieve&db=Protein&list_uids=31652244&dopt=GenPept&RID=T2JDDAMS01S&log$=prottop&blast_rank=21) | transcription factor 20 isoform 1 [Homo sapiens] | [19.7](http://blast.ncbi.nlm.nih.gov/Blast.cgi#31652244%2331652244) | 19.7 | 80% | 200 |
| [NP_945322.1](http://www.ncbi.nlm.nih.gov/entrez/query.fcgi?cmd=Retrieve&db=Protein&list_uids=39725944&dopt=GenPept&RID=T2JDDAMS01S&log$=prottop&blast_rank=22) | MBD2 (methyl-CpG-binding protein)-interacting zinc finger protein [Homo sapiens] >ref|NP_056332.2| MBD2 (methyl-CpG-binding protein)-interacting zinc finger protein [Homo sapiens] | [19.7](http://blast.ncbi.nlm.nih.gov/Blast.cgi#39725944%2339725944) | 19.7 | 50% | 200 |
| [NP_002835.2](http://www.ncbi.nlm.nih.gov/entrez/query.fcgi?cmd=Retrieve&db=Protein&list_uids=18860902&dopt=GenPept&RID=T2JDDAMS01S&log$=prottop&blast_rank=23) | protein tyrosine phosphatase, receptor type, K isoform b [Homo sapiens] | [19.7](http://blast.ncbi.nlm.nih.gov/Blast.cgi#18860902%2318860902) | 29.7 | 90% | 200 |
| [NP_443072.3](http://www.ncbi.nlm.nih.gov/entrez/query.fcgi?cmd=Retrieve&db=Protein&list_uids=70780381&dopt=GenPept&RID=T2JDDAMS01S&log$=prottop&blast_rank=24) | CUG-BP- and ETR-3-like factor 6 isoform 1 [Homo sapiens] | [19.7](http://blast.ncbi.nlm.nih.gov/Blast.cgi#70780381%2370780381) | 19.7 | 50% | 200 |
| [NP_068757.2](http://www.ncbi.nlm.nih.gov/entrez/query.fcgi?cmd=Retrieve&db=Protein&list_uids=24762232&dopt=GenPept&RID=T2JDDAMS01S&log$=prottop&blast_rank=25) | CUG-BP- and ETR-3-like factor 5 isoform 1 [Homo sapiens] | [19.7](http://blast.ncbi.nlm.nih.gov/Blast.cgi#24762232%2324762232) | 19.7 | 50% | 200 |
| [NP_001020260.1](http://www.ncbi.nlm.nih.gov/entrez/query.fcgi?cmd=Retrieve&db=Protein&list_uids=187761303&dopt=GenPept&RID=T2JDDAMS01S&log$=prottop&blast_rank=26) | CUG-BP- and ETR-3-like factor 4 isoform 4 [Homo sapiens] | [19.7](http://blast.ncbi.nlm.nih.gov/Blast.cgi#187761303%23187761303) | 19.7 | 50% | 200 |
| [NP_002363.2](http://www.ncbi.nlm.nih.gov/entrez/query.fcgi?cmd=Retrieve&db=Protein&list_uids=51477714&dopt=GenPept&RID=T2JDDAMS01S&log$=prottop&blast_rank=27) | mannosidase, alpha, class 2A, member 1 [Homo sapiens] | [19.7](http://blast.ncbi.nlm.nih.gov/Blast.cgi#51477714%2351477714) | 19.7 | 50% | 200 |
| [NP_064565.1](http://www.ncbi.nlm.nih.gov/entrez/query.fcgi?cmd=Retrieve&db=Protein&list_uids=13123778&dopt=GenPept&RID=T2JDDAMS01S&log$=prottop&blast_rank=28) | CUG-BP- and ETR-3-like factor 4 isoform 1 [Homo sapiens] | [19.7](http://blast.ncbi.nlm.nih.gov/Blast.cgi#13123778%2313123778) | 19.7 | 50% | 200 |
| [NP_065744.2](http://www.ncbi.nlm.nih.gov/entrez/query.fcgi?cmd=Retrieve&db=Protein&list_uids=21359935&dopt=GenPept&RID=T2JDDAMS01S&log$=prottop&blast_rank=29) | Down syndrome cell adhesion molecule like 1 [Homo sapiens] | [19.7](http://blast.ncbi.nlm.nih.gov/Blast.cgi#21359935%2321359935) | 19.7 | 70% | 200 |
| [NP_009116.3](http://www.ncbi.nlm.nih.gov/entrez/query.fcgi?cmd=Retrieve&db=Protein&list_uids=71164894&dopt=GenPept&RID=T2JDDAMS01S&log$=prottop&blast_rank=30) | CUG-BP- and ETR-3-like factor 3 isoform 1 [Homo sapiens] | [19.7](http://blast.ncbi.nlm.nih.gov/Blast.cgi#71164894%2371164894) | 19.7 | 50% | 200 |
| [NP_055833.2](http://www.ncbi.nlm.nih.gov/entrez/query.fcgi?cmd=Retrieve&db=Protein&list_uids=34577118&dopt=GenPept&RID=T2JDDAMS01S&log$=prottop&blast_rank=31) | dopey family member 1 [Homo sapiens] | [19.3](http://blast.ncbi.nlm.nih.gov/Blast.cgi#34577118%2334577118) | 19.3 | 50% | 268 |
| [NP_973729.1](http://www.ncbi.nlm.nih.gov/entrez/query.fcgi?cmd=Retrieve&db=Protein&list_uids=42544134&dopt=GenPept&RID=T2JDDAMS01S&log$=prottop&blast_rank=32) | SA hypertension-associated homolog isoform 2 [Homo sapiens] | [19.3](http://blast.ncbi.nlm.nih.gov/Blast.cgi#42544134%2342544134) | 19.3 | 70% | 268 |
| [NP_057374.3](http://www.ncbi.nlm.nih.gov/entrez/query.fcgi?cmd=Retrieve&db=Protein&list_uids=42476130&dopt=GenPept&RID=T2JDDAMS01S&log$=prottop&blast_rank=33) | ubiquitin interaction motif containing 1 [Homo sapiens] | [19.3](http://blast.ncbi.nlm.nih.gov/Blast.cgi#42476130%2342476130) | 19.3 | 60% | 268 |
| [NP_004935.1](http://www.ncbi.nlm.nih.gov/entrez/query.fcgi?cmd=Retrieve&db=Protein&list_uids=4826698&dopt=GenPept&RID=T2JDDAMS01S&log$=prottop&blast_rank=34) | deoxyribonuclease I-like 3 precursor [Homo sapiens] | [19.3](http://blast.ncbi.nlm.nih.gov/Blast.cgi#4826698%234826698) | 19.3 | 50% | 268 |
| [NP_055135.1](http://www.ncbi.nlm.nih.gov/entrez/query.fcgi?cmd=Retrieve&db=Protein&list_uids=7657603&dopt=GenPept&RID=T2JDDAMS01S&log$=prottop&blast_rank=35) | heme binding protein 2 [Homo sapiens] | [19.3](http://blast.ncbi.nlm.nih.gov/Blast.cgi#7657603%237657603) | 19.3 | 50% | 268 |
| [NP_060686.1](http://www.ncbi.nlm.nih.gov/entrez/query.fcgi?cmd=Retrieve&db=Protein&list_uids=8922665&dopt=GenPept&RID=T2JDDAMS01S&log$=prottop&blast_rank=36) | pantothenate kinase 4 [Homo sapiens] | [19.3](http://blast.ncbi.nlm.nih.gov/Blast.cgi#8922665%238922665) | 19.3 | 50% | 268 |
| [NP_004973.1](http://www.ncbi.nlm.nih.gov/entrez/query.fcgi?cmd=Retrieve&db=Protein&list_uids=4826802&dopt=GenPept&RID=T2JDDAMS01S&log$=prottop&blast_rank=37) | potassium inwardly-rectifying channel J8 [Homo sapiens] | [19.3](http://blast.ncbi.nlm.nih.gov/Blast.cgi#4826802%234826802) | 36.1 | 90% | 268 |
| [NP_006819.2](http://www.ncbi.nlm.nih.gov/entrez/query.fcgi?cmd=Retrieve&db=Protein&list_uids=76880486&dopt=GenPept&RID=T2JDDAMS01S&log$=prottop&blast_rank=38) | activating signal cointegrator 1 complex subunit 3 isoform a [Homo sapiens] | [18.9](http://blast.ncbi.nlm.nih.gov/Blast.cgi#76880486%2376880486) | 43.5 | 90% | 359 |
| [NP_116201.7](http://www.ncbi.nlm.nih.gov/entrez/query.fcgi?cmd=Retrieve&db=Protein&list_uids=40255005&dopt=GenPept&RID=T2JDDAMS01S&log$=prottop&blast_rank=39) | plexin domain containing 2 precursor [Homo sapiens] | [18.9](http://blast.ncbi.nlm.nih.gov/Blast.cgi#40255005%2340255005) | 18.9 | 80% | 359 |
| [NP_001018066.1](http://www.ncbi.nlm.nih.gov/entrez/query.fcgi?cmd=Retrieve&db=Protein&list_uids=65301164&dopt=GenPept&RID=T2JDDAMS01S&log$=prottop&blast_rank=40) | very low density lipoprotein receptor isoform b [Homo sapiens] | [18.9](http://blast.ncbi.nlm.nih.gov/Blast.cgi#65301164%2365301164) | 18.9 | 90% | 359 |
| [NP_001259.1](http://www.ncbi.nlm.nih.gov/entrez/query.fcgi?cmd=Retrieve&db=Protein&list_uids=4557445&dopt=GenPept&RID=T2JDDAMS01S&log$=prottop&blast_rank=41) | regulator of chromosome condensation and BTB domain containing protein 2 [Homo sapiens] | [18.9](http://blast.ncbi.nlm.nih.gov/Blast.cgi#4557445%234557445) | 18.9 | 60% | 359 |
| [NP_003374.3](http://www.ncbi.nlm.nih.gov/entrez/query.fcgi?cmd=Retrieve&db=Protein&list_uids=65301167&dopt=GenPept&RID=T2JDDAMS01S&log$=prottop&blast_rank=42) | very low density lipoprotein receptor isoform a [Homo sapiens] | [18.9](http://blast.ncbi.nlm.nih.gov/Blast.cgi#65301167%2365301167) | 18.9 | 90% | 359 |
| [NP_005391.1](http://www.ncbi.nlm.nih.gov/entrez/query.fcgi?cmd=Retrieve&db=Protein&list_uids=4885563&dopt=GenPept&RID=T2JDDAMS01S&log$=prottop&blast_rank=43) | protein kinase C, epsilon [Homo sapiens] | [18.9](http://blast.ncbi.nlm.nih.gov/Blast.cgi#4885563%234885563) | 18.9 | 70% | 359 |
| [NP_004681.1](http://www.ncbi.nlm.nih.gov/entrez/query.fcgi?cmd=Retrieve&db=Protein&list_uids=4758666&dopt=GenPept&RID=T2JDDAMS01S&log$=prottop&blast_rank=44) | LATS homolog 1 [Homo sapiens] | [18.9](http://blast.ncbi.nlm.nih.gov/Blast.cgi#4758666%234758666) | 18.9 | 70% | 359 |
| [XP_002344832.1](http://www.ncbi.nlm.nih.gov/entrez/query.fcgi?cmd=Retrieve&db=Protein&list_uids=239756174&dopt=GenPept&RID=T2JDDAMS01S&log$=prottop&blast_rank=45) | PREDICTED: hypothetical protein [Homo sapiens] | [18.5](http://blast.ncbi.nlm.nih.gov/Blast.cgi#239756174%23239756174) | 18.5 | 60% | 482 |
| [XP_001718394.2](http://www.ncbi.nlm.nih.gov/entrez/query.fcgi?cmd=Retrieve&db=Protein&list_uids=239744982&dopt=GenPept&RID=T2JDDAMS01S&log$=prottop&blast_rank=46) | PREDICTED: hypothetical protein [Homo sapiens] >ref|XP_001716267.2| PREDICTED: hypothetical protein [Homo sapiens] | [18.5](http://blast.ncbi.nlm.nih.gov/Blast.cgi#239744982%23239744982) | 18.5 | 60% | 482 |
| [NP_757344.2](http://www.ncbi.nlm.nih.gov/entrez/query.fcgi?cmd=Retrieve&db=Protein&list_uids=148806859&dopt=GenPept&RID=T2JDDAMS01S&log$=prottop&blast_rank=47) | calcium/calmodulin-dependent protein kinase 1 alpha isoform b [Homo sapiens] | [18.5](http://blast.ncbi.nlm.nih.gov/Blast.cgi#148806859%23148806859) | 18.5 | 60% | 482 |
| [NP_065941.2](http://www.ncbi.nlm.nih.gov/entrez/query.fcgi?cmd=Retrieve&db=Protein&list_uids=190194355&dopt=GenPept&RID=T2JDDAMS01S&log$=prottop&blast_rank=48) | p90 autoantigen [Homo sapiens] | [18.5](http://blast.ncbi.nlm.nih.gov/Blast.cgi#190194355%23190194355) | 18.5 | 60% | 482 |
| [NP_005206.2](http://www.ncbi.nlm.nih.gov/entrez/query.fcgi?cmd=Retrieve&db=Protein&list_uids=110431348&dopt=GenPept&RID=T2JDDAMS01S&log$=prottop&blast_rank=49) | netrin receptor DCC precursor [Homo sapiens] | [18.5](http://blast.ncbi.nlm.nih.gov/Blast.cgi#110431348%23110431348) | 18.5 | 60% | 482 |
| [NP_757365.1](http://www.ncbi.nlm.nih.gov/entrez/query.fcgi?cmd=Retrieve&db=Protein&list_uids=27437025&dopt=GenPept&RID=T2JDDAMS01S&log$=prottop&blast_rank=50) | calcium/calmodulin-dependent protein kinase kinase 2 beta isoform 3 [Homo sapiens] | [18.5](http://blast.ncbi.nlm.nih.gov/Blast.cgi#27437025%2327437025) | 18.5 | 60% | 482 |
| [NP_006540.3](http://www.ncbi.nlm.nih.gov/entrez/query.fcgi?cmd=Retrieve&db=Protein&list_uids=27437015&dopt=GenPept&RID=T2JDDAMS01S&log$=prottop&blast_rank=51) | calcium/calmodulin-dependent protein kinase kinase 2 beta isoform 1 [Homo sapiens] | [18.5](http://blast.ncbi.nlm.nih.gov/Blast.cgi#27437015%2327437015) | 18.5 | 60% | 482 |
| [NP_757363.1](http://www.ncbi.nlm.nih.gov/entrez/query.fcgi?cmd=Retrieve&db=Protein&list_uids=27437021&dopt=GenPept&RID=T2JDDAMS01S&log$=prottop&blast_rank=52) | calcium/calmodulin-dependent protein kinase kinase 2 beta isoform 5 [Homo sapiens] | [18.5](http://blast.ncbi.nlm.nih.gov/Blast.cgi#27437021%2327437021) | 18.5 | 60% | 482 |
| [NP_001136145.1](http://www.ncbi.nlm.nih.gov/entrez/query.fcgi?cmd=Retrieve&db=Protein&list_uids=218082953&dopt=GenPept&RID=T2JDDAMS01S&log$=prottop&blast_rank=53) | autophagy-related protein 13 isoform 1 [Homo sapiens] | [18.5](http://blast.ncbi.nlm.nih.gov/Blast.cgi#218082953%23218082953) | 18.5 | 60% | 482 |
| [NP_757364.1](http://www.ncbi.nlm.nih.gov/entrez/query.fcgi?cmd=Retrieve&db=Protein&list_uids=27437023&dopt=GenPept&RID=T2JDDAMS01S&log$=prottop&blast_rank=54) | calcium/calmodulin-dependent protein kinase kinase 2 beta isoform 6 [Homo sapiens] | [18.5](http://blast.ncbi.nlm.nih.gov/Blast.cgi#27437023%2327437023) | 18.5 | 60% | 482 |
| [NP_003263.1](http://www.ncbi.nlm.nih.gov/entrez/query.fcgi?cmd=Retrieve&db=Protein&list_uids=4507545&dopt=GenPept&RID=T2JDDAMS01S&log$=prottop&blast_rank=55) | G protein-coupled receptor 137B [Homo sapiens] | [18.5](http://blast.ncbi.nlm.nih.gov/Blast.cgi#4507545%234507545) | 18.5 | 60% | 482 |
| [NP_055556.2](http://www.ncbi.nlm.nih.gov/entrez/query.fcgi?cmd=Retrieve&db=Protein&list_uids=41281462&dopt=GenPept&RID=T2JDDAMS01S&log$=prottop&blast_rank=56) | autophagy-related protein 13 isoform 2 [Homo sapiens] | [18.5](http://blast.ncbi.nlm.nih.gov/Blast.cgi#41281462%2341281462) | 18.5 | 60% | 482 |
| [NP_115670.1](http://www.ncbi.nlm.nih.gov/entrez/query.fcgi?cmd=Retrieve&db=Protein&list_uids=14150045&dopt=GenPept&RID=T2JDDAMS01S&log$=prottop&blast_rank=57) | calcium/calmodulin-dependent protein kinase 1 alpha isoform a [Homo sapiens] >ref|NP_757343.1| calcium/calmodulin-dependent protein kinase 1 alpha isoform a [Homo sapiens] | [18.5](http://blast.ncbi.nlm.nih.gov/Blast.cgi#14150045%2314150045) | 18.5 | 60% | 482 |
| [NP_705719.2](http://www.ncbi.nlm.nih.gov/entrez/query.fcgi?cmd=Retrieve&db=Protein&list_uids=27437017&dopt=GenPept&RID=T2JDDAMS01S&log$=prottop&blast_rank=58) | calcium/calmodulin-dependent protein kinase kinase 2 beta isoform 2 [Homo sapiens] >ref|NP_757380.1| calcium/calmodulin-dependent protein kinase kinase 2 beta isoform 2 [Homo sapiens] | [18.5](http://blast.ncbi.nlm.nih.gov/Blast.cgi#27437017%2327437017) | 18.5 | 60% | 482 |
| [NP_705720.1](http://www.ncbi.nlm.nih.gov/entrez/query.fcgi?cmd=Retrieve&db=Protein&list_uids=27437019&dopt=GenPept&RID=T2JDDAMS01S&log$=prottop&blast_rank=59) | calcium/calmodulin-dependent protein kinase kinase 2 beta isoform 4 [Homo sapiens] | [18.5](http://blast.ncbi.nlm.nih.gov/Blast.cgi#27437019%2327437019) | 18.5 | 60% | 482 |
| [XP_001716559.1](http://www.ncbi.nlm.nih.gov/entrez/query.fcgi?cmd=Retrieve&db=Protein&list_uids=169179422&dopt=GenPept&RID=T2JDDAMS01S&log$=prottop&blast_rank=60) | PREDICTED: hypothetical protein [Homo sapiens] | [18.0](http://blast.ncbi.nlm.nih.gov/Blast.cgi#169179422%23169179422) | 18.0 | 60% | 647 |
| [NP_008832.2](http://www.ncbi.nlm.nih.gov/entrez/query.fcgi?cmd=Retrieve&db=Protein&list_uids=156119615&dopt=GenPept&RID=T2JDDAMS01S&log$=prottop&blast_rank=61) | myosin IXA [Homo sapiens] | [18.0](http://blast.ncbi.nlm.nih.gov/Blast.cgi#156119615%23156119615) | 18.0 | 60% | 647 |
| [NP_001008781.2](http://www.ncbi.nlm.nih.gov/entrez/query.fcgi?cmd=Retrieve&db=Protein&list_uids=148886692&dopt=GenPept&RID=T2JDDAMS01S&log$=prottop&blast_rank=62) | FAT tumor suppressor homolog 3 [Homo sapiens] | [18.0](http://blast.ncbi.nlm.nih.gov/Blast.cgi#148886692%23148886692) | 18.0 | 80% | 647 |
| [NP_056110.2](http://www.ncbi.nlm.nih.gov/entrez/query.fcgi?cmd=Retrieve&db=Protein&list_uids=148839305&dopt=GenPept&RID=T2JDDAMS01S&log$=prottop&blast_rank=63) | structural maintenance of chromosomes flexible hinge domain containing 1 [Homo sapiens] | [18.0](http://blast.ncbi.nlm.nih.gov/Blast.cgi#148839305%23148839305) | 28.0 | 70% | 647 |
| [NP_689957.3](http://www.ncbi.nlm.nih.gov/entrez/query.fcgi?cmd=Retrieve&db=Protein&list_uids=119220552&dopt=GenPept&RID=T2JDDAMS01S&log$=prottop&blast_rank=64) | sidekick 1 precursor [Homo sapiens] | [18.0](http://blast.ncbi.nlm.nih.gov/Blast.cgi#119220552%23119220552) | 18.0 | 80% | 647 |
| [NP_004516.2](http://www.ncbi.nlm.nih.gov/entrez/query.fcgi?cmd=Retrieve&db=Protein&list_uids=126012573&dopt=GenPept&RID=T2JDDAMS01S&log$=prottop&blast_rank=65) | low density lipoprotein-related protein 2 precursor [Homo sapiens] | [18.0](http://blast.ncbi.nlm.nih.gov/Blast.cgi#126012573%23126012573) | 49.8 | 50% | 647 |
| [NP_056386.2](http://www.ncbi.nlm.nih.gov/entrez/query.fcgi?cmd=Retrieve&db=Protein&list_uids=156105701&dopt=GenPept&RID=T2JDDAMS01S&log$=prottop&blast_rank=66) | SUMO1/sentrin specific peptidase 6 isoform 1 [Homo sapiens] | [18.0](http://blast.ncbi.nlm.nih.gov/Blast.cgi#156105701%23156105701) | 18.0 | 50% | 647 |
| [NP_060222.2](http://www.ncbi.nlm.nih.gov/entrez/query.fcgi?cmd=Retrieve&db=Protein&list_uids=38570101&dopt=GenPept&RID=T2JDDAMS01S&log$=prottop&blast_rank=67) | TBC1 domain family, member 8B (with GRAM domain) isoform a [Homo sapiens] | [18.0](http://blast.ncbi.nlm.nih.gov/Blast.cgi#38570101%2338570101) | 18.0 | 80% | 647 |
| [NP_037376.2](http://www.ncbi.nlm.nih.gov/entrez/query.fcgi?cmd=Retrieve&db=Protein&list_uids=166197698&dopt=GenPept&RID=T2JDDAMS01S&log$=prottop&blast_rank=68) | alpha-1,3-mannosyl-glycoprotein 4-beta-N-acetylglucosaminyltransferase C [Homo sapiens] | [18.0](http://blast.ncbi.nlm.nih.gov/Blast.cgi#166197698%23166197698) | 18.0 | 50% | 647 |
| [NP_055949.2](http://www.ncbi.nlm.nih.gov/entrez/query.fcgi?cmd=Retrieve&db=Protein&list_uids=50980307&dopt=GenPept&RID=T2JDDAMS01S&log$=prottop&blast_rank=69) | myosin phosphatase-Rho interacting protein isoform 1 [Homo sapiens] | [18.0](http://blast.ncbi.nlm.nih.gov/Blast.cgi#50980307%2350980307) | 18.0 | 50% | 647 |
| [NP_958431.2](http://www.ncbi.nlm.nih.gov/entrez/query.fcgi?cmd=Retrieve&db=Protein&list_uids=50980301&dopt=GenPept&RID=T2JDDAMS01S&log$=prottop&blast_rank=70) | myosin phosphatase-Rho interacting protein isoform 2 [Homo sapiens] | [18.0](http://blast.ncbi.nlm.nih.gov/Blast.cgi#50980301%2350980301) | 18.0 | 50% | 647 |
| [NP_919410.1](http://www.ncbi.nlm.nih.gov/entrez/query.fcgi?cmd=Retrieve&db=Protein&list_uids=36287110&dopt=GenPept&RID=T2JDDAMS01S&log$=prottop&blast_rank=71) | FGFR1 oncogene partner isoform b [Homo sapiens] | [18.0](http://blast.ncbi.nlm.nih.gov/Blast.cgi#36287110%2336287110) | 18.0 | 50% | 647 |
| [NP_065866.1](http://www.ncbi.nlm.nih.gov/entrez/query.fcgi?cmd=Retrieve&db=Protein&list_uids=14589914&dopt=GenPept&RID=T2JDDAMS01S&log$=prottop&blast_rank=72) | protocadherin 10 isoform 2 precursor [Homo sapiens] | [18.0](http://blast.ncbi.nlm.nih.gov/Blast.cgi#14589914%2314589914) | 34.4 | 90% | 647 |
| [NP_612195.1](http://www.ncbi.nlm.nih.gov/entrez/query.fcgi?cmd=Retrieve&db=Protein&list_uids=20336186&dopt=GenPept&RID=T2JDDAMS01S&log$=prottop&blast_rank=73) | paired basic amino acid cleaving system 4 isoform c preproprotein [Homo sapiens] | [18.0](http://blast.ncbi.nlm.nih.gov/Blast.cgi#20336186%2320336186) | 18.0 | 80% | 647 |
| [NP_062570.1](http://www.ncbi.nlm.nih.gov/entrez/query.fcgi?cmd=Retrieve&db=Protein&list_uids=9955968&dopt=GenPept&RID=T2JDDAMS01S&log$=prottop&blast_rank=74) | ATP-binding cassette, sub-family B (MDR/TAP), member 9 isoform 2 [Homo sapiens] | [18.0](http://blast.ncbi.nlm.nih.gov/Blast.cgi#9955968%239955968) | 18.0 | 60% | 647 |
| [NP_001017373.2](http://www.ncbi.nlm.nih.gov/entrez/query.fcgi?cmd=Retrieve&db=Protein&list_uids=126032305&dopt=GenPept&RID=T2JDDAMS01S&log$=prottop&blast_rank=75) | sterile alpha motif domain containing 3 isoform a [Homo sapiens] | [18.0](http://blast.ncbi.nlm.nih.gov/Blast.cgi#126032305%23126032305) | 18.0 | 60% | 647 |
| [NP_148937.1](http://www.ncbi.nlm.nih.gov/entrez/query.fcgi?cmd=Retrieve&db=Protein&list_uids=15451786&dopt=GenPept&RID=T2JDDAMS01S&log$=prottop&blast_rank=76) | platelet-derived growth factor beta isoform 2 preproprotein [Homo sapiens] | [18.0](http://blast.ncbi.nlm.nih.gov/Blast.cgi#15451786%2315451786) | 18.0 | 60% | 647 |
| [NP_150643.2](http://www.ncbi.nlm.nih.gov/entrez/query.fcgi?cmd=Retrieve&db=Protein&list_uids=66082554&dopt=GenPept&RID=T2JDDAMS01S&log$=prottop&blast_rank=77) | low density lipoprotein receptor-related protein 8 isoform 2 precursor [Homo sapiens] | [18.0](http://blast.ncbi.nlm.nih.gov/Blast.cgi#66082554%2366082554) | 18.0 | 50% | 647 |
| [NP_891550.1](http://www.ncbi.nlm.nih.gov/entrez/query.fcgi?cmd=Retrieve&db=Protein&list_uids=33624896&dopt=GenPept&RID=T2JDDAMS01S&log$=prottop&blast_rank=78) | ADAM metallopeptidase with thrombospondin type 1 motif, 9 preproprotein [Homo sapiens] | [18.0](http://blast.ncbi.nlm.nih.gov/Blast.cgi#33624896%2333624896) | 18.0 | 90% | 647 |
| [NP_612193.1](http://www.ncbi.nlm.nih.gov/entrez/query.fcgi?cmd=Retrieve&db=Protein&list_uids=20336182&dopt=GenPept&RID=T2JDDAMS01S&log$=prottop&blast_rank=79) | paired basic amino acid cleaving system 4 isoform g preproprotein [Homo sapiens] | [18.0](http://blast.ncbi.nlm.nih.gov/Blast.cgi#20336182%2320336182) | 18.0 | 80% | 647 |
| [NP_612198.2](http://www.ncbi.nlm.nih.gov/entrez/query.fcgi?cmd=Retrieve&db=Protein&list_uids=27894285&dopt=GenPept&RID=T2JDDAMS01S&log$=prottop&blast_rank=80) | paired basic amino acid cleaving system 4 isoform f preproprotein [Homo sapiens] | [18.0](http://blast.ncbi.nlm.nih.gov/Blast.cgi#27894285%2327894285) | 18.0 | 80% | 647 |
| [NP_612194.1](http://www.ncbi.nlm.nih.gov/entrez/query.fcgi?cmd=Retrieve&db=Protein&list_uids=20336184&dopt=GenPept&RID=T2JDDAMS01S&log$=prottop&blast_rank=81) | paired basic amino acid cleaving system 4 isoform h preproprotein [Homo sapiens] | [18.0](http://blast.ncbi.nlm.nih.gov/Blast.cgi#20336184%2320336184) | 18.0 | 80% | 647 |
| [NP_079328.3](http://www.ncbi.nlm.nih.gov/entrez/query.fcgi?cmd=Retrieve&db=Protein&list_uids=68077164&dopt=GenPept&RID=T2JDDAMS01S&log$=prottop&blast_rank=82) | Yeast Sps1/Ste20-related kinase 4 isoform 1 [Homo sapiens] | [18.0](http://blast.ncbi.nlm.nih.gov/Blast.cgi#68077164%2368077164) | 18.0 | 50% | 647 |
| [NP_116586.1](http://www.ncbi.nlm.nih.gov/entrez/query.fcgi?cmd=Retrieve&db=Protein&list_uids=14589916&dopt=GenPept&RID=T2JDDAMS01S&log$=prottop&blast_rank=83) | protocadherin 10 isoform 1 precursor [Homo sapiens] | [18.0](http://blast.ncbi.nlm.nih.gov/Blast.cgi#14589916%2314589916) | 34.4 | 90% | 647 |
| [NP_001093879.1](http://www.ncbi.nlm.nih.gov/entrez/query.fcgi?cmd=Retrieve&db=Protein&list_uids=156105703&dopt=GenPept&RID=T2JDDAMS01S&log$=prottop&blast_rank=84) | SUMO1/sentrin specific peptidase 6 isoform 2 [Homo sapiens] | [18.0](http://blast.ncbi.nlm.nih.gov/Blast.cgi#156105703%23156105703) | 18.0 | 50% | 647 |
| [NP_059992.3](http://www.ncbi.nlm.nih.gov/entrez/query.fcgi?cmd=Retrieve&db=Protein&list_uids=61744467&dopt=GenPept&RID=T2JDDAMS01S&log$=prottop&blast_rank=85) | low density lipoprotein receptor-related protein 8 isoform 3 precursor [Homo sapiens] | [18.0](http://blast.ncbi.nlm.nih.gov/Blast.cgi#61744467%2361744467) | 18.0 | 50% | 647 |
| [NP_004622.2](http://www.ncbi.nlm.nih.gov/entrez/query.fcgi?cmd=Retrieve&db=Protein&list_uids=61744471&dopt=GenPept&RID=T2JDDAMS01S&log$=prottop&blast_rank=86) | low density lipoprotein receptor-related protein 8 isoform 1 precursor [Homo sapiens] | [18.0](http://blast.ncbi.nlm.nih.gov/Blast.cgi#61744471%2361744471) | 18.0 | 50% | 647 |
| [NP_001018064.1](http://www.ncbi.nlm.nih.gov/entrez/query.fcgi?cmd=Retrieve&db=Protein&list_uids=65301119&dopt=GenPept&RID=T2JDDAMS01S&log$=prottop&blast_rank=87) | low density lipoprotein receptor-related protein 8 isoform 4 precursor [Homo sapiens] | [18.0](http://blast.ncbi.nlm.nih.gov/Blast.cgi#65301119%2365301119) | 18.0 | 50% | 647 |
| [NP_004651.2](http://www.ncbi.nlm.nih.gov/entrez/query.fcgi?cmd=Retrieve&db=Protein&list_uids=13514809&dopt=GenPept&RID=T2JDDAMS01S&log$=prottop&blast_rank=88) | DEAD (Asp-Glu-Ala-Asp) box polypeptide 3, Y-linked [Homo sapiens] >ref|NP_001116137.1| DEAD (Asp-Glu-Ala-Asp) box polypeptide 3, Y-linked [Homo sapiens] | [18.0](http://blast.ncbi.nlm.nih.gov/Blast.cgi#13514809%2313514809) | 28.4 | 100% | 647 |
| [NP_000518.1](http://www.ncbi.nlm.nih.gov/entrez/query.fcgi?cmd=Retrieve&db=Protein&list_uids=4504975&dopt=GenPept&RID=T2JDDAMS01S&log$=prottop&blast_rank=89) | low density lipoprotein receptor precursor [Homo sapiens] | [18.0](http://blast.ncbi.nlm.nih.gov/Blast.cgi#4504975%234504975) | 18.0 | 50% | 647 |
| [NP_008976.1](http://www.ncbi.nlm.nih.gov/entrez/query.fcgi?cmd=Retrieve&db=Protein&list_uids=5901954&dopt=GenPept&RID=T2JDDAMS01S&log$=prottop&blast_rank=90) | FGFR1 oncogene partner isoform a [Homo sapiens] | [18.0](http://blast.ncbi.nlm.nih.gov/Blast.cgi#5901954%235901954) | 18.0 | 50% | 647 |
| [NP_112224.1](http://www.ncbi.nlm.nih.gov/entrez/query.fcgi?cmd=Retrieve&db=Protein&list_uids=29788755&dopt=GenPept&RID=T2JDDAMS01S&log$=prottop&blast_rank=91) | SET binding factor 2 [Homo sapiens] | [18.0](http://blast.ncbi.nlm.nih.gov/Blast.cgi#29788755%2329788755) | 31.8 | 80% | 647 |
| [NP_078832.1](http://www.ncbi.nlm.nih.gov/entrez/query.fcgi?cmd=Retrieve&db=Protein&list_uids=13375721&dopt=GenPept&RID=T2JDDAMS01S&log$=prottop&blast_rank=92) | hypothetical protein LOC79607 [Homo sapiens] | [18.0](http://blast.ncbi.nlm.nih.gov/Blast.cgi#13375721%2313375721) | 18.0 | 50% | 647 |
| [NP_001460.1](http://www.ncbi.nlm.nih.gov/entrez/query.fcgi?cmd=Retrieve&db=Protein&list_uids=4503841&dopt=GenPept&RID=T2JDDAMS01S&log$=prottop&blast_rank=93) | ATP-dependent DNA helicase II, 70 kDa subunit [Homo sapiens] | [18.0](http://blast.ncbi.nlm.nih.gov/Blast.cgi#4503841%234503841) | 18.0 | 60% | 647 |
| [NP_062571.1](http://www.ncbi.nlm.nih.gov/entrez/query.fcgi?cmd=Retrieve&db=Protein&list_uids=9955966&dopt=GenPept&RID=T2JDDAMS01S&log$=prottop&blast_rank=94) | ATP-binding cassette, sub-family B (MDR/TAP), member 9 isoform 1 [Homo sapiens] >ref|NP_982269.1| ATP-binding cassette, sub-family B (MDR/TAP), member 9 isoform 1 [Homo sapiens] | [18.0](http://blast.ncbi.nlm.nih.gov/Blast.cgi#9955966%239955966) | 18.0 | 60% | 647 |
| [NP_002599.1](http://www.ncbi.nlm.nih.gov/entrez/query.fcgi?cmd=Retrieve&db=Protein&list_uids=4505681&dopt=GenPept&RID=T2JDDAMS01S&log$=prottop&blast_rank=95) | platelet-derived growth factor beta isoform 1 preproprotein [Homo sapiens] | [18.0](http://blast.ncbi.nlm.nih.gov/Blast.cgi#4505681%234505681) | 18.0 | 60% | 647 |
| [NP_001075.1](http://www.ncbi.nlm.nih.gov/entrez/query.fcgi?cmd=Retrieve&db=Protein&list_uids=4505891&dopt=GenPept&RID=T2JDDAMS01S&log$=prottop&blast_rank=96) | procollagen-lysine, 2-oxoglutarate 5-dioxygenase 3 precursor [Homo sapiens] | [18.0](http://blast.ncbi.nlm.nih.gov/Blast.cgi#4505891%234505891) | 18.0 | 50% | 647 |
| [NP_612192.1](http://www.ncbi.nlm.nih.gov/entrez/query.fcgi?cmd=Retrieve&db=Protein&list_uids=20336180&dopt=GenPept&RID=T2JDDAMS01S&log$=prottop&blast_rank=97) | paired basic amino acid cleaving system 4 isoform b preproprotein [Homo sapiens] | [18.0](http://blast.ncbi.nlm.nih.gov/Blast.cgi#20336180%2320336180) | 18.0 | 80% | 647 |
| [NP_751948.1](http://www.ncbi.nlm.nih.gov/entrez/query.fcgi?cmd=Retrieve&db=Protein&list_uids=27436986&dopt=GenPept&RID=T2JDDAMS01S&log$=prottop&blast_rank=98) | potassium voltage-gated channel, Shal-related subfamily, member 3 isoform 2 [Homo sapiens] | [18.0](http://blast.ncbi.nlm.nih.gov/Blast.cgi#27436986%2327436986) | 18.0 | 60% | 647 |
| [NP_055129.2](http://www.ncbi.nlm.nih.gov/entrez/query.fcgi?cmd=Retrieve&db=Protein&list_uids=27881482&dopt=GenPept&RID=T2JDDAMS01S&log$=prottop&blast_rank=99) | DEAD/H (Asp-Glu-Ala-Asp/His) box polypeptide RIG-I [Homo sapiens] | [18.0](http://blast.ncbi.nlm.nih.gov/Blast.cgi#27881482%2327881482) | 18.0 | 70% | 647 |
| [NP_612197.1](http://www.ncbi.nlm.nih.gov/entrez/query.fcgi?cmd=Retrieve&db=Protein&list_uids=20336190&dopt=GenPept&RID=T2JDDAMS01S&log$=prottop&blast_rank=100) | paired basic amino acid cleaving system 4 isoform d preproprotein [Homo sapiens] | [18.0](http://blast.ncbi.nlm.nih.gov/Blast.cgi#20336190%2320336190) | 18.0 | 80% | 647 |

| **Accession** | **Proteins with a match to ANTPWSKTL peptide** | **[Max score](http://blast.ncbi.nlm.nih.gov/Blast.cgi?CMD=Get&ALIGNMENTS=100&ALIGNMENT_VIEW=Pairwise&CDD_SEARCH_STATE=1&DATABASE_SORT=0&DESCRIPTIONS=100&ENTREZ_QUERY=txid9606 %5BORGN%5D&FIRST_QUERY_NUM=0&FORMAT_OBJECT=Alignment&FORMAT_PAGE_TARGET=&FORMAT_TYPE=HTML&GET_SEQUENCE=yes&I_THRESH=&MASK_CHAR=2&MASK_COLOR=1&NEW_DESIGN=on&NEW_VIEW=yes&NUM_OVERVIEW=100&OLD_BLAST=false&PAGE=Proteins&QUERY_INDEX=0&QUERY_NUMBER=0&RESULTS_PAGE_TARGET=&RID=T2NUM92A014&SHOW_LINKOUT=yes&SHOW_OVERVIEW=yes&STEP_NUMBER=&WORD_SIZE=2&DISPLAY_SORT=1&HSP_SORT=1" \l "sort_mark)** | **[Total score](http://blast.ncbi.nlm.nih.gov/Blast.cgi?CMD=Get&ALIGNMENTS=100&ALIGNMENT_VIEW=Pairwise&CDD_SEARCH_STATE=1&DATABASE_SORT=0&DESCRIPTIONS=100&ENTREZ_QUERY=txid9606 %5BORGN%5D&FIRST_QUERY_NUM=0&FORMAT_OBJECT=Alignment&FORMAT_PAGE_TARGET=&FORMAT_TYPE=HTML&GET_SEQUENCE=yes&I_THRESH=&MASK_CHAR=2&MASK_COLOR=1&NEW_DESIGN=on&NEW_VIEW=yes&NUM_OVERVIEW=100&OLD_BLAST=false&PAGE=Proteins&QUERY_INDEX=0&QUERY_NUMBER=0&RESULTS_PAGE_TARGET=&RID=T2NUM92A014&SHOW_LINKOUT=yes&SHOW_OVERVIEW=yes&STEP_NUMBER=&WORD_SIZE=2&DISPLAY_SORT=2&HSP_SORT=1" \l "sort_mark)** | **[Query coverage](http://blast.ncbi.nlm.nih.gov/Blast.cgi?CMD=Get&ALIGNMENTS=100&ALIGNMENT_VIEW=Pairwise&CDD_SEARCH_STATE=1&DATABASE_SORT=0&DESCRIPTIONS=100&ENTREZ_QUERY=txid9606 %5BORGN%5D&FIRST_QUERY_NUM=0&FORMAT_OBJECT=Alignment&FORMAT_PAGE_TARGET=&FORMAT_TYPE=HTML&GET_SEQUENCE=yes&I_THRESH=&MASK_CHAR=2&MASK_COLOR=1&NEW_DESIGN=on&NEW_VIEW=yes&NUM_OVERVIEW=100&OLD_BLAST=false&PAGE=Proteins&QUERY_INDEX=0&QUERY_NUMBER=0&RESULTS_PAGE_TARGET=&RID=T2NUM92A014&SHOW_LINKOUT=yes&SHOW_OVERVIEW=yes&STEP_NUMBER=&WORD_SIZE=2&DISPLAY_SORT=4&HSP_SORT=0" \l "sort_mark)** | **[E value](http://blast.ncbi.nlm.nih.gov/Blast.cgi?CMD=Get&ALIGNMENTS=100&ALIGNMENT_VIEW=Pairwise&CDD_SEARCH_STATE=1&DATABASE_SORT=0&DESCRIPTIONS=100&ENTREZ_QUERY=txid9606 %5BORGN%5D&FIRST_QUERY_NUM=0&FORMAT_OBJECT=Alignment&FORMAT_PAGE_TARGET=&FORMAT_TYPE=HTML&GET_SEQUENCE=yes&I_THRESH=&MASK_CHAR=2&MASK_COLOR=1&NEW_DESIGN=on&NEW_VIEW=yes&NUM_OVERVIEW=100&OLD_BLAST=false&PAGE=Proteins&QUERY_INDEX=0&QUERY_NUMBER=0&RESULTS_PAGE_TARGET=&RID=T2NUM92A014&SHOW_LINKOUT=yes&SHOW_OVERVIEW=yes&STEP_NUMBER=&WORD_SIZE=2&DISPLAY_SORT=0&HSP_SORT=0" \l "sort_mark)** |
| --- | --- | --- | --- | --- | --- |
| [NP_078836.1](http://www.ncbi.nlm.nih.gov/entrez/query.fcgi?cmd=Retrieve&db=Protein&list_uids=13375727&dopt=GenPept&RID=T2NUM92A014&log$=prottop&blast_rank=1) | acyl-CoA synthetase short-chain family member 3 precursor [Homo sapiens] | [21.0](http://blast.ncbi.nlm.nih.gov/Blast.cgi#13375727%2313375727) | 21.0 | 66% | 74 |
| [NP_003719.2](http://www.ncbi.nlm.nih.gov/entrez/query.fcgi?cmd=Retrieve&db=Protein&list_uids=16933525&dopt=GenPept&RID=T2NUM92A014&log$=prottop&blast_rank=2) | unc5C precursor [Homo sapiens] | [20.6](http://blast.ncbi.nlm.nih.gov/Blast.cgi#16933525%2316933525) | 20.6 | 55% | 100 |
| [XP_937083.1](http://www.ncbi.nlm.nih.gov/entrez/query.fcgi?cmd=Retrieve&db=Protein&list_uids=88990495&dopt=GenPept&RID=T2NUM92A014&log$=prottop&blast_rank=3) | PREDICTED: hypothetical protein [Homo sapiens] >ref|XP_948685.1| PREDICTED: hypothetical protein [Homo sapiens] >ref|XP_001724847.1| PREDICTED: hypothetical protein [Homo sapiens] | [20.6](http://blast.ncbi.nlm.nih.gov/Blast.cgi#88990495%2388990495) | 20.6 | 66% | 100 |
| [NP_065784.1](http://www.ncbi.nlm.nih.gov/entrez/query.fcgi?cmd=Retrieve&db=Protein&list_uids=153792110&dopt=GenPept&RID=T2NUM92A014&log$=prottop&blast_rank=4) | HEG homolog 1 precursor [Homo sapiens] | [20.2](http://blast.ncbi.nlm.nih.gov/Blast.cgi#153792110%23153792110) | 20.2 | 66% | 134 |
| [XP_002348185.1](http://www.ncbi.nlm.nih.gov/entrez/query.fcgi?cmd=Retrieve&db=Protein&list_uids=239752263&dopt=GenPept&RID=T2NUM92A014&log$=prottop&blast_rank=5) | PREDICTED: hypothetical protein XP_002348185 [Homo sapiens] | [19.7](http://blast.ncbi.nlm.nih.gov/Blast.cgi#239752263%23239752263) | 19.7 | 66% | 180 |
| [NP_115895.2](http://www.ncbi.nlm.nih.gov/entrez/query.fcgi?cmd=Retrieve&db=Protein&list_uids=194018431&dopt=GenPept&RID=T2NUM92A014&log$=prottop&blast_rank=6) | KIAA1841 protein isoform b [Homo sapiens] | [19.3](http://blast.ncbi.nlm.nih.gov/Blast.cgi#194018431%23194018431) | 19.3 | 77% | 241 |
| [NP_658988.2](http://www.ncbi.nlm.nih.gov/entrez/query.fcgi?cmd=Retrieve&db=Protein&list_uids=146260268&dopt=GenPept&RID=T2NUM92A014&log$=prottop&blast_rank=7) | Smith-Magenis syndrome chromosome region, candidate 8 [Homo sapiens] | [19.3](http://blast.ncbi.nlm.nih.gov/Blast.cgi#146260268%23146260268) | 19.3 | 77% | 241 |
| [NP_001123465.1](http://www.ncbi.nlm.nih.gov/entrez/query.fcgi?cmd=Retrieve&db=Protein&list_uids=194018433&dopt=GenPept&RID=T2NUM92A014&log$=prottop&blast_rank=8) | KIAA1841 protein isoform a [Homo sapiens] | [19.3](http://blast.ncbi.nlm.nih.gov/Blast.cgi#194018433%23194018433) | 19.3 | 77% | 241 |
| [NP_001106878.1](http://www.ncbi.nlm.nih.gov/entrez/query.fcgi?cmd=Retrieve&db=Protein&list_uids=164663816&dopt=GenPept&RID=T2NUM92A014&log$=prottop&blast_rank=9) | LIM domain binding 1 isoform 1 [Homo sapiens] | [19.3](http://blast.ncbi.nlm.nih.gov/Blast.cgi#164663816%23164663816) | 19.3 | 77% | 241 |
| [NP_789786.2](http://www.ncbi.nlm.nih.gov/entrez/query.fcgi?cmd=Retrieve&db=Protein&list_uids=88014609&dopt=GenPept&RID=T2NUM92A014&log$=prottop&blast_rank=10) | coiled-coil domain containing 125 [Homo sapiens] | [19.3](http://blast.ncbi.nlm.nih.gov/Blast.cgi#88014609%2388014609) | 19.3 | 77% | 241 |
| [NP_056209.2](http://www.ncbi.nlm.nih.gov/entrez/query.fcgi?cmd=Retrieve&db=Protein&list_uids=21314662&dopt=GenPept&RID=T2NUM92A014&log$=prottop&blast_rank=11) | zinc finger protein 10 [Homo sapiens] | [19.3](http://blast.ncbi.nlm.nih.gov/Blast.cgi#21314662%2321314662) | 19.3 | 77% | 241 |
| [NP_003884.1](http://www.ncbi.nlm.nih.gov/entrez/query.fcgi?cmd=Retrieve&db=Protein&list_uids=4504969&dopt=GenPept&RID=T2NUM92A014&log$=prottop&blast_rank=12) | LIM domain binding 1 isoform 3 [Homo sapiens] | [19.3](http://blast.ncbi.nlm.nih.gov/Blast.cgi#4504969%234504969) | 19.3 | 77% | 241 |
| [NP_008969.2](http://www.ncbi.nlm.nih.gov/entrez/query.fcgi?cmd=Retrieve&db=Protein&list_uids=195539372&dopt=GenPept&RID=T2NUM92A014&log$=prottop&blast_rank=13) | ADAM metallopeptidase with thrombospondin type 1 motif, 5 preproprotein [Homo sapiens] | [18.9](http://blast.ncbi.nlm.nih.gov/Blast.cgi#195539372%23195539372) | 18.9 | 77% | 324 |
| [NP_473366.1](http://www.ncbi.nlm.nih.gov/entrez/query.fcgi?cmd=Retrieve&db=Protein&list_uids=16905510&dopt=GenPept&RID=T2NUM92A014&log$=prottop&blast_rank=14) | beta-1,3-glucuronyltransferase 1 [Homo sapiens] >ref|NP_061114.2| beta-1,3-glucuronyltransferase 1 [Homo sapiens] | [18.9](http://blast.ncbi.nlm.nih.gov/Blast.cgi#16905510%2316905510) | 18.9 | 66% | 324 |
| [NP_000298.2](http://www.ncbi.nlm.nih.gov/entrez/query.fcgi?cmd=Retrieve&db=Protein&list_uids=110624763&dopt=GenPept&RID=T2NUM92A014&log$=prottop&blast_rank=15) | POU domain, class 3, transcription factor 4 [Homo sapiens] | [18.9](http://blast.ncbi.nlm.nih.gov/Blast.cgi#110624763%23110624763) | 18.9 | 66% | 324 |
| [NP_006400.2](http://www.ncbi.nlm.nih.gov/entrez/query.fcgi?cmd=Retrieve&db=Protein&list_uids=22907052&dopt=GenPept&RID=T2NUM92A014&log$=prottop&blast_rank=16) | actin related protein 2/3 complex subunit 1A [Homo sapiens] | [18.9](http://blast.ncbi.nlm.nih.gov/Blast.cgi#22907052%2322907052) | 18.9 | 77% | 324 |
| [NP_001139334.1](http://www.ncbi.nlm.nih.gov/entrez/query.fcgi?cmd=Retrieve&db=Protein&list_uids=224994251&dopt=GenPept&RID=T2NUM92A014&log$=prottop&blast_rank=17) | myotubularin related protein 11 isoform a [Homo sapiens] | [18.5](http://blast.ncbi.nlm.nih.gov/Blast.cgi#224994251%23224994251) | 18.5 | 44% | 434 |
| [NP_002340.2](http://www.ncbi.nlm.nih.gov/entrez/query.fcgi?cmd=Retrieve&db=Protein&list_uids=144446030&dopt=GenPept&RID=T2NUM92A014&log$=prottop&blast_rank=18) | lymphocyte antigen 75 precursor [Homo sapiens] | [18.5](http://blast.ncbi.nlm.nih.gov/Blast.cgi#144446030%23144446030) | 18.5 | 44% | 434 |
| [NP_006855.2](http://www.ncbi.nlm.nih.gov/entrez/query.fcgi?cmd=Retrieve&db=Protein&list_uids=125661045&dopt=GenPept&RID=T2NUM92A014&log$=prottop&blast_rank=19) | leukocyte immunoglobulin-like receptor, subfamily B, member 3 isoform 2 [Homo sapiens] | [18.5](http://blast.ncbi.nlm.nih.gov/Blast.cgi#125661045%23125661045) | 18.5 | 44% | 434 |
| [NP_001074919.1](http://www.ncbi.nlm.nih.gov/entrez/query.fcgi?cmd=Retrieve&db=Protein&list_uids=125661043&dopt=GenPept&RID=T2NUM92A014&log$=prottop&blast_rank=20) | leukocyte immunoglobulin-like receptor, subfamily B, member 3 isoform 1 [Homo sapiens] | [18.5](http://blast.ncbi.nlm.nih.gov/Blast.cgi#125661043%23125661043) | 18.5 | 44% | 434 |
| [NP_001026872.2](http://www.ncbi.nlm.nih.gov/entrez/query.fcgi?cmd=Retrieve&db=Protein&list_uids=91982767&dopt=GenPept&RID=T2NUM92A014&log$=prottop&blast_rank=21) | semaphorin 5B isoform 1 [Homo sapiens] | [18.5](http://blast.ncbi.nlm.nih.gov/Blast.cgi#91982767%2391982767) | 51.1 | 66% | 434 |
| [NP_870988.2](http://www.ncbi.nlm.nih.gov/entrez/query.fcgi?cmd=Retrieve&db=Protein&list_uids=89111941&dopt=GenPept&RID=T2NUM92A014&log$=prottop&blast_rank=22) | myotubularin related protein 11 isoform b [Homo sapiens] | [18.5](http://blast.ncbi.nlm.nih.gov/Blast.cgi#89111941%2389111941) | 18.5 | 44% | 434 |
| [NP_060442.2](http://www.ncbi.nlm.nih.gov/entrez/query.fcgi?cmd=Retrieve&db=Protein&list_uids=40018644&dopt=GenPept&RID=T2NUM92A014&log$=prottop&blast_rank=23) | hypothetical protein LOC55668 isoform 2 [Homo sapiens] | [18.5](http://blast.ncbi.nlm.nih.gov/Blast.cgi#40018644%2340018644) | 30.1 | 55% | 434 |
| [NP_060396.2](http://www.ncbi.nlm.nih.gov/entrez/query.fcgi?cmd=Retrieve&db=Protein&list_uids=40018646&dopt=GenPept&RID=T2NUM92A014&log$=prottop&blast_rank=24) | hypothetical protein LOC55668 isoform 1 [Homo sapiens] | [18.5](http://blast.ncbi.nlm.nih.gov/Blast.cgi#40018646%2340018646) | 30.1 | 55% | 434 |
| [NP_071927.1](http://www.ncbi.nlm.nih.gov/entrez/query.fcgi?cmd=Retrieve&db=Protein&list_uids=11968150&dopt=GenPept&RID=T2NUM92A014&log$=prottop&blast_rank=25) | GDNF-inducible zinc finger protein 1 [Homo sapiens] | [18.5](http://blast.ncbi.nlm.nih.gov/Blast.cgi#11968150%2311968150) | 18.5 | 44% | 434 |
| [NP_060716.2](http://www.ncbi.nlm.nih.gov/entrez/query.fcgi?cmd=Retrieve&db=Protein&list_uids=108936950&dopt=GenPept&RID=T2NUM92A014&log$=prottop&blast_rank=26) | coiled-coil domain containing 25 [Homo sapiens] | [18.5](http://blast.ncbi.nlm.nih.gov/Blast.cgi#108936950%23108936950) | 18.5 | 66% | 434 |
| [NP_004404.1](http://www.ncbi.nlm.nih.gov/entrez/query.fcgi?cmd=Retrieve&db=Protein&list_uids=4758190&dopt=GenPept&RID=T2NUM92A014&log$=prottop&blast_rank=27) | dipeptidase 1 precursor [Homo sapiens] >ref|NP_001121613.1| dipeptidase 1 precursor [Homo sapiens] | [18.5](http://blast.ncbi.nlm.nih.gov/Blast.cgi#4758190%234758190) | 18.5 | 44% | 434 |
| [NP_071750.1](http://www.ncbi.nlm.nih.gov/entrez/query.fcgi?cmd=Retrieve&db=Protein&list_uids=11641269&dopt=GenPept&RID=T2NUM92A014&log$=prottop&blast_rank=28) | dipeptidase 2 precursor [Homo sapiens] | [18.5](http://blast.ncbi.nlm.nih.gov/Blast.cgi#11641269%2311641269) | 18.5 | 44% | 434 |
| [NP_057175.1](http://www.ncbi.nlm.nih.gov/entrez/query.fcgi?cmd=Retrieve&db=Protein&list_uids=7705433&dopt=GenPept&RID=T2NUM92A014&log$=prottop&blast_rank=29) | eukaryotic translation initiation factor 3 subunit 6 interacting protein [Homo sapiens] | [18.5](http://blast.ncbi.nlm.nih.gov/Blast.cgi#7705433%237705433) | 18.5 | 44% | 434 |
| [NP_001127895.1](http://www.ncbi.nlm.nih.gov/entrez/query.fcgi?cmd=Retrieve&db=Protein&list_uids=197313674&dopt=GenPept&RID=T2NUM92A014&log$=prottop&blast_rank=30) | carnitine deficiency-associated gene expressed in ventricle 3 isoform c [Homo sapiens] | [18.0](http://blast.ncbi.nlm.nih.gov/Blast.cgi#197313674%23197313674) | 18.0 | 55% | 583 |
| [NP_006717.2](http://www.ncbi.nlm.nih.gov/entrez/query.fcgi?cmd=Retrieve&db=Protein&list_uids=148233596&dopt=GenPept&RID=T2NUM92A014&log$=prottop&blast_rank=31) | LPS-responsive vesicle trafficking, beach and anchor containing [Homo sapiens] | [18.0](http://blast.ncbi.nlm.nih.gov/Blast.cgi#148233596%23148233596) | 18.0 | 55% | 583 |
| [NP_001094385.1](http://www.ncbi.nlm.nih.gov/entrez/query.fcgi?cmd=Retrieve&db=Protein&list_uids=155369221&dopt=GenPept&RID=T2NUM92A014&log$=prottop&blast_rank=32) | potassium channel tetramerisation domain containing 19 [Homo sapiens] | [18.0](http://blast.ncbi.nlm.nih.gov/Blast.cgi#155369221%23155369221) | 18.0 | 55% | 583 |
| [NP_963863.1](http://www.ncbi.nlm.nih.gov/entrez/query.fcgi?cmd=Retrieve&db=Protein&list_uids=42476066&dopt=GenPept&RID=T2NUM92A014&log$=prottop&blast_rank=33) | SMG-7 homolog isoform 4 [Homo sapiens] | [18.0](http://blast.ncbi.nlm.nih.gov/Blast.cgi#42476066%2342476066) | 18.0 | 77% | 583 |
| [NP_963862.1](http://www.ncbi.nlm.nih.gov/entrez/query.fcgi?cmd=Retrieve&db=Protein&list_uids=42476070&dopt=GenPept&RID=T2NUM92A014&log$=prottop&blast_rank=34) | SMG-7 homolog isoform 2 [Homo sapiens] | [18.0](http://blast.ncbi.nlm.nih.gov/Blast.cgi#42476070%2342476070) | 18.0 | 77% | 583 |
| [NP_001137304.1](http://www.ncbi.nlm.nih.gov/entrez/query.fcgi?cmd=Retrieve&db=Protein&list_uids=219802098&dopt=GenPept&RID=T2NUM92A014&log$=prottop&blast_rank=35) | leucine twenty homeobox [Homo sapiens] | [18.0](http://blast.ncbi.nlm.nih.gov/Blast.cgi#219802098%23219802098) | 18.0 | 88% | 583 |
| [NP_015563.2](http://www.ncbi.nlm.nih.gov/entrez/query.fcgi?cmd=Retrieve&db=Protein&list_uids=47717112&dopt=GenPept&RID=T2NUM92A014&log$=prottop&blast_rank=36) | zinc finger, FYVE domain containing 9 isoform 1 [Homo sapiens] | [18.0](http://blast.ncbi.nlm.nih.gov/Blast.cgi#47717112%2347717112) | 18.0 | 55% | 583 |
| [NP_004841.2](http://www.ncbi.nlm.nih.gov/entrez/query.fcgi?cmd=Retrieve&db=Protein&list_uids=41872583&dopt=GenPept&RID=T2NUM92A014&log$=prottop&blast_rank=37) | Rho-associated, coiled-coil containing protein kinase 2 [Homo sapiens] | [18.0](http://blast.ncbi.nlm.nih.gov/Blast.cgi#41872583%2341872583) | 18.0 | 55% | 583 |
| [NP_060018.1](http://www.ncbi.nlm.nih.gov/entrez/query.fcgi?cmd=Retrieve&db=Protein&list_uids=8923710&dopt=GenPept&RID=T2NUM92A014&log$=prottop&blast_rank=38) | carnitine deficiency-associated gene expressed in ventricle 3 isoform b [Homo sapiens] | [18.0](http://blast.ncbi.nlm.nih.gov/Blast.cgi#8923710%238923710) | 18.0 | 55% | 583 |
| [NP_001127894.1](http://www.ncbi.nlm.nih.gov/entrez/query.fcgi?cmd=Retrieve&db=Protein&list_uids=197313672&dopt=GenPept&RID=T2NUM92A014&log$=prottop&blast_rank=39) | carnitine deficiency-associated gene expressed in ventricle 3 isoform a [Homo sapiens] | [18.0](http://blast.ncbi.nlm.nih.gov/Blast.cgi#197313672%23197313672) | 18.0 | 55% | 583 |
| [NP_775179.1](http://www.ncbi.nlm.nih.gov/entrez/query.fcgi?cmd=Retrieve&db=Protein&list_uids=42475558&dopt=GenPept&RID=T2NUM92A014&log$=prottop&blast_rank=40) | SMG-7 homolog isoform 1 [Homo sapiens] | [18.0](http://blast.ncbi.nlm.nih.gov/Blast.cgi#42475558%2342475558) | 18.0 | 77% | 583 |
| [NP_055289.2](http://www.ncbi.nlm.nih.gov/entrez/query.fcgi?cmd=Retrieve&db=Protein&list_uids=57242798&dopt=GenPept&RID=T2NUM92A014&log$=prottop&blast_rank=41) | acid sphingomyelinase-like phosphodiesterase 3B isoform 1 [Homo sapiens] | [18.0](http://blast.ncbi.nlm.nih.gov/Blast.cgi#57242798%2357242798) | 18.0 | 77% | 583 |
| [NP_001009568.1](http://www.ncbi.nlm.nih.gov/entrez/query.fcgi?cmd=Retrieve&db=Protein&list_uids=57242800&dopt=GenPept&RID=T2NUM92A014&log$=prottop&blast_rank=42) | acid sphingomyelinase-like phosphodiesterase 3B isoform 2 [Homo sapiens] | [18.0](http://blast.ncbi.nlm.nih.gov/Blast.cgi#57242800%2357242800) | 18.0 | 77% | 583 |
| [NP_004790.2](http://www.ncbi.nlm.nih.gov/entrez/query.fcgi?cmd=Retrieve&db=Protein&list_uids=47717106&dopt=GenPept&RID=T2NUM92A014&log$=prottop&blast_rank=43) | zinc finger, FYVE domain containing 9 isoform 3 [Homo sapiens] | [18.0](http://blast.ncbi.nlm.nih.gov/Blast.cgi#47717106%2347717106) | 18.0 | 55% | 583 |
| [NP_005397.1](http://www.ncbi.nlm.nih.gov/entrez/query.fcgi?cmd=Retrieve&db=Protein&list_uids=4885583&dopt=GenPept&RID=T2NUM92A014&log$=prottop&blast_rank=44) | Rho-associated, coiled-coil containing protein kinase 1 [Homo sapiens] | [18.0](http://blast.ncbi.nlm.nih.gov/Blast.cgi#4885583%234885583) | 18.0 | 55% | 583 |
| [NP_005432.1](http://www.ncbi.nlm.nih.gov/entrez/query.fcgi?cmd=Retrieve&db=Protein&list_uids=4885105&dopt=GenPept&RID=T2NUM92A014&log$=prottop&blast_rank=45) | chromatin assembly factor 1 subunit B [Homo sapiens] | [18.0](http://blast.ncbi.nlm.nih.gov/Blast.cgi#4885105%234885105) | 18.0 | 77% | 583 |
| [NP_653249.1](http://www.ncbi.nlm.nih.gov/entrez/query.fcgi?cmd=Retrieve&db=Protein&list_uids=21389483&dopt=GenPept&RID=T2NUM92A014&log$=prottop&blast_rank=46) | leucine-rich repeats and guanylate kinase domain containing [Homo sapiens] | [18.0](http://blast.ncbi.nlm.nih.gov/Blast.cgi#21389483%2321389483) | 18.0 | 66% | 583 |
| [NP_001004316.2](http://www.ncbi.nlm.nih.gov/entrez/query.fcgi?cmd=Retrieve&db=Protein&list_uids=262263420&dopt=GenPept&RID=T2NUM92A014&log$=prottop&blast_rank=47) | leucine, glutamate and lysine rich 1 [Homo sapiens] | [17.6](http://blast.ncbi.nlm.nih.gov/Blast.cgi#262263420%23262263420) | 17.6 | 55% | 782 |
| [NP_003835.3](http://www.ncbi.nlm.nih.gov/entrez/query.fcgi?cmd=Retrieve&db=Protein&list_uids=259906438&dopt=GenPept&RID=T2NUM92A014&log$=prottop&blast_rank=48) | tumor necrosis factor receptor superfamily, member 10a precursor [Homo sapiens] | [17.6](http://blast.ncbi.nlm.nih.gov/Blast.cgi#259906438%23259906438) | 17.6 | 44% | 782 |
| [NP_001157412.1](http://www.ncbi.nlm.nih.gov/entrez/query.fcgi?cmd=Retrieve&db=Protein&list_uids=255653002&dopt=GenPept&RID=T2NUM92A014&log$=prottop&blast_rank=49) | liver glycogen phosphorylase isoform 2 [Homo sapiens] | [17.6](http://blast.ncbi.nlm.nih.gov/Blast.cgi#255653002%23255653002) | 17.6 | 44% | 782 |
| [XP_002347935.1](http://www.ncbi.nlm.nih.gov/entrez/query.fcgi?cmd=Retrieve&db=Protein&list_uids=239751607&dopt=GenPept&RID=T2NUM92A014&log$=prottop&blast_rank=50) | PREDICTED: hypothetical protein, partial [Homo sapiens] | [17.6](http://blast.ncbi.nlm.nih.gov/Blast.cgi#239751607%23239751607) | 17.6 | 55% | 782 |
| [NP_003833.4](http://www.ncbi.nlm.nih.gov/entrez/query.fcgi?cmd=Retrieve&db=Protein&list_uids=224494019&dopt=GenPept&RID=T2NUM92A014&log$=prottop&blast_rank=51) | tumor necrosis factor receptor superfamily, member 10b isoform 1 precursor [Homo sapiens] | [17.6](http://blast.ncbi.nlm.nih.gov/Blast.cgi#224494019%23224494019) | 17.6 | 44% | 782 |
| [NP_001128686.1](http://www.ncbi.nlm.nih.gov/entrez/query.fcgi?cmd=Retrieve&db=Protein&list_uids=206725422&dopt=GenPept&RID=T2NUM92A014&log$=prottop&blast_rank=52) | G protein-coupled receptor kinase interacting ArfGAP 2 isoform 5 [Homo sapiens] | [17.6](http://blast.ncbi.nlm.nih.gov/Blast.cgi#206725422%23206725422) | 17.6 | 77% | 782 |
| [NP_003604.3](http://www.ncbi.nlm.nih.gov/entrez/query.fcgi?cmd=Retrieve&db=Protein&list_uids=192449445&dopt=GenPept&RID=T2NUM92A014&log$=prottop&blast_rank=53) | cartilage intermediate layer protein [Homo sapiens] | [17.6](http://blast.ncbi.nlm.nih.gov/Blast.cgi#192449445%23192449445) | 17.6 | 44% | 782 |
| [NP_001120727.1](http://www.ncbi.nlm.nih.gov/entrez/query.fcgi?cmd=Retrieve&db=Protein&list_uids=187937176&dopt=GenPept&RID=T2NUM92A014&log$=prottop&blast_rank=54) | NACHT, leucine rich repeat and PYD containing 7 isoform 3 [Homo sapiens] | [17.6](http://blast.ncbi.nlm.nih.gov/Blast.cgi#187937176%23187937176) | 17.6 | 44% | 782 |
| [XP_942245.3](http://www.ncbi.nlm.nih.gov/entrez/query.fcgi?cmd=Retrieve&db=Protein&list_uids=169211815&dopt=GenPept&RID=T2NUM92A014&log$=prottop&blast_rank=55) | PREDICTED: keratin associated protein 4.6 [Homo sapiens] | [17.6](http://blast.ncbi.nlm.nih.gov/Blast.cgi#169211815%23169211815) | 17.6 | 44% | 782 |
| [XP_001720844.1](http://www.ncbi.nlm.nih.gov/entrez/query.fcgi?cmd=Retrieve&db=Protein&list_uids=169173161&dopt=GenPept&RID=T2NUM92A014&log$=prottop&blast_rank=56) | PREDICTED: hypothetical protein [Homo sapiens] | [17.6](http://blast.ncbi.nlm.nih.gov/Blast.cgi#169173161%23169173161) | 17.6 | 44% | 782 |
| [XP_001716104.1](http://www.ncbi.nlm.nih.gov/entrez/query.fcgi?cmd=Retrieve&db=Protein&list_uids=169168282&dopt=GenPept&RID=T2NUM92A014&log$=prottop&blast_rank=57) | PREDICTED: hypothetical protein [Homo sapiens] | [17.6](http://blast.ncbi.nlm.nih.gov/Blast.cgi#169168282%23169168282) | 17.6 | 44% | 782 |
| [XP_001716843.1](http://www.ncbi.nlm.nih.gov/entrez/query.fcgi?cmd=Retrieve&db=Protein&list_uids=169168103&dopt=GenPept&RID=T2NUM92A014&log$=prottop&blast_rank=58) | PREDICTED: hypothetical protein [Homo sapiens] | [17.6](http://blast.ncbi.nlm.nih.gov/Blast.cgi#169168103%23169168103) | 17.6 | 44% | 782 |
| [XP_001716057.1](http://www.ncbi.nlm.nih.gov/entrez/query.fcgi?cmd=Retrieve&db=Protein&list_uids=169167521&dopt=GenPept&RID=T2NUM92A014&log$=prottop&blast_rank=59) | PREDICTED: hypothetical protein [Homo sapiens] | [17.6](http://blast.ncbi.nlm.nih.gov/Blast.cgi#169167521%23169167521) | 17.6 | 44% | 782 |
| [NP_001128685.1](http://www.ncbi.nlm.nih.gov/entrez/query.fcgi?cmd=Retrieve&db=Protein&list_uids=206725420&dopt=GenPept&RID=T2NUM92A014&log$=prottop&blast_rank=60) | G protein-coupled receptor kinase interacting ArfGAP 2 isoform 6 [Homo sapiens] | [17.6](http://blast.ncbi.nlm.nih.gov/Blast.cgi#206725420%23206725420) | 17.6 | 77% | 782 |
| [NP_055932.2](http://www.ncbi.nlm.nih.gov/entrez/query.fcgi?cmd=Retrieve&db=Protein&list_uids=155722994&dopt=GenPept&RID=T2NUM92A014&log$=prottop&blast_rank=61) | zinc finger CCCH-type containing 3 [Homo sapiens] | [17.6](http://blast.ncbi.nlm.nih.gov/Blast.cgi#155722994%23155722994) | 17.6 | 44% | 782 |
| [NP_853530.2](http://www.ncbi.nlm.nih.gov/entrez/query.fcgi?cmd=Retrieve&db=Protein&list_uids=148277064&dopt=GenPept&RID=T2NUM92A014&log$=prottop&blast_rank=62) | cut-like homeobox 1 isoform a [Homo sapiens] | [17.6](http://blast.ncbi.nlm.nih.gov/Blast.cgi#148277064%23148277064) | 17.6 | 44% | 782 |
| [NP_997320.2](http://www.ncbi.nlm.nih.gov/entrez/query.fcgi?cmd=Retrieve&db=Protein&list_uids=198442844&dopt=GenPept&RID=T2NUM92A014&log$=prottop&blast_rank=63) | dynein, axonemal, heavy chain 10 [Homo sapiens] | [17.6](http://blast.ncbi.nlm.nih.gov/Blast.cgi#198442844%23198442844) | 63.6 | 100% | 782 |
| [NP_940857.2](http://www.ncbi.nlm.nih.gov/entrez/query.fcgi?cmd=Retrieve&db=Protein&list_uids=134031945&dopt=GenPept&RID=T2NUM92A014&log$=prottop&blast_rank=64) | SCO-spondin precursor [Homo sapiens] | [17.6](http://blast.ncbi.nlm.nih.gov/Blast.cgi#134031945%23134031945) | 71.5 | 100% | 782 |
| [NP_620714.2](http://www.ncbi.nlm.nih.gov/entrez/query.fcgi?cmd=Retrieve&db=Protein&list_uids=157502181&dopt=GenPept&RID=T2NUM92A014&log$=prottop&blast_rank=65) | two pore segment channel 2 [Homo sapiens] | [17.6](http://blast.ncbi.nlm.nih.gov/Blast.cgi#157502181%23157502181) | 17.6 | 44% | 782 |
| [NP_004843.2](http://www.ncbi.nlm.nih.gov/entrez/query.fcgi?cmd=Retrieve&db=Protein&list_uids=119220564&dopt=GenPept&RID=T2NUM92A014&log$=prottop&blast_rank=66) | one cut domain, family member 2 [Homo sapiens] | [17.6](http://blast.ncbi.nlm.nih.gov/Blast.cgi#119220564%23119220564) | 17.6 | 44% | 782 |
| [NP_689472.3](http://www.ncbi.nlm.nih.gov/entrez/query.fcgi?cmd=Retrieve&db=Protein&list_uids=118421085&dopt=GenPept&RID=T2NUM92A014&log$=prottop&blast_rank=67) | leucine-rich repeat kinase 1 [Homo sapiens] | [17.6](http://blast.ncbi.nlm.nih.gov/Blast.cgi#118421085%23118421085) | 28.4 | 77% | 782 |
| [NP_056082.2](http://www.ncbi.nlm.nih.gov/entrez/query.fcgi?cmd=Retrieve&db=Protein&list_uids=116517292&dopt=GenPept&RID=T2NUM92A014&log$=prottop&blast_rank=68) | cut-like 2 [Homo sapiens] | [17.6](http://blast.ncbi.nlm.nih.gov/Blast.cgi#116517292%23116517292) | 17.6 | 44% | 782 |
| [NP_001363.2](http://www.ncbi.nlm.nih.gov/entrez/query.fcgi?cmd=Retrieve&db=Protein&list_uids=114155133&dopt=GenPept&RID=T2NUM92A014&log$=prottop&blast_rank=69) | dynein, axonemal, heavy chain 9 isoform 2 [Homo sapiens] | [17.6](http://blast.ncbi.nlm.nih.gov/Blast.cgi#114155133%23114155133) | 17.6 | 44% | 782 |
| [NP_001073871.1](http://www.ncbi.nlm.nih.gov/entrez/query.fcgi?cmd=Retrieve&db=Protein&list_uids=122937207&dopt=GenPept&RID=T2NUM92A014&log$=prottop&blast_rank=70) | coiled-coil domain containing 61 [Homo sapiens] | [17.6](http://blast.ncbi.nlm.nih.gov/Blast.cgi#122937207%23122937207) | 17.6 | 44% | 782 |
| [NP_001073898.1](http://www.ncbi.nlm.nih.gov/entrez/query.fcgi?cmd=Retrieve&db=Protein&list_uids=122937255&dopt=GenPept&RID=T2NUM92A014&log$=prottop&blast_rank=71) | NEZHA isoform 1 [Homo sapiens] | [17.6](http://blast.ncbi.nlm.nih.gov/Blast.cgi#122937255%23122937255) | 17.6 | 55% | 782 |
| [NP_001073896.1](http://www.ncbi.nlm.nih.gov/entrez/query.fcgi?cmd=Retrieve&db=Protein&list_uids=122937257&dopt=GenPept&RID=T2NUM92A014&log$=prottop&blast_rank=72) | thrombospondin, type I, domain containing 7B [Homo sapiens] | [17.6](http://blast.ncbi.nlm.nih.gov/Blast.cgi#122937257%23122937257) | 33.9 | 55% | 782 |
| [NP_005112.2](http://www.ncbi.nlm.nih.gov/entrez/query.fcgi?cmd=Retrieve&db=Protein&list_uids=102468717&dopt=GenPept&RID=T2NUM92A014&log$=prottop&blast_rank=73) | mediator complex subunit 13 [Homo sapiens] | [17.6](http://blast.ncbi.nlm.nih.gov/Blast.cgi#102468717%23102468717) | 47.3 | 100% | 782 |
| [NP_000325.4](http://www.ncbi.nlm.nih.gov/entrez/query.fcgi?cmd=Retrieve&db=Protein&list_uids=93587342&dopt=GenPept&RID=T2NUM92A014&log$=prottop&blast_rank=74) | voltage-gated sodium channel type 4 alpha [Homo sapiens] | [17.6](http://blast.ncbi.nlm.nih.gov/Blast.cgi#93587342%2393587342) | 28.4 | 77% | 782 |
| [NP_055591.2](http://www.ncbi.nlm.nih.gov/entrez/query.fcgi?cmd=Retrieve&db=Protein&list_uids=21237786&dopt=GenPept&RID=T2NUM92A014&log$=prottop&blast_rank=75) | G protein-coupled receptor kinase interacting ArfGAP 2 isoform 3 [Homo sapiens] | [17.6](http://blast.ncbi.nlm.nih.gov/Blast.cgi#21237786%2321237786) | 17.6 | 77% | 782 |
| [NP_476511.1](http://www.ncbi.nlm.nih.gov/entrez/query.fcgi?cmd=Retrieve&db=Protein&list_uids=17149832&dopt=GenPept&RID=T2NUM92A014&log$=prottop&blast_rank=76) | G protein-coupled receptor kinase interacting ArfGAP 2 isoform 2 [Homo sapiens] | [17.6](http://blast.ncbi.nlm.nih.gov/Blast.cgi#17149832%2317149832) | 17.6 | 77% | 782 |
| [NP_001018092.1](http://www.ncbi.nlm.nih.gov/entrez/query.fcgi?cmd=Retrieve&db=Protein&list_uids=66275526&dopt=GenPept&RID=T2NUM92A014&log$=prottop&blast_rank=77) | small adipocyte factor 1 [Homo sapiens] | [17.6](http://blast.ncbi.nlm.nih.gov/Blast.cgi#66275526%2366275526) | 17.6 | 44% | 782 |
| [NP_056420.3](http://www.ncbi.nlm.nih.gov/entrez/query.fcgi?cmd=Retrieve&db=Protein&list_uids=45243554&dopt=GenPept&RID=T2NUM92A014&log$=prottop&blast_rank=78) | protein tyrosine phosphatase, non-receptor type 20 isoform 2 [Homo sapiens] >ref|NP_001035846.1| protein tyrosine phosphatase, non-receptor type 20A isoform 2 [Homo sapiens] | [17.6](http://blast.ncbi.nlm.nih.gov/Blast.cgi#45243554%2345243554) | 17.6 | 66% | 782 |
| [NP_964014.1](http://www.ncbi.nlm.nih.gov/entrez/query.fcgi?cmd=Retrieve&db=Protein&list_uids=42542383&dopt=GenPept&RID=T2NUM92A014&log$=prottop&blast_rank=79) | solute carrier family 28, member 1 isoform 2 [Homo sapiens] | [17.6](http://blast.ncbi.nlm.nih.gov/Blast.cgi#42542383%2342542383) | 17.6 | 44% | 782 |
| [NP_001129496.1](http://www.ncbi.nlm.nih.gov/entrez/query.fcgi?cmd=Retrieve&db=Protein&list_uids=209862849&dopt=GenPept&RID=T2NUM92A014&log$=prottop&blast_rank=80) | Nance-Horan syndrome protein isoform 2 [Homo sapiens] | [17.6](http://blast.ncbi.nlm.nih.gov/Blast.cgi#209862849%23209862849) | 33.1 | 66% | 782 |
| [NP_940873.2](http://www.ncbi.nlm.nih.gov/entrez/query.fcgi?cmd=Retrieve&db=Protein&list_uids=157504499&dopt=GenPept&RID=T2NUM92A014&log$=prottop&blast_rank=81) | ankyrin repeat domain 47 [Homo sapiens] | [17.6](http://blast.ncbi.nlm.nih.gov/Blast.cgi#157504499%23157504499) | 17.6 | 44% | 782 |
| [XP_001726145.1](http://www.ncbi.nlm.nih.gov/entrez/query.fcgi?cmd=Retrieve&db=Protein&list_uids=169213465&dopt=GenPept&RID=T2NUM92A014&log$=prottop&blast_rank=82) | PREDICTED: hypothetical protein [Homo sapiens] >ref|XP_001726164.1| PREDICTED: hypothetical protein [Homo sapiens] | [17.6](http://blast.ncbi.nlm.nih.gov/Blast.cgi#169213465%23169213465) | 17.6 | 55% | 782 |
| [NP_055926.1](http://www.ncbi.nlm.nih.gov/entrez/query.fcgi?cmd=Retrieve&db=Protein&list_uids=62177129&dopt=GenPept&RID=T2NUM92A014&log$=prottop&blast_rank=83) | Nedd4 binding protein 3 [Homo sapiens] | [17.6](http://blast.ncbi.nlm.nih.gov/Blast.cgi#62177129%2362177129) | 17.6 | 44% | 782 |
| [NP_620477.1](http://www.ncbi.nlm.nih.gov/entrez/query.fcgi?cmd=Retrieve&db=Protein&list_uids=21040362&dopt=GenPept&RID=T2NUM92A014&log$=prottop&blast_rank=84) | beta-site APP-cleaving enzyme 2 isoform B preproprotein [Homo sapiens] | [17.6](http://blast.ncbi.nlm.nih.gov/Blast.cgi#21040362%2321040362) | 17.6 | 44% | 782 |
| [NP_001035816.1](http://www.ncbi.nlm.nih.gov/entrez/query.fcgi?cmd=Retrieve&db=Protein&list_uids=108802604&dopt=GenPept&RID=T2NUM92A014&log$=prottop&blast_rank=85) | protein tyrosine phosphatase, non-receptor type 20 isoform 1 [Homo sapiens] >ref|NP_001035848.1| protein tyrosine phosphatase, non-receptor type 20A isoform 1 [Homo sapiens] | [17.6](http://blast.ncbi.nlm.nih.gov/Blast.cgi#108802604%23108802604) | 17.6 | 66% | 782 |
| [NP_109592.1](http://www.ncbi.nlm.nih.gov/entrez/query.fcgi?cmd=Retrieve&db=Protein&list_uids=13677214&dopt=GenPept&RID=T2NUM92A014&log$=prottop&blast_rank=86) | receptor-type protein tyrosine phosphatase O isoform a precursor [Homo sapiens] | [17.6](http://blast.ncbi.nlm.nih.gov/Blast.cgi#13677214%2313677214) | 17.6 | 44% | 782 |
| [NP_003568.2](http://www.ncbi.nlm.nih.gov/entrez/query.fcgi?cmd=Retrieve&db=Protein&list_uids=71043876&dopt=GenPept&RID=T2NUM92A014&log$=prottop&blast_rank=87) | undifferentiated embryonic cell transcription factor 1 [Homo sapiens] | [17.6](http://blast.ncbi.nlm.nih.gov/Blast.cgi#71043876%2371043876) | 17.6 | 44% | 782 |
| [NP_001017995.1](http://www.ncbi.nlm.nih.gov/entrez/query.fcgi?cmd=Retrieve&db=Protein&list_uids=63055059&dopt=GenPept&RID=T2NUM92A014&log$=prottop&blast_rank=88) | SH3 and PX domains 2B [Homo sapiens] | [17.6](http://blast.ncbi.nlm.nih.gov/Blast.cgi#63055059%2363055059) | 17.6 | 44% | 782 |
| [NP_060482.2](http://www.ncbi.nlm.nih.gov/entrez/query.fcgi?cmd=Retrieve&db=Protein&list_uids=124430752&dopt=GenPept&RID=T2NUM92A014&log$=prottop&blast_rank=89) | kinesin family member 26B [Homo sapiens] | [17.6](http://blast.ncbi.nlm.nih.gov/Blast.cgi#124430752%23124430752) | 17.6 | 44% | 782 |
| [NP_588610.2](http://www.ncbi.nlm.nih.gov/entrez/query.fcgi?cmd=Retrieve&db=Protein&list_uids=62243567&dopt=GenPept&RID=T2NUM92A014&log$=prottop&blast_rank=90) | netrin receptor Unc5h1 precursor [Homo sapiens] | [17.6](http://blast.ncbi.nlm.nih.gov/Blast.cgi#62243567%2362243567) | 17.6 | 44% | 782 |
| [NP_001035818.1](http://www.ncbi.nlm.nih.gov/entrez/query.fcgi?cmd=Retrieve&db=Protein&list_uids=108802609&dopt=GenPept&RID=T2NUM92A014&log$=prottop&blast_rank=91) | protein tyrosine phosphatase, non-receptor type 20 isoform 4 [Homo sapiens] >ref|NP_001035850.1| protein tyrosine phosphatase, non-receptor type 20A isoform 4 [Homo sapiens] | [17.6](http://blast.ncbi.nlm.nih.gov/Blast.cgi#108802609%23108802609) | 17.6 | 66% | 782 |
| [NP_001035820.1](http://www.ncbi.nlm.nih.gov/entrez/query.fcgi?cmd=Retrieve&db=Protein&list_uids=108802613&dopt=GenPept&RID=T2NUM92A014&log$=prottop&blast_rank=92) | protein tyrosine phosphatase, non-receptor type 20 isoform 6 [Homo sapiens] >ref|NP_001035852.1| protein tyrosine phosphatase, non-receptor type 20A isoform 6 [Homo sapiens] | [17.6](http://blast.ncbi.nlm.nih.gov/Blast.cgi#108802613%23108802613) | 17.6 | 66% | 782 |
| [NP_001035821.1](http://www.ncbi.nlm.nih.gov/entrez/query.fcgi?cmd=Retrieve&db=Protein&list_uids=108802615&dopt=GenPept&RID=T2NUM92A014&log$=prottop&blast_rank=93) | protein tyrosine phosphatase, non-receptor type 20 isoform 7 [Homo sapiens] >ref|NP_001035853.1| protein tyrosine phosphatase, non-receptor type 20A isoform 7 [Homo sapiens] | [17.6](http://blast.ncbi.nlm.nih.gov/Blast.cgi#108802615%23108802615) | 17.6 | 66% | 782 |
| [NP_001035817.1](http://www.ncbi.nlm.nih.gov/entrez/query.fcgi?cmd=Retrieve&db=Protein&list_uids=108802607&dopt=GenPept&RID=T2NUM92A014&log$=prottop&blast_rank=94) | protein tyrosine phosphatase, non-receptor type 20 isoform 3 [Homo sapiens] >ref|NP_001035849.1| protein tyrosine phosphatase, non-receptor type 20A isoform 3 [Homo sapiens] | [17.6](http://blast.ncbi.nlm.nih.gov/Blast.cgi#108802607%23108802607) | 17.6 | 66% | 782 |
| [NP_061905.2](http://www.ncbi.nlm.nih.gov/entrez/query.fcgi?cmd=Retrieve&db=Protein&list_uids=38016904&dopt=GenPept&RID=T2NUM92A014&log$=prottop&blast_rank=95) | thrombospondin repeat containing 1 isoform 1 [Homo sapiens] | [17.6](http://blast.ncbi.nlm.nih.gov/Blast.cgi#38016904%2338016904) | 17.6 | 44% | 782 |
| [NP_109594.1](http://www.ncbi.nlm.nih.gov/entrez/query.fcgi?cmd=Retrieve&db=Protein&list_uids=13677218&dopt=GenPept&RID=T2NUM92A014&log$=prottop&blast_rank=96) | receptor-type protein tyrosine phosphatase O isoform c precursor [Homo sapiens] >ref|NP_109596.1| receptor-type protein tyrosine phosphatase O isoform c precursor [Homo sapiens] | [17.6](http://blast.ncbi.nlm.nih.gov/Blast.cgi#13677218%2313677218) | 17.6 | 44% | 782 |
| [NP_777584.1](http://www.ncbi.nlm.nih.gov/entrez/query.fcgi?cmd=Retrieve&db=Protein&list_uids=28372543&dopt=GenPept&RID=T2NUM92A014&log$=prottop&blast_rank=97) | protein disulfide isomerase-like, testis expressed precursor [Homo sapiens] | [17.6](http://blast.ncbi.nlm.nih.gov/Blast.cgi#28372543%2328372543) | 17.6 | 44% | 782 |
| [NP_116565.2](http://www.ncbi.nlm.nih.gov/entrez/query.fcgi?cmd=Retrieve&db=Protein&list_uids=40805108&dopt=GenPept&RID=T2NUM92A014&log$=prottop&blast_rank=98) | putative small membrane protein NID67 [Homo sapiens] | [17.6](http://blast.ncbi.nlm.nih.gov/Blast.cgi#40805108%2340805108) | 17.6 | 44% | 782 |
| [NP_036237.2](http://www.ncbi.nlm.nih.gov/entrez/query.fcgi?cmd=Retrieve&db=Protein&list_uids=19923395&dopt=GenPept&RID=T2NUM92A014&log$=prottop&blast_rank=99) | beta-site APP-cleaving enzyme 2 isoform A preproprotein [Homo sapiens] | [17.6](http://blast.ncbi.nlm.nih.gov/Blast.cgi#19923395%2319923395) | 17.6 | 44% | 782 |
| [NP_002854.3](http://www.ncbi.nlm.nih.gov/entrez/query.fcgi?cmd=Retrieve&db=Protein&list_uids=71037379&dopt=GenPept&RID=T2NUM92A014&log$=prottop&blast_rank=100) | liver glycogen phosphorylase isoform 1 [Homo sapiens] | [17.6](http://blast.ncbi.nlm.nih.gov/Blast.cgi#71037379%2371037379) | 17.6 | 44% | 782 |

| **Accession** | **Proteins with a match to IPLPPPSRPF peptide** | **[Max score](http://blast.ncbi.nlm.nih.gov/Blast.cgi?CMD=Get&ALIGNMENTS=100&ALIGNMENT_VIEW=Pairwise&CDD_SEARCH_STATE=1&DATABASE_SORT=0&DESCRIPTIONS=100&ENTREZ_QUERY=txid9606 %5BORGN%5D&FIRST_QUERY_NUM=0&FORMAT_OBJECT=Alignment&FORMAT_PAGE_TARGET=&FORMAT_TYPE=HTML&GET_SEQUENCE=yes&I_THRESH=&MASK_CHAR=2&MASK_COLOR=1&NEW_DESIGN=on&NEW_VIEW=yes&NUM_OVERVIEW=100&OLD_BLAST=false&PAGE=Proteins&QUERY_INDEX=0&QUERY_NUMBER=0&RESULTS_PAGE_TARGET=&RID=T2NZCYV001S&SHOW_LINKOUT=yes&SHOW_OVERVIEW=yes&STEP_NUMBER=&WORD_SIZE=2&DISPLAY_SORT=1&HSP_SORT=1" \l "sort_mark)** | **[Total score](http://blast.ncbi.nlm.nih.gov/Blast.cgi?CMD=Get&ALIGNMENTS=100&ALIGNMENT_VIEW=Pairwise&CDD_SEARCH_STATE=1&DATABASE_SORT=0&DESCRIPTIONS=100&ENTREZ_QUERY=txid9606 %5BORGN%5D&FIRST_QUERY_NUM=0&FORMAT_OBJECT=Alignment&FORMAT_PAGE_TARGET=&FORMAT_TYPE=HTML&GET_SEQUENCE=yes&I_THRESH=&MASK_CHAR=2&MASK_COLOR=1&NEW_DESIGN=on&NEW_VIEW=yes&NUM_OVERVIEW=100&OLD_BLAST=false&PAGE=Proteins&QUERY_INDEX=0&QUERY_NUMBER=0&RESULTS_PAGE_TARGET=&RID=T2NZCYV001S&SHOW_LINKOUT=yes&SHOW_OVERVIEW=yes&STEP_NUMBER=&WORD_SIZE=2&DISPLAY_SORT=2&HSP_SORT=1" \l "sort_mark)** | **[Query coverage](http://blast.ncbi.nlm.nih.gov/Blast.cgi?CMD=Get&ALIGNMENTS=100&ALIGNMENT_VIEW=Pairwise&CDD_SEARCH_STATE=1&DATABASE_SORT=0&DESCRIPTIONS=100&ENTREZ_QUERY=txid9606 %5BORGN%5D&FIRST_QUERY_NUM=0&FORMAT_OBJECT=Alignment&FORMAT_PAGE_TARGET=&FORMAT_TYPE=HTML&GET_SEQUENCE=yes&I_THRESH=&MASK_CHAR=2&MASK_COLOR=1&NEW_DESIGN=on&NEW_VIEW=yes&NUM_OVERVIEW=100&OLD_BLAST=false&PAGE=Proteins&QUERY_INDEX=0&QUERY_NUMBER=0&RESULTS_PAGE_TARGET=&RID=T2NZCYV001S&SHOW_LINKOUT=yes&SHOW_OVERVIEW=yes&STEP_NUMBER=&WORD_SIZE=2&DISPLAY_SORT=4&HSP_SORT=0" \l "sort_mark)** | **[E value](http://blast.ncbi.nlm.nih.gov/Blast.cgi?CMD=Get&ALIGNMENTS=100&ALIGNMENT_VIEW=Pairwise&CDD_SEARCH_STATE=1&DATABASE_SORT=0&DESCRIPTIONS=100&ENTREZ_QUERY=txid9606 %5BORGN%5D&FIRST_QUERY_NUM=0&FORMAT_OBJECT=Alignment&FORMAT_PAGE_TARGET=&FORMAT_TYPE=HTML&GET_SEQUENCE=yes&I_THRESH=&MASK_CHAR=2&MASK_COLOR=1&NEW_DESIGN=on&NEW_VIEW=yes&NUM_OVERVIEW=100&OLD_BLAST=false&PAGE=Proteins&QUERY_INDEX=0&QUERY_NUMBER=0&RESULTS_PAGE_TARGET=&RID=T2NZCYV001S&SHOW_LINKOUT=yes&SHOW_OVERVIEW=yes&STEP_NUMBER=&WORD_SIZE=2&DISPLAY_SORT=0&HSP_SORT=0" \l "sort_mark)** |
| --- | --- | --- | --- | --- | --- |
| [NP_443196.2](http://www.ncbi.nlm.nih.gov/entrez/query.fcgi?cmd=Retrieve&db=Protein&list_uids=193083221&dopt=GenPept&RID=T2NZCYV001S&log$=prottop&blast_rank=1) | mast cell immunoreceptor signal transducer [Homo sapiens] | [25.7](http://blast.ncbi.nlm.nih.gov/Blast.cgi#193083221%23193083221) | 45.8 | 90% | 3.3 |
| [XP_002343551.1](http://www.ncbi.nlm.nih.gov/entrez/query.fcgi?cmd=Retrieve&db=Protein&list_uids=239745775&dopt=GenPept&RID=T2NZCYV001S&log$=prottop&blast_rank=2) | PREDICTED: hypothetical protein XP_002343551 [Homo sapiens] >ref|XP_002347784.1| PREDICTED: hypothetical protein XP_002347784 [Homo sapiens] >ref|XP_002345054.1| PREDICTED: hypothetical protein [Homo sapiens] | [25.2](http://blast.ncbi.nlm.nih.gov/Blast.cgi#239745775%23239745775) | 25.2 | 90% | 4.4 |
| [NP_009217.3](http://www.ncbi.nlm.nih.gov/entrez/query.fcgi?cmd=Retrieve&db=Protein&list_uids=40353727&dopt=GenPept&RID=T2NZCYV001S&log$=prottop&blast_rank=3) | synaptopodin isoform A [Homo sapiens] | [25.2](http://blast.ncbi.nlm.nih.gov/Blast.cgi#40353727%2340353727) | 78.1 | 90% | 4.4 |
| [NP_004454.2](http://www.ncbi.nlm.nih.gov/entrez/query.fcgi?cmd=Retrieve&db=Protein&list_uids=24797153&dopt=GenPept&RID=T2NZCYV001S&log$=prottop&blast_rank=4) | faciogenital dysplasia protein [Homo sapiens] | [25.2](http://blast.ncbi.nlm.nih.gov/Blast.cgi#24797153%2324797153) | 25.2 | 90% | 4.4 |
| [NP_068773.2](http://www.ncbi.nlm.nih.gov/entrez/query.fcgi?cmd=Retrieve&db=Protein&list_uids=22779877&dopt=GenPept&RID=T2NZCYV001S&log$=prottop&blast_rank=5) | gap junction protein, alpha 3 [Homo sapiens] | [24.8](http://blast.ncbi.nlm.nih.gov/Blast.cgi#22779877%2322779877) | 24.8 | 80% | 5.9 |
| [NP_005482.2](http://www.ncbi.nlm.nih.gov/entrez/query.fcgi?cmd=Retrieve&db=Protein&list_uids=120952764&dopt=GenPept&RID=T2NZCYV001S&log$=prottop&blast_rank=6) | mastermind-like domain containing 1 [Homo sapiens] | [24.4](http://blast.ncbi.nlm.nih.gov/Blast.cgi#120952764%23120952764) | 70.8 | 90% | 7.9 |
| [NP_067081.2](http://www.ncbi.nlm.nih.gov/entrez/query.fcgi?cmd=Retrieve&db=Protein&list_uids=31317239&dopt=GenPept&RID=T2NZCYV001S&log$=prottop&blast_rank=7) | interleukin 22 receptor, alpha 1 precursor [Homo sapiens] | [24.4](http://blast.ncbi.nlm.nih.gov/Blast.cgi#31317239%2331317239) | 24.4 | 80% | 7.9 |
| [NP_005698.1](http://www.ncbi.nlm.nih.gov/entrez/query.fcgi?cmd=Retrieve&db=Protein&list_uids=22027541&dopt=GenPept&RID=T2NZCYV001S&log$=prottop&blast_rank=8) | programmed cell death 7 [Homo sapiens] | [24.4](http://blast.ncbi.nlm.nih.gov/Blast.cgi#22027541%2322027541) | 68.3 | 90% | 7.9 |
| [XP_001715317.1](http://www.ncbi.nlm.nih.gov/entrez/query.fcgi?cmd=Retrieve&db=Protein&list_uids=169162921&dopt=GenPept&RID=T2NZCYV001S&log$=prottop&blast_rank=9) | PREDICTED: hypothetical protein [Homo sapiens] | [24.0](http://blast.ncbi.nlm.nih.gov/Blast.cgi#169162921%23169162921) | 24.0 | 100% | 11 |
| [NP_689594.4](http://www.ncbi.nlm.nih.gov/entrez/query.fcgi?cmd=Retrieve&db=Protein&list_uids=119372317&dopt=GenPept&RID=T2NZCYV001S&log$=prottop&blast_rank=10) | xin actin-binding repeat containing 2 isoform 1 [Homo sapiens] | [24.0](http://blast.ncbi.nlm.nih.gov/Blast.cgi#119372317%23119372317) | 163 | 80% | 11 |
| [NP_055309.2](http://www.ncbi.nlm.nih.gov/entrez/query.fcgi?cmd=Retrieve&db=Protein&list_uids=116805348&dopt=GenPept&RID=T2NZCYV001S&log$=prottop&blast_rank=11) | trinucleotide repeat containing 6A [Homo sapiens] | [24.0](http://blast.ncbi.nlm.nih.gov/Blast.cgi#116805348%23116805348) | 36.1 | 90% | 11 |
| [NP_065973.2](http://www.ncbi.nlm.nih.gov/entrez/query.fcgi?cmd=Retrieve&db=Protein&list_uids=50845418&dopt=GenPept&RID=T2NZCYV001S&log$=prottop&blast_rank=12) | WNK lysine deficient protein kinase 3 isoform 1 [Homo sapiens] | [24.0](http://blast.ncbi.nlm.nih.gov/Blast.cgi#50845418%2350845418) | 24.0 | 90% | 11 |
| [NP_597701.1](http://www.ncbi.nlm.nih.gov/entrez/query.fcgi?cmd=Retrieve&db=Protein&list_uids=45331217&dopt=GenPept&RID=T2NZCYV001S&log$=prottop&blast_rank=13) | zinc finger protein 526 [Homo sapiens] | [24.0](http://blast.ncbi.nlm.nih.gov/Blast.cgi#45331217%2345331217) | 43.7 | 80% | 11 |
| [NP_001397.1](http://www.ncbi.nlm.nih.gov/entrez/query.fcgi?cmd=Retrieve&db=Protein&list_uids=4503489&dopt=GenPept&RID=T2NZCYV001S&log$=prottop&blast_rank=14) | ephrin-B3 precursor [Homo sapiens] | [24.0](http://blast.ncbi.nlm.nih.gov/Blast.cgi#4503489%234503489) | 24.0 | 80% | 11 |
| [NP_945344.1](http://www.ncbi.nlm.nih.gov/entrez/query.fcgi?cmd=Retrieve&db=Protein&list_uids=39752673&dopt=GenPept&RID=T2NZCYV001S&log$=prottop&blast_rank=15) | SH3 and cysteine rich domain 2 [Homo sapiens] | [24.0](http://blast.ncbi.nlm.nih.gov/Blast.cgi#39752673%2339752673) | 24.0 | 80% | 11 |
| [NP_000917.3](http://www.ncbi.nlm.nih.gov/entrez/query.fcgi?cmd=Retrieve&db=Protein&list_uids=110611914&dopt=GenPept&RID=T2NZCYV001S&log$=prottop&blast_rank=16) | progesterone receptor isoform B [Homo sapiens] | [24.0](http://blast.ncbi.nlm.nih.gov/Blast.cgi#110611914%23110611914) | 34.4 | 90% | 11 |
| [NP_073572.2](http://www.ncbi.nlm.nih.gov/entrez/query.fcgi?cmd=Retrieve&db=Protein&list_uids=15826852&dopt=GenPept&RID=T2NZCYV001S&log$=prottop&blast_rank=17) | acyl-Coenzyme A binding domain containing 3 [Homo sapiens] | [24.0](http://blast.ncbi.nlm.nih.gov/Blast.cgi#15826852%2315826852) | 53.7 | 80% | 11 |
| [NP_116223.3](http://www.ncbi.nlm.nih.gov/entrez/query.fcgi?cmd=Retrieve&db=Protein&list_uids=147904264&dopt=GenPept&RID=T2NZCYV001S&log$=prottop&blast_rank=18) | asparagine-linked glycosylation 10 homolog (yeast, alpha-1,2-glucosyltransferase) [Homo sapiens] | [24.0](http://blast.ncbi.nlm.nih.gov/Blast.cgi#147904264%23147904264) | 35.2 | 100% | 11 |
| [NP_001002838.1](http://www.ncbi.nlm.nih.gov/entrez/query.fcgi?cmd=Retrieve&db=Protein&list_uids=50845416&dopt=GenPept&RID=T2NZCYV001S&log$=prottop&blast_rank=19) | WNK lysine deficient protein kinase 3 isoform 2 [Homo sapiens] | [24.0](http://blast.ncbi.nlm.nih.gov/Blast.cgi#50845416%2350845416) | 24.0 | 90% | 11 |
| [NP_036200.2](http://www.ncbi.nlm.nih.gov/entrez/query.fcgi?cmd=Retrieve&db=Protein&list_uids=12597625&dopt=GenPept&RID=T2NZCYV001S&log$=prottop&blast_rank=20) | activating transcription factor 5 [Homo sapiens] | [24.0](http://blast.ncbi.nlm.nih.gov/Blast.cgi#12597625%2312597625) | 130 | 80% | 11 |
| [NP_005376.2](http://www.ncbi.nlm.nih.gov/entrez/query.fcgi?cmd=Retrieve&db=Protein&list_uids=6631100&dopt=GenPept&RID=T2NZCYV001S&log$=prottop&blast_rank=21) | natural killer-tumor recognition sequence [Homo sapiens] | [24.0](http://blast.ncbi.nlm.nih.gov/Blast.cgi#6631100%236631100) | 38.2 | 80% | 11 |
| [NP_115811.2](http://www.ncbi.nlm.nih.gov/entrez/query.fcgi?cmd=Retrieve&db=Protein&list_uids=155723000&dopt=GenPept&RID=T2NZCYV001S&log$=prottop&blast_rank=22) | mixed lineage kinase 4 [Homo sapiens] | [23.5](http://blast.ncbi.nlm.nih.gov/Blast.cgi#155723000%23155723000) | 35.6 | 80% | 14 |
| [NP_787050.6](http://www.ncbi.nlm.nih.gov/entrez/query.fcgi?cmd=Retrieve&db=Protein&list_uids=281427224&dopt=GenPept&RID=T2NZCYV001S&log$=prottop&blast_rank=23) | PAB-dependent poly(A)-specific ribonuclease subunit 3 [Homo sapiens] | [23.1](http://blast.ncbi.nlm.nih.gov/Blast.cgi#281427224%23281427224) | 42.0 | 70% | 19 |
| [XP_002347385.1](http://www.ncbi.nlm.nih.gov/entrez/query.fcgi?cmd=Retrieve&db=Protein&list_uids=239750384&dopt=GenPept&RID=T2NZCYV001S&log$=prottop&blast_rank=24) | PREDICTED: hypothetical protein XP_002347385 [Homo sapiens] | [23.1](http://blast.ncbi.nlm.nih.gov/Blast.cgi#239750384%23239750384) | 33.1 | 70% | 19 |
| [NP_064450.3](http://www.ncbi.nlm.nih.gov/entrez/query.fcgi?cmd=Retrieve&db=Protein&list_uids=160707881&dopt=GenPept&RID=T2NZCYV001S&log$=prottop&blast_rank=25) | formin 2 [Homo sapiens] | [23.1](http://blast.ncbi.nlm.nih.gov/Blast.cgi#160707881%23160707881) | 587 | 90% | 19 |
| [NP_659410.3](http://www.ncbi.nlm.nih.gov/entrez/query.fcgi?cmd=Retrieve&db=Protein&list_uids=122891862&dopt=GenPept&RID=T2NZCYV001S&log$=prottop&blast_rank=26) | DENN/MADD domain containing 5B [Homo sapiens] | [23.1](http://blast.ncbi.nlm.nih.gov/Blast.cgi#122891862%23122891862) | 23.1 | 80% | 19 |
| [NP_056028.2](http://www.ncbi.nlm.nih.gov/entrez/query.fcgi?cmd=Retrieve&db=Protein&list_uids=44889475&dopt=GenPept&RID=T2NZCYV001S&log$=prottop&blast_rank=27) | RAB6 interacting protein 1 [Homo sapiens] | [23.1](http://blast.ncbi.nlm.nih.gov/Blast.cgi#44889475%2344889475) | 23.1 | 80% | 19 |
| [NP_002025.2](http://www.ncbi.nlm.nih.gov/entrez/query.fcgi?cmd=Retrieve&db=Protein&list_uids=52485799&dopt=GenPept&RID=T2NZCYV001S&log$=prottop&blast_rank=28) | fucosyltransferase 5 [Homo sapiens] | [23.1](http://blast.ncbi.nlm.nih.gov/Blast.cgi#52485799%2352485799) | 23.1 | 70% | 19 |
| [NP_612351.2](http://www.ncbi.nlm.nih.gov/entrez/query.fcgi?cmd=Retrieve&db=Protein&list_uids=31543093&dopt=GenPept&RID=T2NZCYV001S&log$=prottop&blast_rank=29) | galactosidase, beta 1-like 2 precursor [Homo sapiens] | [23.1](http://blast.ncbi.nlm.nih.gov/Blast.cgi#31543093%2331543093) | 23.1 | 60% | 19 |
| [XP_001719523.1](http://www.ncbi.nlm.nih.gov/entrez/query.fcgi?cmd=Retrieve&db=Protein&list_uids=169218206&dopt=GenPept&RID=T2NZCYV001S&log$=prottop&blast_rank=30) | PREDICTED: similar to hCG2042704 [Homo sapiens] | [23.1](http://blast.ncbi.nlm.nih.gov/Blast.cgi#169218206%23169218206) | 36.5 | 70% | 19 |
| [NP_003378.3](http://www.ncbi.nlm.nih.gov/entrez/query.fcgi?cmd=Retrieve&db=Protein&list_uids=38373695&dopt=GenPept&RID=T2NZCYV001S&log$=prottop&blast_rank=31) | WAS/WASL interacting protein family, member 1 [Homo sapiens] >ref|NP_001070737.1| WAS/WASL interacting protein family, member 1 [Homo sapiens] | [23.1](http://blast.ncbi.nlm.nih.gov/Blast.cgi#38373695%2338373695) | 217 | 100% | 19 |
| [NP_036459.1](http://www.ncbi.nlm.nih.gov/entrez/query.fcgi?cmd=Retrieve&db=Protein&list_uids=6912500&dopt=GenPept&RID=T2NZCYV001S&log$=prottop&blast_rank=32) | phosphatidylinositol glycan anchor biosynthesis, class N [Homo sapiens] >ref|NP_789744.1| phosphatidylinositol glycan anchor biosynthesis, class N [Homo sapiens] | [23.1](http://blast.ncbi.nlm.nih.gov/Blast.cgi#6912500%236912500) | 33.5 | 90% | 19 |
| [NP_006550.1](http://www.ncbi.nlm.nih.gov/entrez/query.fcgi?cmd=Retrieve&db=Protein&list_uids=5730027&dopt=GenPept&RID=T2NZCYV001S&log$=prottop&blast_rank=33) | KH domain containing, RNA binding, signal transduction associated 1 [Homo sapiens] | [23.1](http://blast.ncbi.nlm.nih.gov/Blast.cgi#5730027%235730027) | 87.2 | 90% | 19 |
| [NP_003932.3](http://www.ncbi.nlm.nih.gov/entrez/query.fcgi?cmd=Retrieve&db=Protein&list_uids=51702526&dopt=GenPept&RID=T2NZCYV001S&log$=prottop&blast_rank=34) | Wiskott-Aldrich syndrome gene-like protein [Homo sapiens] | [23.1](http://blast.ncbi.nlm.nih.gov/Blast.cgi#51702526%2351702526) | 78.9 | 80% | 19 |
| [NP_443129.3](http://www.ncbi.nlm.nih.gov/entrez/query.fcgi?cmd=Retrieve&db=Protein&list_uids=46852161&dopt=GenPept&RID=T2NZCYV001S&log$=prottop&blast_rank=35) | methyl-CpG binding domain protein 6 [Homo sapiens] | [23.1](http://blast.ncbi.nlm.nih.gov/Blast.cgi#46852161%2346852161) | 224 | 90% | 19 |
| [NP_115605.2](http://www.ncbi.nlm.nih.gov/entrez/query.fcgi?cmd=Retrieve&db=Protein&list_uids=40217825&dopt=GenPept&RID=T2NZCYV001S&log$=prottop&blast_rank=36) | slit and trk like 6 precursor [Homo sapiens] | [23.1](http://blast.ncbi.nlm.nih.gov/Blast.cgi#40217825%2340217825) | 23.1 | 60% | 19 |
| [NP_476507.3](http://www.ncbi.nlm.nih.gov/entrez/query.fcgi?cmd=Retrieve&db=Protein&list_uids=240255535&dopt=GenPept&RID=T2NZCYV001S&log$=prottop&blast_rank=37) | alpha 3 type VI collagen isoform 4 precursor [Homo sapiens] | [22.7](http://blast.ncbi.nlm.nih.gov/Blast.cgi#240255535%23240255535) | 22.7 | 60% | 25 |
| [XP_002343711.1](http://www.ncbi.nlm.nih.gov/entrez/query.fcgi?cmd=Retrieve&db=Protein&list_uids=239746239&dopt=GenPept&RID=T2NZCYV001S&log$=prottop&blast_rank=38) | PREDICTED: hypothetical protein XP_002343711 [Homo sapiens] >ref|XP_002347978.1| PREDICTED: hypothetical protein [Homo sapiens] >ref|XP_002345213.1| PREDICTED: hypothetical protein [Homo sapiens] | [22.7](http://blast.ncbi.nlm.nih.gov/Blast.cgi#239746239%23239746239) | 22.7 | 60% | 25 |
| [XP_002343324.1](http://www.ncbi.nlm.nih.gov/entrez/query.fcgi?cmd=Retrieve&db=Protein&list_uids=239744993&dopt=GenPept&RID=T2NZCYV001S&log$=prottop&blast_rank=39) | PREDICTED: hypothetical protein XP_002343324 [Homo sapiens] >ref|XP_002347505.1| PREDICTED: hypothetical protein XP_002347505 [Homo sapiens] >ref|XP_002344830.1| PREDICTED: hypothetical protein [Homo sapiens] | [22.7](http://blast.ncbi.nlm.nih.gov/Blast.cgi#239744993%23239744993) | 22.7 | 80% | 25 |
| [NP_001129494.1](http://www.ncbi.nlm.nih.gov/entrez/query.fcgi?cmd=Retrieve&db=Protein&list_uids=209862845&dopt=GenPept&RID=T2NZCYV001S&log$=prottop&blast_rank=40) | nuclear factor of activated T-cells, cytoplasmic, calcineurin-dependent 4 isoform 1 [Homo sapiens] | [22.7](http://blast.ncbi.nlm.nih.gov/Blast.cgi#209862845%23209862845) | 68.7 | 90% | 25 |
| [NP_542417.2](http://www.ncbi.nlm.nih.gov/entrez/query.fcgi?cmd=Retrieve&db=Protein&list_uids=149158690&dopt=GenPept&RID=T2NZCYV001S&log$=prottop&blast_rank=41) | HLA-B associated transcript-2 [Homo sapiens] | [22.7](http://blast.ncbi.nlm.nih.gov/Blast.cgi#149158690%23149158690) | 242 | 100% | 25 |
| [NP_000144.2](http://www.ncbi.nlm.nih.gov/entrez/query.fcgi?cmd=Retrieve&db=Protein&list_uids=83281450&dopt=GenPept&RID=T2NZCYV001S&log$=prottop&blast_rank=42) | galactosylceramidase isoform a precursor [Homo sapiens] | [22.7](http://blast.ncbi.nlm.nih.gov/Blast.cgi#83281450%2383281450) | 22.7 | 90% | 25 |
| [NP_476508.2](http://www.ncbi.nlm.nih.gov/entrez/query.fcgi?cmd=Retrieve&db=Protein&list_uids=55743106&dopt=GenPept&RID=T2NZCYV001S&log$=prottop&blast_rank=43) | alpha 3 type VI collagen isoform 5 precursor [Homo sapiens] | [22.7](http://blast.ncbi.nlm.nih.gov/Blast.cgi#55743106%2355743106) | 22.7 | 60% | 25 |
| [NP_004360.2](http://www.ncbi.nlm.nih.gov/entrez/query.fcgi?cmd=Retrieve&db=Protein&list_uids=55743098&dopt=GenPept&RID=T2NZCYV001S&log$=prottop&blast_rank=44) | alpha 3 type VI collagen isoform 1 precursor [Homo sapiens] | [22.7](http://blast.ncbi.nlm.nih.gov/Blast.cgi#55743098%2355743098) | 22.7 | 60% | 25 |
| [NP_004545.2](http://www.ncbi.nlm.nih.gov/entrez/query.fcgi?cmd=Retrieve&db=Protein&list_uids=27886563&dopt=GenPept&RID=T2NZCYV001S&log$=prottop&blast_rank=45) | nuclear factor of activated T-cells, cytoplasmic, calcineurin-dependent 4 isoform 2 [Homo sapiens] | [22.7](http://blast.ncbi.nlm.nih.gov/Blast.cgi#27886563%2327886563) | 68.7 | 90% | 25 |
| [NP_150377.1](http://www.ncbi.nlm.nih.gov/entrez/query.fcgi?cmd=Retrieve&db=Protein&list_uids=15451844&dopt=GenPept&RID=T2NZCYV001S&log$=prottop&blast_rank=46) | ADAM metallopeptidase domain 19 preproprotein [Homo sapiens] | [22.7](http://blast.ncbi.nlm.nih.gov/Blast.cgi#15451844%2315451844) | 66.6 | 90% | 25 |
| [NP_115584.1](http://www.ncbi.nlm.nih.gov/entrez/query.fcgi?cmd=Retrieve&db=Protein&list_uids=14149904&dopt=GenPept&RID=T2NZCYV001S&log$=prottop&blast_rank=47) | anthrax toxin receptor 1 isoform 1 precursor [Homo sapiens] | [22.7](http://blast.ncbi.nlm.nih.gov/Blast.cgi#14149904%2314149904) | 39.5 | 80% | 25 |
| [NP_055428.1](http://www.ncbi.nlm.nih.gov/entrez/query.fcgi?cmd=Retrieve&db=Protein&list_uids=24797106&dopt=GenPept&RID=T2NZCYV001S&log$=prottop&blast_rank=48) | UBX domain containing 8 [Homo sapiens] | [22.7](http://blast.ncbi.nlm.nih.gov/Blast.cgi#24797106%2324797106) | 22.7 | 60% | 25 |
| [NP_005689.2](http://www.ncbi.nlm.nih.gov/entrez/query.fcgi?cmd=Retrieve&db=Protein&list_uids=16445419&dopt=GenPept&RID=T2NZCYV001S&log$=prottop&blast_rank=49) | secretory carrier membrane protein 3 isoform 1 [Homo sapiens] | [22.7](http://blast.ncbi.nlm.nih.gov/Blast.cgi#16445419%2316445419) | 22.7 | 80% | 25 |
| [NP_061918.3](http://www.ncbi.nlm.nih.gov/entrez/query.fcgi?cmd=Retrieve&db=Protein&list_uids=45238858&dopt=GenPept&RID=T2NZCYV001S&log$=prottop&blast_rank=50) | WD repeat domain 44 protein [Homo sapiens] | [22.7](http://blast.ncbi.nlm.nih.gov/Blast.cgi#45238858%2345238858) | 38.6 | 60% | 25 |
| [NP_115587.6](http://www.ncbi.nlm.nih.gov/entrez/query.fcgi?cmd=Retrieve&db=Protein&list_uids=67782349&dopt=GenPept&RID=T2NZCYV001S&log$=prottop&blast_rank=51) | lysyl oxidase-like 4 precursor [Homo sapiens] | [22.7](http://blast.ncbi.nlm.nih.gov/Blast.cgi#67782349%2367782349) | 22.7 | 60% | 25 |
| [NP_443069.1](http://www.ncbi.nlm.nih.gov/entrez/query.fcgi?cmd=Retrieve&db=Protein&list_uids=16445421&dopt=GenPept&RID=T2NZCYV001S&log$=prottop&blast_rank=52) | secretory carrier membrane protein 3 isoform 2 [Homo sapiens] | [22.7](http://blast.ncbi.nlm.nih.gov/Blast.cgi#16445421%2316445421) | 22.7 | 80% | 25 |
| [NP_001166158.1](http://www.ncbi.nlm.nih.gov/entrez/query.fcgi?cmd=Retrieve&db=Protein&list_uids=289577051&dopt=GenPept&RID=T2NZCYV001S&log$=prottop&blast_rank=53) | ADP-ribosylation factor-binding protein GGA1 isoform 4 [Homo sapiens] | [22.3](http://blast.ncbi.nlm.nih.gov/Blast.cgi#289577051%23289577051) | 22.3 | 80% | 34 |
| [XP_002344629.1](http://www.ncbi.nlm.nih.gov/entrez/query.fcgi?cmd=Retrieve&db=Protein&list_uids=239755589&dopt=GenPept&RID=T2NZCYV001S&log$=prottop&blast_rank=54) | PREDICTED: hypothetical protein [Homo sapiens] | [22.3](http://blast.ncbi.nlm.nih.gov/Blast.cgi#239755589%23239755589) | 22.3 | 80% | 34 |
| [XP_002346011.1](http://www.ncbi.nlm.nih.gov/entrez/query.fcgi?cmd=Retrieve&db=Protein&list_uids=239754512&dopt=GenPept&RID=T2NZCYV001S&log$=prottop&blast_rank=55) | PREDICTED: hypothetical protein [Homo sapiens] | [22.3](http://blast.ncbi.nlm.nih.gov/Blast.cgi#239754512%23239754512) | 22.3 | 70% | 34 |
| [XP_002347453.1](http://www.ncbi.nlm.nih.gov/entrez/query.fcgi?cmd=Retrieve&db=Protein&list_uids=239750584&dopt=GenPept&RID=T2NZCYV001S&log$=prottop&blast_rank=56) | PREDICTED: hypothetical protein [Homo sapiens] >ref|XP_002344780.1| PREDICTED: hypothetical protein [Homo sapiens] | [22.3](http://blast.ncbi.nlm.nih.gov/Blast.cgi#239750584%23239750584) | 22.3 | 60% | 34 |
| [XP_002346897.1](http://www.ncbi.nlm.nih.gov/entrez/query.fcgi?cmd=Retrieve&db=Protein&list_uids=239749073&dopt=GenPept&RID=T2NZCYV001S&log$=prottop&blast_rank=57) | PREDICTED: hypothetical protein [Homo sapiens] | [22.3](http://blast.ncbi.nlm.nih.gov/Blast.cgi#239749073%23239749073) | 67.9 | 90% | 34 |
| [XP_002344129.1](http://www.ncbi.nlm.nih.gov/entrez/query.fcgi?cmd=Retrieve&db=Protein&list_uids=239744881&dopt=GenPept&RID=T2NZCYV001S&log$=prottop&blast_rank=58) | PREDICTED: hypothetical protein XP_002344129 [Homo sapiens] | [22.3](http://blast.ncbi.nlm.nih.gov/Blast.cgi#239744881%23239744881) | 22.3 | 60% | 34 |
| [XP_002343212.1](http://www.ncbi.nlm.nih.gov/entrez/query.fcgi?cmd=Retrieve&db=Protein&list_uids=239744616&dopt=GenPept&RID=T2NZCYV001S&log$=prottop&blast_rank=59) | PREDICTED: hypothetical protein XP_002343212 [Homo sapiens] >ref|XP_002347349.1| PREDICTED: hypothetical protein XP_002347349 [Homo sapiens] >ref|XP_002344696.1| PREDICTED: hypothetical protein [Homo sapiens] | [22.3](http://blast.ncbi.nlm.nih.gov/Blast.cgi#239744616%23239744616) | 22.3 | 80% | 34 |
| [XP_002344337.1](http://www.ncbi.nlm.nih.gov/entrez/query.fcgi?cmd=Retrieve&db=Protein&list_uids=239508751&dopt=GenPept&RID=T2NZCYV001S&log$=prottop&blast_rank=60) | PREDICTED: hypothetical protein [Homo sapiens] >ref|XP_002342754.1| PREDICTED: hypothetical protein XP_002342754 [Homo sapiens] | [22.3](http://blast.ncbi.nlm.nih.gov/Blast.cgi#239508751%23239508751) | 67.9 | 90% | 34 |
| [XP_002346504.1](http://www.ncbi.nlm.nih.gov/entrez/query.fcgi?cmd=Retrieve&db=Protein&list_uids=239747993&dopt=GenPept&RID=T2NZCYV001S&log$=prottop&blast_rank=61) | PREDICTED: hypothetical protein XP_002346504 [Homo sapiens] >ref|XP_002345651.1| PREDICTED: hypothetical protein XP_002345651 [Homo sapiens] | [22.3](http://blast.ncbi.nlm.nih.gov/Blast.cgi#239747993%23239747993) | 87.0 | 80% | 34 |
| [NP_057726.3](http://www.ncbi.nlm.nih.gov/entrez/query.fcgi?cmd=Retrieve&db=Protein&list_uids=170016061&dopt=GenPept&RID=T2NZCYV001S&log$=prottop&blast_rank=62) | spectrin, beta, non-erythrocytic 5 [Homo sapiens] | [22.3](http://blast.ncbi.nlm.nih.gov/Blast.cgi#170016061%23170016061) | 22.3 | 60% | 34 |
| [NP_001166159.1](http://www.ncbi.nlm.nih.gov/entrez/query.fcgi?cmd=Retrieve&db=Protein&list_uids=289577053&dopt=GenPept&RID=T2NZCYV001S&log$=prottop&blast_rank=63) | ADP-ribosylation factor-binding protein GGA1 isoform 5 [Homo sapiens] | [22.3](http://blast.ncbi.nlm.nih.gov/Blast.cgi#289577053%23289577053) | 22.3 | 80% | 34 |
| [NP_001120979.2](http://www.ncbi.nlm.nih.gov/entrez/query.fcgi?cmd=Retrieve&db=Protein&list_uids=260763963&dopt=GenPept&RID=T2NZCYV001S&log$=prottop&blast_rank=64) | B-cell scaffold protein with ankyrin repeats 1 isoform 3 [Homo sapiens] | [22.3](http://blast.ncbi.nlm.nih.gov/Blast.cgi#260763963%23260763963) | 22.3 | 80% | 34 |
| [NP_001077376.2](http://www.ncbi.nlm.nih.gov/entrez/query.fcgi?cmd=Retrieve&db=Protein&list_uids=260763966&dopt=GenPept&RID=T2NZCYV001S&log$=prottop&blast_rank=65) | B-cell scaffold protein with ankyrin repeats 1 isoform 2 [Homo sapiens] | [22.3](http://blast.ncbi.nlm.nih.gov/Blast.cgi#260763966%23260763966) | 22.3 | 80% | 34 |
| [NP_057710.3](http://www.ncbi.nlm.nih.gov/entrez/query.fcgi?cmd=Retrieve&db=Protein&list_uids=148229134&dopt=GenPept&RID=T2NZCYV001S&log$=prottop&blast_rank=66) | ring finger and KH domain containing 2 [Homo sapiens] | [22.3](http://blast.ncbi.nlm.nih.gov/Blast.cgi#148229134%23148229134) | 67.9 | 80% | 34 |
| [NP_001137466.1](http://www.ncbi.nlm.nih.gov/entrez/query.fcgi?cmd=Retrieve&db=Protein&list_uids=221136876&dopt=GenPept&RID=T2NZCYV001S&log$=prottop&blast_rank=67) | Ras association (RalGDS/AF-6) domain family (N-terminal) member 7 isoform 3 [Homo sapiens] | [22.3](http://blast.ncbi.nlm.nih.gov/Blast.cgi#221136876%23221136876) | 22.3 | 70% | 34 |
| [NP_001104595.1](http://www.ncbi.nlm.nih.gov/entrez/query.fcgi?cmd=Retrieve&db=Protein&list_uids=162138911&dopt=GenPept&RID=T2NZCYV001S&log$=prottop&blast_rank=68) | IQ motif and Sec7 domain 2 isoform1 [Homo sapiens] | [22.3](http://blast.ncbi.nlm.nih.gov/Blast.cgi#162138911%23162138911) | 119 | 90% | 34 |
| [NP_001137465.1](http://www.ncbi.nlm.nih.gov/entrez/query.fcgi?cmd=Retrieve&db=Protein&list_uids=221136872&dopt=GenPept&RID=T2NZCYV001S&log$=prottop&blast_rank=69) | Ras association (RalGDS/AF-6) domain family (N-terminal) member 7 isoform 2 [Homo sapiens] | [22.3](http://blast.ncbi.nlm.nih.gov/Blast.cgi#221136872%23221136872) | 22.3 | 70% | 34 |
| [NP_001096654.1](http://www.ncbi.nlm.nih.gov/entrez/query.fcgi?cmd=Retrieve&db=Protein&list_uids=157168329&dopt=GenPept&RID=T2NZCYV001S&log$=prottop&blast_rank=70) | formin 1 [Homo sapiens] | [22.3](http://blast.ncbi.nlm.nih.gov/Blast.cgi#157168329%23157168329) | 76.4 | 90% | 34 |
| [NP_060959.2](http://www.ncbi.nlm.nih.gov/entrez/query.fcgi?cmd=Retrieve&db=Protein&list_uids=110349788&dopt=GenPept&RID=T2NZCYV001S&log$=prottop&blast_rank=71) | absent, small, or homeotic 1-like [Homo sapiens] | [22.3](http://blast.ncbi.nlm.nih.gov/Blast.cgi#110349788%23110349788) | 54.1 | 90% | 34 |
| [NP_543019.2](http://www.ncbi.nlm.nih.gov/entrez/query.fcgi?cmd=Retrieve&db=Protein&list_uids=109689705&dopt=GenPept&RID=T2NZCYV001S&log$=prottop&blast_rank=72) | hypothetical protein LOC140876 [Homo sapiens] | [22.3](http://blast.ncbi.nlm.nih.gov/Blast.cgi#109689705%23109689705) | 54.5 | 70% | 34 |
| [NP_653177.3](http://www.ncbi.nlm.nih.gov/entrez/query.fcgi?cmd=Retrieve&db=Protein&list_uids=151101384&dopt=GenPept&RID=T2NZCYV001S&log$=prottop&blast_rank=73) | coenzyme Q10 homolog A isoform a [Homo sapiens] | [22.3](http://blast.ncbi.nlm.nih.gov/Blast.cgi#151101384%23151101384) | 22.3 | 80% | 34 |
| [NP_001001560.1](http://www.ncbi.nlm.nih.gov/entrez/query.fcgi?cmd=Retrieve&db=Protein&list_uids=48527952&dopt=GenPept&RID=T2NZCYV001S&log$=prottop&blast_rank=74) | ADP-ribosylation factor-binding protein GGA1 isoform 2 [Homo sapiens] | [22.3](http://blast.ncbi.nlm.nih.gov/Blast.cgi#48527952%2348527952) | 22.3 | 80% | 34 |
| [NP_055863.1](http://www.ncbi.nlm.nih.gov/entrez/query.fcgi?cmd=Retrieve&db=Protein&list_uids=210032580&dopt=GenPept&RID=T2NZCYV001S&log$=prottop&blast_rank=75) | SET domain containing 1B [Homo sapiens] | [22.3](http://blast.ncbi.nlm.nih.gov/Blast.cgi#210032580%23210032580) | 212 | 100% | 34 |
| [NP_852668.1](http://www.ncbi.nlm.nih.gov/entrez/query.fcgi?cmd=Retrieve&db=Protein&list_uids=31563512&dopt=GenPept&RID=T2NZCYV001S&log$=prottop&blast_rank=76) | N-acetyltransferase 5 isoform b [Homo sapiens] | [22.3](http://blast.ncbi.nlm.nih.gov/Blast.cgi#31563512%2331563512) | 22.3 | 90% | 34 |
| [NP_078782.3](http://www.ncbi.nlm.nih.gov/entrez/query.fcgi?cmd=Retrieve&db=Protein&list_uids=40255043&dopt=GenPept&RID=T2NZCYV001S&log$=prottop&blast_rank=77) | galactosidase, beta 1-like precursor [Homo sapiens] | [22.3](http://blast.ncbi.nlm.nih.gov/Blast.cgi#40255043%2340255043) | 22.3 | 60% | 34 |
| [NP_004761.2](http://www.ncbi.nlm.nih.gov/entrez/query.fcgi?cmd=Retrieve&db=Protein&list_uids=27436974&dopt=GenPept&RID=T2NZCYV001S&log$=prottop&blast_rank=78) | potassium voltage-gated channel, Shab-related subfamily, member 2 [Homo sapiens] | [22.3](http://blast.ncbi.nlm.nih.gov/Blast.cgi#27436974%2327436974) | 22.3 | 60% | 34 |
| [NP_001396.2](http://www.ncbi.nlm.nih.gov/entrez/query.fcgi?cmd=Retrieve&db=Protein&list_uids=27894381&dopt=GenPept&RID=T2NZCYV001S&log$=prottop&blast_rank=79) | ephrin-A2 precursor [Homo sapiens] | [22.3](http://blast.ncbi.nlm.nih.gov/Blast.cgi#27894381%2327894381) | 39.5 | 90% | 34 |
| [NP_060405.4](http://www.ncbi.nlm.nih.gov/entrez/query.fcgi?cmd=Retrieve&db=Protein&list_uids=260763960&dopt=GenPept&RID=T2NZCYV001S&log$=prottop&blast_rank=80) | B-cell scaffold protein with ankyrin repeats 1 isoform 1 [Homo sapiens] | [22.3](http://blast.ncbi.nlm.nih.gov/Blast.cgi#260763960%23260763960) | 22.3 | 80% | 34 |
| [NP_055542.1](http://www.ncbi.nlm.nih.gov/entrez/query.fcgi?cmd=Retrieve&db=Protein&list_uids=7662046&dopt=GenPept&RID=T2NZCYV001S&log$=prottop&blast_rank=81) | myeloid/lymphoid or mixed-lineage leukemia 4 [Homo sapiens] | [22.3](http://blast.ncbi.nlm.nih.gov/Blast.cgi#7662046%237662046) | 201 | 80% | 34 |
| [NP_000732.2](http://www.ncbi.nlm.nih.gov/entrez/query.fcgi?cmd=Retrieve&db=Protein&list_uids=52426748&dopt=GenPept&RID=T2NZCYV001S&log$=prottop&blast_rank=82) | cholinergic receptor, muscarinic 4 [Homo sapiens] | [22.3](http://blast.ncbi.nlm.nih.gov/Blast.cgi#52426748%2352426748) | 22.3 | 70% | 34 |
| [NP_003466.1](http://www.ncbi.nlm.nih.gov/entrez/query.fcgi?cmd=Retrieve&db=Protein&list_uids=4502483&dopt=GenPept&RID=T2NZCYV001S&log$=prottop&blast_rank=83) | Ras association (RalGDS/AF-6) domain family (N-terminal) member 7 isoform 1 [Homo sapiens] | [22.3](http://blast.ncbi.nlm.nih.gov/Blast.cgi#4502483%234502483) | 38.6 | 80% | 34 |
| [NP_005841.1](http://www.ncbi.nlm.nih.gov/entrez/query.fcgi?cmd=Retrieve&db=Protein&list_uids=5032069&dopt=GenPept&RID=T2NZCYV001S&log$=prottop&blast_rank=84) | splicing factor 3b, subunit 4 [Homo sapiens] | [22.3](http://blast.ncbi.nlm.nih.gov/Blast.cgi#5032069%235032069) | 109 | 90% | 34 |
| [NP_057184.1](http://www.ncbi.nlm.nih.gov/entrez/query.fcgi?cmd=Retrieve&db=Protein&list_uids=7705823&dopt=GenPept&RID=T2NZCYV001S&log$=prottop&blast_rank=85) | N-acetyltransferase 5 isoform a [Homo sapiens] | [22.3](http://blast.ncbi.nlm.nih.gov/Blast.cgi#7705823%237705823) | 22.3 | 90% | 34 |
| [NP_036387.2](http://www.ncbi.nlm.nih.gov/entrez/query.fcgi?cmd=Retrieve&db=Protein&list_uids=18860916&dopt=GenPept&RID=T2NZCYV001S&log$=prottop&blast_rank=86) | 5'-3' exoribonuclease 2 [Homo sapiens] | [22.3](http://blast.ncbi.nlm.nih.gov/Blast.cgi#18860916%2318860916) | 22.3 | 60% | 34 |
| [NP_006284.2](http://www.ncbi.nlm.nih.gov/entrez/query.fcgi?cmd=Retrieve&db=Protein&list_uids=27597078&dopt=GenPept&RID=T2NZCYV001S&log$=prottop&blast_rank=87) | TYRO3 protein tyrosine kinase precursor [Homo sapiens] | [22.3](http://blast.ncbi.nlm.nih.gov/Blast.cgi#27597078%2327597078) | 50.7 | 90% | 34 |
| [NP_054787.1](http://www.ncbi.nlm.nih.gov/entrez/query.fcgi?cmd=Retrieve&db=Protein&list_uids=7662661&dopt=GenPept&RID=T2NZCYV001S&log$=prottop&blast_rank=88) | SEEK1 protein [Homo sapiens] | [22.3](http://blast.ncbi.nlm.nih.gov/Blast.cgi#7662661%237662661) | 22.3 | 60% | 34 |
| [NP_056207.3](http://www.ncbi.nlm.nih.gov/entrez/query.fcgi?cmd=Retrieve&db=Protein&list_uids=223029449&dopt=GenPept&RID=T2NZCYV001S&log$=prottop&blast_rank=89) | neural proliferation, differentiation and control, 1 precursor [Homo sapiens] | [22.3](http://blast.ncbi.nlm.nih.gov/Blast.cgi#223029449%23223029449) | 42.4 | 80% | 34 |
| [NP_037497.1](http://www.ncbi.nlm.nih.gov/entrez/query.fcgi?cmd=Retrieve&db=Protein&list_uids=9558729&dopt=GenPept&RID=T2NZCYV001S&log$=prottop&blast_rank=90) | ADP-ribosylation factor-binding protein GGA1 isoform 1 [Homo sapiens] | [22.3](http://blast.ncbi.nlm.nih.gov/Blast.cgi#9558729%239558729) | 22.3 | 80% | 34 |
| [NP_149015.2](http://www.ncbi.nlm.nih.gov/entrez/query.fcgi?cmd=Retrieve&db=Protein&list_uids=150378539&dopt=GenPept&RID=T2NZCYV001S&log$=prottop&blast_rank=91) | piccolo isoform 1 [Homo sapiens] | [21.8](http://blast.ncbi.nlm.nih.gov/Blast.cgi#150378539%23150378539) | 138 | 90% | 46 |
| [NP_055325.2](http://www.ncbi.nlm.nih.gov/entrez/query.fcgi?cmd=Retrieve&db=Protein&list_uids=150170670&dopt=GenPept&RID=T2NZCYV001S&log$=prottop&blast_rank=92) | piccolo isoform 2 [Homo sapiens] | [21.8](http://blast.ncbi.nlm.nih.gov/Blast.cgi#150170670%23150170670) | 125 | 90% | 46 |
| [NP_722520.2](http://www.ncbi.nlm.nih.gov/entrez/query.fcgi?cmd=Retrieve&db=Protein&list_uids=110556644&dopt=GenPept&RID=T2NZCYV001S&log$=prottop&blast_rank=93) | zinc finger protein, multitype 1 [Homo sapiens] | [21.8](http://blast.ncbi.nlm.nih.gov/Blast.cgi#110556644%23110556644) | 56.6 | 80% | 46 |
| [NP_001140.2](http://www.ncbi.nlm.nih.gov/entrez/query.fcgi?cmd=Retrieve&db=Protein&list_uids=32967599&dopt=GenPept&RID=T2NZCYV001S&log$=prottop&blast_rank=94) | ankyrin 3 isoform 2 [Homo sapiens] | [21.8](http://blast.ncbi.nlm.nih.gov/Blast.cgi#32967599%2332967599) | 37.3 | 80% | 46 |
| [NP_066267.2](http://www.ncbi.nlm.nih.gov/entrez/query.fcgi?cmd=Retrieve&db=Protein&list_uids=32967601&dopt=GenPept&RID=T2NZCYV001S&log$=prottop&blast_rank=95) | ankyrin 3 isoform 1 [Homo sapiens] | [21.8](http://blast.ncbi.nlm.nih.gov/Blast.cgi#32967601%2332967601) | 21.8 | 70% | 46 |
| [NP_064423.2](http://www.ncbi.nlm.nih.gov/entrez/query.fcgi?cmd=Retrieve&db=Protein&list_uids=21536351&dopt=GenPept&RID=T2NZCYV001S&log$=prottop&blast_rank=96) | amiloride-sensitive cation channel 2, neuronal isoform a [Homo sapiens] | [21.8](http://blast.ncbi.nlm.nih.gov/Blast.cgi#21536351%2321536351) | 38.2 | 80% | 46 |
| [NP_054790.2](http://www.ncbi.nlm.nih.gov/entrez/query.fcgi?cmd=Retrieve&db=Protein&list_uids=32307128&dopt=GenPept&RID=T2NZCYV001S&log$=prottop&blast_rank=97) | nuclear receptor coactivator 6 [Homo sapiens] | [21.8](http://blast.ncbi.nlm.nih.gov/Blast.cgi#32307128%2332307128) | 72.1 | 100% | 46 |
| [NP_067652.1](http://www.ncbi.nlm.nih.gov/entrez/query.fcgi?cmd=Retrieve&db=Protein&list_uids=11056016&dopt=GenPept&RID=T2NZCYV001S&log$=prottop&blast_rank=98) | GC-rich promoter binding protein 1-like 1 [Homo sapiens] | [21.8](http://blast.ncbi.nlm.nih.gov/Blast.cgi#11056016%2311056016) | 21.8 | 80% | 46 |
| [NP_055245.2](http://www.ncbi.nlm.nih.gov/entrez/query.fcgi?cmd=Retrieve&db=Protein&list_uids=93102358&dopt=GenPept&RID=T2NZCYV001S&log$=prottop&blast_rank=99) | cell death-inducing DFFA-like effector b [Homo sapiens] | [21.8](http://blast.ncbi.nlm.nih.gov/Blast.cgi#93102358%2393102358) | 21.8 | 70% | 46 |
| [NP_001164209.1](http://www.ncbi.nlm.nih.gov/entrez/query.fcgi?cmd=Retrieve&db=Protein&list_uids=282721018&dopt=GenPept&RID=T2NZCYV001S&log$=prottop&blast_rank=100) | IQ motif and Sec7 domain 3 isoform 1 [Homo sapiens] | [21.4](http://blast.ncbi.nlm.nih.gov/Blast.cgi#282721018%23282721018) | 41.1 | 90% | 62 |

| **Accession** | **Proteins with a match to HNTRNWTLPP peptide** | **[Max score](http://blast.ncbi.nlm.nih.gov/Blast.cgi?CMD=Get&ALIGNMENTS=100&ALIGNMENT_VIEW=Pairwise&CDD_SEARCH_STATE=1&DATABASE_SORT=0&DESCRIPTIONS=100&ENTREZ_QUERY=txid9606 %5BORGN%5D&FIRST_QUERY_NUM=0&FORMAT_OBJECT=Alignment&FORMAT_PAGE_TARGET=&FORMAT_TYPE=HTML&GET_SEQUENCE=yes&I_THRESH=&MASK_CHAR=2&MASK_COLOR=1&NEW_DESIGN=on&NEW_VIEW=yes&NUM_OVERVIEW=100&OLD_BLAST=false&PAGE=Proteins&QUERY_INDEX=0&QUERY_NUMBER=0&RESULTS_PAGE_TARGET=&RID=T2P7JMMV016&SHOW_LINKOUT=yes&SHOW_OVERVIEW=yes&STEP_NUMBER=&WORD_SIZE=2&DISPLAY_SORT=1&HSP_SORT=1" \l "sort_mark)** | **[Total score](http://blast.ncbi.nlm.nih.gov/Blast.cgi?CMD=Get&ALIGNMENTS=100&ALIGNMENT_VIEW=Pairwise&CDD_SEARCH_STATE=1&DATABASE_SORT=0&DESCRIPTIONS=100&ENTREZ_QUERY=txid9606 %5BORGN%5D&FIRST_QUERY_NUM=0&FORMAT_OBJECT=Alignment&FORMAT_PAGE_TARGET=&FORMAT_TYPE=HTML&GET_SEQUENCE=yes&I_THRESH=&MASK_CHAR=2&MASK_COLOR=1&NEW_DESIGN=on&NEW_VIEW=yes&NUM_OVERVIEW=100&OLD_BLAST=false&PAGE=Proteins&QUERY_INDEX=0&QUERY_NUMBER=0&RESULTS_PAGE_TARGET=&RID=T2P7JMMV016&SHOW_LINKOUT=yes&SHOW_OVERVIEW=yes&STEP_NUMBER=&WORD_SIZE=2&DISPLAY_SORT=2&HSP_SORT=1" \l "sort_mark)** | **[Query coverage](http://blast.ncbi.nlm.nih.gov/Blast.cgi?CMD=Get&ALIGNMENTS=100&ALIGNMENT_VIEW=Pairwise&CDD_SEARCH_STATE=1&DATABASE_SORT=0&DESCRIPTIONS=100&ENTREZ_QUERY=txid9606 %5BORGN%5D&FIRST_QUERY_NUM=0&FORMAT_OBJECT=Alignment&FORMAT_PAGE_TARGET=&FORMAT_TYPE=HTML&GET_SEQUENCE=yes&I_THRESH=&MASK_CHAR=2&MASK_COLOR=1&NEW_DESIGN=on&NEW_VIEW=yes&NUM_OVERVIEW=100&OLD_BLAST=false&PAGE=Proteins&QUERY_INDEX=0&QUERY_NUMBER=0&RESULTS_PAGE_TARGET=&RID=T2P7JMMV016&SHOW_LINKOUT=yes&SHOW_OVERVIEW=yes&STEP_NUMBER=&WORD_SIZE=2&DISPLAY_SORT=4&HSP_SORT=0" \l "sort_mark)** | **[E value](http://blast.ncbi.nlm.nih.gov/Blast.cgi?CMD=Get&ALIGNMENTS=100&ALIGNMENT_VIEW=Pairwise&CDD_SEARCH_STATE=1&DATABASE_SORT=0&DESCRIPTIONS=100&ENTREZ_QUERY=txid9606 %5BORGN%5D&FIRST_QUERY_NUM=0&FORMAT_OBJECT=Alignment&FORMAT_PAGE_TARGET=&FORMAT_TYPE=HTML&GET_SEQUENCE=yes&I_THRESH=&MASK_CHAR=2&MASK_COLOR=1&NEW_DESIGN=on&NEW_VIEW=yes&NUM_OVERVIEW=100&OLD_BLAST=false&PAGE=Proteins&QUERY_INDEX=0&QUERY_NUMBER=0&RESULTS_PAGE_TARGET=&RID=T2P7JMMV016&SHOW_LINKOUT=yes&SHOW_OVERVIEW=yes&STEP_NUMBER=&WORD_SIZE=2&DISPLAY_SORT=0&HSP_SORT=0" \l "sort_mark)** |
| --- | --- | --- | --- | --- | --- |
| [NP_036525.1](http://www.ncbi.nlm.nih.gov/entrez/query.fcgi?cmd=Retrieve&db=Protein&list_uids=31657129&dopt=GenPept&RID=T2P7JMMV016&log$=prottop&blast_rank=1) | phosphoribosylformylglycinamidine synthase [Homo sapiens] | [24.4](http://blast.ncbi.nlm.nih.gov/Blast.cgi#31657129%2331657129) | 36.5 | 80% | 7.9 |
| [NP_001122311.1](http://www.ncbi.nlm.nih.gov/entrez/query.fcgi?cmd=Retrieve&db=Protein&list_uids=192807298&dopt=GenPept&RID=T2P7JMMV016&log$=prottop&blast_rank=2) | calcium channel, voltage-dependent, L type, alpha 1D subunit isoform c [Homo sapiens] | [22.3](http://blast.ncbi.nlm.nih.gov/Blast.cgi#192807298%23192807298) | 56.2 | 100% | 34 |
| [NP_001124183.1](http://www.ncbi.nlm.nih.gov/entrez/query.fcgi?cmd=Retrieve&db=Protein&list_uids=195222725&dopt=GenPept&RID=T2P7JMMV016&log$=prottop&blast_rank=3) | C-type lectin domain family 2, member A [Homo sapiens] | [22.3](http://blast.ncbi.nlm.nih.gov/Blast.cgi#195222725%23195222725) | 22.3 | 60% | 34 |
| [NP_001122312.1](http://www.ncbi.nlm.nih.gov/entrez/query.fcgi?cmd=Retrieve&db=Protein&list_uids=192807300&dopt=GenPept&RID=T2P7JMMV016&log$=prottop&blast_rank=4) | calcium channel, voltage-dependent, L type, alpha 1D subunit isoform b [Homo sapiens] | [22.3](http://blast.ncbi.nlm.nih.gov/Blast.cgi#192807300%23192807300) | 56.2 | 100% | 34 |
| [NP_000711.1](http://www.ncbi.nlm.nih.gov/entrez/query.fcgi?cmd=Retrieve&db=Protein&list_uids=4502527&dopt=GenPept&RID=T2P7JMMV016&log$=prottop&blast_rank=5) | calcium channel, voltage-dependent, L type, alpha 1D subunit isoform a [Homo sapiens] | [22.3](http://blast.ncbi.nlm.nih.gov/Blast.cgi#4502527%234502527) | 56.2 | 100% | 34 |
| [XP_002346497.1](http://www.ncbi.nlm.nih.gov/entrez/query.fcgi?cmd=Retrieve&db=Protein&list_uids=239748036&dopt=GenPept&RID=T2P7JMMV016&log$=prottop&blast_rank=6) | PREDICTED: hypothetical protein XP_002346497 [Homo sapiens] | [21.8](http://blast.ncbi.nlm.nih.gov/Blast.cgi#239748036%23239748036) | 39.9 | 90% | 46 |
| [XP_002342355.1](http://www.ncbi.nlm.nih.gov/entrez/query.fcgi?cmd=Retrieve&db=Protein&list_uids=239741930&dopt=GenPept&RID=T2P7JMMV016&log$=prottop&blast_rank=7) | PREDICTED: hypothetical protein XP_002342355 [Homo sapiens] | [21.8](http://blast.ncbi.nlm.nih.gov/Blast.cgi#239741930%23239741930) | 39.9 | 90% | 46 |
| [XP_002342354.1](http://www.ncbi.nlm.nih.gov/entrez/query.fcgi?cmd=Retrieve&db=Protein&list_uids=239741928&dopt=GenPept&RID=T2P7JMMV016&log$=prottop&blast_rank=8) | PREDICTED: hypothetical protein XP_002342354 [Homo sapiens] | [21.8](http://blast.ncbi.nlm.nih.gov/Blast.cgi#239741928%23239741928) | 39.9 | 90% | 46 |
| [NP_001030589.1](http://www.ncbi.nlm.nih.gov/entrez/query.fcgi?cmd=Retrieve&db=Protein&list_uids=78711822&dopt=GenPept&RID=T2P7JMMV016&log$=prottop&blast_rank=9) | succinate dehydrogenase complex, subunit C isoform 3 precursor [Homo sapiens] | [21.8](http://blast.ncbi.nlm.nih.gov/Blast.cgi#78711822%2378711822) | 21.8 | 60% | 46 |
| [NP_872617.1](http://www.ncbi.nlm.nih.gov/entrez/query.fcgi?cmd=Retrieve&db=Protein&list_uids=33356541&dopt=GenPept&RID=T2P7JMMV016&log$=prottop&blast_rank=10) | phospholipid transfer protein isoform b precursor [Homo sapiens] | [21.8](http://blast.ncbi.nlm.nih.gov/Blast.cgi#33356541%2333356541) | 21.8 | 60% | 46 |
| [NP_006218.1](http://www.ncbi.nlm.nih.gov/entrez/query.fcgi?cmd=Retrieve&db=Protein&list_uids=5453914&dopt=GenPept&RID=T2P7JMMV016&log$=prottop&blast_rank=11) | phospholipid transfer protein isoform a precursor [Homo sapiens] | [21.8](http://blast.ncbi.nlm.nih.gov/Blast.cgi#5453914%235453914) | 21.8 | 60% | 46 |
| [XP_002348298.1](http://www.ncbi.nlm.nih.gov/entrez/query.fcgi?cmd=Retrieve&db=Protein&list_uids=239752604&dopt=GenPept&RID=T2P7JMMV016&log$=prottop&blast_rank=12) | PREDICTED: similar to immunoglobulin lambda-like polypeptide 1 [Homo sapiens] | [21.4](http://blast.ncbi.nlm.nih.gov/Blast.cgi#239752604%23239752604) | 37.3 | 70% | 62 |
| [NP_056667.2](http://www.ncbi.nlm.nih.gov/entrez/query.fcgi?cmd=Retrieve&db=Protein&list_uids=221316634&dopt=GenPept&RID=T2P7JMMV016&log$=prottop&blast_rank=13) | LIM domain only 7 isoform 2 [Homo sapiens] | [21.4](http://blast.ncbi.nlm.nih.gov/Blast.cgi#221316634%23221316634) | 56.4 | 100% | 62 |
| [NP_001137149.1](http://www.ncbi.nlm.nih.gov/entrez/query.fcgi?cmd=Retrieve&db=Protein&list_uids=219521878&dopt=GenPept&RID=T2P7JMMV016&log$=prottop&blast_rank=14) | serum/glucocorticoid regulated kinase 1 isoform 3 [Homo sapiens] | [21.4](http://blast.ncbi.nlm.nih.gov/Blast.cgi#219521878%23219521878) | 21.4 | 60% | 62 |
| [NP_001093322.1](http://www.ncbi.nlm.nih.gov/entrez/query.fcgi?cmd=Retrieve&db=Protein&list_uids=153792112&dopt=GenPept&RID=T2P7JMMV016&log$=prottop&blast_rank=15) | PRAME family member 20 [Homo sapiens] >ref|NP_001093584.1| PRAME family member 21 [Homo sapiens] | [21.4](http://blast.ncbi.nlm.nih.gov/Blast.cgi#153792112%23153792112) | 21.4 | 50% | 62 |
| [NP_060530.3](http://www.ncbi.nlm.nih.gov/entrez/query.fcgi?cmd=Retrieve&db=Protein&list_uids=46852147&dopt=GenPept&RID=T2P7JMMV016&log$=prottop&blast_rank=16) | mitochondrial isoleucine tRNA synthetase precursor [Homo sapiens] | [21.4](http://blast.ncbi.nlm.nih.gov/Blast.cgi#46852147%2346852147) | 21.4 | 50% | 62 |
| [NP_001985.2](http://www.ncbi.nlm.nih.gov/entrez/query.fcgi?cmd=Retrieve&db=Protein&list_uids=110611237&dopt=GenPept&RID=T2P7JMMV016&log$=prottop&blast_rank=17) | coagulation factor XIII B subunit precursor [Homo sapiens] | [21.4](http://blast.ncbi.nlm.nih.gov/Blast.cgi#110611237%23110611237) | 73.6 | 50% | 62 |
| [NP_940914.1](http://www.ncbi.nlm.nih.gov/entrez/query.fcgi?cmd=Retrieve&db=Protein&list_uids=38348340&dopt=GenPept&RID=T2P7JMMV016&log$=prottop&blast_rank=18) | diacylglycerol O-acyltransferase 2-like 6 [Homo sapiens] | [21.4](http://blast.ncbi.nlm.nih.gov/Blast.cgi#38348340%2338348340) | 21.4 | 50% | 62 |
| [NP_055542.1](http://www.ncbi.nlm.nih.gov/entrez/query.fcgi?cmd=Retrieve&db=Protein&list_uids=7662046&dopt=GenPept&RID=T2P7JMMV016&log$=prottop&blast_rank=19) | myeloid/lymphoid or mixed-lineage leukemia 4 [Homo sapiens] | [21.4](http://blast.ncbi.nlm.nih.gov/Blast.cgi#7662046%237662046) | 33.1 | 50% | 62 |
| [NP_006677.1](http://www.ncbi.nlm.nih.gov/entrez/query.fcgi?cmd=Retrieve&db=Protein&list_uids=5729722&dopt=GenPept&RID=T2P7JMMV016&log$=prottop&blast_rank=20) | actin-like 7B [Homo sapiens] | [21.4](http://blast.ncbi.nlm.nih.gov/Blast.cgi#5729722%235729722) | 21.4 | 70% | 62 |
| [NP_005746.2](http://www.ncbi.nlm.nih.gov/entrez/query.fcgi?cmd=Retrieve&db=Protein&list_uids=14577917&dopt=GenPept&RID=T2P7JMMV016&log$=prottop&blast_rank=21) | Epstein-Barr virus induced 3 precursor [Homo sapiens] | [21.4](http://blast.ncbi.nlm.nih.gov/Blast.cgi#14577917%2314577917) | 37.3 | 60% | 62 |
| [NP_001135440.1](http://www.ncbi.nlm.nih.gov/entrez/query.fcgi?cmd=Retrieve&db=Protein&list_uids=213972593&dopt=GenPept&RID=T2P7JMMV016&log$=prottop&blast_rank=22) | TPTE and PTEN homologous inositol lipid phosphatase isoform delta [Homo sapiens] | [21.0](http://blast.ncbi.nlm.nih.gov/Blast.cgi#213972593%23213972593) | 21.0 | 60% | 83 |
| [NP_954863.2](http://www.ncbi.nlm.nih.gov/entrez/query.fcgi?cmd=Retrieve&db=Protein&list_uids=213972591&dopt=GenPept&RID=T2P7JMMV016&log$=prottop&blast_rank=23) | TPTE and PTEN homologous inositol lipid phosphatase isoform gamma [Homo sapiens] | [21.0](http://blast.ncbi.nlm.nih.gov/Blast.cgi#213972591%23213972591) | 21.0 | 60% | 83 |
| [NP_570141.3](http://www.ncbi.nlm.nih.gov/entrez/query.fcgi?cmd=Retrieve&db=Protein&list_uids=213972589&dopt=GenPept&RID=T2P7JMMV016&log$=prottop&blast_rank=24) | TPTE and PTEN homologous inositol lipid phosphatase isoform alpha [Homo sapiens] | [21.0](http://blast.ncbi.nlm.nih.gov/Blast.cgi#213972589%23213972589) | 21.0 | 60% | 83 |
| [NP_954868.1](http://www.ncbi.nlm.nih.gov/entrez/query.fcgi?cmd=Retrieve&db=Protein&list_uids=40549433&dopt=GenPept&RID=T2P7JMMV016&log$=prottop&blast_rank=25) | transmembrane phosphatase with tensin homology isoform beta [Homo sapiens] | [21.0](http://blast.ncbi.nlm.nih.gov/Blast.cgi#40549433%2340549433) | 21.0 | 60% | 83 |
| [NP_954869.1](http://www.ncbi.nlm.nih.gov/entrez/query.fcgi?cmd=Retrieve&db=Protein&list_uids=40549435&dopt=GenPept&RID=T2P7JMMV016&log$=prottop&blast_rank=26) | transmembrane phosphatase with tensin homology isoform gamma [Homo sapiens] | [21.0](http://blast.ncbi.nlm.nih.gov/Blast.cgi#40549435%2340549435) | 21.0 | 60% | 83 |
| [NP_954870.2](http://www.ncbi.nlm.nih.gov/entrez/query.fcgi?cmd=Retrieve&db=Protein&list_uids=109689707&dopt=GenPept&RID=T2P7JMMV016&log$=prottop&blast_rank=27) | transmembrane phosphatase with tensin homology isoform alpha [Homo sapiens] | [21.0](http://blast.ncbi.nlm.nih.gov/Blast.cgi#109689707%23109689707) | 21.0 | 60% | 83 |
| [NP_848018.1](http://www.ncbi.nlm.nih.gov/entrez/query.fcgi?cmd=Retrieve&db=Protein&list_uids=41281911&dopt=GenPept&RID=T2P7JMMV016&log$=prottop&blast_rank=28) | Ymer protein long isoform [Homo sapiens] | [21.0](http://blast.ncbi.nlm.nih.gov/Blast.cgi#41281911%2341281911) | 21.0 | 60% | 83 |
| [NP_065996.1](http://www.ncbi.nlm.nih.gov/entrez/query.fcgi?cmd=Retrieve&db=Protein&list_uids=191252801&dopt=GenPept&RID=T2P7JMMV016&log$=prottop&blast_rank=29) | WDFY family member 4 [Homo sapiens] | [20.6](http://blast.ncbi.nlm.nih.gov/Blast.cgi#191252801%23191252801) | 20.6 | 90% | 111 |
| [NP_060203.3](http://www.ncbi.nlm.nih.gov/entrez/query.fcgi?cmd=Retrieve&db=Protein&list_uids=187608438&dopt=GenPept&RID=T2P7JMMV016&log$=prottop&blast_rank=30) | phosphatidylinositol glycan anchor biosynthesis, class G isoform 2 [Homo sapiens] | [20.6](http://blast.ncbi.nlm.nih.gov/Blast.cgi#187608438%23187608438) | 20.6 | 60% | 111 |
| [NP_803875.2](http://www.ncbi.nlm.nih.gov/entrez/query.fcgi?cmd=Retrieve&db=Protein&list_uids=126116589&dopt=GenPept&RID=T2P7JMMV016&log$=prottop&blast_rank=31) | fibrocystin L precursor [Homo sapiens] | [20.6](http://blast.ncbi.nlm.nih.gov/Blast.cgi#126116589%23126116589) | 20.6 | 90% | 111 |
| [NP_001120650.1](http://www.ncbi.nlm.nih.gov/entrez/query.fcgi?cmd=Retrieve&db=Protein&list_uids=187608446&dopt=GenPept&RID=T2P7JMMV016&log$=prottop&blast_rank=32) | phosphatidylinositol glycan anchor biosynthesis, class G isoform 1 [Homo sapiens] | [20.6](http://blast.ncbi.nlm.nih.gov/Blast.cgi#187608446%23187608446) | 20.6 | 60% | 111 |
| [NP_001367.2](http://www.ncbi.nlm.nih.gov/entrez/query.fcgi?cmd=Retrieve&db=Protein&list_uids=33350932&dopt=GenPept&RID=T2P7JMMV016&log$=prottop&blast_rank=33) | cytoplasmic dynein 1 heavy chain 1 [Homo sapiens] | [20.6](http://blast.ncbi.nlm.nih.gov/Blast.cgi#33350932%2333350932) | 20.6 | 60% | 111 |
| [NP_036538.3](http://www.ncbi.nlm.nih.gov/entrez/query.fcgi?cmd=Retrieve&db=Protein&list_uids=41349474&dopt=GenPept&RID=T2P7JMMV016&log$=prottop&blast_rank=34) | PR domain containing 4 [Homo sapiens] | [20.6](http://blast.ncbi.nlm.nih.gov/Blast.cgi#41349474%2341349474) | 20.6 | 70% | 111 |
| [NP_055812.1](http://www.ncbi.nlm.nih.gov/entrez/query.fcgi?cmd=Retrieve&db=Protein&list_uids=44917619&dopt=GenPept&RID=T2P7JMMV016&log$=prottop&blast_rank=35) | kelch domain containing 10 [Homo sapiens] | [20.6](http://blast.ncbi.nlm.nih.gov/Blast.cgi#44917619%2344917619) | 20.6 | 60% | 111 |
| [NP_060819.3](http://www.ncbi.nlm.nih.gov/entrez/query.fcgi?cmd=Retrieve&db=Protein&list_uids=227496440&dopt=GenPept&RID=T2P7JMMV016&log$=prottop&blast_rank=36) | multiple C2 domains, transmembrane 2 isoform 1 [Homo sapiens] | [20.2](http://blast.ncbi.nlm.nih.gov/Blast.cgi#227496440%23227496440) | 20.2 | 80% | 149 |
| [NP_078993.4](http://www.ncbi.nlm.nih.gov/entrez/query.fcgi?cmd=Retrieve&db=Protein&list_uids=205361109&dopt=GenPept&RID=T2P7JMMV016&log$=prottop&blast_rank=37) | multiple C2 domains, transmembrane 1 isoform L [Homo sapiens] | [20.2](http://blast.ncbi.nlm.nih.gov/Blast.cgi#205361109%23205361109) | 31.0 | 90% | 149 |
| [NP_001124390.1](http://www.ncbi.nlm.nih.gov/entrez/query.fcgi?cmd=Retrieve&db=Protein&list_uids=195963418&dopt=GenPept&RID=T2P7JMMV016&log$=prottop&blast_rank=38) | tubulin tyrosine ligase-like family, member 6 isoform 1 [Homo sapiens] | [20.2](http://blast.ncbi.nlm.nih.gov/Blast.cgi#195963418%23195963418) | 49.8 | 80% | 149 |
| [NP_001153115.1](http://www.ncbi.nlm.nih.gov/entrez/query.fcgi?cmd=Retrieve&db=Protein&list_uids=227496489&dopt=GenPept&RID=T2P7JMMV016&log$=prottop&blast_rank=39) | multiple C2 domains, transmembrane 2 isoform 2 [Homo sapiens] | [20.2](http://blast.ncbi.nlm.nih.gov/Blast.cgi#227496489%23227496489) | 20.2 | 80% | 149 |
| [NP_775894.2](http://www.ncbi.nlm.nih.gov/entrez/query.fcgi?cmd=Retrieve&db=Protein&list_uids=195963416&dopt=GenPept&RID=T2P7JMMV016&log$=prottop&blast_rank=40) | tubulin tyrosine ligase-like family, member 6 isoform 2 [Homo sapiens] | [20.2](http://blast.ncbi.nlm.nih.gov/Blast.cgi#195963416%23195963416) | 20.2 | 70% | 149 |
| [NP_001009899.2](http://www.ncbi.nlm.nih.gov/entrez/query.fcgi?cmd=Retrieve&db=Protein&list_uids=114431248&dopt=GenPept&RID=T2P7JMMV016&log$=prottop&blast_rank=41) | hypothetical protein LOC205717 [Homo sapiens] | [20.2](http://blast.ncbi.nlm.nih.gov/Blast.cgi#114431248%23114431248) | 42.6 | 90% | 149 |
| [NP_783852.1](http://www.ncbi.nlm.nih.gov/entrez/query.fcgi?cmd=Retrieve&db=Protein&list_uids=28559025&dopt=GenPept&RID=T2P7JMMV016&log$=prottop&blast_rank=42) | interleukin 5 receptor, alpha isoform 3 precursor [Homo sapiens] >ref|NP_783855.1| interleukin 5 receptor, alpha isoform 3 precursor [Homo sapiens] | [20.2](http://blast.ncbi.nlm.nih.gov/Blast.cgi#28559025%2328559025) | 20.2 | 50% | 149 |
| [NP_001106954.1](http://www.ncbi.nlm.nih.gov/entrez/query.fcgi?cmd=Retrieve&db=Protein&list_uids=164698460&dopt=GenPept&RID=T2P7JMMV016&log$=prottop&blast_rank=43) | mannosidase, endo-alpha-like isoform 3 [Homo sapiens] | [20.2](http://blast.ncbi.nlm.nih.gov/Blast.cgi#164698460%23164698460) | 32.7 | 60% | 149 |
| [NP_000555.2](http://www.ncbi.nlm.nih.gov/entrez/query.fcgi?cmd=Retrieve&db=Protein&list_uids=28559021&dopt=GenPept&RID=T2P7JMMV016&log$=prottop&blast_rank=44) | interleukin 5 receptor, alpha isoform 1 precursor [Homo sapiens] >ref|NP_783853.1| interleukin 5 receptor, alpha isoform 1 precursor [Homo sapiens] | [20.2](http://blast.ncbi.nlm.nih.gov/Blast.cgi#28559021%2328559021) | 20.2 | 50% | 149 |
| [NP_689709.1](http://www.ncbi.nlm.nih.gov/entrez/query.fcgi?cmd=Retrieve&db=Protein&list_uids=22749033&dopt=GenPept&RID=T2P7JMMV016&log$=prottop&blast_rank=45) | mannosidase, endo-alpha-like isoform 2 [Homo sapiens] | [20.2](http://blast.ncbi.nlm.nih.gov/Blast.cgi#22749033%2322749033) | 32.7 | 60% | 149 |
| [NP_001002796.1](http://www.ncbi.nlm.nih.gov/entrez/query.fcgi?cmd=Retrieve&db=Protein&list_uids=50582996&dopt=GenPept&RID=T2P7JMMV016&log$=prottop&blast_rank=46) | multiple C2 domains, transmembrane 1 isoform S [Homo sapiens] | [20.2](http://blast.ncbi.nlm.nih.gov/Blast.cgi#50582996%2350582996) | 31.0 | 90% | 149 |
| [NP_699170.1](http://www.ncbi.nlm.nih.gov/entrez/query.fcgi?cmd=Retrieve&db=Protein&list_uids=23503259&dopt=GenPept&RID=T2P7JMMV016&log$=prottop&blast_rank=47) | pseudouridylate synthase-like 1 [Homo sapiens] | [20.2](http://blast.ncbi.nlm.nih.gov/Blast.cgi#23503259%2323503259) | 20.2 | 60% | 149 |
| [NP_783851.1](http://www.ncbi.nlm.nih.gov/entrez/query.fcgi?cmd=Retrieve&db=Protein&list_uids=28559023&dopt=GenPept&RID=T2P7JMMV016&log$=prottop&blast_rank=48) | interleukin 5 receptor, alpha isoform 2 precursor [Homo sapiens] >ref|NP_783854.1| interleukin 5 receptor, alpha isoform 2 precursor [Homo sapiens] | [20.2](http://blast.ncbi.nlm.nih.gov/Blast.cgi#28559023%2328559023) | 20.2 | 50% | 149 |
| [NP_001119809.1](http://www.ncbi.nlm.nih.gov/entrez/query.fcgi?cmd=Retrieve&db=Protein&list_uids=187607364&dopt=GenPept&RID=T2P7JMMV016&log$=prottop&blast_rank=49) | tuftelin 1 isoform 2 [Homo sapiens] | [20.2](http://blast.ncbi.nlm.nih.gov/Blast.cgi#187607364%23187607364) | 20.2 | 60% | 149 |
| [NP_064512.1](http://www.ncbi.nlm.nih.gov/entrez/query.fcgi?cmd=Retrieve&db=Protein&list_uids=9910596&dopt=GenPept&RID=T2P7JMMV016&log$=prottop&blast_rank=50) | tuftelin 1 isoform 1 [Homo sapiens] | [20.2](http://blast.ncbi.nlm.nih.gov/Blast.cgi#9910596%239910596) | 20.2 | 60% | 149 |
| [NP_077007.1](http://www.ncbi.nlm.nih.gov/entrez/query.fcgi?cmd=Retrieve&db=Protein&list_uids=13129110&dopt=GenPept&RID=T2P7JMMV016&log$=prottop&blast_rank=51) | WD repeat domain 77 [Homo sapiens] | [20.2](http://blast.ncbi.nlm.nih.gov/Blast.cgi#13129110%2313129110) | 20.2 | 70% | 149 |
| [NP_671714.1](http://www.ncbi.nlm.nih.gov/entrez/query.fcgi?cmd=Retrieve&db=Protein&list_uids=22538393&dopt=GenPept&RID=T2P7JMMV016&log$=prottop&blast_rank=52) | A-kinase anchor protein 9 isoform 3 [Homo sapiens] | [20.2](http://blast.ncbi.nlm.nih.gov/Blast.cgi#22538393%2322538393) | 20.2 | 60% | 149 |
| [NP_005742.4](http://www.ncbi.nlm.nih.gov/entrez/query.fcgi?cmd=Retrieve&db=Protein&list_uids=22538387&dopt=GenPept&RID=T2P7JMMV016&log$=prottop&blast_rank=53) | A-kinase anchor protein 9 isoform 2 [Homo sapiens] | [20.2](http://blast.ncbi.nlm.nih.gov/Blast.cgi#22538387%2322538387) | 20.2 | 60% | 149 |
| [XP_002342195.1](http://www.ncbi.nlm.nih.gov/entrez/query.fcgi?cmd=Retrieve&db=Protein&list_uids=239741404&dopt=GenPept&RID=T2P7JMMV016&log$=prottop&blast_rank=54) | PREDICTED: hypothetical protein XP_002342195 [Homo sapiens] >ref|XP_002347645.1| PREDICTED: similar to hCG2042913 [Homo sapiens] >ref|XP_002345324.1| PREDICTED: similar to hCG2042913 [Homo sapiens] | [19.7](http://blast.ncbi.nlm.nih.gov/Blast.cgi#239741404%23239741404) | 19.7 | 70% | 200 |
| [NP_001129074.1](http://www.ncbi.nlm.nih.gov/entrez/query.fcgi?cmd=Retrieve&db=Protein&list_uids=208022658&dopt=GenPept&RID=T2P7JMMV016&log$=prottop&blast_rank=55) | galactosidase, beta 1 isoform c preproprotein [Homo sapiens] | [19.7](http://blast.ncbi.nlm.nih.gov/Blast.cgi#208022658%23208022658) | 19.7 | 90% | 200 |
| [NP_000395.2](http://www.ncbi.nlm.nih.gov/entrez/query.fcgi?cmd=Retrieve&db=Protein&list_uids=119372308&dopt=GenPept&RID=T2P7JMMV016&log$=prottop&blast_rank=56) | galactosidase, beta 1 isoform a preproprotein [Homo sapiens] | [19.7](http://blast.ncbi.nlm.nih.gov/Blast.cgi#119372308%23119372308) | 19.7 | 90% | 200 |
| [NP_001073279.1](http://www.ncbi.nlm.nih.gov/entrez/query.fcgi?cmd=Retrieve&db=Protein&list_uids=119372312&dopt=GenPept&RID=T2P7JMMV016&log$=prottop&blast_rank=57) | galactosidase, beta 1 isoform b [Homo sapiens] | [19.7](http://blast.ncbi.nlm.nih.gov/Blast.cgi#119372312%23119372312) | 19.7 | 90% | 200 |
| [NP_055476.3](http://www.ncbi.nlm.nih.gov/entrez/query.fcgi?cmd=Retrieve&db=Protein&list_uids=47078281&dopt=GenPept&RID=T2P7JMMV016&log$=prottop&blast_rank=58) | hypothetical protein LOC9679 [Homo sapiens] | [19.7](http://blast.ncbi.nlm.nih.gov/Blast.cgi#47078281%2347078281) | 19.7 | 60% | 200 |
| [NP_001531.1](http://www.ncbi.nlm.nih.gov/entrez/query.fcgi?cmd=Retrieve&db=Protein&list_uids=4504517&dopt=GenPept&RID=T2P7JMMV016&log$=prottop&blast_rank=59) | heat shock protein beta-1 [Homo sapiens] | [19.7](http://blast.ncbi.nlm.nih.gov/Blast.cgi#4504517%234504517) | 19.7 | 80% | 200 |
| [NP_000437.3](http://www.ncbi.nlm.nih.gov/entrez/query.fcgi?cmd=Retrieve&db=Protein&list_uids=19923106&dopt=GenPept&RID=T2P7JMMV016&log$=prottop&blast_rank=60) | paraoxonase 1 precursor [Homo sapiens] | [19.7](http://blast.ncbi.nlm.nih.gov/Blast.cgi#19923106%2319923106) | 19.7 | 60% | 200 |
| [NP_001158058.1](http://www.ncbi.nlm.nih.gov/entrez/query.fcgi?cmd=Retrieve&db=Protein&list_uids=257196151&dopt=GenPept&RID=T2P7JMMV016&log$=prottop&blast_rank=61) | immunoglobulin-like and fibronectin type III domain containing 1 [Homo sapiens] | [19.3](http://blast.ncbi.nlm.nih.gov/Blast.cgi#257196151%23257196151) | 19.3 | 50% | 268 |
| [NP_001154848.1](http://www.ncbi.nlm.nih.gov/entrez/query.fcgi?cmd=Retrieve&db=Protein&list_uids=238624195&dopt=GenPept&RID=T2P7JMMV016&log$=prottop&blast_rank=62) | hypothetical protein LOC221545 isoform 3 [Homo sapiens] | [19.3](http://blast.ncbi.nlm.nih.gov/Blast.cgi#238624195%23238624195) | 19.3 | 60% | 268 |
| [NP_001398.2](http://www.ncbi.nlm.nih.gov/entrez/query.fcgi?cmd=Retrieve&db=Protein&list_uids=145309304&dopt=GenPept&RID=T2P7JMMV016&log$=prottop&blast_rank=63) | cadherin EGF LAG seven-pass G-type receptor 3 precursor [Homo sapiens] | [19.3](http://blast.ncbi.nlm.nih.gov/Blast.cgi#145309304%23145309304) | 35.6 | 90% | 268 |
| [NP_659466.2](http://www.ncbi.nlm.nih.gov/entrez/query.fcgi?cmd=Retrieve&db=Protein&list_uids=238624193&dopt=GenPept&RID=T2P7JMMV016&log$=prottop&blast_rank=64) | hypothetical protein LOC221545 isoform 2 [Homo sapiens] | [19.3](http://blast.ncbi.nlm.nih.gov/Blast.cgi#238624193%23238624193) | 19.3 | 60% | 268 |
| [NP_001103408.1](http://www.ncbi.nlm.nih.gov/entrez/query.fcgi?cmd=Retrieve&db=Protein&list_uids=158262031&dopt=GenPept&RID=T2P7JMMV016&log$=prottop&blast_rank=65) | hypothetical protein LOC221545 isoform 1 [Homo sapiens] | [19.3](http://blast.ncbi.nlm.nih.gov/Blast.cgi#158262031%23158262031) | 19.3 | 60% | 268 |
| [NP_071336.1](http://www.ncbi.nlm.nih.gov/entrez/query.fcgi?cmd=Retrieve&db=Protein&list_uids=13430854&dopt=GenPept&RID=T2P7JMMV016&log$=prottop&blast_rank=66) | nuclear RNA export factor 2 [Homo sapiens] >ref|NP_060279.2| nuclear RNA export factor 2 [Homo sapiens] >ref|NP_001093156.1| nuclear RNA export factor 2B [Homo sapiens] | [19.3](http://blast.ncbi.nlm.nih.gov/Blast.cgi#13430854%2313430854) | 19.3 | 70% | 268 |
| [NP_008944.1](http://www.ncbi.nlm.nih.gov/entrez/query.fcgi?cmd=Retrieve&db=Protein&list_uids=13654239&dopt=GenPept&RID=T2P7JMMV016&log$=prottop&blast_rank=67) | WW domain containing E3 ubiquitin protein ligase 1 [Homo sapiens] | [19.3](http://blast.ncbi.nlm.nih.gov/Blast.cgi#13654239%2313654239) | 52.8 | 100% | 268 |
| [NP_006281.1](http://www.ncbi.nlm.nih.gov/entrez/query.fcgi?cmd=Retrieve&db=Protein&list_uids=5454132&dopt=GenPept&RID=T2P7JMMV016&log$=prottop&blast_rank=68) | tumor necrosis factor, alpha-induced protein 3 [Homo sapiens] | [19.3](http://blast.ncbi.nlm.nih.gov/Blast.cgi#5454132%235454132) | 19.3 | 50% | 268 |
| [XP_002342714.1](http://www.ncbi.nlm.nih.gov/entrez/query.fcgi?cmd=Retrieve&db=Protein&list_uids=239742995&dopt=GenPept&RID=T2P7JMMV016&log$=prottop&blast_rank=69) | PREDICTED: hypothetical protein XP_002342714 [Homo sapiens] >ref|XP_002346872.1| PREDICTED: hypothetical protein [Homo sapiens] >ref|XP_002345970.1| PREDICTED: hypothetical protein [Homo sapiens] | [18.9](http://blast.ncbi.nlm.nih.gov/Blast.cgi#239742995%23239742995) | 18.9 | 60% | 360 |
| [XP_002342391.1](http://www.ncbi.nlm.nih.gov/entrez/query.fcgi?cmd=Retrieve&db=Protein&list_uids=239742011&dopt=GenPept&RID=T2P7JMMV016&log$=prottop&blast_rank=70) | PREDICTED: hypothetical protein XP_002342391 [Homo sapiens] >ref|XP_002346555.1| PREDICTED: hypothetical protein XP_002346555 [Homo sapiens] | [18.9](http://blast.ncbi.nlm.nih.gov/Blast.cgi#239742011%23239742011) | 18.9 | 60% | 360 |
| [NP_001159444.1](http://www.ncbi.nlm.nih.gov/entrez/query.fcgi?cmd=Retrieve&db=Protein&list_uids=260166672&dopt=GenPept&RID=T2P7JMMV016&log$=prottop&blast_rank=71) | neuregulin 3 isoform 2 [Homo sapiens] | [18.9](http://blast.ncbi.nlm.nih.gov/Blast.cgi#260166672%23260166672) | 18.9 | 100% | 360 |
| [NP_001123640.1](http://www.ncbi.nlm.nih.gov/entrez/query.fcgi?cmd=Retrieve&db=Protein&list_uids=194328712&dopt=GenPept&RID=T2P7JMMV016&log$=prottop&blast_rank=72) | pregnancy specific beta-1-glycoprotein 8 isoform c [Homo sapiens] | [18.9](http://blast.ncbi.nlm.nih.gov/Blast.cgi#194328712%23194328712) | 18.9 | 50% | 360 |
| [NP_002774.2](http://www.ncbi.nlm.nih.gov/entrez/query.fcgi?cmd=Retrieve&db=Protein&list_uids=157805480&dopt=GenPept&RID=T2P7JMMV016&log$=prottop&blast_rank=73) | pregnancy specific beta-1-glycoprotein 7 precursor [Homo sapiens] | [18.9](http://blast.ncbi.nlm.nih.gov/Blast.cgi#157805480%23157805480) | 18.9 | 50% | 360 |
| [NP_001123639.1](http://www.ncbi.nlm.nih.gov/entrez/query.fcgi?cmd=Retrieve&db=Protein&list_uids=194328710&dopt=GenPept&RID=T2P7JMMV016&log$=prottop&blast_rank=74) | pregnancy specific beta-1-glycoprotein 8 isoform b [Homo sapiens] | [18.9](http://blast.ncbi.nlm.nih.gov/Blast.cgi#194328710%23194328710) | 18.9 | 50% | 360 |
| [NP_997320.2](http://www.ncbi.nlm.nih.gov/entrez/query.fcgi?cmd=Retrieve&db=Protein&list_uids=198442844&dopt=GenPept&RID=T2P7JMMV016&log$=prottop&blast_rank=75) | dynein, axonemal, heavy chain 10 [Homo sapiens] | [18.9](http://blast.ncbi.nlm.nih.gov/Blast.cgi#198442844%23198442844) | 29.3 | 60% | 360 |
| [NP_056507.2](http://www.ncbi.nlm.nih.gov/entrez/query.fcgi?cmd=Retrieve&db=Protein&list_uids=118600977&dopt=GenPept&RID=T2P7JMMV016&log$=prottop&blast_rank=76) | C3 and PZP-like, alpha-2-macroglobulin domain containing 8 [Homo sapiens] | [18.9](http://blast.ncbi.nlm.nih.gov/Blast.cgi#118600977%23118600977) | 36.9 | 90% | 360 |
| [NP_700357.1](http://www.ncbi.nlm.nih.gov/entrez/query.fcgi?cmd=Retrieve&db=Protein&list_uids=24497487&dopt=GenPept&RID=T2P7JMMV016&log$=prottop&blast_rank=77) | urate anion exchanger 1 isoform b [Homo sapiens] | [18.9](http://blast.ncbi.nlm.nih.gov/Blast.cgi#24497487%2324497487) | 18.9 | 50% | 360 |
| [NP_079273.2](http://www.ncbi.nlm.nih.gov/entrez/query.fcgi?cmd=Retrieve&db=Protein&list_uids=38569430&dopt=GenPept&RID=T2P7JMMV016&log$=prottop&blast_rank=78) | activating transcription factor 7 interacting protein 2 [Homo sapiens] | [18.9](http://blast.ncbi.nlm.nih.gov/Blast.cgi#38569430%2338569430) | 18.9 | 80% | 360 |
| [NP_006245.2](http://www.ncbi.nlm.nih.gov/entrez/query.fcgi?cmd=Retrieve&db=Protein&list_uids=31377782&dopt=GenPept&RID=T2P7JMMV016&log$=prottop&blast_rank=79) | protein kinase C, delta [Homo sapiens] >ref|NP_997704.1| protein kinase C, delta [Homo sapiens] | [18.9](http://blast.ncbi.nlm.nih.gov/Blast.cgi#31377782%2331377782) | 18.9 | 60% | 360 |
| [NP_001010848.2](http://www.ncbi.nlm.nih.gov/entrez/query.fcgi?cmd=Retrieve&db=Protein&list_uids=66730481&dopt=GenPept&RID=T2P7JMMV016&log$=prottop&blast_rank=80) | neuregulin 3 isoform 1 [Homo sapiens] | [18.9](http://blast.ncbi.nlm.nih.gov/Blast.cgi#66730481%2366730481) | 18.9 | 100% | 360 |
| [NP_991331.1](http://www.ncbi.nlm.nih.gov/entrez/query.fcgi?cmd=Retrieve&db=Protein&list_uids=45439302&dopt=GenPept&RID=T2P7JMMV016&log$=prottop&blast_rank=81) | zinc finger protein 238 isoform 1 [Homo sapiens] | [18.9](http://blast.ncbi.nlm.nih.gov/Blast.cgi#45439302%2345439302) | 18.9 | 50% | 360 |
| [NP_001032221.1](http://www.ncbi.nlm.nih.gov/entrez/query.fcgi?cmd=Retrieve&db=Protein&list_uids=80861396&dopt=GenPept&RID=T2P7JMMV016&log$=prottop&blast_rank=82) | centrobin, centrosomal BRCA2 interacting protein isoform beta [Homo sapiens] | [18.9](http://blast.ncbi.nlm.nih.gov/Blast.cgi#80861396%2380861396) | 34.8 | 50% | 360 |
| [NP_848631.2](http://www.ncbi.nlm.nih.gov/entrez/query.fcgi?cmd=Retrieve&db=Protein&list_uids=89886464&dopt=GenPept&RID=T2P7JMMV016&log$=prottop&blast_rank=83) | lipocalcin 12 precursor [Homo sapiens] | [18.9](http://blast.ncbi.nlm.nih.gov/Blast.cgi#89886464%2389886464) | 18.9 | 70% | 360 |
| [NP_444279.2](http://www.ncbi.nlm.nih.gov/entrez/query.fcgi?cmd=Retrieve&db=Protein&list_uids=191252785&dopt=GenPept&RID=T2P7JMMV016&log$=prottop&blast_rank=84) | centrobin, centrosomal BRCA2 interacting protein isoform alpha [Homo sapiens] | [18.9](http://blast.ncbi.nlm.nih.gov/Blast.cgi#191252785%23191252785) | 34.8 | 50% | 360 |
| [NP_006343.2](http://www.ncbi.nlm.nih.gov/entrez/query.fcgi?cmd=Retrieve&db=Protein&list_uids=19923354&dopt=GenPept&RID=T2P7JMMV016&log$=prottop&blast_rank=85) | zinc finger protein 238 isoform 2 [Homo sapiens] | [18.9](http://blast.ncbi.nlm.nih.gov/Blast.cgi#19923354%2319923354) | 18.9 | 50% | 360 |
| [NP_653186.2](http://www.ncbi.nlm.nih.gov/entrez/query.fcgi?cmd=Retrieve&db=Protein&list_uids=24497485&dopt=GenPept&RID=T2P7JMMV016&log$=prottop&blast_rank=86) | urate anion exchanger 1 isoform a [Homo sapiens] | [18.9](http://blast.ncbi.nlm.nih.gov/Blast.cgi#24497485%2324497485) | 18.9 | 50% | 360 |
| [NP_056244.2](http://www.ncbi.nlm.nih.gov/entrez/query.fcgi?cmd=Retrieve&db=Protein&list_uids=33667044&dopt=GenPept&RID=T2P7JMMV016&log$=prottop&blast_rank=87) | ABI gene family, member 3 (NESH) binding protein precursor [Homo sapiens] | [18.9](http://blast.ncbi.nlm.nih.gov/Blast.cgi#33667044%2333667044) | 18.9 | 50% | 360 |
| [XP_002345003.1](http://www.ncbi.nlm.nih.gov/entrez/query.fcgi?cmd=Retrieve&db=Protein&list_uids=239756495&dopt=GenPept&RID=T2P7JMMV016&log$=prottop&blast_rank=88) | PREDICTED: hypothetical protein [Homo sapiens] | [18.5](http://blast.ncbi.nlm.nih.gov/Blast.cgi#239756495%23239756495) | 18.5 | 40% | 482 |
| [XP_002344549.1](http://www.ncbi.nlm.nih.gov/entrez/query.fcgi?cmd=Retrieve&db=Protein&list_uids=239755376&dopt=GenPept&RID=T2P7JMMV016&log$=prottop&blast_rank=89) | PREDICTED: similar to hCG2044968 [Homo sapiens] | [18.5](http://blast.ncbi.nlm.nih.gov/Blast.cgi#239755376%23239755376) | 18.5 | 50% | 482 |
| [XP_002345666.1](http://www.ncbi.nlm.nih.gov/entrez/query.fcgi?cmd=Retrieve&db=Protein&list_uids=239753468&dopt=GenPept&RID=T2P7JMMV016&log$=prottop&blast_rank=90) | PREDICTED: hypothetical protein [Homo sapiens] | [18.5](http://blast.ncbi.nlm.nih.gov/Blast.cgi#239753468%23239753468) | 36.5 | 90% | 482 |
| [XP_002342863.1](http://www.ncbi.nlm.nih.gov/entrez/query.fcgi?cmd=Retrieve&db=Protein&list_uids=239743538&dopt=GenPept&RID=T2P7JMMV016&log$=prottop&blast_rank=91) | PREDICTED: hypothetical protein XP_002342863 [Homo sapiens] >ref|XP_002347022.1| PREDICTED: hypothetical protein [Homo sapiens] | [18.5](http://blast.ncbi.nlm.nih.gov/Blast.cgi#239743538%23239743538) | 18.5 | 50% | 482 |
| [NP_001162.4](http://www.ncbi.nlm.nih.gov/entrez/query.fcgi?cmd=Retrieve&db=Protein&list_uids=190343023&dopt=GenPept&RID=T2P7JMMV016&log$=prottop&blast_rank=92) | ATP-binding cassette, sub-family C, member 6 isoform 1 [Homo sapiens] | [18.5](http://blast.ncbi.nlm.nih.gov/Blast.cgi#190343023%23190343023) | 18.5 | 40% | 482 |
| [NP_079420.3](http://www.ncbi.nlm.nih.gov/entrez/query.fcgi?cmd=Retrieve&db=Protein&list_uids=156151441&dopt=GenPept&RID=T2P7JMMV016&log$=prottop&blast_rank=93) | alpha-kinase 1 [Homo sapiens] >ref|NP_001095876.1| alpha-kinase 1 [Homo sapiens] | [18.5](http://blast.ncbi.nlm.nih.gov/Blast.cgi#156151441%23156151441) | 18.5 | 40% | 482 |
| [NP_115560.1](http://www.ncbi.nlm.nih.gov/entrez/query.fcgi?cmd=Retrieve&db=Protein&list_uids=149274655&dopt=GenPept&RID=T2P7JMMV016&log$=prottop&blast_rank=94) | synapse defective 1, Rho GTPase, homolog 2 [Homo sapiens] | [18.5](http://blast.ncbi.nlm.nih.gov/Blast.cgi#149274655%23149274655) | 32.2 | 70% | 482 |
| [XP_001714591.2](http://www.ncbi.nlm.nih.gov/entrez/query.fcgi?cmd=Retrieve&db=Protein&list_uids=239752250&dopt=GenPept&RID=T2P7JMMV016&log$=prottop&blast_rank=95) | PREDICTED: similar to acetylserotonin O-methyltransferase-like [Homo sapiens] | [18.5](http://blast.ncbi.nlm.nih.gov/Blast.cgi#239752250%23239752250) | 18.5 | 100% | 482 |
| [NP_872344.3](http://www.ncbi.nlm.nih.gov/entrez/query.fcgi?cmd=Retrieve&db=Protein&list_uids=194239645&dopt=GenPept&RID=T2P7JMMV016&log$=prottop&blast_rank=96) | spinster homolog 3 [Homo sapiens] | [18.5](http://blast.ncbi.nlm.nih.gov/Blast.cgi#194239645%23194239645) | 18.5 | 50% | 482 |
| [XP_002347286.1](http://www.ncbi.nlm.nih.gov/entrez/query.fcgi?cmd=Retrieve&db=Protein&list_uids=239750069&dopt=GenPept&RID=T2P7JMMV016&log$=prottop&blast_rank=97) | PREDICTED: hypothetical protein XP_002347286 [Homo sapiens] | [18.5](http://blast.ncbi.nlm.nih.gov/Blast.cgi#239750069%23239750069) | 18.5 | 50% | 482 |
| [NP_848639.2](http://www.ncbi.nlm.nih.gov/entrez/query.fcgi?cmd=Retrieve&db=Protein&list_uids=194018469&dopt=GenPept&RID=T2P7JMMV016&log$=prottop&blast_rank=98) | zinc finger protein 546 [Homo sapiens] | [18.5](http://blast.ncbi.nlm.nih.gov/Blast.cgi#194018469%23194018469) | 18.5 | 40% | 482 |
| [NP_775483.1](http://www.ncbi.nlm.nih.gov/entrez/query.fcgi?cmd=Retrieve&db=Protein&list_uids=190886446&dopt=GenPept&RID=T2P7JMMV016&log$=prottop&blast_rank=99) | hypothetical protein LOC317662 [Homo sapiens] | [18.5](http://blast.ncbi.nlm.nih.gov/Blast.cgi#190886446%23190886446) | 18.5 | 40% | 482 |

| **Accession** | **Proteins with a match to SNVISYPDV peptide** | **[Max score](http://blast.ncbi.nlm.nih.gov/Blast.cgi?CMD=Get&ALIGNMENTS=100&ALIGNMENT_VIEW=Pairwise&CDD_SEARCH_STATE=1&DATABASE_SORT=0&DESCRIPTIONS=100&ENTREZ_QUERY=txid9606 %5BORGN%5D&FIRST_QUERY_NUM=0&FORMAT_OBJECT=Alignment&FORMAT_PAGE_TARGET=&FORMAT_TYPE=HTML&GET_SEQUENCE=yes&I_THRESH=&MASK_CHAR=2&MASK_COLOR=1&NEW_DESIGN=on&NEW_VIEW=yes&NUM_OVERVIEW=100&OLD_BLAST=false&PAGE=Proteins&QUERY_INDEX=0&QUERY_NUMBER=0&RESULTS_PAGE_TARGET=&RID=T2PJYNMM01N&SHOW_LINKOUT=yes&SHOW_OVERVIEW=yes&STEP_NUMBER=&WORD_SIZE=2&DISPLAY_SORT=1&HSP_SORT=1" \l "sort_mark)** | **[Total score](http://blast.ncbi.nlm.nih.gov/Blast.cgi?CMD=Get&ALIGNMENTS=100&ALIGNMENT_VIEW=Pairwise&CDD_SEARCH_STATE=1&DATABASE_SORT=0&DESCRIPTIONS=100&ENTREZ_QUERY=txid9606 %5BORGN%5D&FIRST_QUERY_NUM=0&FORMAT_OBJECT=Alignment&FORMAT_PAGE_TARGET=&FORMAT_TYPE=HTML&GET_SEQUENCE=yes&I_THRESH=&MASK_CHAR=2&MASK_COLOR=1&NEW_DESIGN=on&NEW_VIEW=yes&NUM_OVERVIEW=100&OLD_BLAST=false&PAGE=Proteins&QUERY_INDEX=0&QUERY_NUMBER=0&RESULTS_PAGE_TARGET=&RID=T2PJYNMM01N&SHOW_LINKOUT=yes&SHOW_OVERVIEW=yes&STEP_NUMBER=&WORD_SIZE=2&DISPLAY_SORT=2&HSP_SORT=1" \l "sort_mark)** | **[Query coverage](http://blast.ncbi.nlm.nih.gov/Blast.cgi?CMD=Get&ALIGNMENTS=100&ALIGNMENT_VIEW=Pairwise&CDD_SEARCH_STATE=1&DATABASE_SORT=0&DESCRIPTIONS=100&ENTREZ_QUERY=txid9606 %5BORGN%5D&FIRST_QUERY_NUM=0&FORMAT_OBJECT=Alignment&FORMAT_PAGE_TARGET=&FORMAT_TYPE=HTML&GET_SEQUENCE=yes&I_THRESH=&MASK_CHAR=2&MASK_COLOR=1&NEW_DESIGN=on&NEW_VIEW=yes&NUM_OVERVIEW=100&OLD_BLAST=false&PAGE=Proteins&QUERY_INDEX=0&QUERY_NUMBER=0&RESULTS_PAGE_TARGET=&RID=T2PJYNMM01N&SHOW_LINKOUT=yes&SHOW_OVERVIEW=yes&STEP_NUMBER=&WORD_SIZE=2&DISPLAY_SORT=4&HSP_SORT=0" \l "sort_mark)** | **[E value](http://blast.ncbi.nlm.nih.gov/Blast.cgi?CMD=Get&ALIGNMENTS=100&ALIGNMENT_VIEW=Pairwise&CDD_SEARCH_STATE=1&DATABASE_SORT=0&DESCRIPTIONS=100&ENTREZ_QUERY=txid9606 %5BORGN%5D&FIRST_QUERY_NUM=0&FORMAT_OBJECT=Alignment&FORMAT_PAGE_TARGET=&FORMAT_TYPE=HTML&GET_SEQUENCE=yes&I_THRESH=&MASK_CHAR=2&MASK_COLOR=1&NEW_DESIGN=on&NEW_VIEW=yes&NUM_OVERVIEW=100&OLD_BLAST=false&PAGE=Proteins&QUERY_INDEX=0&QUERY_NUMBER=0&RESULTS_PAGE_TARGET=&RID=T2PJYNMM01N&SHOW_LINKOUT=yes&SHOW_OVERVIEW=yes&STEP_NUMBER=&WORD_SIZE=2&DISPLAY_SORT=0&HSP_SORT=0" \l "sort_mark)** |
| --- | --- | --- | --- | --- | --- |
| [NP_689909.2](http://www.ncbi.nlm.nih.gov/entrez/query.fcgi?cmd=Retrieve&db=Protein&list_uids=38201638&dopt=GenPept&RID=T2PJYNMM01N&log$=prottop&blast_rank=1) | homeodomain-interacting protein kinase 1 isoform 2 [Homo sapiens] | [22.7](http://blast.ncbi.nlm.nih.gov/Blast.cgi#38201638%2338201638) | 22.7 | 88% | 23 |
| [NP_852003.1](http://www.ncbi.nlm.nih.gov/entrez/query.fcgi?cmd=Retrieve&db=Protein&list_uids=38201644&dopt=GenPept&RID=T2PJYNMM01N&log$=prottop&blast_rank=2) | homeodomain-interacting protein kinase 1 isoform 4 [Homo sapiens] | [22.7](http://blast.ncbi.nlm.nih.gov/Blast.cgi#38201644%2338201644) | 22.7 | 88% | 23 |
| [NP_060051.2](http://www.ncbi.nlm.nih.gov/entrez/query.fcgi?cmd=Retrieve&db=Protein&list_uids=70995130&dopt=GenPept&RID=T2PJYNMM01N&log$=prottop&blast_rank=3) | cholinergic receptor, nicotinic, alpha 9 precursor [Homo sapiens] | [22.7](http://blast.ncbi.nlm.nih.gov/Blast.cgi#70995130%2370995130) | 22.7 | 88% | 23 |
| [NP_938010.1](http://www.ncbi.nlm.nih.gov/entrez/query.fcgi?cmd=Retrieve&db=Protein&list_uids=38201642&dopt=GenPept&RID=T2PJYNMM01N&log$=prottop&blast_rank=4) | homeodomain-interacting protein kinase 1 isoform 3 [Homo sapiens] | [22.7](http://blast.ncbi.nlm.nih.gov/Blast.cgi#38201642%2338201642) | 22.7 | 88% | 23 |
| [NP_938009.1](http://www.ncbi.nlm.nih.gov/entrez/query.fcgi?cmd=Retrieve&db=Protein&list_uids=38201640&dopt=GenPept&RID=T2PJYNMM01N&log$=prottop&blast_rank=5) | homeodomain-interacting protein kinase 1 isoform 1 [Homo sapiens] | [22.7](http://blast.ncbi.nlm.nih.gov/Blast.cgi#38201640%2338201640) | 22.7 | 88% | 23 |
| [NP_008911.1](http://www.ncbi.nlm.nih.gov/entrez/query.fcgi?cmd=Retrieve&db=Protein&list_uids=5902010&dopt=GenPept&RID=T2PJYNMM01N&log$=prottop&blast_rank=6) | mitochondrial transcription termination factor precursor [Homo sapiens] | [22.3](http://blast.ncbi.nlm.nih.gov/Blast.cgi#5902010%235902010) | 22.3 | 77% | 31 |
| [NP_001005198.1](http://www.ncbi.nlm.nih.gov/entrez/query.fcgi?cmd=Retrieve&db=Protein&list_uids=52353354&dopt=GenPept&RID=T2PJYNMM01N&log$=prottop&blast_rank=7) | olfactory receptor, family 8, subfamily G, member 5 [Homo sapiens] | [21.8](http://blast.ncbi.nlm.nih.gov/Blast.cgi#52353354%2352353354) | 21.8 | 77% | 41 |
| [NP_001007250.1](http://www.ncbi.nlm.nih.gov/entrez/query.fcgi?cmd=Retrieve&db=Protein&list_uids=55925593&dopt=GenPept&RID=T2PJYNMM01N&log$=prottop&blast_rank=8) | olfactory receptor, family 8, subfamily G, member 2 [Homo sapiens] | [21.8](http://blast.ncbi.nlm.nih.gov/Blast.cgi#55925593%2355925593) | 21.8 | 77% | 41 |
| [NP_001002905.1](http://www.ncbi.nlm.nih.gov/entrez/query.fcgi?cmd=Retrieve&db=Protein&list_uids=50897298&dopt=GenPept&RID=T2PJYNMM01N&log$=prottop&blast_rank=9) | olfactory receptor, family 8, subfamily G, member 1 [Homo sapiens] | [21.8](http://blast.ncbi.nlm.nih.gov/Blast.cgi#50897298%2350897298) | 21.8 | 77% | 41 |
| [NP_060445.3](http://www.ncbi.nlm.nih.gov/entrez/query.fcgi?cmd=Retrieve&db=Protein&list_uids=110578655&dopt=GenPept&RID=T2PJYNMM01N&log$=prottop&blast_rank=10) | Zwilch [Homo sapiens] | [21.0](http://blast.ncbi.nlm.nih.gov/Blast.cgi#110578655%23110578655) | 21.0 | 88% | 74 |
| [NP_005405.2](http://www.ncbi.nlm.nih.gov/entrez/query.fcgi?cmd=Retrieve&db=Protein&list_uids=223029418&dopt=GenPept&RID=T2PJYNMM01N&log$=prottop&blast_rank=11) | SKI-like isoform 1 [Homo sapiens] | [20.6](http://blast.ncbi.nlm.nih.gov/Blast.cgi#223029418%23223029418) | 20.6 | 66% | 100 |
| [NP_001138570.1](http://www.ncbi.nlm.nih.gov/entrez/query.fcgi?cmd=Retrieve&db=Protein&list_uids=223029422&dopt=GenPept&RID=T2PJYNMM01N&log$=prottop&blast_rank=12) | SKI-like isoform 3 [Homo sapiens] | [20.6](http://blast.ncbi.nlm.nih.gov/Blast.cgi#223029422%23223029422) | 20.6 | 66% | 100 |
| [NP_004561.3](http://www.ncbi.nlm.nih.gov/entrez/query.fcgi?cmd=Retrieve&db=Protein&list_uids=194353959&dopt=GenPept&RID=T2PJYNMM01N&log$=prottop&blast_rank=13) | phosphoinositide-3-kinase, class 2 gamma polypeptide [Homo sapiens] | [20.6](http://blast.ncbi.nlm.nih.gov/Blast.cgi#194353959%23194353959) | 20.6 | 77% | 100 |
| [NP_004954.2](http://www.ncbi.nlm.nih.gov/entrez/query.fcgi?cmd=Retrieve&db=Protein&list_uids=222080083&dopt=GenPept&RID=T2PJYNMM01N&log$=prottop&blast_rank=14) | guanylate cyclase 2C precursor [Homo sapiens] | [20.2](http://blast.ncbi.nlm.nih.gov/Blast.cgi#222080083%23222080083) | 35.6 | 88% | 134 |
| [NP_001002907.1](http://www.ncbi.nlm.nih.gov/entrez/query.fcgi?cmd=Retrieve&db=Protein&list_uids=50897270&dopt=GenPept&RID=T2PJYNMM01N&log$=prottop&blast_rank=15) | olfactory receptor, family 8, subfamily K, member 1 [Homo sapiens] | [20.2](http://blast.ncbi.nlm.nih.gov/Blast.cgi#50897270%2350897270) | 20.2 | 66% | 134 |
| [NP_001138600.2](http://www.ncbi.nlm.nih.gov/entrez/query.fcgi?cmd=Retrieve&db=Protein&list_uids=237858799&dopt=GenPept&RID=T2PJYNMM01N&log$=prottop&blast_rank=16) | adenylate kinase domain containing 1 isoform 1 [Homo sapiens] | [19.7](http://blast.ncbi.nlm.nih.gov/Blast.cgi#237858799%23237858799) | 19.7 | 55% | 180 |
| [NP_001165911.1](http://www.ncbi.nlm.nih.gov/entrez/query.fcgi?cmd=Retrieve&db=Protein&list_uids=289063435&dopt=GenPept&RID=T2PJYNMM01N&log$=prottop&blast_rank=17) | endonuclease, polyU-specific isoform 3 precursor [Homo sapiens] | [19.7](http://blast.ncbi.nlm.nih.gov/Blast.cgi#289063435%23289063435) | 19.7 | 55% | 180 |
| [NP_001138815.1](http://www.ncbi.nlm.nih.gov/entrez/query.fcgi?cmd=Retrieve&db=Protein&list_uids=223890210&dopt=GenPept&RID=T2PJYNMM01N&log$=prottop&blast_rank=18) | zinc finger protein 566 isoform 2 [Homo sapiens] | [19.7](http://blast.ncbi.nlm.nih.gov/Blast.cgi#223890210%23223890210) | 19.7 | 55% | 180 |
| [NP_001009877.2](http://www.ncbi.nlm.nih.gov/entrez/query.fcgi?cmd=Retrieve&db=Protein&list_uids=242247075&dopt=GenPept&RID=T2PJYNMM01N&log$=prottop&blast_rank=19) | bromodomain containing 9 isoform 2 [Homo sapiens] | [19.7](http://blast.ncbi.nlm.nih.gov/Blast.cgi#242247075%23242247075) | 19.7 | 55% | 180 |
| [NP_076413.3](http://www.ncbi.nlm.nih.gov/entrez/query.fcgi?cmd=Retrieve&db=Protein&list_uids=237649104&dopt=GenPept&RID=T2PJYNMM01N&log$=prottop&blast_rank=20) | bromodomain containing 9 isoform 1 [Homo sapiens] | [19.7](http://blast.ncbi.nlm.nih.gov/Blast.cgi#237649104%23237649104) | 19.7 | 55% | 180 |
| [NP_079461.2](http://www.ncbi.nlm.nih.gov/entrez/query.fcgi?cmd=Retrieve&db=Protein&list_uids=157739945&dopt=GenPept&RID=T2PJYNMM01N&log$=prottop&blast_rank=21) | tetratricopeptide repeat, ankyrin repeat and coiled-coil containing 2 [Homo sapiens] | [19.7](http://blast.ncbi.nlm.nih.gov/Blast.cgi#157739945%23157739945) | 32.2 | 100% | 180 |
| [NP_001165910.1](http://www.ncbi.nlm.nih.gov/entrez/query.fcgi?cmd=Retrieve&db=Protein&list_uids=289063433&dopt=GenPept&RID=T2PJYNMM01N&log$=prottop&blast_rank=22) | endonuclease, polyU-specific isoform 1 precursor [Homo sapiens] | [19.7](http://blast.ncbi.nlm.nih.gov/Blast.cgi#289063433%23289063433) | 19.7 | 55% | 180 |
| [NP_001073286.1](http://www.ncbi.nlm.nih.gov/entrez/query.fcgi?cmd=Retrieve&db=Protein&list_uids=119395740&dopt=GenPept&RID=T2PJYNMM01N&log$=prottop&blast_rank=23) | integrin alpha chain, alpha 6 isoform a precursor [Homo sapiens] | [19.7](http://blast.ncbi.nlm.nih.gov/Blast.cgi#119395740%23119395740) | 19.7 | 55% | 180 |
| [NP_003980.2](http://www.ncbi.nlm.nih.gov/entrez/query.fcgi?cmd=Retrieve&db=Protein&list_uids=34878716&dopt=GenPept&RID=T2PJYNMM01N&log$=prottop&blast_rank=24) | paired box protein 2 isoform d [Homo sapiens] | [19.7](http://blast.ncbi.nlm.nih.gov/Blast.cgi#34878716%2334878716) | 19.7 | 55% | 180 |
| [NP_000269.2](http://www.ncbi.nlm.nih.gov/entrez/query.fcgi?cmd=Retrieve&db=Protein&list_uids=34878701&dopt=GenPept&RID=T2PJYNMM01N&log$=prottop&blast_rank=25) | paired box protein 2 isoform b [Homo sapiens] | [19.7](http://blast.ncbi.nlm.nih.gov/Blast.cgi#34878701%2334878701) | 19.7 | 55% | 180 |
| [NP_003981.2](http://www.ncbi.nlm.nih.gov/entrez/query.fcgi?cmd=Retrieve&db=Protein&list_uids=34878703&dopt=GenPept&RID=T2PJYNMM01N&log$=prottop&blast_rank=26) | paired box protein 2 isoform e [Homo sapiens] | [19.7](http://blast.ncbi.nlm.nih.gov/Blast.cgi#34878703%2334878703) | 19.7 | 55% | 180 |
| [NP_003978.2](http://www.ncbi.nlm.nih.gov/entrez/query.fcgi?cmd=Retrieve&db=Protein&list_uids=34878699&dopt=GenPept&RID=T2PJYNMM01N&log$=prottop&blast_rank=27) | paired box protein 2 isoform a [Homo sapiens] | [19.7](http://blast.ncbi.nlm.nih.gov/Blast.cgi#34878699%2334878699) | 19.7 | 55% | 180 |
| [NP_003979.2](http://www.ncbi.nlm.nih.gov/entrez/query.fcgi?cmd=Retrieve&db=Protein&list_uids=34878709&dopt=GenPept&RID=T2PJYNMM01N&log$=prottop&blast_rank=28) | paired box protein 2 isoform c [Homo sapiens] | [19.7](http://blast.ncbi.nlm.nih.gov/Blast.cgi#34878709%2334878709) | 19.7 | 55% | 180 |
| [NP_689578.2](http://www.ncbi.nlm.nih.gov/entrez/query.fcgi?cmd=Retrieve&db=Protein&list_uids=282399147&dopt=GenPept&RID=T2PJYNMM01N&log$=prottop&blast_rank=29) | hypothetical protein LOC126695 [Homo sapiens] | [19.7](http://blast.ncbi.nlm.nih.gov/Blast.cgi#282399147%23282399147) | 19.7 | 55% | 180 |
| [NP_001073996.1](http://www.ncbi.nlm.nih.gov/entrez/query.fcgi?cmd=Retrieve&db=Protein&list_uids=122937512&dopt=GenPept&RID=T2PJYNMM01N&log$=prottop&blast_rank=30) | myosin VIIB [Homo sapiens] | [19.7](http://blast.ncbi.nlm.nih.gov/Blast.cgi#122937512%23122937512) | 19.7 | 55% | 180 |
| [NP_000201.2](http://www.ncbi.nlm.nih.gov/entrez/query.fcgi?cmd=Retrieve&db=Protein&list_uids=119395742&dopt=GenPept&RID=T2PJYNMM01N&log$=prottop&blast_rank=31) | integrin alpha chain, alpha 6 isoform b precursor [Homo sapiens] | [19.7](http://blast.ncbi.nlm.nih.gov/Blast.cgi#119395742%23119395742) | 19.7 | 55% | 180 |
| [NP_116227.1](http://www.ncbi.nlm.nih.gov/entrez/query.fcgi?cmd=Retrieve&db=Protein&list_uids=14523054&dopt=GenPept&RID=T2PJYNMM01N&log$=prottop&blast_rank=32) | zinc finger protein 566 isoform 1 [Homo sapiens] >ref|NP_001138816.1| zinc finger protein 566 isoform 1 [Homo sapiens] >ref|NP_001138817.1| zinc finger protein 566 isoform 1 [Homo sapiens] | [19.7](http://blast.ncbi.nlm.nih.gov/Blast.cgi#14523054%2314523054) | 19.7 | 55% | 180 |
| [NP_000614.1](http://www.ncbi.nlm.nih.gov/entrez/query.fcgi?cmd=Retrieve&db=Protein&list_uids=4557359&dopt=GenPept&RID=T2PJYNMM01N&log$=prottop&blast_rank=33) | bradykinin receptor B2 [Homo sapiens] | [19.7](http://blast.ncbi.nlm.nih.gov/Blast.cgi#4557359%234557359) | 19.7 | 55% | 180 |
| [NP_009190.2](http://www.ncbi.nlm.nih.gov/entrez/query.fcgi?cmd=Retrieve&db=Protein&list_uids=18105063&dopt=GenPept&RID=T2PJYNMM01N&log$=prottop&blast_rank=34) | vacuolar protein sorting 45A [Homo sapiens] | [19.7](http://blast.ncbi.nlm.nih.gov/Blast.cgi#18105063%2318105063) | 19.7 | 100% | 180 |
| [NP_066983.1](http://www.ncbi.nlm.nih.gov/entrez/query.fcgi?cmd=Retrieve&db=Protein&list_uids=15100151&dopt=GenPept&RID=T2PJYNMM01N&log$=prottop&blast_rank=35) | HLA-B associated transcript 5 [Homo sapiens] | [19.7](http://blast.ncbi.nlm.nih.gov/Blast.cgi#15100151%2315100151) | 19.7 | 55% | 180 |
| [NP_005756.2](http://www.ncbi.nlm.nih.gov/entrez/query.fcgi?cmd=Retrieve&db=Protein&list_uids=15011918&dopt=GenPept&RID=T2PJYNMM01N&log$=prottop&blast_rank=36) | ATPase, H+ transporting, lysosomal accessory protein 2 [Homo sapiens] | [19.7](http://blast.ncbi.nlm.nih.gov/Blast.cgi#15011918%2315011918) | 19.7 | 55% | 180 |
| [NP_006016.1](http://www.ncbi.nlm.nih.gov/entrez/query.fcgi?cmd=Retrieve&db=Protein&list_uids=5174623&dopt=GenPept&RID=T2PJYNMM01N&log$=prottop&blast_rank=37) | endonuclease, polyU-specific isoform 2 precursor [Homo sapiens] | [19.7](http://blast.ncbi.nlm.nih.gov/Blast.cgi#5174623%235174623) | 19.7 | 55% | 180 |
| [NP_002509.2](http://www.ncbi.nlm.nih.gov/entrez/query.fcgi?cmd=Retrieve&db=Protein&list_uids=22027471&dopt=GenPept&RID=T2PJYNMM01N&log$=prottop&blast_rank=38) | neuronal PAS domain protein 2 [Homo sapiens] | [19.3](http://blast.ncbi.nlm.nih.gov/Blast.cgi#22027471%2322027471) | 19.3 | 77% | 241 |
| [NP_075052.1](http://www.ncbi.nlm.nih.gov/entrez/query.fcgi?cmd=Retrieve&db=Protein&list_uids=12597641&dopt=GenPept&RID=T2PJYNMM01N&log$=prottop&blast_rank=39) | leucine rich repeat containing 19 precursor [Homo sapiens] | [19.3](http://blast.ncbi.nlm.nih.gov/Blast.cgi#12597641%2312597641) | 19.3 | 88% | 241 |
| [NP_563578.1](http://www.ncbi.nlm.nih.gov/entrez/query.fcgi?cmd=Retrieve&db=Protein&list_uids=18641362&dopt=GenPept&RID=T2PJYNMM01N&log$=prottop&blast_rank=40) | protein tyrosine phosphatase, receptor type, C isoform 2 precursor [Homo sapiens] | [18.9](http://blast.ncbi.nlm.nih.gov/Blast.cgi#18641362%2318641362) | 18.9 | 66% | 323 |
| [NP_689920.1](http://www.ncbi.nlm.nih.gov/entrez/query.fcgi?cmd=Retrieve&db=Protein&list_uids=27544933&dopt=GenPept&RID=T2PJYNMM01N&log$=prottop&blast_rank=41) | solute carrier family 25, member 16 [Homo sapiens] | [18.9](http://blast.ncbi.nlm.nih.gov/Blast.cgi#27544933%2327544933) | 18.9 | 66% | 323 |
| [NP_563579.1](http://www.ncbi.nlm.nih.gov/entrez/query.fcgi?cmd=Retrieve&db=Protein&list_uids=18641364&dopt=GenPept&RID=T2PJYNMM01N&log$=prottop&blast_rank=42) | protein tyrosine phosphatase, receptor type, C isoform 3 precursor [Homo sapiens] | [18.9](http://blast.ncbi.nlm.nih.gov/Blast.cgi#18641364%2318641364) | 33.1 | 66% | 323 |
| [NP_443193.1](http://www.ncbi.nlm.nih.gov/entrez/query.fcgi?cmd=Retrieve&db=Protein&list_uids=16418457&dopt=GenPept&RID=T2PJYNMM01N&log$=prottop&blast_rank=43) | solute carrier family 26, member 8 isoform a [Homo sapiens] | [18.9](http://blast.ncbi.nlm.nih.gov/Blast.cgi#16418457%2316418457) | 18.9 | 66% | 323 |
| [NP_619732.1](http://www.ncbi.nlm.nih.gov/entrez/query.fcgi?cmd=Retrieve&db=Protein&list_uids=20336285&dopt=GenPept&RID=T2PJYNMM01N&log$=prottop&blast_rank=44) | solute carrier family 26, member 8 isoform b [Homo sapiens] | [18.9](http://blast.ncbi.nlm.nih.gov/Blast.cgi#20336285%2320336285) | 18.9 | 66% | 323 |
| [NP_002829.2](http://www.ncbi.nlm.nih.gov/entrez/query.fcgi?cmd=Retrieve&db=Protein&list_uids=18641347&dopt=GenPept&RID=T2PJYNMM01N&log$=prottop&blast_rank=45) | protein tyrosine phosphatase, receptor type, C isoform 1 precursor [Homo sapiens] | [18.9](http://blast.ncbi.nlm.nih.gov/Blast.cgi#18641347%2318641347) | 34.8 | 100% | 323 |
| [NP_001137293.1](http://www.ncbi.nlm.nih.gov/entrez/query.fcgi?cmd=Retrieve&db=Protein&list_uids=219689123&dopt=GenPept&RID=T2PJYNMM01N&log$=prottop&blast_rank=46) | pleckstrin homology domain containing, family A member 5 isoform 2 [Homo sapiens] | [18.5](http://blast.ncbi.nlm.nih.gov/Blast.cgi#219689123%23219689123) | 34.8 | 77% | 434 |
| [NP_001009880.1](http://www.ncbi.nlm.nih.gov/entrez/query.fcgi?cmd=Retrieve&db=Protein&list_uids=57863295&dopt=GenPept&RID=T2PJYNMM01N&log$=prottop&blast_rank=47) | hypothetical protein LOC23313 isoform b [Homo sapiens] | [18.5](http://blast.ncbi.nlm.nih.gov/Blast.cgi#57863295%2357863295) | 18.5 | 66% | 434 |
| [NP_056079.1](http://www.ncbi.nlm.nih.gov/entrez/query.fcgi?cmd=Retrieve&db=Protein&list_uids=57863293&dopt=GenPept&RID=T2PJYNMM01N&log$=prottop&blast_rank=48) | hypothetical protein LOC23313 isoform a [Homo sapiens] | [18.5](http://blast.ncbi.nlm.nih.gov/Blast.cgi#57863293%2357863293) | 18.5 | 66% | 434 |
| [NP_296373.1](http://www.ncbi.nlm.nih.gov/entrez/query.fcgi?cmd=Retrieve&db=Protein&list_uids=16306502&dopt=GenPept&RID=T2PJYNMM01N&log$=prottop&blast_rank=49) | F-box only protein 21 isoform 1 [Homo sapiens] | [18.5](http://blast.ncbi.nlm.nih.gov/Blast.cgi#16306502%2316306502) | 18.5 | 88% | 434 |
| [NP_076985.4](http://www.ncbi.nlm.nih.gov/entrez/query.fcgi?cmd=Retrieve&db=Protein&list_uids=109689695&dopt=GenPept&RID=T2PJYNMM01N&log$=prottop&blast_rank=50) | transient receptor potential cation channel, subfamily M, member 8 [Homo sapiens] | [18.5](http://blast.ncbi.nlm.nih.gov/Blast.cgi#109689695%23109689695) | 18.5 | 66% | 434 |
| [NP_061885.2](http://www.ncbi.nlm.nih.gov/entrez/query.fcgi?cmd=Retrieve&db=Protein&list_uids=19923493&dopt=GenPept&RID=T2PJYNMM01N&log$=prottop&blast_rank=51) | pleckstrin homology domain containing, family A member 5 isoform 1 [Homo sapiens] | [18.5](http://blast.ncbi.nlm.nih.gov/Blast.cgi#19923493%2319923493) | 34.8 | 77% | 434 |
| [NP_005908.1](http://www.ncbi.nlm.nih.gov/entrez/query.fcgi?cmd=Retrieve&db=Protein&list_uids=5174539&dopt=GenPept&RID=T2PJYNMM01N&log$=prottop&blast_rank=52) | cytosolic malate dehydrogenase [Homo sapiens] | [18.5](http://blast.ncbi.nlm.nih.gov/Blast.cgi#5174539%235174539) | 18.5 | 88% | 434 |
| [NP_055817.1](http://www.ncbi.nlm.nih.gov/entrez/query.fcgi?cmd=Retrieve&db=Protein&list_uids=16306500&dopt=GenPept&RID=T2PJYNMM01N&log$=prottop&blast_rank=53) | F-box only protein 21 isoform 2 [Homo sapiens] | [18.5](http://blast.ncbi.nlm.nih.gov/Blast.cgi#16306500%2316306500) | 18.5 | 88% | 434 |
| [NP_001138295.1](http://www.ncbi.nlm.nih.gov/entrez/query.fcgi?cmd=Retrieve&db=Protein&list_uids=221307568&dopt=GenPept&RID=T2PJYNMM01N&log$=prottop&blast_rank=54) | DENN/MADD domain containing 4A isoform 1 [Homo sapiens] | [18.0](http://blast.ncbi.nlm.nih.gov/Blast.cgi#221307568%23221307568) | 18.0 | 55% | 582 |
| [NP_055640.2](http://www.ncbi.nlm.nih.gov/entrez/query.fcgi?cmd=Retrieve&db=Protein&list_uids=194394141&dopt=GenPept&RID=T2PJYNMM01N&log$=prottop&blast_rank=55) | URB1 ribosome biogenesis 1 homolog [Homo sapiens] | [18.0](http://blast.ncbi.nlm.nih.gov/Blast.cgi#194394141%23194394141) | 28.0 | 77% | 582 |
| [NP_001129036.1](http://www.ncbi.nlm.nih.gov/entrez/query.fcgi?cmd=Retrieve&db=Protein&list_uids=207113147&dopt=GenPept&RID=T2PJYNMM01N&log$=prottop&blast_rank=56) | heat shock transcription factor 2 isoform b [Homo sapiens] | [18.0](http://blast.ncbi.nlm.nih.gov/Blast.cgi#207113147%23207113147) | 18.0 | 88% | 582 |
| [NP_001004488.1](http://www.ncbi.nlm.nih.gov/entrez/query.fcgi?cmd=Retrieve&db=Protein&list_uids=52317188&dopt=GenPept&RID=T2PJYNMM01N&log$=prottop&blast_rank=57) | olfactory receptor, family 2, subfamily A, member 25 [Homo sapiens] | [18.0](http://blast.ncbi.nlm.nih.gov/Blast.cgi#52317188%2352317188) | 18.0 | 66% | 582 |
| [NP_000755.2](http://www.ncbi.nlm.nih.gov/entrez/query.fcgi?cmd=Retrieve&db=Protein&list_uids=15147330&dopt=GenPept&RID=T2PJYNMM01N&log$=prottop&blast_rank=58) | cytochrome P450, family 2, subfamily A, polypeptide 7 isoform 1 [Homo sapiens] | [18.0](http://blast.ncbi.nlm.nih.gov/Blast.cgi#15147330%2315147330) | 18.0 | 55% | 582 |
| [NP_005115.2](http://www.ncbi.nlm.nih.gov/entrez/query.fcgi?cmd=Retrieve&db=Protein&list_uids=24430146&dopt=GenPept&RID=T2PJYNMM01N&log$=prottop&blast_rank=59) | nucleoporin 153kDa [Homo sapiens] | [18.0](http://blast.ncbi.nlm.nih.gov/Blast.cgi#24430146%2324430146) | 18.0 | 100% | 582 |
| [NP_659453.3](http://www.ncbi.nlm.nih.gov/entrez/query.fcgi?cmd=Retrieve&db=Protein&list_uids=150010619&dopt=GenPept&RID=T2PJYNMM01N&log$=prottop&blast_rank=60) | glycine-N-acyltransferase-like 2 [Homo sapiens] | [18.0](http://blast.ncbi.nlm.nih.gov/Blast.cgi#150010619%23150010619) | 18.0 | 55% | 582 |
| [NP_085079.2](http://www.ncbi.nlm.nih.gov/entrez/query.fcgi?cmd=Retrieve&db=Protein&list_uids=15147328&dopt=GenPept&RID=T2PJYNMM01N&log$=prottop&blast_rank=61) | cytochrome P450, family 2, subfamily A, polypeptide 7 isoform 2 [Homo sapiens] | [18.0](http://blast.ncbi.nlm.nih.gov/Blast.cgi#15147328%2315147328) | 18.0 | 55% | 582 |
| [NP_000753.3](http://www.ncbi.nlm.nih.gov/entrez/query.fcgi?cmd=Retrieve&db=Protein&list_uids=189339233&dopt=GenPept&RID=T2PJYNMM01N&log$=prottop&blast_rank=62) | cytochrome P450, family 2, subfamily A, polypeptide 6 [Homo sapiens] | [18.0](http://blast.ncbi.nlm.nih.gov/Blast.cgi#189339233%23189339233) | 18.0 | 55% | 582 |
| [NP_004497.1](http://www.ncbi.nlm.nih.gov/entrez/query.fcgi?cmd=Retrieve&db=Protein&list_uids=4758568&dopt=GenPept&RID=T2PJYNMM01N&log$=prottop&blast_rank=63) | heat shock transcription factor 2 isoform a [Homo sapiens] | [18.0](http://blast.ncbi.nlm.nih.gov/Blast.cgi#4758568%234758568) | 18.0 | 88% | 582 |
| [NP_001004456.1](http://www.ncbi.nlm.nih.gov/entrez/query.fcgi?cmd=Retrieve&db=Protein&list_uids=52218828&dopt=GenPept&RID=T2PJYNMM01N&log$=prottop&blast_rank=64) | olfactory receptor, family 1, subfamily M, member 1 [Homo sapiens] | [18.0](http://blast.ncbi.nlm.nih.gov/Blast.cgi#52218828%2352218828) | 18.0 | 77% | 582 |
| [NP_005839.3](http://www.ncbi.nlm.nih.gov/entrez/query.fcgi?cmd=Retrieve&db=Protein&list_uids=221307566&dopt=GenPept&RID=T2PJYNMM01N&log$=prottop&blast_rank=65) | DENN/MADD domain containing 4A isoform 2 [Homo sapiens] | [18.0](http://blast.ncbi.nlm.nih.gov/Blast.cgi#221307566%23221307566) | 18.0 | 55% | 582 |
| [NP_000757.2](http://www.ncbi.nlm.nih.gov/entrez/query.fcgi?cmd=Retrieve&db=Protein&list_uids=13699809&dopt=GenPept&RID=T2PJYNMM01N&log$=prottop&blast_rank=66) | cytochrome P450, family 2, subfamily A, polypeptide 13 [Homo sapiens] | [18.0](http://blast.ncbi.nlm.nih.gov/Blast.cgi#13699809%2313699809) | 18.0 | 55% | 582 |
| [NP_055312.2](http://www.ncbi.nlm.nih.gov/entrez/query.fcgi?cmd=Retrieve&db=Protein&list_uids=21626468&dopt=GenPept&RID=T2PJYNMM01N&log$=prottop&blast_rank=67) | zinc finger protein 638 [Homo sapiens] >ref|NP_001014972.1| zinc finger protein 638 [Homo sapiens] | [18.0](http://blast.ncbi.nlm.nih.gov/Blast.cgi#21626468%2321626468) | 18.0 | 66% | 582 |
| [NP_071899.1](http://www.ncbi.nlm.nih.gov/entrez/query.fcgi?cmd=Retrieve&db=Protein&list_uids=11967991&dopt=GenPept&RID=T2PJYNMM01N&log$=prottop&blast_rank=68) | SRY-box 17 [Homo sapiens] | [18.0](http://blast.ncbi.nlm.nih.gov/Blast.cgi#11967991%2311967991) | 18.0 | 100% | 582 |
| [NP_000349.1](http://www.ncbi.nlm.nih.gov/entrez/query.fcgi?cmd=Retrieve&db=Protein&list_uids=4507467&dopt=GenPept&RID=T2PJYNMM01N&log$=prottop&blast_rank=69) | transforming growth factor, beta-induced, 68kDa precursor [Homo sapiens] | [18.0](http://blast.ncbi.nlm.nih.gov/Blast.cgi#4507467%234507467) | 18.0 | 66% | 582 |
| [NP_005430.1](http://www.ncbi.nlm.nih.gov/entrez/query.fcgi?cmd=Retrieve&db=Protein&list_uids=4885487&dopt=GenPept&RID=T2PJYNMM01N&log$=prottop&blast_rank=70) | myeloid leukemia factor 2 [Homo sapiens] | [18.0](http://blast.ncbi.nlm.nih.gov/Blast.cgi#4885487%234885487) | 18.0 | 66% | 582 |
| [NP_001004058.2](http://www.ncbi.nlm.nih.gov/entrez/query.fcgi?cmd=Retrieve&db=Protein&list_uids=145279179&dopt=GenPept&RID=T2PJYNMM01N&log$=prottop&blast_rank=71) | olfactory receptor, family 8, subfamily K, member 5 [Homo sapiens] | [18.0](http://blast.ncbi.nlm.nih.gov/Blast.cgi#145279179%23145279179) | 18.0 | 55% | 582 |
| [NP_057664.1](http://www.ncbi.nlm.nih.gov/entrez/query.fcgi?cmd=Retrieve&db=Protein&list_uids=7706113&dopt=GenPept&RID=T2PJYNMM01N&log$=prottop&blast_rank=72) | protocadherin 12 precursor [Homo sapiens] | [18.0](http://blast.ncbi.nlm.nih.gov/Blast.cgi#7706113%237706113) | 28.0 | 77% | 582 |
| [NP_071504.2](http://www.ncbi.nlm.nih.gov/entrez/query.fcgi?cmd=Retrieve&db=Protein&list_uids=106049292&dopt=GenPept&RID=T2PJYNMM01N&log$=prottop&blast_rank=73) | pyruvate carboxylase precursor [Homo sapiens] >ref|NP_000911.2| pyruvate carboxylase precursor [Homo sapiens] >ref|NP_001035806.1| pyruvate carboxylase precursor [Homo sapiens] | [18.0](http://blast.ncbi.nlm.nih.gov/Blast.cgi#106049292%23106049292) | 18.0 | 88% | 582 |
| [NP_005159.1](http://www.ncbi.nlm.nih.gov/entrez/query.fcgi?cmd=Retrieve&db=Protein&list_uids=4885069&dopt=GenPept&RID=T2PJYNMM01N&log$=prottop&blast_rank=74) | ras homolog gene family, member E precursor [Homo sapiens] | [18.0](http://blast.ncbi.nlm.nih.gov/Blast.cgi#4885069%234885069) | 18.0 | 77% | 582 |
| [NP_001136022.1](http://www.ncbi.nlm.nih.gov/entrez/query.fcgi?cmd=Retrieve&db=Protein&list_uids=216548378&dopt=GenPept&RID=T2PJYNMM01N&log$=prottop&blast_rank=75) | WD repeat domain 47 isoform 1 [Homo sapiens] | [17.6](http://blast.ncbi.nlm.nih.gov/Blast.cgi#216548378%23216548378) | 17.6 | 77% | 781 |
| [NP_001129217.1](http://www.ncbi.nlm.nih.gov/entrez/query.fcgi?cmd=Retrieve&db=Protein&list_uids=209364523&dopt=GenPept&RID=T2PJYNMM01N&log$=prottop&blast_rank=76) | alsin isoform 2 [Homo sapiens] | [17.6](http://blast.ncbi.nlm.nih.gov/Blast.cgi#209364523%23209364523) | 17.6 | 77% | 781 |
| [XP_001724537.2](http://www.ncbi.nlm.nih.gov/entrez/query.fcgi?cmd=Retrieve&db=Protein&list_uids=239744057&dopt=GenPept&RID=T2PJYNMM01N&log$=prottop&blast_rank=77) | PREDICTED: PRO1102 [Homo sapiens] | [17.6](http://blast.ncbi.nlm.nih.gov/Blast.cgi#239744057%23239744057) | 17.6 | 55% | 781 |
| [NP_057344.2](http://www.ncbi.nlm.nih.gov/entrez/query.fcgi?cmd=Retrieve&db=Protein&list_uids=119220596&dopt=GenPept&RID=T2PJYNMM01N&log$=prottop&blast_rank=78) | helios isoform 1 [Homo sapiens] | [17.6](http://blast.ncbi.nlm.nih.gov/Blast.cgi#119220596%23119220596) | 17.6 | 55% | 781 |
| [NP_003808.2](http://www.ncbi.nlm.nih.gov/entrez/query.fcgi?cmd=Retrieve&db=Protein&list_uids=114326453&dopt=GenPept&RID=T2PJYNMM01N&log$=prottop&blast_rank=79) | a disintegrin and metalloproteinase domain 7 precursor [Homo sapiens] | [17.6](http://blast.ncbi.nlm.nih.gov/Blast.cgi#114326453%23114326453) | 17.6 | 77% | 781 |
| [NP_055935.4](http://www.ncbi.nlm.nih.gov/entrez/query.fcgi?cmd=Retrieve&db=Protein&list_uids=110349786&dopt=GenPept&RID=T2PJYNMM01N&log$=prottop&blast_rank=80) | Alstrom syndrome 1 [Homo sapiens] | [17.6](http://blast.ncbi.nlm.nih.gov/Blast.cgi#110349786%23110349786) | 38.8 | 77% | 781 |
| [NP_055784.3](http://www.ncbi.nlm.nih.gov/entrez/query.fcgi?cmd=Retrieve&db=Protein&list_uids=216548347&dopt=GenPept&RID=T2PJYNMM01N&log$=prottop&blast_rank=81) | WD repeat domain 47 isoform 2 [Homo sapiens] | [17.6](http://blast.ncbi.nlm.nih.gov/Blast.cgi#216548347%23216548347) | 17.6 | 77% | 781 |
| [NP_001071062.1](http://www.ncbi.nlm.nih.gov/entrez/query.fcgi?cmd=Retrieve&db=Protein&list_uids=117938266&dopt=GenPept&RID=T2PJYNMM01N&log$=prottop&blast_rank=82) | hypothetical protein LOC91828 [Homo sapiens] | [17.6](http://blast.ncbi.nlm.nih.gov/Blast.cgi#117938266%23117938266) | 28.8 | 88% | 781 |
| [NP_001010892.1](http://www.ncbi.nlm.nih.gov/entrez/query.fcgi?cmd=Retrieve&db=Protein&list_uids=58219006&dopt=GenPept&RID=T2PJYNMM01N&log$=prottop&blast_rank=83) | radial spoke head 4 homolog A isoform 1 [Homo sapiens] | [17.6](http://blast.ncbi.nlm.nih.gov/Blast.cgi#58219006%2358219006) | 17.6 | 55% | 781 |
| [NP_001726.2](http://www.ncbi.nlm.nih.gov/entrez/query.fcgi?cmd=Retrieve&db=Protein&list_uids=38016947&dopt=GenPept&RID=T2PJYNMM01N&log$=prottop&blast_rank=84) | complement component 5 preproprotein [Homo sapiens] | [17.6](http://blast.ncbi.nlm.nih.gov/Blast.cgi#38016947%2338016947) | 34.4 | 66% | 781 |
| [NP_542416.1](http://www.ncbi.nlm.nih.gov/entrez/query.fcgi?cmd=Retrieve&db=Protein&list_uids=18375650&dopt=GenPept&RID=T2PJYNMM01N&log$=prottop&blast_rank=85) | protein tyrosine phosphatase, non-receptor type 13 isoform 4 [Homo sapiens] | [17.6](http://blast.ncbi.nlm.nih.gov/Blast.cgi#18375650%2318375650) | 17.6 | 66% | 781 |
| [NP_001072994.1](http://www.ncbi.nlm.nih.gov/entrez/query.fcgi?cmd=Retrieve&db=Protein&list_uids=119220592&dopt=GenPept&RID=T2PJYNMM01N&log$=prottop&blast_rank=86) | helios isoform 2 [Homo sapiens] | [17.6](http://blast.ncbi.nlm.nih.gov/Blast.cgi#119220592%23119220592) | 17.6 | 55% | 781 |
| [NP_001155136.1](http://www.ncbi.nlm.nih.gov/entrez/query.fcgi?cmd=Retrieve&db=Protein&list_uids=239582735&dopt=GenPept&RID=T2PJYNMM01N&log$=prottop&blast_rank=87) | radial spoke head 4 homolog A isoform 2 [Homo sapiens] | [17.6](http://blast.ncbi.nlm.nih.gov/Blast.cgi#239582735%23239582735) | 17.6 | 55% | 781 |
| [NP_001001925.1](http://www.ncbi.nlm.nih.gov/entrez/query.fcgi?cmd=Retrieve&db=Protein&list_uids=50348617&dopt=GenPept&RID=T2PJYNMM01N&log$=prottop&blast_rank=88) | mitochondrial tumor suppressor 1 isoform 2 [Homo sapiens] | [17.6](http://blast.ncbi.nlm.nih.gov/Blast.cgi#50348617%2350348617) | 29.7 | 66% | 781 |
| [NP_001001924.1](http://www.ncbi.nlm.nih.gov/entrez/query.fcgi?cmd=Retrieve&db=Protein&list_uids=50348611&dopt=GenPept&RID=T2PJYNMM01N&log$=prottop&blast_rank=89) | mitochondrial tumor suppressor 1 isoform 1 [Homo sapiens] | [17.6](http://blast.ncbi.nlm.nih.gov/Blast.cgi#50348611%2350348611) | 29.7 | 66% | 781 |
| [NP_061142.2](http://www.ncbi.nlm.nih.gov/entrez/query.fcgi?cmd=Retrieve&db=Protein&list_uids=27262624&dopt=GenPept&RID=T2PJYNMM01N&log$=prottop&blast_rank=90) | ATP-binding cassette, sub-family A , member 5 [Homo sapiens] >ref|NP_758424.1| ATP-binding cassette, sub-family A , member 5 [Homo sapiens] | [17.6](http://blast.ncbi.nlm.nih.gov/Blast.cgi#27262624%2327262624) | 17.6 | 66% | 781 |
| [NP_065970.2](http://www.ncbi.nlm.nih.gov/entrez/query.fcgi?cmd=Retrieve&db=Protein&list_uids=40316935&dopt=GenPept&RID=T2PJYNMM01N&log$=prottop&blast_rank=91) | alsin isoform 1 [Homo sapiens] | [17.6](http://blast.ncbi.nlm.nih.gov/Blast.cgi#40316935%2340316935) | 17.6 | 77% | 781 |
| [NP_001003841.1](http://www.ncbi.nlm.nih.gov/entrez/query.fcgi?cmd=Retrieve&db=Protein&list_uids=51468073&dopt=GenPept&RID=T2PJYNMM01N&log$=prottop&blast_rank=92) | solute carrier family 6, member 19 [Homo sapiens] | [17.6](http://blast.ncbi.nlm.nih.gov/Blast.cgi#51468073%2351468073) | 17.6 | 55% | 781 |
| [NP_008841.2](http://www.ncbi.nlm.nih.gov/entrez/query.fcgi?cmd=Retrieve&db=Protein&list_uids=33620769&dopt=GenPept&RID=T2PJYNMM01N&log$=prottop&blast_rank=93) | retinoblastoma-binding protein 6 isoform 1 [Homo sapiens] | [17.6](http://blast.ncbi.nlm.nih.gov/Blast.cgi#33620769%2333620769) | 17.6 | 88% | 781 |
| [NP_061173.1](http://www.ncbi.nlm.nih.gov/entrez/query.fcgi?cmd=Retrieve&db=Protein&list_uids=33620716&dopt=GenPept&RID=T2PJYNMM01N&log$=prottop&blast_rank=94) | retinoblastoma-binding protein 6 isoform 2 [Homo sapiens] | [17.6](http://blast.ncbi.nlm.nih.gov/Blast.cgi#33620716%2333620716) | 17.6 | 88% | 781 |
| [NP_003622.2](http://www.ncbi.nlm.nih.gov/entrez/query.fcgi?cmd=Retrieve&db=Protein&list_uids=70610136&dopt=GenPept&RID=T2PJYNMM01N&log$=prottop&blast_rank=95) | poly (ADP-ribose) glycohydrolase [Homo sapiens] | [17.6](http://blast.ncbi.nlm.nih.gov/Blast.cgi#70610136%2370610136) | 17.6 | 55% | 781 |
| [NP_003876.1](http://www.ncbi.nlm.nih.gov/entrez/query.fcgi?cmd=Retrieve&db=Protein&list_uids=4502737&dopt=GenPept&RID=T2PJYNMM01N&log$=prottop&blast_rank=96) | cyclin-dependent kinase 5, regulatory subunit 1 [Homo sapiens] | [17.6](http://blast.ncbi.nlm.nih.gov/Blast.cgi#4502737%234502737) | 17.6 | 66% | 781 |
| [NP_000069.2](http://www.ncbi.nlm.nih.gov/entrez/query.fcgi?cmd=Retrieve&db=Protein&list_uids=169636439&dopt=GenPept&RID=T2PJYNMM01N&log$=prottop&blast_rank=97) | cholesteryl ester transfer protein, plasma precursor [Homo sapiens] | [17.6](http://blast.ncbi.nlm.nih.gov/Blast.cgi#169636439%23169636439) | 33.1 | 88% | 781 |
| [NP_001136023.1](http://www.ncbi.nlm.nih.gov/entrez/query.fcgi?cmd=Retrieve&db=Protein&list_uids=216548382&dopt=GenPept&RID=T2PJYNMM01N&log$=prottop&blast_rank=98) | WD repeat domain 47 isoform 3 [Homo sapiens] | [17.6](http://blast.ncbi.nlm.nih.gov/Blast.cgi#216548382%23216548382) | 17.6 | 77% | 781 |
| [NP_003927.1](http://www.ncbi.nlm.nih.gov/entrez/query.fcgi?cmd=Retrieve&db=Protein&list_uids=4502739&dopt=GenPept&RID=T2PJYNMM01N&log$=prottop&blast_rank=99) | cyclin-dependent kinase 5, regulatory subunit 2 precursor [Homo sapiens] | [17.6](http://blast.ncbi.nlm.nih.gov/Blast.cgi#4502739%234502739) | 17.6 | 66% | 781 |
| [NP_001005188.1](http://www.ncbi.nlm.nih.gov/entrez/query.fcgi?cmd=Retrieve&db=Protein&list_uids=52353274&dopt=GenPept&RID=T2PJYNMM01N&log$=prottop&blast_rank=100) | olfactory receptor, family 6, subfamily X, member 1 [Homo sapiens] | [14.2](http://blast.ncbi.nlm.nih.gov/Blast.cgi#52353274%2352353274) | 14.2 | 55% | 8210 |

| **Accession** | **Proteins with a match to GNPWSKQINI peptide** | **[Max score](http://blast.ncbi.nlm.nih.gov/Blast.cgi?CMD=Get&ALIGNMENTS=100&ALIGNMENT_VIEW=Pairwise&CDD_SEARCH_STATE=1&DATABASE_SORT=0&DESCRIPTIONS=100&ENTREZ_QUERY=txid9606 %5BORGN%5D&FIRST_QUERY_NUM=0&FORMAT_OBJECT=Alignment&FORMAT_PAGE_TARGET=&FORMAT_TYPE=HTML&GET_SEQUENCE=yes&I_THRESH=&MASK_CHAR=2&MASK_COLOR=1&NEW_DESIGN=on&NEW_VIEW=yes&NUM_OVERVIEW=100&OLD_BLAST=false&PAGE=Proteins&QUERY_INDEX=0&QUERY_NUMBER=0&RESULTS_PAGE_TARGET=&RID=T2PYS44A01S&SHOW_LINKOUT=yes&SHOW_OVERVIEW=yes&STEP_NUMBER=&WORD_SIZE=2&DISPLAY_SORT=1&HSP_SORT=1" \l "sort_mark)** | **[Total score](http://blast.ncbi.nlm.nih.gov/Blast.cgi?CMD=Get&ALIGNMENTS=100&ALIGNMENT_VIEW=Pairwise&CDD_SEARCH_STATE=1&DATABASE_SORT=0&DESCRIPTIONS=100&ENTREZ_QUERY=txid9606 %5BORGN%5D&FIRST_QUERY_NUM=0&FORMAT_OBJECT=Alignment&FORMAT_PAGE_TARGET=&FORMAT_TYPE=HTML&GET_SEQUENCE=yes&I_THRESH=&MASK_CHAR=2&MASK_COLOR=1&NEW_DESIGN=on&NEW_VIEW=yes&NUM_OVERVIEW=100&OLD_BLAST=false&PAGE=Proteins&QUERY_INDEX=0&QUERY_NUMBER=0&RESULTS_PAGE_TARGET=&RID=T2PYS44A01S&SHOW_LINKOUT=yes&SHOW_OVERVIEW=yes&STEP_NUMBER=&WORD_SIZE=2&DISPLAY_SORT=2&HSP_SORT=1" \l "sort_mark)** | **[Query coverage](http://blast.ncbi.nlm.nih.gov/Blast.cgi?CMD=Get&ALIGNMENTS=100&ALIGNMENT_VIEW=Pairwise&CDD_SEARCH_STATE=1&DATABASE_SORT=0&DESCRIPTIONS=100&ENTREZ_QUERY=txid9606 %5BORGN%5D&FIRST_QUERY_NUM=0&FORMAT_OBJECT=Alignment&FORMAT_PAGE_TARGET=&FORMAT_TYPE=HTML&GET_SEQUENCE=yes&I_THRESH=&MASK_CHAR=2&MASK_COLOR=1&NEW_DESIGN=on&NEW_VIEW=yes&NUM_OVERVIEW=100&OLD_BLAST=false&PAGE=Proteins&QUERY_INDEX=0&QUERY_NUMBER=0&RESULTS_PAGE_TARGET=&RID=T2PYS44A01S&SHOW_LINKOUT=yes&SHOW_OVERVIEW=yes&STEP_NUMBER=&WORD_SIZE=2&DISPLAY_SORT=4&HSP_SORT=0" \l "sort_mark)** | **[E value](http://blast.ncbi.nlm.nih.gov/Blast.cgi?CMD=Get&ALIGNMENTS=100&ALIGNMENT_VIEW=Pairwise&CDD_SEARCH_STATE=1&DATABASE_SORT=0&DESCRIPTIONS=100&ENTREZ_QUERY=txid9606 %5BORGN%5D&FIRST_QUERY_NUM=0&FORMAT_OBJECT=Alignment&FORMAT_PAGE_TARGET=&FORMAT_TYPE=HTML&GET_SEQUENCE=yes&I_THRESH=&MASK_CHAR=2&MASK_COLOR=1&NEW_DESIGN=on&NEW_VIEW=yes&NUM_OVERVIEW=100&OLD_BLAST=false&PAGE=Proteins&QUERY_INDEX=0&QUERY_NUMBER=0&RESULTS_PAGE_TARGET=&RID=T2PYS44A01S&SHOW_LINKOUT=yes&SHOW_OVERVIEW=yes&STEP_NUMBER=&WORD_SIZE=2&DISPLAY_SORT=0&HSP_SORT=0" \l "sort_mark)** |
| --- | --- | --- | --- | --- | --- |
| [NP_000713.2](http://www.ncbi.nlm.nih.gov/entrez/query.fcgi?cmd=Retrieve&db=Protein&list_uids=54112390&dopt=GenPept&RID=T2PYS44A01S&log$=prottop&blast_rank=1) | calcium channel, voltage-dependent, alpha 2/delta subunit 1 [Homo sapiens] | [21.8](http://blast.ncbi.nlm.nih.gov/Blast.cgi#54112390%2354112390) | 21.8 | 70% | 46 |
| [XP_942285.2](http://www.ncbi.nlm.nih.gov/entrez/query.fcgi?cmd=Retrieve&db=Protein&list_uids=169166778&dopt=GenPept&RID=T2PYS44A01S&log$=prottop&blast_rank=2) | PREDICTED: similar to hCG2040199 [Homo sapiens] >ref|XP_001715526.2| PREDICTED: similar to hCG2040199 [Homo sapiens] | [21.0](http://blast.ncbi.nlm.nih.gov/Blast.cgi#169166778%23169166778) | 21.0 | 60% | 83 |
| [XP_932787.2](http://www.ncbi.nlm.nih.gov/entrez/query.fcgi?cmd=Retrieve&db=Protein&list_uids=169166363&dopt=GenPept&RID=T2PYS44A01S&log$=prottop&blast_rank=3) | PREDICTED: similar to hCG2040199 [Homo sapiens] | [21.0](http://blast.ncbi.nlm.nih.gov/Blast.cgi#169166363%23169166363) | 21.0 | 60% | 83 |
| [NP_658988.2](http://www.ncbi.nlm.nih.gov/entrez/query.fcgi?cmd=Retrieve&db=Protein&list_uids=146260268&dopt=GenPept&RID=T2PYS44A01S&log$=prottop&blast_rank=4) | Smith-Magenis syndrome chromosome region, candidate 8 [Homo sapiens] | [21.0](http://blast.ncbi.nlm.nih.gov/Blast.cgi#146260268%23146260268) | 21.0 | 50% | 83 |
| [NP_109592.1](http://www.ncbi.nlm.nih.gov/entrez/query.fcgi?cmd=Retrieve&db=Protein&list_uids=13677214&dopt=GenPept&RID=T2PYS44A01S&log$=prottop&blast_rank=5) | receptor-type protein tyrosine phosphatase O isoform a precursor [Homo sapiens] | [21.0](http://blast.ncbi.nlm.nih.gov/Blast.cgi#13677214%2313677214) | 21.0 | 50% | 83 |
| [NP_109594.1](http://www.ncbi.nlm.nih.gov/entrez/query.fcgi?cmd=Retrieve&db=Protein&list_uids=13677218&dopt=GenPept&RID=T2PYS44A01S&log$=prottop&blast_rank=6) | receptor-type protein tyrosine phosphatase O isoform c precursor [Homo sapiens] >ref|NP_109596.1| receptor-type protein tyrosine phosphatase O isoform c precursor [Homo sapiens] | [21.0](http://blast.ncbi.nlm.nih.gov/Blast.cgi#13677218%2313677218) | 21.0 | 50% | 83 |
| [NP_002052.1](http://www.ncbi.nlm.nih.gov/entrez/query.fcgi?cmd=Retrieve&db=Protein&list_uids=4504011&dopt=GenPept&RID=T2PYS44A01S&log$=prottop&blast_rank=7) | glutamate-cysteine ligase regulatory protein [Homo sapiens] | [21.0](http://blast.ncbi.nlm.nih.gov/Blast.cgi#4504011%234504011) | 21.0 | 80% | 83 |
| [NP_001127880.1](http://www.ncbi.nlm.nih.gov/entrez/query.fcgi?cmd=Retrieve&db=Protein&list_uids=197313638&dopt=GenPept&RID=T2PYS44A01S&log$=prottop&blast_rank=8) | N-methyl-D-aspartate receptor subunit 2A isoform 2 precursor [Homo sapiens] | [20.6](http://blast.ncbi.nlm.nih.gov/Blast.cgi#197313638%23197313638) | 20.6 | 90% | 111 |
| [NP_000824.1](http://www.ncbi.nlm.nih.gov/entrez/query.fcgi?cmd=Retrieve&db=Protein&list_uids=4504125&dopt=GenPept&RID=T2PYS44A01S&log$=prottop&blast_rank=9) | N-methyl-D-aspartate receptor subunit 2A isoform 1 precursor [Homo sapiens] >ref|NP_001127879.1| N-methyl-D-aspartate receptor subunit 2A isoform 1 precursor [Homo sapiens] | [20.6](http://blast.ncbi.nlm.nih.gov/Blast.cgi#4504125%234504125) | 20.6 | 90% | 111 |
| [NP_001116105.1](http://www.ncbi.nlm.nih.gov/entrez/query.fcgi?cmd=Retrieve&db=Protein&list_uids=169790915&dopt=GenPept&RID=T2PYS44A01S&log$=prottop&blast_rank=10) | carbamoyl-phosphate synthetase 1 isoform a precursor [Homo sapiens] | [20.2](http://blast.ncbi.nlm.nih.gov/Blast.cgi#169790915%23169790915) | 20.2 | 70% | 149 |
| [XP_001725513.1](http://www.ncbi.nlm.nih.gov/entrez/query.fcgi?cmd=Retrieve&db=Protein&list_uids=169205475&dopt=GenPept&RID=T2PYS44A01S&log$=prottop&blast_rank=11) | PREDICTED: similar to peroxiredoxin 6 [Homo sapiens] | [20.2](http://blast.ncbi.nlm.nih.gov/Blast.cgi#169205475%23169205475) | 20.2 | 60% | 149 |
| [NP_872357.2](http://www.ncbi.nlm.nih.gov/entrez/query.fcgi?cmd=Retrieve&db=Protein&list_uids=42558246&dopt=GenPept&RID=T2PYS44A01S&log$=prottop&blast_rank=12) | lysocardiolipin acyltransferase 1 isoform 1 [Homo sapiens] | [20.2](http://blast.ncbi.nlm.nih.gov/Blast.cgi#42558246%2342558246) | 31.8 | 80% | 149 |
| [NP_001116106.1](http://www.ncbi.nlm.nih.gov/entrez/query.fcgi?cmd=Retrieve&db=Protein&list_uids=170295797&dopt=GenPept&RID=T2PYS44A01S&log$=prottop&blast_rank=13) | carbamoyl-phosphate synthetase 1 isoform c [Homo sapiens] | [20.2](http://blast.ncbi.nlm.nih.gov/Blast.cgi#170295797%23170295797) | 20.2 | 70% | 149 |
| [NP_058515.1](http://www.ncbi.nlm.nih.gov/entrez/query.fcgi?cmd=Retrieve&db=Protein&list_uids=8567388&dopt=GenPept&RID=T2PYS44A01S&log$=prottop&blast_rank=14) | period 3 [Homo sapiens] | [20.2](http://blast.ncbi.nlm.nih.gov/Blast.cgi#8567388%238567388) | 20.2 | 70% | 149 |
| [NP_001866.2](http://www.ncbi.nlm.nih.gov/entrez/query.fcgi?cmd=Retrieve&db=Protein&list_uids=21361331&dopt=GenPept&RID=T2PYS44A01S&log$=prottop&blast_rank=15) | carbamoyl-phosphate synthetase 1 isoform b precursor [Homo sapiens] | [20.2](http://blast.ncbi.nlm.nih.gov/Blast.cgi#21361331%2321361331) | 20.2 | 70% | 149 |
| [NP_004896.1](http://www.ncbi.nlm.nih.gov/entrez/query.fcgi?cmd=Retrieve&db=Protein&list_uids=4758638&dopt=GenPept&RID=T2PYS44A01S&log$=prottop&blast_rank=16) | peroxiredoxin 6 [Homo sapiens] | [20.2](http://blast.ncbi.nlm.nih.gov/Blast.cgi#4758638%234758638) | 20.2 | 60% | 149 |
| [NP_073728.1](http://www.ncbi.nlm.nih.gov/entrez/query.fcgi?cmd=Retrieve&db=Protein&list_uids=12707562&dopt=GenPept&RID=T2PYS44A01S&log$=prottop&blast_rank=17) | period 2 [Homo sapiens] | [20.2](http://blast.ncbi.nlm.nih.gov/Blast.cgi#12707562%2312707562) | 31.0 | 80% | 149 |
| [NP_056327.4](http://www.ncbi.nlm.nih.gov/entrez/query.fcgi?cmd=Retrieve&db=Protein&list_uids=197927452&dopt=GenPept&RID=T2PYS44A01S&log$=prottop&blast_rank=18) | dynein, axonemal, heavy chain 1 [Homo sapiens] | [19.7](http://blast.ncbi.nlm.nih.gov/Blast.cgi#197927452%23197927452) | 32.2 | 90% | 200 |
| [NP_149014.3](http://www.ncbi.nlm.nih.gov/entrez/query.fcgi?cmd=Retrieve&db=Protein&list_uids=42475970&dopt=GenPept&RID=T2PYS44A01S&log$=prottop&blast_rank=19) | synapse defective 1, Rho GTPase, homolog 1 [Homo sapiens] | [19.7](http://blast.ncbi.nlm.nih.gov/Blast.cgi#42475970%2342475970) | 19.7 | 60% | 200 |
| [NP_001025444.1](http://www.ncbi.nlm.nih.gov/entrez/query.fcgi?cmd=Retrieve&db=Protein&list_uids=71852582&dopt=GenPept&RID=T2PYS44A01S&log$=prottop&blast_rank=20) | aryl hydrocarbon receptor nuclear translocator-like isoform b [Homo sapiens] | [19.7](http://blast.ncbi.nlm.nih.gov/Blast.cgi#71852582%2371852582) | 19.7 | 70% | 200 |
| [NP_001169.3](http://www.ncbi.nlm.nih.gov/entrez/query.fcgi?cmd=Retrieve&db=Protein&list_uids=42716311&dopt=GenPept&RID=T2PYS44A01S&log$=prottop&blast_rank=21) | aryl hydrocarbon receptor nuclear translocator-like isoform a [Homo sapiens] >ref|NP_001025443.1| aryl hydrocarbon receptor nuclear translocator-like isoform a [Homo sapiens] | [19.7](http://blast.ncbi.nlm.nih.gov/Blast.cgi#42716311%2342716311) | 19.7 | 70% | 200 |
| [NP_060762.3](http://www.ncbi.nlm.nih.gov/entrez/query.fcgi?cmd=Retrieve&db=Protein&list_uids=222831590&dopt=GenPept&RID=T2PYS44A01S&log$=prottop&blast_rank=22) | glutaminyl-tRNA synthase (glutamine-hydrolyzing)-like 1 [Homo sapiens] | [19.7](http://blast.ncbi.nlm.nih.gov/Blast.cgi#222831590%23222831590) | 19.7 | 60% | 200 |
| [NP_002913.3](http://www.ncbi.nlm.nih.gov/entrez/query.fcgi?cmd=Retrieve&db=Protein&list_uids=115387108&dopt=GenPept&RID=T2PYS44A01S&log$=prottop&blast_rank=23) | regulator of G-protein signalling 1 [Homo sapiens] | [19.7](http://blast.ncbi.nlm.nih.gov/Blast.cgi#115387108%23115387108) | 19.7 | 50% | 200 |
| [NP_057291.1](http://www.ncbi.nlm.nih.gov/entrez/query.fcgi?cmd=Retrieve&db=Protein&list_uids=7706427&dopt=GenPept&RID=T2PYS44A01S&log$=prottop&blast_rank=24) | cleavage and polyadenylation specific factor 3, 73kDa [Homo sapiens] | [19.7](http://blast.ncbi.nlm.nih.gov/Blast.cgi#7706427%237706427) | 19.7 | 50% | 200 |
| [NP_001157412.1](http://www.ncbi.nlm.nih.gov/entrez/query.fcgi?cmd=Retrieve&db=Protein&list_uids=255653002&dopt=GenPept&RID=T2PYS44A01S&log$=prottop&blast_rank=25) | liver glycogen phosphorylase isoform 2 [Homo sapiens] | [19.3](http://blast.ncbi.nlm.nih.gov/Blast.cgi#255653002%23255653002) | 51.5 | 90% | 268 |
| [NP_001158188.1](http://www.ncbi.nlm.nih.gov/entrez/query.fcgi?cmd=Retrieve&db=Protein&list_uids=257900462&dopt=GenPept&RID=T2PYS44A01S&log$=prottop&blast_rank=26) | muscle glycogen phosphorylase isoform 2 [Homo sapiens] | [19.3](http://blast.ncbi.nlm.nih.gov/Blast.cgi#257900462%23257900462) | 19.3 | 60% | 268 |
| [NP_065705.3](http://www.ncbi.nlm.nih.gov/entrez/query.fcgi?cmd=Retrieve&db=Protein&list_uids=116174746&dopt=GenPept&RID=T2PYS44A01S&log$=prottop&blast_rank=27) | sentrin/SUMO-specific protease 7 isoform 1 [Homo sapiens] | [19.3](http://blast.ncbi.nlm.nih.gov/Blast.cgi#116174746%23116174746) | 19.3 | 70% | 268 |
| [NP_001070671.1](http://www.ncbi.nlm.nih.gov/entrez/query.fcgi?cmd=Retrieve&db=Protein&list_uids=116174736&dopt=GenPept&RID=T2PYS44A01S&log$=prottop&blast_rank=28) | sentrin/SUMO-specific protease 7 isoform 2 [Homo sapiens] | [19.3](http://blast.ncbi.nlm.nih.gov/Blast.cgi#116174736%23116174736) | 19.3 | 70% | 268 |
| [NP_008847.1](http://www.ncbi.nlm.nih.gov/entrez/query.fcgi?cmd=Retrieve&db=Protein&list_uids=24307923&dopt=GenPept&RID=T2PYS44A01S&log$=prottop&blast_rank=29) | ribulose-5-phosphate-3-epimerase isoform 2 [Homo sapiens] | [19.3](http://blast.ncbi.nlm.nih.gov/Blast.cgi#24307923%2324307923) | 19.3 | 70% | 268 |
| [NP_001367.2](http://www.ncbi.nlm.nih.gov/entrez/query.fcgi?cmd=Retrieve&db=Protein&list_uids=33350932&dopt=GenPept&RID=T2PYS44A01S&log$=prottop&blast_rank=30) | cytoplasmic dynein 1 heavy chain 1 [Homo sapiens] | [19.3](http://blast.ncbi.nlm.nih.gov/Blast.cgi#33350932%2333350932) | 29.3 | 70% | 268 |
| [NP_001137381.1](http://www.ncbi.nlm.nih.gov/entrez/query.fcgi?cmd=Retrieve&db=Protein&list_uids=219879828&dopt=GenPept&RID=T2PYS44A01S&log$=prottop&blast_rank=31) | rcRPE protein [Homo sapiens] | [19.3](http://blast.ncbi.nlm.nih.gov/Blast.cgi#219879828%23219879828) | 19.3 | 70% | 268 |
| [NP_114432.1](http://www.ncbi.nlm.nih.gov/entrez/query.fcgi?cmd=Retrieve&db=Protein&list_uids=14042978&dopt=GenPept&RID=T2PYS44A01S&log$=prottop&blast_rank=32) | BRCA1 interacting protein C-terminal helicase 1 [Homo sapiens] | [19.3](http://blast.ncbi.nlm.nih.gov/Blast.cgi#14042978%2314042978) | 29.7 | 70% | 268 |
| [NP_002854.3](http://www.ncbi.nlm.nih.gov/entrez/query.fcgi?cmd=Retrieve&db=Protein&list_uids=71037379&dopt=GenPept&RID=T2PYS44A01S&log$=prottop&blast_rank=33) | liver glycogen phosphorylase isoform 1 [Homo sapiens] | [19.3](http://blast.ncbi.nlm.nih.gov/Blast.cgi#71037379%2371037379) | 51.5 | 90% | 268 |
| [NP_005600.1](http://www.ncbi.nlm.nih.gov/entrez/query.fcgi?cmd=Retrieve&db=Protein&list_uids=5032009&dopt=GenPept&RID=T2PYS44A01S&log$=prottop&blast_rank=34) | muscle glycogen phosphorylase isoform 1 [Homo sapiens] | [19.3](http://blast.ncbi.nlm.nih.gov/Blast.cgi#5032009%235032009) | 19.3 | 60% | 268 |
| [NP_002853.2](http://www.ncbi.nlm.nih.gov/entrez/query.fcgi?cmd=Retrieve&db=Protein&list_uids=21361370&dopt=GenPept&RID=T2PYS44A01S&log$=prottop&blast_rank=35) | brain glycogen phosphorylase [Homo sapiens] | [19.3](http://blast.ncbi.nlm.nih.gov/Blast.cgi#21361370%2321361370) | 19.3 | 60% | 268 |
| [NP_954699.1](http://www.ncbi.nlm.nih.gov/entrez/query.fcgi?cmd=Retrieve&db=Protein&list_uids=40385883&dopt=GenPept&RID=T2PYS44A01S&log$=prottop&blast_rank=36) | ribulose-5-phosphate-3-epimerase isoform 1 [Homo sapiens] | [19.3](http://blast.ncbi.nlm.nih.gov/Blast.cgi#40385883%2340385883) | 19.3 | 70% | 268 |
| [XP_002346371.1](http://www.ncbi.nlm.nih.gov/entrez/query.fcgi?cmd=Retrieve&db=Protein&list_uids=239757898&dopt=GenPept&RID=T2PYS44A01S&log$=prottop&blast_rank=37) | PREDICTED: similar to testis expressed sequence 13A [Homo sapiens] | [18.9](http://blast.ncbi.nlm.nih.gov/Blast.cgi#239757898%23239757898) | 18.9 | 70% | 359 |
| [NP_001165464.1](http://www.ncbi.nlm.nih.gov/entrez/query.fcgi?cmd=Retrieve&db=Protein&list_uids=285002264&dopt=GenPept&RID=T2PYS44A01S&log$=prottop&blast_rank=38) | 4-hydroxyphenylpyruvate dioxygenase isoform 2 [Homo sapiens] | [18.9](http://blast.ncbi.nlm.nih.gov/Blast.cgi#285002264%23285002264) | 18.9 | 60% | 359 |
| [NP_004511.2](http://www.ncbi.nlm.nih.gov/entrez/query.fcgi?cmd=Retrieve&db=Protein&list_uids=148612877&dopt=GenPept&RID=T2PYS44A01S&log$=prottop&blast_rank=39) | kinesin heavy chain member 2 isoform 1 [Homo sapiens] | [18.9](http://blast.ncbi.nlm.nih.gov/Blast.cgi#148612877%23148612877) | 18.9 | 50% | 359 |
| [NP_001091981.1](http://www.ncbi.nlm.nih.gov/entrez/query.fcgi?cmd=Retrieve&db=Protein&list_uids=148612849&dopt=GenPept&RID=T2PYS44A01S&log$=prottop&blast_rank=40) | kinesin heavy chain member 2 isoform 2 [Homo sapiens] | [18.9](http://blast.ncbi.nlm.nih.gov/Blast.cgi#148612849%23148612849) | 18.9 | 50% | 359 |
| [NP_064568.3](http://www.ncbi.nlm.nih.gov/entrez/query.fcgi?cmd=Retrieve&db=Protein&list_uids=31745180&dopt=GenPept&RID=T2PYS44A01S&log$=prottop&blast_rank=41) | aryl hydrocarbon receptor nuclear translocator-like 2 [Homo sapiens] | [18.9](http://blast.ncbi.nlm.nih.gov/Blast.cgi#31745180%2331745180) | 18.9 | 60% | 359 |
| [NP_001006115.1](http://www.ncbi.nlm.nih.gov/entrez/query.fcgi?cmd=Retrieve&db=Protein&list_uids=55769518&dopt=GenPept&RID=T2PYS44A01S&log$=prottop&blast_rank=42) | inositol hexakisphosphate kinase 1 isoform 2 [Homo sapiens] | [18.9](http://blast.ncbi.nlm.nih.gov/Blast.cgi#55769518%2355769518) | 18.9 | 60% | 359 |
| [XP_002348264.1](http://www.ncbi.nlm.nih.gov/entrez/query.fcgi?cmd=Retrieve&db=Protein&list_uids=239752412&dopt=GenPept&RID=T2PYS44A01S&log$=prottop&blast_rank=43) | PREDICTED: similar to testis expressed sequence 13A [Homo sapiens] | [18.9](http://blast.ncbi.nlm.nih.gov/Blast.cgi#239752412%23239752412) | 18.9 | 70% | 359 |
| [XP_001715046.1](http://www.ncbi.nlm.nih.gov/entrez/query.fcgi?cmd=Retrieve&db=Protein&list_uids=169216868&dopt=GenPept&RID=T2PYS44A01S&log$=prottop&blast_rank=44) | PREDICTED: similar to testis expressed sequence 13A [Homo sapiens] | [18.9](http://blast.ncbi.nlm.nih.gov/Blast.cgi#169216868%23169216868) | 18.9 | 70% | 359 |
| [NP_000523.2](http://www.ncbi.nlm.nih.gov/entrez/query.fcgi?cmd=Retrieve&db=Protein&list_uids=119943100&dopt=GenPept&RID=T2PYS44A01S&log$=prottop&blast_rank=45) | propionyl Coenzyme A carboxylase, beta polypeptide precursor [Homo sapiens] | [18.9](http://blast.ncbi.nlm.nih.gov/Blast.cgi#119943100%23119943100) | 18.9 | 80% | 359 |
| [NP_695005.1](http://www.ncbi.nlm.nih.gov/entrez/query.fcgi?cmd=Retrieve&db=Protein&list_uids=23510335&dopt=GenPept&RID=T2PYS44A01S&log$=prottop&blast_rank=46) | inositol hexakisphosphate kinase 1 isoform 1 [Homo sapiens] | [18.9](http://blast.ncbi.nlm.nih.gov/Blast.cgi#23510335%2323510335) | 18.9 | 60% | 359 |
| [NP_002141.1](http://www.ncbi.nlm.nih.gov/entrez/query.fcgi?cmd=Retrieve&db=Protein&list_uids=4504477&dopt=GenPept&RID=T2PYS44A01S&log$=prottop&blast_rank=47) | 4-hydroxyphenylpyruvate dioxygenase isoform 1 [Homo sapiens] | [18.9](http://blast.ncbi.nlm.nih.gov/Blast.cgi#4504477%234504477) | 18.9 | 60% | 359 |
| [NP_001010904.1](http://www.ncbi.nlm.nih.gov/entrez/query.fcgi?cmd=Retrieve&db=Protein&list_uids=222831633&dopt=GenPept&RID=T2PYS44A01S&log$=prottop&blast_rank=48) | glycine N-acyltransferase-like protein 3 [Homo sapiens] | [18.5](http://blast.ncbi.nlm.nih.gov/Blast.cgi#222831633%23222831633) | 18.5 | 60% | 482 |
| [NP_001075042.1](http://www.ncbi.nlm.nih.gov/entrez/query.fcgi?cmd=Retrieve&db=Protein&list_uids=126091130&dopt=GenPept&RID=T2PYS44A01S&log$=prottop&blast_rank=49) | Gab3 protein isoform 1 [Homo sapiens] | [18.5](http://blast.ncbi.nlm.nih.gov/Blast.cgi#126091130%23126091130) | 18.5 | 60% | 482 |
| [NP_689799.3](http://www.ncbi.nlm.nih.gov/entrez/query.fcgi?cmd=Retrieve&db=Protein&list_uids=124001558&dopt=GenPept&RID=T2PYS44A01S&log$=prottop&blast_rank=50) | ubiquitin specific peptidase 54 [Homo sapiens] | [18.5](http://blast.ncbi.nlm.nih.gov/Blast.cgi#124001558%23124001558) | 18.5 | 50% | 482 |
| [NP_001036069.1](http://www.ncbi.nlm.nih.gov/entrez/query.fcgi?cmd=Retrieve&db=Protein&list_uids=110624787&dopt=GenPept&RID=T2PYS44A01S&log$=prottop&blast_rank=51) | 5'-3' exoribonuclease 1 isoform b [Homo sapiens] | [18.5](http://blast.ncbi.nlm.nih.gov/Blast.cgi#110624787%23110624787) | 28.4 | 60% | 482 |
| [NP_115740.5](http://www.ncbi.nlm.nih.gov/entrez/query.fcgi?cmd=Retrieve&db=Protein&list_uids=119943096&dopt=GenPept&RID=T2PYS44A01S&log$=prottop&blast_rank=52) | dopamine receptor interacting protein [Homo sapiens] | [18.5](http://blast.ncbi.nlm.nih.gov/Blast.cgi#119943096%23119943096) | 18.5 | 70% | 482 |
| [NP_055178.3](http://www.ncbi.nlm.nih.gov/entrez/query.fcgi?cmd=Retrieve&db=Protein&list_uids=163659918&dopt=GenPept&RID=T2PYS44A01S&log$=prottop&blast_rank=53) | sacsin [Homo sapiens] | [18.5](http://blast.ncbi.nlm.nih.gov/Blast.cgi#163659918%23163659918) | 33.1 | 80% | 482 |
| [NP_665699.1](http://www.ncbi.nlm.nih.gov/entrez/query.fcgi?cmd=Retrieve&db=Protein&list_uids=21955239&dopt=GenPept&RID=T2PYS44A01S&log$=prottop&blast_rank=54) | zinc finger protein 396 [Homo sapiens] | [18.5](http://blast.ncbi.nlm.nih.gov/Blast.cgi#21955239%2321955239) | 18.5 | 70% | 482 |
| [NP_542179.1](http://www.ncbi.nlm.nih.gov/entrez/query.fcgi?cmd=Retrieve&db=Protein&list_uids=18079323&dopt=GenPept&RID=T2PYS44A01S&log$=prottop&blast_rank=55) | Gab3 protein isoform 2 [Homo sapiens] | [18.5](http://blast.ncbi.nlm.nih.gov/Blast.cgi#18079323%2318079323) | 18.5 | 60% | 482 |
| [NP_057629.2](http://www.ncbi.nlm.nih.gov/entrez/query.fcgi?cmd=Retrieve&db=Protein&list_uids=142976757&dopt=GenPept&RID=T2PYS44A01S&log$=prottop&blast_rank=56) | immediate early response 5 [Homo sapiens] | [18.5](http://blast.ncbi.nlm.nih.gov/Blast.cgi#142976757%23142976757) | 18.5 | 70% | 482 |
| [NP_291029.2](http://www.ncbi.nlm.nih.gov/entrez/query.fcgi?cmd=Retrieve&db=Protein&list_uids=61102725&dopt=GenPept&RID=T2PYS44A01S&log$=prottop&blast_rank=57) | la related protein isoform 2 [Homo sapiens] | [18.5](http://blast.ncbi.nlm.nih.gov/Blast.cgi#61102725%2361102725) | 18.5 | 50% | 482 |
| [NP_006436.3](http://www.ncbi.nlm.nih.gov/entrez/query.fcgi?cmd=Retrieve&db=Protein&list_uids=91208426&dopt=GenPept&RID=T2PYS44A01S&log$=prottop&blast_rank=58) | U5 snRNP-specific protein [Homo sapiens] | [18.5](http://blast.ncbi.nlm.nih.gov/Blast.cgi#91208426%2391208426) | 43.5 | 70% | 482 |
| [NP_061874.3](http://www.ncbi.nlm.nih.gov/entrez/query.fcgi?cmd=Retrieve&db=Protein&list_uids=110624792&dopt=GenPept&RID=T2PYS44A01S&log$=prottop&blast_rank=59) | 5'-3' exoribonuclease 1 isoform a [Homo sapiens] | [18.5](http://blast.ncbi.nlm.nih.gov/Blast.cgi#110624792%23110624792) | 28.4 | 60% | 482 |
| [NP_001829.1](http://www.ncbi.nlm.nih.gov/entrez/query.fcgi?cmd=Retrieve&db=Protein&list_uids=4502641&dopt=GenPept&RID=T2PYS44A01S&log$=prottop&blast_rank=60) | chemokine (C-C motif) receptor 7 precursor [Homo sapiens] | [18.5](http://blast.ncbi.nlm.nih.gov/Blast.cgi#4502641%234502641) | 18.5 | 60% | 482 |
| [NP_055472.1](http://www.ncbi.nlm.nih.gov/entrez/query.fcgi?cmd=Retrieve&db=Protein&list_uids=24307961&dopt=GenPept&RID=T2PYS44A01S&log$=prottop&blast_rank=61) | hypothetical protein LOC9675 [Homo sapiens] | [18.5](http://blast.ncbi.nlm.nih.gov/Blast.cgi#24307961%2324307961) | 18.5 | 60% | 482 |
| [NP_005655.1](http://www.ncbi.nlm.nih.gov/entrez/query.fcgi?cmd=Retrieve&db=Protein&list_uids=5032243&dopt=GenPept&RID=T2PYS44A01S&log$=prottop&blast_rank=62) | makorin ring finger protein 3 [Homo sapiens] | [18.5](http://blast.ncbi.nlm.nih.gov/Blast.cgi#5032243%235032243) | 18.5 | 50% | 482 |
| [XP_001713994.2](http://www.ncbi.nlm.nih.gov/entrez/query.fcgi?cmd=Retrieve&db=Protein&list_uids=239752702&dopt=GenPept&RID=T2PYS44A01S&log$=prottop&blast_rank=63) | PREDICTED: leucine rich repeat containing 38, partial [Homo sapiens] | [18.0](http://blast.ncbi.nlm.nih.gov/Blast.cgi#239752702%23239752702) | 18.0 | 40% | 647 |
| [XP_059074.6](http://www.ncbi.nlm.nih.gov/entrez/query.fcgi?cmd=Retrieve&db=Protein&list_uids=239741057&dopt=GenPept&RID=T2PYS44A01S&log$=prottop&blast_rank=64) | PREDICTED: leucine rich repeat containing 38 [Homo sapiens] >ref|XP_948754.3| PREDICTED: leucine rich repeat containing 38 [Homo sapiens] | [18.0](http://blast.ncbi.nlm.nih.gov/Blast.cgi#239741057%23239741057) | 18.0 | 40% | 647 |
| [XP_002344096.1](http://www.ncbi.nlm.nih.gov/entrez/query.fcgi?cmd=Retrieve&db=Protein&list_uids=239740900&dopt=GenPept&RID=T2PYS44A01S&log$=prottop&blast_rank=65) | PREDICTED: hypothetical protein XP_002344096 [Homo sapiens] >ref|XP_002342661.1| PREDICTED: hypothetical protein XP_002342661 [Homo sapiens] >ref|XP_002346828.1| PREDICTED: hypothetical protein XP_002346828 [Homo sapiens] >ref|XP_002345927.1| PREDICTED: hypothetical protein [Homo sapiens] | [18.0](http://blast.ncbi.nlm.nih.gov/Blast.cgi#239740900%23239740900) | 18.0 | 40% | 647 |
| [XP_002344068.1](http://www.ncbi.nlm.nih.gov/entrez/query.fcgi?cmd=Retrieve&db=Protein&list_uids=239740830&dopt=GenPept&RID=T2PYS44A01S&log$=prottop&blast_rank=66) | PREDICTED: hypothetical protein XP_002344068 [Homo sapiens] | [18.0](http://blast.ncbi.nlm.nih.gov/Blast.cgi#239740830%23239740830) | 18.0 | 40% | 647 |
| [XP_002343964.1](http://www.ncbi.nlm.nih.gov/entrez/query.fcgi?cmd=Retrieve&db=Protein&list_uids=239513944&dopt=GenPept&RID=T2PYS44A01S&log$=prottop&blast_rank=67) | PREDICTED: hypothetical protein [Homo sapiens] >ref|XP_002343987.1| PREDICTED: hypothetical protein [Homo sapiens] >ref|XP_002344036.1| PREDICTED: hypothetical protein [Homo sapiens] | [18.0](http://blast.ncbi.nlm.nih.gov/Blast.cgi#239513944%23239513944) | 18.0 | 40% | 647 |
| [NP_001139586.1](http://www.ncbi.nlm.nih.gov/entrez/query.fcgi?cmd=Retrieve&db=Protein&list_uids=225735625&dopt=GenPept&RID=T2PYS44A01S&log$=prottop&blast_rank=68) | disco-interacting protein 2A isoform e [Homo sapiens] | [18.0](http://blast.ncbi.nlm.nih.gov/Blast.cgi#225735625%23225735625) | 18.0 | 40% | 647 |
| [NP_001139478.1](http://www.ncbi.nlm.nih.gov/entrez/query.fcgi?cmd=Retrieve&db=Protein&list_uids=225579152&dopt=GenPept&RID=T2PYS44A01S&log$=prottop&blast_rank=69) | insulin-like growth factor binding protein, acid labile subunit isoform 1 precursor [Homo sapiens] | [18.0](http://blast.ncbi.nlm.nih.gov/Blast.cgi#225579152%23225579152) | 18.0 | 40% | 647 |
| [NP_001137291.1](http://www.ncbi.nlm.nih.gov/entrez/query.fcgi?cmd=Retrieve&db=Protein&list_uids=219689113&dopt=GenPept&RID=T2PYS44A01S&log$=prottop&blast_rank=70) | two pore segment channel 1 isoform 1 [Homo sapiens] | [18.0](http://blast.ncbi.nlm.nih.gov/Blast.cgi#219689113%23219689113) | 18.0 | 40% | 647 |
| [NP_001173.2](http://www.ncbi.nlm.nih.gov/entrez/query.fcgi?cmd=Retrieve&db=Protein&list_uids=188035924&dopt=GenPept&RID=T2PYS44A01S&log$=prottop&blast_rank=71) | aldehyde dehydrogenase 7 family, member A1 [Homo sapiens] | [18.0](http://blast.ncbi.nlm.nih.gov/Blast.cgi#188035924%23188035924) | 18.0 | 40% | 647 |
| [XP_001714626.1](http://www.ncbi.nlm.nih.gov/entrez/query.fcgi?cmd=Retrieve&db=Protein&list_uids=169178065&dopt=GenPept&RID=T2PYS44A01S&log$=prottop&blast_rank=72) | PREDICTED: hypothetical protein [Homo sapiens] | [18.0](http://blast.ncbi.nlm.nih.gov/Blast.cgi#169178065%23169178065) | 18.0 | 40% | 647 |
| [XP_001719763.1](http://www.ncbi.nlm.nih.gov/entrez/query.fcgi?cmd=Retrieve&db=Protein&list_uids=169179313&dopt=GenPept&RID=T2PYS44A01S&log$=prottop&blast_rank=73) | PREDICTED: hypothetical protein [Homo sapiens] >ref|XP_001725929.1| PREDICTED: hypothetical protein [Homo sapiens] | [18.0](http://blast.ncbi.nlm.nih.gov/Blast.cgi#169179313%23169179313) | 18.0 | 40% | 647 |
| [NP_742067.3](http://www.ncbi.nlm.nih.gov/entrez/query.fcgi?cmd=Retrieve&db=Protein&list_uids=160948610&dopt=GenPept&RID=T2PYS44A01S&log$=prottop&blast_rank=74) | E3 ubiquitin-protein ligase UBR3 [Homo sapiens] | [18.0](http://blast.ncbi.nlm.nih.gov/Blast.cgi#160948610%23160948610) | 18.0 | 40% | 647 |
| [NP_000164.4](http://www.ncbi.nlm.nih.gov/entrez/query.fcgi?cmd=Retrieve&db=Protein&list_uids=150417978&dopt=GenPept&RID=T2PYS44A01S&log$=prottop&blast_rank=75) | platelet glycoprotein Ib alpha polypeptide precursor [Homo sapiens] | [18.0](http://blast.ncbi.nlm.nih.gov/Blast.cgi#150417978%23150417978) | 18.0 | 40% | 647 |
| [NP_056234.2](http://www.ncbi.nlm.nih.gov/entrez/query.fcgi?cmd=Retrieve&db=Protein&list_uids=139948432&dopt=GenPept&RID=T2PYS44A01S&log$=prottop&blast_rank=76) | adlican precursor [Homo sapiens] | [18.0](http://blast.ncbi.nlm.nih.gov/Blast.cgi#139948432%23139948432) | 34.4 | 80% | 647 |
| [NP_001073860.1](http://www.ncbi.nlm.nih.gov/entrez/query.fcgi?cmd=Retrieve&db=Protein&list_uids=122939208&dopt=GenPept&RID=T2PYS44A01S&log$=prottop&blast_rank=77) | nuclear antigen Sp100 isoform 1 [Homo sapiens] | [18.0](http://blast.ncbi.nlm.nih.gov/Blast.cgi#122939208%23122939208) | 18.0 | 40% | 647 |
| [NP_060146.2](http://www.ncbi.nlm.nih.gov/entrez/query.fcgi?cmd=Retrieve&db=Protein&list_uids=116812606&dopt=GenPept&RID=T2PYS44A01S&log$=prottop&blast_rank=78) | zinc finger, H2C2 domain containing [Homo sapiens] | [18.0](http://blast.ncbi.nlm.nih.gov/Blast.cgi#116812606%23116812606) | 34.8 | 80% | 647 |
| [NP_751946.1](http://www.ncbi.nlm.nih.gov/entrez/query.fcgi?cmd=Retrieve&db=Protein&list_uids=26787966&dopt=GenPept&RID=T2PYS44A01S&log$=prottop&blast_rank=79) | general transcription factor IIA, 1-like isoform 2 [Homo sapiens] | [18.0](http://blast.ncbi.nlm.nih.gov/Blast.cgi#26787966%2326787966) | 18.0 | 40% | 647 |
| [NP_001073896.1](http://www.ncbi.nlm.nih.gov/entrez/query.fcgi?cmd=Retrieve&db=Protein&list_uids=122937257&dopt=GenPept&RID=T2PYS44A01S&log$=prottop&blast_rank=80) | thrombospondin, type I, domain containing 7B [Homo sapiens] | [18.0](http://blast.ncbi.nlm.nih.gov/Blast.cgi#122937257%23122937257) | 32.2 | 70% | 647 |
| [NP_001082.2](http://www.ncbi.nlm.nih.gov/entrez/query.fcgi?cmd=Retrieve&db=Protein&list_uids=73486661&dopt=GenPept&RID=T2PYS44A01S&log$=prottop&blast_rank=81) | amiloride binding protein 1 precursor [Homo sapiens] | [18.0](http://blast.ncbi.nlm.nih.gov/Blast.cgi#73486661%2373486661) | 33.5 | 40% | 647 |
| [NP_005913.2](http://www.ncbi.nlm.nih.gov/entrez/query.fcgi?cmd=Retrieve&db=Protein&list_uids=55956904&dopt=GenPept&RID=T2PYS44A01S&log$=prottop&blast_rank=82) | mitogen-activated protein kinase kinase kinase 4 isoform a [Homo sapiens] | [18.0](http://blast.ncbi.nlm.nih.gov/Blast.cgi#55956904%2355956904) | 18.0 | 60% | 647 |
| [NP_006715.2](http://www.ncbi.nlm.nih.gov/entrez/query.fcgi?cmd=Retrieve&db=Protein&list_uids=55956902&dopt=GenPept&RID=T2PYS44A01S&log$=prottop&blast_rank=83) | mitogen-activated protein kinase kinase kinase 4 isoform b [Homo sapiens] | [18.0](http://blast.ncbi.nlm.nih.gov/Blast.cgi#55956902%2355956902) | 18.0 | 60% | 647 |
| [NP_775873.2](http://www.ncbi.nlm.nih.gov/entrez/query.fcgi?cmd=Retrieve&db=Protein&list_uids=55749758&dopt=GenPept&RID=T2PYS44A01S&log$=prottop&blast_rank=84) | DIP2 disco-interacting protein 2 homolog B [Homo sapiens] | [18.0](http://blast.ncbi.nlm.nih.gov/Blast.cgi#55749758%2355749758) | 18.0 | 40% | 647 |
| [NP_964014.1](http://www.ncbi.nlm.nih.gov/entrez/query.fcgi?cmd=Retrieve&db=Protein&list_uids=42542383&dopt=GenPept&RID=T2PYS44A01S&log$=prottop&blast_rank=85) | solute carrier family 28, member 1 isoform 2 [Homo sapiens] | [18.0](http://blast.ncbi.nlm.nih.gov/Blast.cgi#42542383%2342542383) | 18.0 | 50% | 647 |
| [NP_001013675.1](http://www.ncbi.nlm.nih.gov/entrez/query.fcgi?cmd=Retrieve&db=Protein&list_uids=61966761&dopt=GenPept&RID=T2PYS44A01S&log$=prottop&blast_rank=86) | leucine rich repeat containing 26 precursor [Homo sapiens] | [18.0](http://blast.ncbi.nlm.nih.gov/Blast.cgi#61966761%2361966761) | 18.0 | 40% | 647 |
| [NP_001005210.1](http://www.ncbi.nlm.nih.gov/entrez/query.fcgi?cmd=Retrieve&db=Protein&list_uids=52353306&dopt=GenPept&RID=T2PYS44A01S&log$=prottop&blast_rank=87) | leucine rich repeat containing 55 [Homo sapiens] | [18.0](http://blast.ncbi.nlm.nih.gov/Blast.cgi#52353306%2352353306) | 18.0 | 40% | 647 |
| [NP_849144.2](http://www.ncbi.nlm.nih.gov/entrez/query.fcgi?cmd=Retrieve&db=Protein&list_uids=38490688&dopt=GenPept&RID=T2PYS44A01S&log$=prottop&blast_rank=88) | immunoglobulin superfamily, member 10 precursor [Homo sapiens] | [18.0](http://blast.ncbi.nlm.nih.gov/Blast.cgi#38490688%2338490688) | 32.2 | 90% | 647 |
| [NP_877429.2](http://www.ncbi.nlm.nih.gov/entrez/query.fcgi?cmd=Retrieve&db=Protein&list_uids=38679907&dopt=GenPept&RID=T2PYS44A01S&log$=prottop&blast_rank=89) | tumor protein p63 regulated 1-like [Homo sapiens] | [18.0](http://blast.ncbi.nlm.nih.gov/Blast.cgi#38679907%2338679907) | 18.0 | 40% | 647 |
| [NP_932343.1](http://www.ncbi.nlm.nih.gov/entrez/query.fcgi?cmd=Retrieve&db=Protein&list_uids=37620206&dopt=GenPept&RID=T2PYS44A01S&log$=prottop&blast_rank=90) | hypothetical protein LOC148423 [Homo sapiens] | [18.0](http://blast.ncbi.nlm.nih.gov/Blast.cgi#37620206%2337620206) | 18.0 | 80% | 647 |
| [NP_006863.2](http://www.ncbi.nlm.nih.gov/entrez/query.fcgi?cmd=Retrieve&db=Protein&list_uids=26787968&dopt=GenPept&RID=T2PYS44A01S&log$=prottop&blast_rank=91) | general transcription factor IIA, 1-like isoform 1 [Homo sapiens] | [18.0](http://blast.ncbi.nlm.nih.gov/Blast.cgi#26787968%2326787968) | 18.0 | 40% | 647 |
| [NP_057331.2](http://www.ncbi.nlm.nih.gov/entrez/query.fcgi?cmd=Retrieve&db=Protein&list_uids=57242793&dopt=GenPept&RID=T2PYS44A01S&log$=prottop&blast_rank=92) | interphotoreceptor matrix proteoglycan 2 precursor [Homo sapiens] | [18.0](http://blast.ncbi.nlm.nih.gov/Blast.cgi#57242793%2357242793) | 18.0 | 40% | 647 |
| [NP_060426.2](http://www.ncbi.nlm.nih.gov/entrez/query.fcgi?cmd=Retrieve&db=Protein&list_uids=157388919&dopt=GenPept&RID=T2PYS44A01S&log$=prottop&blast_rank=93) | homolog of yeast tRNA methyltransferase 12 [Homo sapiens] | [18.0](http://blast.ncbi.nlm.nih.gov/Blast.cgi#157388919%23157388919) | 18.0 | 80% | 647 |
| [NP_001139588.1](http://www.ncbi.nlm.nih.gov/entrez/query.fcgi?cmd=Retrieve&db=Protein&list_uids=225735629&dopt=GenPept&RID=T2PYS44A01S&log$=prottop&blast_rank=94) | disco-interacting protein 2A isoform g [Homo sapiens] | [18.0](http://blast.ncbi.nlm.nih.gov/Blast.cgi#225735629%23225735629) | 18.0 | 40% | 647 |
| [NP_001059.2](http://www.ncbi.nlm.nih.gov/entrez/query.fcgi?cmd=Retrieve&db=Protein&list_uids=19913408&dopt=GenPept&RID=T2PYS44A01S&log$=prottop&blast_rank=95) | DNA topoisomerase II, beta isozyme [Homo sapiens] | [18.0](http://blast.ncbi.nlm.nih.gov/Blast.cgi#19913408%2319913408) | 18.0 | 40% | 647 |
| [NP_001005214.2](http://www.ncbi.nlm.nih.gov/entrez/query.fcgi?cmd=Retrieve&db=Protein&list_uids=194018474&dopt=GenPept&RID=T2PYS44A01S&log$=prottop&blast_rank=96) | leucine rich repeat containing 52 precursor [Homo sapiens] | [18.0](http://blast.ncbi.nlm.nih.gov/Blast.cgi#194018474%23194018474) | 18.0 | 40% | 647 |
| [NP_001106205.1](http://www.ncbi.nlm.nih.gov/entrez/query.fcgi?cmd=Retrieve&db=Protein&list_uids=163644333&dopt=GenPept&RID=T2PYS44A01S&log$=prottop&blast_rank=97) | zinc finger protein 397 opposite strand [Homo sapiens] >ref|NP_001159484.1| zinc finger protein 397 opposite strand [Homo sapiens] | [18.0](http://blast.ncbi.nlm.nih.gov/Blast.cgi#163644333%23163644333) | 18.0 | 40% | 647 |
| [NP_996774.1](http://www.ncbi.nlm.nih.gov/entrez/query.fcgi?cmd=Retrieve&db=Protein&list_uids=45827698&dopt=GenPept&RID=T2PYS44A01S&log$=prottop&blast_rank=98) | disco-interacting protein 2A isoform d [Homo sapiens] | [18.0](http://blast.ncbi.nlm.nih.gov/Blast.cgi#45827698%2345827698) | 18.0 | 40% | 647 |
| [NP_443142.1](http://www.ncbi.nlm.nih.gov/entrez/query.fcgi?cmd=Retrieve&db=Protein&list_uids=40217817&dopt=GenPept&RID=T2PYS44A01S&log$=prottop&blast_rank=99) | slit and trk like 1 protein precursor [Homo sapiens] | [18.0](http://blast.ncbi.nlm.nih.gov/Blast.cgi#40217817%2340217817) | 18.0 | 40% | 647 |
| [NP_689801.1](http://www.ncbi.nlm.nih.gov/entrez/query.fcgi?cmd=Retrieve&db=Protein&list_uids=22749211&dopt=GenPept&RID=T2PYS44A01S&log$=prottop&blast_rank=100) | transmembrane and tetratricopeptide repeat containing 2 [Homo sapiens] | [18.0](http://blast.ncbi.nlm.nih.gov/Blast.cgi#22749211%2322749211) | 18.0 | 40% | 647 |

| **Accession** | **Proteins with a match to VNTTSYNMRP peptide** | **[Max score](http://blast.ncbi.nlm.nih.gov/Blast.cgi?CMD=Get&ALIGNMENTS=100&ALIGNMENT_VIEW=Pairwise&CDD_SEARCH_STATE=1&DATABASE_SORT=0&DESCRIPTIONS=100&ENTREZ_QUERY=txid9606 %5BORGN%5D&FIRST_QUERY_NUM=0&FORMAT_OBJECT=Alignment&FORMAT_PAGE_TARGET=&FORMAT_TYPE=HTML&GET_SEQUENCE=yes&I_THRESH=&MASK_CHAR=2&MASK_COLOR=1&NEW_DESIGN=on&NEW_VIEW=yes&NUM_OVERVIEW=100&OLD_BLAST=false&PAGE=Proteins&QUERY_INDEX=0&QUERY_NUMBER=0&RESULTS_PAGE_TARGET=&RID=T2R2S8VP014&SHOW_LINKOUT=yes&SHOW_OVERVIEW=yes&STEP_NUMBER=&WORD_SIZE=2&DISPLAY_SORT=1&HSP_SORT=1" \l "sort_mark)** | **[Total score](http://blast.ncbi.nlm.nih.gov/Blast.cgi?CMD=Get&ALIGNMENTS=100&ALIGNMENT_VIEW=Pairwise&CDD_SEARCH_STATE=1&DATABASE_SORT=0&DESCRIPTIONS=100&ENTREZ_QUERY=txid9606 %5BORGN%5D&FIRST_QUERY_NUM=0&FORMAT_OBJECT=Alignment&FORMAT_PAGE_TARGET=&FORMAT_TYPE=HTML&GET_SEQUENCE=yes&I_THRESH=&MASK_CHAR=2&MASK_COLOR=1&NEW_DESIGN=on&NEW_VIEW=yes&NUM_OVERVIEW=100&OLD_BLAST=false&PAGE=Proteins&QUERY_INDEX=0&QUERY_NUMBER=0&RESULTS_PAGE_TARGET=&RID=T2R2S8VP014&SHOW_LINKOUT=yes&SHOW_OVERVIEW=yes&STEP_NUMBER=&WORD_SIZE=2&DISPLAY_SORT=2&HSP_SORT=1" \l "sort_mark)** | **[Query coverage](http://blast.ncbi.nlm.nih.gov/Blast.cgi?CMD=Get&ALIGNMENTS=100&ALIGNMENT_VIEW=Pairwise&CDD_SEARCH_STATE=1&DATABASE_SORT=0&DESCRIPTIONS=100&ENTREZ_QUERY=txid9606 %5BORGN%5D&FIRST_QUERY_NUM=0&FORMAT_OBJECT=Alignment&FORMAT_PAGE_TARGET=&FORMAT_TYPE=HTML&GET_SEQUENCE=yes&I_THRESH=&MASK_CHAR=2&MASK_COLOR=1&NEW_DESIGN=on&NEW_VIEW=yes&NUM_OVERVIEW=100&OLD_BLAST=false&PAGE=Proteins&QUERY_INDEX=0&QUERY_NUMBER=0&RESULTS_PAGE_TARGET=&RID=T2R2S8VP014&SHOW_LINKOUT=yes&SHOW_OVERVIEW=yes&STEP_NUMBER=&WORD_SIZE=2&DISPLAY_SORT=4&HSP_SORT=0" \l "sort_mark)** | **[E value](http://blast.ncbi.nlm.nih.gov/Blast.cgi?CMD=Get&ALIGNMENTS=100&ALIGNMENT_VIEW=Pairwise&CDD_SEARCH_STATE=1&DATABASE_SORT=0&DESCRIPTIONS=100&ENTREZ_QUERY=txid9606 %5BORGN%5D&FIRST_QUERY_NUM=0&FORMAT_OBJECT=Alignment&FORMAT_PAGE_TARGET=&FORMAT_TYPE=HTML&GET_SEQUENCE=yes&I_THRESH=&MASK_CHAR=2&MASK_COLOR=1&NEW_DESIGN=on&NEW_VIEW=yes&NUM_OVERVIEW=100&OLD_BLAST=false&PAGE=Proteins&QUERY_INDEX=0&QUERY_NUMBER=0&RESULTS_PAGE_TARGET=&RID=T2R2S8VP014&SHOW_LINKOUT=yes&SHOW_OVERVIEW=yes&STEP_NUMBER=&WORD_SIZE=2&DISPLAY_SORT=0&HSP_SORT=0" \l "sort_mark)** |
| --- | --- | --- | --- | --- | --- |
| [NP_001161744.1](http://www.ncbi.nlm.nih.gov/entrez/query.fcgi?cmd=Retrieve&db=Protein&list_uids=269954694&dopt=GenPept&RID=T2R2S8VP014&log$=prottop&blast_rank=1) | inositol 1,4,5-triphosphate receptor, type 1 isoform 3 [Homo sapiens] | [22.3](http://blast.ncbi.nlm.nih.gov/Blast.cgi#269954694%23269954694) | 46.9 | 70% | 34 |
| [NP_001093422.2](http://www.ncbi.nlm.nih.gov/entrez/query.fcgi?cmd=Retrieve&db=Protein&list_uids=269954690&dopt=GenPept&RID=T2R2S8VP014&log$=prottop&blast_rank=2) | inositol 1,4,5-triphosphate receptor, type 1 isoform 1 [Homo sapiens] | [22.3](http://blast.ncbi.nlm.nih.gov/Blast.cgi#269954690%23269954690) | 46.9 | 70% | 34 |
| [NP_002213.5](http://www.ncbi.nlm.nih.gov/entrez/query.fcgi?cmd=Retrieve&db=Protein&list_uids=269954692&dopt=GenPept&RID=T2R2S8VP014&log$=prottop&blast_rank=3) | inositol 1,4,5-triphosphate receptor, type 1 isoform 2 [Homo sapiens] | [22.3](http://blast.ncbi.nlm.nih.gov/Blast.cgi#269954692%23269954692) | 46.9 | 70% | 34 |
| [NP_054721.1](http://www.ncbi.nlm.nih.gov/entrez/query.fcgi?cmd=Retrieve&db=Protein&list_uids=7661946&dopt=GenPept&RID=T2R2S8VP014&log$=prottop&blast_rank=4) | IKK-related kinase epsilon [Homo sapiens] | [22.3](http://blast.ncbi.nlm.nih.gov/Blast.cgi#7661946%237661946) | 22.3 | 90% | 34 |
| [NP_065944.1](http://www.ncbi.nlm.nih.gov/entrez/query.fcgi?cmd=Retrieve&db=Protein&list_uids=55741661&dopt=GenPept&RID=T2R2S8VP014&log$=prottop&blast_rank=5) | hypothetical protein LOC57653 [Homo sapiens] | [21.4](http://blast.ncbi.nlm.nih.gov/Blast.cgi#55741661%2355741661) | 21.4 | 70% | 62 |
| [NP_115656.1](http://www.ncbi.nlm.nih.gov/entrez/query.fcgi?cmd=Retrieve&db=Protein&list_uids=14150027&dopt=GenPept&RID=T2R2S8VP014&log$=prottop&blast_rank=6) | zinc finger, CCHC domain containing 9 [Homo sapiens] >ref|NP_001124507.1| zinc finger, CCHC domain containing 9 [Homo sapiens] >ref|NP_001124508.1| zinc finger, CCHC domain containing 9 [Homo sapiens] | [21.4](http://blast.ncbi.nlm.nih.gov/Blast.cgi#14150027%2314150027) | 21.4 | 100% | 62 |
| [NP_940912.1](http://www.ncbi.nlm.nih.gov/entrez/query.fcgi?cmd=Retrieve&db=Protein&list_uids=38348336&dopt=GenPept&RID=T2R2S8VP014&log$=prottop&blast_rank=7) | inter-alpha (globulin) inhibitor H5-like precursor [Homo sapiens] | [21.0](http://blast.ncbi.nlm.nih.gov/Blast.cgi#38348336%2338348336) | 21.0 | 60% | 83 |
| [NP_005516.1](http://www.ncbi.nlm.nih.gov/entrez/query.fcgi?cmd=Retrieve&db=Protein&list_uids=5031765&dopt=GenPept&RID=T2R2S8VP014&log$=prottop&blast_rank=8) | 11-beta-hydroxysteroid dehydrogenase 1 [Homo sapiens] >ref|NP_861420.1| 11-beta-hydroxysteroid dehydrogenase 1 [Homo sapiens] | [21.0](http://blast.ncbi.nlm.nih.gov/Blast.cgi#5031765%235031765) | 31.0 | 50% | 83 |
| [NP_000712.2](http://www.ncbi.nlm.nih.gov/entrez/query.fcgi?cmd=Retrieve&db=Protein&list_uids=53832005&dopt=GenPept&RID=T2R2S8VP014&log$=prottop&blast_rank=9) | calcium channel, voltage-dependent, R type, alpha 1E subunit [Homo sapiens] | [20.6](http://blast.ncbi.nlm.nih.gov/Blast.cgi#53832005%2353832005) | 20.6 | 80% | 111 |
| [NP_001154826.1](http://www.ncbi.nlm.nih.gov/entrez/query.fcgi?cmd=Retrieve&db=Protein&list_uids=238859653&dopt=GenPept&RID=T2R2S8VP014&log$=prottop&blast_rank=10) | pleckstrin homology domain containing family A member 4 isoform 2 [Homo sapiens] | [19.7](http://blast.ncbi.nlm.nih.gov/Blast.cgi#238859653%23238859653) | 19.7 | 60% | 200 |
| [NP_055448.1](http://www.ncbi.nlm.nih.gov/entrez/query.fcgi?cmd=Retrieve&db=Protein&list_uids=7661950&dopt=GenPept&RID=T2R2S8VP014&log$=prottop&blast_rank=11) | SH2 domain binding protein 1 [Homo sapiens] | [19.7](http://blast.ncbi.nlm.nih.gov/Blast.cgi#7661950%237661950) | 19.7 | 60% | 200 |
| [NP_002151.2](http://www.ncbi.nlm.nih.gov/entrez/query.fcgi?cmd=Retrieve&db=Protein&list_uids=153946395&dopt=GenPept&RID=T2R2S8VP014&log$=prottop&blast_rank=12) | tenascin C precursor [Homo sapiens] | [19.7](http://blast.ncbi.nlm.nih.gov/Blast.cgi#153946395%23153946395) | 47.3 | 90% | 200 |
| [NP_055950.1](http://www.ncbi.nlm.nih.gov/entrez/query.fcgi?cmd=Retrieve&db=Protein&list_uids=57634534&dopt=GenPept&RID=T2R2S8VP014&log$=prottop&blast_rank=13) | nucleoporin 205kDa [Homo sapiens] | [19.7](http://blast.ncbi.nlm.nih.gov/Blast.cgi#57634534%2357634534) | 19.7 | 50% | 200 |
| [NP_000832.1](http://www.ncbi.nlm.nih.gov/entrez/query.fcgi?cmd=Retrieve&db=Protein&list_uids=4504141&dopt=GenPept&RID=T2R2S8VP014&log$=prottop&blast_rank=14) | glutamate receptor, metabotropic 4 precursor [Homo sapiens] | [19.7](http://blast.ncbi.nlm.nih.gov/Blast.cgi#4504141%234504141) | 19.7 | 50% | 200 |
| [NP_065955.2](http://www.ncbi.nlm.nih.gov/entrez/query.fcgi?cmd=Retrieve&db=Protein&list_uids=238859651&dopt=GenPept&RID=T2R2S8VP014&log$=prottop&blast_rank=15) | pleckstrin homology domain containing family A member 4 isoform 1 [Homo sapiens] | [19.7](http://blast.ncbi.nlm.nih.gov/Blast.cgi#238859651%23238859651) | 19.7 | 60% | 200 |
| [NP_002476.2](http://www.ncbi.nlm.nih.gov/entrez/query.fcgi?cmd=Retrieve&db=Protein&list_uids=33356172&dopt=GenPept&RID=T2R2S8VP014&log$=prottop&blast_rank=16) | nibrin [Homo sapiens] | [19.7](http://blast.ncbi.nlm.nih.gov/Blast.cgi#33356172%2333356172) | 19.7 | 60% | 200 |
| [NP_001018196.1](http://www.ncbi.nlm.nih.gov/entrez/query.fcgi?cmd=Retrieve&db=Protein&list_uids=66882524&dopt=GenPept&RID=T2R2S8VP014&log$=prottop&blast_rank=17) | farnesyltransferase, CAAX box, alpha isoform b [Homo sapiens] | [19.3](http://blast.ncbi.nlm.nih.gov/Blast.cgi#66882524%2366882524) | 19.3 | 60% | 268 |
| [NP_001018197.1](http://www.ncbi.nlm.nih.gov/entrez/query.fcgi?cmd=Retrieve&db=Protein&list_uids=66882532&dopt=GenPept&RID=T2R2S8VP014&log$=prottop&blast_rank=18) | farnesyltransferase, CAAX box, alpha isoform c [Homo sapiens] | [19.3](http://blast.ncbi.nlm.nih.gov/Blast.cgi#66882532%2366882532) | 19.3 | 60% | 268 |
| [NP_055201.2](http://www.ncbi.nlm.nih.gov/entrez/query.fcgi?cmd=Retrieve&db=Protein&list_uids=116812588&dopt=GenPept&RID=T2R2S8VP014&log$=prottop&blast_rank=19) | polycystic kidney disease 2-like 2 [Homo sapiens] | [19.3](http://blast.ncbi.nlm.nih.gov/Blast.cgi#116812588%23116812588) | 19.3 | 70% | 268 |
| [NP_536350.2](http://www.ncbi.nlm.nih.gov/entrez/query.fcgi?cmd=Retrieve&db=Protein&list_uids=117938759&dopt=GenPept&RID=T2R2S8VP014&log$=prottop&blast_rank=20) | GNAS complex locus XLas [Homo sapiens] | [19.3](http://blast.ncbi.nlm.nih.gov/Blast.cgi#117938759%23117938759) | 19.3 | 80% | 268 |
| [NP_005059.2](http://www.ncbi.nlm.nih.gov/entrez/query.fcgi?cmd=Retrieve&db=Protein&list_uids=21614542&dopt=GenPept&RID=T2R2S8VP014&log$=prottop&blast_rank=21) | single-minded homolog 1 [Homo sapiens] | [19.3](http://blast.ncbi.nlm.nih.gov/Blast.cgi#21614542%2321614542) | 19.3 | 70% | 268 |
| [NP_002935.2](http://www.ncbi.nlm.nih.gov/entrez/query.fcgi?cmd=Retrieve&db=Protein&list_uids=19924165&dopt=GenPept&RID=T2R2S8VP014&log$=prottop&blast_rank=22) | proto-oncogene c-ros-1 protein precursor [Homo sapiens] | [19.3](http://blast.ncbi.nlm.nih.gov/Blast.cgi#19924165%2319924165) | 19.3 | 60% | 268 |
| [NP_001075145.1](http://www.ncbi.nlm.nih.gov/entrez/query.fcgi?cmd=Retrieve&db=Protein&list_uids=126362947&dopt=GenPept&RID=T2R2S8VP014&log$=prottop&blast_rank=23) | sodium channel, voltage-gated, type III, alpha isoform 2 [Homo sapiens] | [19.3](http://blast.ncbi.nlm.nih.gov/Blast.cgi#126362947%23126362947) | 32.2 | 80% | 268 |
| [NP_008853.3](http://www.ncbi.nlm.nih.gov/entrez/query.fcgi?cmd=Retrieve&db=Protein&list_uids=126362949&dopt=GenPept&RID=T2R2S8VP014&log$=prottop&blast_rank=24) | sodium channel, voltage-gated, type III, alpha isoform 1 [Homo sapiens] | [19.3](http://blast.ncbi.nlm.nih.gov/Blast.cgi#126362949%23126362949) | 32.2 | 80% | 268 |
| [NP_033664.2](http://www.ncbi.nlm.nih.gov/entrez/query.fcgi?cmd=Retrieve&db=Protein&list_uids=194239684&dopt=GenPept&RID=T2R2S8VP014&log$=prottop&blast_rank=25) | single-minded homolog 2 short isoform [Homo sapiens] | [19.3](http://blast.ncbi.nlm.nih.gov/Blast.cgi#194239684%23194239684) | 19.3 | 70% | 268 |
| [NP_006277.1](http://www.ncbi.nlm.nih.gov/entrez/query.fcgi?cmd=Retrieve&db=Protein&list_uids=5454112&dopt=GenPept&RID=T2R2S8VP014&log$=prottop&blast_rank=26) | transcription factor Dp-2 (E2F dimerization partner 2) [Homo sapiens] | [19.3](http://blast.ncbi.nlm.nih.gov/Blast.cgi#5454112%235454112) | 19.3 | 50% | 268 |
| [NP_001032405.1](http://www.ncbi.nlm.nih.gov/entrez/query.fcgi?cmd=Retrieve&db=Protein&list_uids=82659091&dopt=GenPept&RID=T2R2S8VP014&log$=prottop&blast_rank=27) | staufen isoform c [Homo sapiens] | [19.3](http://blast.ncbi.nlm.nih.gov/Blast.cgi#82659091%2382659091) | 19.3 | 70% | 268 |
| [NP_000407.1](http://www.ncbi.nlm.nih.gov/entrez/query.fcgi?cmd=Retrieve&db=Protein&list_uids=4557880&dopt=GenPept&RID=T2R2S8VP014&log$=prottop&blast_rank=28) | interferon gamma receptor 1 precursor [Homo sapiens] | [19.3](http://blast.ncbi.nlm.nih.gov/Blast.cgi#4557880%234557880) | 19.3 | 90% | 268 |
| [NP_001070957.1](http://www.ncbi.nlm.nih.gov/entrez/query.fcgi?cmd=Retrieve&db=Protein&list_uids=117938765&dopt=GenPept&RID=T2R2S8VP014&log$=prottop&blast_rank=29) | GNAS complex locus isoform g [Homo sapiens] | [19.3](http://blast.ncbi.nlm.nih.gov/Blast.cgi#117938765%23117938765) | 19.3 | 80% | 268 |
| [NP_004593.2](http://www.ncbi.nlm.nih.gov/entrez/query.fcgi?cmd=Retrieve&db=Protein&list_uids=82659083&dopt=GenPept&RID=T2R2S8VP014&log$=prottop&blast_rank=30) | staufen isoform a [Homo sapiens] >ref|NP_059346.2| staufen isoform a [Homo sapiens] >ref|NP_059348.2| staufen isoform a [Homo sapiens] | [19.3](http://blast.ncbi.nlm.nih.gov/Blast.cgi#82659083%2382659083) | 19.3 | 70% | 268 |
| [NP_536351.1](http://www.ncbi.nlm.nih.gov/entrez/query.fcgi?cmd=Retrieve&db=Protein&list_uids=18426900&dopt=GenPept&RID=T2R2S8VP014&log$=prottop&blast_rank=31) | GNAS complex locus GNASS [Homo sapiens] | [19.3](http://blast.ncbi.nlm.nih.gov/Blast.cgi#18426900%2318426900) | 19.3 | 80% | 268 |
| [NP_005060.1](http://www.ncbi.nlm.nih.gov/entrez/query.fcgi?cmd=Retrieve&db=Protein&list_uids=4827004&dopt=GenPept&RID=T2R2S8VP014&log$=prottop&blast_rank=32) | single-minded homolog 2 long isoform [Homo sapiens] | [19.3](http://blast.ncbi.nlm.nih.gov/Blast.cgi#4827004%234827004) | 19.3 | 70% | 268 |
| [NP_059347.2](http://www.ncbi.nlm.nih.gov/entrez/query.fcgi?cmd=Retrieve&db=Protein&list_uids=82659087&dopt=GenPept&RID=T2R2S8VP014&log$=prottop&blast_rank=33) | staufen isoform b [Homo sapiens] | [19.3](http://blast.ncbi.nlm.nih.gov/Blast.cgi#82659087%2382659087) | 19.3 | 70% | 268 |
| [NP_000507.1](http://www.ncbi.nlm.nih.gov/entrez/query.fcgi?cmd=Retrieve&db=Protein&list_uids=4504047&dopt=GenPept&RID=T2R2S8VP014&log$=prottop&blast_rank=34) | GNAS complex locus GNASL [Homo sapiens] | [19.3](http://blast.ncbi.nlm.nih.gov/Blast.cgi#4504047%234504047) | 19.3 | 80% | 268 |
| [NP_009042.1](http://www.ncbi.nlm.nih.gov/entrez/query.fcgi?cmd=Retrieve&db=Protein&list_uids=6005900&dopt=GenPept&RID=T2R2S8VP014&log$=prottop&blast_rank=35) | transcription factor Dp-1 [Homo sapiens] | [19.3](http://blast.ncbi.nlm.nih.gov/Blast.cgi#6005900%236005900) | 19.3 | 50% | 268 |
| [NP_001070956.1](http://www.ncbi.nlm.nih.gov/entrez/query.fcgi?cmd=Retrieve&db=Protein&list_uids=117938762&dopt=GenPept&RID=T2R2S8VP014&log$=prottop&blast_rank=36) | GNAS complex locus isoform f [Homo sapiens] | [19.3](http://blast.ncbi.nlm.nih.gov/Blast.cgi#117938762%23117938762) | 19.3 | 80% | 268 |
| [NP_002018.1](http://www.ncbi.nlm.nih.gov/entrez/query.fcgi?cmd=Retrieve&db=Protein&list_uids=4503771&dopt=GenPept&RID=T2R2S8VP014&log$=prottop&blast_rank=37) | farnesyltransferase, CAAX box, alpha isoform a [Homo sapiens] | [19.3](http://blast.ncbi.nlm.nih.gov/Blast.cgi#4503771%234503771) | 19.3 | 60% | 268 |
| [NP_001075146.1](http://www.ncbi.nlm.nih.gov/entrez/query.fcgi?cmd=Retrieve&db=Protein&list_uids=126362955&dopt=GenPept&RID=T2R2S8VP014&log$=prottop&blast_rank=38) | sodium channel, voltage-gated, type III, alpha isoform 3 [Homo sapiens] | [19.3](http://blast.ncbi.nlm.nih.gov/Blast.cgi#126362955%23126362955) | 32.2 | 80% | 268 |
| [XP_001718110.2](http://www.ncbi.nlm.nih.gov/entrez/query.fcgi?cmd=Retrieve&db=Protein&list_uids=239750374&dopt=GenPept&RID=T2R2S8VP014&log$=prottop&blast_rank=39) | PREDICTED: chromosome 12 open reading frame 28 [Homo sapiens] | [18.9](http://blast.ncbi.nlm.nih.gov/Blast.cgi#239750374%23239750374) | 18.9 | 70% | 360 |
| [XP_002344113.1](http://www.ncbi.nlm.nih.gov/entrez/query.fcgi?cmd=Retrieve&db=Protein&list_uids=239740950&dopt=GenPept&RID=T2R2S8VP014&log$=prottop&blast_rank=40) | PREDICTED: similar to mitogen-activated protein kinase 8 interacting protein 1 [Homo sapiens] | [18.9](http://blast.ncbi.nlm.nih.gov/Blast.cgi#239740950%23239740950) | 18.9 | 40% | 360 |
| [NP_996816.2](http://www.ncbi.nlm.nih.gov/entrez/query.fcgi?cmd=Retrieve&db=Protein&list_uids=219842266&dopt=GenPept&RID=T2R2S8VP014&log$=prottop&blast_rank=41) | usherin isoform B [Homo sapiens] | [18.9](http://blast.ncbi.nlm.nih.gov/Blast.cgi#219842266%23219842266) | 30.1 | 100% | 360 |
| [XP_001716702.1](http://www.ncbi.nlm.nih.gov/entrez/query.fcgi?cmd=Retrieve&db=Protein&list_uids=169204830&dopt=GenPept&RID=T2R2S8VP014&log$=prottop&blast_rank=42) | PREDICTED: hypothetical protein LOC196446 [Homo sapiens] | [18.9](http://blast.ncbi.nlm.nih.gov/Blast.cgi#169204830%23169204830) | 18.9 | 70% | 360 |
| [XP_001718960.1](http://www.ncbi.nlm.nih.gov/entrez/query.fcgi?cmd=Retrieve&db=Protein&list_uids=169204180&dopt=GenPept&RID=T2R2S8VP014&log$=prottop&blast_rank=43) | PREDICTED: hypothetical protein LOC196446 [Homo sapiens] | [18.9](http://blast.ncbi.nlm.nih.gov/Blast.cgi#169204180%23169204180) | 18.9 | 70% | 360 |
| [NP_001106271.1](http://www.ncbi.nlm.nih.gov/entrez/query.fcgi?cmd=Retrieve&db=Protein&list_uids=163914369&dopt=GenPept&RID=T2R2S8VP014&log$=prottop&blast_rank=44) | solute carrier family 8 (sodium/calcium exchanger), member 1 isoform B [Homo sapiens] | [18.9](http://blast.ncbi.nlm.nih.gov/Blast.cgi#163914369%23163914369) | 18.9 | 40% | 360 |
| [NP_004516.2](http://www.ncbi.nlm.nih.gov/entrez/query.fcgi?cmd=Retrieve&db=Protein&list_uids=126012573&dopt=GenPept&RID=T2R2S8VP014&log$=prottop&blast_rank=45) | low density lipoprotein-related protein 2 precursor [Homo sapiens] | [18.9](http://blast.ncbi.nlm.nih.gov/Blast.cgi#126012573%23126012573) | 34.4 | 80% | 360 |
| [NP_060229.3](http://www.ncbi.nlm.nih.gov/entrez/query.fcgi?cmd=Retrieve&db=Protein&list_uids=148539591&dopt=GenPept&RID=T2R2S8VP014&log$=prottop&blast_rank=46) | INO80 complex subunit D [Homo sapiens] | [18.9](http://blast.ncbi.nlm.nih.gov/Blast.cgi#148539591%23148539591) | 18.9 | 60% | 360 |
| [NP_001120864.1](http://www.ncbi.nlm.nih.gov/entrez/query.fcgi?cmd=Retrieve&db=Protein&list_uids=188528652&dopt=GenPept&RID=T2R2S8VP014&log$=prottop&blast_rank=47) | myelin gene regulatory factor isoform 2 [Homo sapiens] | [18.9](http://blast.ncbi.nlm.nih.gov/Blast.cgi#188528652%23188528652) | 18.9 | 70% | 360 |
| [NP_060250.2](http://www.ncbi.nlm.nih.gov/entrez/query.fcgi?cmd=Retrieve&db=Protein&list_uids=54112403&dopt=GenPept&RID=T2R2S8VP014&log$=prottop&blast_rank=48) | chromodomain helicase DNA binding protein 7 [Homo sapiens] | [18.9](http://blast.ncbi.nlm.nih.gov/Blast.cgi#54112403%2354112403) | 33.5 | 50% | 360 |
| [NP_127509.1](http://www.ncbi.nlm.nih.gov/entrez/query.fcgi?cmd=Retrieve&db=Protein&list_uids=14702169&dopt=GenPept&RID=T2R2S8VP014&log$=prottop&blast_rank=49) | plasminogen activator, tissue isoform 3 preproprotein [Homo sapiens] | [18.9](http://blast.ncbi.nlm.nih.gov/Blast.cgi#14702169%2314702169) | 18.9 | 70% | 360 |
| [NP_001026.2](http://www.ncbi.nlm.nih.gov/entrez/query.fcgi?cmd=Retrieve&db=Protein&list_uids=112799847&dopt=GenPept&RID=T2R2S8VP014&log$=prottop&blast_rank=50) | cardiac muscle ryanodine receptor [Homo sapiens] | [18.9](http://blast.ncbi.nlm.nih.gov/Blast.cgi#112799847%23112799847) | 33.9 | 100% | 360 |
| [NP_003033.3](http://www.ncbi.nlm.nih.gov/entrez/query.fcgi?cmd=Retrieve&db=Protein&list_uids=188528618&dopt=GenPept&RID=T2R2S8VP014&log$=prottop&blast_rank=51) | solute carrier family 6 (neurotransmitter transporter, GABA), member 1 [Homo sapiens] | [18.9](http://blast.ncbi.nlm.nih.gov/Blast.cgi#188528618%23188528618) | 18.9 | 80% | 360 |
| [NP_002214.2](http://www.ncbi.nlm.nih.gov/entrez/query.fcgi?cmd=Retrieve&db=Protein&list_uids=95147335&dopt=GenPept&RID=T2R2S8VP014&log$=prottop&blast_rank=52) | inositol 1,4,5-triphosphate receptor, type 2 [Homo sapiens] | [18.9](http://blast.ncbi.nlm.nih.gov/Blast.cgi#95147335%2395147335) | 18.9 | 50% | 360 |
| [NP_878253.1](http://www.ncbi.nlm.nih.gov/entrez/query.fcgi?cmd=Retrieve&db=Protein&list_uids=33457301&dopt=GenPept&RID=T2R2S8VP014&log$=prottop&blast_rank=53) | glycerophosphodiester phosphodiesterase domain containing 4 [Homo sapiens] | [18.9](http://blast.ncbi.nlm.nih.gov/Blast.cgi#33457301%2333457301) | 18.9 | 50% | 360 |
| [NP_848647.1](http://www.ncbi.nlm.nih.gov/entrez/query.fcgi?cmd=Retrieve&db=Protein&list_uids=30425524&dopt=GenPept&RID=T2R2S8VP014&log$=prottop&blast_rank=54) | hypothetical protein LOC339669 isoform 2 [Homo sapiens] | [18.9](http://blast.ncbi.nlm.nih.gov/Blast.cgi#30425524%2330425524) | 18.9 | 40% | 360 |
| [NP_777280.1](http://www.ncbi.nlm.nih.gov/entrez/query.fcgi?cmd=Retrieve&db=Protein&list_uids=28178816&dopt=GenPept&RID=T2R2S8VP014&log$=prottop&blast_rank=55) | isocitrate dehydrogenase 3, beta subunit isoform b precursor [Homo sapiens] | [18.9](http://blast.ncbi.nlm.nih.gov/Blast.cgi#28178816%2328178816) | 18.9 | 50% | 360 |
| [NP_008830.2](http://www.ncbi.nlm.nih.gov/entrez/query.fcgi?cmd=Retrieve&db=Protein&list_uids=28178821&dopt=GenPept&RID=T2R2S8VP014&log$=prottop&blast_rank=56) | isocitrate dehydrogenase 3, beta subunit isoform a precursor [Homo sapiens] | [18.9](http://blast.ncbi.nlm.nih.gov/Blast.cgi#28178821%2328178821) | 18.9 | 50% | 360 |
| [NP_008980.1](http://www.ncbi.nlm.nih.gov/entrez/query.fcgi?cmd=Retrieve&db=Protein&list_uids=5921461&dopt=GenPept&RID=T2R2S8VP014&log$=prottop&blast_rank=57) | butyrophilin, subfamily 2, member A1 isoform 1 precursor [Homo sapiens] | [18.9](http://blast.ncbi.nlm.nih.gov/Blast.cgi#5921461%235921461) | 18.9 | 40% | 360 |
| [XP_001719209.1](http://www.ncbi.nlm.nih.gov/entrez/query.fcgi?cmd=Retrieve&db=Protein&list_uids=169211037&dopt=GenPept&RID=T2R2S8VP014&log$=prottop&blast_rank=58) | PREDICTED: similar to mitogen-activated protein kinase 8 interacting protein 1 [Homo sapiens] | [18.9](http://blast.ncbi.nlm.nih.gov/Blast.cgi#169211037%23169211037) | 18.9 | 40% | 360 |
| [NP_001106273.1](http://www.ncbi.nlm.nih.gov/entrez/query.fcgi?cmd=Retrieve&db=Protein&list_uids=163914373&dopt=GenPept&RID=T2R2S8VP014&log$=prottop&blast_rank=59) | solute carrier family 8 (sodium/calcium exchanger), member 1 isoform D [Homo sapiens] | [18.9](http://blast.ncbi.nlm.nih.gov/Blast.cgi#163914373%23163914373) | 18.9 | 40% | 360 |
| [NP_000456.2](http://www.ncbi.nlm.nih.gov/entrez/query.fcgi?cmd=Retrieve&db=Protein&list_uids=157266328&dopt=GenPept&RID=T2R2S8VP014&log$=prottop&blast_rank=60) | BRCA1 associated RING domain 1 [Homo sapiens] | [18.9](http://blast.ncbi.nlm.nih.gov/Blast.cgi#157266328%23157266328) | 36.9 | 100% | 360 |
| [NP_853509.1](http://www.ncbi.nlm.nih.gov/entrez/query.fcgi?cmd=Retrieve&db=Protein&list_uids=31881693&dopt=GenPept&RID=T2R2S8VP014&log$=prottop&blast_rank=61) | butyrophilin, subfamily 2, member A2 isoform b [Homo sapiens] | [18.9](http://blast.ncbi.nlm.nih.gov/Blast.cgi#31881693%2331881693) | 18.9 | 40% | 360 |
| [NP_001106272.1](http://www.ncbi.nlm.nih.gov/entrez/query.fcgi?cmd=Retrieve&db=Protein&list_uids=163914371&dopt=GenPept&RID=T2R2S8VP014&log$=prottop&blast_rank=62) | solute carrier family 8 (sodium/calcium exchanger), member 1 isoform C [Homo sapiens] | [18.9](http://blast.ncbi.nlm.nih.gov/Blast.cgi#163914371%23163914371) | 18.9 | 40% | 360 |
| [NP_037411.1](http://www.ncbi.nlm.nih.gov/entrez/query.fcgi?cmd=Retrieve&db=Protein&list_uids=7019335&dopt=GenPept&RID=T2R2S8VP014&log$=prottop&blast_rank=63) | myelin gene regulatory factor isoform 1 [Homo sapiens] | [18.9](http://blast.ncbi.nlm.nih.gov/Blast.cgi#7019335%237019335) | 18.9 | 70% | 360 |
| [NP_003120.2](http://www.ncbi.nlm.nih.gov/entrez/query.fcgi?cmd=Retrieve&db=Protein&list_uids=62865635&dopt=GenPept&RID=T2R2S8VP014&log$=prottop&blast_rank=64) | squalene epoxidase [Homo sapiens] | [18.9](http://blast.ncbi.nlm.nih.gov/Blast.cgi#62865635%2362865635) | 18.9 | 40% | 360 |
| [NP_817089.1](http://www.ncbi.nlm.nih.gov/entrez/query.fcgi?cmd=Retrieve&db=Protein&list_uids=29570800&dopt=GenPept&RID=T2R2S8VP014&log$=prottop&blast_rank=65) | cadherin-like 26 isoform a [Homo sapiens] | [18.9](http://blast.ncbi.nlm.nih.gov/Blast.cgi#29570800%2329570800) | 18.9 | 70% | 360 |
| [NP_001157329.1](http://www.ncbi.nlm.nih.gov/entrez/query.fcgi?cmd=Retrieve&db=Protein&list_uids=255759947&dopt=GenPept&RID=T2R2S8VP014&log$=prottop&blast_rank=66) | hypothetical protein LOC339669 isoform 1 [Homo sapiens] | [18.9](http://blast.ncbi.nlm.nih.gov/Blast.cgi#255759947%23255759947) | 18.9 | 40% | 360 |
| [NP_861454.2](http://www.ncbi.nlm.nih.gov/entrez/query.fcgi?cmd=Retrieve&db=Protein&list_uids=98986321&dopt=GenPept&RID=T2R2S8VP014&log$=prottop&blast_rank=67) | gliomedin [Homo sapiens] | [18.9](http://blast.ncbi.nlm.nih.gov/Blast.cgi#98986321%2398986321) | 34.4 | 80% | 360 |
| [NP_000921.1](http://www.ncbi.nlm.nih.gov/entrez/query.fcgi?cmd=Retrieve&db=Protein&list_uids=4505861&dopt=GenPept&RID=T2R2S8VP014&log$=prottop&blast_rank=68) | plasminogen activator, tissue isoform 1 preproprotein [Homo sapiens] | [18.9](http://blast.ncbi.nlm.nih.gov/Blast.cgi#4505861%234505861) | 18.9 | 70% | 360 |
| [NP_004995.1](http://www.ncbi.nlm.nih.gov/entrez/query.fcgi?cmd=Retrieve&db=Protein&list_uids=4826854&dopt=GenPept&RID=T2R2S8VP014&log$=prottop&blast_rank=69) | NADH dehydrogenase (ubiquinone) 1 beta subcomplex, 8 precursor [Homo sapiens] | [18.9](http://blast.ncbi.nlm.nih.gov/Blast.cgi#4826854%234826854) | 18.9 | 40% | 360 |
| [NP_001002907.1](http://www.ncbi.nlm.nih.gov/entrez/query.fcgi?cmd=Retrieve&db=Protein&list_uids=50897270&dopt=GenPept&RID=T2R2S8VP014&log$=prottop&blast_rank=70) | olfactory receptor, family 8, subfamily K, member 1 [Homo sapiens] | [18.9](http://blast.ncbi.nlm.nih.gov/Blast.cgi#50897270%2350897270) | 18.9 | 60% | 360 |
| [NP_005447.1](http://www.ncbi.nlm.nih.gov/entrez/query.fcgi?cmd=Retrieve&db=Protein&list_uids=4885433&dopt=GenPept&RID=T2R2S8VP014&log$=prottop&blast_rank=71) | mitogen-activated protein kinase 8 interacting protein 1 [Homo sapiens] | [18.9](http://blast.ncbi.nlm.nih.gov/Blast.cgi#4885433%234885433) | 18.9 | 40% | 360 |
| [NP_149094.3](http://www.ncbi.nlm.nih.gov/entrez/query.fcgi?cmd=Retrieve&db=Protein&list_uids=228008333&dopt=GenPept&RID=T2R2S8VP014&log$=prottop&blast_rank=72) | rhophilin, Rho GTPase binding protein 2 [Homo sapiens] | [18.9](http://blast.ncbi.nlm.nih.gov/Blast.cgi#228008333%23228008333) | 18.9 | 80% | 360 |
| [NP_066920.1](http://www.ncbi.nlm.nih.gov/entrez/query.fcgi?cmd=Retrieve&db=Protein&list_uids=10863913&dopt=GenPept&RID=T2R2S8VP014&log$=prottop&blast_rank=73) | solute carrier family 8 (sodium/calcium exchanger), member 1 isoform A [Homo sapiens] | [18.9](http://blast.ncbi.nlm.nih.gov/Blast.cgi#10863913%2310863913) | 18.9 | 40% | 360 |
| [NP_008926.2](http://www.ncbi.nlm.nih.gov/entrez/query.fcgi?cmd=Retrieve&db=Protein&list_uids=6453813&dopt=GenPept&RID=T2R2S8VP014&log$=prottop&blast_rank=74) | butyrophilin, subfamily 2, member A2 isoform a [Homo sapiens] | [18.9](http://blast.ncbi.nlm.nih.gov/Blast.cgi#6453813%236453813) | 18.9 | 40% | 360 |
| [NP_000040.1](http://www.ncbi.nlm.nih.gov/entrez/query.fcgi?cmd=Retrieve&db=Protein&list_uids=4557335&dopt=GenPept&RID=T2R2S8VP014&log$=prottop&blast_rank=75) | aspartoacylase [Homo sapiens] >ref|NP_001121557.1| aspartoacylase [Homo sapiens] | [18.9](http://blast.ncbi.nlm.nih.gov/Blast.cgi#4557335%234557335) | 18.9 | 70% | 360 |
| [NP_071351.2](http://www.ncbi.nlm.nih.gov/entrez/query.fcgi?cmd=Retrieve&db=Protein&list_uids=257900451&dopt=GenPept&RID=T2R2S8VP014&log$=prottop&blast_rank=76) | family with sequence similarity 38, member B [Homo sapiens] | [18.5](http://blast.ncbi.nlm.nih.gov/Blast.cgi#257900451%23257900451) | 18.5 | 60% | 482 |
| [NP_001124390.1](http://www.ncbi.nlm.nih.gov/entrez/query.fcgi?cmd=Retrieve&db=Protein&list_uids=195963418&dopt=GenPept&RID=T2R2S8VP014&log$=prottop&blast_rank=77) | tubulin tyrosine ligase-like family, member 6 isoform 1 [Homo sapiens] | [18.5](http://blast.ncbi.nlm.nih.gov/Blast.cgi#195963418%23195963418) | 18.5 | 80% | 482 |
| [NP_835469.1](http://www.ncbi.nlm.nih.gov/entrez/query.fcgi?cmd=Retrieve&db=Protein&list_uids=30039704&dopt=GenPept&RID=T2R2S8VP014&log$=prottop&blast_rank=78) | lipoma HMGIC fusion partner-like 1 precursor [Homo sapiens] | [18.5](http://blast.ncbi.nlm.nih.gov/Blast.cgi#30039704%2330039704) | 18.5 | 70% | 482 |
| [NP_055758.1](http://www.ncbi.nlm.nih.gov/entrez/query.fcgi?cmd=Retrieve&db=Protein&list_uids=7662342&dopt=GenPept&RID=T2R2S8VP014&log$=prottop&blast_rank=79) | zinc fingers and homeoboxes 2 [Homo sapiens] | [18.5](http://blast.ncbi.nlm.nih.gov/Blast.cgi#7662342%237662342) | 18.5 | 60% | 482 |
| [XP_002346262.1](http://www.ncbi.nlm.nih.gov/entrez/query.fcgi?cmd=Retrieve&db=Protein&list_uids=239757991&dopt=GenPept&RID=T2R2S8VP014&log$=prottop&blast_rank=80) | PREDICTED: similar to inward rectifying K+ channel negative regulator Kir2.2v isoform 2 [Homo sapiens] | [18.0](http://blast.ncbi.nlm.nih.gov/Blast.cgi#239757991%23239757991) | 18.0 | 80% | 647 |
| [XP_002344378.1](http://www.ncbi.nlm.nih.gov/entrez/query.fcgi?cmd=Retrieve&db=Protein&list_uids=239508877&dopt=GenPept&RID=T2R2S8VP014&log$=prottop&blast_rank=81) | PREDICTED: hypothetical LOC100129126 [Homo sapiens] >ref|XP_001715380.2| PREDICTED: hypothetical LOC100129126 [Homo sapiens] >ref|XP_001715107.2| PREDICTED: hypothetical LOC100129126 [Homo sapiens] >ref|XP_001715529.2| PREDICTED: hypothetical LOC100129126 [Homo sapiens] | [18.0](http://blast.ncbi.nlm.nih.gov/Blast.cgi#239508877%23239508877) | 18.0 | 50% | 647 |
| [NP_001129493.1](http://www.ncbi.nlm.nih.gov/entrez/query.fcgi?cmd=Retrieve&db=Protein&list_uids=209862843&dopt=GenPept&RID=T2R2S8VP014&log$=prottop&blast_rank=82) | nuclear factor of activated T-cells, cytoplasmic, calcineurin-dependent 2 isoform D [Homo sapiens] | [18.0](http://blast.ncbi.nlm.nih.gov/Blast.cgi#209862843%23209862843) | 18.0 | 60% | 647 |
| [NP_001098718.1](http://www.ncbi.nlm.nih.gov/entrez/query.fcgi?cmd=Retrieve&db=Protein&list_uids=157426860&dopt=GenPept&RID=T2R2S8VP014&log$=prottop&blast_rank=83) | transmembrane channel-like 5 isoform a [Homo sapiens] | [18.0](http://blast.ncbi.nlm.nih.gov/Blast.cgi#157426860%23157426860) | 18.0 | 40% | 647 |
| [NP_060264.3](http://www.ncbi.nlm.nih.gov/entrez/query.fcgi?cmd=Retrieve&db=Protein&list_uids=150456451&dopt=GenPept&RID=T2R2S8VP014&log$=prottop&blast_rank=84) | hypothetical protein LOC54914 [Homo sapiens] | [18.0](http://blast.ncbi.nlm.nih.gov/Blast.cgi#150456451%23150456451) | 18.0 | 60% | 647 |
| [NP_001073327.1](http://www.ncbi.nlm.nih.gov/entrez/query.fcgi?cmd=Retrieve&db=Protein&list_uids=119943120&dopt=GenPept&RID=T2R2S8VP014&log$=prottop&blast_rank=85) | G protein-coupled receptor 64 isoform 1 [Homo sapiens] | [18.0](http://blast.ncbi.nlm.nih.gov/Blast.cgi#119943120%23119943120) | 18.0 | 50% | 647 |
| [NP_001073328.1](http://www.ncbi.nlm.nih.gov/entrez/query.fcgi?cmd=Retrieve&db=Protein&list_uids=119943116&dopt=GenPept&RID=T2R2S8VP014&log$=prottop&blast_rank=86) | G protein-coupled receptor 64 isoform 2 [Homo sapiens] | [18.0](http://blast.ncbi.nlm.nih.gov/Blast.cgi#119943116%23119943116) | 18.0 | 50% | 647 |
| [NP_055765.2](http://www.ncbi.nlm.nih.gov/entrez/query.fcgi?cmd=Retrieve&db=Protein&list_uids=182509178&dopt=GenPept&RID=T2R2S8VP014&log$=prottop&blast_rank=87) | zinc finger and BTB domain containing 1 isoform 2 [Homo sapiens] | [18.0](http://blast.ncbi.nlm.nih.gov/Blast.cgi#182509178%23182509178) | 31.8 | 90% | 647 |
| [NP_149115.2](http://www.ncbi.nlm.nih.gov/entrez/query.fcgi?cmd=Retrieve&db=Protein&list_uids=224586875&dopt=GenPept&RID=T2R2S8VP014&log$=prottop&blast_rank=88) | coiled-coil domain containing 65 [Homo sapiens] | [18.0](http://blast.ncbi.nlm.nih.gov/Blast.cgi#224586875%23224586875) | 18.0 | 40% | 647 |
| [NP_056099.2](http://www.ncbi.nlm.nih.gov/entrez/query.fcgi?cmd=Retrieve&db=Protein&list_uids=71999153&dopt=GenPept&RID=T2R2S8VP014&log$=prottop&blast_rank=89) | hypothetical protein LOC23334 [Homo sapiens] | [18.0](http://blast.ncbi.nlm.nih.gov/Blast.cgi#71999153%2371999153) | 28.0 | 60% | 647 |
| [NP_001073329.1](http://www.ncbi.nlm.nih.gov/entrez/query.fcgi?cmd=Retrieve&db=Protein&list_uids=119943125&dopt=GenPept&RID=T2R2S8VP014&log$=prottop&blast_rank=90) | G protein-coupled receptor 64 isoform 3 [Homo sapiens] | [18.0](http://blast.ncbi.nlm.nih.gov/Blast.cgi#119943125%23119943125) | 18.0 | 50% | 647 |
| [NP_005747.2](http://www.ncbi.nlm.nih.gov/entrez/query.fcgi?cmd=Retrieve&db=Protein&list_uids=119943129&dopt=GenPept&RID=T2R2S8VP014&log$=prottop&blast_rank=91) | G protein-coupled receptor 64 isoform 4 [Homo sapiens] | [18.0](http://blast.ncbi.nlm.nih.gov/Blast.cgi#119943129%23119943129) | 18.0 | 50% | 647 |
| [NP_071442.2](http://www.ncbi.nlm.nih.gov/entrez/query.fcgi?cmd=Retrieve&db=Protein&list_uids=148806925&dopt=GenPept&RID=T2R2S8VP014&log$=prottop&blast_rank=92) | EGF, latrophilin and seven transmembrane domain containing 1 precursor [Homo sapiens] | [18.0](http://blast.ncbi.nlm.nih.gov/Blast.cgi#148806925%23148806925) | 18.0 | 40% | 647 |
| [NP_003226.4](http://www.ncbi.nlm.nih.gov/entrez/query.fcgi?cmd=Retrieve&db=Protein&list_uids=55770862&dopt=GenPept&RID=T2R2S8VP014&log$=prottop&blast_rank=93) | thyroglobulin precursor [Homo sapiens] | [18.0](http://blast.ncbi.nlm.nih.gov/Blast.cgi#55770862%2355770862) | 18.0 | 70% | 647 |
| [NP_055692.2](http://www.ncbi.nlm.nih.gov/entrez/query.fcgi?cmd=Retrieve&db=Protein&list_uids=54792138&dopt=GenPept&RID=T2R2S8VP014&log$=prottop&blast_rank=94) | helicase with zinc finger domain [Homo sapiens] | [18.0](http://blast.ncbi.nlm.nih.gov/Blast.cgi#54792138%2354792138) | 18.0 | 50% | 647 |
| [NP_001098719.1](http://www.ncbi.nlm.nih.gov/entrez/query.fcgi?cmd=Retrieve&db=Protein&list_uids=157426862&dopt=GenPept&RID=T2R2S8VP014&log$=prottop&blast_rank=95) | transmembrane channel-like 5 isoform b [Homo sapiens] | [18.0](http://blast.ncbi.nlm.nih.gov/Blast.cgi#157426862%23157426862) | 18.0 | 40% | 647 |
| [NP_001035961.1](http://www.ncbi.nlm.nih.gov/entrez/query.fcgi?cmd=Retrieve&db=Protein&list_uids=110224456&dopt=GenPept&RID=T2R2S8VP014&log$=prottop&blast_rank=96) | solute carrier family 12, member 6 isoform d [Homo sapiens] | [18.0](http://blast.ncbi.nlm.nih.gov/Blast.cgi#110224456%23110224456) | 18.0 | 40% | 647 |
| [NP_001107849.1](http://www.ncbi.nlm.nih.gov/entrez/query.fcgi?cmd=Retrieve&db=Protein&list_uids=167466190&dopt=GenPept&RID=T2R2S8VP014&log$=prottop&blast_rank=97) | forkhead box P3 isoform b [Homo sapiens] | [18.0](http://blast.ncbi.nlm.nih.gov/Blast.cgi#167466190%23167466190) | 18.0 | 40% | 647 |
| [NP_776297.2](http://www.ncbi.nlm.nih.gov/entrez/query.fcgi?cmd=Retrieve&db=Protein&list_uids=55770840&dopt=GenPept&RID=T2R2S8VP014&log$=prottop&blast_rank=98) | chloride channel 3 isoform e [Homo sapiens] | [18.0](http://blast.ncbi.nlm.nih.gov/Blast.cgi#55770840%2355770840) | 18.0 | 70% | 647 |
| [NP_001035962.1](http://www.ncbi.nlm.nih.gov/entrez/query.fcgi?cmd=Retrieve&db=Protein&list_uids=110224458&dopt=GenPept&RID=T2R2S8VP014&log$=prottop&blast_rank=99) | solute carrier family 12, member 6 isoform e [Homo sapiens] | [18.0](http://blast.ncbi.nlm.nih.gov/Blast.cgi#110224458%23110224458) | 18.0 | 40% | 647 |
| [NP_001035959.1](http://www.ncbi.nlm.nih.gov/entrez/query.fcgi?cmd=Retrieve&db=Protein&list_uids=110224452&dopt=GenPept&RID=T2R2S8VP014&log$=prottop&blast_rank=100) | solute carrier family 12, member 6 isoform c [Homo sapiens] >ref|NP_001035960.1| solute carrier family 12, member 6 isoform c [Homo sapiens] | [18.0](http://blast.ncbi.nlm.nih.gov/Blast.cgi#110224452%23110224452) | 18.0 | 40% | 647 |

| **Accession** | **Proteins with a match to LPWSKLSSPS peptide** | **[Max score](http://blast.ncbi.nlm.nih.gov/Blast.cgi?CMD=Get&ALIGNMENTS=100&ALIGNMENT_VIEW=Pairwise&CDD_SEARCH_STATE=1&DATABASE_SORT=0&DESCRIPTIONS=100&ENTREZ_QUERY=txid9606 %5BORGN%5D&FIRST_QUERY_NUM=0&FORMAT_OBJECT=Alignment&FORMAT_PAGE_TARGET=&FORMAT_TYPE=HTML&GET_SEQUENCE=yes&I_THRESH=&MASK_CHAR=2&MASK_COLOR=1&NEW_DESIGN=on&NEW_VIEW=yes&NUM_OVERVIEW=100&OLD_BLAST=false&PAGE=Proteins&QUERY_INDEX=0&QUERY_NUMBER=0&RESULTS_PAGE_TARGET=&RID=T2R8NYZT012&SHOW_LINKOUT=yes&SHOW_OVERVIEW=yes&STEP_NUMBER=&WORD_SIZE=2&DISPLAY_SORT=1&HSP_SORT=1" \l "sort_mark)** | **[Total score](http://blast.ncbi.nlm.nih.gov/Blast.cgi?CMD=Get&ALIGNMENTS=100&ALIGNMENT_VIEW=Pairwise&CDD_SEARCH_STATE=1&DATABASE_SORT=0&DESCRIPTIONS=100&ENTREZ_QUERY=txid9606 %5BORGN%5D&FIRST_QUERY_NUM=0&FORMAT_OBJECT=Alignment&FORMAT_PAGE_TARGET=&FORMAT_TYPE=HTML&GET_SEQUENCE=yes&I_THRESH=&MASK_CHAR=2&MASK_COLOR=1&NEW_DESIGN=on&NEW_VIEW=yes&NUM_OVERVIEW=100&OLD_BLAST=false&PAGE=Proteins&QUERY_INDEX=0&QUERY_NUMBER=0&RESULTS_PAGE_TARGET=&RID=T2R8NYZT012&SHOW_LINKOUT=yes&SHOW_OVERVIEW=yes&STEP_NUMBER=&WORD_SIZE=2&DISPLAY_SORT=2&HSP_SORT=1" \l "sort_mark)** | **[Query coverage](http://blast.ncbi.nlm.nih.gov/Blast.cgi?CMD=Get&ALIGNMENTS=100&ALIGNMENT_VIEW=Pairwise&CDD_SEARCH_STATE=1&DATABASE_SORT=0&DESCRIPTIONS=100&ENTREZ_QUERY=txid9606 %5BORGN%5D&FIRST_QUERY_NUM=0&FORMAT_OBJECT=Alignment&FORMAT_PAGE_TARGET=&FORMAT_TYPE=HTML&GET_SEQUENCE=yes&I_THRESH=&MASK_CHAR=2&MASK_COLOR=1&NEW_DESIGN=on&NEW_VIEW=yes&NUM_OVERVIEW=100&OLD_BLAST=false&PAGE=Proteins&QUERY_INDEX=0&QUERY_NUMBER=0&RESULTS_PAGE_TARGET=&RID=T2R8NYZT012&SHOW_LINKOUT=yes&SHOW_OVERVIEW=yes&STEP_NUMBER=&WORD_SIZE=2&DISPLAY_SORT=4&HSP_SORT=0" \l "sort_mark)** | **[E value](http://blast.ncbi.nlm.nih.gov/Blast.cgi?CMD=Get&ALIGNMENTS=100&ALIGNMENT_VIEW=Pairwise&CDD_SEARCH_STATE=1&DATABASE_SORT=0&DESCRIPTIONS=100&ENTREZ_QUERY=txid9606 %5BORGN%5D&FIRST_QUERY_NUM=0&FORMAT_OBJECT=Alignment&FORMAT_PAGE_TARGET=&FORMAT_TYPE=HTML&GET_SEQUENCE=yes&I_THRESH=&MASK_CHAR=2&MASK_COLOR=1&NEW_DESIGN=on&NEW_VIEW=yes&NUM_OVERVIEW=100&OLD_BLAST=false&PAGE=Proteins&QUERY_INDEX=0&QUERY_NUMBER=0&RESULTS_PAGE_TARGET=&RID=T2R8NYZT012&SHOW_LINKOUT=yes&SHOW_OVERVIEW=yes&STEP_NUMBER=&WORD_SIZE=2&DISPLAY_SORT=0&HSP_SORT=0" \l "sort_mark)** |
| --- | --- | --- | --- | --- | --- |
| [NP_658988.2](http://www.ncbi.nlm.nih.gov/entrez/query.fcgi?cmd=Retrieve&db=Protein&list_uids=146260268&dopt=GenPept&RID=T2R8NYZT012&log$=prottop&blast_rank=1) | Smith-Magenis syndrome chromosome region, candidate 8 [Homo sapiens] | [23.1](http://blast.ncbi.nlm.nih.gov/Blast.cgi#146260268%23146260268) | 23.1 | 60% | 19 |
| [NP_000027.2](http://www.ncbi.nlm.nih.gov/entrez/query.fcgi?cmd=Retrieve&db=Protein&list_uids=289547500&dopt=GenPept&RID=T2R8NYZT012&log$=prottop&blast_rank=2) | adenosine monophosphate deaminase 1 (isoform M) isoform 1 [Homo sapiens] | [22.3](http://blast.ncbi.nlm.nih.gov/Blast.cgi#289547500%23289547500) | 22.3 | 60% | 34 |
| [NP_001136278.1](http://www.ncbi.nlm.nih.gov/entrez/query.fcgi?cmd=Retrieve&db=Protein&list_uids=218563758&dopt=GenPept&RID=T2R8NYZT012&log$=prottop&blast_rank=3) | solute carrier family 6 member 8 isoform 3 [Homo sapiens] | [22.3](http://blast.ncbi.nlm.nih.gov/Blast.cgi#218563758%23218563758) | 37.3 | 90% | 34 |
| [NP_001136277.1](http://www.ncbi.nlm.nih.gov/entrez/query.fcgi?cmd=Retrieve&db=Protein&list_uids=218563756&dopt=GenPept&RID=T2R8NYZT012&log$=prottop&blast_rank=4) | solute carrier family 6 member 8 isoform 2 [Homo sapiens] | [22.3](http://blast.ncbi.nlm.nih.gov/Blast.cgi#218563756%23218563756) | 37.3 | 90% | 34 |
| [NP_001166097.1](http://www.ncbi.nlm.nih.gov/entrez/query.fcgi?cmd=Retrieve&db=Protein&list_uids=289547498&dopt=GenPept&RID=T2R8NYZT012&log$=prottop&blast_rank=5) | adenosine monophosphate deaminase 1 (isoform M) isoform 2 [Homo sapiens] | [22.3](http://blast.ncbi.nlm.nih.gov/Blast.cgi#289547498%23289547498) | 22.3 | 60% | 34 |
| [NP_005620.1](http://www.ncbi.nlm.nih.gov/entrez/query.fcgi?cmd=Retrieve&db=Protein&list_uids=5032097&dopt=GenPept&RID=T2R8NYZT012&log$=prottop&blast_rank=6) | solute carrier family 6 member 8 isoform 1 [Homo sapiens] | [22.3](http://blast.ncbi.nlm.nih.gov/Blast.cgi#5032097%235032097) | 37.3 | 90% | 34 |
| [XP_001722508.2](http://www.ncbi.nlm.nih.gov/entrez/query.fcgi?cmd=Retrieve&db=Protein&list_uids=239753803&dopt=GenPept&RID=T2R8NYZT012&log$=prottop&blast_rank=7) | PREDICTED: hypothetical protein [Homo sapiens] | [21.8](http://blast.ncbi.nlm.nih.gov/Blast.cgi#239753803%23239753803) | 21.8 | 80% | 46 |
| [XP_001720370.2](http://www.ncbi.nlm.nih.gov/entrez/query.fcgi?cmd=Retrieve&db=Protein&list_uids=239742260&dopt=GenPept&RID=T2R8NYZT012&log$=prottop&blast_rank=8) | PREDICTED: hypothetical protein [Homo sapiens] >ref|XP_001721315.2| PREDICTED: hypothetical protein [Homo sapiens] | [21.8](http://blast.ncbi.nlm.nih.gov/Blast.cgi#239742260%23239742260) | 21.8 | 80% | 46 |
| [NP_005520.4](http://www.ncbi.nlm.nih.gov/entrez/query.fcgi?cmd=Retrieve&db=Protein&list_uids=126012571&dopt=GenPept&RID=T2R8NYZT012&log$=prottop&blast_rank=9) | basement membrane-specific heparan sulfate proteoglycan core protein precursor [Homo sapiens] | [21.8](http://blast.ncbi.nlm.nih.gov/Blast.cgi#126012571%23126012571) | 66.6 | 80% | 46 |
| [NP_733796.2](http://www.ncbi.nlm.nih.gov/entrez/query.fcgi?cmd=Retrieve&db=Protein&list_uids=28178843&dopt=GenPept&RID=T2R8NYZT012&log$=prottop&blast_rank=10) | TG-interacting factor isoform a [Homo sapiens] | [21.8](http://blast.ncbi.nlm.nih.gov/Blast.cgi#28178843%2328178843) | 21.8 | 90% | 46 |
| [NP_004843.2](http://www.ncbi.nlm.nih.gov/entrez/query.fcgi?cmd=Retrieve&db=Protein&list_uids=119220564&dopt=GenPept&RID=T2R8NYZT012&log$=prottop&blast_rank=11) | one cut domain, family member 2 [Homo sapiens] | [21.4](http://blast.ncbi.nlm.nih.gov/Blast.cgi#119220564%23119220564) | 21.4 | 70% | 62 |
| [NP_060036.2](http://www.ncbi.nlm.nih.gov/entrez/query.fcgi?cmd=Retrieve&db=Protein&list_uids=21314675&dopt=GenPept&RID=T2R8NYZT012&log$=prottop&blast_rank=12) | kelch domain containing 4 [Homo sapiens] | [21.4](http://blast.ncbi.nlm.nih.gov/Blast.cgi#21314675%2321314675) | 33.5 | 80% | 62 |
| [NP_001073957.1](http://www.ncbi.nlm.nih.gov/entrez/query.fcgi?cmd=Retrieve&db=Protein&list_uids=122937424&dopt=GenPept&RID=T2R8NYZT012&log$=prottop&blast_rank=13) | one cut homeobox 3 [Homo sapiens] | [21.4](http://blast.ncbi.nlm.nih.gov/Blast.cgi#122937424%23122937424) | 21.4 | 70% | 62 |
| [XP_173015.1](http://www.ncbi.nlm.nih.gov/entrez/query.fcgi?cmd=Retrieve&db=Protein&list_uids=22043463&dopt=GenPept&RID=T2R8NYZT012&log$=prottop&blast_rank=14) | PREDICTED: hypothetical protein [Homo sapiens] >ref|XP_950736.1| PREDICTED: hypothetical protein [Homo sapiens] >ref|XP_001725741.1| PREDICTED: hypothetical protein [Homo sapiens] | [21.4](http://blast.ncbi.nlm.nih.gov/Blast.cgi#22043463%2322043463) | 21.4 | 70% | 62 |
| [NP_683765.1](http://www.ncbi.nlm.nih.gov/entrez/query.fcgi?cmd=Retrieve&db=Protein&list_uids=22507376&dopt=GenPept&RID=T2R8NYZT012&log$=prottop&blast_rank=15) | G-protein coupled receptor TG1019 [Homo sapiens] | [21.4](http://blast.ncbi.nlm.nih.gov/Blast.cgi#22507376%2322507376) | 21.4 | 90% | 62 |
| [NP_004489.1](http://www.ncbi.nlm.nih.gov/entrez/query.fcgi?cmd=Retrieve&db=Protein&list_uids=24307887&dopt=GenPept&RID=T2R8NYZT012&log$=prottop&blast_rank=16) | one cut homeobox 1 [Homo sapiens] | [21.4](http://blast.ncbi.nlm.nih.gov/Blast.cgi#24307887%2324307887) | 21.4 | 70% | 62 |
| [NP_001166170.1](http://www.ncbi.nlm.nih.gov/entrez/query.fcgi?cmd=Retrieve&db=Protein&list_uids=289577092&dopt=GenPept&RID=T2R8NYZT012&log$=prottop&blast_rank=17) | peroxisome proliferator-activated receptor gamma, coactivator 1 beta isoform 3 [Homo sapiens] | [21.0](http://blast.ncbi.nlm.nih.gov/Blast.cgi#289577092%23289577092) | 32.2 | 100% | 83 |
| [NP_940869.2](http://www.ncbi.nlm.nih.gov/entrez/query.fcgi?cmd=Retrieve&db=Protein&list_uids=158534055&dopt=GenPept&RID=T2R8NYZT012&log$=prottop&blast_rank=18) | round spermatid basic protein 1-like [Homo sapiens] | [21.0](http://blast.ncbi.nlm.nih.gov/Blast.cgi#158534055%23158534055) | 21.0 | 80% | 83 |
| [NP_004819.2](http://www.ncbi.nlm.nih.gov/entrez/query.fcgi?cmd=Retrieve&db=Protein&list_uids=153945782&dopt=GenPept&RID=T2R8NYZT012&log$=prottop&blast_rank=19) | natural cytotoxicity triggering receptor 2 [Homo sapiens] | [21.0](http://blast.ncbi.nlm.nih.gov/Blast.cgi#153945782%23153945782) | 21.0 | 90% | 83 |
| [NP_060834.2](http://www.ncbi.nlm.nih.gov/entrez/query.fcgi?cmd=Retrieve&db=Protein&list_uids=38683851&dopt=GenPept&RID=T2R8NYZT012&log$=prottop&blast_rank=20) | round spermatid basic protein 1 [Homo sapiens] | [21.0](http://blast.ncbi.nlm.nih.gov/Blast.cgi#38683851%2338683851) | 21.0 | 80% | 83 |
| [NP_573570.3](http://www.ncbi.nlm.nih.gov/entrez/query.fcgi?cmd=Retrieve&db=Protein&list_uids=289577088&dopt=GenPept&RID=T2R8NYZT012&log$=prottop&blast_rank=21) | peroxisome proliferator-activated receptor gamma, coactivator 1 beta isoform 1 [Homo sapiens] | [21.0](http://blast.ncbi.nlm.nih.gov/Blast.cgi#289577088%23289577088) | 32.2 | 100% | 83 |
| [NP_001166169.1](http://www.ncbi.nlm.nih.gov/entrez/query.fcgi?cmd=Retrieve&db=Protein&list_uids=289577090&dopt=GenPept&RID=T2R8NYZT012&log$=prottop&blast_rank=22) | peroxisome proliferator-activated receptor gamma, coactivator 1 beta isoform 2 [Homo sapiens] | [21.0](http://blast.ncbi.nlm.nih.gov/Blast.cgi#289577090%23289577090) | 32.2 | 100% | 83 |
| [NP_065784.1](http://www.ncbi.nlm.nih.gov/entrez/query.fcgi?cmd=Retrieve&db=Protein&list_uids=153792110&dopt=GenPept&RID=T2R8NYZT012&log$=prottop&blast_rank=23) | HEG homolog 1 precursor [Homo sapiens] | [21.0](http://blast.ncbi.nlm.nih.gov/Blast.cgi#153792110%23153792110) | 21.0 | 90% | 83 |
| [NP_001157412.1](http://www.ncbi.nlm.nih.gov/entrez/query.fcgi?cmd=Retrieve&db=Protein&list_uids=255653002&dopt=GenPept&RID=T2R8NYZT012&log$=prottop&blast_rank=24) | liver glycogen phosphorylase isoform 2 [Homo sapiens] | [20.6](http://blast.ncbi.nlm.nih.gov/Blast.cgi#255653002%23255653002) | 20.6 | 50% | 111 |
| [NP_001155134.1](http://www.ncbi.nlm.nih.gov/entrez/query.fcgi?cmd=Retrieve&db=Protein&list_uids=242247257&dopt=GenPept&RID=T2R8NYZT012&log$=prottop&blast_rank=25) | WW and C2 domain containing 1 isoform 2 [Homo sapiens] | [20.6](http://blast.ncbi.nlm.nih.gov/Blast.cgi#242247257%23242247257) | 31.8 | 90% | 111 |
| [XP_002348185.1](http://www.ncbi.nlm.nih.gov/entrez/query.fcgi?cmd=Retrieve&db=Protein&list_uids=239752263&dopt=GenPept&RID=T2R8NYZT012&log$=prottop&blast_rank=26) | PREDICTED: hypothetical protein XP_002348185 [Homo sapiens] | [20.6](http://blast.ncbi.nlm.nih.gov/Blast.cgi#239752263%23239752263) | 20.6 | 50% | 111 |
| [XP_002343134.1](http://www.ncbi.nlm.nih.gov/entrez/query.fcgi?cmd=Retrieve&db=Protein&list_uids=239744362&dopt=GenPept&RID=T2R8NYZT012&log$=prottop&blast_rank=27) | PREDICTED: hypothetical protein XP_002343134 [Homo sapiens] >ref|XP_002347281.1| PREDICTED: hypothetical protein XP_002347281 [Homo sapiens] >ref|XP_002344613.1| PREDICTED: similar to NADH dehydrogenase (ubiquinone) 1 alpha subcomplex, 4 [Homo sapiens] | [20.6](http://blast.ncbi.nlm.nih.gov/Blast.cgi#239744362%23239744362) | 20.6 | 60% | 111 |
| [NP_001155133.1](http://www.ncbi.nlm.nih.gov/entrez/query.fcgi?cmd=Retrieve&db=Protein&list_uids=242247251&dopt=GenPept&RID=T2R8NYZT012&log$=prottop&blast_rank=28) | WW and C2 domain containing 1 isoform 1 [Homo sapiens] | [20.6](http://blast.ncbi.nlm.nih.gov/Blast.cgi#242247251%23242247251) | 31.8 | 90% | 111 |
| [NP_853530.2](http://www.ncbi.nlm.nih.gov/entrez/query.fcgi?cmd=Retrieve&db=Protein&list_uids=148277064&dopt=GenPept&RID=T2R8NYZT012&log$=prottop&blast_rank=29) | cut-like homeobox 1 isoform a [Homo sapiens] | [20.6](http://blast.ncbi.nlm.nih.gov/Blast.cgi#148277064%23148277064) | 66.6 | 90% | 111 |
| [NP_997320.2](http://www.ncbi.nlm.nih.gov/entrez/query.fcgi?cmd=Retrieve&db=Protein&list_uids=198442844&dopt=GenPept&RID=T2R8NYZT012&log$=prottop&blast_rank=30) | dynein, axonemal, heavy chain 10 [Homo sapiens] | [20.6](http://blast.ncbi.nlm.nih.gov/Blast.cgi#198442844%23198442844) | 20.6 | 50% | 111 |
| [NP_056082.2](http://www.ncbi.nlm.nih.gov/entrez/query.fcgi?cmd=Retrieve&db=Protein&list_uids=116517292&dopt=GenPept&RID=T2R8NYZT012&log$=prottop&blast_rank=31) | cut-like 2 [Homo sapiens] | [20.6](http://blast.ncbi.nlm.nih.gov/Blast.cgi#116517292%23116517292) | 69.6 | 90% | 111 |
| [NP_001363.2](http://www.ncbi.nlm.nih.gov/entrez/query.fcgi?cmd=Retrieve&db=Protein&list_uids=114155133&dopt=GenPept&RID=T2R8NYZT012&log$=prottop&blast_rank=32) | dynein, axonemal, heavy chain 9 isoform 2 [Homo sapiens] | [20.6](http://blast.ncbi.nlm.nih.gov/Blast.cgi#114155133%23114155133) | 50.7 | 70% | 111 |
| [NP_001025035.1](http://www.ncbi.nlm.nih.gov/entrez/query.fcgi?cmd=Retrieve&db=Protein&list_uids=71274186&dopt=GenPept&RID=T2R8NYZT012&log$=prottop&blast_rank=33) | hypothetical protein LOC85449 [Homo sapiens] | [20.6](http://blast.ncbi.nlm.nih.gov/Blast.cgi#71274186%2371274186) | 35.2 | 80% | 111 |
| [NP_056053.1](http://www.ncbi.nlm.nih.gov/entrez/query.fcgi?cmd=Retrieve&db=Protein&list_uids=29789058&dopt=GenPept&RID=T2R8NYZT012&log$=prottop&blast_rank=34) | WW and C2 domain containing 1 isoform 3 [Homo sapiens] | [20.6](http://blast.ncbi.nlm.nih.gov/Blast.cgi#29789058%2329789058) | 31.8 | 90% | 111 |
| [NP_078963.2](http://www.ncbi.nlm.nih.gov/entrez/query.fcgi?cmd=Retrieve&db=Protein&list_uids=87196347&dopt=GenPept&RID=T2R8NYZT012&log$=prottop&blast_rank=35) | zinc finger, B-box domain containing [Homo sapiens] | [20.6](http://blast.ncbi.nlm.nih.gov/Blast.cgi#87196347%2387196347) | 37.8 | 50% | 111 |
| [NP_001017995.1](http://www.ncbi.nlm.nih.gov/entrez/query.fcgi?cmd=Retrieve&db=Protein&list_uids=63055059&dopt=GenPept&RID=T2R8NYZT012&log$=prottop&blast_rank=36) | SH3 and PX domains 2B [Homo sapiens] | [20.6](http://blast.ncbi.nlm.nih.gov/Blast.cgi#63055059%2363055059) | 20.6 | 50% | 111 |
| [NP_758857.1](http://www.ncbi.nlm.nih.gov/entrez/query.fcgi?cmd=Retrieve&db=Protein&list_uids=27436996&dopt=GenPept&RID=T2R8NYZT012&log$=prottop&blast_rank=37) | potassium voltage-gated channel, subfamily G, member 4 [Homo sapiens] | [20.6](http://blast.ncbi.nlm.nih.gov/Blast.cgi#27436996%2327436996) | 39.9 | 90% | 111 |
| [NP_002854.3](http://www.ncbi.nlm.nih.gov/entrez/query.fcgi?cmd=Retrieve&db=Protein&list_uids=71037379&dopt=GenPept&RID=T2R8NYZT012&log$=prottop&blast_rank=38) | liver glycogen phosphorylase isoform 1 [Homo sapiens] | [20.6](http://blast.ncbi.nlm.nih.gov/Blast.cgi#71037379%2371037379) | 20.6 | 50% | 111 |
| [NP_060846.2](http://www.ncbi.nlm.nih.gov/entrez/query.fcgi?cmd=Retrieve&db=Protein&list_uids=21361743&dopt=GenPept&RID=T2R8NYZT012&log$=prottop&blast_rank=39) | nipsnap homolog 3B [Homo sapiens] | [20.6](http://blast.ncbi.nlm.nih.gov/Blast.cgi#21361743%2321361743) | 20.6 | 50% | 111 |
| [NP_940972.2](http://www.ncbi.nlm.nih.gov/entrez/query.fcgi?cmd=Retrieve&db=Protein&list_uids=260654089&dopt=GenPept&RID=T2R8NYZT012&log$=prottop&blast_rank=40) | von Willebrand factor C domain containing 2 precursor [Homo sapiens] | [20.6](http://blast.ncbi.nlm.nih.gov/Blast.cgi#260654089%23260654089) | 20.6 | 50% | 111 |
| [NP_001139395.1](http://www.ncbi.nlm.nih.gov/entrez/query.fcgi?cmd=Retrieve&db=Protein&list_uids=225579095&dopt=GenPept&RID=T2R8NYZT012&log$=prottop&blast_rank=41) | S-phase cyclin A-associated protein in the ER isoform b [Homo sapiens] | [20.2](http://blast.ncbi.nlm.nih.gov/Blast.cgi#225579095%23225579095) | 20.2 | 60% | 149 |
| [NP_065894.2](http://www.ncbi.nlm.nih.gov/entrez/query.fcgi?cmd=Retrieve&db=Protein&list_uids=225579092&dopt=GenPept&RID=T2R8NYZT012&log$=prottop&blast_rank=42) | S-phase cyclin A-associated protein in the ER isoform a [Homo sapiens] | [20.2](http://blast.ncbi.nlm.nih.gov/Blast.cgi#225579092%23225579092) | 20.2 | 60% | 149 |
| [NP_543147.2](http://www.ncbi.nlm.nih.gov/entrez/query.fcgi?cmd=Retrieve&db=Protein&list_uids=215598806&dopt=GenPept&RID=T2R8NYZT012&log$=prottop&blast_rank=43) | ankyrin repeat and SOCS box-containing 10 isoform 3 [Homo sapiens] | [20.2](http://blast.ncbi.nlm.nih.gov/Blast.cgi#215598806%23215598806) | 20.2 | 90% | 149 |
| [NP_714912.1](http://www.ncbi.nlm.nih.gov/entrez/query.fcgi?cmd=Retrieve&db=Protein&list_uids=24497440&dopt=GenPept&RID=T2R8NYZT012&log$=prottop&blast_rank=44) | interleukin 12 receptor, beta 1 isoform 2 precursor [Homo sapiens] | [20.2](http://blast.ncbi.nlm.nih.gov/Blast.cgi#24497440%2324497440) | 20.2 | 70% | 149 |
| [NP_001108198.1](http://www.ncbi.nlm.nih.gov/entrez/query.fcgi?cmd=Retrieve&db=Protein&list_uids=168229226&dopt=GenPept&RID=T2R8NYZT012&log$=prottop&blast_rank=45) | proline-rich transmembrane protein 4 [Homo sapiens] | [20.2](http://blast.ncbi.nlm.nih.gov/Blast.cgi#168229226%23168229226) | 62.8 | 80% | 149 |
| [NP_003768.2](http://www.ncbi.nlm.nih.gov/entrez/query.fcgi?cmd=Retrieve&db=Protein&list_uids=51479173&dopt=GenPept&RID=T2R8NYZT012&log$=prottop&blast_rank=46) | dynein, axonemal, heavy chain 11 [Homo sapiens] | [20.2](http://blast.ncbi.nlm.nih.gov/Blast.cgi#51479173%2351479173) | 48.2 | 100% | 149 |
| [NP_065994.1](http://www.ncbi.nlm.nih.gov/entrez/query.fcgi?cmd=Retrieve&db=Protein&list_uids=55749769&dopt=GenPept&RID=T2R8NYZT012&log$=prottop&blast_rank=47) | CWC22 spliceosome-associated protein homolog [Homo sapiens] | [20.2](http://blast.ncbi.nlm.nih.gov/Blast.cgi#55749769%2355749769) | 34.4 | 100% | 149 |
| [NP_001561.3](http://www.ncbi.nlm.nih.gov/entrez/query.fcgi?cmd=Retrieve&db=Protein&list_uids=58530886&dopt=GenPept&RID=T2R8NYZT012&log$=prottop&blast_rank=48) | interleukin-1 receptor-associated kinase 2 [Homo sapiens] | [20.2](http://blast.ncbi.nlm.nih.gov/Blast.cgi#58530886%2358530886) | 35.2 | 70% | 149 |
| [NP_689412.1](http://www.ncbi.nlm.nih.gov/entrez/query.fcgi?cmd=Retrieve&db=Protein&list_uids=23312378&dopt=GenPept&RID=T2R8NYZT012&log$=prottop&blast_rank=49) | protein phosphatase with EF hand calcium-binding domain 1 isoform 3 [Homo sapiens] | [20.2](http://blast.ncbi.nlm.nih.gov/Blast.cgi#23312378%2323312378) | 20.2 | 80% | 149 |
| [NP_689410.1](http://www.ncbi.nlm.nih.gov/entrez/query.fcgi?cmd=Retrieve&db=Protein&list_uids=23312374&dopt=GenPept&RID=T2R8NYZT012&log$=prottop&blast_rank=50) | protein phosphatase with EF hand calcium-binding domain 1 isoform 1b [Homo sapiens] | [20.2](http://blast.ncbi.nlm.nih.gov/Blast.cgi#23312374%2323312374) | 20.2 | 80% | 149 |
| [NP_060621.3](http://www.ncbi.nlm.nih.gov/entrez/query.fcgi?cmd=Retrieve&db=Protein&list_uids=56676335&dopt=GenPept&RID=T2R8NYZT012&log$=prottop&blast_rank=51) | RAP1 interacting factor 1 [Homo sapiens] | [20.2](http://blast.ncbi.nlm.nih.gov/Blast.cgi#56676335%2356676335) | 71.1 | 80% | 149 |
| [NP_006231.2](http://www.ncbi.nlm.nih.gov/entrez/query.fcgi?cmd=Retrieve&db=Protein&list_uids=23312380&dopt=GenPept&RID=T2R8NYZT012&log$=prottop&blast_rank=52) | protein phosphatase with EF hand calcium-binding domain 1 isoform 1 [Homo sapiens] | [20.2](http://blast.ncbi.nlm.nih.gov/Blast.cgi#23312380%2323312380) | 20.2 | 80% | 149 |
| [NP_036552.1](http://www.ncbi.nlm.nih.gov/entrez/query.fcgi?cmd=Retrieve&db=Protein&list_uids=6912630&dopt=GenPept&RID=T2R8NYZT012&log$=prottop&blast_rank=53) | interferon-induced protein with tetratricopeptide repeats 5 [Homo sapiens] | [20.2](http://blast.ncbi.nlm.nih.gov/Blast.cgi#6912630%236912630) | 20.2 | 60% | 149 |
| [NP_005526.1](http://www.ncbi.nlm.nih.gov/entrez/query.fcgi?cmd=Retrieve&db=Protein&list_uids=5031785&dopt=GenPept&RID=T2R8NYZT012&log$=prottop&blast_rank=54) | interleukin 12 receptor, beta 1 isoform 1 precursor [Homo sapiens] | [20.2](http://blast.ncbi.nlm.nih.gov/Blast.cgi#5031785%235031785) | 20.2 | 70% | 149 |
| [NP_858047.2](http://www.ncbi.nlm.nih.gov/entrez/query.fcgi?cmd=Retrieve&db=Protein&list_uids=119874215&dopt=GenPept&RID=T2R8NYZT012&log$=prottop&blast_rank=55) | vacuolar protein sorting 13B isoform 4 [Homo sapiens] | [19.7](http://blast.ncbi.nlm.nih.gov/Blast.cgi#119874215%23119874215) | 19.7 | 70% | 200 |
| [NP_056058.2](http://www.ncbi.nlm.nih.gov/entrez/query.fcgi?cmd=Retrieve&db=Protein&list_uids=35493725&dopt=GenPept&RID=T2R8NYZT012&log$=prottop&blast_rank=56) | vacuolar protein sorting 13B isoform 3 [Homo sapiens] | [19.7](http://blast.ncbi.nlm.nih.gov/Blast.cgi#35493725%2335493725) | 19.7 | 70% | 200 |
| [NP_001073942.1](http://www.ncbi.nlm.nih.gov/entrez/query.fcgi?cmd=Retrieve&db=Protein&list_uids=122937339&dopt=GenPept&RID=T2R8NYZT012&log$=prottop&blast_rank=57) | major facilitator superfamily domain containing 2B [Homo sapiens] | [19.7](http://blast.ncbi.nlm.nih.gov/Blast.cgi#122937339%23122937339) | 19.7 | 60% | 200 |
| [NP_001129965.1](http://www.ncbi.nlm.nih.gov/entrez/query.fcgi?cmd=Retrieve&db=Protein&list_uids=211063479&dopt=GenPept&RID=T2R8NYZT012&log$=prottop&blast_rank=58) | major facilitator superfamily domain containing 2 isoform 1 [Homo sapiens] | [19.7](http://blast.ncbi.nlm.nih.gov/Blast.cgi#211063479%23211063479) | 19.7 | 60% | 200 |
| [NP_620168.1](http://www.ncbi.nlm.nih.gov/entrez/query.fcgi?cmd=Retrieve&db=Protein&list_uids=44888835&dopt=GenPept&RID=T2R8NYZT012&log$=prottop&blast_rank=59) | ATPase, class I, type 8B, member 3 [Homo sapiens] | [19.7](http://blast.ncbi.nlm.nih.gov/Blast.cgi#44888835%2344888835) | 34.4 | 100% | 200 |
| [NP_689777.3](http://www.ncbi.nlm.nih.gov/entrez/query.fcgi?cmd=Retrieve&db=Protein&list_uids=35493701&dopt=GenPept&RID=T2R8NYZT012&log$=prottop&blast_rank=60) | vacuolar protein sorting 13B isoform 1 [Homo sapiens] | [19.7](http://blast.ncbi.nlm.nih.gov/Blast.cgi#35493701%2335493701) | 46.0 | 80% | 200 |
| [NP_060360.3](http://www.ncbi.nlm.nih.gov/entrez/query.fcgi?cmd=Retrieve&db=Protein&list_uids=35493713&dopt=GenPept&RID=T2R8NYZT012&log$=prottop&blast_rank=61) | vacuolar protein sorting 13B isoform 5 [Homo sapiens] | [19.7](http://blast.ncbi.nlm.nih.gov/Blast.cgi#35493713%2335493713) | 46.0 | 80% | 200 |
| [NP_116182.2](http://www.ncbi.nlm.nih.gov/entrez/query.fcgi?cmd=Retrieve&db=Protein&list_uids=42713696&dopt=GenPept&RID=T2R8NYZT012&log$=prottop&blast_rank=62) | major facilitator superfamily domain containing 2 isoform 2 [Homo sapiens] | [19.7](http://blast.ncbi.nlm.nih.gov/Blast.cgi#42713696%2342713696) | 19.7 | 60% | 200 |
| [NP_001157745.1](http://www.ncbi.nlm.nih.gov/entrez/query.fcgi?cmd=Retrieve&db=Protein&list_uids=256017163&dopt=GenPept&RID=T2R8NYZT012&log$=prottop&blast_rank=63) | MAX-interacting protein isoform 1 [Homo sapiens] | [19.3](http://blast.ncbi.nlm.nih.gov/Blast.cgi#256017163%23256017163) | 48.6 | 100% | 268 |
| [NP_001074010.2](http://www.ncbi.nlm.nih.gov/entrez/query.fcgi?cmd=Retrieve&db=Protein&list_uids=256017159&dopt=GenPept&RID=T2R8NYZT012&log$=prottop&blast_rank=64) | MAX-interacting protein isoform 2 [Homo sapiens] | [19.3](http://blast.ncbi.nlm.nih.gov/Blast.cgi#256017159%23256017159) | 48.6 | 100% | 268 |
| [XP_002344625.1](http://www.ncbi.nlm.nih.gov/entrez/query.fcgi?cmd=Retrieve&db=Protein&list_uids=239755573&dopt=GenPept&RID=T2R8NYZT012&log$=prottop&blast_rank=65) | PREDICTED: similar to ectonucleotide pyrophosphatase/phosphodiesterase 5 (putative function) [Homo sapiens] | [19.3](http://blast.ncbi.nlm.nih.gov/Blast.cgi#239755573%23239755573) | 31.4 | 100% | 268 |
| [XP_001716104.1](http://www.ncbi.nlm.nih.gov/entrez/query.fcgi?cmd=Retrieve&db=Protein&list_uids=169168282&dopt=GenPept&RID=T2R8NYZT012&log$=prottop&blast_rank=66) | PREDICTED: hypothetical protein [Homo sapiens] | [19.3](http://blast.ncbi.nlm.nih.gov/Blast.cgi#169168282%23169168282) | 38.6 | 100% | 268 |
| [XP_001716843.1](http://www.ncbi.nlm.nih.gov/entrez/query.fcgi?cmd=Retrieve&db=Protein&list_uids=169168103&dopt=GenPept&RID=T2R8NYZT012&log$=prottop&blast_rank=67) | PREDICTED: hypothetical protein [Homo sapiens] | [19.3](http://blast.ncbi.nlm.nih.gov/Blast.cgi#169168103%23169168103) | 38.6 | 100% | 268 |
| [XP_001716057.1](http://www.ncbi.nlm.nih.gov/entrez/query.fcgi?cmd=Retrieve&db=Protein&list_uids=169167521&dopt=GenPept&RID=T2R8NYZT012&log$=prottop&blast_rank=68) | PREDICTED: hypothetical protein [Homo sapiens] | [19.3](http://blast.ncbi.nlm.nih.gov/Blast.cgi#169167521%23169167521) | 19.3 | 60% | 268 |
| [NP_775760.3](http://www.ncbi.nlm.nih.gov/entrez/query.fcgi?cmd=Retrieve&db=Protein&list_uids=154240671&dopt=GenPept&RID=T2R8NYZT012&log$=prottop&blast_rank=69) | HEAT repeat family member 7B2 [Homo sapiens] | [19.3](http://blast.ncbi.nlm.nih.gov/Blast.cgi#154240671%23154240671) | 48.6 | 80% | 268 |
| [XP_001129377.1](http://www.ncbi.nlm.nih.gov/entrez/query.fcgi?cmd=Retrieve&db=Protein&list_uids=113429326&dopt=GenPept&RID=T2R8NYZT012&log$=prottop&blast_rank=70) | PREDICTED: gamma-glutamyltransferase 2 isoform 1 [Homo sapiens] | [19.3](http://blast.ncbi.nlm.nih.gov/Blast.cgi#113429326%23113429326) | 31.8 | 100% | 268 |
| [XP_001129425.1](http://www.ncbi.nlm.nih.gov/entrez/query.fcgi?cmd=Retrieve&db=Protein&list_uids=113429324&dopt=GenPept&RID=T2R8NYZT012&log$=prottop&blast_rank=71) | PREDICTED: gamma-glutamyltransferase 2 isoform 3 [Homo sapiens] | [19.3](http://blast.ncbi.nlm.nih.gov/Blast.cgi#113429324%23113429324) | 31.8 | 100% | 268 |
| [NP_005256.2](http://www.ncbi.nlm.nih.gov/entrez/query.fcgi?cmd=Retrieve&db=Protein&list_uids=73915090&dopt=GenPept&RID=T2R8NYZT012&log$=prottop&blast_rank=72) | gamma-glutamyltransferase 1 precursor [Homo sapiens] >ref|NP_038347.2| gamma-glutamyltransferase 1 precursor [Homo sapiens] >ref|NP_001027536.1| gamma-glutamyltransferase 1 precursor [Homo sapiens] >ref|NP_001027537.1| gamma-glutamyltransferase 1 precursor [Homo sapiens] | [19.3](http://blast.ncbi.nlm.nih.gov/Blast.cgi#73915090%2373915090) | 46.0 | 100% | 268 |
| [NP_001129396.1](http://www.ncbi.nlm.nih.gov/entrez/query.fcgi?cmd=Retrieve&db=Protein&list_uids=209571555&dopt=GenPept&RID=T2R8NYZT012&log$=prottop&blast_rank=73) | von Willebrand factor D and EGF domains precursor [Homo sapiens] | [19.3](http://blast.ncbi.nlm.nih.gov/Blast.cgi#209571555%23209571555) | 19.3 | 60% | 268 |
| [NP_757374.1](http://www.ncbi.nlm.nih.gov/entrez/query.fcgi?cmd=Retrieve&db=Protein&list_uids=27437051&dopt=GenPept&RID=T2R8NYZT012&log$=prottop&blast_rank=74) | colony stimulating factor 3 isoform c [Homo sapiens] | [19.3](http://blast.ncbi.nlm.nih.gov/Blast.cgi#27437051%2327437051) | 19.3 | 90% | 268 |
| [NP_000338.3](http://www.ncbi.nlm.nih.gov/entrez/query.fcgi?cmd=Retrieve&db=Protein&list_uids=67782319&dopt=GenPept&RID=T2R8NYZT012&log$=prottop&blast_rank=75) | spectrin beta isoform b [Homo sapiens] | [19.3](http://blast.ncbi.nlm.nih.gov/Blast.cgi#67782319%2367782319) | 19.3 | 60% | 268 |
| [NP_579889.1](http://www.ncbi.nlm.nih.gov/entrez/query.fcgi?cmd=Retrieve&db=Protein&list_uids=19913356&dopt=GenPept&RID=T2R8NYZT012&log$=prottop&blast_rank=76) | Wolf-Hirschhorn syndrome candidate 1 protein isoform 3 [Homo sapiens] | [19.3](http://blast.ncbi.nlm.nih.gov/Blast.cgi#19913356%2319913356) | 49.0 | 80% | 268 |
| [NP_015627.1](http://www.ncbi.nlm.nih.gov/entrez/query.fcgi?cmd=Retrieve&db=Protein&list_uids=6594683&dopt=GenPept&RID=T2R8NYZT012&log$=prottop&blast_rank=77) | Wolf-Hirschhorn syndrome candidate 1 protein isoform 4 [Homo sapiens] | [19.3](http://blast.ncbi.nlm.nih.gov/Blast.cgi#6594683%236594683) | 49.0 | 80% | 268 |
| [NP_579877.1](http://www.ncbi.nlm.nih.gov/entrez/query.fcgi?cmd=Retrieve&db=Protein&list_uids=19913348&dopt=GenPept&RID=T2R8NYZT012&log$=prottop&blast_rank=78) | Wolf-Hirschhorn syndrome candidate 1 protein isoform 1 [Homo sapiens] >ref|NP_579878.1| Wolf-Hirschhorn syndrome candidate 1 protein isoform 1 [Homo sapiens] >ref|NP_579890.1| Wolf-Hirschhorn syndrome candidate 1 protein isoform 1 [Homo sapiens] >ref|NP_001035889.1| Wolf-Hirschhorn syndrome candidate 1 protein isoform 1 [Homo sapiens] | [19.3](http://blast.ncbi.nlm.nih.gov/Blast.cgi#19913348%2319913348) | 49.0 | 80% | 268 |
| [NP_000750.1](http://www.ncbi.nlm.nih.gov/entrez/query.fcgi?cmd=Retrieve&db=Protein&list_uids=4503079&dopt=GenPept&RID=T2R8NYZT012&log$=prottop&blast_rank=79) | colony stimulating factor 3 isoform a precursor [Homo sapiens] | [19.3](http://blast.ncbi.nlm.nih.gov/Blast.cgi#4503079%234503079) | 19.3 | 90% | 268 |
| [NP_757373.1](http://www.ncbi.nlm.nih.gov/entrez/query.fcgi?cmd=Retrieve&db=Protein&list_uids=27437049&dopt=GenPept&RID=T2R8NYZT012&log$=prottop&blast_rank=80) | colony stimulating factor 3 isoform b precursor [Homo sapiens] | [19.3](http://blast.ncbi.nlm.nih.gov/Blast.cgi#27437049%2327437049) | 19.3 | 90% | 268 |
| [NP_079121.1](http://www.ncbi.nlm.nih.gov/entrez/query.fcgi?cmd=Retrieve&db=Protein&list_uids=13376261&dopt=GenPept&RID=T2R8NYZT012&log$=prottop&blast_rank=81) | N-acetyltransferase 15 [Homo sapiens] >ref|NP_001077069.1| N-acetyltransferase 15 [Homo sapiens] >ref|NP_001077070.1| N-acetyltransferase 15 [Homo sapiens] | [19.3](http://blast.ncbi.nlm.nih.gov/Blast.cgi#13376261%2313376261) | 19.3 | 80% | 268 |
| [XP_002347269.1](http://www.ncbi.nlm.nih.gov/entrez/query.fcgi?cmd=Retrieve&db=Protein&list_uids=239750067&dopt=GenPept&RID=T2R8NYZT012&log$=prottop&blast_rank=82) | PREDICTED: hypothetical protein XP_002347269 [Homo sapiens] | [18.9](http://blast.ncbi.nlm.nih.gov/Blast.cgi#239750067%23239750067) | 18.9 | 90% | 360 |
| [XP_002348119.1](http://www.ncbi.nlm.nih.gov/entrez/query.fcgi?cmd=Retrieve&db=Protein&list_uids=239747649&dopt=GenPept&RID=T2R8NYZT012&log$=prottop&blast_rank=83) | PREDICTED: similar to Putative uncharacterized protein FLJ44672 [Homo sapiens] | [18.9](http://blast.ncbi.nlm.nih.gov/Blast.cgi#239747649%23239747649) | 48.2 | 100% | 360 |
| [XP_001714475.2](http://www.ncbi.nlm.nih.gov/entrez/query.fcgi?cmd=Retrieve&db=Protein&list_uids=239744229&dopt=GenPept&RID=T2R8NYZT012&log$=prottop&blast_rank=84) | PREDICTED: similar to hCG2038970 [Homo sapiens] | [18.9](http://blast.ncbi.nlm.nih.gov/Blast.cgi#239744229%23239744229) | 33.1 | 80% | 360 |
| [XP_002342948.1](http://www.ncbi.nlm.nih.gov/entrez/query.fcgi?cmd=Retrieve&db=Protein&list_uids=239743680&dopt=GenPept&RID=T2R8NYZT012&log$=prottop&blast_rank=85) | PREDICTED: similar to selenoprotein T [Homo sapiens] >ref|XP_002347091.1| PREDICTED: similar to selenoprotein T [Homo sapiens] >ref|XP_002346187.1| PREDICTED: similar to selenoprotein T [Homo sapiens] | [18.9](http://blast.ncbi.nlm.nih.gov/Blast.cgi#239743680%23239743680) | 18.9 | 80% | 360 |
| [NP_001364.1](http://www.ncbi.nlm.nih.gov/entrez/query.fcgi?cmd=Retrieve&db=Protein&list_uids=223555935&dopt=GenPept&RID=T2R8NYZT012&log$=prottop&blast_rank=86) | dynein, axonemal, heavy polypeptide 14 isoform 1 [Homo sapiens] | [18.9](http://blast.ncbi.nlm.nih.gov/Blast.cgi#223555935%23223555935) | 33.1 | 70% | 360 |
| [XP_001717442.1](http://www.ncbi.nlm.nih.gov/entrez/query.fcgi?cmd=Retrieve&db=Protein&list_uids=169201839&dopt=GenPept&RID=T2R8NYZT012&log$=prottop&blast_rank=87) | PREDICTED: similar to hCG2038970 [Homo sapiens] | [18.9](http://blast.ncbi.nlm.nih.gov/Blast.cgi#169201839%23169201839) | 33.1 | 80% | 360 |
| [XP_001719271.1](http://www.ncbi.nlm.nih.gov/entrez/query.fcgi?cmd=Retrieve&db=Protein&list_uids=169201482&dopt=GenPept&RID=T2R8NYZT012&log$=prottop&blast_rank=88) | PREDICTED: similar to hCG2038970 [Homo sapiens] | [18.9](http://blast.ncbi.nlm.nih.gov/Blast.cgi#169201482%23169201482) | 33.1 | 80% | 360 |
| [XP_001716482.1](http://www.ncbi.nlm.nih.gov/entrez/query.fcgi?cmd=Retrieve&db=Protein&list_uids=169169009&dopt=GenPept&RID=T2R8NYZT012&log$=prottop&blast_rank=89) | PREDICTED: hypothetical protein [Homo sapiens] | [18.9](http://blast.ncbi.nlm.nih.gov/Blast.cgi#169169009%23169169009) | 18.9 | 60% | 360 |
| [NP_059995.2](http://www.ncbi.nlm.nih.gov/entrez/query.fcgi?cmd=Retrieve&db=Protein&list_uids=156766068&dopt=GenPept&RID=T2R8NYZT012&log$=prottop&blast_rank=90) | CDC42 binding protein kinase gamma (DMPK-like) [Homo sapiens] | [18.9](http://blast.ncbi.nlm.nih.gov/Blast.cgi#156766068%23156766068) | 47.3 | 90% | 360 |
| [NP_001073992.1](http://www.ncbi.nlm.nih.gov/entrez/query.fcgi?cmd=Retrieve&db=Protein&list_uids=122937478&dopt=GenPept&RID=T2R8NYZT012&log$=prottop&blast_rank=91) | arrestin domain containing 5 [Homo sapiens] | [18.9](http://blast.ncbi.nlm.nih.gov/Blast.cgi#122937478%23122937478) | 18.9 | 80% | 360 |
| [NP_112567.2](http://www.ncbi.nlm.nih.gov/entrez/query.fcgi?cmd=Retrieve&db=Protein&list_uids=94421471&dopt=GenPept&RID=T2R8NYZT012&log$=prottop&blast_rank=92) | ring finger protein 17 [Homo sapiens] | [18.9](http://blast.ncbi.nlm.nih.gov/Blast.cgi#94421471%2394421471) | 18.9 | 60% | 360 |
| [NP_075463.2](http://www.ncbi.nlm.nih.gov/entrez/query.fcgi?cmd=Retrieve&db=Protein&list_uids=117606355&dopt=GenPept&RID=T2R8NYZT012&log$=prottop&blast_rank=93) | furry homolog [Homo sapiens] | [18.9](http://blast.ncbi.nlm.nih.gov/Blast.cgi#117606355%23117606355) | 18.9 | 100% | 360 |
| [NP_079452.3](http://www.ncbi.nlm.nih.gov/entrez/query.fcgi?cmd=Retrieve&db=Protein&list_uids=55749678&dopt=GenPept&RID=T2R8NYZT012&log$=prottop&blast_rank=94) | ninein-like [Homo sapiens] | [18.9](http://blast.ncbi.nlm.nih.gov/Blast.cgi#55749678%2355749678) | 18.9 | 60% | 360 |
| [NP_003398.1](http://www.ncbi.nlm.nih.gov/entrez/query.fcgi?cmd=Retrieve&db=Protein&list_uids=4507961&dopt=GenPept&RID=T2R8NYZT012&log$=prottop&blast_rank=95) | zinc finger protein 36, C3H type, homolog [Homo sapiens] | [18.9](http://blast.ncbi.nlm.nih.gov/Blast.cgi#4507961%234507961) | 18.9 | 90% | 360 |
| [NP_057359.2](http://www.ncbi.nlm.nih.gov/entrez/query.fcgi?cmd=Retrieve&db=Protein&list_uids=42789380&dopt=GenPept&RID=T2R8NYZT012&log$=prottop&blast_rank=96) | selenoprotein T precursor [Homo sapiens] | [18.9](http://blast.ncbi.nlm.nih.gov/Blast.cgi#42789380%2342789380) | 18.9 | 80% | 360 |
| [NP_000177.2](http://www.ncbi.nlm.nih.gov/entrez/query.fcgi?cmd=Retrieve&db=Protein&list_uids=62739186&dopt=GenPept&RID=T2R8NYZT012&log$=prottop&blast_rank=97) | complement factor H isoform a precursor [Homo sapiens] | [18.9](http://blast.ncbi.nlm.nih.gov/Blast.cgi#62739186%2362739186) | 30.1 | 80% | 360 |
| [NP_061918.3](http://www.ncbi.nlm.nih.gov/entrez/query.fcgi?cmd=Retrieve&db=Protein&list_uids=45238858&dopt=GenPept&RID=T2R8NYZT012&log$=prottop&blast_rank=98) | WD repeat domain 44 protein [Homo sapiens] | [18.9](http://blast.ncbi.nlm.nih.gov/Blast.cgi#45238858%2345238858) | 18.9 | 80% | 360 |
| [NP_002401.1](http://www.ncbi.nlm.nih.gov/entrez/query.fcgi?cmd=Retrieve&db=Protein&list_uids=4505167&dopt=GenPept&RID=T2R8NYZT012&log$=prottop&blast_rank=99) | N-acetylglucosaminyltransferase V [Homo sapiens] | [18.9](http://blast.ncbi.nlm.nih.gov/Blast.cgi#4505167%234505167) | 29.7 | 70% | 360 |
| [NP_067653.3](http://www.ncbi.nlm.nih.gov/entrez/query.fcgi?cmd=Retrieve&db=Protein&list_uids=145275185&dopt=GenPept&RID=T2R8NYZT012&log$=prottop&blast_rank=100) | MYG1 protein precursor [Homo sapiens] | [12.5](http://blast.ncbi.nlm.nih.gov/Blast.cgi#145275185%23145275185) | 12.5 | 70% | 29577 |

| **Accession** | **Proteins with a match to SNVKNYMAIP peptide** | **[Max score](http://blast.ncbi.nlm.nih.gov/Blast.cgi?CMD=Get&ALIGNMENTS=100&ALIGNMENT_VIEW=Pairwise&CDD_SEARCH_STATE=1&DATABASE_SORT=0&DESCRIPTIONS=100&ENTREZ_QUERY=txid9606 %5BORGN%5D&FIRST_QUERY_NUM=0&FORMAT_OBJECT=Alignment&FORMAT_PAGE_TARGET=&FORMAT_TYPE=HTML&GET_SEQUENCE=yes&I_THRESH=&MASK_CHAR=2&MASK_COLOR=1&NEW_DESIGN=on&NEW_VIEW=yes&NUM_OVERVIEW=100&OLD_BLAST=false&PAGE=Proteins&QUERY_INDEX=0&QUERY_NUMBER=0&RESULTS_PAGE_TARGET=&RID=T2RDE0YZ01N&SHOW_LINKOUT=yes&SHOW_OVERVIEW=yes&STEP_NUMBER=&WORD_SIZE=2&DISPLAY_SORT=1&HSP_SORT=1" \l "sort_mark)** | **[Total score](http://blast.ncbi.nlm.nih.gov/Blast.cgi?CMD=Get&ALIGNMENTS=100&ALIGNMENT_VIEW=Pairwise&CDD_SEARCH_STATE=1&DATABASE_SORT=0&DESCRIPTIONS=100&ENTREZ_QUERY=txid9606 %5BORGN%5D&FIRST_QUERY_NUM=0&FORMAT_OBJECT=Alignment&FORMAT_PAGE_TARGET=&FORMAT_TYPE=HTML&GET_SEQUENCE=yes&I_THRESH=&MASK_CHAR=2&MASK_COLOR=1&NEW_DESIGN=on&NEW_VIEW=yes&NUM_OVERVIEW=100&OLD_BLAST=false&PAGE=Proteins&QUERY_INDEX=0&QUERY_NUMBER=0&RESULTS_PAGE_TARGET=&RID=T2RDE0YZ01N&SHOW_LINKOUT=yes&SHOW_OVERVIEW=yes&STEP_NUMBER=&WORD_SIZE=2&DISPLAY_SORT=2&HSP_SORT=1" \l "sort_mark)** | **[Query coverage](http://blast.ncbi.nlm.nih.gov/Blast.cgi?CMD=Get&ALIGNMENTS=100&ALIGNMENT_VIEW=Pairwise&CDD_SEARCH_STATE=1&DATABASE_SORT=0&DESCRIPTIONS=100&ENTREZ_QUERY=txid9606 %5BORGN%5D&FIRST_QUERY_NUM=0&FORMAT_OBJECT=Alignment&FORMAT_PAGE_TARGET=&FORMAT_TYPE=HTML&GET_SEQUENCE=yes&I_THRESH=&MASK_CHAR=2&MASK_COLOR=1&NEW_DESIGN=on&NEW_VIEW=yes&NUM_OVERVIEW=100&OLD_BLAST=false&PAGE=Proteins&QUERY_INDEX=0&QUERY_NUMBER=0&RESULTS_PAGE_TARGET=&RID=T2RDE0YZ01N&SHOW_LINKOUT=yes&SHOW_OVERVIEW=yes&STEP_NUMBER=&WORD_SIZE=2&DISPLAY_SORT=4&HSP_SORT=0" \l "sort_mark)** | **[E value](http://blast.ncbi.nlm.nih.gov/Blast.cgi?CMD=Get&ALIGNMENTS=100&ALIGNMENT_VIEW=Pairwise&CDD_SEARCH_STATE=1&DATABASE_SORT=0&DESCRIPTIONS=100&ENTREZ_QUERY=txid9606 %5BORGN%5D&FIRST_QUERY_NUM=0&FORMAT_OBJECT=Alignment&FORMAT_PAGE_TARGET=&FORMAT_TYPE=HTML&GET_SEQUENCE=yes&I_THRESH=&MASK_CHAR=2&MASK_COLOR=1&NEW_DESIGN=on&NEW_VIEW=yes&NUM_OVERVIEW=100&OLD_BLAST=false&PAGE=Proteins&QUERY_INDEX=0&QUERY_NUMBER=0&RESULTS_PAGE_TARGET=&RID=T2RDE0YZ01N&SHOW_LINKOUT=yes&SHOW_OVERVIEW=yes&STEP_NUMBER=&WORD_SIZE=2&DISPLAY_SORT=0&HSP_SORT=0" \l "sort_mark)** |
| --- | --- | --- | --- | --- | --- |
| [NP_001036148.2](http://www.ncbi.nlm.nih.gov/entrez/query.fcgi?cmd=Retrieve&db=Protein&list_uids=289547541&dopt=GenPept&RID=T2RDE0YZ01N&log$=prottop&blast_rank=1) | SNF2 histone linker PHD RING helicase isoform a [Homo sapiens] | [22.3](http://blast.ncbi.nlm.nih.gov/Blast.cgi#289547541%23289547541) | 38.2 | 90% | 34 |
| [NP_775105.1](http://www.ncbi.nlm.nih.gov/entrez/query.fcgi?cmd=Retrieve&db=Protein&list_uids=27436873&dopt=GenPept&RID=T2RDE0YZ01N&log$=prottop&blast_rank=2) | SNF2 histone linker PHD RING helicase isoform b [Homo sapiens] | [22.3](http://blast.ncbi.nlm.nih.gov/Blast.cgi#27436873%2327436873) | 38.2 | 90% | 34 |
| [NP_075043.1](http://www.ncbi.nlm.nih.gov/entrez/query.fcgi?cmd=Retrieve&db=Protein&list_uids=119393876&dopt=GenPept&RID=T2RDE0YZ01N&log$=prottop&blast_rank=3) | NLR family, apoptosis inhibitory protein isoform 2 [Homo sapiens] | [21.4](http://blast.ncbi.nlm.nih.gov/Blast.cgi#119393876%23119393876) | 21.4 | 60% | 62 |
| [NP_004527.2](http://www.ncbi.nlm.nih.gov/entrez/query.fcgi?cmd=Retrieve&db=Protein&list_uids=119393878&dopt=GenPept&RID=T2RDE0YZ01N&log$=prottop&blast_rank=4) | NLR family, apoptosis inhibitory protein isoform 1 [Homo sapiens] | [21.4](http://blast.ncbi.nlm.nih.gov/Blast.cgi#119393878%23119393878) | 21.4 | 60% | 62 |
| [NP_663719.1](http://www.ncbi.nlm.nih.gov/entrez/query.fcgi?cmd=Retrieve&db=Protein&list_uids=22035604&dopt=GenPept&RID=T2RDE0YZ01N&log$=prottop&blast_rank=5) | mitogen-activated protein kinase kinase kinase kinase 4 isoform 2 [Homo sapiens] | [21.4](http://blast.ncbi.nlm.nih.gov/Blast.cgi#22035604%2322035604) | 21.4 | 50% | 62 |
| [NP_004825.2](http://www.ncbi.nlm.nih.gov/entrez/query.fcgi?cmd=Retrieve&db=Protein&list_uids=22035602&dopt=GenPept&RID=T2RDE0YZ01N&log$=prottop&blast_rank=6) | mitogen-activated protein kinase kinase kinase kinase 4 isoform 1 [Homo sapiens] | [21.4](http://blast.ncbi.nlm.nih.gov/Blast.cgi#22035602%2322035602) | 21.4 | 50% | 62 |
| [NP_663720.1](http://www.ncbi.nlm.nih.gov/entrez/query.fcgi?cmd=Retrieve&db=Protein&list_uids=22035606&dopt=GenPept&RID=T2RDE0YZ01N&log$=prottop&blast_rank=7) | mitogen-activated protein kinase kinase kinase kinase 4 isoform 3 [Homo sapiens] | [21.4](http://blast.ncbi.nlm.nih.gov/Blast.cgi#22035606%2322035606) | 21.4 | 50% | 62 |
| [NP_001073922.2](http://www.ncbi.nlm.nih.gov/entrez/query.fcgi?cmd=Retrieve&db=Protein&list_uids=160948599&dopt=GenPept&RID=T2RDE0YZ01N&log$=prottop&blast_rank=8) | integrator complex subunit 1 [Homo sapiens] | [21.0](http://blast.ncbi.nlm.nih.gov/Blast.cgi#160948599%23160948599) | 21.0 | 50% | 83 |
| [NP_003631.2](http://www.ncbi.nlm.nih.gov/entrez/query.fcgi?cmd=Retrieve&db=Protein&list_uids=38569394&dopt=GenPept&RID=T2RDE0YZ01N&log$=prottop&blast_rank=9) | inhibitor of kappa light polypeptide gene enhancer in B-cells, kinase complex-associated protein [Homo sapiens] | [21.0](http://blast.ncbi.nlm.nih.gov/Blast.cgi#38569394%2338569394) | 21.0 | 50% | 83 |
| [NP_001123628.1](http://www.ncbi.nlm.nih.gov/entrez/query.fcgi?cmd=Retrieve&db=Protein&list_uids=194328678&dopt=GenPept&RID=T2RDE0YZ01N&log$=prottop&blast_rank=10) | myeloid leukemia factor 1 isoform 2 [Homo sapiens] >ref|NP_001123629.1| myeloid leukemia factor 1 isoform 2 [Homo sapiens] | [21.0](http://blast.ncbi.nlm.nih.gov/Blast.cgi#194328678%23194328678) | 21.0 | 70% | 83 |
| [NP_071888.1](http://www.ncbi.nlm.nih.gov/entrez/query.fcgi?cmd=Retrieve&db=Protein&list_uids=11967975&dopt=GenPept&RID=T2RDE0YZ01N&log$=prottop&blast_rank=11) | myeloid leukemia factor 1 isoform 1 [Homo sapiens] | [21.0](http://blast.ncbi.nlm.nih.gov/Blast.cgi#11967975%2311967975) | 21.0 | 70% | 83 |
| [NP_001099007.1](http://www.ncbi.nlm.nih.gov/entrez/query.fcgi?cmd=Retrieve&db=Protein&list_uids=157676340&dopt=GenPept&RID=T2RDE0YZ01N&log$=prottop&blast_rank=12) | zinc finger protein 142 [Homo sapiens] | [20.6](http://blast.ncbi.nlm.nih.gov/Blast.cgi#157676340%23157676340) | 34.4 | 70% | 111 |
| [NP_000850.1](http://www.ncbi.nlm.nih.gov/entrez/query.fcgi?cmd=Retrieve&db=Protein&list_uids=4557643&dopt=GenPept&RID=T2RDE0YZ01N&log$=prottop&blast_rank=13) | 3-hydroxy-3-methylglutaryl-Coenzyme A reductase isoform 1 [Homo sapiens] | [20.6](http://blast.ncbi.nlm.nih.gov/Blast.cgi#4557643%234557643) | 20.6 | 90% | 111 |
| [NP_001695.1](http://www.ncbi.nlm.nih.gov/entrez/query.fcgi?cmd=Retrieve&db=Protein&list_uids=4502359&dopt=GenPept&RID=T2RDE0YZ01N&log$=prottop&blast_rank=14) | brain-specific angiogenesis inhibitor 3 precursor [Homo sapiens] | [20.6](http://blast.ncbi.nlm.nih.gov/Blast.cgi#4502359%234502359) | 20.6 | 60% | 111 |
| [NP_001034973.2](http://www.ncbi.nlm.nih.gov/entrez/query.fcgi?cmd=Retrieve&db=Protein&list_uids=194239715&dopt=GenPept&RID=T2RDE0YZ01N&log$=prottop&blast_rank=15) | zinc finger protein 826 [Homo sapiens] | [20.2](http://blast.ncbi.nlm.nih.gov/Blast.cgi#194239715%23194239715) | 36.1 | 90% | 149 |
| [NP_000178.2](http://www.ncbi.nlm.nih.gov/entrez/query.fcgi?cmd=Retrieve&db=Protein&list_uids=115527117&dopt=GenPept&RID=T2RDE0YZ01N&log$=prottop&blast_rank=16) | homogentisate 1,2-dioxygenase [Homo sapiens] | [20.2](http://blast.ncbi.nlm.nih.gov/Blast.cgi#115527117%23115527117) | 20.2 | 80% | 149 |
| [NP_775834.1](http://www.ncbi.nlm.nih.gov/entrez/query.fcgi?cmd=Retrieve&db=Protein&list_uids=27734861&dopt=GenPept&RID=T2RDE0YZ01N&log$=prottop&blast_rank=17) | hypothetical protein LOC222826 [Homo sapiens] | [20.2](http://blast.ncbi.nlm.nih.gov/Blast.cgi#27734861%2327734861) | 20.2 | 70% | 149 |
| [NP_000566.3](http://www.ncbi.nlm.nih.gov/entrez/query.fcgi?cmd=Retrieve&db=Protein&list_uids=27894330&dopt=GenPept&RID=T2RDE0YZ01N&log$=prottop&blast_rank=18) | interleukin 1, alpha proprotein [Homo sapiens] | [20.2](http://blast.ncbi.nlm.nih.gov/Blast.cgi#27894330%2327894330) | 20.2 | 90% | 149 |
| [NP_612387.1](http://www.ncbi.nlm.nih.gov/entrez/query.fcgi?cmd=Retrieve&db=Protein&list_uids=149274610&dopt=GenPept&RID=T2RDE0YZ01N&log$=prottop&blast_rank=19) | ferredoxin-fold anticodon binding domain containing 1 [Homo sapiens] | [19.7](http://blast.ncbi.nlm.nih.gov/Blast.cgi#149274610%23149274610) | 19.7 | 70% | 200 |
| [NP_002199.3](http://www.ncbi.nlm.nih.gov/entrez/query.fcgi?cmd=Retrieve&db=Protein&list_uids=148728188&dopt=GenPept&RID=T2RDE0YZ01N&log$=prottop&blast_rank=20) | integrin, alpha E precursor [Homo sapiens] | [19.7](http://blast.ncbi.nlm.nih.gov/Blast.cgi#148728188%23148728188) | 19.7 | 60% | 200 |
| [NP_001367.2](http://www.ncbi.nlm.nih.gov/entrez/query.fcgi?cmd=Retrieve&db=Protein&list_uids=33350932&dopt=GenPept&RID=T2RDE0YZ01N&log$=prottop&blast_rank=21) | cytoplasmic dynein 1 heavy chain 1 [Homo sapiens] | [19.7](http://blast.ncbi.nlm.nih.gov/Blast.cgi#33350932%2333350932) | 30.5 | 70% | 200 |
| [NP_001010932.1](http://www.ncbi.nlm.nih.gov/entrez/query.fcgi?cmd=Retrieve&db=Protein&list_uids=58533170&dopt=GenPept&RID=T2RDE0YZ01N&log$=prottop&blast_rank=22) | hepatocyte growth factor isoform 3 precursor [Homo sapiens] | [19.7](http://blast.ncbi.nlm.nih.gov/Blast.cgi#58533170%2358533170) | 19.7 | 60% | 200 |
| [NP_006144.1](http://www.ncbi.nlm.nih.gov/entrez/query.fcgi?cmd=Retrieve&db=Protein&list_uids=5453754&dopt=GenPept&RID=T2RDE0YZ01N&log$=prottop&blast_rank=23) | NCK adaptor protein 1 [Homo sapiens] | [19.7](http://blast.ncbi.nlm.nih.gov/Blast.cgi#5453754%235453754) | 19.7 | 60% | 200 |
| [NP_000592.3](http://www.ncbi.nlm.nih.gov/entrez/query.fcgi?cmd=Retrieve&db=Protein&list_uids=33859835&dopt=GenPept&RID=T2RDE0YZ01N&log$=prottop&blast_rank=24) | hepatocyte growth factor isoform 1 preproprotein [Homo sapiens] | [19.7](http://blast.ncbi.nlm.nih.gov/Blast.cgi#33859835%2333859835) | 19.7 | 60% | 200 |
| [XP_002345006.1](http://www.ncbi.nlm.nih.gov/entrez/query.fcgi?cmd=Retrieve&db=Protein&list_uids=239756501&dopt=GenPept&RID=T2RDE0YZ01N&log$=prottop&blast_rank=25) | PREDICTED: hypothetical protein XP_002345006 [Homo sapiens] | [19.3](http://blast.ncbi.nlm.nih.gov/Blast.cgi#239756501%23239756501) | 19.3 | 70% | 268 |
| [XP_002345004.1](http://www.ncbi.nlm.nih.gov/entrez/query.fcgi?cmd=Retrieve&db=Protein&list_uids=239756497&dopt=GenPept&RID=T2RDE0YZ01N&log$=prottop&blast_rank=26) | PREDICTED: hypothetical protein [Homo sapiens] | [19.3](http://blast.ncbi.nlm.nih.gov/Blast.cgi#239756497%23239756497) | 19.3 | 70% | 268 |
| [NP_001165276.1](http://www.ncbi.nlm.nih.gov/entrez/query.fcgi?cmd=Retrieve&db=Protein&list_uids=284507293&dopt=GenPept&RID=T2RDE0YZ01N&log$=prottop&blast_rank=27) | DNA polymerase sigma isoform 2 [Homo sapiens] | [19.3](http://blast.ncbi.nlm.nih.gov/Blast.cgi#284507293%23284507293) | 19.3 | 50% | 268 |
| [NP_001121699.1](http://www.ncbi.nlm.nih.gov/entrez/query.fcgi?cmd=Retrieve&db=Protein&list_uids=190014632&dopt=GenPept&RID=T2RDE0YZ01N&log$=prottop&blast_rank=28) | UDP-N-acetylglucosamine-2-epimerase/N-acetylmannosamine kinase isoform 1 [Homo sapiens] | [19.3](http://blast.ncbi.nlm.nih.gov/Blast.cgi#190014632%23190014632) | 19.3 | 60% | 268 |
| [NP_722520.2](http://www.ncbi.nlm.nih.gov/entrez/query.fcgi?cmd=Retrieve&db=Protein&list_uids=110556644&dopt=GenPept&RID=T2RDE0YZ01N&log$=prottop&blast_rank=29) | zinc finger protein, multitype 1 [Homo sapiens] | [19.3](http://blast.ncbi.nlm.nih.gov/Blast.cgi#110556644%23110556644) | 19.3 | 60% | 268 |
| [NP_001005336.1](http://www.ncbi.nlm.nih.gov/entrez/query.fcgi?cmd=Retrieve&db=Protein&list_uids=56549117&dopt=GenPept&RID=T2RDE0YZ01N&log$=prottop&blast_rank=30) | dynamin 1 isoform 2 [Homo sapiens] | [19.3](http://blast.ncbi.nlm.nih.gov/Blast.cgi#56549117%2356549117) | 19.3 | 70% | 268 |
| [NP_061872.2](http://www.ncbi.nlm.nih.gov/entrez/query.fcgi?cmd=Retrieve&db=Protein&list_uids=80861486&dopt=GenPept&RID=T2RDE0YZ01N&log$=prottop&blast_rank=31) | granule cell antiserum positive 14 [Homo sapiens] | [19.3](http://blast.ncbi.nlm.nih.gov/Blast.cgi#80861486%2380861486) | 19.3 | 60% | 268 |
| [NP_055796.1](http://www.ncbi.nlm.nih.gov/entrez/query.fcgi?cmd=Retrieve&db=Protein&list_uids=150010558&dopt=GenPept&RID=T2RDE0YZ01N&log$=prottop&blast_rank=32) | myosin, heavy polypeptide 15 [Homo sapiens] | [19.3](http://blast.ncbi.nlm.nih.gov/Blast.cgi#150010558%23150010558) | 19.3 | 60% | 268 |
| [NP_003990.1](http://www.ncbi.nlm.nih.gov/entrez/query.fcgi?cmd=Retrieve&db=Protein&list_uids=4557040&dopt=GenPept&RID=T2RDE0YZ01N&log$=prottop&blast_rank=33) | oncostatin M receptor isoform 1 precursor [Homo sapiens] | [19.3](http://blast.ncbi.nlm.nih.gov/Blast.cgi#4557040%234557040) | 19.3 | 90% | 268 |
| [NP_009007.2](http://www.ncbi.nlm.nih.gov/entrez/query.fcgi?cmd=Retrieve&db=Protein&list_uids=42794620&dopt=GenPept&RID=T2RDE0YZ01N&log$=prottop&blast_rank=34) | Huntingtin interacting protein E [Homo sapiens] | [19.3](http://blast.ncbi.nlm.nih.gov/Blast.cgi#42794620%2342794620) | 19.3 | 100% | 268 |
| [NP_004399.2](http://www.ncbi.nlm.nih.gov/entrez/query.fcgi?cmd=Retrieve&db=Protein&list_uids=59853099&dopt=GenPept&RID=T2RDE0YZ01N&log$=prottop&blast_rank=35) | dynamin 1 isoform 1 [Homo sapiens] | [19.3](http://blast.ncbi.nlm.nih.gov/Blast.cgi#59853099%2359853099) | 19.3 | 70% | 268 |
| [NP_008930.1](http://www.ncbi.nlm.nih.gov/entrez/query.fcgi?cmd=Retrieve&db=Protein&list_uids=5902142&dopt=GenPept&RID=T2RDE0YZ01N&log$=prottop&blast_rank=36) | DNA polymerase sigma isoform 1 [Homo sapiens] | [19.3](http://blast.ncbi.nlm.nih.gov/Blast.cgi#5902142%235902142) | 19.3 | 50% | 268 |
| [NP_060888.2](http://www.ncbi.nlm.nih.gov/entrez/query.fcgi?cmd=Retrieve&db=Protein&list_uids=94536848&dopt=GenPept&RID=T2RDE0YZ01N&log$=prottop&blast_rank=37) | spermatogenesis-associated protein 7 isoform a [Homo sapiens] | [19.3](http://blast.ncbi.nlm.nih.gov/Blast.cgi#94536848%2394536848) | 19.3 | 70% | 268 |
| [NP_003623.1](http://www.ncbi.nlm.nih.gov/entrez/query.fcgi?cmd=Retrieve&db=Protein&list_uids=4505463&dopt=GenPept&RID=T2RDE0YZ01N&log$=prottop&blast_rank=38) | contactin associated protein 1 precursor [Homo sapiens] | [19.3](http://blast.ncbi.nlm.nih.gov/Blast.cgi#4505463%234505463) | 19.3 | 70% | 268 |
| [NP_005467.1](http://www.ncbi.nlm.nih.gov/entrez/query.fcgi?cmd=Retrieve&db=Protein&list_uids=4885285&dopt=GenPept&RID=T2RDE0YZ01N&log$=prottop&blast_rank=39) | UDP-N-acetylglucosamine-2-epimerase/N-acetylmannosamine kinase isoform 2 [Homo sapiens] | [19.3](http://blast.ncbi.nlm.nih.gov/Blast.cgi#4885285%234885285) | 19.3 | 60% | 268 |
| [NP_001164641.1](http://www.ncbi.nlm.nih.gov/entrez/query.fcgi?cmd=Retrieve&db=Protein&list_uids=283837920&dopt=GenPept&RID=T2RDE0YZ01N&log$=prottop&blast_rank=40) | solute carrier family 25 (mitochondrial oxodicarboxylate carrier), member 21 isoform 2 [Homo sapiens] | [18.9](http://blast.ncbi.nlm.nih.gov/Blast.cgi#283837920%23283837920) | 18.9 | 90% | 359 |
| [XP_002343223.1](http://www.ncbi.nlm.nih.gov/entrez/query.fcgi?cmd=Retrieve&db=Protein&list_uids=239744697&dopt=GenPept&RID=T2RDE0YZ01N&log$=prottop&blast_rank=41) | PREDICTED: hypothetical protein XP_002343223 [Homo sapiens] >ref|XP_002347386.1| PREDICTED: hypothetical protein XP_002347386 [Homo sapiens] >ref|XP_002344736.1| PREDICTED: hypothetical protein [Homo sapiens] | [18.9](http://blast.ncbi.nlm.nih.gov/Blast.cgi#239744697%23239744697) | 18.9 | 50% | 359 |
| [NP_001154825.1](http://www.ncbi.nlm.nih.gov/entrez/query.fcgi?cmd=Retrieve&db=Protein&list_uids=238624132&dopt=GenPept&RID=T2RDE0YZ01N&log$=prottop&blast_rank=42) | large conductance calcium-activated potassium channel subfamily M alpha member 1 isoform d [Homo sapiens] | [18.9](http://blast.ncbi.nlm.nih.gov/Blast.cgi#238624132%23238624132) | 18.9 | 60% | 359 |
| [NP_065873.2](http://www.ncbi.nlm.nih.gov/entrez/query.fcgi?cmd=Retrieve&db=Protein&list_uids=240255505&dopt=GenPept&RID=T2RDE0YZ01N&log$=prottop&blast_rank=43) | potassium channel, subfamily T, member 1 [Homo sapiens] | [18.9](http://blast.ncbi.nlm.nih.gov/Blast.cgi#240255505%23240255505) | 34.4 | 60% | 359 |
| [NP_001014797.1](http://www.ncbi.nlm.nih.gov/entrez/query.fcgi?cmd=Retrieve&db=Protein&list_uids=62388890&dopt=GenPept&RID=T2RDE0YZ01N&log$=prottop&blast_rank=44) | large conductance calcium-activated potassium channel subfamily M alpha member 1 isoform a [Homo sapiens] | [18.9](http://blast.ncbi.nlm.nih.gov/Blast.cgi#62388890%2362388890) | 18.9 | 60% | 359 |
| [NP_919276.2](http://www.ncbi.nlm.nih.gov/entrez/query.fcgi?cmd=Retrieve&db=Protein&list_uids=154937346&dopt=GenPept&RID=T2RDE0YZ01N&log$=prottop&blast_rank=45) | coiled-coil domain containing 129 [Homo sapiens] | [18.9](http://blast.ncbi.nlm.nih.gov/Blast.cgi#154937346%23154937346) | 18.9 | 50% | 359 |
| [NP_060110.3](http://www.ncbi.nlm.nih.gov/entrez/query.fcgi?cmd=Retrieve&db=Protein&list_uids=63054866&dopt=GenPept&RID=T2RDE0YZ01N&log$=prottop&blast_rank=46) | leucine rich repeat containing 16A [Homo sapiens] | [18.9](http://blast.ncbi.nlm.nih.gov/Blast.cgi#63054866%2363054866) | 18.9 | 70% | 359 |
| [NP_060497.3](http://www.ncbi.nlm.nih.gov/entrez/query.fcgi?cmd=Retrieve&db=Protein&list_uids=116063562&dopt=GenPept&RID=T2RDE0YZ01N&log$=prottop&blast_rank=47) | FERM domain containing 4A [Homo sapiens] | [18.9](http://blast.ncbi.nlm.nih.gov/Blast.cgi#116063562%23116063562) | 29.3 | 80% | 359 |
| [NP_057368.3](http://www.ncbi.nlm.nih.gov/entrez/query.fcgi?cmd=Retrieve&db=Protein&list_uids=42716275&dopt=GenPept&RID=T2RDE0YZ01N&log$=prottop&blast_rank=48) | CCR4-NOT transcription complex, subunit 1 isoform a [Homo sapiens] | [18.9](http://blast.ncbi.nlm.nih.gov/Blast.cgi#42716275%2342716275) | 18.9 | 50% | 359 |
| [NP_940905.2](http://www.ncbi.nlm.nih.gov/entrez/query.fcgi?cmd=Retrieve&db=Protein&list_uids=41349443&dopt=GenPept&RID=T2RDE0YZ01N&log$=prottop&blast_rank=49) | potassium channel, subfamily T, member 2 [Homo sapiens] | [18.9](http://blast.ncbi.nlm.nih.gov/Blast.cgi#41349443%2341349443) | 34.4 | 60% | 359 |
| [NP_872415.1](http://www.ncbi.nlm.nih.gov/entrez/query.fcgi?cmd=Retrieve&db=Protein&list_uids=33438598&dopt=GenPept&RID=T2RDE0YZ01N&log$=prottop&blast_rank=50) | zinc finger protein 677 [Homo sapiens] | [18.9](http://blast.ncbi.nlm.nih.gov/Blast.cgi#33438598%2333438598) | 18.9 | 90% | 359 |
| [NP_115597.3](http://www.ncbi.nlm.nih.gov/entrez/query.fcgi?cmd=Retrieve&db=Protein&list_uids=29244924&dopt=GenPept&RID=T2RDE0YZ01N&log$=prottop&blast_rank=51) | chromodomain helicase DNA binding protein 6 [Homo sapiens] | [18.9](http://blast.ncbi.nlm.nih.gov/Blast.cgi#29244924%2329244924) | 28.8 | 60% | 359 |
| [NP_001154824.1](http://www.ncbi.nlm.nih.gov/entrez/query.fcgi?cmd=Retrieve&db=Protein&list_uids=238624130&dopt=GenPept&RID=T2RDE0YZ01N&log$=prottop&blast_rank=52) | large conductance calcium-activated potassium channel subfamily M alpha member 1 isoform c [Homo sapiens] | [18.9](http://blast.ncbi.nlm.nih.gov/Blast.cgi#238624130%23238624130) | 18.9 | 60% | 359 |
| [NP_085134.1](http://www.ncbi.nlm.nih.gov/entrez/query.fcgi?cmd=Retrieve&db=Protein&list_uids=13449279&dopt=GenPept&RID=T2RDE0YZ01N&log$=prottop&blast_rank=53) | solute carrier family 25 (mitochondrial oxodicarboxylate carrier), member 21 isoform 1 [Homo sapiens] | [18.9](http://blast.ncbi.nlm.nih.gov/Blast.cgi#13449279%2313449279) | 18.9 | 90% | 359 |
| [NP_002238.2](http://www.ncbi.nlm.nih.gov/entrez/query.fcgi?cmd=Retrieve&db=Protein&list_uids=26638650&dopt=GenPept&RID=T2RDE0YZ01N&log$=prottop&blast_rank=54) | large conductance calcium-activated potassium channel subfamily M alpha member 1 isoform b [Homo sapiens] | [18.9](http://blast.ncbi.nlm.nih.gov/Blast.cgi#26638650%2326638650) | 18.9 | 60% | 359 |
| [NP_055847.1](http://www.ncbi.nlm.nih.gov/entrez/query.fcgi?cmd=Retrieve&db=Protein&list_uids=7657269&dopt=GenPept&RID=T2RDE0YZ01N&log$=prottop&blast_rank=55) | PDS5, regulator of cohesion maintenance, homolog B [Homo sapiens] | [18.9](http://blast.ncbi.nlm.nih.gov/Blast.cgi#7657269%237657269) | 50.7 | 90% | 359 |
| [NP_060206.2](http://www.ncbi.nlm.nih.gov/entrez/query.fcgi?cmd=Retrieve&db=Protein&list_uids=42476024&dopt=GenPept&RID=T2RDE0YZ01N&log$=prottop&blast_rank=56) | THUMP domain containing 1 [Homo sapiens] | [18.9](http://blast.ncbi.nlm.nih.gov/Blast.cgi#42476024%2342476024) | 18.9 | 50% | 359 |
| [NP_055041.1](http://www.ncbi.nlm.nih.gov/entrez/query.fcgi?cmd=Retrieve&db=Protein&list_uids=7657498&dopt=GenPept&RID=T2RDE0YZ01N&log$=prottop&blast_rank=57) | MAPK/MAK/MRK overlapping kinase [Homo sapiens] | [18.9](http://blast.ncbi.nlm.nih.gov/Blast.cgi#7657498%237657498) | 18.9 | 60% | 359 |
| [NP_060870.1](http://www.ncbi.nlm.nih.gov/entrez/query.fcgi?cmd=Retrieve&db=Protein&list_uids=9055238&dopt=GenPept&RID=T2RDE0YZ01N&log$=prottop&blast_rank=58) | voltage-gated sodium channel beta-3 subunit precursor [Homo sapiens] >ref|NP_001035241.1| voltage-gated sodium channel beta-3 subunit precursor [Homo sapiens] | [18.9](http://blast.ncbi.nlm.nih.gov/Blast.cgi#9055238%239055238) | 28.8 | 90% | 359 |
| [NP_115511.3](http://www.ncbi.nlm.nih.gov/entrez/query.fcgi?cmd=Retrieve&db=Protein&list_uids=209977046&dopt=GenPept&RID=T2RDE0YZ01N&log$=prottop&blast_rank=59) | fibrous sheath CABYR binding protein [Homo sapiens] | [18.5](http://blast.ncbi.nlm.nih.gov/Blast.cgi#209977046%23209977046) | 18.5 | 70% | 482 |
| [NP_940980.3](http://www.ncbi.nlm.nih.gov/entrez/query.fcgi?cmd=Retrieve&db=Protein&list_uids=171846278&dopt=GenPept&RID=T2RDE0YZ01N&log$=prottop&blast_rank=60) | leucine-rich repeat kinase 2 [Homo sapiens] | [18.5](http://blast.ncbi.nlm.nih.gov/Blast.cgi#171846278%23171846278) | 18.5 | 40% | 482 |
| [NP_057336.3](http://www.ncbi.nlm.nih.gov/entrez/query.fcgi?cmd=Retrieve&db=Protein&list_uids=153792694&dopt=GenPept&RID=T2RDE0YZ01N&log$=prottop&blast_rank=61) | baculoviral IAP repeat-containing 6 [Homo sapiens] | [18.5](http://blast.ncbi.nlm.nih.gov/Blast.cgi#153792694%23153792694) | 18.5 | 50% | 482 |
| [NP_001027.3](http://www.ncbi.nlm.nih.gov/entrez/query.fcgi?cmd=Retrieve&db=Protein&list_uids=126032338&dopt=GenPept&RID=T2RDE0YZ01N&log$=prottop&blast_rank=62) | ryanodine receptor 3 [Homo sapiens] | [18.5](http://blast.ncbi.nlm.nih.gov/Blast.cgi#126032338%23126032338) | 28.4 | 70% | 482 |
| [NP_001138912.1](http://www.ncbi.nlm.nih.gov/entrez/query.fcgi?cmd=Retrieve&db=Protein&list_uids=224282123&dopt=GenPept&RID=T2RDE0YZ01N&log$=prottop&blast_rank=63) | tRNA-yW synthesizing protein 1 homolog B isoform 1 [Homo sapiens] | [18.5](http://blast.ncbi.nlm.nih.gov/Blast.cgi#224282123%23224282123) | 18.5 | 50% | 482 |
| [NP_060734.2](http://www.ncbi.nlm.nih.gov/entrez/query.fcgi?cmd=Retrieve&db=Protein&list_uids=50726981&dopt=GenPept&RID=T2RDE0YZ01N&log$=prottop&blast_rank=64) | radical S-adenosyl methionine and flavodoxin domains 1 [Homo sapiens] | [18.5](http://blast.ncbi.nlm.nih.gov/Blast.cgi#50726981%2350726981) | 18.5 | 50% | 482 |
| [NP_003362.2](http://www.ncbi.nlm.nih.gov/entrez/query.fcgi?cmd=Retrieve&db=Protein&list_uids=40549448&dopt=GenPept&RID=T2RDE0YZ01N&log$=prottop&blast_rank=65) | vav 2 guanine nucleotide exchange factor isoform 2 [Homo sapiens] | [18.5](http://blast.ncbi.nlm.nih.gov/Blast.cgi#40549448%2340549448) | 18.5 | 40% | 482 |
| [NP_689804.1](http://www.ncbi.nlm.nih.gov/entrez/query.fcgi?cmd=Retrieve&db=Protein&list_uids=22749217&dopt=GenPept&RID=T2RDE0YZ01N&log$=prottop&blast_rank=66) | coiled-coil domain containing 63 [Homo sapiens] | [18.5](http://blast.ncbi.nlm.nih.gov/Blast.cgi#22749217%2322749217) | 18.5 | 40% | 482 |
| [NP_065897.1](http://www.ncbi.nlm.nih.gov/entrez/query.fcgi?cmd=Retrieve&db=Protein&list_uids=149588928&dopt=GenPept&RID=T2RDE0YZ01N&log$=prottop&blast_rank=67) | SLAIN motif family, member 2 [Homo sapiens] | [18.5](http://blast.ncbi.nlm.nih.gov/Blast.cgi#149588928%23149588928) | 18.5 | 50% | 482 |
| [NP_001008409.1](http://www.ncbi.nlm.nih.gov/entrez/query.fcgi?cmd=Retrieve&db=Protein&list_uids=56606131&dopt=GenPept&RID=T2RDE0YZ01N&log$=prottop&blast_rank=68) | tubulin tyrosine ligase-like family, member 9 [Homo sapiens] | [18.5](http://blast.ncbi.nlm.nih.gov/Blast.cgi#56606131%2356606131) | 18.5 | 40% | 482 |
| [NP_055730.2](http://www.ncbi.nlm.nih.gov/entrez/query.fcgi?cmd=Retrieve&db=Protein&list_uids=154354990&dopt=GenPept&RID=T2RDE0YZ01N&log$=prottop&blast_rank=69) | ankyrin repeat domain 26 [Homo sapiens] | [18.5](http://blast.ncbi.nlm.nih.gov/Blast.cgi#154354990%23154354990) | 46.5 | 70% | 482 |
| [NP_001127870.1](http://www.ncbi.nlm.nih.gov/entrez/query.fcgi?cmd=Retrieve&db=Protein&list_uids=197304715&dopt=GenPept&RID=T2RDE0YZ01N&log$=prottop&blast_rank=70) | vav 2 guanine nucleotide exchange factor isoform 1 [Homo sapiens] | [18.5](http://blast.ncbi.nlm.nih.gov/Blast.cgi#197304715%23197304715) | 18.5 | 40% | 482 |
| [NP_542416.1](http://www.ncbi.nlm.nih.gov/entrez/query.fcgi?cmd=Retrieve&db=Protein&list_uids=18375650&dopt=GenPept&RID=T2RDE0YZ01N&log$=prottop&blast_rank=71) | protein tyrosine phosphatase, non-receptor type 13 isoform 4 [Homo sapiens] | [18.5](http://blast.ncbi.nlm.nih.gov/Blast.cgi#18375650%2318375650) | 32.7 | 50% | 482 |
| [NP_115551.2](http://www.ncbi.nlm.nih.gov/entrez/query.fcgi?cmd=Retrieve&db=Protein&list_uids=50980309&dopt=GenPept&RID=T2RDE0YZ01N&log$=prottop&blast_rank=72) | UTP15, U3 small nucleolar ribonucleoprotein, homolog [Homo sapiens] | [18.5](http://blast.ncbi.nlm.nih.gov/Blast.cgi#50980309%2350980309) | 18.5 | 40% | 482 |
| [NP_839955.1](http://www.ncbi.nlm.nih.gov/entrez/query.fcgi?cmd=Retrieve&db=Protein&list_uids=30410716&dopt=GenPept&RID=T2RDE0YZ01N&log$=prottop&blast_rank=73) | exocyst complex component 1 isoform 2 [Homo sapiens] | [18.5](http://blast.ncbi.nlm.nih.gov/Blast.cgi#30410716%2330410716) | 18.5 | 40% | 482 |
| [NP_997190.1](http://www.ncbi.nlm.nih.gov/entrez/query.fcgi?cmd=Retrieve&db=Protein&list_uids=46409260&dopt=GenPept&RID=T2RDE0YZ01N&log$=prottop&blast_rank=74) | hypothetical protein LOC90288 [Homo sapiens] | [18.5](http://blast.ncbi.nlm.nih.gov/Blast.cgi#46409260%2346409260) | 18.5 | 40% | 482 |
| [NP_694550.1](http://www.ncbi.nlm.nih.gov/entrez/query.fcgi?cmd=Retrieve&db=Protein&list_uids=23510358&dopt=GenPept&RID=T2RDE0YZ01N&log$=prottop&blast_rank=75) | RIO kinase 1 isoform 2 [Homo sapiens] | [18.5](http://blast.ncbi.nlm.nih.gov/Blast.cgi#23510358%2323510358) | 18.5 | 70% | 482 |
| [NP_002075.2](http://www.ncbi.nlm.nih.gov/entrez/query.fcgi?cmd=Retrieve&db=Protein&list_uids=6006001&dopt=GenPept&RID=T2RDE0YZ01N&log$=prottop&blast_rank=76) | glutathione peroxidase 3 precursor [Homo sapiens] | [18.5](http://blast.ncbi.nlm.nih.gov/Blast.cgi#6006001%236006001) | 18.5 | 70% | 482 |
| [NP_775781.1](http://www.ncbi.nlm.nih.gov/entrez/query.fcgi?cmd=Retrieve&db=Protein&list_uids=27735133&dopt=GenPept&RID=T2RDE0YZ01N&log$=prottop&blast_rank=77) | coiled-coil domain containing 117 [Homo sapiens] | [18.5](http://blast.ncbi.nlm.nih.gov/Blast.cgi#27735133%2327735133) | 18.5 | 60% | 482 |
| [NP_113668.2](http://www.ncbi.nlm.nih.gov/entrez/query.fcgi?cmd=Retrieve&db=Protein&list_uids=23510356&dopt=GenPept&RID=T2RDE0YZ01N&log$=prottop&blast_rank=78) | RIO kinase 1 isoform 1 [Homo sapiens] | [18.5](http://blast.ncbi.nlm.nih.gov/Blast.cgi#23510356%2323510356) | 18.5 | 70% | 482 |
| [NP_060731.2](http://www.ncbi.nlm.nih.gov/entrez/query.fcgi?cmd=Retrieve&db=Protein&list_uids=30410720&dopt=GenPept&RID=T2RDE0YZ01N&log$=prottop&blast_rank=79) | exocyst complex component 1 isoform 1 [Homo sapiens] >ref|NP_001020095.1| exocyst complex component 1 isoform 1 [Homo sapiens] | [18.5](http://blast.ncbi.nlm.nih.gov/Blast.cgi#30410720%2330410720) | 18.5 | 40% | 482 |
| [NP_036247.1](http://www.ncbi.nlm.nih.gov/entrez/query.fcgi?cmd=Retrieve&db=Protein&list_uids=6912288&dopt=GenPept&RID=T2RDE0YZ01N&log$=prottop&blast_rank=80) | caspase 8 associated protein 2 [Homo sapiens] >ref|NP_001131140.1| caspase 8 associated protein 2 [Homo sapiens] >ref|NP_001131139.1| caspase 8 associated protein 2 [Homo sapiens] | [18.5](http://blast.ncbi.nlm.nih.gov/Blast.cgi#6912288%236912288) | 18.5 | 40% | 482 |
| [NP_001900.1](http://www.ncbi.nlm.nih.gov/entrez/query.fcgi?cmd=Retrieve&db=Protein&list_uids=4503143&dopt=GenPept&RID=T2RDE0YZ01N&log$=prottop&blast_rank=81) | cathepsin D preproprotein [Homo sapiens] | [18.5](http://blast.ncbi.nlm.nih.gov/Blast.cgi#4503143%234503143) | 18.5 | 40% | 482 |
| [NP_443190.1](http://www.ncbi.nlm.nih.gov/entrez/query.fcgi?cmd=Retrieve&db=Protein&list_uids=16418451&dopt=GenPept&RID=T2RDE0YZ01N&log$=prottop&blast_rank=82) | hypothetical protein LOC116328 [Homo sapiens] | [18.5](http://blast.ncbi.nlm.nih.gov/Blast.cgi#16418451%2316418451) | 18.5 | 40% | 482 |
| [NP_064502.9](http://www.ncbi.nlm.nih.gov/entrez/query.fcgi?cmd=Retrieve&db=Protein&list_uids=108773810&dopt=GenPept&RID=T2RDE0YZ01N&log$=prottop&blast_rank=83) | leucyl-tRNA synthetase [Homo sapiens] | [18.5](http://blast.ncbi.nlm.nih.gov/Blast.cgi#108773810%23108773810) | 18.5 | 40% | 482 |
| [NP_542415.1](http://www.ncbi.nlm.nih.gov/entrez/query.fcgi?cmd=Retrieve&db=Protein&list_uids=18375648&dopt=GenPept&RID=T2RDE0YZ01N&log$=prottop&blast_rank=84) | protein tyrosine phosphatase, non-receptor type 13 isoform 3 [Homo sapiens] | [18.5](http://blast.ncbi.nlm.nih.gov/Blast.cgi#18375648%2318375648) | 32.7 | 50% | 482 |
| [NP_006255.1](http://www.ncbi.nlm.nih.gov/entrez/query.fcgi?cmd=Retrieve&db=Protein&list_uids=5453992&dopt=GenPept&RID=T2RDE0YZ01N&log$=prottop&blast_rank=85) | protein tyrosine phosphatase, non-receptor type 13 isoform 2 [Homo sapiens] | [18.5](http://blast.ncbi.nlm.nih.gov/Blast.cgi#5453992%235453992) | 32.7 | 50% | 482 |
| [NP_002960.2](http://www.ncbi.nlm.nih.gov/entrez/query.fcgi?cmd=Retrieve&db=Protein&list_uids=48255970&dopt=GenPept&RID=T2RDE0YZ01N&log$=prottop&blast_rank=86) | mitogen-activated protein kinase 12 [Homo sapiens] | [18.5](http://blast.ncbi.nlm.nih.gov/Blast.cgi#48255970%2348255970) | 18.5 | 40% | 482 |
| [NP_542414.1](http://www.ncbi.nlm.nih.gov/entrez/query.fcgi?cmd=Retrieve&db=Protein&list_uids=18375646&dopt=GenPept&RID=T2RDE0YZ01N&log$=prottop&blast_rank=87) | protein tyrosine phosphatase, non-receptor type 13 isoform 1 [Homo sapiens] | [18.5](http://blast.ncbi.nlm.nih.gov/Blast.cgi#18375646%2318375646) | 32.7 | 50% | 482 |
| [NP_001161221.1](http://www.ncbi.nlm.nih.gov/entrez/query.fcgi?cmd=Retrieve&db=Protein&list_uids=268370055&dopt=GenPept&RID=T2RDE0YZ01N&log$=prottop&blast_rank=88) | adenylate cyclase 10 isoform 2 [Homo sapiens] | [18.0](http://blast.ncbi.nlm.nih.gov/Blast.cgi#268370055%23268370055) | 18.0 | 40% | 647 |
| [NP_001138409.1](http://www.ncbi.nlm.nih.gov/entrez/query.fcgi?cmd=Retrieve&db=Protein&list_uids=222144290&dopt=GenPept&RID=T2RDE0YZ01N&log$=prottop&blast_rank=89) | fibronectin type III domain containing 7 [Homo sapiens] | [18.0](http://blast.ncbi.nlm.nih.gov/Blast.cgi#222144290%23222144290) | 18.0 | 40% | 647 |
| [XP_001714323.1](http://www.ncbi.nlm.nih.gov/entrez/query.fcgi?cmd=Retrieve&db=Protein&list_uids=169217315&dopt=GenPept&RID=T2RDE0YZ01N&log$=prottop&blast_rank=90) | PREDICTED: hypothetical protein [Homo sapiens] | [18.0](http://blast.ncbi.nlm.nih.gov/Blast.cgi#169217315%23169217315) | 18.0 | 40% | 647 |
| [XP_001719454.1](http://www.ncbi.nlm.nih.gov/entrez/query.fcgi?cmd=Retrieve&db=Protein&list_uids=169216042&dopt=GenPept&RID=T2RDE0YZ01N&log$=prottop&blast_rank=91) | PREDICTED: similar to SEC14p-like protein TAP3 [Homo sapiens] | [18.0](http://blast.ncbi.nlm.nih.gov/Blast.cgi#169216042%23169216042) | 18.0 | 50% | 647 |
| [XP_001132040.2](http://www.ncbi.nlm.nih.gov/entrez/query.fcgi?cmd=Retrieve&db=Protein&list_uids=169215635&dopt=GenPept&RID=T2RDE0YZ01N&log$=prottop&blast_rank=92) | PREDICTED: similar to SEC14p-like protein TAP3 [Homo sapiens] >ref|XP_001718003.1| PREDICTED: similar to SEC14p-like protein TAP3 [Homo sapiens] | [18.0](http://blast.ncbi.nlm.nih.gov/Blast.cgi#169215635%23169215635) | 18.0 | 50% | 647 |
| [NP_694591.2](http://www.ncbi.nlm.nih.gov/entrez/query.fcgi?cmd=Retrieve&db=Protein&list_uids=166851804&dopt=GenPept&RID=T2RDE0YZ01N&log$=prottop&blast_rank=93) | tudor domain containing 9 [Homo sapiens] | [18.0](http://blast.ncbi.nlm.nih.gov/Blast.cgi#166851804%23166851804) | 18.0 | 40% | 647 |
| [NP_001124171.1](http://www.ncbi.nlm.nih.gov/entrez/query.fcgi?cmd=Retrieve&db=Protein&list_uids=194733747&dopt=GenPept&RID=T2RDE0YZ01N&log$=prottop&blast_rank=94) | interaction protein for cytohesin exchange factors 1 isoform 1 [Homo sapiens] >ref|NP_001124172.1| interaction protein for cytohesin exchange factors 1 isoform 1 [Homo sapiens] | [18.0](http://blast.ncbi.nlm.nih.gov/Blast.cgi#194733747%23194733747) | 18.0 | 40% | 647 |
| [NP_002340.2](http://www.ncbi.nlm.nih.gov/entrez/query.fcgi?cmd=Retrieve&db=Protein&list_uids=144446030&dopt=GenPept&RID=T2RDE0YZ01N&log$=prottop&blast_rank=95) | lymphocyte antigen 75 precursor [Homo sapiens] | [18.0](http://blast.ncbi.nlm.nih.gov/Blast.cgi#144446030%23144446030) | 30.5 | 50% | 647 |
| [NP_001077007.1](http://www.ncbi.nlm.nih.gov/entrez/query.fcgi?cmd=Retrieve&db=Protein&list_uids=134133226&dopt=GenPept&RID=T2RDE0YZ01N&log$=prottop&blast_rank=96) | protein expressed in prostate, ovary, testis, and placenta 2 [Homo sapiens] | [18.0](http://blast.ncbi.nlm.nih.gov/Blast.cgi#134133226%23134133226) | 18.0 | 50% | 647 |
| [NP_001121631.1](http://www.ncbi.nlm.nih.gov/entrez/query.fcgi?cmd=Retrieve&db=Protein&list_uids=189491744&dopt=GenPept&RID=T2RDE0YZ01N&log$=prottop&blast_rank=97) | vacuolar protein sorting 53 isoform 1 [Homo sapiens] | [18.0](http://blast.ncbi.nlm.nih.gov/Blast.cgi#189491744%23189491744) | 18.0 | 50% | 647 |
| [NP_000827.2](http://www.ncbi.nlm.nih.gov/entrez/query.fcgi?cmd=Retrieve&db=Protein&list_uids=153946391&dopt=GenPept&RID=T2RDE0YZ01N&log$=prottop&blast_rank=98) | N-methyl-D-aspartate receptor subunit 2D precursor [Homo sapiens] | [18.0](http://blast.ncbi.nlm.nih.gov/Blast.cgi#153946391%23153946391) | 18.0 | 40% | 647 |
| [NP_001138628.1](http://www.ncbi.nlm.nih.gov/entrez/query.fcgi?cmd=Retrieve&db=Protein&list_uids=223555951&dopt=GenPept&RID=T2RDE0YZ01N&log$=prottop&blast_rank=99) | nuclear receptor subfamily 2, group F, member 2 isoform c [Homo sapiens] >ref|NP_001138629.1| nuclear receptor subfamily 2, group F, member 2 isoform c [Homo sapiens] | [18.0](http://blast.ncbi.nlm.nih.gov/Blast.cgi#223555951%23223555951) | 18.0 | 40% | 647 |
| [NP_001138627.1](http://www.ncbi.nlm.nih.gov/entrez/query.fcgi?cmd=Retrieve&db=Protein&list_uids=223555949&dopt=GenPept&RID=T2RDE0YZ01N&log$=prottop&blast_rank=100) | nuclear receptor subfamily 2, group F, member 2 isoform b [Homo sapiens] | [18.0](http://blast.ncbi.nlm.nih.gov/Blast.cgi#223555949%23223555949) | 18.0 | 40% | 647 |

| **Accession** | **Proteins with a match to QLHPHNLHSP peptide** | **[Max score](http://blast.ncbi.nlm.nih.gov/Blast.cgi?CMD=Get&ALIGNMENTS=100&ALIGNMENT_VIEW=Pairwise&CDD_SEARCH_STATE=1&DATABASE_SORT=0&DESCRIPTIONS=100&ENTREZ_QUERY=txid9606 %5BORGN%5D&FIRST_QUERY_NUM=0&FORMAT_OBJECT=Alignment&FORMAT_PAGE_TARGET=&FORMAT_TYPE=HTML&GET_SEQUENCE=yes&I_THRESH=&MASK_CHAR=2&MASK_COLOR=1&NEW_DESIGN=on&NEW_VIEW=yes&NUM_OVERVIEW=100&OLD_BLAST=false&PAGE=Proteins&QUERY_INDEX=0&QUERY_NUMBER=0&RESULTS_PAGE_TARGET=&RID=T2RMG8CM014&SHOW_LINKOUT=yes&SHOW_OVERVIEW=yes&STEP_NUMBER=&WORD_SIZE=2&DISPLAY_SORT=1&HSP_SORT=1" \l "sort_mark)** | **[Total score](http://blast.ncbi.nlm.nih.gov/Blast.cgi?CMD=Get&ALIGNMENTS=100&ALIGNMENT_VIEW=Pairwise&CDD_SEARCH_STATE=1&DATABASE_SORT=0&DESCRIPTIONS=100&ENTREZ_QUERY=txid9606 %5BORGN%5D&FIRST_QUERY_NUM=0&FORMAT_OBJECT=Alignment&FORMAT_PAGE_TARGET=&FORMAT_TYPE=HTML&GET_SEQUENCE=yes&I_THRESH=&MASK_CHAR=2&MASK_COLOR=1&NEW_DESIGN=on&NEW_VIEW=yes&NUM_OVERVIEW=100&OLD_BLAST=false&PAGE=Proteins&QUERY_INDEX=0&QUERY_NUMBER=0&RESULTS_PAGE_TARGET=&RID=T2RMG8CM014&SHOW_LINKOUT=yes&SHOW_OVERVIEW=yes&STEP_NUMBER=&WORD_SIZE=2&DISPLAY_SORT=2&HSP_SORT=1" \l "sort_mark)** | **[Query coverage](http://blast.ncbi.nlm.nih.gov/Blast.cgi?CMD=Get&ALIGNMENTS=100&ALIGNMENT_VIEW=Pairwise&CDD_SEARCH_STATE=1&DATABASE_SORT=0&DESCRIPTIONS=100&ENTREZ_QUERY=txid9606 %5BORGN%5D&FIRST_QUERY_NUM=0&FORMAT_OBJECT=Alignment&FORMAT_PAGE_TARGET=&FORMAT_TYPE=HTML&GET_SEQUENCE=yes&I_THRESH=&MASK_CHAR=2&MASK_COLOR=1&NEW_DESIGN=on&NEW_VIEW=yes&NUM_OVERVIEW=100&OLD_BLAST=false&PAGE=Proteins&QUERY_INDEX=0&QUERY_NUMBER=0&RESULTS_PAGE_TARGET=&RID=T2RMG8CM014&SHOW_LINKOUT=yes&SHOW_OVERVIEW=yes&STEP_NUMBER=&WORD_SIZE=2&DISPLAY_SORT=4&HSP_SORT=0" \l "sort_mark)** | **[E value](http://blast.ncbi.nlm.nih.gov/Blast.cgi?CMD=Get&ALIGNMENTS=100&ALIGNMENT_VIEW=Pairwise&CDD_SEARCH_STATE=1&DATABASE_SORT=0&DESCRIPTIONS=100&ENTREZ_QUERY=txid9606 %5BORGN%5D&FIRST_QUERY_NUM=0&FORMAT_OBJECT=Alignment&FORMAT_PAGE_TARGET=&FORMAT_TYPE=HTML&GET_SEQUENCE=yes&I_THRESH=&MASK_CHAR=2&MASK_COLOR=1&NEW_DESIGN=on&NEW_VIEW=yes&NUM_OVERVIEW=100&OLD_BLAST=false&PAGE=Proteins&QUERY_INDEX=0&QUERY_NUMBER=0&RESULTS_PAGE_TARGET=&RID=T2RMG8CM014&SHOW_LINKOUT=yes&SHOW_OVERVIEW=yes&STEP_NUMBER=&WORD_SIZE=2&DISPLAY_SORT=0&HSP_SORT=0" \l "sort_mark)** |
| --- | --- | --- | --- | --- | --- |
| [NP_002205.1](http://www.ncbi.nlm.nih.gov/entrez/query.fcgi?cmd=Retrieve&db=Protein&list_uids=4504779&dopt=GenPept&RID=T2RMG8CM014&log$=prottop&blast_rank=1) | integrin, beta 8 precursor [Homo sapiens] | [23.5](http://blast.ncbi.nlm.nih.gov/Blast.cgi#4504779%234504779) | 23.5 | 60% | 14 |
| [XP_001126164.3](http://www.ncbi.nlm.nih.gov/entrez/query.fcgi?cmd=Retrieve&db=Protein&list_uids=239508903&dopt=GenPept&RID=T2RMG8CM014&log$=prottop&blast_rank=2) | PREDICTED: WBSCR19-like protein 8-like [Homo sapiens] >ref|XP_499314.4| PREDICTED: WBSCR19-like protein 8-like isoform 2 [Homo sapiens] | [23.1](http://blast.ncbi.nlm.nih.gov/Blast.cgi#239508903%23239508903) | 23.1 | 100% | 19 |
| [XP_001720076.1](http://www.ncbi.nlm.nih.gov/entrez/query.fcgi?cmd=Retrieve&db=Protein&list_uids=169171620&dopt=GenPept&RID=T2RMG8CM014&log$=prottop&blast_rank=3) | PREDICTED: hypothetical protein [Homo sapiens] | [23.1](http://blast.ncbi.nlm.nih.gov/Blast.cgi#169171620%23169171620) | 23.1 | 100% | 19 |
| [NP_001138537.1](http://www.ncbi.nlm.nih.gov/entrez/query.fcgi?cmd=Retrieve&db=Protein&list_uids=222831647&dopt=GenPept&RID=T2RMG8CM014&log$=prottop&blast_rank=4) | KIAA1680 protein isoform 1 [Homo sapiens] | [22.3](http://blast.ncbi.nlm.nih.gov/Blast.cgi#222831647%23222831647) | 22.3 | 80% | 34 |
| [NP_001123553.1](http://www.ncbi.nlm.nih.gov/entrez/query.fcgi?cmd=Retrieve&db=Protein&list_uids=194272176&dopt=GenPept&RID=T2RMG8CM014&log$=prottop&blast_rank=5) | phospholipase D1 isoform b [Homo sapiens] | [22.3](http://blast.ncbi.nlm.nih.gov/Blast.cgi#194272176%23194272176) | 22.3 | 80% | 34 |
| [NP_002653.1](http://www.ncbi.nlm.nih.gov/entrez/query.fcgi?cmd=Retrieve&db=Protein&list_uids=4505873&dopt=GenPept&RID=T2RMG8CM014&log$=prottop&blast_rank=6) | phospholipase D1 isoform a [Homo sapiens] | [22.3](http://blast.ncbi.nlm.nih.gov/Blast.cgi#4505873%234505873) | 22.3 | 80% | 34 |
| [NP_001159764.1](http://www.ncbi.nlm.nih.gov/entrez/query.fcgi?cmd=Retrieve&db=Protein&list_uids=261823993&dopt=GenPept&RID=T2RMG8CM014&log$=prottop&blast_rank=7) | patched 2 isoform 2 [Homo sapiens] | [21.4](http://blast.ncbi.nlm.nih.gov/Blast.cgi#261823993%23261823993) | 21.4 | 90% | 62 |
| [NP_653267.2](http://www.ncbi.nlm.nih.gov/entrez/query.fcgi?cmd=Retrieve&db=Protein&list_uids=222144249&dopt=GenPept&RID=T2RMG8CM014&log$=prottop&blast_rank=8) | dynein heavy chain domain 1 isoform 1 [Homo sapiens] | [21.4](http://blast.ncbi.nlm.nih.gov/Blast.cgi#222144249%23222144249) | 81.2 | 90% | 62 |
| [NP_003729.3](http://www.ncbi.nlm.nih.gov/entrez/query.fcgi?cmd=Retrieve&db=Protein&list_uids=52145305&dopt=GenPept&RID=T2RMG8CM014&log$=prottop&blast_rank=9) | patched 2 isoform 1 [Homo sapiens] | [21.4](http://blast.ncbi.nlm.nih.gov/Blast.cgi#52145305%2352145305) | 21.4 | 90% | 62 |
| [NP_001127846.1](http://www.ncbi.nlm.nih.gov/entrez/query.fcgi?cmd=Retrieve&db=Protein&list_uids=197276670&dopt=GenPept&RID=T2RMG8CM014&log$=prottop&blast_rank=10) | spermatogenesis and centriole associated 1 isoform 2 [Homo sapiens] | [21.0](http://blast.ncbi.nlm.nih.gov/Blast.cgi#197276670%23197276670) | 21.0 | 70% | 83 |
| [NP_904358.2](http://www.ncbi.nlm.nih.gov/entrez/query.fcgi?cmd=Retrieve&db=Protein&list_uids=171460948&dopt=GenPept&RID=T2RMG8CM014&log$=prottop&blast_rank=11) | TSC22 domain family, member 1 isoform 1 [Homo sapiens] | [21.0](http://blast.ncbi.nlm.nih.gov/Blast.cgi#171460948%23171460948) | 21.0 | 60% | 83 |
| [NP_940974.2](http://www.ncbi.nlm.nih.gov/entrez/query.fcgi?cmd=Retrieve&db=Protein&list_uids=197276668&dopt=GenPept&RID=T2RMG8CM014&log$=prottop&blast_rank=12) | spermatogenesis and centriole associated 1 isoform 1 [Homo sapiens] | [21.0](http://blast.ncbi.nlm.nih.gov/Blast.cgi#197276668%23197276668) | 21.0 | 70% | 83 |
| [NP_942146.2](http://www.ncbi.nlm.nih.gov/entrez/query.fcgi?cmd=Retrieve&db=Protein&list_uids=110578665&dopt=GenPept&RID=T2RMG8CM014&log$=prottop&blast_rank=13) | seven in absentia homolog 3 [Homo sapiens] | [21.0](http://blast.ncbi.nlm.nih.gov/Blast.cgi#110578665%23110578665) | 21.0 | 60% | 83 |
| [XP_001716936.2](http://www.ncbi.nlm.nih.gov/entrez/query.fcgi?cmd=Retrieve&db=Protein&list_uids=239745999&dopt=GenPept&RID=T2RMG8CM014&log$=prottop&blast_rank=14) | PREDICTED: hypothetical protein [Homo sapiens] | [20.6](http://blast.ncbi.nlm.nih.gov/Blast.cgi#239745999%23239745999) | 20.6 | 50% | 111 |
| [NP_055699.2](http://www.ncbi.nlm.nih.gov/entrez/query.fcgi?cmd=Retrieve&db=Protein&list_uids=224282117&dopt=GenPept&RID=T2RMG8CM014&log$=prottop&blast_rank=15) | splicing factor, arginine/serine-rich 14 [Homo sapiens] >ref|NP_001017392.2| splicing factor, arginine/serine-rich 14 [Homo sapiens] | [20.6](http://blast.ncbi.nlm.nih.gov/Blast.cgi#224282117%23224282117) | 20.6 | 50% | 111 |
| [NP_001129595.1](http://www.ncbi.nlm.nih.gov/entrez/query.fcgi?cmd=Retrieve&db=Protein&list_uids=209915547&dopt=GenPept&RID=T2RMG8CM014&log$=prottop&blast_rank=16) | hypothetical protein LOC55719 isoform 2 [Homo sapiens] | [20.6](http://blast.ncbi.nlm.nih.gov/Blast.cgi#209915547%23209915547) | 20.6 | 50% | 111 |
| [NP_945339.2](http://www.ncbi.nlm.nih.gov/entrez/query.fcgi?cmd=Retrieve&db=Protein&list_uids=206725452&dopt=GenPept&RID=T2RMG8CM014&log$=prottop&blast_rank=17) | leukocyte receptor cluster (LRC) member 9 [Homo sapiens] | [20.6](http://blast.ncbi.nlm.nih.gov/Blast.cgi#206725452%23206725452) | 20.6 | 50% | 111 |
| [NP_001070254.1](http://www.ncbi.nlm.nih.gov/entrez/query.fcgi?cmd=Retrieve&db=Protein&list_uids=115647981&dopt=GenPept&RID=T2RMG8CM014&log$=prottop&blast_rank=18) | glutamine and serine rich 1 [Homo sapiens] | [20.6](http://blast.ncbi.nlm.nih.gov/Blast.cgi#115647981%23115647981) | 20.6 | 60% | 111 |
| [NP_031375.3](http://www.ncbi.nlm.nih.gov/entrez/query.fcgi?cmd=Retrieve&db=Protein&list_uids=93141033&dopt=GenPept&RID=T2RMG8CM014&log$=prottop&blast_rank=19) | PAX interacting protein 1 [Homo sapiens] | [20.6](http://blast.ncbi.nlm.nih.gov/Blast.cgi#93141033%2393141033) | 20.6 | 100% | 111 |
| [NP_997722.1](http://www.ncbi.nlm.nih.gov/entrez/query.fcgi?cmd=Retrieve&db=Protein&list_uids=47086439&dopt=GenPept&RID=T2RMG8CM014&log$=prottop&blast_rank=20) | amelotin precursor [Homo sapiens] | [20.6](http://blast.ncbi.nlm.nih.gov/Blast.cgi#47086439%2347086439) | 20.6 | 50% | 111 |
| [NP_060591.3](http://www.ncbi.nlm.nih.gov/entrez/query.fcgi?cmd=Retrieve&db=Protein&list_uids=150456436&dopt=GenPept&RID=T2RMG8CM014&log$=prottop&blast_rank=21) | hypothetical protein LOC55719 isoform 1 [Homo sapiens] | [20.6](http://blast.ncbi.nlm.nih.gov/Blast.cgi#150456436%23150456436) | 20.6 | 50% | 111 |
| [NP_056155.1](http://www.ncbi.nlm.nih.gov/entrez/query.fcgi?cmd=Retrieve&db=Protein&list_uids=7661872&dopt=GenPept&RID=T2RMG8CM014&log$=prottop&blast_rank=22) | leucyl-tRNA synthetase 2, mitochondrial precursor [Homo sapiens] | [20.6](http://blast.ncbi.nlm.nih.gov/Blast.cgi#7661872%237661872) | 36.5 | 70% | 111 |
| [NP_631897.1](http://www.ncbi.nlm.nih.gov/entrez/query.fcgi?cmd=Retrieve&db=Protein&list_uids=21040235&dopt=GenPept&RID=T2RMG8CM014&log$=prottop&blast_rank=23) | cyclin-dependent kinase 15 [Homo sapiens] | [20.6](http://blast.ncbi.nlm.nih.gov/Blast.cgi#21040235%2321040235) | 32.7 | 70% | 111 |
| [NP_000403.1](http://www.ncbi.nlm.nih.gov/entrez/query.fcgi?cmd=Retrieve&db=Protein&list_uids=4504489&dopt=GenPept&RID=T2RMG8CM014&log$=prottop&blast_rank=24) | histidine-rich glycoprotein precursor [Homo sapiens] | [20.6](http://blast.ncbi.nlm.nih.gov/Blast.cgi#4504489%234504489) | 20.6 | 80% | 111 |
| [NP_001139134.1](http://www.ncbi.nlm.nih.gov/entrez/query.fcgi?cmd=Retrieve&db=Protein&list_uids=224611701&dopt=GenPept&RID=T2RMG8CM014&log$=prottop&blast_rank=25) | GATA binding protein 2 isoform 2 [Homo sapiens] | [20.2](http://blast.ncbi.nlm.nih.gov/Blast.cgi#224611701%23224611701) | 20.2 | 70% | 149 |
| [NP_116027.2](http://www.ncbi.nlm.nih.gov/entrez/query.fcgi?cmd=Retrieve&db=Protein&list_uids=20070352&dopt=GenPept&RID=T2RMG8CM014&log$=prottop&blast_rank=26) | GATA binding protein 2 isoform 1 [Homo sapiens] >ref|NP_001139133.1| GATA binding protein 2 isoform 1 [Homo sapiens] | [20.2](http://blast.ncbi.nlm.nih.gov/Blast.cgi#20070352%2320070352) | 20.2 | 70% | 149 |
| [NP_003322.3](http://www.ncbi.nlm.nih.gov/entrez/query.fcgi?cmd=Retrieve&db=Protein&list_uids=187608615&dopt=GenPept&RID=T2RMG8CM014&log$=prottop&blast_rank=27) | tyrosine kinase 2 [Homo sapiens] | [20.2](http://blast.ncbi.nlm.nih.gov/Blast.cgi#187608615%23187608615) | 20.2 | 60% | 149 |
| [NP_001158136.1](http://www.ncbi.nlm.nih.gov/entrez/query.fcgi?cmd=Retrieve&db=Protein&list_uids=257467648&dopt=GenPept&RID=T2RMG8CM014&log$=prottop&blast_rank=28) | microtubule associated serine/threonine kinase family member 4 isoform c [Homo sapiens] | [19.7](http://blast.ncbi.nlm.nih.gov/Blast.cgi#257467648%23257467648) | 19.7 | 60% | 200 |
| [NP_055998.1](http://www.ncbi.nlm.nih.gov/entrez/query.fcgi?cmd=Retrieve&db=Protein&list_uids=148727255&dopt=GenPept&RID=T2RMG8CM014&log$=prottop&blast_rank=29) | microtubule associated serine/threonine kinase family member 4 isoform a [Homo sapiens] | [19.7](http://blast.ncbi.nlm.nih.gov/Blast.cgi#148727255%23148727255) | 19.7 | 60% | 200 |
| [NP_059135.2](http://www.ncbi.nlm.nih.gov/entrez/query.fcgi?cmd=Retrieve&db=Protein&list_uids=148612805&dopt=GenPept&RID=T2RMG8CM014&log$=prottop&blast_rank=30) | pigeon homolog [Homo sapiens] | [19.7](http://blast.ncbi.nlm.nih.gov/Blast.cgi#148612805%23148612805) | 19.7 | 50% | 200 |
| [NP_001074016.1](http://www.ncbi.nlm.nih.gov/entrez/query.fcgi?cmd=Retrieve&db=Protein&list_uids=124028521&dopt=GenPept&RID=T2RMG8CM014&log$=prottop&blast_rank=31) | hematopoietic transcription factor PU.1 isoform 1 [Homo sapiens] | [19.7](http://blast.ncbi.nlm.nih.gov/Blast.cgi#124028521%23124028521) | 19.7 | 70% | 200 |
| [NP_787057.2](http://www.ncbi.nlm.nih.gov/entrez/query.fcgi?cmd=Retrieve&db=Protein&list_uids=83415184&dopt=GenPept&RID=T2RMG8CM014&log$=prottop&blast_rank=32) | transmembrane and tetratricopeptide repeat containing 1 [Homo sapiens] | [19.7](http://blast.ncbi.nlm.nih.gov/Blast.cgi#83415184%2383415184) | 33.1 | 70% | 200 |
| [NP_001013757.1](http://www.ncbi.nlm.nih.gov/entrez/query.fcgi?cmd=Retrieve&db=Protein&list_uids=61966923&dopt=GenPept&RID=T2RMG8CM014&log$=prottop&blast_rank=33) | forkhead box B2 [Homo sapiens] | [19.7](http://blast.ncbi.nlm.nih.gov/Blast.cgi#61966923%2361966923) | 19.7 | 100% | 200 |
[truncated: 229,757 more chars]
