# Supplementary material for: A high throughput method for identifying personalized tumor-associated antigens
Source: Oncotarget. 2010 Jun 27;1(2):148–55. doi: 10.18632/oncotarget.118 (PMC2920534; doi:10.18632/oncotarget.118)
Supplement: Supplemental Table 6 [file oncotarget-01-148-s006.doc]

**IgM Patient D**

| **Accession** | **Proteins with a match to QSLDHSSC peptide** | **[Max score](http://blast.ncbi.nlm.nih.gov/Blast.cgi?CMD=Get&ALIGNMENTS=100&ALIGNMENT_VIEW=Pairwise&CDD_SEARCH_STATE=1&DATABASE_SORT=0&DESCRIPTIONS=100&ENTREZ_QUERY=txid9606 %5BORGN%5D&FIRST_QUERY_NUM=0&FORMAT_OBJECT=Alignment&FORMAT_PAGE_TARGET=&FORMAT_TYPE=HTML&GET_SEQUENCE=yes&I_THRESH=&MASK_CHAR=2&MASK_COLOR=1&NEW_DESIGN=on&NEW_VIEW=yes&NUM_OVERVIEW=100&OLD_BLAST=false&PAGE=Proteins&QUERY_INDEX=0&QUERY_NUMBER=0&RESULTS_PAGE_TARGET=&RID=T2WHBG7501N&SHOW_LINKOUT=yes&SHOW_OVERVIEW=yes&STEP_NUMBER=&WORD_SIZE=2&DISPLAY_SORT=1&HSP_SORT=1" \l "sort_mark)** | **[Total score](http://blast.ncbi.nlm.nih.gov/Blast.cgi?CMD=Get&ALIGNMENTS=100&ALIGNMENT_VIEW=Pairwise&CDD_SEARCH_STATE=1&DATABASE_SORT=0&DESCRIPTIONS=100&ENTREZ_QUERY=txid9606 %5BORGN%5D&FIRST_QUERY_NUM=0&FORMAT_OBJECT=Alignment&FORMAT_PAGE_TARGET=&FORMAT_TYPE=HTML&GET_SEQUENCE=yes&I_THRESH=&MASK_CHAR=2&MASK_COLOR=1&NEW_DESIGN=on&NEW_VIEW=yes&NUM_OVERVIEW=100&OLD_BLAST=false&PAGE=Proteins&QUERY_INDEX=0&QUERY_NUMBER=0&RESULTS_PAGE_TARGET=&RID=T2WHBG7501N&SHOW_LINKOUT=yes&SHOW_OVERVIEW=yes&STEP_NUMBER=&WORD_SIZE=2&DISPLAY_SORT=2&HSP_SORT=1" \l "sort_mark)** | **[Query coverage](http://blast.ncbi.nlm.nih.gov/Blast.cgi?CMD=Get&ALIGNMENTS=100&ALIGNMENT_VIEW=Pairwise&CDD_SEARCH_STATE=1&DATABASE_SORT=0&DESCRIPTIONS=100&ENTREZ_QUERY=txid9606 %5BORGN%5D&FIRST_QUERY_NUM=0&FORMAT_OBJECT=Alignment&FORMAT_PAGE_TARGET=&FORMAT_TYPE=HTML&GET_SEQUENCE=yes&I_THRESH=&MASK_CHAR=2&MASK_COLOR=1&NEW_DESIGN=on&NEW_VIEW=yes&NUM_OVERVIEW=100&OLD_BLAST=false&PAGE=Proteins&QUERY_INDEX=0&QUERY_NUMBER=0&RESULTS_PAGE_TARGET=&RID=T2WHBG7501N&SHOW_LINKOUT=yes&SHOW_OVERVIEW=yes&STEP_NUMBER=&WORD_SIZE=2&DISPLAY_SORT=4&HSP_SORT=0" \l "sort_mark)** | **[E value](http://blast.ncbi.nlm.nih.gov/Blast.cgi?CMD=Get&ALIGNMENTS=100&ALIGNMENT_VIEW=Pairwise&CDD_SEARCH_STATE=1&DATABASE_SORT=0&DESCRIPTIONS=100&ENTREZ_QUERY=txid9606 %5BORGN%5D&FIRST_QUERY_NUM=0&FORMAT_OBJECT=Alignment&FORMAT_PAGE_TARGET=&FORMAT_TYPE=HTML&GET_SEQUENCE=yes&I_THRESH=&MASK_CHAR=2&MASK_COLOR=1&NEW_DESIGN=on&NEW_VIEW=yes&NUM_OVERVIEW=100&OLD_BLAST=false&PAGE=Proteins&QUERY_INDEX=0&QUERY_NUMBER=0&RESULTS_PAGE_TARGET=&RID=T2WHBG7501N&SHOW_LINKOUT=yes&SHOW_OVERVIEW=yes&STEP_NUMBER=&WORD_SIZE=2&DISPLAY_SORT=0&HSP_SORT=0" \l "sort_mark)** |
| --- | --- | --- | --- | --- | --- |
| [XP_002347226.1](http://www.ncbi.nlm.nih.gov/entrez/query.fcgi?cmd=Retrieve&db=Protein&list_uids=239749951&dopt=GenPept&RID=T2WHBG7501N&log$=prottop&blast_rank=1) | PREDICTED: similar to mucin 2 [Homo sapiens] | [21.8](http://blast.ncbi.nlm.nih.gov/Blast.cgi#239749951%23239749951) | 21.8 | 100% | 37 |
| [NP_002448.2](http://www.ncbi.nlm.nih.gov/entrez/query.fcgi?cmd=Retrieve&db=Protein&list_uids=116284392&dopt=GenPept&RID=T2WHBG7501N&log$=prottop&blast_rank=2) | mucin 2 precursor [Homo sapiens] | [21.8](http://blast.ncbi.nlm.nih.gov/Blast.cgi#116284392%23116284392) | 21.8 | 100% | 37 |
| [NP_065681.1](http://www.ncbi.nlm.nih.gov/entrez/query.fcgi?cmd=Retrieve&db=Protein&list_uids=10862701&dopt=GenPept&RID=T2WHBG7501N&log$=prottop&blast_rank=3) | ret proto-oncogene isoform c [Homo sapiens] | [21.0](http://blast.ncbi.nlm.nih.gov/Blast.cgi#10862701%2310862701) | 36.9 | 75% | 66 |
| [NP_689804.1](http://www.ncbi.nlm.nih.gov/entrez/query.fcgi?cmd=Retrieve&db=Protein&list_uids=22749217&dopt=GenPept&RID=T2WHBG7501N&log$=prottop&blast_rank=4) | coiled-coil domain containing 63 [Homo sapiens] | [21.0](http://blast.ncbi.nlm.nih.gov/Blast.cgi#22749217%2322749217) | 21.0 | 87% | 66 |
| [NP_116755.1](http://www.ncbi.nlm.nih.gov/entrez/query.fcgi?cmd=Retrieve&db=Protein&list_uids=14589946&dopt=GenPept&RID=T2WHBG7501N&log$=prottop&blast_rank=5) | protocadherin 11 Y-linked isoform c [Homo sapiens] | [21.0](http://blast.ncbi.nlm.nih.gov/Blast.cgi#14589946%2314589946) | 21.0 | 75% | 66 |
| [NP_116751.1](http://www.ncbi.nlm.nih.gov/entrez/query.fcgi?cmd=Retrieve&db=Protein&list_uids=14589922&dopt=GenPept&RID=T2WHBG7501N&log$=prottop&blast_rank=6) | protocadherin 11 X-linked isoform d precursor [Homo sapiens] | [21.0](http://blast.ncbi.nlm.nih.gov/Blast.cgi#14589922%2314589922) | 21.0 | 75% | 66 |
| [NP_116750.1](http://www.ncbi.nlm.nih.gov/entrez/query.fcgi?cmd=Retrieve&db=Protein&list_uids=14589920&dopt=GenPept&RID=T2WHBG7501N&log$=prottop&blast_rank=7) | protocadherin 11 X-linked isoform c precursor [Homo sapiens] | [21.0](http://blast.ncbi.nlm.nih.gov/Blast.cgi#14589920%2314589920) | 21.0 | 75% | 66 |
| [NP_001161835.1](http://www.ncbi.nlm.nih.gov/entrez/query.fcgi?cmd=Retrieve&db=Protein&list_uids=270288731&dopt=GenPept&RID=T2WHBG7501N&log$=prottop&blast_rank=8) | protocadherin 11 X-linked isoform h precursor [Homo sapiens] | [21.0](http://blast.ncbi.nlm.nih.gov/Blast.cgi#270288731%23270288731) | 21.0 | 75% | 66 |
| [NP_001161832.1](http://www.ncbi.nlm.nih.gov/entrez/query.fcgi?cmd=Retrieve&db=Protein&list_uids=270288725&dopt=GenPept&RID=T2WHBG7501N&log$=prottop&blast_rank=9) | protocadherin 11 X-linked isoform e precursor [Homo sapiens] | [21.0](http://blast.ncbi.nlm.nih.gov/Blast.cgi#270288725%23270288725) | 21.0 | 75% | 66 |
| [NP_001161834.1](http://www.ncbi.nlm.nih.gov/entrez/query.fcgi?cmd=Retrieve&db=Protein&list_uids=270288729&dopt=GenPept&RID=T2WHBG7501N&log$=prottop&blast_rank=10) | protocadherin 11 X-linked isoform g precursor [Homo sapiens] | [21.0](http://blast.ncbi.nlm.nih.gov/Blast.cgi#270288729%23270288729) | 21.0 | 75% | 66 |
| [NP_066124.1](http://www.ncbi.nlm.nih.gov/entrez/query.fcgi?cmd=Retrieve&db=Protein&list_uids=10862703&dopt=GenPept&RID=T2WHBG7501N&log$=prottop&blast_rank=11) | ret proto-oncogene isoform a [Homo sapiens] | [21.0](http://blast.ncbi.nlm.nih.gov/Blast.cgi#10862703%2310862703) | 36.9 | 75% | 66 |
| [NP_659444.2](http://www.ncbi.nlm.nih.gov/entrez/query.fcgi?cmd=Retrieve&db=Protein&list_uids=194018484&dopt=GenPept&RID=T2WHBG7501N&log$=prottop&blast_rank=12) | NLR family, pyrin domain containing 11 [Homo sapiens] | [20.2](http://blast.ncbi.nlm.nih.gov/Blast.cgi#194018484%23194018484) | 20.2 | 75% | 119 |
| [NP_872337.2](http://www.ncbi.nlm.nih.gov/entrez/query.fcgi?cmd=Retrieve&db=Protein&list_uids=153792259&dopt=GenPept&RID=T2WHBG7501N&log$=prottop&blast_rank=13) | zinc finger protein 778 [Homo sapiens] | [20.2](http://blast.ncbi.nlm.nih.gov/Blast.cgi#153792259%23153792259) | 20.2 | 87% | 119 |
| [NP_620688.2](http://www.ncbi.nlm.nih.gov/entrez/query.fcgi?cmd=Retrieve&db=Protein&list_uids=110611170&dopt=GenPept&RID=T2WHBG7501N&log$=prottop&blast_rank=14) | ADAM metallopeptidase with thrombospondin type 1 motif, 17 preproprotein [Homo sapiens] | [19.7](http://blast.ncbi.nlm.nih.gov/Blast.cgi#110611170%23110611170) | 19.7 | 62% | 160 |
| [NP_061176.3](http://www.ncbi.nlm.nih.gov/entrez/query.fcgi?cmd=Retrieve&db=Protein&list_uids=38788380&dopt=GenPept&RID=T2WHBG7501N&log$=prottop&blast_rank=15) | dehydrogenase E1 and transketolase domain containing 1 precursor [Homo sapiens] | [19.7](http://blast.ncbi.nlm.nih.gov/Blast.cgi#38788380%2338788380) | 19.7 | 62% | 160 |
| [NP_001123560.1](http://www.ncbi.nlm.nih.gov/entrez/query.fcgi?cmd=Retrieve&db=Protein&list_uids=194272207&dopt=GenPept&RID=T2WHBG7501N&log$=prottop&blast_rank=16) | actin binding LIM protein family, member 2 isoform 7 [Homo sapiens] | [19.3](http://blast.ncbi.nlm.nih.gov/Blast.cgi#194272207%23194272207) | 19.3 | 87% | 214 |
| [NP_001123559.1](http://www.ncbi.nlm.nih.gov/entrez/query.fcgi?cmd=Retrieve&db=Protein&list_uids=194272204&dopt=GenPept&RID=T2WHBG7501N&log$=prottop&blast_rank=17) | actin binding LIM protein family, member 2 isoform 5 [Homo sapiens] | [19.3](http://blast.ncbi.nlm.nih.gov/Blast.cgi#194272204%23194272204) | 19.3 | 87% | 214 |
| [NP_001123555.1](http://www.ncbi.nlm.nih.gov/entrez/query.fcgi?cmd=Retrieve&db=Protein&list_uids=194272196&dopt=GenPept&RID=T2WHBG7501N&log$=prottop&blast_rank=18) | actin binding LIM protein family, member 2 isoform 1 [Homo sapiens] | [19.3](http://blast.ncbi.nlm.nih.gov/Blast.cgi#194272196%23194272196) | 19.3 | 87% | 214 |
| [XP_001719784.1](http://www.ncbi.nlm.nih.gov/entrez/query.fcgi?cmd=Retrieve&db=Protein&list_uids=169168890&dopt=GenPept&RID=T2WHBG7501N&log$=prottop&blast_rank=19) | PREDICTED: hypothetical protein LOC441172 [Homo sapiens] >ref|XP_001717931.1| PREDICTED: hypothetical protein LOC441172 [Homo sapiens] | [19.3](http://blast.ncbi.nlm.nih.gov/Blast.cgi#169168890%23169168890) | 19.3 | 62% | 214 |
| [NP_115808.3](http://www.ncbi.nlm.nih.gov/entrez/query.fcgi?cmd=Retrieve&db=Protein&list_uids=153792514&dopt=GenPept&RID=T2WHBG7501N&log$=prottop&blast_rank=20) | actin binding LIM protein family, member 2 isoform 6 [Homo sapiens] | [19.3](http://blast.ncbi.nlm.nih.gov/Blast.cgi#153792514%23153792514) | 19.3 | 87% | 214 |
| [NP_001123558.1](http://www.ncbi.nlm.nih.gov/entrez/query.fcgi?cmd=Retrieve&db=Protein&list_uids=194272202&dopt=GenPept&RID=T2WHBG7501N&log$=prottop&blast_rank=21) | actin binding LIM protein family, member 2 isoform 4 [Homo sapiens] | [19.3](http://blast.ncbi.nlm.nih.gov/Blast.cgi#194272202%23194272202) | 19.3 | 87% | 214 |
| [NP_004487.2](http://www.ncbi.nlm.nih.gov/entrez/query.fcgi?cmd=Retrieve&db=Protein&list_uids=24497501&dopt=GenPept&RID=T2WHBG7501N&log$=prottop&blast_rank=22) | forkhead box A1 [Homo sapiens] | [19.3](http://blast.ncbi.nlm.nih.gov/Blast.cgi#24497501%2324497501) | 19.3 | 75% | 214 |
| [NP_000746.2](http://www.ncbi.nlm.nih.gov/entrez/query.fcgi?cmd=Retrieve&db=Protein&list_uids=21618331&dopt=GenPept&RID=T2WHBG7501N&log$=prottop&blast_rank=23) | carnitine acetyltransferase precursor [Homo sapiens] | [19.3](http://blast.ncbi.nlm.nih.gov/Blast.cgi#21618331%2321618331) | 19.3 | 62% | 214 |
| [NP_001108446.1](http://www.ncbi.nlm.nih.gov/entrez/query.fcgi?cmd=Retrieve&db=Protein&list_uids=169234647&dopt=GenPept&RID=T2WHBG7501N&log$=prottop&blast_rank=24) | smoothelin-like 2 isoform 1 [Homo sapiens] | [19.3](http://blast.ncbi.nlm.nih.gov/Blast.cgi#169234647%23169234647) | 19.3 | 62% | 214 |
| [NP_001123557.1](http://www.ncbi.nlm.nih.gov/entrez/query.fcgi?cmd=Retrieve&db=Protein&list_uids=194272200&dopt=GenPept&RID=T2WHBG7501N&log$=prottop&blast_rank=25) | actin binding LIM protein family, member 2 isoform 3 [Homo sapiens] | [19.3](http://blast.ncbi.nlm.nih.gov/Blast.cgi#194272200%23194272200) | 19.3 | 87% | 214 |
| [NP_001123556.1](http://www.ncbi.nlm.nih.gov/entrez/query.fcgi?cmd=Retrieve&db=Protein&list_uids=194272198&dopt=GenPept&RID=T2WHBG7501N&log$=prottop&blast_rank=26) | actin binding LIM protein family, member 2 isoform 2 [Homo sapiens] | [19.3](http://blast.ncbi.nlm.nih.gov/Blast.cgi#194272198%23194272198) | 19.3 | 87% | 214 |
| [NP_620594.1](http://www.ncbi.nlm.nih.gov/entrez/query.fcgi?cmd=Retrieve&db=Protein&list_uids=21265034&dopt=GenPept&RID=T2WHBG7501N&log$=prottop&blast_rank=27) | ADAM metallopeptidase with thrombospondin type 1 motif, 13 isoform 1 preproprotein [Homo sapiens] | [19.3](http://blast.ncbi.nlm.nih.gov/Blast.cgi#21265034%2321265034) | 19.3 | 75% | 214 |
| [NP_620596.2](http://www.ncbi.nlm.nih.gov/entrez/query.fcgi?cmd=Retrieve&db=Protein&list_uids=73695936&dopt=GenPept&RID=T2WHBG7501N&log$=prottop&blast_rank=28) | ADAM metallopeptidase with thrombospondin type 1 motif, 13 isoform 2 preproprotein [Homo sapiens] | [19.3](http://blast.ncbi.nlm.nih.gov/Blast.cgi#73695936%2373695936) | 19.3 | 75% | 214 |
| [NP_001339.1](http://www.ncbi.nlm.nih.gov/entrez/query.fcgi?cmd=Retrieve&db=Protein&list_uids=4557511&dopt=GenPept&RID=T2WHBG7501N&log$=prottop&blast_rank=29) | death-associated protein kinase 3 [Homo sapiens] | [19.3](http://blast.ncbi.nlm.nih.gov/Blast.cgi#4557511%234557511) | 19.3 | 75% | 214 |
| [NP_620595.1](http://www.ncbi.nlm.nih.gov/entrez/query.fcgi?cmd=Retrieve&db=Protein&list_uids=21265043&dopt=GenPept&RID=T2WHBG7501N&log$=prottop&blast_rank=30) | ADAM metallopeptidase with thrombospondin type 1 motif, 13 isoform 3 preproprotein [Homo sapiens] | [19.3](http://blast.ncbi.nlm.nih.gov/Blast.cgi#21265043%2321265043) | 19.3 | 75% | 214 |
| [NP_001157018.1](http://www.ncbi.nlm.nih.gov/entrez/query.fcgi?cmd=Retrieve&db=Protein&list_uids=254587992&dopt=GenPept&RID=T2WHBG7501N&log$=prottop&blast_rank=31) | synergin, gamma isoform 6 [Homo sapiens] | [18.9](http://blast.ncbi.nlm.nih.gov/Blast.cgi#254587992%23254587992) | 18.9 | 87% | 288 |
| [NP_942583.1](http://www.ncbi.nlm.nih.gov/entrez/query.fcgi?cmd=Retrieve&db=Protein&list_uids=254587986&dopt=GenPept&RID=T2WHBG7501N&log$=prottop&blast_rank=32) | synergin, gamma isoform 3 [Homo sapiens] | [18.9](http://blast.ncbi.nlm.nih.gov/Blast.cgi#254587986%23254587986) | 18.9 | 87% | 288 |
| [NP_001157016.1](http://www.ncbi.nlm.nih.gov/entrez/query.fcgi?cmd=Retrieve&db=Protein&list_uids=254587988&dopt=GenPept&RID=T2WHBG7501N&log$=prottop&blast_rank=33) | synergin, gamma isoform 4 [Homo sapiens] | [18.9](http://blast.ncbi.nlm.nih.gov/Blast.cgi#254587988%23254587988) | 18.9 | 87% | 288 |
| [NP_001157019.1](http://www.ncbi.nlm.nih.gov/entrez/query.fcgi?cmd=Retrieve&db=Protein&list_uids=254587994&dopt=GenPept&RID=T2WHBG7501N&log$=prottop&blast_rank=34) | synergin, gamma isoform 7 [Homo sapiens] | [18.9](http://blast.ncbi.nlm.nih.gov/Blast.cgi#254587994%23254587994) | 18.9 | 87% | 288 |
| [NP_004794.2](http://www.ncbi.nlm.nih.gov/entrez/query.fcgi?cmd=Retrieve&db=Protein&list_uids=157426885&dopt=GenPept&RID=T2WHBG7501N&log$=prottop&blast_rank=35) | organic cation transporter like 4 [Homo sapiens] | [18.9](http://blast.ncbi.nlm.nih.gov/Blast.cgi#157426885%23157426885) | 18.9 | 87% | 288 |
| [NP_001157017.1](http://www.ncbi.nlm.nih.gov/entrez/query.fcgi?cmd=Retrieve&db=Protein&list_uids=254587990&dopt=GenPept&RID=T2WHBG7501N&log$=prottop&blast_rank=36) | synergin, gamma isoform 5 [Homo sapiens] | [18.9](http://blast.ncbi.nlm.nih.gov/Blast.cgi#254587990%23254587990) | 18.9 | 87% | 288 |
| [NP_001002759.1](http://www.ncbi.nlm.nih.gov/entrez/query.fcgi?cmd=Retrieve&db=Protein&list_uids=50593526&dopt=GenPept&RID=T2WHBG7501N&log$=prottop&blast_rank=37) | hypothetical protein LOC119392 isoform a [Homo sapiens] | [18.9](http://blast.ncbi.nlm.nih.gov/Blast.cgi#50593526%2350593526) | 18.9 | 100% | 288 |
| [NP_009178.3](http://www.ncbi.nlm.nih.gov/entrez/query.fcgi?cmd=Retrieve&db=Protein&list_uids=38569409&dopt=GenPept&RID=T2WHBG7501N&log$=prottop&blast_rank=38) | synergin, gamma isoform 1 [Homo sapiens] | [18.9](http://blast.ncbi.nlm.nih.gov/Blast.cgi#38569409%2338569409) | 18.9 | 87% | 288 |
| [NP_660290.3](http://www.ncbi.nlm.nih.gov/entrez/query.fcgi?cmd=Retrieve&db=Protein&list_uids=50593524&dopt=GenPept&RID=T2WHBG7501N&log$=prottop&blast_rank=39) | hypothetical protein LOC119392 isoform b [Homo sapiens] | [18.9](http://blast.ncbi.nlm.nih.gov/Blast.cgi#50593524%2350593524) | 18.9 | 100% | 288 |
| [NP_542117.3](http://www.ncbi.nlm.nih.gov/entrez/query.fcgi?cmd=Retrieve&db=Protein&list_uids=254587984&dopt=GenPept&RID=T2WHBG7501N&log$=prottop&blast_rank=40) | synergin, gamma isoform 2 [Homo sapiens] | [18.9](http://blast.ncbi.nlm.nih.gov/Blast.cgi#254587984%23254587984) | 18.9 | 87% | 288 |
| [NP_001165124.1](http://www.ncbi.nlm.nih.gov/entrez/query.fcgi?cmd=Retrieve&db=Protein&list_uids=284413746&dopt=GenPept&RID=T2WHBG7501N&log$=prottop&blast_rank=41) | zinc finger homeobox 1b isoform 2 [Homo sapiens] | [18.5](http://blast.ncbi.nlm.nih.gov/Blast.cgi#284413746%23284413746) | 48.2 | 75% | 386 |
| [NP_001139736.1](http://www.ncbi.nlm.nih.gov/entrez/query.fcgi?cmd=Retrieve&db=Protein&list_uids=226342962&dopt=GenPept&RID=T2WHBG7501N&log$=prottop&blast_rank=42) | synaptotagmin XIV isoform 2 [Homo sapiens] | [18.5](http://blast.ncbi.nlm.nih.gov/Blast.cgi#226342962%23226342962) | 18.5 | 87% | 386 |
| [NP_001139733.1](http://www.ncbi.nlm.nih.gov/entrez/query.fcgi?cmd=Retrieve&db=Protein&list_uids=226342955&dopt=GenPept&RID=T2WHBG7501N&log$=prottop&blast_rank=43) | synaptotagmin XIV isoform 1 [Homo sapiens] | [18.5](http://blast.ncbi.nlm.nih.gov/Blast.cgi#226342955%23226342955) | 18.5 | 87% | 386 |
| [NP_001139734.1](http://www.ncbi.nlm.nih.gov/entrez/query.fcgi?cmd=Retrieve&db=Protein&list_uids=226342957&dopt=GenPept&RID=T2WHBG7501N&log$=prottop&blast_rank=44) | synaptotagmin XIV isoform 3 [Homo sapiens] | [18.5](http://blast.ncbi.nlm.nih.gov/Blast.cgi#226342957%23226342957) | 18.5 | 87% | 386 |
| [NP_001035351.3](http://www.ncbi.nlm.nih.gov/entrez/query.fcgi?cmd=Retrieve&db=Protein&list_uids=156713430&dopt=GenPept&RID=T2WHBG7501N&log$=prottop&blast_rank=45) | doublecortin-like kinase 2 isoform b [Homo sapiens] | [18.5](http://blast.ncbi.nlm.nih.gov/Blast.cgi#156713430%23156713430) | 18.5 | 75% | 386 |
| [NP_001035350.2](http://www.ncbi.nlm.nih.gov/entrez/query.fcgi?cmd=Retrieve&db=Protein&list_uids=156713428&dopt=GenPept&RID=T2WHBG7501N&log$=prottop&blast_rank=46) | doublecortin-like kinase 2 isoform a [Homo sapiens] | [18.5](http://blast.ncbi.nlm.nih.gov/Blast.cgi#156713428%23156713428) | 18.5 | 75% | 386 |
| [NP_001034852.2](http://www.ncbi.nlm.nih.gov/entrez/query.fcgi?cmd=Retrieve&db=Protein&list_uids=193290174&dopt=GenPept&RID=T2WHBG7501N&log$=prottop&blast_rank=47) | transmembrane protein 232 [Homo sapiens] | [18.5](http://blast.ncbi.nlm.nih.gov/Blast.cgi#193290174%23193290174) | 18.5 | 75% | 386 |
| [NP_055912.1](http://www.ncbi.nlm.nih.gov/entrez/query.fcgi?cmd=Retrieve&db=Protein&list_uids=57863301&dopt=GenPept&RID=T2WHBG7501N&log$=prottop&blast_rank=48) | CLIP-associating protein 2 [Homo sapiens] | [18.5](http://blast.ncbi.nlm.nih.gov/Blast.cgi#57863301%2357863301) | 28.4 | 62% | 386 |
| [NP_694994.2](http://www.ncbi.nlm.nih.gov/entrez/query.fcgi?cmd=Retrieve&db=Protein&list_uids=144226225&dopt=GenPept&RID=T2WHBG7501N&log$=prottop&blast_rank=49) | synaptotagmin XIV isoform 4 [Homo sapiens] | [18.5](http://blast.ncbi.nlm.nih.gov/Blast.cgi#144226225%23144226225) | 18.5 | 87% | 386 |
| [NP_060749.2](http://www.ncbi.nlm.nih.gov/entrez/query.fcgi?cmd=Retrieve&db=Protein&list_uids=21361720&dopt=GenPept&RID=T2WHBG7501N&log$=prottop&blast_rank=50) | transmembrane protein 19 [Homo sapiens] | [18.5](http://blast.ncbi.nlm.nih.gov/Blast.cgi#21361720%2321361720) | 18.5 | 62% | 386 |
| [NP_663722.1](http://www.ncbi.nlm.nih.gov/entrez/query.fcgi?cmd=Retrieve&db=Protein&list_uids=22035554&dopt=GenPept&RID=T2WHBG7501N&log$=prottop&blast_rank=51) | amyloid beta A4 precursor protein-binding, family B, member 1 isoform delta E9 [Homo sapiens] | [18.5](http://blast.ncbi.nlm.nih.gov/Blast.cgi#22035554%2322035554) | 18.5 | 62% | 386 |
| [XP_931066.1](http://www.ncbi.nlm.nih.gov/entrez/query.fcgi?cmd=Retrieve&db=Protein&list_uids=89030918&dopt=GenPept&RID=T2WHBG7501N&log$=prottop&blast_rank=52) | PREDICTED: similar to hCG1988827 [Homo sapiens] >ref|XP_001715124.1| PREDICTED: similar to hCG1988827 [Homo sapiens] | [18.5](http://blast.ncbi.nlm.nih.gov/Blast.cgi#89030918%2389030918) | 18.5 | 62% | 386 |
| [NP_689659.2](http://www.ncbi.nlm.nih.gov/entrez/query.fcgi?cmd=Retrieve&db=Protein&list_uids=64085252&dopt=GenPept&RID=T2WHBG7501N&log$=prottop&blast_rank=53) | hypothetical protein LOC145508 [Homo sapiens] | [18.5](http://blast.ncbi.nlm.nih.gov/Blast.cgi#64085252%2364085252) | 18.5 | 62% | 386 |
| [NP_115820.2](http://www.ncbi.nlm.nih.gov/entrez/query.fcgi?cmd=Retrieve&db=Protein&list_uids=63252863&dopt=GenPept&RID=T2WHBG7501N&log$=prottop&blast_rank=54) | BTB (POZ) domain containing 12 [Homo sapiens] | [18.5](http://blast.ncbi.nlm.nih.gov/Blast.cgi#63252863%2363252863) | 45.6 | 100% | 386 |
| [NP_000288.1](http://www.ncbi.nlm.nih.gov/entrez/query.fcgi?cmd=Retrieve&db=Protein&list_uids=4505835&dopt=GenPept&RID=T2WHBG7501N&log$=prottop&blast_rank=55) | polycystin 2 [Homo sapiens] | [18.5](http://blast.ncbi.nlm.nih.gov/Blast.cgi#4505835%234505835) | 18.5 | 62% | 386 |
| [NP_150375.2](http://www.ncbi.nlm.nih.gov/entrez/query.fcgi?cmd=Retrieve&db=Protein&list_uids=27886653&dopt=GenPept&RID=T2WHBG7501N&log$=prottop&blast_rank=56) | potassium voltage-gated channel, subfamily H, member 7 isoform 1 [Homo sapiens] | [18.5](http://blast.ncbi.nlm.nih.gov/Blast.cgi#27886653%2327886653) | 18.5 | 62% | 386 |
| [NP_775185.1](http://www.ncbi.nlm.nih.gov/entrez/query.fcgi?cmd=Retrieve&db=Protein&list_uids=27886665&dopt=GenPept&RID=T2WHBG7501N&log$=prottop&blast_rank=57) | potassium voltage-gated channel, subfamily H, member 7 isoform 2 [Homo sapiens] | [18.5](http://blast.ncbi.nlm.nih.gov/Blast.cgi#27886665%2327886665) | 18.5 | 62% | 386 |
| [NP_000912.3](http://www.ncbi.nlm.nih.gov/entrez/query.fcgi?cmd=Retrieve&db=Protein&list_uids=70608155&dopt=GenPept&RID=T2WHBG7501N&log$=prottop&blast_rank=58) | phosphodiesterase 3A [Homo sapiens] | [18.5](http://blast.ncbi.nlm.nih.gov/Blast.cgi#70608155%2370608155) | 18.5 | 87% | 386 |
| [NP_055610.1](http://www.ncbi.nlm.nih.gov/entrez/query.fcgi?cmd=Retrieve&db=Protein&list_uids=7662184&dopt=GenPept&RID=T2WHBG7501N&log$=prottop&blast_rank=59) | zinc finger homeobox 1b isoform 1 [Homo sapiens] | [18.5](http://blast.ncbi.nlm.nih.gov/Blast.cgi#7662184%237662184) | 48.2 | 75% | 386 |
| [NP_004985.2](http://www.ncbi.nlm.nih.gov/entrez/query.fcgi?cmd=Retrieve&db=Protein&list_uids=74272287&dopt=GenPept&RID=T2WHBG7501N&log$=prottop&blast_rank=60) | matrix metalloproteinase 9 preproprotein [Homo sapiens] | [18.5](http://blast.ncbi.nlm.nih.gov/Blast.cgi#74272287%2374272287) | 18.5 | 62% | 386 |
| [NP_001907.2](http://www.ncbi.nlm.nih.gov/entrez/query.fcgi?cmd=Retrieve&db=Protein&list_uids=21359867&dopt=GenPept&RID=T2WHBG7501N&log$=prottop&blast_rank=61) | cytochrome c-1 [Homo sapiens] | [18.5](http://blast.ncbi.nlm.nih.gov/Blast.cgi#21359867%2321359867) | 18.5 | 75% | 386 |
| [NP_005460.2](http://www.ncbi.nlm.nih.gov/entrez/query.fcgi?cmd=Retrieve&db=Protein&list_uids=34577075&dopt=GenPept&RID=T2WHBG7501N&log$=prottop&blast_rank=62) | peroxisomal acyl-CoA thioesterase 1 isoform a [Homo sapiens] | [18.5](http://blast.ncbi.nlm.nih.gov/Blast.cgi#34577075%2334577075) | 18.5 | 62% | 386 |
| [NP_057472.2](http://www.ncbi.nlm.nih.gov/entrez/query.fcgi?cmd=Retrieve&db=Protein&list_uids=54607137&dopt=GenPept&RID=T2WHBG7501N&log$=prottop&blast_rank=63) | T-cell receptor interacting molecule [Homo sapiens] | [18.5](http://blast.ncbi.nlm.nih.gov/Blast.cgi#54607137%2354607137) | 18.5 | 62% | 386 |
| [NP_001155.1](http://www.ncbi.nlm.nih.gov/entrez/query.fcgi?cmd=Retrieve&db=Protein&list_uids=4502131&dopt=GenPept&RID=T2WHBG7501N&log$=prottop&blast_rank=64) | amyloid beta A4 precursor protein-binding, family B, member 1 isoform E9 [Homo sapiens] | [18.5](http://blast.ncbi.nlm.nih.gov/Blast.cgi#4502131%234502131) | 18.5 | 62% | 386 |
| [NP_058649.3](http://www.ncbi.nlm.nih.gov/entrez/query.fcgi?cmd=Retrieve&db=Protein&list_uids=116536085&dopt=GenPept&RID=T2WHBG7501N&log$=prottop&blast_rank=65) | phosphodiesterase 11A isoform 4 [Homo sapiens] | [18.0](http://blast.ncbi.nlm.nih.gov/Blast.cgi#116536085%23116536085) | 18.0 | 87% | 518 |
| [NP_078879.2](http://www.ncbi.nlm.nih.gov/entrez/query.fcgi?cmd=Retrieve&db=Protein&list_uids=187828564&dopt=GenPept&RID=T2WHBG7501N&log$=prottop&blast_rank=66) | BEN domain containing 5 [Homo sapiens] | [18.0](http://blast.ncbi.nlm.nih.gov/Blast.cgi#187828564%23187828564) | 31.4 | 87% | 518 |
| [NP_005824.2](http://www.ncbi.nlm.nih.gov/entrez/query.fcgi?cmd=Retrieve&db=Protein&list_uids=33695109&dopt=GenPept&RID=T2WHBG7501N&log$=prottop&blast_rank=67) | Rab9 effector protein with kelch motifs [Homo sapiens] | [18.0](http://blast.ncbi.nlm.nih.gov/Blast.cgi#33695109%2333695109) | 18.0 | 75% | 518 |
| [NP_001010867.1](http://www.ncbi.nlm.nih.gov/entrez/query.fcgi?cmd=Retrieve&db=Protein&list_uids=58197556&dopt=GenPept&RID=T2WHBG7501N&log$=prottop&blast_rank=68) | C1orf69 precursor [Homo sapiens] | [18.0](http://blast.ncbi.nlm.nih.gov/Blast.cgi#58197556%2358197556) | 18.0 | 75% | 518 |
| [NP_631908.1](http://www.ncbi.nlm.nih.gov/entrez/query.fcgi?cmd=Retrieve&db=Protein&list_uids=21040257&dopt=GenPept&RID=T2WHBG7501N&log$=prottop&blast_rank=69) | TruB pseudouridine (psi) synthase homolog 1 [Homo sapiens] | [18.0](http://blast.ncbi.nlm.nih.gov/Blast.cgi#21040257%2321040257) | 18.0 | 87% | 518 |
| [NP_612401.2](http://www.ncbi.nlm.nih.gov/entrez/query.fcgi?cmd=Retrieve&db=Protein&list_uids=223005950&dopt=GenPept&RID=T2WHBG7501N&log$=prottop&blast_rank=70) | SH3KBP1 binding protein 1 [Homo sapiens] | [18.0](http://blast.ncbi.nlm.nih.gov/Blast.cgi#223005950%23223005950) | 18.0 | 75% | 518 |
| [NP_001020128.1](http://www.ncbi.nlm.nih.gov/entrez/query.fcgi?cmd=Retrieve&db=Protein&list_uids=68348702&dopt=GenPept&RID=T2WHBG7501N&log$=prottop&blast_rank=71) | breast cancer metastasis suppressor 1 isoform 2 [Homo sapiens] | [18.0](http://blast.ncbi.nlm.nih.gov/Blast.cgi#68348702%2368348702) | 18.0 | 87% | 518 |
| [NP_002606.3](http://www.ncbi.nlm.nih.gov/entrez/query.fcgi?cmd=Retrieve&db=Protein&list_uids=39725934&dopt=GenPept&RID=T2WHBG7501N&log$=prottop&blast_rank=72) | serine (or cysteine) proteinase inhibitor, clade F (alpha-2 antiplasmin, pigment epithelium derived factor), member 1 precursor [Homo sapiens] | [18.0](http://blast.ncbi.nlm.nih.gov/Blast.cgi#39725934%2339725934) | 18.0 | 75% | 518 |
| [NP_000149.3](http://www.ncbi.nlm.nih.gov/entrez/query.fcgi?cmd=Retrieve&db=Protein&list_uids=189458812&dopt=GenPept&RID=T2WHBG7501N&log$=prottop&blast_rank=73) | glucan (1,4-alpha-), branching enzyme 1 [Homo sapiens] | [18.0](http://blast.ncbi.nlm.nih.gov/Blast.cgi#189458812%23189458812) | 18.0 | 75% | 518 |
| [NP_060816.1](http://www.ncbi.nlm.nih.gov/entrez/query.fcgi?cmd=Retrieve&db=Protein&list_uids=8922911&dopt=GenPept&RID=T2WHBG7501N&log$=prottop&blast_rank=74) | radical S-adenosyl methionine domain containing 1 precursor [Homo sapiens] | [18.0](http://blast.ncbi.nlm.nih.gov/Blast.cgi#8922911%238922911) | 18.0 | 100% | 518 |
| [NP_008877.1](http://www.ncbi.nlm.nih.gov/entrez/query.fcgi?cmd=Retrieve&db=Protein&list_uids=5902122&dopt=GenPept&RID=T2WHBG7501N&log$=prottop&blast_rank=75) | spectrin, beta, non-erythrocytic 2 [Homo sapiens] | [18.0](http://blast.ncbi.nlm.nih.gov/Blast.cgi#5902122%235902122) | 18.0 | 87% | 518 |
| [NP_056214.1](http://www.ncbi.nlm.nih.gov/entrez/query.fcgi?cmd=Retrieve&db=Protein&list_uids=17530785&dopt=GenPept&RID=T2WHBG7501N&log$=prottop&blast_rank=76) | breast cancer metastasis suppressor 1 isoform 1 [Homo sapiens] | [18.0](http://blast.ncbi.nlm.nih.gov/Blast.cgi#17530785%2317530785) | 18.0 | 87% | 518 |
| [NP_001166122.1](http://www.ncbi.nlm.nih.gov/entrez/query.fcgi?cmd=Retrieve&db=Protein&list_uids=289547611&dopt=GenPept&RID=T2WHBG7501N&log$=prottop&blast_rank=77) | zinc finger protein 177 isoform a [Homo sapiens] | [17.6](http://blast.ncbi.nlm.nih.gov/Blast.cgi#289547611%23289547611) | 17.6 | 87% | 695 |
| [XP_001714433.2](http://www.ncbi.nlm.nih.gov/entrez/query.fcgi?cmd=Retrieve&db=Protein&list_uids=239757877&dopt=GenPept&RID=T2WHBG7501N&log$=prottop&blast_rank=78) | PREDICTED: FERM and PDZ domain containing 3 [Homo sapiens] | [17.6](http://blast.ncbi.nlm.nih.gov/Blast.cgi#239757877%23239757877) | 30.5 | 100% | 695 |
| [NP_055809.2](http://www.ncbi.nlm.nih.gov/entrez/query.fcgi?cmd=Retrieve&db=Protein&list_uids=190684671&dopt=GenPept&RID=T2WHBG7501N&log$=prottop&blast_rank=79) | mitogen-activated protein kinase binding protein 1 isoform a [Homo sapiens] | [17.6](http://blast.ncbi.nlm.nih.gov/Blast.cgi#190684671%23190684671) | 33.9 | 100% | 695 |
| [NP_078858.4](http://www.ncbi.nlm.nih.gov/entrez/query.fcgi?cmd=Retrieve&db=Protein&list_uids=165932370&dopt=GenPept&RID=T2WHBG7501N&log$=prottop&blast_rank=80) | FAT tumor suppressor homolog 4 precursor [Homo sapiens] | [17.6](http://blast.ncbi.nlm.nih.gov/Blast.cgi#165932370%23165932370) | 60.7 | 100% | 695 |
| [NP_061930.2](http://www.ncbi.nlm.nih.gov/entrez/query.fcgi?cmd=Retrieve&db=Protein&list_uids=154426272&dopt=GenPept&RID=T2WHBG7501N&log$=prottop&blast_rank=81) | hypothetical protein LOC54540 [Homo sapiens] | [17.6](http://blast.ncbi.nlm.nih.gov/Blast.cgi#154426272%23154426272) | 17.6 | 87% | 695 |
| [NP_775907.4](http://www.ncbi.nlm.nih.gov/entrez/query.fcgi?cmd=Retrieve&db=Protein&list_uids=145580610&dopt=GenPept&RID=T2WHBG7501N&log$=prottop&blast_rank=82) | WD repeat domain 62 isoform 2 [Homo sapiens] | [17.6](http://blast.ncbi.nlm.nih.gov/Blast.cgi#145580610%23145580610) | 17.6 | 87% | 695 |
| [NP_001077430.1](http://www.ncbi.nlm.nih.gov/entrez/query.fcgi?cmd=Retrieve&db=Protein&list_uids=145580608&dopt=GenPept&RID=T2WHBG7501N&log$=prottop&blast_rank=83) | WD repeat domain 62 isoform 1 [Homo sapiens] | [17.6](http://blast.ncbi.nlm.nih.gov/Blast.cgi#145580608%23145580608) | 17.6 | 87% | 695 |
| [NP_006028.2](http://www.ncbi.nlm.nih.gov/entrez/query.fcgi?cmd=Retrieve&db=Protein&list_uids=153085395&dopt=GenPept&RID=T2WHBG7501N&log$=prottop&blast_rank=84) | histone deacetylase 4 [Homo sapiens] | [17.6](http://blast.ncbi.nlm.nih.gov/Blast.cgi#153085395%23153085395) | 17.6 | 75% | 695 |
| [NP_001122080.1](http://www.ncbi.nlm.nih.gov/entrez/query.fcgi?cmd=Retrieve&db=Protein&list_uids=190684677&dopt=GenPept&RID=T2WHBG7501N&log$=prottop&blast_rank=85) | mitogen-activated protein kinase binding protein 1 isoform b [Homo sapiens] | [17.6](http://blast.ncbi.nlm.nih.gov/Blast.cgi#190684677%23190684677) | 33.9 | 100% | 695 |
| [NP_001015053.1](http://www.ncbi.nlm.nih.gov/entrez/query.fcgi?cmd=Retrieve&db=Protein&list_uids=62750349&dopt=GenPept&RID=T2WHBG7501N&log$=prottop&blast_rank=86) | histone deacetylase 5 isoform 3 [Homo sapiens] | [17.6](http://blast.ncbi.nlm.nih.gov/Blast.cgi#62750349%2362750349) | 17.6 | 75% | 695 |
| [NP_005465.2](http://www.ncbi.nlm.nih.gov/entrez/query.fcgi?cmd=Retrieve&db=Protein&list_uids=62750347&dopt=GenPept&RID=T2WHBG7501N&log$=prottop&blast_rank=87) | histone deacetylase 5 isoform 1 [Homo sapiens] | [17.6](http://blast.ncbi.nlm.nih.gov/Blast.cgi#62750347%2362750347) | 17.6 | 75% | 695 |
| [NP_110394.3](http://www.ncbi.nlm.nih.gov/entrez/query.fcgi?cmd=Retrieve&db=Protein&list_uids=151301137&dopt=GenPept&RID=T2WHBG7501N&log$=prottop&blast_rank=88) | AT-hook transcription factor [Homo sapiens] | [17.6](http://blast.ncbi.nlm.nih.gov/Blast.cgi#151301137%23151301137) | 17.6 | 75% | 695 |
| [NP_056505.2](http://www.ncbi.nlm.nih.gov/entrez/query.fcgi?cmd=Retrieve&db=Protein&list_uids=217330634&dopt=GenPept&RID=T2WHBG7501N&log$=prottop&blast_rank=89) | serine/threonine kinase 36 [Homo sapiens] | [17.6](http://blast.ncbi.nlm.nih.gov/Blast.cgi#217330634%23217330634) | 17.6 | 87% | 695 |
| [NP_055522.1](http://www.ncbi.nlm.nih.gov/entrez/query.fcgi?cmd=Retrieve&db=Protein&list_uids=7662280&dopt=GenPept&RID=T2WHBG7501N&log$=prottop&blast_rank=90) | histone deacetylase 9 isoform 3 [Homo sapiens] | [17.6](http://blast.ncbi.nlm.nih.gov/Blast.cgi#7662280%237662280) | 17.6 | 75% | 695 |
| [XP_042978.5](http://www.ncbi.nlm.nih.gov/entrez/query.fcgi?cmd=Retrieve&db=Protein&list_uids=89059963&dopt=GenPept&RID=T2WHBG7501N&log$=prottop&blast_rank=91) | PREDICTED: FERM and PDZ domain containing 3 [Homo sapiens] >ref|XP_942100.1| PREDICTED: FERM and PDZ domain containing 3 [Homo sapiens] | [17.6](http://blast.ncbi.nlm.nih.gov/Blast.cgi#89059963%2389059963) | 30.5 | 100% | 695 |
| [NP_848512.1](http://www.ncbi.nlm.nih.gov/entrez/query.fcgi?cmd=Retrieve&db=Protein&list_uids=30795204&dopt=GenPept&RID=T2WHBG7501N&log$=prottop&blast_rank=92) | histone deacetylase 9 isoform 5 [Homo sapiens] | [17.6](http://blast.ncbi.nlm.nih.gov/Blast.cgi#30795204%2330795204) | 17.6 | 75% | 695 |
| [NP_478057.1](http://www.ncbi.nlm.nih.gov/entrez/query.fcgi?cmd=Retrieve&db=Protein&list_uids=17158041&dopt=GenPept&RID=T2WHBG7501N&log$=prottop&blast_rank=93) | histone deacetylase 9 isoform 2 [Homo sapiens] | [17.6](http://blast.ncbi.nlm.nih.gov/Blast.cgi#17158041%2317158041) | 17.6 | 75% | 695 |
| [NP_056161.2](http://www.ncbi.nlm.nih.gov/entrez/query.fcgi?cmd=Retrieve&db=Protein&list_uids=38202205&dopt=GenPept&RID=T2WHBG7501N&log$=prottop&blast_rank=94) | zinc finger, FYVE domain containing 26 [Homo sapiens] | [17.6](http://blast.ncbi.nlm.nih.gov/Blast.cgi#38202205%2338202205) | 17.6 | 87% | 695 |
| [NP_001032402.1](http://www.ncbi.nlm.nih.gov/entrez/query.fcgi?cmd=Retrieve&db=Protein&list_uids=82880654&dopt=GenPept&RID=T2WHBG7501N&log$=prottop&blast_rank=95) | coiled-coil domain containing 46 isoform b [Homo sapiens] | [17.6](http://blast.ncbi.nlm.nih.gov/Blast.cgi#82880654%2382880654) | 17.6 | 75% | 695 |
| [NP_848510.1](http://www.ncbi.nlm.nih.gov/entrez/query.fcgi?cmd=Retrieve&db=Protein&list_uids=30795202&dopt=GenPept&RID=T2WHBG7501N&log$=prottop&blast_rank=96) | histone deacetylase 9 isoform 4 [Homo sapiens] | [17.6](http://blast.ncbi.nlm.nih.gov/Blast.cgi#30795202%2330795202) | 17.6 | 75% | 695 |
| [NP_203129.1](http://www.ncbi.nlm.nih.gov/entrez/query.fcgi?cmd=Retrieve&db=Protein&list_uids=15375314&dopt=GenPept&RID=T2WHBG7501N&log$=prottop&blast_rank=97) | LIM homeobox protein 4 [Homo sapiens] | [17.6](http://blast.ncbi.nlm.nih.gov/Blast.cgi#15375314%2315375314) | 17.6 | 75% | 695 |
| [NP_478056.1](http://www.ncbi.nlm.nih.gov/entrez/query.fcgi?cmd=Retrieve&db=Protein&list_uids=17158039&dopt=GenPept&RID=T2WHBG7501N&log$=prottop&blast_rank=98) | histone deacetylase 9 isoform 1 [Homo sapiens] | [17.6](http://blast.ncbi.nlm.nih.gov/Blast.cgi#17158039%2317158039) | 17.6 | 75% | 695 |
| [NP_001640.1](http://www.ncbi.nlm.nih.gov/entrez/query.fcgi?cmd=Retrieve&db=Protein&list_uids=4502175&dopt=GenPept&RID=T2WHBG7501N&log$=prottop&blast_rank=99) | apical protein of Xenopus-like [Homo sapiens] | [17.6](http://blast.ncbi.nlm.nih.gov/Blast.cgi#4502175%234502175) | 44.8 | 87% | 695 |
| [NP_003814.1](http://www.ncbi.nlm.nih.gov/entrez/query.fcgi?cmd=Retrieve&db=Protein&list_uids=4507585&dopt=GenPept&RID=T2WHBG7501N&log$=prottop&blast_rank=100) | tumor necrosis factor receptor superfamily, member 6b precursor [Homo sapiens] >ref|NP_116563.1| tumor necrosis factor receptor superfamily, member 6b precursor [Homo sapiens] | [17.6](http://blast.ncbi.nlm.nih.gov/Blast.cgi#4507585%234507585) | 17.6 | 75% | 695 |

| **Accession** | **Proteins with a match to LNPQSPRD peptide** | **[Max score](http://blast.ncbi.nlm.nih.gov/Blast.cgi?CMD=Get&ALIGNMENTS=100&ALIGNMENT_VIEW=Pairwise&CDD_SEARCH_STATE=1&DATABASE_SORT=0&DESCRIPTIONS=100&ENTREZ_QUERY=txid9606 %5BORGN%5D&FIRST_QUERY_NUM=0&FORMAT_OBJECT=Alignment&FORMAT_PAGE_TARGET=&FORMAT_TYPE=HTML&GET_SEQUENCE=yes&I_THRESH=&MASK_CHAR=2&MASK_COLOR=1&NEW_DESIGN=on&NEW_VIEW=yes&NUM_OVERVIEW=100&OLD_BLAST=false&PAGE=Proteins&QUERY_INDEX=0&QUERY_NUMBER=0&RESULTS_PAGE_TARGET=&RID=T2WPRKGM016&SHOW_LINKOUT=yes&SHOW_OVERVIEW=yes&STEP_NUMBER=&WORD_SIZE=2&DISPLAY_SORT=1&HSP_SORT=1" \l "sort_mark)** | **[Total score](http://blast.ncbi.nlm.nih.gov/Blast.cgi?CMD=Get&ALIGNMENTS=100&ALIGNMENT_VIEW=Pairwise&CDD_SEARCH_STATE=1&DATABASE_SORT=0&DESCRIPTIONS=100&ENTREZ_QUERY=txid9606 %5BORGN%5D&FIRST_QUERY_NUM=0&FORMAT_OBJECT=Alignment&FORMAT_PAGE_TARGET=&FORMAT_TYPE=HTML&GET_SEQUENCE=yes&I_THRESH=&MASK_CHAR=2&MASK_COLOR=1&NEW_DESIGN=on&NEW_VIEW=yes&NUM_OVERVIEW=100&OLD_BLAST=false&PAGE=Proteins&QUERY_INDEX=0&QUERY_NUMBER=0&RESULTS_PAGE_TARGET=&RID=T2WPRKGM016&SHOW_LINKOUT=yes&SHOW_OVERVIEW=yes&STEP_NUMBER=&WORD_SIZE=2&DISPLAY_SORT=2&HSP_SORT=1" \l "sort_mark)** | **[Query coverage](http://blast.ncbi.nlm.nih.gov/Blast.cgi?CMD=Get&ALIGNMENTS=100&ALIGNMENT_VIEW=Pairwise&CDD_SEARCH_STATE=1&DATABASE_SORT=0&DESCRIPTIONS=100&ENTREZ_QUERY=txid9606 %5BORGN%5D&FIRST_QUERY_NUM=0&FORMAT_OBJECT=Alignment&FORMAT_PAGE_TARGET=&FORMAT_TYPE=HTML&GET_SEQUENCE=yes&I_THRESH=&MASK_CHAR=2&MASK_COLOR=1&NEW_DESIGN=on&NEW_VIEW=yes&NUM_OVERVIEW=100&OLD_BLAST=false&PAGE=Proteins&QUERY_INDEX=0&QUERY_NUMBER=0&RESULTS_PAGE_TARGET=&RID=T2WPRKGM016&SHOW_LINKOUT=yes&SHOW_OVERVIEW=yes&STEP_NUMBER=&WORD_SIZE=2&DISPLAY_SORT=4&HSP_SORT=0" \l "sort_mark)** | **[E value](http://blast.ncbi.nlm.nih.gov/Blast.cgi?CMD=Get&ALIGNMENTS=100&ALIGNMENT_VIEW=Pairwise&CDD_SEARCH_STATE=1&DATABASE_SORT=0&DESCRIPTIONS=100&ENTREZ_QUERY=txid9606 %5BORGN%5D&FIRST_QUERY_NUM=0&FORMAT_OBJECT=Alignment&FORMAT_PAGE_TARGET=&FORMAT_TYPE=HTML&GET_SEQUENCE=yes&I_THRESH=&MASK_CHAR=2&MASK_COLOR=1&NEW_DESIGN=on&NEW_VIEW=yes&NUM_OVERVIEW=100&OLD_BLAST=false&PAGE=Proteins&QUERY_INDEX=0&QUERY_NUMBER=0&RESULTS_PAGE_TARGET=&RID=T2WPRKGM016&SHOW_LINKOUT=yes&SHOW_OVERVIEW=yes&STEP_NUMBER=&WORD_SIZE=2&DISPLAY_SORT=0&HSP_SORT=0" \l "sort_mark)** |
| --- | --- | --- | --- | --- | --- |
| [NP_001120712.1](http://www.ncbi.nlm.nih.gov/entrez/query.fcgi?cmd=Retrieve&db=Protein&list_uids=187829730&dopt=GenPept&RID=T2WPRKGM016&log$=prottop&blast_rank=1) | BCL2 binding component 3 isoform 1 [Homo sapiens] | [22.7](http://blast.ncbi.nlm.nih.gov/Blast.cgi#187829730%23187829730) | 22.7 | 75% | 20 |
| [NP_004002.2](http://www.ncbi.nlm.nih.gov/entrez/query.fcgi?cmd=Retrieve&db=Protein&list_uids=150036268&dopt=GenPept&RID=T2WPRKGM016&log$=prottop&blast_rank=2) | dystrophin Dp260-1 isoform [Homo sapiens] | [22.7](http://blast.ncbi.nlm.nih.gov/Blast.cgi#150036268%23150036268) | 36.1 | 100% | 20 |
| [NP_004013.1](http://www.ncbi.nlm.nih.gov/entrez/query.fcgi?cmd=Retrieve&db=Protein&list_uids=5032311&dopt=GenPept&RID=T2WPRKGM016&log$=prottop&blast_rank=3) | dystrophin Dp140ab isoform [Homo sapiens] | [22.7](http://blast.ncbi.nlm.nih.gov/Blast.cgi#5032311%235032311) | 36.1 | 100% | 20 |
| [NP_000100.2](http://www.ncbi.nlm.nih.gov/entrez/query.fcgi?cmd=Retrieve&db=Protein&list_uids=5032281&dopt=GenPept&RID=T2WPRKGM016&log$=prottop&blast_rank=4) | dystrophin Dp427c isoform [Homo sapiens] | [22.7](http://blast.ncbi.nlm.nih.gov/Blast.cgi#5032281%235032281) | 36.1 | 100% | 20 |
| [NP_003997.1](http://www.ncbi.nlm.nih.gov/entrez/query.fcgi?cmd=Retrieve&db=Protein&list_uids=5032283&dopt=GenPept&RID=T2WPRKGM016&log$=prottop&blast_rank=5) | dystrophin Dp427m isoform [Homo sapiens] | [22.7](http://blast.ncbi.nlm.nih.gov/Blast.cgi#5032283%235032283) | 36.1 | 100% | 20 |
| [NP_004003.1](http://www.ncbi.nlm.nih.gov/entrez/query.fcgi?cmd=Retrieve&db=Protein&list_uids=5032291&dopt=GenPept&RID=T2WPRKGM016&log$=prottop&blast_rank=6) | dystrophin Dp260-2 isoform [Homo sapiens] | [22.7](http://blast.ncbi.nlm.nih.gov/Blast.cgi#5032291%235032291) | 36.1 | 100% | 20 |
| [NP_004011.1](http://www.ncbi.nlm.nih.gov/entrez/query.fcgi?cmd=Retrieve&db=Protein&list_uids=5032307&dopt=GenPept&RID=T2WPRKGM016&log$=prottop&blast_rank=7) | dystrophin Dp140c isoform [Homo sapiens] | [22.7](http://blast.ncbi.nlm.nih.gov/Blast.cgi#5032307%235032307) | 22.7 | 75% | 20 |
| [NP_004006.1](http://www.ncbi.nlm.nih.gov/entrez/query.fcgi?cmd=Retrieve&db=Protein&list_uids=5032297&dopt=GenPept&RID=T2WPRKGM016&log$=prottop&blast_rank=8) | dystrophin Dp71 isoform [Homo sapiens] | [22.7](http://blast.ncbi.nlm.nih.gov/Blast.cgi#5032297%235032297) | 36.1 | 100% | 20 |
| [NP_004005.1](http://www.ncbi.nlm.nih.gov/entrez/query.fcgi?cmd=Retrieve&db=Protein&list_uids=5032295&dopt=GenPept&RID=T2WPRKGM016&log$=prottop&blast_rank=9) | dystrophin Dp116 isoform [Homo sapiens] | [22.7](http://blast.ncbi.nlm.nih.gov/Blast.cgi#5032295%235032295) | 36.1 | 100% | 20 |
| [NP_003998.1](http://www.ncbi.nlm.nih.gov/entrez/query.fcgi?cmd=Retrieve&db=Protein&list_uids=5032285&dopt=GenPept&RID=T2WPRKGM016&log$=prottop&blast_rank=10) | dystrophin Dp427l isoform [Homo sapiens] >ref|NP_004001.1| dystrophin Dp427p2 isoform [Homo sapiens] | [22.7](http://blast.ncbi.nlm.nih.gov/Blast.cgi#5032285%235032285) | 36.1 | 100% | 20 |
| [NP_004014.1](http://www.ncbi.nlm.nih.gov/entrez/query.fcgi?cmd=Retrieve&db=Protein&list_uids=5032313&dopt=GenPept&RID=T2WPRKGM016&log$=prottop&blast_rank=11) | dystrophin Dp140bc isoform [Homo sapiens] | [22.7](http://blast.ncbi.nlm.nih.gov/Blast.cgi#5032313%235032313) | 22.7 | 75% | 20 |
| [NP_004000.1](http://www.ncbi.nlm.nih.gov/entrez/query.fcgi?cmd=Retrieve&db=Protein&list_uids=5032287&dopt=GenPept&RID=T2WPRKGM016&log$=prottop&blast_rank=12) | dystrophin Dp427p1 isoform [Homo sapiens] | [22.7](http://blast.ncbi.nlm.nih.gov/Blast.cgi#5032287%235032287) | 36.1 | 100% | 20 |
| [NP_004008.1](http://www.ncbi.nlm.nih.gov/entrez/query.fcgi?cmd=Retrieve&db=Protein&list_uids=5032301&dopt=GenPept&RID=T2WPRKGM016&log$=prottop&blast_rank=13) | dystrophin Dp71a isoform [Homo sapiens] | [22.7](http://blast.ncbi.nlm.nih.gov/Blast.cgi#5032301%235032301) | 36.1 | 100% | 20 |
| [NP_001092285.1](http://www.ncbi.nlm.nih.gov/entrez/query.fcgi?cmd=Retrieve&db=Protein&list_uids=149363704&dopt=GenPept&RID=T2WPRKGM016&log$=prottop&blast_rank=14) | hypothetical protein LOC9840 [Homo sapiens] >ref|NP_001129502.1| hypothetical protein LOC9840 [Homo sapiens] | [22.7](http://blast.ncbi.nlm.nih.gov/Blast.cgi#149363704%23149363704) | 22.7 | 75% | 20 |
| [NP_004012.1](http://www.ncbi.nlm.nih.gov/entrez/query.fcgi?cmd=Retrieve&db=Protein&list_uids=5032309&dopt=GenPept&RID=T2WPRKGM016&log$=prottop&blast_rank=15) | dystrophin Dp140b isoform [Homo sapiens] | [22.7](http://blast.ncbi.nlm.nih.gov/Blast.cgi#5032309%235032309) | 36.1 | 100% | 20 |
| [NP_004004.1](http://www.ncbi.nlm.nih.gov/entrez/query.fcgi?cmd=Retrieve&db=Protein&list_uids=5032293&dopt=GenPept&RID=T2WPRKGM016&log$=prottop&blast_rank=16) | dystrophin Dp140 isoform [Homo sapiens] | [22.7](http://blast.ncbi.nlm.nih.gov/Blast.cgi#5032293%235032293) | 36.1 | 100% | 20 |
| [NP_001120714.1](http://www.ncbi.nlm.nih.gov/entrez/query.fcgi?cmd=Retrieve&db=Protein&list_uids=187829745&dopt=GenPept&RID=T2WPRKGM016&log$=prottop&blast_rank=17) | BCL2 binding component 3 isoform 3 [Homo sapiens] | [22.7](http://blast.ncbi.nlm.nih.gov/Blast.cgi#187829745%23187829745) | 22.7 | 75% | 20 |
| [NP_004007.1](http://www.ncbi.nlm.nih.gov/entrez/query.fcgi?cmd=Retrieve&db=Protein&list_uids=5032299&dopt=GenPept&RID=T2WPRKGM016&log$=prottop&blast_rank=18) | dystrophin Dp71b isoform [Homo sapiens] | [22.7](http://blast.ncbi.nlm.nih.gov/Blast.cgi#5032299%235032299) | 36.1 | 100% | 20 |
| [NP_004009.1](http://www.ncbi.nlm.nih.gov/entrez/query.fcgi?cmd=Retrieve&db=Protein&list_uids=5032303&dopt=GenPept&RID=T2WPRKGM016&log$=prottop&blast_rank=19) | dystrophin Dp71ab isoform [Homo sapiens] | [22.7](http://blast.ncbi.nlm.nih.gov/Blast.cgi#5032303%235032303) | 36.1 | 100% | 20 |
| [NP_078966.2](http://www.ncbi.nlm.nih.gov/entrez/query.fcgi?cmd=Retrieve&db=Protein&list_uids=83367077&dopt=GenPept&RID=T2WPRKGM016&log$=prottop&blast_rank=20) | mucin 16 [Homo sapiens] | [22.3](http://blast.ncbi.nlm.nih.gov/Blast.cgi#83367077%2383367077) | 22.3 | 75% | 27 |
| [NP_055771.4](http://www.ncbi.nlm.nih.gov/entrez/query.fcgi?cmd=Retrieve&db=Protein&list_uids=115648142&dopt=GenPept&RID=T2WPRKGM016&log$=prottop&blast_rank=21) | centrosomal protein 164kDa [Homo sapiens] | [22.3](http://blast.ncbi.nlm.nih.gov/Blast.cgi#115648142%23115648142) | 22.3 | 75% | 27 |
| [NP_078881.3](http://www.ncbi.nlm.nih.gov/entrez/query.fcgi?cmd=Retrieve&db=Protein&list_uids=50843948&dopt=GenPept&RID=T2WPRKGM016&log$=prottop&blast_rank=22) | Rho GTPase activating protein 10 [Homo sapiens] | [22.3](http://blast.ncbi.nlm.nih.gov/Blast.cgi#50843948%2350843948) | 22.3 | 75% | 27 |
| [NP_000765.2](http://www.ncbi.nlm.nih.gov/entrez/query.fcgi?cmd=Retrieve&db=Protein&list_uids=19743565&dopt=GenPept&RID=T2WPRKGM016&log$=prottop&blast_rank=23) | cytochrome P450, family 2, subfamily F, polypeptide 1 [Homo sapiens] | [22.3](http://blast.ncbi.nlm.nih.gov/Blast.cgi#19743565%2319743565) | 22.3 | 100% | 27 |
| [NP_000753.3](http://www.ncbi.nlm.nih.gov/entrez/query.fcgi?cmd=Retrieve&db=Protein&list_uids=189339233&dopt=GenPept&RID=T2WPRKGM016&log$=prottop&blast_rank=24) | cytochrome P450, family 2, subfamily A, polypeptide 6 [Homo sapiens] | [21.8](http://blast.ncbi.nlm.nih.gov/Blast.cgi#189339233%23189339233) | 21.8 | 100% | 37 |
| [NP_062565.2](http://www.ncbi.nlm.nih.gov/entrez/query.fcgi?cmd=Retrieve&db=Protein&list_uids=21361831&dopt=GenPept&RID=T2WPRKGM016&log$=prottop&blast_rank=25) | partitioning-defective protein 3 homolog [Homo sapiens] | [21.8](http://blast.ncbi.nlm.nih.gov/Blast.cgi#21361831%2321361831) | 21.8 | 100% | 37 |
| [NP_000757.2](http://www.ncbi.nlm.nih.gov/entrez/query.fcgi?cmd=Retrieve&db=Protein&list_uids=13699809&dopt=GenPept&RID=T2WPRKGM016&log$=prottop&blast_rank=26) | cytochrome P450, family 2, subfamily A, polypeptide 13 [Homo sapiens] | [21.8](http://blast.ncbi.nlm.nih.gov/Blast.cgi#13699809%2313699809) | 41.1 | 100% | 37 |
| [NP_060718.2](http://www.ncbi.nlm.nih.gov/entrez/query.fcgi?cmd=Retrieve&db=Protein&list_uids=157388969&dopt=GenPept&RID=T2WPRKGM016&log$=prottop&blast_rank=27) | nei endonuclease VIII-like 3 [Homo sapiens] | [21.4](http://blast.ncbi.nlm.nih.gov/Blast.cgi#157388969%23157388969) | 21.4 | 87% | 49 |
| [NP_872327.2](http://www.ncbi.nlm.nih.gov/entrez/query.fcgi?cmd=Retrieve&db=Protein&list_uids=71043932&dopt=GenPept&RID=T2WPRKGM016&log$=prottop&blast_rank=28) | zinc finger, SWIM domain containing 2 [Homo sapiens] | [21.4](http://blast.ncbi.nlm.nih.gov/Blast.cgi#71043932%2371043932) | 21.4 | 100% | 49 |
| [NP_060638.2](http://www.ncbi.nlm.nih.gov/entrez/query.fcgi?cmd=Retrieve&db=Protein&list_uids=190341089&dopt=GenPept&RID=T2WPRKGM016&log$=prottop&blast_rank=29) | hypothetical protein LOC55195 [Homo sapiens] | [20.2](http://blast.ncbi.nlm.nih.gov/Blast.cgi#190341089%23190341089) | 20.2 | 87% | 119 |
| [XP_001129558.1](http://www.ncbi.nlm.nih.gov/entrez/query.fcgi?cmd=Retrieve&db=Protein&list_uids=113415539&dopt=GenPept&RID=T2WPRKGM016&log$=prottop&blast_rank=30) | PREDICTED: hypothetical protein [Homo sapiens] >ref|XP_001131204.1| PREDICTED: hypothetical protein [Homo sapiens] >ref|XP_001717566.1| PREDICTED: hypothetical protein [Homo sapiens] | [20.2](http://blast.ncbi.nlm.nih.gov/Blast.cgi#113415539%23113415539) | 20.2 | 75% | 119 |
| [NP_056099.2](http://www.ncbi.nlm.nih.gov/entrez/query.fcgi?cmd=Retrieve&db=Protein&list_uids=71999153&dopt=GenPept&RID=T2WPRKGM016&log$=prottop&blast_rank=31) | hypothetical protein LOC23334 [Homo sapiens] | [20.2](http://blast.ncbi.nlm.nih.gov/Blast.cgi#71999153%2371999153) | 36.1 | 75% | 119 |
| [NP_002918.1](http://www.ncbi.nlm.nih.gov/entrez/query.fcgi?cmd=Retrieve&db=Protein&list_uids=4506511&dopt=GenPept&RID=T2WPRKGM016&log$=prottop&blast_rank=32) | regulator of G-protein signalling 13 [Homo sapiens] >ref|NP_658912.1| regulator of G-protein signalling 13 [Homo sapiens] | [20.2](http://blast.ncbi.nlm.nih.gov/Blast.cgi#4506511%234506511) | 20.2 | 75% | 119 |
| [NP_057676.1](http://www.ncbi.nlm.nih.gov/entrez/query.fcgi?cmd=Retrieve&db=Protein&list_uids=7706589&dopt=GenPept&RID=T2WPRKGM016&log$=prottop&blast_rank=33) | GNAS complex locus NESP55 [Homo sapiens] | [20.2](http://blast.ncbi.nlm.nih.gov/Blast.cgi#7706589%237706589) | 20.2 | 75% | 119 |
| [NP_001138422.1](http://www.ncbi.nlm.nih.gov/entrez/query.fcgi?cmd=Retrieve&db=Protein&list_uids=222352122&dopt=GenPept&RID=T2WPRKGM016&log$=prottop&blast_rank=34) | scavenger receptor cysteine-rich glycoprotein [Homo sapiens] | [19.7](http://blast.ncbi.nlm.nih.gov/Blast.cgi#222352122%23222352122) | 19.7 | 100% | 160 |
| [NP_001157782.1](http://www.ncbi.nlm.nih.gov/entrez/query.fcgi?cmd=Retrieve&db=Protein&list_uids=256221824&dopt=GenPept&RID=T2WPRKGM016&log$=prottop&blast_rank=35) | hypothetical protein LOC730112 isoform 1 [Homo sapiens] | [19.7](http://blast.ncbi.nlm.nih.gov/Blast.cgi#256221824%23256221824) | 19.7 | 75% | 160 |
| [NP_001093421.1](http://www.ncbi.nlm.nih.gov/entrez/query.fcgi?cmd=Retrieve&db=Protein&list_uids=153791167&dopt=GenPept&RID=T2WPRKGM016&log$=prottop&blast_rank=36) | hypothetical protein LOC730112 isoform 2 [Homo sapiens] | [19.7](http://blast.ncbi.nlm.nih.gov/Blast.cgi#153791167%23153791167) | 19.7 | 75% | 160 |
| [NP_001155855.1](http://www.ncbi.nlm.nih.gov/entrez/query.fcgi?cmd=Retrieve&db=Protein&list_uids=253735775&dopt=GenPept&RID=T2WPRKGM016&log$=prottop&blast_rank=37) | Rho/Rac guanine nucleotide exchange factor 2 isoform 1 [Homo sapiens] | [19.7](http://blast.ncbi.nlm.nih.gov/Blast.cgi#253735775%23253735775) | 19.7 | 75% | 160 |
| [NP_054790.2](http://www.ncbi.nlm.nih.gov/entrez/query.fcgi?cmd=Retrieve&db=Protein&list_uids=32307128&dopt=GenPept&RID=T2WPRKGM016&log$=prottop&blast_rank=38) | nuclear receptor coactivator 6 [Homo sapiens] | [19.7](http://blast.ncbi.nlm.nih.gov/Blast.cgi#32307128%2332307128) | 36.1 | 75% | 160 |
| [NP_001155856.1](http://www.ncbi.nlm.nih.gov/entrez/query.fcgi?cmd=Retrieve&db=Protein&list_uids=253735778&dopt=GenPept&RID=T2WPRKGM016&log$=prottop&blast_rank=39) | Rho/Rac guanine nucleotide exchange factor 2 isoform 2 [Homo sapiens] | [19.7](http://blast.ncbi.nlm.nih.gov/Blast.cgi#253735778%23253735778) | 19.7 | 75% | 160 |
| [NP_005144.2](http://www.ncbi.nlm.nih.gov/entrez/query.fcgi?cmd=Retrieve&db=Protein&list_uids=119220605&dopt=GenPept&RID=T2WPRKGM016&log$=prottop&blast_rank=40) | ubiquitin specific protease 10 [Homo sapiens] | [19.7](http://blast.ncbi.nlm.nih.gov/Blast.cgi#119220605%23119220605) | 19.7 | 75% | 160 |
| [NP_001025181.1](http://www.ncbi.nlm.nih.gov/entrez/query.fcgi?cmd=Retrieve&db=Protein&list_uids=71773324&dopt=GenPept&RID=T2WPRKGM016&log$=prottop&blast_rank=41) | aldehyde dehydrogenase 3B1 isoform b [Homo sapiens] | [19.7](http://blast.ncbi.nlm.nih.gov/Blast.cgi#71773324%2371773324) | 19.7 | 87% | 160 |
| [NP_004714.2](http://www.ncbi.nlm.nih.gov/entrez/query.fcgi?cmd=Retrieve&db=Protein&list_uids=15011974&dopt=GenPept&RID=T2WPRKGM016&log$=prottop&blast_rank=42) | Rho/Rac guanine nucleotide exchange factor 2 isooform 3 [Homo sapiens] | [19.7](http://blast.ncbi.nlm.nih.gov/Blast.cgi#15011974%2315011974) | 19.7 | 75% | 160 |
| [NP_000318.1](http://www.ncbi.nlm.nih.gov/entrez/query.fcgi?cmd=Retrieve&db=Protein&list_uids=4506575&dopt=GenPept&RID=T2WPRKGM016&log$=prottop&blast_rank=43) | retinal outer segment membrane protein 1 [Homo sapiens] | [19.7](http://blast.ncbi.nlm.nih.gov/Blast.cgi#4506575%234506575) | 19.7 | 75% | 160 |
| [NP_000685.1](http://www.ncbi.nlm.nih.gov/entrez/query.fcgi?cmd=Retrieve&db=Protein&list_uids=4502043&dopt=GenPept&RID=T2WPRKGM016&log$=prottop&blast_rank=44) | aldehyde dehydrogenase 3B1 isoform a [Homo sapiens] >ref|NP_001154945.1| aldehyde dehydrogenase 3B1 isoform a [Homo sapiens] | [19.7](http://blast.ncbi.nlm.nih.gov/Blast.cgi#4502043%234502043) | 19.7 | 87% | 160 |
| [NP_001003700.1](http://www.ncbi.nlm.nih.gov/entrez/query.fcgi?cmd=Retrieve&db=Protein&list_uids=270132935&dopt=GenPept&RID=T2WPRKGM016&log$=prottop&blast_rank=45) | ras responsive element binding protein 1 isoform 3 [Homo sapiens] | [19.3](http://blast.ncbi.nlm.nih.gov/Blast.cgi#270132935%23270132935) | 19.3 | 75% | 214 |
| [NP_001158222.1](http://www.ncbi.nlm.nih.gov/entrez/query.fcgi?cmd=Retrieve&db=Protein&list_uids=258613943&dopt=GenPept&RID=T2WPRKGM016&log$=prottop&blast_rank=46) | aspartate beta-hydroxylase isoform f [Homo sapiens] | [19.3](http://blast.ncbi.nlm.nih.gov/Blast.cgi#258613943%23258613943) | 19.3 | 62% | 214 |
| [NP_001156730.1](http://www.ncbi.nlm.nih.gov/entrez/query.fcgi?cmd=Retrieve&db=Protein&list_uids=253795488&dopt=GenPept&RID=T2WPRKGM016&log$=prottop&blast_rank=47) | hypothetical protein LOC55793 isoform 3 [Homo sapiens] | [19.3](http://blast.ncbi.nlm.nih.gov/Blast.cgi#253795488%23253795488) | 36.1 | 75% | 214 |
| [XP_002342926.1](http://www.ncbi.nlm.nih.gov/entrez/query.fcgi?cmd=Retrieve&db=Protein&list_uids=239743660&dopt=GenPept&RID=T2WPRKGM016&log$=prottop&blast_rank=48) | PREDICTED: hypothetical protein XP_002342926 [Homo sapiens] | [19.3](http://blast.ncbi.nlm.nih.gov/Blast.cgi#239743660%23239743660) | 19.3 | 62% | 214 |
| [XP_002344369.1](http://www.ncbi.nlm.nih.gov/entrez/query.fcgi?cmd=Retrieve&db=Protein&list_uids=239508917&dopt=GenPept&RID=T2WPRKGM016&log$=prottop&blast_rank=49) | PREDICTED: hypothetical protein [Homo sapiens] >ref|XP_002342777.1| PREDICTED: hypothetical protein XP_002342777 [Homo sapiens] | [19.3](http://blast.ncbi.nlm.nih.gov/Blast.cgi#239508917%23239508917) | 19.3 | 62% | 214 |
| [NP_001138237.1](http://www.ncbi.nlm.nih.gov/entrez/query.fcgi?cmd=Retrieve&db=Protein&list_uids=221316754&dopt=GenPept&RID=T2WPRKGM016&log$=prottop&blast_rank=50) | exophilin 5 isoform c [Homo sapiens] | [19.3](http://blast.ncbi.nlm.nih.gov/Blast.cgi#221316754%23221316754) | 19.3 | 62% | 214 |
| [NP_055880.2](http://www.ncbi.nlm.nih.gov/entrez/query.fcgi?cmd=Retrieve&db=Protein&list_uids=221316747&dopt=GenPept&RID=T2WPRKGM016&log$=prottop&blast_rank=51) | exophilin 5 isoform a [Homo sapiens] | [19.3](http://blast.ncbi.nlm.nih.gov/Blast.cgi#221316747%23221316747) | 19.3 | 62% | 214 |
| [NP_001156731.1](http://www.ncbi.nlm.nih.gov/entrez/query.fcgi?cmd=Retrieve&db=Protein&list_uids=253795490&dopt=GenPept&RID=T2WPRKGM016&log$=prottop&blast_rank=52) | hypothetical protein LOC55793 isoform 4 [Homo sapiens] | [19.3](http://blast.ncbi.nlm.nih.gov/Blast.cgi#253795490%23253795490) | 36.1 | 75% | 214 |
| [XP_001715639.1](http://www.ncbi.nlm.nih.gov/entrez/query.fcgi?cmd=Retrieve&db=Protein&list_uids=169160873&dopt=GenPept&RID=T2WPRKGM016&log$=prottop&blast_rank=53) | PREDICTED: similar to hCG2040319 [Homo sapiens] >ref|XP_002342138.1| PREDICTED: similar to hCG2040319 [Homo sapiens] >ref|XP_002346643.1| PREDICTED: similar to hCG2040319 [Homo sapiens] | [19.3](http://blast.ncbi.nlm.nih.gov/Blast.cgi#169160873%23169160873) | 19.3 | 62% | 214 |
| [NP_055842.2](http://www.ncbi.nlm.nih.gov/entrez/query.fcgi?cmd=Retrieve&db=Protein&list_uids=190341074&dopt=GenPept&RID=T2WPRKGM016&log$=prottop&blast_rank=54) | pyridoxal-dependent decarboxylase domain containing 1 [Homo sapiens] | [19.3](http://blast.ncbi.nlm.nih.gov/Blast.cgi#190341074%23190341074) | 19.3 | 62% | 214 |
| [NP_001093629.1](http://www.ncbi.nlm.nih.gov/entrez/query.fcgi?cmd=Retrieve&db=Protein&list_uids=154091007&dopt=GenPept&RID=T2WPRKGM016&log$=prottop&blast_rank=55) | hypothetical protein LOC136288 [Homo sapiens] | [19.3](http://blast.ncbi.nlm.nih.gov/Blast.cgi#154091007%23154091007) | 19.3 | 62% | 214 |
| [NP_056131.2](http://www.ncbi.nlm.nih.gov/entrez/query.fcgi?cmd=Retrieve&db=Protein&list_uids=121114287&dopt=GenPept&RID=T2WPRKGM016&log$=prottop&blast_rank=56) | apoptosis-stimulating protein of p53, 1 [Homo sapiens] | [19.3](http://blast.ncbi.nlm.nih.gov/Blast.cgi#121114287%23121114287) | 35.2 | 62% | 214 |
| [NP_001138235.1](http://www.ncbi.nlm.nih.gov/entrez/query.fcgi?cmd=Retrieve&db=Protein&list_uids=221316751&dopt=GenPept&RID=T2WPRKGM016&log$=prottop&blast_rank=57) | exophilin 5 isoform b [Homo sapiens] | [19.3](http://blast.ncbi.nlm.nih.gov/Blast.cgi#221316751%23221316751) | 19.3 | 62% | 214 |
| [NP_076419.2](http://www.ncbi.nlm.nih.gov/entrez/query.fcgi?cmd=Retrieve&db=Protein&list_uids=142356950&dopt=GenPept&RID=T2WPRKGM016&log$=prottop&blast_rank=58) | potassium channel tetramerisation domain containing 14 [Homo sapiens] | [19.3](http://blast.ncbi.nlm.nih.gov/Blast.cgi#142356950%23142356950) | 19.3 | 62% | 214 |
| [NP_006035.2](http://www.ncbi.nlm.nih.gov/entrez/query.fcgi?cmd=Retrieve&db=Protein&list_uids=13128864&dopt=GenPept&RID=T2WPRKGM016&log$=prottop&blast_rank=59) | histone deacetylase 6 [Homo sapiens] | [19.3](http://blast.ncbi.nlm.nih.gov/Blast.cgi#13128864%2313128864) | 19.3 | 62% | 214 |
| [NP_001138441.1](http://www.ncbi.nlm.nih.gov/entrez/query.fcgi?cmd=Retrieve&db=Protein&list_uids=222352090&dopt=GenPept&RID=T2WPRKGM016&log$=prottop&blast_rank=60) | neural precursor cell expressed, developmentally down-regulated 4-like isoform 5 [Homo sapiens] | [19.3](http://blast.ncbi.nlm.nih.gov/Blast.cgi#222352090%23222352090) | 19.3 | 62% | 214 |
| [NP_001138440.1](http://www.ncbi.nlm.nih.gov/entrez/query.fcgi?cmd=Retrieve&db=Protein&list_uids=222352088&dopt=GenPept&RID=T2WPRKGM016&log$=prottop&blast_rank=61) | neural precursor cell expressed, developmentally down-regulated 4-like isoform 4 [Homo sapiens] | [19.3](http://blast.ncbi.nlm.nih.gov/Blast.cgi#222352088%23222352088) | 19.3 | 62% | 214 |
| [NP_001013644.1](http://www.ncbi.nlm.nih.gov/entrez/query.fcgi?cmd=Retrieve&db=Protein&list_uids=61966701&dopt=GenPept&RID=T2WPRKGM016&log$=prottop&blast_rank=62) | dorsal neural-tube nuclear protein [Homo sapiens] | [19.3](http://blast.ncbi.nlm.nih.gov/Blast.cgi#61966701%2361966701) | 34.8 | 87% | 214 |
| [NP_004309.2](http://www.ncbi.nlm.nih.gov/entrez/query.fcgi?cmd=Retrieve&db=Protein&list_uids=14589866&dopt=GenPept&RID=T2WPRKGM016&log$=prottop&blast_rank=63) | aspartate beta-hydroxylase isoform a [Homo sapiens] | [19.3](http://blast.ncbi.nlm.nih.gov/Blast.cgi#14589866%2314589866) | 19.3 | 62% | 214 |
| [NP_003283.2](http://www.ncbi.nlm.nih.gov/entrez/query.fcgi?cmd=Retrieve&db=Protein&list_uids=114155142&dopt=GenPept&RID=T2WPRKGM016&log$=prottop&blast_rank=64) | nuclear pore complex-associated protein TPR [Homo sapiens] | [19.3](http://blast.ncbi.nlm.nih.gov/Blast.cgi#114155142%23114155142) | 19.3 | 62% | 214 |
| [XP_001724422.1](http://www.ncbi.nlm.nih.gov/entrez/query.fcgi?cmd=Retrieve&db=Protein&list_uids=169161476&dopt=GenPept&RID=T2WPRKGM016&log$=prottop&blast_rank=65) | PREDICTED: hypothetical protein [Homo sapiens] | [19.3](http://blast.ncbi.nlm.nih.gov/Blast.cgi#169161476%23169161476) | 19.3 | 62% | 214 |
| [NP_821075.1](http://www.ncbi.nlm.nih.gov/entrez/query.fcgi?cmd=Retrieve&db=Protein&list_uids=41281907&dopt=GenPept&RID=T2WPRKGM016&log$=prottop&blast_rank=66) | StAR-related lipid transfer (START) domain containing 13 isoform beta [Homo sapiens] | [19.3](http://blast.ncbi.nlm.nih.gov/Blast.cgi#41281907%2341281907) | 19.3 | 75% | 214 |
| [NP_071342.2](http://www.ncbi.nlm.nih.gov/entrez/query.fcgi?cmd=Retrieve&db=Protein&list_uids=154816176&dopt=GenPept&RID=T2WPRKGM016&log$=prottop&blast_rank=67) | chemokine (C-X-C motif) ligand 16 [Homo sapiens] >ref|NP_001094282.1| chemokine (C-X-C motif) ligand 16 [Homo sapiens] | [19.3](http://blast.ncbi.nlm.nih.gov/Blast.cgi#154816176%23154816176) | 19.3 | 62% | 214 |
| [NP_821074.1](http://www.ncbi.nlm.nih.gov/entrez/query.fcgi?cmd=Retrieve&db=Protein&list_uids=41281898&dopt=GenPept&RID=T2WPRKGM016&log$=prottop&blast_rank=68) | StAR-related lipid transfer (START) domain containing 13 isoform alpha [Homo sapiens] | [19.3](http://blast.ncbi.nlm.nih.gov/Blast.cgi#41281898%2341281898) | 19.3 | 75% | 214 |
| [NP_001138437.1](http://www.ncbi.nlm.nih.gov/entrez/query.fcgi?cmd=Retrieve&db=Protein&list_uids=222352082&dopt=GenPept&RID=T2WPRKGM016&log$=prottop&blast_rank=69) | neural precursor cell expressed, developmentally down-regulated 4-like isoform 2 [Homo sapiens] >ref|NP_001138438.1| neural precursor cell expressed, developmentally down-regulated 4-like isoform 2 [Homo sapiens] >ref|NP_001138436.1| neural precursor cell expressed, developmentally down-regulated 4-like isoform 2 [Homo sapiens] | [19.3](http://blast.ncbi.nlm.nih.gov/Blast.cgi#222352082%23222352082) | 19.3 | 62% | 214 |
| [NP_001003698.1](http://www.ncbi.nlm.nih.gov/entrez/query.fcgi?cmd=Retrieve&db=Protein&list_uids=51173735&dopt=GenPept&RID=T2WPRKGM016&log$=prottop&blast_rank=70) | ras responsive element binding protein 1 isoform 2 [Homo sapiens] >ref|NP_001161816.1| ras responsive element binding protein 1 isoform 2 [Homo sapiens] | [19.3](http://blast.ncbi.nlm.nih.gov/Blast.cgi#51173735%2351173735) | 19.3 | 75% | 214 |
| [NP_071754.3](http://www.ncbi.nlm.nih.gov/entrez/query.fcgi?cmd=Retrieve&db=Protein&list_uids=50658075&dopt=GenPept&RID=T2WPRKGM016&log$=prottop&blast_rank=71) | phosphodiesterase 4D interacting protein isoform 3 [Homo sapiens] | [19.3](http://blast.ncbi.nlm.nih.gov/Blast.cgi#50658075%2350658075) | 19.3 | 62% | 214 |
| [NP_149038.2](http://www.ncbi.nlm.nih.gov/entrez/query.fcgi?cmd=Retrieve&db=Protein&list_uids=51593090&dopt=GenPept&RID=T2WPRKGM016&log$=prottop&blast_rank=72) | mucin 13, epithelial transmembrane [Homo sapiens] | [19.3](http://blast.ncbi.nlm.nih.gov/Blast.cgi#51593090%2351593090) | 19.3 | 62% | 214 |
| [NP_001074001.1](http://www.ncbi.nlm.nih.gov/entrez/query.fcgi?cmd=Retrieve&db=Protein&list_uids=122937502&dopt=GenPept&RID=T2WPRKGM016&log$=prottop&blast_rank=73) | transmembrane channel-like 3 [Homo sapiens] | [19.3](http://blast.ncbi.nlm.nih.gov/Blast.cgi#122937502%23122937502) | 19.3 | 62% | 214 |
| [NP_078947.3](http://www.ncbi.nlm.nih.gov/entrez/query.fcgi?cmd=Retrieve&db=Protein&list_uids=146198654&dopt=GenPept&RID=T2WPRKGM016&log$=prottop&blast_rank=74) | zinc finger protein 768 [Homo sapiens] | [19.3](http://blast.ncbi.nlm.nih.gov/Blast.cgi#146198654%23146198654) | 85.1 | 87% | 214 |
| [NP_001138442.1](http://www.ncbi.nlm.nih.gov/entrez/query.fcgi?cmd=Retrieve&db=Protein&list_uids=222352092&dopt=GenPept&RID=T2WPRKGM016&log$=prottop&blast_rank=75) | neural precursor cell expressed, developmentally down-regulated 4-like isoform 6 [Homo sapiens] >ref|NP_001138443.1| neural precursor cell expressed, developmentally down-regulated 4-like isoform 6 [Homo sapiens] | [19.3](http://blast.ncbi.nlm.nih.gov/Blast.cgi#222352092%23222352092) | 19.3 | 62% | 214 |
| [NP_001003699.1](http://www.ncbi.nlm.nih.gov/entrez/query.fcgi?cmd=Retrieve&db=Protein&list_uids=51173737&dopt=GenPept&RID=T2WPRKGM016&log$=prottop&blast_rank=76) | ras responsive element binding protein 1 isoform 1 [Homo sapiens] | [19.3](http://blast.ncbi.nlm.nih.gov/Blast.cgi#51173737%2351173737) | 19.3 | 75% | 214 |
| [NP_004707.2](http://www.ncbi.nlm.nih.gov/entrez/query.fcgi?cmd=Retrieve&db=Protein&list_uids=20336248&dopt=GenPept&RID=T2WPRKGM016&log$=prottop&blast_rank=77) | proprotein convertase subtilisin/kexin type 7 preproprotein [Homo sapiens] | [19.3](http://blast.ncbi.nlm.nih.gov/Blast.cgi#20336248%2320336248) | 19.3 | 62% | 214 |
| [NP_060849.2](http://www.ncbi.nlm.nih.gov/entrez/query.fcgi?cmd=Retrieve&db=Protein&list_uids=31377841&dopt=GenPept&RID=T2WPRKGM016&log$=prottop&blast_rank=78) | hypothetical protein LOC55793 isoform 1 [Homo sapiens] | [19.3](http://blast.ncbi.nlm.nih.gov/Blast.cgi#31377841%2331377841) | 36.1 | 75% | 214 |
| [NP_055243.1](http://www.ncbi.nlm.nih.gov/entrez/query.fcgi?cmd=Retrieve&db=Protein&list_uids=10092691&dopt=GenPept&RID=T2WPRKGM016&log$=prottop&blast_rank=79) | tight junction protein 3 [Homo sapiens] | [19.3](http://blast.ncbi.nlm.nih.gov/Blast.cgi#10092691%2310092691) | 19.3 | 62% | 214 |
| [NP_001138439.1](http://www.ncbi.nlm.nih.gov/entrez/query.fcgi?cmd=Retrieve&db=Protein&list_uids=222352086&dopt=GenPept&RID=T2WPRKGM016&log$=prottop&blast_rank=80) | neural precursor cell expressed, developmentally down-regulated 4-like isoform 1 [Homo sapiens] | [19.3](http://blast.ncbi.nlm.nih.gov/Blast.cgi#222352086%23222352086) | 19.3 | 62% | 214 |
| [NP_064553.1](http://www.ncbi.nlm.nih.gov/entrez/query.fcgi?cmd=Retrieve&db=Protein&list_uids=9910476&dopt=GenPept&RID=T2WPRKGM016&log$=prottop&blast_rank=81) | p21-activated kinase 6 [Homo sapiens] >ref|NP_001122100.1| p21-activated kinase 6 [Homo sapiens] >ref|NP_001122101.1| p21-activated kinase 6 [Homo sapiens] | [19.3](http://blast.ncbi.nlm.nih.gov/Blast.cgi#9910476%239910476) | 19.3 | 62% | 214 |
| [NP_689867.1](http://www.ncbi.nlm.nih.gov/entrez/query.fcgi?cmd=Retrieve&db=Protein&list_uids=22749329&dopt=GenPept&RID=T2WPRKGM016&log$=prottop&blast_rank=82) | dante precursor [Homo sapiens] | [19.3](http://blast.ncbi.nlm.nih.gov/Blast.cgi#22749329%2322749329) | 35.6 | 87% | 214 |
| [NP_072091.1](http://www.ncbi.nlm.nih.gov/entrez/query.fcgi?cmd=Retrieve&db=Protein&list_uids=12007650&dopt=GenPept&RID=T2WPRKGM016&log$=prottop&blast_rank=83) | N-deacetylase/N-sulfotransferase 4 [Homo sapiens] | [19.3](http://blast.ncbi.nlm.nih.gov/Blast.cgi#12007650%2312007650) | 19.3 | 62% | 214 |
| [NP_056092.2](http://www.ncbi.nlm.nih.gov/entrez/query.fcgi?cmd=Retrieve&db=Protein&list_uids=21361472&dopt=GenPept&RID=T2WPRKGM016&log$=prottop&blast_rank=84) | neural precursor cell expressed, developmentally down-regulated 4-like isoform 3 [Homo sapiens] | [19.3](http://blast.ncbi.nlm.nih.gov/Blast.cgi#21361472%2321361472) | 19.3 | 62% | 214 |
| [NP_001008910.1](http://www.ncbi.nlm.nih.gov/entrez/query.fcgi?cmd=Retrieve&db=Protein&list_uids=57165436&dopt=GenPept&RID=T2WPRKGM016&log$=prottop&blast_rank=85) | serine/threonine kinase 16 [Homo sapiens] | [19.3](http://blast.ncbi.nlm.nih.gov/Blast.cgi#57165436%2357165436) | 19.3 | 62% | 214 |
| [NP_443083.1](http://www.ncbi.nlm.nih.gov/entrez/query.fcgi?cmd=Retrieve&db=Protein&list_uids=16445031&dopt=GenPept&RID=T2WPRKGM016&log$=prottop&blast_rank=86) | StAR-related lipid transfer (START) domain containing 13 isoform gamma [Homo sapiens] | [19.3](http://blast.ncbi.nlm.nih.gov/Blast.cgi#16445031%2316445031) | 19.3 | 75% | 214 |
| [NP_001977.1](http://www.ncbi.nlm.nih.gov/entrez/query.fcgi?cmd=Retrieve&db=Protein&list_uids=24307883&dopt=GenPept&RID=T2WPRKGM016&log$=prottop&blast_rank=87) | ets variant gene 4 (E1A enhancer binding protein, E1AF) [Homo sapiens] >ref|NP_001073143.1| ets variant gene 4 (E1A enhancer binding protein, E1AF) [Homo sapiens] | [19.3](http://blast.ncbi.nlm.nih.gov/Blast.cgi#24307883%2324307883) | 19.3 | 62% | 214 |
| [NP_005517.1](http://www.ncbi.nlm.nih.gov/entrez/query.fcgi?cmd=Retrieve&db=Protein&list_uids=5031767&dopt=GenPept&RID=T2WPRKGM016&log$=prottop&blast_rank=88) | heat shock transcription factor 1 [Homo sapiens] | [19.3](http://blast.ncbi.nlm.nih.gov/Blast.cgi#5031767%235031767) | 19.3 | 62% | 214 |
| [NP_057737.2](http://www.ncbi.nlm.nih.gov/entrez/query.fcgi?cmd=Retrieve&db=Protein&list_uids=82880648&dopt=GenPept&RID=T2WPRKGM016&log$=prottop&blast_rank=89) | MLK-related kinase isoform 1 [Homo sapiens] | [19.3](http://blast.ncbi.nlm.nih.gov/Blast.cgi#82880648%2382880648) | 19.3 | 100% | 214 |
| [NP_001469.1](http://www.ncbi.nlm.nih.gov/entrez/query.fcgi?cmd=Retrieve&db=Protein&list_uids=4503893&dopt=GenPept&RID=T2WPRKGM016&log$=prottop&blast_rank=90) | beta-1,4-N-acetyl-galactosaminyl transferase 1 [Homo sapiens] | [19.3](http://blast.ncbi.nlm.nih.gov/Blast.cgi#4503893%234503893) | 19.3 | 62% | 214 |
| [XP_002345546.1](http://www.ncbi.nlm.nih.gov/entrez/query.fcgi?cmd=Retrieve&db=Protein&list_uids=239757736&dopt=GenPept&RID=T2WPRKGM016&log$=prottop&blast_rank=91) | PREDICTED: hypothetical protein [Homo sapiens] | [18.9](http://blast.ncbi.nlm.nih.gov/Blast.cgi#239757736%23239757736) | 18.9 | 87% | 288 |
| [XP_002348167.1](http://www.ncbi.nlm.nih.gov/entrez/query.fcgi?cmd=Retrieve&db=Protein&list_uids=239752235&dopt=GenPept&RID=T2WPRKGM016&log$=prottop&blast_rank=92) | PREDICTED: hypothetical protein XP_002348167 [Homo sapiens] | [18.9](http://blast.ncbi.nlm.nih.gov/Blast.cgi#239752235%23239752235) | 18.9 | 87% | 288 |
| [XP_002342198.1](http://www.ncbi.nlm.nih.gov/entrez/query.fcgi?cmd=Retrieve&db=Protein&list_uids=239741430&dopt=GenPept&RID=T2WPRKGM016&log$=prottop&blast_rank=93) | PREDICTED: hypothetical protein XP_002342198 [Homo sapiens] >ref|XP_002345321.1| PREDICTED: hypothetical protein XP_002345321 [Homo sapiens] | [18.9](http://blast.ncbi.nlm.nih.gov/Blast.cgi#239741430%23239741430) | 34.8 | 75% | 288 |
| [NP_001156751.1](http://www.ncbi.nlm.nih.gov/entrez/query.fcgi?cmd=Retrieve&db=Protein&list_uids=253970446&dopt=GenPept&RID=T2WPRKGM016&log$=prottop&blast_rank=94) | odz, odd Oz/ten-m homolog 1 isoform 2 [Homo sapiens] | [18.9](http://blast.ncbi.nlm.nih.gov/Blast.cgi#253970446%23253970446) | 18.9 | 62% | 288 |
| [NP_001156750.1](http://www.ncbi.nlm.nih.gov/entrez/query.fcgi?cmd=Retrieve&db=Protein&list_uids=253970444&dopt=GenPept&RID=T2WPRKGM016&log$=prottop&blast_rank=95) | odz, odd Oz/ten-m homolog 1 isoform 1 [Homo sapiens] | [18.9](http://blast.ncbi.nlm.nih.gov/Blast.cgi#253970444%23253970444) | 18.9 | 62% | 288 |
| [NP_001120727.1](http://www.ncbi.nlm.nih.gov/entrez/query.fcgi?cmd=Retrieve&db=Protein&list_uids=187937176&dopt=GenPept&RID=T2WPRKGM016&log$=prottop&blast_rank=96) | NACHT, leucine rich repeat and PYD containing 7 isoform 3 [Homo sapiens] | [18.9](http://blast.ncbi.nlm.nih.gov/Blast.cgi#187937176%23187937176) | 18.9 | 87% | 288 |
| [NP_001099023.1](http://www.ncbi.nlm.nih.gov/entrez/query.fcgi?cmd=Retrieve&db=Protein&list_uids=157738661&dopt=GenPept&RID=T2WPRKGM016&log$=prottop&blast_rank=97) | zinc finger protein 83 isoform b [Homo sapiens] >ref|NP_001099024.1| zinc finger protein 83 isoform b [Homo sapiens] | [18.9](http://blast.ncbi.nlm.nih.gov/Blast.cgi#157738661%23157738661) | 18.9 | 62% | 288 |
| [NP_060770.3](http://www.ncbi.nlm.nih.gov/entrez/query.fcgi?cmd=Retrieve&db=Protein&list_uids=157738651&dopt=GenPept&RID=T2WPRKGM016&log$=prottop&blast_rank=98) | zinc finger protein 83 isoform a [Homo sapiens] >ref|NP_001099019.1| zinc finger protein 83 isoform a [Homo sapiens] >ref|NP_001099020.1| zinc finger protein 83 isoform a [Homo sapiens] >ref|NP_001099021.1| zinc finger protein 83 isoform a [Homo sapiens] >ref|NP_001099022.1| zinc finger protein 83 isoform a [Homo sapiens] | [18.9](http://blast.ncbi.nlm.nih.gov/Blast.cgi#157738651%23157738651) | 18.9 | 62% | 288 |
| [NP_689472.3](http://www.ncbi.nlm.nih.gov/entrez/query.fcgi?cmd=Retrieve&db=Protein&list_uids=118421085&dopt=GenPept&RID=T2WPRKGM016&log$=prottop&blast_rank=99) | leucine-rich repeat kinase 1 [Homo sapiens] | [18.9](http://blast.ncbi.nlm.nih.gov/Blast.cgi#118421085%23118421085) | 34.8 | 100% | 288 |
| [NP_001165408.1](http://www.ncbi.nlm.nih.gov/entrez/query.fcgi?cmd=Retrieve&db=Protein&list_uids=288541308&dopt=GenPept&RID=T2WPRKGM016&log$=prottop&blast_rank=100) | fuzzy homolog isoform 2 [Homo sapiens] | [18.9](http://blast.ncbi.nlm.nih.gov/Blast.cgi#288541308%23288541308) | 35.2 | 100% | 288 |

| **Accession** | **Proteins with a match to YSWRAT peptide** | **[Max score](http://blast.ncbi.nlm.nih.gov/Blast.cgi?CMD=Get&ALIGNMENTS=100&ALIGNMENT_VIEW=Pairwise&CDD_SEARCH_STATE=1&DATABASE_SORT=0&DESCRIPTIONS=100&ENTREZ_QUERY=txid9606 %5BORGN%5D&FIRST_QUERY_NUM=0&FORMAT_OBJECT=Alignment&FORMAT_PAGE_TARGET=&FORMAT_TYPE=HTML&GET_SEQUENCE=yes&I_THRESH=&MASK_CHAR=2&MASK_COLOR=1&NEW_DESIGN=on&NEW_VIEW=yes&NUM_OVERVIEW=100&OLD_BLAST=false&PAGE=Proteins&QUERY_INDEX=0&QUERY_NUMBER=0&RESULTS_PAGE_TARGET=&RID=T2WV9WH201N&SHOW_LINKOUT=yes&SHOW_OVERVIEW=yes&STEP_NUMBER=&WORD_SIZE=2&DISPLAY_SORT=1&HSP_SORT=1" \l "sort_mark)** | **[Total score](http://blast.ncbi.nlm.nih.gov/Blast.cgi?CMD=Get&ALIGNMENTS=100&ALIGNMENT_VIEW=Pairwise&CDD_SEARCH_STATE=1&DATABASE_SORT=0&DESCRIPTIONS=100&ENTREZ_QUERY=txid9606 %5BORGN%5D&FIRST_QUERY_NUM=0&FORMAT_OBJECT=Alignment&FORMAT_PAGE_TARGET=&FORMAT_TYPE=HTML&GET_SEQUENCE=yes&I_THRESH=&MASK_CHAR=2&MASK_COLOR=1&NEW_DESIGN=on&NEW_VIEW=yes&NUM_OVERVIEW=100&OLD_BLAST=false&PAGE=Proteins&QUERY_INDEX=0&QUERY_NUMBER=0&RESULTS_PAGE_TARGET=&RID=T2WV9WH201N&SHOW_LINKOUT=yes&SHOW_OVERVIEW=yes&STEP_NUMBER=&WORD_SIZE=2&DISPLAY_SORT=2&HSP_SORT=1" \l "sort_mark)** | **[Query coverage](http://blast.ncbi.nlm.nih.gov/Blast.cgi?CMD=Get&ALIGNMENTS=100&ALIGNMENT_VIEW=Pairwise&CDD_SEARCH_STATE=1&DATABASE_SORT=0&DESCRIPTIONS=100&ENTREZ_QUERY=txid9606 %5BORGN%5D&FIRST_QUERY_NUM=0&FORMAT_OBJECT=Alignment&FORMAT_PAGE_TARGET=&FORMAT_TYPE=HTML&GET_SEQUENCE=yes&I_THRESH=&MASK_CHAR=2&MASK_COLOR=1&NEW_DESIGN=on&NEW_VIEW=yes&NUM_OVERVIEW=100&OLD_BLAST=false&PAGE=Proteins&QUERY_INDEX=0&QUERY_NUMBER=0&RESULTS_PAGE_TARGET=&RID=T2WV9WH201N&SHOW_LINKOUT=yes&SHOW_OVERVIEW=yes&STEP_NUMBER=&WORD_SIZE=2&DISPLAY_SORT=4&HSP_SORT=0" \l "sort_mark)** | **[E value](http://blast.ncbi.nlm.nih.gov/Blast.cgi?CMD=Get&ALIGNMENTS=100&ALIGNMENT_VIEW=Pairwise&CDD_SEARCH_STATE=1&DATABASE_SORT=0&DESCRIPTIONS=100&ENTREZ_QUERY=txid9606 %5BORGN%5D&FIRST_QUERY_NUM=0&FORMAT_OBJECT=Alignment&FORMAT_PAGE_TARGET=&FORMAT_TYPE=HTML&GET_SEQUENCE=yes&I_THRESH=&MASK_CHAR=2&MASK_COLOR=1&NEW_DESIGN=on&NEW_VIEW=yes&NUM_OVERVIEW=100&OLD_BLAST=false&PAGE=Proteins&QUERY_INDEX=0&QUERY_NUMBER=0&RESULTS_PAGE_TARGET=&RID=T2WV9WH201N&SHOW_LINKOUT=yes&SHOW_OVERVIEW=yes&STEP_NUMBER=&WORD_SIZE=2&DISPLAY_SORT=0&HSP_SORT=0" \l "sort_mark)** |
| --- | --- | --- | --- | --- | --- |
| [NP_001157751.1](http://www.ncbi.nlm.nih.gov/entrez/query.fcgi?cmd=Retrieve&db=Protein&list_uids=256219564&dopt=GenPept&RID=T2WV9WH201N&log$=prottop&blast_rank=1) | solute carrier family 37 (glucose-6-phosphate transporter), member 4 isoform 3 [Homo sapiens] | [21.8](http://blast.ncbi.nlm.nih.gov/Blast.cgi#256219564%23256219564) | 21.8 | 100% | 28 |
| [NP_001458.1](http://www.ncbi.nlm.nih.gov/entrez/query.fcgi?cmd=Retrieve&db=Protein&list_uids=4503847&dopt=GenPept&RID=T2WV9WH201N&log$=prottop&blast_rank=2) | solute carrier family 37 (glucose-6-phosphate transporter), member 4 isoform 1 [Homo sapiens] >ref|NP_001157749.1| solute carrier family 37 (glucose-6-phosphate transporter), member 4 isoform a [Homo sapiens] >ref|NP_001157752.1| solute carrier family 37 (glucose-6-phosphate transporter), member 4 isoform 1 [Homo sapiens] | [21.8](http://blast.ncbi.nlm.nih.gov/Blast.cgi#4503847%234503847) | 21.8 | 100% | 28 |
| [NP_001157750.1](http://www.ncbi.nlm.nih.gov/entrez/query.fcgi?cmd=Retrieve&db=Protein&list_uids=256219543&dopt=GenPept&RID=T2WV9WH201N&log$=prottop&blast_rank=3) | solute carrier family 37 (glucose-6-phosphate transporter), member 4 isoform 2 [Homo sapiens] | [21.8](http://blast.ncbi.nlm.nih.gov/Blast.cgi#256219543%23256219543) | 21.8 | 100% | 28 |
| [NP_001158152.1](http://www.ncbi.nlm.nih.gov/entrez/query.fcgi?cmd=Retrieve&db=Protein&list_uids=257743048&dopt=GenPept&RID=T2WV9WH201N&log$=prottop&blast_rank=4) | CC chemokine receptor 3 isoform 3 [Homo sapiens] | [18.9](http://blast.ncbi.nlm.nih.gov/Blast.cgi#257743048%23257743048) | 18.9 | 66% | 216 |
| [NP_847898.1](http://www.ncbi.nlm.nih.gov/entrez/query.fcgi?cmd=Retrieve&db=Protein&list_uids=257743046&dopt=GenPept&RID=T2WV9WH201N&log$=prottop&blast_rank=5) | CC chemokine receptor 3 isoform 2 [Homo sapiens] | [18.9](http://blast.ncbi.nlm.nih.gov/Blast.cgi#257743046%23257743046) | 18.9 | 66% | 216 |
| [XP_002342595.1](http://www.ncbi.nlm.nih.gov/entrez/query.fcgi?cmd=Retrieve&db=Protein&list_uids=239742682&dopt=GenPept&RID=T2WV9WH201N&log$=prottop&blast_rank=6) | PREDICTED: hypothetical protein XP_002342595 [Homo sapiens] >ref|XP_002346770.1| PREDICTED: hypothetical protein XP_002346770 [Homo sapiens] >ref|XP_002345867.1| PREDICTED: hypothetical protein [Homo sapiens] | [18.9](http://blast.ncbi.nlm.nih.gov/Blast.cgi#239742682%23239742682) | 18.9 | 66% | 216 |
| [NP_001139502.1](http://www.ncbi.nlm.nih.gov/entrez/query.fcgi?cmd=Retrieve&db=Protein&list_uids=225637524&dopt=GenPept&RID=T2WV9WH201N&log$=prottop&blast_rank=7) | semaphorin 7A isoform 3 [Homo sapiens] | [18.9](http://blast.ncbi.nlm.nih.gov/Blast.cgi#225637524%23225637524) | 18.9 | 66% | 216 |
| [NP_001139501.1](http://www.ncbi.nlm.nih.gov/entrez/query.fcgi?cmd=Retrieve&db=Protein&list_uids=225637520&dopt=GenPept&RID=T2WV9WH201N&log$=prottop&blast_rank=8) | semaphorin 7A isoform 2 preproprotein [Homo sapiens] | [18.9](http://blast.ncbi.nlm.nih.gov/Blast.cgi#225637520%23225637520) | 18.9 | 66% | 216 |
| [NP_004354.2](http://www.ncbi.nlm.nih.gov/entrez/query.fcgi?cmd=Retrieve&db=Protein&list_uids=98986445&dopt=GenPept&RID=T2WV9WH201N&log$=prottop&blast_rank=9) | carcinoembryonic antigen-related cell adhesion molecule 5 preproprotein [Homo sapiens] | [18.9](http://blast.ncbi.nlm.nih.gov/Blast.cgi#98986445%2398986445) | 18.9 | 66% | 216 |
| [XP_001724522.1](http://www.ncbi.nlm.nih.gov/entrez/query.fcgi?cmd=Retrieve&db=Protein&list_uids=169168320&dopt=GenPept&RID=T2WV9WH201N&log$=prottop&blast_rank=10) | PREDICTED: similar to hCG39609 [Homo sapiens] >ref|XP_002342586.1| PREDICTED: similar to hCG39609 [Homo sapiens] >ref|XP_002346742.1| PREDICTED: similar to hCG39609 [Homo sapiens] | [18.9](http://blast.ncbi.nlm.nih.gov/Blast.cgi#169168320%23169168320) | 18.9 | 83% | 216 |
| [NP_775838.3](http://www.ncbi.nlm.nih.gov/entrez/query.fcgi?cmd=Retrieve&db=Protein&list_uids=218777837&dopt=GenPept&RID=T2WV9WH201N&log$=prottop&blast_rank=11) | abhydrolase domain containing 7 [Homo sapiens] | [18.9](http://blast.ncbi.nlm.nih.gov/Blast.cgi#218777837%23218777837) | 18.9 | 66% | 216 |
| [NP_775463.1](http://www.ncbi.nlm.nih.gov/entrez/query.fcgi?cmd=Retrieve&db=Protein&list_uids=27894287&dopt=GenPept&RID=T2WV9WH201N&log$=prottop&blast_rank=12) | PHD finger protein 7 isoform 2 [Homo sapiens] | [18.9](http://blast.ncbi.nlm.nih.gov/Blast.cgi#27894287%2327894287) | 18.9 | 66% | 216 |
| [NP_001073896.1](http://www.ncbi.nlm.nih.gov/entrez/query.fcgi?cmd=Retrieve&db=Protein&list_uids=122937257&dopt=GenPept&RID=T2WV9WH201N&log$=prottop&blast_rank=13) | thrombospondin, type I, domain containing 7B [Homo sapiens] | [18.9](http://blast.ncbi.nlm.nih.gov/Blast.cgi#122937257%23122937257) | 18.9 | 66% | 216 |
| [NP_001019851.1](http://www.ncbi.nlm.nih.gov/entrez/query.fcgi?cmd=Retrieve&db=Protein&list_uids=67003566&dopt=GenPept&RID=T2WV9WH201N&log$=prottop&blast_rank=14) | F-box protein 48 [Homo sapiens] | [18.9](http://blast.ncbi.nlm.nih.gov/Blast.cgi#67003566%2367003566) | 18.9 | 66% | 216 |
| [NP_060606.3](http://www.ncbi.nlm.nih.gov/entrez/query.fcgi?cmd=Retrieve&db=Protein&list_uids=126116596&dopt=GenPept&RID=T2WV9WH201N&log$=prottop&blast_rank=15) | asp (abnormal spindle)-like, microcephaly associated [Homo sapiens] | [18.9](http://blast.ncbi.nlm.nih.gov/Blast.cgi#126116596%23126116596) | 18.9 | 66% | 216 |
| [NP_891550.1](http://www.ncbi.nlm.nih.gov/entrez/query.fcgi?cmd=Retrieve&db=Protein&list_uids=33624896&dopt=GenPept&RID=T2WV9WH201N&log$=prottop&blast_rank=16) | ADAM metallopeptidase with thrombospondin type 1 motif, 9 preproprotein [Homo sapiens] | [18.9](http://blast.ncbi.nlm.nih.gov/Blast.cgi#33624896%2333624896) | 18.9 | 83% | 216 |
| [NP_002196.2](http://www.ncbi.nlm.nih.gov/entrez/query.fcgi?cmd=Retrieve&db=Protein&list_uids=56237029&dopt=GenPept&RID=T2WV9WH201N&log$=prottop&blast_rank=17) | integrin alpha 5 precursor [Homo sapiens] | [18.9](http://blast.ncbi.nlm.nih.gov/Blast.cgi#56237029%2356237029) | 18.9 | 66% | 216 |
| [NP_203124.1](http://www.ncbi.nlm.nih.gov/entrez/query.fcgi?cmd=Retrieve&db=Protein&list_uids=15718698&dopt=GenPept&RID=T2WV9WH201N&log$=prottop&blast_rank=18) | caspase 7 isoform delta [Homo sapiens] | [18.9](http://blast.ncbi.nlm.nih.gov/Blast.cgi#15718698%2315718698) | 18.9 | 66% | 216 |
| [NP_001006611.1](http://www.ncbi.nlm.nih.gov/entrez/query.fcgi?cmd=Retrieve&db=Protein&list_uids=55749557&dopt=GenPept&RID=T2WV9WH201N&log$=prottop&blast_rank=19) | seven in absentia homolog 1 isoform b [Homo sapiens] | [18.9](http://blast.ncbi.nlm.nih.gov/Blast.cgi#55749557%2355749557) | 29.3 | 100% | 216 |
| [NP_001098684.1](http://www.ncbi.nlm.nih.gov/entrez/query.fcgi?cmd=Retrieve&db=Protein&list_uids=157412282&dopt=GenPept&RID=T2WV9WH201N&log$=prottop&blast_rank=20) | ash2-like isoform b [Homo sapiens] | [18.9](http://blast.ncbi.nlm.nih.gov/Blast.cgi#157412282%23157412282) | 18.9 | 66% | 216 |
| [NP_742001.1](http://www.ncbi.nlm.nih.gov/entrez/query.fcgi?cmd=Retrieve&db=Protein&list_uids=25188187&dopt=GenPept&RID=T2WV9WH201N&log$=prottop&blast_rank=21) | type II transmembrane protein DCAL1 [Homo sapiens] | [18.9](http://blast.ncbi.nlm.nih.gov/Blast.cgi#25188187%2325188187) | 18.9 | 66% | 216 |
| [NP_689777.3](http://www.ncbi.nlm.nih.gov/entrez/query.fcgi?cmd=Retrieve&db=Protein&list_uids=35493701&dopt=GenPept&RID=T2WV9WH201N&log$=prottop&blast_rank=22) | vacuolar protein sorting 13B isoform 1 [Homo sapiens] | [18.9](http://blast.ncbi.nlm.nih.gov/Blast.cgi#35493701%2335493701) | 18.9 | 66% | 216 |
| [NP_060360.3](http://www.ncbi.nlm.nih.gov/entrez/query.fcgi?cmd=Retrieve&db=Protein&list_uids=35493713&dopt=GenPept&RID=T2WV9WH201N&log$=prottop&blast_rank=23) | vacuolar protein sorting 13B isoform 5 [Homo sapiens] | [18.9](http://blast.ncbi.nlm.nih.gov/Blast.cgi#35493713%2335493713) | 18.9 | 66% | 216 |
| [NP_001106970.1](http://www.ncbi.nlm.nih.gov/entrez/query.fcgi?cmd=Retrieve&db=Protein&list_uids=164698517&dopt=GenPept&RID=T2WV9WH201N&log$=prottop&blast_rank=24) | MAM domain containing 1 isoform 1 [Homo sapiens] | [18.9](http://blast.ncbi.nlm.nih.gov/Blast.cgi#164698517%23164698517) | 18.9 | 66% | 216 |
| [NP_002111.1](http://www.ncbi.nlm.nih.gov/entrez/query.fcgi?cmd=Retrieve&db=Protein&list_uids=4504403&dopt=GenPept&RID=T2WV9WH201N&log$=prottop&blast_rank=25) | major histocompatibility complex, class II, DO beta precursor [Homo sapiens] | [18.9](http://blast.ncbi.nlm.nih.gov/Blast.cgi#4504403%234504403) | 18.9 | 66% | 216 |
| [NP_001218.1](http://www.ncbi.nlm.nih.gov/entrez/query.fcgi?cmd=Retrieve&db=Protein&list_uids=4502581&dopt=GenPept&RID=T2WV9WH201N&log$=prottop&blast_rank=26) | caspase 7 isoform alpha precursor [Homo sapiens] >ref|NP_203125.1| caspase 7 isoform alpha [Homo sapiens] | [18.9](http://blast.ncbi.nlm.nih.gov/Blast.cgi#4502581%234502581) | 18.9 | 66% | 216 |
| [NP_006636.2](http://www.ncbi.nlm.nih.gov/entrez/query.fcgi?cmd=Retrieve&db=Protein&list_uids=116812600&dopt=GenPept&RID=T2WV9WH201N&log$=prottop&blast_rank=27) | START domain containing 10 [Homo sapiens] | [18.9](http://blast.ncbi.nlm.nih.gov/Blast.cgi#116812600%23116812600) | 18.9 | 66% | 216 |
| [NP_001080.2](http://www.ncbi.nlm.nih.gov/entrez/query.fcgi?cmd=Retrieve&db=Protein&list_uids=116734710&dopt=GenPept&RID=T2WV9WH201N&log$=prottop&blast_rank=28) | ATP-binding cassette, sub-family A member 3 [Homo sapiens] | [18.9](http://blast.ncbi.nlm.nih.gov/Blast.cgi#116734710%23116734710) | 32.2 | 83% | 216 |
| [NP_001828.1](http://www.ncbi.nlm.nih.gov/entrez/query.fcgi?cmd=Retrieve&db=Protein&list_uids=4502637&dopt=GenPept&RID=T2WV9WH201N&log$=prottop&blast_rank=29) | CC chemokine receptor 3 isoform 1 [Homo sapiens] >ref|NP_847899.1| CC chemokine receptor 3 isoform 1 [Homo sapiens] | [18.9](http://blast.ncbi.nlm.nih.gov/Blast.cgi#4502637%234502637) | 18.9 | 66% | 216 |
| [NP_004665.2](http://www.ncbi.nlm.nih.gov/entrez/query.fcgi?cmd=Retrieve&db=Protein&list_uids=157412280&dopt=GenPept&RID=T2WV9WH201N&log$=prottop&blast_rank=30) | ash2-like isoform a [Homo sapiens] | [18.9](http://blast.ncbi.nlm.nih.gov/Blast.cgi#157412280%23157412280) | 18.9 | 66% | 216 |
| [NP_116786.1](http://www.ncbi.nlm.nih.gov/entrez/query.fcgi?cmd=Retrieve&db=Protein&list_uids=14790115&dopt=GenPept&RID=T2WV9WH201N&log$=prottop&blast_rank=31) | caspase 3 preproprotein [Homo sapiens] >ref|NP_004337.2| caspase 3 preproprotein [Homo sapiens] | [18.9](http://blast.ncbi.nlm.nih.gov/Blast.cgi#14790115%2314790115) | 18.9 | 66% | 216 |
| [NP_008914.1](http://www.ncbi.nlm.nih.gov/entrez/query.fcgi?cmd=Retrieve&db=Protein&list_uids=5902004&dopt=GenPept&RID=T2WV9WH201N&log$=prottop&blast_rank=32) | matrix metalloproteinase 23B precursor [Homo sapiens] | [18.9](http://blast.ncbi.nlm.nih.gov/Blast.cgi#5902004%235902004) | 34.4 | 66% | 216 |
| [NP_064510.1](http://www.ncbi.nlm.nih.gov/entrez/query.fcgi?cmd=Retrieve&db=Protein&list_uids=9910342&dopt=GenPept&RID=T2WV9WH201N&log$=prottop&blast_rank=33) | SLAM family member 8 precursor [Homo sapiens] | [18.9](http://blast.ncbi.nlm.nih.gov/Blast.cgi#9910342%239910342) | 18.9 | 66% | 216 |
| [NP_003603.1](http://www.ncbi.nlm.nih.gov/entrez/query.fcgi?cmd=Retrieve&db=Protein&list_uids=4504237&dopt=GenPept&RID=T2WV9WH201N&log$=prottop&blast_rank=34) | semaphorin 7A isoform 1 preproprotein [Homo sapiens] | [18.9](http://blast.ncbi.nlm.nih.gov/Blast.cgi#4504237%234504237) | 18.9 | 66% | 216 |
| [NP_775900.1](http://www.ncbi.nlm.nih.gov/entrez/query.fcgi?cmd=Retrieve&db=Protein&list_uids=27734983&dopt=GenPept&RID=T2WV9WH201N&log$=prottop&blast_rank=35) | hypothetical protein LOC284254 [Homo sapiens] | [18.9](http://blast.ncbi.nlm.nih.gov/Blast.cgi#27734983%2327734983) | 18.9 | 66% | 216 |
| [NP_057567.3](http://www.ncbi.nlm.nih.gov/entrez/query.fcgi?cmd=Retrieve&db=Protein&list_uids=21361543&dopt=GenPept&RID=T2WV9WH201N&log$=prottop&blast_rank=36) | PHD finger protein 7 isoform 1 [Homo sapiens] | [18.9](http://blast.ncbi.nlm.nih.gov/Blast.cgi#21361543%2321361543) | 18.9 | 66% | 216 |
| [NP_001970.2](http://www.ncbi.nlm.nih.gov/entrez/query.fcgi?cmd=Retrieve&db=Protein&list_uids=27597073&dopt=GenPept&RID=T2WV9WH201N&log$=prottop&blast_rank=37) | epoxide hydrolase 2, cytoplasmic [Homo sapiens] | [18.9](http://blast.ncbi.nlm.nih.gov/Blast.cgi#27597073%2327597073) | 18.9 | 66% | 216 |
| [NP_001120730.1](http://www.ncbi.nlm.nih.gov/entrez/query.fcgi?cmd=Retrieve&db=Protein&list_uids=187936955&dopt=GenPept&RID=T2WV9WH201N&log$=prottop&blast_rank=38) | HHIP-like protein 1 isoform a [Homo sapiens] | [18.0](http://blast.ncbi.nlm.nih.gov/Blast.cgi#187936955%23187936955) | 18.0 | 83% | 388 |
| [NP_115801.3](http://www.ncbi.nlm.nih.gov/entrez/query.fcgi?cmd=Retrieve&db=Protein&list_uids=187936953&dopt=GenPept&RID=T2WV9WH201N&log$=prottop&blast_rank=39) | HHIP-like protein 1 isoform b [Homo sapiens] | [18.0](http://blast.ncbi.nlm.nih.gov/Blast.cgi#187936953%23187936953) | 18.0 | 83% | 388 |
| [NP_001073939.1](http://www.ncbi.nlm.nih.gov/entrez/query.fcgi?cmd=Retrieve&db=Protein&list_uids=122937333&dopt=GenPept&RID=T2WV9WH201N&log$=prottop&blast_rank=40) | zinc finger protein 697 [Homo sapiens] | [18.0](http://blast.ncbi.nlm.nih.gov/Blast.cgi#122937333%23122937333) | 18.0 | 83% | 388 |
| [NP_079022.2](http://www.ncbi.nlm.nih.gov/entrez/query.fcgi?cmd=Retrieve&db=Protein&list_uids=124248546&dopt=GenPept&RID=T2WV9WH201N&log$=prottop&blast_rank=41) | hedgehog interacting protein-like 2 precursor [Homo sapiens] | [18.0](http://blast.ncbi.nlm.nih.gov/Blast.cgi#124248546%23124248546) | 18.0 | 83% | 388 |
| [NP_003015.2](http://www.ncbi.nlm.nih.gov/entrez/query.fcgi?cmd=Retrieve&db=Protein&list_uids=47717123&dopt=GenPept&RID=T2WV9WH201N&log$=prottop&blast_rank=42) | intersectin 1 isoform ITSN-l [Homo sapiens] | [18.0](http://blast.ncbi.nlm.nih.gov/Blast.cgi#47717123%2347717123) | 18.0 | 83% | 388 |
| [NP_001001132.1](http://www.ncbi.nlm.nih.gov/entrez/query.fcgi?cmd=Retrieve&db=Protein&list_uids=47717125&dopt=GenPept&RID=T2WV9WH201N&log$=prottop&blast_rank=43) | intersectin 1 isoform ITSN-s [Homo sapiens] | [18.0](http://blast.ncbi.nlm.nih.gov/Blast.cgi#47717125%2347717125) | 18.0 | 83% | 388 |
| [NP_005122.2](http://www.ncbi.nlm.nih.gov/entrez/query.fcgi?cmd=Retrieve&db=Protein&list_uids=154448890&dopt=GenPept&RID=T2WV9WH201N&log$=prottop&blast_rank=44) | THO complex 1 [Homo sapiens] | [18.0](http://blast.ncbi.nlm.nih.gov/Blast.cgi#154448890%23154448890) | 18.0 | 83% | 388 |
| [NP_001164225.1](http://www.ncbi.nlm.nih.gov/entrez/query.fcgi?cmd=Retrieve&db=Protein&list_uids=282721090&dopt=GenPept&RID=T2WV9WH201N&log$=prottop&blast_rank=45) | hypothetical protein LOC148345 [Homo sapiens] | [17.6](http://blast.ncbi.nlm.nih.gov/Blast.cgi#282721090%23282721090) | 17.6 | 66% | 521 |
| [NP_001019628.3](http://www.ncbi.nlm.nih.gov/entrez/query.fcgi?cmd=Retrieve&db=Protein&list_uids=262118265&dopt=GenPept&RID=T2WV9WH201N&log$=prottop&blast_rank=46) | RANBP2-like and GRIP domain containing 1 [Homo sapiens] | [17.6](http://blast.ncbi.nlm.nih.gov/Blast.cgi#262118265%23262118265) | 17.6 | 66% | 521 |
| [NP_079350.5](http://www.ncbi.nlm.nih.gov/entrez/query.fcgi?cmd=Retrieve&db=Protein&list_uids=256000767&dopt=GenPept&RID=T2WV9WH201N&log$=prottop&blast_rank=47) | Fraser syndrome 1 protein isoform 1 precursor [Homo sapiens] | [17.6](http://blast.ncbi.nlm.nih.gov/Blast.cgi#256000767%23256000767) | 17.6 | 66% | 521 |
| [XP_002347229.1](http://www.ncbi.nlm.nih.gov/entrez/query.fcgi?cmd=Retrieve&db=Protein&list_uids=239749957&dopt=GenPept&RID=T2WV9WH201N&log$=prottop&blast_rank=48) | PREDICTED: hypothetical protein [Homo sapiens] | [17.6](http://blast.ncbi.nlm.nih.gov/Blast.cgi#239749957%23239749957) | 17.6 | 66% | 521 |
| [XP_002343745.1](http://www.ncbi.nlm.nih.gov/entrez/query.fcgi?cmd=Retrieve&db=Protein&list_uids=239746493&dopt=GenPept&RID=T2WV9WH201N&log$=prottop&blast_rank=49) | PREDICTED: hypothetical protein XP_002343745 [Homo sapiens] | [17.6](http://blast.ncbi.nlm.nih.gov/Blast.cgi#239746493%23239746493) | 17.6 | 66% | 521 |
| [XP_002343068.1](http://www.ncbi.nlm.nih.gov/entrez/query.fcgi?cmd=Retrieve&db=Protein&list_uids=239744174&dopt=GenPept&RID=T2WV9WH201N&log$=prottop&blast_rank=50) | PREDICTED: hypothetical protein XP_002343068 [Homo sapiens] >ref|XP_002347205.1| PREDICTED: hypothetical protein [Homo sapiens] | [17.6](http://blast.ncbi.nlm.nih.gov/Blast.cgi#239744174%23239744174) | 17.6 | 66% | 521 |
| [XP_002342855.1](http://www.ncbi.nlm.nih.gov/entrez/query.fcgi?cmd=Retrieve&db=Protein&list_uids=239743434&dopt=GenPept&RID=T2WV9WH201N&log$=prottop&blast_rank=51) | PREDICTED: hypothetical protein XP_002342855 [Homo sapiens] >ref|XP_002347005.1| PREDICTED: hypothetical protein XP_002347005 [Homo sapiens] >ref|XP_002346118.1| PREDICTED: hypothetical protein [Homo sapiens] | [17.6](http://blast.ncbi.nlm.nih.gov/Blast.cgi#239743434%23239743434) | 17.6 | 66% | 521 |
| [XP_002342672.1](http://www.ncbi.nlm.nih.gov/entrez/query.fcgi?cmd=Retrieve&db=Protein&list_uids=239742903&dopt=GenPept&RID=T2WV9WH201N&log$=prottop&blast_rank=52) | PREDICTED: hypothetical protein XP_002342672 [Homo sapiens] >ref|XP_002346837.1| PREDICTED: hypothetical protein XP_002346837 [Homo sapiens] >ref|XP_002345955.1| PREDICTED: hypothetical protein XP_002345955 [Homo sapiens] | [17.6](http://blast.ncbi.nlm.nih.gov/Blast.cgi#239742903%23239742903) | 17.6 | 66% | 521 |
| [NP_872394.2](http://www.ncbi.nlm.nih.gov/entrez/query.fcgi?cmd=Retrieve&db=Protein&list_uids=211059431&dopt=GenPept&RID=T2WV9WH201N&log$=prottop&blast_rank=53) | RANBP2-like and GRIP domain containing 4 [Homo sapiens] | [17.6](http://blast.ncbi.nlm.nih.gov/Blast.cgi#211059431%23211059431) | 17.6 | 66% | 521 |
| [XP_001131723.2](http://www.ncbi.nlm.nih.gov/entrez/query.fcgi?cmd=Retrieve&db=Protein&list_uids=169205589&dopt=GenPept&RID=T2WV9WH201N&log$=prottop&blast_rank=54) | PREDICTED: chromosome 12 open reading frame 55 [Homo sapiens] | [17.6](http://blast.ncbi.nlm.nih.gov/Blast.cgi#169205589%23169205589) | 17.6 | 66% | 521 |
| [XP_001718035.1](http://www.ncbi.nlm.nih.gov/entrez/query.fcgi?cmd=Retrieve&db=Protein&list_uids=169204921&dopt=GenPept&RID=T2WV9WH201N&log$=prottop&blast_rank=55) | PREDICTED: chromosome 12 open reading frame 55 [Homo sapiens] | [17.6](http://blast.ncbi.nlm.nih.gov/Blast.cgi#169204921%23169204921) | 17.6 | 66% | 521 |
| [XP_001715142.1](http://www.ncbi.nlm.nih.gov/entrez/query.fcgi?cmd=Retrieve&db=Protein&list_uids=169203876&dopt=GenPept&RID=T2WV9WH201N&log$=prottop&blast_rank=56) | PREDICTED: hypothetical protein [Homo sapiens] | [17.6](http://blast.ncbi.nlm.nih.gov/Blast.cgi#169203876%23169203876) | 17.6 | 66% | 521 |
| [NP_689944.2](http://www.ncbi.nlm.nih.gov/entrez/query.fcgi?cmd=Retrieve&db=Protein&list_uids=148806920&dopt=GenPept&RID=T2WV9WH201N&log$=prottop&blast_rank=57) | BEN domain containing 6 [Homo sapiens] | [17.6](http://blast.ncbi.nlm.nih.gov/Blast.cgi#148806920%23148806920) | 17.6 | 66% | 521 |
| [NP_775891.2](http://www.ncbi.nlm.nih.gov/entrez/query.fcgi?cmd=Retrieve&db=Protein&list_uids=150456449&dopt=GenPept&RID=T2WV9WH201N&log$=prottop&blast_rank=58) | hexosaminidase (glycosyl hydrolase family 20, catalytic domain) containing [Homo sapiens] | [17.6](http://blast.ncbi.nlm.nih.gov/Blast.cgi#150456449%23150456449) | 17.6 | 66% | 521 |
| [NP_079366.3](http://www.ncbi.nlm.nih.gov/entrez/query.fcgi?cmd=Retrieve&db=Protein&list_uids=122114651&dopt=GenPept&RID=T2WV9WH201N&log$=prottop&blast_rank=59) | ubiquitin specific peptidase 36 [Homo sapiens] | [17.6](http://blast.ncbi.nlm.nih.gov/Blast.cgi#122114651%23122114651) | 17.6 | 66% | 521 |
| [NP_001091069.1](http://www.ncbi.nlm.nih.gov/entrez/query.fcgi?cmd=Retrieve&db=Protein&list_uids=147907044&dopt=GenPept&RID=T2WV9WH201N&log$=prottop&blast_rank=60) | transmembrane protein 22 [Homo sapiens] >ref|NP_079522.2| transmembrane protein 22 [Homo sapiens] >ref|NP_001091068.1| transmembrane protein 22 [Homo sapiens] | [17.6](http://blast.ncbi.nlm.nih.gov/Blast.cgi#147907044%23147907044) | 17.6 | 66% | 521 |
| [NP_002901.2](http://www.ncbi.nlm.nih.gov/entrez/query.fcgi?cmd=Retrieve&db=Protein&list_uids=213417820&dopt=GenPept&RID=T2WV9WH201N&log$=prottop&blast_rank=61) | renin binding protein [Homo sapiens] | [17.6](http://blast.ncbi.nlm.nih.gov/Blast.cgi#213417820%23213417820) | 17.6 | 66% | 521 |
| [NP_001070729.1](http://www.ncbi.nlm.nih.gov/entrez/query.fcgi?cmd=Retrieve&db=Protein&list_uids=116256445&dopt=GenPept&RID=T2WV9WH201N&log$=prottop&blast_rank=62) | nuclear receptor co-repressor 2 isoform 2 [Homo sapiens] | [17.6](http://blast.ncbi.nlm.nih.gov/Blast.cgi#116256445%23116256445) | 17.6 | 66% | 521 |
| [NP_006303.3](http://www.ncbi.nlm.nih.gov/entrez/query.fcgi?cmd=Retrieve&db=Protein&list_uids=116256453&dopt=GenPept&RID=T2WV9WH201N&log$=prottop&blast_rank=63) | nuclear receptor co-repressor 2 isoform 1 [Homo sapiens] | [17.6](http://blast.ncbi.nlm.nih.gov/Blast.cgi#116256453%23116256453) | 17.6 | 66% | 521 |
| [NP_001011885.1](http://www.ncbi.nlm.nih.gov/entrez/query.fcgi?cmd=Retrieve&db=Protein&list_uids=59814146&dopt=GenPept&RID=T2WV9WH201N&log$=prottop&blast_rank=64) | BTB (POZ) domain containing 1 isoform 2 [Homo sapiens] | [17.6](http://blast.ncbi.nlm.nih.gov/Blast.cgi#59814146%2359814146) | 17.6 | 100% | 521 |
| [NP_751896.1](http://www.ncbi.nlm.nih.gov/entrez/query.fcgi?cmd=Retrieve&db=Protein&list_uids=27262634&dopt=GenPept&RID=T2WV9WH201N&log$=prottop&blast_rank=65) | nuclear autoantigenic sperm protein isoform 1 [Homo sapiens] | [17.6](http://blast.ncbi.nlm.nih.gov/Blast.cgi#27262634%2327262634) | 17.6 | 66% | 521 |
| [XP_001129716.1](http://www.ncbi.nlm.nih.gov/entrez/query.fcgi?cmd=Retrieve&db=Protein&list_uids=113408665&dopt=GenPept&RID=T2WV9WH201N&log$=prottop&blast_rank=66) | PREDICTED: hypothetical protein [Homo sapiens] >ref|XP_001132865.1| PREDICTED: hypothetical protein [Homo sapiens] >ref|XP_001718851.1| PREDICTED: hypothetical protein [Homo sapiens] | [17.6](http://blast.ncbi.nlm.nih.gov/Blast.cgi#113408665%23113408665) | 17.6 | 66% | 521 |
| [NP_001693.2](http://www.ncbi.nlm.nih.gov/entrez/query.fcgi?cmd=Retrieve&db=Protein&list_uids=111118994&dopt=GenPept&RID=T2WV9WH201N&log$=prottop&blast_rank=67) | brain-specific angiogenesis inhibitor 1 precursor [Homo sapiens] | [17.6](http://blast.ncbi.nlm.nih.gov/Blast.cgi#111118994%23111118994) | 17.6 | 66% | 521 |
| [NP_775931.3](http://www.ncbi.nlm.nih.gov/entrez/query.fcgi?cmd=Retrieve&db=Protein&list_uids=111185957&dopt=GenPept&RID=T2WV9WH201N&log$=prottop&blast_rank=68) | downstream of tyrosine kinase 7 isoform 1 [Homo sapiens] | [17.6](http://blast.ncbi.nlm.nih.gov/Blast.cgi#111185957%23111185957) | 17.6 | 66% | 521 |
| [NP_001025126.2](http://www.ncbi.nlm.nih.gov/entrez/query.fcgi?cmd=Retrieve&db=Protein&list_uids=146149345&dopt=GenPept&RID=T2WV9WH201N&log$=prottop&blast_rank=69) | WD repeat domain 21B [Homo sapiens] | [17.6](http://blast.ncbi.nlm.nih.gov/Blast.cgi#146149345%23146149345) | 17.6 | 66% | 521 |
| [NP_001157935.1](http://www.ncbi.nlm.nih.gov/entrez/query.fcgi?cmd=Retrieve&db=Protein&list_uids=256600210&dopt=GenPept&RID=T2WV9WH201N&log$=prottop&blast_rank=70) | RANBP2-like and GRIP domain containing 8 [Homo sapiens] | [17.6](http://blast.ncbi.nlm.nih.gov/Blast.cgi#256600210%23256600210) | 17.6 | 66% | 521 |
| [NP_001784.2](http://www.ncbi.nlm.nih.gov/entrez/query.fcgi?cmd=Retrieve&db=Protein&list_uids=14589891&dopt=GenPept&RID=T2WV9WH201N&log$=prottop&blast_rank=71) | cadherin 3, type 1 preproprotein [Homo sapiens] | [17.6](http://blast.ncbi.nlm.nih.gov/Blast.cgi#14589891%2314589891) | 17.6 | 66% | 521 |
| [NP_065816.2](http://www.ncbi.nlm.nih.gov/entrez/query.fcgi?cmd=Retrieve&db=Protein&list_uids=82659109&dopt=GenPept&RID=T2WV9WH201N&log$=prottop&blast_rank=72) | retinoblastoma-associated factor 600 [Homo sapiens] | [17.6](http://blast.ncbi.nlm.nih.gov/Blast.cgi#82659109%2382659109) | 17.6 | 66% | 521 |
| [NP_031372.2](http://www.ncbi.nlm.nih.gov/entrez/query.fcgi?cmd=Retrieve&db=Protein&list_uids=33286446&dopt=GenPept&RID=T2WV9WH201N&log$=prottop&blast_rank=73) | opioid growth factor receptor [Homo sapiens] | [17.6](http://blast.ncbi.nlm.nih.gov/Blast.cgi#33286446%2333286446) | 17.6 | 66% | 521 |
| [NP_073581.2](http://www.ncbi.nlm.nih.gov/entrez/query.fcgi?cmd=Retrieve&db=Protein&list_uids=257900512&dopt=GenPept&RID=T2WV9WH201N&log$=prottop&blast_rank=74) | hypothetical protein LOC64755 [Homo sapiens] | [17.6](http://blast.ncbi.nlm.nih.gov/Blast.cgi#257900512%23257900512) | 17.6 | 66% | 521 |
| [NP_001071638.2](http://www.ncbi.nlm.nih.gov/entrez/query.fcgi?cmd=Retrieve&db=Protein&list_uids=262118271&dopt=GenPept&RID=T2WV9WH201N&log$=prottop&blast_rank=75) | RANBP2-like and GRIP domain containing 2 [Homo sapiens] | [17.6](http://blast.ncbi.nlm.nih.gov/Blast.cgi#262118271%23262118271) | 17.6 | 66% | 521 |
| [NP_001137485.1](http://www.ncbi.nlm.nih.gov/entrez/query.fcgi?cmd=Retrieve&db=Protein&list_uids=221307607&dopt=GenPept&RID=T2WV9WH201N&log$=prottop&blast_rank=76) | RANBP2-like and GRIP domain containing 3 [Homo sapiens] | [17.6](http://blast.ncbi.nlm.nih.gov/Blast.cgi#221307607%23221307607) | 17.6 | 66% | 521 |
| [NP_001123380.2](http://www.ncbi.nlm.nih.gov/entrez/query.fcgi?cmd=Retrieve&db=Protein&list_uids=207113133&dopt=GenPept&RID=T2WV9WH201N&log$=prottop&blast_rank=77) | hypothetical protein LOC729085 [Homo sapiens] | [17.6](http://blast.ncbi.nlm.nih.gov/Blast.cgi#207113133%23207113133) | 17.6 | 66% | 521 |
| [NP_001005746.1](http://www.ncbi.nlm.nih.gov/entrez/query.fcgi?cmd=Retrieve&db=Protein&list_uids=54607066&dopt=GenPept&RID=T2WV9WH201N&log$=prottop&blast_rank=78) | calcium channel, voltage-dependent, beta 4 subunit isoform c [Homo sapiens] | [17.6](http://blast.ncbi.nlm.nih.gov/Blast.cgi#54607066%2354607066) | 17.6 | 66% | 521 |
| [NP_001005747.1](http://www.ncbi.nlm.nih.gov/entrez/query.fcgi?cmd=Retrieve&db=Protein&list_uids=54607068&dopt=GenPept&RID=T2WV9WH201N&log$=prottop&blast_rank=79) | calcium channel, voltage-dependent, beta 4 subunit isoform a [Homo sapiens] | [17.6](http://blast.ncbi.nlm.nih.gov/Blast.cgi#54607068%2354607068) | 17.6 | 66% | 521 |
| [NP_056156.2](http://www.ncbi.nlm.nih.gov/entrez/query.fcgi?cmd=Retrieve&db=Protein&list_uids=25121987&dopt=GenPept&RID=T2WV9WH201N&log$=prottop&blast_rank=80) | non-SMC condensin I complex, subunit H [Homo sapiens] | [17.6](http://blast.ncbi.nlm.nih.gov/Blast.cgi#25121987%2325121987) | 17.6 | 66% | 521 |
| [NP_000717.2](http://www.ncbi.nlm.nih.gov/entrez/query.fcgi?cmd=Retrieve&db=Protein&list_uids=54607064&dopt=GenPept&RID=T2WV9WH201N&log$=prottop&blast_rank=81) | calcium channel, voltage-dependent, beta 4 subunit isoform b [Homo sapiens] | [17.6](http://blast.ncbi.nlm.nih.gov/Blast.cgi#54607064%2354607064) | 17.6 | 66% | 521 |
| [NP_006258.3](http://www.ncbi.nlm.nih.gov/entrez/query.fcgi?cmd=Retrieve&db=Protein&list_uids=150418007&dopt=GenPept&RID=T2WV9WH201N&log$=prottop&blast_rank=82) | RAN binding protein 2 [Homo sapiens] | [17.6](http://blast.ncbi.nlm.nih.gov/Blast.cgi#150418007%23150418007) | 17.6 | 66% | 521 |
| [NP_060688.1](http://www.ncbi.nlm.nih.gov/entrez/query.fcgi?cmd=Retrieve&db=Protein&list_uids=41055953&dopt=GenPept&RID=T2WV9WH201N&log$=prottop&blast_rank=83) | ubiquitin thioesterase 40 [Homo sapiens] | [17.6](http://blast.ncbi.nlm.nih.gov/Blast.cgi#41055953%2341055953) | 17.6 | 66% | 521 |
| [NP_060795.1](http://www.ncbi.nlm.nih.gov/entrez/query.fcgi?cmd=Retrieve&db=Protein&list_uids=37039612&dopt=GenPept&RID=T2WV9WH201N&log$=prottop&blast_rank=84) | hypothetical protein LOC203228 isoform a [Homo sapiens] | [17.6](http://blast.ncbi.nlm.nih.gov/Blast.cgi#37039612%2337039612) | 17.6 | 66% | 521 |
| [NP_149095.2](http://www.ncbi.nlm.nih.gov/entrez/query.fcgi?cmd=Retrieve&db=Protein&list_uids=21361863&dopt=GenPept&RID=T2WV9WH201N&log$=prottop&blast_rank=85) | stonin 2 [Homo sapiens] | [17.6](http://blast.ncbi.nlm.nih.gov/Blast.cgi#21361863%2321361863) | 17.6 | 66% | 521 |
| [NP_079514.1](http://www.ncbi.nlm.nih.gov/entrez/query.fcgi?cmd=Retrieve&db=Protein&list_uids=13376848&dopt=GenPept&RID=T2WV9WH201N&log$=prottop&blast_rank=86) | BTB (POZ) domain containing 1 isoform 1 [Homo sapiens] | [17.6](http://blast.ncbi.nlm.nih.gov/Blast.cgi#13376848%2313376848) | 17.6 | 100% | 521 |
| [NP_110448.2](http://www.ncbi.nlm.nih.gov/entrez/query.fcgi?cmd=Retrieve&db=Protein&list_uids=21361944&dopt=GenPept&RID=T2WV9WH201N&log$=prottop&blast_rank=87) | phospholipase A2, group XIIA precursor [Homo sapiens] | [17.6](http://blast.ncbi.nlm.nih.gov/Blast.cgi#21361944%2321361944) | 17.6 | 66% | 521 |
| [NP_000716.2](http://www.ncbi.nlm.nih.gov/entrez/query.fcgi?cmd=Retrieve&db=Protein&list_uids=40316928&dopt=GenPept&RID=T2WV9WH201N&log$=prottop&blast_rank=88) | calcium channel, voltage-dependent, beta 3 subunit [Homo sapiens] | [17.6](http://blast.ncbi.nlm.nih.gov/Blast.cgi#40316928%2340316928) | 17.6 | 66% | 521 |
| [NP_003232.2](http://www.ncbi.nlm.nih.gov/entrez/query.fcgi?cmd=Retrieve&db=Protein&list_uids=156627577&dopt=GenPept&RID=T2WV9WH201N&log$=prottop&blast_rank=89) | transglutaminase 4 (prostate) [Homo sapiens] | [17.6](http://blast.ncbi.nlm.nih.gov/Blast.cgi#156627577%23156627577) | 17.6 | 100% | 521 |
| [XP_002345234.1](http://www.ncbi.nlm.nih.gov/entrez/query.fcgi?cmd=Retrieve&db=Protein&list_uids=239757284&dopt=GenPept&RID=T2WV9WH201N&log$=prottop&blast_rank=90) | PREDICTED: similar to Fc fragment of IgG binding protein [Homo sapiens] | [17.2](http://blast.ncbi.nlm.nih.gov/Blast.cgi#239757284%23239757284) | 17.2 | 66% | 699 |
| [XP_001717595.2](http://www.ncbi.nlm.nih.gov/entrez/query.fcgi?cmd=Retrieve&db=Protein&list_uids=239757282&dopt=GenPept&RID=T2WV9WH201N&log$=prottop&blast_rank=91) | PREDICTED: similar to Fc fragment of IgG binding protein [Homo sapiens] | [17.2](http://blast.ncbi.nlm.nih.gov/Blast.cgi#239757282%23239757282) | 17.2 | 66% | 699 |
| [XP_002345218.1](http://www.ncbi.nlm.nih.gov/entrez/query.fcgi?cmd=Retrieve&db=Protein&list_uids=239757244&dopt=GenPept&RID=T2WV9WH201N&log$=prottop&blast_rank=92) | PREDICTED: hypothetical protein [Homo sapiens] | [17.2](http://blast.ncbi.nlm.nih.gov/Blast.cgi#239757244%23239757244) | 17.2 | 66% | 699 |
| [XP_002345619.1](http://www.ncbi.nlm.nih.gov/entrez/query.fcgi?cmd=Retrieve&db=Protein&list_uids=239753314&dopt=GenPept&RID=T2WV9WH201N&log$=prottop&blast_rank=93) | PREDICTED: similar to RIKEN cDNA C230030N03 [Homo sapiens] | [17.2](http://blast.ncbi.nlm.nih.gov/Blast.cgi#239753314%23239753314) | 31.8 | 66% | 699 |
| [XP_002348012.1](http://www.ncbi.nlm.nih.gov/entrez/query.fcgi?cmd=Retrieve&db=Protein&list_uids=239751787&dopt=GenPept&RID=T2WV9WH201N&log$=prottop&blast_rank=94) | PREDICTED: hypothetical protein XP_002348012 [Homo sapiens] | [17.2](http://blast.ncbi.nlm.nih.gov/Blast.cgi#239751787%23239751787) | 31.8 | 66% | 699 |
| [XP_002348293.1](http://www.ncbi.nlm.nih.gov/entrez/query.fcgi?cmd=Retrieve&db=Protein&list_uids=239747780&dopt=GenPept&RID=T2WV9WH201N&log$=prottop&blast_rank=95) | PREDICTED: hypothetical protein XP_002348293 [Homo sapiens] | [17.2](http://blast.ncbi.nlm.nih.gov/Blast.cgi#239747780%23239747780) | 17.2 | 66% | 699 |
| [XP_002343716.1](http://www.ncbi.nlm.nih.gov/entrez/query.fcgi?cmd=Retrieve&db=Protein&list_uids=239746252&dopt=GenPept&RID=T2WV9WH201N&log$=prottop&blast_rank=96) | PREDICTED: hypothetical protein XP_002343716 [Homo sapiens] >ref|XP_002347984.1| PREDICTED: hypothetical protein [Homo sapiens] | [17.2](http://blast.ncbi.nlm.nih.gov/Blast.cgi#239746252%23239746252) | 17.2 | 66% | 699 |
| [XP_002342306.1](http://www.ncbi.nlm.nih.gov/entrez/query.fcgi?cmd=Retrieve&db=Protein&list_uids=239741723&dopt=GenPept&RID=T2WV9WH201N&log$=prottop&blast_rank=97) | PREDICTED: hypothetical protein XP_002342306 [Homo sapiens] | [17.2](http://blast.ncbi.nlm.nih.gov/Blast.cgi#239741723%23239741723) | 17.2 | 66% | 699 |
| [NP_001153574.1](http://www.ncbi.nlm.nih.gov/entrez/query.fcgi?cmd=Retrieve&db=Protein&list_uids=231569458&dopt=GenPept&RID=T2WV9WH201N&log$=prottop&blast_rank=98) | lactoperoxidase isoform 3 preproprotein [Homo sapiens] | [17.2](http://blast.ncbi.nlm.nih.gov/Blast.cgi#231569458%23231569458) | 17.2 | 66% | 699 |
| [NP_001129671.1](http://www.ncbi.nlm.nih.gov/entrez/query.fcgi?cmd=Retrieve&db=Protein&list_uids=209969686&dopt=GenPept&RID=T2WV9WH201N&log$=prottop&blast_rank=99) | GRAM domain containing 1A isoform 2 [Homo sapiens] | [17.2](http://blast.ncbi.nlm.nih.gov/Blast.cgi#209969686%23209969686) | 17.2 | 66% | 699 |
| [NP_003250.3](http://www.ncbi.nlm.nih.gov/entrez/query.fcgi?cmd=Retrieve&db=Protein&list_uids=189571583&dopt=GenPept&RID=T2WV9WH201N&log$=prottop&blast_rank=100) | intercellular adhesion molecule 5 precursor [Homo sapiens] | [17.2](http://blast.ncbi.nlm.nih.gov/Blast.cgi#189571583%23189571583) | 17.2 | 66% | 699 |

| **Accession** | **Proteins with a match to NERSEAR peptide** | **[Max score](http://blast.ncbi.nlm.nih.gov/Blast.cgi?CMD=Get&ALIGNMENTS=100&ALIGNMENT_VIEW=Pairwise&CDD_SEARCH_STATE=1&DATABASE_SORT=0&DESCRIPTIONS=100&ENTREZ_QUERY=txid9606 %5BORGN%5D&FIRST_QUERY_NUM=0&FORMAT_OBJECT=Alignment&FORMAT_PAGE_TARGET=&FORMAT_TYPE=HTML&GET_SEQUENCE=yes&I_THRESH=&MASK_CHAR=2&MASK_COLOR=1&NEW_DESIGN=on&NEW_VIEW=yes&NUM_OVERVIEW=100&OLD_BLAST=false&PAGE=Proteins&QUERY_INDEX=0&QUERY_NUMBER=0&RESULTS_PAGE_TARGET=&RID=T2X4GGAW016&SHOW_LINKOUT=yes&SHOW_OVERVIEW=yes&STEP_NUMBER=&WORD_SIZE=2&DISPLAY_SORT=1&HSP_SORT=1" \l "sort_mark)** | **[Total score](http://blast.ncbi.nlm.nih.gov/Blast.cgi?CMD=Get&ALIGNMENTS=100&ALIGNMENT_VIEW=Pairwise&CDD_SEARCH_STATE=1&DATABASE_SORT=0&DESCRIPTIONS=100&ENTREZ_QUERY=txid9606 %5BORGN%5D&FIRST_QUERY_NUM=0&FORMAT_OBJECT=Alignment&FORMAT_PAGE_TARGET=&FORMAT_TYPE=HTML&GET_SEQUENCE=yes&I_THRESH=&MASK_CHAR=2&MASK_COLOR=1&NEW_DESIGN=on&NEW_VIEW=yes&NUM_OVERVIEW=100&OLD_BLAST=false&PAGE=Proteins&QUERY_INDEX=0&QUERY_NUMBER=0&RESULTS_PAGE_TARGET=&RID=T2X4GGAW016&SHOW_LINKOUT=yes&SHOW_OVERVIEW=yes&STEP_NUMBER=&WORD_SIZE=2&DISPLAY_SORT=2&HSP_SORT=1" \l "sort_mark)** | **[Query coverage](http://blast.ncbi.nlm.nih.gov/Blast.cgi?CMD=Get&ALIGNMENTS=100&ALIGNMENT_VIEW=Pairwise&CDD_SEARCH_STATE=1&DATABASE_SORT=0&DESCRIPTIONS=100&ENTREZ_QUERY=txid9606 %5BORGN%5D&FIRST_QUERY_NUM=0&FORMAT_OBJECT=Alignment&FORMAT_PAGE_TARGET=&FORMAT_TYPE=HTML&GET_SEQUENCE=yes&I_THRESH=&MASK_CHAR=2&MASK_COLOR=1&NEW_DESIGN=on&NEW_VIEW=yes&NUM_OVERVIEW=100&OLD_BLAST=false&PAGE=Proteins&QUERY_INDEX=0&QUERY_NUMBER=0&RESULTS_PAGE_TARGET=&RID=T2X4GGAW016&SHOW_LINKOUT=yes&SHOW_OVERVIEW=yes&STEP_NUMBER=&WORD_SIZE=2&DISPLAY_SORT=4&HSP_SORT=0" \l "sort_mark)** | **[E value](http://blast.ncbi.nlm.nih.gov/Blast.cgi?CMD=Get&ALIGNMENTS=100&ALIGNMENT_VIEW=Pairwise&CDD_SEARCH_STATE=1&DATABASE_SORT=0&DESCRIPTIONS=100&ENTREZ_QUERY=txid9606 %5BORGN%5D&FIRST_QUERY_NUM=0&FORMAT_OBJECT=Alignment&FORMAT_PAGE_TARGET=&FORMAT_TYPE=HTML&GET_SEQUENCE=yes&I_THRESH=&MASK_CHAR=2&MASK_COLOR=1&NEW_DESIGN=on&NEW_VIEW=yes&NUM_OVERVIEW=100&OLD_BLAST=false&PAGE=Proteins&QUERY_INDEX=0&QUERY_NUMBER=0&RESULTS_PAGE_TARGET=&RID=T2X4GGAW016&SHOW_LINKOUT=yes&SHOW_OVERVIEW=yes&STEP_NUMBER=&WORD_SIZE=2&DISPLAY_SORT=0&HSP_SORT=0" \l "sort_mark)** |
| --- | --- | --- | --- | --- | --- |
| [NP_001078933.1](http://www.ncbi.nlm.nih.gov/entrez/query.fcgi?cmd=Retrieve&db=Protein&list_uids=146231991&dopt=GenPept&RID=T2X4GGAW016&log$=prottop&blast_rank=1) | catenin, delta 1 isoform 3AB [Homo sapiens] | [21.8](http://blast.ncbi.nlm.nih.gov/Blast.cgi#146231991%23146231991) | 21.8 | 85% | 32 |
| [NP_001078937.1](http://www.ncbi.nlm.nih.gov/entrez/query.fcgi?cmd=Retrieve&db=Protein&list_uids=146231948&dopt=GenPept&RID=T2X4GGAW016&log$=prottop&blast_rank=2) | catenin, delta 1 isoform 3A [Homo sapiens] >ref|NP_001078938.1| catenin, delta 1 isoform 3A [Homo sapiens] >ref|NP_001078936.1| catenin, delta 1 isoform 3A [Homo sapiens] | [21.8](http://blast.ncbi.nlm.nih.gov/Blast.cgi#146231948%23146231948) | 21.8 | 85% | 32 |
| [NP_001078929.1](http://www.ncbi.nlm.nih.gov/entrez/query.fcgi?cmd=Retrieve&db=Protein&list_uids=146231938&dopt=GenPept&RID=T2X4GGAW016&log$=prottop&blast_rank=3) | catenin, delta 1 isoform 1A [Homo sapiens] >ref|NP_001078931.1| catenin, delta 1 isoform 1A [Homo sapiens] >ref|NP_001078930.1| catenin, delta 1 isoform 1A [Homo sapiens] | [21.8](http://blast.ncbi.nlm.nih.gov/Blast.cgi#146231938%23146231938) | 21.8 | 85% | 32 |
| [NP_001322.1](http://www.ncbi.nlm.nih.gov/entrez/query.fcgi?cmd=Retrieve&db=Protein&list_uids=10835010&dopt=GenPept&RID=T2X4GGAW016&log$=prottop&blast_rank=4) | catenin, delta 1 isoform 1B [Homo sapiens] | [21.8](http://blast.ncbi.nlm.nih.gov/Blast.cgi#10835010%2310835010) | 21.8 | 85% | 32 |
| [NP_001078934.1](http://www.ncbi.nlm.nih.gov/entrez/query.fcgi?cmd=Retrieve&db=Protein&list_uids=146231946&dopt=GenPept&RID=T2X4GGAW016&log$=prottop&blast_rank=5) | catenin, delta 1 isoform 3B [Homo sapiens] | [21.8](http://blast.ncbi.nlm.nih.gov/Blast.cgi#146231946%23146231946) | 21.8 | 85% | 32 |
| [NP_001078932.1](http://www.ncbi.nlm.nih.gov/entrez/query.fcgi?cmd=Retrieve&db=Protein&list_uids=146231970&dopt=GenPept&RID=T2X4GGAW016&log$=prottop&blast_rank=6) | catenin, delta 1 isoform 3ABC [Homo sapiens] | [21.8](http://blast.ncbi.nlm.nih.gov/Blast.cgi#146231970%23146231970) | 21.8 | 85% | 32 |
| [NP_001078935.1](http://www.ncbi.nlm.nih.gov/entrez/query.fcgi?cmd=Retrieve&db=Protein&list_uids=146231977&dopt=GenPept&RID=T2X4GGAW016&log$=prottop&blast_rank=7) | catenin, delta 1 isoform 3AC [Homo sapiens] | [21.8](http://blast.ncbi.nlm.nih.gov/Blast.cgi#146231977%23146231977) | 21.8 | 85% | 32 |
| [NP_001078928.1](http://www.ncbi.nlm.nih.gov/entrez/query.fcgi?cmd=Retrieve&db=Protein&list_uids=146231962&dopt=GenPept&RID=T2X4GGAW016&log$=prottop&blast_rank=8) | catenin, delta 1 isoform 1AB [Homo sapiens] | [21.8](http://blast.ncbi.nlm.nih.gov/Blast.cgi#146231962%23146231962) | 21.8 | 85% | 32 |
| [NP_001078927.1](http://www.ncbi.nlm.nih.gov/entrez/query.fcgi?cmd=Retrieve&db=Protein&list_uids=146231940&dopt=GenPept&RID=T2X4GGAW016&log$=prottop&blast_rank=9) | catenin, delta 1 isoform 1ABC [Homo sapiens] | [21.8](http://blast.ncbi.nlm.nih.gov/Blast.cgi#146231940%23146231940) | 21.8 | 85% | 32 |
| [NP_689658.1](http://www.ncbi.nlm.nih.gov/entrez/query.fcgi?cmd=Retrieve&db=Protein&list_uids=22748943&dopt=GenPept&RID=T2X4GGAW016&log$=prottop&blast_rank=10) | hypothetical protein LOC145483 [Homo sapiens] | [21.8](http://blast.ncbi.nlm.nih.gov/Blast.cgi#22748943%2322748943) | 21.8 | 85% | 32 |
| [XP_002347559.1](http://www.ncbi.nlm.nih.gov/entrez/query.fcgi?cmd=Retrieve&db=Protein&list_uids=239750788&dopt=GenPept&RID=T2X4GGAW016&log$=prottop&blast_rank=11) | PREDICTED: similar to hCG2040392 [Homo sapiens] | [19.3](http://blast.ncbi.nlm.nih.gov/Blast.cgi#239750788%23239750788) | 19.3 | 85% | 188 |
| [XP_002342508.1](http://www.ncbi.nlm.nih.gov/entrez/query.fcgi?cmd=Retrieve&db=Protein&list_uids=239742388&dopt=GenPept&RID=T2X4GGAW016&log$=prottop&blast_rank=12) | PREDICTED: hypothetical protein XP_002342508 [Homo sapiens] >ref|XP_002346681.1| PREDICTED: similar to TRIMCyp [Homo sapiens] >ref|XP_002345812.1| PREDICTED: similar to TRIMCyp [Homo sapiens] | [19.3](http://blast.ncbi.nlm.nih.gov/Blast.cgi#239742388%23239742388) | 19.3 | 85% | 188 |
| [XP_002344164.1](http://www.ncbi.nlm.nih.gov/entrez/query.fcgi?cmd=Retrieve&db=Protein&list_uids=239745658&dopt=GenPept&RID=T2X4GGAW016&log$=prottop&blast_rank=13) | PREDICTED: hypothetical protein XP_002344164 [Homo sapiens] | [19.3](http://blast.ncbi.nlm.nih.gov/Blast.cgi#239745658%23239745658) | 33.1 | 85% | 188 |
| [NP_056052.2](http://www.ncbi.nlm.nih.gov/entrez/query.fcgi?cmd=Retrieve&db=Protein&list_uids=197245440&dopt=GenPept&RID=T2X4GGAW016&log$=prottop&blast_rank=14) | hypothetical protein LOC23285 [Homo sapiens] | [19.3](http://blast.ncbi.nlm.nih.gov/Blast.cgi#197245440%23197245440) | 19.3 | 85% | 188 |
| [NP_055809.2](http://www.ncbi.nlm.nih.gov/entrez/query.fcgi?cmd=Retrieve&db=Protein&list_uids=190684671&dopt=GenPept&RID=T2X4GGAW016&log$=prottop&blast_rank=15) | mitogen-activated protein kinase binding protein 1 isoform a [Homo sapiens] | [19.3](http://blast.ncbi.nlm.nih.gov/Blast.cgi#190684671%23190684671) | 32.7 | 85% | 188 |
| [NP_001078880.1](http://www.ncbi.nlm.nih.gov/entrez/query.fcgi?cmd=Retrieve&db=Protein&list_uids=146134341&dopt=GenPept&RID=T2X4GGAW016&log$=prottop&blast_rank=16) | hypothetical protein LOC133686 isoform 1 [Homo sapiens] | [19.3](http://blast.ncbi.nlm.nih.gov/Blast.cgi#146134341%23146134341) | 35.2 | 85% | 188 |
| [NP_005985.3](http://www.ncbi.nlm.nih.gov/entrez/query.fcgi?cmd=Retrieve&db=Protein&list_uids=197333868&dopt=GenPept&RID=T2X4GGAW016&log$=prottop&blast_rank=17) | T-box 2 [Homo sapiens] | [19.3](http://blast.ncbi.nlm.nih.gov/Blast.cgi#197333868%23197333868) | 19.3 | 85% | 188 |
| [NP_001122080.1](http://www.ncbi.nlm.nih.gov/entrez/query.fcgi?cmd=Retrieve&db=Protein&list_uids=190684677&dopt=GenPept&RID=T2X4GGAW016&log$=prottop&blast_rank=18) | mitogen-activated protein kinase binding protein 1 isoform b [Homo sapiens] | [19.3](http://blast.ncbi.nlm.nih.gov/Blast.cgi#190684677%23190684677) | 32.7 | 85% | 188 |
| [NP_705833.2](http://www.ncbi.nlm.nih.gov/entrez/query.fcgi?cmd=Retrieve&db=Protein&list_uids=90403579&dopt=GenPept&RID=T2X4GGAW016&log$=prottop&blast_rank=19) | beta-gamma crystallin domain containing 3 [Homo sapiens] | [19.3](http://blast.ncbi.nlm.nih.gov/Blast.cgi#90403579%2390403579) | 19.3 | 85% | 188 |
| [NP_001264.2](http://www.ncbi.nlm.nih.gov/entrez/query.fcgi?cmd=Retrieve&db=Protein&list_uids=51599156&dopt=GenPept&RID=T2X4GGAW016&log$=prottop&blast_rank=20) | chromodomain helicase DNA binding protein 4 [Homo sapiens] | [19.3](http://blast.ncbi.nlm.nih.gov/Blast.cgi#51599156%2351599156) | 19.3 | 85% | 188 |
| [NP_071348.3](http://www.ncbi.nlm.nih.gov/entrez/query.fcgi?cmd=Retrieve&db=Protein&list_uids=38348727&dopt=GenPept&RID=T2X4GGAW016&log$=prottop&blast_rank=21) | thyroid adenoma associated [Homo sapiens] >ref|NP_001077422.1| thyroid adenoma associated [Homo sapiens] | [19.3](http://blast.ncbi.nlm.nih.gov/Blast.cgi#38348727%2338348727) | 19.3 | 71% | 188 |
| [NP_955383.1](http://www.ncbi.nlm.nih.gov/entrez/query.fcgi?cmd=Retrieve&db=Protein&list_uids=40786420&dopt=GenPept&RID=T2X4GGAW016&log$=prottop&blast_rank=22) | immunoglobulin-like domain containing receptor 2 [Homo sapiens] | [19.3](http://blast.ncbi.nlm.nih.gov/Blast.cgi#40786420%2340786420) | 19.3 | 85% | 188 |
| [NP_740721.1](http://www.ncbi.nlm.nih.gov/entrez/query.fcgi?cmd=Retrieve&db=Protein&list_uids=25777647&dopt=GenPept&RID=T2X4GGAW016&log$=prottop&blast_rank=23) | small conductance calcium-activated potassium channel protein 2 isoform b [Homo sapiens] | [19.3](http://blast.ncbi.nlm.nih.gov/Blast.cgi#25777647%2325777647) | 19.3 | 71% | 188 |
| [NP_002516.2](http://www.ncbi.nlm.nih.gov/entrez/query.fcgi?cmd=Retrieve&db=Protein&list_uids=156071450&dopt=GenPept&RID=T2X4GGAW016&log$=prottop&blast_rank=24) | nardilysin isoform a [Homo sapiens] | [19.3](http://blast.ncbi.nlm.nih.gov/Blast.cgi#156071450%23156071450) | 19.3 | 85% | 188 |
| [NP_067627.2](http://www.ncbi.nlm.nih.gov/entrez/query.fcgi?cmd=Retrieve&db=Protein&list_uids=25777645&dopt=GenPept&RID=T2X4GGAW016&log$=prottop&blast_rank=25) | small conductance calcium-activated potassium channel protein 2 isoform a [Homo sapiens] | [19.3](http://blast.ncbi.nlm.nih.gov/Blast.cgi#25777645%2325777645) | 19.3 | 71% | 188 |
| [NP_057700.3](http://www.ncbi.nlm.nih.gov/entrez/query.fcgi?cmd=Retrieve&db=Protein&list_uids=148839372&dopt=GenPept&RID=T2X4GGAW016&log$=prottop&blast_rank=26) | thioredoxin domain containing 3 [Homo sapiens] | [19.3](http://blast.ncbi.nlm.nih.gov/Blast.cgi#148839372%23148839372) | 19.3 | 71% | 188 |
| [NP_001095132.1](http://www.ncbi.nlm.nih.gov/entrez/query.fcgi?cmd=Retrieve&db=Protein&list_uids=156071452&dopt=GenPept&RID=T2X4GGAW016&log$=prottop&blast_rank=27) | nardilysin isoform b [Homo sapiens] | [19.3](http://blast.ncbi.nlm.nih.gov/Blast.cgi#156071452%23156071452) | 19.3 | 85% | 188 |
| [NP_694558.1](http://www.ncbi.nlm.nih.gov/entrez/query.fcgi?cmd=Retrieve&db=Protein&list_uids=23308515&dopt=GenPept&RID=T2X4GGAW016&log$=prottop&blast_rank=28) | hypothetical protein LOC133686 isoform 2 [Homo sapiens] | [19.3](http://blast.ncbi.nlm.nih.gov/Blast.cgi#23308515%2323308515) | 35.2 | 85% | 188 |
| [NP_001993.2](http://www.ncbi.nlm.nih.gov/entrez/query.fcgi?cmd=Retrieve&db=Protein&list_uids=20149533&dopt=GenPept&RID=T2X4GGAW016&log$=prottop&blast_rank=29) | Fc fragment of IgE, low affinity II, receptor for (CD23A) [Homo sapiens] | [19.3](http://blast.ncbi.nlm.nih.gov/Blast.cgi#20149533%2320149533) | 19.3 | 85% | 188 |
| [NP_001177.1](http://www.ncbi.nlm.nih.gov/entrez/query.fcgi?cmd=Retrieve&db=Protein&list_uids=4502353&dopt=GenPept&RID=T2X4GGAW016&log$=prottop&blast_rank=30) | BTB and CNC homology 1 transcription factor isoform a [Homo sapiens] >ref|NP_996749.1| BTB and CNC homology 1 transcription factor isoform a [Homo sapiens] | [19.3](http://blast.ncbi.nlm.nih.gov/Blast.cgi#4502353%234502353) | 19.3 | 71% | 188 |
| [NP_115752.1](http://www.ncbi.nlm.nih.gov/entrez/query.fcgi?cmd=Retrieve&db=Protein&list_uids=14150201&dopt=GenPept&RID=T2X4GGAW016&log$=prottop&blast_rank=31) | transmembrane protein 101 [Homo sapiens] | [19.3](http://blast.ncbi.nlm.nih.gov/Blast.cgi#14150201%2314150201) | 19.3 | 85% | 188 |
| [NP_057440.2](http://www.ncbi.nlm.nih.gov/entrez/query.fcgi?cmd=Retrieve&db=Protein&list_uids=117938307&dopt=GenPept&RID=T2X4GGAW016&log$=prottop&blast_rank=32) | doublecortin domain containing 2 [Homo sapiens] | [18.9](http://blast.ncbi.nlm.nih.gov/Blast.cgi#117938307%23117938307) | 18.9 | 85% | 252 |
| [NP_055780.2](http://www.ncbi.nlm.nih.gov/entrez/query.fcgi?cmd=Retrieve&db=Protein&list_uids=45359873&dopt=GenPept&RID=T2X4GGAW016&log$=prottop&blast_rank=33) | OGT(O-Glc-NAc transferase)-interacting protein 106 KDa isoform 2 [Homo sapiens] | [18.9](http://blast.ncbi.nlm.nih.gov/Blast.cgi#45359873%2345359873) | 33.9 | 85% | 252 |
| [NP_005680.1](http://www.ncbi.nlm.nih.gov/entrez/query.fcgi?cmd=Retrieve&db=Protein&list_uids=9955963&dopt=GenPept&RID=T2X4GGAW016&log$=prottop&blast_rank=34) | ATP-binding cassette, sub-family B, member 6 [Homo sapiens] | [18.9](http://blast.ncbi.nlm.nih.gov/Blast.cgi#9955963%239955963) | 18.9 | 85% | 252 |
| [NP_001159693.1](http://www.ncbi.nlm.nih.gov/entrez/query.fcgi?cmd=Retrieve&db=Protein&list_uids=261278315&dopt=GenPept&RID=T2X4GGAW016&log$=prottop&blast_rank=35) | UTP14, U3 small nucleolar ribonucleoprotein, homolog A isoform 2 [Homo sapiens] | [18.5](http://blast.ncbi.nlm.nih.gov/Blast.cgi#261278315%23261278315) | 18.5 | 71% | 338 |
| [XP_002344832.1](http://www.ncbi.nlm.nih.gov/entrez/query.fcgi?cmd=Retrieve&db=Protein&list_uids=239756174&dopt=GenPept&RID=T2X4GGAW016&log$=prottop&blast_rank=36) | PREDICTED: hypothetical protein [Homo sapiens] | [18.5](http://blast.ncbi.nlm.nih.gov/Blast.cgi#239756174%23239756174) | 60.2 | 85% | 338 |
| [XP_001718394.2](http://www.ncbi.nlm.nih.gov/entrez/query.fcgi?cmd=Retrieve&db=Protein&list_uids=239744982&dopt=GenPept&RID=T2X4GGAW016&log$=prottop&blast_rank=37) | PREDICTED: hypothetical protein [Homo sapiens] >ref|XP_001716267.2| PREDICTED: hypothetical protein [Homo sapiens] | [18.5](http://blast.ncbi.nlm.nih.gov/Blast.cgi#239744982%23239744982) | 60.2 | 85% | 338 |
| [XP_002343084.1](http://www.ncbi.nlm.nih.gov/entrez/query.fcgi?cmd=Retrieve&db=Protein&list_uids=239744252&dopt=GenPept&RID=T2X4GGAW016&log$=prottop&blast_rank=38) | PREDICTED: hypothetical protein XP_002343084 [Homo sapiens] | [18.5](http://blast.ncbi.nlm.nih.gov/Blast.cgi#239744252%23239744252) | 32.2 | 85% | 338 |
| [XP_002344291.1](http://www.ncbi.nlm.nih.gov/entrez/query.fcgi?cmd=Retrieve&db=Protein&list_uids=239740976&dopt=GenPept&RID=T2X4GGAW016&log$=prottop&blast_rank=39) | PREDICTED: hypothetical protein XP_002344291 [Homo sapiens] >ref|XP_002342631.1| PREDICTED: hypothetical protein XP_002342631 [Homo sapiens] | [18.5](http://blast.ncbi.nlm.nih.gov/Blast.cgi#239740976%23239740976) | 32.2 | 85% | 338 |
| [XP_002344357.1](http://www.ncbi.nlm.nih.gov/entrez/query.fcgi?cmd=Retrieve&db=Protein&list_uids=239508694&dopt=GenPept&RID=T2X4GGAW016&log$=prottop&blast_rank=40) | PREDICTED: hypothetical protein [Homo sapiens] | [18.5](http://blast.ncbi.nlm.nih.gov/Blast.cgi#239508694%23239508694) | 18.5 | 71% | 338 |
| [NP_001129107.1](http://www.ncbi.nlm.nih.gov/entrez/query.fcgi?cmd=Retrieve&db=Protein&list_uids=208431771&dopt=GenPept&RID=T2X4GGAW016&log$=prottop&blast_rank=41) | basophilic leukemia expressed protein BLES03 isoform 1 [Homo sapiens] | [18.5](http://blast.ncbi.nlm.nih.gov/Blast.cgi#208431771%23208431771) | 33.1 | 85% | 338 |
| [NP_113638.2](http://www.ncbi.nlm.nih.gov/entrez/query.fcgi?cmd=Retrieve&db=Protein&list_uids=208431769&dopt=GenPept&RID=T2X4GGAW016&log$=prottop&blast_rank=42) | basophilic leukemia expressed protein BLES03 isoform 2 [Homo sapiens] | [18.5](http://blast.ncbi.nlm.nih.gov/Blast.cgi#208431769%23208431769) | 33.1 | 85% | 338 |
| [NP_443161.1](http://www.ncbi.nlm.nih.gov/entrez/query.fcgi?cmd=Retrieve&db=Protein&list_uids=190341068&dopt=GenPept&RID=T2X4GGAW016&log$=prottop&blast_rank=43) | forkhead-associated (FHA) phosphopeptide binding domain 1 [Homo sapiens] | [18.5](http://blast.ncbi.nlm.nih.gov/Blast.cgi#190341068%23190341068) | 18.5 | 85% | 338 |
| [NP_001108410.1](http://www.ncbi.nlm.nih.gov/entrez/query.fcgi?cmd=Retrieve&db=Protein&list_uids=169234951&dopt=GenPept&RID=T2X4GGAW016&log$=prottop&blast_rank=44) | coiled-coil domain containing 17 [Homo sapiens] | [18.5](http://blast.ncbi.nlm.nih.gov/Blast.cgi#169234951%23169234951) | 18.5 | 71% | 338 |
| [XP_001715277.1](http://www.ncbi.nlm.nih.gov/entrez/query.fcgi?cmd=Retrieve&db=Protein&list_uids=169171483&dopt=GenPept&RID=T2X4GGAW016&log$=prottop&blast_rank=45) | PREDICTED: hypothetical protein [Homo sapiens] | [18.5](http://blast.ncbi.nlm.nih.gov/Blast.cgi#169171483%23169171483) | 18.5 | 71% | 338 |
| [XP_001717247.1](http://www.ncbi.nlm.nih.gov/entrez/query.fcgi?cmd=Retrieve&db=Protein&list_uids=169167057&dopt=GenPept&RID=T2X4GGAW016&log$=prottop&blast_rank=46) | PREDICTED: hypothetical protein [Homo sapiens] | [18.5](http://blast.ncbi.nlm.nih.gov/Blast.cgi#169167057%23169167057) | 18.5 | 71% | 338 |
| [XP_001717222.1](http://www.ncbi.nlm.nih.gov/entrez/query.fcgi?cmd=Retrieve&db=Protein&list_uids=169166294&dopt=GenPept&RID=T2X4GGAW016&log$=prottop&blast_rank=47) | PREDICTED: hypothetical protein [Homo sapiens] | [18.5](http://blast.ncbi.nlm.nih.gov/Blast.cgi#169166294%23169166294) | 18.5 | 71% | 338 |
| [NP_001028774.2](http://www.ncbi.nlm.nih.gov/entrez/query.fcgi?cmd=Retrieve&db=Protein&list_uids=140161498&dopt=GenPept&RID=T2X4GGAW016&log$=prottop&blast_rank=48) | cardiac zipper protein isoform a [Homo sapiens] | [18.5](http://blast.ncbi.nlm.nih.gov/Blast.cgi#140161498%23140161498) | 18.5 | 71% | 338 |
| [NP_001004310.2](http://www.ncbi.nlm.nih.gov/entrez/query.fcgi?cmd=Retrieve&db=Protein&list_uids=157057547&dopt=GenPept&RID=T2X4GGAW016&log$=prottop&blast_rank=49) | Fc receptor-like 6 precursor [Homo sapiens] | [18.5](http://blast.ncbi.nlm.nih.gov/Blast.cgi#157057547%23157057547) | 34.4 | 85% | 338 |
| [NP_002462.2](http://www.ncbi.nlm.nih.gov/entrez/query.fcgi?cmd=Retrieve&db=Protein&list_uids=156104908&dopt=GenPept&RID=T2X4GGAW016&log$=prottop&blast_rank=50) | myosin heavy chain 6 [Homo sapiens] | [18.5](http://blast.ncbi.nlm.nih.gov/Blast.cgi#156104908%23156104908) | 46.0 | 85% | 338 |
| [NP_001124295.1](http://www.ncbi.nlm.nih.gov/entrez/query.fcgi?cmd=Retrieve&db=Protein&list_uids=195927037&dopt=GenPept&RID=T2X4GGAW016&log$=prottop&blast_rank=51) | DNA (cytosine-5-)-methyltransferase 1 isoform a [Homo sapiens] | [18.5](http://blast.ncbi.nlm.nih.gov/Blast.cgi#195927037%23195927037) | 18.5 | 71% | 338 |
| [NP_001073867.1](http://www.ncbi.nlm.nih.gov/entrez/query.fcgi?cmd=Retrieve&db=Protein&list_uids=122937211&dopt=GenPept&RID=T2X4GGAW016&log$=prottop&blast_rank=52) | KIAA0368 protein [Homo sapiens] | [18.5](http://blast.ncbi.nlm.nih.gov/Blast.cgi#122937211%23122937211) | 18.5 | 71% | 338 |
| [XP_001129094.1](http://www.ncbi.nlm.nih.gov/entrez/query.fcgi?cmd=Retrieve&db=Protein&list_uids=113416171&dopt=GenPept&RID=T2X4GGAW016&log$=prottop&blast_rank=53) | PREDICTED: hypothetical protein [Homo sapiens] | [18.5](http://blast.ncbi.nlm.nih.gov/Blast.cgi#113416171%23113416171) | 18.5 | 71% | 338 |
| [NP_003793.2](http://www.ncbi.nlm.nih.gov/entrez/query.fcgi?cmd=Retrieve&db=Protein&list_uids=110624781&dopt=GenPept&RID=T2X4GGAW016&log$=prottop&blast_rank=54) | myosin, heavy polypeptide 13, skeletal muscle [Homo sapiens] | [18.5](http://blast.ncbi.nlm.nih.gov/Blast.cgi#110624781%23110624781) | 31.4 | 85% | 338 |
| [NP_001139294.1](http://www.ncbi.nlm.nih.gov/entrez/query.fcgi?cmd=Retrieve&db=Protein&list_uids=224922812&dopt=GenPept&RID=T2X4GGAW016&log$=prottop&blast_rank=55) | tropomyosin 2 (beta) isoform 3 [Homo sapiens] | [18.5](http://blast.ncbi.nlm.nih.gov/Blast.cgi#224922812%23224922812) | 18.5 | 85% | 338 |
| [NP_620604.2](http://www.ncbi.nlm.nih.gov/entrez/query.fcgi?cmd=Retrieve&db=Protein&list_uids=164419749&dopt=GenPept&RID=T2X4GGAW016&log$=prottop&blast_rank=56) | SWI/SNF-related matrix-associated actin-dependent regulator of chromatin a1 isoform b [Homo sapiens] | [18.5](http://blast.ncbi.nlm.nih.gov/Blast.cgi#164419749%23164419749) | 32.7 | 85% | 338 |
| [NP_085139.2](http://www.ncbi.nlm.nih.gov/entrez/query.fcgi?cmd=Retrieve&db=Protein&list_uids=71043966&dopt=GenPept&RID=T2X4GGAW016&log$=prottop&blast_rank=57) | endonuclease/exonuclease/phosphatase family domain containing 1 [Homo sapiens] | [18.5](http://blast.ncbi.nlm.nih.gov/Blast.cgi#71043966%2371043966) | 18.5 | 71% | 338 |
| [NP_620712.2](http://www.ncbi.nlm.nih.gov/entrez/query.fcgi?cmd=Retrieve&db=Protein&list_uids=31712020&dopt=GenPept&RID=T2X4GGAW016&log$=prottop&blast_rank=58) | testis and spermatogenesis cell apoptosis related protein 1 [Homo sapiens] | [18.5](http://blast.ncbi.nlm.nih.gov/Blast.cgi#31712020%2331712020) | 18.5 | 71% | 338 |
| [NP_663719.1](http://www.ncbi.nlm.nih.gov/entrez/query.fcgi?cmd=Retrieve&db=Protein&list_uids=22035604&dopt=GenPept&RID=T2X4GGAW016&log$=prottop&blast_rank=59) | mitogen-activated protein kinase kinase kinase kinase 4 isoform 2 [Homo sapiens] | [18.5](http://blast.ncbi.nlm.nih.gov/Blast.cgi#22035604%2322035604) | 18.5 | 71% | 338 |
| [NP_004825.2](http://www.ncbi.nlm.nih.gov/entrez/query.fcgi?cmd=Retrieve&db=Protein&list_uids=22035602&dopt=GenPept&RID=T2X4GGAW016&log$=prottop&blast_rank=60) | mitogen-activated protein kinase kinase kinase kinase 4 isoform 1 [Homo sapiens] | [18.5](http://blast.ncbi.nlm.nih.gov/Blast.cgi#22035602%2322035602) | 18.5 | 71% | 338 |
| [NP_003060.2](http://www.ncbi.nlm.nih.gov/entrez/query.fcgi?cmd=Retrieve&db=Protein&list_uids=21071044&dopt=GenPept&RID=T2X4GGAW016&log$=prottop&blast_rank=61) | SWI/SNF-related matrix-associated actin-dependent regulator of chromatin a1 isoform a [Homo sapiens] | [18.5](http://blast.ncbi.nlm.nih.gov/Blast.cgi#21071044%2321071044) | 18.5 | 85% | 338 |
| [NP_663720.1](http://www.ncbi.nlm.nih.gov/entrez/query.fcgi?cmd=Retrieve&db=Protein&list_uids=22035606&dopt=GenPept&RID=T2X4GGAW016&log$=prottop&blast_rank=62) | mitogen-activated protein kinase kinase kinase kinase 4 isoform 3 [Homo sapiens] | [18.5](http://blast.ncbi.nlm.nih.gov/Blast.cgi#22035606%2322035606) | 18.5 | 71% | 338 |
| [NP_001155036.1](http://www.ncbi.nlm.nih.gov/entrez/query.fcgi?cmd=Retrieve&db=Protein&list_uids=239735588&dopt=GenPept&RID=T2X4GGAW016&log$=prottop&blast_rank=63) | TRAF2 and NCK interacting kinase isoform 6 [Homo sapiens] | [18.5](http://blast.ncbi.nlm.nih.gov/Blast.cgi#239735588%23239735588) | 18.5 | 71% | 338 |
| [NP_001155037.1](http://www.ncbi.nlm.nih.gov/entrez/query.fcgi?cmd=Retrieve&db=Protein&list_uids=239735590&dopt=GenPept&RID=T2X4GGAW016&log$=prottop&blast_rank=64) | TRAF2 and NCK interacting kinase isoform 7 [Homo sapiens] | [18.5](http://blast.ncbi.nlm.nih.gov/Blast.cgi#239735590%23239735590) | 18.5 | 71% | 338 |
| [NP_001155038.1](http://www.ncbi.nlm.nih.gov/entrez/query.fcgi?cmd=Retrieve&db=Protein&list_uids=239735592&dopt=GenPept&RID=T2X4GGAW016&log$=prottop&blast_rank=65) | TRAF2 and NCK interacting kinase isoform 8 [Homo sapiens] | [18.5](http://blast.ncbi.nlm.nih.gov/Blast.cgi#239735592%23239735592) | 18.5 | 71% | 338 |
| [NP_001155032.1](http://www.ncbi.nlm.nih.gov/entrez/query.fcgi?cmd=Retrieve&db=Protein&list_uids=239735580&dopt=GenPept&RID=T2X4GGAW016&log$=prottop&blast_rank=66) | TRAF2 and NCK interacting kinase isoform 2 [Homo sapiens] | [18.5](http://blast.ncbi.nlm.nih.gov/Blast.cgi#239735580%23239735580) | 18.5 | 71% | 338 |
| [NP_001155033.1](http://www.ncbi.nlm.nih.gov/entrez/query.fcgi?cmd=Retrieve&db=Protein&list_uids=239735582&dopt=GenPept&RID=T2X4GGAW016&log$=prottop&blast_rank=67) | TRAF2 and NCK interacting kinase isoform 3 [Homo sapiens] | [18.5](http://blast.ncbi.nlm.nih.gov/Blast.cgi#239735582%23239735582) | 18.5 | 71% | 338 |
| [NP_001155034.1](http://www.ncbi.nlm.nih.gov/entrez/query.fcgi?cmd=Retrieve&db=Protein&list_uids=239735584&dopt=GenPept&RID=T2X4GGAW016&log$=prottop&blast_rank=68) | TRAF2 and NCK interacting kinase isoform 4 [Homo sapiens] | [18.5](http://blast.ncbi.nlm.nih.gov/Blast.cgi#239735584%23239735584) | 18.5 | 71% | 338 |
| [NP_001155035.1](http://www.ncbi.nlm.nih.gov/entrez/query.fcgi?cmd=Retrieve&db=Protein&list_uids=239735586&dopt=GenPept&RID=T2X4GGAW016&log$=prottop&blast_rank=69) | TRAF2 and NCK interacting kinase isoform 5 [Homo sapiens] | [18.5](http://blast.ncbi.nlm.nih.gov/Blast.cgi#239735586%23239735586) | 18.5 | 71% | 338 |
| [NP_003592.2](http://www.ncbi.nlm.nih.gov/entrez/query.fcgi?cmd=Retrieve&db=Protein&list_uids=21071058&dopt=GenPept&RID=T2X4GGAW016&log$=prottop&blast_rank=70) | SWI/SNF-related matrix-associated actin-dependent regulator of chromatin a5 [Homo sapiens] | [18.5](http://blast.ncbi.nlm.nih.gov/Blast.cgi#21071058%2321071058) | 18.5 | 85% | 338 |
| [NP_570613.2](http://www.ncbi.nlm.nih.gov/entrez/query.fcgi?cmd=Retrieve&db=Protein&list_uids=62865651&dopt=GenPept&RID=T2X4GGAW016&log$=prottop&blast_rank=71) | regulator of G-protein signalling 3 isoform 1 [Homo sapiens] | [18.5](http://blast.ncbi.nlm.nih.gov/Blast.cgi#62865651%2362865651) | 18.5 | 71% | 338 |
| [NP_652759.3](http://www.ncbi.nlm.nih.gov/entrez/query.fcgi?cmd=Retrieve&db=Protein&list_uids=114155127&dopt=GenPept&RID=T2X4GGAW016&log$=prottop&blast_rank=72) | regulator of G-protein signalling 3 isoform 6 [Homo sapiens] | [18.5](http://blast.ncbi.nlm.nih.gov/Blast.cgi#114155127%23114155127) | 18.5 | 71% | 338 |
| [NP_998839.1](http://www.ncbi.nlm.nih.gov/entrez/query.fcgi?cmd=Retrieve&db=Protein&list_uids=47519616&dopt=GenPept&RID=T2X4GGAW016&log$=prottop&blast_rank=73) | tropomyosin 2 (beta) isoform 2 [Homo sapiens] | [18.5](http://blast.ncbi.nlm.nih.gov/Blast.cgi#47519616%2347519616) | 18.5 | 85% | 338 |
| [NP_003280.2](http://www.ncbi.nlm.nih.gov/entrez/query.fcgi?cmd=Retrieve&db=Protein&list_uids=42476296&dopt=GenPept&RID=T2X4GGAW016&log$=prottop&blast_rank=74) | tropomyosin 2 (beta) isoform 1 [Homo sapiens] | [18.5](http://blast.ncbi.nlm.nih.gov/Blast.cgi#42476296%2342476296) | 18.5 | 85% | 338 |
| [NP_000248.2](http://www.ncbi.nlm.nih.gov/entrez/query.fcgi?cmd=Retrieve&db=Protein&list_uids=115496169&dopt=GenPept&RID=T2X4GGAW016&log$=prottop&blast_rank=75) | myosin, heavy chain 7, cardiac muscle, beta [Homo sapiens] | [18.5](http://blast.ncbi.nlm.nih.gov/Blast.cgi#115496169%23115496169) | 46.0 | 85% | 338 |
| [NP_085124.2](http://www.ncbi.nlm.nih.gov/entrez/query.fcgi?cmd=Retrieve&db=Protein&list_uids=29294649&dopt=GenPept&RID=T2X4GGAW016&log$=prottop&blast_rank=76) | dicer1 [Homo sapiens] >ref|NP_803187.1| dicer1 [Homo sapiens] | [18.5](http://blast.ncbi.nlm.nih.gov/Blast.cgi#29294649%2329294649) | 18.5 | 85% | 338 |
| [NP_067677.4](http://www.ncbi.nlm.nih.gov/entrez/query.fcgi?cmd=Retrieve&db=Protein&list_uids=51871374&dopt=GenPept&RID=T2X4GGAW016&log$=prottop&blast_rank=77) | UTP14, U3 small nucleolar ribonucleoprotein, homolog C [Homo sapiens] | [18.5](http://blast.ncbi.nlm.nih.gov/Blast.cgi#51871374%2351871374) | 18.5 | 71% | 338 |
| [NP_061873.2](http://www.ncbi.nlm.nih.gov/entrez/query.fcgi?cmd=Retrieve&db=Protein&list_uids=21361617&dopt=GenPept&RID=T2X4GGAW016&log$=prottop&blast_rank=78) | hypothetical protein LOC54463 isoform 2 [Homo sapiens] | [18.5](http://blast.ncbi.nlm.nih.gov/Blast.cgi#21361617%2321361617) | 18.5 | 71% | 338 |
| [NP_006640.2](http://www.ncbi.nlm.nih.gov/entrez/query.fcgi?cmd=Retrieve&db=Protein&list_uids=21361348&dopt=GenPept&RID=T2X4GGAW016&log$=prottop&blast_rank=79) | UTP14, U3 small nucleolar ribonucleoprotein, homolog A isoform 1 [Homo sapiens] | [18.5](http://blast.ncbi.nlm.nih.gov/Blast.cgi#21361348%2321361348) | 18.5 | 71% | 338 |
| [NP_001030022.1](http://www.ncbi.nlm.nih.gov/entrez/query.fcgi?cmd=Retrieve&db=Protein&list_uids=77917617&dopt=GenPept&RID=T2X4GGAW016&log$=prottop&blast_rank=80) | hypothetical protein LOC54463 isoform 1 [Homo sapiens] | [18.5](http://blast.ncbi.nlm.nih.gov/Blast.cgi#77917617%2377917617) | 18.5 | 71% | 338 |
| [NP_001370.1](http://www.ncbi.nlm.nih.gov/entrez/query.fcgi?cmd=Retrieve&db=Protein&list_uids=4503351&dopt=GenPept&RID=T2X4GGAW016&log$=prottop&blast_rank=81) | DNA (cytosine-5-)-methyltransferase 1 isoform b [Homo sapiens] | [18.5](http://blast.ncbi.nlm.nih.gov/Blast.cgi#4503351%234503351) | 18.5 | 71% | 338 |
| [NP_066929.1](http://www.ncbi.nlm.nih.gov/entrez/query.fcgi?cmd=Retrieve&db=Protein&list_uids=10864075&dopt=GenPept&RID=T2X4GGAW016&log$=prottop&blast_rank=82) | regulator of G-protein signalling 3 isoform 2 [Homo sapiens] | [18.5](http://blast.ncbi.nlm.nih.gov/Blast.cgi#10864075%2310864075) | 18.5 | 71% | 338 |
| [NP_055843.1](http://www.ncbi.nlm.nih.gov/entrez/query.fcgi?cmd=Retrieve&db=Protein&list_uids=55741807&dopt=GenPept&RID=T2X4GGAW016&log$=prottop&blast_rank=83) | TRAF2 and NCK interacting kinase isoform 1 [Homo sapiens] | [18.5](http://blast.ncbi.nlm.nih.gov/Blast.cgi#55741807%2355741807) | 18.5 | 71% | 338 |
| [NP_068587.1](http://www.ncbi.nlm.nih.gov/entrez/query.fcgi?cmd=Retrieve&db=Protein&list_uids=11141885&dopt=GenPept&RID=T2X4GGAW016&log$=prottop&blast_rank=84) | solute carrier family 5 (choline transporter), member 7 [Homo sapiens] | [18.5](http://blast.ncbi.nlm.nih.gov/Blast.cgi#11141885%2311141885) | 18.5 | 71% | 338 |
| [NP_066979.2](http://www.ncbi.nlm.nih.gov/entrez/query.fcgi?cmd=Retrieve&db=Protein&list_uids=40254947&dopt=GenPept&RID=T2X4GGAW016&log$=prottop&blast_rank=85) | thioredoxin-related transmembrane protein 4 precursor [Homo sapiens] | [18.5](http://blast.ncbi.nlm.nih.gov/Blast.cgi#40254947%2340254947) | 34.4 | 71% | 338 |
| [XP_002344182.1](http://www.ncbi.nlm.nih.gov/entrez/query.fcgi?cmd=Retrieve&db=Protein&list_uids=239746020&dopt=GenPept&RID=T2X4GGAW016&log$=prottop&blast_rank=86) | PREDICTED: hypothetical protein XP_002344182 [Homo sapiens] | [18.0](http://blast.ncbi.nlm.nih.gov/Blast.cgi#239746020%23239746020) | 18.0 | 85% | 453 |
| [NP_113654.4](http://www.ncbi.nlm.nih.gov/entrez/query.fcgi?cmd=Retrieve&db=Protein&list_uids=238624122&dopt=GenPept&RID=T2X4GGAW016&log$=prottop&blast_rank=87) | trafficking protein particle complex 9 isoform a [Homo sapiens] | [18.0](http://blast.ncbi.nlm.nih.gov/Blast.cgi#238624122%23238624122) | 18.0 | 85% | 453 |
| [NP_001153844.1](http://www.ncbi.nlm.nih.gov/entrez/query.fcgi?cmd=Retrieve&db=Protein&list_uids=238624124&dopt=GenPept&RID=T2X4GGAW016&log$=prottop&blast_rank=88) | trafficking protein particle complex 9 isoform b [Homo sapiens] | [18.0](http://blast.ncbi.nlm.nih.gov/Blast.cgi#238624124%23238624124) | 18.0 | 85% | 453 |
| [NP_002153.2](http://www.ncbi.nlm.nih.gov/entrez/query.fcgi?cmd=Retrieve&db=Protein&list_uids=167466207&dopt=GenPept&RID=T2X4GGAW016&log$=prottop&blast_rank=89) | intercellular adhesion molecule 3 precursor [Homo sapiens] | [18.0](http://blast.ncbi.nlm.nih.gov/Blast.cgi#167466207%23167466207) | 18.0 | 85% | 453 |
| [NP_004413.1](http://www.ncbi.nlm.nih.gov/entrez/query.fcgi?cmd=Retrieve&db=Protein&list_uids=4758216&dopt=GenPept&RID=T2X4GGAW016&log$=prottop&blast_rank=90) | dishevelled 2 [Homo sapiens] | [18.0](http://blast.ncbi.nlm.nih.gov/Blast.cgi#4758216%234758216) | 18.0 | 85% | 453 |
| [NP_004412.2](http://www.ncbi.nlm.nih.gov/entrez/query.fcgi?cmd=Retrieve&db=Protein&list_uids=32479521&dopt=GenPept&RID=T2X4GGAW016&log$=prottop&blast_rank=91) | dishevelled 1 [Homo sapiens] | [18.0](http://blast.ncbi.nlm.nih.gov/Blast.cgi#32479521%2332479521) | 18.0 | 85% | 453 |
| [NP_115822.1](http://www.ncbi.nlm.nih.gov/entrez/query.fcgi?cmd=Retrieve&db=Protein&list_uids=14192943&dopt=GenPept&RID=T2X4GGAW016&log$=prottop&blast_rank=92) | multiple EGF-like-domains 10 precursor [Homo sapiens] | [18.0](http://blast.ncbi.nlm.nih.gov/Blast.cgi#14192943%2314192943) | 18.0 | 85% | 453 |
| [NP_001160174.1](http://www.ncbi.nlm.nih.gov/entrez/query.fcgi?cmd=Retrieve&db=Protein&list_uids=262359953&dopt=GenPept&RID=T2X4GGAW016&log$=prottop&blast_rank=93) | hypothetical protein LOC90736 isoform 5 [Homo sapiens] | [17.6](http://blast.ncbi.nlm.nih.gov/Blast.cgi#262359953%23262359953) | 17.6 | 100% | 608 |
| [NP_001160173.1](http://www.ncbi.nlm.nih.gov/entrez/query.fcgi?cmd=Retrieve&db=Protein&list_uids=262359951&dopt=GenPept&RID=T2X4GGAW016&log$=prottop&blast_rank=94) | hypothetical protein LOC90736 isoform 4 [Homo sapiens] | [17.6](http://blast.ncbi.nlm.nih.gov/Blast.cgi#262359951%23262359951) | 17.6 | 100% | 608 |
| [NP_001160172.1](http://www.ncbi.nlm.nih.gov/entrez/query.fcgi?cmd=Retrieve&db=Protein&list_uids=262359949&dopt=GenPept&RID=T2X4GGAW016&log$=prottop&blast_rank=95) | hypothetical protein LOC90736 isoform 3 [Homo sapiens] | [17.6](http://blast.ncbi.nlm.nih.gov/Blast.cgi#262359949%23262359949) | 17.6 | 100% | 608 |
| [NP_001158136.1](http://www.ncbi.nlm.nih.gov/entrez/query.fcgi?cmd=Retrieve&db=Protein&list_uids=257467648&dopt=GenPept&RID=T2X4GGAW016&log$=prottop&blast_rank=96) | microtubule associated serine/threonine kinase family member 4 isoform c [Homo sapiens] | [17.6](http://blast.ncbi.nlm.nih.gov/Blast.cgi#257467648%23257467648) | 17.6 | 85% | 608 |
| [NP_001156423.1](http://www.ncbi.nlm.nih.gov/entrez/query.fcgi?cmd=Retrieve&db=Protein&list_uids=244790013&dopt=GenPept&RID=T2X4GGAW016&log$=prottop&blast_rank=97) | synaptotagmin-like 2 isoform g [Homo sapiens] | [17.6](http://blast.ncbi.nlm.nih.gov/Blast.cgi#244790013%23244790013) | 17.6 | 85% | 608 |
| [XP_002344493.1](http://www.ncbi.nlm.nih.gov/entrez/query.fcgi?cmd=Retrieve&db=Protein&list_uids=239747100&dopt=GenPept&RID=T2X4GGAW016&log$=prottop&blast_rank=98) | PREDICTED: hypothetical protein XP_002344493 [Homo sapiens] | [17.6](http://blast.ncbi.nlm.nih.gov/Blast.cgi#239747100%23239747100) | 17.6 | 85% | 608 |
| [XP_002342657.1](http://www.ncbi.nlm.nih.gov/entrez/query.fcgi?cmd=Retrieve&db=Protein&list_uids=239742831&dopt=GenPept&RID=T2X4GGAW016&log$=prottop&blast_rank=99) | PREDICTED: hypothetical protein XP_002342657 [Homo sapiens] >ref|XP_002346825.1| PREDICTED: hypothetical protein [Homo sapiens] >ref|XP_002345949.1| PREDICTED: hypothetical protein [Homo sapiens] | [17.6](http://blast.ncbi.nlm.nih.gov/Blast.cgi#239742831%23239742831) | 17.6 | 100% | 608 |
| [NP_001156425.1](http://www.ncbi.nlm.nih.gov/entrez/query.fcgi?cmd=Retrieve&db=Protein&list_uids=244790020&dopt=GenPept&RID=T2X4GGAW016&log$=prottop&blast_rank=100) | synaptotagmin-like 2 isoform i [Homo sapiens] | [17.6](http://blast.ncbi.nlm.nih.gov/Blast.cgi#244790020%23244790020) | 17.6 | 85% | 608 |

| **Accession** | **Proteins with a match to HFHHLAVRGR peptides** | **[Max score](http://blast.ncbi.nlm.nih.gov/Blast.cgi?CMD=Get&ALIGNMENTS=100&ALIGNMENT_VIEW=Pairwise&CDD_SEARCH_STATE=1&DATABASE_SORT=0&DESCRIPTIONS=100&ENTREZ_QUERY=txid9606 %5BORGN%5D&FIRST_QUERY_NUM=0&FORMAT_OBJECT=Alignment&FORMAT_PAGE_TARGET=&FORMAT_TYPE=HTML&GET_SEQUENCE=yes&I_THRESH=&MASK_CHAR=2&MASK_COLOR=1&NEW_DESIGN=on&NEW_VIEW=yes&NUM_OVERVIEW=100&OLD_BLAST=false&PAGE=Proteins&QUERY_INDEX=0&QUERY_NUMBER=0&RESULTS_PAGE_TARGET=&RID=T2X8VGF8012&SHOW_LINKOUT=yes&SHOW_OVERVIEW=yes&STEP_NUMBER=&WORD_SIZE=2&DISPLAY_SORT=1&HSP_SORT=1" \l "sort_mark)** | **[Total score](http://blast.ncbi.nlm.nih.gov/Blast.cgi?CMD=Get&ALIGNMENTS=100&ALIGNMENT_VIEW=Pairwise&CDD_SEARCH_STATE=1&DATABASE_SORT=0&DESCRIPTIONS=100&ENTREZ_QUERY=txid9606 %5BORGN%5D&FIRST_QUERY_NUM=0&FORMAT_OBJECT=Alignment&FORMAT_PAGE_TARGET=&FORMAT_TYPE=HTML&GET_SEQUENCE=yes&I_THRESH=&MASK_CHAR=2&MASK_COLOR=1&NEW_DESIGN=on&NEW_VIEW=yes&NUM_OVERVIEW=100&OLD_BLAST=false&PAGE=Proteins&QUERY_INDEX=0&QUERY_NUMBER=0&RESULTS_PAGE_TARGET=&RID=T2X8VGF8012&SHOW_LINKOUT=yes&SHOW_OVERVIEW=yes&STEP_NUMBER=&WORD_SIZE=2&DISPLAY_SORT=2&HSP_SORT=1" \l "sort_mark)** | **[Query coverage](http://blast.ncbi.nlm.nih.gov/Blast.cgi?CMD=Get&ALIGNMENTS=100&ALIGNMENT_VIEW=Pairwise&CDD_SEARCH_STATE=1&DATABASE_SORT=0&DESCRIPTIONS=100&ENTREZ_QUERY=txid9606 %5BORGN%5D&FIRST_QUERY_NUM=0&FORMAT_OBJECT=Alignment&FORMAT_PAGE_TARGET=&FORMAT_TYPE=HTML&GET_SEQUENCE=yes&I_THRESH=&MASK_CHAR=2&MASK_COLOR=1&NEW_DESIGN=on&NEW_VIEW=yes&NUM_OVERVIEW=100&OLD_BLAST=false&PAGE=Proteins&QUERY_INDEX=0&QUERY_NUMBER=0&RESULTS_PAGE_TARGET=&RID=T2X8VGF8012&SHOW_LINKOUT=yes&SHOW_OVERVIEW=yes&STEP_NUMBER=&WORD_SIZE=2&DISPLAY_SORT=4&HSP_SORT=0" \l "sort_mark)** | **[E value](http://blast.ncbi.nlm.nih.gov/Blast.cgi?CMD=Get&ALIGNMENTS=100&ALIGNMENT_VIEW=Pairwise&CDD_SEARCH_STATE=1&DATABASE_SORT=0&DESCRIPTIONS=100&ENTREZ_QUERY=txid9606 %5BORGN%5D&FIRST_QUERY_NUM=0&FORMAT_OBJECT=Alignment&FORMAT_PAGE_TARGET=&FORMAT_TYPE=HTML&GET_SEQUENCE=yes&I_THRESH=&MASK_CHAR=2&MASK_COLOR=1&NEW_DESIGN=on&NEW_VIEW=yes&NUM_OVERVIEW=100&OLD_BLAST=false&PAGE=Proteins&QUERY_INDEX=0&QUERY_NUMBER=0&RESULTS_PAGE_TARGET=&RID=T2X8VGF8012&SHOW_LINKOUT=yes&SHOW_OVERVIEW=yes&STEP_NUMBER=&WORD_SIZE=2&DISPLAY_SORT=0&HSP_SORT=0" \l "sort_mark)** |
| --- | --- | --- | --- | --- | --- |
| [NP_859059.1](http://www.ncbi.nlm.nih.gov/entrez/query.fcgi?cmd=Retrieve&db=Protein&list_uids=32171233&dopt=GenPept&RID=T2X8VGF8012&log$=prottop&blast_rank=1) | BCDIN3 domain containing [Homo sapiens] | [24.4](http://blast.ncbi.nlm.nih.gov/Blast.cgi#32171233%2332171233) | 24.4 | 90% | 7.9 |
| [NP_001034589.2](http://www.ncbi.nlm.nih.gov/entrez/query.fcgi?cmd=Retrieve&db=Protein&list_uids=210147462&dopt=GenPept&RID=T2X8VGF8012&log$=prottop&blast_rank=2) | von Willebrand factor A domain containing 5B1 [Homo sapiens] | [22.7](http://blast.ncbi.nlm.nih.gov/Blast.cgi#210147462%23210147462) | 22.7 | 70% | 26 |
| [NP_065826.2](http://www.ncbi.nlm.nih.gov/entrez/query.fcgi?cmd=Retrieve&db=Protein&list_uids=38569482&dopt=GenPept&RID=T2X8VGF8012&log$=prottop&blast_rank=3) | hypothetical protein LOC57535 precursor [Homo sapiens] | [22.7](http://blast.ncbi.nlm.nih.gov/Blast.cgi#38569482%2338569482) | 22.7 | 100% | 26 |
| [NP_001092093.1](http://www.ncbi.nlm.nih.gov/entrez/query.fcgi?cmd=Retrieve&db=Protein&list_uids=148833506&dopt=GenPept&RID=T2X8VGF8012&log$=prottop&blast_rank=4) | obscurin, cytoskeletal calmodulin and titin-interacting RhoGEF isoform b [Homo sapiens] | [21.4](http://blast.ncbi.nlm.nih.gov/Blast.cgi#148833506%23148833506) | 48.2 | 90% | 62 |
| [NP_001136430.1](http://www.ncbi.nlm.nih.gov/entrez/query.fcgi?cmd=Retrieve&db=Protein&list_uids=219281831&dopt=GenPept&RID=T2X8VGF8012&log$=prottop&blast_rank=5) | F-box protein 15 isoform 2 [Homo sapiens] | [21.4](http://blast.ncbi.nlm.nih.gov/Blast.cgi#219281831%23219281831) | 21.4 | 50% | 62 |
| [NP_443075.2](http://www.ncbi.nlm.nih.gov/entrez/query.fcgi?cmd=Retrieve&db=Protein&list_uids=58331253&dopt=GenPept&RID=T2X8VGF8012&log$=prottop&blast_rank=6) | obscurin, cytoskeletal calmodulin and titin-interacting RhoGEF isoform a [Homo sapiens] | [21.4](http://blast.ncbi.nlm.nih.gov/Blast.cgi#58331253%2358331253) | 48.2 | 90% | 62 |
| [NP_002493.3](http://www.ncbi.nlm.nih.gov/entrez/query.fcgi?cmd=Retrieve&db=Protein&list_uids=117320527&dopt=GenPept&RID=T2X8VGF8012&log$=prottop&blast_rank=7) | nuclear factor of kappa light polypeptide gene enhancer in B-cells 2 isoform b [Homo sapiens] >ref|NP_001070961.1| nuclear factor of kappa light polypeptide gene enhancer in B-cells 2 isoform b [Homo sapiens] | [21.4](http://blast.ncbi.nlm.nih.gov/Blast.cgi#117320527%23117320527) | 37.8 | 100% | 62 |
| [NP_689889.1](http://www.ncbi.nlm.nih.gov/entrez/query.fcgi?cmd=Retrieve&db=Protein&list_uids=22749363&dopt=GenPept&RID=T2X8VGF8012&log$=prottop&blast_rank=8) | F-box protein 15 isoform 1 [Homo sapiens] | [21.4](http://blast.ncbi.nlm.nih.gov/Blast.cgi#22749363%2322749363) | 21.4 | 50% | 62 |
| [NP_001070962.1](http://www.ncbi.nlm.nih.gov/entrez/query.fcgi?cmd=Retrieve&db=Protein&list_uids=117320531&dopt=GenPept&RID=T2X8VGF8012&log$=prottop&blast_rank=9) | nuclear factor of kappa light polypeptide gene enhancer in B-cells 2 isoform a [Homo sapiens] | [21.4](http://blast.ncbi.nlm.nih.gov/Blast.cgi#117320531%23117320531) | 37.8 | 100% | 62 |
| [XP_002345045.1](http://www.ncbi.nlm.nih.gov/entrez/query.fcgi?cmd=Retrieve&db=Protein&list_uids=239756750&dopt=GenPept&RID=T2X8VGF8012&log$=prottop&blast_rank=10) | PREDICTED: similar to TBC1 domain family, member 28 [Homo sapiens] | [21.0](http://blast.ncbi.nlm.nih.gov/Blast.cgi#239756750%23239756750) | 21.0 | 60% | 83 |
| [NP_055284.3](http://www.ncbi.nlm.nih.gov/entrez/query.fcgi?cmd=Retrieve&db=Protein&list_uids=153252068&dopt=GenPept&RID=T2X8VGF8012&log$=prottop&blast_rank=11) | testes-specific heterogenous nuclear ribonucleoprotein G-T [Homo sapiens] | [21.0](http://blast.ncbi.nlm.nih.gov/Blast.cgi#153252068%23153252068) | 21.0 | 60% | 83 |
| [NP_848666.2](http://www.ncbi.nlm.nih.gov/entrez/query.fcgi?cmd=Retrieve&db=Protein&list_uids=148235002&dopt=GenPept&RID=T2X8VGF8012&log$=prottop&blast_rank=12) | TBC1 domain family, member 26 [Homo sapiens] | [21.0](http://blast.ncbi.nlm.nih.gov/Blast.cgi#148235002%23148235002) | 21.0 | 60% | 83 |
| [NP_001073933.1](http://www.ncbi.nlm.nih.gov/entrez/query.fcgi?cmd=Retrieve&db=Protein&list_uids=122937331&dopt=GenPept&RID=T2X8VGF8012&log$=prottop&blast_rank=13) | 60 kDa lysophospholipase [Homo sapiens] | [21.0](http://blast.ncbi.nlm.nih.gov/Blast.cgi#122937331%23122937331) | 21.0 | 60% | 83 |
| [NP_001034486.2](http://www.ncbi.nlm.nih.gov/entrez/query.fcgi?cmd=Retrieve&db=Protein&list_uids=151108489&dopt=GenPept&RID=T2X8VGF8012&log$=prottop&blast_rank=14) | TBC1 domain family, member 28 [Homo sapiens] | [21.0](http://blast.ncbi.nlm.nih.gov/Blast.cgi#151108489%23151108489) | 21.0 | 60% | 83 |
| [NP_620594.1](http://www.ncbi.nlm.nih.gov/entrez/query.fcgi?cmd=Retrieve&db=Protein&list_uids=21265034&dopt=GenPept&RID=T2X8VGF8012&log$=prottop&blast_rank=15) | ADAM metallopeptidase with thrombospondin type 1 motif, 13 isoform 1 preproprotein [Homo sapiens] | [21.0](http://blast.ncbi.nlm.nih.gov/Blast.cgi#21265034%2321265034) | 49.4 | 90% | 83 |
| [NP_620596.2](http://www.ncbi.nlm.nih.gov/entrez/query.fcgi?cmd=Retrieve&db=Protein&list_uids=73695936&dopt=GenPept&RID=T2X8VGF8012&log$=prottop&blast_rank=16) | ADAM metallopeptidase with thrombospondin type 1 motif, 13 isoform 2 preproprotein [Homo sapiens] | [21.0](http://blast.ncbi.nlm.nih.gov/Blast.cgi#73695936%2373695936) | 49.4 | 90% | 83 |
| [NP_620595.1](http://www.ncbi.nlm.nih.gov/entrez/query.fcgi?cmd=Retrieve&db=Protein&list_uids=21265043&dopt=GenPept&RID=T2X8VGF8012&log$=prottop&blast_rank=17) | ADAM metallopeptidase with thrombospondin type 1 motif, 13 isoform 3 preproprotein [Homo sapiens] | [21.0](http://blast.ncbi.nlm.nih.gov/Blast.cgi#21265043%2321265043) | 49.4 | 90% | 83 |
| [NP_001157980.1](http://www.ncbi.nlm.nih.gov/entrez/query.fcgi?cmd=Retrieve&db=Protein&list_uids=257743025&dopt=GenPept&RID=T2X8VGF8012&log$=prottop&blast_rank=18) | nebulin isoform 2 [Homo sapiens] | [20.6](http://blast.ncbi.nlm.nih.gov/Blast.cgi#257743025%23257743025) | 20.6 | 70% | 111 |
| [NP_001157979.1](http://www.ncbi.nlm.nih.gov/entrez/query.fcgi?cmd=Retrieve&db=Protein&list_uids=257743023&dopt=GenPept&RID=T2X8VGF8012&log$=prottop&blast_rank=19) | nebulin isoform 1 [Homo sapiens] | [20.6](http://blast.ncbi.nlm.nih.gov/Blast.cgi#257743023%23257743023) | 20.6 | 70% | 111 |
| [NP_004534.2](http://www.ncbi.nlm.nih.gov/entrez/query.fcgi?cmd=Retrieve&db=Protein&list_uids=115527120&dopt=GenPept&RID=T2X8VGF8012&log$=prottop&blast_rank=20) | nebulin isoform 3 [Homo sapiens] | [20.6](http://blast.ncbi.nlm.nih.gov/Blast.cgi#115527120%23115527120) | 20.6 | 70% | 111 |
| [NP_079106.3](http://www.ncbi.nlm.nih.gov/entrez/query.fcgi?cmd=Retrieve&db=Protein&list_uids=33946291&dopt=GenPept&RID=T2X8VGF8012&log$=prottop&blast_rank=21) | lysophosphatidylcholine acyltransferase 1 [Homo sapiens] | [20.6](http://blast.ncbi.nlm.nih.gov/Blast.cgi#33946291%2333946291) | 31.0 | 90% | 111 |
| [NP_000825.2](http://www.ncbi.nlm.nih.gov/entrez/query.fcgi?cmd=Retrieve&db=Protein&list_uids=167003331&dopt=GenPept&RID=T2X8VGF8012&log$=prottop&blast_rank=22) | N-methyl-D-aspartate receptor subunit 2B precursor [Homo sapiens] | [20.6](http://blast.ncbi.nlm.nih.gov/Blast.cgi#167003331%23167003331) | 36.1 | 70% | 111 |
| [NP_001018025.1](http://www.ncbi.nlm.nih.gov/entrez/query.fcgi?cmd=Retrieve&db=Protein&list_uids=66346715&dopt=GenPept&RID=T2X8VGF8012&log$=prottop&blast_rank=23) | mature T-cell proliferation 1 [Homo sapiens] | [20.6](http://blast.ncbi.nlm.nih.gov/Blast.cgi#66346715%2366346715) | 20.6 | 70% | 111 |
| [NP_009117.2](http://www.ncbi.nlm.nih.gov/entrez/query.fcgi?cmd=Retrieve&db=Protein&list_uids=21735548&dopt=GenPept&RID=T2X8VGF8012&log$=prottop&blast_rank=24) | centrosomal protein 2 [Homo sapiens] | [20.6](http://blast.ncbi.nlm.nih.gov/Blast.cgi#21735548%2321735548) | 34.4 | 70% | 111 |
| [NP_001092302.1](http://www.ncbi.nlm.nih.gov/entrez/query.fcgi?cmd=Retrieve&db=Protein&list_uids=154350246&dopt=GenPept&RID=T2X8VGF8012&log$=prottop&blast_rank=25) | hypothetical protein LOC84923 isoform 1 [Homo sapiens] | [20.2](http://blast.ncbi.nlm.nih.gov/Blast.cgi#154350246%23154350246) | 20.2 | 100% | 149 |
| [NP_116226.2](http://www.ncbi.nlm.nih.gov/entrez/query.fcgi?cmd=Retrieve&db=Protein&list_uids=154350224&dopt=GenPept&RID=T2X8VGF8012&log$=prottop&blast_rank=26) | hypothetical protein LOC84923 isoform 2 [Homo sapiens] | [20.2](http://blast.ncbi.nlm.nih.gov/Blast.cgi#154350224%23154350224) | 20.2 | 100% | 149 |
| [NP_116277.2](http://www.ncbi.nlm.nih.gov/entrez/query.fcgi?cmd=Retrieve&db=Protein&list_uids=32140760&dopt=GenPept&RID=T2X8VGF8012&log$=prottop&blast_rank=27) | collagen, type XXVII, alpha 1 precursor [Homo sapiens] | [20.2](http://blast.ncbi.nlm.nih.gov/Blast.cgi#32140760%2332140760) | 32.7 | 80% | 149 |
| [NP_071927.1](http://www.ncbi.nlm.nih.gov/entrez/query.fcgi?cmd=Retrieve&db=Protein&list_uids=11968150&dopt=GenPept&RID=T2X8VGF8012&log$=prottop&blast_rank=28) | GDNF-inducible zinc finger protein 1 [Homo sapiens] | [20.2](http://blast.ncbi.nlm.nih.gov/Blast.cgi#11968150%2311968150) | 20.2 | 60% | 149 |
| [NP_060352.1](http://www.ncbi.nlm.nih.gov/entrez/query.fcgi?cmd=Retrieve&db=Protein&list_uids=8923532&dopt=GenPept&RID=T2X8VGF8012&log$=prottop&blast_rank=29) | CLN6 protein [Homo sapiens] | [20.2](http://blast.ncbi.nlm.nih.gov/Blast.cgi#8923532%238923532) | 20.2 | 60% | 149 |
| [NP_000341.2](http://www.ncbi.nlm.nih.gov/entrez/query.fcgi?cmd=Retrieve&db=Protein&list_uids=105990541&dopt=GenPept&RID=T2X8VGF8012&log$=prottop&blast_rank=30) | ATP-binding cassette, sub-family A member 4 [Homo sapiens] | [20.2](http://blast.ncbi.nlm.nih.gov/Blast.cgi#105990541%23105990541) | 20.2 | 60% | 149 |
| [XP_001720383.2](http://www.ncbi.nlm.nih.gov/entrez/query.fcgi?cmd=Retrieve&db=Protein&list_uids=239747766&dopt=GenPept&RID=T2X8VGF8012&log$=prottop&blast_rank=31) | PREDICTED: hypothetical protein [Homo sapiens] >ref|XP_001719428.2| PREDICTED: hypothetical protein [Homo sapiens] | [19.7](http://blast.ncbi.nlm.nih.gov/Blast.cgi#239747766%23239747766) | 19.7 | 70% | 200 |
| [XP_002342252.1](http://www.ncbi.nlm.nih.gov/entrez/query.fcgi?cmd=Retrieve&db=Protein&list_uids=239741632&dopt=GenPept&RID=T2X8VGF8012&log$=prottop&blast_rank=32) | PREDICTED: hypothetical protein XP_002342252 [Homo sapiens] | [19.7](http://blast.ncbi.nlm.nih.gov/Blast.cgi#239741632%23239741632) | 19.7 | 70% | 200 |
| [NP_079044.2](http://www.ncbi.nlm.nih.gov/entrez/query.fcgi?cmd=Retrieve&db=Protein&list_uids=228008351&dopt=GenPept&RID=T2X8VGF8012&log$=prottop&blast_rank=33) | coiled-coil domain containing 48 [Homo sapiens] | [19.7](http://blast.ncbi.nlm.nih.gov/Blast.cgi#228008351%23228008351) | 19.7 | 80% | 200 |
| [XP_001720202.1](http://www.ncbi.nlm.nih.gov/entrez/query.fcgi?cmd=Retrieve&db=Protein&list_uids=169177385&dopt=GenPept&RID=T2X8VGF8012&log$=prottop&blast_rank=34) | PREDICTED: hypothetical protein [Homo sapiens] | [19.7](http://blast.ncbi.nlm.nih.gov/Blast.cgi#169177385%23169177385) | 19.7 | 100% | 200 |
| [XP_931822.2](http://www.ncbi.nlm.nih.gov/entrez/query.fcgi?cmd=Retrieve&db=Protein&list_uids=169177307&dopt=GenPept&RID=T2X8VGF8012&log$=prottop&blast_rank=35) | PREDICTED: hypothetical protein [Homo sapiens] | [19.7](http://blast.ncbi.nlm.nih.gov/Blast.cgi#169177307%23169177307) | 19.7 | 100% | 200 |
| [NP_115928.1](http://www.ncbi.nlm.nih.gov/entrez/query.fcgi?cmd=Retrieve&db=Protein&list_uids=33504581&dopt=GenPept&RID=T2X8VGF8012&log$=prottop&blast_rank=36) | SLIT and NTRK-like family, member 2 precursor [Homo sapiens] >ref|NP_001137475.1| SLIT and NTRK-like family, member 2 precursor [Homo sapiens] >ref|NP_001137476.1| SLIT and NTRK-like family, member 2 precursor [Homo sapiens] >ref|NP_001137477.1| SLIT and NTRK-like family, member 2 precursor [Homo sapiens] >ref|NP_001137478.1| SLIT and NTRK-like family, member 2 precursor [Homo sapiens] >ref|NP_001137480.1| SLIT and NTRK-like family, member 2 precursor [Homo sapiens] >ref|NP_001137481.1| SLIT and NTRK-like family, member 2 precursor [Homo sapiens] >ref|NP_001137482.1| SLIT and NTRK-like family, member 2 precursor [Homo sapiens] | [19.7](http://blast.ncbi.nlm.nih.gov/Blast.cgi#33504581%2333504581) | 33.5 | 90% | 200 |
| [NP_065854.3](http://www.ncbi.nlm.nih.gov/entrez/query.fcgi?cmd=Retrieve&db=Protein&list_uids=34101268&dopt=GenPept&RID=T2X8VGF8012&log$=prottop&blast_rank=37) | kelch-like 8 [Homo sapiens] | [19.7](http://blast.ncbi.nlm.nih.gov/Blast.cgi#34101268%2334101268) | 19.7 | 80% | 200 |
| [XP_002343634.1](http://www.ncbi.nlm.nih.gov/entrez/query.fcgi?cmd=Retrieve&db=Protein&list_uids=239746037&dopt=GenPept&RID=T2X8VGF8012&log$=prottop&blast_rank=38) | PREDICTED: hypothetical protein XP_002343634 [Homo sapiens] >ref|XP_002347889.1| PREDICTED: hypothetical protein [Homo sapiens] >ref|XP_002345147.1| PREDICTED: hypothetical protein [Homo sapiens] | [19.3](http://blast.ncbi.nlm.nih.gov/Blast.cgi#239746037%23239746037) | 19.3 | 50% | 268 |
| [NP_065996.1](http://www.ncbi.nlm.nih.gov/entrez/query.fcgi?cmd=Retrieve&db=Protein&list_uids=191252801&dopt=GenPept&RID=T2X8VGF8012&log$=prottop&blast_rank=39) | WDFY family member 4 [Homo sapiens] | [19.3](http://blast.ncbi.nlm.nih.gov/Blast.cgi#191252801%23191252801) | 33.9 | 90% | 268 |
| [NP_057726.3](http://www.ncbi.nlm.nih.gov/entrez/query.fcgi?cmd=Retrieve&db=Protein&list_uids=170016061&dopt=GenPept&RID=T2X8VGF8012&log$=prottop&blast_rank=40) | spectrin, beta, non-erythrocytic 5 [Homo sapiens] | [19.3](http://blast.ncbi.nlm.nih.gov/Blast.cgi#170016061%23170016061) | 31.4 | 60% | 268 |
| [XP_002344438.1](http://www.ncbi.nlm.nih.gov/entrez/query.fcgi?cmd=Retrieve&db=Protein&list_uids=239509071&dopt=GenPept&RID=T2X8VGF8012&log$=prottop&blast_rank=41) | PREDICTED: similar to zinc finger protein 30 [Homo sapiens] >ref|XP_002342831.1| PREDICTED: hypothetical protein XP_002342831 [Homo sapiens] >ref|XP_002346980.1| PREDICTED: hypothetical protein XP_002346980 [Homo sapiens] >ref|XP_002346096.1| PREDICTED: similar to zinc finger protein 30 [Homo sapiens] | [19.3](http://blast.ncbi.nlm.nih.gov/Blast.cgi#239509071%23239509071) | 19.3 | 50% | 268 |
| [NP_001092289.1](http://www.ncbi.nlm.nih.gov/entrez/query.fcgi?cmd=Retrieve&db=Protein&list_uids=149363666&dopt=GenPept&RID=T2X8VGF8012&log$=prottop&blast_rank=42) | phosphodiesterase 4C isoform PDE4C-2 [Homo sapiens] | [19.3](http://blast.ncbi.nlm.nih.gov/Blast.cgi#149363666%23149363666) | 19.3 | 50% | 268 |
| [NP_001092288.1](http://www.ncbi.nlm.nih.gov/entrez/query.fcgi?cmd=Retrieve&db=Protein&list_uids=149363646&dopt=GenPept&RID=T2X8VGF8012&log$=prottop&blast_rank=43) | phosphodiesterase 4C isoform PDE4C-3 [Homo sapiens] | [19.3](http://blast.ncbi.nlm.nih.gov/Blast.cgi#149363646%23149363646) | 19.3 | 50% | 268 |
| [NP_055059.2](http://www.ncbi.nlm.nih.gov/entrez/query.fcgi?cmd=Retrieve&db=Protein&list_uids=110825974&dopt=GenPept&RID=T2X8VGF8012&log$=prottop&blast_rank=44) | ADAM metallopeptidase with thrombospondin type 1 motif, 2 isoform 1 preproprotein [Homo sapiens] | [19.3](http://blast.ncbi.nlm.nih.gov/Blast.cgi#110825974%23110825974) | 19.3 | 50% | 268 |
| [NP_001026973.1](http://www.ncbi.nlm.nih.gov/entrez/query.fcgi?cmd=Retrieve&db=Protein&list_uids=73427803&dopt=GenPept&RID=T2X8VGF8012&log$=prottop&blast_rank=45) | lethal giant larvae homolog 2 isoform c [Homo sapiens] | [19.3](http://blast.ncbi.nlm.nih.gov/Blast.cgi#73427803%2373427803) | 19.3 | 50% | 268 |
| [NP_004515.2](http://www.ncbi.nlm.nih.gov/entrez/query.fcgi?cmd=Retrieve&db=Protein&list_uids=62739161&dopt=GenPept&RID=T2X8VGF8012&log$=prottop&blast_rank=46) | lethal giant larvae homolog 2 isoform a [Homo sapiens] | [19.3](http://blast.ncbi.nlm.nih.gov/Blast.cgi#62739161%2362739161) | 19.3 | 50% | 268 |
| [NP_001073940.1](http://www.ncbi.nlm.nih.gov/entrez/query.fcgi?cmd=Retrieve&db=Protein&list_uids=122937343&dopt=GenPept&RID=T2X8VGF8012&log$=prottop&blast_rank=47) | platelet endothelial aggregation receptor 1 precursor [Homo sapiens] | [19.3](http://blast.ncbi.nlm.nih.gov/Blast.cgi#122937343%23122937343) | 19.3 | 50% | 268 |
| [NP_001032242.1](http://www.ncbi.nlm.nih.gov/entrez/query.fcgi?cmd=Retrieve&db=Protein&list_uids=82546824&dopt=GenPept&RID=T2X8VGF8012&log$=prottop&blast_rank=48) | forkhead box K1 [Homo sapiens] | [19.3](http://blast.ncbi.nlm.nih.gov/Blast.cgi#82546824%2382546824) | 19.3 | 60% | 268 |
| [NP_001104779.1](http://www.ncbi.nlm.nih.gov/entrez/query.fcgi?cmd=Retrieve&db=Protein&list_uids=162329612&dopt=GenPept&RID=T2X8VGF8012&log$=prottop&blast_rank=49) | phosphodiesterase 4A isoform 3 [Homo sapiens] | [19.3](http://blast.ncbi.nlm.nih.gov/Blast.cgi#162329612%23162329612) | 19.3 | 50% | 268 |
| [NP_001159371.1](http://www.ncbi.nlm.nih.gov/entrez/query.fcgi?cmd=Retrieve&db=Protein&list_uids=259906420&dopt=GenPept&RID=T2X8VGF8012&log$=prottop&blast_rank=50) | phosphodiesterase 4D isoform 3 [Homo sapiens] | [19.3](http://blast.ncbi.nlm.nih.gov/Blast.cgi#259906420%23259906420) | 19.3 | 50% | 268 |
| [NP_001104777.1](http://www.ncbi.nlm.nih.gov/entrez/query.fcgi?cmd=Retrieve&db=Protein&list_uids=162329608&dopt=GenPept&RID=T2X8VGF8012&log$=prottop&blast_rank=51) | phosphodiesterase 4A isoform 1 [Homo sapiens] | [19.3](http://blast.ncbi.nlm.nih.gov/Blast.cgi#162329608%23162329608) | 19.3 | 50% | 268 |
| [NP_001104778.1](http://www.ncbi.nlm.nih.gov/entrez/query.fcgi?cmd=Retrieve&db=Protein&list_uids=162329610&dopt=GenPept&RID=T2X8VGF8012&log$=prottop&blast_rank=52) | phosphodiesterase 4A isoform 2 [Homo sapiens] | [19.3](http://blast.ncbi.nlm.nih.gov/Blast.cgi#162329610%23162329610) | 19.3 | 50% | 268 |
| [NP_006193.1](http://www.ncbi.nlm.nih.gov/entrez/query.fcgi?cmd=Retrieve&db=Protein&list_uids=5453862&dopt=GenPept&RID=T2X8VGF8012&log$=prottop&blast_rank=53) | phosphodiesterase 4A isoform 4 [Homo sapiens] | [19.3](http://blast.ncbi.nlm.nih.gov/Blast.cgi#5453862%235453862) | 19.3 | 50% | 268 |
| [NP_958443.1](http://www.ncbi.nlm.nih.gov/entrez/query.fcgi?cmd=Retrieve&db=Protein&list_uids=41152235&dopt=GenPept&RID=T2X8VGF8012&log$=prottop&blast_rank=54) | ubiquitin specific protease 51 [Homo sapiens] | [19.3](http://blast.ncbi.nlm.nih.gov/Blast.cgi#41152235%2341152235) | 19.3 | 50% | 268 |
| [NP_001032417.1](http://www.ncbi.nlm.nih.gov/entrez/query.fcgi?cmd=Retrieve&db=Protein&list_uids=82799484&dopt=GenPept&RID=T2X8VGF8012&log$=prottop&blast_rank=55) | phosphodiesterase 4B isoform 3 [Homo sapiens] | [19.3](http://blast.ncbi.nlm.nih.gov/Blast.cgi#82799484%2382799484) | 19.3 | 50% | 268 |
| [NP_001098101.1](http://www.ncbi.nlm.nih.gov/entrez/query.fcgi?cmd=Retrieve&db=Protein&list_uids=157277988&dopt=GenPept&RID=T2X8VGF8012&log$=prottop&blast_rank=56) | phosphodiesterase 4D isoform 1 [Homo sapiens] | [19.3](http://blast.ncbi.nlm.nih.gov/Blast.cgi#157277988%23157277988) | 19.3 | 50% | 268 |
| [NP_000914.2](http://www.ncbi.nlm.nih.gov/entrez/query.fcgi?cmd=Retrieve&db=Protein&list_uids=115529445&dopt=GenPept&RID=T2X8VGF8012&log$=prottop&blast_rank=57) | phosphodiesterase 4C isoform PDE4C-1 [Homo sapiens] | [19.3](http://blast.ncbi.nlm.nih.gov/Blast.cgi#115529445%23115529445) | 19.3 | 50% | 268 |
| [NP_001032416.1](http://www.ncbi.nlm.nih.gov/entrez/query.fcgi?cmd=Retrieve&db=Protein&list_uids=82799482&dopt=GenPept&RID=T2X8VGF8012&log$=prottop&blast_rank=58) | phosphodiesterase 4B isoform 2 [Homo sapiens] | [19.3](http://blast.ncbi.nlm.nih.gov/Blast.cgi#82799482%2382799482) | 19.3 | 50% | 268 |
| [NP_002591.2](http://www.ncbi.nlm.nih.gov/entrez/query.fcgi?cmd=Retrieve&db=Protein&list_uids=32171241&dopt=GenPept&RID=T2X8VGF8012&log$=prottop&blast_rank=59) | phosphodiesterase 4B isoform 1 [Homo sapiens] >ref|NP_001032418.1| phosphodiesterase 4B isoform 1 [Homo sapiens] | [19.3](http://blast.ncbi.nlm.nih.gov/Blast.cgi#32171241%2332171241) | 19.3 | 50% | 268 |
| [NP_006194.2](http://www.ncbi.nlm.nih.gov/entrez/query.fcgi?cmd=Retrieve&db=Protein&list_uids=32306513&dopt=GenPept&RID=T2X8VGF8012&log$=prottop&blast_rank=60) | phosphodiesterase 4D isoform 2 [Homo sapiens] | [19.3](http://blast.ncbi.nlm.nih.gov/Blast.cgi#32306513%2332306513) | 19.3 | 50% | 268 |
| [NP_694962.1](http://www.ncbi.nlm.nih.gov/entrez/query.fcgi?cmd=Retrieve&db=Protein&list_uids=23397500&dopt=GenPept&RID=T2X8VGF8012&log$=prottop&blast_rank=61) | F-box protein 39 [Homo sapiens] | [19.3](http://blast.ncbi.nlm.nih.gov/Blast.cgi#23397500%2323397500) | 19.3 | 50% | 268 |
| [NP_001019628.3](http://www.ncbi.nlm.nih.gov/entrez/query.fcgi?cmd=Retrieve&db=Protein&list_uids=262118265&dopt=GenPept&RID=T2X8VGF8012&log$=prottop&blast_rank=62) | RANBP2-like and GRIP domain containing 1 [Homo sapiens] | [18.9](http://blast.ncbi.nlm.nih.gov/Blast.cgi#262118265%23262118265) | 36.5 | 70% | 360 |
| [XP_002343747.1](http://www.ncbi.nlm.nih.gov/entrez/query.fcgi?cmd=Retrieve&db=Protein&list_uids=239746497&dopt=GenPept&RID=T2X8VGF8012&log$=prottop&blast_rank=63) | PREDICTED: hypothetical protein XP_002343747 [Homo sapiens] >ref|XP_002348067.1| PREDICTED: hypothetical protein XP_002348067 [Homo sapiens] >ref|XP_002345465.1| PREDICTED: hypothetical protein [Homo sapiens] | [18.9](http://blast.ncbi.nlm.nih.gov/Blast.cgi#239746497%23239746497) | 18.9 | 60% | 360 |
| [NP_000404.2](http://www.ncbi.nlm.nih.gov/entrez/query.fcgi?cmd=Retrieve&db=Protein&list_uids=223718074&dopt=GenPept&RID=T2X8VGF8012&log$=prottop&blast_rank=64) | hydroxysteroid (17-beta) dehydrogenase 1 [Homo sapiens] | [18.9](http://blast.ncbi.nlm.nih.gov/Blast.cgi#223718074%23223718074) | 18.9 | 50% | 360 |
| [NP_001138740.1](http://www.ncbi.nlm.nih.gov/entrez/query.fcgi?cmd=Retrieve&db=Protein&list_uids=223671925&dopt=GenPept&RID=T2X8VGF8012&log$=prottop&blast_rank=65) | hypothetical protein LOC222234 isoform 1 [Homo sapiens] | [18.9](http://blast.ncbi.nlm.nih.gov/Blast.cgi#223671925%23223671925) | 18.9 | 50% | 360 |
| [NP_872394.2](http://www.ncbi.nlm.nih.gov/entrez/query.fcgi?cmd=Retrieve&db=Protein&list_uids=211059431&dopt=GenPept&RID=T2X8VGF8012&log$=prottop&blast_rank=66) | RANBP2-like and GRIP domain containing 4 [Homo sapiens] | [18.9](http://blast.ncbi.nlm.nih.gov/Blast.cgi#211059431%23211059431) | 36.5 | 70% | 360 |
| [NP_001157915.1](http://www.ncbi.nlm.nih.gov/entrez/query.fcgi?cmd=Retrieve&db=Protein&list_uids=256574792&dopt=GenPept&RID=T2X8VGF8012&log$=prottop&blast_rank=67) | ankyrin repeat domain 31 [Homo sapiens] >ref|XP_942490.4| PREDICTED: ankyrin repeat domain 31 [Homo sapiens] >ref|XP_001714521.2| PREDICTED: ankyrin repeat domain 31 [Homo sapiens] | [18.9](http://blast.ncbi.nlm.nih.gov/Blast.cgi#256574792%23256574792) | 18.9 | 50% | 360 |
| [NP_001128663.1](http://www.ncbi.nlm.nih.gov/entrez/query.fcgi?cmd=Retrieve&db=Protein&list_uids=206597522&dopt=GenPept&RID=T2X8VGF8012&log$=prottop&blast_rank=68) | ArfGAP with SH3 domain, ankyrin repeat and PH domain 2 isoform b [Homo sapiens] | [18.9](http://blast.ncbi.nlm.nih.gov/Blast.cgi#206597522%23206597522) | 18.9 | 50% | 360 |
| [NP_689856.6](http://www.ncbi.nlm.nih.gov/entrez/query.fcgi?cmd=Retrieve&db=Protein&list_uids=116268127&dopt=GenPept&RID=T2X8VGF8012&log$=prottop&blast_rank=69) | kinase non-catalytic C-lobe domain (KIND) containing 1 isoform a [Homo sapiens] | [18.9](http://blast.ncbi.nlm.nih.gov/Blast.cgi#116268127%23116268127) | 18.9 | 50% | 360 |
| [NP_874362.3](http://www.ncbi.nlm.nih.gov/entrez/query.fcgi?cmd=Retrieve&db=Protein&list_uids=96975023&dopt=GenPept&RID=T2X8VGF8012&log$=prottop&blast_rank=70) | ankyrin repeat and death domain containing 1A [Homo sapiens] | [18.9](http://blast.ncbi.nlm.nih.gov/Blast.cgi#96975023%2396975023) | 18.9 | 50% | 360 |
| [NP_003768.2](http://www.ncbi.nlm.nih.gov/entrez/query.fcgi?cmd=Retrieve&db=Protein&list_uids=51479173&dopt=GenPept&RID=T2X8VGF8012&log$=prottop&blast_rank=71) | dynein, axonemal, heavy chain 11 [Homo sapiens] | [18.9](http://blast.ncbi.nlm.nih.gov/Blast.cgi#51479173%2351479173) | 49.4 | 80% | 360 |
| [NP_060952.2](http://www.ncbi.nlm.nih.gov/entrez/query.fcgi?cmd=Retrieve&db=Protein&list_uids=46094081&dopt=GenPept&RID=T2X8VGF8012&log$=prottop&blast_rank=72) | development and differentiation enhancing factor 1 [Homo sapiens] | [18.9](http://blast.ncbi.nlm.nih.gov/Blast.cgi#46094081%2346094081) | 18.9 | 50% | 360 |
| [NP_067545.3](http://www.ncbi.nlm.nih.gov/entrez/query.fcgi?cmd=Retrieve&db=Protein&list_uids=153218470&dopt=GenPept&RID=T2X8VGF8012&log$=prottop&blast_rank=73) | BARX homeobox 1 [Homo sapiens] | [18.9](http://blast.ncbi.nlm.nih.gov/Blast.cgi#153218470%23153218470) | 18.9 | 60% | 360 |
| [NP_001157935.1](http://www.ncbi.nlm.nih.gov/entrez/query.fcgi?cmd=Retrieve&db=Protein&list_uids=256600210&dopt=GenPept&RID=T2X8VGF8012&log$=prottop&blast_rank=74) | RANBP2-like and GRIP domain containing 8 [Homo sapiens] | [18.9](http://blast.ncbi.nlm.nih.gov/Blast.cgi#256600210%23256600210) | 18.9 | 50% | 360 |
| [NP_001071638.2](http://www.ncbi.nlm.nih.gov/entrez/query.fcgi?cmd=Retrieve&db=Protein&list_uids=262118271&dopt=GenPept&RID=T2X8VGF8012&log$=prottop&blast_rank=75) | RANBP2-like and GRIP domain containing 2 [Homo sapiens] | [18.9](http://blast.ncbi.nlm.nih.gov/Blast.cgi#262118271%23262118271) | 36.5 | 70% | 360 |
| [NP_001137485.1](http://www.ncbi.nlm.nih.gov/entrez/query.fcgi?cmd=Retrieve&db=Protein&list_uids=221307607&dopt=GenPept&RID=T2X8VGF8012&log$=prottop&blast_rank=76) | RANBP2-like and GRIP domain containing 3 [Homo sapiens] | [18.9](http://blast.ncbi.nlm.nih.gov/Blast.cgi#221307607%23221307607) | 36.5 | 70% | 360 |
| [NP_004929.2](http://www.ncbi.nlm.nih.gov/entrez/query.fcgi?cmd=Retrieve&db=Protein&list_uids=89363047&dopt=GenPept&RID=T2X8VGF8012&log$=prottop&blast_rank=77) | death-associated protein kinase 1 [Homo sapiens] | [18.9](http://blast.ncbi.nlm.nih.gov/Blast.cgi#89363047%2389363047) | 30.1 | 60% | 360 |
| [NP_006258.3](http://www.ncbi.nlm.nih.gov/entrez/query.fcgi?cmd=Retrieve&db=Protein&list_uids=150418007&dopt=GenPept&RID=T2X8VGF8012&log$=prottop&blast_rank=78) | RAN binding protein 2 [Homo sapiens] | [18.9](http://blast.ncbi.nlm.nih.gov/Blast.cgi#150418007%23150418007) | 52.0 | 70% | 360 |
| [NP_003878.1](http://www.ncbi.nlm.nih.gov/entrez/query.fcgi?cmd=Retrieve&db=Protein&list_uids=4502249&dopt=GenPept&RID=T2X8VGF8012&log$=prottop&blast_rank=79) | ArfGAP with SH3 domain, ankyrin repeat and PH domain 2 isoform a [Homo sapiens] | [18.9](http://blast.ncbi.nlm.nih.gov/Blast.cgi#4502249%234502249) | 18.9 | 50% | 360 |
| [NP_005045.2](http://www.ncbi.nlm.nih.gov/entrez/query.fcgi?cmd=Retrieve&db=Protein&list_uids=83267877&dopt=GenPept&RID=T2X8VGF8012&log$=prottop&blast_rank=80) | RANBP2-like and GRIP domain containing 5 isoform 1 [Homo sapiens] >ref|NP_001116835.1| RANBP2-like and GRIP domain containing 6 isoform 1 [Homo sapiens] | [18.9](http://blast.ncbi.nlm.nih.gov/Blast.cgi#83267877%2383267877) | 18.9 | 50% | 360 |
| [NP_001153047.1](http://www.ncbi.nlm.nih.gov/entrez/query.fcgi?cmd=Retrieve&db=Protein&list_uids=227430287&dopt=GenPept&RID=T2X8VGF8012&log$=prottop&blast_rank=81) | sodium channel, nonvoltage-gated 1 alpha isoform 3 [Homo sapiens] | [18.5](http://blast.ncbi.nlm.nih.gov/Blast.cgi#227430287%23227430287) | 18.5 | 40% | 482 |
| [NP_116203.2](http://www.ncbi.nlm.nih.gov/entrez/query.fcgi?cmd=Retrieve&db=Protein&list_uids=158186612&dopt=GenPept&RID=T2X8VGF8012&log$=prottop&blast_rank=82) | transmembrane protein 118 isoform 2 [Homo sapiens] | [18.5](http://blast.ncbi.nlm.nih.gov/Blast.cgi#158186612%23158186612) | 18.5 | 40% | 482 |
| [NP_060318.3](http://www.ncbi.nlm.nih.gov/entrez/query.fcgi?cmd=Retrieve&db=Protein&list_uids=157388908&dopt=GenPept&RID=T2X8VGF8012&log$=prottop&blast_rank=83) | hypothetical protein LOC54954 [Homo sapiens] | [18.5](http://blast.ncbi.nlm.nih.gov/Blast.cgi#157388908%23157388908) | 29.7 | 100% | 482 |
| [NP_001103373.1](http://www.ncbi.nlm.nih.gov/entrez/query.fcgi?cmd=Retrieve&db=Protein&list_uids=158186614&dopt=GenPept&RID=T2X8VGF8012&log$=prottop&blast_rank=84) | transmembrane protein 118 isoform 1 [Homo sapiens] | [18.5](http://blast.ncbi.nlm.nih.gov/Blast.cgi#158186614%23158186614) | 18.5 | 40% | 482 |
| [NP_114120.2](http://www.ncbi.nlm.nih.gov/entrez/query.fcgi?cmd=Retrieve&db=Protein&list_uids=123173752&dopt=GenPept&RID=T2X8VGF8012&log$=prottop&blast_rank=85) | synaptotagmin XIV-like [Homo sapiens] | [18.5](http://blast.ncbi.nlm.nih.gov/Blast.cgi#123173752%23123173752) | 18.5 | 70% | 482 |
| [NP_001153048.1](http://www.ncbi.nlm.nih.gov/entrez/query.fcgi?cmd=Retrieve&db=Protein&list_uids=227430289&dopt=GenPept&RID=T2X8VGF8012&log$=prottop&blast_rank=86) | sodium channel, nonvoltage-gated 1 alpha isoform 2 [Homo sapiens] | [18.5](http://blast.ncbi.nlm.nih.gov/Blast.cgi#227430289%23227430289) | 18.5 | 40% | 482 |
| [NP_057613.4](http://www.ncbi.nlm.nih.gov/entrez/query.fcgi?cmd=Retrieve&db=Protein&list_uids=117168245&dopt=GenPept&RID=T2X8VGF8012&log$=prottop&blast_rank=87) | ATPase, aminophospholipid transporter-like, Class I, type 8A, member 2 [Homo sapiens] | [18.5](http://blast.ncbi.nlm.nih.gov/Blast.cgi#117168245%23117168245) | 31.0 | 90% | 482 |
| [NP_001003788.1](http://www.ncbi.nlm.nih.gov/entrez/query.fcgi?cmd=Retrieve&db=Protein&list_uids=51242957&dopt=GenPept&RID=T2X8VGF8012&log$=prottop&blast_rank=88) | STE20-related kinase adaptor alpha isoform 4 [Homo sapiens] | [18.5](http://blast.ncbi.nlm.nih.gov/Blast.cgi#51242957%2351242957) | 18.5 | 40% | 482 |
| [NP_699166.2](http://www.ncbi.nlm.nih.gov/entrez/query.fcgi?cmd=Retrieve&db=Protein&list_uids=31982873&dopt=GenPept&RID=T2X8VGF8012&log$=prottop&blast_rank=89) | STE20-related kinase adaptor alpha isoform 3 [Homo sapiens] | [18.5](http://blast.ncbi.nlm.nih.gov/Blast.cgi#31982873%2331982873) | 18.5 | 40% | 482 |
| [NP_001137447.1](http://www.ncbi.nlm.nih.gov/entrez/query.fcgi?cmd=Retrieve&db=Protein&list_uids=221136785&dopt=GenPept&RID=T2X8VGF8012&log$=prottop&blast_rank=90) | upstream binding transcription factor, RNA polymerase I-like 1 [Homo sapiens] | [18.5](http://blast.ncbi.nlm.nih.gov/Blast.cgi#221136785%23221136785) | 18.5 | 80% | 482 |
| [NP_001027450.1](http://www.ncbi.nlm.nih.gov/entrez/query.fcgi?cmd=Retrieve&db=Protein&list_uids=73858575&dopt=GenPept&RID=T2X8VGF8012&log$=prottop&blast_rank=91) | prenyl protein peptidase RCE1 isoform 2 [Homo sapiens] | [18.5](http://blast.ncbi.nlm.nih.gov/Blast.cgi#73858575%2373858575) | 18.5 | 40% | 482 |
| [NP_055271.2](http://www.ncbi.nlm.nih.gov/entrez/query.fcgi?cmd=Retrieve&db=Protein&list_uids=21735596&dopt=GenPept&RID=T2X8VGF8012&log$=prottop&blast_rank=92) | programmed cell death 4 isoform 1 [Homo sapiens] | [18.5](http://blast.ncbi.nlm.nih.gov/Blast.cgi#21735596%2321735596) | 18.5 | 40% | 482 |
| [NP_001003787.1](http://www.ncbi.nlm.nih.gov/entrez/query.fcgi?cmd=Retrieve&db=Protein&list_uids=51242955&dopt=GenPept&RID=T2X8VGF8012&log$=prottop&blast_rank=93) | STE20-related kinase adaptor alpha isoform 1 [Homo sapiens] | [18.5](http://blast.ncbi.nlm.nih.gov/Blast.cgi#51242955%2351242955) | 18.5 | 40% | 482 |
| [NP_001338.2](http://www.ncbi.nlm.nih.gov/entrez/query.fcgi?cmd=Retrieve&db=Protein&list_uids=40806175&dopt=GenPept&RID=T2X8VGF8012&log$=prottop&blast_rank=94) | diacylglycerol kinase, theta [Homo sapiens] | [18.5](http://blast.ncbi.nlm.nih.gov/Blast.cgi#40806175%2340806175) | 18.5 | 60% | 482 |
| [NP_115958.2](http://www.ncbi.nlm.nih.gov/entrez/query.fcgi?cmd=Retrieve&db=Protein&list_uids=40556376&dopt=GenPept&RID=T2X8VGF8012&log$=prottop&blast_rank=95) | cytokine-like nuclear factor n-pac [Homo sapiens] | [18.5](http://blast.ncbi.nlm.nih.gov/Blast.cgi#40556376%2340556376) | 18.5 | 40% | 482 |
| [NP_001107611.1](http://www.ncbi.nlm.nih.gov/entrez/query.fcgi?cmd=Retrieve&db=Protein&list_uids=166706885&dopt=GenPept&RID=T2X8VGF8012&log$=prottop&blast_rank=96) | erythrocyte membrane protein band 4.9 isoform 3 [Homo sapiens] | [18.5](http://blast.ncbi.nlm.nih.gov/Blast.cgi#166706885%23166706885) | 18.5 | 40% | 482 |
| [NP_006709.2](http://www.ncbi.nlm.nih.gov/entrez/query.fcgi?cmd=Retrieve&db=Protein&list_uids=27894293&dopt=GenPept&RID=T2X8VGF8012&log$=prottop&blast_rank=97) | pleiomorphic adenoma gene-like 1 isoform 2 [Homo sapiens] >ref|NP_001074420.1| pleiomorphic adenoma gene-like 1 isoform 2 [Homo sapiens] >ref|NP_001074421.1| pleiomorphic adenoma gene-like 1 isoform 2 [Homo sapiens] >ref|NP_001074422.1| pleiomorphic adenoma gene-like 1 isoform 2 [Homo sapiens] >ref|NP_001074423.1| pleiomorphic adenoma gene-like 1 isoform 2 [Homo sapiens] | [18.5](http://blast.ncbi.nlm.nih.gov/Blast.cgi#27894293%2327894293) | 18.5 | 40% | 482 |
| [NP_003176.2](http://www.ncbi.nlm.nih.gov/entrez/query.fcgi?cmd=Retrieve&db=Protein&list_uids=110832843&dopt=GenPept&RID=T2X8VGF8012&log$=prottop&blast_rank=98) | TBP-associated factor 4 [Homo sapiens] | [18.5](http://blast.ncbi.nlm.nih.gov/Blast.cgi#110832843%23110832843) | 18.5 | 80% | 482 |
| [NP_001003786.1](http://www.ncbi.nlm.nih.gov/entrez/query.fcgi?cmd=Retrieve&db=Protein&list_uids=51242960&dopt=GenPept&RID=T2X8VGF8012&log$=prottop&blast_rank=99) | STE20-related kinase adaptor alpha isoform 2 [Homo sapiens] | [18.5](http://blast.ncbi.nlm.nih.gov/Blast.cgi#51242960%2351242960) | 18.5 | 40% | 482 |
| [NP_061893.2](http://www.ncbi.nlm.nih.gov/entrez/query.fcgi?cmd=Retrieve&db=Protein&list_uids=33563376&dopt=GenPept&RID=T2X8VGF8012&log$=prottop&blast_rank=100) | TBC1 domain family, member 16 [Homo sapiens] | [18.5](http://blast.ncbi.nlm.nih.gov/Blast.cgi#33563376%2333563376) | 31.0 | 60% | 482 |

| **Accession** | **Proteins with a match to PQGWLGW peptide** | **[Max score](http://blast.ncbi.nlm.nih.gov/Blast.cgi?CMD=Get&ALIGNMENTS=100&ALIGNMENT_VIEW=Pairwise&CDD_SEARCH_STATE=1&DATABASE_SORT=0&DESCRIPTIONS=100&ENTREZ_QUERY=txid9606 %5BORGN%5D&FIRST_QUERY_NUM=0&FORMAT_OBJECT=Alignment&FORMAT_PAGE_TARGET=&FORMAT_TYPE=HTML&GET_SEQUENCE=yes&I_THRESH=&MASK_CHAR=2&MASK_COLOR=1&NEW_DESIGN=on&NEW_VIEW=yes&NUM_OVERVIEW=100&OLD_BLAST=false&PAGE=Proteins&QUERY_INDEX=0&QUERY_NUMBER=0&RESULTS_PAGE_TARGET=&RID=T2ZED13D01S&SHOW_LINKOUT=yes&SHOW_OVERVIEW=yes&STEP_NUMBER=&WORD_SIZE=2&DISPLAY_SORT=1&HSP_SORT=1" \l "sort_mark)** | **[Total score](http://blast.ncbi.nlm.nih.gov/Blast.cgi?CMD=Get&ALIGNMENTS=100&ALIGNMENT_VIEW=Pairwise&CDD_SEARCH_STATE=1&DATABASE_SORT=0&DESCRIPTIONS=100&ENTREZ_QUERY=txid9606 %5BORGN%5D&FIRST_QUERY_NUM=0&FORMAT_OBJECT=Alignment&FORMAT_PAGE_TARGET=&FORMAT_TYPE=HTML&GET_SEQUENCE=yes&I_THRESH=&MASK_CHAR=2&MASK_COLOR=1&NEW_DESIGN=on&NEW_VIEW=yes&NUM_OVERVIEW=100&OLD_BLAST=false&PAGE=Proteins&QUERY_INDEX=0&QUERY_NUMBER=0&RESULTS_PAGE_TARGET=&RID=T2ZED13D01S&SHOW_LINKOUT=yes&SHOW_OVERVIEW=yes&STEP_NUMBER=&WORD_SIZE=2&DISPLAY_SORT=2&HSP_SORT=1" \l "sort_mark)** | **[Query coverage](http://blast.ncbi.nlm.nih.gov/Blast.cgi?CMD=Get&ALIGNMENTS=100&ALIGNMENT_VIEW=Pairwise&CDD_SEARCH_STATE=1&DATABASE_SORT=0&DESCRIPTIONS=100&ENTREZ_QUERY=txid9606 %5BORGN%5D&FIRST_QUERY_NUM=0&FORMAT_OBJECT=Alignment&FORMAT_PAGE_TARGET=&FORMAT_TYPE=HTML&GET_SEQUENCE=yes&I_THRESH=&MASK_CHAR=2&MASK_COLOR=1&NEW_DESIGN=on&NEW_VIEW=yes&NUM_OVERVIEW=100&OLD_BLAST=false&PAGE=Proteins&QUERY_INDEX=0&QUERY_NUMBER=0&RESULTS_PAGE_TARGET=&RID=T2ZED13D01S&SHOW_LINKOUT=yes&SHOW_OVERVIEW=yes&STEP_NUMBER=&WORD_SIZE=2&DISPLAY_SORT=4&HSP_SORT=0" \l "sort_mark)** | **[E value](http://blast.ncbi.nlm.nih.gov/Blast.cgi?CMD=Get&ALIGNMENTS=100&ALIGNMENT_VIEW=Pairwise&CDD_SEARCH_STATE=1&DATABASE_SORT=0&DESCRIPTIONS=100&ENTREZ_QUERY=txid9606 %5BORGN%5D&FIRST_QUERY_NUM=0&FORMAT_OBJECT=Alignment&FORMAT_PAGE_TARGET=&FORMAT_TYPE=HTML&GET_SEQUENCE=yes&I_THRESH=&MASK_CHAR=2&MASK_COLOR=1&NEW_DESIGN=on&NEW_VIEW=yes&NUM_OVERVIEW=100&OLD_BLAST=false&PAGE=Proteins&QUERY_INDEX=0&QUERY_NUMBER=0&RESULTS_PAGE_TARGET=&RID=T2ZED13D01S&SHOW_LINKOUT=yes&SHOW_OVERVIEW=yes&STEP_NUMBER=&WORD_SIZE=2&DISPLAY_SORT=0&HSP_SORT=0" \l "sort_mark)** |
| --- | --- | --- | --- | --- | --- |
| [XP_002347299.1](http://www.ncbi.nlm.nih.gov/entrez/query.fcgi?cmd=Retrieve&db=Protein&list_uids=239750143&dopt=GenPept&RID=T2ZED13D01S&log$=prottop&blast_rank=1) | PREDICTED: tripartite motif-containing 77 [Homo sapiens] >ref|XP_002344647.1| PREDICTED: tripartite motif-containing 77 [Homo sapiens] | [23.5](http://blast.ncbi.nlm.nih.gov/Blast.cgi#239750143%23239750143) | 23.5 | 85% | 9.9 |
| [NP_001139634.1](http://www.ncbi.nlm.nih.gov/entrez/query.fcgi?cmd=Retrieve&db=Protein&list_uids=225903455&dopt=GenPept&RID=T2ZED13D01S&log$=prottop&blast_rank=2) | tripartite motif-containing 77 [Homo sapiens] >ref|XP_002343148.1| PREDICTED: hypothetical protein XP_002343148 [Homo sapiens] | [23.5](http://blast.ncbi.nlm.nih.gov/Blast.cgi#225903455%23225903455) | 23.5 | 85% | 9.9 |
| [XP_002343003.1](http://www.ncbi.nlm.nih.gov/entrez/query.fcgi?cmd=Retrieve&db=Protein&list_uids=239743999&dopt=GenPept&RID=T2ZED13D01S&log$=prottop&blast_rank=3) | PREDICTED: hypothetical protein XP_002343003 [Homo sapiens] >ref|XP_002347155.1| PREDICTED: hypothetical protein XP_002347155 [Homo sapiens] >ref|XP_002344512.1| PREDICTED: hypothetical protein [Homo sapiens] | [22.7](http://blast.ncbi.nlm.nih.gov/Blast.cgi#239743999%23239743999) | 22.7 | 100% | 18 |
| [NP_036546.2](http://www.ncbi.nlm.nih.gov/entrez/query.fcgi?cmd=Retrieve&db=Protein&list_uids=19923790&dopt=GenPept&RID=T2ZED13D01S&log$=prottop&blast_rank=4) | rab3 GTPase-activating protein, non-catalytic subunit [Homo sapiens] | [22.3](http://blast.ncbi.nlm.nih.gov/Blast.cgi#19923790%2319923790) | 33.9 | 71% | 24 |
| [NP_858047.2](http://www.ncbi.nlm.nih.gov/entrez/query.fcgi?cmd=Retrieve&db=Protein&list_uids=119874215&dopt=GenPept&RID=T2ZED13D01S&log$=prottop&blast_rank=5) | vacuolar protein sorting 13B isoform 4 [Homo sapiens] | [21.8](http://blast.ncbi.nlm.nih.gov/Blast.cgi#119874215%23119874215) | 21.8 | 100% | 32 |
| [NP_056058.2](http://www.ncbi.nlm.nih.gov/entrez/query.fcgi?cmd=Retrieve&db=Protein&list_uids=35493725&dopt=GenPept&RID=T2ZED13D01S&log$=prottop&blast_rank=6) | vacuolar protein sorting 13B isoform 3 [Homo sapiens] | [21.8](http://blast.ncbi.nlm.nih.gov/Blast.cgi#35493725%2335493725) | 21.8 | 100% | 32 |
| [NP_689777.3](http://www.ncbi.nlm.nih.gov/entrez/query.fcgi?cmd=Retrieve&db=Protein&list_uids=35493701&dopt=GenPept&RID=T2ZED13D01S&log$=prottop&blast_rank=7) | vacuolar protein sorting 13B isoform 1 [Homo sapiens] | [21.8](http://blast.ncbi.nlm.nih.gov/Blast.cgi#35493701%2335493701) | 21.8 | 100% | 32 |
| [NP_060360.3](http://www.ncbi.nlm.nih.gov/entrez/query.fcgi?cmd=Retrieve&db=Protein&list_uids=35493713&dopt=GenPept&RID=T2ZED13D01S&log$=prottop&blast_rank=8) | vacuolar protein sorting 13B isoform 5 [Homo sapiens] | [21.8](http://blast.ncbi.nlm.nih.gov/Blast.cgi#35493713%2335493713) | 21.8 | 100% | 32 |
| [NP_997465.1](http://www.ncbi.nlm.nih.gov/entrez/query.fcgi?cmd=Retrieve&db=Protein&list_uids=46485773&dopt=GenPept&RID=T2ZED13D01S&log$=prottop&blast_rank=9) | syncytin 2 [Homo sapiens] | [21.8](http://blast.ncbi.nlm.nih.gov/Blast.cgi#46485773%2346485773) | 21.8 | 85% | 32 |
| [XP_002347839.1](http://www.ncbi.nlm.nih.gov/entrez/query.fcgi?cmd=Retrieve&db=Protein&list_uids=239751407&dopt=GenPept&RID=T2ZED13D01S&log$=prottop&blast_rank=10) | PREDICTED: hypothetical protein XP_002347839 [Homo sapiens] | [21.0](http://blast.ncbi.nlm.nih.gov/Blast.cgi#239751407%23239751407) | 21.0 | 71% | 58 |
| [NP_001013668.2](http://www.ncbi.nlm.nih.gov/entrez/query.fcgi?cmd=Retrieve&db=Protein&list_uids=71043642&dopt=GenPept&RID=T2ZED13D01S&log$=prottop&blast_rank=11) | hypothetical protein LOC388799 precursor [Homo sapiens] | [21.0](http://blast.ncbi.nlm.nih.gov/Blast.cgi#71043642%2371043642) | 21.0 | 85% | 58 |
| [NP_112200.2](http://www.ncbi.nlm.nih.gov/entrez/query.fcgi?cmd=Retrieve&db=Protein&list_uids=20070349&dopt=GenPept&RID=T2ZED13D01S&log$=prottop&blast_rank=12) | transmembrane protein 49 [Homo sapiens] | [21.0](http://blast.ncbi.nlm.nih.gov/Blast.cgi#20070349%2320070349) | 21.0 | 100% | 58 |
| [NP_001012989.2](http://www.ncbi.nlm.nih.gov/entrez/query.fcgi?cmd=Retrieve&db=Protein&list_uids=71043622&dopt=GenPept&RID=T2ZED13D01S&log$=prottop&blast_rank=13) | hypothetical protein LOC200232 precursor [Homo sapiens] | [21.0](http://blast.ncbi.nlm.nih.gov/Blast.cgi#71043622%2371043622) | 21.0 | 85% | 58 |
| [XP_002343156.1](http://www.ncbi.nlm.nih.gov/entrez/query.fcgi?cmd=Retrieve&db=Protein&list_uids=239744411&dopt=GenPept&RID=T2ZED13D01S&log$=prottop&blast_rank=14) | PREDICTED: hypothetical protein XP_002343156 [Homo sapiens] >ref|XP_002347294.1| PREDICTED: hypothetical protein XP_002347294 [Homo sapiens] >ref|XP_002344631.1| PREDICTED: hypothetical protein [Homo sapiens] | [20.6](http://blast.ncbi.nlm.nih.gov/Blast.cgi#239744411%23239744411) | 20.6 | 85% | 78 |
| [NP_000614.1](http://www.ncbi.nlm.nih.gov/entrez/query.fcgi?cmd=Retrieve&db=Protein&list_uids=4557359&dopt=GenPept&RID=T2ZED13D01S&log$=prottop&blast_rank=15) | bradykinin receptor B2 [Homo sapiens] | [20.6](http://blast.ncbi.nlm.nih.gov/Blast.cgi#4557359%234557359) | 20.6 | 100% | 78 |
| [NP_000253.1](http://www.ncbi.nlm.nih.gov/entrez/query.fcgi?cmd=Retrieve&db=Protein&list_uids=4557781&dopt=GenPept&RID=T2ZED13D01S&log$=prottop&blast_rank=16) | alpha-N-acetylgalactosaminidase precursor [Homo sapiens] | [20.6](http://blast.ncbi.nlm.nih.gov/Blast.cgi#4557781%234557781) | 20.6 | 100% | 78 |
| [XP_002342080.1](http://www.ncbi.nlm.nih.gov/entrez/query.fcgi?cmd=Retrieve&db=Protein&list_uids=239741061&dopt=GenPept&RID=T2ZED13D01S&log$=prottop&blast_rank=17) | PREDICTED: hypothetical protein XP_002342080 [Homo sapiens] >ref|XP_002347554.1| PREDICTED: hypothetical protein [Homo sapiens] | [20.2](http://blast.ncbi.nlm.nih.gov/Blast.cgi#239741061%23239741061) | 20.2 | 71% | 104 |
| [NP_036423.4](http://www.ncbi.nlm.nih.gov/entrez/query.fcgi?cmd=Retrieve&db=Protein&list_uids=134276943&dopt=GenPept&RID=T2ZED13D01S&log$=prottop&blast_rank=18) | separase [Homo sapiens] | [20.2](http://blast.ncbi.nlm.nih.gov/Blast.cgi#134276943%23134276943) | 20.2 | 71% | 104 |
| [NP_775908.2](http://www.ncbi.nlm.nih.gov/entrez/query.fcgi?cmd=Retrieve&db=Protein&list_uids=149274607&dopt=GenPept&RID=T2ZED13D01S&log$=prottop&blast_rank=19) | solute carrier family 25, member 41 [Homo sapiens] | [20.2](http://blast.ncbi.nlm.nih.gov/Blast.cgi#149274607%23149274607) | 20.2 | 71% | 104 |
| [XP_002345348.1](http://www.ncbi.nlm.nih.gov/entrez/query.fcgi?cmd=Retrieve&db=Protein&list_uids=239752708&dopt=GenPept&RID=T2ZED13D01S&log$=prottop&blast_rank=20) | PREDICTED: hypothetical protein [Homo sapiens] | [20.2](http://blast.ncbi.nlm.nih.gov/Blast.cgi#239752708%23239752708) | 20.2 | 71% | 104 |
| [NP_001010865.1](http://www.ncbi.nlm.nih.gov/entrez/query.fcgi?cmd=Retrieve&db=Protein&list_uids=58197552&dopt=GenPept&RID=T2ZED13D01S&log$=prottop&blast_rank=21) | essential meiotic endonuclease 1 homolog 2 [Homo sapiens] | [20.2](http://blast.ncbi.nlm.nih.gov/Blast.cgi#58197552%2358197552) | 20.2 | 71% | 104 |
| [NP_653080.1](http://www.ncbi.nlm.nih.gov/entrez/query.fcgi?cmd=Retrieve&db=Protein&list_uids=21493024&dopt=GenPept&RID=T2ZED13D01S&log$=prottop&blast_rank=22) | A kinase (PRKA) anchor protein 12 isoform 2 [Homo sapiens] | [20.2](http://blast.ncbi.nlm.nih.gov/Blast.cgi#21493024%2321493024) | 20.2 | 85% | 104 |
| [NP_005091.2](http://www.ncbi.nlm.nih.gov/entrez/query.fcgi?cmd=Retrieve&db=Protein&list_uids=21493022&dopt=GenPept&RID=T2ZED13D01S&log$=prottop&blast_rank=23) | A kinase (PRKA) anchor protein 12 isoform 1 [Homo sapiens] | [20.2](http://blast.ncbi.nlm.nih.gov/Blast.cgi#21493022%2321493022) | 20.2 | 85% | 104 |
| [NP_001161842.1](http://www.ncbi.nlm.nih.gov/entrez/query.fcgi?cmd=Retrieve&db=Protein&list_uids=270265835&dopt=GenPept&RID=T2ZED13D01S&log$=prottop&blast_rank=24) | cullin 7 isoform 1 [Homo sapiens] | [19.7](http://blast.ncbi.nlm.nih.gov/Blast.cgi#270265835%23270265835) | 19.7 | 57% | 140 |
| [XP_002348003.1](http://www.ncbi.nlm.nih.gov/entrez/query.fcgi?cmd=Retrieve&db=Protein&list_uids=239751791&dopt=GenPept&RID=T2ZED13D01S&log$=prottop&blast_rank=25) | PREDICTED: hypothetical protein [Homo sapiens] | [19.7](http://blast.ncbi.nlm.nih.gov/Blast.cgi#239751791%23239751791) | 19.7 | 85% | 140 |
| [XP_002343034.1](http://www.ncbi.nlm.nih.gov/entrez/query.fcgi?cmd=Retrieve&db=Protein&list_uids=239744106&dopt=GenPept&RID=T2ZED13D01S&log$=prottop&blast_rank=26) | PREDICTED: hypothetical protein XP_002343034 [Homo sapiens] >ref|XP_002347178.1| PREDICTED: hypothetical protein [Homo sapiens] >ref|XP_002344527.1| PREDICTED: hypothetical protein XP_002344527 [Homo sapiens] | [19.7](http://blast.ncbi.nlm.nih.gov/Blast.cgi#239744106%23239744106) | 19.7 | 57% | 140 |
| [XP_002342748.1](http://www.ncbi.nlm.nih.gov/entrez/query.fcgi?cmd=Retrieve&db=Protein&list_uids=239743053&dopt=GenPept&RID=T2ZED13D01S&log$=prottop&blast_rank=27) | PREDICTED: hypothetical protein XP_002342748 [Homo sapiens] | [19.7](http://blast.ncbi.nlm.nih.gov/Blast.cgi#239743053%23239743053) | 19.7 | 57% | 140 |
| [XP_002342387.1](http://www.ncbi.nlm.nih.gov/entrez/query.fcgi?cmd=Retrieve&db=Protein&list_uids=239741989&dopt=GenPept&RID=T2ZED13D01S&log$=prottop&blast_rank=28) | PREDICTED: hypothetical protein XP_002342387 [Homo sapiens] >ref|XP_002346551.1| PREDICTED: hypothetical protein XP_002346551 [Homo sapiens] >ref|XP_002345713.1| PREDICTED: hypothetical protein [Homo sapiens] | [19.7](http://blast.ncbi.nlm.nih.gov/Blast.cgi#239741989%23239741989) | 19.7 | 57% | 140 |
| [NP_001030014.2](http://www.ncbi.nlm.nih.gov/entrez/query.fcgi?cmd=Retrieve&db=Protein&list_uids=194018513&dopt=GenPept&RID=T2ZED13D01S&log$=prottop&blast_rank=29) | patched domain containing 3 [Homo sapiens] | [19.7](http://blast.ncbi.nlm.nih.gov/Blast.cgi#194018513%23194018513) | 19.7 | 57% | 140 |
| [NP_061985.2](http://www.ncbi.nlm.nih.gov/entrez/query.fcgi?cmd=Retrieve&db=Protein&list_uids=150417984&dopt=GenPept&RID=T2ZED13D01S&log$=prottop&blast_rank=30) | ATP-binding cassette, sub-family A, member 7 [Homo sapiens] | [19.7](http://blast.ncbi.nlm.nih.gov/Blast.cgi#150417984%23150417984) | 19.7 | 57% | 140 |
| [NP_001012302.2](http://www.ncbi.nlm.nih.gov/entrez/query.fcgi?cmd=Retrieve&db=Protein&list_uids=145611446&dopt=GenPept&RID=T2ZED13D01S&log$=prottop&blast_rank=31) | tumor protein p53 inducible protein 5 [Homo sapiens] | [19.7](http://blast.ncbi.nlm.nih.gov/Blast.cgi#145611446%23145611446) | 19.7 | 57% | 140 |
| [NP_849148.2](http://www.ncbi.nlm.nih.gov/entrez/query.fcgi?cmd=Retrieve&db=Protein&list_uids=218084958&dopt=GenPept&RID=T2ZED13D01S&log$=prottop&blast_rank=32) | anoctamin 4 [Homo sapiens] | [19.7](http://blast.ncbi.nlm.nih.gov/Blast.cgi#218084958%23218084958) | 19.7 | 57% | 140 |
| [NP_001012681.1](http://www.ncbi.nlm.nih.gov/entrez/query.fcgi?cmd=Retrieve&db=Protein&list_uids=61744479&dopt=GenPept&RID=T2ZED13D01S&log$=prottop&blast_rank=33) | solute carrier family 3, member 2 isoform d [Homo sapiens] | [19.7](http://blast.ncbi.nlm.nih.gov/Blast.cgi#61744479%2361744479) | 36.5 | 71% | 140 |
| [NP_113606.2](http://www.ncbi.nlm.nih.gov/entrez/query.fcgi?cmd=Retrieve&db=Protein&list_uids=156766084&dopt=GenPept&RID=T2ZED13D01S&log$=prottop&blast_rank=34) | transmembrane protein 16C [Homo sapiens] | [19.7](http://blast.ncbi.nlm.nih.gov/Blast.cgi#156766084%23156766084) | 19.7 | 57% | 140 |
| [NP_001073923.1](http://www.ncbi.nlm.nih.gov/entrez/query.fcgi?cmd=Retrieve&db=Protein&list_uids=122937307&dopt=GenPept&RID=T2ZED13D01S&log$=prottop&blast_rank=35) | acyl-CoA synthetase medium-chain family member 4 precursor [Homo sapiens] | [19.7](http://blast.ncbi.nlm.nih.gov/Blast.cgi#122937307%23122937307) | 19.7 | 100% | 140 |
| [NP_001012680.1](http://www.ncbi.nlm.nih.gov/entrez/query.fcgi?cmd=Retrieve&db=Protein&list_uids=61744477&dopt=GenPept&RID=T2ZED13D01S&log$=prottop&blast_rank=36) | solute carrier family 3, member 2 isoform b [Homo sapiens] | [19.7](http://blast.ncbi.nlm.nih.gov/Blast.cgi#61744477%2361744477) | 36.5 | 71% | 140 |
| [NP_056344.2](http://www.ncbi.nlm.nih.gov/entrez/query.fcgi?cmd=Retrieve&db=Protein&list_uids=118421087&dopt=GenPept&RID=T2ZED13D01S&log$=prottop&blast_rank=37) | monooxygenase, DBH-like 1 isoform 2 [Homo sapiens] | [19.7](http://blast.ncbi.nlm.nih.gov/Blast.cgi#118421087%23118421087) | 19.7 | 57% | 140 |
| [NP_001137252.1](http://www.ncbi.nlm.nih.gov/entrez/query.fcgi?cmd=Retrieve&db=Protein&list_uids=219555665&dopt=GenPept&RID=T2ZED13D01S&log$=prottop&blast_rank=38) | solute carrier family 25, member 39 isoform a [Homo sapiens] | [19.7](http://blast.ncbi.nlm.nih.gov/Blast.cgi#219555665%23219555665) | 19.7 | 57% | 140 |
| [NP_002385.3](http://www.ncbi.nlm.nih.gov/entrez/query.fcgi?cmd=Retrieve&db=Protein&list_uids=65506891&dopt=GenPept&RID=T2ZED13D01S&log$=prottop&blast_rank=39) | solute carrier family 3, member 2 isoform c [Homo sapiens] | [19.7](http://blast.ncbi.nlm.nih.gov/Blast.cgi#65506891%2365506891) | 36.5 | 71% | 140 |
| [NP_001012682.1](http://www.ncbi.nlm.nih.gov/entrez/query.fcgi?cmd=Retrieve&db=Protein&list_uids=61744481&dopt=GenPept&RID=T2ZED13D01S&log$=prottop&blast_rank=40) | solute carrier family 3, member 2 isoform e [Homo sapiens] | [19.7](http://blast.ncbi.nlm.nih.gov/Blast.cgi#61744481%2361744481) | 36.5 | 71% | 140 |
| [NP_001012679.1](http://www.ncbi.nlm.nih.gov/entrez/query.fcgi?cmd=Retrieve&db=Protein&list_uids=61744475&dopt=GenPept&RID=T2ZED13D01S&log$=prottop&blast_rank=41) | solute carrier family 3, member 2 isoform a [Homo sapiens] | [19.7](http://blast.ncbi.nlm.nih.gov/Blast.cgi#61744475%2361744475) | 36.5 | 71% | 140 |
| [NP_055688.1](http://www.ncbi.nlm.nih.gov/entrez/query.fcgi?cmd=Retrieve&db=Protein&list_uids=7661996&dopt=GenPept&RID=T2ZED13D01S&log$=prottop&blast_rank=42) | lysophosphatidylglycerol acyltransferase 1 [Homo sapiens] | [19.7](http://blast.ncbi.nlm.nih.gov/Blast.cgi#7661996%237661996) | 19.7 | 57% | 140 |
| [NP_057438.3](http://www.ncbi.nlm.nih.gov/entrez/query.fcgi?cmd=Retrieve&db=Protein&list_uids=39777594&dopt=GenPept&RID=T2ZED13D01S&log$=prottop&blast_rank=43) | solute carrier organic anion transporter family member 4A1 [Homo sapiens] | [19.7](http://blast.ncbi.nlm.nih.gov/Blast.cgi#39777594%2339777594) | 19.7 | 57% | 140 |
| [NP_001013269.1](http://www.ncbi.nlm.nih.gov/entrez/query.fcgi?cmd=Retrieve&db=Protein&list_uids=61744483&dopt=GenPept&RID=T2ZED13D01S&log$=prottop&blast_rank=44) | solute carrier family 3, member 2 isoform f [Homo sapiens] | [19.7](http://blast.ncbi.nlm.nih.gov/Blast.cgi#61744483%2361744483) | 36.5 | 71% | 140 |
| [NP_057100.1](http://www.ncbi.nlm.nih.gov/entrez/query.fcgi?cmd=Retrieve&db=Protein&list_uids=7706306&dopt=GenPept&RID=T2ZED13D01S&log$=prottop&blast_rank=45) | solute carrier family 25, member 39 isoform b [Homo sapiens] | [19.7](http://blast.ncbi.nlm.nih.gov/Blast.cgi#7706306%237706306) | 19.7 | 57% | 140 |
| [NP_004637.1](http://www.ncbi.nlm.nih.gov/entrez/query.fcgi?cmd=Retrieve&db=Protein&list_uids=4758822&dopt=GenPept&RID=T2ZED13D01S&log$=prottop&blast_rank=46) | nephrin precursor [Homo sapiens] | [19.7](http://blast.ncbi.nlm.nih.gov/Blast.cgi#4758822%234758822) | 19.7 | 57% | 140 |
| [NP_055595.2](http://www.ncbi.nlm.nih.gov/entrez/query.fcgi?cmd=Retrieve&db=Protein&list_uids=41872646&dopt=GenPept&RID=T2ZED13D01S&log$=prottop&blast_rank=47) | cullin 7 isoform 2 [Homo sapiens] | [19.7](http://blast.ncbi.nlm.nih.gov/Blast.cgi#41872646%2341872646) | 19.7 | 57% | 140 |
| [XP_496693.4](http://www.ncbi.nlm.nih.gov/entrez/query.fcgi?cmd=Retrieve&db=Protein&list_uids=169166418&dopt=GenPept&RID=T2ZED13D01S&log$=prottop&blast_rank=48) | PREDICTED: similar to COMM domain containing 5 [Homo sapiens] >ref|XP_941557.3| PREDICTED: similar to COMM domain containing 5 [Homo sapiens] >ref|XP_001714919.1| PREDICTED: similar to COMM domain containing 5 [Homo sapiens] | [19.3](http://blast.ncbi.nlm.nih.gov/Blast.cgi#169166418%23169166418) | 19.3 | 85% | 188 |
| [NP_001106853.1](http://www.ncbi.nlm.nih.gov/entrez/query.fcgi?cmd=Retrieve&db=Protein&list_uids=164607133&dopt=GenPept&RID=T2ZED13D01S&log$=prottop&blast_rank=49) | fer-1-like 5 isoform 2 [Homo sapiens] | [19.3](http://blast.ncbi.nlm.nih.gov/Blast.cgi#164607133%23164607133) | 37.3 | 100% | 188 |
| [NP_653216.2](http://www.ncbi.nlm.nih.gov/entrez/query.fcgi?cmd=Retrieve&db=Protein&list_uids=281306838&dopt=GenPept&RID=T2ZED13D01S&log$=prottop&blast_rank=50) | transmembrane and immunoglobulin domain containing 2 isoform 1 precursor [Homo sapiens] | [19.3](http://blast.ncbi.nlm.nih.gov/Blast.cgi#281306838%23281306838) | 19.3 | 100% | 188 |
| [NP_055764.2](http://www.ncbi.nlm.nih.gov/entrez/query.fcgi?cmd=Retrieve&db=Protein&list_uids=55741675&dopt=GenPept&RID=T2ZED13D01S&log$=prottop&blast_rank=51) | hypothetical protein LOC22889 [Homo sapiens] | [19.3](http://blast.ncbi.nlm.nih.gov/Blast.cgi#55741675%2355741675) | 19.3 | 85% | 188 |
| [NP_001162597.1](http://www.ncbi.nlm.nih.gov/entrez/query.fcgi?cmd=Retrieve&db=Protein&list_uids=281306840&dopt=GenPept&RID=T2ZED13D01S&log$=prottop&blast_rank=52) | transmembrane and immunoglobulin domain containing 2 isoform 2 precursor [Homo sapiens] | [19.3](http://blast.ncbi.nlm.nih.gov/Blast.cgi#281306840%23281306840) | 19.3 | 100% | 188 |
| [NP_006276.2](http://www.ncbi.nlm.nih.gov/entrez/query.fcgi?cmd=Retrieve&db=Protein&list_uids=66932999&dopt=GenPept&RID=T2ZED13D01S&log$=prottop&blast_rank=53) | testis-specific protein kinase 1 [Homo sapiens] | [18.9](http://blast.ncbi.nlm.nih.gov/Blast.cgi#66932999%2366932999) | 18.9 | 100% | 252 |
| [NP_002297.2](http://www.ncbi.nlm.nih.gov/entrez/query.fcgi?cmd=Retrieve&db=Protein&list_uids=115430223&dopt=GenPept&RID=T2ZED13D01S&log$=prottop&blast_rank=54) | galectin 3 [Homo sapiens] | [18.9](http://blast.ncbi.nlm.nih.gov/Blast.cgi#115430223%23115430223) | 18.9 | 100% | 252 |
| [NP_003384.2](http://www.ncbi.nlm.nih.gov/entrez/query.fcgi?cmd=Retrieve&db=Protein&list_uids=110735437&dopt=GenPept&RID=T2ZED13D01S&log$=prottop&blast_rank=55) | wingless-type MMTV integration site family, member 8B precursor [Homo sapiens] | [18.9](http://blast.ncbi.nlm.nih.gov/Blast.cgi#110735437%23110735437) | 18.9 | 85% | 252 |
| [NP_055927.2](http://www.ncbi.nlm.nih.gov/entrez/query.fcgi?cmd=Retrieve&db=Protein&list_uids=112363080&dopt=GenPept&RID=T2ZED13D01S&log$=prottop&blast_rank=56) | microtubule associated serine/threonine kinase 2 [Homo sapiens] | [18.9](http://blast.ncbi.nlm.nih.gov/Blast.cgi#112363080%23112363080) | 18.9 | 85% | 252 |
| [XP_002347495.1](http://www.ncbi.nlm.nih.gov/entrez/query.fcgi?cmd=Retrieve&db=Protein&list_uids=239750745&dopt=GenPept&RID=T2ZED13D01S&log$=prottop&blast_rank=57) | PREDICTED: hypothetical protein XP_002347495 [Homo sapiens] >ref|XP_002344852.1| PREDICTED: similar to pre-B lymphocyte gene 2 [Homo sapiens] | [18.5](http://blast.ncbi.nlm.nih.gov/Blast.cgi#239750745%23239750745) | 18.5 | 85% | 338 |
| [NP_064506.3](http://www.ncbi.nlm.nih.gov/entrez/query.fcgi?cmd=Retrieve&db=Protein&list_uids=238859593&dopt=GenPept&RID=T2ZED13D01S&log$=prottop&blast_rank=58) | UDP-glucose ceramide glucosyltransferase-like 2 precursor [Homo sapiens] | [18.5](http://blast.ncbi.nlm.nih.gov/Blast.cgi#238859593%23238859593) | 18.5 | 100% | 338 |
| [XP_002347494.1](http://www.ncbi.nlm.nih.gov/entrez/query.fcgi?cmd=Retrieve&db=Protein&list_uids=239750752&dopt=GenPept&RID=T2ZED13D01S&log$=prottop&blast_rank=59) | PREDICTED: hypothetical protein XP_002347494 [Homo sapiens] >ref|XP_002344851.1| PREDICTED: similar to pre-B lymphocyte gene 2 [Homo sapiens] | [18.5](http://blast.ncbi.nlm.nih.gov/Blast.cgi#239750752%23239750752) | 18.5 | 85% | 338 |
| [NP_000426.2](http://www.ncbi.nlm.nih.gov/entrez/query.fcgi?cmd=Retrieve&db=Protein&list_uids=134244285&dopt=GenPept&RID=T2ZED13D01S&log$=prottop&blast_rank=60) | Notch homolog 3 precursor [Homo sapiens] | [18.5](http://blast.ncbi.nlm.nih.gov/Blast.cgi#134244285%23134244285) | 18.5 | 85% | 338 |
| [NP_001015072.2](http://www.ncbi.nlm.nih.gov/entrez/query.fcgi?cmd=Retrieve&db=Protein&list_uids=146231966&dopt=GenPept&RID=T2ZED13D01S&log$=prottop&blast_rank=61) | inactive Ufm1-specific protease 1 [Homo sapiens] | [18.5](http://blast.ncbi.nlm.nih.gov/Blast.cgi#146231966%23146231966) | 18.5 | 71% | 338 |
| [XP_002345235.1](http://www.ncbi.nlm.nih.gov/entrez/query.fcgi?cmd=Retrieve&db=Protein&list_uids=239757286&dopt=GenPept&RID=T2ZED13D01S&log$=prottop&blast_rank=62) | PREDICTED: similar to Fc fragment of IgG binding protein [Homo sapiens] | [18.0](http://blast.ncbi.nlm.nih.gov/Blast.cgi#239757286%23239757286) | 18.0 | 57% | 453 |
| [XP_002348190.1](http://www.ncbi.nlm.nih.gov/entrez/query.fcgi?cmd=Retrieve&db=Protein&list_uids=239752280&dopt=GenPept&RID=T2ZED13D01S&log$=prottop&blast_rank=63) | PREDICTED: similar to family with sequence similarity 48, member B2 [Homo sapiens] | [18.0](http://blast.ncbi.nlm.nih.gov/Blast.cgi#239752280%23239752280) | 18.0 | 57% | 453 |
| [XP_002344225.1](http://www.ncbi.nlm.nih.gov/entrez/query.fcgi?cmd=Retrieve&db=Protein&list_uids=239746447&dopt=GenPept&RID=T2ZED13D01S&log$=prottop&blast_rank=64) | PREDICTED: hypothetical protein XP_002344225 [Homo sapiens] >ref|XP_002348061.1| PREDICTED: similar to mCG146261 [Homo sapiens] >ref|XP_002345439.1| PREDICTED: similar to mCG146261 [Homo sapiens] | [18.0](http://blast.ncbi.nlm.nih.gov/Blast.cgi#239746447%23239746447) | 18.0 | 85% | 453 |
| [NP_001138483.1](http://www.ncbi.nlm.nih.gov/entrez/query.fcgi?cmd=Retrieve&db=Protein&list_uids=222446620&dopt=GenPept&RID=T2ZED13D01S&log$=prottop&blast_rank=65) | hypothetical protein LOC342346 [Homo sapiens] | [18.0](http://blast.ncbi.nlm.nih.gov/Blast.cgi#222446620%23222446620) | 18.0 | 57% | 453 |
| [NP_001129705.1](http://www.ncbi.nlm.nih.gov/entrez/query.fcgi?cmd=Retrieve&db=Protein&list_uids=210032463&dopt=GenPept&RID=T2ZED13D01S&log$=prottop&blast_rank=66) | family with sequence similarity 48, member B2 [Homo sapiens] | [18.0](http://blast.ncbi.nlm.nih.gov/Blast.cgi#210032463%23210032463) | 18.0 | 57% | 453 |
| [NP_001127836.1](http://www.ncbi.nlm.nih.gov/entrez/query.fcgi?cmd=Retrieve&db=Protein&list_uids=197276600&dopt=GenPept&RID=T2ZED13D01S&log$=prottop&blast_rank=67) | microtubule-associated protein 4 isoform 4 [Homo sapiens] | [18.0](http://blast.ncbi.nlm.nih.gov/Blast.cgi#197276600%23197276600) | 18.0 | 57% | 453 |
| [XP_001722870.1](http://www.ncbi.nlm.nih.gov/entrez/query.fcgi?cmd=Retrieve&db=Protein&list_uids=169215350&dopt=GenPept&RID=T2ZED13D01S&log$=prottop&blast_rank=68) | PREDICTED: hypothetical protein [Homo sapiens] | [18.0](http://blast.ncbi.nlm.nih.gov/Blast.cgi#169215350%23169215350) | 18.0 | 57% | 453 |
| [XP_002345125.1](http://www.ncbi.nlm.nih.gov/entrez/query.fcgi?cmd=Retrieve&db=Protein&list_uids=239756966&dopt=GenPept&RID=T2ZED13D01S&log$=prottop&blast_rank=69) | PREDICTED: similar to arg tyrosine kinase [Homo sapiens] | [18.0](http://blast.ncbi.nlm.nih.gov/Blast.cgi#239756966%23239756966) | 18.0 | 57% | 453 |
| [NP_001368.2](http://www.ncbi.nlm.nih.gov/entrez/query.fcgi?cmd=Retrieve&db=Protein&list_uids=283806679&dopt=GenPept&RID=T2ZED13D01S&log$=prottop&blast_rank=70) | dynein, cytoplasmic 2, heavy chain 1 isoform 1 [Homo sapiens] | [18.0](http://blast.ncbi.nlm.nih.gov/Blast.cgi#283806679%23283806679) | 18.0 | 57% | 453 |
| [NP_002438.2](http://www.ncbi.nlm.nih.gov/entrez/query.fcgi?cmd=Retrieve&db=Protein&list_uids=153946393&dopt=GenPept&RID=T2ZED13D01S&log$=prottop&blast_rank=71) | macrophage stimulating 1 receptor precursor [Homo sapiens] | [18.0](http://blast.ncbi.nlm.nih.gov/Blast.cgi#153946393%23153946393) | 18.0 | 57% | 453 |
| [NP_001401.2](http://www.ncbi.nlm.nih.gov/entrez/query.fcgi?cmd=Retrieve&db=Protein&list_uids=145701025&dopt=GenPept&RID=T2ZED13D01S&log$=prottop&blast_rank=72) | multiple EGF-like-domains 8 [Homo sapiens] | [18.0](http://blast.ncbi.nlm.nih.gov/Blast.cgi#145701025%23145701025) | 33.5 | 85% | 453 |
| [NP_940857.2](http://www.ncbi.nlm.nih.gov/entrez/query.fcgi?cmd=Retrieve&db=Protein&list_uids=134031945&dopt=GenPept&RID=T2ZED13D01S&log$=prottop&blast_rank=73) | SCO-spondin precursor [Homo sapiens] | [18.0](http://blast.ncbi.nlm.nih.gov/Blast.cgi#134031945%23134031945) | 91.4 | 100% | 453 |
| [XP_001726340.1](http://www.ncbi.nlm.nih.gov/entrez/query.fcgi?cmd=Retrieve&db=Protein&list_uids=169215453&dopt=GenPept&RID=T2ZED13D01S&log$=prottop&blast_rank=74) | PREDICTED: hypothetical protein [Homo sapiens] | [18.0](http://blast.ncbi.nlm.nih.gov/Blast.cgi#169215453%23169215453) | 18.0 | 57% | 453 |
| [NP_003676.2](http://www.ncbi.nlm.nih.gov/entrez/query.fcgi?cmd=Retrieve&db=Protein&list_uids=154355000&dopt=GenPept&RID=T2ZED13D01S&log$=prottop&blast_rank=75) | KH-type splicing regulatory protein [Homo sapiens] | [18.0](http://blast.ncbi.nlm.nih.gov/Blast.cgi#154355000%23154355000) | 18.0 | 57% | 453 |
| [NP_937986.1](http://www.ncbi.nlm.nih.gov/entrez/query.fcgi?cmd=Retrieve&db=Protein&list_uids=38201702&dopt=GenPept&RID=T2ZED13D01S&log$=prottop&blast_rank=76) | telomerase reverse transcriptase isoform 2 [Homo sapiens] | [18.0](http://blast.ncbi.nlm.nih.gov/Blast.cgi#38201702%2338201702) | 18.0 | 57% | 453 |
| [NP_001363.2](http://www.ncbi.nlm.nih.gov/entrez/query.fcgi?cmd=Retrieve&db=Protein&list_uids=114155133&dopt=GenPept&RID=T2ZED13D01S&log$=prottop&blast_rank=77) | dynein, axonemal, heavy chain 9 isoform 2 [Homo sapiens] | [18.0](http://blast.ncbi.nlm.nih.gov/Blast.cgi#114155133%23114155133) | 18.0 | 57% | 453 |
| [NP_004653.2](http://www.ncbi.nlm.nih.gov/entrez/query.fcgi?cmd=Retrieve&db=Protein&list_uids=114155135&dopt=GenPept&RID=T2ZED13D01S&log$=prottop&blast_rank=78) | dynein, axonemal, heavy chain 9 isoform 1 [Homo sapiens] | [18.0](http://blast.ncbi.nlm.nih.gov/Blast.cgi#114155135%23114155135) | 18.0 | 57% | 453 |
| [NP_001073932.1](http://www.ncbi.nlm.nih.gov/entrez/query.fcgi?cmd=Retrieve&db=Protein&list_uids=122937398&dopt=GenPept&RID=T2ZED13D01S&log$=prottop&blast_rank=79) | dynein, cytoplasmic 2, heavy chain 1 isoform 2 [Homo sapiens] | [18.0](http://blast.ncbi.nlm.nih.gov/Blast.cgi#122937398%23122937398) | 18.0 | 57% | 453 |
| [NP_006030.2](http://www.ncbi.nlm.nih.gov/entrez/query.fcgi?cmd=Retrieve&db=Protein&list_uids=110624774&dopt=GenPept&RID=T2ZED13D01S&log$=prottop&blast_rank=80) | mannose receptor, C type 2 [Homo sapiens] | [18.0](http://blast.ncbi.nlm.nih.gov/Blast.cgi#110624774%23110624774) | 32.2 | 85% | 453 |
| [NP_775931.3](http://www.ncbi.nlm.nih.gov/entrez/query.fcgi?cmd=Retrieve&db=Protein&list_uids=111185957&dopt=GenPept&RID=T2ZED13D01S&log$=prottop&blast_rank=81) | downstream of tyrosine kinase 7 isoform 1 [Homo sapiens] | [18.0](http://blast.ncbi.nlm.nih.gov/Blast.cgi#111185957%23111185957) | 18.0 | 85% | 453 |
| [NP_003768.2](http://www.ncbi.nlm.nih.gov/entrez/query.fcgi?cmd=Retrieve&db=Protein&list_uids=51479173&dopt=GenPept&RID=T2ZED13D01S&log$=prottop&blast_rank=82) | dynein, axonemal, heavy chain 11 [Homo sapiens] | [18.0](http://blast.ncbi.nlm.nih.gov/Blast.cgi#51479173%2351479173) | 18.0 | 57% | 453 |
| [NP_065170.2](http://www.ncbi.nlm.nih.gov/entrez/query.fcgi?cmd=Retrieve&db=Protein&list_uids=194328779&dopt=GenPept&RID=T2ZED13D01S&log$=prottop&blast_rank=83) | aspartate beta-hydroxylase domain containing 2 [Homo sapiens] | [18.0](http://blast.ncbi.nlm.nih.gov/Blast.cgi#194328779%23194328779) | 18.0 | 57% | 453 |
| [NP_002366.2](http://www.ncbi.nlm.nih.gov/entrez/query.fcgi?cmd=Retrieve&db=Protein&list_uids=47519639&dopt=GenPept&RID=T2ZED13D01S&log$=prottop&blast_rank=84) | microtubule-associated protein 4 isoform 1 [Homo sapiens] | [18.0](http://blast.ncbi.nlm.nih.gov/Blast.cgi#47519639%2347519639) | 18.0 | 57% | 453 |
| [NP_663696.1](http://www.ncbi.nlm.nih.gov/entrez/query.fcgi?cmd=Retrieve&db=Protein&list_uids=22035682&dopt=GenPept&RID=T2ZED13D01S&log$=prottop&blast_rank=85) | DBF4 homolog B isoform 1 [Homo sapiens] | [18.0](http://blast.ncbi.nlm.nih.gov/Blast.cgi#22035682%2322035682) | 18.0 | 57% | 453 |
| [NP_203700.1](http://www.ncbi.nlm.nih.gov/entrez/query.fcgi?cmd=Retrieve&db=Protein&list_uids=15890088&dopt=GenPept&RID=T2ZED13D01S&log$=prottop&blast_rank=86) | type IV collagen alpha 5 isoform 3 precursor [Homo sapiens] | [18.0](http://blast.ncbi.nlm.nih.gov/Blast.cgi#15890088%2315890088) | 18.0 | 57% | 453 |
| [NP_003893.2](http://www.ncbi.nlm.nih.gov/entrez/query.fcgi?cmd=Retrieve&db=Protein&list_uids=17402900&dopt=GenPept&RID=T2ZED13D01S&log$=prottop&blast_rank=87) | far upstream element-binding protein [Homo sapiens] | [18.0](http://blast.ncbi.nlm.nih.gov/Blast.cgi#17402900%2317402900) | 18.0 | 57% | 453 |
| [NP_057063.2](http://www.ncbi.nlm.nih.gov/entrez/query.fcgi?cmd=Retrieve&db=Protein&list_uids=28558969&dopt=GenPept&RID=T2ZED13D01S&log$=prottop&blast_rank=88) | mediator complex subunit 23 isoform b [Homo sapiens] | [18.0](http://blast.ncbi.nlm.nih.gov/Blast.cgi#28558969%2328558969) | 18.0 | 57% | 453 |
| [NP_149163.2](http://www.ncbi.nlm.nih.gov/entrez/query.fcgi?cmd=Retrieve&db=Protein&list_uids=15149474&dopt=GenPept&RID=T2ZED13D01S&log$=prottop&blast_rank=89) | ATP-binding cassette, sub-family C, member 11 isoform a [Homo sapiens] >ref|NP_115972.2| ATP-binding cassette, sub-family C, member 11 isoform a [Homo sapiens] | [18.0](http://blast.ncbi.nlm.nih.gov/Blast.cgi#15149474%2315149474) | 18.0 | 57% | 453 |
| [NP_660187.1](http://www.ncbi.nlm.nih.gov/entrez/query.fcgi?cmd=Retrieve&db=Protein&list_uids=21729876&dopt=GenPept&RID=T2ZED13D01S&log$=prottop&blast_rank=90) | ATP-binding cassette, sub-family C, member 11 isoform b [Homo sapiens] | [18.0](http://blast.ncbi.nlm.nih.gov/Blast.cgi#21729876%2321729876) | 18.0 | 57% | 453 |
| [NP_963918.1](http://www.ncbi.nlm.nih.gov/entrez/query.fcgi?cmd=Retrieve&db=Protein&list_uids=42516565&dopt=GenPept&RID=T2ZED13D01S&log$=prottop&blast_rank=91) | ubiquitin specific protease 33 isoform 2 [Homo sapiens] | [18.0](http://blast.ncbi.nlm.nih.gov/Blast.cgi#42516565%2342516565) | 18.0 | 57% | 453 |
| [NP_055832.3](http://www.ncbi.nlm.nih.gov/entrez/query.fcgi?cmd=Retrieve&db=Protein&list_uids=42516567&dopt=GenPept&RID=T2ZED13D01S&log$=prottop&blast_rank=92) | ubiquitin specific protease 33 isoform 1 [Homo sapiens] | [18.0](http://blast.ncbi.nlm.nih.gov/Blast.cgi#42516567%2342516567) | 18.0 | 57% | 453 |
| [NP_963920.1](http://www.ncbi.nlm.nih.gov/entrez/query.fcgi?cmd=Retrieve&db=Protein&list_uids=42516561&dopt=GenPept&RID=T2ZED13D01S&log$=prottop&blast_rank=93) | ubiquitin specific protease 33 isoform 3 [Homo sapiens] | [18.0](http://blast.ncbi.nlm.nih.gov/Blast.cgi#42516561%2342516561) | 18.0 | 57% | 453 |
| [NP_006862.2](http://www.ncbi.nlm.nih.gov/entrez/query.fcgi?cmd=Retrieve&db=Protein&list_uids=40254844&dopt=GenPept&RID=T2ZED13D01S&log$=prottop&blast_rank=94) | receptor-interacting serine-threonine kinase 3 [Homo sapiens] | [18.0](http://blast.ncbi.nlm.nih.gov/Blast.cgi#40254844%2340254844) | 32.7 | 57% | 453 |
| [NP_064505.1](http://www.ncbi.nlm.nih.gov/entrez/query.fcgi?cmd=Retrieve&db=Protein&list_uids=9910280&dopt=GenPept&RID=T2ZED13D01S&log$=prottop&blast_rank=95) | UDP-glucose ceramide glucosyltransferase-like 1 precursor [Homo sapiens] | [18.0](http://blast.ncbi.nlm.nih.gov/Blast.cgi#9910280%239910280) | 18.0 | 100% | 453 |
| [NP_203699.1](http://www.ncbi.nlm.nih.gov/entrez/query.fcgi?cmd=Retrieve&db=Protein&list_uids=15890086&dopt=GenPept&RID=T2ZED13D01S&log$=prottop&blast_rank=96) | type IV collagen alpha 5 isoform 2 precursor [Homo sapiens] | [18.0](http://blast.ncbi.nlm.nih.gov/Blast.cgi#15890086%2315890086) | 18.0 | 57% | 453 |
| [NP_653337.1](http://www.ncbi.nlm.nih.gov/entrez/query.fcgi?cmd=Retrieve&db=Protein&list_uids=21396487&dopt=GenPept&RID=T2ZED13D01S&log$=prottop&blast_rank=97) | hypothetical protein LOC55471 isoform 1 [Homo sapiens] | [18.0](http://blast.ncbi.nlm.nih.gov/Blast.cgi#21396487%2321396487) | 18.0 | 57% | 453 |
| [NP_940910.1](http://www.ncbi.nlm.nih.gov/entrez/query.fcgi?cmd=Retrieve&db=Protein&list_uids=38348332&dopt=GenPept&RID=T2ZED13D01S&log$=prottop&blast_rank=98) | killer cell lectin-like receptor subfamily G, member 2 [Homo sapiens] | [18.0](http://blast.ncbi.nlm.nih.gov/Blast.cgi#38348332%2338348332) | 18.0 | 57% | 453 |
| [NP_001077415.1](http://www.ncbi.nlm.nih.gov/entrez/query.fcgi?cmd=Retrieve&db=Protein&list_uids=145701028&dopt=GenPept&RID=T2ZED13D01S&log$=prottop&blast_rank=99) | hypothetical protein LOC55471 isoform 3 [Homo sapiens] | [18.0](http://blast.ncbi.nlm.nih.gov/Blast.cgi#145701028%23145701028) | 18.0 | 57% | 453 |
| [NP_077001.1](http://www.ncbi.nlm.nih.gov/entrez/query.fcgi?cmd=Retrieve&db=Protein&list_uids=13129100&dopt=GenPept&RID=T2ZED13D01S&log$=prottop&blast_rank=100) | dCTP pyrophosphatase 1 [Homo sapiens] | [18.0](http://blast.ncbi.nlm.nih.gov/Blast.cgi#13129100%2313129100) | 18.0 | 57% | 453 |

| **Accession** | **Proteins with a match to GTVEPD peptide** | **[Max score](http://blast.ncbi.nlm.nih.gov/Blast.cgi?CMD=Get&ALIGNMENTS=100&ALIGNMENT_VIEW=Pairwise&CDD_SEARCH_STATE=1&DATABASE_SORT=0&DESCRIPTIONS=100&ENTREZ_QUERY=txid9606 %5BORGN%5D&FIRST_QUERY_NUM=0&FORMAT_OBJECT=Alignment&FORMAT_PAGE_TARGET=&FORMAT_TYPE=HTML&GET_SEQUENCE=yes&I_THRESH=&MASK_CHAR=2&MASK_COLOR=1&NEW_DESIGN=on&NEW_VIEW=yes&NUM_OVERVIEW=100&OLD_BLAST=false&PAGE=Proteins&QUERY_INDEX=0&QUERY_NUMBER=0&RESULTS_PAGE_TARGET=&RID=T2ZJKRC5012&SHOW_LINKOUT=yes&SHOW_OVERVIEW=yes&STEP_NUMBER=&WORD_SIZE=2&DISPLAY_SORT=1&HSP_SORT=1" \l "sort_mark)** | **[Total score](http://blast.ncbi.nlm.nih.gov/Blast.cgi?CMD=Get&ALIGNMENTS=100&ALIGNMENT_VIEW=Pairwise&CDD_SEARCH_STATE=1&DATABASE_SORT=0&DESCRIPTIONS=100&ENTREZ_QUERY=txid9606 %5BORGN%5D&FIRST_QUERY_NUM=0&FORMAT_OBJECT=Alignment&FORMAT_PAGE_TARGET=&FORMAT_TYPE=HTML&GET_SEQUENCE=yes&I_THRESH=&MASK_CHAR=2&MASK_COLOR=1&NEW_DESIGN=on&NEW_VIEW=yes&NUM_OVERVIEW=100&OLD_BLAST=false&PAGE=Proteins&QUERY_INDEX=0&QUERY_NUMBER=0&RESULTS_PAGE_TARGET=&RID=T2ZJKRC5012&SHOW_LINKOUT=yes&SHOW_OVERVIEW=yes&STEP_NUMBER=&WORD_SIZE=2&DISPLAY_SORT=2&HSP_SORT=1" \l "sort_mark)** | **[Query coverage](http://blast.ncbi.nlm.nih.gov/Blast.cgi?CMD=Get&ALIGNMENTS=100&ALIGNMENT_VIEW=Pairwise&CDD_SEARCH_STATE=1&DATABASE_SORT=0&DESCRIPTIONS=100&ENTREZ_QUERY=txid9606 %5BORGN%5D&FIRST_QUERY_NUM=0&FORMAT_OBJECT=Alignment&FORMAT_PAGE_TARGET=&FORMAT_TYPE=HTML&GET_SEQUENCE=yes&I_THRESH=&MASK_CHAR=2&MASK_COLOR=1&NEW_DESIGN=on&NEW_VIEW=yes&NUM_OVERVIEW=100&OLD_BLAST=false&PAGE=Proteins&QUERY_INDEX=0&QUERY_NUMBER=0&RESULTS_PAGE_TARGET=&RID=T2ZJKRC5012&SHOW_LINKOUT=yes&SHOW_OVERVIEW=yes&STEP_NUMBER=&WORD_SIZE=2&DISPLAY_SORT=4&HSP_SORT=0" \l "sort_mark)** | **[E value](http://blast.ncbi.nlm.nih.gov/Blast.cgi?CMD=Get&ALIGNMENTS=100&ALIGNMENT_VIEW=Pairwise&CDD_SEARCH_STATE=1&DATABASE_SORT=0&DESCRIPTIONS=100&ENTREZ_QUERY=txid9606 %5BORGN%5D&FIRST_QUERY_NUM=0&FORMAT_OBJECT=Alignment&FORMAT_PAGE_TARGET=&FORMAT_TYPE=HTML&GET_SEQUENCE=yes&I_THRESH=&MASK_CHAR=2&MASK_COLOR=1&NEW_DESIGN=on&NEW_VIEW=yes&NUM_OVERVIEW=100&OLD_BLAST=false&PAGE=Proteins&QUERY_INDEX=0&QUERY_NUMBER=0&RESULTS_PAGE_TARGET=&RID=T2ZJKRC5012&SHOW_LINKOUT=yes&SHOW_OVERVIEW=yes&STEP_NUMBER=&WORD_SIZE=2&DISPLAY_SORT=0&HSP_SORT=0" \l "sort_mark)** |
| --- | --- | --- | --- | --- | --- |
| [NP_001135445.1](http://www.ncbi.nlm.nih.gov/entrez/query.fcgi?cmd=Retrieve&db=Protein&list_uids=213972619&dopt=GenPept&RID=T2ZJKRC5012&log$=prottop&blast_rank=1) | ATPase type 13A2 isoform 2 [Homo sapiens] | [19.3](http://blast.ncbi.nlm.nih.gov/Blast.cgi#213972619%23213972619) | 19.3 | 83% | 161 |
| [NP_001783.2](http://www.ncbi.nlm.nih.gov/entrez/query.fcgi?cmd=Retrieve&db=Protein&list_uids=14589889&dopt=GenPept&RID=T2ZJKRC5012&log$=prottop&blast_rank=2) | cadherin 2, type 1 preproprotein [Homo sapiens] | [19.3](http://blast.ncbi.nlm.nih.gov/Blast.cgi#14589889%2314589889) | 19.3 | 83% | 161 |
| [NP_115791.3](http://www.ncbi.nlm.nih.gov/entrez/query.fcgi?cmd=Retrieve&db=Protein&list_uids=157743265&dopt=GenPept&RID=T2ZJKRC5012&log$=prottop&blast_rank=3) | caspase recruitment domain family, member 11 [Homo sapiens] | [19.3](http://blast.ncbi.nlm.nih.gov/Blast.cgi#157743265%23157743265) | 19.3 | 83% | 161 |
| [NP_071372.1](http://www.ncbi.nlm.nih.gov/entrez/query.fcgi?cmd=Retrieve&db=Protein&list_uids=13435129&dopt=GenPept&RID=T2ZJKRC5012&log$=prottop&blast_rank=4) | ATPase type 13A2 isoform 1 [Homo sapiens] | [19.3](http://blast.ncbi.nlm.nih.gov/Blast.cgi#13435129%2313435129) | 19.3 | 83% | 161 |
| [NP_005458.1](http://www.ncbi.nlm.nih.gov/entrez/query.fcgi?cmd=Retrieve&db=Protein&list_uids=4885505&dopt=GenPept&RID=T2ZJKRC5012&log$=prottop&blast_rank=5) | N-acetylated alpha-linked acidic dipeptidase 2 [Homo sapiens] | [18.9](http://blast.ncbi.nlm.nih.gov/Blast.cgi#4885505%234885505) | 18.9 | 100% | 216 |
| [XP_002345671.1](http://www.ncbi.nlm.nih.gov/entrez/query.fcgi?cmd=Retrieve&db=Protein&list_uids=239753480&dopt=GenPept&RID=T2ZJKRC5012&log$=prottop&blast_rank=6) | PREDICTED: hypothetical protein [Homo sapiens] | [18.5](http://blast.ncbi.nlm.nih.gov/Blast.cgi#239753480%23239753480) | 18.5 | 83% | 289 |
| [XP_001724495.1](http://www.ncbi.nlm.nih.gov/entrez/query.fcgi?cmd=Retrieve&db=Protein&list_uids=169218196&dopt=GenPept&RID=T2ZJKRC5012&log$=prottop&blast_rank=7) | PREDICTED: similar to Werner syndrome ATP-dependent helicase, partial [Homo sapiens] | [18.5](http://blast.ncbi.nlm.nih.gov/Blast.cgi#169218196%23169218196) | 18.5 | 83% | 289 |
| [NP_001032622.1](http://www.ncbi.nlm.nih.gov/entrez/query.fcgi?cmd=Retrieve&db=Protein&list_uids=82830424&dopt=GenPept&RID=T2ZJKRC5012&log$=prottop&blast_rank=8) | gon-4-like isoform a [Homo sapiens] | [18.5](http://blast.ncbi.nlm.nih.gov/Blast.cgi#82830424%2382830424) | 18.5 | 83% | 289 |
| [XP_001134101.1](http://www.ncbi.nlm.nih.gov/entrez/query.fcgi?cmd=Retrieve&db=Protein&list_uids=113413492&dopt=GenPept&RID=T2ZJKRC5012&log$=prottop&blast_rank=9) | PREDICTED: similar to zinc finger and BTB domain containing 8 opposite strand [Homo sapiens] >ref|XP_001133395.1| PREDICTED: similar to zinc finger and BTB domain containing 8 opposite strand [Homo sapiens] >ref|XP_001721028.1| PREDICTED: similar to zinc finger and BTB domain containing 8 opposite strand [Homo sapiens] | [18.5](http://blast.ncbi.nlm.nih.gov/Blast.cgi#113413492%23113413492) | 18.5 | 83% | 289 |
| [NP_005459.2](http://www.ncbi.nlm.nih.gov/entrez/query.fcgi?cmd=Retrieve&db=Protein&list_uids=57232740&dopt=GenPept&RID=T2ZJKRC5012&log$=prottop&blast_rank=10) | N-acetylated alpha-linked acidic dipeptidase-like 1 [Homo sapiens] | [18.5](http://blast.ncbi.nlm.nih.gov/Blast.cgi#57232740%2357232740) | 18.5 | 100% | 289 |
| [NP_000544.2](http://www.ncbi.nlm.nih.gov/entrez/query.fcgi?cmd=Retrieve&db=Protein&list_uids=110735439&dopt=GenPept&RID=T2ZJKRC5012&log$=prottop&blast_rank=11) | Werner syndrome protein [Homo sapiens] | [18.5](http://blast.ncbi.nlm.nih.gov/Blast.cgi#110735439%23110735439) | 18.5 | 83% | 289 |
| [NP_001447.2](http://www.ncbi.nlm.nih.gov/entrez/query.fcgi?cmd=Retrieve&db=Protein&list_uids=116063573&dopt=GenPept&RID=T2ZJKRC5012&log$=prottop&blast_rank=12) | filamin A, alpha isoform 1 [Homo sapiens] | [18.5](http://blast.ncbi.nlm.nih.gov/Blast.cgi#116063573%23116063573) | 18.5 | 83% | 289 |
| [NP_001014986.1](http://www.ncbi.nlm.nih.gov/entrez/query.fcgi?cmd=Retrieve&db=Protein&list_uids=62548858&dopt=GenPept&RID=T2ZJKRC5012&log$=prottop&blast_rank=13) | folate hydrolase 1 isoform 2 [Homo sapiens] | [18.5](http://blast.ncbi.nlm.nih.gov/Blast.cgi#62548858%2362548858) | 18.5 | 100% | 289 |
| [NP_710163.1](http://www.ncbi.nlm.nih.gov/entrez/query.fcgi?cmd=Retrieve&db=Protein&list_uids=24308545&dopt=GenPept&RID=T2ZJKRC5012&log$=prottop&blast_rank=14) | folate hydrolase 1B [Homo sapiens] | [18.5](http://blast.ncbi.nlm.nih.gov/Blast.cgi#24308545%2324308545) | 18.5 | 100% | 289 |
| [NP_001104026.1](http://www.ncbi.nlm.nih.gov/entrez/query.fcgi?cmd=Retrieve&db=Protein&list_uids=160420317&dopt=GenPept&RID=T2ZJKRC5012&log$=prottop&blast_rank=15) | filamin A, alpha isoform 2 [Homo sapiens] | [18.5](http://blast.ncbi.nlm.nih.gov/Blast.cgi#160420317%23160420317) | 18.5 | 83% | 289 |
| [NP_065908.1](http://www.ncbi.nlm.nih.gov/entrez/query.fcgi?cmd=Retrieve&db=Protein&list_uids=17978485&dopt=GenPept&RID=T2ZJKRC5012&log$=prottop&blast_rank=16) | vacuolar protein sorting 18 [Homo sapiens] | [18.5](http://blast.ncbi.nlm.nih.gov/Blast.cgi#17978485%2317978485) | 18.5 | 83% | 289 |
| [NP_004467.1](http://www.ncbi.nlm.nih.gov/entrez/query.fcgi?cmd=Retrieve&db=Protein&list_uids=4758398&dopt=GenPept&RID=T2ZJKRC5012&log$=prottop&blast_rank=17) | folate hydrolase 1 isoform 1 [Homo sapiens] | [18.5](http://blast.ncbi.nlm.nih.gov/Blast.cgi#4758398%234758398) | 18.5 | 100% | 289 |
| [NP_848642.1](http://www.ncbi.nlm.nih.gov/entrez/query.fcgi?cmd=Retrieve&db=Protein&list_uids=30425510&dopt=GenPept&RID=T2ZJKRC5012&log$=prottop&blast_rank=18) | zinc finger and BTB domain containing 8 opposite strand [Homo sapiens] | [18.5](http://blast.ncbi.nlm.nih.gov/Blast.cgi#30425510%2330425510) | 18.5 | 83% | 289 |
| [NP_874364.1](http://www.ncbi.nlm.nih.gov/entrez/query.fcgi?cmd=Retrieve&db=Protein&list_uids=33186901&dopt=GenPept&RID=T2ZJKRC5012&log$=prottop&blast_rank=19) | hypothetical protein LOC359845 [Homo sapiens] | [18.0](http://blast.ncbi.nlm.nih.gov/Blast.cgi#33186901%2333186901) | 18.0 | 100% | 388 |
| [NP_002651.2](http://www.ncbi.nlm.nih.gov/entrez/query.fcgi?cmd=Retrieve&db=Protein&list_uids=33598948&dopt=GenPept&RID=T2ZJKRC5012&log$=prottop&blast_rank=20) | phospholipase C, gamma 1 isoform a [Homo sapiens] | [18.0](http://blast.ncbi.nlm.nih.gov/Blast.cgi#33598948%2333598948) | 18.0 | 100% | 388 |
| [NP_877963.1](http://www.ncbi.nlm.nih.gov/entrez/query.fcgi?cmd=Retrieve&db=Protein&list_uids=33598946&dopt=GenPept&RID=T2ZJKRC5012&log$=prottop&blast_rank=21) | phospholipase C, gamma 1 isoform b [Homo sapiens] | [18.0](http://blast.ncbi.nlm.nih.gov/Blast.cgi#33598946%2333598946) | 18.0 | 100% | 388 |
| [NP_055309.2](http://www.ncbi.nlm.nih.gov/entrez/query.fcgi?cmd=Retrieve&db=Protein&list_uids=116805348&dopt=GenPept&RID=T2ZJKRC5012&log$=prottop&blast_rank=22) | trinucleotide repeat containing 6A [Homo sapiens] | [17.6](http://blast.ncbi.nlm.nih.gov/Blast.cgi#116805348%23116805348) | 17.6 | 100% | 521 |
| [NP_787069.3](http://www.ncbi.nlm.nih.gov/entrez/query.fcgi?cmd=Retrieve&db=Protein&list_uids=95147559&dopt=GenPept&RID=T2ZJKRC5012&log$=prottop&blast_rank=23) | ankyrin repeat domain 43 precursor [Homo sapiens] | [17.6](http://blast.ncbi.nlm.nih.gov/Blast.cgi#95147559%2395147559) | 17.6 | 100% | 521 |
| [NP_065166.2](http://www.ncbi.nlm.nih.gov/entrez/query.fcgi?cmd=Retrieve&db=Protein&list_uids=21704281&dopt=GenPept&RID=T2ZJKRC5012&log$=prottop&blast_rank=24) | junctophilin 2 isoform 1 [Homo sapiens] | [17.6](http://blast.ncbi.nlm.nih.gov/Blast.cgi#21704281%2321704281) | 17.6 | 100% | 521 |
| [NP_066565.1](http://www.ncbi.nlm.nih.gov/entrez/query.fcgi?cmd=Retrieve&db=Protein&list_uids=10835073&dopt=GenPept&RID=T2ZJKRC5012&log$=prottop&blast_rank=25) | N-myristoyltransferase 1 [Homo sapiens] | [17.2](http://blast.ncbi.nlm.nih.gov/Blast.cgi#10835073%2310835073) | 17.2 | 100% | 699 |
| [NP_001157862.1](http://www.ncbi.nlm.nih.gov/entrez/query.fcgi?cmd=Retrieve&db=Protein&list_uids=256600204&dopt=GenPept&RID=T2ZJKRC5012&log$=prottop&blast_rank=26) | PDZ domain-containing guanine nucleotide exchange factor I isoform 6 [Homo sapiens] | [16.8](http://blast.ncbi.nlm.nih.gov/Blast.cgi#256600204%23256600204) | 16.8 | 100% | 938 |
| [NP_057424.3](http://www.ncbi.nlm.nih.gov/entrez/query.fcgi?cmd=Retrieve&db=Protein&list_uids=256600194&dopt=GenPept&RID=T2ZJKRC5012&log$=prottop&blast_rank=27) | PDZ domain-containing guanine nucleotide exchange factor I isoform 2 [Homo sapiens] | [16.8](http://blast.ncbi.nlm.nih.gov/Blast.cgi#256600194%23256600194) | 16.8 | 100% | 938 |
| [NP_001157859.1](http://www.ncbi.nlm.nih.gov/entrez/query.fcgi?cmd=Retrieve&db=Protein&list_uids=256600198&dopt=GenPept&RID=T2ZJKRC5012&log$=prottop&blast_rank=28) | PDZ domain-containing guanine nucleotide exchange factor I isoform 3 [Homo sapiens] | [16.8](http://blast.ncbi.nlm.nih.gov/Blast.cgi#256600198%23256600198) | 16.8 | 100% | 938 |
| [NP_001157858.1](http://www.ncbi.nlm.nih.gov/entrez/query.fcgi?cmd=Retrieve&db=Protein&list_uids=256600196&dopt=GenPept&RID=T2ZJKRC5012&log$=prottop&blast_rank=29) | PDZ domain-containing guanine nucleotide exchange factor I isoform 1 [Homo sapiens] | [16.8](http://blast.ncbi.nlm.nih.gov/Blast.cgi#256600196%23256600196) | 16.8 | 100% | 938 |
| [NP_001124151.1](http://www.ncbi.nlm.nih.gov/entrez/query.fcgi?cmd=Retrieve&db=Protein&list_uids=194578909&dopt=GenPept&RID=T2ZJKRC5012&log$=prottop&blast_rank=30) | eukaryotic translation initiation factor 4E isoform 2 [Homo sapiens] | [16.8](http://blast.ncbi.nlm.nih.gov/Blast.cgi#194578909%23194578909) | 16.8 | 83% | 938 |
| [NP_008832.2](http://www.ncbi.nlm.nih.gov/entrez/query.fcgi?cmd=Retrieve&db=Protein&list_uids=156119615&dopt=GenPept&RID=T2ZJKRC5012&log$=prottop&blast_rank=31) | myosin IXA [Homo sapiens] | [16.8](http://blast.ncbi.nlm.nih.gov/Blast.cgi#156119615%23156119615) | 16.8 | 83% | 938 |
| [NP_573400.3](http://www.ncbi.nlm.nih.gov/entrez/query.fcgi?cmd=Retrieve&db=Protein&list_uids=148539858&dopt=GenPept&RID=T2ZJKRC5012&log$=prottop&blast_rank=32) | protein tyrosine phosphatase, receptor type, T isoform 1 precursor [Homo sapiens] | [16.8](http://blast.ncbi.nlm.nih.gov/Blast.cgi#148539858%23148539858) | 16.8 | 83% | 938 |
| [NP_001157860.1](http://www.ncbi.nlm.nih.gov/entrez/query.fcgi?cmd=Retrieve&db=Protein&list_uids=256600200&dopt=GenPept&RID=T2ZJKRC5012&log$=prottop&blast_rank=33) | PDZ domain-containing guanine nucleotide exchange factor I isoform 4 [Homo sapiens] | [16.8](http://blast.ncbi.nlm.nih.gov/Blast.cgi#256600200%23256600200) | 16.8 | 100% | 938 |
| [NP_071349.3](http://www.ncbi.nlm.nih.gov/entrez/query.fcgi?cmd=Retrieve&db=Protein&list_uids=192449449&dopt=GenPept&RID=T2ZJKRC5012&log$=prottop&blast_rank=34) | ubiquitin-conjugating enzyme E2O [Homo sapiens] | [16.8](http://blast.ncbi.nlm.nih.gov/Blast.cgi#192449449%23192449449) | 16.8 | 83% | 938 |
| [XP_001726634.1](http://www.ncbi.nlm.nih.gov/entrez/query.fcgi?cmd=Retrieve&db=Protein&list_uids=169210992&dopt=GenPept&RID=T2ZJKRC5012&log$=prottop&blast_rank=35) | PREDICTED: hypothetical protein LOC100131693 [Homo sapiens] >ref|XP_001723156.1| PREDICTED: similar to hCG1777996 [Homo sapiens] >ref|XP_001725246.1| PREDICTED: similar to hCG1777996 [Homo sapiens] | [16.8](http://blast.ncbi.nlm.nih.gov/Blast.cgi#169210992%23169210992) | 16.8 | 83% | 938 |
| [NP_001157861.1](http://www.ncbi.nlm.nih.gov/entrez/query.fcgi?cmd=Retrieve&db=Protein&list_uids=256600202&dopt=GenPept&RID=T2ZJKRC5012&log$=prottop&blast_rank=36) | PDZ domain-containing guanine nucleotide exchange factor I isoform 5 [Homo sapiens] | [16.8](http://blast.ncbi.nlm.nih.gov/Blast.cgi#256600202%23256600202) | 16.8 | 100% | 938 |
| [NP_001288.3](http://www.ncbi.nlm.nih.gov/entrez/query.fcgi?cmd=Retrieve&db=Protein&list_uids=114205399&dopt=GenPept&RID=T2ZJKRC5012&log$=prottop&blast_rank=37) | cyclic nucleotide gated channel beta 1 isoform a [Homo sapiens] | [16.8](http://blast.ncbi.nlm.nih.gov/Blast.cgi#114205399%23114205399) | 16.8 | 100% | 938 |
| [NP_008981.4](http://www.ncbi.nlm.nih.gov/entrez/query.fcgi?cmd=Retrieve&db=Protein&list_uids=148539860&dopt=GenPept&RID=T2ZJKRC5012&log$=prottop&blast_rank=38) | protein tyrosine phosphatase, receptor type, T isoform 2 precursor [Homo sapiens] | [16.8](http://blast.ncbi.nlm.nih.gov/Blast.cgi#148539860%23148539860) | 16.8 | 83% | 938 |
| [NP_000713.2](http://www.ncbi.nlm.nih.gov/entrez/query.fcgi?cmd=Retrieve&db=Protein&list_uids=54112390&dopt=GenPept&RID=T2ZJKRC5012&log$=prottop&blast_rank=39) | calcium channel, voltage-dependent, alpha 2/delta subunit 1 [Homo sapiens] | [16.8](http://blast.ncbi.nlm.nih.gov/Blast.cgi#54112390%2354112390) | 16.8 | 83% | 938 |
| [NP_000126.2](http://www.ncbi.nlm.nih.gov/entrez/query.fcgi?cmd=Retrieve&db=Protein&list_uids=66880553&dopt=GenPept&RID=T2ZJKRC5012&log$=prottop&blast_rank=40) | Fanconi anemia, complementation group A isoform a [Homo sapiens] | [16.8](http://blast.ncbi.nlm.nih.gov/Blast.cgi#66880553%2366880553) | 16.8 | 83% | 938 |
| [NP_291021.2](http://www.ncbi.nlm.nih.gov/entrez/query.fcgi?cmd=Retrieve&db=Protein&list_uids=52317156&dopt=GenPept&RID=T2ZJKRC5012&log$=prottop&blast_rank=41) | carcinoembryonic antigen-related cell adhesion molecule isoform 2 [Homo sapiens] | [16.8](http://blast.ncbi.nlm.nih.gov/Blast.cgi#52317156%2352317156) | 16.8 | 83% | 938 |
| [NP_066267.2](http://www.ncbi.nlm.nih.gov/entrez/query.fcgi?cmd=Retrieve&db=Protein&list_uids=32967601&dopt=GenPept&RID=T2ZJKRC5012&log$=prottop&blast_rank=42) | ankyrin 3 isoform 1 [Homo sapiens] | [16.8](http://blast.ncbi.nlm.nih.gov/Blast.cgi#32967601%2332967601) | 32.7 | 83% | 938 |
| [NP_073583.3](http://www.ncbi.nlm.nih.gov/entrez/query.fcgi?cmd=Retrieve&db=Protein&list_uids=224600454&dopt=GenPept&RID=T2ZJKRC5012&log$=prottop&blast_rank=43) | MOCO sulphurase C-terminal domain containing 1 precursor [Homo sapiens] | [16.8](http://blast.ncbi.nlm.nih.gov/Blast.cgi#224600454%23224600454) | 16.8 | 83% | 938 |
| [NP_001513.2](http://www.ncbi.nlm.nih.gov/entrez/query.fcgi?cmd=Retrieve&db=Protein&list_uids=134152694&dopt=GenPept&RID=T2ZJKRC5012&log$=prottop&blast_rank=44) | guanylate cyclase 2F precursor [Homo sapiens] | [16.8](http://blast.ncbi.nlm.nih.gov/Blast.cgi#134152694%23134152694) | 16.8 | 83% | 938 |
| [NP_001018122.1](http://www.ncbi.nlm.nih.gov/entrez/query.fcgi?cmd=Retrieve&db=Protein&list_uids=66879666&dopt=GenPept&RID=T2ZJKRC5012&log$=prottop&blast_rank=45) | Fanconi anemia, complementation group A isoform b [Homo sapiens] | [16.8](http://blast.ncbi.nlm.nih.gov/Blast.cgi#66879666%2366879666) | 16.8 | 83% | 938 |
| [NP_065830.2](http://www.ncbi.nlm.nih.gov/entrez/query.fcgi?cmd=Retrieve&db=Protein&list_uids=56243599&dopt=GenPept&RID=T2ZJKRC5012&log$=prottop&blast_rank=46) | WD repeat domain 35 isoform 2 [Homo sapiens] | [16.8](http://blast.ncbi.nlm.nih.gov/Blast.cgi#56243599%2356243599) | 16.8 | 100% | 938 |
| [NP_001091976.1](http://www.ncbi.nlm.nih.gov/entrez/query.fcgi?cmd=Retrieve&db=Protein&list_uids=148612793&dopt=GenPept&RID=T2ZJKRC5012&log$=prottop&blast_rank=47) | carcinoembryonic antigen-related cell adhesion molecule isoform 1 [Homo sapiens] | [16.8](http://blast.ncbi.nlm.nih.gov/Blast.cgi#148612793%23148612793) | 16.8 | 83% | 938 |
| [NP_060482.2](http://www.ncbi.nlm.nih.gov/entrez/query.fcgi?cmd=Retrieve&db=Protein&list_uids=124430752&dopt=GenPept&RID=T2ZJKRC5012&log$=prottop&blast_rank=48) | kinesin family member 26B [Homo sapiens] | [16.8](http://blast.ncbi.nlm.nih.gov/Blast.cgi#124430752%23124430752) | 16.8 | 83% | 938 |
| [NP_006527.1](http://www.ncbi.nlm.nih.gov/entrez/query.fcgi?cmd=Retrieve&db=Protein&list_uids=5729769&dopt=GenPept&RID=T2ZJKRC5012&log$=prottop&blast_rank=49) | chloride channel accessory 2 precursor [Homo sapiens] | [16.8](http://blast.ncbi.nlm.nih.gov/Blast.cgi#5729769%235729769) | 16.8 | 83% | 938 |
| [NP_060368.2](http://www.ncbi.nlm.nih.gov/entrez/query.fcgi?cmd=Retrieve&db=Protein&list_uids=31542713&dopt=GenPept&RID=T2ZJKRC5012&log$=prottop&blast_rank=50) | MOCO sulphurase C-terminal domain containing 2 precursor [Homo sapiens] | [16.8](http://blast.ncbi.nlm.nih.gov/Blast.cgi#31542713%2331542713) | 16.8 | 83% | 938 |
| [NP_001959.1](http://www.ncbi.nlm.nih.gov/entrez/query.fcgi?cmd=Retrieve&db=Protein&list_uids=4503535&dopt=GenPept&RID=T2ZJKRC5012&log$=prottop&blast_rank=51) | eukaryotic translation initiation factor 4E isoform 1 [Homo sapiens] | [16.8](http://blast.ncbi.nlm.nih.gov/Blast.cgi#4503535%234503535) | 16.8 | 83% | 938 |
| [NP_036231.1](http://www.ncbi.nlm.nih.gov/entrez/query.fcgi?cmd=Retrieve&db=Protein&list_uids=6912246&dopt=GenPept&RID=T2ZJKRC5012&log$=prottop&blast_rank=52) | CD3E antigen, epsilon polypeptide associated protein [Homo sapiens] | [16.8](http://blast.ncbi.nlm.nih.gov/Blast.cgi#6912246%236912246) | 16.8 | 83% | 938 |
| [NP_001013633.1](http://www.ncbi.nlm.nih.gov/entrez/query.fcgi?cmd=Retrieve&db=Protein&list_uids=61966689&dopt=GenPept&RID=T2ZJKRC5012&log$=prottop&blast_rank=53) | hypothetical protein LOC541468 [Homo sapiens] | [16.8](http://blast.ncbi.nlm.nih.gov/Blast.cgi#61966689%2361966689) | 16.8 | 100% | 938 |
| [NP_597728.1](http://www.ncbi.nlm.nih.gov/entrez/query.fcgi?cmd=Retrieve&db=Protein&list_uids=145386517&dopt=GenPept&RID=T2ZJKRC5012&log$=prottop&blast_rank=54) | phostensin [Homo sapiens] >ref|NP_001128342.1| phostensin [Homo sapiens] | [16.8](http://blast.ncbi.nlm.nih.gov/Blast.cgi#145386517%23145386517) | 16.8 | 100% | 938 |
| [NP_001006658.1](http://www.ncbi.nlm.nih.gov/entrez/query.fcgi?cmd=Retrieve&db=Protein&list_uids=55743161&dopt=GenPept&RID=T2ZJKRC5012&log$=prottop&blast_rank=55) | WD repeat domain 35 isoform 1 [Homo sapiens] | [16.8](http://blast.ncbi.nlm.nih.gov/Blast.cgi#55743161%2355743161) | 16.8 | 100% | 938 |
| [NP_001165401.1](http://www.ncbi.nlm.nih.gov/entrez/query.fcgi?cmd=Retrieve&db=Protein&list_uids=284925128&dopt=GenPept&RID=T2ZJKRC5012&log$=prottop&blast_rank=56) | cadherin-like 23 isoform 3 [Homo sapiens] | [16.3](http://blast.ncbi.nlm.nih.gov/Blast.cgi#284925128%23284925128) | 16.3 | 66% | 1259 |
| [NP_001161407.1](http://www.ncbi.nlm.nih.gov/entrez/query.fcgi?cmd=Retrieve&db=Protein&list_uids=269308190&dopt=GenPept&RID=T2ZJKRC5012&log$=prottop&blast_rank=57) | cerebral cavernous malformation 2 isoform 4 [Homo sapiens] | [16.3](http://blast.ncbi.nlm.nih.gov/Blast.cgi#269308190%23269308190) | 16.3 | 66% | 1259 |
| [NP_079350.5](http://www.ncbi.nlm.nih.gov/entrez/query.fcgi?cmd=Retrieve&db=Protein&list_uids=256000767&dopt=GenPept&RID=T2ZJKRC5012&log$=prottop&blast_rank=58) | Fraser syndrome 1 protein isoform 1 precursor [Homo sapiens] | [16.3](http://blast.ncbi.nlm.nih.gov/Blast.cgi#256000767%23256000767) | 29.7 | 100% | 1259 |
| [XP_002345529.1](http://www.ncbi.nlm.nih.gov/entrez/query.fcgi?cmd=Retrieve&db=Protein&list_uids=239757714&dopt=GenPept&RID=T2ZJKRC5012&log$=prottop&blast_rank=59) | PREDICTED: hypothetical protein XP_002345529 [Homo sapiens] | [16.3](http://blast.ncbi.nlm.nih.gov/Blast.cgi#239757714%23239757714) | 16.3 | 83% | 1259 |
| [XP_002344564.1](http://www.ncbi.nlm.nih.gov/entrez/query.fcgi?cmd=Retrieve&db=Protein&list_uids=239755412&dopt=GenPept&RID=T2ZJKRC5012&log$=prottop&blast_rank=60) | PREDICTED: hypothetical protein [Homo sapiens] | [16.3](http://blast.ncbi.nlm.nih.gov/Blast.cgi#239755412%23239755412) | 16.3 | 66% | 1259 |
| [XP_002348147.1](http://www.ncbi.nlm.nih.gov/entrez/query.fcgi?cmd=Retrieve&db=Protein&list_uids=239752201&dopt=GenPept&RID=T2ZJKRC5012&log$=prottop&blast_rank=61) | PREDICTED: hypothetical protein XP_002348147 [Homo sapiens] | [16.3](http://blast.ncbi.nlm.nih.gov/Blast.cgi#239752201%23239752201) | 16.3 | 83% | 1259 |
| [XP_002344242.1](http://www.ncbi.nlm.nih.gov/entrez/query.fcgi?cmd=Retrieve&db=Protein&list_uids=239747010&dopt=GenPept&RID=T2ZJKRC5012&log$=prottop&blast_rank=62) | PREDICTED: hypothetical protein XP_002344242 [Homo sapiens] >ref|XP_002348285.1| PREDICTED: hypothetical protein [Homo sapiens] | [16.3](http://blast.ncbi.nlm.nih.gov/Blast.cgi#239747010%23239747010) | 16.3 | 66% | 1259 |
| [XP_002342471.1](http://www.ncbi.nlm.nih.gov/entrez/query.fcgi?cmd=Retrieve&db=Protein&list_uids=239742276&dopt=GenPept&RID=T2ZJKRC5012&log$=prottop&blast_rank=63) | PREDICTED: hypothetical protein XP_002342471 [Homo sapiens] | [16.3](http://blast.ncbi.nlm.nih.gov/Blast.cgi#239742276%23239742276) | 16.3 | 100% | 1259 |
| [NP_001130038.2](http://www.ncbi.nlm.nih.gov/entrez/query.fcgi?cmd=Retrieve&db=Protein&list_uids=222136644&dopt=GenPept&RID=T2ZJKRC5012&log$=prottop&blast_rank=64) | RAD21-like 1 [Homo sapiens] | [16.3](http://blast.ncbi.nlm.nih.gov/Blast.cgi#222136644%23222136644) | 16.3 | 66% | 1259 |
| [NP_071407.4](http://www.ncbi.nlm.nih.gov/entrez/query.fcgi?cmd=Retrieve&db=Protein&list_uids=189571674&dopt=GenPept&RID=T2ZJKRC5012&log$=prottop&blast_rank=65) | cadherin-like 23 isoform 1 [Homo sapiens] | [16.3](http://blast.ncbi.nlm.nih.gov/Blast.cgi#189571674%23189571674) | 16.3 | 66% | 1259 |
| [NP_001127835.1](http://www.ncbi.nlm.nih.gov/entrez/query.fcgi?cmd=Retrieve&db=Protein&list_uids=197276594&dopt=GenPept&RID=T2ZJKRC5012&log$=prottop&blast_rank=66) | RNA binding motif protein 20 [Homo sapiens] | [16.3](http://blast.ncbi.nlm.nih.gov/Blast.cgi#197276594%23197276594) | 16.3 | 66% | 1259 |
| [NP_071934.3](http://www.ncbi.nlm.nih.gov/entrez/query.fcgi?cmd=Retrieve&db=Protein&list_uids=149999380&dopt=GenPept&RID=T2ZJKRC5012&log$=prottop&blast_rank=67) | inverted formin 2 isoform 1 [Homo sapiens] | [16.3](http://blast.ncbi.nlm.nih.gov/Blast.cgi#149999380%23149999380) | 16.3 | 66% | 1259 |
| [NP_001026884.3](http://www.ncbi.nlm.nih.gov/entrez/query.fcgi?cmd=Retrieve&db=Protein&list_uids=149999378&dopt=GenPept&RID=T2ZJKRC5012&log$=prottop&blast_rank=68) | inverted formin 2 isoform 2 [Homo sapiens] | [16.3](http://blast.ncbi.nlm.nih.gov/Blast.cgi#149999378%23149999378) | 16.3 | 66% | 1259 |
| [NP_085135.1](http://www.ncbi.nlm.nih.gov/entrez/query.fcgi?cmd=Retrieve&db=Protein&list_uids=149944526&dopt=GenPept&RID=T2ZJKRC5012&log$=prottop&blast_rank=69) | additional sex combs like 3 [Homo sapiens] | [16.3](http://blast.ncbi.nlm.nih.gov/Blast.cgi#149944526%23149944526) | 16.3 | 66% | 1259 |
| [NP_001165403.1](http://www.ncbi.nlm.nih.gov/entrez/query.fcgi?cmd=Retrieve&db=Protein&list_uids=284925132&dopt=GenPept&RID=T2ZJKRC5012&log$=prottop&blast_rank=70) | cadherin-like 23 isoform 5 [Homo sapiens] | [16.3](http://blast.ncbi.nlm.nih.gov/Blast.cgi#284925132%23284925132) | 16.3 | 66% | 1259 |
| [NP_001401.2](http://www.ncbi.nlm.nih.gov/entrez/query.fcgi?cmd=Retrieve&db=Protein&list_uids=145701025&dopt=GenPept&RID=T2ZJKRC5012&log$=prottop&blast_rank=71) | multiple EGF-like-domains 8 [Homo sapiens] | [16.3](http://blast.ncbi.nlm.nih.gov/Blast.cgi#145701025%23145701025) | 16.3 | 66% | 1259 |
| [NP_775901.3](http://www.ncbi.nlm.nih.gov/entrez/query.fcgi?cmd=Retrieve&db=Protein&list_uids=145046269&dopt=GenPept&RID=T2ZJKRC5012&log$=prottop&blast_rank=72) | rotatin [Homo sapiens] | [16.3](http://blast.ncbi.nlm.nih.gov/Blast.cgi#145046269%23145046269) | 16.3 | 66% | 1259 |
| [NP_055196.2](http://www.ncbi.nlm.nih.gov/entrez/query.fcgi?cmd=Retrieve&db=Protein&list_uids=91992160&dopt=GenPept&RID=T2ZJKRC5012&log$=prottop&blast_rank=73) | mutL homolog 3 isoform 2 [Homo sapiens] | [16.3](http://blast.ncbi.nlm.nih.gov/Blast.cgi#91992160%2391992160) | 16.3 | 66% | 1259 |
| [NP_001035197.1](http://www.ncbi.nlm.nih.gov/entrez/query.fcgi?cmd=Retrieve&db=Protein&list_uids=91992162&dopt=GenPept&RID=T2ZJKRC5012&log$=prottop&blast_rank=74) | mutL homolog 3 isoform 1 [Homo sapiens] | [16.3](http://blast.ncbi.nlm.nih.gov/Blast.cgi#91992162%2391992162) | 16.3 | 66% | 1259 |
| [NP_001164402.1](http://www.ncbi.nlm.nih.gov/entrez/query.fcgi?cmd=Retrieve&db=Protein&list_uids=283436083&dopt=GenPept&RID=T2ZJKRC5012&log$=prottop&blast_rank=75) | forkhead box O4 isoform 2 [Homo sapiens] | [16.3](http://blast.ncbi.nlm.nih.gov/Blast.cgi#283436083%23283436083) | 16.3 | 66% | 1259 |
| [NP_001127923.1](http://www.ncbi.nlm.nih.gov/entrez/query.fcgi?cmd=Retrieve&db=Protein&list_uids=197313781&dopt=GenPept&RID=T2ZJKRC5012&log$=prottop&blast_rank=76) | transmembrane protein 130 isoform c [Homo sapiens] | [16.3](http://blast.ncbi.nlm.nih.gov/Blast.cgi#197313781%23197313781) | 16.3 | 66% | 1259 |
| [NP_689761.2](http://www.ncbi.nlm.nih.gov/entrez/query.fcgi?cmd=Retrieve&db=Protein&list_uids=115430110&dopt=GenPept&RID=T2ZJKRC5012&log$=prottop&blast_rank=77) | hypothetical protein LOC153643 [Homo sapiens] | [16.3](http://blast.ncbi.nlm.nih.gov/Blast.cgi#115430110%23115430110) | 16.3 | 66% | 1259 |
| [NP_001035088.2](http://www.ncbi.nlm.nih.gov/entrez/query.fcgi?cmd=Retrieve&db=Protein&list_uids=115392150&dopt=GenPept&RID=T2ZJKRC5012&log$=prottop&blast_rank=78) | hypothetical protein LOC644815 [Homo sapiens] | [16.3](http://blast.ncbi.nlm.nih.gov/Blast.cgi#115392150%23115392150) | 16.3 | 66% | 1259 |
| [NP_001026.2](http://www.ncbi.nlm.nih.gov/entrez/query.fcgi?cmd=Retrieve&db=Protein&list_uids=112799847&dopt=GenPept&RID=T2ZJKRC5012&log$=prottop&blast_rank=79) | cardiac muscle ryanodine receptor [Homo sapiens] | [16.3](http://blast.ncbi.nlm.nih.gov/Blast.cgi#112799847%23112799847) | 16.3 | 66% | 1259 |
| [NP_001035743.1](http://www.ncbi.nlm.nih.gov/entrez/query.fcgi?cmd=Retrieve&db=Protein&list_uids=98961138&dopt=GenPept&RID=T2ZJKRC5012&log$=prottop&blast_rank=80) | ZXD family zinc finger C isoform 2 [Homo sapiens] | [16.3](http://blast.ncbi.nlm.nih.gov/Blast.cgi#98961138%2398961138) | 16.3 | 66% | 1259 |
| [NP_079388.3](http://www.ncbi.nlm.nih.gov/entrez/query.fcgi?cmd=Retrieve&db=Protein&list_uids=98961133&dopt=GenPept&RID=T2ZJKRC5012&log$=prottop&blast_rank=81) | ZXD family zinc finger C isoform 1 [Homo sapiens] | [16.3](http://blast.ncbi.nlm.nih.gov/Blast.cgi#98961133%2398961133) | 16.3 | 66% | 1259 |
| [NP_001073922.2](http://www.ncbi.nlm.nih.gov/entrez/query.fcgi?cmd=Retrieve&db=Protein&list_uids=160948599&dopt=GenPept&RID=T2ZJKRC5012&log$=prottop&blast_rank=82) | integrator complex subunit 1 [Homo sapiens] | [16.3](http://blast.ncbi.nlm.nih.gov/Blast.cgi#160948599%23160948599) | 16.3 | 66% | 1259 |
| [NP_056041.1](http://www.ncbi.nlm.nih.gov/entrez/query.fcgi?cmd=Retrieve&db=Protein&list_uids=54792782&dopt=GenPept&RID=T2ZJKRC5012&log$=prottop&blast_rank=83) | C-type lectin domain family 16, member A [Homo sapiens] | [16.3](http://blast.ncbi.nlm.nih.gov/Blast.cgi#54792782%2354792782) | 16.3 | 66% | 1259 |
| [NP_004662.2](http://www.ncbi.nlm.nih.gov/entrez/query.fcgi?cmd=Retrieve&db=Protein&list_uids=56699458&dopt=GenPept&RID=T2ZJKRC5012&log$=prottop&blast_rank=84) | protein inhibitor of activated STAT X isoform beta [Homo sapiens] | [16.3](http://blast.ncbi.nlm.nih.gov/Blast.cgi#56699458%2356699458) | 16.3 | 66% | 1259 |
| [NP_443128.2](http://www.ncbi.nlm.nih.gov/entrez/query.fcgi?cmd=Retrieve&db=Protein&list_uids=92110053&dopt=GenPept&RID=T2ZJKRC5012&log$=prottop&blast_rank=85) | CUB and Sushi multiple domains 2 [Homo sapiens] | [16.3](http://blast.ncbi.nlm.nih.gov/Blast.cgi#92110053%2392110053) | 84.0 | 100% | 1259 |
| [NP_004275.3](http://www.ncbi.nlm.nih.gov/entrez/query.fcgi?cmd=Retrieve&db=Protein&list_uids=148612870&dopt=GenPept&RID=T2ZJKRC5012&log$=prottop&blast_rank=86) | chromodomain helicase DNA binding protein 1-like [Homo sapiens] | [16.3](http://blast.ncbi.nlm.nih.gov/Blast.cgi#148612870%23148612870) | 16.3 | 66% | 1259 |
| [NP_001124387.1](http://www.ncbi.nlm.nih.gov/entrez/query.fcgi?cmd=Retrieve&db=Protein&list_uids=195963410&dopt=GenPept&RID=T2ZJKRC5012&log$=prottop&blast_rank=87) | MEF2 activating motif and SAP domain containing transcriptional regulator isoform 1 [Homo sapiens] | [16.3](http://blast.ncbi.nlm.nih.gov/Blast.cgi#195963410%23195963410) | 16.3 | 66% | 1259 |
| [NP_001012724.1](http://www.ncbi.nlm.nih.gov/entrez/query.fcgi?cmd=Retrieve&db=Protein&list_uids=61676098&dopt=GenPept&RID=T2ZJKRC5012&log$=prottop&blast_rank=88) | hypothetical protein LOC283551 [Homo sapiens] | [16.3](http://blast.ncbi.nlm.nih.gov/Blast.cgi#61676098%2361676098) | 16.3 | 66% | 1259 |
| [NP_079440.2](http://www.ncbi.nlm.nih.gov/entrez/query.fcgi?cmd=Retrieve&db=Protein&list_uids=38569491&dopt=GenPept&RID=T2ZJKRC5012&log$=prottop&blast_rank=89) | serine/threonine-protein kinase QSK [Homo sapiens] | [16.3](http://blast.ncbi.nlm.nih.gov/Blast.cgi#38569491%2338569491) | 16.3 | 66% | 1259 |
| [NP_071401.3](http://www.ncbi.nlm.nih.gov/entrez/query.fcgi?cmd=Retrieve&db=Protein&list_uids=31652264&dopt=GenPept&RID=T2ZJKRC5012&log$=prottop&blast_rank=90) | RNA binding motif protein 26 [Homo sapiens] | [16.3](http://blast.ncbi.nlm.nih.gov/Blast.cgi#31652264%2331652264) | 16.3 | 66% | 1259 |
| [NP_001006939.2](http://www.ncbi.nlm.nih.gov/entrez/query.fcgi?cmd=Retrieve&db=Protein&list_uids=118600971&dopt=GenPept&RID=T2ZJKRC5012&log$=prottop&blast_rank=91) | transcription elongation factor A (SII)-like 6 [Homo sapiens] | [16.3](http://blast.ncbi.nlm.nih.gov/Blast.cgi#118600971%23118600971) | 16.3 | 66% | 1259 |
| [NP_060060.3](http://www.ncbi.nlm.nih.gov/entrez/query.fcgi?cmd=Retrieve&db=Protein&list_uids=27881484&dopt=GenPept&RID=T2ZJKRC5012&log$=prottop&blast_rank=92) | zinc finger CCCH-type containing 7B [Homo sapiens] | [16.3](http://blast.ncbi.nlm.nih.gov/Blast.cgi#27881484%2327881484) | 16.3 | 66% | 1259 |
| [NP_003105.2](http://www.ncbi.nlm.nih.gov/entrez/query.fcgi?cmd=Retrieve&db=Protein&list_uids=27262636&dopt=GenPept&RID=T2ZJKRC5012&log$=prottop&blast_rank=93) | sperm associated antigen 1 [Homo sapiens] >ref|NP_757367.1| sperm associated antigen 1 [Homo sapiens] | [16.3](http://blast.ncbi.nlm.nih.gov/Blast.cgi#27262636%2327262636) | 16.3 | 66% | 1259 |
| [NP_001161971.1](http://www.ncbi.nlm.nih.gov/entrez/query.fcgi?cmd=Retrieve&db=Protein&list_uids=271398277&dopt=GenPept&RID=T2ZJKRC5012&log$=prottop&blast_rank=94) | CNDP dipeptidase 2 isoform 2 [Homo sapiens] | [16.3](http://blast.ncbi.nlm.nih.gov/Blast.cgi#271398277%23271398277) | 16.3 | 66% | 1259 |
| [NP_061894.2](http://www.ncbi.nlm.nih.gov/entrez/query.fcgi?cmd=Retrieve&db=Protein&list_uids=169790777&dopt=GenPept&RID=T2ZJKRC5012&log$=prottop&blast_rank=95) | hypothetical protein LOC54494 [Homo sapiens] | [16.3](http://blast.ncbi.nlm.nih.gov/Blast.cgi#169790777%23169790777) | 16.3 | 66% | 1259 |
| [NP_005929.2](http://www.ncbi.nlm.nih.gov/entrez/query.fcgi?cmd=Retrieve&db=Protein&list_uids=103472003&dopt=GenPept&RID=T2ZJKRC5012&log$=prottop&blast_rank=96) | forkhead box O4 isoform 1 [Homo sapiens] | [16.3](http://blast.ncbi.nlm.nih.gov/Blast.cgi#103472003%23103472003) | 16.3 | 66% | 1259 |
| [NP_056364.1](http://www.ncbi.nlm.nih.gov/entrez/query.fcgi?cmd=Retrieve&db=Protein&list_uids=55749621&dopt=GenPept&RID=T2ZJKRC5012&log$=prottop&blast_rank=97) | pleckstrin homology domain containing, family G, member 3 [Homo sapiens] | [16.3](http://blast.ncbi.nlm.nih.gov/Blast.cgi#55749621%2355749621) | 32.7 | 66% | 1259 |
| [NP_060915.2](http://www.ncbi.nlm.nih.gov/entrez/query.fcgi?cmd=Retrieve&db=Protein&list_uids=33285002&dopt=GenPept&RID=T2ZJKRC5012&log$=prottop&blast_rank=98) | selenoprotein S [Homo sapiens] >ref|NP_982298.1| selenoprotein S [Homo sapiens] | [16.3](http://blast.ncbi.nlm.nih.gov/Blast.cgi#33285002%2333285002) | 16.3 | 66% | 1259 |
| [NP_857634.1](http://www.ncbi.nlm.nih.gov/entrez/query.fcgi?cmd=Retrieve&db=Protein&list_uids=32455260&dopt=GenPept&RID=T2ZJKRC5012&log$=prottop&blast_rank=99) | peroxiredoxin 5 isoform b precursor [Homo sapiens] | [16.3](http://blast.ncbi.nlm.nih.gov/Blast.cgi#32455260%2332455260) | 16.3 | 66% | 1259 |
| [NP_857635.1](http://www.ncbi.nlm.nih.gov/entrez/query.fcgi?cmd=Retrieve&db=Protein&list_uids=32455262&dopt=GenPept&RID=T2ZJKRC5012&log$=prottop&blast_rank=100) | peroxiredoxin 5 isoform c precursor [Homo sapiens] | [16.3](http://blast.ncbi.nlm.nih.gov/Blast.cgi#32455262%2332455262) | 16.3 | 66% | 1259 |

| **Accession** | **Proteins with a match to PTRWGARLVK peptide** | **[Max score](http://blast.ncbi.nlm.nih.gov/Blast.cgi?CMD=Get&ALIGNMENTS=100&ALIGNMENT_VIEW=Pairwise&CDD_SEARCH_STATE=1&DATABASE_SORT=0&DESCRIPTIONS=100&ENTREZ_QUERY=txid9606 %5BORGN%5D&FIRST_QUERY_NUM=0&FORMAT_OBJECT=Alignment&FORMAT_PAGE_TARGET=&FORMAT_TYPE=HTML&GET_SEQUENCE=yes&I_THRESH=&MASK_CHAR=2&MASK_COLOR=1&NEW_DESIGN=on&NEW_VIEW=yes&NUM_OVERVIEW=100&OLD_BLAST=false&PAGE=Proteins&QUERY_INDEX=0&QUERY_NUMBER=0&RESULTS_PAGE_TARGET=&RID=T2ZRF97E012&SHOW_LINKOUT=yes&SHOW_OVERVIEW=yes&STEP_NUMBER=&WORD_SIZE=2&DISPLAY_SORT=1&HSP_SORT=1" \l "sort_mark)** | **[Total score](http://blast.ncbi.nlm.nih.gov/Blast.cgi?CMD=Get&ALIGNMENTS=100&ALIGNMENT_VIEW=Pairwise&CDD_SEARCH_STATE=1&DATABASE_SORT=0&DESCRIPTIONS=100&ENTREZ_QUERY=txid9606 %5BORGN%5D&FIRST_QUERY_NUM=0&FORMAT_OBJECT=Alignment&FORMAT_PAGE_TARGET=&FORMAT_TYPE=HTML&GET_SEQUENCE=yes&I_THRESH=&MASK_CHAR=2&MASK_COLOR=1&NEW_DESIGN=on&NEW_VIEW=yes&NUM_OVERVIEW=100&OLD_BLAST=false&PAGE=Proteins&QUERY_INDEX=0&QUERY_NUMBER=0&RESULTS_PAGE_TARGET=&RID=T2ZRF97E012&SHOW_LINKOUT=yes&SHOW_OVERVIEW=yes&STEP_NUMBER=&WORD_SIZE=2&DISPLAY_SORT=2&HSP_SORT=1" \l "sort_mark)** | **[Query coverage](http://blast.ncbi.nlm.nih.gov/Blast.cgi?CMD=Get&ALIGNMENTS=100&ALIGNMENT_VIEW=Pairwise&CDD_SEARCH_STATE=1&DATABASE_SORT=0&DESCRIPTIONS=100&ENTREZ_QUERY=txid9606 %5BORGN%5D&FIRST_QUERY_NUM=0&FORMAT_OBJECT=Alignment&FORMAT_PAGE_TARGET=&FORMAT_TYPE=HTML&GET_SEQUENCE=yes&I_THRESH=&MASK_CHAR=2&MASK_COLOR=1&NEW_DESIGN=on&NEW_VIEW=yes&NUM_OVERVIEW=100&OLD_BLAST=false&PAGE=Proteins&QUERY_INDEX=0&QUERY_NUMBER=0&RESULTS_PAGE_TARGET=&RID=T2ZRF97E012&SHOW_LINKOUT=yes&SHOW_OVERVIEW=yes&STEP_NUMBER=&WORD_SIZE=2&DISPLAY_SORT=4&HSP_SORT=0" \l "sort_mark)** | **[E value](http://blast.ncbi.nlm.nih.gov/Blast.cgi?CMD=Get&ALIGNMENTS=100&ALIGNMENT_VIEW=Pairwise&CDD_SEARCH_STATE=1&DATABASE_SORT=0&DESCRIPTIONS=100&ENTREZ_QUERY=txid9606 %5BORGN%5D&FIRST_QUERY_NUM=0&FORMAT_OBJECT=Alignment&FORMAT_PAGE_TARGET=&FORMAT_TYPE=HTML&GET_SEQUENCE=yes&I_THRESH=&MASK_CHAR=2&MASK_COLOR=1&NEW_DESIGN=on&NEW_VIEW=yes&NUM_OVERVIEW=100&OLD_BLAST=false&PAGE=Proteins&QUERY_INDEX=0&QUERY_NUMBER=0&RESULTS_PAGE_TARGET=&RID=T2ZRF97E012&SHOW_LINKOUT=yes&SHOW_OVERVIEW=yes&STEP_NUMBER=&WORD_SIZE=2&DISPLAY_SORT=0&HSP_SORT=0" \l "sort_mark)** |
| --- | --- | --- | --- | --- | --- |
| [NP_699188.1](http://www.ncbi.nlm.nih.gov/entrez/query.fcgi?cmd=Retrieve&db=Protein&list_uids=23503293&dopt=GenPept&RID=T2ZRF97E012&log$=prottop&blast_rank=1) | solute carrier family 16, member 11 [Homo sapiens] | [23.5](http://blast.ncbi.nlm.nih.gov/Blast.cgi#23503293%2323503293) | 52.4 | 80% | 14 |
| [XP_002342877.1](http://www.ncbi.nlm.nih.gov/entrez/query.fcgi?cmd=Retrieve&db=Protein&list_uids=239743507&dopt=GenPept&RID=T2ZRF97E012&log$=prottop&blast_rank=2) | PREDICTED: hypothetical protein XP_002342877 [Homo sapiens] >ref|XP_002347016.1| PREDICTED: hypothetical protein XP_002347016 [Homo sapiens] >ref|XP_002346123.1| PREDICTED: hypothetical protein [Homo sapiens] | [22.3](http://blast.ncbi.nlm.nih.gov/Blast.cgi#239743507%23239743507) | 22.3 | 80% | 34 |
| [NP_055334.2](http://www.ncbi.nlm.nih.gov/entrez/query.fcgi?cmd=Retrieve&db=Protein&list_uids=37574601&dopt=GenPept&RID=T2ZRF97E012&log$=prottop&blast_rank=3) | zinc finger protein 232 [Homo sapiens] | [22.3](http://blast.ncbi.nlm.nih.gov/Blast.cgi#37574601%2337574601) | 22.3 | 70% | 34 |
| [NP_001073375.1](http://www.ncbi.nlm.nih.gov/entrez/query.fcgi?cmd=Retrieve&db=Protein&list_uids=120952829&dopt=GenPept&RID=T2ZRF97E012&log$=prottop&blast_rank=4) | zinc finger protein 331 [Homo sapiens] >ref|NP_001073376.1| zinc finger protein 331 [Homo sapiens] >ref|NP_061025.5| zinc finger protein 331 [Homo sapiens] | [22.3](http://blast.ncbi.nlm.nih.gov/Blast.cgi#120952829%23120952829) | 41.6 | 80% | 34 |
| [XP_001726352.1](http://www.ncbi.nlm.nih.gov/entrez/query.fcgi?cmd=Retrieve&db=Protein&list_uids=169211031&dopt=GenPept&RID=T2ZRF97E012&log$=prottop&blast_rank=5) | PREDICTED: hypothetical protein [Homo sapiens] >ref|XP_002345081.1| PREDICTED: hypothetical protein [Homo sapiens] | [21.8](http://blast.ncbi.nlm.nih.gov/Blast.cgi#169211031%23169211031) | 21.8 | 70% | 46 |
| [XP_002343430.1](http://www.ncbi.nlm.nih.gov/entrez/query.fcgi?cmd=Retrieve&db=Protein&list_uids=239745342&dopt=GenPept&RID=T2ZRF97E012&log$=prottop&blast_rank=6) | PREDICTED: hypothetical protein XP_002343430 [Homo sapiens] | [20.6](http://blast.ncbi.nlm.nih.gov/Blast.cgi#239745342%23239745342) | 20.6 | 50% | 111 |
| [NP_663327.2](http://www.ncbi.nlm.nih.gov/entrez/query.fcgi?cmd=Retrieve&db=Protein&list_uids=145309306&dopt=GenPept&RID=T2ZRF97E012&log$=prottop&blast_rank=7) | scavenger receptor class F, member 1 isoform 5 precursor [Homo sapiens] | [20.6](http://blast.ncbi.nlm.nih.gov/Blast.cgi#145309306%23145309306) | 20.6 | 50% | 111 |
| [NP_060880.3](http://www.ncbi.nlm.nih.gov/entrez/query.fcgi?cmd=Retrieve&db=Protein&list_uids=83816964&dopt=GenPept&RID=T2ZRF97E012&log$=prottop&blast_rank=8) | Holliday junction recognition protein [Homo sapiens] | [20.6](http://blast.ncbi.nlm.nih.gov/Blast.cgi#83816964%2383816964) | 20.6 | 70% | 111 |
| [NP_699165.2](http://www.ncbi.nlm.nih.gov/entrez/query.fcgi?cmd=Retrieve&db=Protein&list_uids=24586659&dopt=GenPept&RID=T2ZRF97E012&log$=prottop&blast_rank=9) | scavenger receptor class F, member 2 isoform 1 [Homo sapiens] | [20.6](http://blast.ncbi.nlm.nih.gov/Blast.cgi#24586659%2324586659) | 45.2 | 70% | 111 |
| [NP_003684.2](http://www.ncbi.nlm.nih.gov/entrez/query.fcgi?cmd=Retrieve&db=Protein&list_uids=33598929&dopt=GenPept&RID=T2ZRF97E012&log$=prottop&blast_rank=10) | scavenger receptor class F, member 1 isoform 1 precursor [Homo sapiens] | [20.6](http://blast.ncbi.nlm.nih.gov/Blast.cgi#33598929%2333598929) | 20.6 | 50% | 111 |
| [NP_878315.1](http://www.ncbi.nlm.nih.gov/entrez/query.fcgi?cmd=Retrieve&db=Protein&list_uids=33598937&dopt=GenPept&RID=T2ZRF97E012&log$=prottop&blast_rank=11) | scavenger receptor class F, member 2 isoform 2 [Homo sapiens] | [20.6](http://blast.ncbi.nlm.nih.gov/Blast.cgi#33598937%2333598937) | 45.2 | 70% | 111 |
| [NP_663325.1](http://www.ncbi.nlm.nih.gov/entrez/query.fcgi?cmd=Retrieve&db=Protein&list_uids=33598931&dopt=GenPept&RID=T2ZRF97E012&log$=prottop&blast_rank=12) | scavenger receptor class F, member 1 isoform 3 precursor [Homo sapiens] | [20.6](http://blast.ncbi.nlm.nih.gov/Blast.cgi#33598931%2333598931) | 20.6 | 50% | 111 |
| [NP_110437.2](http://www.ncbi.nlm.nih.gov/entrez/query.fcgi?cmd=Retrieve&db=Protein&list_uids=42794771&dopt=GenPept&RID=T2ZRF97E012&log$=prottop&blast_rank=13) | thioredoxin domain containing 5 isoform 1 precursor [Homo sapiens] | [20.6](http://blast.ncbi.nlm.nih.gov/Blast.cgi#42794771%2342794771) | 20.6 | 50% | 111 |
| [XP_001718731.2](http://www.ncbi.nlm.nih.gov/entrez/query.fcgi?cmd=Retrieve&db=Protein&list_uids=239757641&dopt=GenPept&RID=T2ZRF97E012&log$=prottop&blast_rank=14) | PREDICTED: similar to hCG1646661 [Homo sapiens] | [20.2](http://blast.ncbi.nlm.nih.gov/Blast.cgi#239757641%23239757641) | 20.2 | 60% | 149 |
| [XP_001719433.2](http://www.ncbi.nlm.nih.gov/entrez/query.fcgi?cmd=Retrieve&db=Protein&list_uids=239746647&dopt=GenPept&RID=T2ZRF97E012&log$=prottop&blast_rank=15) | PREDICTED: similar to hCG1646661 [Homo sapiens] | [20.2](http://blast.ncbi.nlm.nih.gov/Blast.cgi#239746647%23239746647) | 20.2 | 60% | 149 |
| [XP_002343029.1](http://www.ncbi.nlm.nih.gov/entrez/query.fcgi?cmd=Retrieve&db=Protein&list_uids=239744088&dopt=GenPept&RID=T2ZRF97E012&log$=prottop&blast_rank=16) | PREDICTED: hypothetical protein XP_002343029 [Homo sapiens] | [20.2](http://blast.ncbi.nlm.nih.gov/Blast.cgi#239744088%23239744088) | 20.2 | 60% | 149 |
| [NP_001037858.1](http://www.ncbi.nlm.nih.gov/entrez/query.fcgi?cmd=Retrieve&db=Protein&list_uids=113206030&dopt=GenPept&RID=T2ZRF97E012&log$=prottop&blast_rank=17) | mucin 1 isoform 8 precursor [Homo sapiens] | [20.2](http://blast.ncbi.nlm.nih.gov/Blast.cgi#113206030%23113206030) | 20.2 | 50% | 149 |
| [XP_001719377.2](http://www.ncbi.nlm.nih.gov/entrez/query.fcgi?cmd=Retrieve&db=Protein&list_uids=239746634&dopt=GenPept&RID=T2ZRF97E012&log$=prottop&blast_rank=18) | PREDICTED: similar to hCG1646661 [Homo sapiens] >ref|XP_002348121.1| PREDICTED: similar to hCG1646661 [Homo sapiens] >ref|XP_002345505.1| PREDICTED: similar to hCG1646661 [Homo sapiens] | [20.2](http://blast.ncbi.nlm.nih.gov/Blast.cgi#239746634%23239746634) | 20.2 | 60% | 149 |
| [NP_055987.2](http://www.ncbi.nlm.nih.gov/entrez/query.fcgi?cmd=Retrieve&db=Protein&list_uids=115298682&dopt=GenPept&RID=T2ZRF97E012&log$=prottop&blast_rank=19) | HBxAg transactivated protein 2 [Homo sapiens] | [20.2](http://blast.ncbi.nlm.nih.gov/Blast.cgi#115298682%23115298682) | 20.2 | 60% | 149 |
| [NP_542417.2](http://www.ncbi.nlm.nih.gov/entrez/query.fcgi?cmd=Retrieve&db=Protein&list_uids=149158690&dopt=GenPept&RID=T2ZRF97E012&log$=prottop&blast_rank=20) | HLA-B associated transcript-2 [Homo sapiens] | [20.2](http://blast.ncbi.nlm.nih.gov/Blast.cgi#149158690%23149158690) | 20.2 | 60% | 149 |
| [NP_115513.3](http://www.ncbi.nlm.nih.gov/entrez/query.fcgi?cmd=Retrieve&db=Protein&list_uids=68163572&dopt=GenPept&RID=T2ZRF97E012&log$=prottop&blast_rank=21) | hypothetical protein LOC84077 [Homo sapiens] | [20.2](http://blast.ncbi.nlm.nih.gov/Blast.cgi#68163572%2368163572) | 32.7 | 90% | 149 |
| [NP_005123.2](http://www.ncbi.nlm.nih.gov/entrez/query.fcgi?cmd=Retrieve&db=Protein&list_uids=115430229&dopt=GenPept&RID=T2ZRF97E012&log$=prottop&blast_rank=22) | REC8 homolog [Homo sapiens] >ref|NP_001041670.1| REC8 homolog [Homo sapiens] | [20.2](http://blast.ncbi.nlm.nih.gov/Blast.cgi#115430229%23115430229) | 20.2 | 90% | 149 |
| [NP_620058.1](http://www.ncbi.nlm.nih.gov/entrez/query.fcgi?cmd=Retrieve&db=Protein&list_uids=23238231&dopt=GenPept&RID=T2ZRF97E012&log$=prottop&blast_rank=23) | high mobility group nucleosomal binding domain 3 isoform HMGN3b [Homo sapiens] | [20.2](http://blast.ncbi.nlm.nih.gov/Blast.cgi#23238231%2323238231) | 20.2 | 80% | 149 |
| [NP_004233.1](http://www.ncbi.nlm.nih.gov/entrez/query.fcgi?cmd=Retrieve&db=Protein&list_uids=20270186&dopt=GenPept&RID=T2ZRF97E012&log$=prottop&blast_rank=24) | high mobility group nucleosomal binding domain 3 isoform HMGN3a [Homo sapiens] | [20.2](http://blast.ncbi.nlm.nih.gov/Blast.cgi#20270186%2320270186) | 20.2 | 80% | 149 |
| [XP_002345107.1](http://www.ncbi.nlm.nih.gov/entrez/query.fcgi?cmd=Retrieve&db=Protein&list_uids=239756916&dopt=GenPept&RID=T2ZRF97E012&log$=prottop&blast_rank=25) | PREDICTED: hypothetical protein [Homo sapiens] | [19.7](http://blast.ncbi.nlm.nih.gov/Blast.cgi#239756916%23239756916) | 34.4 | 70% | 200 |
| [XP_002347839.1](http://www.ncbi.nlm.nih.gov/entrez/query.fcgi?cmd=Retrieve&db=Protein&list_uids=239751407&dopt=GenPept&RID=T2ZRF97E012&log$=prottop&blast_rank=26) | PREDICTED: hypothetical protein XP_002347839 [Homo sapiens] | [19.7](http://blast.ncbi.nlm.nih.gov/Blast.cgi#239751407%23239751407) | 34.4 | 70% | 200 |
| [XP_002343616.1](http://www.ncbi.nlm.nih.gov/entrez/query.fcgi?cmd=Retrieve&db=Protein&list_uids=239745917&dopt=GenPept&RID=T2ZRF97E012&log$=prottop&blast_rank=27) | PREDICTED: hypothetical protein XP_002343616 [Homo sapiens] | [19.7](http://blast.ncbi.nlm.nih.gov/Blast.cgi#239745917%23239745917) | 34.4 | 70% | 200 |
| [NP_787114.2](http://www.ncbi.nlm.nih.gov/entrez/query.fcgi?cmd=Retrieve&db=Protein&list_uids=154937359&dopt=GenPept&RID=T2ZRF97E012&log$=prottop&blast_rank=28) | cysteine-rich PAK1 inhibitor [Homo sapiens] | [19.7](http://blast.ncbi.nlm.nih.gov/Blast.cgi#154937359%23154937359) | 133 | 90% | 200 |
| [NP_060091.1](http://www.ncbi.nlm.nih.gov/entrez/query.fcgi?cmd=Retrieve&db=Protein&list_uids=8923019&dopt=GenPept&RID=T2ZRF97E012&log$=prottop&blast_rank=29) | alkB, alkylation repair homolog 4 [Homo sapiens] | [19.7](http://blast.ncbi.nlm.nih.gov/Blast.cgi#8923019%238923019) | 19.7 | 60% | 200 |
| [NP_001157918.1](http://www.ncbi.nlm.nih.gov/entrez/query.fcgi?cmd=Retrieve&db=Protein&list_uids=256574805&dopt=GenPept&RID=T2ZRF97E012&log$=prottop&blast_rank=30) | hypothetical protein LOC647024 [Homo sapiens] >ref|XP_946637.4| PREDICTED: chromosome 6 open reading frame 132 [Homo sapiens] >ref|XP_001724365.2| PREDICTED: chromosome 6 open reading frame 132 [Homo sapiens] | [19.3](http://blast.ncbi.nlm.nih.gov/Blast.cgi#256574805%23256574805) | 19.3 | 80% | 268 |
| [NP_065910.3](http://www.ncbi.nlm.nih.gov/entrez/query.fcgi?cmd=Retrieve&db=Protein&list_uids=203098098&dopt=GenPept&RID=T2ZRF97E012&log$=prottop&blast_rank=31) | shroom family member 3 protein [Homo sapiens] | [19.3](http://blast.ncbi.nlm.nih.gov/Blast.cgi#203098098%23203098098) | 19.3 | 70% | 268 |
| [XP_001720570.1](http://www.ncbi.nlm.nih.gov/entrez/query.fcgi?cmd=Retrieve&db=Protein&list_uids=169173146&dopt=GenPept&RID=T2ZRF97E012&log$=prottop&blast_rank=32) | PREDICTED: hypothetical protein [Homo sapiens] | [19.3](http://blast.ncbi.nlm.nih.gov/Blast.cgi#169173146%23169173146) | 19.3 | 90% | 268 |
| [XP_001726997.1](http://www.ncbi.nlm.nih.gov/entrez/query.fcgi?cmd=Retrieve&db=Protein&list_uids=169172766&dopt=GenPept&RID=T2ZRF97E012&log$=prottop&blast_rank=33) | PREDICTED: hypothetical protein LOC389630 [Homo sapiens] | [19.3](http://blast.ncbi.nlm.nih.gov/Blast.cgi#169172766%23169172766) | 19.3 | 90% | 268 |
| [NP_001138673.1](http://www.ncbi.nlm.nih.gov/entrez/query.fcgi?cmd=Retrieve&db=Protein&list_uids=223633957&dopt=GenPept&RID=T2ZRF97E012&log$=prottop&blast_rank=34) | proline-rich acidic protein 1 isoform 2 [Homo sapiens] | [19.3](http://blast.ncbi.nlm.nih.gov/Blast.cgi#223633957%23223633957) | 19.3 | 60% | 268 |
| [NP_660203.3](http://www.ncbi.nlm.nih.gov/entrez/query.fcgi?cmd=Retrieve&db=Protein&list_uids=223633959&dopt=GenPept&RID=T2ZRF97E012&log$=prottop&blast_rank=35) | proline-rich acidic protein 1 isoform 1 [Homo sapiens] | [19.3](http://blast.ncbi.nlm.nih.gov/Blast.cgi#223633959%23223633959) | 19.3 | 60% | 268 |
| [NP_001157925.1](http://www.ncbi.nlm.nih.gov/entrez/query.fcgi?cmd=Retrieve&db=Protein&list_uids=256574822&dopt=GenPept&RID=T2ZRF97E012&log$=prottop&blast_rank=36) | hypothetical protein LOC728430 [Homo sapiens] | [19.3](http://blast.ncbi.nlm.nih.gov/Blast.cgi#256574822%23256574822) | 19.3 | 90% | 268 |
| [NP_000447.2](http://www.ncbi.nlm.nih.gov/entrez/query.fcgi?cmd=Retrieve&db=Protein&list_uids=74099694&dopt=GenPept&RID=T2ZRF97E012&log$=prottop&blast_rank=37) | sulfite oxidase precursor [Homo sapiens] >ref|NP_001027558.1| sulfite oxidase precursor [Homo sapiens] >ref|NP_001027559.1| sulfite oxidase precursor [Homo sapiens] | [19.3](http://blast.ncbi.nlm.nih.gov/Blast.cgi#74099694%2374099694) | 34.4 | 80% | 268 |
| [NP_002903.3](http://www.ncbi.nlm.nih.gov/entrez/query.fcgi?cmd=Retrieve&db=Protein&list_uids=153792012&dopt=GenPept&RID=T2ZRF97E012&log$=prottop&blast_rank=38) | DNA polymerase zeta [Homo sapiens] | [19.3](http://blast.ncbi.nlm.nih.gov/Blast.cgi#153792012%23153792012) | 19.3 | 60% | 268 |
| [NP_056114.1](http://www.ncbi.nlm.nih.gov/entrez/query.fcgi?cmd=Retrieve&db=Protein&list_uids=54792090&dopt=GenPept&RID=T2ZRF97E012&log$=prottop&blast_rank=39) | KH and NYN domain containing [Homo sapiens] | [19.3](http://blast.ncbi.nlm.nih.gov/Blast.cgi#54792090%2354792090) | 19.3 | 70% | 268 |
| [NP_997227.1](http://www.ncbi.nlm.nih.gov/entrez/query.fcgi?cmd=Retrieve&db=Protein&list_uids=46409324&dopt=GenPept&RID=T2ZRF97E012&log$=prottop&blast_rank=40) | SPRY domain containing 4 [Homo sapiens] | [19.3](http://blast.ncbi.nlm.nih.gov/Blast.cgi#46409324%2346409324) | 19.3 | 60% | 268 |
| [NP_001157924.1](http://www.ncbi.nlm.nih.gov/entrez/query.fcgi?cmd=Retrieve&db=Protein&list_uids=256574820&dopt=GenPept&RID=T2ZRF97E012&log$=prottop&blast_rank=41) | hypothetical protein LOC645651 [Homo sapiens] | [19.3](http://blast.ncbi.nlm.nih.gov/Blast.cgi#256574820%23256574820) | 19.3 | 90% | 268 |
| [NP_001157927.1](http://www.ncbi.nlm.nih.gov/entrez/query.fcgi?cmd=Retrieve&db=Protein&list_uids=256574826&dopt=GenPept&RID=T2ZRF97E012&log$=prottop&blast_rank=42) | hypothetical protein LOC441315 [Homo sapiens] | [19.3](http://blast.ncbi.nlm.nih.gov/Blast.cgi#256574826%23256574826) | 19.3 | 90% | 268 |
| [NP_001157928.1](http://www.ncbi.nlm.nih.gov/entrez/query.fcgi?cmd=Retrieve&db=Protein&list_uids=256574828&dopt=GenPept&RID=T2ZRF97E012&log$=prottop&blast_rank=43) | hypothetical protein LOC441314 [Homo sapiens] | [19.3](http://blast.ncbi.nlm.nih.gov/Blast.cgi#256574828%23256574828) | 19.3 | 90% | 268 |
| [XP_372013.2](http://www.ncbi.nlm.nih.gov/entrez/query.fcgi?cmd=Retrieve&db=Protein&list_uids=42658817&dopt=GenPept&RID=T2ZRF97E012&log$=prottop&blast_rank=44) | PREDICTED: hypothetical protein [Homo sapiens] | [19.3](http://blast.ncbi.nlm.nih.gov/Blast.cgi#42658817%2342658817) | 19.3 | 90% | 268 |
| [NP_005693.1](http://www.ncbi.nlm.nih.gov/entrez/query.fcgi?cmd=Retrieve&db=Protein&list_uids=24307899&dopt=GenPept&RID=T2ZRF97E012&log$=prottop&blast_rank=45) | Era-like 1 [Homo sapiens] | [19.3](http://blast.ncbi.nlm.nih.gov/Blast.cgi#24307899%2324307899) | 19.3 | 90% | 268 |
| [NP_149043.2](http://www.ncbi.nlm.nih.gov/entrez/query.fcgi?cmd=Retrieve&db=Protein&list_uids=239582755&dopt=GenPept&RID=T2ZRF97E012&log$=prottop&blast_rank=46) | myosin IG [Homo sapiens] | [18.9](http://blast.ncbi.nlm.nih.gov/Blast.cgi#239582755%23239582755) | 18.9 | 80% | 360 |
| [NP_862828.1](http://www.ncbi.nlm.nih.gov/entrez/query.fcgi?cmd=Retrieve&db=Protein&list_uids=149588643&dopt=GenPept&RID=T2ZRF97E012&log$=prottop&blast_rank=47) | zinc finger protein 283 [Homo sapiens] | [18.9](http://blast.ncbi.nlm.nih.gov/Blast.cgi#149588643%23149588643) | 56.6 | 70% | 360 |
| [NP_001010982.2](http://www.ncbi.nlm.nih.gov/entrez/query.fcgi?cmd=Retrieve&db=Protein&list_uids=192807282&dopt=GenPept&RID=T2ZRF97E012&log$=prottop&blast_rank=48) | arylformamidase isoform 1 [Homo sapiens] | [18.9](http://blast.ncbi.nlm.nih.gov/Blast.cgi#192807282%23192807282) | 18.9 | 80% | 360 |
| [NP_001138998.1](http://www.ncbi.nlm.nih.gov/entrez/query.fcgi?cmd=Retrieve&db=Protein&list_uids=224465228&dopt=GenPept&RID=T2ZRF97E012&log$=prottop&blast_rank=49) | arylformamidase isoform 2 [Homo sapiens] | [18.9](http://blast.ncbi.nlm.nih.gov/Blast.cgi#224465228%23224465228) | 18.9 | 80% | 360 |
| [NP_055627.2](http://www.ncbi.nlm.nih.gov/entrez/query.fcgi?cmd=Retrieve&db=Protein&list_uids=109255228&dopt=GenPept&RID=T2ZRF97E012&log$=prottop&blast_rank=50) | centrosomal protein 170kDa isoform alpha [Homo sapiens] | [18.9](http://blast.ncbi.nlm.nih.gov/Blast.cgi#109255228%23109255228) | 18.9 | 70% | 360 |
| [NP_001035863.1](http://www.ncbi.nlm.nih.gov/entrez/query.fcgi?cmd=Retrieve&db=Protein&list_uids=109255230&dopt=GenPept&RID=T2ZRF97E012&log$=prottop&blast_rank=51) | centrosomal protein 170kDa isoform beta [Homo sapiens] | [18.9](http://blast.ncbi.nlm.nih.gov/Blast.cgi#109255230%23109255230) | 18.9 | 70% | 360 |
| [NP_001035864.1](http://www.ncbi.nlm.nih.gov/entrez/query.fcgi?cmd=Retrieve&db=Protein&list_uids=109255232&dopt=GenPept&RID=T2ZRF97E012&log$=prottop&blast_rank=52) | centrosomal protein 170kDa isoform gamma [Homo sapiens] | [18.9](http://blast.ncbi.nlm.nih.gov/Blast.cgi#109255232%23109255232) | 18.9 | 70% | 360 |
| [NP_004841.2](http://www.ncbi.nlm.nih.gov/entrez/query.fcgi?cmd=Retrieve&db=Protein&list_uids=41872583&dopt=GenPept&RID=T2ZRF97E012&log$=prottop&blast_rank=53) | Rho-associated, coiled-coil containing protein kinase 2 [Homo sapiens] | [18.9](http://blast.ncbi.nlm.nih.gov/Blast.cgi#41872583%2341872583) | 18.9 | 70% | 360 |
| [NP_001124499.1](http://www.ncbi.nlm.nih.gov/entrez/query.fcgi?cmd=Retrieve&db=Protein&list_uids=196162711&dopt=GenPept&RID=T2ZRF97E012&log$=prottop&blast_rank=54) | PDZ and LIM domain 4 isoform 2 [Homo sapiens] | [18.9](http://blast.ncbi.nlm.nih.gov/Blast.cgi#196162711%23196162711) | 18.9 | 90% | 360 |
| [NP_057342.2](http://www.ncbi.nlm.nih.gov/entrez/query.fcgi?cmd=Retrieve&db=Protein&list_uids=116812575&dopt=GenPept&RID=T2ZRF97E012&log$=prottop&blast_rank=55) | high glucose-regulated protein 8 [Homo sapiens] | [18.9](http://blast.ncbi.nlm.nih.gov/Blast.cgi#116812575%23116812575) | 18.9 | 60% | 360 |
| [NP_003678.2](http://www.ncbi.nlm.nih.gov/entrez/query.fcgi?cmd=Retrieve&db=Protein&list_uids=19923181&dopt=GenPept&RID=T2ZRF97E012&log$=prottop&blast_rank=56) | PDZ and LIM domain 4 isoform 1 [Homo sapiens] | [18.9](http://blast.ncbi.nlm.nih.gov/Blast.cgi#19923181%2319923181) | 18.9 | 90% | 360 |
| [NP_076936.1](http://www.ncbi.nlm.nih.gov/entrez/query.fcgi?cmd=Retrieve&db=Protein&list_uids=13128980&dopt=GenPept&RID=T2ZRF97E012&log$=prottop&blast_rank=57) | proline rich 14 [Homo sapiens] | [18.9](http://blast.ncbi.nlm.nih.gov/Blast.cgi#13128980%2313128980) | 18.9 | 60% | 360 |
| [NP_001138984.1](http://www.ncbi.nlm.nih.gov/entrez/query.fcgi?cmd=Retrieve&db=Protein&list_uids=224465184&dopt=GenPept&RID=T2ZRF97E012&log$=prottop&blast_rank=58) | nuclear factor I/A isoform 4 [Homo sapiens] | [18.5](http://blast.ncbi.nlm.nih.gov/Blast.cgi#224465184%23224465184) | 49.8 | 100% | 482 |
| [NP_848658.3](http://www.ncbi.nlm.nih.gov/entrez/query.fcgi?cmd=Retrieve&db=Protein&list_uids=222352158&dopt=GenPept&RID=T2ZRF97E012&log$=prottop&blast_rank=59) | carboxypeptidase 3, cytosolic [Homo sapiens] | [18.5](http://blast.ncbi.nlm.nih.gov/Blast.cgi#222352158%23222352158) | 33.9 | 80% | 482 |
| [XP_002343445.1](http://www.ncbi.nlm.nih.gov/entrez/query.fcgi?cmd=Retrieve&db=Protein&list_uids=239745356&dopt=GenPept&RID=T2ZRF97E012&log$=prottop&blast_rank=60) | PREDICTED: hypothetical protein XP_002343445 [Homo sapiens] >ref|XP_002347615.1| PREDICTED: hypothetical protein XP_002347615 [Homo sapiens] >ref|XP_002344980.1| PREDICTED: hypothetical protein [Homo sapiens] | [18.5](http://blast.ncbi.nlm.nih.gov/Blast.cgi#239745356%23239745356) | 18.5 | 60% | 482 |
| [XP_947146.3](http://www.ncbi.nlm.nih.gov/entrez/query.fcgi?cmd=Retrieve&db=Protein&list_uids=169217928&dopt=GenPept&RID=T2ZRF97E012&log$=prottop&blast_rank=61) | PREDICTED: similar to piwi like homolog 2 [Homo sapiens] | [18.5](http://blast.ncbi.nlm.nih.gov/Blast.cgi#169217928%23169217928) | 18.5 | 60% | 482 |
| [XP_001715475.1](http://www.ncbi.nlm.nih.gov/entrez/query.fcgi?cmd=Retrieve&db=Protein&list_uids=169173184&dopt=GenPept&RID=T2ZRF97E012&log$=prottop&blast_rank=62) | PREDICTED: hypothetical protein [Homo sapiens] | [18.5](http://blast.ncbi.nlm.nih.gov/Blast.cgi#169173184%23169173184) | 18.5 | 40% | 482 |
| [XP_001714827.1](http://www.ncbi.nlm.nih.gov/entrez/query.fcgi?cmd=Retrieve&db=Protein&list_uids=169168219&dopt=GenPept&RID=T2ZRF97E012&log$=prottop&blast_rank=63) | PREDICTED: hypothetical protein [Homo sapiens] | [18.5](http://blast.ncbi.nlm.nih.gov/Blast.cgi#169168219%23169168219) | 18.5 | 40% | 482 |
| [XP_001716040.1](http://www.ncbi.nlm.nih.gov/entrez/query.fcgi?cmd=Retrieve&db=Protein&list_uids=169167696&dopt=GenPept&RID=T2ZRF97E012&log$=prottop&blast_rank=64) | PREDICTED: hypothetical protein [Homo sapiens] | [18.5](http://blast.ncbi.nlm.nih.gov/Blast.cgi#169167696%23169167696) | 18.5 | 40% | 482 |
| [NP_061985.2](http://www.ncbi.nlm.nih.gov/entrez/query.fcgi?cmd=Retrieve&db=Protein&list_uids=150417984&dopt=GenPept&RID=T2ZRF97E012&log$=prottop&blast_rank=65) | ATP-binding cassette, sub-family A, member 7 [Homo sapiens] | [18.5](http://blast.ncbi.nlm.nih.gov/Blast.cgi#150417984%23150417984) | 29.3 | 90% | 482 |
| [NP_001093243.1](http://www.ncbi.nlm.nih.gov/entrez/query.fcgi?cmd=Retrieve&db=Protein&list_uids=153218654&dopt=GenPept&RID=T2ZRF97E012&log$=prottop&blast_rank=66) | cytochrome P450, family 11, subfamily A, polypeptide 1 isoform b [Homo sapiens] | [18.5](http://blast.ncbi.nlm.nih.gov/Blast.cgi#153218654%23153218654) | 18.5 | 40% | 482 |
| [NP_001073898.1](http://www.ncbi.nlm.nih.gov/entrez/query.fcgi?cmd=Retrieve&db=Protein&list_uids=122937255&dopt=GenPept&RID=T2ZRF97E012&log$=prottop&blast_rank=67) | NEZHA isoform 1 [Homo sapiens] | [18.5](http://blast.ncbi.nlm.nih.gov/Blast.cgi#122937255%23122937255) | 18.5 | 40% | 482 |
| [NP_001157940.1](http://www.ncbi.nlm.nih.gov/entrez/query.fcgi?cmd=Retrieve&db=Protein&list_uids=256985100&dopt=GenPept&RID=T2ZRF97E012&log$=prottop&blast_rank=68) | TRAM adaptor with GOLD domain isoform 1 [Homo sapiens] | [18.5](http://blast.ncbi.nlm.nih.gov/Blast.cgi#256985100%23256985100) | 18.5 | 60% | 482 |
| [NP_840101.1](http://www.ncbi.nlm.nih.gov/entrez/query.fcgi?cmd=Retrieve&db=Protein&list_uids=77404438&dopt=GenPept&RID=T2ZRF97E012&log$=prottop&blast_rank=69) | snail homolog 3 [Homo sapiens] | [18.5](http://blast.ncbi.nlm.nih.gov/Blast.cgi#77404438%2377404438) | 18.5 | 40% | 482 |
| [NP_116222.3](http://www.ncbi.nlm.nih.gov/entrez/query.fcgi?cmd=Retrieve&db=Protein&list_uids=188219559&dopt=GenPept&RID=T2ZRF97E012&log$=prottop&blast_rank=70) | protein phosphatase 1, regulatory subunit 15B [Homo sapiens] | [18.5](http://blast.ncbi.nlm.nih.gov/Blast.cgi#188219559%23188219559) | 18.5 | 40% | 482 |
| [NP_031376.3](http://www.ncbi.nlm.nih.gov/entrez/query.fcgi?cmd=Retrieve&db=Protein&list_uids=83977459&dopt=GenPept&RID=T2ZRF97E012&log$=prottop&blast_rank=71) | pleckstrin homology-like domain, family A, member 1 [Homo sapiens] | [18.5](http://blast.ncbi.nlm.nih.gov/Blast.cgi#83977459%2383977459) | 18.5 | 60% | 482 |
| [NP_705872.1](http://www.ncbi.nlm.nih.gov/entrez/query.fcgi?cmd=Retrieve&db=Protein&list_uids=24234744&dopt=GenPept&RID=T2ZRF97E012&log$=prottop&blast_rank=72) | semaphorin 6D isoform 5 precursor [Homo sapiens] | [18.5](http://blast.ncbi.nlm.nih.gov/Blast.cgi#24234744%2324234744) | 18.5 | 40% | 482 |
| [NP_000772.2](http://www.ncbi.nlm.nih.gov/entrez/query.fcgi?cmd=Retrieve&db=Protein&list_uids=153218646&dopt=GenPept&RID=T2ZRF97E012&log$=prottop&blast_rank=73) | cytochrome P450, family 11, subfamily A, polypeptide 1 isoform a precursor [Homo sapiens] | [18.5](http://blast.ncbi.nlm.nih.gov/Blast.cgi#153218646%23153218646) | 18.5 | 40% | 482 |
| [NP_689617.3](http://www.ncbi.nlm.nih.gov/entrez/query.fcgi?cmd=Retrieve&db=Protein&list_uids=288541302&dopt=GenPept&RID=T2ZRF97E012&log$=prottop&blast_rank=74) | UDP glycosyltransferase 3 family, polypeptide A1 isoform 1 precursor [Homo sapiens] | [18.5](http://blast.ncbi.nlm.nih.gov/Blast.cgi#288541302%23288541302) | 18.5 | 80% | 482 |
| [NP_002804.2](http://www.ncbi.nlm.nih.gov/entrez/query.fcgi?cmd=Retrieve&db=Protein&list_uids=18543329&dopt=GenPept&RID=T2ZRF97E012&log$=prottop&blast_rank=75) | proteasome 26S non-ATPase subunit 9 [Homo sapiens] | [18.5](http://blast.ncbi.nlm.nih.gov/Blast.cgi#18543329%2318543329) | 18.5 | 40% | 482 |
| [NP_006246.2](http://www.ncbi.nlm.nih.gov/entrez/query.fcgi?cmd=Retrieve&db=Protein&list_uids=28557781&dopt=GenPept&RID=T2ZRF97E012&log$=prottop&blast_rank=76) | protein kinase C, eta [Homo sapiens] | [18.5](http://blast.ncbi.nlm.nih.gov/Blast.cgi#28557781%2328557781) | 18.5 | 40% | 482 |
| [NP_079334.3](http://www.ncbi.nlm.nih.gov/entrez/query.fcgi?cmd=Retrieve&db=Protein&list_uids=47174859&dopt=GenPept&RID=T2ZRF97E012&log$=prottop&blast_rank=77) | tripartite motif-containing 46 [Homo sapiens] | [18.5](http://blast.ncbi.nlm.nih.gov/Blast.cgi#47174859%2347174859) | 36.9 | 80% | 482 |
| [NP_861974.1](http://www.ncbi.nlm.nih.gov/entrez/query.fcgi?cmd=Retrieve&db=Protein&list_uids=32996709&dopt=GenPept&RID=T2ZRF97E012&log$=prottop&blast_rank=78) | transmembrane emp24 protein transport domain containing 7 precursor [Homo sapiens] | [18.5](http://blast.ncbi.nlm.nih.gov/Blast.cgi#32996709%2332996709) | 18.5 | 60% | 482 |
| [NP_062565.2](http://www.ncbi.nlm.nih.gov/entrez/query.fcgi?cmd=Retrieve&db=Protein&list_uids=21361831&dopt=GenPept&RID=T2ZRF97E012&log$=prottop&blast_rank=79) | partitioning-defective protein 3 homolog [Homo sapiens] | [18.5](http://blast.ncbi.nlm.nih.gov/Blast.cgi#21361831%2321361831) | 18.5 | 40% | 482 |
| [NP_000119.1](http://www.ncbi.nlm.nih.gov/entrez/query.fcgi?cmd=Retrieve&db=Protein&list_uids=4503627&dopt=GenPept&RID=T2ZRF97E012&log$=prottop&blast_rank=80) | coagulation factor XI precursor [Homo sapiens] | [18.5](http://blast.ncbi.nlm.nih.gov/Blast.cgi#4503627%234503627) | 18.5 | 40% | 482 |
| [NP_060538.2](http://www.ncbi.nlm.nih.gov/entrez/query.fcgi?cmd=Retrieve&db=Protein&list_uids=24431985&dopt=GenPept&RID=T2ZRF97E012&log$=prottop&blast_rank=81) | piwi-like 2 [Homo sapiens] >ref|NP_001129193.1| piwi-like 2 [Homo sapiens] | [18.5](http://blast.ncbi.nlm.nih.gov/Blast.cgi#24431985%2324431985) | 18.5 | 60% | 482 |
| [NP_001157941.1](http://www.ncbi.nlm.nih.gov/entrez/query.fcgi?cmd=Retrieve&db=Protein&list_uids=256985102&dopt=GenPept&RID=T2ZRF97E012&log$=prottop&blast_rank=82) | TRAM adaptor with GOLD domain isoform 2 [Homo sapiens] | [18.5](http://blast.ncbi.nlm.nih.gov/Blast.cgi#256985102%23256985102) | 18.5 | 60% | 482 |
| [NP_005435.1](http://www.ncbi.nlm.nih.gov/entrez/query.fcgi?cmd=Retrieve&db=Protein&list_uids=4885579&dopt=GenPept&RID=T2ZRF97E012&log$=prottop&blast_rank=83) | RCD1 required for cell differentiation1 homolog [Homo sapiens] | [18.5](http://blast.ncbi.nlm.nih.gov/Blast.cgi#4885579%234885579) | 18.5 | 80% | 482 |
| [NP_001128348.1](http://www.ncbi.nlm.nih.gov/entrez/query.fcgi?cmd=Retrieve&db=Protein&list_uids=199560438&dopt=GenPept&RID=T2ZRF97E012&log$=prottop&blast_rank=84) | hypothetical protein LOC283643 isoform 2 [Homo sapiens] | [18.0](http://blast.ncbi.nlm.nih.gov/Blast.cgi#199560438%23199560438) | 33.1 | 50% | 647 |
| [NP_001128347.1](http://www.ncbi.nlm.nih.gov/entrez/query.fcgi?cmd=Retrieve&db=Protein&list_uids=199560355&dopt=GenPept&RID=T2ZRF97E012&log$=prottop&blast_rank=85) | hypothetical protein LOC283643 isoform 1 [Homo sapiens] | [18.0](http://blast.ncbi.nlm.nih.gov/Blast.cgi#199560355%23199560355) | 33.1 | 50% | 647 |
| [NP_001128349.1](http://www.ncbi.nlm.nih.gov/entrez/query.fcgi?cmd=Retrieve&db=Protein&list_uids=199560536&dopt=GenPept&RID=T2ZRF97E012&log$=prottop&blast_rank=86) | hypothetical protein LOC283643 isoform 4 [Homo sapiens] | [18.0](http://blast.ncbi.nlm.nih.gov/Blast.cgi#199560536%23199560536) | 33.1 | 50% | 647 |
| [NP_001164106.1](http://www.ncbi.nlm.nih.gov/entrez/query.fcgi?cmd=Retrieve&db=Protein&list_uids=282400944&dopt=GenPept&RID=T2ZRF97E012&log$=prottop&blast_rank=87) | lipid phosphate phosphatase-related protein type 2 isoform 2 [Homo sapiens] | [18.0](http://blast.ncbi.nlm.nih.gov/Blast.cgi#282400944%23282400944) | 18.0 | 60% | 647 |
| [NP_055738.3](http://www.ncbi.nlm.nih.gov/entrez/query.fcgi?cmd=Retrieve&db=Protein&list_uids=118918397&dopt=GenPept&RID=T2ZRF97E012&log$=prottop&blast_rank=88) | fibronectin type III domain containing 3A isoform 2 [Homo sapiens] | [18.0](http://blast.ncbi.nlm.nih.gov/Blast.cgi#118918397%23118918397) | 18.0 | 70% | 647 |
| [NP_203754.2](http://www.ncbi.nlm.nih.gov/entrez/query.fcgi?cmd=Retrieve&db=Protein&list_uids=110556636&dopt=GenPept&RID=T2ZRF97E012&log$=prottop&blast_rank=89) | tankyrase 1-binding protein 1 [Homo sapiens] | [18.0](http://blast.ncbi.nlm.nih.gov/Blast.cgi#110556636%23110556636) | 18.0 | 80% | 647 |
| [NP_001026997.1](http://www.ncbi.nlm.nih.gov/entrez/query.fcgi?cmd=Retrieve&db=Protein&list_uids=73622130&dopt=GenPept&RID=T2ZRF97E012&log$=prottop&blast_rank=90) | BolA-like protein 2 [Homo sapiens] >ref|NP_001034271.1| bolA-like protein 2B [Homo sapiens] | [18.0](http://blast.ncbi.nlm.nih.gov/Blast.cgi#73622130%2373622130) | 18.0 | 60% | 647 |
| [NP_055926.1](http://www.ncbi.nlm.nih.gov/entrez/query.fcgi?cmd=Retrieve&db=Protein&list_uids=62177129&dopt=GenPept&RID=T2ZRF97E012&log$=prottop&blast_rank=91) | Nedd4 binding protein 3 [Homo sapiens] | [18.0](http://blast.ncbi.nlm.nih.gov/Blast.cgi#62177129%2362177129) | 18.0 | 80% | 647 |
| [NP_060707.2](http://www.ncbi.nlm.nih.gov/entrez/query.fcgi?cmd=Retrieve&db=Protein&list_uids=46852388&dopt=GenPept&RID=T2ZRF97E012&log$=prottop&blast_rank=92) | cell-cycle and apoptosis regulatory protein 1 [Homo sapiens] | [18.0](http://blast.ncbi.nlm.nih.gov/Blast.cgi#46852388%2346852388) | 32.2 | 90% | 647 |
| [NP_658985.2](http://www.ncbi.nlm.nih.gov/entrez/query.fcgi?cmd=Retrieve&db=Protein&list_uids=91984773&dopt=GenPept&RID=T2ZRF97E012&log$=prottop&blast_rank=93) | apolipoprotein A-I binding protein precursor [Homo sapiens] | [18.0](http://blast.ncbi.nlm.nih.gov/Blast.cgi#91984773%2391984773) | 18.0 | 80% | 647 |
| [NP_653188.2](http://www.ncbi.nlm.nih.gov/entrez/query.fcgi?cmd=Retrieve&db=Protein&list_uids=50345833&dopt=GenPept&RID=T2ZRF97E012&log$=prottop&blast_rank=94) | BTB (POZ) domain containing 16 [Homo sapiens] | [18.0](http://blast.ncbi.nlm.nih.gov/Blast.cgi#50345833%2350345833) | 18.0 | 50% | 647 |
| [XP_937998.1](http://www.ncbi.nlm.nih.gov/entrez/query.fcgi?cmd=Retrieve&db=Protein&list_uids=89058207&dopt=GenPept&RID=T2ZRF97E012&log$=prottop&blast_rank=95) | PREDICTED: hypothetical protein [Homo sapiens] >ref|XP_950434.1| PREDICTED: hypothetical protein [Homo sapiens] >ref|XP_001714468.1| PREDICTED: hypothetical protein [Homo sapiens] | [18.0](http://blast.ncbi.nlm.nih.gov/Blast.cgi#89058207%2389058207) | 18.0 | 60% | 647 |
| [NP_001032626.1](http://www.ncbi.nlm.nih.gov/entrez/query.fcgi?cmd=Retrieve&db=Protein&list_uids=83281447&dopt=GenPept&RID=T2ZRF97E012&log$=prottop&blast_rank=96) | phytanoyl-CoA 2-hydroxylase isoform b precursor [Homo sapiens] | [18.0](http://blast.ncbi.nlm.nih.gov/Blast.cgi#83281447%2383281447) | 18.0 | 50% | 647 |
| [NP_001034930.1](http://www.ncbi.nlm.nih.gov/entrez/query.fcgi?cmd=Retrieve&db=Protein&list_uids=89886350&dopt=GenPept&RID=T2ZRF97E012&log$=prottop&blast_rank=97) | Rho GTPase activating protein 11B [Homo sapiens] | [18.0](http://blast.ncbi.nlm.nih.gov/Blast.cgi#89886350%2389886350) | 18.0 | 70% | 647 |
| [NP_777601.2](http://www.ncbi.nlm.nih.gov/entrez/query.fcgi?cmd=Retrieve&db=Protein&list_uids=50659091&dopt=GenPept&RID=T2ZRF97E012&log$=prottop&blast_rank=98) | scavenger receptor cysteine-rich type 1 precursor [Homo sapiens] | [18.0](http://blast.ncbi.nlm.nih.gov/Blast.cgi#50659091%2350659091) | 18.0 | 60% | 647 |
| [NP_000084.3](http://www.ncbi.nlm.nih.gov/entrez/query.fcgi?cmd=Retrieve&db=Protein&list_uids=89276751&dopt=GenPept&RID=T2ZRF97E012&log$=prottop&blast_rank=99) | alpha 1 type V collagen preproprotein [Homo sapiens] | [18.0](http://blast.ncbi.nlm.nih.gov/Blast.cgi#89276751%2389276751) | 18.0 | 60% | 647 |
| [NP_006205.1](http://www.ncbi.nlm.nih.gov/entrez/query.fcgi?cmd=Retrieve&db=Protein&list_uids=5453884&dopt=GenPept&RID=T2ZRF97E012&log$=prottop&blast_rank=100) | phytanoyl-CoA 2-hydroxylase isoform a precursor [Homo sapiens] | [18.0](http://blast.ncbi.nlm.nih.gov/Blast.cgi#5453884%235453884) | 18.0 | 50% | 647 |

| **Accession** | **Proteins with a match to GGRWNR peptide** | **[Max score](http://blast.ncbi.nlm.nih.gov/Blast.cgi?CMD=Get&ALIGNMENTS=100&ALIGNMENT_VIEW=Pairwise&CDD_SEARCH_STATE=1&DATABASE_SORT=0&DESCRIPTIONS=100&ENTREZ_QUERY=txid9606 %5BORGN%5D&FIRST_QUERY_NUM=0&FORMAT_OBJECT=Alignment&FORMAT_PAGE_TARGET=&FORMAT_TYPE=HTML&GET_SEQUENCE=yes&I_THRESH=&MASK_CHAR=2&MASK_COLOR=1&NEW_VIEW=yes&NUM_OVERVIEW=100&OLD_BLAST=false&PAGE=Proteins&QUERY_INDEX=0&QUERY_NUMBER=0&RESULTS_PAGE_TARGET=&RID=TAJX8JRE01N&SHOW_LINKOUT=yes&SHOW_OVERVIEW=yes&STEP_NUMBER=&WORD_SIZE=2&DISPLAY_SORT=1&HSP_SORT=1" \l "sort_mark)** | **[Total score](http://blast.ncbi.nlm.nih.gov/Blast.cgi?CMD=Get&ALIGNMENTS=100&ALIGNMENT_VIEW=Pairwise&CDD_SEARCH_STATE=1&DATABASE_SORT=0&DESCRIPTIONS=100&ENTREZ_QUERY=txid9606 %5BORGN%5D&FIRST_QUERY_NUM=0&FORMAT_OBJECT=Alignment&FORMAT_PAGE_TARGET=&FORMAT_TYPE=HTML&GET_SEQUENCE=yes&I_THRESH=&MASK_CHAR=2&MASK_COLOR=1&NEW_VIEW=yes&NUM_OVERVIEW=100&OLD_BLAST=false&PAGE=Proteins&QUERY_INDEX=0&QUERY_NUMBER=0&RESULTS_PAGE_TARGET=&RID=TAJX8JRE01N&SHOW_LINKOUT=yes&SHOW_OVERVIEW=yes&STEP_NUMBER=&WORD_SIZE=2&DISPLAY_SORT=2&HSP_SORT=1" \l "sort_mark)** | **[Query coverage](http://blast.ncbi.nlm.nih.gov/Blast.cgi?CMD=Get&ALIGNMENTS=100&ALIGNMENT_VIEW=Pairwise&CDD_SEARCH_STATE=1&DATABASE_SORT=0&DESCRIPTIONS=100&ENTREZ_QUERY=txid9606 %5BORGN%5D&FIRST_QUERY_NUM=0&FORMAT_OBJECT=Alignment&FORMAT_PAGE_TARGET=&FORMAT_TYPE=HTML&GET_SEQUENCE=yes&I_THRESH=&MASK_CHAR=2&MASK_COLOR=1&NEW_VIEW=yes&NUM_OVERVIEW=100&OLD_BLAST=false&PAGE=Proteins&QUERY_INDEX=0&QUERY_NUMBER=0&RESULTS_PAGE_TARGET=&RID=TAJX8JRE01N&SHOW_LINKOUT=yes&SHOW_OVERVIEW=yes&STEP_NUMBER=&WORD_SIZE=2&DISPLAY_SORT=4&HSP_SORT=0" \l "sort_mark)** | **[E value](http://blast.ncbi.nlm.nih.gov/Blast.cgi?CMD=Get&ALIGNMENTS=100&ALIGNMENT_VIEW=Pairwise&CDD_SEARCH_STATE=1&DATABASE_SORT=0&DESCRIPTIONS=100&ENTREZ_QUERY=txid9606 %5BORGN%5D&FIRST_QUERY_NUM=0&FORMAT_OBJECT=Alignment&FORMAT_PAGE_TARGET=&FORMAT_TYPE=HTML&GET_SEQUENCE=yes&I_THRESH=&MASK_CHAR=2&MASK_COLOR=1&NEW_VIEW=yes&NUM_OVERVIEW=100&OLD_BLAST=false&PAGE=Proteins&QUERY_INDEX=0&QUERY_NUMBER=0&RESULTS_PAGE_TARGET=&RID=TAJX8JRE01N&SHOW_LINKOUT=yes&SHOW_OVERVIEW=yes&STEP_NUMBER=&WORD_SIZE=2&DISPLAY_SORT=0&HSP_SORT=0" \l "sort_mark)** |
| --- | --- | --- | --- | --- | --- |
| [NP_001364.1](http://www.ncbi.nlm.nih.gov/entrez/query.fcgi?cmd=Retrieve&db=Protein&list_uids=223555935&dopt=GenPept&RID=TAJX8JRE01N&log$=prottop&blast_rank=1) | dynein heavy chain 14, axonemal isoform 1 [Homo sapiens] | [20.6](http://blast.ncbi.nlm.nih.gov/Blast.cgi#223555935%23223555935) | 30.5 | 100% | 67 |
| [NP_849153.3](http://www.ncbi.nlm.nih.gov/entrez/query.fcgi?cmd=Retrieve&db=Protein&list_uids=54020722&dopt=GenPept&RID=TAJX8JRE01N&log$=prottop&blast_rank=2) | putative protein GATS [Homo sapiens] | [20.6](http://blast.ncbi.nlm.nih.gov/Blast.cgi#54020722%2354020722) | 20.6 | 83% | 67 |
| [NP_848614.1](http://www.ncbi.nlm.nih.gov/entrez/query.fcgi?cmd=Retrieve&db=Protein&list_uids=30425462&dopt=GenPept&RID=TAJX8JRE01N&log$=prottop&blast_rank=3) | hypothetical protein LOC284185 [Homo sapiens] | [19.7](http://blast.ncbi.nlm.nih.gov/Blast.cgi#30425462%2330425462) | 19.7 | 100% | 120 |
| [NP_001136007.1](http://www.ncbi.nlm.nih.gov/entrez/query.fcgi?cmd=Retrieve&db=Protein&list_uids=216547809&dopt=GenPept&RID=TAJX8JRE01N&log$=prottop&blast_rank=4) | lectin, galactoside-binding, soluble, 12 isoform 1 [Homo sapiens] | [19.3](http://blast.ncbi.nlm.nih.gov/Blast.cgi#216547809%23216547809) | 19.3 | 100% | 161 |
| [NP_001158067.1](http://www.ncbi.nlm.nih.gov/entrez/query.fcgi?cmd=Retrieve&db=Protein&list_uids=257196172&dopt=GenPept&RID=TAJX8JRE01N&log$=prottop&blast_rank=5) | PDZ domain-containing RING finger protein 4 isoform 1 [Homo sapiens] | [19.3](http://blast.ncbi.nlm.nih.gov/Blast.cgi#257196172%23257196172) | 19.3 | 100% | 161 |
| [NP_001136010.1](http://www.ncbi.nlm.nih.gov/entrez/query.fcgi?cmd=Retrieve&db=Protein&list_uids=216547832&dopt=GenPept&RID=TAJX8JRE01N&log$=prottop&blast_rank=6) | lectin, galactoside-binding, soluble, 12 isoform 5 [Homo sapiens] | [19.3](http://blast.ncbi.nlm.nih.gov/Blast.cgi#216547832%23216547832) | 19.3 | 100% | 161 |
| [NP_001136008.1](http://www.ncbi.nlm.nih.gov/entrez/query.fcgi?cmd=Retrieve&db=Protein&list_uids=216547811&dopt=GenPept&RID=TAJX8JRE01N&log$=prottop&blast_rank=7) | lectin, galactoside-binding, soluble, 12 isoform 3 [Homo sapiens] | [19.3](http://blast.ncbi.nlm.nih.gov/Blast.cgi#216547811%23216547811) | 19.3 | 100% | 161 |
| [NP_001136009.1](http://www.ncbi.nlm.nih.gov/entrez/query.fcgi?cmd=Retrieve&db=Protein&list_uids=216547818&dopt=GenPept&RID=TAJX8JRE01N&log$=prottop&blast_rank=8) | lectin, galactoside-binding, soluble, 12 isoform 4 [Homo sapiens] | [19.3](http://blast.ncbi.nlm.nih.gov/Blast.cgi#216547818%23216547818) | 19.3 | 100% | 161 |
| [NP_149092.2](http://www.ncbi.nlm.nih.gov/entrez/query.fcgi?cmd=Retrieve&db=Protein&list_uids=20127659&dopt=GenPept&RID=TAJX8JRE01N&log$=prottop&blast_rank=9) | lectin, galactoside-binding, soluble, 12 isoform 2 [Homo sapiens] | [19.3](http://blast.ncbi.nlm.nih.gov/Blast.cgi#20127659%2320127659) | 19.3 | 100% | 161 |
| [NP_001164638.1](http://www.ncbi.nlm.nih.gov/entrez/query.fcgi?cmd=Retrieve&db=Protein&list_uids=284005537&dopt=GenPept&RID=TAJX8JRE01N&log$=prottop&blast_rank=10) | calmodulin-binding transcription activator 2 isoform 4 [Homo sapiens] | [18.9](http://blast.ncbi.nlm.nih.gov/Blast.cgi#284005537%23284005537) | 18.9 | 66% | 216 |
| [NP_001164637.1](http://www.ncbi.nlm.nih.gov/entrez/query.fcgi?cmd=Retrieve&db=Protein&list_uids=284005535&dopt=GenPept&RID=TAJX8JRE01N&log$=prottop&blast_rank=11) | calmodulin-binding transcription activator 2 isoform 2 [Homo sapiens] | [18.9](http://blast.ncbi.nlm.nih.gov/Blast.cgi#284005535%23284005535) | 18.9 | 66% | 216 |
| [NP_001161097.1](http://www.ncbi.nlm.nih.gov/entrez/query.fcgi?cmd=Retrieve&db=Protein&list_uids=264681420&dopt=GenPept&RID=TAJX8JRE01N&log$=prottop&blast_rank=12) | voltage-dependent L-type calcium channel subunit alpha-1C isoform 23 [Homo sapiens] | [18.9](http://blast.ncbi.nlm.nih.gov/Blast.cgi#264681420%23264681420) | 18.9 | 66% | 216 |
| [NP_001161096.1](http://www.ncbi.nlm.nih.gov/entrez/query.fcgi?cmd=Retrieve&db=Protein&list_uids=264681418&dopt=GenPept&RID=TAJX8JRE01N&log$=prottop&blast_rank=13) | voltage-dependent L-type calcium channel subunit alpha-1C isoform 22 [Homo sapiens] | [18.9](http://blast.ncbi.nlm.nih.gov/Blast.cgi#264681418%23264681418) | 18.9 | 66% | 216 |
| [NP_001161095.1](http://www.ncbi.nlm.nih.gov/entrez/query.fcgi?cmd=Retrieve&db=Protein&list_uids=264681416&dopt=GenPept&RID=TAJX8JRE01N&log$=prottop&blast_rank=14) | voltage-dependent L-type calcium channel subunit alpha-1C isoform 21 [Homo sapiens] | [18.9](http://blast.ncbi.nlm.nih.gov/Blast.cgi#264681416%23264681416) | 18.9 | 66% | 216 |
| [NP_001153199.1](http://www.ncbi.nlm.nih.gov/entrez/query.fcgi?cmd=Retrieve&db=Protein&list_uids=229091322&dopt=GenPept&RID=TAJX8JRE01N&log$=prottop&blast_rank=15) | putative phospholipase B-like 2 isoform 2 precursor [Homo sapiens] | [18.9](http://blast.ncbi.nlm.nih.gov/Blast.cgi#229091322%23229091322) | 18.9 | 100% | 216 |
| [NP_775813.2](http://www.ncbi.nlm.nih.gov/entrez/query.fcgi?cmd=Retrieve&db=Protein&list_uids=229093316&dopt=GenPept&RID=TAJX8JRE01N&log$=prottop&blast_rank=16) | putative phospholipase B-like 2 isoform 1 precursor [Homo sapiens] | [18.9](http://blast.ncbi.nlm.nih.gov/Blast.cgi#229093316%23229093316) | 18.9 | 100% | 216 |
| [NP_001123311.1](http://www.ncbi.nlm.nih.gov/entrez/query.fcgi?cmd=Retrieve&db=Protein&list_uids=193794832&dopt=GenPept&RID=TAJX8JRE01N&log$=prottop&blast_rank=17) | voltage-dependent L-type calcium channel subunit alpha-1C isoforom 13 [Homo sapiens] | [18.9](http://blast.ncbi.nlm.nih.gov/Blast.cgi#193794832%23193794832) | 18.9 | 66% | 216 |
| [NP_001123308.1](http://www.ncbi.nlm.nih.gov/entrez/query.fcgi?cmd=Retrieve&db=Protein&list_uids=193794830&dopt=GenPept&RID=TAJX8JRE01N&log$=prottop&blast_rank=18) | voltage-dependent L-type calcium channel subunit alpha-1C isoform 10 [Homo sapiens] | [18.9](http://blast.ncbi.nlm.nih.gov/Blast.cgi#193794830%23193794830) | 18.9 | 66% | 216 |
| [NP_001123305.1](http://www.ncbi.nlm.nih.gov/entrez/query.fcgi?cmd=Retrieve&db=Protein&list_uids=193794828&dopt=GenPept&RID=TAJX8JRE01N&log$=prottop&blast_rank=19) | voltage-dependent L-type calcium channel subunit alpha-1C isoform 7 [Homo sapiens] | [18.9](http://blast.ncbi.nlm.nih.gov/Blast.cgi#193794828%23193794828) | 18.9 | 66% | 216 |
| [NP_001123303.1](http://www.ncbi.nlm.nih.gov/entrez/query.fcgi?cmd=Retrieve&db=Protein&list_uids=193788732&dopt=GenPept&RID=TAJX8JRE01N&log$=prottop&blast_rank=20) | voltage-dependent L-type calcium channel subunit alpha-1C isoform 5 [Homo sapiens] | [18.9](http://blast.ncbi.nlm.nih.gov/Blast.cgi#193788732%23193788732) | 18.9 | 66% | 216 |
| [NP_001123302.1](http://www.ncbi.nlm.nih.gov/entrez/query.fcgi?cmd=Retrieve&db=Protein&list_uids=193788730&dopt=GenPept&RID=TAJX8JRE01N&log$=prottop&blast_rank=21) | voltage-dependent L-type calcium channel subunit alpha-1C isoform 4 [Homo sapiens] | [18.9](http://blast.ncbi.nlm.nih.gov/Blast.cgi#193788730%23193788730) | 18.9 | 66% | 216 |
| [NP_001123301.1](http://www.ncbi.nlm.nih.gov/entrez/query.fcgi?cmd=Retrieve&db=Protein&list_uids=193788728&dopt=GenPept&RID=TAJX8JRE01N&log$=prottop&blast_rank=22) | voltage-dependent L-type calcium channel subunit alpha-1C isoform 3 [Homo sapiens] | [18.9](http://blast.ncbi.nlm.nih.gov/Blast.cgi#193788728%23193788728) | 18.9 | 66% | 216 |
| [NP_001123299.1](http://www.ncbi.nlm.nih.gov/entrez/query.fcgi?cmd=Retrieve&db=Protein&list_uids=193788724&dopt=GenPept&RID=TAJX8JRE01N&log$=prottop&blast_rank=23) | voltage-dependent L-type calcium channel subunit alpha-1C isoform 2 [Homo sapiens] | [18.9](http://blast.ncbi.nlm.nih.gov/Blast.cgi#193788724%23193788724) | 18.9 | 66% | 216 |
| [NP_955630.2](http://www.ncbi.nlm.nih.gov/entrez/query.fcgi?cmd=Retrieve&db=Protein&list_uids=193788720&dopt=GenPept&RID=TAJX8JRE01N&log$=prottop&blast_rank=24) | voltage-dependent L-type calcium channel subunit alpha-1C isoform 1 [Homo sapiens] | [18.9](http://blast.ncbi.nlm.nih.gov/Blast.cgi#193788720%23193788720) | 18.9 | 66% | 216 |
| [NP_001123318.1](http://www.ncbi.nlm.nih.gov/entrez/query.fcgi?cmd=Retrieve&db=Protein&list_uids=193788548&dopt=GenPept&RID=TAJX8JRE01N&log$=prottop&blast_rank=25) | voltage-dependent L-type calcium channel subunit alpha-1C isoform 20 [Homo sapiens] | [18.9](http://blast.ncbi.nlm.nih.gov/Blast.cgi#193788548%23193788548) | 18.9 | 66% | 216 |
| [NP_001123316.1](http://www.ncbi.nlm.nih.gov/entrez/query.fcgi?cmd=Retrieve&db=Protein&list_uids=193788544&dopt=GenPept&RID=TAJX8JRE01N&log$=prottop&blast_rank=26) | voltage-dependent L-type calcium channel subunit alpha-1C isoform 19 [Homo sapiens] | [18.9](http://blast.ncbi.nlm.nih.gov/Blast.cgi#193788544%23193788544) | 18.9 | 66% | 216 |
| [NP_001123315.1](http://www.ncbi.nlm.nih.gov/entrez/query.fcgi?cmd=Retrieve&db=Protein&list_uids=193788542&dopt=GenPept&RID=TAJX8JRE01N&log$=prottop&blast_rank=27) | voltage-dependent L-type calcium channel subunit alpha-1C isoform 17 [Homo sapiens] | [18.9](http://blast.ncbi.nlm.nih.gov/Blast.cgi#193788542%23193788542) | 18.9 | 66% | 216 |
| [NP_001123314.1](http://www.ncbi.nlm.nih.gov/entrez/query.fcgi?cmd=Retrieve&db=Protein&list_uids=193788540&dopt=GenPept&RID=TAJX8JRE01N&log$=prottop&blast_rank=28) | voltage-dependent L-type calcium channel subunit alpha-1C isoform 16 [Homo sapiens] | [18.9](http://blast.ncbi.nlm.nih.gov/Blast.cgi#193788540%23193788540) | 18.9 | 66% | 216 |
| [NP_001123313.1](http://www.ncbi.nlm.nih.gov/entrez/query.fcgi?cmd=Retrieve&db=Protein&list_uids=193788538&dopt=GenPept&RID=TAJX8JRE01N&log$=prottop&blast_rank=29) | voltage-dependent L-type calcium channel subunit alpha-1C isoform 15 [Homo sapiens] | [18.9](http://blast.ncbi.nlm.nih.gov/Blast.cgi#193788538%23193788538) | 18.9 | 66% | 216 |
| [NP_001123312.1](http://www.ncbi.nlm.nih.gov/entrez/query.fcgi?cmd=Retrieve&db=Protein&list_uids=193788536&dopt=GenPept&RID=TAJX8JRE01N&log$=prottop&blast_rank=30) | voltage-dependent L-type calcium channel subunit alpha-1C isoform 14 [Homo sapiens] | [18.9](http://blast.ncbi.nlm.nih.gov/Blast.cgi#193788536%23193788536) | 18.9 | 66% | 216 |
| [NP_001123310.1](http://www.ncbi.nlm.nih.gov/entrez/query.fcgi?cmd=Retrieve&db=Protein&list_uids=193788534&dopt=GenPept&RID=TAJX8JRE01N&log$=prottop&blast_rank=31) | voltage-dependent L-type calcium channel subunit alpha-1C isoform 12 [Homo sapiens] | [18.9](http://blast.ncbi.nlm.nih.gov/Blast.cgi#193788534%23193788534) | 18.9 | 66% | 216 |
| [NP_001123309.1](http://www.ncbi.nlm.nih.gov/entrez/query.fcgi?cmd=Retrieve&db=Protein&list_uids=193788532&dopt=GenPept&RID=TAJX8JRE01N&log$=prottop&blast_rank=32) | voltage-dependent L-type calcium channel subunit alpha-1C isoform 11 [Homo sapiens] | [18.9](http://blast.ncbi.nlm.nih.gov/Blast.cgi#193788532%23193788532) | 18.9 | 66% | 216 |
| [NP_001123306.1](http://www.ncbi.nlm.nih.gov/entrez/query.fcgi?cmd=Retrieve&db=Protein&list_uids=193788528&dopt=GenPept&RID=TAJX8JRE01N&log$=prottop&blast_rank=33) | voltage-dependent L-type calcium channel subunit alpha-1C isoform 8 [Homo sapiens] | [18.9](http://blast.ncbi.nlm.nih.gov/Blast.cgi#193788528%23193788528) | 18.9 | 66% | 216 |
| [NP_001123304.1](http://www.ncbi.nlm.nih.gov/entrez/query.fcgi?cmd=Retrieve&db=Protein&list_uids=193788526&dopt=GenPept&RID=TAJX8JRE01N&log$=prottop&blast_rank=34) | voltage-dependent L-type calcium channel subunit alpha-1C isoform 6 [Homo sapiens] | [18.9](http://blast.ncbi.nlm.nih.gov/Blast.cgi#193788526%23193788526) | 18.9 | 66% | 216 |
| [NP_001123307.1](http://www.ncbi.nlm.nih.gov/entrez/query.fcgi?cmd=Retrieve&db=Protein&list_uids=193788530&dopt=GenPept&RID=TAJX8JRE01N&log$=prottop&blast_rank=35) | voltage-dependent L-type calcium channel subunit alpha-1C isoform 9 [Homo sapiens] | [18.9](http://blast.ncbi.nlm.nih.gov/Blast.cgi#193788530%23193788530) | 18.9 | 66% | 216 |
| [NP_001121621.1](http://www.ncbi.nlm.nih.gov/entrez/query.fcgi?cmd=Retrieve&db=Protein&list_uids=189491742&dopt=GenPept&RID=TAJX8JRE01N&log$=prottop&blast_rank=36) | ataxin-7 isoform b [Homo sapiens] | [18.9](http://blast.ncbi.nlm.nih.gov/Blast.cgi#189491742%23189491742) | 18.9 | 66% | 216 |
| [NP_001073991.2](http://www.ncbi.nlm.nih.gov/entrez/query.fcgi?cmd=Retrieve&db=Protein&list_uids=197209974&dopt=GenPept&RID=TAJX8JRE01N&log$=prottop&blast_rank=37) | coiled-coil and C2 domain-containing protein 2A isoform a [Homo sapiens] | [18.9](http://blast.ncbi.nlm.nih.gov/Blast.cgi#197209974%23197209974) | 18.9 | 66% | 216 |
| [XP_001716026.1](http://www.ncbi.nlm.nih.gov/entrez/query.fcgi?cmd=Retrieve&db=Protein&list_uids=169209171&dopt=GenPept&RID=TAJX8JRE01N&log$=prottop&blast_rank=38) | PREDICTED: hypothetical protein [Homo sapiens] | [18.9](http://blast.ncbi.nlm.nih.gov/Blast.cgi#169209171%23169209171) | 18.9 | 83% | 216 |
| [XP_001716002.1](http://www.ncbi.nlm.nih.gov/entrez/query.fcgi?cmd=Retrieve&db=Protein&list_uids=169168280&dopt=GenPept&RID=TAJX8JRE01N&log$=prottop&blast_rank=39) | PREDICTED: hypothetical protein [Homo sapiens] | [18.9](http://blast.ncbi.nlm.nih.gov/Blast.cgi#169168280%23169168280) | 18.9 | 66% | 216 |
| [XP_001716937.1](http://www.ncbi.nlm.nih.gov/entrez/query.fcgi?cmd=Retrieve&db=Protein&list_uids=169168105&dopt=GenPept&RID=TAJX8JRE01N&log$=prottop&blast_rank=40) | PREDICTED: hypothetical protein [Homo sapiens] | [18.9](http://blast.ncbi.nlm.nih.gov/Blast.cgi#169168105%23169168105) | 18.9 | 66% | 216 |
| [XP_001716139.1](http://www.ncbi.nlm.nih.gov/entrez/query.fcgi?cmd=Retrieve&db=Protein&list_uids=169167523&dopt=GenPept&RID=TAJX8JRE01N&log$=prottop&blast_rank=41) | PREDICTED: hypothetical protein [Homo sapiens] | [18.9](http://blast.ncbi.nlm.nih.gov/Blast.cgi#169167523%23169167523) | 18.9 | 66% | 216 |
| [NP_001122311.1](http://www.ncbi.nlm.nih.gov/entrez/query.fcgi?cmd=Retrieve&db=Protein&list_uids=192807298&dopt=GenPept&RID=TAJX8JRE01N&log$=prottop&blast_rank=42) | voltage-dependent L-type calcium channel subunit alpha-1D isoform c [Homo sapiens] | [18.9](http://blast.ncbi.nlm.nih.gov/Blast.cgi#192807298%23192807298) | 18.9 | 66% | 216 |
| [NP_892006.2](http://www.ncbi.nlm.nih.gov/entrez/query.fcgi?cmd=Retrieve&db=Protein&list_uids=154277116&dopt=GenPept&RID=TAJX8JRE01N&log$=prottop&blast_rank=43) | nesprin-1 isoform 1 [Homo sapiens] | [18.9](http://blast.ncbi.nlm.nih.gov/Blast.cgi#154277116%23154277116) | 32.7 | 83% | 216 |
| [NP_001120932.1](http://www.ncbi.nlm.nih.gov/entrez/query.fcgi?cmd=Retrieve&db=Protein&list_uids=188536114&dopt=GenPept&RID=TAJX8JRE01N&log$=prottop&blast_rank=44) | PAB-dependent poly(A)-specific ribonuclease subunit 2 isoform 1 [Homo sapiens] | [18.9](http://blast.ncbi.nlm.nih.gov/Blast.cgi#188536114%23188536114) | 18.9 | 66% | 216 |
| [NP_001159751.1](http://www.ncbi.nlm.nih.gov/entrez/query.fcgi?cmd=Retrieve&db=Protein&list_uids=261878449&dopt=GenPept&RID=TAJX8JRE01N&log$=prottop&blast_rank=45) | PAB-dependent poly(A)-specific ribonuclease subunit 2 isoform 2 [Homo sapiens] | [18.9](http://blast.ncbi.nlm.nih.gov/Blast.cgi#261878449%23261878449) | 18.9 | 66% | 216 |
| [NP_060035.2](http://www.ncbi.nlm.nih.gov/entrez/query.fcgi?cmd=Retrieve&db=Protein&list_uids=241896898&dopt=GenPept&RID=TAJX8JRE01N&log$=prottop&blast_rank=46) | family with sequence similarity 20, member A precursor [Homo sapiens] | [18.9](http://blast.ncbi.nlm.nih.gov/Blast.cgi#241896898%23241896898) | 18.9 | 66% | 216 |
| [NP_079223.3](http://www.ncbi.nlm.nih.gov/entrez/query.fcgi?cmd=Retrieve&db=Protein&list_uids=148612879&dopt=GenPept&RID=TAJX8JRE01N&log$=prottop&blast_rank=47) | polyhomeotic-like protein 3 [Homo sapiens] | [18.9](http://blast.ncbi.nlm.nih.gov/Blast.cgi#148612879%23148612879) | 18.9 | 66% | 216 |
| [NP_001122312.1](http://www.ncbi.nlm.nih.gov/entrez/query.fcgi?cmd=Retrieve&db=Protein&list_uids=192807300&dopt=GenPept&RID=TAJX8JRE01N&log$=prottop&blast_rank=48) | voltage-dependent L-type calcium channel subunit alpha-1D isoform b [Homo sapiens] | [18.9](http://blast.ncbi.nlm.nih.gov/Blast.cgi#192807300%23192807300) | 18.9 | 66% | 216 |
| [NP_653299.3](http://www.ncbi.nlm.nih.gov/entrez/query.fcgi?cmd=Retrieve&db=Protein&list_uids=121582655&dopt=GenPept&RID=TAJX8JRE01N&log$=prottop&blast_rank=49) | ankyrin repeat domain-containing protein 35 [Homo sapiens] | [18.9](http://blast.ncbi.nlm.nih.gov/Blast.cgi#121582655%23121582655) | 18.9 | 66% | 216 |
| [NP_598411.2](http://www.ncbi.nlm.nih.gov/entrez/query.fcgi?cmd=Retrieve&db=Protein&list_uids=154277120&dopt=GenPept&RID=TAJX8JRE01N&log$=prottop&blast_rank=50) | nesprin-1 isoform 3 [Homo sapiens] | [18.9](http://blast.ncbi.nlm.nih.gov/Blast.cgi#154277120%23154277120) | 18.9 | 83% | 216 |
| [NP_009055.2](http://www.ncbi.nlm.nih.gov/entrez/query.fcgi?cmd=Retrieve&db=Protein&list_uids=110611228&dopt=GenPept&RID=TAJX8JRE01N&log$=prottop&blast_rank=51) | utrophin [Homo sapiens] | [18.9](http://blast.ncbi.nlm.nih.gov/Blast.cgi#110611228%23110611228) | 34.4 | 66% | 216 |
| [NP_055914.2](http://www.ncbi.nlm.nih.gov/entrez/query.fcgi?cmd=Retrieve&db=Protein&list_uids=29826341&dopt=GenPept&RID=TAJX8JRE01N&log$=prottop&blast_rank=52) | calmodulin-binding transcription activator 2 isoform 1 [Homo sapiens] | [18.9](http://blast.ncbi.nlm.nih.gov/Blast.cgi#29826341%2329826341) | 18.9 | 66% | 216 |
| [NP_005644.2](http://www.ncbi.nlm.nih.gov/entrez/query.fcgi?cmd=Retrieve&db=Protein&list_uids=21361278&dopt=GenPept&RID=TAJX8JRE01N&log$=prottop&blast_rank=53) | alpha-globin transcription factor CP2 [Homo sapiens] | [18.9](http://blast.ncbi.nlm.nih.gov/Blast.cgi#21361278%2321361278) | 18.9 | 66% | 216 |
| [NP_000710.5](http://www.ncbi.nlm.nih.gov/entrez/query.fcgi?cmd=Retrieve&db=Protein&list_uids=120433602&dopt=GenPept&RID=TAJX8JRE01N&log$=prottop&blast_rank=54) | voltage-dependent L-type calcium channel subunit alpha-1C isoform 18 [Homo sapiens] | [18.9](http://blast.ncbi.nlm.nih.gov/Blast.cgi#120433602%23120433602) | 18.9 | 66% | 216 |
| [NP_000620.2](http://www.ncbi.nlm.nih.gov/entrez/query.fcgi?cmd=Retrieve&db=Protein&list_uids=46488932&dopt=GenPept&RID=TAJX8JRE01N&log$=prottop&blast_rank=55) | interferon-alpha receptor 1 precursor [Homo sapiens] | [18.9](http://blast.ncbi.nlm.nih.gov/Blast.cgi#46488932%2346488932) | 18.9 | 66% | 216 |
| [NP_660339.1](http://www.ncbi.nlm.nih.gov/entrez/query.fcgi?cmd=Retrieve&db=Protein&list_uids=21686977&dopt=GenPept&RID=TAJX8JRE01N&log$=prottop&blast_rank=56) | cell adhesion molecule 4 precursor [Homo sapiens] | [18.9](http://blast.ncbi.nlm.nih.gov/Blast.cgi#21686977%2321686977) | 18.9 | 66% | 216 |
| [NP_004817.2](http://www.ncbi.nlm.nih.gov/entrez/query.fcgi?cmd=Retrieve&db=Protein&list_uids=157426891&dopt=GenPept&RID=TAJX8JRE01N&log$=prottop&blast_rank=57) | endothelin-converting enzyme-like 1 [Homo sapiens] | [18.9](http://blast.ncbi.nlm.nih.gov/Blast.cgi#157426891%23157426891) | 18.9 | 66% | 216 |
| [NP_945353.1](http://www.ncbi.nlm.nih.gov/entrez/query.fcgi?cmd=Retrieve&db=Protein&list_uids=39777606&dopt=GenPept&RID=TAJX8JRE01N&log$=prottop&blast_rank=58) | rho guanine nucleotide exchange factor 1 isoform 1 [Homo sapiens] | [18.9](http://blast.ncbi.nlm.nih.gov/Blast.cgi#39777606%2339777606) | 18.9 | 66% | 216 |
| [NP_783302.1](http://www.ncbi.nlm.nih.gov/entrez/query.fcgi?cmd=Retrieve&db=Protein&list_uids=28373126&dopt=GenPept&RID=TAJX8JRE01N&log$=prottop&blast_rank=59) | contactin 4 isoform c precursor [Homo sapiens] | [18.9](http://blast.ncbi.nlm.nih.gov/Blast.cgi#28373126%2328373126) | 18.9 | 66% | 216 |
| [NP_783301.1](http://www.ncbi.nlm.nih.gov/entrez/query.fcgi?cmd=Retrieve&db=Protein&list_uids=28373124&dopt=GenPept&RID=TAJX8JRE01N&log$=prottop&blast_rank=60) | contactin 4 isoform b precursor [Homo sapiens] | [18.9](http://blast.ncbi.nlm.nih.gov/Blast.cgi#28373124%2328373124) | 18.9 | 66% | 216 |
| [NP_945328.1](http://www.ncbi.nlm.nih.gov/entrez/query.fcgi?cmd=Retrieve&db=Protein&list_uids=39777604&dopt=GenPept&RID=TAJX8JRE01N&log$=prottop&blast_rank=61) | rho guanine nucleotide exchange factor 1 isoform 3 [Homo sapiens] | [18.9](http://blast.ncbi.nlm.nih.gov/Blast.cgi#39777604%2339777604) | 18.9 | 66% | 216 |
| [NP_149062.1](http://www.ncbi.nlm.nih.gov/entrez/query.fcgi?cmd=Retrieve&db=Protein&list_uids=23097308&dopt=GenPept&RID=TAJX8JRE01N&log$=prottop&blast_rank=62) | nesprin-1 isoform 2 [Homo sapiens] | [18.9](http://blast.ncbi.nlm.nih.gov/Blast.cgi#23097308%2323097308) | 32.7 | 83% | 216 |
| [NP_004697.2](http://www.ncbi.nlm.nih.gov/entrez/query.fcgi?cmd=Retrieve&db=Protein&list_uids=15011972&dopt=GenPept&RID=TAJX8JRE01N&log$=prottop&blast_rank=63) | rho guanine nucleotide exchange factor 1 isoform 2 [Homo sapiens] | [18.9](http://blast.ncbi.nlm.nih.gov/Blast.cgi#15011972%2315011972) | 18.9 | 66% | 216 |
| [NP_116231.2](http://www.ncbi.nlm.nih.gov/entrez/query.fcgi?cmd=Retrieve&db=Protein&list_uids=66348165&dopt=GenPept&RID=TAJX8JRE01N&log$=prottop&blast_rank=64) | transmembrane protein 209 [Homo sapiens] | [18.9](http://blast.ncbi.nlm.nih.gov/Blast.cgi#66348165%2366348165) | 18.9 | 66% | 216 |
| [NP_056108.2](http://www.ncbi.nlm.nih.gov/entrez/query.fcgi?cmd=Retrieve&db=Protein&list_uids=154277118&dopt=GenPept&RID=TAJX8JRE01N&log$=prottop&blast_rank=65) | nesprin-1 isoform 4 [Homo sapiens] | [18.9](http://blast.ncbi.nlm.nih.gov/Blast.cgi#154277118%23154277118) | 18.9 | 83% | 216 |
| [NP_055686.3](http://www.ncbi.nlm.nih.gov/entrez/query.fcgi?cmd=Retrieve&db=Protein&list_uids=188536112&dopt=GenPept&RID=TAJX8JRE01N&log$=prottop&blast_rank=66) | PAB-dependent poly(A)-specific ribonuclease subunit 2 isoform 3 [Homo sapiens] | [18.9](http://blast.ncbi.nlm.nih.gov/Blast.cgi#188536112%23188536112) | 18.9 | 66% | 216 |
| [NP_001164639.1](http://www.ncbi.nlm.nih.gov/entrez/query.fcgi?cmd=Retrieve&db=Protein&list_uids=284005543&dopt=GenPept&RID=TAJX8JRE01N&log$=prottop&blast_rank=67) | calmodulin-binding transcription activator 2 isoform 3 [Homo sapiens] | [18.9](http://blast.ncbi.nlm.nih.gov/Blast.cgi#284005543%23284005543) | 18.9 | 66% | 216 |
| [NP_000711.1](http://www.ncbi.nlm.nih.gov/entrez/query.fcgi?cmd=Retrieve&db=Protein&list_uids=4502527&dopt=GenPept&RID=TAJX8JRE01N&log$=prottop&blast_rank=68) | voltage-dependent L-type calcium channel subunit alpha-1D isoform a [Homo sapiens] | [18.9](http://blast.ncbi.nlm.nih.gov/Blast.cgi#4502527%234502527) | 18.9 | 66% | 216 |
| [NP_001846.3](http://www.ncbi.nlm.nih.gov/entrez/query.fcgi?cmd=Retrieve&db=Protein&list_uids=116008152&dopt=GenPept&RID=TAJX8JRE01N&log$=prottop&blast_rank=69) | alpha 1 type XV collagen precursor [Homo sapiens] | [18.9](http://blast.ncbi.nlm.nih.gov/Blast.cgi#116008152%23116008152) | 18.9 | 66% | 216 |
| [NP_056273.2](http://www.ncbi.nlm.nih.gov/entrez/query.fcgi?cmd=Retrieve&db=Protein&list_uids=19923424&dopt=GenPept&RID=TAJX8JRE01N&log$=prottop&blast_rank=70) | myotubularin-related protein 9 [Homo sapiens] | [18.9](http://blast.ncbi.nlm.nih.gov/Blast.cgi#19923424%2319923424) | 18.9 | 66% | 216 |
| [XP_002343055.1](http://www.ncbi.nlm.nih.gov/entrez/query.fcgi?cmd=Retrieve&db=Protein&list_uids=239744205&dopt=GenPept&RID=TAJX8JRE01N&log$=prottop&blast_rank=71) | PREDICTED: hypothetical protein XP_002343055 [Homo sapiens] >ref|XP_002347200.1| PREDICTED: hypothetical protein XP_002347200 [Homo sapiens] >ref|XP_002344555.1| PREDICTED: hypothetical protein [Homo sapiens] | [18.9](http://blast.ncbi.nlm.nih.gov/Blast.cgi#239744205%23239744205) | 18.9 | 66% | 216 |
| [NP_066022.2](http://www.ncbi.nlm.nih.gov/entrez/query.fcgi?cmd=Retrieve&db=Protein&list_uids=115430237&dopt=GenPept&RID=TAJX8JRE01N&log$=prottop&blast_rank=72) | spectrin beta chain, brain 3 isoform sigma1 [Homo sapiens] | [18.9](http://blast.ncbi.nlm.nih.gov/Blast.cgi#115430237%23115430237) | 18.9 | 66% | 216 |
| [NP_000324.1](http://www.ncbi.nlm.nih.gov/entrez/query.fcgi?cmd=Retrieve&db=Protein&list_uids=4506797&dopt=GenPept&RID=TAJX8JRE01N&log$=prottop&blast_rank=73) | ataxin-7 isoform a [Homo sapiens] | [18.9](http://blast.ncbi.nlm.nih.gov/Blast.cgi#4506797%234506797) | 18.9 | 66% | 216 |
| [NP_000960.2](http://www.ncbi.nlm.nih.gov/entrez/query.fcgi?cmd=Retrieve&db=Protein&list_uids=14591909&dopt=GenPept&RID=TAJX8JRE01N&log$=prottop&blast_rank=74) | ribosomal protein L5 [Homo sapiens] | [18.9](http://blast.ncbi.nlm.nih.gov/Blast.cgi#14591909%2314591909) | 18.9 | 66% | 216 |
| [NP_783200.1](http://www.ncbi.nlm.nih.gov/entrez/query.fcgi?cmd=Retrieve&db=Protein&list_uids=28373122&dopt=GenPept&RID=TAJX8JRE01N&log$=prottop&blast_rank=75) | contactin 4 isoform a precursor [Homo sapiens] | [18.9](http://blast.ncbi.nlm.nih.gov/Blast.cgi#28373122%2328373122) | 18.9 | 66% | 216 |
| [NP_060605.1](http://www.ncbi.nlm.nih.gov/entrez/query.fcgi?cmd=Retrieve&db=Protein&list_uids=8922511&dopt=GenPept&RID=TAJX8JRE01N&log$=prottop&blast_rank=76) | mitochondrial ribosomal protein S18A precursor [Homo sapiens] | [18.9](http://blast.ncbi.nlm.nih.gov/Blast.cgi#8922511%238922511) | 18.9 | 66% | 216 |
| [NP_000127.2](http://www.ncbi.nlm.nih.gov/entrez/query.fcgi?cmd=Retrieve&db=Protein&list_uids=56118236&dopt=GenPept&RID=TAJX8JRE01N&log$=prottop&blast_rank=77) | fanconi anemia group C protein [Homo sapiens] | [18.9](http://blast.ncbi.nlm.nih.gov/Blast.cgi#56118236%2356118236) | 18.9 | 66% | 216 |
| [NP_061833.1](http://www.ncbi.nlm.nih.gov/entrez/query.fcgi?cmd=Retrieve&db=Protein&list_uids=9506741&dopt=GenPept&RID=TAJX8JRE01N&log$=prottop&blast_rank=78) | glycine N-methyltransferase [Homo sapiens] | [18.9](http://blast.ncbi.nlm.nih.gov/Blast.cgi#9506741%239506741) | 18.9 | 66% | 216 |
| [NP_150094.5](http://www.ncbi.nlm.nih.gov/entrez/query.fcgi?cmd=Retrieve&db=Protein&list_uids=259013213&dopt=GenPept&RID=TAJX8JRE01N&log$=prottop&blast_rank=79) | CUB and sushi domain-containing protein 1 precursor [Homo sapiens] | [18.0](http://blast.ncbi.nlm.nih.gov/Blast.cgi#259013213%23259013213) | 28.4 | 100% | 388 |
| [XP_002344738.1](http://www.ncbi.nlm.nih.gov/entrez/query.fcgi?cmd=Retrieve&db=Protein&list_uids=239755904&dopt=GenPept&RID=TAJX8JRE01N&log$=prottop&blast_rank=80) | PREDICTED: hypothetical protein XP_002344738 [Homo sapiens] | [18.0](http://blast.ncbi.nlm.nih.gov/Blast.cgi#239755904%23239755904) | 18.0 | 83% | 388 |
| [XP_002343770.1](http://www.ncbi.nlm.nih.gov/entrez/query.fcgi?cmd=Retrieve&db=Protein&list_uids=239746469&dopt=GenPept&RID=TAJX8JRE01N&log$=prottop&blast_rank=81) | PREDICTED: hypothetical protein XP_002343770 [Homo sapiens] >ref|XP_002348093.1| PREDICTED: hypothetical protein XP_002348093 [Homo sapiens] >ref|XP_002345450.1| PREDICTED: hypothetical protein [Homo sapiens] | [18.0](http://blast.ncbi.nlm.nih.gov/Blast.cgi#239746469%23239746469) | 18.0 | 83% | 388 |
| [XP_002348164.1](http://www.ncbi.nlm.nih.gov/entrez/query.fcgi?cmd=Retrieve&db=Protein&list_uids=239752226&dopt=GenPept&RID=TAJX8JRE01N&log$=prottop&blast_rank=82) | PREDICTED: hypothetical protein XP_002348164 [Homo sapiens] | [18.0](http://blast.ncbi.nlm.nih.gov/Blast.cgi#239752226%23239752226) | 18.0 | 66% | 388 |
| [NP_001138910.1](http://www.ncbi.nlm.nih.gov/entrez/query.fcgi?cmd=Retrieve&db=Protein&list_uids=224177563&dopt=GenPept&RID=TAJX8JRE01N&log$=prottop&blast_rank=83) | post-GPI attachment to proteins factor 2 isoform 2 [Homo sapiens] | [18.0](http://blast.ncbi.nlm.nih.gov/Blast.cgi#224177563%23224177563) | 18.0 | 66% | 388 |
| [NP_001139540.1](http://www.ncbi.nlm.nih.gov/entrez/query.fcgi?cmd=Retrieve&db=Protein&list_uids=225703100&dopt=GenPept&RID=TAJX8JRE01N&log$=prottop&blast_rank=84) | calpain-2 catalytic subunit isoform 2 [Homo sapiens] | [18.0](http://blast.ncbi.nlm.nih.gov/Blast.cgi#225703100%23225703100) | 18.0 | 66% | 388 |
| [NP_002281.2](http://www.ncbi.nlm.nih.gov/entrez/query.fcgi?cmd=Retrieve&db=Protein&list_uids=157419122&dopt=GenPept&RID=TAJX8JRE01N&log$=prottop&blast_rank=85) | laminin, alpha 4 isoform 2 precursor [Homo sapiens] >ref|NP_001098677.1| laminin, alpha 4 isoform 2 precursor [Homo sapiens] | [18.0](http://blast.ncbi.nlm.nih.gov/Blast.cgi#157419122%23157419122) | 18.0 | 83% | 388 |
| [NP_001098676.1](http://www.ncbi.nlm.nih.gov/entrez/query.fcgi?cmd=Retrieve&db=Protein&list_uids=157419124&dopt=GenPept&RID=TAJX8JRE01N&log$=prottop&blast_rank=86) | laminin, alpha 4 isoform 1 precursor [Homo sapiens] | [18.0](http://blast.ncbi.nlm.nih.gov/Blast.cgi#157419124%23157419124) | 18.0 | 83% | 388 |
| [NP_056126.1](http://www.ncbi.nlm.nih.gov/entrez/query.fcgi?cmd=Retrieve&db=Protein&list_uids=144226847&dopt=GenPept&RID=TAJX8JRE01N&log$=prottop&blast_rank=87) | obscurin-like protein 1 [Homo sapiens] | [18.0](http://blast.ncbi.nlm.nih.gov/Blast.cgi#144226847%23144226847) | 32.7 | 83% | 388 |
| [NP_001092877.1](http://www.ncbi.nlm.nih.gov/entrez/query.fcgi?cmd=Retrieve&db=Protein&list_uids=150378537&dopt=GenPept&RID=TAJX8JRE01N&log$=prottop&blast_rank=88) | ankyrin repeat-containing protein C20orf12 [Homo sapiens] | [18.0](http://blast.ncbi.nlm.nih.gov/Blast.cgi#150378537%23150378537) | 18.0 | 66% | 388 |
| [NP_001073286.1](http://www.ncbi.nlm.nih.gov/entrez/query.fcgi?cmd=Retrieve&db=Protein&list_uids=119395740&dopt=GenPept&RID=TAJX8JRE01N&log$=prottop&blast_rank=89) | integrin alpha chain, alpha 6 isoform a precursor [Homo sapiens] | [18.0](http://blast.ncbi.nlm.nih.gov/Blast.cgi#119395740%23119395740) | 27.6 | 83% | 388 |
| [NP_056534.2](http://www.ncbi.nlm.nih.gov/entrez/query.fcgi?cmd=Retrieve&db=Protein&list_uids=110735435&dopt=GenPept&RID=TAJX8JRE01N&log$=prottop&blast_rank=90) | collagen, type V, alpha 3 preproprotein [Homo sapiens] | [18.0](http://blast.ncbi.nlm.nih.gov/Blast.cgi#110735435%23110735435) | 18.0 | 83% | 388 |
| [NP_006030.2](http://www.ncbi.nlm.nih.gov/entrez/query.fcgi?cmd=Retrieve&db=Protein&list_uids=110624774&dopt=GenPept&RID=TAJX8JRE01N&log$=prottop&blast_rank=91) | C-type mannose receptor 2 [Homo sapiens] | [18.0](http://blast.ncbi.nlm.nih.gov/Blast.cgi#110624774%23110624774) | 61.1 | 100% | 388 |
| [NP_004377.2](http://www.ncbi.nlm.nih.gov/entrez/query.fcgi?cmd=Retrieve&db=Protein&list_uids=118600983&dopt=GenPept&RID=TAJX8JRE01N&log$=prottop&blast_rank=92) | neurocan core protein precursor [Homo sapiens] | [18.0](http://blast.ncbi.nlm.nih.gov/Blast.cgi#118600983%23118600983) | 18.0 | 66% | 388 |
| [NP_694984.3](http://www.ncbi.nlm.nih.gov/entrez/query.fcgi?cmd=Retrieve&db=Protein&list_uids=153791630&dopt=GenPept&RID=TAJX8JRE01N&log$=prottop&blast_rank=93) | bromodomain and WD repeat-containing protein 3 [Homo sapiens] | [18.0](http://blast.ncbi.nlm.nih.gov/Blast.cgi#153791630%23153791630) | 18.0 | 83% | 388 |
| [NP_055178.3](http://www.ncbi.nlm.nih.gov/entrez/query.fcgi?cmd=Retrieve&db=Protein&list_uids=163659918&dopt=GenPept&RID=TAJX8JRE01N&log$=prottop&blast_rank=94) | sacsin [Homo sapiens] | [18.0](http://blast.ncbi.nlm.nih.gov/Blast.cgi#163659918%23163659918) | 18.0 | 66% | 388 |
| [NP_005520.4](http://www.ncbi.nlm.nih.gov/entrez/query.fcgi?cmd=Retrieve&db=Protein&list_uids=126012571&dopt=GenPept&RID=TAJX8JRE01N&log$=prottop&blast_rank=95) | basement membrane-specific heparan sulfate proteoglycan core protein precursor [Homo sapiens] | [18.0](http://blast.ncbi.nlm.nih.gov/Blast.cgi#126012571%23126012571) | 18.0 | 83% | 388 |
| [NP_937838.1](http://www.ncbi.nlm.nih.gov/entrez/query.fcgi?cmd=Retrieve&db=Protein&list_uids=38045944&dopt=GenPept&RID=TAJX8JRE01N&log$=prottop&blast_rank=96) | NEDD8-activating enzyme E1 catalytic subunit isoform 2 [Homo sapiens] | [18.0](http://blast.ncbi.nlm.nih.gov/Blast.cgi#38045944%2338045944) | 18.0 | 66% | 388 |
| [NP_001138489.1](http://www.ncbi.nlm.nih.gov/entrez/query.fcgi?cmd=Retrieve&db=Protein&list_uids=222446642&dopt=GenPept&RID=TAJX8JRE01N&log$=prottop&blast_rank=97) | proton-coupled amino acid transporter 3 isoform 1 [Homo sapiens] | [18.0](http://blast.ncbi.nlm.nih.gov/Blast.cgi#222446642%23222446642) | 18.0 | 66% | 388 |
| [NP_877951.1](http://www.ncbi.nlm.nih.gov/entrez/query.fcgi?cmd=Retrieve&db=Protein&list_uids=33469935&dopt=GenPept&RID=TAJX8JRE01N&log$=prottop&blast_rank=98) | EGF-like, fibronectin type III and laminin G domains isoform 3 [Homo sapiens] | [18.0](http://blast.ncbi.nlm.nih.gov/Blast.cgi#33469935%2333469935) | 18.0 | 83% | 388 |
| [NP_003959.3](http://www.ncbi.nlm.nih.gov/entrez/query.fcgi?cmd=Retrieve&db=Protein&list_uids=38045942&dopt=GenPept&RID=TAJX8JRE01N&log$=prottop&blast_rank=99) | NEDD8-activating enzyme E1 catalytic subunit isoform 1 [Homo sapiens] | [18.0](http://blast.ncbi.nlm.nih.gov/Blast.cgi#38045942%2338045942) | 18.0 | 66% | 388 |
| [NP_550436.1](http://www.ncbi.nlm.nih.gov/entrez/query.fcgi?cmd=Retrieve&db=Protein&list_uids=18426877&dopt=GenPept&RID=TAJX8JRE01N&log$=prottop&blast_rank=100) | asialoglycoprotein receptor 2 isoform c [Homo sapiens] | [18.0](http://blast.ncbi.nlm.nih.gov/Blast.cgi#18426877%2318426877) | 18.0 | 66% | 388 |

| **Accession** | **Proteins with a match to PETTDK peptide** | **[Max score](http://blast.ncbi.nlm.nih.gov/Blast.cgi?CMD=Get&ALIGNMENTS=100&ALIGNMENT_VIEW=Pairwise&CDD_SEARCH_STATE=1&DATABASE_SORT=0&DESCRIPTIONS=100&ENTREZ_QUERY=txid9606 %5BORGN%5D&FIRST_QUERY_NUM=0&FORMAT_OBJECT=Alignment&FORMAT_PAGE_TARGET=&FORMAT_TYPE=HTML&GET_SEQUENCE=yes&I_THRESH=&MASK_CHAR=2&MASK_COLOR=1&NEW_VIEW=yes&NUM_OVERVIEW=100&OLD_BLAST=false&PAGE=Proteins&QUERY_INDEX=0&QUERY_NUMBER=0&RESULTS_PAGE_TARGET=&RID=TAK4YPNW01S&SHOW_LINKOUT=yes&SHOW_OVERVIEW=yes&STEP_NUMBER=&WORD_SIZE=2&DISPLAY_SORT=1&HSP_SORT=1" \l "sort_mark)** | **[Total score](http://blast.ncbi.nlm.nih.gov/Blast.cgi?CMD=Get&ALIGNMENTS=100&ALIGNMENT_VIEW=Pairwise&CDD_SEARCH_STATE=1&DATABASE_SORT=0&DESCRIPTIONS=100&ENTREZ_QUERY=txid9606 %5BORGN%5D&FIRST_QUERY_NUM=0&FORMAT_OBJECT=Alignment&FORMAT_PAGE_TARGET=&FORMAT_TYPE=HTML&GET_SEQUENCE=yes&I_THRESH=&MASK_CHAR=2&MASK_COLOR=1&NEW_VIEW=yes&NUM_OVERVIEW=100&OLD_BLAST=false&PAGE=Proteins&QUERY_INDEX=0&QUERY_NUMBER=0&RESULTS_PAGE_TARGET=&RID=TAK4YPNW01S&SHOW_LINKOUT=yes&SHOW_OVERVIEW=yes&STEP_NUMBER=&WORD_SIZE=2&DISPLAY_SORT=2&HSP_SORT=1" \l "sort_mark)** | **[Query coverage](http://blast.ncbi.nlm.nih.gov/Blast.cgi?CMD=Get&ALIGNMENTS=100&ALIGNMENT_VIEW=Pairwise&CDD_SEARCH_STATE=1&DATABASE_SORT=0&DESCRIPTIONS=100&ENTREZ_QUERY=txid9606 %5BORGN%5D&FIRST_QUERY_NUM=0&FORMAT_OBJECT=Alignment&FORMAT_PAGE_TARGET=&FORMAT_TYPE=HTML&GET_SEQUENCE=yes&I_THRESH=&MASK_CHAR=2&MASK_COLOR=1&NEW_VIEW=yes&NUM_OVERVIEW=100&OLD_BLAST=false&PAGE=Proteins&QUERY_INDEX=0&QUERY_NUMBER=0&RESULTS_PAGE_TARGET=&RID=TAK4YPNW01S&SHOW_LINKOUT=yes&SHOW_OVERVIEW=yes&STEP_NUMBER=&WORD_SIZE=2&DISPLAY_SORT=4&HSP_SORT=0" \l "sort_mark)** | **[E value](http://blast.ncbi.nlm.nih.gov/Blast.cgi?CMD=Get&ALIGNMENTS=100&ALIGNMENT_VIEW=Pairwise&CDD_SEARCH_STATE=1&DATABASE_SORT=0&DESCRIPTIONS=100&ENTREZ_QUERY=txid9606 %5BORGN%5D&FIRST_QUERY_NUM=0&FORMAT_OBJECT=Alignment&FORMAT_PAGE_TARGET=&FORMAT_TYPE=HTML&GET_SEQUENCE=yes&I_THRESH=&MASK_CHAR=2&MASK_COLOR=1&NEW_VIEW=yes&NUM_OVERVIEW=100&OLD_BLAST=false&PAGE=Proteins&QUERY_INDEX=0&QUERY_NUMBER=0&RESULTS_PAGE_TARGET=&RID=TAK4YPNW01S&SHOW_LINKOUT=yes&SHOW_OVERVIEW=yes&STEP_NUMBER=&WORD_SIZE=2&DISPLAY_SORT=0&HSP_SORT=0" \l "sort_mark)** |
| --- | --- | --- | --- | --- | --- |
| [XP_002344507.1](http://www.ncbi.nlm.nih.gov/entrez/query.fcgi?cmd=Retrieve&db=Protein&list_uids=239755232&dopt=GenPept&RID=TAK4YPNW01S&log$=prottop&blast_rank=1) | PREDICTED: hypothetical protein [Homo sapiens] | [19.3](http://blast.ncbi.nlm.nih.gov/Blast.cgi#239755232%23239755232) | 19.3 | 83% | 161 |
| [XP_002343007.1](http://www.ncbi.nlm.nih.gov/entrez/query.fcgi?cmd=Retrieve&db=Protein&list_uids=239744027&dopt=GenPept&RID=TAK4YPNW01S&log$=prottop&blast_rank=2) | PREDICTED: hypothetical protein XP_002343007 [Homo sapiens] >ref|XP_002347159.1| PREDICTED: hypothetical protein [Homo sapiens] | [19.3](http://blast.ncbi.nlm.nih.gov/Blast.cgi#239744027%23239744027) | 19.3 | 83% | 161 |
| [XP_001717740.2](http://www.ncbi.nlm.nih.gov/entrez/query.fcgi?cmd=Retrieve&db=Protein&list_uids=239743976&dopt=GenPept&RID=TAK4YPNW01S&log$=prottop&blast_rank=3) | PREDICTED: hypothetical protein [Homo sapiens] >ref|XP_001724587.2| PREDICTED: hypothetical protein [Homo sapiens] | [19.3](http://blast.ncbi.nlm.nih.gov/Blast.cgi#239743976%23239743976) | 19.3 | 83% | 161 |
| [NP_006624.2](http://www.ncbi.nlm.nih.gov/entrez/query.fcgi?cmd=Retrieve&db=Protein&list_uids=116089337&dopt=GenPept&RID=TAK4YPNW01S&log$=prottop&blast_rank=4) | ras GTPase-activating-like protein IQGAP2 [Homo sapiens] | [19.3](http://blast.ncbi.nlm.nih.gov/Blast.cgi#116089337%23116089337) | 31.8 | 100% | 161 |
| [NP_055863.1](http://www.ncbi.nlm.nih.gov/entrez/query.fcgi?cmd=Retrieve&db=Protein&list_uids=210032580&dopt=GenPept&RID=TAK4YPNW01S&log$=prottop&blast_rank=5) | histone-lysine N-methyltransferase SETD1B [Homo sapiens] | [19.3](http://blast.ncbi.nlm.nih.gov/Blast.cgi#210032580%23210032580) | 19.3 | 83% | 161 |
| [NP_839943.2](http://www.ncbi.nlm.nih.gov/entrez/query.fcgi?cmd=Retrieve&db=Protein&list_uids=39753961&dopt=GenPept&RID=TAK4YPNW01S&log$=prottop&blast_rank=6) | ras GTPase-activating-like protein IQGAP3 [Homo sapiens] | [19.3](http://blast.ncbi.nlm.nih.gov/Blast.cgi#39753961%2339753961) | 19.3 | 83% | 161 |
| [NP_775954.2](http://www.ncbi.nlm.nih.gov/entrez/query.fcgi?cmd=Retrieve&db=Protein&list_uids=89886484&dopt=GenPept&RID=TAK4YPNW01S&log$=prottop&blast_rank=7) | XK, Kell blood group complex subunit-related family, member 6 isoform b [Homo sapiens] | [19.3](http://blast.ncbi.nlm.nih.gov/Blast.cgi#89886484%2389886484) | 19.3 | 83% | 161 |
| [NP_055319.1](http://www.ncbi.nlm.nih.gov/entrez/query.fcgi?cmd=Retrieve&db=Protein&list_uids=7657496&dopt=GenPept&RID=TAK4YPNW01S&log$=prottop&blast_rank=8) | RAB guanine nucleotide exchange factor (GEF) 1 [Homo sapiens] | [19.3](http://blast.ncbi.nlm.nih.gov/Blast.cgi#7657496%237657496) | 19.3 | 83% | 161 |
| [NP_003861.1](http://www.ncbi.nlm.nih.gov/entrez/query.fcgi?cmd=Retrieve&db=Protein&list_uids=4506787&dopt=GenPept&RID=TAK4YPNW01S&log$=prottop&blast_rank=9) | ras GTPase-activating-like protein IQGAP1 [Homo sapiens] | [19.3](http://blast.ncbi.nlm.nih.gov/Blast.cgi#4506787%234506787) | 30.5 | 100% | 161 |
| [NP_001124388.1](http://www.ncbi.nlm.nih.gov/entrez/query.fcgi?cmd=Retrieve&db=Protein&list_uids=195963412&dopt=GenPept&RID=TAK4YPNW01S&log$=prottop&blast_rank=10) | transforming growth factor, beta receptor I isoform 2 precursor [Homo sapiens] | [18.9](http://blast.ncbi.nlm.nih.gov/Blast.cgi#195963412%23195963412) | 18.9 | 83% | 216 |
| [NP_056173.1](http://www.ncbi.nlm.nih.gov/entrez/query.fcgi?cmd=Retrieve&db=Protein&list_uids=28872812&dopt=GenPept&RID=TAK4YPNW01S&log$=prottop&blast_rank=11) | MORC family CW-type zinc finger protein 3 [Homo sapiens] | [18.9](http://blast.ncbi.nlm.nih.gov/Blast.cgi#28872812%2328872812) | 18.9 | 83% | 216 |
| [NP_005236.2](http://www.ncbi.nlm.nih.gov/entrez/query.fcgi?cmd=Retrieve&db=Protein&list_uids=66346693&dopt=GenPept&RID=TAK4YPNW01S&log$=prottop&blast_rank=12) | protocadherin Fat 1 precursor [Homo sapiens] | [18.9](http://blast.ncbi.nlm.nih.gov/Blast.cgi#66346693%2366346693) | 18.9 | 83% | 216 |
| [NP_002825.3](http://www.ncbi.nlm.nih.gov/entrez/query.fcgi?cmd=Retrieve&db=Protein&list_uids=33356177&dopt=GenPept&RID=TAK4YPNW01S&log$=prottop&blast_rank=13) | tyrosine-protein phosphatase non-receptor type 11 [Homo sapiens] | [18.9](http://blast.ncbi.nlm.nih.gov/Blast.cgi#33356177%2333356177) | 18.9 | 83% | 216 |
| [NP_004603.1](http://www.ncbi.nlm.nih.gov/entrez/query.fcgi?cmd=Retrieve&db=Protein&list_uids=4759226&dopt=GenPept&RID=TAK4YPNW01S&log$=prottop&blast_rank=14) | transforming growth factor, beta receptor I isoform 1 precursor [Homo sapiens] | [18.9](http://blast.ncbi.nlm.nih.gov/Blast.cgi#4759226%234759226) | 18.9 | 83% | 216 |
| [NP_789783.1](http://www.ncbi.nlm.nih.gov/entrez/query.fcgi?cmd=Retrieve&db=Protein&list_uids=28827801&dopt=GenPept&RID=TAK4YPNW01S&log$=prottop&blast_rank=15) | anterior gradient protein 3 homolog precursor [Homo sapiens] | [18.9](http://blast.ncbi.nlm.nih.gov/Blast.cgi#28827801%2328827801) | 18.9 | 83% | 216 |
| [NP_006399.1](http://www.ncbi.nlm.nih.gov/entrez/query.fcgi?cmd=Retrieve&db=Protein&list_uids=5453541&dopt=GenPept&RID=TAK4YPNW01S&log$=prottop&blast_rank=16) | anterior gradient protein 2 homolog precursor [Homo sapiens] | [18.9](http://blast.ncbi.nlm.nih.gov/Blast.cgi#5453541%235453541) | 18.9 | 83% | 216 |
| [NP_079350.5](http://www.ncbi.nlm.nih.gov/entrez/query.fcgi?cmd=Retrieve&db=Protein&list_uids=256000767&dopt=GenPept&RID=TAK4YPNW01S&log$=prottop&blast_rank=17) | Fraser syndrome 1 protein isoform 1 precursor [Homo sapiens] | [18.0](http://blast.ncbi.nlm.nih.gov/Blast.cgi#256000767%23256000767) | 18.0 | 100% | 388 |
| [XP_002345619.1](http://www.ncbi.nlm.nih.gov/entrez/query.fcgi?cmd=Retrieve&db=Protein&list_uids=239753314&dopt=GenPept&RID=TAK4YPNW01S&log$=prottop&blast_rank=18) | PREDICTED: similar to RIKEN cDNA C230030N03 [Homo sapiens] | [18.0](http://blast.ncbi.nlm.nih.gov/Blast.cgi#239753314%23239753314) | 36.1 | 100% | 388 |
| [NP_054777.2](http://www.ncbi.nlm.nih.gov/entrez/query.fcgi?cmd=Retrieve&db=Protein&list_uids=40254871&dopt=GenPept&RID=TAK4YPNW01S&log$=prottop&blast_rank=19) | transmembrane protease serine 11E [Homo sapiens] | [18.0](http://blast.ncbi.nlm.nih.gov/Blast.cgi#40254871%2340254871) | 33.5 | 100% | 388 |
| [NP_000637.2](http://www.ncbi.nlm.nih.gov/entrez/query.fcgi?cmd=Retrieve&db=Protein&list_uids=116734853&dopt=GenPept&RID=TAK4YPNW01S&log$=prottop&blast_rank=20) | amylo-1,6-glucosidase, 4-alpha-glucanotransferase isoform 3 [Homo sapiens] | [17.6](http://blast.ncbi.nlm.nih.gov/Blast.cgi#116734853%23116734853) | 17.6 | 100% | 521 |
| [NP_000636.2](http://www.ncbi.nlm.nih.gov/entrez/query.fcgi?cmd=Retrieve&db=Protein&list_uids=116734849&dopt=GenPept&RID=TAK4YPNW01S&log$=prottop&blast_rank=21) | amylo-1,6-glucosidase, 4-alpha-glucanotransferase isoform 2 [Homo sapiens] | [17.6](http://blast.ncbi.nlm.nih.gov/Blast.cgi#116734849%23116734849) | 17.6 | 100% | 521 |
| [NP_004272.2](http://www.ncbi.nlm.nih.gov/entrez/query.fcgi?cmd=Retrieve&db=Protein&list_uids=14043024&dopt=GenPept&RID=TAK4YPNW01S&log$=prottop&blast_rank=22) | BAG family molecular chaperone regulator 3 [Homo sapiens] | [17.6](http://blast.ncbi.nlm.nih.gov/Blast.cgi#14043024%2314043024) | 17.6 | 100% | 521 |
| [NP_000019.2](http://www.ncbi.nlm.nih.gov/entrez/query.fcgi?cmd=Retrieve&db=Protein&list_uids=116734847&dopt=GenPept&RID=TAK4YPNW01S&log$=prottop&blast_rank=23) | amylo-1,6-glucosidase, 4-alpha-glucanotransferase isoform 1 [Homo sapiens] >ref|NP_000634.2| amylo-1,6-glucosidase, 4-alpha-glucanotransferase isoform 1 [Homo sapiens] >ref|NP_000635.2| amylo-1,6-glucosidase, 4-alpha-glucanotransferase isoform 1 [Homo sapiens] >ref|NP_000633.2| amylo-1,6-glucosidase, 4-alpha-glucanotransferase isoform 1 [Homo sapiens] | [17.6](http://blast.ncbi.nlm.nih.gov/Blast.cgi#116734847%23116734847) | 17.6 | 100% | 521 |
| [NP_001166130.1](http://www.ncbi.nlm.nih.gov/entrez/query.fcgi?cmd=Retrieve&db=Protein&list_uids=289547652&dopt=GenPept&RID=TAK4YPNW01S&log$=prottop&blast_rank=24) | lateral signaling target protein 2 homolog isoform 1 [Homo sapiens] | [17.2](http://blast.ncbi.nlm.nih.gov/Blast.cgi#289547652%23289547652) | 17.2 | 100% | 699 |
| [NP_001166127.1](http://www.ncbi.nlm.nih.gov/entrez/query.fcgi?cmd=Retrieve&db=Protein&list_uids=289547645&dopt=GenPept&RID=TAK4YPNW01S&log$=prottop&blast_rank=25) | lateral signaling target protein 2 homolog isoform 3 [Homo sapiens] | [17.2](http://blast.ncbi.nlm.nih.gov/Blast.cgi#289547645%23289547645) | 17.2 | 100% | 699 |
| [NP_001138525.1](http://www.ncbi.nlm.nih.gov/entrez/query.fcgi?cmd=Retrieve&db=Protein&list_uids=223029500&dopt=GenPept&RID=TAK4YPNW01S&log$=prottop&blast_rank=26) | EF-hand calcium-binding domain-containing protein 5 isoform b [Homo sapiens] | [17.2](http://blast.ncbi.nlm.nih.gov/Blast.cgi#223029500%23223029500) | 17.2 | 100% | 699 |
| [NP_066023.2](http://www.ncbi.nlm.nih.gov/entrez/query.fcgi?cmd=Retrieve&db=Protein&list_uids=289547643&dopt=GenPept&RID=TAK4YPNW01S&log$=prottop&blast_rank=27) | lateral signaling target protein 2 homolog isoform 2 [Homo sapiens] | [17.2](http://blast.ncbi.nlm.nih.gov/Blast.cgi#289547643%23289547643) | 17.2 | 100% | 699 |
| [NP_940931.2](http://www.ncbi.nlm.nih.gov/entrez/query.fcgi?cmd=Retrieve&db=Protein&list_uids=90652855&dopt=GenPept&RID=TAK4YPNW01S&log$=prottop&blast_rank=28) | EF-hand calcium-binding domain-containing protein 5 isoform a [Homo sapiens] | [17.2](http://blast.ncbi.nlm.nih.gov/Blast.cgi#90652855%2390652855) | 17.2 | 100% | 699 |
| [NP_001004458.1](http://www.ncbi.nlm.nih.gov/entrez/query.fcgi?cmd=Retrieve&db=Protein&list_uids=52317174&dopt=GenPept&RID=TAK4YPNW01S&log$=prottop&blast_rank=29) | olfactory receptor 1S1 [Homo sapiens] | [17.2](http://blast.ncbi.nlm.nih.gov/Blast.cgi#52317174%2352317174) | 17.2 | 100% | 699 |
| [NP_055532.1](http://www.ncbi.nlm.nih.gov/entrez/query.fcgi?cmd=Retrieve&db=Protein&list_uids=7662092&dopt=GenPept&RID=TAK4YPNW01S&log$=prottop&blast_rank=30) | zinc finger protein 536 [Homo sapiens] | [17.2](http://blast.ncbi.nlm.nih.gov/Blast.cgi#7662092%237662092) | 17.2 | 100% | 699 |
| [NP_001004459.1](http://www.ncbi.nlm.nih.gov/entrez/query.fcgi?cmd=Retrieve&db=Protein&list_uids=52317168&dopt=GenPept&RID=TAK4YPNW01S&log$=prottop&blast_rank=31) | olfactory receptor 1S2 [Homo sapiens] | [17.2](http://blast.ncbi.nlm.nih.gov/Blast.cgi#52317168%2352317168) | 17.2 | 100% | 699 |
| [NP_001026913.1](http://www.ncbi.nlm.nih.gov/entrez/query.fcgi?cmd=Retrieve&db=Protein&list_uids=72534780&dopt=GenPept&RID=TAK4YPNW01S&log$=prottop&blast_rank=32) | hypothetical protein LOC154313 [Homo sapiens] | [17.2](http://blast.ncbi.nlm.nih.gov/Blast.cgi#72534780%2372534780) | 17.2 | 100% | 699 |
| [NP_075389.1](http://www.ncbi.nlm.nih.gov/entrez/query.fcgi?cmd=Retrieve&db=Protein&list_uids=12738831&dopt=GenPept&RID=TAK4YPNW01S&log$=prottop&blast_rank=33) | PRAME family member 1 [Homo sapiens] | [17.2](http://blast.ncbi.nlm.nih.gov/Blast.cgi#12738831%2312738831) | 17.2 | 100% | 699 |
| [XP_001727055.2](http://www.ncbi.nlm.nih.gov/entrez/query.fcgi?cmd=Retrieve&db=Protein&list_uids=239747708&dopt=GenPept&RID=TAK4YPNW01S&log$=prottop&blast_rank=34) | PREDICTED: similar to unr-interacting protein [Homo sapiens] >ref|XP_001727087.2| PREDICTED: similar to unr-interacting protein [Homo sapiens] | [16.8](http://blast.ncbi.nlm.nih.gov/Blast.cgi#239747708%23239747708) | 16.8 | 83% | 938 |
| [NP_848599.3](http://www.ncbi.nlm.nih.gov/entrez/query.fcgi?cmd=Retrieve&db=Protein&list_uids=194440727&dopt=GenPept&RID=TAK4YPNW01S&log$=prottop&blast_rank=35) | axonemal dynein heavy chain 12-like protein isoform 1 [Homo sapiens] | [16.8](http://blast.ncbi.nlm.nih.gov/Blast.cgi#194440727%23194440727) | 16.8 | 83% | 938 |
| [XP_001725450.1](http://www.ncbi.nlm.nih.gov/entrez/query.fcgi?cmd=Retrieve&db=Protein&list_uids=169211489&dopt=GenPept&RID=TAK4YPNW01S&log$=prottop&blast_rank=36) | PREDICTED: hypothetical protein [Homo sapiens] | [16.8](http://blast.ncbi.nlm.nih.gov/Blast.cgi#169211489%23169211489) | 16.8 | 100% | 938 |
| [XP_001720324.1](http://www.ncbi.nlm.nih.gov/entrez/query.fcgi?cmd=Retrieve&db=Protein&list_uids=169210715&dopt=GenPept&RID=TAK4YPNW01S&log$=prottop&blast_rank=37) | PREDICTED: hypothetical protein [Homo sapiens] | [16.8](http://blast.ncbi.nlm.nih.gov/Blast.cgi#169210715%23169210715) | 16.8 | 100% | 938 |
| [NP_061720.2](http://www.ncbi.nlm.nih.gov/entrez/query.fcgi?cmd=Retrieve&db=Protein&list_uids=151301127&dopt=GenPept&RID=TAK4YPNW01S&log$=prottop&blast_rank=38) | dynein heavy chain 7, axonemal [Homo sapiens] | [16.8](http://blast.ncbi.nlm.nih.gov/Blast.cgi#151301127%23151301127) | 16.8 | 83% | 938 |
| [NP_060742.3](http://www.ncbi.nlm.nih.gov/entrez/query.fcgi?cmd=Retrieve&db=Protein&list_uids=131888105&dopt=GenPept&RID=TAK4YPNW01S&log$=prottop&blast_rank=39) | cancer susceptibility candidate protein 1 isoform a [Homo sapiens] | [16.8](http://blast.ncbi.nlm.nih.gov/Blast.cgi#131888105%23131888105) | 16.8 | 83% | 938 |
| [NP_001034589.2](http://www.ncbi.nlm.nih.gov/entrez/query.fcgi?cmd=Retrieve&db=Protein&list_uids=210147462&dopt=GenPept&RID=TAK4YPNW01S&log$=prottop&blast_rank=40) | von Willebrand factor A domain-containing protein 5B1 [Homo sapiens] | [16.8](http://blast.ncbi.nlm.nih.gov/Blast.cgi#210147462%23210147462) | 16.8 | 83% | 938 |
| [NP_001120683.1](http://www.ncbi.nlm.nih.gov/entrez/query.fcgi?cmd=Retrieve&db=Protein&list_uids=187761324&dopt=GenPept&RID=TAK4YPNW01S&log$=prottop&blast_rank=41) | shootin1 isoform a [Homo sapiens] | [16.8](http://blast.ncbi.nlm.nih.gov/Blast.cgi#187761324%23187761324) | 16.8 | 83% | 938 |
| [NP_000682.3](http://www.ncbi.nlm.nih.gov/entrez/query.fcgi?cmd=Retrieve&db=Protein&list_uids=22907049&dopt=GenPept&RID=TAK4YPNW01S&log$=prottop&blast_rank=42) | aldehyde dehydrogenase, dimeric NADP-preferring [Homo sapiens] >ref|NP_001128639.1| aldehyde dehydrogenase, dimeric NADP-preferring [Homo sapiens] >ref|NP_001128640.1| aldehyde dehydrogenase, dimeric NADP-preferring [Homo sapiens] | [16.8](http://blast.ncbi.nlm.nih.gov/Blast.cgi#22907049%2322907049) | 16.8 | 83% | 938 |
| [XP_293026.6](http://www.ncbi.nlm.nih.gov/entrez/query.fcgi?cmd=Retrieve&db=Protein&list_uids=113412837&dopt=GenPept&RID=TAK4YPNW01S&log$=prottop&blast_rank=43) | PREDICTED: similar to unr-interacting protein [Homo sapiens] | [16.8](http://blast.ncbi.nlm.nih.gov/Blast.cgi#113412837%23113412837) | 16.8 | 83% | 938 |
| [NP_009045.2](http://www.ncbi.nlm.nih.gov/entrez/query.fcgi?cmd=Retrieve&db=Protein&list_uids=110347443&dopt=GenPept&RID=TAK4YPNW01S&log$=prottop&blast_rank=44) | TATA element modulatory factor [Homo sapiens] | [16.8](http://blast.ncbi.nlm.nih.gov/Blast.cgi#110347443%23110347443) | 32.7 | 83% | 938 |
| [NP_003648.2](http://www.ncbi.nlm.nih.gov/entrez/query.fcgi?cmd=Retrieve&db=Protein&list_uids=191251777&dopt=GenPept&RID=TAK4YPNW01S&log$=prottop&blast_rank=45) | breast carcinoma-amplified sequence 1 [Homo sapiens] | [16.8](http://blast.ncbi.nlm.nih.gov/Blast.cgi#191251777%23191251777) | 16.8 | 83% | 938 |
| [NP_060800.2](http://www.ncbi.nlm.nih.gov/entrez/query.fcgi?cmd=Retrieve&db=Protein&list_uids=50511340&dopt=GenPept&RID=TAK4YPNW01S&log$=prottop&blast_rank=46) | shootin1 isoform b [Homo sapiens] | [16.8](http://blast.ncbi.nlm.nih.gov/Blast.cgi#50511340%2350511340) | 16.8 | 83% | 938 |
| [NP_001076442.1](http://www.ncbi.nlm.nih.gov/entrez/query.fcgi?cmd=Retrieve&db=Protein&list_uids=131889097&dopt=GenPept&RID=TAK4YPNW01S&log$=prottop&blast_rank=47) | cancer susceptibility candidate protein 1 isoform b [Homo sapiens] | [16.8](http://blast.ncbi.nlm.nih.gov/Blast.cgi#131889097%23131889097) | 16.8 | 83% | 938 |
| [NP_002365.3](http://www.ncbi.nlm.nih.gov/entrez/query.fcgi?cmd=Retrieve&db=Protein&list_uids=87578396&dopt=GenPept&RID=TAK4YPNW01S&log$=prottop&blast_rank=48) | microtubule-associated protein 2 isoform 1 [Homo sapiens] | [16.8](http://blast.ncbi.nlm.nih.gov/Blast.cgi#87578396%2387578396) | 16.8 | 100% | 938 |
| [NP_612488.2](http://www.ncbi.nlm.nih.gov/entrez/query.fcgi?cmd=Retrieve&db=Protein&list_uids=57232752&dopt=GenPept&RID=TAK4YPNW01S&log$=prottop&blast_rank=49) | putative uncharacterized protein C1orf213 isoform 1 [Homo sapiens] | [16.8](http://blast.ncbi.nlm.nih.gov/Blast.cgi#57232752%2357232752) | 16.8 | 83% | 938 |
| [NP_001076441.1](http://www.ncbi.nlm.nih.gov/entrez/query.fcgi?cmd=Retrieve&db=Protein&list_uids=131889447&dopt=GenPept&RID=TAK4YPNW01S&log$=prottop&blast_rank=50) | cancer susceptibility candidate protein 1 isoform c [Homo sapiens] | [16.8](http://blast.ncbi.nlm.nih.gov/Blast.cgi#131889447%23131889447) | 16.8 | 83% | 938 |
| [NP_001035962.1](http://www.ncbi.nlm.nih.gov/entrez/query.fcgi?cmd=Retrieve&db=Protein&list_uids=110224458&dopt=GenPept&RID=TAK4YPNW01S&log$=prottop&blast_rank=51) | solute carrier family 12 member 6 isoform e [Homo sapiens] | [16.8](http://blast.ncbi.nlm.nih.gov/Blast.cgi#110224458%23110224458) | 16.8 | 100% | 938 |
| [NP_110445.1](http://www.ncbi.nlm.nih.gov/entrez/query.fcgi?cmd=Retrieve&db=Protein&list_uids=13540614&dopt=GenPept&RID=TAK4YPNW01S&log$=prottop&blast_rank=52) | coiled-coil domain-containing protein 130 [Homo sapiens] | [16.8](http://blast.ncbi.nlm.nih.gov/Blast.cgi#13540614%2313540614) | 16.8 | 83% | 938 |
| [NP_003425.2](http://www.ncbi.nlm.nih.gov/entrez/query.fcgi?cmd=Retrieve&db=Protein&list_uids=27545332&dopt=GenPept&RID=TAK4YPNW01S&log$=prottop&blast_rank=53) | zinc finger protein 133 [Homo sapiens] >ref|NP_001076799.1| zinc finger protein 133 [Homo sapiens] | [16.8](http://blast.ncbi.nlm.nih.gov/Blast.cgi#27545332%2327545332) | 16.8 | 100% | 938 |
| [NP_114382.1](http://www.ncbi.nlm.nih.gov/entrez/query.fcgi?cmd=Retrieve&db=Protein&list_uids=14196459&dopt=GenPept&RID=TAK4YPNW01S&log$=prottop&blast_rank=54) | protocadherin gamma subfamily A, 1 isoform 2 precursor [Homo sapiens] | [16.8](http://blast.ncbi.nlm.nih.gov/Blast.cgi#14196459%2314196459) | 16.8 | 100% | 938 |
| [NP_061735.1](http://www.ncbi.nlm.nih.gov/entrez/query.fcgi?cmd=Retrieve&db=Protein&list_uids=11056032&dopt=GenPept&RID=TAK4YPNW01S&log$=prottop&blast_rank=55) | protocadherin gamma subfamily A, 1 isoform 1 precursor [Homo sapiens] | [16.8](http://blast.ncbi.nlm.nih.gov/Blast.cgi#11056032%2311056032) | 16.8 | 100% | 938 |
| [NP_002989.2](http://www.ncbi.nlm.nih.gov/entrez/query.fcgi?cmd=Retrieve&db=Protein&list_uids=40548378&dopt=GenPept&RID=TAK4YPNW01S&log$=prottop&blast_rank=56) | syndecan 2 precursor [Homo sapiens] | [16.8](http://blast.ncbi.nlm.nih.gov/Blast.cgi#40548378%2340548378) | 16.8 | 100% | 938 |
| [NP_660282.2](http://www.ncbi.nlm.nih.gov/entrez/query.fcgi?cmd=Retrieve&db=Protein&list_uids=156523246&dopt=GenPept&RID=TAK4YPNW01S&log$=prottop&blast_rank=57) | proline-rich transmembrane protein 2 [Homo sapiens] | [16.8](http://blast.ncbi.nlm.nih.gov/Blast.cgi#156523246%23156523246) | 16.8 | 83% | 938 |
| [NP_114477.1](http://www.ncbi.nlm.nih.gov/entrez/query.fcgi?cmd=Retrieve&db=Protein&list_uids=14270484&dopt=GenPept&RID=TAK4YPNW01S&log$=prottop&blast_rank=58) | protocadherin gamma subfamily A, 8 isoform 1 precursor [Homo sapiens] | [16.8](http://blast.ncbi.nlm.nih.gov/Blast.cgi#14270484%2314270484) | 16.8 | 100% | 938 |
| [NP_054723.1](http://www.ncbi.nlm.nih.gov/entrez/query.fcgi?cmd=Retrieve&db=Protein&list_uids=7662056&dopt=GenPept&RID=TAK4YPNW01S&log$=prottop&blast_rank=59) | protocadherin gamma subfamily A, 8 isoform 2 precursor [Homo sapiens] | [16.8](http://blast.ncbi.nlm.nih.gov/Blast.cgi#7662056%237662056) | 16.8 | 100% | 938 |
| [NP_570612.1](http://www.ncbi.nlm.nih.gov/entrez/query.fcgi?cmd=Retrieve&db=Protein&list_uids=19882249&dopt=GenPept&RID=TAK4YPNW01S&log$=prottop&blast_rank=60) | cystatin 11 isoform 1 precursor [Homo sapiens] | [16.8](http://blast.ncbi.nlm.nih.gov/Blast.cgi#19882249%2319882249) | 16.8 | 83% | 938 |
| [NP_005266.2](http://www.ncbi.nlm.nih.gov/entrez/query.fcgi?cmd=Retrieve&db=Protein&list_uids=38788319&dopt=GenPept&RID=TAK4YPNW01S&log$=prottop&blast_rank=61) | guanine nucleotide-binding protein-like 1 [Homo sapiens] | [16.8](http://blast.ncbi.nlm.nih.gov/Blast.cgi#38788319%2338788319) | 16.8 | 83% | 938 |
| [NP_060555.2](http://www.ncbi.nlm.nih.gov/entrez/query.fcgi?cmd=Retrieve&db=Protein&list_uids=21361659&dopt=GenPept&RID=TAK4YPNW01S&log$=prottop&blast_rank=62) | importin-9 [Homo sapiens] | [16.8](http://blast.ncbi.nlm.nih.gov/Blast.cgi#21361659%2321361659) | 16.8 | 83% | 938 |
| [NP_002493.3](http://www.ncbi.nlm.nih.gov/entrez/query.fcgi?cmd=Retrieve&db=Protein&list_uids=117320527&dopt=GenPept&RID=TAK4YPNW01S&log$=prottop&blast_rank=63) | nuclear factor NF-kappa-B p100 subunit isoform b [Homo sapiens] >ref|NP_001070961.1| nuclear factor NF-kappa-B p100 subunit isoform b [Homo sapiens] | [16.8](http://blast.ncbi.nlm.nih.gov/Blast.cgi#117320527%23117320527) | 16.8 | 100% | 938 |
| [NP_055527.1](http://www.ncbi.nlm.nih.gov/entrez/query.fcgi?cmd=Retrieve&db=Protein&list_uids=55741677&dopt=GenPept&RID=TAK4YPNW01S&log$=prottop&blast_rank=64) | histone-lysine N-methyltransferase SETD1A [Homo sapiens] | [16.8](http://blast.ncbi.nlm.nih.gov/Blast.cgi#55741677%2355741677) | 16.8 | 83% | 938 |
| [NP_291032.2](http://www.ncbi.nlm.nih.gov/entrez/query.fcgi?cmd=Retrieve&db=Protein&list_uids=24797074&dopt=GenPept&RID=TAK4YPNW01S&log$=prottop&blast_rank=65) | major histocompatibility complex, class II, DP alpha 1 precursor [Homo sapiens] | [16.8](http://blast.ncbi.nlm.nih.gov/Blast.cgi#24797074%2324797074) | 16.8 | 83% | 938 |
| [NP_000928.1](http://www.ncbi.nlm.nih.gov/entrez/query.fcgi?cmd=Retrieve&db=Protein&list_uids=4505939&dopt=GenPept&RID=TAK4YPNW01S&log$=prottop&blast_rank=66) | DNA-directed RNA polymerase II subunit RPB1 [Homo sapiens] | [16.8](http://blast.ncbi.nlm.nih.gov/Blast.cgi#4505939%234505939) | 16.8 | 83% | 938 |
| [NP_061984.2](http://www.ncbi.nlm.nih.gov/entrez/query.fcgi?cmd=Retrieve&db=Protein&list_uids=52426774&dopt=GenPept&RID=TAK4YPNW01S&log$=prottop&blast_rank=67) | major histocompatibility complex, class II, DR alpha precursor [Homo sapiens] | [16.8](http://blast.ncbi.nlm.nih.gov/Blast.cgi#52426774%2352426774) | 16.8 | 83% | 938 |
| [NP_009109.3](http://www.ncbi.nlm.nih.gov/entrez/query.fcgi?cmd=Retrieve&db=Protein&list_uids=148727341&dopt=GenPept&RID=TAK4YPNW01S&log$=prottop&blast_rank=68) | serine-threonine kinase receptor-associated protein [Homo sapiens] | [16.8](http://blast.ncbi.nlm.nih.gov/Blast.cgi#148727341%23148727341) | 16.8 | 83% | 938 |
| [NP_598408.1](http://www.ncbi.nlm.nih.gov/entrez/query.fcgi?cmd=Retrieve&db=Protein&list_uids=110224449&dopt=GenPept&RID=TAK4YPNW01S&log$=prottop&blast_rank=69) | solute carrier family 12 member 6 isoform a [Homo sapiens] | [16.8](http://blast.ncbi.nlm.nih.gov/Blast.cgi#110224449%23110224449) | 16.8 | 100% | 938 |
| [NP_001070962.1](http://www.ncbi.nlm.nih.gov/entrez/query.fcgi?cmd=Retrieve&db=Protein&list_uids=117320531&dopt=GenPept&RID=TAK4YPNW01S&log$=prottop&blast_rank=70) | nuclear factor NF-kappa-B p100 subunit isoform a [Homo sapiens] | [16.8](http://blast.ncbi.nlm.nih.gov/Blast.cgi#117320531%23117320531) | 16.8 | 100% | 938 |
| [NP_001153610.1](http://www.ncbi.nlm.nih.gov/entrez/query.fcgi?cmd=Retrieve&db=Protein&list_uids=236463300&dopt=GenPept&RID=TAK4YPNW01S&log$=prottop&blast_rank=71) | hypothetical protein LOC79846 isoform 2 [Homo sapiens] | [16.3](http://blast.ncbi.nlm.nih.gov/Blast.cgi#236463300%23236463300) | 16.3 | 100% | 1259 |
| [NP_001034795.2](http://www.ncbi.nlm.nih.gov/entrez/query.fcgi?cmd=Retrieve&db=Protein&list_uids=236463164&dopt=GenPept&RID=TAK4YPNW01S&log$=prottop&blast_rank=72) | hypothetical protein LOC79846 isoform 1 [Homo sapiens] | [16.3](http://blast.ncbi.nlm.nih.gov/Blast.cgi#236463164%23236463164) | 16.3 | 100% | 1259 |
| [NP_065952.2](http://www.ncbi.nlm.nih.gov/entrez/query.fcgi?cmd=Retrieve&db=Protein&list_uids=221139764&dopt=GenPept&RID=TAK4YPNW01S&log$=prottop&blast_rank=73) | PHD and RING finger domain-containing protein 1 [Homo sapiens] | [16.3](http://blast.ncbi.nlm.nih.gov/Blast.cgi#221139764%23221139764) | 16.3 | 83% | 1259 |
| [NP_001368.2](http://www.ncbi.nlm.nih.gov/entrez/query.fcgi?cmd=Retrieve&db=Protein&list_uids=283806679&dopt=GenPept&RID=TAK4YPNW01S&log$=prottop&blast_rank=74) | cytoplasmic dynein 2 heavy chain 1 isoform 1 [Homo sapiens] | [16.3](http://blast.ncbi.nlm.nih.gov/Blast.cgi#283806679%23283806679) | 16.3 | 100% | 1259 |
| [NP_004658.3](http://www.ncbi.nlm.nih.gov/entrez/query.fcgi?cmd=Retrieve&db=Protein&list_uids=126032348&dopt=GenPept&RID=TAK4YPNW01S&log$=prottop&blast_rank=75) | probable E3 ubiquitin-protein ligase HERC2 [Homo sapiens] | [16.3](http://blast.ncbi.nlm.nih.gov/Blast.cgi#126032348%23126032348) | 31.8 | 100% | 1259 |
| [NP_001073932.1](http://www.ncbi.nlm.nih.gov/entrez/query.fcgi?cmd=Retrieve&db=Protein&list_uids=122937398&dopt=GenPept&RID=TAK4YPNW01S&log$=prottop&blast_rank=76) | cytoplasmic dynein 2 heavy chain 1 isoform 2 [Homo sapiens] | [16.3](http://blast.ncbi.nlm.nih.gov/Blast.cgi#122937398%23122937398) | 16.3 | 100% | 1259 |
| [NP_001121698.1](http://www.ncbi.nlm.nih.gov/entrez/query.fcgi?cmd=Retrieve&db=Protein&list_uids=190014625&dopt=GenPept&RID=TAK4YPNW01S&log$=prottop&blast_rank=77) | exosome complex exonuclease RRP44 isoform b [Homo sapiens] | [16.3](http://blast.ncbi.nlm.nih.gov/Blast.cgi#190014625%23190014625) | 16.3 | 100% | 1259 |
| [NP_001007157.1](http://www.ncbi.nlm.nih.gov/entrez/query.fcgi?cmd=Retrieve&db=Protein&list_uids=55956794&dopt=GenPept&RID=TAK4YPNW01S&log$=prottop&blast_rank=78) | neurotrophic tyrosine kinase, receptor, type 3 isoform c precursor [Homo sapiens] | [16.3](http://blast.ncbi.nlm.nih.gov/Blast.cgi#55956794%2355956794) | 16.3 | 83% | 1259 |
| [NP_001092103.1](http://www.ncbi.nlm.nih.gov/entrez/query.fcgi?cmd=Retrieve&db=Protein&list_uids=148806884&dopt=GenPept&RID=TAK4YPNW01S&log$=prottop&blast_rank=79) | proline-rich AKT1 substrate 1 [Homo sapiens] >ref|NP_115751.2| proline-rich AKT1 substrate 1 [Homo sapiens] >ref|NP_001092102.1| proline-rich AKT1 substrate 1 [Homo sapiens] | [16.3](http://blast.ncbi.nlm.nih.gov/Blast.cgi#148806884%23148806884) | 16.3 | 83% | 1259 |
| [NP_057458.4](http://www.ncbi.nlm.nih.gov/entrez/query.fcgi?cmd=Retrieve&db=Protein&list_uids=22035677&dopt=GenPept&RID=TAK4YPNW01S&log$=prottop&blast_rank=80) | AT-rich interactive domain-containing protein 4B isoform 1 [Homo sapiens] | [16.3](http://blast.ncbi.nlm.nih.gov/Blast.cgi#22035677%2322035677) | 16.3 | 83% | 1259 |
| [NP_002521.2](http://www.ncbi.nlm.nih.gov/entrez/query.fcgi?cmd=Retrieve&db=Protein&list_uids=59889560&dopt=GenPept&RID=TAK4YPNW01S&log$=prottop&blast_rank=81) | neurotrophic tyrosine kinase, receptor, type 3 isoform b precursor [Homo sapiens] | [16.3](http://blast.ncbi.nlm.nih.gov/Blast.cgi#59889560%2359889560) | 16.3 | 83% | 1259 |
| [NP_112739.2](http://www.ncbi.nlm.nih.gov/entrez/query.fcgi?cmd=Retrieve&db=Protein&list_uids=22035679&dopt=GenPept&RID=TAK4YPNW01S&log$=prottop&blast_rank=82) | AT-rich interactive domain-containing protein 4B isoform 2 [Homo sapiens] | [16.3](http://blast.ncbi.nlm.nih.gov/Blast.cgi#22035679%2322035679) | 16.3 | 83% | 1259 |
| [NP_777567.1](http://www.ncbi.nlm.nih.gov/entrez/query.fcgi?cmd=Retrieve&db=Protein&list_uids=28372531&dopt=GenPept&RID=TAK4YPNW01S&log$=prottop&blast_rank=83) | serine/threonine-protein phosphatase 4 regulatory subunit 2 [Homo sapiens] | [16.3](http://blast.ncbi.nlm.nih.gov/Blast.cgi#28372531%2328372531) | 16.3 | 83% | 1259 |
| [NP_954575.1](http://www.ncbi.nlm.nih.gov/entrez/query.fcgi?cmd=Retrieve&db=Protein&list_uids=40217801&dopt=GenPept&RID=TAK4YPNW01S&log$=prottop&blast_rank=84) | hypothetical protein LOC79864 isoform 2 [Homo sapiens] | [16.3](http://blast.ncbi.nlm.nih.gov/Blast.cgi#40217801%2340217801) | 16.3 | 83% | 1259 |
| [NP_079082.2](http://www.ncbi.nlm.nih.gov/entrez/query.fcgi?cmd=Retrieve&db=Protein&list_uids=40217799&dopt=GenPept&RID=TAK4YPNW01S&log$=prottop&blast_rank=85) | hypothetical protein LOC79864 isoform 1 [Homo sapiens] | [16.3](http://blast.ncbi.nlm.nih.gov/Blast.cgi#40217799%2340217799) | 16.3 | 83% | 1259 |
| [NP_945353.1](http://www.ncbi.nlm.nih.gov/entrez/query.fcgi?cmd=Retrieve&db=Protein&list_uids=39777606&dopt=GenPept&RID=TAK4YPNW01S&log$=prottop&blast_rank=86) | rho guanine nucleotide exchange factor 1 isoform 1 [Homo sapiens] | [16.3](http://blast.ncbi.nlm.nih.gov/Blast.cgi#39777606%2339777606) | 16.3 | 83% | 1259 |
| [NP_003898.2](http://www.ncbi.nlm.nih.gov/entrez/query.fcgi?cmd=Retrieve&db=Protein&list_uids=83267879&dopt=GenPept&RID=TAK4YPNW01S&log$=prottop&blast_rank=87) | translation initiation factor eIF-2B subunit epsilon [Homo sapiens] | [16.3](http://blast.ncbi.nlm.nih.gov/Blast.cgi#83267879%2383267879) | 16.3 | 83% | 1259 |
| [NP_945328.1](http://www.ncbi.nlm.nih.gov/entrez/query.fcgi?cmd=Retrieve&db=Protein&list_uids=39777604&dopt=GenPept&RID=TAK4YPNW01S&log$=prottop&blast_rank=88) | rho guanine nucleotide exchange factor 1 isoform 3 [Homo sapiens] | [16.3](http://blast.ncbi.nlm.nih.gov/Blast.cgi#39777604%2339777604) | 16.3 | 83% | 1259 |
| [NP_004697.2](http://www.ncbi.nlm.nih.gov/entrez/query.fcgi?cmd=Retrieve&db=Protein&list_uids=15011972&dopt=GenPept&RID=TAK4YPNW01S&log$=prottop&blast_rank=89) | rho guanine nucleotide exchange factor 1 isoform 2 [Homo sapiens] | [16.3](http://blast.ncbi.nlm.nih.gov/Blast.cgi#15011972%2315011972) | 16.3 | 83% | 1259 |
| [NP_001106697.1](http://www.ncbi.nlm.nih.gov/entrez/query.fcgi?cmd=Retrieve&db=Protein&list_uids=164414431&dopt=GenPept&RID=TAK4YPNW01S&log$=prottop&blast_rank=90) | netrin-G1 isoform 1 [Homo sapiens] | [16.3](http://blast.ncbi.nlm.nih.gov/Blast.cgi#164414431%23164414431) | 16.3 | 83% | 1259 |
| [NP_066941.1](http://www.ncbi.nlm.nih.gov/entrez/query.fcgi?cmd=Retrieve&db=Protein&list_uids=117935038&dopt=GenPept&RID=TAK4YPNW01S&log$=prottop&blast_rank=91) | cylicin-1 [Homo sapiens] | [16.3](http://blast.ncbi.nlm.nih.gov/Blast.cgi#117935038%23117935038) | 16.3 | 83% | 1259 |
| [NP_958357.1](http://www.ncbi.nlm.nih.gov/entrez/query.fcgi?cmd=Retrieve&db=Protein&list_uids=41152093&dopt=GenPept&RID=TAK4YPNW01S&log$=prottop&blast_rank=92) | zinc finger protein 644 isoform 1 [Homo sapiens] | [16.3](http://blast.ncbi.nlm.nih.gov/Blast.cgi#41152093%2341152093) | 31.0 | 100% | 1259 |
| [NP_008877.1](http://www.ncbi.nlm.nih.gov/entrez/query.fcgi?cmd=Retrieve&db=Protein&list_uids=5902122&dopt=GenPept&RID=TAK4YPNW01S&log$=prottop&blast_rank=93) | spectrin beta chain, brain 2 [Homo sapiens] | [16.3](http://blast.ncbi.nlm.nih.gov/Blast.cgi#5902122%235902122) | 16.3 | 83% | 1259 |
| [NP_055732.2](http://www.ncbi.nlm.nih.gov/entrez/query.fcgi?cmd=Retrieve&db=Protein&list_uids=54312078&dopt=GenPept&RID=TAK4YPNW01S&log$=prottop&blast_rank=94) | netrin-G1 isoform 3 [Homo sapiens] | [16.3](http://blast.ncbi.nlm.nih.gov/Blast.cgi#54312078%2354312078) | 16.3 | 83% | 1259 |
| [NP_079034.3](http://www.ncbi.nlm.nih.gov/entrez/query.fcgi?cmd=Retrieve&db=Protein&list_uids=37537722&dopt=GenPept&RID=TAK4YPNW01S&log$=prottop&blast_rank=95) | agmatinase, mitochondrial precursor [Homo sapiens] | [16.3](http://blast.ncbi.nlm.nih.gov/Blast.cgi#37537722%2337537722) | 16.3 | 83% | 1259 |
| [NP_055768.3](http://www.ncbi.nlm.nih.gov/entrez/query.fcgi?cmd=Retrieve&db=Protein&list_uids=190014623&dopt=GenPept&RID=TAK4YPNW01S&log$=prottop&blast_rank=96) | exosome complex exonuclease RRP44 isoform a [Homo sapiens] | [16.3](http://blast.ncbi.nlm.nih.gov/Blast.cgi#190014623%23190014623) | 16.3 | 100% | 1259 |
| [NP_001012338.1](http://www.ncbi.nlm.nih.gov/entrez/query.fcgi?cmd=Retrieve&db=Protein&list_uids=59889562&dopt=GenPept&RID=TAK4YPNW01S&log$=prottop&blast_rank=97) | neurotrophic tyrosine kinase, receptor, type 3 isoform a precursor [Homo sapiens] | [16.3](http://blast.ncbi.nlm.nih.gov/Blast.cgi#59889562%2359889562) | 16.3 | 83% | 1259 |
| [NP_001106699.1](http://www.ncbi.nlm.nih.gov/entrez/query.fcgi?cmd=Retrieve&db=Protein&list_uids=164414437&dopt=GenPept&RID=TAK4YPNW01S&log$=prottop&blast_rank=98) | netrin-G1 isoform 2 [Homo sapiens] | [16.3](http://blast.ncbi.nlm.nih.gov/Blast.cgi#164414437%23164414437) | 16.3 | 83% | 1259 |
| [NP_001041683.1](http://www.ncbi.nlm.nih.gov/entrez/query.fcgi?cmd=Retrieve&db=Protein&list_uids=115430243&dopt=GenPept&RID=TAK4YPNW01S&log$=prottop&blast_rank=99) | N-terminal kinase-like protein isoform B [Homo sapiens] | [16.3](http://blast.ncbi.nlm.nih.gov/Blast.cgi#115430243%23115430243) | 16.3 | 100% | 1259 |
| [NP_065731.3](http://www.ncbi.nlm.nih.gov/entrez/query.fcgi?cmd=Retrieve&db=Protein&list_uids=115430241&dopt=GenPept&RID=TAK4YPNW01S&log$=prottop&blast_rank=100) | N-terminal kinase-like protein isoform A [Homo sapiens] | [16.3](http://blast.ncbi.nlm.nih.gov/Blast.cgi#115430241%23115430241) | 16.3 | 100% | 1259 |

| **Accession** | **Proteins with a match to HVRGTLGR peptide** | **[Max score](http://blast.ncbi.nlm.nih.gov/Blast.cgi?CMD=Get&ALIGNMENTS=100&ALIGNMENT_VIEW=Pairwise&CDD_SEARCH_STATE=1&DATABASE_SORT=0&DESCRIPTIONS=100&ENTREZ_QUERY=txid9606 %5BORGN%5D&FIRST_QUERY_NUM=0&FORMAT_OBJECT=Alignment&FORMAT_PAGE_TARGET=&FORMAT_TYPE=HTML&GET_SEQUENCE=yes&I_THRESH=&MASK_CHAR=2&MASK_COLOR=1&NEW_VIEW=yes&NUM_OVERVIEW=100&OLD_BLAST=false&PAGE=Proteins&QUERY_INDEX=0&QUERY_NUMBER=0&RESULTS_PAGE_TARGET=&RID=TAK97135016&SHOW_LINKOUT=yes&SHOW_OVERVIEW=yes&STEP_NUMBER=&WORD_SIZE=2&DISPLAY_SORT=1&HSP_SORT=1" \l "sort_mark)** | **[Total score](http://blast.ncbi.nlm.nih.gov/Blast.cgi?CMD=Get&ALIGNMENTS=100&ALIGNMENT_VIEW=Pairwise&CDD_SEARCH_STATE=1&DATABASE_SORT=0&DESCRIPTIONS=100&ENTREZ_QUERY=txid9606 %5BORGN%5D&FIRST_QUERY_NUM=0&FORMAT_OBJECT=Alignment&FORMAT_PAGE_TARGET=&FORMAT_TYPE=HTML&GET_SEQUENCE=yes&I_THRESH=&MASK_CHAR=2&MASK_COLOR=1&NEW_VIEW=yes&NUM_OVERVIEW=100&OLD_BLAST=false&PAGE=Proteins&QUERY_INDEX=0&QUERY_NUMBER=0&RESULTS_PAGE_TARGET=&RID=TAK97135016&SHOW_LINKOUT=yes&SHOW_OVERVIEW=yes&STEP_NUMBER=&WORD_SIZE=2&DISPLAY_SORT=2&HSP_SORT=1" \l "sort_mark)** | **[Query coverage](http://blast.ncbi.nlm.nih.gov/Blast.cgi?CMD=Get&ALIGNMENTS=100&ALIGNMENT_VIEW=Pairwise&CDD_SEARCH_STATE=1&DATABASE_SORT=0&DESCRIPTIONS=100&ENTREZ_QUERY=txid9606 %5BORGN%5D&FIRST_QUERY_NUM=0&FORMAT_OBJECT=Alignment&FORMAT_PAGE_TARGET=&FORMAT_TYPE=HTML&GET_SEQUENCE=yes&I_THRESH=&MASK_CHAR=2&MASK_COLOR=1&NEW_VIEW=yes&NUM_OVERVIEW=100&OLD_BLAST=false&PAGE=Proteins&QUERY_INDEX=0&QUERY_NUMBER=0&RESULTS_PAGE_TARGET=&RID=TAK97135016&SHOW_LINKOUT=yes&SHOW_OVERVIEW=yes&STEP_NUMBER=&WORD_SIZE=2&DISPLAY_SORT=4&HSP_SORT=0" \l "sort_mark)** | **[E value](http://blast.ncbi.nlm.nih.gov/Blast.cgi?CMD=Get&ALIGNMENTS=100&ALIGNMENT_VIEW=Pairwise&CDD_SEARCH_STATE=1&DATABASE_SORT=0&DESCRIPTIONS=100&ENTREZ_QUERY=txid9606 %5BORGN%5D&FIRST_QUERY_NUM=0&FORMAT_OBJECT=Alignment&FORMAT_PAGE_TARGET=&FORMAT_TYPE=HTML&GET_SEQUENCE=yes&I_THRESH=&MASK_CHAR=2&MASK_COLOR=1&NEW_VIEW=yes&NUM_OVERVIEW=100&OLD_BLAST=false&PAGE=Proteins&QUERY_INDEX=0&QUERY_NUMBER=0&RESULTS_PAGE_TARGET=&RID=TAK97135016&SHOW_LINKOUT=yes&SHOW_OVERVIEW=yes&STEP_NUMBER=&WORD_SIZE=2&DISPLAY_SORT=0&HSP_SORT=0" \l "sort_mark)** |
| --- | --- | --- | --- | --- | --- |
| [XP_002346352.1](http://www.ncbi.nlm.nih.gov/entrez/query.fcgi?cmd=Retrieve&db=Protein&list_uids=239757832&dopt=GenPept&RID=TAK97135016&log$=prottop&blast_rank=1) | PREDICTED: hypothetical protein [Homo sapiens] | [21.0](http://blast.ncbi.nlm.nih.gov/Blast.cgi#239757832%23239757832) | 21.0 | 75% | 66 |
| [XP_002347677.1](http://www.ncbi.nlm.nih.gov/entrez/query.fcgi?cmd=Retrieve&db=Protein&list_uids=239751024&dopt=GenPept&RID=TAK97135016&log$=prottop&blast_rank=2) | PREDICTED: hypothetical protein XP_002347677 [Homo sapiens] | [21.0](http://blast.ncbi.nlm.nih.gov/Blast.cgi#239751024%23239751024) | 21.0 | 75% | 66 |
| [XP_002343859.1](http://www.ncbi.nlm.nih.gov/entrez/query.fcgi?cmd=Retrieve&db=Protein&list_uids=239746856&dopt=GenPept&RID=TAK97135016&log$=prottop&blast_rank=3) | PREDICTED: hypothetical protein XP_002343859 [Homo sapiens] >ref|XP_002348203.1| PREDICTED: hypothetical protein [Homo sapiens] | [21.0](http://blast.ncbi.nlm.nih.gov/Blast.cgi#239746856%23239746856) | 21.0 | 75% | 66 |
| [XP_002343495.1](http://www.ncbi.nlm.nih.gov/entrez/query.fcgi?cmd=Retrieve&db=Protein&list_uids=239745453&dopt=GenPept&RID=TAK97135016&log$=prottop&blast_rank=4) | PREDICTED: hypothetical protein XP_002343495 [Homo sapiens] | [21.0](http://blast.ncbi.nlm.nih.gov/Blast.cgi#239745453%23239745453) | 21.0 | 75% | 66 |
| [NP_055809.2](http://www.ncbi.nlm.nih.gov/entrez/query.fcgi?cmd=Retrieve&db=Protein&list_uids=190684671&dopt=GenPept&RID=TAK97135016&log$=prottop&blast_rank=5) | mitogen-activated protein kinase-binding protein 1 isoform a [Homo sapiens] | [21.0](http://blast.ncbi.nlm.nih.gov/Blast.cgi#190684671%23190684671) | 21.0 | 75% | 66 |
| [NP_001074419.1](http://www.ncbi.nlm.nih.gov/entrez/query.fcgi?cmd=Retrieve&db=Protein&list_uids=124494247&dopt=GenPept&RID=TAK97135016&log$=prottop&blast_rank=6) | myosin-Ic isoform b [Homo sapiens] | [21.0](http://blast.ncbi.nlm.nih.gov/Blast.cgi#124494247%23124494247) | 33.1 | 100% | 66 |
| [NP_001074248.1](http://www.ncbi.nlm.nih.gov/entrez/query.fcgi?cmd=Retrieve&db=Protein&list_uids=124494238&dopt=GenPept&RID=TAK97135016&log$=prottop&blast_rank=7) | myosin-Ic isoform a [Homo sapiens] | [21.0](http://blast.ncbi.nlm.nih.gov/Blast.cgi#124494238%23124494238) | 33.1 | 100% | 66 |
| [NP_203693.3](http://www.ncbi.nlm.nih.gov/entrez/query.fcgi?cmd=Retrieve&db=Protein&list_uids=124494240&dopt=GenPept&RID=TAK97135016&log$=prottop&blast_rank=8) | myosin-Ic isoform c [Homo sapiens] | [21.0](http://blast.ncbi.nlm.nih.gov/Blast.cgi#124494240%23124494240) | 33.1 | 100% | 66 |
| [NP_001122080.1](http://www.ncbi.nlm.nih.gov/entrez/query.fcgi?cmd=Retrieve&db=Protein&list_uids=190684677&dopt=GenPept&RID=TAK97135016&log$=prottop&blast_rank=9) | mitogen-activated protein kinase-binding protein 1 isoform b [Homo sapiens] | [21.0](http://blast.ncbi.nlm.nih.gov/Blast.cgi#190684677%23190684677) | 21.0 | 75% | 66 |
| [NP_001035805.1](http://www.ncbi.nlm.nih.gov/entrez/query.fcgi?cmd=Retrieve&db=Protein&list_uids=106507204&dopt=GenPept&RID=TAK97135016&log$=prottop&blast_rank=10) | hypothetical protein LOC653319 [Homo sapiens] | [21.0](http://blast.ncbi.nlm.nih.gov/Blast.cgi#106507204%23106507204) | 21.0 | 75% | 66 |
| [NP_001012768.1](http://www.ncbi.nlm.nih.gov/entrez/query.fcgi?cmd=Retrieve&db=Protein&list_uids=61743944&dopt=GenPept&RID=TAK97135016&log$=prottop&blast_rank=11) | abl interactor 1 isoform b [Homo sapiens] | [21.0](http://blast.ncbi.nlm.nih.gov/Blast.cgi#61743944%2361743944) | 21.0 | 75% | 66 |
| [NP_001012770.1](http://www.ncbi.nlm.nih.gov/entrez/query.fcgi?cmd=Retrieve&db=Protein&list_uids=61743948&dopt=GenPept&RID=TAK97135016&log$=prottop&blast_rank=12) | abl interactor 1 isoform d [Homo sapiens] | [21.0](http://blast.ncbi.nlm.nih.gov/Blast.cgi#61743948%2361743948) | 21.0 | 75% | 66 |
| [NP_005461.2](http://www.ncbi.nlm.nih.gov/entrez/query.fcgi?cmd=Retrieve&db=Protein&list_uids=61743942&dopt=GenPept&RID=TAK97135016&log$=prottop&blast_rank=13) | abl interactor 1 isoform a [Homo sapiens] | [21.0](http://blast.ncbi.nlm.nih.gov/Blast.cgi#61743942%2361743942) | 21.0 | 75% | 66 |
| [NP_001012769.1](http://www.ncbi.nlm.nih.gov/entrez/query.fcgi?cmd=Retrieve&db=Protein&list_uids=61743946&dopt=GenPept&RID=TAK97135016&log$=prottop&blast_rank=14) | abl interactor 1 isoform c [Homo sapiens] | [21.0](http://blast.ncbi.nlm.nih.gov/Blast.cgi#61743946%2361743946) | 21.0 | 75% | 66 |
| [NP_006613.2](http://www.ncbi.nlm.nih.gov/entrez/query.fcgi?cmd=Retrieve&db=Protein&list_uids=93004081&dopt=GenPept&RID=TAK97135016&log$=prottop&blast_rank=15) | serine/threonine-protein kinase PLK2 [Homo sapiens] | [20.6](http://blast.ncbi.nlm.nih.gov/Blast.cgi#93004081%2393004081) | 20.6 | 75% | 89 |
| [NP_006694.1](http://www.ncbi.nlm.nih.gov/entrez/query.fcgi?cmd=Retrieve&db=Protein&list_uids=5729804&dopt=GenPept&RID=TAK97135016&log$=prottop&blast_rank=16) | diphosphoinositol polyphosphate phosphohydrolase 1 [Homo sapiens] | [20.6](http://blast.ncbi.nlm.nih.gov/Blast.cgi#5729804%235729804) | 20.6 | 87% | 89 |
| [NP_004938.1](http://www.ncbi.nlm.nih.gov/entrez/query.fcgi?cmd=Retrieve&db=Protein&list_uids=31415870&dopt=GenPept&RID=TAK97135016&log$=prottop&blast_rank=17) | dedicator of cytokinesis protein 3 [Homo sapiens] | [20.6](http://blast.ncbi.nlm.nih.gov/Blast.cgi#31415870%2331415870) | 20.6 | 75% | 89 |
| [XP_001715497.2](http://www.ncbi.nlm.nih.gov/entrez/query.fcgi?cmd=Retrieve&db=Protein&list_uids=239743415&dopt=GenPept&RID=TAK97135016&log$=prottop&blast_rank=18) | PREDICTED: hypothetical protein [Homo sapiens] >ref|XP_002346114.1| PREDICTED: hypothetical protein [Homo sapiens] | [20.2](http://blast.ncbi.nlm.nih.gov/Blast.cgi#239743415%23239743415) | 20.2 | 87% | 119 |
| [XP_002344479.1](http://www.ncbi.nlm.nih.gov/entrez/query.fcgi?cmd=Retrieve&db=Protein&list_uids=239509122&dopt=GenPept&RID=TAK97135016&log$=prottop&blast_rank=19) | PREDICTED: hypothetical protein [Homo sapiens] >ref|XP_001714494.2| PREDICTED: hypothetical protein [Homo sapiens] | [20.2](http://blast.ncbi.nlm.nih.gov/Blast.cgi#239509122%23239509122) | 20.2 | 87% | 119 |
| [XP_002345359.1](http://www.ncbi.nlm.nih.gov/entrez/query.fcgi?cmd=Retrieve&db=Protein&list_uids=239752732&dopt=GenPept&RID=TAK97135016&log$=prottop&blast_rank=20) | PREDICTED: hypothetical protein [Homo sapiens] | [18.9](http://blast.ncbi.nlm.nih.gov/Blast.cgi#239752732%23239752732) | 18.9 | 100% | 288 |
| [NP_653267.2](http://www.ncbi.nlm.nih.gov/entrez/query.fcgi?cmd=Retrieve&db=Protein&list_uids=222144249&dopt=GenPept&RID=TAK97135016&log$=prottop&blast_rank=21) | dynein heavy chain domain 1 isoform 1 [Homo sapiens] | [18.9](http://blast.ncbi.nlm.nih.gov/Blast.cgi#222144249%23222144249) | 18.9 | 87% | 288 |
| [NP_001124335.1](http://www.ncbi.nlm.nih.gov/entrez/query.fcgi?cmd=Retrieve&db=Protein&list_uids=195947389&dopt=GenPept&RID=TAK97135016&log$=prottop&blast_rank=22) | autotaxin isoform 3 preproprotein [Homo sapiens] | [18.9](http://blast.ncbi.nlm.nih.gov/Blast.cgi#195947389%23195947389) | 18.9 | 87% | 288 |
| [NP_001035181.1](http://www.ncbi.nlm.nih.gov/entrez/query.fcgi?cmd=Retrieve&db=Protein&list_uids=91823602&dopt=GenPept&RID=TAK97135016&log$=prottop&blast_rank=23) | autotaxin isoform 2 preproprotein [Homo sapiens] | [18.9](http://blast.ncbi.nlm.nih.gov/Blast.cgi#91823602%2391823602) | 18.9 | 87% | 288 |
| [NP_006200.3](http://www.ncbi.nlm.nih.gov/entrez/query.fcgi?cmd=Retrieve&db=Protein&list_uids=91823274&dopt=GenPept&RID=TAK97135016&log$=prottop&blast_rank=24) | autotaxin isoform 1 preproprotein [Homo sapiens] | [18.9](http://blast.ncbi.nlm.nih.gov/Blast.cgi#91823274%2391823274) | 18.9 | 87% | 288 |
| [NP_945320.1](http://www.ncbi.nlm.nih.gov/entrez/query.fcgi?cmd=Retrieve&db=Protein&list_uids=39812019&dopt=GenPept&RID=TAK97135016&log$=prottop&blast_rank=25) | amino-terminal enhancer of split isoform a [Homo sapiens] | [18.9](http://blast.ncbi.nlm.nih.gov/Blast.cgi#39812019%2339812019) | 18.9 | 62% | 288 |
| [NP_037371.2](http://www.ncbi.nlm.nih.gov/entrez/query.fcgi?cmd=Retrieve&db=Protein&list_uids=33695078&dopt=GenPept&RID=TAK97135016&log$=prottop&blast_rank=26) | serine/threonine-protein phosphatase 2A regulatory subunit B'' subunit beta isoform 1 [Homo sapiens] | [18.9](http://blast.ncbi.nlm.nih.gov/Blast.cgi#33695078%2333695078) | 18.9 | 62% | 288 |
| [XP_001716437.2](http://www.ncbi.nlm.nih.gov/entrez/query.fcgi?cmd=Retrieve&db=Protein&list_uids=239756862&dopt=GenPept&RID=TAK97135016&log$=prottop&blast_rank=27) | PREDICTED: hypothetical protein [Homo sapiens] | [18.5](http://blast.ncbi.nlm.nih.gov/Blast.cgi#239756862%23239756862) | 28.8 | 75% | 386 |
| [XP_370932.2](http://www.ncbi.nlm.nih.gov/entrez/query.fcgi?cmd=Retrieve&db=Protein&list_uids=113425870&dopt=GenPept&RID=TAK97135016&log$=prottop&blast_rank=28) | PREDICTED: hypothetical protein LOC283876 [Homo sapiens] >ref|XP_945182.2| PREDICTED: hypothetical protein FLJ39639 [Homo sapiens] >ref|XP_001715803.1| PREDICTED: hypothetical protein FLJ39639 [Homo sapiens] | [18.5](http://blast.ncbi.nlm.nih.gov/Blast.cgi#113425870%23113425870) | 18.5 | 75% | 386 |
| [NP_062831.3](http://www.ncbi.nlm.nih.gov/entrez/query.fcgi?cmd=Retrieve&db=Protein&list_uids=28559083&dopt=GenPept&RID=TAK97135016&log$=prottop&blast_rank=29) | CTP synthase 2 [Homo sapiens] >ref|NP_787055.1| CTP synthase 2 [Homo sapiens] >ref|NP_001137474.1| CTP synthase 2 [Homo sapiens] | [18.5](http://blast.ncbi.nlm.nih.gov/Blast.cgi#28559083%2328559083) | 18.5 | 75% | 386 |
| [XP_002346550.1](http://www.ncbi.nlm.nih.gov/entrez/query.fcgi?cmd=Retrieve&db=Protein&list_uids=239748088&dopt=GenPept&RID=TAK97135016&log$=prottop&blast_rank=30) | PREDICTED: hypothetical protein XP_002346550 [Homo sapiens] >ref|XP_002345681.1| PREDICTED: hypothetical protein [Homo sapiens] | [18.0](http://blast.ncbi.nlm.nih.gov/Blast.cgi#239748088%23239748088) | 18.0 | 62% | 518 |
| [XP_002344169.1](http://www.ncbi.nlm.nih.gov/entrez/query.fcgi?cmd=Retrieve&db=Protein&list_uids=239745652&dopt=GenPept&RID=TAK97135016&log$=prottop&blast_rank=31) | PREDICTED: hypothetical protein XP_002344169 [Homo sapiens] >ref|XP_002347734.1| PREDICTED: hypothetical protein XP_002347734 [Homo sapiens] >ref|XP_002344974.1| PREDICTED: hypothetical protein [Homo sapiens] | [18.0](http://blast.ncbi.nlm.nih.gov/Blast.cgi#239745652%23239745652) | 18.0 | 62% | 518 |
| [XP_002342357.1](http://www.ncbi.nlm.nih.gov/entrez/query.fcgi?cmd=Retrieve&db=Protein&list_uids=239741935&dopt=GenPept&RID=TAK97135016&log$=prottop&blast_rank=32) | PREDICTED: hypothetical protein XP_002342357 [Homo sapiens] >ref|XP_002346500.1| PREDICTED: hypothetical protein XP_002346500 [Homo sapiens] >ref|XP_002345669.1| PREDICTED: hypothetical protein [Homo sapiens] | [18.0](http://blast.ncbi.nlm.nih.gov/Blast.cgi#239741935%23239741935) | 18.0 | 62% | 518 |
| [NP_006812.3](http://www.ncbi.nlm.nih.gov/entrez/query.fcgi?cmd=Retrieve&db=Protein&list_uids=148727286&dopt=GenPept&RID=TAK97135016&log$=prottop&blast_rank=33) | acyl-coenzyme A thioesterase 2, mitochondrial [Homo sapiens] | [18.0](http://blast.ncbi.nlm.nih.gov/Blast.cgi#148727286%23148727286) | 18.0 | 62% | 518 |
| [NP_001096654.1](http://www.ncbi.nlm.nih.gov/entrez/query.fcgi?cmd=Retrieve&db=Protein&list_uids=157168329&dopt=GenPept&RID=TAK97135016&log$=prottop&blast_rank=34) | formin-1 [Homo sapiens] | [18.0](http://blast.ncbi.nlm.nih.gov/Blast.cgi#157168329%23157168329) | 31.8 | 75% | 518 |
| [NP_001020414.1](http://www.ncbi.nlm.nih.gov/entrez/query.fcgi?cmd=Retrieve&db=Protein&list_uids=68800350&dopt=GenPept&RID=TAK97135016&log$=prottop&blast_rank=35) | interleukin-1 receptor-associated kinase 1 isoform 3 [Homo sapiens] | [18.0](http://blast.ncbi.nlm.nih.gov/Blast.cgi#68800350%2368800350) | 18.0 | 62% | 518 |
| [NP_958435.1](http://www.ncbi.nlm.nih.gov/entrez/query.fcgi?cmd=Retrieve&db=Protein&list_uids=41350316&dopt=GenPept&RID=TAK97135016&log$=prottop&blast_rank=36) | myotubularin-related protein 2 isoform 2 [Homo sapiens] >ref|NP_958438.1| myotubularin-related protein 2 isoform 2 [Homo sapiens] | [18.0](http://blast.ncbi.nlm.nih.gov/Blast.cgi#41350316%2341350316) | 18.0 | 62% | 518 |
| [NP_057240.3](http://www.ncbi.nlm.nih.gov/entrez/query.fcgi?cmd=Retrieve&db=Protein&list_uids=44680154&dopt=GenPept&RID=TAK97135016&log$=prottop&blast_rank=37) | myotubularin-related protein 2 isoform 1 [Homo sapiens] | [18.0](http://blast.ncbi.nlm.nih.gov/Blast.cgi#44680154%2344680154) | 18.0 | 62% | 518 |
| [NP_001020413.1](http://www.ncbi.nlm.nih.gov/entrez/query.fcgi?cmd=Retrieve&db=Protein&list_uids=68800343&dopt=GenPept&RID=TAK97135016&log$=prottop&blast_rank=38) | interleukin-1 receptor-associated kinase 1 isoform 2 [Homo sapiens] | [18.0](http://blast.ncbi.nlm.nih.gov/Blast.cgi#68800343%2368800343) | 18.0 | 62% | 518 |
| [NP_001122105.1](http://www.ncbi.nlm.nih.gov/entrez/query.fcgi?cmd=Retrieve&db=Protein&list_uids=191252806&dopt=GenPept&RID=TAK97135016&log$=prottop&blast_rank=39) | RIMS-binding protein 3C [Homo sapiens] | [18.0](http://blast.ncbi.nlm.nih.gov/Blast.cgi#191252806%23191252806) | 18.0 | 87% | 518 |
| [NP_056487.1](http://www.ncbi.nlm.nih.gov/entrez/query.fcgi?cmd=Retrieve&db=Protein&list_uids=153792195&dopt=GenPept&RID=TAK97135016&log$=prottop&blast_rank=40) | RIMS-binding protein 3A [Homo sapiens] | [18.0](http://blast.ncbi.nlm.nih.gov/Blast.cgi#153792195%23153792195) | 18.0 | 87% | 518 |
| [NP_001122107.1](http://www.ncbi.nlm.nih.gov/entrez/query.fcgi?cmd=Retrieve&db=Protein&list_uids=191252814&dopt=GenPept&RID=TAK97135016&log$=prottop&blast_rank=41) | RIMS-binding protein 3B [Homo sapiens] | [18.0](http://blast.ncbi.nlm.nih.gov/Blast.cgi#191252814%23191252814) | 18.0 | 87% | 518 |
| [NP_115820.2](http://www.ncbi.nlm.nih.gov/entrez/query.fcgi?cmd=Retrieve&db=Protein&list_uids=63252863&dopt=GenPept&RID=TAK97135016&log$=prottop&blast_rank=42) | structure-specific endonuclease subunit SLX4 [Homo sapiens] | [18.0](http://blast.ncbi.nlm.nih.gov/Blast.cgi#63252863%2363252863) | 18.0 | 75% | 518 |
| [NP_001032238.1](http://www.ncbi.nlm.nih.gov/entrez/query.fcgi?cmd=Retrieve&db=Protein&list_uids=81230485&dopt=GenPept&RID=TAK97135016&log$=prottop&blast_rank=43) | acyl-coenzyme A thioesterase 1 [Homo sapiens] | [18.0](http://blast.ncbi.nlm.nih.gov/Blast.cgi#81230485%2381230485) | 18.0 | 62% | 518 |
| [NP_055365.2](http://www.ncbi.nlm.nih.gov/entrez/query.fcgi?cmd=Retrieve&db=Protein&list_uids=51093861&dopt=GenPept&RID=TAK97135016&log$=prottop&blast_rank=44) | caspase recruitment domain-containing protein 10 [Homo sapiens] | [18.0](http://blast.ncbi.nlm.nih.gov/Blast.cgi#51093861%2351093861) | 18.0 | 87% | 518 |
| [NP_061918.3](http://www.ncbi.nlm.nih.gov/entrez/query.fcgi?cmd=Retrieve&db=Protein&list_uids=45238858&dopt=GenPept&RID=TAK97135016&log$=prottop&blast_rank=45) | WD repeat domain 44 protein [Homo sapiens] | [18.0](http://blast.ncbi.nlm.nih.gov/Blast.cgi#45238858%2345238858) | 18.0 | 100% | 518 |
| [NP_076997.1](http://www.ncbi.nlm.nih.gov/entrez/query.fcgi?cmd=Retrieve&db=Protein&list_uids=13129092&dopt=GenPept&RID=TAK97135016&log$=prottop&blast_rank=46) | transmembrane protein 109 precursor [Homo sapiens] | [18.0](http://blast.ncbi.nlm.nih.gov/Blast.cgi#13129092%2313129092) | 18.0 | 62% | 518 |
| [NP_001560.2](http://www.ncbi.nlm.nih.gov/entrez/query.fcgi?cmd=Retrieve&db=Protein&list_uids=68800243&dopt=GenPept&RID=TAK97135016&log$=prottop&blast_rank=47) | interleukin-1 receptor-associated kinase 1 isoform 1 [Homo sapiens] | [18.0](http://blast.ncbi.nlm.nih.gov/Blast.cgi#68800243%2368800243) | 18.0 | 62% | 518 |
| [NP_002053.3](http://www.ncbi.nlm.nih.gov/entrez/query.fcgi?cmd=Retrieve&db=Protein&list_uids=166795283&dopt=GenPept&RID=TAK97135016&log$=prottop&blast_rank=48) | glucagon-like peptide 1 receptor precursor [Homo sapiens] | [18.0](http://blast.ncbi.nlm.nih.gov/Blast.cgi#166795283%23166795283) | 18.0 | 75% | 518 |
| [NP_056520.2](http://www.ncbi.nlm.nih.gov/entrez/query.fcgi?cmd=Retrieve&db=Protein&list_uids=29648315&dopt=GenPept&RID=TAK97135016&log$=prottop&blast_rank=49) | small G protein signaling modulator 3 [Homo sapiens] | [18.0](http://blast.ncbi.nlm.nih.gov/Blast.cgi#29648315%2329648315) | 18.0 | 62% | 518 |
| [XP_002343336.1](http://www.ncbi.nlm.nih.gov/entrez/query.fcgi?cmd=Retrieve&db=Protein&list_uids=239745032&dopt=GenPept&RID=TAK97135016&log$=prottop&blast_rank=50) | PREDICTED: hypothetical protein XP_002343336 [Homo sapiens] | [17.6](http://blast.ncbi.nlm.nih.gov/Blast.cgi#239745032%23239745032) | 17.6 | 62% | 695 |
| [NP_149043.2](http://www.ncbi.nlm.nih.gov/entrez/query.fcgi?cmd=Retrieve&db=Protein&list_uids=239582755&dopt=GenPept&RID=TAK97135016&log$=prottop&blast_rank=51) | myosin-Ig [Homo sapiens] | [17.6](http://blast.ncbi.nlm.nih.gov/Blast.cgi#239582755%23239582755) | 17.6 | 75% | 695 |
| [NP_001135775.1](http://www.ncbi.nlm.nih.gov/entrez/query.fcgi?cmd=Retrieve&db=Protein&list_uids=214831008&dopt=GenPept&RID=TAK97135016&log$=prottop&blast_rank=52) | high affinity immunoglobulin epsilon receptor subunit beta isoform 2 [Homo sapiens] | [17.6](http://blast.ncbi.nlm.nih.gov/Blast.cgi#214831008%23214831008) | 17.6 | 75% | 695 |
| [NP_001157586.1](http://www.ncbi.nlm.nih.gov/entrez/query.fcgi?cmd=Retrieve&db=Protein&list_uids=255759930&dopt=GenPept&RID=TAK97135016&log$=prottop&blast_rank=53) | cas scaffolding protein family member 4 isoform b [Homo sapiens] | [17.6](http://blast.ncbi.nlm.nih.gov/Blast.cgi#255759930%23255759930) | 17.6 | 62% | 695 |
| [NP_001129175.1](http://www.ncbi.nlm.nih.gov/entrez/query.fcgi?cmd=Retrieve&db=Protein&list_uids=208973262&dopt=GenPept&RID=TAK97135016&log$=prottop&blast_rank=54) | low density lipoprotein-related protein 12 isoform b precursor [Homo sapiens] | [17.6](http://blast.ncbi.nlm.nih.gov/Blast.cgi#208973262%23208973262) | 29.7 | 87% | 695 |
| [XP_001714323.1](http://www.ncbi.nlm.nih.gov/entrez/query.fcgi?cmd=Retrieve&db=Protein&list_uids=169217315&dopt=GenPept&RID=TAK97135016&log$=prottop&blast_rank=55) | PREDICTED: hypothetical protein [Homo sapiens] | [17.6](http://blast.ncbi.nlm.nih.gov/Blast.cgi#169217315%23169217315) | 32.7 | 75% | 695 |
| [NP_078964.2](http://www.ncbi.nlm.nih.gov/entrez/query.fcgi?cmd=Retrieve&db=Protein&list_uids=157739936&dopt=GenPept&RID=TAK97135016&log$=prottop&blast_rank=56) | chromosome 10 open reading frame 68 [Homo sapiens] | [17.6](http://blast.ncbi.nlm.nih.gov/Blast.cgi#157739936%23157739936) | 17.6 | 75% | 695 |
| [NP_061985.2](http://www.ncbi.nlm.nih.gov/entrez/query.fcgi?cmd=Retrieve&db=Protein&list_uids=150417984&dopt=GenPept&RID=TAK97135016&log$=prottop&blast_rank=57) | ATP-binding cassette sub-family A member 7 [Homo sapiens] | [17.6](http://blast.ncbi.nlm.nih.gov/Blast.cgi#150417984%23150417984) | 17.6 | 62% | 695 |
| [NP_733837.2](http://www.ncbi.nlm.nih.gov/entrez/query.fcgi?cmd=Retrieve&db=Protein&list_uids=125858485&dopt=GenPept&RID=TAK97135016&log$=prottop&blast_rank=58) | hypothetical protein LOC80209 isoform 2 [Homo sapiens] | [17.6](http://blast.ncbi.nlm.nih.gov/Blast.cgi#125858485%23125858485) | 31.8 | 62% | 695 |
| [NP_001131024.1](http://www.ncbi.nlm.nih.gov/entrez/query.fcgi?cmd=Retrieve&db=Protein&list_uids=212276096&dopt=GenPept&RID=TAK97135016&log$=prottop&blast_rank=59) | leucine rich repeat (in FLII) interacting protein 1 isoform 3 [Homo sapiens] | [17.6](http://blast.ncbi.nlm.nih.gov/Blast.cgi#212276096%23212276096) | 17.6 | 62% | 695 |
| [NP_001374.3](http://www.ncbi.nlm.nih.gov/entrez/query.fcgi?cmd=Retrieve&db=Protein&list_uids=116686106&dopt=GenPept&RID=TAK97135016&log$=prottop&blast_rank=60) | diphthamide biosynthesis protein 1 [Homo sapiens] | [17.6](http://blast.ncbi.nlm.nih.gov/Blast.cgi#116686106%23116686106) | 17.6 | 62% | 695 |
| [NP_997245.2](http://www.ncbi.nlm.nih.gov/entrez/query.fcgi?cmd=Retrieve&db=Protein&list_uids=116256464&dopt=GenPept&RID=TAK97135016&log$=prottop&blast_rank=61) | hypothetical protein LOC343990 [Homo sapiens] | [17.6](http://blast.ncbi.nlm.nih.gov/Blast.cgi#116256464%23116256464) | 32.7 | 62% | 695 |
| [XP_001127959.1](http://www.ncbi.nlm.nih.gov/entrez/query.fcgi?cmd=Retrieve&db=Protein&list_uids=113430659&dopt=GenPept&RID=TAK97135016&log$=prottop&blast_rank=62) | PREDICTED: hypothetical protein [Homo sapiens] | [17.6](http://blast.ncbi.nlm.nih.gov/Blast.cgi#113430659%23113430659) | 32.7 | 75% | 695 |
| [XP_001127139.1](http://www.ncbi.nlm.nih.gov/entrez/query.fcgi?cmd=Retrieve&db=Protein&list_uids=113430175&dopt=GenPept&RID=TAK97135016&log$=prottop&blast_rank=63) | PREDICTED: hypothetical protein [Homo sapiens] | [17.6](http://blast.ncbi.nlm.nih.gov/Blast.cgi#113430175%23113430175) | 32.7 | 75% | 695 |
| [XP_001127165.1](http://www.ncbi.nlm.nih.gov/entrez/query.fcgi?cmd=Retrieve&db=Protein&list_uids=113430177&dopt=GenPept&RID=TAK97135016&log$=prottop&blast_rank=64) | PREDICTED: hypothetical protein [Homo sapiens] | [17.6](http://blast.ncbi.nlm.nih.gov/Blast.cgi#113430177%23113430177) | 32.7 | 75% | 695 |
| [NP_079390.3](http://www.ncbi.nlm.nih.gov/entrez/query.fcgi?cmd=Retrieve&db=Protein&list_uids=109255234&dopt=GenPept&RID=TAK97135016&log$=prottop&blast_rank=65) | centrosomal protein of 290 kDa [Homo sapiens] | [17.6](http://blast.ncbi.nlm.nih.gov/Blast.cgi#109255234%23109255234) | 17.6 | 100% | 695 |
| [NP_112733.2](http://www.ncbi.nlm.nih.gov/entrez/query.fcgi?cmd=Retrieve&db=Protein&list_uids=89142735&dopt=GenPept&RID=TAK97135016&log$=prottop&blast_rank=66) | alpha 3 type IV collagen isoform 4 precursor [Homo sapiens] | [17.6](http://blast.ncbi.nlm.nih.gov/Blast.cgi#89142735%2389142735) | 66.0 | 87% | 695 |
| [NP_112730.2](http://www.ncbi.nlm.nih.gov/entrez/query.fcgi?cmd=Retrieve&db=Protein&list_uids=89142733&dopt=GenPept&RID=TAK97135016&log$=prottop&blast_rank=67) | alpha 3 type IV collagen isoform 2 precursor [Homo sapiens] | [17.6](http://blast.ncbi.nlm.nih.gov/Blast.cgi#89142733%2389142733) | 80.2 | 87% | 695 |
| [NP_112734.2](http://www.ncbi.nlm.nih.gov/entrez/query.fcgi?cmd=Retrieve&db=Protein&list_uids=89142737&dopt=GenPept&RID=TAK97135016&log$=prottop&blast_rank=68) | alpha 3 type IV collagen isoform 5 precursor [Homo sapiens] | [17.6](http://blast.ncbi.nlm.nih.gov/Blast.cgi#89142737%2389142737) | 66.0 | 87% | 695 |
| [NP_060465.1](http://www.ncbi.nlm.nih.gov/entrez/query.fcgi?cmd=Retrieve&db=Protein&list_uids=186700625&dopt=GenPept&RID=TAK97135016&log$=prottop&blast_rank=69) | putative GTP-binding protein Parf isoform 2 [Homo sapiens] | [17.6](http://blast.ncbi.nlm.nih.gov/Blast.cgi#186700625%23186700625) | 17.6 | 75% | 695 |
| [NP_005409.3](http://www.ncbi.nlm.nih.gov/entrez/query.fcgi?cmd=Retrieve&db=Protein&list_uids=47132529&dopt=GenPept&RID=TAK97135016&log$=prottop&blast_rank=70) | suppression of tumorigenicity 5 protein isoform 1 [Homo sapiens] >ref|NP_998783.1| suppression of tumorigenicity 5 protein isoform 1 [Homo sapiens] | [17.6](http://blast.ncbi.nlm.nih.gov/Blast.cgi#47132529%2347132529) | 31.8 | 75% | 695 |
| [NP_001157587.1](http://www.ncbi.nlm.nih.gov/entrez/query.fcgi?cmd=Retrieve&db=Protein&list_uids=255759932&dopt=GenPept&RID=TAK97135016&log$=prottop&blast_rank=71) | cas scaffolding protein family member 4 isoform c [Homo sapiens] | [17.6](http://blast.ncbi.nlm.nih.gov/Blast.cgi#255759932%23255759932) | 17.6 | 62% | 695 |
| [NP_055601.2](http://www.ncbi.nlm.nih.gov/entrez/query.fcgi?cmd=Retrieve&db=Protein&list_uids=21361458&dopt=GenPept&RID=TAK97135016&log$=prottop&blast_rank=72) | rho guanine nucleotide exchange factor 17 [Homo sapiens] | [17.6](http://blast.ncbi.nlm.nih.gov/Blast.cgi#21361458%2321361458) | 17.6 | 62% | 695 |
| [NP_000082.2](http://www.ncbi.nlm.nih.gov/entrez/query.fcgi?cmd=Retrieve&db=Protein&list_uids=89142730&dopt=GenPept&RID=TAK97135016&log$=prottop&blast_rank=73) | alpha 3 type IV collagen isoform 1 precursor [Homo sapiens] | [17.6](http://blast.ncbi.nlm.nih.gov/Blast.cgi#89142730%2389142730) | 80.2 | 87% | 695 |
| [NP_710142.1](http://www.ncbi.nlm.nih.gov/entrez/query.fcgi?cmd=Retrieve&db=Protein&list_uids=41281808&dopt=GenPept&RID=TAK97135016&log$=prottop&blast_rank=74) | harmonin isoform b3 [Homo sapiens] | [17.6](http://blast.ncbi.nlm.nih.gov/Blast.cgi#41281808%2341281808) | 17.6 | 75% | 695 |
| [NP_079215.2](http://www.ncbi.nlm.nih.gov/entrez/query.fcgi?cmd=Retrieve&db=Protein&list_uids=45935393&dopt=GenPept&RID=TAK97135016&log$=prottop&blast_rank=75) | RNA binding motif protein 35B [Homo sapiens] | [17.6](http://blast.ncbi.nlm.nih.gov/Blast.cgi#45935393%2345935393) | 17.6 | 62% | 695 |
| [NP_005750.4](http://www.ncbi.nlm.nih.gov/entrez/query.fcgi?cmd=Retrieve&db=Protein&list_uids=109689723&dopt=GenPept&RID=TAK97135016&log$=prottop&blast_rank=76) | abl interactor 2 [Homo sapiens] | [17.6](http://blast.ncbi.nlm.nih.gov/Blast.cgi#109689723%23109689723) | 17.6 | 62% | 695 |
| [NP_004726.2](http://www.ncbi.nlm.nih.gov/entrez/query.fcgi?cmd=Retrieve&db=Protein&list_uids=212276070&dopt=GenPept&RID=TAK97135016&log$=prottop&blast_rank=77) | leucine rich repeat (in FLII) interacting protein 1 isoform 4 [Homo sapiens] | [17.6](http://blast.ncbi.nlm.nih.gov/Blast.cgi#212276070%23212276070) | 17.6 | 62% | 695 |
| [NP_005551.3](http://www.ncbi.nlm.nih.gov/entrez/query.fcgi?cmd=Retrieve&db=Protein&list_uids=21264602&dopt=GenPept&RID=TAK97135016&log$=prottop&blast_rank=78) | laminin alpha 5 precursor [Homo sapiens] | [17.6](http://blast.ncbi.nlm.nih.gov/Blast.cgi#21264602%2321264602) | 33.5 | 100% | 695 |
| [NP_002490.2](http://www.ncbi.nlm.nih.gov/entrez/query.fcgi?cmd=Retrieve&db=Protein&list_uids=157311649&dopt=GenPept&RID=TAK97135016&log$=prottop&blast_rank=79) | neogenin isoform 1 precursor [Homo sapiens] | [17.6](http://blast.ncbi.nlm.nih.gov/Blast.cgi#157311649%23157311649) | 17.6 | 62% | 695 |
| [NP_849172.2](http://www.ncbi.nlm.nih.gov/entrez/query.fcgi?cmd=Retrieve&db=Protein&list_uids=118918429&dopt=GenPept&RID=TAK97135016&log$=prottop&blast_rank=80) | protein NLRC3 [Homo sapiens] | [17.6](http://blast.ncbi.nlm.nih.gov/Blast.cgi#118918429%23118918429) | 17.6 | 62% | 695 |
| [NP_689673.2](http://www.ncbi.nlm.nih.gov/entrez/query.fcgi?cmd=Retrieve&db=Protein&list_uids=48255962&dopt=GenPept&RID=TAK97135016&log$=prottop&blast_rank=81) | hypothetical protein LOC146723 [Homo sapiens] | [17.6](http://blast.ncbi.nlm.nih.gov/Blast.cgi#48255962%2348255962) | 17.6 | 75% | 695 |
| [NP_001131025.1](http://www.ncbi.nlm.nih.gov/entrez/query.fcgi?cmd=Retrieve&db=Protein&list_uids=212276104&dopt=GenPept&RID=TAK97135016&log$=prottop&blast_rank=82) | leucine rich repeat (in FLII) interacting protein 1 isoform 5 [Homo sapiens] | [17.6](http://blast.ncbi.nlm.nih.gov/Blast.cgi#212276104%23212276104) | 17.6 | 62% | 695 |
| [NP_631896.1](http://www.ncbi.nlm.nih.gov/entrez/query.fcgi?cmd=Retrieve&db=Protein&list_uids=21264614&dopt=GenPept&RID=TAK97135016&log$=prottop&blast_rank=83) | suppression of tumorigenicity 5 protein isoform 2 [Homo sapiens] | [17.6](http://blast.ncbi.nlm.nih.gov/Blast.cgi#21264614%2321264614) | 17.6 | 75% | 695 |
| [NP_852662.1](http://www.ncbi.nlm.nih.gov/entrez/query.fcgi?cmd=Retrieve&db=Protein&list_uids=31657096&dopt=GenPept&RID=TAK97135016&log$=prottop&blast_rank=84) | CKLF-like MARVEL transmembrane domain-containing protein 4 isoform 2 [Homo sapiens] | [17.6](http://blast.ncbi.nlm.nih.gov/Blast.cgi#31657096%2331657096) | 17.6 | 75% | 695 |
| [NP_076926.2](http://www.ncbi.nlm.nih.gov/entrez/query.fcgi?cmd=Retrieve&db=Protein&list_uids=20070328&dopt=GenPept&RID=TAK97135016&log$=prottop&blast_rank=85) | membrane-spanning 4-domains subfamily A member 4A isoform a [Homo sapiens] | [17.6](http://blast.ncbi.nlm.nih.gov/Blast.cgi#20070328%2320070328) | 17.6 | 75% | 695 |
| [NP_001004060.1](http://www.ncbi.nlm.nih.gov/entrez/query.fcgi?cmd=Retrieve&db=Protein&list_uids=51944971&dopt=GenPept&RID=TAK97135016&log$=prottop&blast_rank=86) | nodal modulator 2 isoform 1 [Homo sapiens] | [17.6](http://blast.ncbi.nlm.nih.gov/Blast.cgi#51944971%2351944971) | 29.7 | 87% | 695 |
| [NP_078939.3](http://www.ncbi.nlm.nih.gov/entrez/query.fcgi?cmd=Retrieve&db=Protein&list_uids=47155554&dopt=GenPept&RID=TAK97135016&log$=prottop&blast_rank=87) | probable aminopeptidase NPEPL1 [Homo sapiens] | [17.6](http://blast.ncbi.nlm.nih.gov/Blast.cgi#47155554%2347155554) | 17.6 | 75% | 695 |
| [NP_079414.3](http://www.ncbi.nlm.nih.gov/entrez/query.fcgi?cmd=Retrieve&db=Protein&list_uids=125656155&dopt=GenPept&RID=TAK97135016&log$=prottop&blast_rank=88) | hypothetical protein LOC80209 isoform 1 [Homo sapiens] | [17.6](http://blast.ncbi.nlm.nih.gov/Blast.cgi#125656155%23125656155) | 31.8 | 62% | 695 |
| [NP_055315.2](http://www.ncbi.nlm.nih.gov/entrez/query.fcgi?cmd=Retrieve&db=Protein&list_uids=21361437&dopt=GenPept&RID=TAK97135016&log$=prottop&blast_rank=89) | HIV Tat-specific factor 1 [Homo sapiens] >ref|NP_001156752.1| HIV Tat-specific factor 1 [Homo sapiens] | [17.6](http://blast.ncbi.nlm.nih.gov/Blast.cgi#21361437%2321361437) | 17.6 | 62% | 695 |
| [NP_038465.1](http://www.ncbi.nlm.nih.gov/entrez/query.fcgi?cmd=Retrieve&db=Protein&list_uids=7305525&dopt=GenPept&RID=TAK97135016&log$=prottop&blast_rank=90) | low density lipoprotein-related protein 12 isoform a precursor [Homo sapiens] | [17.6](http://blast.ncbi.nlm.nih.gov/Blast.cgi#7305525%237305525) | 29.7 | 87% | 695 |
| [NP_065089.2](http://www.ncbi.nlm.nih.gov/entrez/query.fcgi?cmd=Retrieve&db=Protein&list_uids=55769585&dopt=GenPept&RID=TAK97135016&log$=prottop&blast_rank=91) | cas scaffolding protein family member 4 isoform a [Homo sapiens] >ref|NP_001157588.1| cas scaffolding protein family member 4 isoform a [Homo sapiens] | [17.6](http://blast.ncbi.nlm.nih.gov/Blast.cgi#55769585%2355769585) | 17.6 | 62% | 695 |
| [NP_848933.1](http://www.ncbi.nlm.nih.gov/entrez/query.fcgi?cmd=Retrieve&db=Protein&list_uids=30520333&dopt=GenPept&RID=TAK97135016&log$=prottop&blast_rank=92) | CKLF-like MARVEL transmembrane domain-containing protein 4 isoform 1 [Homo sapiens] | [17.6](http://blast.ncbi.nlm.nih.gov/Blast.cgi#30520333%2330520333) | 17.6 | 75% | 695 |
| [NP_683876.1](http://www.ncbi.nlm.nih.gov/entrez/query.fcgi?cmd=Retrieve&db=Protein&list_uids=23110995&dopt=GenPept&RID=TAK97135016&log$=prottop&blast_rank=93) | membrane-spanning 4-domains subfamily A member 4A isoform b [Homo sapiens] | [17.6](http://blast.ncbi.nlm.nih.gov/Blast.cgi#23110995%2323110995) | 17.6 | 75% | 695 |
| [NP_714928.1](http://www.ncbi.nlm.nih.gov/entrez/query.fcgi?cmd=Retrieve&db=Protein&list_uids=24497531&dopt=GenPept&RID=TAK97135016&log$=prottop&blast_rank=94) | ellis-van Creveld syndrome protein [Homo sapiens] | [17.6](http://blast.ncbi.nlm.nih.gov/Blast.cgi#24497531%2324497531) | 30.1 | 87% | 695 |
| [NP_758454.1](http://www.ncbi.nlm.nih.gov/entrez/query.fcgi?cmd=Retrieve&db=Protein&list_uids=26892295&dopt=GenPept&RID=TAK97135016&log$=prottop&blast_rank=95) | methylmalonic aciduria type A precursor [Homo sapiens] | [17.6](http://blast.ncbi.nlm.nih.gov/Blast.cgi#26892295%2326892295) | 17.6 | 62% | 695 |
| [NP_060636.1](http://www.ncbi.nlm.nih.gov/entrez/query.fcgi?cmd=Retrieve&db=Protein&list_uids=8922567&dopt=GenPept&RID=TAK97135016&log$=prottop&blast_rank=96) | hypothetical protein LOC55194 [Homo sapiens] | [17.6](http://blast.ncbi.nlm.nih.gov/Blast.cgi#8922567%238922567) | 31.8 | 62% | 695 |
| [NP_005700.2](http://www.ncbi.nlm.nih.gov/entrez/query.fcgi?cmd=Retrieve&db=Protein&list_uids=71480164&dopt=GenPept&RID=TAK97135016&log$=prottop&blast_rank=97) | harmonin isoform a [Homo sapiens] | [17.6](http://blast.ncbi.nlm.nih.gov/Blast.cgi#71480164%2371480164) | 17.6 | 75% | 695 |
| [NP_542763.1](http://www.ncbi.nlm.nih.gov/entrez/query.fcgi?cmd=Retrieve&db=Protein&list_uids=22129778&dopt=GenPept&RID=TAK97135016&log$=prottop&blast_rank=98) | sulfiredoxin-1 [Homo sapiens] | [17.6](http://blast.ncbi.nlm.nih.gov/Blast.cgi#22129778%2322129778) | 17.6 | 62% | 695 |
| [NP_056009.1](http://www.ncbi.nlm.nih.gov/entrez/query.fcgi?cmd=Retrieve&db=Protein&list_uids=51100974&dopt=GenPept&RID=TAK97135016&log$=prottop&blast_rank=99) | myosin-Id [Homo sapiens] | [17.6](http://blast.ncbi.nlm.nih.gov/Blast.cgi#51100974%2351100974) | 17.6 | 75% | 695 |
| [NP_057622.1](http://www.ncbi.nlm.nih.gov/entrez/query.fcgi?cmd=Retrieve&db=Protein&list_uids=7706712&dopt=GenPept&RID=TAK97135016&log$=prottop&blast_rank=100) | NAD-dependent deacetylase sirtuin-7 [Homo sapiens] | [17.6](http://blast.ncbi.nlm.nih.gov/Blast.cgi#7706712%237706712) | 17.6 | 62% | 695 |

| **Accession** | **Proteins with a match to YVDTLSKL peptide** | **[Max score](http://blast.ncbi.nlm.nih.gov/Blast.cgi?CMD=Get&ALIGNMENTS=100&ALIGNMENT_VIEW=Pairwise&CDD_SEARCH_STATE=1&DATABASE_SORT=0&DESCRIPTIONS=100&ENTREZ_QUERY=txid9606 %5BORGN%5D&FIRST_QUERY_NUM=0&FORMAT_OBJECT=Alignment&FORMAT_PAGE_TARGET=&FORMAT_TYPE=HTML&GET_SEQUENCE=yes&I_THRESH=&MASK_CHAR=2&MASK_COLOR=1&NEW_VIEW=yes&NUM_OVERVIEW=100&OLD_BLAST=false&PAGE=Proteins&QUERY_INDEX=0&QUERY_NUMBER=0&RESULTS_PAGE_TARGET=&RID=TAKDV1V5014&SHOW_LINKOUT=yes&SHOW_OVERVIEW=yes&STEP_NUMBER=&WORD_SIZE=2&DISPLAY_SORT=1&HSP_SORT=1" \l "sort_mark)** | **[Total score](http://blast.ncbi.nlm.nih.gov/Blast.cgi?CMD=Get&ALIGNMENTS=100&ALIGNMENT_VIEW=Pairwise&CDD_SEARCH_STATE=1&DATABASE_SORT=0&DESCRIPTIONS=100&ENTREZ_QUERY=txid9606 %5BORGN%5D&FIRST_QUERY_NUM=0&FORMAT_OBJECT=Alignment&FORMAT_PAGE_TARGET=&FORMAT_TYPE=HTML&GET_SEQUENCE=yes&I_THRESH=&MASK_CHAR=2&MASK_COLOR=1&NEW_VIEW=yes&NUM_OVERVIEW=100&OLD_BLAST=false&PAGE=Proteins&QUERY_INDEX=0&QUERY_NUMBER=0&RESULTS_PAGE_TARGET=&RID=TAKDV1V5014&SHOW_LINKOUT=yes&SHOW_OVERVIEW=yes&STEP_NUMBER=&WORD_SIZE=2&DISPLAY_SORT=2&HSP_SORT=1" \l "sort_mark)** | **[Query coverage](http://blast.ncbi.nlm.nih.gov/Blast.cgi?CMD=Get&ALIGNMENTS=100&ALIGNMENT_VIEW=Pairwise&CDD_SEARCH_STATE=1&DATABASE_SORT=0&DESCRIPTIONS=100&ENTREZ_QUERY=txid9606 %5BORGN%5D&FIRST_QUERY_NUM=0&FORMAT_OBJECT=Alignment&FORMAT_PAGE_TARGET=&FORMAT_TYPE=HTML&GET_SEQUENCE=yes&I_THRESH=&MASK_CHAR=2&MASK_COLOR=1&NEW_VIEW=yes&NUM_OVERVIEW=100&OLD_BLAST=false&PAGE=Proteins&QUERY_INDEX=0&QUERY_NUMBER=0&RESULTS_PAGE_TARGET=&RID=TAKDV1V5014&SHOW_LINKOUT=yes&SHOW_OVERVIEW=yes&STEP_NUMBER=&WORD_SIZE=2&DISPLAY_SORT=4&HSP_SORT=0" \l "sort_mark)** | **[E value](http://blast.ncbi.nlm.nih.gov/Blast.cgi?CMD=Get&ALIGNMENTS=100&ALIGNMENT_VIEW=Pairwise&CDD_SEARCH_STATE=1&DATABASE_SORT=0&DESCRIPTIONS=100&ENTREZ_QUERY=txid9606 %5BORGN%5D&FIRST_QUERY_NUM=0&FORMAT_OBJECT=Alignment&FORMAT_PAGE_TARGET=&FORMAT_TYPE=HTML&GET_SEQUENCE=yes&I_THRESH=&MASK_CHAR=2&MASK_COLOR=1&NEW_VIEW=yes&NUM_OVERVIEW=100&OLD_BLAST=false&PAGE=Proteins&QUERY_INDEX=0&QUERY_NUMBER=0&RESULTS_PAGE_TARGET=&RID=TAKDV1V5014&SHOW_LINKOUT=yes&SHOW_OVERVIEW=yes&STEP_NUMBER=&WORD_SIZE=2&DISPLAY_SORT=0&HSP_SORT=0" \l "sort_mark)** |
| --- | --- | --- | --- | --- | --- |
| [NP_056069.2](http://www.ncbi.nlm.nih.gov/entrez/query.fcgi?cmd=Retrieve&db=Protein&list_uids=46852172&dopt=GenPept&RID=TAKDV1V5014&log$=prottop&blast_rank=1) | kinesin-like protein KIF13B [Homo sapiens] | [22.7](http://blast.ncbi.nlm.nih.gov/Blast.cgi#46852172%2346852172) | 22.7 | 100% | 20 |
| [NP_072047.4](http://www.ncbi.nlm.nih.gov/entrez/query.fcgi?cmd=Retrieve&db=Protein&list_uids=73747799&dopt=GenPept&RID=TAKDV1V5014&log$=prottop&blast_rank=2) | vacuolar protein sorting-associated protein 52 homolog [Homo sapiens] | [22.7](http://blast.ncbi.nlm.nih.gov/Blast.cgi#73747799%2373747799) | 22.7 | 87% | 20 |
| [NP_001027460.1](http://www.ncbi.nlm.nih.gov/entrez/query.fcgi?cmd=Retrieve&db=Protein&list_uids=73808086&dopt=GenPept&RID=TAKDV1V5014&log$=prottop&blast_rank=3) | solute carrier family 35, member A2 isoform b [Homo sapiens] | [21.4](http://blast.ncbi.nlm.nih.gov/Blast.cgi#73808086%2373808086) | 21.4 | 100% | 49 |
| [NP_699192.1](http://www.ncbi.nlm.nih.gov/entrez/query.fcgi?cmd=Retrieve&db=Protein&list_uids=23503303&dopt=GenPept&RID=TAKDV1V5014&log$=prottop&blast_rank=4) | serine/threonine-protein kinase NIM1 [Homo sapiens] | [21.4](http://blast.ncbi.nlm.nih.gov/Blast.cgi#23503303%2323503303) | 21.4 | 87% | 49 |
| [NP_005651.1](http://www.ncbi.nlm.nih.gov/entrez/query.fcgi?cmd=Retrieve&db=Protein&list_uids=5032211&dopt=GenPept&RID=TAKDV1V5014&log$=prottop&blast_rank=5) | solute carrier family 35, member A2 isoform a [Homo sapiens] | [21.4](http://blast.ncbi.nlm.nih.gov/Blast.cgi#5032211%235032211) | 21.4 | 100% | 49 |
| [NP_001035963.1](http://www.ncbi.nlm.nih.gov/entrez/query.fcgi?cmd=Retrieve&db=Protein&list_uids=109948265&dopt=GenPept&RID=TAKDV1V5014&log$=prottop&blast_rank=6) | solute carrier family 35, member A2 isoform c [Homo sapiens] | [21.4](http://blast.ncbi.nlm.nih.gov/Blast.cgi#109948265%23109948265) | 21.4 | 100% | 49 |
| [NP_001166184.1](http://www.ncbi.nlm.nih.gov/entrez/query.fcgi?cmd=Retrieve&db=Protein&list_uids=289577134&dopt=GenPept&RID=TAKDV1V5014&log$=prottop&blast_rank=7) | golgin subfamily A member 4 isoform 1 [Homo sapiens] | [21.0](http://blast.ncbi.nlm.nih.gov/Blast.cgi#289577134%23289577134) | 34.4 | 87% | 66 |
| [NP_002069.2](http://www.ncbi.nlm.nih.gov/entrez/query.fcgi?cmd=Retrieve&db=Protein&list_uids=6715600&dopt=GenPept&RID=TAKDV1V5014&log$=prottop&blast_rank=8) | golgin subfamily A member 4 isoform 2 [Homo sapiens] | [21.0](http://blast.ncbi.nlm.nih.gov/Blast.cgi#6715600%236715600) | 34.4 | 87% | 66 |
| [NP_001004704.1](http://www.ncbi.nlm.nih.gov/entrez/query.fcgi?cmd=Retrieve&db=Protein&list_uids=52317237&dopt=GenPept&RID=TAKDV1V5014&log$=prottop&blast_rank=9) | olfactory receptor 4C6 [Homo sapiens] | [21.0](http://blast.ncbi.nlm.nih.gov/Blast.cgi#52317237%2352317237) | 21.0 | 75% | 66 |
| [NP_008996.1](http://www.ncbi.nlm.nih.gov/entrez/query.fcgi?cmd=Retrieve&db=Protein&list_uids=5901922&dopt=GenPept&RID=TAKDV1V5014&log$=prottop&blast_rank=10) | cell division cycle 37 protein [Homo sapiens] | [21.0](http://blast.ncbi.nlm.nih.gov/Blast.cgi#5901922%235901922) | 21.0 | 75% | 66 |
| [NP_219499.1](http://www.ncbi.nlm.nih.gov/entrez/query.fcgi?cmd=Retrieve&db=Protein&list_uids=16975496&dopt=GenPept&RID=TAKDV1V5014&log$=prottop&blast_rank=11) | cortactin-binding protein 2 [Homo sapiens] | [21.0](http://blast.ncbi.nlm.nih.gov/Blast.cgi#16975496%2316975496) | 21.0 | 75% | 66 |
| [NP_904325.2](http://www.ncbi.nlm.nih.gov/entrez/query.fcgi?cmd=Retrieve&db=Protein&list_uids=41393559&dopt=GenPept&RID=TAKDV1V5014&log$=prottop&blast_rank=12) | kinesin-like protein KIF1B isoform alpha [Homo sapiens] | [20.6](http://blast.ncbi.nlm.nih.gov/Blast.cgi#41393559%2341393559) | 20.6 | 100% | 89 |
| [NP_055889.2](http://www.ncbi.nlm.nih.gov/entrez/query.fcgi?cmd=Retrieve&db=Protein&list_uids=41393563&dopt=GenPept&RID=TAKDV1V5014&log$=prottop&blast_rank=13) | kinesin-like protein KIF1B isoform b [Homo sapiens] | [20.6](http://blast.ncbi.nlm.nih.gov/Blast.cgi#41393563%2341393563) | 20.6 | 100% | 89 |
| [NP_004312.2](http://www.ncbi.nlm.nih.gov/entrez/query.fcgi?cmd=Retrieve&db=Protein&list_uids=19924175&dopt=GenPept&RID=TAKDV1V5014&log$=prottop&blast_rank=14) | kinesin-like protein KIF1A [Homo sapiens] | [20.6](http://blast.ncbi.nlm.nih.gov/Blast.cgi#19924175%2319924175) | 33.9 | 100% | 89 |
| [NP_065829.3](http://www.ncbi.nlm.nih.gov/entrez/query.fcgi?cmd=Retrieve&db=Protein&list_uids=115511036&dopt=GenPept&RID=TAKDV1V5014&log$=prottop&blast_rank=15) | alpha-protein kinase 3 [Homo sapiens] | [20.2](http://blast.ncbi.nlm.nih.gov/Blast.cgi#115511036%23115511036) | 20.2 | 87% | 119 |
| [NP_056374.2](http://www.ncbi.nlm.nih.gov/entrez/query.fcgi?cmd=Retrieve&db=Protein&list_uids=194294554&dopt=GenPept&RID=TAKDV1V5014&log$=prottop&blast_rank=16) | SET-binding protein isoform a [Homo sapiens] | [19.7](http://blast.ncbi.nlm.nih.gov/Blast.cgi#194294554%23194294554) | 19.7 | 87% | 160 |
| [NP_002963.2](http://www.ncbi.nlm.nih.gov/entrez/query.fcgi?cmd=Retrieve&db=Protein&list_uids=239735519&dopt=GenPept&RID=TAKDV1V5014&log$=prottop&blast_rank=17) | SET binding factor 1 [Homo sapiens] | [19.7](http://blast.ncbi.nlm.nih.gov/Blast.cgi#239735519%23239735519) | 19.7 | 87% | 160 |
| [NP_848622.2](http://www.ncbi.nlm.nih.gov/entrez/query.fcgi?cmd=Retrieve&db=Protein&list_uids=224465231&dopt=GenPept&RID=TAKDV1V5014&log$=prottop&blast_rank=18) | sodium/hydrogen exchanger 11 [Homo sapiens] | [19.7](http://blast.ncbi.nlm.nih.gov/Blast.cgi#224465231%23224465231) | 34.4 | 100% | 160 |
| [NP_006775.1](http://www.ncbi.nlm.nih.gov/entrez/query.fcgi?cmd=Retrieve&db=Protein&list_uids=5803221&dopt=GenPept&RID=TAKDV1V5014&log$=prottop&blast_rank=19) | WD repeat-containing protein 3 [Homo sapiens] | [19.7](http://blast.ncbi.nlm.nih.gov/Blast.cgi#5803221%235803221) | 19.7 | 62% | 160 |
| [NP_036218.1](http://www.ncbi.nlm.nih.gov/entrez/query.fcgi?cmd=Retrieve&db=Protein&list_uids=6912398&dopt=GenPept&RID=TAKDV1V5014&log$=prottop&blast_rank=20) | general transcription factor 3C polypeptide 3 [Homo sapiens] | [19.7](http://blast.ncbi.nlm.nih.gov/Blast.cgi#6912398%236912398) | 19.7 | 62% | 160 |
| [NP_055467.3](http://www.ncbi.nlm.nih.gov/entrez/query.fcgi?cmd=Retrieve&db=Protein&list_uids=224831257&dopt=GenPept&RID=TAKDV1V5014&log$=prottop&blast_rank=21) | importin-13 [Homo sapiens] | [19.7](http://blast.ncbi.nlm.nih.gov/Blast.cgi#224831257%23224831257) | 32.2 | 100% | 160 |
| [NP_056646.1](http://www.ncbi.nlm.nih.gov/entrez/query.fcgi?cmd=Retrieve&db=Protein&list_uids=7710112&dopt=GenPept&RID=TAKDV1V5014&log$=prottop&blast_rank=22) | acetylcholinesterase isoform E4-E5 precursor [Homo sapiens] | [19.7](http://blast.ncbi.nlm.nih.gov/Blast.cgi#7710112%237710112) | 19.7 | 62% | 160 |
| [NP_000656.1](http://www.ncbi.nlm.nih.gov/entrez/query.fcgi?cmd=Retrieve&db=Protein&list_uids=4557239&dopt=GenPept&RID=TAKDV1V5014&log$=prottop&blast_rank=23) | acetylcholinesterase isoform E4-E6 precursor [Homo sapiens] | [19.7](http://blast.ncbi.nlm.nih.gov/Blast.cgi#4557239%234557239) | 19.7 | 62% | 160 |
| [NP_001008496.2](http://www.ncbi.nlm.nih.gov/entrez/query.fcgi?cmd=Retrieve&db=Protein&list_uids=153792298&dopt=GenPept&RID=TAKDV1V5014&log$=prottop&blast_rank=24) | piwi-like protein 3 [Homo sapiens] | [19.3](http://blast.ncbi.nlm.nih.gov/Blast.cgi#153792298%23153792298) | 19.3 | 87% | 214 |
| [NP_001035536.1](http://www.ncbi.nlm.nih.gov/entrez/query.fcgi?cmd=Retrieve&db=Protein&list_uids=94721263&dopt=GenPept&RID=TAKDV1V5014&log$=prottop&blast_rank=25) | myotubularin-related protein 12 [Homo sapiens] | [19.3](http://blast.ncbi.nlm.nih.gov/Blast.cgi#94721263%2394721263) | 32.2 | 87% | 214 |
| [NP_059111.2](http://www.ncbi.nlm.nih.gov/entrez/query.fcgi?cmd=Retrieve&db=Protein&list_uids=166235129&dopt=GenPept&RID=TAKDV1V5014&log$=prottop&blast_rank=26) | kelch-like protein 3 [Homo sapiens] | [19.3](http://blast.ncbi.nlm.nih.gov/Blast.cgi#166235129%23166235129) | 19.3 | 87% | 214 |
| [XP_002346270.1](http://www.ncbi.nlm.nih.gov/entrez/query.fcgi?cmd=Retrieve&db=Protein&list_uids=239758017&dopt=GenPept&RID=TAKDV1V5014&log$=prottop&blast_rank=27) | PREDICTED: similar to pseudoautosomal GTP-binding protein-like protein [Homo sapiens] | [18.9](http://blast.ncbi.nlm.nih.gov/Blast.cgi#239758017%23239758017) | 18.9 | 100% | 288 |
| [NP_001002254.1](http://www.ncbi.nlm.nih.gov/entrez/query.fcgi?cmd=Retrieve&db=Protein&list_uids=51317389&dopt=GenPept&RID=TAKDV1V5014&log$=prottop&blast_rank=28) | wax synthase 2 [Homo sapiens] | [18.9](http://blast.ncbi.nlm.nih.gov/Blast.cgi#51317389%2351317389) | 18.9 | 100% | 288 |
| [NP_006603.2](http://www.ncbi.nlm.nih.gov/entrez/query.fcgi?cmd=Retrieve&db=Protein&list_uids=40254834&dopt=GenPept&RID=TAKDV1V5014&log$=prottop&blast_rank=29) | kinesin family member 1C [Homo sapiens] | [18.9](http://blast.ncbi.nlm.nih.gov/Blast.cgi#40254834%2340254834) | 18.9 | 100% | 288 |
| [NP_036359.1](http://www.ncbi.nlm.nih.gov/entrez/query.fcgi?cmd=Retrieve&db=Protein&list_uids=6912588&dopt=GenPept&RID=TAKDV1V5014&log$=prottop&blast_rank=30) | putative GTP-binding protein 6 [Homo sapiens] | [18.9](http://blast.ncbi.nlm.nih.gov/Blast.cgi#6912588%236912588) | 18.9 | 100% | 288 |
| [NP_000827.2](http://www.ncbi.nlm.nih.gov/entrez/query.fcgi?cmd=Retrieve&db=Protein&list_uids=153946391&dopt=GenPept&RID=TAKDV1V5014&log$=prottop&blast_rank=31) | glutamate [NMDA] receptor subunit epsilon-4 precursor [Homo sapiens] | [18.5](http://blast.ncbi.nlm.nih.gov/Blast.cgi#153946391%23153946391) | 18.5 | 75% | 386 |
[truncated: 443,663 more chars]
