# Supplementary material for: A high throughput method for identifying personalized tumor-associated antigens
Source: Oncotarget. 2010 Jun 27;1(2):148–55. doi: 10.18632/oncotarget.118 (PMC2920534; doi:10.18632/oncotarget.118)
Supplement: Supplemental Table 7 [file oncotarget-01-148-s007.doc]

**IgG Normal**

| **Accession** | **Proteins with a match to DIRLSAQL peptide** | **[Max score](http://blast.ncbi.nlm.nih.gov/Blast.cgi?CMD=Get&ALIGNMENTS=100&ALIGNMENT_VIEW=Pairwise&CDD_SEARCH_STATE=1&DATABASE_SORT=0&DESCRIPTIONS=100&ENTREZ_QUERY=txid9606 %5BORGN%5D&FIRST_QUERY_NUM=0&FORMAT_OBJECT=Alignment&FORMAT_PAGE_TARGET=&FORMAT_TYPE=HTML&GET_SEQUENCE=yes&I_THRESH=&MASK_CHAR=2&MASK_COLOR=1&NEW_VIEW=yes&NUM_OVERVIEW=100&OLD_BLAST=false&PAGE=Proteins&QUERY_INDEX=0&QUERY_NUMBER=0&RESULTS_PAGE_TARGET=&RID=TFM9K94S01S&SHOW_LINKOUT=yes&SHOW_OVERVIEW=yes&STEP_NUMBER=&WORD_SIZE=2&DISPLAY_SORT=1&HSP_SORT=1" \l "sort_mark)** | **[Total score](http://blast.ncbi.nlm.nih.gov/Blast.cgi?CMD=Get&ALIGNMENTS=100&ALIGNMENT_VIEW=Pairwise&CDD_SEARCH_STATE=1&DATABASE_SORT=0&DESCRIPTIONS=100&ENTREZ_QUERY=txid9606 %5BORGN%5D&FIRST_QUERY_NUM=0&FORMAT_OBJECT=Alignment&FORMAT_PAGE_TARGET=&FORMAT_TYPE=HTML&GET_SEQUENCE=yes&I_THRESH=&MASK_CHAR=2&MASK_COLOR=1&NEW_VIEW=yes&NUM_OVERVIEW=100&OLD_BLAST=false&PAGE=Proteins&QUERY_INDEX=0&QUERY_NUMBER=0&RESULTS_PAGE_TARGET=&RID=TFM9K94S01S&SHOW_LINKOUT=yes&SHOW_OVERVIEW=yes&STEP_NUMBER=&WORD_SIZE=2&DISPLAY_SORT=2&HSP_SORT=1" \l "sort_mark)** | **[Query coverage](http://blast.ncbi.nlm.nih.gov/Blast.cgi?CMD=Get&ALIGNMENTS=100&ALIGNMENT_VIEW=Pairwise&CDD_SEARCH_STATE=1&DATABASE_SORT=0&DESCRIPTIONS=100&ENTREZ_QUERY=txid9606 %5BORGN%5D&FIRST_QUERY_NUM=0&FORMAT_OBJECT=Alignment&FORMAT_PAGE_TARGET=&FORMAT_TYPE=HTML&GET_SEQUENCE=yes&I_THRESH=&MASK_CHAR=2&MASK_COLOR=1&NEW_VIEW=yes&NUM_OVERVIEW=100&OLD_BLAST=false&PAGE=Proteins&QUERY_INDEX=0&QUERY_NUMBER=0&RESULTS_PAGE_TARGET=&RID=TFM9K94S01S&SHOW_LINKOUT=yes&SHOW_OVERVIEW=yes&STEP_NUMBER=&WORD_SIZE=2&DISPLAY_SORT=4&HSP_SORT=0" \l "sort_mark)** | **[E value](http://blast.ncbi.nlm.nih.gov/Blast.cgi?CMD=Get&ALIGNMENTS=100&ALIGNMENT_VIEW=Pairwise&CDD_SEARCH_STATE=1&DATABASE_SORT=0&DESCRIPTIONS=100&ENTREZ_QUERY=txid9606 %5BORGN%5D&FIRST_QUERY_NUM=0&FORMAT_OBJECT=Alignment&FORMAT_PAGE_TARGET=&FORMAT_TYPE=HTML&GET_SEQUENCE=yes&I_THRESH=&MASK_CHAR=2&MASK_COLOR=1&NEW_VIEW=yes&NUM_OVERVIEW=100&OLD_BLAST=false&PAGE=Proteins&QUERY_INDEX=0&QUERY_NUMBER=0&RESULTS_PAGE_TARGET=&RID=TFM9K94S01S&SHOW_LINKOUT=yes&SHOW_OVERVIEW=yes&STEP_NUMBER=&WORD_SIZE=2&DISPLAY_SORT=0&HSP_SORT=0" \l "sort_mark)** |
| --- | --- | --- | --- | --- | --- |
| [NP_060327.3](http://www.ncbi.nlm.nih.gov/entrez/query.fcgi?cmd=Retrieve&db=Protein&list_uids=239582767&dopt=GenPept&RID=TFM9K94S01S&log$=prottop&blast_rank=1) | protein phosphatase Slingshot homolog 3 [Homo sapiens] | [25.2](http://blast.ncbi.nlm.nih.gov/Blast.cgi#239582767%23239582767) | 42.4 | 100% | 3.5 |
| [NP_073752.5](http://www.ncbi.nlm.nih.gov/entrez/query.fcgi?cmd=Retrieve&db=Protein&list_uids=148613886&dopt=GenPept&RID=TFM9K94S01S&log$=prottop&blast_rank=2) | DNA-binding protein RFX7 [Homo sapiens] | [22.3](http://blast.ncbi.nlm.nih.gov/Blast.cgi#148613886%23148613886) | 22.3 | 100% | 27 |
| [NP_001138243.1](http://www.ncbi.nlm.nih.gov/entrez/query.fcgi?cmd=Retrieve&db=Protein&list_uids=221316624&dopt=GenPept&RID=TFM9K94S01S&log$=prottop&blast_rank=3) | bullous pemphigoid antigen 1, isoform 7 isoform 4 [Homo sapiens] | [21.8](http://blast.ncbi.nlm.nih.gov/Blast.cgi#221316624%23221316624) | 33.9 | 100% | 37 |
| [NP_001138242.1](http://www.ncbi.nlm.nih.gov/entrez/query.fcgi?cmd=Retrieve&db=Protein&list_uids=221316622&dopt=GenPept&RID=TFM9K94S01S&log$=prottop&blast_rank=4) | bullous pemphigoid antigen 1, isoform 7 isoform 3 [Homo sapiens] | [21.8](http://blast.ncbi.nlm.nih.gov/Blast.cgi#221316622%23221316622) | 33.9 | 100% | 37 |
| [NP_001138241.1](http://www.ncbi.nlm.nih.gov/entrez/query.fcgi?cmd=Retrieve&db=Protein&list_uids=221316618&dopt=GenPept&RID=TFM9K94S01S&log$=prottop&blast_rank=5) | bullous pemphigoid antigen 1, isoform 7 isoform 2 [Homo sapiens] | [21.8](http://blast.ncbi.nlm.nih.gov/Blast.cgi#221316618%23221316618) | 33.9 | 100% | 37 |
| [NP_056363.2](http://www.ncbi.nlm.nih.gov/entrez/query.fcgi?cmd=Retrieve&db=Protein&list_uids=34577049&dopt=GenPept&RID=TFM9K94S01S&log$=prottop&blast_rank=6) | dystonin isoform 1eA precursor [Homo sapiens] | [21.8](http://blast.ncbi.nlm.nih.gov/Blast.cgi#34577049%2334577049) | 33.9 | 100% | 37 |
| [NP_899236.1](http://www.ncbi.nlm.nih.gov/entrez/query.fcgi?cmd=Retrieve&db=Protein&list_uids=34577047&dopt=GenPept&RID=TFM9K94S01S&log$=prottop&blast_rank=7) | bullous pemphigoid antigen 1, isoform 7 isoform 1 [Homo sapiens] | [21.8](http://blast.ncbi.nlm.nih.gov/Blast.cgi#34577047%2334577047) | 33.9 | 100% | 37 |
| [NP_004264.2](http://www.ncbi.nlm.nih.gov/entrez/query.fcgi?cmd=Retrieve&db=Protein&list_uids=19923281&dopt=GenPept&RID=TFM9K94S01S&log$=prottop&blast_rank=8) | carbohydrate sulfotransferase 3 [Homo sapiens] | [21.4](http://blast.ncbi.nlm.nih.gov/Blast.cgi#19923281%2319923281) | 33.9 | 87% | 49 |
| [NP_996829.3](http://www.ncbi.nlm.nih.gov/entrez/query.fcgi?cmd=Retrieve&db=Protein&list_uids=154091308&dopt=GenPept&RID=TFM9K94S01S&log$=prottop&blast_rank=9) | transient receptor potential cation channel subfamily M member 3 isoform f [Homo sapiens] | [21.0](http://blast.ncbi.nlm.nih.gov/Blast.cgi#154091308%23154091308) | 31.0 | 100% | 66 |
| [NP_001007472.2](http://www.ncbi.nlm.nih.gov/entrez/query.fcgi?cmd=Retrieve&db=Protein&list_uids=154091314&dopt=GenPept&RID=TFM9K94S01S&log$=prottop&blast_rank=10) | transient receptor potential cation channel subfamily M member 3 isoform k [Homo sapiens] | [21.0](http://blast.ncbi.nlm.nih.gov/Blast.cgi#154091314%23154091314) | 31.0 | 100% | 66 |
| [NP_996830.3](http://www.ncbi.nlm.nih.gov/entrez/query.fcgi?cmd=Retrieve&db=Protein&list_uids=154091310&dopt=GenPept&RID=TFM9K94S01S&log$=prottop&blast_rank=11) | transient receptor potential cation channel subfamily M member 3 isoform g [Homo sapiens] | [21.0](http://blast.ncbi.nlm.nih.gov/Blast.cgi#154091310%23154091310) | 31.0 | 100% | 66 |
| [NP_079247.5](http://www.ncbi.nlm.nih.gov/entrez/query.fcgi?cmd=Retrieve&db=Protein&list_uids=154091312&dopt=GenPept&RID=TFM9K94S01S&log$=prottop&blast_rank=12) | transient receptor potential cation channel subfamily M member 3 isoform b [Homo sapiens] | [21.0](http://blast.ncbi.nlm.nih.gov/Blast.cgi#154091312%23154091312) | 31.0 | 100% | 66 |
| [NP_066003.3](http://www.ncbi.nlm.nih.gov/entrez/query.fcgi?cmd=Retrieve&db=Protein&list_uids=154091320&dopt=GenPept&RID=TFM9K94S01S&log$=prottop&blast_rank=13) | transient receptor potential cation channel subfamily M member 3 isoform a [Homo sapiens] | [21.0](http://blast.ncbi.nlm.nih.gov/Blast.cgi#154091320%23154091320) | 31.0 | 100% | 66 |
| [NP_996827.3](http://www.ncbi.nlm.nih.gov/entrez/query.fcgi?cmd=Retrieve&db=Protein&list_uids=154091318&dopt=GenPept&RID=TFM9K94S01S&log$=prottop&blast_rank=14) | transient receptor potential cation channel subfamily M member 3 isoform d [Homo sapiens] | [21.0](http://blast.ncbi.nlm.nih.gov/Blast.cgi#154091318%23154091318) | 31.0 | 100% | 66 |
| [NP_996828.3](http://www.ncbi.nlm.nih.gov/entrez/query.fcgi?cmd=Retrieve&db=Protein&list_uids=154091316&dopt=GenPept&RID=TFM9K94S01S&log$=prottop&blast_rank=15) | transient receptor potential cation channel subfamily M member 3 isoform e [Homo sapiens] | [21.0](http://blast.ncbi.nlm.nih.gov/Blast.cgi#154091316%23154091316) | 31.0 | 100% | 66 |
| [NP_060295.1](http://www.ncbi.nlm.nih.gov/entrez/query.fcgi?cmd=Retrieve&db=Protein&list_uids=8923417&dopt=GenPept&RID=TFM9K94S01S&log$=prottop&blast_rank=16) | ADP-ribosylhydrolase like 2 [Homo sapiens] | [21.0](http://blast.ncbi.nlm.nih.gov/Blast.cgi#8923417%238923417) | 21.0 | 75% | 66 |
| [NP_003015.2](http://www.ncbi.nlm.nih.gov/entrez/query.fcgi?cmd=Retrieve&db=Protein&list_uids=47717123&dopt=GenPept&RID=TFM9K94S01S&log$=prottop&blast_rank=17) | intersectin-1 isoform ITSN-l [Homo sapiens] | [20.2](http://blast.ncbi.nlm.nih.gov/Blast.cgi#47717123%2347717123) | 46.9 | 100% | 119 |
| [NP_079230.1](http://www.ncbi.nlm.nih.gov/entrez/query.fcgi?cmd=Retrieve&db=Protein&list_uids=13376439&dopt=GenPept&RID=TFM9K94S01S&log$=prottop&blast_rank=18) | ubiquitin domain-containing protein 1 [Homo sapiens] | [19.7](http://blast.ncbi.nlm.nih.gov/Blast.cgi#13376439%2313376439) | 19.7 | 100% | 160 |
| [NP_002642.1](http://www.ncbi.nlm.nih.gov/entrez/query.fcgi?cmd=Retrieve&db=Protein&list_uids=4505809&dopt=GenPept&RID=TFM9K94S01S&log$=prottop&blast_rank=19) | phosphatidylinositol 4-kinase beta [Homo sapiens] | [19.7](http://blast.ncbi.nlm.nih.gov/Blast.cgi#4505809%234505809) | 34.4 | 100% | 160 |
| [NP_005693.1](http://www.ncbi.nlm.nih.gov/entrez/query.fcgi?cmd=Retrieve&db=Protein&list_uids=24307899&dopt=GenPept&RID=TFM9K94S01S&log$=prottop&blast_rank=20) | GTP-binding protein era homolog [Homo sapiens] | [19.7](http://blast.ncbi.nlm.nih.gov/Blast.cgi#24307899%2324307899) | 47.3 | 100% | 160 |
| [XP_001716973.1](http://www.ncbi.nlm.nih.gov/entrez/query.fcgi?cmd=Retrieve&db=Protein&list_uids=169171558&dopt=GenPept&RID=TFM9K94S01S&log$=prottop&blast_rank=21) | PREDICTED: hypothetical protein [Homo sapiens] | [19.3](http://blast.ncbi.nlm.nih.gov/Blast.cgi#169171558%23169171558) | 19.3 | 100% | 214 |
| [XP_001714480.1](http://www.ncbi.nlm.nih.gov/entrez/query.fcgi?cmd=Retrieve&db=Protein&list_uids=169171016&dopt=GenPept&RID=TFM9K94S01S&log$=prottop&blast_rank=22) | PREDICTED: hypothetical protein [Homo sapiens] >ref|XP_001716389.1| PREDICTED: hypothetical protein [Homo sapiens] >ref|XP_002344468.1| PREDICTED: hypothetical protein [Homo sapiens] | [19.3](http://blast.ncbi.nlm.nih.gov/Blast.cgi#169171016%23169171016) | 19.3 | 100% | 214 |
| [NP_612429.2](http://www.ncbi.nlm.nih.gov/entrez/query.fcgi?cmd=Retrieve&db=Protein&list_uids=156766050&dopt=GenPept&RID=TFM9K94S01S&log$=prottop&blast_rank=23) | AHNAK nucleoprotein 2 [Homo sapiens] | [19.3](http://blast.ncbi.nlm.nih.gov/Blast.cgi#156766050%23156766050) | 90.8 | 100% | 214 |
| [NP_001138526.1](http://www.ncbi.nlm.nih.gov/entrez/query.fcgi?cmd=Retrieve&db=Protein&list_uids=222831614&dopt=GenPept&RID=TFM9K94S01S&log$=prottop&blast_rank=24) | hypothetical protein LOC388963 [Homo sapiens] | [19.3](http://blast.ncbi.nlm.nih.gov/Blast.cgi#222831614%23222831614) | 19.3 | 87% | 214 |
| [NP_733822.1](http://www.ncbi.nlm.nih.gov/entrez/query.fcgi?cmd=Retrieve&db=Protein&list_uids=27436948&dopt=GenPept&RID=TFM9K94S01S&log$=prottop&blast_rank=25) | lamin-A/C isoform 3 [Homo sapiens] | [19.3](http://blast.ncbi.nlm.nih.gov/Blast.cgi#27436948%2327436948) | 19.3 | 87% | 214 |
| [NP_056193.2](http://www.ncbi.nlm.nih.gov/entrez/query.fcgi?cmd=Retrieve&db=Protein&list_uids=54607139&dopt=GenPept&RID=TFM9K94S01S&log$=prottop&blast_rank=26) | vacuolar protein sorting-associated protein 13D isoform 1 [Homo sapiens] | [19.3](http://blast.ncbi.nlm.nih.gov/Blast.cgi#54607139%2354607139) | 19.3 | 100% | 214 |
| [NP_060626.2](http://www.ncbi.nlm.nih.gov/entrez/query.fcgi?cmd=Retrieve&db=Protein&list_uids=54607141&dopt=GenPept&RID=TFM9K94S01S&log$=prottop&blast_rank=27) | vacuolar protein sorting-associated protein 13D isoform 2 [Homo sapiens] | [19.3](http://blast.ncbi.nlm.nih.gov/Blast.cgi#54607141%2354607141) | 19.3 | 100% | 214 |
| [NP_005563.1](http://www.ncbi.nlm.nih.gov/entrez/query.fcgi?cmd=Retrieve&db=Protein&list_uids=5031875&dopt=GenPept&RID=TFM9K94S01S&log$=prottop&blast_rank=28) | lamin-A/C isoform 2 [Homo sapiens] | [19.3](http://blast.ncbi.nlm.nih.gov/Blast.cgi#5031875%235031875) | 19.3 | 87% | 214 |
| [NP_733821.1](http://www.ncbi.nlm.nih.gov/entrez/query.fcgi?cmd=Retrieve&db=Protein&list_uids=27436946&dopt=GenPept&RID=TFM9K94S01S&log$=prottop&blast_rank=29) | lamin A/C isoform 1 precursor [Homo sapiens] | [19.3](http://blast.ncbi.nlm.nih.gov/Blast.cgi#27436946%2327436946) | 19.3 | 87% | 214 |
| [NP_065945.2](http://www.ncbi.nlm.nih.gov/entrez/query.fcgi?cmd=Retrieve&db=Protein&list_uids=190358543&dopt=GenPept&RID=TFM9K94S01S&log$=prottop&blast_rank=30) | hypothetical protein LOC57654 [Homo sapiens] | [18.9](http://blast.ncbi.nlm.nih.gov/Blast.cgi#190358543%23190358543) | 18.9 | 75% | 288 |
| [XP_002347374.1](http://www.ncbi.nlm.nih.gov/entrez/query.fcgi?cmd=Retrieve&db=Protein&list_uids=239750339&dopt=GenPept&RID=TFM9K94S01S&log$=prottop&blast_rank=31) | PREDICTED: hypothetical protein XP_002347374 [Homo sapiens] | [18.9](http://blast.ncbi.nlm.nih.gov/Blast.cgi#239750339%23239750339) | 18.9 | 62% | 288 |
| [NP_055740.3](http://www.ncbi.nlm.nih.gov/entrez/query.fcgi?cmd=Retrieve&db=Protein&list_uids=162951884&dopt=GenPept&RID=TFM9K94S01S&log$=prottop&blast_rank=32) | R3H domain-containing protein 2 [Homo sapiens] | [18.9](http://blast.ncbi.nlm.nih.gov/Blast.cgi#162951884%23162951884) | 18.9 | 62% | 288 |
| [XP_002346633.1](http://www.ncbi.nlm.nih.gov/entrez/query.fcgi?cmd=Retrieve&db=Protein&list_uids=239748340&dopt=GenPept&RID=TFM9K94S01S&log$=prottop&blast_rank=33) | PREDICTED: hypothetical protein XP_002346633 [Homo sapiens] | [18.9](http://blast.ncbi.nlm.nih.gov/Blast.cgi#239748340%23239748340) | 18.9 | 75% | 288 |
| [NP_001009899.2](http://www.ncbi.nlm.nih.gov/entrez/query.fcgi?cmd=Retrieve&db=Protein&list_uids=114431248&dopt=GenPept&RID=TFM9K94S01S&log$=prottop&blast_rank=34) | hypothetical protein LOC205717 [Homo sapiens] | [18.9](http://blast.ncbi.nlm.nih.gov/Blast.cgi#114431248%23114431248) | 18.9 | 62% | 288 |
| [NP_114119.2](http://www.ncbi.nlm.nih.gov/entrez/query.fcgi?cmd=Retrieve&db=Protein&list_uids=103472031&dopt=GenPept&RID=TFM9K94S01S&log$=prottop&blast_rank=35) | family with sequence similarity 62 (C2 domain containing), member C [Homo sapiens] | [18.9](http://blast.ncbi.nlm.nih.gov/Blast.cgi#103472031%23103472031) | 18.9 | 100% | 288 |
| [NP_003226.4](http://www.ncbi.nlm.nih.gov/entrez/query.fcgi?cmd=Retrieve&db=Protein&list_uids=55770862&dopt=GenPept&RID=TFM9K94S01S&log$=prottop&blast_rank=36) | thyroglobulin precursor [Homo sapiens] | [18.9](http://blast.ncbi.nlm.nih.gov/Blast.cgi#55770862%2355770862) | 18.9 | 75% | 288 |
| [NP_079440.2](http://www.ncbi.nlm.nih.gov/entrez/query.fcgi?cmd=Retrieve&db=Protein&list_uids=38569491&dopt=GenPept&RID=TFM9K94S01S&log$=prottop&blast_rank=37) | serine/threonine-protein kinase SIK3 [Homo sapiens] | [18.9](http://blast.ncbi.nlm.nih.gov/Blast.cgi#38569491%2338569491) | 43.1 | 100% | 288 |
| [NP_000419.1](http://www.ncbi.nlm.nih.gov/entrez/query.fcgi?cmd=Retrieve&db=Protein&list_uids=4557733&dopt=GenPept&RID=TFM9K94S01S&log$=prottop&blast_rank=38) | latent transforming growth factor beta binding protein 2 precursor [Homo sapiens] | [18.9](http://blast.ncbi.nlm.nih.gov/Blast.cgi#4557733%234557733) | 32.2 | 100% | 288 |
| [NP_065385.2](http://www.ncbi.nlm.nih.gov/entrez/query.fcgi?cmd=Retrieve&db=Protein&list_uids=19923830&dopt=GenPept&RID=TFM9K94S01S&log$=prottop&blast_rank=39) | pre-B-cell leukemia transcription factor-interacting protein 1 [Homo sapiens] | [18.9](http://blast.ncbi.nlm.nih.gov/Blast.cgi#19923830%2319923830) | 18.9 | 100% | 288 |
| [NP_001447.2](http://www.ncbi.nlm.nih.gov/entrez/query.fcgi?cmd=Retrieve&db=Protein&list_uids=116063573&dopt=GenPept&RID=TFM9K94S01S&log$=prottop&blast_rank=40) | filamin-A isoform 1 [Homo sapiens] | [18.9](http://blast.ncbi.nlm.nih.gov/Blast.cgi#116063573%23116063573) | 18.9 | 62% | 288 |
| [NP_620060.1](http://www.ncbi.nlm.nih.gov/entrez/query.fcgi?cmd=Retrieve&db=Protein&list_uids=21166380&dopt=GenPept&RID=TFM9K94S01S&log$=prottop&blast_rank=41) | neurexin 2 isoform alpha-2 precursor [Homo sapiens] | [18.9](http://blast.ncbi.nlm.nih.gov/Blast.cgi#21166380%2321166380) | 18.9 | 75% | 288 |
| [NP_150090.1](http://www.ncbi.nlm.nih.gov/entrez/query.fcgi?cmd=Retrieve&db=Protein&list_uids=15208669&dopt=GenPept&RID=TFM9K94S01S&log$=prottop&blast_rank=42) | tripartite motif-containing protein 14 isoform beta [Homo sapiens] | [18.9](http://blast.ncbi.nlm.nih.gov/Blast.cgi#15208669%2315208669) | 18.9 | 62% | 288 |
| [NP_001104026.1](http://www.ncbi.nlm.nih.gov/entrez/query.fcgi?cmd=Retrieve&db=Protein&list_uids=160420317&dopt=GenPept&RID=TFM9K94S01S&log$=prottop&blast_rank=43) | filamin-A isoform 2 [Homo sapiens] | [18.9](http://blast.ncbi.nlm.nih.gov/Blast.cgi#160420317%23160420317) | 18.9 | 62% | 288 |
| [NP_002420.1](http://www.ncbi.nlm.nih.gov/entrez/query.fcgi?cmd=Retrieve&db=Protein&list_uids=4505213&dopt=GenPept&RID=TFM9K94S01S&log$=prottop&blast_rank=44) | matrix metalloproteinase 19 isoform rasi-1 preproprotein [Homo sapiens] | [18.9](http://blast.ncbi.nlm.nih.gov/Blast.cgi#4505213%234505213) | 33.1 | 100% | 288 |
| [NP_055895.1](http://www.ncbi.nlm.nih.gov/entrez/query.fcgi?cmd=Retrieve&db=Protein&list_uids=14211536&dopt=GenPept&RID=TFM9K94S01S&log$=prottop&blast_rank=45) | neurexin 2 isoform alpha-1 precursor [Homo sapiens] | [18.9](http://blast.ncbi.nlm.nih.gov/Blast.cgi#14211536%2314211536) | 18.9 | 75% | 288 |
| [NP_620077.1](http://www.ncbi.nlm.nih.gov/entrez/query.fcgi?cmd=Retrieve&db=Protein&list_uids=41281688&dopt=GenPept&RID=TFM9K94S01S&log$=prottop&blast_rank=46) | hypothetical protein LOC25912 isoform 2 [Homo sapiens] | [18.9](http://blast.ncbi.nlm.nih.gov/Blast.cgi#41281688%2341281688) | 18.9 | 62% | 288 |
| [NP_001036.1](http://www.ncbi.nlm.nih.gov/entrez/query.fcgi?cmd=Retrieve&db=Protein&list_uids=4507043&dopt=GenPept&RID=TFM9K94S01S&log$=prottop&blast_rank=47) | solute carrier family 6 member 4 [Homo sapiens] | [18.9](http://blast.ncbi.nlm.nih.gov/Blast.cgi#4507043%234507043) | 18.9 | 75% | 288 |
| [NP_002401.1](http://www.ncbi.nlm.nih.gov/entrez/query.fcgi?cmd=Retrieve&db=Protein&list_uids=4505167&dopt=GenPept&RID=TFM9K94S01S&log$=prottop&blast_rank=48) | alpha-1,6-mannosylglycoprotein 6-beta-N-acetylglucosaminyltransferase A [Homo sapiens] | [18.9](http://blast.ncbi.nlm.nih.gov/Blast.cgi#4505167%234505167) | 28.8 | 100% | 288 |
| [NP_056264.1](http://www.ncbi.nlm.nih.gov/entrez/query.fcgi?cmd=Retrieve&db=Protein&list_uids=14149688&dopt=GenPept&RID=TFM9K94S01S&log$=prottop&blast_rank=49) | hypothetical protein LOC25912 isoform 1 [Homo sapiens] | [18.9](http://blast.ncbi.nlm.nih.gov/Blast.cgi#14149688%2314149688) | 18.9 | 62% | 288 |
| [NP_066997.3](http://www.ncbi.nlm.nih.gov/entrez/query.fcgi?cmd=Retrieve&db=Protein&list_uids=24432106&dopt=GenPept&RID=TFM9K94S01S&log$=prottop&blast_rank=50) | p30 DBC protein [Homo sapiens] >ref|NP_954675.1| p30 DBC protein [Homo sapiens] | [18.9](http://blast.ncbi.nlm.nih.gov/Blast.cgi#24432106%2324432106) | 18.9 | 87% | 288 |
| [NP_001092086.1](http://www.ncbi.nlm.nih.gov/entrez/query.fcgi?cmd=Retrieve&db=Protein&list_uids=148833486&dopt=GenPept&RID=TFM9K94S01S&log$=prottop&blast_rank=51) | hypothetical protein LOC25912 isoform 3 [Homo sapiens] | [18.9](http://blast.ncbi.nlm.nih.gov/Blast.cgi#148833486%23148833486) | 18.9 | 62% | 288 |
| [XP_002343852.1](http://www.ncbi.nlm.nih.gov/entrez/query.fcgi?cmd=Retrieve&db=Protein&list_uids=239746824&dopt=GenPept&RID=TFM9K94S01S&log$=prottop&blast_rank=52) | PREDICTED: hypothetical protein XP_002343852 [Homo sapiens] >ref|XP_002348196.1| PREDICTED: hypothetical protein XP_002348196 [Homo sapiens] >ref|XP_002346344.1| PREDICTED: hypothetical protein [Homo sapiens] | [18.5](http://blast.ncbi.nlm.nih.gov/Blast.cgi#239746824%23239746824) | 18.5 | 75% | 386 |
| [XP_002342243.1](http://www.ncbi.nlm.nih.gov/entrez/query.fcgi?cmd=Retrieve&db=Protein&list_uids=239741585&dopt=GenPept&RID=TFM9K94S01S&log$=prottop&blast_rank=53) | PREDICTED: hypothetical protein XP_002342243 [Homo sapiens] | [18.5](http://blast.ncbi.nlm.nih.gov/Blast.cgi#239741585%23239741585) | 18.5 | 75% | 386 |
| [NP_001138663.1](http://www.ncbi.nlm.nih.gov/entrez/query.fcgi?cmd=Retrieve&db=Protein&list_uids=223633919&dopt=GenPept&RID=TFM9K94S01S&log$=prottop&blast_rank=54) | hypothetical protein LOC285550 [Homo sapiens] | [18.5](http://blast.ncbi.nlm.nih.gov/Blast.cgi#223633919%23223633919) | 18.5 | 87% | 386 |
| [NP_001005290.1](http://www.ncbi.nlm.nih.gov/entrez/query.fcgi?cmd=Retrieve&db=Protein&list_uids=52630338&dopt=GenPept&RID=TFM9K94S01S&log$=prottop&blast_rank=55) | proline/serine-rich coiled-coil protein 1 isoform b [Homo sapiens] | [18.5](http://blast.ncbi.nlm.nih.gov/Blast.cgi#52630338%2352630338) | 18.5 | 75% | 386 |
| [NP_055862.1](http://www.ncbi.nlm.nih.gov/entrez/query.fcgi?cmd=Retrieve&db=Protein&list_uids=22095331&dopt=GenPept&RID=TFM9K94S01S&log$=prottop&blast_rank=56) | hypothetical protein LOC23065 precursor [Homo sapiens] | [18.5](http://blast.ncbi.nlm.nih.gov/Blast.cgi#22095331%2322095331) | 18.5 | 75% | 386 |
| [NP_055705.2](http://www.ncbi.nlm.nih.gov/entrez/query.fcgi?cmd=Retrieve&db=Protein&list_uids=109659849&dopt=GenPept&RID=TFM9K94S01S&log$=prottop&blast_rank=57) | filamin A-interacting protein 1-like isoform 2 [Homo sapiens] | [18.5](http://blast.ncbi.nlm.nih.gov/Blast.cgi#109659849%23109659849) | 18.5 | 75% | 386 |
| [NP_002363.2](http://www.ncbi.nlm.nih.gov/entrez/query.fcgi?cmd=Retrieve&db=Protein&list_uids=51477714&dopt=GenPept&RID=TFM9K94S01S&log$=prottop&blast_rank=58) | alpha-mannosidase 2 [Homo sapiens] | [18.5](http://blast.ncbi.nlm.nih.gov/Blast.cgi#51477714%2351477714) | 33.1 | 75% | 386 |
| [NP_878913.2](http://www.ncbi.nlm.nih.gov/entrez/query.fcgi?cmd=Retrieve&db=Protein&list_uids=109659845&dopt=GenPept&RID=TFM9K94S01S&log$=prottop&blast_rank=59) | filamin A-interacting protein 1-like isoform 1 [Homo sapiens] | [18.5](http://blast.ncbi.nlm.nih.gov/Blast.cgi#109659845%23109659845) | 18.5 | 75% | 386 |
| [NP_001035924.1](http://www.ncbi.nlm.nih.gov/entrez/query.fcgi?cmd=Retrieve&db=Protein&list_uids=109659847&dopt=GenPept&RID=TFM9K94S01S&log$=prottop&blast_rank=60) | filamin A-interacting protein 1-like isoform 3 [Homo sapiens] | [18.5](http://blast.ncbi.nlm.nih.gov/Blast.cgi#109659847%23109659847) | 18.5 | 75% | 386 |
| [NP_703145.1](http://www.ncbi.nlm.nih.gov/entrez/query.fcgi?cmd=Retrieve&db=Protein&list_uids=23592222&dopt=GenPept&RID=TFM9K94S01S&log$=prottop&blast_rank=61) | olfactory receptor 5P2 [Homo sapiens] | [18.5](http://blast.ncbi.nlm.nih.gov/Blast.cgi#23592222%2323592222) | 18.5 | 87% | 386 |
| [NP_000862.1](http://www.ncbi.nlm.nih.gov/entrez/query.fcgi?cmd=Retrieve&db=Protein&list_uids=4504545&dopt=GenPept&RID=TFM9K94S01S&log$=prottop&blast_rank=62) | 5-hydroxytryptamine receptor 6 [Homo sapiens] | [18.5](http://blast.ncbi.nlm.nih.gov/Blast.cgi#4504545%234504545) | 18.5 | 75% | 386 |
| [NP_006168.1](http://www.ncbi.nlm.nih.gov/entrez/query.fcgi?cmd=Retrieve&db=Protein&list_uids=5453802&dopt=GenPept&RID=TFM9K94S01S&log$=prottop&blast_rank=63) | neural retina-specific leucine zipper protein [Homo sapiens] | [18.5](http://blast.ncbi.nlm.nih.gov/Blast.cgi#5453802%235453802) | 18.5 | 75% | 386 |
| [NP_060559.2](http://www.ncbi.nlm.nih.gov/entrez/query.fcgi?cmd=Retrieve&db=Protein&list_uids=109150425&dopt=GenPept&RID=TFM9K94S01S&log$=prottop&blast_rank=64) | ankyrin repeat and zinc finger domain-containing protein 1 [Homo sapiens] >ref|NP_001035869.1| ankyrin repeat and zinc finger domain-containing protein 1 [Homo sapiens] | [18.5](http://blast.ncbi.nlm.nih.gov/Blast.cgi#109150425%23109150425) | 18.5 | 75% | 386 |
| [NP_116025.1](http://www.ncbi.nlm.nih.gov/entrez/query.fcgi?cmd=Retrieve&db=Protein&list_uids=14249168&dopt=GenPept&RID=TFM9K94S01S&log$=prottop&blast_rank=65) | proline/serine-rich coiled-coil protein 1 isoform a [Homo sapiens] >ref|NP_001027462.1| proline/serine-rich coiled-coil protein 1 isoform a [Homo sapiens] | [18.5](http://blast.ncbi.nlm.nih.gov/Blast.cgi#14249168%2314249168) | 18.5 | 75% | 386 |
| [NP_848578.1](http://www.ncbi.nlm.nih.gov/entrez/query.fcgi?cmd=Retrieve&db=Protein&list_uids=30425406&dopt=GenPept&RID=TFM9K94S01S&log$=prottop&blast_rank=66) | hypothetical protein LOC140856 [Homo sapiens] | [18.5](http://blast.ncbi.nlm.nih.gov/Blast.cgi#30425406%2330425406) | 18.5 | 100% | 386 |
| [NP_219499.1](http://www.ncbi.nlm.nih.gov/entrez/query.fcgi?cmd=Retrieve&db=Protein&list_uids=16975496&dopt=GenPept&RID=TFM9K94S01S&log$=prottop&blast_rank=67) | cortactin-binding protein 2 [Homo sapiens] | [18.5](http://blast.ncbi.nlm.nih.gov/Blast.cgi#16975496%2316975496) | 18.5 | 75% | 386 |
| [NP_001158508.1](http://www.ncbi.nlm.nih.gov/entrez/query.fcgi?cmd=Retrieve&db=Protein&list_uids=259013553&dopt=GenPept&RID=TFM9K94S01S&log$=prottop&blast_rank=68) | oxoglutarate dehydrogenase isoform 3 precursor [Homo sapiens] | [18.0](http://blast.ncbi.nlm.nih.gov/Blast.cgi#259013553%23259013553) | 18.0 | 75% | 518 |
| [NP_001155180.1](http://www.ncbi.nlm.nih.gov/entrez/query.fcgi?cmd=Retrieve&db=Protein&list_uids=239787821&dopt=GenPept&RID=TFM9K94S01S&log$=prottop&blast_rank=69) | syncoilin, intermediate filament 1 isoform 2 [Homo sapiens] | [18.0](http://blast.ncbi.nlm.nih.gov/Blast.cgi#239787821%23239787821) | 18.0 | 62% | 518 |
| [XP_002346979.1](http://www.ncbi.nlm.nih.gov/entrez/query.fcgi?cmd=Retrieve&db=Protein&list_uids=239749281&dopt=GenPept&RID=TFM9K94S01S&log$=prottop&blast_rank=70) | PREDICTED: hypothetical protein XP_002346979 [Homo sapiens] >ref|XP_002346093.1| PREDICTED: hypothetical protein [Homo sapiens] | [18.0](http://blast.ncbi.nlm.nih.gov/Blast.cgi#239749281%23239749281) | 18.0 | 62% | 518 |
| [XP_002344469.1](http://www.ncbi.nlm.nih.gov/entrez/query.fcgi?cmd=Retrieve&db=Protein&list_uids=239509062&dopt=GenPept&RID=TFM9K94S01S&log$=prottop&blast_rank=71) | PREDICTED: hypothetical protein [Homo sapiens] | [18.0](http://blast.ncbi.nlm.nih.gov/Blast.cgi#239509062%23239509062) | 18.0 | 62% | 518 |
| [NP_055919.2](http://www.ncbi.nlm.nih.gov/entrez/query.fcgi?cmd=Retrieve&db=Protein&list_uids=239047271&dopt=GenPept&RID=TFM9K94S01S&log$=prottop&blast_rank=72) | autophagy-related protein 2 homolog A [Homo sapiens] | [18.0](http://blast.ncbi.nlm.nih.gov/Blast.cgi#239047271%23239047271) | 32.7 | 62% | 518 |
| [XP_001719513.1](http://www.ncbi.nlm.nih.gov/entrez/query.fcgi?cmd=Retrieve&db=Protein&list_uids=169218211&dopt=GenPept&RID=TFM9K94S01S&log$=prottop&blast_rank=73) | PREDICTED: similar to plectin 1, partial [Homo sapiens] | [18.0](http://blast.ncbi.nlm.nih.gov/Blast.cgi#169218211%23169218211) | 63.2 | 100% | 518 |
| [NP_001138461.1](http://www.ncbi.nlm.nih.gov/entrez/query.fcgi?cmd=Retrieve&db=Protein&list_uids=222418585&dopt=GenPept&RID=TFM9K94S01S&log$=prottop&blast_rank=74) | zinc finger protein 814 [Homo sapiens] | [18.0](http://blast.ncbi.nlm.nih.gov/Blast.cgi#222418585%23222418585) | 18.0 | 62% | 518 |
| [NP_110413.2](http://www.ncbi.nlm.nih.gov/entrez/query.fcgi?cmd=Retrieve&db=Protein&list_uids=166706887&dopt=GenPept&RID=TFM9K94S01S&log$=prottop&blast_rank=75) | syncoilin, intermediate filament 1 isoform 1 [Homo sapiens] | [18.0](http://blast.ncbi.nlm.nih.gov/Blast.cgi#166706887%23166706887) | 18.0 | 62% | 518 |
| [NP_001104489.1](http://www.ncbi.nlm.nih.gov/entrez/query.fcgi?cmd=Retrieve&db=Protein&list_uids=161169017&dopt=GenPept&RID=TFM9K94S01S&log$=prottop&blast_rank=76) | neuron navigator 2 isoform 4 [Homo sapiens] | [18.0](http://blast.ncbi.nlm.nih.gov/Blast.cgi#161169017%23161169017) | 32.2 | 100% | 518 |
| [NP_001104488.1](http://www.ncbi.nlm.nih.gov/entrez/query.fcgi?cmd=Retrieve&db=Protein&list_uids=161169015&dopt=GenPept&RID=TFM9K94S01S&log$=prottop&blast_rank=77) | neuron navigator 2 isoform 3 [Homo sapiens] | [18.0](http://blast.ncbi.nlm.nih.gov/Blast.cgi#161169015%23161169015) | 32.2 | 100% | 518 |
| [NP_892009.3](http://www.ncbi.nlm.nih.gov/entrez/query.fcgi?cmd=Retrieve&db=Protein&list_uids=161169013&dopt=GenPept&RID=TFM9K94S01S&log$=prottop&blast_rank=78) | neuron navigator 2 isoform 1 [Homo sapiens] | [18.0](http://blast.ncbi.nlm.nih.gov/Blast.cgi#161169013%23161169013) | 32.2 | 100% | 518 |
| [NP_056288.1](http://www.ncbi.nlm.nih.gov/entrez/query.fcgi?cmd=Retrieve&db=Protein&list_uids=150456415&dopt=GenPept&RID=TFM9K94S01S&log$=prottop&blast_rank=79) | HEAT repeat containing 5A [Homo sapiens] | [18.0](http://blast.ncbi.nlm.nih.gov/Blast.cgi#150456415%23150456415) | 18.0 | 62% | 518 |
| [NP_149015.2](http://www.ncbi.nlm.nih.gov/entrez/query.fcgi?cmd=Retrieve&db=Protein&list_uids=150378539&dopt=GenPept&RID=TFM9K94S01S&log$=prottop&blast_rank=80) | protein piccolo isoform 1 [Homo sapiens] | [18.0](http://blast.ncbi.nlm.nih.gov/Blast.cgi#150378539%23150378539) | 31.8 | 75% | 518 |
| [NP_055325.2](http://www.ncbi.nlm.nih.gov/entrez/query.fcgi?cmd=Retrieve&db=Protein&list_uids=150170670&dopt=GenPept&RID=TFM9K94S01S&log$=prottop&blast_rank=81) | protein piccolo isoform 2 [Homo sapiens] | [18.0](http://blast.ncbi.nlm.nih.gov/Blast.cgi#150170670%23150170670) | 31.8 | 75% | 518 |
| [NP_061198.2](http://www.ncbi.nlm.nih.gov/entrez/query.fcgi?cmd=Retrieve&db=Protein&list_uids=153945715&dopt=GenPept&RID=TFM9K94S01S&log$=prottop&blast_rank=82) | myosin-Vc [Homo sapiens] | [18.0](http://blast.ncbi.nlm.nih.gov/Blast.cgi#153945715%23153945715) | 31.4 | 87% | 518 |
| [NP_065176.3](http://www.ncbi.nlm.nih.gov/entrez/query.fcgi?cmd=Retrieve&db=Protein&list_uids=267844811&dopt=GenPept&RID=TFM9K94S01S&log$=prottop&blast_rank=83) | neuron navigator 1 isoform 1 [Homo sapiens] | [18.0](http://blast.ncbi.nlm.nih.gov/Blast.cgi#267844811%23267844811) | 18.0 | 100% | 518 |
| [NP_001161210.1](http://www.ncbi.nlm.nih.gov/entrez/query.fcgi?cmd=Retrieve&db=Protein&list_uids=267844813&dopt=GenPept&RID=TFM9K94S01S&log$=prottop&blast_rank=84) | neuron navigator 1 isoform 2 [Homo sapiens] | [18.0](http://blast.ncbi.nlm.nih.gov/Blast.cgi#267844813%23267844813) | 18.0 | 100% | 518 |
| [NP_951008.1](http://www.ncbi.nlm.nih.gov/entrez/query.fcgi?cmd=Retrieve&db=Protein&list_uids=39995078&dopt=GenPept&RID=TFM9K94S01S&log$=prottop&blast_rank=85) | hypothetical protein LOC60684 isoform b [Homo sapiens] | [18.0](http://blast.ncbi.nlm.nih.gov/Blast.cgi#39995078%2339995078) | 18.0 | 75% | 518 |
| [NP_056450.2](http://www.ncbi.nlm.nih.gov/entrez/query.fcgi?cmd=Retrieve&db=Protein&list_uids=51093832&dopt=GenPept&RID=TFM9K94S01S&log$=prottop&blast_rank=86) | GTPase-activating protein and VPS9 domain-containing protein 1 [Homo sapiens] | [18.0](http://blast.ncbi.nlm.nih.gov/Blast.cgi#51093832%2351093832) | 28.8 | 75% | 518 |
| [NP_001026859.1](http://www.ncbi.nlm.nih.gov/entrez/query.fcgi?cmd=Retrieve&db=Protein&list_uids=72534670&dopt=GenPept&RID=TFM9K94S01S&log$=prottop&blast_rank=87) | phospholipase A-2-activating protein [Homo sapiens] | [18.0](http://blast.ncbi.nlm.nih.gov/Blast.cgi#72534670%2372534670) | 18.0 | 75% | 518 |
| [NP_660093.2](http://www.ncbi.nlm.nih.gov/entrez/query.fcgi?cmd=Retrieve&db=Protein&list_uids=38044282&dopt=GenPept&RID=TFM9K94S01S&log$=prottop&blast_rank=88) | neuron navigator 2 isoform 2 [Homo sapiens] | [18.0](http://blast.ncbi.nlm.nih.gov/Blast.cgi#38044282%2338044282) | 32.2 | 100% | 518 |
| [NP_000594.2](http://www.ncbi.nlm.nih.gov/entrez/query.fcgi?cmd=Retrieve&db=Protein&list_uids=40254422&dopt=GenPept&RID=TFM9K94S01S&log$=prottop&blast_rank=89) | nitric oxide synthase, endothelial isoform 1 [Homo sapiens] | [18.0](http://blast.ncbi.nlm.nih.gov/Blast.cgi#40254422%2340254422) | 32.7 | 62% | 518 |
| [NP_056173.1](http://www.ncbi.nlm.nih.gov/entrez/query.fcgi?cmd=Retrieve&db=Protein&list_uids=28872812&dopt=GenPept&RID=TFM9K94S01S&log$=prottop&blast_rank=90) | MORC family CW-type zinc finger protein 3 [Homo sapiens] | [18.0](http://blast.ncbi.nlm.nih.gov/Blast.cgi#28872812%2328872812) | 18.0 | 62% | 518 |
| [NP_958780.1](http://www.ncbi.nlm.nih.gov/entrez/query.fcgi?cmd=Retrieve&db=Protein&list_uids=41322912&dopt=GenPept&RID=TFM9K94S01S&log$=prottop&blast_rank=91) | plectin-1 isoform 1f [Homo sapiens] | [18.0](http://blast.ncbi.nlm.nih.gov/Blast.cgi#41322912%2341322912) | 60.7 | 100% | 518 |
| [NP_958786.1](http://www.ncbi.nlm.nih.gov/entrez/query.fcgi?cmd=Retrieve&db=Protein&list_uids=41322923&dopt=GenPept&RID=TFM9K94S01S&log$=prottop&blast_rank=92) | plectin-1 isoform 1a [Homo sapiens] | [18.0](http://blast.ncbi.nlm.nih.gov/Blast.cgi#41322923%2341322923) | 60.7 | 100% | 518 |
| [NP_000436.2](http://www.ncbi.nlm.nih.gov/entrez/query.fcgi?cmd=Retrieve&db=Protein&list_uids=47607492&dopt=GenPept&RID=TFM9K94S01S&log$=prottop&blast_rank=93) | plectin-1 isoform 1c [Homo sapiens] | [18.0](http://blast.ncbi.nlm.nih.gov/Blast.cgi#47607492%2347607492) | 60.7 | 100% | 518 |
| [NP_958783.1](http://www.ncbi.nlm.nih.gov/entrez/query.fcgi?cmd=Retrieve&db=Protein&list_uids=41322910&dopt=GenPept&RID=TFM9K94S01S&log$=prottop&blast_rank=94) | plectin-1 isoform 1d [Homo sapiens] | [18.0](http://blast.ncbi.nlm.nih.gov/Blast.cgi#41322910%2341322910) | 60.7 | 100% | 518 |
| [NP_958785.1](http://www.ncbi.nlm.nih.gov/entrez/query.fcgi?cmd=Retrieve&db=Protein&list_uids=41322914&dopt=GenPept&RID=TFM9K94S01S&log$=prottop&blast_rank=95) | plectin-1 isoform 1g [Homo sapiens] | [18.0](http://blast.ncbi.nlm.nih.gov/Blast.cgi#41322914%2341322914) | 60.7 | 100% | 518 |
| [NP_958781.1](http://www.ncbi.nlm.nih.gov/entrez/query.fcgi?cmd=Retrieve&db=Protein&list_uids=41322908&dopt=GenPept&RID=TFM9K94S01S&log$=prottop&blast_rank=96) | plectin-1 isoform 1e [Homo sapiens] | [18.0](http://blast.ncbi.nlm.nih.gov/Blast.cgi#41322908%2341322908) | 60.7 | 100% | 518 |
| [NP_958782.1](http://www.ncbi.nlm.nih.gov/entrez/query.fcgi?cmd=Retrieve&db=Protein&list_uids=41322916&dopt=GenPept&RID=TFM9K94S01S&log$=prottop&blast_rank=97) | plectin-1 isoform 1 [Homo sapiens] | [18.0](http://blast.ncbi.nlm.nih.gov/Blast.cgi#41322916%2341322916) | 60.7 | 100% | 518 |
| [NP_958784.1](http://www.ncbi.nlm.nih.gov/entrez/query.fcgi?cmd=Retrieve&db=Protein&list_uids=41322919&dopt=GenPept&RID=TFM9K94S01S&log$=prottop&blast_rank=98) | plectin-1 isoform 1b [Homo sapiens] | [18.0](http://blast.ncbi.nlm.nih.gov/Blast.cgi#41322919%2341322919) | 60.7 | 100% | 518 |
| [NP_071919.2](http://www.ncbi.nlm.nih.gov/entrez/query.fcgi?cmd=Retrieve&db=Protein&list_uids=38570142&dopt=GenPept&RID=TFM9K94S01S&log$=prottop&blast_rank=99) | membrane protein, palmitoylated 5 [Homo sapiens] | [18.0](http://blast.ncbi.nlm.nih.gov/Blast.cgi#38570142%2338570142) | 30.1 | 100% | 518 |
| [NP_705717.1](http://www.ncbi.nlm.nih.gov/entrez/query.fcgi?cmd=Retrieve&db=Protein&list_uids=41281798&dopt=GenPept&RID=TFM9K94S01S&log$=prottop&blast_rank=100) | mitogen-activated protein kinase kinase kinase 7-interacting protein 1 isoform beta [Homo sapiens] | [18.0](http://blast.ncbi.nlm.nih.gov/Blast.cgi#41281798%2341281798) | 18.0 | 75% | 518 |

| **Accession** | **Proteins with a match to SWSGYYTY peptide** | **[Max score](http://blast.ncbi.nlm.nih.gov/Blast.cgi?CMD=Get&ALIGNMENTS=100&ALIGNMENT_VIEW=Pairwise&CDD_SEARCH_STATE=1&DATABASE_SORT=0&DESCRIPTIONS=100&ENTREZ_QUERY=txid9606 %5BORGN%5D&FIRST_QUERY_NUM=0&FORMAT_OBJECT=Alignment&FORMAT_PAGE_TARGET=&FORMAT_TYPE=HTML&GET_SEQUENCE=yes&I_THRESH=&MASK_CHAR=2&MASK_COLOR=1&NEW_VIEW=yes&NUM_OVERVIEW=100&OLD_BLAST=false&PAGE=Proteins&QUERY_INDEX=0&QUERY_NUMBER=0&RESULTS_PAGE_TARGET=&RID=TFMFHDKY01S&SHOW_LINKOUT=yes&SHOW_OVERVIEW=yes&STEP_NUMBER=&WORD_SIZE=2&DISPLAY_SORT=1&HSP_SORT=1" \l "sort_mark)** | **[Total score](http://blast.ncbi.nlm.nih.gov/Blast.cgi?CMD=Get&ALIGNMENTS=100&ALIGNMENT_VIEW=Pairwise&CDD_SEARCH_STATE=1&DATABASE_SORT=0&DESCRIPTIONS=100&ENTREZ_QUERY=txid9606 %5BORGN%5D&FIRST_QUERY_NUM=0&FORMAT_OBJECT=Alignment&FORMAT_PAGE_TARGET=&FORMAT_TYPE=HTML&GET_SEQUENCE=yes&I_THRESH=&MASK_CHAR=2&MASK_COLOR=1&NEW_VIEW=yes&NUM_OVERVIEW=100&OLD_BLAST=false&PAGE=Proteins&QUERY_INDEX=0&QUERY_NUMBER=0&RESULTS_PAGE_TARGET=&RID=TFMFHDKY01S&SHOW_LINKOUT=yes&SHOW_OVERVIEW=yes&STEP_NUMBER=&WORD_SIZE=2&DISPLAY_SORT=2&HSP_SORT=1" \l "sort_mark)** | **[Query coverage](http://blast.ncbi.nlm.nih.gov/Blast.cgi?CMD=Get&ALIGNMENTS=100&ALIGNMENT_VIEW=Pairwise&CDD_SEARCH_STATE=1&DATABASE_SORT=0&DESCRIPTIONS=100&ENTREZ_QUERY=txid9606 %5BORGN%5D&FIRST_QUERY_NUM=0&FORMAT_OBJECT=Alignment&FORMAT_PAGE_TARGET=&FORMAT_TYPE=HTML&GET_SEQUENCE=yes&I_THRESH=&MASK_CHAR=2&MASK_COLOR=1&NEW_VIEW=yes&NUM_OVERVIEW=100&OLD_BLAST=false&PAGE=Proteins&QUERY_INDEX=0&QUERY_NUMBER=0&RESULTS_PAGE_TARGET=&RID=TFMFHDKY01S&SHOW_LINKOUT=yes&SHOW_OVERVIEW=yes&STEP_NUMBER=&WORD_SIZE=2&DISPLAY_SORT=4&HSP_SORT=0" \l "sort_mark)** | **[E value](http://blast.ncbi.nlm.nih.gov/Blast.cgi?CMD=Get&ALIGNMENTS=100&ALIGNMENT_VIEW=Pairwise&CDD_SEARCH_STATE=1&DATABASE_SORT=0&DESCRIPTIONS=100&ENTREZ_QUERY=txid9606 %5BORGN%5D&FIRST_QUERY_NUM=0&FORMAT_OBJECT=Alignment&FORMAT_PAGE_TARGET=&FORMAT_TYPE=HTML&GET_SEQUENCE=yes&I_THRESH=&MASK_CHAR=2&MASK_COLOR=1&NEW_VIEW=yes&NUM_OVERVIEW=100&OLD_BLAST=false&PAGE=Proteins&QUERY_INDEX=0&QUERY_NUMBER=0&RESULTS_PAGE_TARGET=&RID=TFMFHDKY01S&SHOW_LINKOUT=yes&SHOW_OVERVIEW=yes&STEP_NUMBER=&WORD_SIZE=2&DISPLAY_SORT=0&HSP_SORT=0" \l "sort_mark)** |
| --- | --- | --- | --- | --- | --- |
| [NP_006113.2](http://www.ncbi.nlm.nih.gov/entrez/query.fcgi?cmd=Retrieve&db=Protein&list_uids=51477716&dopt=GenPept&RID=TFMFHDKY01S&log$=prottop&blast_rank=1) | alpha-mannosidase 2x [Homo sapiens] | [22.7](http://blast.ncbi.nlm.nih.gov/Blast.cgi#51477716%2351477716) | 22.7 | 75% | 20 |
| [NP_002363.2](http://www.ncbi.nlm.nih.gov/entrez/query.fcgi?cmd=Retrieve&db=Protein&list_uids=51477714&dopt=GenPept&RID=TFMFHDKY01S&log$=prottop&blast_rank=2) | alpha-mannosidase 2 [Homo sapiens] | [21.8](http://blast.ncbi.nlm.nih.gov/Blast.cgi#51477714%2351477714) | 21.8 | 75% | 37 |
| [NP_060017.1](http://www.ncbi.nlm.nih.gov/entrez/query.fcgi?cmd=Retrieve&db=Protein&list_uids=8923708&dopt=GenPept&RID=TFMFHDKY01S&log$=prottop&blast_rank=3) | FAD-dependent oxidoreductase domain-containing protein 1 [Homo sapiens] | [21.8](http://blast.ncbi.nlm.nih.gov/Blast.cgi#8923708%238923708) | 21.8 | 87% | 37 |
| [NP_001136091.1](http://www.ncbi.nlm.nih.gov/entrez/query.fcgi?cmd=Retrieve&db=Protein&list_uids=217330584&dopt=GenPept&RID=TFMFHDKY01S&log$=prottop&blast_rank=4) | stimulated by retinoic acid gene 6 protein homolog isoform b [Homo sapiens] | [21.4](http://blast.ncbi.nlm.nih.gov/Blast.cgi#217330584%23217330584) | 31.8 | 62% | 49 |
| [NP_071764.3](http://www.ncbi.nlm.nih.gov/entrez/query.fcgi?cmd=Retrieve&db=Protein&list_uids=217330578&dopt=GenPept&RID=TFMFHDKY01S&log$=prottop&blast_rank=5) | stimulated by retinoic acid gene 6 protein homolog isoform a [Homo sapiens] >ref|NP_001136089.1| stimulated by retinoic acid gene 6 protein homolog isoform a [Homo sapiens] >ref|NP_001136090.1| stimulated by retinoic acid gene 6 protein homolog isoform a [Homo sapiens] | [21.4](http://blast.ncbi.nlm.nih.gov/Blast.cgi#217330578%23217330578) | 31.8 | 62% | 49 |
| [NP_006681.1](http://www.ncbi.nlm.nih.gov/entrez/query.fcgi?cmd=Retrieve&db=Protein&list_uids=5729929&dopt=GenPept&RID=TFMFHDKY01S&log$=prottop&blast_rank=6) | matrix metalloproteinase 24 preproprotein [Homo sapiens] | [21.4](http://blast.ncbi.nlm.nih.gov/Blast.cgi#5729929%235729929) | 21.4 | 62% | 49 |
| [NP_055798.2](http://www.ncbi.nlm.nih.gov/entrez/query.fcgi?cmd=Retrieve&db=Protein&list_uids=241982802&dopt=GenPept&RID=TFMFHDKY01S&log$=prottop&blast_rank=7) | HMG domain-containing protein 3 [Homo sapiens] | [21.0](http://blast.ncbi.nlm.nih.gov/Blast.cgi#241982802%23241982802) | 21.0 | 75% | 66 |
| [NP_061862.1](http://www.ncbi.nlm.nih.gov/entrez/query.fcgi?cmd=Retrieve&db=Protein&list_uids=168229174&dopt=GenPept&RID=TFMFHDKY01S&log$=prottop&blast_rank=8) | RNA-binding protein 27 [Homo sapiens] | [21.0](http://blast.ncbi.nlm.nih.gov/Blast.cgi#168229174%23168229174) | 21.0 | 75% | 66 |
| [NP_055434.2](http://www.ncbi.nlm.nih.gov/entrez/query.fcgi?cmd=Retrieve&db=Protein&list_uids=29029595&dopt=GenPept&RID=TFMFHDKY01S&log$=prottop&blast_rank=9) | glutamate receptor, ionotropic kainate 4 precursor [Homo sapiens] | [20.6](http://blast.ncbi.nlm.nih.gov/Blast.cgi#29029595%2329029595) | 20.6 | 75% | 89 |
| [NP_699191.1](http://www.ncbi.nlm.nih.gov/entrez/query.fcgi?cmd=Retrieve&db=Protein&list_uids=23503291&dopt=GenPept&RID=TFMFHDKY01S&log$=prottop&blast_rank=10) | protein APCDD1-like precursor [Homo sapiens] | [20.6](http://blast.ncbi.nlm.nih.gov/Blast.cgi#23503291%2323503291) | 20.6 | 75% | 89 |
| [NP_079150.3](http://www.ncbi.nlm.nih.gov/entrez/query.fcgi?cmd=Retrieve&db=Protein&list_uids=33359221&dopt=GenPept&RID=TFMFHDKY01S&log$=prottop&blast_rank=11) | dyslexia susceptibility 2-like [Homo sapiens] | [20.2](http://blast.ncbi.nlm.nih.gov/Blast.cgi#33359221%2333359221) | 20.2 | 75% | 119 |
| [NP_001032251.1](http://www.ncbi.nlm.nih.gov/entrez/query.fcgi?cmd=Retrieve&db=Protein&list_uids=82546836&dopt=GenPept&RID=TFMFHDKY01S&log$=prottop&blast_rank=12) | ADP-ribosylation factor-like protein 5A isoform 2 [Homo sapiens] >ref|NP_817114.2| ADP-ribosylation factor-like protein 5A isoform 2 [Homo sapiens] | [20.2](http://blast.ncbi.nlm.nih.gov/Blast.cgi#82546836%2382546836) | 20.2 | 87% | 119 |
| [NP_036229.1](http://www.ncbi.nlm.nih.gov/entrez/query.fcgi?cmd=Retrieve&db=Protein&list_uids=6912244&dopt=GenPept&RID=TFMFHDKY01S&log$=prottop&blast_rank=13) | ADP-ribosylation factor-like protein 5A isoform 1 [Homo sapiens] | [20.2](http://blast.ncbi.nlm.nih.gov/Blast.cgi#6912244%236912244) | 20.2 | 87% | 119 |
| [NP_065770.1](http://www.ncbi.nlm.nih.gov/entrez/query.fcgi?cmd=Retrieve&db=Protein&list_uids=153792074&dopt=GenPept&RID=TFMFHDKY01S&log$=prottop&blast_rank=14) | proline-rich protein 12 [Homo sapiens] | [19.7](http://blast.ncbi.nlm.nih.gov/Blast.cgi#153792074%23153792074) | 19.7 | 62% | 160 |
| [NP_001034852.2](http://www.ncbi.nlm.nih.gov/entrez/query.fcgi?cmd=Retrieve&db=Protein&list_uids=193290174&dopt=GenPept&RID=TFMFHDKY01S&log$=prottop&blast_rank=15) | transmembrane protein 232 [Homo sapiens] | [19.7](http://blast.ncbi.nlm.nih.gov/Blast.cgi#193290174%23193290174) | 19.7 | 62% | 160 |
| [NP_000203.2](http://www.ncbi.nlm.nih.gov/entrez/query.fcgi?cmd=Retrieve&db=Protein&list_uids=47078292&dopt=GenPept&RID=TFMFHDKY01S&log$=prottop&blast_rank=16) | integrin beta chain, beta 3 precursor [Homo sapiens] | [19.7](http://blast.ncbi.nlm.nih.gov/Blast.cgi#47078292%2347078292) | 19.7 | 62% | 160 |
| [NP_056655.2](http://www.ncbi.nlm.nih.gov/entrez/query.fcgi?cmd=Retrieve&db=Protein&list_uids=70166944&dopt=GenPept&RID=TFMFHDKY01S&log$=prottop&blast_rank=17) | adenosine deaminase, RNA-specific isoform b [Homo sapiens] | [19.7](http://blast.ncbi.nlm.nih.gov/Blast.cgi#70166944%2370166944) | 19.7 | 62% | 160 |
| [NP_056656.2](http://www.ncbi.nlm.nih.gov/entrez/query.fcgi?cmd=Retrieve&db=Protein&list_uids=70167032&dopt=GenPept&RID=TFMFHDKY01S&log$=prottop&blast_rank=18) | adenosine deaminase, RNA-specific isoform c [Homo sapiens] | [19.7](http://blast.ncbi.nlm.nih.gov/Blast.cgi#70167032%2370167032) | 19.7 | 62% | 160 |
| [NP_060049.2](http://www.ncbi.nlm.nih.gov/entrez/query.fcgi?cmd=Retrieve&db=Protein&list_uids=148539844&dopt=GenPept&RID=TFMFHDKY01S&log$=prottop&blast_rank=19) | deleted in malignant brain tumors 1 isoform c precursor [Homo sapiens] | [19.7](http://blast.ncbi.nlm.nih.gov/Blast.cgi#148539844%23148539844) | 19.7 | 62% | 160 |
| [NP_004397.2](http://www.ncbi.nlm.nih.gov/entrez/query.fcgi?cmd=Retrieve&db=Protein&list_uids=148539840&dopt=GenPept&RID=TFMFHDKY01S&log$=prottop&blast_rank=20) | deleted in malignant brain tumors 1 isoform a precursor [Homo sapiens] | [19.7](http://blast.ncbi.nlm.nih.gov/Blast.cgi#148539840%23148539840) | 19.7 | 62% | 160 |
| [NP_775873.2](http://www.ncbi.nlm.nih.gov/entrez/query.fcgi?cmd=Retrieve&db=Protein&list_uids=55749758&dopt=GenPept&RID=TFMFHDKY01S&log$=prottop&blast_rank=21) | disco-interacting protein 2 homolog B [Homo sapiens] | [19.7](http://blast.ncbi.nlm.nih.gov/Blast.cgi#55749758%2355749758) | 19.7 | 62% | 160 |
| [NP_001102.2](http://www.ncbi.nlm.nih.gov/entrez/query.fcgi?cmd=Retrieve&db=Protein&list_uids=70166852&dopt=GenPept&RID=TFMFHDKY01S&log$=prottop&blast_rank=22) | adenosine deaminase, RNA-specific isoform a [Homo sapiens] | [19.7](http://blast.ncbi.nlm.nih.gov/Blast.cgi#70166852%2370166852) | 19.7 | 62% | 160 |
| [NP_065744.2](http://www.ncbi.nlm.nih.gov/entrez/query.fcgi?cmd=Retrieve&db=Protein&list_uids=21359935&dopt=GenPept&RID=TFMFHDKY01S&log$=prottop&blast_rank=23) | down syndrome cell adhesion molecule-like protein 1 [Homo sapiens] | [19.7](http://blast.ncbi.nlm.nih.gov/Blast.cgi#21359935%2321359935) | 19.7 | 62% | 160 |
| [NP_015568.2](http://www.ncbi.nlm.nih.gov/entrez/query.fcgi?cmd=Retrieve&db=Protein&list_uids=148539842&dopt=GenPept&RID=TFMFHDKY01S&log$=prottop&blast_rank=24) | deleted in malignant brain tumors 1 isoform b precursor [Homo sapiens] | [19.7](http://blast.ncbi.nlm.nih.gov/Blast.cgi#148539842%23148539842) | 19.7 | 62% | 160 |
| [NP_001136151.1](http://www.ncbi.nlm.nih.gov/entrez/query.fcgi?cmd=Retrieve&db=Protein&list_uids=218156305&dopt=GenPept&RID=TFMFHDKY01S&log$=prottop&blast_rank=25) | anoctamin-6 isoform c [Homo sapiens] >ref|NP_001136152.1| anoctamin-6 isoform c [Homo sapiens] | [19.3](http://blast.ncbi.nlm.nih.gov/Blast.cgi#218156305%23218156305) | 29.7 | 87% | 214 |
| [NP_001136150.1](http://www.ncbi.nlm.nih.gov/entrez/query.fcgi?cmd=Retrieve&db=Protein&list_uids=218156303&dopt=GenPept&RID=TFMFHDKY01S&log$=prottop&blast_rank=26) | anoctamin-6 isoform b [Homo sapiens] | [19.3](http://blast.ncbi.nlm.nih.gov/Blast.cgi#218156303%23218156303) | 29.7 | 87% | 214 |
| [NP_001129518.1](http://www.ncbi.nlm.nih.gov/entrez/query.fcgi?cmd=Retrieve&db=Protein&list_uids=209862893&dopt=GenPept&RID=TFMFHDKY01S&log$=prottop&blast_rank=27) | zinc finger MYND domain-containing protein 15 isoform 1 [Homo sapiens] | [19.3](http://blast.ncbi.nlm.nih.gov/Blast.cgi#209862893%23209862893) | 19.3 | 87% | 214 |
| [NP_001020527.2](http://www.ncbi.nlm.nih.gov/entrez/query.fcgi?cmd=Retrieve&db=Protein&list_uids=218156299&dopt=GenPept&RID=TFMFHDKY01S&log$=prottop&blast_rank=28) | anoctamin-6 isoform a [Homo sapiens] | [19.3](http://blast.ncbi.nlm.nih.gov/Blast.cgi#218156299%23218156299) | 29.7 | 87% | 214 |
| [NP_056089.1](http://www.ncbi.nlm.nih.gov/entrez/query.fcgi?cmd=Retrieve&db=Protein&list_uids=50659093&dopt=GenPept&RID=TFMFHDKY01S&log$=prottop&blast_rank=29) | epididymis-specific alpha-mannosidase precursor [Homo sapiens] | [19.3](http://blast.ncbi.nlm.nih.gov/Blast.cgi#50659093%2350659093) | 32.7 | 75% | 214 |
| [NP_057332.1](http://www.ncbi.nlm.nih.gov/entrez/query.fcgi?cmd=Retrieve&db=Protein&list_uids=7706457&dopt=GenPept&RID=TFMFHDKY01S&log$=prottop&blast_rank=30) | A-kinase anchor protein 11 [Homo sapiens] | [19.3](http://blast.ncbi.nlm.nih.gov/Blast.cgi#7706457%237706457) | 19.3 | 75% | 214 |
| [NP_150094.5](http://www.ncbi.nlm.nih.gov/entrez/query.fcgi?cmd=Retrieve&db=Protein&list_uids=259013213&dopt=GenPept&RID=TFMFHDKY01S&log$=prottop&blast_rank=31) | CUB and sushi domain-containing protein 1 precursor [Homo sapiens] | [18.9](http://blast.ncbi.nlm.nih.gov/Blast.cgi#259013213%23259013213) | 62.8 | 75% | 288 |
| [XP_002344706.1](http://www.ncbi.nlm.nih.gov/entrez/query.fcgi?cmd=Retrieve&db=Protein&list_uids=239755805&dopt=GenPept&RID=TFMFHDKY01S&log$=prottop&blast_rank=32) | PREDICTED: hypothetical protein [Homo sapiens] | [18.9](http://blast.ncbi.nlm.nih.gov/Blast.cgi#239755805%23239755805) | 18.9 | 50% | 288 |
| [XP_002345809.1](http://www.ncbi.nlm.nih.gov/entrez/query.fcgi?cmd=Retrieve&db=Protein&list_uids=239753878&dopt=GenPept&RID=TFMFHDKY01S&log$=prottop&blast_rank=33) | PREDICTED: hypothetical protein XP_002345809, partial [Homo sapiens] | [18.9](http://blast.ncbi.nlm.nih.gov/Blast.cgi#239753878%23239753878) | 18.9 | 50% | 288 |
| [NP_443132.3](http://www.ncbi.nlm.nih.gov/entrez/query.fcgi?cmd=Retrieve&db=Protein&list_uids=205277354&dopt=GenPept&RID=TFMFHDKY01S&log$=prottop&blast_rank=34) | CUB and sushi domain-containing protein 3 isoform 3 [Homo sapiens] | [18.9](http://blast.ncbi.nlm.nih.gov/Blast.cgi#205277354%23205277354) | 101 | 62% | 288 |
| [NP_067008.3](http://www.ncbi.nlm.nih.gov/entrez/query.fcgi?cmd=Retrieve&db=Protein&list_uids=187761334&dopt=GenPept&RID=TFMFHDKY01S&log$=prottop&blast_rank=35) | cation channel sperm-associated protein subunit gamma precursor [Homo sapiens] | [18.9](http://blast.ncbi.nlm.nih.gov/Blast.cgi#187761334%23187761334) | 18.9 | 50% | 288 |
| [NP_001120680.1](http://www.ncbi.nlm.nih.gov/entrez/query.fcgi?cmd=Retrieve&db=Protein&list_uids=187761317&dopt=GenPept&RID=TFMFHDKY01S&log$=prottop&blast_rank=36) | probable methylcytosine dioxygenase TET2 isoform a [Homo sapiens] | [18.9](http://blast.ncbi.nlm.nih.gov/Blast.cgi#187761317%23187761317) | 18.9 | 75% | 288 |
| [XP_001719515.1](http://www.ncbi.nlm.nih.gov/entrez/query.fcgi?cmd=Retrieve&db=Protein&list_uids=169218213&dopt=GenPept&RID=TFMFHDKY01S&log$=prottop&blast_rank=37) | PREDICTED: similar to complement component C3, partial [Homo sapiens] | [18.9](http://blast.ncbi.nlm.nih.gov/Blast.cgi#169218213%23169218213) | 18.9 | 50% | 288 |
| [NP_068556.2](http://www.ncbi.nlm.nih.gov/entrez/query.fcgi?cmd=Retrieve&db=Protein&list_uids=194394143&dopt=GenPept&RID=TFMFHDKY01S&log$=prottop&blast_rank=38) | hepatocyte nuclear factor 3-beta isoform 1 [Homo sapiens] | [18.9](http://blast.ncbi.nlm.nih.gov/Blast.cgi#194394143%23194394143) | 18.9 | 62% | 288 |
| [NP_001165084.1](http://www.ncbi.nlm.nih.gov/entrez/query.fcgi?cmd=Retrieve&db=Protein&list_uids=284172431&dopt=GenPept&RID=TFMFHDKY01S&log$=prottop&blast_rank=39) | prolyl endopeptidase-like isoform 4 [Homo sapiens] >ref|NP_001165088.1| prolyl endopeptidase-like isoform 4 [Homo sapiens] | [18.9](http://blast.ncbi.nlm.nih.gov/Blast.cgi#284172431%23284172431) | 18.9 | 50% | 288 |
| [NP_001035844.1](http://www.ncbi.nlm.nih.gov/entrez/query.fcgi?cmd=Retrieve&db=Protein&list_uids=108860686&dopt=GenPept&RID=TFMFHDKY01S&log$=prottop&blast_rank=40) | prolyl endopeptidase-like isoform 2 [Homo sapiens] | [18.9](http://blast.ncbi.nlm.nih.gov/Blast.cgi#108860686%23108860686) | 18.9 | 50% | 288 |
| [NP_848621.2](http://www.ncbi.nlm.nih.gov/entrez/query.fcgi?cmd=Retrieve&db=Protein&list_uids=258547124&dopt=GenPept&RID=TFMFHDKY01S&log$=prottop&blast_rank=41) | solute carrier family 25 member 42 [Homo sapiens] | [18.9](http://blast.ncbi.nlm.nih.gov/Blast.cgi#258547124%23258547124) | 18.9 | 50% | 288 |
| [NP_001206.2](http://www.ncbi.nlm.nih.gov/entrez/query.fcgi?cmd=Retrieve&db=Protein&list_uids=70167127&dopt=GenPept&RID=TFMFHDKY01S&log$=prottop&blast_rank=42) | carbonic anhydrase VI precursor [Homo sapiens] | [18.9](http://blast.ncbi.nlm.nih.gov/Blast.cgi#70167127%2370167127) | 18.9 | 50% | 288 |
| [NP_004425.2](http://www.ncbi.nlm.nih.gov/entrez/query.fcgi?cmd=Retrieve&db=Protein&list_uids=56790930&dopt=GenPept&RID=TFMFHDKY01S&log$=prottop&blast_rank=43) | echinoderm microtubule-associated protein-like 1 isoform b [Homo sapiens] | [18.9](http://blast.ncbi.nlm.nih.gov/Blast.cgi#56790930%2356790930) | 18.9 | 75% | 288 |
| [NP_000055.2](http://www.ncbi.nlm.nih.gov/entrez/query.fcgi?cmd=Retrieve&db=Protein&list_uids=115298678&dopt=GenPept&RID=TFMFHDKY01S&log$=prottop&blast_rank=44) | complement component 3 precursor [Homo sapiens] | [18.9](http://blast.ncbi.nlm.nih.gov/Blast.cgi#115298678%23115298678) | 18.9 | 50% | 288 |
| [NP_001035114.1](http://www.ncbi.nlm.nih.gov/entrez/query.fcgi?cmd=Retrieve&db=Protein&list_uids=91199552&dopt=GenPept&RID=TFMFHDKY01S&log$=prottop&blast_rank=45) | ADP-ribosylation factor-like protein 16 [Homo sapiens] | [18.9](http://blast.ncbi.nlm.nih.gov/Blast.cgi#91199552%2391199552) | 18.9 | 62% | 288 |
| [NP_006027.2](http://www.ncbi.nlm.nih.gov/entrez/query.fcgi?cmd=Retrieve&db=Protein&list_uids=70778815&dopt=GenPept&RID=TFMFHDKY01S&log$=prottop&blast_rank=46) | prolyl endopeptidase-like isoform 1 [Homo sapiens] >ref|NP_001165074.1| prolyl endopeptidase-like isoform 1 [Homo sapiens] >ref|NP_001165077.1| prolyl endopeptidase-like isoform 1 [Homo sapiens] | [18.9](http://blast.ncbi.nlm.nih.gov/Blast.cgi#70778815%2370778815) | 18.9 | 50% | 288 |
| [NP_001008707.1](http://www.ncbi.nlm.nih.gov/entrez/query.fcgi?cmd=Retrieve&db=Protein&list_uids=56790932&dopt=GenPept&RID=TFMFHDKY01S&log$=prottop&blast_rank=47) | echinoderm microtubule-associated protein-like 1 isoform a [Homo sapiens] | [18.9](http://blast.ncbi.nlm.nih.gov/Blast.cgi#56790932%2356790932) | 18.9 | 75% | 288 |
| [NP_861522.2](http://www.ncbi.nlm.nih.gov/entrez/query.fcgi?cmd=Retrieve&db=Protein&list_uids=45580730&dopt=GenPept&RID=TFMFHDKY01S&log$=prottop&blast_rank=48) | acyl-CoA synthetase family member 4 [Homo sapiens] | [18.9](http://blast.ncbi.nlm.nih.gov/Blast.cgi#45580730%2345580730) | 18.9 | 50% | 288 |
| [NP_919254.2](http://www.ncbi.nlm.nih.gov/entrez/query.fcgi?cmd=Retrieve&db=Protein&list_uids=44890062&dopt=GenPept&RID=TFMFHDKY01S&log$=prottop&blast_rank=49) | hypothetical protein LOC91748 [Homo sapiens] >ref|NP_001036783.1| hypothetical protein LOC91748 [Homo sapiens] | [18.9](http://blast.ncbi.nlm.nih.gov/Blast.cgi#44890062%2344890062) | 18.9 | 50% | 288 |
| [NP_937757.1](http://www.ncbi.nlm.nih.gov/entrez/query.fcgi?cmd=Retrieve&db=Protein&list_uids=38045890&dopt=GenPept&RID=TFMFHDKY01S&log$=prottop&blast_rank=50) | CUB and sushi domain-containing protein 3 isoform 2 [Homo sapiens] | [18.9](http://blast.ncbi.nlm.nih.gov/Blast.cgi#38045890%2338045890) | 101 | 62% | 288 |
| [NP_937756.1](http://www.ncbi.nlm.nih.gov/entrez/query.fcgi?cmd=Retrieve&db=Protein&list_uids=38045888&dopt=GenPept&RID=TFMFHDKY01S&log$=prottop&blast_rank=51) | CUB and sushi domain-containing protein 3 isoform 1 [Homo sapiens] | [18.9](http://blast.ncbi.nlm.nih.gov/Blast.cgi#38045888%2338045888) | 101 | 62% | 288 |
| [NP_899068.1](http://www.ncbi.nlm.nih.gov/entrez/query.fcgi?cmd=Retrieve&db=Protein&list_uids=34304379&dopt=GenPept&RID=TFMFHDKY01S&log$=prottop&blast_rank=52) | inversin isoform b [Homo sapiens] | [18.9](http://blast.ncbi.nlm.nih.gov/Blast.cgi#34304379%2334304379) | 18.9 | 62% | 288 |
| [NP_055240.2](http://www.ncbi.nlm.nih.gov/entrez/query.fcgi?cmd=Retrieve&db=Protein&list_uids=34304381&dopt=GenPept&RID=TFMFHDKY01S&log$=prottop&blast_rank=53) | inversin isoform a [Homo sapiens] | [18.9](http://blast.ncbi.nlm.nih.gov/Blast.cgi#34304381%2334304381) | 18.9 | 62% | 288 |
| [NP_710141.1](http://www.ncbi.nlm.nih.gov/entrez/query.fcgi?cmd=Retrieve&db=Protein&list_uids=24497504&dopt=GenPept&RID=TFMFHDKY01S&log$=prottop&blast_rank=54) | hepatocyte nuclear factor 3-beta isoform 2 [Homo sapiens] | [18.9](http://blast.ncbi.nlm.nih.gov/Blast.cgi#24497504%2324497504) | 18.9 | 62% | 288 |
| [NP_005041.1](http://www.ncbi.nlm.nih.gov/entrez/query.fcgi?cmd=Retrieve&db=Protein&list_uids=4826958&dopt=GenPept&RID=TFMFHDKY01S&log$=prottop&blast_rank=55) | ATP-binding cassette sub-family D member 4 [Homo sapiens] | [18.9](http://blast.ncbi.nlm.nih.gov/Blast.cgi#4826958%234826958) | 18.9 | 50% | 288 |
| [NP_689926.1](http://www.ncbi.nlm.nih.gov/entrez/query.fcgi?cmd=Retrieve&db=Protein&list_uids=22749415&dopt=GenPept&RID=TFMFHDKY01S&log$=prottop&blast_rank=56) | dolichyl-diphosphooligosaccharide--protein glycosyltransferase subunit STT3A [Homo sapiens] | [18.9](http://blast.ncbi.nlm.nih.gov/Blast.cgi#22749415%2322749415) | 36.1 | 75% | 288 |
| [NP_444508.1](http://www.ncbi.nlm.nih.gov/entrez/query.fcgi?cmd=Retrieve&db=Protein&list_uids=16751917&dopt=GenPept&RID=TFMFHDKY01S&log$=prottop&blast_rank=57) | trace amine associated receptor 8 [Homo sapiens] | [18.9](http://blast.ncbi.nlm.nih.gov/Blast.cgi#16751917%2316751917) | 29.3 | 87% | 288 |
| [NP_005849.1](http://www.ncbi.nlm.nih.gov/entrez/query.fcgi?cmd=Retrieve&db=Protein&list_uids=5031579&dopt=GenPept&RID=TFMFHDKY01S&log$=prottop&blast_rank=58) | A-kinase anchor protein 8 [Homo sapiens] | [18.9](http://blast.ncbi.nlm.nih.gov/Blast.cgi#5031579%235031579) | 18.9 | 100% | 288 |
| [NP_000677.2](http://www.ncbi.nlm.nih.gov/entrez/query.fcgi?cmd=Retrieve&db=Protein&list_uids=23238240&dopt=GenPept&RID=TFMFHDKY01S&log$=prottop&blast_rank=59) | type-2 angiotensin II receptor [Homo sapiens] | [18.9](http://blast.ncbi.nlm.nih.gov/Blast.cgi#23238240%2323238240) | 18.9 | 87% | 288 |
| [NP_001012301.1](http://www.ncbi.nlm.nih.gov/entrez/query.fcgi?cmd=Retrieve&db=Protein&list_uids=59797060&dopt=GenPept&RID=TFMFHDKY01S&log$=prottop&blast_rank=60) | arylsulfatase family, member I precursor [Homo sapiens] | [18.9](http://blast.ncbi.nlm.nih.gov/Blast.cgi#59797060%2359797060) | 18.9 | 50% | 288 |
| [NP_055429.2](http://www.ncbi.nlm.nih.gov/entrez/query.fcgi?cmd=Retrieve&db=Protein&list_uids=163644283&dopt=GenPept&RID=TFMFHDKY01S&log$=prottop&blast_rank=61) | proteasome activator complex subunit 4 [Homo sapiens] | [18.5](http://blast.ncbi.nlm.nih.gov/Blast.cgi#163644283%23163644283) | 29.3 | 87% | 386 |
| [NP_001036069.1](http://www.ncbi.nlm.nih.gov/entrez/query.fcgi?cmd=Retrieve&db=Protein&list_uids=110624787&dopt=GenPept&RID=TFMFHDKY01S&log$=prottop&blast_rank=62) | 5'-3' exoribonuclease 1 isoform b [Homo sapiens] | [18.5](http://blast.ncbi.nlm.nih.gov/Blast.cgi#110624787%23110624787) | 18.5 | 100% | 386 |
| [NP_079105.4](http://www.ncbi.nlm.nih.gov/entrez/query.fcgi?cmd=Retrieve&db=Protein&list_uids=110227598&dopt=GenPept&RID=TFMFHDKY01S&log$=prottop&blast_rank=63) | putative phospholipase B-like 1 precursor [Homo sapiens] | [18.5](http://blast.ncbi.nlm.nih.gov/Blast.cgi#110227598%23110227598) | 18.5 | 87% | 386 |
| [NP_055958.2](http://www.ncbi.nlm.nih.gov/entrez/query.fcgi?cmd=Retrieve&db=Protein&list_uids=164420681&dopt=GenPept&RID=TFMFHDKY01S&log$=prottop&blast_rank=64) | methionine aminopeptidase 1 [Homo sapiens] | [18.5](http://blast.ncbi.nlm.nih.gov/Blast.cgi#164420681%23164420681) | 18.5 | 75% | 386 |
| [NP_061874.3](http://www.ncbi.nlm.nih.gov/entrez/query.fcgi?cmd=Retrieve&db=Protein&list_uids=110624792&dopt=GenPept&RID=TFMFHDKY01S&log$=prottop&blast_rank=65) | 5'-3' exoribonuclease 1 isoform a [Homo sapiens] | [18.5](http://blast.ncbi.nlm.nih.gov/Blast.cgi#110624792%23110624792) | 18.5 | 100% | 386 |
| [NP_003236.3](http://www.ncbi.nlm.nih.gov/entrez/query.fcgi?cmd=Retrieve&db=Protein&list_uids=189458821&dopt=GenPept&RID=TFMFHDKY01S&log$=prottop&blast_rank=66) | transglutaminase 3 precursor [Homo sapiens] | [18.5](http://blast.ncbi.nlm.nih.gov/Blast.cgi#189458821%23189458821) | 18.5 | 75% | 386 |
| [XP_002344743.1](http://www.ncbi.nlm.nih.gov/entrez/query.fcgi?cmd=Retrieve&db=Protein&list_uids=239755911&dopt=GenPept&RID=TFMFHDKY01S&log$=prottop&blast_rank=67) | PREDICTED: hypothetical protein [Homo sapiens] | [18.0](http://blast.ncbi.nlm.nih.gov/Blast.cgi#239755911%23239755911) | 18.0 | 50% | 518 |
| [XP_002346533.1](http://www.ncbi.nlm.nih.gov/entrez/query.fcgi?cmd=Retrieve&db=Protein&list_uids=239748112&dopt=GenPept&RID=TFMFHDKY01S&log$=prottop&blast_rank=68) | PREDICTED: hypothetical protein [Homo sapiens] >ref|XP_002345694.1| PREDICTED: hypothetical protein XP_002345694 [Homo sapiens] | [18.0](http://blast.ncbi.nlm.nih.gov/Blast.cgi#239748112%23239748112) | 18.0 | 50% | 518 |
| [NP_001165911.1](http://www.ncbi.nlm.nih.gov/entrez/query.fcgi?cmd=Retrieve&db=Protein&list_uids=289063435&dopt=GenPept&RID=TFMFHDKY01S&log$=prottop&blast_rank=69) | endonuclease, polyU-specific isoform 3 precursor [Homo sapiens] | [18.0](http://blast.ncbi.nlm.nih.gov/Blast.cgi#289063435%23289063435) | 18.0 | 62% | 518 |
| [XP_001719139.1](http://www.ncbi.nlm.nih.gov/entrez/query.fcgi?cmd=Retrieve&db=Protein&list_uids=169218238&dopt=GenPept&RID=TFMFHDKY01S&log$=prottop&blast_rank=70) | PREDICTED: similar to ataxia telangiectasia and Rad3 related protein, partial [Homo sapiens] | [18.0](http://blast.ncbi.nlm.nih.gov/Blast.cgi#169218238%23169218238) | 18.0 | 62% | 518 |
| [NP_060024.2](http://www.ncbi.nlm.nih.gov/entrez/query.fcgi?cmd=Retrieve&db=Protein&list_uids=154813199&dopt=GenPept&RID=TFMFHDKY01S&log$=prottop&blast_rank=71) | poly [ADP-ribose] polymerase 14 [Homo sapiens] | [18.0](http://blast.ncbi.nlm.nih.gov/Blast.cgi#154813199%23154813199) | 18.0 | 50% | 518 |
| [NP_060453.3](http://www.ncbi.nlm.nih.gov/entrez/query.fcgi?cmd=Retrieve&db=Protein&list_uids=157388939&dopt=GenPept&RID=TFMFHDKY01S&log$=prottop&blast_rank=72) | WD repeat domain phosphoinositide-interacting protein 1 [Homo sapiens] | [18.0](http://blast.ncbi.nlm.nih.gov/Blast.cgi#157388939%23157388939) | 18.0 | 50% | 518 |
| [NP_001165910.1](http://www.ncbi.nlm.nih.gov/entrez/query.fcgi?cmd=Retrieve&db=Protein&list_uids=289063433&dopt=GenPept&RID=TFMFHDKY01S&log$=prottop&blast_rank=73) | endonuclease, polyU-specific isoform 1 precursor [Homo sapiens] | [18.0](http://blast.ncbi.nlm.nih.gov/Blast.cgi#289063433%23289063433) | 18.0 | 62% | 518 |
| [NP_001837.2](http://www.ncbi.nlm.nih.gov/entrez/query.fcgi?cmd=Retrieve&db=Protein&list_uids=116256354&dopt=GenPept&RID=TFMFHDKY01S&log$=prottop&blast_rank=74) | alpha 2 type IV collagen preproprotein [Homo sapiens] | [18.0](http://blast.ncbi.nlm.nih.gov/Blast.cgi#116256354%23116256354) | 18.0 | 50% | 518 |
| [NP_000080.2](http://www.ncbi.nlm.nih.gov/entrez/query.fcgi?cmd=Retrieve&db=Protein&list_uids=48762934&dopt=GenPept&RID=TFMFHDKY01S&log$=prottop&blast_rank=75) | alpha 2 type I collagen precursor [Homo sapiens] | [18.0](http://blast.ncbi.nlm.nih.gov/Blast.cgi#48762934%2348762934) | 18.0 | 62% | 518 |
| [NP_056169.1](http://www.ncbi.nlm.nih.gov/entrez/query.fcgi?cmd=Retrieve&db=Protein&list_uids=62955803&dopt=GenPept&RID=TFMFHDKY01S&log$=prottop&blast_rank=76) | nucleoporin NUP188 homolog [Homo sapiens] | [18.0](http://blast.ncbi.nlm.nih.gov/Blast.cgi#62955803%2362955803) | 18.0 | 50% | 518 |
| [NP_001096121.1](http://www.ncbi.nlm.nih.gov/entrez/query.fcgi?cmd=Retrieve&db=Protein&list_uids=156627557&dopt=GenPept&RID=TFMFHDKY01S&log$=prottop&blast_rank=77) | zinc finger protein 554 [Homo sapiens] | [18.0](http://blast.ncbi.nlm.nih.gov/Blast.cgi#156627557%23156627557) | 18.0 | 62% | 518 |
| [NP_036307.2](http://www.ncbi.nlm.nih.gov/entrez/query.fcgi?cmd=Retrieve&db=Protein&list_uids=15812186&dopt=GenPept&RID=TFMFHDKY01S&log$=prottop&blast_rank=78) | F-box only protein 3 isoform 1 [Homo sapiens] | [18.0](http://blast.ncbi.nlm.nih.gov/Blast.cgi#15812186%2315812186) | 18.0 | 62% | 518 |
| [NP_114441.1](http://www.ncbi.nlm.nih.gov/entrez/query.fcgi?cmd=Retrieve&db=Protein&list_uids=14670366&dopt=GenPept&RID=TFMFHDKY01S&log$=prottop&blast_rank=79) | POZ-, AT hook-, and zinc finger-containing protein 1 long B isoform [Homo sapiens] | [18.0](http://blast.ncbi.nlm.nih.gov/Blast.cgi#14670366%2314670366) | 18.0 | 75% | 518 |
| [NP_114440.1](http://www.ncbi.nlm.nih.gov/entrez/query.fcgi?cmd=Retrieve&db=Protein&list_uids=14670364&dopt=GenPept&RID=TFMFHDKY01S&log$=prottop&blast_rank=80) | POZ-, AT hook-, and zinc finger-containing protein 1 short isoform [Homo sapiens] | [18.0](http://blast.ncbi.nlm.nih.gov/Blast.cgi#14670364%2314670364) | 18.0 | 75% | 518 |
| [NP_848535.1](http://www.ncbi.nlm.nih.gov/entrez/query.fcgi?cmd=Retrieve&db=Protein&list_uids=30795182&dopt=GenPept&RID=TFMFHDKY01S&log$=prottop&blast_rank=81) | zinc finger FYVE domain-containing protein 1 isoform 2 [Homo sapiens] | [18.0](http://blast.ncbi.nlm.nih.gov/Blast.cgi#30795182%2330795182) | 18.0 | 50% | 518 |
| [NP_208385.1](http://www.ncbi.nlm.nih.gov/entrez/query.fcgi?cmd=Retrieve&db=Protein&list_uids=15812188&dopt=GenPept&RID=TFMFHDKY01S&log$=prottop&blast_rank=82) | F-box only protein 3 isoform 2 [Homo sapiens] | [18.0](http://blast.ncbi.nlm.nih.gov/Blast.cgi#15812188%2315812188) | 18.0 | 62% | 518 |
| [NP_000889.3](http://www.ncbi.nlm.nih.gov/entrez/query.fcgi?cmd=Retrieve&db=Protein&list_uids=38202207&dopt=GenPept&RID=TFMFHDKY01S&log$=prottop&blast_rank=83) | amine oxidase [flavin-containing] B [Homo sapiens] | [18.0](http://blast.ncbi.nlm.nih.gov/Blast.cgi#38202207%2338202207) | 18.0 | 50% | 518 |
| [NP_000065.1](http://www.ncbi.nlm.nih.gov/entrez/query.fcgi?cmd=Retrieve&db=Protein&list_uids=4557433&dopt=GenPept&RID=TFMFHDKY01S&log$=prottop&blast_rank=84) | CD40 ligand [Homo sapiens] | [18.0](http://blast.ncbi.nlm.nih.gov/Blast.cgi#4557433%234557433) | 18.0 | 75% | 518 |
| [NP_003782.1](http://www.ncbi.nlm.nih.gov/entrez/query.fcgi?cmd=Retrieve&db=Protein&list_uids=4506775&dopt=GenPept&RID=TFMFHDKY01S&log$=prottop&blast_rank=85) | membrane-bound transcription factor site-1 protease preproprotein [Homo sapiens] | [18.0](http://blast.ncbi.nlm.nih.gov/Blast.cgi#4506775%234506775) | 18.0 | 50% | 518 |
| [NP_060095.2](http://www.ncbi.nlm.nih.gov/entrez/query.fcgi?cmd=Retrieve&db=Protein&list_uids=31542986&dopt=GenPept&RID=TFMFHDKY01S&log$=prottop&blast_rank=86) | intelectin-1 precursor [Homo sapiens] | [18.0](http://blast.ncbi.nlm.nih.gov/Blast.cgi#31542986%2331542986) | 18.0 | 50% | 518 |
| [NP_114439.1](http://www.ncbi.nlm.nih.gov/entrez/query.fcgi?cmd=Retrieve&db=Protein&list_uids=14670362&dopt=GenPept&RID=TFMFHDKY01S&log$=prottop&blast_rank=87) | POZ-, AT hook-, and zinc finger-containing protein 1 long A isoform [Homo sapiens] | [18.0](http://blast.ncbi.nlm.nih.gov/Blast.cgi#14670362%2314670362) | 18.0 | 75% | 518 |
| [NP_055138.2](http://www.ncbi.nlm.nih.gov/entrez/query.fcgi?cmd=Retrieve&db=Protein&list_uids=14670360&dopt=GenPept&RID=TFMFHDKY01S&log$=prottop&blast_rank=88) | POZ-, AT hook-, and zinc finger-containing protein 1 long C isoform [Homo sapiens] | [18.0](http://blast.ncbi.nlm.nih.gov/Blast.cgi#14670360%2314670360) | 18.0 | 75% | 518 |
| [NP_004164.2](http://www.ncbi.nlm.nih.gov/entrez/query.fcgi?cmd=Retrieve&db=Protein&list_uids=110347453&dopt=GenPept&RID=TFMFHDKY01S&log$=prottop&blast_rank=89) | cationic amino acid transporter 4 [Homo sapiens] | [18.0](http://blast.ncbi.nlm.nih.gov/Blast.cgi#110347453%23110347453) | 18.0 | 50% | 518 |
| [NP_067083.1](http://www.ncbi.nlm.nih.gov/entrez/query.fcgi?cmd=Retrieve&db=Protein&list_uids=30795180&dopt=GenPept&RID=TFMFHDKY01S&log$=prottop&blast_rank=90) | zinc finger FYVE domain-containing protein 1 isoform 1 [Homo sapiens] | [18.0](http://blast.ncbi.nlm.nih.gov/Blast.cgi#30795180%2330795180) | 18.0 | 50% | 518 |
| [NP_000231.1](http://www.ncbi.nlm.nih.gov/entrez/query.fcgi?cmd=Retrieve&db=Protein&list_uids=4557735&dopt=GenPept&RID=TFMFHDKY01S&log$=prottop&blast_rank=91) | amine oxidase [flavin-containing] A [Homo sapiens] | [18.0](http://blast.ncbi.nlm.nih.gov/Blast.cgi#4557735%234557735) | 18.0 | 50% | 518 |
| [NP_055041.1](http://www.ncbi.nlm.nih.gov/entrez/query.fcgi?cmd=Retrieve&db=Protein&list_uids=7657498&dopt=GenPept&RID=TFMFHDKY01S&log$=prottop&blast_rank=92) | MAPK/MAK/MRK overlapping kinase [Homo sapiens] | [18.0](http://blast.ncbi.nlm.nih.gov/Blast.cgi#7657498%237657498) | 18.0 | 62% | 518 |
| [NP_006016.1](http://www.ncbi.nlm.nih.gov/entrez/query.fcgi?cmd=Retrieve&db=Protein&list_uids=5174623&dopt=GenPept&RID=TFMFHDKY01S&log$=prottop&blast_rank=93) | endonuclease, polyU-specific isoform 2 precursor [Homo sapiens] | [18.0](http://blast.ncbi.nlm.nih.gov/Blast.cgi#5174623%235174623) | 18.0 | 62% | 518 |
| [NP_001175.2](http://www.ncbi.nlm.nih.gov/entrez/query.fcgi?cmd=Retrieve&db=Protein&list_uids=157266317&dopt=GenPept&RID=TFMFHDKY01S&log$=prottop&blast_rank=94) | serine/threonine-protein kinase ATR [Homo sapiens] | [18.0](http://blast.ncbi.nlm.nih.gov/Blast.cgi#157266317%23157266317) | 18.0 | 62% | 518 |
| [NP_003595.1](http://www.ncbi.nlm.nih.gov/entrez/query.fcgi?cmd=Retrieve&db=Protein&list_uids=4504733&dopt=GenPept&RID=TFMFHDKY01S&log$=prottop&blast_rank=95) | insulin receptor substrate 4 [Homo sapiens] | [18.0](http://blast.ncbi.nlm.nih.gov/Blast.cgi#4504733%234504733) | 18.0 | 75% | 518 |
| [NP_783161.1](http://www.ncbi.nlm.nih.gov/entrez/query.fcgi?cmd=Retrieve&db=Protein&list_uids=28416956&dopt=GenPept&RID=TFMFHDKY01S&log$=prottop&blast_rank=96) | GTPase IMAP family member 8 [Homo sapiens] | [18.0](http://blast.ncbi.nlm.nih.gov/Blast.cgi#28416956%2328416956) | 18.0 | 50% | 518 |
| [XP_002346332.1](http://www.ncbi.nlm.nih.gov/entrez/query.fcgi?cmd=Retrieve&db=Protein&list_uids=239757765&dopt=GenPept&RID=TFMFHDKY01S&log$=prottop&blast_rank=97) | PREDICTED: similar to thioredoxin peroxidase [Homo sapiens] | [17.6](http://blast.ncbi.nlm.nih.gov/Blast.cgi#239757765%23239757765) | 17.6 | 87% | 695 |
| [NP_056160.2](http://www.ncbi.nlm.nih.gov/entrez/query.fcgi?cmd=Retrieve&db=Protein&list_uids=40548415&dopt=GenPept&RID=TFMFHDKY01S&log$=prottop&blast_rank=98) | disheveled-associated activator of morphogenesis 2 [Homo sapiens] | [17.6](http://blast.ncbi.nlm.nih.gov/Blast.cgi#40548415%2340548415) | 17.6 | 75% | 695 |
| [NP_001026866.1](http://www.ncbi.nlm.nih.gov/entrez/query.fcgi?cmd=Retrieve&db=Protein&list_uids=72534684&dopt=GenPept&RID=TFMFHDKY01S&log$=prottop&blast_rank=99) | phospholipase D3 [Homo sapiens] >ref|NP_036400.2| phospholipase D3 [Homo sapiens] | [17.6](http://blast.ncbi.nlm.nih.gov/Blast.cgi#72534684%2372534684) | 17.6 | 75% | 695 |
| [NP_001077.2](http://www.ncbi.nlm.nih.gov/entrez/query.fcgi?cmd=Retrieve&db=Protein&list_uids=68299767&dopt=GenPept&RID=TFMFHDKY01S&log$=prottop&blast_rank=100) | arylacetamide deacetylase [Homo sapiens] | [17.6](http://blast.ncbi.nlm.nih.gov/Blast.cgi#68299767%2368299767) | 17.6 | 75% | 695 |

| **Accession** | **Proteins with a match to TNGVHHGR peptide** | **[Max score](http://blast.ncbi.nlm.nih.gov/Blast.cgi?CMD=Get&ALIGNMENTS=100&ALIGNMENT_VIEW=Pairwise&CDD_SEARCH_STATE=1&DATABASE_SORT=0&DESCRIPTIONS=100&ENTREZ_QUERY=txid9606 %5BORGN%5D&FIRST_QUERY_NUM=0&FORMAT_OBJECT=Alignment&FORMAT_PAGE_TARGET=&FORMAT_TYPE=HTML&GET_SEQUENCE=yes&I_THRESH=&MASK_CHAR=2&MASK_COLOR=1&NEW_VIEW=yes&NUM_OVERVIEW=100&OLD_BLAST=false&PAGE=Proteins&QUERY_INDEX=0&QUERY_NUMBER=0&RESULTS_PAGE_TARGET=&RID=TFMM7SSM01S&SHOW_LINKOUT=yes&SHOW_OVERVIEW=yes&STEP_NUMBER=&WORD_SIZE=2&DISPLAY_SORT=1&HSP_SORT=1" \l "sort_mark)** | **[Total score](http://blast.ncbi.nlm.nih.gov/Blast.cgi?CMD=Get&ALIGNMENTS=100&ALIGNMENT_VIEW=Pairwise&CDD_SEARCH_STATE=1&DATABASE_SORT=0&DESCRIPTIONS=100&ENTREZ_QUERY=txid9606 %5BORGN%5D&FIRST_QUERY_NUM=0&FORMAT_OBJECT=Alignment&FORMAT_PAGE_TARGET=&FORMAT_TYPE=HTML&GET_SEQUENCE=yes&I_THRESH=&MASK_CHAR=2&MASK_COLOR=1&NEW_VIEW=yes&NUM_OVERVIEW=100&OLD_BLAST=false&PAGE=Proteins&QUERY_INDEX=0&QUERY_NUMBER=0&RESULTS_PAGE_TARGET=&RID=TFMM7SSM01S&SHOW_LINKOUT=yes&SHOW_OVERVIEW=yes&STEP_NUMBER=&WORD_SIZE=2&DISPLAY_SORT=2&HSP_SORT=1" \l "sort_mark)** | **[Query coverage](http://blast.ncbi.nlm.nih.gov/Blast.cgi?CMD=Get&ALIGNMENTS=100&ALIGNMENT_VIEW=Pairwise&CDD_SEARCH_STATE=1&DATABASE_SORT=0&DESCRIPTIONS=100&ENTREZ_QUERY=txid9606 %5BORGN%5D&FIRST_QUERY_NUM=0&FORMAT_OBJECT=Alignment&FORMAT_PAGE_TARGET=&FORMAT_TYPE=HTML&GET_SEQUENCE=yes&I_THRESH=&MASK_CHAR=2&MASK_COLOR=1&NEW_VIEW=yes&NUM_OVERVIEW=100&OLD_BLAST=false&PAGE=Proteins&QUERY_INDEX=0&QUERY_NUMBER=0&RESULTS_PAGE_TARGET=&RID=TFMM7SSM01S&SHOW_LINKOUT=yes&SHOW_OVERVIEW=yes&STEP_NUMBER=&WORD_SIZE=2&DISPLAY_SORT=4&HSP_SORT=0" \l "sort_mark)** | **[E value](http://blast.ncbi.nlm.nih.gov/Blast.cgi?CMD=Get&ALIGNMENTS=100&ALIGNMENT_VIEW=Pairwise&CDD_SEARCH_STATE=1&DATABASE_SORT=0&DESCRIPTIONS=100&ENTREZ_QUERY=txid9606 %5BORGN%5D&FIRST_QUERY_NUM=0&FORMAT_OBJECT=Alignment&FORMAT_PAGE_TARGET=&FORMAT_TYPE=HTML&GET_SEQUENCE=yes&I_THRESH=&MASK_CHAR=2&MASK_COLOR=1&NEW_VIEW=yes&NUM_OVERVIEW=100&OLD_BLAST=false&PAGE=Proteins&QUERY_INDEX=0&QUERY_NUMBER=0&RESULTS_PAGE_TARGET=&RID=TFMM7SSM01S&SHOW_LINKOUT=yes&SHOW_OVERVIEW=yes&STEP_NUMBER=&WORD_SIZE=2&DISPLAY_SORT=0&HSP_SORT=0" \l "sort_mark)** |
| --- | --- | --- | --- | --- | --- |
| [NP_116166.7](http://www.ncbi.nlm.nih.gov/entrez/query.fcgi?cmd=Retrieve&db=Protein&list_uids=194272183&dopt=GenPept&RID=TFMM7SSM01S&log$=prottop&blast_rank=1) | probable G-protein coupled receptor 124 precursor [Homo sapiens] | [21.8](http://blast.ncbi.nlm.nih.gov/Blast.cgi#194272183%23194272183) | 21.8 | 87% | 37 |
| [NP_001159817.1](http://www.ncbi.nlm.nih.gov/entrez/query.fcgi?cmd=Retrieve&db=Protein&list_uids=261599042&dopt=GenPept&RID=TFMM7SSM01S&log$=prottop&blast_rank=2) | myoD family inhibitor domain-containing protein isoform p32 [Homo sapiens] | [20.2](http://blast.ncbi.nlm.nih.gov/Blast.cgi#261599042%23261599042) | 20.2 | 75% | 119 |
| [NP_951038.1](http://www.ncbi.nlm.nih.gov/entrez/query.fcgi?cmd=Retrieve&db=Protein&list_uids=40018612&dopt=GenPept&RID=TFMM7SSM01S&log$=prottop&blast_rank=3) | myoD family inhibitor domain-containing protein isoform p40 [Homo sapiens] | [20.2](http://blast.ncbi.nlm.nih.gov/Blast.cgi#40018612%2340018612) | 20.2 | 75% | 119 |
| [NP_001159580.1](http://www.ncbi.nlm.nih.gov/entrez/query.fcgi?cmd=Retrieve&db=Protein&list_uids=260656030&dopt=GenPept&RID=TFMM7SSM01S&log$=prottop&blast_rank=4) | palladin isoform 1 [Homo sapiens] | [19.7](http://blast.ncbi.nlm.nih.gov/Blast.cgi#260656030%23260656030) | 35.6 | 100% | 160 |
| [NP_001159581.1](http://www.ncbi.nlm.nih.gov/entrez/query.fcgi?cmd=Retrieve&db=Protein&list_uids=260656032&dopt=GenPept&RID=TFMM7SSM01S&log$=prottop&blast_rank=5) | palladin isoform 3 [Homo sapiens] | [19.7](http://blast.ncbi.nlm.nih.gov/Blast.cgi#260656032%23260656032) | 35.6 | 100% | 160 |
| [NP_057165.3](http://www.ncbi.nlm.nih.gov/entrez/query.fcgi?cmd=Retrieve&db=Protein&list_uids=93102361&dopt=GenPept&RID=TFMM7SSM01S&log$=prottop&blast_rank=6) | palladin isoform 2 [Homo sapiens] | [19.7](http://blast.ncbi.nlm.nih.gov/Blast.cgi#93102361%2393102361) | 35.6 | 100% | 160 |
| [NP_000043.3](http://www.ncbi.nlm.nih.gov/entrez/query.fcgi?cmd=Retrieve&db=Protein&list_uids=115529486&dopt=GenPept&RID=TFMM7SSM01S&log$=prottop&blast_rank=7) | ATPase, Cu++ transporting, alpha polypeptide [Homo sapiens] | [19.7](http://blast.ncbi.nlm.nih.gov/Blast.cgi#115529486%23115529486) | 19.7 | 62% | 160 |
| [NP_001004320.1](http://www.ncbi.nlm.nih.gov/entrez/query.fcgi?cmd=Retrieve&db=Protein&list_uids=51972212&dopt=GenPept&RID=TFMM7SSM01S&log$=prottop&blast_rank=8) | transmembrane protein 195 [Homo sapiens] | [19.7](http://blast.ncbi.nlm.nih.gov/Blast.cgi#51972212%2351972212) | 19.7 | 62% | 160 |
| [NP_001159506.1](http://www.ncbi.nlm.nih.gov/entrez/query.fcgi?cmd=Retrieve&db=Protein&list_uids=260436922&dopt=GenPept&RID=TFMM7SSM01S&log$=prottop&blast_rank=9) | suprabasin isoform 1 precursor [Homo sapiens] | [18.9](http://blast.ncbi.nlm.nih.gov/Blast.cgi#260436922%23260436922) | 187 | 62% | 288 |
| [XP_002345432.1](http://www.ncbi.nlm.nih.gov/entrez/query.fcgi?cmd=Retrieve&db=Protein&list_uids=239757414&dopt=GenPept&RID=TFMM7SSM01S&log$=prottop&blast_rank=10) | PREDICTED: hypothetical protein XP_002345432 [Homo sapiens] | [18.9](http://blast.ncbi.nlm.nih.gov/Blast.cgi#239757414%23239757414) | 18.9 | 75% | 288 |
| [XP_002344217.1](http://www.ncbi.nlm.nih.gov/entrez/query.fcgi?cmd=Retrieve&db=Protein&list_uids=239746415&dopt=GenPept&RID=TFMM7SSM01S&log$=prottop&blast_rank=11) | PREDICTED: hypothetical protein XP_002344217 [Homo sapiens] >ref|XP_002348054.1| PREDICTED: hypothetical protein [Homo sapiens] | [18.9](http://blast.ncbi.nlm.nih.gov/Blast.cgi#239746415%23239746415) | 18.9 | 75% | 288 |
| [XP_002342945.1](http://www.ncbi.nlm.nih.gov/entrez/query.fcgi?cmd=Retrieve&db=Protein&list_uids=239743682&dopt=GenPept&RID=TFMM7SSM01S&log$=prottop&blast_rank=12) | PREDICTED: hypothetical protein XP_002342945 [Homo sapiens] >ref|XP_002347079.1| PREDICTED: hypothetical protein XP_002347079 [Homo sapiens] >ref|XP_002346199.1| PREDICTED: hypothetical protein XP_002346199 [Homo sapiens] | [18.9](http://blast.ncbi.nlm.nih.gov/Blast.cgi#239743682%23239743682) | 18.9 | 62% | 288 |
| [NP_689957.3](http://www.ncbi.nlm.nih.gov/entrez/query.fcgi?cmd=Retrieve&db=Protein&list_uids=119220552&dopt=GenPept&RID=TFMM7SSM01S&log$=prottop&blast_rank=13) | protein sidekick-1 precursor [Homo sapiens] | [18.9](http://blast.ncbi.nlm.nih.gov/Blast.cgi#119220552%23119220552) | 18.9 | 62% | 288 |
| [NP_061867.1](http://www.ncbi.nlm.nih.gov/entrez/query.fcgi?cmd=Retrieve&db=Protein&list_uids=62955044&dopt=GenPept&RID=TFMM7SSM01S&log$=prottop&blast_rank=14) | F-box only protein 42 [Homo sapiens] | [18.9](http://blast.ncbi.nlm.nih.gov/Blast.cgi#62955044%2362955044) | 18.9 | 62% | 288 |
| [NP_006070.2](http://www.ncbi.nlm.nih.gov/entrez/query.fcgi?cmd=Retrieve&db=Protein&list_uids=19923343&dopt=GenPept&RID=TFMM7SSM01S&log$=prottop&blast_rank=15) | cbp/p300-interacting transactivator 2 [Homo sapiens] >ref|NP_001161860.1| cbp/p300-interacting transactivator 2 [Homo sapiens] >ref|NP_001161861.1| cbp/p300-interacting transactivator 2 [Homo sapiens] | [18.9](http://blast.ncbi.nlm.nih.gov/Blast.cgi#19923343%2319923343) | 18.9 | 75% | 288 |
| [NP_705835.2](http://www.ncbi.nlm.nih.gov/entrez/query.fcgi?cmd=Retrieve&db=Protein&list_uids=270309176&dopt=GenPept&RID=TFMM7SSM01S&log$=prottop&blast_rank=16) | luman-recruiting factor isoform 1 [Homo sapiens] | [18.5](http://blast.ncbi.nlm.nih.gov/Blast.cgi#270309176%23270309176) | 18.5 | 75% | 386 |
| [NP_000154.1](http://www.ncbi.nlm.nih.gov/entrez/query.fcgi?cmd=Retrieve&db=Protein&list_uids=4503993&dopt=GenPept&RID=TFMM7SSM01S&log$=prottop&blast_rank=17) | growth hormone receptor precursor [Homo sapiens] | [18.5](http://blast.ncbi.nlm.nih.gov/Blast.cgi#4503993%234503993) | 18.5 | 87% | 386 |
| [NP_001006659.1](http://www.ncbi.nlm.nih.gov/entrez/query.fcgi?cmd=Retrieve&db=Protein&list_uids=54792123&dopt=GenPept&RID=TFMM7SSM01S&log$=prottop&blast_rank=18) | complement component (3d/Epstein Barr virus) receptor 2 isoform 1 precursor [Homo sapiens] | [18.5](http://blast.ncbi.nlm.nih.gov/Blast.cgi#54792123%2354792123) | 18.5 | 75% | 386 |
| [NP_001098714.1](http://www.ncbi.nlm.nih.gov/entrez/query.fcgi?cmd=Retrieve&db=Protein&list_uids=157419152&dopt=GenPept&RID=TFMM7SSM01S&log$=prottop&blast_rank=19) | protein tyrosine phosphatase, receptor type, M isoform 1 precursor [Homo sapiens] | [18.0](http://blast.ncbi.nlm.nih.gov/Blast.cgi#157419152%23157419152) | 18.0 | 75% | 518 |
| [NP_002836.3](http://www.ncbi.nlm.nih.gov/entrez/query.fcgi?cmd=Retrieve&db=Protein&list_uids=157419150&dopt=GenPept&RID=TFMM7SSM01S&log$=prottop&blast_rank=20) | protein tyrosine phosphatase, receptor type, M isoform 2 precursor [Homo sapiens] | [18.0](http://blast.ncbi.nlm.nih.gov/Blast.cgi#157419150%23157419150) | 18.0 | 75% | 518 |
| [NP_005535.1](http://www.ncbi.nlm.nih.gov/entrez/query.fcgi?cmd=Retrieve&db=Protein&list_uids=5031805&dopt=GenPept&RID=TFMM7SSM01S&log$=prottop&blast_rank=21) | insulin receptor substrate 1 [Homo sapiens] | [18.0](http://blast.ncbi.nlm.nih.gov/Blast.cgi#5031805%235031805) | 18.0 | 75% | 518 |
| [XP_002343000.1](http://www.ncbi.nlm.nih.gov/entrez/query.fcgi?cmd=Retrieve&db=Protein&list_uids=239743894&dopt=GenPept&RID=TFMM7SSM01S&log$=prottop&blast_rank=22) | PREDICTED: hypothetical protein XP_002343000 [Homo sapiens] >ref|XP_002347106.1| PREDICTED: hypothetical protein XP_002347106 [Homo sapiens] >ref|XP_002346217.1| PREDICTED: hypothetical protein XP_002346217 [Homo sapiens] | [17.6](http://blast.ncbi.nlm.nih.gov/Blast.cgi#239743894%23239743894) | 17.6 | 75% | 695 |
| [NP_835228.2](http://www.ncbi.nlm.nih.gov/entrez/query.fcgi?cmd=Retrieve&db=Protein&list_uids=187960047&dopt=GenPept&RID=TFMM7SSM01S&log$=prottop&blast_rank=23) | angiopoietin-related protein 5 precursor [Homo sapiens] | [17.6](http://blast.ncbi.nlm.nih.gov/Blast.cgi#187960047%23187960047) | 17.6 | 62% | 695 |
| [NP_006647.3](http://www.ncbi.nlm.nih.gov/entrez/query.fcgi?cmd=Retrieve&db=Protein&list_uids=117190519&dopt=GenPept&RID=TFMM7SSM01S&log$=prottop&blast_rank=24) | sialidase-3 [Homo sapiens] | [17.6](http://blast.ncbi.nlm.nih.gov/Blast.cgi#117190519%23117190519) | 17.6 | 75% | 695 |
| [NP_061163.2](http://www.ncbi.nlm.nih.gov/entrez/query.fcgi?cmd=Retrieve&db=Protein&list_uids=30089924&dopt=GenPept&RID=TFMM7SSM01S&log$=prottop&blast_rank=25) | F-box only protein 11 isoform 2 [Homo sapiens] | [17.6](http://blast.ncbi.nlm.nih.gov/Blast.cgi#30089924%2330089924) | 44.3 | 87% | 695 |
| [NP_036299.1](http://www.ncbi.nlm.nih.gov/entrez/query.fcgi?cmd=Retrieve&db=Protein&list_uids=30089922&dopt=GenPept&RID=TFMM7SSM01S&log$=prottop&blast_rank=26) | F-box only protein 11 isoform 3 [Homo sapiens] | [17.6](http://blast.ncbi.nlm.nih.gov/Blast.cgi#30089922%2330089922) | 31.8 | 62% | 695 |
| [NP_001018099.1](http://www.ncbi.nlm.nih.gov/entrez/query.fcgi?cmd=Retrieve&db=Protein&list_uids=66346704&dopt=GenPept&RID=TFMM7SSM01S&log$=prottop&blast_rank=27) | NMDA receptor-regulated protein 2 isoform b [Homo sapiens] | [17.6](http://blast.ncbi.nlm.nih.gov/Blast.cgi#66346704%2366346704) | 17.6 | 62% | 695 |
| [NP_849144.2](http://www.ncbi.nlm.nih.gov/entrez/query.fcgi?cmd=Retrieve&db=Protein&list_uids=38490688&dopt=GenPept&RID=TFMM7SSM01S&log$=prottop&blast_rank=28) | immunoglobulin superfamily member 10 precursor [Homo sapiens] | [17.6](http://blast.ncbi.nlm.nih.gov/Blast.cgi#38490688%2338490688) | 31.4 | 62% | 695 |
| [NP_078887.2](http://www.ncbi.nlm.nih.gov/entrez/query.fcgi?cmd=Retrieve&db=Protein&list_uids=37202123&dopt=GenPept&RID=TFMM7SSM01S&log$=prottop&blast_rank=29) | NMDA receptor-regulated protein 2 isoform a [Homo sapiens] | [17.6](http://blast.ncbi.nlm.nih.gov/Blast.cgi#37202123%2337202123) | 17.6 | 62% | 695 |
| [NP_005236.2](http://www.ncbi.nlm.nih.gov/entrez/query.fcgi?cmd=Retrieve&db=Protein&list_uids=66346693&dopt=GenPept&RID=TFMM7SSM01S&log$=prottop&blast_rank=30) | protocadherin Fat 1 precursor [Homo sapiens] | [17.6](http://blast.ncbi.nlm.nih.gov/Blast.cgi#66346693%2366346693) | 33.9 | 62% | 695 |
| [NP_079409.3](http://www.ncbi.nlm.nih.gov/entrez/query.fcgi?cmd=Retrieve&db=Protein&list_uids=30089926&dopt=GenPept&RID=TFMM7SSM01S&log$=prottop&blast_rank=31) | F-box only protein 11 isoform 1 [Homo sapiens] | [17.6](http://blast.ncbi.nlm.nih.gov/Blast.cgi#30089926%2330089926) | 44.3 | 87% | 695 |
| [NP_001208.2](http://www.ncbi.nlm.nih.gov/entrez/query.fcgi?cmd=Retrieve&db=Protein&list_uids=9951923&dopt=GenPept&RID=TFMM7SSM01S&log$=prottop&blast_rank=32) | carbonic anhydrase XI precursor [Homo sapiens] | [17.6](http://blast.ncbi.nlm.nih.gov/Blast.cgi#9951923%239951923) | 17.6 | 87% | 695 |
| [NP_079021.3](http://www.ncbi.nlm.nih.gov/entrez/query.fcgi?cmd=Retrieve&db=Protein&list_uids=190358504&dopt=GenPept&RID=TFMM7SSM01S&log$=prottop&blast_rank=33) | SHC SH2 domain-binding protein 1 [Homo sapiens] | [17.6](http://blast.ncbi.nlm.nih.gov/Blast.cgi#190358504%23190358504) | 17.6 | 62% | 695 |
| [NP_001161707.1](http://www.ncbi.nlm.nih.gov/entrez/query.fcgi?cmd=Retrieve&db=Protein&list_uids=270265871&dopt=GenPept&RID=TFMM7SSM01S&log$=prottop&blast_rank=34) | FRAS1-related extracellular matrix protein 3 precursor [Homo sapiens] | [17.2](http://blast.ncbi.nlm.nih.gov/Blast.cgi#270265871%23270265871) | 28.4 | 75% | 932 |
| [NP_660337.3](http://www.ncbi.nlm.nih.gov/entrez/query.fcgi?cmd=Retrieve&db=Protein&list_uids=94400919&dopt=GenPept&RID=TFMM7SSM01S&log$=prottop&blast_rank=35) | WD repeat-containing protein 90 [Homo sapiens] | [17.2](http://blast.ncbi.nlm.nih.gov/Blast.cgi#94400919%2394400919) | 17.2 | 75% | 932 |
| [NP_065166.2](http://www.ncbi.nlm.nih.gov/entrez/query.fcgi?cmd=Retrieve&db=Protein&list_uids=21704281&dopt=GenPept&RID=TFMM7SSM01S&log$=prottop&blast_rank=36) | junctophilin-2 isoform 1 [Homo sapiens] | [17.2](http://blast.ncbi.nlm.nih.gov/Blast.cgi#21704281%2321704281) | 17.2 | 87% | 932 |
| [NP_057105.2](http://www.ncbi.nlm.nih.gov/entrez/query.fcgi?cmd=Retrieve&db=Protein&list_uids=37577122&dopt=GenPept&RID=TFMM7SSM01S&log$=prottop&blast_rank=37) | ubiquitin-conjugating enzyme E2 J1 [Homo sapiens] | [17.2](http://blast.ncbi.nlm.nih.gov/Blast.cgi#37577122%2337577122) | 17.2 | 75% | 932 |
| [NP_079437.5](http://www.ncbi.nlm.nih.gov/entrez/query.fcgi?cmd=Retrieve&db=Protein&list_uids=289547536&dopt=GenPept&RID=TFMM7SSM01S&log$=prottop&blast_rank=38) | fanconi anemia-associated protein of 100 kDa isoform b [Homo sapiens] | [16.8](http://blast.ncbi.nlm.nih.gov/Blast.cgi#289547536%23289547536) | 16.8 | 50% | 1251 |
| [XP_002345907.1](http://www.ncbi.nlm.nih.gov/entrez/query.fcgi?cmd=Retrieve&db=Protein&list_uids=239754195&dopt=GenPept&RID=TFMM7SSM01S&log$=prottop&blast_rank=39) | PREDICTED: hypothetical protein [Homo sapiens] | [16.8](http://blast.ncbi.nlm.nih.gov/Blast.cgi#239754195%23239754195) | 33.1 | 75% | 1251 |
| [XP_002348035.1](http://www.ncbi.nlm.nih.gov/entrez/query.fcgi?cmd=Retrieve&db=Protein&list_uids=239751877&dopt=GenPept&RID=TFMM7SSM01S&log$=prottop&blast_rank=40) | PREDICTED: hypothetical protein XP_002348035 [Homo sapiens] | [16.8](http://blast.ncbi.nlm.nih.gov/Blast.cgi#239751877%23239751877) | 16.8 | 50% | 1251 |
| [XP_002347848.1](http://www.ncbi.nlm.nih.gov/entrez/query.fcgi?cmd=Retrieve&db=Protein&list_uids=239751487&dopt=GenPept&RID=TFMM7SSM01S&log$=prottop&blast_rank=41) | PREDICTED: hypothetical protein XP_002347848 [Homo sapiens] >ref|XP_002345129.1| PREDICTED: hypothetical protein XP_002345129 [Homo sapiens] | [16.8](http://blast.ncbi.nlm.nih.gov/Blast.cgi#239751487%23239751487) | 16.8 | 75% | 1251 |
| [XP_002346783.1](http://www.ncbi.nlm.nih.gov/entrez/query.fcgi?cmd=Retrieve&db=Protein&list_uids=239748723&dopt=GenPept&RID=TFMM7SSM01S&log$=prottop&blast_rank=42) | PREDICTED: hypothetical protein XP_002346783 [Homo sapiens] | [16.8](http://blast.ncbi.nlm.nih.gov/Blast.cgi#239748723%23239748723) | 33.1 | 75% | 1251 |
| [XP_002346764.1](http://www.ncbi.nlm.nih.gov/entrez/query.fcgi?cmd=Retrieve&db=Protein&list_uids=239748683&dopt=GenPept&RID=TFMM7SSM01S&log$=prottop&blast_rank=43) | PREDICTED: hypothetical protein XP_002346764 [Homo sapiens] >ref|XP_002345895.1| PREDICTED: hypothetical protein XP_002345895 [Homo sapiens] | [16.8](http://blast.ncbi.nlm.nih.gov/Blast.cgi#239748683%23239748683) | 16.8 | 50% | 1251 |
| [XP_002346501.1](http://www.ncbi.nlm.nih.gov/entrez/query.fcgi?cmd=Retrieve&db=Protein&list_uids=239748046&dopt=GenPept&RID=TFMM7SSM01S&log$=prottop&blast_rank=44) | PREDICTED: hypothetical protein XP_002346501 [Homo sapiens] | [16.8](http://blast.ncbi.nlm.nih.gov/Blast.cgi#239748046%23239748046) | 16.8 | 75% | 1251 |
| [XP_002343607.1](http://www.ncbi.nlm.nih.gov/entrez/query.fcgi?cmd=Retrieve&db=Protein&list_uids=239745867&dopt=GenPept&RID=TFMM7SSM01S&log$=prottop&blast_rank=45) | PREDICTED: hypothetical protein XP_002343607 [Homo sapiens] >ref|XP_002347829.1| PREDICTED: hypothetical protein XP_002347829 [Homo sapiens] >ref|XP_002345093.1| PREDICTED: hypothetical protein [Homo sapiens] | [16.8](http://blast.ncbi.nlm.nih.gov/Blast.cgi#239745867%23239745867) | 16.8 | 50% | 1251 |
| [XP_002342618.1](http://www.ncbi.nlm.nih.gov/entrez/query.fcgi?cmd=Retrieve&db=Protein&list_uids=239742758&dopt=GenPept&RID=TFMM7SSM01S&log$=prottop&blast_rank=46) | PREDICTED: hypothetical protein XP_002342618 [Homo sapiens] | [16.8](http://blast.ncbi.nlm.nih.gov/Blast.cgi#239742758%23239742758) | 33.1 | 75% | 1251 |
| [XP_002342607.1](http://www.ncbi.nlm.nih.gov/entrez/query.fcgi?cmd=Retrieve&db=Protein&list_uids=239742706&dopt=GenPept&RID=TFMM7SSM01S&log$=prottop&blast_rank=47) | PREDICTED: hypothetical protein XP_002342607 [Homo sapiens] | [16.8](http://blast.ncbi.nlm.nih.gov/Blast.cgi#239742706%23239742706) | 16.8 | 50% | 1251 |
| [NP_001153509.1](http://www.ncbi.nlm.nih.gov/entrez/query.fcgi?cmd=Retrieve&db=Protein&list_uids=237649111&dopt=GenPept&RID=TFMM7SSM01S&log$=prottop&blast_rank=48) | rho-related BTB domain-containing protein 2 isoform 2 [Homo sapiens] | [16.8](http://blast.ncbi.nlm.nih.gov/Blast.cgi#237649111%23237649111) | 16.8 | 50% | 1251 |
| [NP_001153508.1](http://www.ncbi.nlm.nih.gov/entrez/query.fcgi?cmd=Retrieve&db=Protein&list_uids=237649109&dopt=GenPept&RID=TFMM7SSM01S&log$=prottop&blast_rank=49) | rho-related BTB domain-containing protein 2 isoform 1 [Homo sapiens] | [16.8](http://blast.ncbi.nlm.nih.gov/Blast.cgi#237649109%23237649109) | 16.8 | 50% | 1251 |
| [NP_001139147.1](http://www.ncbi.nlm.nih.gov/entrez/query.fcgi?cmd=Retrieve&db=Protein&list_uids=224586855&dopt=GenPept&RID=TFMM7SSM01S&log$=prottop&blast_rank=50) | sorbin and SH3 domain-containing protein 2 isoform 8 [Homo sapiens] | [16.8](http://blast.ncbi.nlm.nih.gov/Blast.cgi#224586855%23224586855) | 16.8 | 50% | 1251 |
| [NP_001139145.1](http://www.ncbi.nlm.nih.gov/entrez/query.fcgi?cmd=Retrieve&db=Protein&list_uids=224586851&dopt=GenPept&RID=TFMM7SSM01S&log$=prottop&blast_rank=51) | sorbin and SH3 domain-containing protein 2 isoform 6 [Homo sapiens] | [16.8](http://blast.ncbi.nlm.nih.gov/Blast.cgi#224586851%23224586851) | 16.8 | 50% | 1251 |
| [NP_001139144.1](http://www.ncbi.nlm.nih.gov/entrez/query.fcgi?cmd=Retrieve&db=Protein&list_uids=224586849&dopt=GenPept&RID=TFMM7SSM01S&log$=prottop&blast_rank=52) | sorbin and SH3 domain-containing protein 2 isoform 5 [Homo sapiens] | [16.8](http://blast.ncbi.nlm.nih.gov/Blast.cgi#224586849%23224586849) | 16.8 | 50% | 1251 |
| [NP_001139142.1](http://www.ncbi.nlm.nih.gov/entrez/query.fcgi?cmd=Retrieve&db=Protein&list_uids=224586844&dopt=GenPept&RID=TFMM7SSM01S&log$=prottop&blast_rank=53) | sorbin and SH3 domain-containing protein 2 isoform 3 [Homo sapiens] | [16.8](http://blast.ncbi.nlm.nih.gov/Blast.cgi#224586844%23224586844) | 16.8 | 50% | 1251 |
| [NP_444282.3](http://www.ncbi.nlm.nih.gov/entrez/query.fcgi?cmd=Retrieve&db=Protein&list_uids=221316609&dopt=GenPept&RID=TFMM7SSM01S&log$=prottop&blast_rank=54) | cation channel sperm-associated protein 1 [Homo sapiens] | [16.8](http://blast.ncbi.nlm.nih.gov/Blast.cgi#221316609%23221316609) | 33.5 | 50% | 1251 |
| [NP_001139146.1](http://www.ncbi.nlm.nih.gov/entrez/query.fcgi?cmd=Retrieve&db=Protein&list_uids=224586853&dopt=GenPept&RID=TFMM7SSM01S&log$=prottop&blast_rank=55) | sorbin and SH3 domain-containing protein 2 isoform 7 [Homo sapiens] | [16.8](http://blast.ncbi.nlm.nih.gov/Blast.cgi#224586853%23224586853) | 16.8 | 50% | 1251 |
| [NP_056052.2](http://www.ncbi.nlm.nih.gov/entrez/query.fcgi?cmd=Retrieve&db=Protein&list_uids=197245440&dopt=GenPept&RID=TFMM7SSM01S&log$=prottop&blast_rank=56) | hypothetical protein LOC23285 [Homo sapiens] | [16.8](http://blast.ncbi.nlm.nih.gov/Blast.cgi#197245440%23197245440) | 16.8 | 50% | 1251 |
| [NP_001139143.1](http://www.ncbi.nlm.nih.gov/entrez/query.fcgi?cmd=Retrieve&db=Protein&list_uids=224586846&dopt=GenPept&RID=TFMM7SSM01S&log$=prottop&blast_rank=57) | sorbin and SH3 domain-containing protein 2 isoform 4 [Homo sapiens] | [16.8](http://blast.ncbi.nlm.nih.gov/Blast.cgi#224586846%23224586846) | 16.8 | 50% | 1251 |
| [NP_001162.4](http://www.ncbi.nlm.nih.gov/entrez/query.fcgi?cmd=Retrieve&db=Protein&list_uids=190343023&dopt=GenPept&RID=TFMM7SSM01S&log$=prottop&blast_rank=58) | multidrug resistance-associated protein 6 isoform 1 [Homo sapiens] | [16.8](http://blast.ncbi.nlm.nih.gov/Blast.cgi#190343023%23190343023) | 16.8 | 50% | 1251 |
| [XP_001718549.1](http://www.ncbi.nlm.nih.gov/entrez/query.fcgi?cmd=Retrieve&db=Protein&list_uids=169161633&dopt=GenPept&RID=TFMM7SSM01S&log$=prottop&blast_rank=59) | PREDICTED: hypothetical protein [Homo sapiens] | [16.8](http://blast.ncbi.nlm.nih.gov/Blast.cgi#169161633%23169161633) | 16.8 | 50% | 1251 |
| [XP_001718594.1](http://www.ncbi.nlm.nih.gov/entrez/query.fcgi?cmd=Retrieve&db=Protein&list_uids=169161340&dopt=GenPept&RID=TFMM7SSM01S&log$=prottop&blast_rank=60) | PREDICTED: hypothetical protein [Homo sapiens] | [16.8](http://blast.ncbi.nlm.nih.gov/Blast.cgi#169161340%23169161340) | 16.8 | 50% | 1251 |
| [NP_055699.2](http://www.ncbi.nlm.nih.gov/entrez/query.fcgi?cmd=Retrieve&db=Protein&list_uids=224282117&dopt=GenPept&RID=TFMM7SSM01S&log$=prottop&blast_rank=61) | putative splicing factor, arginine/serine-rich 14 [Homo sapiens] >ref|NP_001017392.2| putative splicing factor, arginine/serine-rich 14 [Homo sapiens] | [16.8](http://blast.ncbi.nlm.nih.gov/Blast.cgi#224282117%23224282117) | 16.8 | 50% | 1251 |
| [NP_689576.4](http://www.ncbi.nlm.nih.gov/entrez/query.fcgi?cmd=Retrieve&db=Protein&list_uids=168229256&dopt=GenPept&RID=TFMM7SSM01S&log$=prottop&blast_rank=62) | ankyrin repeat and LEM domain-containing protein 1 [Homo sapiens] | [16.8](http://blast.ncbi.nlm.nih.gov/Blast.cgi#168229256%23168229256) | 16.8 | 50% | 1251 |
| [NP_085095.1](http://www.ncbi.nlm.nih.gov/entrez/query.fcgi?cmd=Retrieve&db=Protein&list_uids=13699836&dopt=GenPept&RID=TFMM7SSM01S&log$=prottop&blast_rank=63) | matrilin 4 isoform 3 precursor [Homo sapiens] | [16.8](http://blast.ncbi.nlm.nih.gov/Blast.cgi#13699836%2313699836) | 16.8 | 75% | 1251 |
| [NP_005766.3](http://www.ncbi.nlm.nih.gov/entrez/query.fcgi?cmd=Retrieve&db=Protein&list_uids=155030230&dopt=GenPept&RID=TFMM7SSM01S&log$=prottop&blast_rank=64) | sorbin and SH3 domain containing 3 isoform 1 [Homo sapiens] | [16.8](http://blast.ncbi.nlm.nih.gov/Blast.cgi#155030230%23155030230) | 16.8 | 50% | 1251 |
| [NP_003594.3](http://www.ncbi.nlm.nih.gov/entrez/query.fcgi?cmd=Retrieve&db=Protein&list_uids=194733749&dopt=GenPept&RID=TFMM7SSM01S&log$=prottop&blast_rank=65) | sorbin and SH3 domain-containing protein 2 isoform 1 [Homo sapiens] | [16.8](http://blast.ncbi.nlm.nih.gov/Blast.cgi#194733749%23194733749) | 16.8 | 50% | 1251 |
| [NP_001120864.1](http://www.ncbi.nlm.nih.gov/entrez/query.fcgi?cmd=Retrieve&db=Protein&list_uids=188528652&dopt=GenPept&RID=TFMM7SSM01S&log$=prottop&blast_rank=66) | myelin gene regulatory factor isoform 2 [Homo sapiens] | [16.8](http://blast.ncbi.nlm.nih.gov/Blast.cgi#188528652%23188528652) | 33.1 | 75% | 1251 |
| [NP_115479.3](http://www.ncbi.nlm.nih.gov/entrez/query.fcgi?cmd=Retrieve&db=Protein&list_uids=117938330&dopt=GenPept&RID=TFMM7SSM01S&log$=prottop&blast_rank=67) | lethal(3)malignant brain tumor-like protein isoform II [Homo sapiens] | [16.8](http://blast.ncbi.nlm.nih.gov/Blast.cgi#117938330%23117938330) | 16.8 | 50% | 1251 |
| [NP_203754.2](http://www.ncbi.nlm.nih.gov/entrez/query.fcgi?cmd=Retrieve&db=Protein&list_uids=110556636&dopt=GenPept&RID=TFMM7SSM01S&log$=prottop&blast_rank=68) | 182 kDa tankyrase-1-binding protein [Homo sapiens] | [16.8](http://blast.ncbi.nlm.nih.gov/Blast.cgi#110556636%23110556636) | 16.8 | 50% | 1251 |
| [NP_001030127.1](http://www.ncbi.nlm.nih.gov/entrez/query.fcgi?cmd=Retrieve&db=Protein&list_uids=78000165&dopt=GenPept&RID=TFMM7SSM01S&log$=prottop&blast_rank=69) | sorbin and SH3 domain-containing protein 1 isoform 4 [Homo sapiens] | [16.8](http://blast.ncbi.nlm.nih.gov/Blast.cgi#78000165%2378000165) | 16.8 | 50% | 1251 |
| [NP_001030129.1](http://www.ncbi.nlm.nih.gov/entrez/query.fcgi?cmd=Retrieve&db=Protein&list_uids=78000171&dopt=GenPept&RID=TFMM7SSM01S&log$=prottop&blast_rank=70) | sorbin and SH3 domain-containing protein 1 isoform 7 [Homo sapiens] | [16.8](http://blast.ncbi.nlm.nih.gov/Blast.cgi#78000171%2378000171) | 16.8 | 50% | 1251 |
| [NP_006425.2](http://www.ncbi.nlm.nih.gov/entrez/query.fcgi?cmd=Retrieve&db=Protein&list_uids=78000160&dopt=GenPept&RID=TFMM7SSM01S&log$=prottop&blast_rank=71) | sorbin and SH3 domain-containing protein 1 isoform 1 [Homo sapiens] | [16.8](http://blast.ncbi.nlm.nih.gov/Blast.cgi#78000160%2378000160) | 16.8 | 50% | 1251 |
| [NP_079267.1](http://www.ncbi.nlm.nih.gov/entrez/query.fcgi?cmd=Retrieve&db=Protein&list_uids=78000169&dopt=GenPept&RID=TFMM7SSM01S&log$=prottop&blast_rank=72) | sorbin and SH3 domain-containing protein 1 isoform 6 [Homo sapiens] | [16.8](http://blast.ncbi.nlm.nih.gov/Blast.cgi#78000169%2378000169) | 16.8 | 50% | 1251 |
| [NP_001030126.1](http://www.ncbi.nlm.nih.gov/entrez/query.fcgi?cmd=Retrieve&db=Protein&list_uids=78000163&dopt=GenPept&RID=TFMM7SSM01S&log$=prottop&blast_rank=73) | sorbin and SH3 domain-containing protein 1 isoform 3 [Homo sapiens] | [16.8](http://blast.ncbi.nlm.nih.gov/Blast.cgi#78000163%2378000163) | 16.8 | 50% | 1251 |
| [NP_937784.2](http://www.ncbi.nlm.nih.gov/entrez/query.fcgi?cmd=Retrieve&db=Protein&list_uids=66346737&dopt=GenPept&RID=TFMM7SSM01S&log$=prottop&blast_rank=74) | glucosidase, alpha; neutral C [Homo sapiens] | [16.8](http://blast.ncbi.nlm.nih.gov/Blast.cgi#66346737%2366346737) | 16.8 | 75% | 1251 |
| [NP_001009608.1](http://www.ncbi.nlm.nih.gov/entrez/query.fcgi?cmd=Retrieve&db=Protein&list_uids=61102723&dopt=GenPept&RID=TFMM7SSM01S&log$=prottop&blast_rank=75) | hypothetical protein LOC128710 [Homo sapiens] | [16.8](http://blast.ncbi.nlm.nih.gov/Blast.cgi#61102723%2361102723) | 16.8 | 50% | 1251 |
| [NP_001121189.1](http://www.ncbi.nlm.nih.gov/entrez/query.fcgi?cmd=Retrieve&db=Protein&list_uids=189217425&dopt=GenPept&RID=TFMM7SSM01S&log$=prottop&blast_rank=76) | laminin subunit alpha-3 isoform 3 [Homo sapiens] | [16.8](http://blast.ncbi.nlm.nih.gov/Blast.cgi#189217425%23189217425) | 16.8 | 50% | 1251 |
| [NP_937762.1](http://www.ncbi.nlm.nih.gov/entrez/query.fcgi?cmd=Retrieve&db=Protein&list_uids=38045910&dopt=GenPept&RID=TFMM7SSM01S&log$=prottop&blast_rank=77) | laminin subunit alpha-3 isoform 1 [Homo sapiens] | [16.8](http://blast.ncbi.nlm.nih.gov/Blast.cgi#38045910%2338045910) | 16.8 | 50% | 1251 |
| [NP_001001665.3](http://www.ncbi.nlm.nih.gov/entrez/query.fcgi?cmd=Retrieve&db=Protein&list_uids=167466231&dopt=GenPept&RID=TFMM7SSM01S&log$=prottop&blast_rank=78) | cytochrome P450, family 27, subfamily C, polypeptide 1 [Homo sapiens] | [16.8](http://blast.ncbi.nlm.nih.gov/Blast.cgi#167466231%23167466231) | 16.8 | 87% | 1251 |
| [NP_066547.1](http://www.ncbi.nlm.nih.gov/entrez/query.fcgi?cmd=Retrieve&db=Protein&list_uids=10947118&dopt=GenPept&RID=TFMM7SSM01S&log$=prottop&blast_rank=79) | sorbin and SH3 domain-containing protein 2 isoform 2 [Homo sapiens] | [16.8](http://blast.ncbi.nlm.nih.gov/Blast.cgi#10947118%2310947118) | 16.8 | 50% | 1251 |
| [NP_006742.2](http://www.ncbi.nlm.nih.gov/entrez/query.fcgi?cmd=Retrieve&db=Protein&list_uids=28557788&dopt=GenPept&RID=TFMM7SSM01S&log$=prottop&blast_rank=80) | sperm-specific antigen 2 isoform 2 [Homo sapiens] | [16.8](http://blast.ncbi.nlm.nih.gov/Blast.cgi#28557788%2328557788) | 16.8 | 50% | 1251 |
| [NP_003824.2](http://www.ncbi.nlm.nih.gov/entrez/query.fcgi?cmd=Retrieve&db=Protein&list_uids=13699830&dopt=GenPept&RID=TFMM7SSM01S&log$=prottop&blast_rank=81) | matrilin 4 isoform 1 precursor [Homo sapiens] | [16.8](http://blast.ncbi.nlm.nih.gov/Blast.cgi#13699830%2313699830) | 16.8 | 75% | 1251 |
| [NP_001810.2](http://www.ncbi.nlm.nih.gov/entrez/query.fcgi?cmd=Retrieve&db=Protein&list_uids=221316599&dopt=GenPept&RID=TFMM7SSM01S&log$=prottop&blast_rank=82) | chromogranin B precursor [Homo sapiens] | [16.8](http://blast.ncbi.nlm.nih.gov/Blast.cgi#221316599%23221316599) | 16.8 | 50% | 1251 |
| [NP_001018003.1](http://www.ncbi.nlm.nih.gov/entrez/query.fcgi?cmd=Retrieve&db=Protein&list_uids=65301112&dopt=GenPept&RID=TFMM7SSM01S&log$=prottop&blast_rank=83) | sorbin and SH3 domain containing 3 isoform 2 [Homo sapiens] | [16.8](http://blast.ncbi.nlm.nih.gov/Blast.cgi#65301112%2365301112) | 16.8 | 50% | 1251 |
| [NP_542775.2](http://www.ncbi.nlm.nih.gov/entrez/query.fcgi?cmd=Retrieve&db=Protein&list_uids=193804860&dopt=GenPept&RID=TFMM7SSM01S&log$=prottop&blast_rank=84) | synaptotagmin-like protein 4 [Homo sapiens] >ref|NP_001123368.1| synaptotagmin-like protein 4 [Homo sapiens] | [16.8](http://blast.ncbi.nlm.nih.gov/Blast.cgi#193804860%23193804860) | 16.8 | 50% | 1251 |
| [NP_085080.1](http://www.ncbi.nlm.nih.gov/entrez/query.fcgi?cmd=Retrieve&db=Protein&list_uids=13699834&dopt=GenPept&RID=TFMM7SSM01S&log$=prottop&blast_rank=85) | matrilin 4 isoform 2 precursor [Homo sapiens] | [16.8](http://blast.ncbi.nlm.nih.gov/Blast.cgi#13699834%2313699834) | 16.8 | 75% | 1251 |
| [NP_001073893.1](http://www.ncbi.nlm.nih.gov/entrez/query.fcgi?cmd=Retrieve&db=Protein&list_uids=122937251&dopt=GenPept&RID=TFMM7SSM01S&log$=prottop&blast_rank=86) | lysine-specific demethylase 6B [Homo sapiens] | [16.8](http://blast.ncbi.nlm.nih.gov/Blast.cgi#122937251%23122937251) | 16.8 | 50% | 1251 |
| [NP_000426.2](http://www.ncbi.nlm.nih.gov/entrez/query.fcgi?cmd=Retrieve&db=Protein&list_uids=134244285&dopt=GenPept&RID=TFMM7SSM01S&log$=prottop&blast_rank=87) | Notch homolog 3 precursor [Homo sapiens] | [16.8](http://blast.ncbi.nlm.nih.gov/Blast.cgi#134244285%23134244285) | 16.8 | 50% | 1251 |
| [NP_001072996.1](http://www.ncbi.nlm.nih.gov/entrez/query.fcgi?cmd=Retrieve&db=Protein&list_uids=118582251&dopt=GenPept&RID=TFMM7SSM01S&log$=prottop&blast_rank=88) | multidrug resistance-associated protein 6 isoform 2 [Homo sapiens] | [16.8](http://blast.ncbi.nlm.nih.gov/Blast.cgi#118582251%23118582251) | 16.8 | 50% | 1251 |
| [NP_037411.1](http://www.ncbi.nlm.nih.gov/entrez/query.fcgi?cmd=Retrieve&db=Protein&list_uids=7019335&dopt=GenPept&RID=TFMM7SSM01S&log$=prottop&blast_rank=89) | myelin gene regulatory factor isoform 1 [Homo sapiens] | [16.8](http://blast.ncbi.nlm.nih.gov/Blast.cgi#7019335%237019335) | 33.1 | 75% | 1251 |
| [NP_003120.2](http://www.ncbi.nlm.nih.gov/entrez/query.fcgi?cmd=Retrieve&db=Protein&list_uids=62865635&dopt=GenPept&RID=TFMM7SSM01S&log$=prottop&blast_rank=90) | squalene monooxygenase [Homo sapiens] | [16.8](http://blast.ncbi.nlm.nih.gov/Blast.cgi#62865635%2362865635) | 16.8 | 50% | 1251 |
| [NP_803172.1](http://www.ncbi.nlm.nih.gov/entrez/query.fcgi?cmd=Retrieve&db=Protein&list_uids=29171753&dopt=GenPept&RID=TFMM7SSM01S&log$=prottop&blast_rank=91) | liprin-alpha-1 isoform a [Homo sapiens] | [16.8](http://blast.ncbi.nlm.nih.gov/Blast.cgi#29171753%2329171753) | 16.8 | 50% | 1251 |
| [NP_001030128.1](http://www.ncbi.nlm.nih.gov/entrez/query.fcgi?cmd=Retrieve&db=Protein&list_uids=78000167&dopt=GenPept&RID=TFMM7SSM01S&log$=prottop&blast_rank=92) | sorbin and SH3 domain-containing protein 1 isoform 5 [Homo sapiens] | [16.8](http://blast.ncbi.nlm.nih.gov/Blast.cgi#78000167%2378000167) | 16.8 | 50% | 1251 |
| [NP_060043.2](http://www.ncbi.nlm.nih.gov/entrez/query.fcgi?cmd=Retrieve&db=Protein&list_uids=76443679&dopt=GenPept&RID=TFMM7SSM01S&log$=prottop&blast_rank=93) | proprotein convertase subtilisin/kexin type 4 precursor [Homo sapiens] | [16.8](http://blast.ncbi.nlm.nih.gov/Blast.cgi#76443679%2376443679) | 30.1 | 75% | 1251 |
| [NP_055868.1](http://www.ncbi.nlm.nih.gov/entrez/query.fcgi?cmd=Retrieve&db=Protein&list_uids=55769554&dopt=GenPept&RID=TFMM7SSM01S&log$=prottop&blast_rank=94) | liprin-alpha-4 [Homo sapiens] | [16.8](http://blast.ncbi.nlm.nih.gov/Blast.cgi#55769554%2355769554) | 16.8 | 50% | 1251 |
| [NP_001123917.1](http://www.ncbi.nlm.nih.gov/entrez/query.fcgi?cmd=Retrieve&db=Protein&list_uids=194363771&dopt=GenPept&RID=TFMM7SSM01S&log$=prottop&blast_rank=95) | sperm-specific antigen 2 isoform 1 [Homo sapiens] | [16.8](http://blast.ncbi.nlm.nih.gov/Blast.cgi#194363771%23194363771) | 16.8 | 50% | 1251 |
| [NP_056175.3](http://www.ncbi.nlm.nih.gov/entrez/query.fcgi?cmd=Retrieve&db=Protein&list_uids=193211480&dopt=GenPept&RID=TFMM7SSM01S&log$=prottop&blast_rank=96) | superkiller viralicidic activity 2-like 2 [Homo sapiens] | [16.8](http://blast.ncbi.nlm.nih.gov/Blast.cgi#193211480%23193211480) | 16.8 | 62% | 1251 |
| [NP_003617.1](http://www.ncbi.nlm.nih.gov/entrez/query.fcgi?cmd=Retrieve&db=Protein&list_uids=4505983&dopt=GenPept&RID=TFMM7SSM01S&log$=prottop&blast_rank=97) | liprin-alpha-1 isoform b [Homo sapiens] | [16.8](http://blast.ncbi.nlm.nih.gov/Blast.cgi#4505983%234505983) | 16.8 | 50% | 1251 |
| [NP_008858.1](http://www.ncbi.nlm.nih.gov/entrez/query.fcgi?cmd=Retrieve&db=Protein&list_uids=5902082&dopt=GenPept&RID=TFMM7SSM01S&log$=prottop&blast_rank=98) | CMP-N-acetylneuraminate-beta-galactosamide-alpha-2,3-sialyltransferase 2 [Homo sapiens] | [16.8](http://blast.ncbi.nlm.nih.gov/Blast.cgi#5902082%235902082) | 16.8 | 50% | 1251 |
| [NP_872319.1](http://www.ncbi.nlm.nih.gov/entrez/query.fcgi?cmd=Retrieve&db=Protein&list_uids=32698866&dopt=GenPept&RID=TFMM7SSM01S&log$=prottop&blast_rank=99) | spindle pole body component 24 homolog [Homo sapiens] | [16.8](http://blast.ncbi.nlm.nih.gov/Blast.cgi#32698866%2332698866) | 16.8 | 62% | 1251 |
| [NP_005038.1](http://www.ncbi.nlm.nih.gov/entrez/query.fcgi?cmd=Retrieve&db=Protein&list_uids=4826952&dopt=GenPept&RID=TFMM7SSM01S&log$=prottop&blast_rank=100) | proteasome 26S non-ATPase subunit 5 [Homo sapiens] | [16.8](http://blast.ncbi.nlm.nih.gov/Blast.cgi#4826952%234826952) | 16.8 | 50% | 1251 |

| **Accession** | **Proteins with a match to GLYNFMGK peptide** | **[Max score](http://blast.ncbi.nlm.nih.gov/Blast.cgi?CMD=Get&ALIGNMENTS=100&ALIGNMENT_VIEW=Pairwise&CDD_SEARCH_STATE=2&DATABASE_SORT=0&DESCRIPTIONS=100&ENTREZ_QUERY=txid9606 %5BORGN%5D&FIRST_QUERY_NUM=0&FORMAT_OBJECT=Alignment&FORMAT_PAGE_TARGET=&FORMAT_TYPE=HTML&GET_SEQUENCE=yes&I_THRESH=&MASK_CHAR=2&MASK_COLOR=1&NEW_VIEW=yes&NUM_OVERVIEW=100&OLD_BLAST=false&PAGE=Proteins&QUERY_INDEX=0&QUERY_NUMBER=0&RESULTS_PAGE_TARGET=&RID=TFMTSW8D01N&SHOW_LINKOUT=yes&SHOW_OVERVIEW=yes&STEP_NUMBER=&WORD_SIZE=2&DISPLAY_SORT=1&HSP_SORT=1" \l "sort_mark)** | **[Total score](http://blast.ncbi.nlm.nih.gov/Blast.cgi?CMD=Get&ALIGNMENTS=100&ALIGNMENT_VIEW=Pairwise&CDD_SEARCH_STATE=2&DATABASE_SORT=0&DESCRIPTIONS=100&ENTREZ_QUERY=txid9606 %5BORGN%5D&FIRST_QUERY_NUM=0&FORMAT_OBJECT=Alignment&FORMAT_PAGE_TARGET=&FORMAT_TYPE=HTML&GET_SEQUENCE=yes&I_THRESH=&MASK_CHAR=2&MASK_COLOR=1&NEW_VIEW=yes&NUM_OVERVIEW=100&OLD_BLAST=false&PAGE=Proteins&QUERY_INDEX=0&QUERY_NUMBER=0&RESULTS_PAGE_TARGET=&RID=TFMTSW8D01N&SHOW_LINKOUT=yes&SHOW_OVERVIEW=yes&STEP_NUMBER=&WORD_SIZE=2&DISPLAY_SORT=2&HSP_SORT=1" \l "sort_mark)** | **[Query coverage](http://blast.ncbi.nlm.nih.gov/Blast.cgi?CMD=Get&ALIGNMENTS=100&ALIGNMENT_VIEW=Pairwise&CDD_SEARCH_STATE=2&DATABASE_SORT=0&DESCRIPTIONS=100&ENTREZ_QUERY=txid9606 %5BORGN%5D&FIRST_QUERY_NUM=0&FORMAT_OBJECT=Alignment&FORMAT_PAGE_TARGET=&FORMAT_TYPE=HTML&GET_SEQUENCE=yes&I_THRESH=&MASK_CHAR=2&MASK_COLOR=1&NEW_VIEW=yes&NUM_OVERVIEW=100&OLD_BLAST=false&PAGE=Proteins&QUERY_INDEX=0&QUERY_NUMBER=0&RESULTS_PAGE_TARGET=&RID=TFMTSW8D01N&SHOW_LINKOUT=yes&SHOW_OVERVIEW=yes&STEP_NUMBER=&WORD_SIZE=2&DISPLAY_SORT=4&HSP_SORT=0" \l "sort_mark)** | **[E value](http://blast.ncbi.nlm.nih.gov/Blast.cgi?CMD=Get&ALIGNMENTS=100&ALIGNMENT_VIEW=Pairwise&CDD_SEARCH_STATE=2&DATABASE_SORT=0&DESCRIPTIONS=100&ENTREZ_QUERY=txid9606 %5BORGN%5D&FIRST_QUERY_NUM=0&FORMAT_OBJECT=Alignment&FORMAT_PAGE_TARGET=&FORMAT_TYPE=HTML&GET_SEQUENCE=yes&I_THRESH=&MASK_CHAR=2&MASK_COLOR=1&NEW_VIEW=yes&NUM_OVERVIEW=100&OLD_BLAST=false&PAGE=Proteins&QUERY_INDEX=0&QUERY_NUMBER=0&RESULTS_PAGE_TARGET=&RID=TFMTSW8D01N&SHOW_LINKOUT=yes&SHOW_OVERVIEW=yes&STEP_NUMBER=&WORD_SIZE=2&DISPLAY_SORT=0&HSP_SORT=0" \l "sort_mark)** |
| --- | --- | --- | --- | --- | --- |
| [NP_075053.2](http://www.ncbi.nlm.nih.gov/entrez/query.fcgi?cmd=Retrieve&db=Protein&list_uids=20070323&dopt=GenPept&RID=TFMTSW8D01N&log$=prottop&blast_rank=1) | solute carrier family 30 (zinc transporter), member 5 isoform 1 [Homo sapiens] | [24.8](http://blast.ncbi.nlm.nih.gov/Blast.cgi#20070323%2320070323) | 24.8 | 75% | 4.7 |
| [NP_001108451.1](http://www.ncbi.nlm.nih.gov/entrez/query.fcgi?cmd=Retrieve&db=Protein&list_uids=169234659&dopt=GenPept&RID=TFMTSW8D01N&log$=prottop&blast_rank=2) | tumor protein 63 isoform 3 [Homo sapiens] | [22.3](http://blast.ncbi.nlm.nih.gov/Blast.cgi#169234659%23169234659) | 22.3 | 62% | 27 |
| [NP_001108454.1](http://www.ncbi.nlm.nih.gov/entrez/query.fcgi?cmd=Retrieve&db=Protein&list_uids=169234665&dopt=GenPept&RID=TFMTSW8D01N&log$=prottop&blast_rank=3) | tumor protein 63 isoform 6 [Homo sapiens] | [22.3](http://blast.ncbi.nlm.nih.gov/Blast.cgi#169234665%23169234665) | 22.3 | 62% | 27 |
| [NP_003713.3](http://www.ncbi.nlm.nih.gov/entrez/query.fcgi?cmd=Retrieve&db=Protein&list_uids=31543818&dopt=GenPept&RID=TFMTSW8D01N&log$=prottop&blast_rank=4) | tumor protein 63 isoform 1 [Homo sapiens] | [22.3](http://blast.ncbi.nlm.nih.gov/Blast.cgi#31543818%2331543818) | 22.3 | 62% | 27 |
| [NP_001108453.1](http://www.ncbi.nlm.nih.gov/entrez/query.fcgi?cmd=Retrieve&db=Protein&list_uids=169234663&dopt=GenPept&RID=TFMTSW8D01N&log$=prottop&blast_rank=5) | tumor protein 63 isoform 5 [Homo sapiens] | [22.3](http://blast.ncbi.nlm.nih.gov/Blast.cgi#169234663%23169234663) | 22.3 | 62% | 27 |
| [NP_001108450.1](http://www.ncbi.nlm.nih.gov/entrez/query.fcgi?cmd=Retrieve&db=Protein&list_uids=169234657&dopt=GenPept&RID=TFMTSW8D01N&log$=prottop&blast_rank=6) | tumor protein 63 isoform 2 [Homo sapiens] | [22.3](http://blast.ncbi.nlm.nih.gov/Blast.cgi#169234657%23169234657) | 22.3 | 62% | 27 |
| [NP_001108452.1](http://www.ncbi.nlm.nih.gov/entrez/query.fcgi?cmd=Retrieve&db=Protein&list_uids=169234661&dopt=GenPept&RID=TFMTSW8D01N&log$=prottop&blast_rank=7) | tumor protein 63 isoform 4 [Homo sapiens] | [22.3](http://blast.ncbi.nlm.nih.gov/Blast.cgi#169234661%23169234661) | 22.3 | 62% | 27 |
| [NP_001119714.1](http://www.ncbi.nlm.nih.gov/entrez/query.fcgi?cmd=Retrieve&db=Protein&list_uids=187829052&dopt=GenPept&RID=TFMTSW8D01N&log$=prottop&blast_rank=8) | tumor protein p73 isoform d [Homo sapiens] | [22.3](http://blast.ncbi.nlm.nih.gov/Blast.cgi#187829052%23187829052) | 22.3 | 62% | 27 |
| [NP_001119713.1](http://www.ncbi.nlm.nih.gov/entrez/query.fcgi?cmd=Retrieve&db=Protein&list_uids=187829017&dopt=GenPept&RID=TFMTSW8D01N&log$=prottop&blast_rank=9) | tumor protein p73 isoform c [Homo sapiens] | [22.3](http://blast.ncbi.nlm.nih.gov/Blast.cgi#187829017%23187829017) | 22.3 | 62% | 27 |
| [NP_001119712.1](http://www.ncbi.nlm.nih.gov/entrez/query.fcgi?cmd=Retrieve&db=Protein&list_uids=187828902&dopt=GenPept&RID=TFMTSW8D01N&log$=prottop&blast_rank=10) | tumor protein p73 isoform b [Homo sapiens] | [22.3](http://blast.ncbi.nlm.nih.gov/Blast.cgi#187828902%23187828902) | 22.3 | 62% | 27 |
| [NP_005418.1](http://www.ncbi.nlm.nih.gov/entrez/query.fcgi?cmd=Retrieve&db=Protein&list_uids=4885645&dopt=GenPept&RID=TFMTSW8D01N&log$=prottop&blast_rank=11) | tumor protein p73 isoform a [Homo sapiens] | [22.3](http://blast.ncbi.nlm.nih.gov/Blast.cgi#4885645%234885645) | 22.3 | 62% | 27 |
| [NP_060711.2](http://www.ncbi.nlm.nih.gov/entrez/query.fcgi?cmd=Retrieve&db=Protein&list_uids=190358512&dopt=GenPept&RID=TFMTSW8D01N&log$=prottop&blast_rank=12) | transmembrane protein 184C [Homo sapiens] | [21.8](http://blast.ncbi.nlm.nih.gov/Blast.cgi#190358512%23190358512) | 21.8 | 62% | 37 |
| [NP_060524.4](http://www.ncbi.nlm.nih.gov/entrez/query.fcgi?cmd=Retrieve&db=Protein&list_uids=54860105&dopt=GenPept&RID=TFMTSW8D01N&log$=prottop&blast_rank=13) | rho GTPase-activating protein 17 isoform 2 [Homo sapiens] | [21.8](http://blast.ncbi.nlm.nih.gov/Blast.cgi#54860105%2354860105) | 21.8 | 87% | 37 |
| [NP_001006635.1](http://www.ncbi.nlm.nih.gov/entrez/query.fcgi?cmd=Retrieve&db=Protein&list_uids=54860079&dopt=GenPept&RID=TFMTSW8D01N&log$=prottop&blast_rank=14) | rho GTPase-activating protein 17 isoform 1 [Homo sapiens] | [21.8](http://blast.ncbi.nlm.nih.gov/Blast.cgi#54860079%2354860079) | 21.8 | 87% | 37 |
| [NP_006436.3](http://www.ncbi.nlm.nih.gov/entrez/query.fcgi?cmd=Retrieve&db=Protein&list_uids=91208426&dopt=GenPept&RID=TFMTSW8D01N&log$=prottop&blast_rank=15) | pre-mRNA-processing-splicing factor 8 [Homo sapiens] | [21.8](http://blast.ncbi.nlm.nih.gov/Blast.cgi#91208426%2391208426) | 21.8 | 62% | 37 |
| [NP_001025029.2](http://www.ncbi.nlm.nih.gov/entrez/query.fcgi?cmd=Retrieve&db=Protein&list_uids=189027121&dopt=GenPept&RID=TFMTSW8D01N&log$=prottop&blast_rank=16) | solute carrier family 35 member F1 [Homo sapiens] | [21.4](http://blast.ncbi.nlm.nih.gov/Blast.cgi#189027121%23189027121) | 21.4 | 75% | 49 |
| [NP_110411.1](http://www.ncbi.nlm.nih.gov/entrez/query.fcgi?cmd=Retrieve&db=Protein&list_uids=13540557&dopt=GenPept&RID=TFMTSW8D01N&log$=prottop&blast_rank=17) | probable G-protein coupled receptor 63 [Homo sapiens] >ref|NP_001137429.1| probable G-protein coupled receptor 63 [Homo sapiens] | [21.4](http://blast.ncbi.nlm.nih.gov/Blast.cgi#13540557%2313540557) | 21.4 | 75% | 49 |
| [NP_005067.1](http://www.ncbi.nlm.nih.gov/entrez/query.fcgi?cmd=Retrieve&db=Protein&list_uids=4827022&dopt=GenPept&RID=TFMTSW8D01N&log$=prottop&blast_rank=18) | contactin 2 precursor [Homo sapiens] | [20.6](http://blast.ncbi.nlm.nih.gov/Blast.cgi#4827022%234827022) | 20.6 | 62% | 89 |
| [NP_065873.2](http://www.ncbi.nlm.nih.gov/entrez/query.fcgi?cmd=Retrieve&db=Protein&list_uids=240255505&dopt=GenPept&RID=TFMTSW8D01N&log$=prottop&blast_rank=19) | potassium channel, subfamily T, member 1 [Homo sapiens] | [20.2](http://blast.ncbi.nlm.nih.gov/Blast.cgi#240255505%23240255505) | 20.2 | 62% | 119 |
| [NP_940905.2](http://www.ncbi.nlm.nih.gov/entrez/query.fcgi?cmd=Retrieve&db=Protein&list_uids=41349443&dopt=GenPept&RID=TFMTSW8D01N&log$=prottop&blast_rank=20) | potassium channel subfamily T member 2 [Homo sapiens] | [20.2](http://blast.ncbi.nlm.nih.gov/Blast.cgi#41349443%2341349443) | 20.2 | 62% | 119 |
| [NP_000535.3](http://www.ncbi.nlm.nih.gov/entrez/query.fcgi?cmd=Retrieve&db=Protein&list_uids=73747915&dopt=GenPept&RID=TFMTSW8D01N&log$=prottop&blast_rank=21) | transporter 2, ATP-binding cassette, sub-family B isoform 1 [Homo sapiens] | [20.2](http://blast.ncbi.nlm.nih.gov/Blast.cgi#73747915%2373747915) | 20.2 | 75% | 119 |
| [NP_061313.2](http://www.ncbi.nlm.nih.gov/entrez/query.fcgi?cmd=Retrieve&db=Protein&list_uids=73747917&dopt=GenPept&RID=TFMTSW8D01N&log$=prottop&blast_rank=22) | transporter 2, ATP-binding cassette, sub-family B isoform 2 [Homo sapiens] | [20.2](http://blast.ncbi.nlm.nih.gov/Blast.cgi#73747917%2373747917) | 20.2 | 75% | 119 |
| [NP_000199.2](http://www.ncbi.nlm.nih.gov/entrez/query.fcgi?cmd=Retrieve&db=Protein&list_uids=119395736&dopt=GenPept&RID=TFMTSW8D01N&log$=prottop&blast_rank=23) | insulin receptor isoform Long precursor [Homo sapiens] | [19.7](http://blast.ncbi.nlm.nih.gov/Blast.cgi#119395736%23119395736) | 19.7 | 75% | 160 |
| [NP_001073285.1](http://www.ncbi.nlm.nih.gov/entrez/query.fcgi?cmd=Retrieve&db=Protein&list_uids=119395738&dopt=GenPept&RID=TFMTSW8D01N&log$=prottop&blast_rank=24) | insulin receptor isoform Short precursor [Homo sapiens] | [19.7](http://blast.ncbi.nlm.nih.gov/Blast.cgi#119395738%23119395738) | 19.7 | 75% | 160 |
| [NP_004981.2](http://www.ncbi.nlm.nih.gov/entrez/query.fcgi?cmd=Retrieve&db=Protein&list_uids=14043022&dopt=GenPept&RID=TFMTSW8D01N&log$=prottop&blast_rank=25) | methionyl-tRNA synthetase, cytoplasmic [Homo sapiens] | [19.7](http://blast.ncbi.nlm.nih.gov/Blast.cgi#14043022%2314043022) | 19.7 | 87% | 160 |
| [NP_001164632.1](http://www.ncbi.nlm.nih.gov/entrez/query.fcgi?cmd=Retrieve&db=Protein&list_uids=283837888&dopt=GenPept&RID=TFMTSW8D01N&log$=prottop&blast_rank=26) | sialic acid-binding Ig-like lectin 10 isoform 7 precursor [Homo sapiens] | [19.3](http://blast.ncbi.nlm.nih.gov/Blast.cgi#283837888%23283837888) | 19.3 | 50% | 214 |
| [NP_001164631.1](http://www.ncbi.nlm.nih.gov/entrez/query.fcgi?cmd=Retrieve&db=Protein&list_uids=283837886&dopt=GenPept&RID=TFMTSW8D01N&log$=prottop&blast_rank=27) | sialic acid-binding Ig-like lectin 10 isoform 6 precursor [Homo sapiens] | [19.3](http://blast.ncbi.nlm.nih.gov/Blast.cgi#283837886%23283837886) | 19.3 | 50% | 214 |
| [NP_001164629.1](http://www.ncbi.nlm.nih.gov/entrez/query.fcgi?cmd=Retrieve&db=Protein&list_uids=283837880&dopt=GenPept&RID=TFMTSW8D01N&log$=prottop&blast_rank=28) | sialic acid-binding Ig-like lectin 10 isoform 4 precursor [Homo sapiens] | [19.3](http://blast.ncbi.nlm.nih.gov/Blast.cgi#283837880%23283837880) | 19.3 | 50% | 214 |
| [NP_001164630.1](http://www.ncbi.nlm.nih.gov/entrez/query.fcgi?cmd=Retrieve&db=Protein&list_uids=283837882&dopt=GenPept&RID=TFMTSW8D01N&log$=prottop&blast_rank=29) | sialic acid-binding Ig-like lectin 10 isoform 5 precursor [Homo sapiens] | [19.3](http://blast.ncbi.nlm.nih.gov/Blast.cgi#283837882%23283837882) | 19.3 | 50% | 214 |
| [NP_001164627.1](http://www.ncbi.nlm.nih.gov/entrez/query.fcgi?cmd=Retrieve&db=Protein&list_uids=283837876&dopt=GenPept&RID=TFMTSW8D01N&log$=prottop&blast_rank=30) | sialic acid-binding Ig-like lectin 10 isoform 2 precursor [Homo sapiens] | [19.3](http://blast.ncbi.nlm.nih.gov/Blast.cgi#283837876%23283837876) | 19.3 | 50% | 214 |
| [NP_659412.3](http://www.ncbi.nlm.nih.gov/entrez/query.fcgi?cmd=Retrieve&db=Protein&list_uids=145580600&dopt=GenPept&RID=TFMTSW8D01N&log$=prottop&blast_rank=31) | schlafen family member 5 [Homo sapiens] | [19.3](http://blast.ncbi.nlm.nih.gov/Blast.cgi#145580600%23145580600) | 19.3 | 50% | 214 |
| [NP_149121.2](http://www.ncbi.nlm.nih.gov/entrez/query.fcgi?cmd=Retrieve&db=Protein&list_uids=31377639&dopt=GenPept&RID=TFMTSW8D01N&log$=prottop&blast_rank=32) | sialic acid-binding Ig-like lectin 10 isoform 1 precursor [Homo sapiens] | [19.3](http://blast.ncbi.nlm.nih.gov/Blast.cgi#31377639%2331377639) | 19.3 | 50% | 214 |
| [NP_001164628.1](http://www.ncbi.nlm.nih.gov/entrez/query.fcgi?cmd=Retrieve&db=Protein&list_uids=283837878&dopt=GenPept&RID=TFMTSW8D01N&log$=prottop&blast_rank=33) | sialic acid-binding Ig-like lectin 10 isoform 3 precursor [Homo sapiens] | [19.3](http://blast.ncbi.nlm.nih.gov/Blast.cgi#283837878%23283837878) | 19.3 | 50% | 214 |
| [NP_699185.1](http://www.ncbi.nlm.nih.gov/entrez/query.fcgi?cmd=Retrieve&db=Protein&list_uids=23503279&dopt=GenPept&RID=TFMTSW8D01N&log$=prottop&blast_rank=34) | transmembrane protein 161B [Homo sapiens] | [19.3](http://blast.ncbi.nlm.nih.gov/Blast.cgi#23503279%2323503279) | 19.3 | 50% | 214 |
| [NP_003584.2](http://www.ncbi.nlm.nih.gov/entrez/query.fcgi?cmd=Retrieve&db=Protein&list_uids=18201913&dopt=GenPept&RID=TFMTSW8D01N&log$=prottop&blast_rank=35) | forkhead box protein N1 [Homo sapiens] | [19.3](http://blast.ncbi.nlm.nih.gov/Blast.cgi#18201913%2318201913) | 19.3 | 50% | 214 |
| [NP_000566.3](http://www.ncbi.nlm.nih.gov/entrez/query.fcgi?cmd=Retrieve&db=Protein&list_uids=27894330&dopt=GenPept&RID=TFMTSW8D01N&log$=prottop&blast_rank=36) | interleukin-1 alpha proprotein [Homo sapiens] | [19.3](http://blast.ncbi.nlm.nih.gov/Blast.cgi#27894330%2327894330) | 19.3 | 50% | 214 |
| [NP_055052.1](http://www.ncbi.nlm.nih.gov/entrez/query.fcgi?cmd=Retrieve&db=Protein&list_uids=7656861&dopt=GenPept&RID=TFMTSW8D01N&log$=prottop&blast_rank=37) | a disintegrin and metalloprotease domain 18 preproprotein [Homo sapiens] | [19.3](http://blast.ncbi.nlm.nih.gov/Blast.cgi#7656861%237656861) | 19.3 | 75% | 214 |
| [NP_689801.1](http://www.ncbi.nlm.nih.gov/entrez/query.fcgi?cmd=Retrieve&db=Protein&list_uids=22749211&dopt=GenPept&RID=TFMTSW8D01N&log$=prottop&blast_rank=38) | transmembrane and tetratricopeptide repeat containing 2 [Homo sapiens] | [19.3](http://blast.ncbi.nlm.nih.gov/Blast.cgi#22749211%2322749211) | 19.3 | 75% | 214 |
| [NP_005871.1](http://www.ncbi.nlm.nih.gov/entrez/query.fcgi?cmd=Retrieve&db=Protein&list_uids=5031741&dopt=GenPept&RID=TFMTSW8D01N&log$=prottop&blast_rank=39) | dnaJ homolog subfamily A member 2 [Homo sapiens] | [19.3](http://blast.ncbi.nlm.nih.gov/Blast.cgi#5031741%235031741) | 19.3 | 87% | 214 |
| [NP_001364.1](http://www.ncbi.nlm.nih.gov/entrez/query.fcgi?cmd=Retrieve&db=Protein&list_uids=223555935&dopt=GenPept&RID=TFMTSW8D01N&log$=prottop&blast_rank=40) | dynein heavy chain 14, axonemal isoform 1 [Homo sapiens] | [18.9](http://blast.ncbi.nlm.nih.gov/Blast.cgi#223555935%23223555935) | 18.9 | 75% | 288 |
| [NP_078828.2](http://www.ncbi.nlm.nih.gov/entrez/query.fcgi?cmd=Retrieve&db=Protein&list_uids=205360977&dopt=GenPept&RID=TFMTSW8D01N&log$=prottop&blast_rank=41) | LAG1 homolog, ceramide synthase 4 [Homo sapiens] | [18.9](http://blast.ncbi.nlm.nih.gov/Blast.cgi#205360977%23205360977) | 18.9 | 62% | 288 |
| [XP_001719681.1](http://www.ncbi.nlm.nih.gov/entrez/query.fcgi?cmd=Retrieve&db=Protein&list_uids=169217711&dopt=GenPept&RID=TFMTSW8D01N&log$=prottop&blast_rank=42) | PREDICTED: similar to Ovostatin homolog 2 [Homo sapiens] | [18.9](http://blast.ncbi.nlm.nih.gov/Blast.cgi#169217711%23169217711) | 18.9 | 62% | 288 |
| [NP_056507.2](http://www.ncbi.nlm.nih.gov/entrez/query.fcgi?cmd=Retrieve&db=Protein&list_uids=118600977&dopt=GenPept&RID=TFMTSW8D01N&log$=prottop&blast_rank=43) | C3 and PZP-like alpha-2-macroglobulin domain-containing protein 8 [Homo sapiens] | [18.9](http://blast.ncbi.nlm.nih.gov/Blast.cgi#118600977%23118600977) | 18.9 | 62% | 288 |
| [NP_598014.1](http://www.ncbi.nlm.nih.gov/entrez/query.fcgi?cmd=Retrieve&db=Protein&list_uids=19743854&dopt=GenPept&RID=TFMTSW8D01N&log$=prottop&blast_rank=44) | decorin isoform e precursor [Homo sapiens] | [18.9](http://blast.ncbi.nlm.nih.gov/Blast.cgi#19743854%2319743854) | 18.9 | 75% | 288 |
| [NP_598013.1](http://www.ncbi.nlm.nih.gov/entrez/query.fcgi?cmd=Retrieve&db=Protein&list_uids=19743852&dopt=GenPept&RID=TFMTSW8D01N&log$=prottop&blast_rank=45) | decorin isoform d precursor [Homo sapiens] | [18.9](http://blast.ncbi.nlm.nih.gov/Blast.cgi#19743852%2319743852) | 18.9 | 75% | 288 |
| [NP_598012.1](http://www.ncbi.nlm.nih.gov/entrez/query.fcgi?cmd=Retrieve&db=Protein&list_uids=19743850&dopt=GenPept&RID=TFMTSW8D01N&log$=prottop&blast_rank=46) | decorin isoform c precursor [Homo sapiens] | [18.9](http://blast.ncbi.nlm.nih.gov/Blast.cgi#19743850%2319743850) | 18.9 | 75% | 288 |
| [NP_598011.1](http://www.ncbi.nlm.nih.gov/entrez/query.fcgi?cmd=Retrieve&db=Protein&list_uids=19743848&dopt=GenPept&RID=TFMTSW8D01N&log$=prottop&blast_rank=47) | decorin isoform b precursor [Homo sapiens] | [18.9](http://blast.ncbi.nlm.nih.gov/Blast.cgi#19743848%2319743848) | 18.9 | 75% | 288 |
| [NP_085915.2](http://www.ncbi.nlm.nih.gov/entrez/query.fcgi?cmd=Retrieve&db=Protein&list_uids=21361300&dopt=GenPept&RID=TFMTSW8D01N&log$=prottop&blast_rank=48) | lens intrinsic membrane protein 2, 19kDa isoform 1 [Homo sapiens] | [18.9](http://blast.ncbi.nlm.nih.gov/Blast.cgi#21361300%2321361300) | 18.9 | 75% | 288 |
| [NP_059985.2](http://www.ncbi.nlm.nih.gov/entrez/query.fcgi?cmd=Retrieve&db=Protein&list_uids=31542943&dopt=GenPept&RID=TFMTSW8D01N&log$=prottop&blast_rank=49) | solute carrier family 35 member F2 [Homo sapiens] | [18.9](http://blast.ncbi.nlm.nih.gov/Blast.cgi#31542943%2331542943) | 18.9 | 62% | 288 |
| [NP_055431.1](http://www.ncbi.nlm.nih.gov/entrez/query.fcgi?cmd=Retrieve&db=Protein&list_uids=62632750&dopt=GenPept&RID=TFMTSW8D01N&log$=prottop&blast_rank=50) | probable phospholipid-transporting ATPase IF [Homo sapiens] | [18.9](http://blast.ncbi.nlm.nih.gov/Blast.cgi#62632750%2362632750) | 36.5 | 75% | 288 |
| [NP_001155220.1](http://www.ncbi.nlm.nih.gov/entrez/query.fcgi?cmd=Retrieve&db=Protein&list_uids=239916002&dopt=GenPept&RID=TFMTSW8D01N&log$=prottop&blast_rank=51) | lens intrinsic membrane protein 2, 19kDa isoform 2 [Homo sapiens] | [18.9](http://blast.ncbi.nlm.nih.gov/Blast.cgi#239916002%23239916002) | 18.9 | 75% | 288 |
| [NP_001911.1](http://www.ncbi.nlm.nih.gov/entrez/query.fcgi?cmd=Retrieve&db=Protein&list_uids=4503271&dopt=GenPept&RID=TFMTSW8D01N&log$=prottop&blast_rank=52) | decorin isoform a preproprotein [Homo sapiens] >ref|NP_598010.1| decorin isoform a preproprotein [Homo sapiens] | [18.9](http://blast.ncbi.nlm.nih.gov/Blast.cgi#4503271%234503271) | 18.9 | 75% | 288 |
| [NP_001938.2](http://www.ncbi.nlm.nih.gov/entrez/query.fcgi?cmd=Retrieve&db=Protein&list_uids=238231453&dopt=GenPept&RID=TFMTSW8D01N&log$=prottop&blast_rank=53) | dual specificity protein phosphatase 7 [Homo sapiens] | [18.5](http://blast.ncbi.nlm.nih.gov/Blast.cgi#238231453%23238231453) | 18.5 | 62% | 386 |
| [NP_001138472.1](http://www.ncbi.nlm.nih.gov/entrez/query.fcgi?cmd=Retrieve&db=Protein&list_uids=223468597&dopt=GenPept&RID=TFMTSW8D01N&log$=prottop&blast_rank=54) | integrin alpha-V isoform 3 precursor [Homo sapiens] | [18.5](http://blast.ncbi.nlm.nih.gov/Blast.cgi#223468597%23223468597) | 18.5 | 75% | 386 |
| [NP_001138471.1](http://www.ncbi.nlm.nih.gov/entrez/query.fcgi?cmd=Retrieve&db=Protein&list_uids=223468595&dopt=GenPept&RID=TFMTSW8D01N&log$=prottop&blast_rank=55) | integrin alpha-V isoform 2 [Homo sapiens] | [18.5](http://blast.ncbi.nlm.nih.gov/Blast.cgi#223468595%23223468595) | 18.5 | 75% | 386 |
| [NP_001156751.1](http://www.ncbi.nlm.nih.gov/entrez/query.fcgi?cmd=Retrieve&db=Protein&list_uids=253970446&dopt=GenPept&RID=TFMTSW8D01N&log$=prottop&blast_rank=56) | teneurin-1 isoform 2 [Homo sapiens] | [18.5](http://blast.ncbi.nlm.nih.gov/Blast.cgi#253970446%23253970446) | 18.5 | 100% | 386 |
| [NP_001156750.1](http://www.ncbi.nlm.nih.gov/entrez/query.fcgi?cmd=Retrieve&db=Protein&list_uids=253970444&dopt=GenPept&RID=TFMTSW8D01N&log$=prottop&blast_rank=57) | teneurin-1 isoform 1 [Homo sapiens] | [18.5](http://blast.ncbi.nlm.nih.gov/Blast.cgi#253970444%23253970444) | 18.5 | 100% | 386 |
| [NP_940857.2](http://www.ncbi.nlm.nih.gov/entrez/query.fcgi?cmd=Retrieve&db=Protein&list_uids=134031945&dopt=GenPept&RID=TFMTSW8D01N&log$=prottop&blast_rank=58) | SCO-spondin precursor [Homo sapiens] | [18.5](http://blast.ncbi.nlm.nih.gov/Blast.cgi#134031945%23134031945) | 18.5 | 75% | 386 |
| [NP_942153.1](http://www.ncbi.nlm.nih.gov/entrez/query.fcgi?cmd=Retrieve&db=Protein&list_uids=38570109&dopt=GenPept&RID=TFMTSW8D01N&log$=prottop&blast_rank=59) | EF-hand calcium-binding domain-containing protein 6 isoform b [Homo sapiens] | [18.5](http://blast.ncbi.nlm.nih.gov/Blast.cgi#38570109%2338570109) | 18.5 | 62% | 386 |
| [NP_075377.2](http://www.ncbi.nlm.nih.gov/entrez/query.fcgi?cmd=Retrieve&db=Protein&list_uids=115334679&dopt=GenPept&RID=TFMTSW8D01N&log$=prottop&blast_rank=60) | retinoblastoma-binding protein 1 isoform III [Homo sapiens] | [18.5](http://blast.ncbi.nlm.nih.gov/Blast.cgi#115334679%23115334679) | 18.5 | 62% | 386 |
| [NP_075376.2](http://www.ncbi.nlm.nih.gov/entrez/query.fcgi?cmd=Retrieve&db=Protein&list_uids=115334673&dopt=GenPept&RID=TFMTSW8D01N&log$=prottop&blast_rank=61) | retinoblastoma-binding protein 1 isoform II [Homo sapiens] | [18.5](http://blast.ncbi.nlm.nih.gov/Blast.cgi#115334673%23115334673) | 18.5 | 62% | 386 |
| [NP_002883.3](http://www.ncbi.nlm.nih.gov/entrez/query.fcgi?cmd=Retrieve&db=Protein&list_uids=115334677&dopt=GenPept&RID=TFMTSW8D01N&log$=prottop&blast_rank=62) | retinoblastoma-binding protein 1 isoform I [Homo sapiens] | [18.5](http://blast.ncbi.nlm.nih.gov/Blast.cgi#115334677%23115334677) | 18.5 | 62% | 386 |
| [NP_073622.2](http://www.ncbi.nlm.nih.gov/entrez/query.fcgi?cmd=Retrieve&db=Protein&list_uids=38570107&dopt=GenPept&RID=TFMTSW8D01N&log$=prottop&blast_rank=63) | EF-hand calcium-binding domain-containing protein 6 isoform a [Homo sapiens] | [18.5](http://blast.ncbi.nlm.nih.gov/Blast.cgi#38570107%2338570107) | 18.5 | 62% | 386 |
| [NP_997646.1](http://www.ncbi.nlm.nih.gov/entrez/query.fcgi?cmd=Retrieve&db=Protein&list_uids=47078224&dopt=GenPept&RID=TFMTSW8D01N&log$=prottop&blast_rank=64) | AT rich interactive domain 5A [Homo sapiens] | [18.5](http://blast.ncbi.nlm.nih.gov/Blast.cgi#47078224%2347078224) | 18.5 | 62% | 386 |
| [NP_073143.2](http://www.ncbi.nlm.nih.gov/entrez/query.fcgi?cmd=Retrieve&db=Protein&list_uids=42764687&dopt=GenPept&RID=TFMTSW8D01N&log$=prottop&blast_rank=65) | dual specificity protein phosphatase 6 isoform b [Homo sapiens] | [18.5](http://blast.ncbi.nlm.nih.gov/Blast.cgi#42764687%2342764687) | 18.5 | 62% | 386 |
| [NP_057458.4](http://www.ncbi.nlm.nih.gov/entrez/query.fcgi?cmd=Retrieve&db=Protein&list_uids=22035677&dopt=GenPept&RID=TFMTSW8D01N&log$=prottop&blast_rank=66) | AT-rich interactive domain-containing protein 4B isoform 1 [Homo sapiens] | [18.5](http://blast.ncbi.nlm.nih.gov/Blast.cgi#22035677%2322035677) | 18.5 | 62% | 386 |
| [NP_001937.2](http://www.ncbi.nlm.nih.gov/entrez/query.fcgi?cmd=Retrieve&db=Protein&list_uids=42764683&dopt=GenPept&RID=TFMTSW8D01N&log$=prottop&blast_rank=67) | dual specificity protein phosphatase 6 isoform a [Homo sapiens] | [18.5](http://blast.ncbi.nlm.nih.gov/Blast.cgi#42764683%2342764683) | 18.5 | 62% | 386 |
| [NP_112739.2](http://www.ncbi.nlm.nih.gov/entrez/query.fcgi?cmd=Retrieve&db=Protein&list_uids=22035679&dopt=GenPept&RID=TFMTSW8D01N&log$=prottop&blast_rank=68) | AT-rich interactive domain-containing protein 4B isoform 2 [Homo sapiens] | [18.5](http://blast.ncbi.nlm.nih.gov/Blast.cgi#22035679%2322035679) | 18.5 | 62% | 386 |
| [NP_055068.2](http://www.ncbi.nlm.nih.gov/entrez/query.fcgi?cmd=Retrieve&db=Protein&list_uids=110347400&dopt=GenPept&RID=TFMTSW8D01N&log$=prottop&blast_rank=69) | teneurin-1 isoform 3 [Homo sapiens] | [18.5](http://blast.ncbi.nlm.nih.gov/Blast.cgi#110347400%23110347400) | 18.5 | 100% | 386 |
| [NP_002201.1](http://www.ncbi.nlm.nih.gov/entrez/query.fcgi?cmd=Retrieve&db=Protein&list_uids=4504763&dopt=GenPept&RID=TFMTSW8D01N&log$=prottop&blast_rank=70) | integrin alpha-V isoform 1 precursor [Homo sapiens] | [18.5](http://blast.ncbi.nlm.nih.gov/Blast.cgi#4504763%234504763) | 18.5 | 75% | 386 |
| [NP_001386.1](http://www.ncbi.nlm.nih.gov/entrez/query.fcgi?cmd=Retrieve&db=Protein&list_uids=4503421&dopt=GenPept&RID=TFMTSW8D01N&log$=prottop&blast_rank=71) | dual specificity protein phosphatase 9 [Homo sapiens] | [18.5](http://blast.ncbi.nlm.nih.gov/Blast.cgi#4503421%234503421) | 18.5 | 62% | 386 |
| [NP_001455.3](http://www.ncbi.nlm.nih.gov/entrez/query.fcgi?cmd=Retrieve&db=Protein&list_uids=55743080&dopt=GenPept&RID=TFMTSW8D01N&log$=prottop&blast_rank=72) | disintegrin and metalloproteinase domain-containing protein 2 proprotein [Homo sapiens] | [18.5](http://blast.ncbi.nlm.nih.gov/Blast.cgi#55743080%2355743080) | 18.5 | 75% | 386 |
| [NP_000435.3](http://www.ncbi.nlm.nih.gov/entrez/query.fcgi?cmd=Retrieve&db=Protein&list_uids=90403592&dopt=GenPept&RID=TFMTSW8D01N&log$=prottop&blast_rank=73) | phosphate-regulating neutral endopeptidase [Homo sapiens] | [18.5](http://blast.ncbi.nlm.nih.gov/Blast.cgi#90403592%2390403592) | 18.5 | 62% | 386 |
| [NP_003045.2](http://www.ncbi.nlm.nih.gov/entrez/query.fcgi?cmd=Retrieve&db=Protein&list_uids=42476325&dopt=GenPept&RID=TFMTSW8D01N&log$=prottop&blast_rank=74) | synaptic vesicular amine transporter [Homo sapiens] | [18.5](http://blast.ncbi.nlm.nih.gov/Blast.cgi#42476325%2342476325) | 18.5 | 87% | 386 |
| [XP_002344821.1](http://www.ncbi.nlm.nih.gov/entrez/query.fcgi?cmd=Retrieve&db=Protein&list_uids=239756124&dopt=GenPept&RID=TFMTSW8D01N&log$=prottop&blast_rank=75) | PREDICTED: hypothetical protein [Homo sapiens] | [18.0](http://blast.ncbi.nlm.nih.gov/Blast.cgi#239756124%23239756124) | 18.0 | 62% | 518 |
| [NP_001128230.1](http://www.ncbi.nlm.nih.gov/entrez/query.fcgi?cmd=Retrieve&db=Protein&list_uids=197927203&dopt=GenPept&RID=TFMTSW8D01N&log$=prottop&blast_rank=76) | ADP-ribosylation factor related protein 1 isoform b [Homo sapiens] | [18.0](http://blast.ncbi.nlm.nih.gov/Blast.cgi#197927203%23197927203) | 18.0 | 75% | 518 |
| [NP_061978.6](http://www.ncbi.nlm.nih.gov/entrez/query.fcgi?cmd=Retrieve&db=Protein&list_uids=188528648&dopt=GenPept&RID=TFMTSW8D01N&log$=prottop&blast_rank=77) | tenascin XB isoform 1 precursor [Homo sapiens] | [18.0](http://blast.ncbi.nlm.nih.gov/Blast.cgi#188528648%23188528648) | 31.8 | 87% | 518 |
| [NP_065770.1](http://www.ncbi.nlm.nih.gov/entrez/query.fcgi?cmd=Retrieve&db=Protein&list_uids=153792074&dopt=GenPept&RID=TFMTSW8D01N&log$=prottop&blast_rank=78) | proline-rich protein 12 [Homo sapiens] | [18.0](http://blast.ncbi.nlm.nih.gov/Blast.cgi#153792074%23153792074) | 18.0 | 75% | 518 |
| [NP_872310.2](http://www.ncbi.nlm.nih.gov/entrez/query.fcgi?cmd=Retrieve&db=Protein&list_uids=153281226&dopt=GenPept&RID=TFMTSW8D01N&log$=prottop&blast_rank=79) | hypothetical protein LOC135886 [Homo sapiens] | [18.0](http://blast.ncbi.nlm.nih.gov/Blast.cgi#153281226%23153281226) | 18.0 | 62% | 518 |
| [NP_001116427.1](http://www.ncbi.nlm.nih.gov/entrez/query.fcgi?cmd=Retrieve&db=Protein&list_uids=171906569&dopt=GenPept&RID=TFMTSW8D01N&log$=prottop&blast_rank=80) | seipin isoform 1 [Homo sapiens] >ref|NP_001124174.1| seipin isoform 1 [Homo sapiens] | [18.0](http://blast.ncbi.nlm.nih.gov/Blast.cgi#171906569%23171906569) | 18.0 | 62% | 518 |
| [NP_001363.2](http://www.ncbi.nlm.nih.gov/entrez/query.fcgi?cmd=Retrieve&db=Protein&list_uids=114155133&dopt=GenPept&RID=TFMTSW8D01N&log$=prottop&blast_rank=81) | dynein heavy chain 9, axonemal isoform 2 [Homo sapiens] | [18.0](http://blast.ncbi.nlm.nih.gov/Blast.cgi#114155133%23114155133) | 29.3 | 87% | 518 |
| [NP_004653.2](http://www.ncbi.nlm.nih.gov/entrez/query.fcgi?cmd=Retrieve&db=Protein&list_uids=114155135&dopt=GenPept&RID=TFMTSW8D01N&log$=prottop&blast_rank=82) | dynein heavy chain 9, axonemal isoform 1 [Homo sapiens] | [18.0](http://blast.ncbi.nlm.nih.gov/Blast.cgi#114155135%23114155135) | 18.0 | 62% | 518 |
| [NP_115495.3](http://www.ncbi.nlm.nih.gov/entrez/query.fcgi?cmd=Retrieve&db=Protein&list_uids=113722120&dopt=GenPept&RID=TFMTSW8D01N&log$=prottop&blast_rank=83) | G protein-coupled receptor 98 precursor [Homo sapiens] | [18.0](http://blast.ncbi.nlm.nih.gov/Blast.cgi#113722120%23113722120) | 28.0 | 75% | 518 |
| [NP_005112.2](http://www.ncbi.nlm.nih.gov/entrez/query.fcgi?cmd=Retrieve&db=Protein&list_uids=102468717&dopt=GenPept&RID=TFMTSW8D01N&log$=prottop&blast_rank=84) | mediator of RNA polymerase II transcription subunit 13 [Homo sapiens] | [18.0](http://blast.ncbi.nlm.nih.gov/Blast.cgi#102468717%23102468717) | 18.0 | 62% | 518 |
| [NP_060143.4](http://www.ncbi.nlm.nih.gov/entrez/query.fcgi?cmd=Retrieve&db=Protein&list_uids=157502207&dopt=GenPept&RID=TFMTSW8D01N&log$=prottop&blast_rank=85) | hypothetical protein LOC54823 [Homo sapiens] >ref|NP_001098988.1| hypothetical protein LOC54823 [Homo sapiens] | [18.0](http://blast.ncbi.nlm.nih.gov/Blast.cgi#157502207%23157502207) | 18.0 | 62% | 518 |
| [NP_659402.1](http://www.ncbi.nlm.nih.gov/entrez/query.fcgi?cmd=Retrieve&db=Protein&list_uids=21450645&dopt=GenPept&RID=TFMTSW8D01N&log$=prottop&blast_rank=86) | tetratricopeptide repeat protein 16 [Homo sapiens] | [18.0](http://blast.ncbi.nlm.nih.gov/Blast.cgi#21450645%2321450645) | 18.0 | 62% | 518 |
| [NP_877576.1](http://www.ncbi.nlm.nih.gov/entrez/query.fcgi?cmd=Retrieve&db=Protein&list_uids=33359217&dopt=GenPept&RID=TFMTSW8D01N&log$=prottop&blast_rank=87) | ALS2 C-terminal-like protein isoform 3 [Homo sapiens] | [18.0](http://blast.ncbi.nlm.nih.gov/Blast.cgi#33359217%2333359217) | 18.0 | 75% | 518 |
| [NP_002196.2](http://www.ncbi.nlm.nih.gov/entrez/query.fcgi?cmd=Retrieve&db=Protein&list_uids=56237029&dopt=GenPept&RID=TFMTSW8D01N&log$=prottop&blast_rank=88) | integrin alpha 5 precursor [Homo sapiens] | [18.0](http://blast.ncbi.nlm.nih.gov/Blast.cgi#56237029%2356237029) | 18.0 | 75% | 518 |
| [NP_004742.1](http://www.ncbi.nlm.nih.gov/entrez/query.fcgi?cmd=Retrieve&db=Protein&list_uids=4758422&dopt=GenPept&RID=TFMTSW8D01N&log$=prottop&blast_rank=89) | beta-1,3-galactosyl-O-glycosyl-glycoprotein beta-1,6-N-acetylglucosaminyltransferase 3 [Homo sapiens] | [18.0](http://blast.ncbi.nlm.nih.gov/Blast.cgi#4758422%234758422) | 18.0 | 62% | 518 |
| [NP_002020.1](http://www.ncbi.nlm.nih.gov/entrez/query.fcgi?cmd=Retrieve&db=Protein&list_uids=4503779&dopt=GenPept&RID=TFMTSW8D01N&log$=prottop&blast_rank=90) | fMet-Leu-Phe receptor [Homo sapiens] | [18.0](http://blast.ncbi.nlm.nih.gov/Blast.cgi#4503779%234503779) | 18.0 | 75% | 518 |
| [NP_620153.1](http://www.ncbi.nlm.nih.gov/entrez/query.fcgi?cmd=Retrieve&db=Protein&list_uids=20270349&dopt=GenPept&RID=TFMTSW8D01N&log$=prottop&blast_rank=91) | MIT domain-containing protein 1 [Homo sapiens] | [18.0](http://blast.ncbi.nlm.nih.gov/Blast.cgi#20270349%2320270349) | 18.0 | 62% | 518 |
| [NP_667340.2](http://www.ncbi.nlm.nih.gov/entrez/query.fcgi?cmd=Retrieve&db=Protein&list_uids=33359215&dopt=GenPept&RID=TFMTSW8D01N&log$=prottop&blast_rank=92) | ALS2 C-terminal-like protein isoform 1 [Homo sapiens] | [18.0](http://blast.ncbi.nlm.nih.gov/Blast.cgi#33359215%2333359215) | 18.0 | 75% | 518 |
| [NP_004063.1](http://www.ncbi.nlm.nih.gov/entrez/query.fcgi?cmd=Retrieve&db=Protein&list_uids=4758014&dopt=GenPept&RID=TFMTSW8D01N&log$=prottop&blast_rank=93) | chemokine receptor-like 1 isoform b [Homo sapiens] | [18.0](http://blast.ncbi.nlm.nih.gov/Blast.cgi#4758014%234758014) | 18.0 | 75% | 518 |
| [NP_647603.1](http://www.ncbi.nlm.nih.gov/entrez/query.fcgi?cmd=Retrieve&db=Protein&list_uids=21536362&dopt=GenPept&RID=TFMTSW8D01N&log$=prottop&blast_rank=94) | TATA box-binding protein-associated factor RNA polymerase I subunit A isoform 2 [Homo sapiens] | [18.0](http://blast.ncbi.nlm.nih.gov/Blast.cgi#21536362%2321536362) | 18.0 | 62% | 518 |
| [NP_001135815.1](http://www.ncbi.nlm.nih.gov/entrez/query.fcgi?cmd=Retrieve&db=Protein&list_uids=215272317&dopt=GenPept&RID=TFMTSW8D01N&log$=prottop&blast_rank=95) | chemokine receptor-like 1 isoform a [Homo sapiens] >ref|NP_001135816.1| chemokine receptor-like 1 isoform a [Homo sapiens] >ref|NP_001135817.1| chemokine receptor-like 1 isoform a [Homo sapiens] | [18.0](http://blast.ncbi.nlm.nih.gov/Blast.cgi#215272317%23215272317) | 18.0 | 75% | 518 |
| [NP_775825.1](http://www.ncbi.nlm.nih.gov/entrez/query.fcgi?cmd=Retrieve&db=Protein&list_uids=27734885&dopt=GenPept&RID=TFMTSW8D01N&log$=prottop&blast_rank=96) | hypothetical protein LOC219621 [Homo sapiens] | [18.0](http://blast.ncbi.nlm.nih.gov/Blast.cgi#27734885%2327734885) | 18.0 | 62% | 518 |
| [NP_009195.1](http://www.ncbi.nlm.nih.gov/entrez/query.fcgi?cmd=Retrieve&db=Protein&list_uids=6005705&dopt=GenPept&RID=TFMTSW8D01N&log$=prottop&blast_rank=97) | G-protein coupled receptor 182 [Homo sapiens] | [18.0](http://blast.ncbi.nlm.nih.gov/Blast.cgi#6005705%236005705) | 18.0 | 62% | 518 |
| [NP_002021.3](http://www.ncbi.nlm.nih.gov/entrez/query.fcgi?cmd=Retrieve&db=Protein&list_uids=38455413&dopt=GenPept&RID=TFMTSW8D01N&log$=prottop&blast_rank=98) | N-formyl peptide receptor 3 [Homo sapiens] | [18.0](http://blast.ncbi.nlm.nih.gov/Blast.cgi#38455413%2338455413) | 18.0 | 75% | 518 |
| [NP_057641.1](http://www.ncbi.nlm.nih.gov/entrez/query.fcgi?cmd=Retrieve&db=Protein&list_uids=7706769&dopt=GenPept&RID=TFMTSW8D01N&log$=prottop&blast_rank=99) | chemokine (C-C motif) receptor-like 1 [Homo sapiens] >ref|NP_848540.1| chemokine (C-C motif) receptor-like 1 [Homo sapiens] | [18.0](http://blast.ncbi.nlm.nih.gov/Blast.cgi#7706769%237706769) | 18.0 | 75% | 518 |
| [NP_005672.1](http://www.ncbi.nlm.nih.gov/entrez/query.fcgi?cmd=Retrieve&db=Protein&list_uids=5032143&dopt=GenPept&RID=TFMTSW8D01N&log$=prottop&blast_rank=100) | TATA box-binding protein-associated factor RNA polymerase I subunit A isoform 1 [Homo sapiens] | [18.0](http://blast.ncbi.nlm.nih.gov/Blast.cgi#5032143%235032143) | 18.0 | 62% | 518 |

| **Accession** | **Proteins with a match to RRTDYLLNG peptide** | **[Max score](http://blast.ncbi.nlm.nih.gov/Blast.cgi?CMD=Get&ALIGNMENTS=100&ALIGNMENT_VIEW=Pairwise&CDD_SEARCH_STATE=1&DATABASE_SORT=0&DESCRIPTIONS=100&ENTREZ_QUERY=txid9606 %5BORGN%5D&FIRST_QUERY_NUM=0&FORMAT_OBJECT=Alignment&FORMAT_PAGE_TARGET=&FORMAT_TYPE=HTML&GET_SEQUENCE=yes&I_THRESH=&MASK_CHAR=2&MASK_COLOR=1&NEW_VIEW=yes&NUM_OVERVIEW=100&OLD_BLAST=false&PAGE=Proteins&QUERY_INDEX=0&QUERY_NUMBER=0&RESULTS_PAGE_TARGET=&RID=TFMYP1MP01N&SHOW_LINKOUT=yes&SHOW_OVERVIEW=yes&STEP_NUMBER=&WORD_SIZE=2&DISPLAY_SORT=1&HSP_SORT=1" \l "sort_mark)** | **[Total score](http://blast.ncbi.nlm.nih.gov/Blast.cgi?CMD=Get&ALIGNMENTS=100&ALIGNMENT_VIEW=Pairwise&CDD_SEARCH_STATE=1&DATABASE_SORT=0&DESCRIPTIONS=100&ENTREZ_QUERY=txid9606 %5BORGN%5D&FIRST_QUERY_NUM=0&FORMAT_OBJECT=Alignment&FORMAT_PAGE_TARGET=&FORMAT_TYPE=HTML&GET_SEQUENCE=yes&I_THRESH=&MASK_CHAR=2&MASK_COLOR=1&NEW_VIEW=yes&NUM_OVERVIEW=100&OLD_BLAST=false&PAGE=Proteins&QUERY_INDEX=0&QUERY_NUMBER=0&RESULTS_PAGE_TARGET=&RID=TFMYP1MP01N&SHOW_LINKOUT=yes&SHOW_OVERVIEW=yes&STEP_NUMBER=&WORD_SIZE=2&DISPLAY_SORT=2&HSP_SORT=1" \l "sort_mark)** | **[Query coverage](http://blast.ncbi.nlm.nih.gov/Blast.cgi?CMD=Get&ALIGNMENTS=100&ALIGNMENT_VIEW=Pairwise&CDD_SEARCH_STATE=1&DATABASE_SORT=0&DESCRIPTIONS=100&ENTREZ_QUERY=txid9606 %5BORGN%5D&FIRST_QUERY_NUM=0&FORMAT_OBJECT=Alignment&FORMAT_PAGE_TARGET=&FORMAT_TYPE=HTML&GET_SEQUENCE=yes&I_THRESH=&MASK_CHAR=2&MASK_COLOR=1&NEW_VIEW=yes&NUM_OVERVIEW=100&OLD_BLAST=false&PAGE=Proteins&QUERY_INDEX=0&QUERY_NUMBER=0&RESULTS_PAGE_TARGET=&RID=TFMYP1MP01N&SHOW_LINKOUT=yes&SHOW_OVERVIEW=yes&STEP_NUMBER=&WORD_SIZE=2&DISPLAY_SORT=4&HSP_SORT=0" \l "sort_mark)** | **[E value](http://blast.ncbi.nlm.nih.gov/Blast.cgi?CMD=Get&ALIGNMENTS=100&ALIGNMENT_VIEW=Pairwise&CDD_SEARCH_STATE=1&DATABASE_SORT=0&DESCRIPTIONS=100&ENTREZ_QUERY=txid9606 %5BORGN%5D&FIRST_QUERY_NUM=0&FORMAT_OBJECT=Alignment&FORMAT_PAGE_TARGET=&FORMAT_TYPE=HTML&GET_SEQUENCE=yes&I_THRESH=&MASK_CHAR=2&MASK_COLOR=1&NEW_VIEW=yes&NUM_OVERVIEW=100&OLD_BLAST=false&PAGE=Proteins&QUERY_INDEX=0&QUERY_NUMBER=0&RESULTS_PAGE_TARGET=&RID=TFMYP1MP01N&SHOW_LINKOUT=yes&SHOW_OVERVIEW=yes&STEP_NUMBER=&WORD_SIZE=2&DISPLAY_SORT=0&HSP_SORT=0" \l "sort_mark)** |
| --- | --- | --- | --- | --- | --- |
| [NP_001028289.1](http://www.ncbi.nlm.nih.gov/entrez/query.fcgi?cmd=Retrieve&db=Protein&list_uids=75677374&dopt=GenPept&RID=TFMYP1MP01N&log$=prottop&blast_rank=1) | SLIT-ROBO Rho GTPase activating protein 3 isoform b [Homo sapiens] | [22.7](http://blast.ncbi.nlm.nih.gov/Blast.cgi#75677374%2375677374) | 22.7 | 77% | 23 |
| [NP_055665.1](http://www.ncbi.nlm.nih.gov/entrez/query.fcgi?cmd=Retrieve&db=Protein&list_uids=24307967&dopt=GenPept&RID=TFMYP1MP01N&log$=prottop&blast_rank=2) | SLIT-ROBO Rho GTPase activating protein 3 isoform a [Homo sapiens] | [22.7](http://blast.ncbi.nlm.nih.gov/Blast.cgi#24307967%2324307967) | 22.7 | 77% | 23 |
| [NP_004997.4](http://www.ncbi.nlm.nih.gov/entrez/query.fcgi?cmd=Retrieve&db=Protein&list_uids=33519475&dopt=GenPept&RID=TFMYP1MP01N&log$=prottop&blast_rank=3) | NADH dehydrogenase (ubiquinone) Fe-S protein 1, 75kDa precursor [Homo sapiens] | [21.0](http://blast.ncbi.nlm.nih.gov/Blast.cgi#33519475%2333519475) | 21.0 | 77% | 74 |
| [XP_002343933.1](http://www.ncbi.nlm.nih.gov/entrez/query.fcgi?cmd=Retrieve&db=Protein&list_uids=239747180&dopt=GenPept&RID=TFMYP1MP01N&log$=prottop&blast_rank=4) | PREDICTED: hypothetical protein XP_002343933 [Homo sapiens] | [20.6](http://blast.ncbi.nlm.nih.gov/Blast.cgi#239747180%23239747180) | 35.6 | 100% | 100 |
| [NP_001157912.1](http://www.ncbi.nlm.nih.gov/entrez/query.fcgi?cmd=Retrieve&db=Protein&list_uids=256574784&dopt=GenPept&RID=TFMYP1MP01N&log$=prottop&blast_rank=5) | ankyrin repeat domain-containing protein 33B [Homo sapiens] >ref|XP_001723826.1| PREDICTED: ankyrin repeat domain 33B [Homo sapiens] | [20.6](http://blast.ncbi.nlm.nih.gov/Blast.cgi#256574784%23256574784) | 20.6 | 66% | 100 |
| [XP_946054.2](http://www.ncbi.nlm.nih.gov/entrez/query.fcgi?cmd=Retrieve&db=Protein&list_uids=113416996&dopt=GenPept&RID=TFMYP1MP01N&log$=prottop&blast_rank=6) | PREDICTED: ankyrin repeat domain 33B [Homo sapiens] | [20.6](http://blast.ncbi.nlm.nih.gov/Blast.cgi#113416996%23113416996) | 20.6 | 66% | 100 |
| [NP_001138678.1](http://www.ncbi.nlm.nih.gov/entrez/query.fcgi?cmd=Retrieve&db=Protein&list_uids=223633988&dopt=GenPept&RID=TFMYP1MP01N&log$=prottop&blast_rank=7) | hypothetical protein LOC85379 [Homo sapiens] | [20.6](http://blast.ncbi.nlm.nih.gov/Blast.cgi#223633988%23223633988) | 35.6 | 100% | 100 |
| [XP_002342992.1](http://www.ncbi.nlm.nih.gov/entrez/query.fcgi?cmd=Retrieve&db=Protein&list_uids=239743930&dopt=GenPept&RID=TFMYP1MP01N&log$=prottop&blast_rank=8) | PREDICTED: hypothetical protein XP_002342992 [Homo sapiens] >ref|XP_002347139.1| PREDICTED: hypothetical protein XP_002347139 [Homo sapiens] | [20.2](http://blast.ncbi.nlm.nih.gov/Blast.cgi#239743930%23239743930) | 20.2 | 55% | 134 |
| [NP_001012302.2](http://www.ncbi.nlm.nih.gov/entrez/query.fcgi?cmd=Retrieve&db=Protein&list_uids=145611446&dopt=GenPept&RID=TFMYP1MP01N&log$=prottop&blast_rank=9) | anoctamin-9 [Homo sapiens] | [20.2](http://blast.ncbi.nlm.nih.gov/Blast.cgi#145611446%23145611446) | 20.2 | 88% | 134 |
| [NP_005682.2](http://www.ncbi.nlm.nih.gov/entrez/query.fcgi?cmd=Retrieve&db=Protein&list_uids=110832835&dopt=GenPept&RID=TFMYP1MP01N&log$=prottop&blast_rank=10) | ATP-binding cassette transporter sub-family C member 9 isoform SUR2A [Homo sapiens] | [20.2](http://blast.ncbi.nlm.nih.gov/Blast.cgi#110832835%23110832835) | 20.2 | 55% | 134 |
| [NP_064694.2](http://www.ncbi.nlm.nih.gov/entrez/query.fcgi?cmd=Retrieve&db=Protein&list_uids=110832839&dopt=GenPept&RID=TFMYP1MP01N&log$=prottop&blast_rank=11) | ATP-binding cassette transporter sub-family C member 9 isoform SUR2A-delta-14 [Homo sapiens] | [20.2](http://blast.ncbi.nlm.nih.gov/Blast.cgi#110832839%23110832839) | 20.2 | 55% | 134 |
| [NP_064693.2](http://www.ncbi.nlm.nih.gov/entrez/query.fcgi?cmd=Retrieve&db=Protein&list_uids=110832837&dopt=GenPept&RID=TFMYP1MP01N&log$=prottop&blast_rank=12) | ATP-binding cassette transporter sub-family C member 9 isoform SUR2B [Homo sapiens] | [20.2](http://blast.ncbi.nlm.nih.gov/Blast.cgi#110832837%23110832837) | 20.2 | 55% | 134 |
| [NP_258260.1](http://www.ncbi.nlm.nih.gov/entrez/query.fcgi?cmd=Retrieve&db=Protein&list_uids=28875786&dopt=GenPept&RID=TFMYP1MP01N&log$=prottop&blast_rank=13) | FCH and double SH3 domains 1 [Homo sapiens] | [20.2](http://blast.ncbi.nlm.nih.gov/Blast.cgi#28875786%2328875786) | 20.2 | 77% | 134 |
| [NP_061137.1](http://www.ncbi.nlm.nih.gov/entrez/query.fcgi?cmd=Retrieve&db=Protein&list_uids=8923946&dopt=GenPept&RID=TFMYP1MP01N&log$=prottop&blast_rank=14) | sphingomyelin phosphodiesterase 3 [Homo sapiens] | [20.2](http://blast.ncbi.nlm.nih.gov/Blast.cgi#8923946%238923946) | 20.2 | 77% | 134 |
| [NP_056382.1](http://www.ncbi.nlm.nih.gov/entrez/query.fcgi?cmd=Retrieve&db=Protein&list_uids=40217823&dopt=GenPept&RID=TFMYP1MP01N&log$=prottop&blast_rank=15) | SLIT and NTRK-like protein 5 precursor [Homo sapiens] | [20.2](http://blast.ncbi.nlm.nih.gov/Blast.cgi#40217823%2340217823) | 20.2 | 66% | 134 |
| [NP_057332.1](http://www.ncbi.nlm.nih.gov/entrez/query.fcgi?cmd=Retrieve&db=Protein&list_uids=7706457&dopt=GenPept&RID=TFMYP1MP01N&log$=prottop&blast_rank=16) | A-kinase anchor protein 11 [Homo sapiens] | [20.2](http://blast.ncbi.nlm.nih.gov/Blast.cgi#7706457%237706457) | 20.2 | 55% | 134 |
| [NP_002798.2](http://www.ncbi.nlm.nih.gov/entrez/query.fcgi?cmd=Retrieve&db=Protein&list_uids=25777600&dopt=GenPept&RID=TFMYP1MP01N&log$=prottop&blast_rank=17) | proteasome 26S non-ATPase subunit 1 [Homo sapiens] | [20.2](http://blast.ncbi.nlm.nih.gov/Blast.cgi#25777600%2325777600) | 20.2 | 55% | 134 |
| [NP_851782.3](http://www.ncbi.nlm.nih.gov/entrez/query.fcgi?cmd=Retrieve&db=Protein&list_uids=266457048&dopt=GenPept&RID=TFMYP1MP01N&log$=prottop&blast_rank=18) | WD repeat domain 17 isoform 2 [Homo sapiens] | [19.7](http://blast.ncbi.nlm.nih.gov/Blast.cgi#266457048%23266457048) | 32.2 | 66% | 180 |
| [NP_001078906.1](http://www.ncbi.nlm.nih.gov/entrez/query.fcgi?cmd=Retrieve&db=Protein&list_uids=146198549&dopt=GenPept&RID=TFMYP1MP01N&log$=prottop&blast_rank=19) | hypothetical protein LOC79919 isoform 1 [Homo sapiens] | [19.7](http://blast.ncbi.nlm.nih.gov/Blast.cgi#146198549%23146198549) | 19.7 | 55% | 180 |
| [NP_000199.2](http://www.ncbi.nlm.nih.gov/entrez/query.fcgi?cmd=Retrieve&db=Protein&list_uids=119395736&dopt=GenPept&RID=TFMYP1MP01N&log$=prottop&blast_rank=20) | insulin receptor isoform Long precursor [Homo sapiens] | [19.7](http://blast.ncbi.nlm.nih.gov/Blast.cgi#119395736%23119395736) | 36.5 | 55% | 180 |
| [NP_001073285.1](http://www.ncbi.nlm.nih.gov/entrez/query.fcgi?cmd=Retrieve&db=Protein&list_uids=119395738&dopt=GenPept&RID=TFMYP1MP01N&log$=prottop&blast_rank=21) | insulin receptor isoform Short precursor [Homo sapiens] | [19.7](http://blast.ncbi.nlm.nih.gov/Blast.cgi#119395738%23119395738) | 36.5 | 55% | 180 |
| [NP_001262.3](http://www.ncbi.nlm.nih.gov/entrez/query.fcgi?cmd=Retrieve&db=Protein&list_uids=118421089&dopt=GenPept&RID=TFMYP1MP01N&log$=prottop&blast_rank=22) | chromodomain-helicase-DNA-binding protein 2 isoform 1 [Homo sapiens] | [19.7](http://blast.ncbi.nlm.nih.gov/Blast.cgi#118421089%23118421089) | 31.8 | 66% | 180 |
| [NP_940922.2](http://www.ncbi.nlm.nih.gov/entrez/query.fcgi?cmd=Retrieve&db=Protein&list_uids=112420960&dopt=GenPept&RID=TFMYP1MP01N&log$=prottop&blast_rank=23) | putative uncharacterized protein C12orf63 [Homo sapiens] | [19.7](http://blast.ncbi.nlm.nih.gov/Blast.cgi#112420960%23112420960) | 19.7 | 55% | 180 |
| [NP_877497.2](http://www.ncbi.nlm.nih.gov/entrez/query.fcgi?cmd=Retrieve&db=Protein&list_uids=190341084&dopt=GenPept&RID=TFMYP1MP01N&log$=prottop&blast_rank=24) | probable E3 ubiquitin-protein ligase HECTD2 isoform a [Homo sapiens] | [19.7](http://blast.ncbi.nlm.nih.gov/Blast.cgi#190341084%23190341084) | 19.7 | 66% | 180 |
| [NP_898884.1](http://www.ncbi.nlm.nih.gov/entrez/query.fcgi?cmd=Retrieve&db=Protein&list_uids=56786136&dopt=GenPept&RID=TFMYP1MP01N&log$=prottop&blast_rank=25) | sodium/hydrogen exchanger 10 [Homo sapiens] | [19.7](http://blast.ncbi.nlm.nih.gov/Blast.cgi#56786136%2356786136) | 33.9 | 77% | 180 |
| [NP_109592.1](http://www.ncbi.nlm.nih.gov/entrez/query.fcgi?cmd=Retrieve&db=Protein&list_uids=13677214&dopt=GenPept&RID=TFMYP1MP01N&log$=prottop&blast_rank=26) | receptor-type protein tyrosine phosphatase O isoform a precursor [Homo sapiens] | [19.7](http://blast.ncbi.nlm.nih.gov/Blast.cgi#13677214%2313677214) | 19.7 | 55% | 180 |
| [NP_733828.2](http://www.ncbi.nlm.nih.gov/entrez/query.fcgi?cmd=Retrieve&db=Protein&list_uids=31317311&dopt=GenPept&RID=TFMYP1MP01N&log$=prottop&blast_rank=27) | WD repeat domain 17 isoform 1 [Homo sapiens] | [19.7](http://blast.ncbi.nlm.nih.gov/Blast.cgi#31317311%2331317311) | 32.2 | 66% | 180 |
| [NP_079137.2](http://www.ncbi.nlm.nih.gov/entrez/query.fcgi?cmd=Retrieve&db=Protein&list_uids=146198668&dopt=GenPept&RID=TFMYP1MP01N&log$=prottop&blast_rank=28) | hypothetical protein LOC79919 isoform 2 [Homo sapiens] | [19.7](http://blast.ncbi.nlm.nih.gov/Blast.cgi#146198668%23146198668) | 19.7 | 55% | 180 |
| [NP_659422.2](http://www.ncbi.nlm.nih.gov/entrez/query.fcgi?cmd=Retrieve&db=Protein&list_uids=32880203&dopt=GenPept&RID=TFMYP1MP01N&log$=prottop&blast_rank=29) | cadherin-24 isoform 2 [Homo sapiens] | [19.7](http://blast.ncbi.nlm.nih.gov/Blast.cgi#32880203%2332880203) | 19.7 | 88% | 180 |
| [NP_665806.1](http://www.ncbi.nlm.nih.gov/entrez/query.fcgi?cmd=Retrieve&db=Protein&list_uids=24308065&dopt=GenPept&RID=TFMYP1MP01N&log$=prottop&blast_rank=30) | netrin 5 precursor [Homo sapiens] | [19.7](http://blast.ncbi.nlm.nih.gov/Blast.cgi#24308065%2324308065) | 19.7 | 55% | 180 |
| [NP_109594.1](http://www.ncbi.nlm.nih.gov/entrez/query.fcgi?cmd=Retrieve&db=Protein&list_uids=13677218&dopt=GenPept&RID=TFMYP1MP01N&log$=prottop&blast_rank=31) | receptor-type protein tyrosine phosphatase O isoform c precursor [Homo sapiens] >ref|NP_109596.1| receptor-type protein tyrosine phosphatase O isoform c precursor [Homo sapiens] | [19.7](http://blast.ncbi.nlm.nih.gov/Blast.cgi#13677218%2313677218) | 19.7 | 55% | 180 |
| [NP_071923.2](http://www.ncbi.nlm.nih.gov/entrez/query.fcgi?cmd=Retrieve&db=Protein&list_uids=32880206&dopt=GenPept&RID=TFMYP1MP01N&log$=prottop&blast_rank=32) | cadherin-24 isoform 1 [Homo sapiens] | [19.7](http://blast.ncbi.nlm.nih.gov/Blast.cgi#32880206%2332880206) | 19.7 | 88% | 180 |
| [NP_835465.2](http://www.ncbi.nlm.nih.gov/entrez/query.fcgi?cmd=Retrieve&db=Protein&list_uids=150456463&dopt=GenPept&RID=TFMYP1MP01N&log$=prottop&blast_rank=33) | gasdermin-A [Homo sapiens] | [19.7](http://blast.ncbi.nlm.nih.gov/Blast.cgi#150456463%23150456463) | 19.7 | 77% | 180 |
| [NP_060634.2](http://www.ncbi.nlm.nih.gov/entrez/query.fcgi?cmd=Retrieve&db=Protein&list_uids=155030185&dopt=GenPept&RID=TFMYP1MP01N&log$=prottop&blast_rank=34) | cell cycle regulator Mat89Bb homolog [Homo sapiens] | [19.7](http://blast.ncbi.nlm.nih.gov/Blast.cgi#155030185%23155030185) | 19.7 | 77% | 180 |
| [NP_060520.2](http://www.ncbi.nlm.nih.gov/entrez/query.fcgi?cmd=Retrieve&db=Protein&list_uids=31542650&dopt=GenPept&RID=TFMYP1MP01N&log$=prottop&blast_rank=35) | MANSC domain-containing protein 1 precursor [Homo sapiens] | [19.7](http://blast.ncbi.nlm.nih.gov/Blast.cgi#31542650%2331542650) | 19.7 | 88% | 180 |
| [NP_067637.2](http://www.ncbi.nlm.nih.gov/entrez/query.fcgi?cmd=Retrieve&db=Protein&list_uids=14251205&dopt=GenPept&RID=TFMYP1MP01N&log$=prottop&blast_rank=36) | histamine H4 receptor isoform 1 [Homo sapiens] | [19.7](http://blast.ncbi.nlm.nih.gov/Blast.cgi#14251205%2314251205) | 19.7 | 55% | 180 |
| [NP_055030.1](http://www.ncbi.nlm.nih.gov/entrez/query.fcgi?cmd=Retrieve&db=Protein&list_uids=31657140&dopt=GenPept&RID=TFMYP1MP01N&log$=prottop&blast_rank=37) | insulin receptor-related protein precursor [Homo sapiens] | [19.7](http://blast.ncbi.nlm.nih.gov/Blast.cgi#31657140%2331657140) | 19.7 | 55% | 180 |
| [NP_060625.2](http://www.ncbi.nlm.nih.gov/entrez/query.fcgi?cmd=Retrieve&db=Protein&list_uids=157388991&dopt=GenPept&RID=TFMYP1MP01N&log$=prottop&blast_rank=38) | solute carrier family 25 member 36 isoform b [Homo sapiens] | [19.3](http://blast.ncbi.nlm.nih.gov/Blast.cgi#157388991%23157388991) | 19.3 | 55% | 241 |
| [NP_000110.2](http://www.ncbi.nlm.nih.gov/entrez/query.fcgi?cmd=Retrieve&db=Protein&list_uids=166362735&dopt=GenPept&RID=TFMYP1MP01N&log$=prottop&blast_rank=39) | erythrocyte membrane protein band 4.2 isoform 1 [Homo sapiens] | [19.3](http://blast.ncbi.nlm.nih.gov/Blast.cgi#166362735%23166362735) | 19.3 | 77% | 241 |
| [NP_008968.4](http://www.ncbi.nlm.nih.gov/entrez/query.fcgi?cmd=Retrieve&db=Protein&list_uids=153792351&dopt=GenPept&RID=TFMYP1MP01N&log$=prottop&blast_rank=40) | ADAM metallopeptidase with thrombospondin type 1 motif, 8 preproprotein [Homo sapiens] | [19.3](http://blast.ncbi.nlm.nih.gov/Blast.cgi#153792351%23153792351) | 19.3 | 55% | 241 |
| [NP_055639.2](http://www.ncbi.nlm.nih.gov/entrez/query.fcgi?cmd=Retrieve&db=Protein&list_uids=182765466&dopt=GenPept&RID=TFMYP1MP01N&log$=prottop&blast_rank=41) | FCH and double SH3 domains protein 2 [Homo sapiens] | [19.3](http://blast.ncbi.nlm.nih.gov/Blast.cgi#182765466%23182765466) | 19.3 | 66% | 241 |
| [NP_001098117.1](http://www.ncbi.nlm.nih.gov/entrez/query.fcgi?cmd=Retrieve&db=Protein&list_uids=157388989&dopt=GenPept&RID=TFMYP1MP01N&log$=prottop&blast_rank=42) | solute carrier family 25 member 36 isoform a [Homo sapiens] | [19.3](http://blast.ncbi.nlm.nih.gov/Blast.cgi#157388989%23157388989) | 19.3 | 55% | 241 |
| [NP_006334.2](http://www.ncbi.nlm.nih.gov/entrez/query.fcgi?cmd=Retrieve&db=Protein&list_uids=66932918&dopt=GenPept&RID=TFMYP1MP01N&log$=prottop&blast_rank=43) | MER receptor tyrosine kinase precursor [Homo sapiens] | [19.3](http://blast.ncbi.nlm.nih.gov/Blast.cgi#66932918%2366932918) | 19.3 | 66% | 241 |
| [NP_001107606.1](http://www.ncbi.nlm.nih.gov/entrez/query.fcgi?cmd=Retrieve&db=Protein&list_uids=166362737&dopt=GenPept&RID=TFMYP1MP01N&log$=prottop&blast_rank=44) | erythrocyte membrane protein band 4.2 isoform 2 [Homo sapiens] | [19.3](http://blast.ncbi.nlm.nih.gov/Blast.cgi#166362737%23166362737) | 19.3 | 77% | 241 |
| [XP_001715192.1](http://www.ncbi.nlm.nih.gov/entrez/query.fcgi?cmd=Retrieve&db=Protein&list_uids=169174934&dopt=GenPept&RID=TFMYP1MP01N&log$=prottop&blast_rank=45) | PREDICTED: similar to ADAMTS-like 2 [Homo sapiens] | [19.3](http://blast.ncbi.nlm.nih.gov/Blast.cgi#169174934%23169174934) | 19.3 | 55% | 241 |
| [NP_055509.2](http://www.ncbi.nlm.nih.gov/entrez/query.fcgi?cmd=Retrieve&db=Protein&list_uids=41281450&dopt=GenPept&RID=TFMYP1MP01N&log$=prottop&blast_rank=46) | ADAMTS-like 2 precursor [Homo sapiens] >ref|NP_001138792.1| ADAMTS-like 2 precursor [Homo sapiens] | [19.3](http://blast.ncbi.nlm.nih.gov/Blast.cgi#41281450%2341281450) | 19.3 | 55% | 241 |
| [NP_071733.1](http://www.ncbi.nlm.nih.gov/entrez/query.fcgi?cmd=Retrieve&db=Protein&list_uids=11641239&dopt=GenPept&RID=TFMYP1MP01N&log$=prottop&blast_rank=47) | hypothetical protein LOC53838 precursor [Homo sapiens] | [19.3](http://blast.ncbi.nlm.nih.gov/Blast.cgi#11641239%2311641239) | 19.3 | 66% | 241 |
| [NP_003295.1](http://www.ncbi.nlm.nih.gov/entrez/query.fcgi?cmd=Retrieve&db=Protein&list_uids=4507685&dopt=GenPept&RID=TFMYP1MP01N&log$=prottop&blast_rank=48) | short transient receptor potential channel 1 [Homo sapiens] | [19.3](http://blast.ncbi.nlm.nih.gov/Blast.cgi#4507685%234507685) | 19.3 | 66% | 241 |
| [NP_620686.1](http://www.ncbi.nlm.nih.gov/entrez/query.fcgi?cmd=Retrieve&db=Protein&list_uids=21265058&dopt=GenPept&RID=TFMYP1MP01N&log$=prottop&blast_rank=49) | a disintegrin-like and metalloprotease (reprolysin type) with thrombospondin type 1 motif, 15 preproprotein [Homo sapiens] | [19.3](http://blast.ncbi.nlm.nih.gov/Blast.cgi#21265058%2321265058) | 19.3 | 55% | 241 |
| [NP_057083.2](http://www.ncbi.nlm.nih.gov/entrez/query.fcgi?cmd=Retrieve&db=Protein&list_uids=21361519&dopt=GenPept&RID=TFMYP1MP01N&log$=prottop&blast_rank=50) | adiponectin receptor protein 1 [Homo sapiens] >ref|NP_001121159.1| adiponectin receptor protein 1 [Homo sapiens] | [19.3](http://blast.ncbi.nlm.nih.gov/Blast.cgi#21361519%2321361519) | 19.3 | 66% | 241 |
| [NP_065392.1](http://www.ncbi.nlm.nih.gov/entrez/query.fcgi?cmd=Retrieve&db=Protein&list_uids=24308201&dopt=GenPept&RID=TFMYP1MP01N&log$=prottop&blast_rank=51) | adipocyte plasma membrane-associated protein [Homo sapiens] | [19.3](http://blast.ncbi.nlm.nih.gov/Blast.cgi#24308201%2324308201) | 19.3 | 77% | 241 |
| [NP_116221.3](http://www.ncbi.nlm.nih.gov/entrez/query.fcgi?cmd=Retrieve&db=Protein&list_uids=239787919&dopt=GenPept&RID=TFMYP1MP01N&log$=prottop&blast_rank=52) | low-density lipoprotein receptor-related protein 11 precursor [Homo sapiens] | [19.3](http://blast.ncbi.nlm.nih.gov/Blast.cgi#239787919%23239787919) | 19.3 | 66% | 241 |
| [NP_588614.1](http://www.ncbi.nlm.nih.gov/entrez/query.fcgi?cmd=Retrieve&db=Protein&list_uids=19115964&dopt=GenPept&RID=TFMYP1MP01N&log$=prottop&blast_rank=53) | phospholipase C delta 3 [Homo sapiens] | [19.3](http://blast.ncbi.nlm.nih.gov/Blast.cgi#19115964%2319115964) | 19.3 | 66% | 241 |
| [NP_001164645.1](http://www.ncbi.nlm.nih.gov/entrez/query.fcgi?cmd=Retrieve&db=Protein&list_uids=283837934&dopt=GenPept&RID=TFMYP1MP01N&log$=prottop&blast_rank=54) | CX3C chemokine receptor 1 isoform a [Homo sapiens] | [18.9](http://blast.ncbi.nlm.nih.gov/Blast.cgi#283837934%23283837934) | 18.9 | 66% | 324 |
| [XP_001725523.1](http://www.ncbi.nlm.nih.gov/entrez/query.fcgi?cmd=Retrieve&db=Protein&list_uids=169169865&dopt=GenPept&RID=TFMYP1MP01N&log$=prottop&blast_rank=55) | PREDICTED: hypothetical protein LOC285754 [Homo sapiens] | [18.9](http://blast.ncbi.nlm.nih.gov/Blast.cgi#169169865%23169169865) | 18.9 | 77% | 324 |
| [XP_001722448.1](http://www.ncbi.nlm.nih.gov/entrez/query.fcgi?cmd=Retrieve&db=Protein&list_uids=169169319&dopt=GenPept&RID=TFMYP1MP01N&log$=prottop&blast_rank=56) | PREDICTED: hypothetical protein LOC285754 [Homo sapiens] | [18.9](http://blast.ncbi.nlm.nih.gov/Blast.cgi#169169319%23169169319) | 18.9 | 77% | 324 |
| [NP_001008781.2](http://www.ncbi.nlm.nih.gov/entrez/query.fcgi?cmd=Retrieve&db=Protein&list_uids=148886692&dopt=GenPept&RID=TFMYP1MP01N&log$=prottop&blast_rank=57) | protocadherin Fat 3 [Homo sapiens] | [18.9](http://blast.ncbi.nlm.nih.gov/Blast.cgi#148886692%23148886692) | 33.1 | 66% | 324 |
| [NP_055160.2](http://www.ncbi.nlm.nih.gov/entrez/query.fcgi?cmd=Retrieve&db=Protein&list_uids=120587019&dopt=GenPept&RID=TFMYP1MP01N&log$=prottop&blast_rank=58) | zinc finger protein 318 [Homo sapiens] | [18.9](http://blast.ncbi.nlm.nih.gov/Blast.cgi#120587019%23120587019) | 18.9 | 77% | 324 |
| [XP_001726182.1](http://www.ncbi.nlm.nih.gov/entrez/query.fcgi?cmd=Retrieve&db=Protein&list_uids=169168870&dopt=GenPept&RID=TFMYP1MP01N&log$=prottop&blast_rank=59) | PREDICTED: hypothetical protein LOC285754 [Homo sapiens] | [18.9](http://blast.ncbi.nlm.nih.gov/Blast.cgi#169168870%23169168870) | 18.9 | 77% | 324 |
| [NP_076933.3](http://www.ncbi.nlm.nih.gov/entrez/query.fcgi?cmd=Retrieve&db=Protein&list_uids=226442763&dopt=GenPept&RID=TFMYP1MP01N&log$=prottop&blast_rank=60) | prenylcysteine oxidase-like precursor [Homo sapiens] | [18.9](http://blast.ncbi.nlm.nih.gov/Blast.cgi#226442763%23226442763) | 18.9 | 66% | 324 |
| [NP_060250.2](http://www.ncbi.nlm.nih.gov/entrez/query.fcgi?cmd=Retrieve&db=Protein&list_uids=54112403&dopt=GenPept&RID=TFMYP1MP01N&log$=prottop&blast_rank=61) | chromodomain-helicase-DNA-binding protein 7 [Homo sapiens] | [18.9](http://blast.ncbi.nlm.nih.gov/Blast.cgi#54112403%2354112403) | 49.8 | 88% | 324 |
| [NP_008870.2](http://www.ncbi.nlm.nih.gov/entrez/query.fcgi?cmd=Retrieve&db=Protein&list_uids=154689780&dopt=GenPept&RID=TFMYP1MP01N&log$=prottop&blast_rank=62) | son of sevenless homolog 2 [Homo sapiens] | [18.9](http://blast.ncbi.nlm.nih.gov/Blast.cgi#154689780%23154689780) | 18.9 | 66% | 324 |
| [NP_899631.1](http://www.ncbi.nlm.nih.gov/entrez/query.fcgi?cmd=Retrieve&db=Protein&list_uids=34452713&dopt=GenPept&RID=TFMYP1MP01N&log$=prottop&blast_rank=63) | calcium-dependent secretion activator 1 isoform 2 [Homo sapiens] | [18.9](http://blast.ncbi.nlm.nih.gov/Blast.cgi#34452713%2334452713) | 18.9 | 88% | 324 |
| [NP_899630.1](http://www.ncbi.nlm.nih.gov/entrez/query.fcgi?cmd=Retrieve&db=Protein&list_uids=34452711&dopt=GenPept&RID=TFMYP1MP01N&log$=prottop&blast_rank=64) | calcium-dependent secretion activator 1 isoform 3 [Homo sapiens] | [18.9](http://blast.ncbi.nlm.nih.gov/Blast.cgi#34452711%2334452711) | 18.9 | 88% | 324 |
| [NP_056068.1](http://www.ncbi.nlm.nih.gov/entrez/query.fcgi?cmd=Retrieve&db=Protein&list_uids=54792094&dopt=GenPept&RID=TFMYP1MP01N&log$=prottop&blast_rank=65) | WSC domain-containing protein 1 [Homo sapiens] | [18.9](http://blast.ncbi.nlm.nih.gov/Blast.cgi#54792094%2354792094) | 18.9 | 77% | 324 |
| [NP_005096.1](http://www.ncbi.nlm.nih.gov/entrez/query.fcgi?cmd=Retrieve&db=Protein&list_uids=4826972&dopt=GenPept&RID=TFMYP1MP01N&log$=prottop&blast_rank=66) | RNA-binding protein 8A [Homo sapiens] | [18.9](http://blast.ncbi.nlm.nih.gov/Blast.cgi#4826972%234826972) | 18.9 | 66% | 324 |
| [NP_005192.1](http://www.ncbi.nlm.nih.gov/entrez/query.fcgi?cmd=Retrieve&db=Protein&list_uids=4885121&dopt=GenPept&RID=TFMYP1MP01N&log$=prottop&blast_rank=67) | C-C chemokine receptor type 8 [Homo sapiens] | [18.9](http://blast.ncbi.nlm.nih.gov/Blast.cgi#4885121%234885121) | 18.9 | 66% | 324 |
| [NP_055174.1](http://www.ncbi.nlm.nih.gov/entrez/query.fcgi?cmd=Retrieve&db=Protein&list_uids=7657419&dopt=GenPept&RID=TFMYP1MP01N&log$=prottop&blast_rank=68) | opticin precursor [Homo sapiens] | [18.9](http://blast.ncbi.nlm.nih.gov/Blast.cgi#7657419%237657419) | 18.9 | 66% | 324 |
| [NP_001116513.2](http://www.ncbi.nlm.nih.gov/entrez/query.fcgi?cmd=Retrieve&db=Protein&list_uids=183979980&dopt=GenPept&RID=TFMYP1MP01N&log$=prottop&blast_rank=69) | C-C chemokine receptor type 2 isoform A [Homo sapiens] | [18.9](http://blast.ncbi.nlm.nih.gov/Blast.cgi#183979980%23183979980) | 18.9 | 66% | 324 |
| [NP_001328.1](http://www.ncbi.nlm.nih.gov/entrez/query.fcgi?cmd=Retrieve&db=Protein&list_uids=4503171&dopt=GenPept&RID=TFMYP1MP01N&log$=prottop&blast_rank=70) | CX3C chemokine receptor 1 isoform b [Homo sapiens] >ref|NP_001164642.1| CX3C chemokine receptor 1 isoform b [Homo sapiens] >ref|NP_001164643.1| CX3C chemokine receptor 1 isoform b [Homo sapiens] | [18.9](http://blast.ncbi.nlm.nih.gov/Blast.cgi#4503171%234503171) | 18.9 | 66% | 324 |
| [NP_001829.1](http://www.ncbi.nlm.nih.gov/entrez/query.fcgi?cmd=Retrieve&db=Protein&list_uids=4502641&dopt=GenPept&RID=TFMYP1MP01N&log$=prottop&blast_rank=71) | chemokine (C-C motif) receptor 7 precursor [Homo sapiens] | [18.9](http://blast.ncbi.nlm.nih.gov/Blast.cgi#4502641%234502641) | 18.9 | 66% | 324 |
| [NP_000072.2](http://www.ncbi.nlm.nih.gov/entrez/query.fcgi?cmd=Retrieve&db=Protein&list_uids=54292123&dopt=GenPept&RID=TFMYP1MP01N&log$=prottop&blast_rank=72) | lysosomal-trafficking regulator [Homo sapiens] | [18.9](http://blast.ncbi.nlm.nih.gov/Blast.cgi#54292123%2354292123) | 30.5 | 66% | 324 |
| [NP_009121.1](http://www.ncbi.nlm.nih.gov/entrez/query.fcgi?cmd=Retrieve&db=Protein&list_uids=6005824&dopt=GenPept&RID=TFMYP1MP01N&log$=prottop&blast_rank=73) | SEC23-interacting protein [Homo sapiens] | [18.9](http://blast.ncbi.nlm.nih.gov/Blast.cgi#6005824%236005824) | 18.9 | 77% | 324 |
| [NP_001116868.1](http://www.ncbi.nlm.nih.gov/entrez/query.fcgi?cmd=Retrieve&db=Protein&list_uids=183979982&dopt=GenPept&RID=TFMYP1MP01N&log$=prottop&blast_rank=74) | C-C chemokine receptor type 2 isoform B [Homo sapiens] | [18.9](http://blast.ncbi.nlm.nih.gov/Blast.cgi#183979982%23183979982) | 18.9 | 66% | 324 |
| [NP_003707.2](http://www.ncbi.nlm.nih.gov/entrez/query.fcgi?cmd=Retrieve&db=Protein&list_uids=34452715&dopt=GenPept&RID=TFMYP1MP01N&log$=prottop&blast_rank=75) | calcium-dependent secretion activator 1 isoform 1 [Homo sapiens] | [18.9](http://blast.ncbi.nlm.nih.gov/Blast.cgi#34452715%2334452715) | 18.9 | 88% | 324 |
| [NP_001548.1](http://www.ncbi.nlm.nih.gov/entrez/query.fcgi?cmd=Retrieve&db=Protein&list_uids=4504683&dopt=GenPept&RID=TFMYP1MP01N&log$=prottop&blast_rank=76) | C-X-C chemokine receptor type 2 [Homo sapiens] >ref|NP_001161770.1| C-X-C chemokine receptor type 2 [Homo sapiens] | [18.9](http://blast.ncbi.nlm.nih.gov/Blast.cgi#4504683%234504683) | 18.9 | 66% | 324 |
| [NP_005499.1](http://www.ncbi.nlm.nih.gov/entrez/query.fcgi?cmd=Retrieve&db=Protein&list_uids=5031627&dopt=GenPept&RID=TFMYP1MP01N&log$=prottop&blast_rank=77) | C-C chemokine receptor type 4 [Homo sapiens] | [18.9](http://blast.ncbi.nlm.nih.gov/Blast.cgi#5031627%235031627) | 18.9 | 66% | 324 |
| [NP_000570.1](http://www.ncbi.nlm.nih.gov/entrez/query.fcgi?cmd=Retrieve&db=Protein&list_uids=4502639&dopt=GenPept&RID=TFMYP1MP01N&log$=prottop&blast_rank=78) | chemokine (C-C motif) receptor 5 [Homo sapiens] >ref|NP_001093638.1| chemokine (C-C motif) receptor 5 [Homo sapiens] | [18.9](http://blast.ncbi.nlm.nih.gov/Blast.cgi#4502639%234502639) | 33.1 | 66% | 324 |
| [NP_787125.1](http://www.ncbi.nlm.nih.gov/entrez/query.fcgi?cmd=Retrieve&db=Protein&list_uids=28872756&dopt=GenPept&RID=TFMYP1MP01N&log$=prottop&blast_rank=79) | fibroblast growth factor 14 isoform 1B [Homo sapiens] | [18.9](http://blast.ncbi.nlm.nih.gov/Blast.cgi#28872756%2328872756) | 18.9 | 77% | 324 |
| [NP_004358.2](http://www.ncbi.nlm.nih.gov/entrez/query.fcgi?cmd=Retrieve&db=Protein&list_uids=37187860&dopt=GenPept&RID=TFMYP1MP01N&log$=prottop&blast_rank=80) | C-C chemokine receptor type 6 [Homo sapiens] >ref|NP_113597.2| C-C chemokine receptor type 6 [Homo sapiens] | [18.9](http://blast.ncbi.nlm.nih.gov/Blast.cgi#37187860%2337187860) | 18.9 | 66% | 324 |
| [NP_005624.2](http://www.ncbi.nlm.nih.gov/entrez/query.fcgi?cmd=Retrieve&db=Protein&list_uids=15529996&dopt=GenPept&RID=TFMYP1MP01N&log$=prottop&blast_rank=81) | son of sevenless homolog 1 [Homo sapiens] | [18.9](http://blast.ncbi.nlm.nih.gov/Blast.cgi#15529996%2315529996) | 18.9 | 66% | 324 |
| [NP_000625.1](http://www.ncbi.nlm.nih.gov/entrez/query.fcgi?cmd=Retrieve&db=Protein&list_uids=4504681&dopt=GenPept&RID=TFMYP1MP01N&log$=prottop&blast_rank=82) | C-X-C chemokine receptor type 1 [Homo sapiens] | [18.9](http://blast.ncbi.nlm.nih.gov/Blast.cgi#4504681%234504681) | 18.9 | 66% | 324 |
| [NP_055472.1](http://www.ncbi.nlm.nih.gov/entrez/query.fcgi?cmd=Retrieve&db=Protein&list_uids=24307961&dopt=GenPept&RID=TFMYP1MP01N&log$=prottop&blast_rank=83) | hypothetical protein LOC9675 [Homo sapiens] | [18.9](http://blast.ncbi.nlm.nih.gov/Blast.cgi#24307961%2324307961) | 18.9 | 66% | 324 |
| [NP_006779.1](http://www.ncbi.nlm.nih.gov/entrez/query.fcgi?cmd=Retrieve&db=Protein&list_uids=5803145&dopt=GenPept&RID=TFMYP1MP01N&log$=prottop&blast_rank=84) | ralA binding protein 1 [Homo sapiens] | [18.9](http://blast.ncbi.nlm.nih.gov/Blast.cgi#5803145%235803145) | 33.1 | 88% | 324 |
| [NP_001165888.1](http://www.ncbi.nlm.nih.gov/entrez/query.fcgi?cmd=Retrieve&db=Protein&list_uids=289547204&dopt=GenPept&RID=TFMYP1MP01N&log$=prottop&blast_rank=85) | inward rectifier potassium channel 13 isoform 3 [Homo sapiens] | [18.5](http://blast.ncbi.nlm.nih.gov/Blast.cgi#289547204%23289547204) | 18.5 | 66% | 434 |
| [NP_065927.1](http://www.ncbi.nlm.nih.gov/entrez/query.fcgi?cmd=Retrieve&db=Protein&list_uids=224458284&dopt=GenPept&RID=TFMYP1MP01N&log$=prottop&blast_rank=86) | rho GTPase-activating protein 23 [Homo sapiens] | [18.5](http://blast.ncbi.nlm.nih.gov/Blast.cgi#224458284%23224458284) | 32.7 | 77% | 434 |
| [NP_112190.2](http://www.ncbi.nlm.nih.gov/entrez/query.fcgi?cmd=Retrieve&db=Protein&list_uids=188497689&dopt=GenPept&RID=TFMYP1MP01N&log$=prottop&blast_rank=87) | DNA replication factor Cdt1 [Homo sapiens] | [18.5](http://blast.ncbi.nlm.nih.gov/Blast.cgi#188497689%23188497689) | 18.5 | 77% | 434 |
| [NP_037493.3](http://www.ncbi.nlm.nih.gov/entrez/query.fcgi?cmd=Retrieve&db=Protein&list_uids=156547039&dopt=GenPept&RID=TFMYP1MP01N&log$=prottop&blast_rank=88) | zinc finger protein 223 [Homo sapiens] | [18.5](http://blast.ncbi.nlm.nih.gov/Blast.cgi#156547039%23156547039) | 18.5 | 77% | 434 |
| [NP_008833.1](http://www.ncbi.nlm.nih.gov/entrez/query.fcgi?cmd=Retrieve&db=Protein&list_uids=5902024&dopt=GenPept&RID=TFMYP1MP01N&log$=prottop&blast_rank=89) | paired mesoderm homeobox protein 1 isoform pmx-1a [Homo sapiens] | [18.5](http://blast.ncbi.nlm.nih.gov/Blast.cgi#5902024%235902024) | 18.5 | 66% | 434 |
| [NP_001157936.1](http://www.ncbi.nlm.nih.gov/entrez/query.fcgi?cmd=Retrieve&db=Protein&list_uids=256600212&dopt=GenPept&RID=TFMYP1MP01N&log$=prottop&blast_rank=90) | tripartite motif-containing protein 43B [Homo sapiens] | [18.5](http://blast.ncbi.nlm.nih.gov/Blast.cgi#256600212%23256600212) | 18.5 | 77% | 434 |
| [NP_599025.2](http://www.ncbi.nlm.nih.gov/entrez/query.fcgi?cmd=Retrieve&db=Protein&list_uids=94721255&dopt=GenPept&RID=TFMYP1MP01N&log$=prottop&blast_rank=91) | solute carrier family 26 member 6 isoform 2 [Homo sapiens] | [18.5](http://blast.ncbi.nlm.nih.gov/Blast.cgi#94721255%2394721255) | 18.5 | 88% | 434 |
| [NP_005144.2](http://www.ncbi.nlm.nih.gov/entrez/query.fcgi?cmd=Retrieve&db=Protein&list_uids=119220605&dopt=GenPept&RID=TFMYP1MP01N&log$=prottop&blast_rank=92) | ubiquitin specific protease 10 [Homo sapiens] | [18.5](http://blast.ncbi.nlm.nih.gov/Blast.cgi#119220605%23119220605) | 18.5 | 66% | 434 |
| [NP_002208.3](http://www.ncbi.nlm.nih.gov/entrez/query.fcgi?cmd=Retrieve&db=Protein&list_uids=133925809&dopt=GenPept&RID=TFMYP1MP01N&log$=prottop&blast_rank=93) | inter-alpha (globulin) inhibitor H3 preproprotein [Homo sapiens] | [18.5](http://blast.ncbi.nlm.nih.gov/Blast.cgi#133925809%23133925809) | 18.5 | 77% | 434 |
| [NP_001035544.1](http://www.ncbi.nlm.nih.gov/entrez/query.fcgi?cmd=Retrieve&db=Protein&list_uids=94721259&dopt=GenPept&RID=TFMYP1MP01N&log$=prottop&blast_rank=94) | solute carrier family 26 member 6 isoform 4 [Homo sapiens] | [18.5](http://blast.ncbi.nlm.nih.gov/Blast.cgi#94721259%2394721259) | 18.5 | 88% | 434 |
| [NP_075062.2](http://www.ncbi.nlm.nih.gov/entrez/query.fcgi?cmd=Retrieve&db=Protein&list_uids=94721253&dopt=GenPept&RID=TFMYP1MP01N&log$=prottop&blast_rank=95) | solute carrier family 26 member 6 isoform 1 [Homo sapiens] | [18.5](http://blast.ncbi.nlm.nih.gov/Blast.cgi#94721253%2394721253) | 18.5 | 88% | 434 |
| [NP_002233.2](http://www.ncbi.nlm.nih.gov/entrez/query.fcgi?cmd=Retrieve&db=Protein&list_uids=156119627&dopt=GenPept&RID=TFMYP1MP01N&log$=prottop&blast_rank=96) | inward rectifier potassium channel 13 isoform 1 [Homo sapiens] | [18.5](http://blast.ncbi.nlm.nih.gov/Blast.cgi#156119627%23156119627) | 18.5 | 66% | 434 |
| [NP_001816.2](http://www.ncbi.nlm.nih.gov/entrez/query.fcgi?cmd=Retrieve&db=Protein&list_uids=153251982&dopt=GenPept&RID=TFMYP1MP01N&log$=prottop&blast_rank=97) | sarcomeric mitochondrial creatine kinase precursor [Homo sapiens] >ref|NP_001093205.1| sarcomeric mitochondrial creatine kinase precursor [Homo sapiens] >ref|NP_001093206.1| sarcomeric mitochondrial creatine kinase precursor [Homo sapiens] | [18.5](http://blast.ncbi.nlm.nih.gov/Blast.cgi#153251982%23153251982) | 18.5 | 66% | 434 |
| [NP_073207.1](http://www.ncbi.nlm.nih.gov/entrez/query.fcgi?cmd=Retrieve&db=Protein&list_uids=12707577&dopt=GenPept&RID=TFMYP1MP01N&log$=prottop&blast_rank=98) | paired mesoderm homeobox protein 1 isoform pmx-1b [Homo sapiens] | [18.5](http://blast.ncbi.nlm.nih.gov/Blast.cgi#12707577%2312707577) | 18.5 | 66% | 434 |
| [NP_602298.2](http://www.ncbi.nlm.nih.gov/entrez/query.fcgi?cmd=Retrieve&db=Protein&list_uids=94721257&dopt=GenPept&RID=TFMYP1MP01N&log$=prottop&blast_rank=99) | solute carrier family 26 member 6 isoform 3 [Homo sapiens] | [18.5](http://blast.ncbi.nlm.nih.gov/Blast.cgi#94721257%2394721257) | 18.5 | 88% | 434 |
| [XP_002347488.1](http://www.ncbi.nlm.nih.gov/entrez/query.fcgi?cmd=Retrieve&db=Protein&list_uids=239750703&dopt=GenPept&RID=TFMYP1MP01N&log$=prottop&blast_rank=100) | PREDICTED: hypothetical protein XP_002347488 [Homo sapiens] >ref|XP_002344842.1| PREDICTED: hypothetical protein [Homo sapiens] | [18.0](http://blast.ncbi.nlm.nih.gov/Blast.cgi#239750703%23239750703) | 18.0 | 77% | 583 |

| **Accession** | **Proteins with a match to DPTVSESS peptide** | **[Max score](http://blast.ncbi.nlm.nih.gov/Blast.cgi?CMD=Get&ALIGNMENTS=100&ALIGNMENT_VIEW=Pairwise&CDD_SEARCH_STATE=1&DATABASE_SORT=0&DESCRIPTIONS=100&ENTREZ_QUERY=txid9606 %5BORGN%5D&FIRST_QUERY_NUM=0&FORMAT_OBJECT=Alignment&FORMAT_PAGE_TARGET=&FORMAT_TYPE=HTML&GET_SEQUENCE=yes&I_THRESH=&MASK_CHAR=2&MASK_COLOR=1&NEW_VIEW=yes&NUM_OVERVIEW=100&OLD_BLAST=false&PAGE=Proteins&QUERY_INDEX=0&QUERY_NUMBER=0&RESULTS_PAGE_TARGET=&RID=TFN1Y92J01S&SHOW_LINKOUT=yes&SHOW_OVERVIEW=yes&STEP_NUMBER=&WORD_SIZE=2&DISPLAY_SORT=1&HSP_SORT=1" \l "sort_mark)** | **[Total score](http://blast.ncbi.nlm.nih.gov/Blast.cgi?CMD=Get&ALIGNMENTS=100&ALIGNMENT_VIEW=Pairwise&CDD_SEARCH_STATE=1&DATABASE_SORT=0&DESCRIPTIONS=100&ENTREZ_QUERY=txid9606 %5BORGN%5D&FIRST_QUERY_NUM=0&FORMAT_OBJECT=Alignment&FORMAT_PAGE_TARGET=&FORMAT_TYPE=HTML&GET_SEQUENCE=yes&I_THRESH=&MASK_CHAR=2&MASK_COLOR=1&NEW_VIEW=yes&NUM_OVERVIEW=100&OLD_BLAST=false&PAGE=Proteins&QUERY_INDEX=0&QUERY_NUMBER=0&RESULTS_PAGE_TARGET=&RID=TFN1Y92J01S&SHOW_LINKOUT=yes&SHOW_OVERVIEW=yes&STEP_NUMBER=&WORD_SIZE=2&DISPLAY_SORT=2&HSP_SORT=1" \l "sort_mark)** | **[Query coverage](http://blast.ncbi.nlm.nih.gov/Blast.cgi?CMD=Get&ALIGNMENTS=100&ALIGNMENT_VIEW=Pairwise&CDD_SEARCH_STATE=1&DATABASE_SORT=0&DESCRIPTIONS=100&ENTREZ_QUERY=txid9606 %5BORGN%5D&FIRST_QUERY_NUM=0&FORMAT_OBJECT=Alignment&FORMAT_PAGE_TARGET=&FORMAT_TYPE=HTML&GET_SEQUENCE=yes&I_THRESH=&MASK_CHAR=2&MASK_COLOR=1&NEW_VIEW=yes&NUM_OVERVIEW=100&OLD_BLAST=false&PAGE=Proteins&QUERY_INDEX=0&QUERY_NUMBER=0&RESULTS_PAGE_TARGET=&RID=TFN1Y92J01S&SHOW_LINKOUT=yes&SHOW_OVERVIEW=yes&STEP_NUMBER=&WORD_SIZE=2&DISPLAY_SORT=4&HSP_SORT=0" \l "sort_mark)** | **[E value](http://blast.ncbi.nlm.nih.gov/Blast.cgi?CMD=Get&ALIGNMENTS=100&ALIGNMENT_VIEW=Pairwise&CDD_SEARCH_STATE=1&DATABASE_SORT=0&DESCRIPTIONS=100&ENTREZ_QUERY=txid9606 %5BORGN%5D&FIRST_QUERY_NUM=0&FORMAT_OBJECT=Alignment&FORMAT_PAGE_TARGET=&FORMAT_TYPE=HTML&GET_SEQUENCE=yes&I_THRESH=&MASK_CHAR=2&MASK_COLOR=1&NEW_VIEW=yes&NUM_OVERVIEW=100&OLD_BLAST=false&PAGE=Proteins&QUERY_INDEX=0&QUERY_NUMBER=0&RESULTS_PAGE_TARGET=&RID=TFN1Y92J01S&SHOW_LINKOUT=yes&SHOW_OVERVIEW=yes&STEP_NUMBER=&WORD_SIZE=2&DISPLAY_SORT=0&HSP_SORT=0" \l "sort_mark)** |
| --- | --- | --- | --- | --- | --- |
| [NP_060049.2](http://www.ncbi.nlm.nih.gov/entrez/query.fcgi?cmd=Retrieve&db=Protein&list_uids=148539844&dopt=GenPept&RID=TFN1Y92J01S&log$=prottop&blast_rank=1) | deleted in malignant brain tumors 1 isoform c precursor [Homo sapiens] | [21.0](http://blast.ncbi.nlm.nih.gov/Blast.cgi#148539844%23148539844) | 141 | 100% | 66 |
| [NP_004397.2](http://www.ncbi.nlm.nih.gov/entrez/query.fcgi?cmd=Retrieve&db=Protein&list_uids=148539840&dopt=GenPept&RID=TFN1Y92J01S&log$=prottop&blast_rank=2) | deleted in malignant brain tumors 1 isoform a precursor [Homo sapiens] | [21.0](http://blast.ncbi.nlm.nih.gov/Blast.cgi#148539840%23148539840) | 95.5 | 100% | 66 |
| [NP_015568.2](http://www.ncbi.nlm.nih.gov/entrez/query.fcgi?cmd=Retrieve&db=Protein&list_uids=148539842&dopt=GenPept&RID=TFN1Y92J01S&log$=prottop&blast_rank=3) | deleted in malignant brain tumors 1 isoform b precursor [Homo sapiens] | [21.0](http://blast.ncbi.nlm.nih.gov/Blast.cgi#148539842%23148539842) | 141 | 100% | 66 |
| [NP_919227.2](http://www.ncbi.nlm.nih.gov/entrez/query.fcgi?cmd=Retrieve&db=Protein&list_uids=57977305&dopt=GenPept&RID=TFN1Y92J01S&log$=prottop&blast_rank=4) | probable G-protein coupled receptor 151 [Homo sapiens] | [21.0](http://blast.ncbi.nlm.nih.gov/Blast.cgi#57977305%2357977305) | 21.0 | 75% | 66 |
| [NP_001139808.1](http://www.ncbi.nlm.nih.gov/entrez/query.fcgi?cmd=Retrieve&db=Protein&list_uids=226437592&dopt=GenPept&RID=TFN1Y92J01S&log$=prottop&blast_rank=5) | transmembrane protein 114 [Homo sapiens] | [20.2](http://blast.ncbi.nlm.nih.gov/Blast.cgi#226437592%23226437592) | 20.2 | 75% | 119 |
| [NP_055598.1](http://www.ncbi.nlm.nih.gov/entrez/query.fcgi?cmd=Retrieve&db=Protein&list_uids=7661858&dopt=GenPept&RID=TFN1Y92J01S&log$=prottop&blast_rank=6) | rho GTPase-activating protein 11A isoform 1 [Homo sapiens] | [20.2](http://blast.ncbi.nlm.nih.gov/Blast.cgi#7661858%237661858) | 20.2 | 75% | 119 |
| [NP_064617.2](http://www.ncbi.nlm.nih.gov/entrez/query.fcgi?cmd=Retrieve&db=Protein&list_uids=22726189&dopt=GenPept&RID=TFN1Y92J01S&log$=prottop&blast_rank=7) | proteasome assembly chaperone 2 [Homo sapiens] | [20.2](http://blast.ncbi.nlm.nih.gov/Blast.cgi#22726189%2322726189) | 20.2 | 87% | 119 |
| [NP_055759.3](http://www.ncbi.nlm.nih.gov/entrez/query.fcgi?cmd=Retrieve&db=Protein&list_uids=57242755&dopt=GenPept&RID=TFN1Y92J01S&log$=prottop&blast_rank=8) | calsyntenin-1 isoform 2 [Homo sapiens] | [19.7](http://blast.ncbi.nlm.nih.gov/Blast.cgi#57242755%2357242755) | 33.9 | 100% | 160 |
| [NP_001009566.1](http://www.ncbi.nlm.nih.gov/entrez/query.fcgi?cmd=Retrieve&db=Protein&list_uids=57242757&dopt=GenPept&RID=TFN1Y92J01S&log$=prottop&blast_rank=9) | calsyntenin-1 isoform 1 [Homo sapiens] | [19.7](http://blast.ncbi.nlm.nih.gov/Blast.cgi#57242757%2357242757) | 33.9 | 100% | 160 |
| [NP_065952.2](http://www.ncbi.nlm.nih.gov/entrez/query.fcgi?cmd=Retrieve&db=Protein&list_uids=221139764&dopt=GenPept&RID=TFN1Y92J01S&log$=prottop&blast_rank=10) | PHD and RING finger domain-containing protein 1 [Homo sapiens] | [19.3](http://blast.ncbi.nlm.nih.gov/Blast.cgi#221139764%23221139764) | 33.5 | 100% | 214 |
| [NP_114157.3](http://www.ncbi.nlm.nih.gov/entrez/query.fcgi?cmd=Retrieve&db=Protein&list_uids=221219020&dopt=GenPept&RID=TFN1Y92J01S&log$=prottop&blast_rank=11) | WD repeat-containing protein 87 [Homo sapiens] | [19.3](http://blast.ncbi.nlm.nih.gov/Blast.cgi#221219020%23221219020) | 31.0 | 87% | 214 |
| [NP_056164.1](http://www.ncbi.nlm.nih.gov/entrez/query.fcgi?cmd=Retrieve&db=Protein&list_uids=54792088&dopt=GenPept&RID=TFN1Y92J01S&log$=prottop&blast_rank=12) | hypothetical protein LOC23506 [Homo sapiens] | [19.3](http://blast.ncbi.nlm.nih.gov/Blast.cgi#54792088%2354792088) | 19.3 | 87% | 214 |
| [NP_065775.1](http://www.ncbi.nlm.nih.gov/entrez/query.fcgi?cmd=Retrieve&db=Protein&list_uids=58331204&dopt=GenPept&RID=TFN1Y92J01S&log$=prottop&blast_rank=13) | ring finger protein 150 precursor [Homo sapiens] | [19.3](http://blast.ncbi.nlm.nih.gov/Blast.cgi#58331204%2358331204) | 19.3 | 100% | 214 |
| [NP_612480.1](http://www.ncbi.nlm.nih.gov/entrez/query.fcgi?cmd=Retrieve&db=Protein&list_uids=39930523&dopt=GenPept&RID=TFN1Y92J01S&log$=prottop&blast_rank=14) | hypothetical protein LOC144097 [Homo sapiens] | [19.3](http://blast.ncbi.nlm.nih.gov/Blast.cgi#39930523%2339930523) | 19.3 | 87% | 214 |
| [XP_001719387.1](http://www.ncbi.nlm.nih.gov/entrez/query.fcgi?cmd=Retrieve&db=Protein&list_uids=169212026&dopt=GenPept&RID=TFN1Y92J01S&log$=prottop&blast_rank=15) | PREDICTED: keratin associated protein [Homo sapiens] | [18.9](http://blast.ncbi.nlm.nih.gov/Blast.cgi#169212026%23169212026) | 18.9 | 100% | 288 |
| [NP_757366.2](http://www.ncbi.nlm.nih.gov/entrez/query.fcgi?cmd=Retrieve&db=Protein&list_uids=148833504&dopt=GenPept&RID=TFN1Y92J01S&log$=prottop&blast_rank=16) | pro-interleukin-16 isoform 2 [Homo sapiens] | [18.9](http://blast.ncbi.nlm.nih.gov/Blast.cgi#148833504%23148833504) | 35.6 | 87% | 288 |
| [NP_001165599.1](http://www.ncbi.nlm.nih.gov/entrez/query.fcgi?cmd=Retrieve&db=Protein&list_uids=289063395&dopt=GenPept&RID=TFN1Y92J01S&log$=prottop&blast_rank=17) | pro-interleukin-16 isoform 3 [Homo sapiens] | [18.9](http://blast.ncbi.nlm.nih.gov/Blast.cgi#289063395%23289063395) | 35.6 | 87% | 288 |
| [NP_001073903.1](http://www.ncbi.nlm.nih.gov/entrez/query.fcgi?cmd=Retrieve&db=Protein&list_uids=122937267&dopt=GenPept&RID=TFN1Y92J01S&log$=prottop&blast_rank=18) | serine/threonine-protein kinase LMTK3 [Homo sapiens] | [18.9](http://blast.ncbi.nlm.nih.gov/Blast.cgi#122937267%23122937267) | 34.8 | 100% | 288 |
| [NP_004504.3](http://www.ncbi.nlm.nih.gov/entrez/query.fcgi?cmd=Retrieve&db=Protein&list_uids=27262655&dopt=GenPept&RID=TFN1Y92J01S&log$=prottop&blast_rank=19) | pro-interleukin-16 isoform 1 [Homo sapiens] | [18.9](http://blast.ncbi.nlm.nih.gov/Blast.cgi#27262655%2327262655) | 18.9 | 87% | 288 |
| [NP_036336.2](http://www.ncbi.nlm.nih.gov/entrez/query.fcgi?cmd=Retrieve&db=Protein&list_uids=156119605&dopt=GenPept&RID=TFN1Y92J01S&log$=prottop&blast_rank=20) | general transcription factor 3C polypeptide 4 [Homo sapiens] | [18.9](http://blast.ncbi.nlm.nih.gov/Blast.cgi#156119605%23156119605) | 18.9 | 75% | 288 |
| [NP_065983.1](http://www.ncbi.nlm.nih.gov/entrez/query.fcgi?cmd=Retrieve&db=Protein&list_uids=20143482&dopt=GenPept&RID=TFN1Y92J01S&log$=prottop&blast_rank=21) | melanoma-associated antigen E1 [Homo sapiens] | [18.9](http://blast.ncbi.nlm.nih.gov/Blast.cgi#20143482%2320143482) | 75.7 | 87% | 288 |
| [NP_001159921.1](http://www.ncbi.nlm.nih.gov/entrez/query.fcgi?cmd=Retrieve&db=Protein&list_uids=262050538&dopt=GenPept&RID=TFN1Y92J01S&log$=prottop&blast_rank=22) | inter-alpha (globulin) inhibitor H4 isoform 2 precursor [Homo sapiens] | [18.5](http://blast.ncbi.nlm.nih.gov/Blast.cgi#262050538%23262050538) | 33.5 | 75% | 386 |
| [NP_001158137.1](http://www.ncbi.nlm.nih.gov/entrez/query.fcgi?cmd=Retrieve&db=Protein&list_uids=257467654&dopt=GenPept&RID=TFN1Y92J01S&log$=prottop&blast_rank=23) | hypothetical protein LOC57670 isoform 2 [Homo sapiens] | [18.5](http://blast.ncbi.nlm.nih.gov/Blast.cgi#257467654%23257467654) | 18.5 | 100% | 386 |
| [NP_065961.2](http://www.ncbi.nlm.nih.gov/entrez/query.fcgi?cmd=Retrieve&db=Protein&list_uids=257467652&dopt=GenPept&RID=TFN1Y92J01S&log$=prottop&blast_rank=24) | hypothetical protein LOC57670 isoform 1 [Homo sapiens] | [18.5](http://blast.ncbi.nlm.nih.gov/Blast.cgi#257467652%23257467652) | 18.5 | 100% | 386 |
| [XP_001722870.1](http://www.ncbi.nlm.nih.gov/entrez/query.fcgi?cmd=Retrieve&db=Protein&list_uids=169215350&dopt=GenPept&RID=TFN1Y92J01S&log$=prottop&blast_rank=25) | PREDICTED: hypothetical protein [Homo sapiens] | [18.5](http://blast.ncbi.nlm.nih.gov/Blast.cgi#169215350%23169215350) | 36.9 | 62% | 386 |
| [XP_001726340.1](http://www.ncbi.nlm.nih.gov/entrez/query.fcgi?cmd=Retrieve&db=Protein&list_uids=169215453&dopt=GenPept&RID=TFN1Y92J01S&log$=prottop&blast_rank=26) | PREDICTED: hypothetical protein [Homo sapiens] | [18.5](http://blast.ncbi.nlm.nih.gov/Blast.cgi#169215453%23169215453) | 36.9 | 62% | 386 |
| [NP_038477.2](http://www.ncbi.nlm.nih.gov/entrez/query.fcgi?cmd=Retrieve&db=Protein&list_uids=91176325&dopt=GenPept&RID=TFN1Y92J01S&log$=prottop&blast_rank=27) | bromodomain adjacent to zinc finger domain protein 2A [Homo sapiens] | [18.5](http://blast.ncbi.nlm.nih.gov/Blast.cgi#91176325%2391176325) | 18.5 | 62% | 386 |
| [NP_059129.3](http://www.ncbi.nlm.nih.gov/entrez/query.fcgi?cmd=Retrieve&db=Protein&list_uids=145275208&dopt=GenPept&RID=TFN1Y92J01S&log$=prottop&blast_rank=28) | myosin-IIIa [Homo sapiens] | [18.5](http://blast.ncbi.nlm.nih.gov/Blast.cgi#145275208%23145275208) | 33.5 | 62% | 386 |
| [NP_002207.2](http://www.ncbi.nlm.nih.gov/entrez/query.fcgi?cmd=Retrieve&db=Protein&list_uids=70778918&dopt=GenPept&RID=TFN1Y92J01S&log$=prottop&blast_rank=29) | inter-alpha globulin inhibitor H2 polypeptide [Homo sapiens] | [18.5](http://blast.ncbi.nlm.nih.gov/Blast.cgi#70778918%2370778918) | 18.5 | 75% | 386 |
| [NP_078853.2](http://www.ncbi.nlm.nih.gov/entrez/query.fcgi?cmd=Retrieve&db=Protein&list_uids=38488692&dopt=GenPept&RID=TFN1Y92J01S&log$=prottop&blast_rank=30) | SH3 domain and tetratricopeptide repeats-containing protein 2 [Homo sapiens] | [18.5](http://blast.ncbi.nlm.nih.gov/Blast.cgi#38488692%2338488692) | 18.5 | 62% | 386 |
| [NP_004823.1](http://www.ncbi.nlm.nih.gov/entrez/query.fcgi?cmd=Retrieve&db=Protein&list_uids=4758484&dopt=GenPept&RID=TFN1Y92J01S&log$=prottop&blast_rank=31) | glutathione S-transferase omega-1 [Homo sapiens] | [18.5](http://blast.ncbi.nlm.nih.gov/Blast.cgi#4758484%234758484) | 18.5 | 62% | 386 |
| [NP_056511.2](http://www.ncbi.nlm.nih.gov/entrez/query.fcgi?cmd=Retrieve&db=Protein&list_uids=15618997&dopt=GenPept&RID=TFN1Y92J01S&log$=prottop&blast_rank=32) | glutathione peroxidase 7 precursor [Homo sapiens] | [18.5](http://blast.ncbi.nlm.nih.gov/Blast.cgi#15618997%2315618997) | 18.5 | 62% | 386 |
| [NP_001429.2](http://www.ncbi.nlm.nih.gov/entrez/query.fcgi?cmd=Retrieve&db=Protein&list_uids=45545433&dopt=GenPept&RID=TFN1Y92J01S&log$=prottop&blast_rank=33) | estrogen-related receptor gamma isoform 1 [Homo sapiens] | [18.5](http://blast.ncbi.nlm.nih.gov/Blast.cgi#45545433%2345545433) | 18.5 | 87% | 386 |
| [NP_005517.1](http://www.ncbi.nlm.nih.gov/entrez/query.fcgi?cmd=Retrieve&db=Protein&list_uids=5031767&dopt=GenPept&RID=TFN1Y92J01S&log$=prottop&blast_rank=34) | heat shock factor protein 1 [Homo sapiens] | [18.5](http://blast.ncbi.nlm.nih.gov/Blast.cgi#5031767%235031767) | 18.5 | 62% | 386 |
| [NP_001242.2](http://www.ncbi.nlm.nih.gov/entrez/query.fcgi?cmd=Retrieve&db=Protein&list_uids=91199548&dopt=GenPept&RID=TFN1Y92J01S&log$=prottop&blast_rank=35) | CD68 antigen isoform A [Homo sapiens] | [18.5](http://blast.ncbi.nlm.nih.gov/Blast.cgi#91199548%2391199548) | 18.5 | 75% | 386 |
| [NP_005119.2](http://www.ncbi.nlm.nih.gov/entrez/query.fcgi?cmd=Retrieve&db=Protein&list_uids=45827701&dopt=GenPept&RID=TFN1Y92J01S&log$=prottop&blast_rank=36) | protein dopey-2 [Homo sapiens] | [18.5](http://blast.ncbi.nlm.nih.gov/Blast.cgi#45827701%2345827701) | 18.5 | 62% | 386 |
| [NP_002209.2](http://www.ncbi.nlm.nih.gov/entrez/query.fcgi?cmd=Retrieve&db=Protein&list_uids=31542984&dopt=GenPept&RID=TFN1Y92J01S&log$=prottop&blast_rank=37) | inter-alpha (globulin) inhibitor H4 isoform 1 precursor [Homo sapiens] | [18.5](http://blast.ncbi.nlm.nih.gov/Blast.cgi#31542984%2331542984) | 33.5 | 75% | 386 |
| [NP_004053.1](http://www.ncbi.nlm.nih.gov/entrez/query.fcgi?cmd=Retrieve&db=Protein&list_uids=4757956&dopt=GenPept&RID=TFN1Y92J01S&log$=prottop&blast_rank=38) | cadherin 16 precursor [Homo sapiens] | [18.5](http://blast.ncbi.nlm.nih.gov/Blast.cgi#4757956%234757956) | 18.5 | 62% | 386 |
| [NP_996317.1](http://www.ncbi.nlm.nih.gov/entrez/query.fcgi?cmd=Retrieve&db=Protein&list_uids=45545427&dopt=GenPept&RID=TFN1Y92J01S&log$=prottop&blast_rank=39) | estrogen-related receptor gamma isoform 2 [Homo sapiens] >ref|NP_996318.1| estrogen-related receptor gamma isoform 2 [Homo sapiens] >ref|NP_001127757.1| estrogen-related receptor gamma isoform 2 [Homo sapiens] | [18.5](http://blast.ncbi.nlm.nih.gov/Blast.cgi#45545427%2345545427) | 18.5 | 87% | 386 |
| [NP_000536.5](http://www.ncbi.nlm.nih.gov/entrez/query.fcgi?cmd=Retrieve&db=Protein&list_uids=256542297&dopt=GenPept&RID=TFN1Y92J01S&log$=prottop&blast_rank=40) | hepatocyte nuclear factor 1-alpha [Homo sapiens] | [18.5](http://blast.ncbi.nlm.nih.gov/Blast.cgi#256542297%23256542297) | 18.5 | 87% | 386 |
| [NP_085126.2](http://www.ncbi.nlm.nih.gov/entrez/query.fcgi?cmd=Retrieve&db=Protein&list_uids=217416352&dopt=GenPept&RID=TFN1Y92J01S&log$=prottop&blast_rank=41) | A-kinase anchor protein SPHKAP isoform 2 [Homo sapiens] | [18.0](http://blast.ncbi.nlm.nih.gov/Blast.cgi#217416352%23217416352) | 18.0 | 75% | 518 |
| [XP_001717064.1](http://www.ncbi.nlm.nih.gov/entrez/query.fcgi?cmd=Retrieve&db=Protein&list_uids=169216354&dopt=GenPept&RID=TFN1Y92J01S&log$=prottop&blast_rank=42) | PREDICTED: similar to armadillo repeat containing, X-linked 6, partial [Homo sapiens] | [18.0](http://blast.ncbi.nlm.nih.gov/Blast.cgi#169216354%23169216354) | 18.0 | 75% | 518 |
| [XP_001717940.1](http://www.ncbi.nlm.nih.gov/entrez/query.fcgi?cmd=Retrieve&db=Protein&list_uids=169216627&dopt=GenPept&RID=TFN1Y92J01S&log$=prottop&blast_rank=43) | PREDICTED: similar to armadillo repeat containing, X-linked 6 [Homo sapiens] >ref|XP_001713950.1| PREDICTED: similar to armadillo repeat containing, X-linked 6 [Homo sapiens] | [18.0](http://blast.ncbi.nlm.nih.gov/Blast.cgi#169216627%23169216627) | 18.0 | 75% | 518 |
| [NP_775840.3](http://www.ncbi.nlm.nih.gov/entrez/query.fcgi?cmd=Retrieve&db=Protein&list_uids=236459851&dopt=GenPept&RID=TFN1Y92J01S&log$=prottop&blast_rank=44) | ubinuclein-2 [Homo sapiens] | [18.0](http://blast.ncbi.nlm.nih.gov/Blast.cgi#236459851%23236459851) | 30.1 | 87% | 518 |
| [NP_055504.2](http://www.ncbi.nlm.nih.gov/entrez/query.fcgi?cmd=Retrieve&db=Protein&list_uids=154146189&dopt=GenPept&RID=TFN1Y92J01S&log$=prottop&blast_rank=45) | dedicator of cytokinesis protein 10 [Homo sapiens] | [18.0](http://blast.ncbi.nlm.nih.gov/Blast.cgi#154146189%23154146189) | 18.0 | 87% | 518 |
| [NP_004232.2](http://www.ncbi.nlm.nih.gov/entrez/query.fcgi?cmd=Retrieve&db=Protein&list_uids=68342036&dopt=GenPept&RID=TFN1Y92J01S&log$=prottop&blast_rank=46) | probable JmjC domain-containing histone demethylation protein 2C isoform b [Homo sapiens] | [18.0](http://blast.ncbi.nlm.nih.gov/Blast.cgi#68342036%2368342036) | 32.2 | 100% | 518 |
| [NP_001073881.2](http://www.ncbi.nlm.nih.gov/entrez/query.fcgi?cmd=Retrieve&db=Protein&list_uids=148276990&dopt=GenPept&RID=TFN1Y92J01S&log$=prottop&blast_rank=47) | zinc finger and BTB domain-containing protein 38 [Homo sapiens] | [18.0](http://blast.ncbi.nlm.nih.gov/Blast.cgi#148276990%23148276990) | 29.3 | 100% | 518 |
| [NP_060649.3](http://www.ncbi.nlm.nih.gov/entrez/query.fcgi?cmd=Retrieve&db=Protein&list_uids=38261962&dopt=GenPept&RID=TFN1Y92J01S&log$=prottop&blast_rank=48) | activating transcription factor 7-interacting protein 1 [Homo sapiens] | [18.0](http://blast.ncbi.nlm.nih.gov/Blast.cgi#38261962%2338261962) | 47.7 | 100% | 518 |
| [NP_740721.1](http://www.ncbi.nlm.nih.gov/entrez/query.fcgi?cmd=Retrieve&db=Protein&list_uids=25777647&dopt=GenPept&RID=TFN1Y92J01S&log$=prottop&blast_rank=49) | small conductance calcium-activated potassium channel protein 2 isoform b [Homo sapiens] | [18.0](http://blast.ncbi.nlm.nih.gov/Blast.cgi#25777647%2325777647) | 18.0 | 87% | 518 |
| [NP_067627.2](http://www.ncbi.nlm.nih.gov/entrez/query.fcgi?cmd=Retrieve&db=Protein&list_uids=25777645&dopt=GenPept&RID=TFN1Y92J01S&log$=prottop&blast_rank=50) | small conductance calcium-activated potassium channel protein 2 isoform a [Homo sapiens] | [18.0](http://blast.ncbi.nlm.nih.gov/Blast.cgi#25777645%2325777645) | 18.0 | 87% | 518 |
| [NP_116165.1](http://www.ncbi.nlm.nih.gov/entrez/query.fcgi?cmd=Retrieve&db=Protein&list_uids=118600981&dopt=GenPept&RID=TFN1Y92J01S&log$=prottop&blast_rank=51) | probable JmjC domain-containing histone demethylation protein 2C isoform a [Homo sapiens] | [18.0](http://blast.ncbi.nlm.nih.gov/Blast.cgi#118600981%23118600981) | 32.2 | 100% | 518 |
| [NP_001136116.1](http://www.ncbi.nlm.nih.gov/entrez/query.fcgi?cmd=Retrieve&db=Protein&list_uids=217416354&dopt=GenPept&RID=TFN1Y92J01S&log$=prottop&blast_rank=52) | A-kinase anchor protein SPHKAP isoform 1 [Homo sapiens] | [18.0](http://blast.ncbi.nlm.nih.gov/Blast.cgi#217416354%23217416354) | 18.0 | 75% | 518 |
| [NP_060774.2](http://www.ncbi.nlm.nih.gov/entrez/query.fcgi?cmd=Retrieve&db=Protein&list_uids=88703045&dopt=GenPept&RID=TFN1Y92J01S&log$=prottop&blast_rank=53) | proline-rich protein 11 [Homo sapiens] | [18.0](http://blast.ncbi.nlm.nih.gov/Blast.cgi#88703045%2388703045) | 18.0 | 75% | 518 |
| [NP_061857.3](http://www.ncbi.nlm.nih.gov/entrez/query.fcgi?cmd=Retrieve&db=Protein&list_uids=239047414&dopt=GenPept&RID=TFN1Y92J01S&log$=prottop&blast_rank=54) | protein phosphatase Slingshot homolog 1 isoform 1 [Homo sapiens] | [18.0](http://blast.ncbi.nlm.nih.gov/Blast.cgi#239047414%23239047414) | 18.0 | 100% | 518 |
| [NP_941966.1](http://www.ncbi.nlm.nih.gov/entrez/query.fcgi?cmd=Retrieve&db=Protein&list_uids=46250735&dopt=GenPept&RID=TFN1Y92J01S&log$=prottop&blast_rank=55) | keratin-associated protein 10-2 [Homo sapiens] | [18.0](http://blast.ncbi.nlm.nih.gov/Blast.cgi#46250735%2346250735) | 18.0 | 87% | 518 |
| [NP_061880.2](http://www.ncbi.nlm.nih.gov/entrez/query.fcgi?cmd=Retrieve&db=Protein&list_uids=34147335&dopt=GenPept&RID=TFN1Y92J01S&log$=prottop&blast_rank=56) | protein ARMCX6 [Homo sapiens] >ref|NP_001009584.1| protein ARMCX6 [Homo sapiens] | [18.0](http://blast.ncbi.nlm.nih.gov/Blast.cgi#34147335%2334147335) | 18.0 | 75% | 518 |
| [NP_859016.1](http://www.ncbi.nlm.nih.gov/entrez/query.fcgi?cmd=Retrieve&db=Protein&list_uids=32140182&dopt=GenPept&RID=TFN1Y92J01S&log$=prottop&blast_rank=57) | keratin-associated protein 10-10 [Homo sapiens] | [18.0](http://blast.ncbi.nlm.nih.gov/Blast.cgi#32140182%2332140182) | 18.0 | 87% | 518 |
| [NP_588609.1](http://www.ncbi.nlm.nih.gov/entrez/query.fcgi?cmd=Retrieve&db=Protein&list_uids=45387949&dopt=GenPept&RID=TFN1Y92J01S&log$=prottop&blast_rank=58) | RING finger and SPRY domain-containing protein 1 precursor [Homo sapiens] | [18.0](http://blast.ncbi.nlm.nih.gov/Blast.cgi#45387949%2345387949) | 18.0 | 75% | 518 |
| [NP_001156506.1](http://www.ncbi.nlm.nih.gov/entrez/query.fcgi?cmd=Retrieve&db=Protein&list_uids=251823858&dopt=GenPept&RID=TFN1Y92J01S&log$=prottop&blast_rank=59) | regulatory-associated protein of mTOR isoform 2 [Homo sapiens] | [17.6](http://blast.ncbi.nlm.nih.gov/Blast.cgi#251823858%23251823858) | 29.7 | 87% | 695 |
| [XP_002344508.1](http://www.ncbi.nlm.nih.gov/entrez/query.fcgi?cmd=Retrieve&db=Protein&list_uids=239755234&dopt=GenPept&RID=TFN1Y92J01S&log$=prottop&blast_rank=60) | PREDICTED: similar to armadillo repeat containing 4 [Homo sapiens] | [17.6](http://blast.ncbi.nlm.nih.gov/Blast.cgi#239755234%23239755234) | 17.6 | 75% | 695 |
| [XP_002347158.1](http://www.ncbi.nlm.nih.gov/entrez/query.fcgi?cmd=Retrieve&db=Protein&list_uids=239749766&dopt=GenPept&RID=TFN1Y92J01S&log$=prottop&blast_rank=61) | PREDICTED: similar to armadillo repeat containing 4 [Homo sapiens] | [17.6](http://blast.ncbi.nlm.nih.gov/Blast.cgi#239749766%23239749766) | 17.6 | 75% | 695 |
| [XP_002343006.1](http://www.ncbi.nlm.nih.gov/entrez/query.fcgi?cmd=Retrieve&db=Protein&list_uids=239744025&dopt=GenPept&RID=TFN1Y92J01S&log$=prottop&blast_rank=62) | PREDICTED: hypothetical protein XP_002343006 [Homo sapiens] | [17.6](http://blast.ncbi.nlm.nih.gov/Blast.cgi#239744025%23239744025) | 17.6 | 75% | 695 |
| [NP_001157918.1](http://www.ncbi.nlm.nih.gov/entrez/query.fcgi?cmd=Retrieve&db=Protein&list_uids=256574805&dopt=GenPept&RID=TFN1Y92J01S&log$=prottop&blast_rank=63) | hypothetical protein LOC647024 [Homo sapiens] >ref|XP_946637.4| PREDICTED: chromosome 6 open reading frame 132 [Homo sapiens] >ref|XP_001724365.2| PREDICTED: chromosome 6 open reading frame 132 [Homo sapiens] | [17.6](http://blast.ncbi.nlm.nih.gov/Blast.cgi#256574805%23256574805) | 17.6 | 62% | 695 |
| [NP_056667.2](http://www.ncbi.nlm.nih.gov/entrez/query.fcgi?cmd=Retrieve&db=Protein&list_uids=221316634&dopt=GenPept&RID=TFN1Y92J01S&log$=prottop&blast_rank=64) | LIM domain only protein 7 isoform 2 [Homo sapiens] | [17.6](http://blast.ncbi.nlm.nih.gov/Blast.cgi#221316634%23221316634) | 17.6 | 75% | 695 |
| [NP_996816.2](http://www.ncbi.nlm.nih.gov/entrez/query.fcgi?cmd=Retrieve&db=Protein&list_uids=219842266&dopt=GenPept&RID=TFN1Y92J01S&log$=prottop&blast_rank=65) | usherin isoform B [Homo sapiens] | [17.6](http://blast.ncbi.nlm.nih.gov/Blast.cgi#219842266%23219842266) | 32.7 | 87% | 695 |
| [NP_619727.2](http://www.ncbi.nlm.nih.gov/entrez/query.fcgi?cmd=Retrieve&db=Protein&list_uids=164419744&dopt=GenPept&RID=TFN1Y92J01S&log$=prottop&blast_rank=66) | nuclear factor of activated T-cells 5 isoform b [Homo sapiens] | [17.6](http://blast.ncbi.nlm.nih.gov/Blast.cgi#164419744%23164419744) | 33.5 | 87% | 695 |
| [NP_056280.2](http://www.ncbi.nlm.nih.gov/entrez/query.fcgi?cmd=Retrieve&db=Protein&list_uids=157739942&dopt=GenPept&RID=TFN1Y92J01S&log$=prottop&blast_rank=67) | gem-associated protein 5 [Homo sapiens] | [17.6](http://blast.ncbi.nlm.nih.gov/Blast.cgi#157739942%23157739942) | 32.2 | 100% | 695 |
| [NP_055932.2](http://www.ncbi.nlm.nih.gov/entrez/query.fcgi?cmd=Retrieve&db=Protein&list_uids=155722994&dopt=GenPept&RID=TFN1Y92J01S&log$=prottop&blast_rank=68) | zinc finger CCCH domain-containing protein 3 [Homo sapiens] | [17.6](http://blast.ncbi.nlm.nih.gov/Blast.cgi#155722994%23155722994) | 17.6 | 62% | 695 |
| [NP_612636.2](http://www.ncbi.nlm.nih.gov/entrez/query.fcgi?cmd=Retrieve&db=Protein&list_uids=154426278&dopt=GenPept&RID=TFN1Y92J01S&log$=prottop&blast_rank=69) | hypothetical protein LOC90523 [Homo sapiens] | [17.6](http://blast.ncbi.nlm.nih.gov/Blast.cgi#154426278%23154426278) | 17.6 | 62% | 695 |
| [NP_060529.4](http://www.ncbi.nlm.nih.gov/entrez/query.fcgi?cmd=Retrieve&db=Protein&list_uids=148612825&dopt=GenPept&RID=TFN1Y92J01S&log$=prottop&blast_rank=70) | ras-associating and dilute domain-containing protein [Homo sapiens] | [17.6](http://blast.ncbi.nlm.nih.gov/Blast.cgi#148612825%23148612825) | 17.6 | 75% | 695 |
| [NP_076427.2](http://www.ncbi.nlm.nih.gov/entrez/query.fcgi?cmd=Retrieve&db=Protein&list_uids=145275189&dopt=GenPept&RID=TFN1Y92J01S&log$=prottop&blast_rank=71) | specifically androgen-regulated gene protein isoform 1 [Homo sapiens] | [17.6](http://blast.ncbi.nlm.nih.gov/Blast.cgi#145275189%23145275189) | 31.8 | 75% | 695 |
| [NP_001106649.1](http://www.ncbi.nlm.nih.gov/entrez/query.fcgi?cmd=Retrieve&db=Protein&list_uids=164419746&dopt=GenPept&RID=TFN1Y92J01S&log$=prottop&blast_rank=72) | nuclear factor of activated T-cells 5 isoform d [Homo sapiens] | [17.6](http://blast.ncbi.nlm.nih.gov/Blast.cgi#164419746%23164419746) | 33.5 | 87% | 695 |
| [NP_002909.4](http://www.ncbi.nlm.nih.gov/entrez/query.fcgi?cmd=Retrieve&db=Protein&list_uids=238859557&dopt=GenPept&RID=TFN1Y92J01S&log$=prottop&blast_rank=73) | MHC class II regulatory factor RFX1 [Homo sapiens] | [17.6](http://blast.ncbi.nlm.nih.gov/Blast.cgi#238859557%23238859557) | 32.7 | 75% | 695 |
| [NP_066550.2](http://www.ncbi.nlm.nih.gov/entrez/query.fcgi?cmd=Retrieve&db=Protein&list_uids=116325989&dopt=GenPept&RID=TFN1Y92J01S&log$=prottop&blast_rank=74) | potassium/sodium hyperpolarization-activated cyclic nucleotide-gated channel 1 [Homo sapiens] | [17.6](http://blast.ncbi.nlm.nih.gov/Blast.cgi#116325989%23116325989) | 17.6 | 75% | 695 |
| [NP_078966.2](http://www.ncbi.nlm.nih.gov/entrez/query.fcgi?cmd=Retrieve&db=Protein&list_uids=83367077&dopt=GenPept&RID=TFN1Y92J01S&log$=prottop&blast_rank=75) | mucin-16 [Homo sapiens] | [17.6](http://blast.ncbi.nlm.nih.gov/Blast.cgi#83367077%2383367077) | 294 | 100% | 695 |
| [NP_060338.3](http://www.ncbi.nlm.nih.gov/entrez/query.fcgi?cmd=Retrieve&db=Protein&list_uids=90669931&dopt=GenPept&RID=TFN1Y92J01S&log$=prottop&blast_rank=76) | tetratricopeptide repeat protein 12 [Homo sapiens] | [17.6](http://blast.ncbi.nlm.nih.gov/Blast.cgi#90669931%2390669931) | 17.6 | 100% | 695 |
| [NP_001013764.1](http://www.ncbi.nlm.nih.gov/entrez/query.fcgi?cmd=Retrieve&db=Protein&list_uids=62000702&dopt=GenPept&RID=TFN1Y92J01S&log$=prottop&blast_rank=77) | diacylglycerol kinase kappa [Homo sapiens] | [17.6](http://blast.ncbi.nlm.nih.gov/Blast.cgi#62000702%2362000702) | 17.6 | 75% | 695 |
| [NP_694984.3](http://www.ncbi.nlm.nih.gov/entrez/query.fcgi?cmd=Retrieve&db=Protein&list_uids=153791630&dopt=GenPept&RID=TFN1Y92J01S&log$=prottop&blast_rank=78) | bromodomain and WD repeat-containing protein 3 [Homo sapiens] | [17.6](http://blast.ncbi.nlm.nih.gov/Blast.cgi#153791630%23153791630) | 17.6 | 100% | 695 |
| [NP_060080.6](http://www.ncbi.nlm.nih.gov/entrez/query.fcgi?cmd=Retrieve&db=Protein&list_uids=37595553&dopt=GenPept&RID=TFN1Y92J01S&log$=prottop&blast_rank=79) | E3 ubiquitin-protein ligase Arkadia [Homo sapiens] | [17.6](http://blast.ncbi.nlm.nih.gov/Blast.cgi#37595553%2337595553) | 46.0 | 75% | 695 |
| [NP_005349.3](http://www.ncbi.nlm.nih.gov/entrez/query.fcgi?cmd=Retrieve&db=Protein&list_uids=33598968&dopt=GenPept&RID=TFN1Y92J01S&log$=prottop&blast_rank=80) | LIM domain only protein 7 isoform 1 [Homo sapiens] | [17.6](http://blast.ncbi.nlm.nih.gov/Blast.cgi#33598968%2333598968) | 17.6 | 75% | 695 |
| [NP_060546.2](http://www.ncbi.nlm.nih.gov/entrez/query.fcgi?cmd=Retrieve&db=Protein&list_uids=31657114&dopt=GenPept&RID=TFN1Y92J01S&log$=prottop&blast_rank=81) | armadillo repeat-containing protein 4 [Homo sapiens] | [17.6](http://blast.ncbi.nlm.nih.gov/Blast.cgi#31657114%2331657114) | 29.3 | 75% | 695 |
| [NP_057290.2](http://www.ncbi.nlm.nih.gov/entrez/query.fcgi?cmd=Retrieve&db=Protein&list_uids=56550035&dopt=GenPept&RID=TFN1Y92J01S&log$=prottop&blast_rank=82) | colon carcinoma related protein [Homo sapiens] | [17.6](http://blast.ncbi.nlm.nih.gov/Blast.cgi#56550035%2356550035) | 17.6 | 75% | 695 |
| [NP_115699.1](http://www.ncbi.nlm.nih.gov/entrez/query.fcgi?cmd=Retrieve&db=Protein&list_uids=14150098&dopt=GenPept&RID=TFN1Y92J01S&log$=prottop&blast_rank=83) | transmembrane protein 79 [Homo sapiens] | [17.6](http://blast.ncbi.nlm.nih.gov/Blast.cgi#14150098%2314150098) | 17.6 | 75% | 695 |
| [NP_659451.1](http://www.ncbi.nlm.nih.gov/entrez/query.fcgi?cmd=Retrieve&db=Protein&list_uids=21450731&dopt=GenPept&RID=TFN1Y92J01S&log$=prottop&blast_rank=84) | hydrolethalus syndrome protein 1 [Homo sapiens] >ref|NP_001128265.1| hydrolethalus syndrome protein 1 [Homo sapiens] | [17.6](http://blast.ncbi.nlm.nih.gov/Blast.cgi#21450731%2321450731) | 17.6 | 75% | 695 |
| [NP_056469.2](http://www.ncbi.nlm.nih.gov/entrez/query.fcgi?cmd=Retrieve&db=Protein&list_uids=31542521&dopt=GenPept&RID=TFN1Y92J01S&log$=prottop&blast_rank=85) | N-acetyltransferase 9 [Homo sapiens] | [17.6](http://blast.ncbi.nlm.nih.gov/Blast.cgi#31542521%2331542521) | 17.6 | 62% | 695 |
| [NP_687034.1](http://www.ncbi.nlm.nih.gov/entrez/query.fcgi?cmd=Retrieve&db=Protein&list_uids=23199971&dopt=GenPept&RID=TFN1Y92J01S&log$=prottop&blast_rank=86) | T-cell activation Rho GTPase-activating protein isoform a [Homo sapiens] | [17.6](http://blast.ncbi.nlm.nih.gov/Blast.cgi#23199971%2323199971) | 17.6 | 62% | 695 |
| [NP_054767.3](http://www.ncbi.nlm.nih.gov/entrez/query.fcgi?cmd=Retrieve&db=Protein&list_uids=38569480&dopt=GenPept&RID=TFN1Y92J01S&log$=prottop&blast_rank=87) | MKL/myocardin-like protein 2 [Homo sapiens] | [17.6](http://blast.ncbi.nlm.nih.gov/Blast.cgi#38569480%2338569480) | 17.6 | 87% | 695 |
| [NP_057688.2](http://www.ncbi.nlm.nih.gov/entrez/query.fcgi?cmd=Retrieve&db=Protein&list_uids=38372909&dopt=GenPept&RID=TFN1Y92J01S&log$=prottop&blast_rank=88) | lysine-specific demethylase 3B [Homo sapiens] | [17.6](http://blast.ncbi.nlm.nih.gov/Blast.cgi#38372909%2338372909) | 17.6 | 62% | 695 |
| [NP_003972.1](http://www.ncbi.nlm.nih.gov/entrez/query.fcgi?cmd=Retrieve&db=Protein&list_uids=4506039&dopt=GenPept&RID=TFN1Y92J01S&log$=prottop&blast_rank=89) | protein regulator of cytokinesis 1 isoform 1 [Homo sapiens] | [17.6](http://blast.ncbi.nlm.nih.gov/Blast.cgi#4506039%234506039) | 17.6 | 100% | 695 |
| [NP_619728.2](http://www.ncbi.nlm.nih.gov/entrez/query.fcgi?cmd=Retrieve&db=Protein&list_uids=27886520&dopt=GenPept&RID=TFN1Y92J01S&log$=prottop&blast_rank=90) | nuclear factor of activated T-cells 5 isoform a [Homo sapiens] >ref|NP_775321.1| nuclear factor of activated T-cells 5 isoform a [Homo sapiens] | [17.6](http://blast.ncbi.nlm.nih.gov/Blast.cgi#27886520%2327886520) | 33.5 | 87% | 695 |
| [NP_060413.2](http://www.ncbi.nlm.nih.gov/entrez/query.fcgi?cmd=Retrieve&db=Protein&list_uids=40807493&dopt=GenPept&RID=TFN1Y92J01S&log$=prottop&blast_rank=91) | protein CGI-301 [Homo sapiens] >ref|NP_689417.1| protein CGI-301 [Homo sapiens] | [17.6](http://blast.ncbi.nlm.nih.gov/Blast.cgi#40807493%2340807493) | 17.6 | 75% | 695 |
| [NP_110412.1](http://www.ncbi.nlm.nih.gov/entrez/query.fcgi?cmd=Retrieve&db=Protein&list_uids=13540559&dopt=GenPept&RID=TFN1Y92J01S&log$=prottop&blast_rank=92) | radial spoke head-like protein 1 [Homo sapiens] | [17.6](http://blast.ncbi.nlm.nih.gov/Blast.cgi#13540559%2313540559) | 17.6 | 75% | 695 |
| [NP_036338.2](http://www.ncbi.nlm.nih.gov/entrez/query.fcgi?cmd=Retrieve&db=Protein&list_uids=153085427&dopt=GenPept&RID=TFN1Y92J01S&log$=prottop&blast_rank=93) | hepatitis A virus cellular receptor 1 precursor [Homo sapiens] >ref|NP_001092884.1| hepatitis A virus cellular receptor 1 precursor [Homo sapiens] | [17.6](http://blast.ncbi.nlm.nih.gov/Blast.cgi#153085427%23153085427) | 17.6 | 75% | 695 |
| [NP_473455.2](http://www.ncbi.nlm.nih.gov/entrez/query.fcgi?cmd=Retrieve&db=Protein&list_uids=21314774&dopt=GenPept&RID=TFN1Y92J01S&log$=prottop&blast_rank=94) | T-cell activation Rho GTPase-activating protein isoform b [Homo sapiens] | [17.6](http://blast.ncbi.nlm.nih.gov/Blast.cgi#21314774%2321314774) | 17.6 | 62% | 695 |
| [NP_006590.1](http://www.ncbi.nlm.nih.gov/entrez/query.fcgi?cmd=Retrieve&db=Protein&list_uids=5729945&dopt=GenPept&RID=TFN1Y92J01S&log$=prottop&blast_rank=95) | nuclear factor of activated T-cells 5 isoform c [Homo sapiens] | [17.6](http://blast.ncbi.nlm.nih.gov/Blast.cgi#5729945%235729945) | 33.5 | 87% | 695 |
| [NP_710157.2](http://www.ncbi.nlm.nih.gov/entrez/query.fcgi?cmd=Retrieve&db=Protein&list_uids=31377557&dopt=GenPept&RID=TFN1Y92J01S&log$=prottop&blast_rank=96) | hypothetical protein LOC131583 [Homo sapiens] | [17.6](http://blast.ncbi.nlm.nih.gov/Blast.cgi#31377557%2331377557) | 17.6 | 100% | 695 |
| [NP_002103.2](http://www.ncbi.nlm.nih.gov/entrez/query.fcgi?cmd=Retrieve&db=Protein&list_uids=92110055&dopt=GenPept&RID=TFN1Y92J01S&log$=prottop&blast_rank=97) | histidine decarboxylase [Homo sapiens] | [17.6](http://blast.ncbi.nlm.nih.gov/Blast.cgi#92110055%2392110055) | 17.6 | 62% | 695 |
| [NP_036596.3](http://www.ncbi.nlm.nih.gov/entrez/query.fcgi?cmd=Retrieve&db=Protein&list_uids=22547221&dopt=GenPept&RID=TFN1Y92J01S&log$=prottop&blast_rank=98) | tolloid-like 1 precursor [Homo sapiens] | [17.6](http://blast.ncbi.nlm.nih.gov/Blast.cgi#22547221%2322547221) | 17.6 | 62% | 695 |
| [NP_071801.1](http://www.ncbi.nlm.nih.gov/entrez/query.fcgi?cmd=Retrieve&db=Protein&list_uids=12408647&dopt=GenPept&RID=TFN1Y92J01S&log$=prottop&blast_rank=99) | X-ray repair cross complementing protein 4 isoform 2 [Homo sapiens] | [17.6](http://blast.ncbi.nlm.nih.gov/Blast.cgi#12408647%2312408647) | 27.6 | 87% | 695 |
| [NP_003418.2](http://www.ncbi.nlm.nih.gov/entrez/query.fcgi?cmd=Retrieve&db=Protein&list_uids=21361146&dopt=GenPept&RID=TFN1Y92J01S&log$=prottop&blast_rank=100) | zinc finger protein 76 [Homo sapiens] | [17.6](http://blast.ncbi.nlm.nih.gov/Blast.cgi#21361146%2321361146) | 17.6 | 62% | 695 |

| **Accession** | **Proteins with a match to APQGYLFK peptide** | **[Max score](http://blast.ncbi.nlm.nih.gov/Blast.cgi?CMD=Get&ALIGNMENTS=100&ALIGNMENT_VIEW=Pairwise&CDD_SEARCH_STATE=1&DATABASE_SORT=0&DESCRIPTIONS=100&ENTREZ_QUERY=txid9606 %5BORGN%5D&FIRST_QUERY_NUM=0&FORMAT_OBJECT=Alignment&FORMAT_PAGE_TARGET=&FORMAT_TYPE=HTML&GET_SEQUENCE=yes&I_THRESH=&MASK_CHAR=2&MASK_COLOR=1&NEW_VIEW=yes&NUM_OVERVIEW=100&OLD_BLAST=false&PAGE=Proteins&QUERY_INDEX=0&QUERY_NUMBER=0&RESULTS_PAGE_TARGET=&RID=TFN66FGG01S&SHOW_LINKOUT=yes&SHOW_OVERVIEW=yes&STEP_NUMBER=&WORD_SIZE=2&DISPLAY_SORT=1&HSP_SORT=1" \l "sort_mark)** | **[Total score](http://blast.ncbi.nlm.nih.gov/Blast.cgi?CMD=Get&ALIGNMENTS=100&ALIGNMENT_VIEW=Pairwise&CDD_SEARCH_STATE=1&DATABASE_SORT=0&DESCRIPTIONS=100&ENTREZ_QUERY=txid9606 %5BORGN%5D&FIRST_QUERY_NUM=0&FORMAT_OBJECT=Alignment&FORMAT_PAGE_TARGET=&FORMAT_TYPE=HTML&GET_SEQUENCE=yes&I_THRESH=&MASK_CHAR=2&MASK_COLOR=1&NEW_VIEW=yes&NUM_OVERVIEW=100&OLD_BLAST=false&PAGE=Proteins&QUERY_INDEX=0&QUERY_NUMBER=0&RESULTS_PAGE_TARGET=&RID=TFN66FGG01S&SHOW_LINKOUT=yes&SHOW_OVERVIEW=yes&STEP_NUMBER=&WORD_SIZE=2&DISPLAY_SORT=2&HSP_SORT=1" \l "sort_mark)** | **[Query coverage](http://blast.ncbi.nlm.nih.gov/Blast.cgi?CMD=Get&ALIGNMENTS=100&ALIGNMENT_VIEW=Pairwise&CDD_SEARCH_STATE=1&DATABASE_SORT=0&DESCRIPTIONS=100&ENTREZ_QUERY=txid9606 %5BORGN%5D&FIRST_QUERY_NUM=0&FORMAT_OBJECT=Alignment&FORMAT_PAGE_TARGET=&FORMAT_TYPE=HTML&GET_SEQUENCE=yes&I_THRESH=&MASK_CHAR=2&MASK_COLOR=1&NEW_VIEW=yes&NUM_OVERVIEW=100&OLD_BLAST=false&PAGE=Proteins&QUERY_INDEX=0&QUERY_NUMBER=0&RESULTS_PAGE_TARGET=&RID=TFN66FGG01S&SHOW_LINKOUT=yes&SHOW_OVERVIEW=yes&STEP_NUMBER=&WORD_SIZE=2&DISPLAY_SORT=4&HSP_SORT=0" \l "sort_mark)** | **[E value](http://blast.ncbi.nlm.nih.gov/Blast.cgi?CMD=Get&ALIGNMENTS=100&ALIGNMENT_VIEW=Pairwise&CDD_SEARCH_STATE=1&DATABASE_SORT=0&DESCRIPTIONS=100&ENTREZ_QUERY=txid9606 %5BORGN%5D&FIRST_QUERY_NUM=0&FORMAT_OBJECT=Alignment&FORMAT_PAGE_TARGET=&FORMAT_TYPE=HTML&GET_SEQUENCE=yes&I_THRESH=&MASK_CHAR=2&MASK_COLOR=1&NEW_VIEW=yes&NUM_OVERVIEW=100&OLD_BLAST=false&PAGE=Proteins&QUERY_INDEX=0&QUERY_NUMBER=0&RESULTS_PAGE_TARGET=&RID=TFN66FGG01S&SHOW_LINKOUT=yes&SHOW_OVERVIEW=yes&STEP_NUMBER=&WORD_SIZE=2&DISPLAY_SORT=0&HSP_SORT=0" \l "sort_mark)** |
| --- | --- | --- | --- | --- | --- |
| [NP_001135763.1](http://www.ncbi.nlm.nih.gov/entrez/query.fcgi?cmd=Retrieve&db=Protein&list_uids=214010238&dopt=GenPept&RID=TFN66FGG01S&log$=prottop&blast_rank=1) | probable E3 ubiquitin-protein ligase MGRN1 isoform 4 [Homo sapiens] | [20.6](http://blast.ncbi.nlm.nih.gov/Blast.cgi#214010238%23214010238) | 20.6 | 75% | 89 |
| [NP_001135761.1](http://www.ncbi.nlm.nih.gov/entrez/query.fcgi?cmd=Retrieve&db=Protein&list_uids=214010234&dopt=GenPept&RID=TFN66FGG01S&log$=prottop&blast_rank=2) | probable E3 ubiquitin-protein ligase MGRN1 isoform 2 [Homo sapiens] | [20.6](http://blast.ncbi.nlm.nih.gov/Blast.cgi#214010234%23214010234) | 20.6 | 75% | 89 |
| [NP_005844.4](http://www.ncbi.nlm.nih.gov/entrez/query.fcgi?cmd=Retrieve&db=Protein&list_uids=139394646&dopt=GenPept&RID=TFN66FGG01S&log$=prottop&blast_rank=3) | iroquois-class homeodomain protein IRX-5 [Homo sapiens] | [20.6](http://blast.ncbi.nlm.nih.gov/Blast.cgi#139394646%23139394646) | 20.6 | 75% | 89 |
| [NP_001135762.1](http://www.ncbi.nlm.nih.gov/entrez/query.fcgi?cmd=Retrieve&db=Protein&list_uids=214010236&dopt=GenPept&RID=TFN66FGG01S&log$=prottop&blast_rank=4) | probable E3 ubiquitin-protein ligase MGRN1 isoform 3 [Homo sapiens] | [20.6](http://blast.ncbi.nlm.nih.gov/Blast.cgi#214010236%23214010236) | 20.6 | 75% | 89 |
| [NP_056061.1](http://www.ncbi.nlm.nih.gov/entrez/query.fcgi?cmd=Retrieve&db=Protein&list_uids=44917608&dopt=GenPept&RID=TFN66FGG01S&log$=prottop&blast_rank=5) | probable E3 ubiquitin-protein ligase MGRN1 isoform 1 [Homo sapiens] | [20.6](http://blast.ncbi.nlm.nih.gov/Blast.cgi#44917608%2344917608) | 20.6 | 75% | 89 |
| [NP_443148.1](http://www.ncbi.nlm.nih.gov/entrez/query.fcgi?cmd=Retrieve&db=Protein&list_uids=58743365&dopt=GenPept&RID=TFN66FGG01S&log$=prottop&blast_rank=6) | ring finger protein 157 [Homo sapiens] | [20.6](http://blast.ncbi.nlm.nih.gov/Blast.cgi#58743365%2358743365) | 20.6 | 75% | 89 |
| [NP_150366.1](http://www.ncbi.nlm.nih.gov/entrez/query.fcgi?cmd=Retrieve&db=Protein&list_uids=39930475&dopt=GenPept&RID=TFN66FGG01S&log$=prottop&blast_rank=7) | iroquois-class homeodomain protein IRX-2 [Homo sapiens] >ref|NP_001127694.1| iroquois-class homeodomain protein IRX-2 [Homo sapiens] | [20.6](http://blast.ncbi.nlm.nih.gov/Blast.cgi#39930475%2339930475) | 20.6 | 75% | 89 |
| [NP_036419.3](http://www.ncbi.nlm.nih.gov/entrez/query.fcgi?cmd=Retrieve&db=Protein&list_uids=187608516&dopt=GenPept&RID=TFN66FGG01S&log$=prottop&blast_rank=8) | arf-GAP with coiled-coil, ANK repeat and PH domain-containing protein 2 [Homo sapiens] | [20.2](http://blast.ncbi.nlm.nih.gov/Blast.cgi#187608516%23187608516) | 20.2 | 75% | 119 |
| [NP_085152.2](http://www.ncbi.nlm.nih.gov/entrez/query.fcgi?cmd=Retrieve&db=Protein&list_uids=218563749&dopt=GenPept&RID=TFN66FGG01S&log$=prottop&blast_rank=9) | arf-GAP with coiled-coil, ANK repeat and PH domain-containing protein 3 [Homo sapiens] | [20.2](http://blast.ncbi.nlm.nih.gov/Blast.cgi#218563749%23218563749) | 20.2 | 75% | 119 |
| [NP_000318.1](http://www.ncbi.nlm.nih.gov/entrez/query.fcgi?cmd=Retrieve&db=Protein&list_uids=4506575&dopt=GenPept&RID=TFN66FGG01S&log$=prottop&blast_rank=10) | retinal outer segment membrane protein 1 [Homo sapiens] | [20.2](http://blast.ncbi.nlm.nih.gov/Blast.cgi#4506575%234506575) | 20.2 | 62% | 119 |
| [XP_001130897.2](http://www.ncbi.nlm.nih.gov/entrez/query.fcgi?cmd=Retrieve&db=Protein&list_uids=169161624&dopt=GenPept&RID=TFN66FGG01S&log$=prottop&blast_rank=11) | PREDICTED: similar to nucleophosmin 1 [Homo sapiens] >ref|XP_001722227.1| PREDICTED: similar to nucleophosmin 1 [Homo sapiens] >ref|XP_496355.4| PREDICTED: similar to nucleophosmin 1 [Homo sapiens] | [19.7](http://blast.ncbi.nlm.nih.gov/Blast.cgi#169161624%23169161624) | 19.7 | 75% | 160 |
| [NP_000129.3](http://www.ncbi.nlm.nih.gov/entrez/query.fcgi?cmd=Retrieve&db=Protein&list_uids=281485550&dopt=GenPept&RID=TFN66FGG01S&log$=prottop&blast_rank=12) | fibrillin-1 precursor [Homo sapiens] | [19.7](http://blast.ncbi.nlm.nih.gov/Blast.cgi#281485550%23281485550) | 32.7 | 75% | 160 |
| [NP_001011722.2](http://www.ncbi.nlm.nih.gov/entrez/query.fcgi?cmd=Retrieve&db=Protein&list_uids=74136547&dopt=GenPept&RID=TFN66FGG01S&log$=prottop&blast_rank=13) | rho guanine nucleotide exchange factor 10-like protein isoform 2 [Homo sapiens] | [19.7](http://blast.ncbi.nlm.nih.gov/Blast.cgi#74136547%2374136547) | 19.7 | 62% | 160 |
| [NP_060595.3](http://www.ncbi.nlm.nih.gov/entrez/query.fcgi?cmd=Retrieve&db=Protein&list_uids=58761492&dopt=GenPept&RID=TFN66FGG01S&log$=prottop&blast_rank=14) | rho guanine nucleotide exchange factor 10-like protein isoform 1 [Homo sapiens] | [19.7](http://blast.ncbi.nlm.nih.gov/Blast.cgi#58761492%2358761492) | 19.7 | 62% | 160 |
| [NP_954654.1](http://www.ncbi.nlm.nih.gov/entrez/query.fcgi?cmd=Retrieve&db=Protein&list_uids=40353734&dopt=GenPept&RID=TFN66FGG01S&log$=prottop&blast_rank=15) | nucleophosmin isoform 2 [Homo sapiens] | [19.7](http://blast.ncbi.nlm.nih.gov/Blast.cgi#40353734%2340353734) | 19.7 | 75% | 160 |
| [NP_055872.3](http://www.ncbi.nlm.nih.gov/entrez/query.fcgi?cmd=Retrieve&db=Protein&list_uids=126116565&dopt=GenPept&RID=TFN66FGG01S&log$=prottop&blast_rank=16) | probable E3 ubiquitin-protein ligase MYCBP2 [Homo sapiens] | [19.7](http://blast.ncbi.nlm.nih.gov/Blast.cgi#126116565%23126116565) | 36.1 | 62% | 160 |
| [NP_002511.1](http://www.ncbi.nlm.nih.gov/entrez/query.fcgi?cmd=Retrieve&db=Protein&list_uids=10835063&dopt=GenPept&RID=TFN66FGG01S&log$=prottop&blast_rank=17) | nucleophosmin isoform 1 [Homo sapiens] | [19.7](http://blast.ncbi.nlm.nih.gov/Blast.cgi#10835063%2310835063) | 19.7 | 75% | 160 |
| [NP_001032827.1](http://www.ncbi.nlm.nih.gov/entrez/query.fcgi?cmd=Retrieve&db=Protein&list_uids=83641870&dopt=GenPept&RID=TFN66FGG01S&log$=prottop&blast_rank=18) | nucleophosmin isoform 3 [Homo sapiens] | [19.7](http://blast.ncbi.nlm.nih.gov/Blast.cgi#83641870%2383641870) | 19.7 | 75% | 160 |
| [NP_001092142.1](http://www.ncbi.nlm.nih.gov/entrez/query.fcgi?cmd=Retrieve&db=Protein&list_uids=148886707&dopt=GenPept&RID=TFN66FGG01S&log$=prottop&blast_rank=19) | hephaestin-like protein 1 precursor [Homo sapiens] | [19.3](http://blast.ncbi.nlm.nih.gov/Blast.cgi#148886707%23148886707) | 36.1 | 87% | 214 |
| [NP_775735.2](http://www.ncbi.nlm.nih.gov/entrez/query.fcgi?cmd=Retrieve&db=Protein&list_uids=158138528&dopt=GenPept&RID=TFN66FGG01S&log$=prottop&blast_rank=20) | lethal(3)malignant brain tumor-like protein 4 [Homo sapiens] | [19.3](http://blast.ncbi.nlm.nih.gov/Blast.cgi#158138528%23158138528) | 19.3 | 62% | 214 |
| [NP_861525.2](http://www.ncbi.nlm.nih.gov/entrez/query.fcgi?cmd=Retrieve&db=Protein&list_uids=145611428&dopt=GenPept&RID=TFN66FGG01S&log$=prottop&blast_rank=21) | bone morphogenetic protein 8A precursor [Homo sapiens] | [19.3](http://blast.ncbi.nlm.nih.gov/Blast.cgi#145611428%23145611428) | 19.3 | 62% | 214 |
| [NP_001711.2](http://www.ncbi.nlm.nih.gov/entrez/query.fcgi?cmd=Retrieve&db=Protein&list_uids=29571106&dopt=GenPept&RID=TFN66FGG01S&log$=prottop&blast_rank=22) | bone morphogenetic protein 8B preproprotein [Homo sapiens] | [19.3](http://blast.ncbi.nlm.nih.gov/Blast.cgi#29571106%2329571106) | 19.3 | 62% | 214 |
| [NP_001153879.1](http://www.ncbi.nlm.nih.gov/entrez/query.fcgi?cmd=Retrieve&db=Protein&list_uids=237874289&dopt=GenPept&RID=TFN66FGG01S&log$=prottop&blast_rank=23) | TLC domain-containing protein 1 isoform 2 [Homo sapiens] | [18.9](http://blast.ncbi.nlm.nih.gov/Blast.cgi#237874289%23237874289) | 18.9 | 75% | 288 |
| [NP_036428.1](http://www.ncbi.nlm.nih.gov/entrez/query.fcgi?cmd=Retrieve&db=Protein&list_uids=6912460&dopt=GenPept&RID=TFN66FGG01S&log$=prottop&blast_rank=24) | GRB2-associated-binding protein 2 isoform b [Homo sapiens] | [18.9](http://blast.ncbi.nlm.nih.gov/Blast.cgi#6912460%236912460) | 18.9 | 87% | 288 |
| [NP_612472.1](http://www.ncbi.nlm.nih.gov/entrez/query.fcgi?cmd=Retrieve&db=Protein&list_uids=19923999&dopt=GenPept&RID=TFN66FGG01S&log$=prottop&blast_rank=25) | TLC domain-containing protein 1 isoform 1 [Homo sapiens] | [18.9](http://blast.ncbi.nlm.nih.gov/Blast.cgi#19923999%2319923999) | 18.9 | 75% | 288 |
| [NP_006104.4](http://www.ncbi.nlm.nih.gov/entrez/query.fcgi?cmd=Retrieve&db=Protein&list_uids=120432044&dopt=GenPept&RID=TFN66FGG01S&log$=prottop&blast_rank=26) | guanine nucleotide exchange factor VAV3 isoform 1 [Homo sapiens] | [18.9](http://blast.ncbi.nlm.nih.gov/Blast.cgi#120432044%23120432044) | 18.9 | 75% | 288 |
| [NP_536739.1](http://www.ncbi.nlm.nih.gov/entrez/query.fcgi?cmd=Retrieve&db=Protein&list_uids=18105042&dopt=GenPept&RID=TFN66FGG01S&log$=prottop&blast_rank=27) | GRB2-associated-binding protein 2 isoform a [Homo sapiens] | [18.9](http://blast.ncbi.nlm.nih.gov/Blast.cgi#18105042%2318105042) | 18.9 | 87% | 288 |
| [XP_001718487.1](http://www.ncbi.nlm.nih.gov/entrez/query.fcgi?cmd=Retrieve&db=Protein&list_uids=169215858&dopt=GenPept&RID=TFN66FGG01S&log$=prottop&blast_rank=28) | PREDICTED: similar to ribosomal protein L3, partial [Homo sapiens] | [18.5](http://blast.ncbi.nlm.nih.gov/Blast.cgi#169215858%23169215858) | 18.5 | 75% | 386 |
| [NP_597681.2](http://www.ncbi.nlm.nih.gov/entrez/query.fcgi?cmd=Retrieve&db=Protein&list_uids=110349717&dopt=GenPept&RID=TFN66FGG01S&log$=prottop&blast_rank=29) | titin isoform novex-2 [Homo sapiens] | [18.5](http://blast.ncbi.nlm.nih.gov/Blast.cgi#110349717%23110349717) | 28.4 | 75% | 386 |
| [NP_596869.3](http://www.ncbi.nlm.nih.gov/entrez/query.fcgi?cmd=Retrieve&db=Protein&list_uids=110349719&dopt=GenPept&RID=TFN66FGG01S&log$=prottop&blast_rank=30) | titin isoform N2-A [Homo sapiens] | [18.5](http://blast.ncbi.nlm.nih.gov/Blast.cgi#110349719%23110349719) | 18.5 | 75% | 386 |
| [NP_597676.2](http://www.ncbi.nlm.nih.gov/entrez/query.fcgi?cmd=Retrieve&db=Protein&list_uids=110349713&dopt=GenPept&RID=TFN66FGG01S&log$=prottop&blast_rank=31) | titin isoform novex-1 [Homo sapiens] | [18.5](http://blast.ncbi.nlm.nih.gov/Blast.cgi#110349713%23110349713) | 28.4 | 75% | 386 |
| [NP_003310.3](http://www.ncbi.nlm.nih.gov/entrez/query.fcgi?cmd=Retrieve&db=Protein&list_uids=110349715&dopt=GenPept&RID=TFN66FGG01S&log$=prottop&blast_rank=32) | titin isoform N2-B [Homo sapiens] | [18.5](http://blast.ncbi.nlm.nih.gov/Blast.cgi#110349715%23110349715) | 28.4 | 75% | 386 |
| [NP_001123959.1](http://www.ncbi.nlm.nih.gov/entrez/query.fcgi?cmd=Retrieve&db=Protein&list_uids=194440722&dopt=GenPept&RID=TFN66FGG01S&log$=prottop&blast_rank=33) | protein NDRG4 isoform 2 [Homo sapiens] | [18.5](http://blast.ncbi.nlm.nih.gov/Blast.cgi#194440722%23194440722) | 18.5 | 75% | 386 |
| [NP_001029025.1](http://www.ncbi.nlm.nih.gov/entrez/query.fcgi?cmd=Retrieve&db=Protein&list_uids=76496472&dopt=GenPept&RID=TFN66FGG01S&log$=prottop&blast_rank=34) | ribosomal protein L3 isoform b [Homo sapiens] | [18.5](http://blast.ncbi.nlm.nih.gov/Blast.cgi#76496472%2376496472) | 18.5 | 75% | 386 |
| [NP_075061.1](http://www.ncbi.nlm.nih.gov/entrez/query.fcgi?cmd=Retrieve&db=Protein&list_uids=13430864&dopt=GenPept&RID=TFN66FGG01S&log$=prottop&blast_rank=35) | protein NDRG4 isoform 1 [Homo sapiens] >ref|NP_065198.1| protein NDRG4 isoform 1 [Homo sapiens] | [18.5](http://blast.ncbi.nlm.nih.gov/Blast.cgi#13430864%2313430864) | 18.5 | 75% | 386 |
| [NP_000629.3](http://www.ncbi.nlm.nih.gov/entrez/query.fcgi?cmd=Retrieve&db=Protein&list_uids=88853069&dopt=GenPept&RID=TFN66FGG01S&log$=prottop&blast_rank=36) | vitronectin precursor [Homo sapiens] | [18.5](http://blast.ncbi.nlm.nih.gov/Blast.cgi#88853069%2388853069) | 18.5 | 75% | 386 |
| [NP_000958.1](http://www.ncbi.nlm.nih.gov/entrez/query.fcgi?cmd=Retrieve&db=Protein&list_uids=4506649&dopt=GenPept&RID=TFN66FGG01S&log$=prottop&blast_rank=37) | ribosomal protein L3 isoform a [Homo sapiens] | [18.5](http://blast.ncbi.nlm.nih.gov/Blast.cgi#4506649%234506649) | 18.5 | 75% | 386 |
| [NP_001158095.1](http://www.ncbi.nlm.nih.gov/entrez/query.fcgi?cmd=Retrieve&db=Protein&list_uids=257467482&dopt=GenPept&RID=TFN66FGG01S&log$=prottop&blast_rank=38) | protein argonaute-2 isoform 2 [Homo sapiens] | [18.0](http://blast.ncbi.nlm.nih.gov/Blast.cgi#257467482%23257467482) | 18.0 | 100% | 518 |
| [NP_001139291.1](http://www.ncbi.nlm.nih.gov/entrez/query.fcgi?cmd=Retrieve&db=Protein&list_uids=224967054&dopt=GenPept&RID=TFN66FGG01S&log$=prottop&blast_rank=39) | SRY (sex determining region Y)-box 6 isoform 4 [Homo sapiens] | [18.0](http://blast.ncbi.nlm.nih.gov/Blast.cgi#224967054%23224967054) | 18.0 | 87% | 518 |
| [NP_001139283.1](http://www.ncbi.nlm.nih.gov/entrez/query.fcgi?cmd=Retrieve&db=Protein&list_uids=224967050&dopt=GenPept&RID=TFN66FGG01S&log$=prottop&blast_rank=40) | SRY (sex determining region Y)-box 6 isoform 3 [Homo sapiens] | [18.0](http://blast.ncbi.nlm.nih.gov/Blast.cgi#224967050%23224967050) | 18.0 | 87% | 518 |
| [NP_001026.2](http://www.ncbi.nlm.nih.gov/entrez/query.fcgi?cmd=Retrieve&db=Protein&list_uids=112799847&dopt=GenPept&RID=TFN66FGG01S&log$=prottop&blast_rank=41) | cardiac muscle ryanodine receptor [Homo sapiens] | [18.0](http://blast.ncbi.nlm.nih.gov/Blast.cgi#112799847%23112799847) | 18.0 | 75% | 518 |
| [NP_056099.2](http://www.ncbi.nlm.nih.gov/entrez/query.fcgi?cmd=Retrieve&db=Protein&list_uids=71999153&dopt=GenPept&RID=TFN66FGG01S&log$=prottop&blast_rank=42) | hypothetical protein LOC23334 [Homo sapiens] | [18.0](http://blast.ncbi.nlm.nih.gov/Blast.cgi#71999153%2371999153) | 18.0 | 75% | 518 |
| [NP_059978.1](http://www.ncbi.nlm.nih.gov/entrez/query.fcgi?cmd=Retrieve&db=Protein&list_uids=70909338&dopt=GenPept&RID=TFN66FGG01S&log$=prottop&blast_rank=43) | SRY (sex determining region Y)-box 6 isoform 1 [Homo sapiens] | [18.0](http://blast.ncbi.nlm.nih.gov/Blast.cgi#70909338%2370909338) | 18.0 | 87% | 518 |
| [NP_201583.2](http://www.ncbi.nlm.nih.gov/entrez/query.fcgi?cmd=Retrieve&db=Protein&list_uids=71037389&dopt=GenPept&RID=TFN66FGG01S&log$=prottop&blast_rank=44) | SRY (sex determining region Y)-box 6 isoform 2 [Homo sapiens] | [18.0](http://blast.ncbi.nlm.nih.gov/Blast.cgi#71037389%2371037389) | 18.0 | 87% | 518 |
| [NP_877954.1](http://www.ncbi.nlm.nih.gov/entrez/query.fcgi?cmd=Retrieve&db=Protein&list_uids=33469924&dopt=GenPept&RID=TFN66FGG01S&log$=prottop&blast_rank=45) | DNA replication licensing factor MCM8 isoform 2 [Homo sapiens] | [18.0](http://blast.ncbi.nlm.nih.gov/Blast.cgi#33469924%2333469924) | 18.0 | 75% | 518 |
| [NP_036286.2](http://www.ncbi.nlm.nih.gov/entrez/query.fcgi?cmd=Retrieve&db=Protein&list_uids=29171734&dopt=GenPept&RID=TFN66FGG01S&log$=prottop&blast_rank=46) | protein argonaute-2 isoform 1 [Homo sapiens] | [18.0](http://blast.ncbi.nlm.nih.gov/Blast.cgi#29171734%2329171734) | 18.0 | 100% | 518 |
| [NP_068758.3](http://www.ncbi.nlm.nih.gov/entrez/query.fcgi?cmd=Retrieve&db=Protein&list_uids=192448443&dopt=GenPept&RID=TFN66FGG01S&log$=prottop&blast_rank=47) | peptidyl-prolyl cis-trans isomerase FKBP10 precursor [Homo sapiens] | [18.0](http://blast.ncbi.nlm.nih.gov/Blast.cgi#192448443%23192448443) | 18.0 | 75% | 518 |
| [NP_037458.3](http://www.ncbi.nlm.nih.gov/entrez/query.fcgi?cmd=Retrieve&db=Protein&list_uids=89242141&dopt=GenPept&RID=TFN66FGG01S&log$=prottop&blast_rank=48) | colon cancer-associated protein Mic1 [Homo sapiens] | [18.0](http://blast.ncbi.nlm.nih.gov/Blast.cgi#89242141%2389242141) | 18.0 | 75% | 518 |
| [NP_115874.3](http://www.ncbi.nlm.nih.gov/entrez/query.fcgi?cmd=Retrieve&db=Protein&list_uids=19923727&dopt=GenPept&RID=TFN66FGG01S&log$=prottop&blast_rank=49) | DNA replication licensing factor MCM8 isoform 1 [Homo sapiens] | [18.0](http://blast.ncbi.nlm.nih.gov/Blast.cgi#19923727%2319923727) | 18.0 | 75% | 518 |
| [NP_001159409.1](http://www.ncbi.nlm.nih.gov/entrez/query.fcgi?cmd=Retrieve&db=Protein&list_uids=260763904&dopt=GenPept&RID=TFN66FGG01S&log$=prottop&blast_rank=50) | stAR-related lipid transfer protein 3 isoform 2 [Homo sapiens] | [17.6](http://blast.ncbi.nlm.nih.gov/Blast.cgi#260763904%23260763904) | 17.6 | 75% | 695 |
| [NP_071330.3](http://www.ncbi.nlm.nih.gov/entrez/query.fcgi?cmd=Retrieve&db=Protein&list_uids=145312241&dopt=GenPept&RID=TFN66FGG01S&log$=prottop&blast_rank=51) | differentially expressed in FDCP 6 homolog [Homo sapiens] | [17.6](http://blast.ncbi.nlm.nih.gov/Blast.cgi#145312241%23145312241) | 31.4 | 75% | 695 |
| [NP_114440.1](http://www.ncbi.nlm.nih.gov/entrez/query.fcgi?cmd=Retrieve&db=Protein&list_uids=14670364&dopt=GenPept&RID=TFN66FGG01S&log$=prottop&blast_rank=52) | POZ-, AT hook-, and zinc finger-containing protein 1 short isoform [Homo sapiens] | [17.6](http://blast.ncbi.nlm.nih.gov/Blast.cgi#14670364%2314670364) | 17.6 | 75% | 695 |
| [NP_055649.4](http://www.ncbi.nlm.nih.gov/entrez/query.fcgi?cmd=Retrieve&db=Protein&list_uids=289547512&dopt=GenPept&RID=TFN66FGG01S&log$=prottop&blast_rank=53) | leucine-rich repeat-containing protein 37A precursor [Homo sapiens] | [17.2](http://blast.ncbi.nlm.nih.gov/Blast.cgi#289547512%23289547512) | 17.2 | 50% | 932 |
| [XP_002344803.1](http://www.ncbi.nlm.nih.gov/entrez/query.fcgi?cmd=Retrieve&db=Protein&list_uids=239756104&dopt=GenPept&RID=TFN66FGG01S&log$=prottop&blast_rank=54) | PREDICTED: tetratricopeptide repeat domain 6 [Homo sapiens] | [17.2](http://blast.ncbi.nlm.nih.gov/Blast.cgi#239756104%23239756104) | 31.8 | 50% | 932 |
| [XP_002343340.1](http://www.ncbi.nlm.nih.gov/entrez/query.fcgi?cmd=Retrieve&db=Protein&list_uids=239744942&dopt=GenPept&RID=TFN66FGG01S&log$=prottop&blast_rank=55) | PREDICTED: chromosome 14 open reading frame 25 [Homo sapiens] | [17.2](http://blast.ncbi.nlm.nih.gov/Blast.cgi#239744942%23239744942) | 31.8 | 50% | 932 |
| [XP_002342459.1](http://www.ncbi.nlm.nih.gov/entrez/query.fcgi?cmd=Retrieve&db=Protein&list_uids=239742247&dopt=GenPept&RID=TFN66FGG01S&log$=prottop&blast_rank=56) | PREDICTED: hypothetical protein XP_002342459 [Homo sapiens] >ref|XP_002346634.1| PREDICTED: hypothetical protein XP_002346634 [Homo sapiens] >ref|XP_002345778.1| PREDICTED: hypothetical protein [Homo sapiens] | [17.2](http://blast.ncbi.nlm.nih.gov/Blast.cgi#239742247%23239742247) | 17.2 | 75% | 932 |
| [XP_002344102.1](http://www.ncbi.nlm.nih.gov/entrez/query.fcgi?cmd=Retrieve&db=Protein&list_uids=239740955&dopt=GenPept&RID=TFN66FGG01S&log$=prottop&blast_rank=57) | PREDICTED: similar to c114 SLIT-like testicular protein isoform 1 [Homo sapiens] | [17.2](http://blast.ncbi.nlm.nih.gov/Blast.cgi#239740955%23239740955) | 17.2 | 50% | 932 |
| [XP_002344103.1](http://www.ncbi.nlm.nih.gov/entrez/query.fcgi?cmd=Retrieve&db=Protein&list_uids=239740953&dopt=GenPept&RID=TFN66FGG01S&log$=prottop&blast_rank=58) | PREDICTED: similar to c114 SLIT-like testicular protein isoform 2 [Homo sapiens] | [17.2](http://blast.ncbi.nlm.nih.gov/Blast.cgi#239740953%23239740953) | 17.2 | 50% | 932 |
| [NP_001138498.1](http://www.ncbi.nlm.nih.gov/entrez/query.fcgi?cmd=Retrieve&db=Protein&list_uids=222537743&dopt=GenPept&RID=TFN66FGG01S&log$=prottop&blast_rank=59) | phosphotidylinositol phosphatase PTPRQ [Homo sapiens] | [17.2](http://blast.ncbi.nlm.nih.gov/Blast.cgi#222537743%23222537743) | 17.2 | 50% | 932 |
| [NP_001153519.1](http://www.ncbi.nlm.nih.gov/entrez/query.fcgi?cmd=Retrieve&db=Protein&list_uids=237648961&dopt=GenPept&RID=TFN66FGG01S&log$=prottop&blast_rank=60) | thioredoxin domain-containing protein 16 isoform 2 [Homo sapiens] | [17.2](http://blast.ncbi.nlm.nih.gov/Blast.cgi#237648961%23237648961) | 17.2 | 50% | 932 |
| [NP_001121600.1](http://www.ncbi.nlm.nih.gov/entrez/query.fcgi?cmd=Retrieve&db=Protein&list_uids=189409130&dopt=GenPept&RID=TFN66FGG01S&log$=prottop&blast_rank=61) | zinc finger E-box-binding homeobox 1 isoform a [Homo sapiens] | [17.2](http://blast.ncbi.nlm.nih.gov/Blast.cgi#189409130%23189409130) | 17.2 | 62% | 932 |
| [NP_004002.2](http://www.ncbi.nlm.nih.gov/entrez/query.fcgi?cmd=Retrieve&db=Protein&list_uids=150036268&dopt=GenPept&RID=TFN66FGG01S&log$=prottop&blast_rank=62) | dystrophin Dp260-1 isoform [Homo sapiens] | [17.2](http://blast.ncbi.nlm.nih.gov/Blast.cgi#150036268%23150036268) | 17.2 | 50% | 932 |
| [NP_004013.1](http://www.ncbi.nlm.nih.gov/entrez/query.fcgi?cmd=Retrieve&db=Protein&list_uids=5032311&dopt=GenPept&RID=TFN66FGG01S&log$=prottop&blast_rank=63) | dystrophin Dp140ab isoform [Homo sapiens] | [17.2](http://blast.ncbi.nlm.nih.gov/Blast.cgi#5032311%235032311) | 17.2 | 50% | 932 |
| [NP_000100.2](http://www.ncbi.nlm.nih.gov/entrez/query.fcgi?cmd=Retrieve&db=Protein&list_uids=5032281&dopt=GenPept&RID=TFN66FGG01S&log$=prottop&blast_rank=64) | dystrophin Dp427c isoform [Homo sapiens] | [17.2](http://blast.ncbi.nlm.nih.gov/Blast.cgi#5032281%235032281) | 17.2 | 50% | 932 |
| [NP_003997.1](http://www.ncbi.nlm.nih.gov/entrez/query.fcgi?cmd=Retrieve&db=Protein&list_uids=5032283&dopt=GenPept&RID=TFN66FGG01S&log$=prottop&blast_rank=65) | dystrophin Dp427m isoform [Homo sapiens] | [17.2](http://blast.ncbi.nlm.nih.gov/Blast.cgi#5032283%235032283) | 17.2 | 50% | 932 |
| [NP_076991.3](http://www.ncbi.nlm.nih.gov/entrez/query.fcgi?cmd=Retrieve&db=Protein&list_uids=122114654&dopt=GenPept&RID=TFN66FGG01S&log$=prottop&blast_rank=66) | putative methyltransferase METT10D [Homo sapiens] | [17.2](http://blast.ncbi.nlm.nih.gov/Blast.cgi#122114654%23122114654) | 17.2 | 50% | 932 |
| [XP_002347467.1](http://www.ncbi.nlm.nih.gov/entrez/query.fcgi?cmd=Retrieve&db=Protein&list_uids=239750633&dopt=GenPept&RID=TFN66FGG01S&log$=prottop&blast_rank=67) | PREDICTED: tetratricopeptide repeat domain 6 [Homo sapiens] | [17.2](http://blast.ncbi.nlm.nih.gov/Blast.cgi#239750633%23239750633) | 31.8 | 50% | 932 |
| [NP_065835.2](http://www.ncbi.nlm.nih.gov/entrez/query.fcgi?cmd=Retrieve&db=Protein&list_uids=237648959&dopt=GenPept&RID=TFN66FGG01S&log$=prottop&blast_rank=68) | thioredoxin domain-containing protein 16 isoform 1 [Homo sapiens] | [17.2](http://blast.ncbi.nlm.nih.gov/Blast.cgi#237648959%23237648959) | 17.2 | 50% | 932 |
| [NP_001006608.2](http://www.ncbi.nlm.nih.gov/entrez/query.fcgi?cmd=Retrieve&db=Protein&list_uids=116325993&dopt=GenPept&RID=TFN66FGG01S&log$=prottop&blast_rank=69) | c114 SLIT-like testicular protein precursor [Homo sapiens] | [17.2](http://blast.ncbi.nlm.nih.gov/Blast.cgi#116325993%23116325993) | 17.2 | 50% | 932 |
| [NP_006624.2](http://www.ncbi.nlm.nih.gov/entrez/query.fcgi?cmd=Retrieve&db=Protein&list_uids=116089337&dopt=GenPept&RID=TFN66FGG01S&log$=prottop&blast_rank=70) | ras GTPase-activating-like protein IQGAP2 [Homo sapiens] | [17.2](http://blast.ncbi.nlm.nih.gov/Blast.cgi#116089337%23116089337) | 17.2 | 50% | 932 |
| [NP_055907.3](http://www.ncbi.nlm.nih.gov/entrez/query.fcgi?cmd=Retrieve&db=Protein&list_uids=62243658&dopt=GenPept&RID=TFN66FGG01S&log$=prottop&blast_rank=71) | serine/threonine-protein kinase SMG1 [Homo sapiens] | [17.2](http://blast.ncbi.nlm.nih.gov/Blast.cgi#62243658%2362243658) | 17.2 | 50% | 932 |
| [NP_001008749.2](http://www.ncbi.nlm.nih.gov/entrez/query.fcgi?cmd=Retrieve&db=Protein&list_uids=239915976&dopt=GenPept&RID=TFN66FGG01S&log$=prottop&blast_rank=72) | ras-related protein Rab-19 [Homo sapiens] | [17.2](http://blast.ncbi.nlm.nih.gov/Blast.cgi#239915976%23239915976) | 17.2 | 50% | 932 |
| [NP_057209.3](http://www.ncbi.nlm.nih.gov/entrez/query.fcgi?cmd=Retrieve&db=Protein&list_uids=109134327&dopt=GenPept&RID=TFN66FGG01S&log$=prottop&blast_rank=73) | RING finger and transmembrane domain-containing protein 1 [Homo sapiens] | [17.2](http://blast.ncbi.nlm.nih.gov/Blast.cgi#109134327%23109134327) | 17.2 | 50% | 932 |
| [NP_066015.2](http://www.ncbi.nlm.nih.gov/entrez/query.fcgi?cmd=Retrieve&db=Protein&list_uids=93204865&dopt=GenPept&RID=TFN66FGG01S&log$=prottop&blast_rank=74) | hypothetical protein LOC57724 [Homo sapiens] | [17.2](http://blast.ncbi.nlm.nih.gov/Blast.cgi#93204865%2393204865) | 17.2 | 50% | 932 |
| [NP_004012.1](http://www.ncbi.nlm.nih.gov/entrez/query.fcgi?cmd=Retrieve&db=Protein&list_uids=5032309&dopt=GenPept&RID=TFN66FGG01S&log$=prottop&blast_rank=75) | dystrophin Dp140b isoform [Homo sapiens] | [17.2](http://blast.ncbi.nlm.nih.gov/Blast.cgi#5032309%235032309) | 17.2 | 50% | 932 |
| [NP_004010.1](http://www.ncbi.nlm.nih.gov/entrez/query.fcgi?cmd=Retrieve&db=Protein&list_uids=5032305&dopt=GenPept&RID=TFN66FGG01S&log$=prottop&blast_rank=76) | dystrophin Dp40 isoform [Homo sapiens] | [17.2](http://blast.ncbi.nlm.nih.gov/Blast.cgi#5032305%235032305) | 17.2 | 50% | 932 |
| [NP_004004.1](http://www.ncbi.nlm.nih.gov/entrez/query.fcgi?cmd=Retrieve&db=Protein&list_uids=5032293&dopt=GenPept&RID=TFN66FGG01S&log$=prottop&blast_rank=77) | dystrophin Dp140 isoform [Homo sapiens] | [17.2](http://blast.ncbi.nlm.nih.gov/Blast.cgi#5032293%235032293) | 17.2 | 50% | 932 |
| [NP_689976.2](http://www.ncbi.nlm.nih.gov/entrez/query.fcgi?cmd=Retrieve&db=Protein&list_uids=91754185&dopt=GenPept&RID=TFN66FGG01S&log$=prottop&blast_rank=78) | AKNA domain containing 1 [Homo sapiens] | [17.2](http://blast.ncbi.nlm.nih.gov/Blast.cgi#91754185%2391754185) | 17.2 | 75% | 932 |
| [NP_009055.2](http://www.ncbi.nlm.nih.gov/entrez/query.fcgi?cmd=Retrieve&db=Protein&list_uids=110611228&dopt=GenPept&RID=TFN66FGG01S&log$=prottop&blast_rank=79) | utrophin [Homo sapiens] | [17.2](http://blast.ncbi.nlm.nih.gov/Blast.cgi#110611228%23110611228) | 17.2 | 50% | 932 |
| [NP_733842.2](http://www.ncbi.nlm.nih.gov/entrez/query.fcgi?cmd=Retrieve&db=Protein&list_uids=126131104&dopt=GenPept&RID=TFN66FGG01S&log$=prottop&blast_rank=80) | fibrocystin isoform 2 [Homo sapiens] | [17.2](http://blast.ncbi.nlm.nih.gov/Blast.cgi#126131104%23126131104) | 29.3 | 100% | 932 |
| [NP_008995.2](http://www.ncbi.nlm.nih.gov/entrez/query.fcgi?cmd=Retrieve&db=Protein&list_uids=68362740&dopt=GenPept&RID=TFN66FGG01S&log$=prottop&blast_rank=81) | kalirin isoform 3 [Homo sapiens] | [17.2](http://blast.ncbi.nlm.nih.gov/Blast.cgi#68362740%2368362740) | 17.2 | 62% | 932 |
| [NP_000144.2](http://www.ncbi.nlm.nih.gov/entrez/query.fcgi?cmd=Retrieve&db=Protein&list_uids=83281450&dopt=GenPept&RID=TFN66FGG01S&log$=prottop&blast_rank=82) | galactosylceramidase isoform a precursor [Homo sapiens] | [17.2](http://blast.ncbi.nlm.nih.gov/Blast.cgi#83281450%2383281450) | 17.2 | 50% | 932 |
| [NP_057605.3](http://www.ncbi.nlm.nih.gov/entrez/query.fcgi?cmd=Retrieve&db=Protein&list_uids=189409125&dopt=GenPept&RID=TFN66FGG01S&log$=prottop&blast_rank=83) | transcription factor Dp family member 3 [Homo sapiens] | [17.2](http://blast.ncbi.nlm.nih.gov/Blast.cgi#189409125%23189409125) | 17.2 | 50% | 932 |
| [NP_653312.2](http://www.ncbi.nlm.nih.gov/entrez/query.fcgi?cmd=Retrieve&db=Protein&list_uids=40255071&dopt=GenPept&RID=TFN66FGG01S&log$=prottop&blast_rank=84) | kelch-like protein 23 [Homo sapiens] | [17.2](http://blast.ncbi.nlm.nih.gov/Blast.cgi#40255071%2340255071) | 17.2 | 50% | 932 |
| [NP_004007.1](http://www.ncbi.nlm.nih.gov/entrez/query.fcgi?cmd=Retrieve&db=Protein&list_uids=5032299&dopt=GenPept&RID=TFN66FGG01S&log$=prottop&blast_rank=85) | dystrophin Dp71b isoform [Homo sapiens] | [17.2](http://blast.ncbi.nlm.nih.gov/Blast.cgi#5032299%235032299) | 17.2 | 50% | 932 |
| [NP_001034806.1](http://www.ncbi.nlm.nih.gov/entrez/query.fcgi?cmd=Retrieve&db=Protein&list_uids=89363030&dopt=GenPept&RID=TFN66FGG01S&log$=prottop&blast_rank=86) | hypothetical protein LOC80167 precursor [Homo sapiens] | [17.2](http://blast.ncbi.nlm.nih.gov/Blast.cgi#89363030%2389363030) | 17.2 | 50% | 932 |
| [NP_079082.2](http://www.ncbi.nlm.nih.gov/entrez/query.fcgi?cmd=Retrieve&db=Protein&list_uids=40217799&dopt=GenPept&RID=TFN66FGG01S&log$=prottop&blast_rank=87) | hypothetical protein LOC79864 isoform 1 [Homo sapiens] | [17.2](http://blast.ncbi.nlm.nih.gov/Blast.cgi#40217799%2340217799) | 17.2 | 62% | 932 |
| [NP_004009.1](http://www.ncbi.nlm.nih.gov/entrez/query.fcgi?cmd=Retrieve&db=Protein&list_uids=5032303&dopt=GenPept&RID=TFN66FGG01S&log$=prottop&blast_rank=88) | dystrophin Dp71ab isoform [Homo sapiens] | [17.2](http://blast.ncbi.nlm.nih.gov/Blast.cgi#5032303%235032303) | 17.2 | 50% | 932 |
| [NP_000430.3](http://www.ncbi.nlm.nih.gov/entrez/query.fcgi?cmd=Retrieve&db=Protein&list_uids=20336242&dopt=GenPept&RID=TFN66FGG01S&log$=prottop&blast_rank=89) | proprotein convertase subtilisin/kexin type 1 preproprotein [Homo sapiens] | [17.2](http://blast.ncbi.nlm.nih.gov/Blast.cgi#20336242%2320336242) | 17.2 | 50% | 932 |
| [NP_001032614.1](http://www.ncbi.nlm.nih.gov/entrez/query.fcgi?cmd=Retrieve&db=Protein&list_uids=83281452&dopt=GenPept&RID=TFN66FGG01S&log$=prottop&blast_rank=90) | galactosylceramidase isoform b precursor [Homo sapiens] | [17.2](http://blast.ncbi.nlm.nih.gov/Blast.cgi#83281452%2383281452) | 17.2 | 50% | 932 |
| [NP_001093632.1](http://www.ncbi.nlm.nih.gov/entrez/query.fcgi?cmd=Retrieve&db=Protein&list_uids=154448896&dopt=GenPept&RID=TFN66FGG01S&log$=prottop&blast_rank=91) | exportin-7 isoform c [Homo sapiens] | [17.2](http://blast.ncbi.nlm.nih.gov/Blast.cgi#154448896%23154448896) | 17.2 | 50% | 932 |
| [NP_060200.2](http://www.ncbi.nlm.nih.gov/entrez/query.fcgi?cmd=Retrieve&db=Protein&list_uids=38570097&dopt=GenPept&RID=TFN66FGG01S&log$=prottop&blast_rank=92) | glutamine-rich protein 1 [Homo sapiens] >ref|NP_942581.1| glutamine-rich protein 1 [Homo sapiens] | [17.2](http://blast.ncbi.nlm.nih.gov/Blast.cgi#38570097%2338570097) | 17.2 | 50% | 932 |
| [NP_061722.1](http://www.ncbi.nlm.nih.gov/entrez/query.fcgi?cmd=Retrieve&db=Protein&list_uids=9256600&dopt=GenPept&RID=TFN66FGG01S&log$=prottop&blast_rank=93) | protocadherin alpha subfamily C, 2 isoform 1 precursor [Homo sapiens] | [17.2](http://blast.ncbi.nlm.nih.gov/Blast.cgi#9256600%239256600) | 17.2 | 75% | 932 |
| [NP_114089.1](http://www.ncbi.nlm.nih.gov/entrez/query.fcgi?cmd=Retrieve&db=Protein&list_uids=14165431&dopt=GenPept&RID=TFN66FGG01S&log$=prottop&blast_rank=94) | protocadherin alpha subfamily C, 2 isoform 2 precursor [Homo sapiens] | [17.2](http://blast.ncbi.nlm.nih.gov/Blast.cgi#14165431%2314165431) | 17.2 | 75% | 932 |
| [NP_003875.3](http://www.ncbi.nlm.nih.gov/entrez/query.fcgi?cmd=Retrieve&db=Protein&list_uids=40805843&dopt=GenPept&RID=TFN66FGG01S&log$=prottop&blast_rank=95) | histone acetyltransferase KAT2B [Homo sapiens] | [17.2](http://blast.ncbi.nlm.nih.gov/Blast.cgi#40805843%2340805843) | 17.2 | 50% | 932 |
| [NP_065716.1](http://www.ncbi.nlm.nih.gov/entrez/query.fcgi?cmd=Retrieve&db=Protein&list_uids=15042969&dopt=GenPept&RID=TFN66FGG01S&log$=prottop&blast_rank=96) | transmembrane protein 27 precursor [Homo sapiens] | [17.2](http://blast.ncbi.nlm.nih.gov/Blast.cgi#15042969%2315042969) | 17.2 | 50% | 932 |
| [NP_066564.2](http://www.ncbi.nlm.nih.gov/entrez/query.fcgi?cmd=Retrieve&db=Protein&list_uids=153791535&dopt=GenPept&RID=TFN66FGG01S&log$=prottop&blast_rank=97) | histone acetyltransferase KAT2A [Homo sapiens] | [17.2](http://blast.ncbi.nlm.nih.gov/Blast.cgi#153791535%23153791535) | 17.2 | 50% | 932 |
| [NP_955372.2](http://www.ncbi.nlm.nih.gov/entrez/query.fcgi?cmd=Retrieve&db=Protein&list_uids=75677612&dopt=GenPept&RID=TFN66FGG01S&log$=prottop&blast_rank=98) | leucine-rich repeat-containing protein 37A3 precursor [Homo sapiens] | [17.2](http://blast.ncbi.nlm.nih.gov/Blast.cgi#75677612%2375677612) | 17.2 | 50% | 932 |
| [NP_940959.1](http://www.ncbi.nlm.nih.gov/entrez/query.fcgi?cmd=Retrieve&db=Protein&list_uids=38348394&dopt=GenPept&RID=TFN66FGG01S&log$=prottop&blast_rank=99) | RNA-binding protein 43 [Homo sapiens] | [17.2](http://blast.ncbi.nlm.nih.gov/Blast.cgi#38348394%2338348394) | 17.2 | 50% | 932 |
| [NP_055839.3](http://www.ncbi.nlm.nih.gov/entrez/query.fcgi?cmd=Retrieve&db=Protein&list_uids=154448892&dopt=GenPept&RID=TFN66FGG01S&log$=prottop&blast_rank=100) | exportin-7 isoform b [Homo sapiens] | [17.2](http://blast.ncbi.nlm.nih.gov/Blast.cgi#154448892%23154448892) | 17.2 | 50% | 932 |

| **Accession** | **Proteins with a match to ESSTKSE peptide** | **[Max score](http://blast.ncbi.nlm.nih.gov/Blast.cgi?CMD=Get&ALIGNMENTS=100&ALIGNMENT_VIEW=Pairwise&CDD_SEARCH_STATE=1&DATABASE_SORT=0&DESCRIPTIONS=100&ENTREZ_QUERY=txid9606 %5BORGN%5D&FIRST_QUERY_NUM=0&FORMAT_OBJECT=Alignment&FORMAT_PAGE_TARGET=&FORMAT_TYPE=HTML&GET_SEQUENCE=yes&I_THRESH=&MASK_CHAR=2&MASK_COLOR=1&NEW_VIEW=yes&NUM_OVERVIEW=100&OLD_BLAST=false&PAGE=Proteins&QUERY_INDEX=0&QUERY_NUMBER=0&RESULTS_PAGE_TARGET=&RID=TFNA0JEC01N&SHOW_LINKOUT=yes&SHOW_OVERVIEW=yes&STEP_NUMBER=&WORD_SIZE=2&DISPLAY_SORT=1&HSP_SORT=1" \l "sort_mark)** | **[Total score](http://blast.ncbi.nlm.nih.gov/Blast.cgi?CMD=Get&ALIGNMENTS=100&ALIGNMENT_VIEW=Pairwise&CDD_SEARCH_STATE=1&DATABASE_SORT=0&DESCRIPTIONS=100&ENTREZ_QUERY=txid9606 %5BORGN%5D&FIRST_QUERY_NUM=0&FORMAT_OBJECT=Alignment&FORMAT_PAGE_TARGET=&FORMAT_TYPE=HTML&GET_SEQUENCE=yes&I_THRESH=&MASK_CHAR=2&MASK_COLOR=1&NEW_VIEW=yes&NUM_OVERVIEW=100&OLD_BLAST=false&PAGE=Proteins&QUERY_INDEX=0&QUERY_NUMBER=0&RESULTS_PAGE_TARGET=&RID=TFNA0JEC01N&SHOW_LINKOUT=yes&SHOW_OVERVIEW=yes&STEP_NUMBER=&WORD_SIZE=2&DISPLAY_SORT=2&HSP_SORT=1" \l "sort_mark)** | **[Query coverage](http://blast.ncbi.nlm.nih.gov/Blast.cgi?CMD=Get&ALIGNMENTS=100&ALIGNMENT_VIEW=Pairwise&CDD_SEARCH_STATE=1&DATABASE_SORT=0&DESCRIPTIONS=100&ENTREZ_QUERY=txid9606 %5BORGN%5D&FIRST_QUERY_NUM=0&FORMAT_OBJECT=Alignment&FORMAT_PAGE_TARGET=&FORMAT_TYPE=HTML&GET_SEQUENCE=yes&I_THRESH=&MASK_CHAR=2&MASK_COLOR=1&NEW_VIEW=yes&NUM_OVERVIEW=100&OLD_BLAST=false&PAGE=Proteins&QUERY_INDEX=0&QUERY_NUMBER=0&RESULTS_PAGE_TARGET=&RID=TFNA0JEC01N&SHOW_LINKOUT=yes&SHOW_OVERVIEW=yes&STEP_NUMBER=&WORD_SIZE=2&DISPLAY_SORT=4&HSP_SORT=0" \l "sort_mark)** | **[E value](http://blast.ncbi.nlm.nih.gov/Blast.cgi?CMD=Get&ALIGNMENTS=100&ALIGNMENT_VIEW=Pairwise&CDD_SEARCH_STATE=1&DATABASE_SORT=0&DESCRIPTIONS=100&ENTREZ_QUERY=txid9606 %5BORGN%5D&FIRST_QUERY_NUM=0&FORMAT_OBJECT=Alignment&FORMAT_PAGE_TARGET=&FORMAT_TYPE=HTML&GET_SEQUENCE=yes&I_THRESH=&MASK_CHAR=2&MASK_COLOR=1&NEW_VIEW=yes&NUM_OVERVIEW=100&OLD_BLAST=false&PAGE=Proteins&QUERY_INDEX=0&QUERY_NUMBER=0&RESULTS_PAGE_TARGET=&RID=TFNA0JEC01N&SHOW_LINKOUT=yes&SHOW_OVERVIEW=yes&STEP_NUMBER=&WORD_SIZE=2&DISPLAY_SORT=0&HSP_SORT=0" \l "sort_mark)** |
| --- | --- | --- | --- | --- | --- |
| [NP_667345.1](http://www.ncbi.nlm.nih.gov/entrez/query.fcgi?cmd=Retrieve&db=Protein&list_uids=22212927&dopt=GenPept&RID=TFNA0JEC01N&log$=prottop&blast_rank=1) | nuclear transcription factor, X-box binding 1 isoform 3 [Homo sapiens] | [20.6](http://blast.ncbi.nlm.nih.gov/Blast.cgi#22212927%2322212927) | 20.6 | 100% | 78 |
| [NP_002495.2](http://www.ncbi.nlm.nih.gov/entrez/query.fcgi?cmd=Retrieve&db=Protein&list_uids=21361137&dopt=GenPept&RID=TFNA0JEC01N&log$=prottop&blast_rank=2) | nuclear transcription factor, X-box binding 1 isoform 1 [Homo sapiens] | [20.6](http://blast.ncbi.nlm.nih.gov/Blast.cgi#21361137%2321361137) | 20.6 | 100% | 78 |
| [NP_667344.1](http://www.ncbi.nlm.nih.gov/entrez/query.fcgi?cmd=Retrieve&db=Protein&list_uids=22212925&dopt=GenPept&RID=TFNA0JEC01N&log$=prottop&blast_rank=3) | nuclear transcription factor, X-box binding 1 isoform 2 [Homo sapiens] | [20.6](http://blast.ncbi.nlm.nih.gov/Blast.cgi#22212925%2322212925) | 20.6 | 100% | 78 |
| [NP_997246.2](http://www.ncbi.nlm.nih.gov/entrez/query.fcgi?cmd=Retrieve&db=Protein&list_uids=126362967&dopt=GenPept&RID=TFNA0JEC01N&log$=prottop&blast_rank=4) | nck-associated protein 5 isoform 1 [Homo sapiens] | [20.2](http://blast.ncbi.nlm.nih.gov/Blast.cgi#126362967%23126362967) | 31.4 | 85% | 104 |
| [NP_683692.2](http://www.ncbi.nlm.nih.gov/entrez/query.fcgi?cmd=Retrieve&db=Protein&list_uids=87299628&dopt=GenPept&RID=TFNA0JEC01N&log$=prottop&blast_rank=5) | biorientation of chromosomes in cell division protein 1-like [Homo sapiens] | [20.2](http://blast.ncbi.nlm.nih.gov/Blast.cgi#87299628%2387299628) | 20.2 | 85% | 104 |
| [NP_001019837.1](http://www.ncbi.nlm.nih.gov/entrez/query.fcgi?cmd=Retrieve&db=Protein&list_uids=67514036&dopt=GenPept&RID=TFNA0JEC01N&log$=prottop&blast_rank=6) | SH3 domain-containing kinase-binding protein 1 isoform b [Homo sapiens] | [20.2](http://blast.ncbi.nlm.nih.gov/Blast.cgi#67514036%2367514036) | 20.2 | 85% | 104 |
| [NP_114098.1](http://www.ncbi.nlm.nih.gov/entrez/query.fcgi?cmd=Retrieve&db=Protein&list_uids=13994242&dopt=GenPept&RID=TFNA0JEC01N&log$=prottop&blast_rank=7) | SH3 domain-containing kinase-binding protein 1 isoform a [Homo sapiens] | [20.2](http://blast.ncbi.nlm.nih.gov/Blast.cgi#13994242%2313994242) | 20.2 | 85% | 104 |
| [NP_001139542.1](http://www.ncbi.nlm.nih.gov/entrez/query.fcgi?cmd=Retrieve&db=Protein&list_uids=225703106&dopt=GenPept&RID=TFNA0JEC01N&log$=prottop&blast_rank=8) | tudor domain containing 3 isoform 1 [Homo sapiens] | [19.3](http://blast.ncbi.nlm.nih.gov/Blast.cgi#225703106%23225703106) | 19.3 | 100% | 188 |
| [NP_071349.3](http://www.ncbi.nlm.nih.gov/entrez/query.fcgi?cmd=Retrieve&db=Protein&list_uids=192449449&dopt=GenPept&RID=TFNA0JEC01N&log$=prottop&blast_rank=9) | ubiquitin-conjugating enzyme E2 O [Homo sapiens] | [19.3](http://blast.ncbi.nlm.nih.gov/Blast.cgi#192449449%23192449449) | 33.5 | 100% | 188 |
| [NP_055625.4](http://www.ncbi.nlm.nih.gov/entrez/query.fcgi?cmd=Retrieve&db=Protein&list_uids=171184451&dopt=GenPept&RID=TFNA0JEC01N&log$=prottop&blast_rank=10) | centrosome-associated protein 350 [Homo sapiens] | [19.3](http://blast.ncbi.nlm.nih.gov/Blast.cgi#171184451%23171184451) | 59.8 | 100% | 188 |
| [NP_110421.1](http://www.ncbi.nlm.nih.gov/entrez/query.fcgi?cmd=Retrieve&db=Protein&list_uids=13540576&dopt=GenPept&RID=TFNA0JEC01N&log$=prottop&blast_rank=11) | tudor domain containing 3 isoform 2 [Homo sapiens] >ref|NP_001139543.1| tudor domain containing 3 isoform 2 [Homo sapiens] | [19.3](http://blast.ncbi.nlm.nih.gov/Blast.cgi#13540576%2313540576) | 19.3 | 100% | 188 |
| [NP_036228.1](http://www.ncbi.nlm.nih.gov/entrez/query.fcgi?cmd=Retrieve&db=Protein&list_uids=6912242&dopt=GenPept&RID=TFNA0JEC01N&log$=prottop&blast_rank=12) | DCC-interacting protein 13-alpha [Homo sapiens] | [18.9](http://blast.ncbi.nlm.nih.gov/Blast.cgi#6912242%236912242) | 43.5 | 100% | 252 |
| [NP_659475.2](http://www.ncbi.nlm.nih.gov/entrez/query.fcgi?cmd=Retrieve&db=Protein&list_uids=217416374&dopt=GenPept&RID=TFNA0JEC01N&log$=prottop&blast_rank=13) | hypothetical protein LOC92749 [Homo sapiens] | [18.0](http://blast.ncbi.nlm.nih.gov/Blast.cgi#217416374%23217416374) | 18.0 | 100% | 453 |
| [NP_001002814.2](http://www.ncbi.nlm.nih.gov/entrez/query.fcgi?cmd=Retrieve&db=Protein&list_uids=289547524&dopt=GenPept&RID=TFNA0JEC01N&log$=prottop&blast_rank=14) | rab11 family-interacting protein 1 isoform 3 [Homo sapiens] | [17.6](http://blast.ncbi.nlm.nih.gov/Blast.cgi#289547524%23289547524) | 17.6 | 71% | 608 |
| [NP_001159698.1](http://www.ncbi.nlm.nih.gov/entrez/query.fcgi?cmd=Retrieve&db=Protein&list_uids=261245061&dopt=GenPept&RID=TFNA0JEC01N&log$=prottop&blast_rank=15) | centrosomal protein of 120 kDa isoform 2 [Homo sapiens] | [17.6](http://blast.ncbi.nlm.nih.gov/Blast.cgi#261245061%23261245061) | 17.6 | 85% | 608 |
| [XP_002344033.1](http://www.ncbi.nlm.nih.gov/entrez/query.fcgi?cmd=Retrieve&db=Protein&list_uids=239740773&dopt=GenPept&RID=TFNA0JEC01N&log$=prottop&blast_rank=16) | PREDICTED: similar to diffuse panbronchiolitis critical region 1 [Homo sapiens] | [17.6](http://blast.ncbi.nlm.nih.gov/Blast.cgi#239740773%23239740773) | 49.4 | 100% | 608 |
| [NP_000251.3](http://www.ncbi.nlm.nih.gov/entrez/query.fcgi?cmd=Retrieve&db=Protein&list_uids=189083798&dopt=GenPept&RID=TFNA0JEC01N&log$=prottop&blast_rank=17) | myosin-VIIa isoform 1 [Homo sapiens] | [17.6](http://blast.ncbi.nlm.nih.gov/Blast.cgi#189083798%23189083798) | 17.6 | 71% | 608 |
| [NP_060639.3](http://www.ncbi.nlm.nih.gov/entrez/query.fcgi?cmd=Retrieve&db=Protein&list_uids=256773222&dopt=GenPept&RID=TFNA0JEC01N&log$=prottop&blast_rank=18) | hypothetical protein LOC55196 [Homo sapiens] | [17.6](http://blast.ncbi.nlm.nih.gov/Blast.cgi#256773222%23256773222) | 28.4 | 85% | 608 |
| [NP_001107590.1](http://www.ncbi.nlm.nih.gov/entrez/query.fcgi?cmd=Retrieve&db=Protein&list_uids=166295179&dopt=GenPept&RID=TFNA0JEC01N&log$=prottop&blast_rank=19) | ELG protein isoform a [Homo sapiens] | [17.6](http://blast.ncbi.nlm.nih.gov/Blast.cgi#166295179%23166295179) | 32.2 | 100% | 608 |
| [NP_001092093.1](http://www.ncbi.nlm.nih.gov/entrez/query.fcgi?cmd=Retrieve&db=Protein&list_uids=148833506&dopt=GenPept&RID=TFNA0JEC01N&log$=prottop&blast_rank=20) | obscurin, cytoskeletal calmodulin and titin-interacting RhoGEF isoform b [Homo sapiens] | [17.6](http://blast.ncbi.nlm.nih.gov/Blast.cgi#148833506%23148833506) | 33.5 | 100% | 608 |
| [NP_073751.3](http://www.ncbi.nlm.nih.gov/entrez/query.fcgi?cmd=Retrieve&db=Protein&list_uids=145275206&dopt=GenPept&RID=TFNA0JEC01N&log$=prottop&blast_rank=21) | methyltransferase like 4 [Homo sapiens] | [17.6](http://blast.ncbi.nlm.nih.gov/Blast.cgi#145275206%23145275206) | 17.6 | 100% | 608 |
| [NP_001123230.1](http://www.ncbi.nlm.nih.gov/entrez/query.fcgi?cmd=Retrieve&db=Protein&list_uids=193211610&dopt=GenPept&RID=TFNA0JEC01N&log$=prottop&blast_rank=22) | dipeptidase 3 isoform b [Homo sapiens] | [17.6](http://blast.ncbi.nlm.nih.gov/Blast.cgi#193211610%23193211610) | 17.6 | 71% | 608 |
| [NP_001120652.1](http://www.ncbi.nlm.nih.gov/entrez/query.fcgi?cmd=Retrieve&db=Protein&list_uids=189083802&dopt=GenPept&RID=TFNA0JEC01N&log$=prottop&blast_rank=23) | myosin-VIIa isoform 2 [Homo sapiens] | [17.6](http://blast.ncbi.nlm.nih.gov/Blast.cgi#189083802%23189083802) | 17.6 | 71% | 608 |
| [NP_001120936.1](http://www.ncbi.nlm.nih.gov/entrez/query.fcgi?cmd=Retrieve&db=Protein&list_uids=188536004&dopt=GenPept&RID=TFNA0JEC01N&log$=prottop&blast_rank=24) | zinc finger protein 469 [Homo sapiens] | [17.6](http://blast.ncbi.nlm.nih.gov/Blast.cgi#188536004%23188536004) | 38.0 | 100% | 608 |
| [NP_002702.2](http://www.ncbi.nlm.nih.gov/entrez/query.fcgi?cmd=Retrieve&db=Protein&list_uids=167614502&dopt=GenPept&RID=TFNA0JEC01N&log$=prottop&blast_rank=25) | protein phosphatase 1 regulatory subunit 3A [Homo sapiens] | [17.6](http://blast.ncbi.nlm.nih.gov/Blast.cgi#167614502%23167614502) | 42.6 | 100% | 608 |
| [NP_000375.2](http://www.ncbi.nlm.nih.gov/entrez/query.fcgi?cmd=Retrieve&db=Protein&list_uids=105990532&dopt=GenPept&RID=TFNA0JEC01N&log$=prottop&blast_rank=26) | apolipoprotein B precursor [Homo sapiens] | [17.6](http://blast.ncbi.nlm.nih.gov/Blast.cgi#105990532%23105990532) | 31.8 | 85% | 608 |
| [NP_694955.2](http://www.ncbi.nlm.nih.gov/entrez/query.fcgi?cmd=Retrieve&db=Protein&list_uids=96975038&dopt=GenPept&RID=TFNA0JEC01N&log$=prottop&blast_rank=27) | centrosomal protein of 120 kDa isoform 1 [Homo sapiens] | [17.6](http://blast.ncbi.nlm.nih.gov/Blast.cgi#96975038%2396975038) | 17.6 | 85% | 608 |
| [NP_060252.3](http://www.ncbi.nlm.nih.gov/entrez/query.fcgi?cmd=Retrieve&db=Protein&list_uids=148806891&dopt=GenPept&RID=TFNA0JEC01N&log$=prottop&blast_rank=28) | hypothetical protein LOC54906 [Homo sapiens] | [17.6](http://blast.ncbi.nlm.nih.gov/Blast.cgi#148806891%23148806891) | 17.6 | 85% | 608 |
| [NP_443723.2](http://www.ncbi.nlm.nih.gov/entrez/query.fcgi?cmd=Retrieve&db=Protein&list_uids=115495445&dopt=GenPept&RID=TFNA0JEC01N&log$=prottop&blast_rank=29) | ankyrin repeat domain-containing protein 30A [Homo sapiens] | [17.6](http://blast.ncbi.nlm.nih.gov/Blast.cgi#115495445%23115495445) | 29.3 | 71% | 608 |
| [NP_001139.3](http://www.ncbi.nlm.nih.gov/entrez/query.fcgi?cmd=Retrieve&db=Protein&list_uids=52426735&dopt=GenPept&RID=TFNA0JEC01N&log$=prottop&blast_rank=30) | ankyrin-2 isoform 1 [Homo sapiens] | [17.6](http://blast.ncbi.nlm.nih.gov/Blast.cgi#52426735%2352426735) | 59.0 | 100% | 608 |
| [NP_001002843.1](http://www.ncbi.nlm.nih.gov/entrez/query.fcgi?cmd=Retrieve&db=Protein&list_uids=50811873&dopt=GenPept&RID=TFNA0JEC01N&log$=prottop&blast_rank=31) | suppressor of hairy wing homolog 4 isoform 2 [Homo sapiens] | [17.6](http://blast.ncbi.nlm.nih.gov/Blast.cgi#50811873%2350811873) | 17.6 | 85% | 608 |
| [NP_001073950.1](http://www.ncbi.nlm.nih.gov/entrez/query.fcgi?cmd=Retrieve&db=Protein&list_uids=122937412&dopt=GenPept&RID=TFNA0JEC01N&log$=prottop&blast_rank=32) | ubiquitin carboxyl-terminal hydrolase 45 [Homo sapiens] | [17.6](http://blast.ncbi.nlm.nih.gov/Blast.cgi#122937412%23122937412) | 17.6 | 71% | 608 |
| [NP_056958.3](http://www.ncbi.nlm.nih.gov/entrez/query.fcgi?cmd=Retrieve&db=Protein&list_uids=42560229&dopt=GenPept&RID=TFNA0JEC01N&log$=prottop&blast_rank=33) | recombining binding protein suppressor of hairless isoform 2 [Homo sapiens] | [17.6](http://blast.ncbi.nlm.nih.gov/Blast.cgi#42560229%2342560229) | 17.6 | 100% | 608 |
| [NP_005340.2](http://www.ncbi.nlm.nih.gov/entrez/query.fcgi?cmd=Retrieve&db=Protein&list_uids=42560227&dopt=GenPept&RID=TFNA0JEC01N&log$=prottop&blast_rank=34) | recombining binding protein suppressor of hairless isoform 1 [Homo sapiens] | [17.6](http://blast.ncbi.nlm.nih.gov/Blast.cgi#42560227%2342560227) | 17.6 | 100% | 608 |
| [NP_849144.2](http://www.ncbi.nlm.nih.gov/entrez/query.fcgi?cmd=Retrieve&db=Protein&list_uids=38490688&dopt=GenPept&RID=TFNA0JEC01N&log$=prottop&blast_rank=35) | immunoglobulin superfamily member 10 precursor [Homo sapiens] | [17.6](http://blast.ncbi.nlm.nih.gov/Blast.cgi#38490688%2338490688) | 54.7 | 100% | 608 |
| [NP_060009.1](http://www.ncbi.nlm.nih.gov/entrez/query.fcgi?cmd=Retrieve&db=Protein&list_uids=24308169&dopt=GenPept&RID=TFNA0JEC01N&log$=prottop&blast_rank=36) | dynein heavy chain 3, axonemal [Homo sapiens] | [17.6](http://blast.ncbi.nlm.nih.gov/Blast.cgi#24308169%2324308169) | 17.6 | 71% | 608 |
| [NP_690618.2](http://www.ncbi.nlm.nih.gov/entrez/query.fcgi?cmd=Retrieve&db=Protein&list_uids=25777598&dopt=GenPept&RID=TFNA0JEC01N&log$=prottop&blast_rank=37) | diacylglycerol kinase delta isoform 2 [Homo sapiens] | [17.6](http://blast.ncbi.nlm.nih.gov/Blast.cgi#25777598%2325777598) | 42.6 | 100% | 608 |
| [NP_002903.3](http://www.ncbi.nlm.nih.gov/entrez/query.fcgi?cmd=Retrieve&db=Protein&list_uids=153792012&dopt=GenPept&RID=TFNA0JEC01N&log$=prottop&blast_rank=38) | DNA polymerase zeta catalytic subunit [Homo sapiens] | [17.6](http://blast.ncbi.nlm.nih.gov/Blast.cgi#153792012%23153792012) | 31.8 | 100% | 608 |
| [NP_085128.2](http://www.ncbi.nlm.nih.gov/entrez/query.fcgi?cmd=Retrieve&db=Protein&list_uids=156139122&dopt=GenPept&RID=TFNA0JEC01N&log$=prottop&blast_rank=39) | methylcytosine dioxygenase TET1 [Homo sapiens] | [17.6](http://blast.ncbi.nlm.nih.gov/Blast.cgi#156139122%23156139122) | 60.7 | 100% | 608 |
| [NP_003639.2](http://www.ncbi.nlm.nih.gov/entrez/query.fcgi?cmd=Retrieve&db=Protein&list_uids=25777596&dopt=GenPept&RID=TFNA0JEC01N&log$=prottop&blast_rank=40) | diacylglycerol kinase delta isoform 1 [Homo sapiens] | [17.6](http://blast.ncbi.nlm.nih.gov/Blast.cgi#25777596%2325777596) | 42.6 | 100% | 608 |
| [NP_659402.1](http://www.ncbi.nlm.nih.gov/entrez/query.fcgi?cmd=Retrieve&db=Protein&list_uids=21450645&dopt=GenPept&RID=TFNA0JEC01N&log$=prottop&blast_rank=41) | tetratricopeptide repeat protein 16 [Homo sapiens] | [17.6](http://blast.ncbi.nlm.nih.gov/Blast.cgi#21450645%2321450645) | 17.6 | 85% | 608 |
| [NP_071752.3](http://www.ncbi.nlm.nih.gov/entrez/query.fcgi?cmd=Retrieve&db=Protein&list_uids=193211608&dopt=GenPept&RID=TFNA0JEC01N&log$=prottop&blast_rank=42) | dipeptidase 3 isoform a [Homo sapiens] | [17.6](http://blast.ncbi.nlm.nih.gov/Blast.cgi#193211608%23193211608) | 17.6 | 71% | 608 |
| [NP_079427.4](http://www.ncbi.nlm.nih.gov/entrez/query.fcgi?cmd=Retrieve&db=Protein&list_uids=289547526&dopt=GenPept&RID=TFNA0JEC01N&log$=prottop&blast_rank=43) | rab11 family-interacting protein 1 isoform 1 [Homo sapiens] | [17.6](http://blast.ncbi.nlm.nih.gov/Blast.cgi#289547526%23289547526) | 17.6 | 71% | 608 |
| [NP_976029.1](http://www.ncbi.nlm.nih.gov/entrez/query.fcgi?cmd=Retrieve&db=Protein&list_uids=42560223&dopt=GenPept&RID=TFNA0JEC01N&log$=prottop&blast_rank=44) | recombining binding protein suppressor of hairless isoform 4 [Homo sapiens] | [17.6](http://blast.ncbi.nlm.nih.gov/Blast.cgi#42560223%2342560223) | 17.6 | 100% | 608 |
| [NP_976028.1](http://www.ncbi.nlm.nih.gov/entrez/query.fcgi?cmd=Retrieve&db=Protein&list_uids=42560225&dopt=GenPept&RID=TFNA0JEC01N&log$=prottop&blast_rank=45) | recombining binding protein suppressor of hairless isoform 3 [Homo sapiens] | [17.6](http://blast.ncbi.nlm.nih.gov/Blast.cgi#42560225%2342560225) | 17.6 | 100% | 608 |
| [NP_006209.2](http://www.ncbi.nlm.nih.gov/entrez/query.fcgi?cmd=Retrieve&db=Protein&list_uids=54792082&dopt=GenPept&RID=TFNA0JEC01N&log$=prottop&blast_rank=46) | phosphatidylinositol-4,5-bisphosphate 3-kinase catalytic subunit alpha isoform [Homo sapiens] | [17.6](http://blast.ncbi.nlm.nih.gov/Blast.cgi#54792082%2354792082) | 17.6 | 85% | 608 |
| [NP_058632.2](http://www.ncbi.nlm.nih.gov/entrez/query.fcgi?cmd=Retrieve&db=Protein&list_uids=118572601&dopt=GenPept&RID=TFNA0JEC01N&log$=prottop&blast_rank=47) | ubinuclein-1 [Homo sapiens] >ref|NP_001072982.1| ubinuclein-1 [Homo sapiens] | [17.6](http://blast.ncbi.nlm.nih.gov/Blast.cgi#118572601%23118572601) | 17.6 | 71% | 608 |
| [NP_005603.2](http://www.ncbi.nlm.nih.gov/entrez/query.fcgi?cmd=Retrieve&db=Protein&list_uids=15559211&dopt=GenPept&RID=TFNA0JEC01N&log$=prottop&blast_rank=48) | RE1-silencing transcription factor [Homo sapiens] | [17.6](http://blast.ncbi.nlm.nih.gov/Blast.cgi#15559211%2315559211) | 30.1 | 85% | 608 |
| [NP_001285.1](http://www.ncbi.nlm.nih.gov/entrez/query.fcgi?cmd=Retrieve&db=Protein&list_uids=4502897&dopt=GenPept&RID=TFNA0JEC01N&log$=prottop&blast_rank=49) | cleft lip and palate transmembrane protein 1 [Homo sapiens] | [17.6](http://blast.ncbi.nlm.nih.gov/Blast.cgi#4502897%234502897) | 31.8 | 85% | 608 |
| [NP_443075.2](http://www.ncbi.nlm.nih.gov/entrez/query.fcgi?cmd=Retrieve&db=Protein&list_uids=58331253&dopt=GenPept&RID=TFNA0JEC01N&log$=prottop&blast_rank=50) | obscurin, cytoskeletal calmodulin and titin-interacting RhoGEF isoform a [Homo sapiens] | [17.6](http://blast.ncbi.nlm.nih.gov/Blast.cgi#58331253%2358331253) | 33.5 | 100% | 608 |
| [NP_060131.2](http://www.ncbi.nlm.nih.gov/entrez/query.fcgi?cmd=Retrieve&db=Protein&list_uids=50811871&dopt=GenPept&RID=TFNA0JEC01N&log$=prottop&blast_rank=51) | suppressor of hairy wing homolog 4 isoform 1 [Homo sapiens] | [17.6](http://blast.ncbi.nlm.nih.gov/Blast.cgi#50811871%2350811871) | 17.6 | 85% | 608 |
| [NP_001010895.1](http://www.ncbi.nlm.nih.gov/entrez/query.fcgi?cmd=Retrieve&db=Protein&list_uids=58219008&dopt=GenPept&RID=TFNA0JEC01N&log$=prottop&blast_rank=52) | RAD26L hypothetical protein [Homo sapiens] | [17.6](http://blast.ncbi.nlm.nih.gov/Blast.cgi#58219008%2358219008) | 17.6 | 71% | 608 |
| [NP_079330.2](http://www.ncbi.nlm.nih.gov/entrez/query.fcgi?cmd=Retrieve&db=Protein&list_uids=36029914&dopt=GenPept&RID=TFNA0JEC01N&log$=prottop&blast_rank=53) | deubiquitinating protein VCIP135 [Homo sapiens] | [17.6](http://blast.ncbi.nlm.nih.gov/Blast.cgi#36029914%2336029914) | 44.8 | 85% | 608 |
| [NP_689838.1](http://www.ncbi.nlm.nih.gov/entrez/query.fcgi?cmd=Retrieve&db=Protein&list_uids=22749279&dopt=GenPept&RID=TFNA0JEC01N&log$=prottop&blast_rank=54) | zinc finger protein 366 [Homo sapiens] | [17.6](http://blast.ncbi.nlm.nih.gov/Blast.cgi#22749279%2322749279) | 17.6 | 71% | 608 |
| [NP_700359.1](http://www.ncbi.nlm.nih.gov/entrez/query.fcgi?cmd=Retrieve&db=Protein&list_uids=23510455&dopt=GenPept&RID=TFNA0JEC01N&log$=prottop&blast_rank=55) | zinc finger protein 41 [Homo sapiens] >ref|NP_009061.1| zinc finger protein 41 [Homo sapiens] | [17.6](http://blast.ncbi.nlm.nih.gov/Blast.cgi#23510455%2323510455) | 17.6 | 85% | 608 |
| [NP_003122.1](http://www.ncbi.nlm.nih.gov/entrez/query.fcgi?cmd=Retrieve&db=Protein&list_uids=4507205&dopt=GenPept&RID=TFNA0JEC01N&log$=prottop&blast_rank=56) | serum response factor [Homo sapiens] | [17.6](http://blast.ncbi.nlm.nih.gov/Blast.cgi#4507205%234507205) | 17.6 | 71% | 608 |
| [NP_061023.1](http://www.ncbi.nlm.nih.gov/entrez/query.fcgi?cmd=Retrieve&db=Protein&list_uids=8923771&dopt=GenPept&RID=TFNA0JEC01N&log$=prottop&blast_rank=57) | ELG protein isoform b [Homo sapiens] | [17.6](http://blast.ncbi.nlm.nih.gov/Blast.cgi#8923771%238923771) | 32.2 | 100% | 608 |
| [NP_003774.1](http://www.ncbi.nlm.nih.gov/entrez/query.fcgi?cmd=Retrieve&db=Protein&list_uids=4502339&dopt=GenPept&RID=TFNA0JEC01N&log$=prottop&blast_rank=58) | beta-1,3-galactosyltransferase 2 [Homo sapiens] | [17.6](http://blast.ncbi.nlm.nih.gov/Blast.cgi#4502339%234502339) | 17.6 | 71% | 608 |
| [NP_066960.1](http://www.ncbi.nlm.nih.gov/entrez/query.fcgi?cmd=Retrieve&db=Protein&list_uids=10863937&dopt=GenPept&RID=TFNA0JEC01N&log$=prottop&blast_rank=59) | BTB/POZ domain-containing protein TNFAIP1 [Homo sapiens] | [17.6](http://blast.ncbi.nlm.nih.gov/Blast.cgi#10863937%2310863937) | 17.6 | 71% | 608 |
| [NP_000433.3](http://www.ncbi.nlm.nih.gov/entrez/query.fcgi?cmd=Retrieve&db=Protein&list_uids=110347451&dopt=GenPept&RID=TFNA0JEC01N&log$=prottop&blast_rank=60) | platelet endothelial cell adhesion molecule precursor [Homo sapiens] | [17.6](http://blast.ncbi.nlm.nih.gov/Blast.cgi#110347451%23110347451) | 29.7 | 85% | 608 |
| [NP_005857.1](http://www.ncbi.nlm.nih.gov/entrez/query.fcgi?cmd=Retrieve&db=Protein&list_uids=5032117&dopt=GenPept&RID=TFNA0JEC01N&log$=prottop&blast_rank=61) | sigma 1-type opioid receptor isoform 1 [Homo sapiens] | [17.6](http://blast.ncbi.nlm.nih.gov/Blast.cgi#5032117%235032117) | 17.6 | 100% | 608 |
| [NP_037452.1](http://www.ncbi.nlm.nih.gov/entrez/query.fcgi?cmd=Retrieve&db=Protein&list_uids=7019405&dopt=GenPept&RID=TFNA0JEC01N&log$=prottop&blast_rank=62) | host cell factor C2 [Homo sapiens] | [17.6](http://blast.ncbi.nlm.nih.gov/Blast.cgi#7019405%237019405) | 17.6 | 71% | 608 |
| [XP_002346314.1](http://www.ncbi.nlm.nih.gov/entrez/query.fcgi?cmd=Retrieve&db=Protein&list_uids=239758151&dopt=GenPept&RID=TFNA0JEC01N&log$=prottop&blast_rank=63) | PREDICTED: hypothetical protein XP_002346314 [Homo sapiens] | [17.2](http://blast.ncbi.nlm.nih.gov/Blast.cgi#239758151%23239758151) | 17.2 | 85% | 816 |
| [XP_002347697.1](http://www.ncbi.nlm.nih.gov/entrez/query.fcgi?cmd=Retrieve&db=Protein&list_uids=239752632&dopt=GenPept&RID=TFNA0JEC01N&log$=prottop&blast_rank=64) | PREDICTED: hypothetical protein XP_002347697 [Homo sapiens] | [17.2](http://blast.ncbi.nlm.nih.gov/Blast.cgi#239752632%23239752632) | 17.2 | 85% | 816 |
| [XP_001718966.2](http://www.ncbi.nlm.nih.gov/entrez/query.fcgi?cmd=Retrieve&db=Protein&list_uids=239741638&dopt=GenPept&RID=TFNA0JEC01N&log$=prottop&blast_rank=65) | PREDICTED: hypothetical protein [Homo sapiens] >ref|XP_001720390.2| PREDICTED: hypothetical protein [Homo sapiens] >ref|XP_001719440.2| PREDICTED: hypothetical protein [Homo sapiens] | [17.2](http://blast.ncbi.nlm.nih.gov/Blast.cgi#239741638%23239741638) | 17.2 | 85% | 816 |
| [NP_066566.3](http://www.ncbi.nlm.nih.gov/entrez/query.fcgi?cmd=Retrieve&db=Protein&list_uids=33350928&dopt=GenPept&RID=TFNA0JEC01N&log$=prottop&blast_rank=66) | disabled homolog 1 [Homo sapiens] | [17.2](http://blast.ncbi.nlm.nih.gov/Blast.cgi#33350928%2333350928) | 17.2 | 100% | 816 |
| [NP_663719.1](http://www.ncbi.nlm.nih.gov/entrez/query.fcgi?cmd=Retrieve&db=Protein&list_uids=22035604&dopt=GenPept&RID=TFNA0JEC01N&log$=prottop&blast_rank=67) | mitogen-activated protein kinase kinase kinase kinase 4 isoform 2 [Homo sapiens] | [17.2](http://blast.ncbi.nlm.nih.gov/Blast.cgi#22035604%2322035604) | 17.2 | 85% | 816 |
| [NP_004825.2](http://www.ncbi.nlm.nih.gov/entrez/query.fcgi?cmd=Retrieve&db=Protein&list_uids=22035602&dopt=GenPept&RID=TFNA0JEC01N&log$=prottop&blast_rank=68) | mitogen-activated protein kinase kinase kinase kinase 4 isoform 1 [Homo sapiens] | [17.2](http://blast.ncbi.nlm.nih.gov/Blast.cgi#22035602%2322035602) | 17.2 | 85% | 816 |
| [NP_663720.1](http://www.ncbi.nlm.nih.gov/entrez/query.fcgi?cmd=Retrieve&db=Protein&list_uids=22035606&dopt=GenPept&RID=TFNA0JEC01N&log$=prottop&blast_rank=69) | mitogen-activated protein kinase kinase kinase kinase 4 isoform 3 [Homo sapiens] | [17.2](http://blast.ncbi.nlm.nih.gov/Blast.cgi#22035606%2322035606) | 17.2 | 85% | 816 |
| [NP_002122.1](http://www.ncbi.nlm.nih.gov/entrez/query.fcgi?cmd=Retrieve&db=Protein&list_uids=4504433&dopt=GenPept&RID=TFNA0JEC01N&log$=prottop&blast_rank=70) | high mobility group protein HMG-I/HMG-Y isoform b [Homo sapiens] >ref|NP_665909.1| high mobility group protein HMG-I/HMG-Y isoform b [Homo sapiens] >ref|NP_665910.1| high mobility group protein HMG-I/HMG-Y isoform b [Homo sapiens] >ref|NP_665912.1| high mobility group protein HMG-I/HMG-Y isoform b [Homo sapiens] | [17.2](http://blast.ncbi.nlm.nih.gov/Blast.cgi#4504433%234504433) | 17.2 | 85% | 816 |
| [NP_055422.1](http://www.ncbi.nlm.nih.gov/entrez/query.fcgi?cmd=Retrieve&db=Protein&list_uids=24307965&dopt=GenPept&RID=TFNA0JEC01N&log$=prottop&blast_rank=71) | UBX domain-containing protein 4 [Homo sapiens] | [17.2](http://blast.ncbi.nlm.nih.gov/Blast.cgi#24307965%2324307965) | 32.2 | 85% | 816 |
| [NP_665906.1](http://www.ncbi.nlm.nih.gov/entrez/query.fcgi?cmd=Retrieve&db=Protein&list_uids=22208967&dopt=GenPept&RID=TFNA0JEC01N&log$=prottop&blast_rank=72) | high mobility group protein HMG-I/HMG-Y isoform a [Homo sapiens] >ref|NP_665908.1| high mobility group protein HMG-I/HMG-Y isoform a [Homo sapiens] | [17.2](http://blast.ncbi.nlm.nih.gov/Blast.cgi#22208967%2322208967) | 17.2 | 85% | 816 |
| [NP_036389.2](http://www.ncbi.nlm.nih.gov/entrez/query.fcgi?cmd=Retrieve&db=Protein&list_uids=21361411&dopt=GenPept&RID=TFNA0JEC01N&log$=prottop&blast_rank=73) | HMG box-containing protein 1 [Homo sapiens] | [17.2](http://blast.ncbi.nlm.nih.gov/Blast.cgi#21361411%2321361411) | 17.2 | 85% | 816 |
| [NP_848644.2](http://www.ncbi.nlm.nih.gov/entrez/query.fcgi?cmd=Retrieve&db=Protein&list_uids=282395053&dopt=GenPept&RID=TFNA0JEC01N&log$=prottop&blast_rank=74) | zinc finger protein 678 [Homo sapiens] | [16.8](http://blast.ncbi.nlm.nih.gov/Blast.cgi#282395053%23282395053) | 16.8 | 71% | 1095 |
| [NP_001160165.1](http://www.ncbi.nlm.nih.gov/entrez/query.fcgi?cmd=Retrieve&db=Protein&list_uids=262359911&dopt=GenPept&RID=TFNA0JEC01N&log$=prottop&blast_rank=75) | AF4/FMR2 family member 1 isoform 1 [Homo sapiens] | [16.8](http://blast.ncbi.nlm.nih.gov/Blast.cgi#262359911%23262359911) | 68.9 | 100% | 1095 |
| [XP_002347871.1](http://www.ncbi.nlm.nih.gov/entrez/query.fcgi?cmd=Retrieve&db=Protein&list_uids=239751461&dopt=GenPept&RID=TFNA0JEC01N&log$=prottop&blast_rank=76) | PREDICTED: hypothetical protein XP_002347871 [Homo sapiens] | [16.8](http://blast.ncbi.nlm.nih.gov/Blast.cgi#239751461%23239751461) | 16.8 | 85% | 1095 |
| [XP_002343836.1](http://www.ncbi.nlm.nih.gov/entrez/query.fcgi?cmd=Retrieve&db=Protein&list_uids=239746759&dopt=GenPept&RID=TFNA0JEC01N&log$=prottop&blast_rank=77) | PREDICTED: hypothetical protein XP_002343836 [Homo sapiens] >ref|XP_002348169.1| PREDICTED: hypothetical protein XP_002348169 [Homo sapiens] | [16.8](http://blast.ncbi.nlm.nih.gov/Blast.cgi#239746759%23239746759) | 16.8 | 71% | 1095 |
| [XP_001715921.2](http://www.ncbi.nlm.nih.gov/entrez/query.fcgi?cmd=Retrieve&db=Protein&list_uids=239743587&dopt=GenPept&RID=TFNA0JEC01N&log$=prottop&blast_rank=78) | PREDICTED: hypothetical protein [Homo sapiens] | [16.8](http://blast.ncbi.nlm.nih.gov/Blast.cgi#239743587%23239743587) | 16.8 | 100% | 1095 |
| [NP_001138600.2](http://www.ncbi.nlm.nih.gov/entrez/query.fcgi?cmd=Retrieve&db=Protein&list_uids=237858799&dopt=GenPept&RID=TFNA0JEC01N&log$=prottop&blast_rank=79) | adenylate kinase domain containing 1 isoform 1 [Homo sapiens] | [16.8](http://blast.ncbi.nlm.nih.gov/Blast.cgi#237858799%23237858799) | 16.8 | 100% | 1095 |
| [NP_001139328.1](http://www.ncbi.nlm.nih.gov/entrez/query.fcgi?cmd=Retrieve&db=Protein&list_uids=224994221&dopt=GenPept&RID=TFNA0JEC01N&log$=prottop&blast_rank=80) | SH3 domain-binding protein 2 isoform b [Homo sapiens] | [16.8](http://blast.ncbi.nlm.nih.gov/Blast.cgi#224994221%23224994221) | 16.8 | 71% | 1095 |
| [NP_115511.3](http://www.ncbi.nlm.nih.gov/entrez/query.fcgi?cmd=Retrieve&db=Protein&list_uids=209977046&dopt=GenPept&RID=TFNA0JEC01N&log$=prottop&blast_rank=81) | fibrous sheath CABYR-binding protein [Homo sapiens] | [16.8](http://blast.ncbi.nlm.nih.gov/Blast.cgi#209977046%23209977046) | 16.8 | 71% | 1095 |
| [NP_056052.2](http://www.ncbi.nlm.nih.gov/entrez/query.fcgi?cmd=Retrieve&db=Protein&list_uids=197245440&dopt=GenPept&RID=TFNA0JEC01N&log$=prottop&blast_rank=82) | hypothetical protein LOC23285 [Homo sapiens] | [16.8](http://blast.ncbi.nlm.nih.gov/Blast.cgi#197245440%23197245440) | 16.8 | 71% | 1095 |
| [NP_001139327.1](http://www.ncbi.nlm.nih.gov/entrez/query.fcgi?cmd=Retrieve&db=Protein&list_uids=224994219&dopt=GenPept&RID=TFNA0JEC01N&log$=prottop&blast_rank=83) | SH3 domain-binding protein 2 isoform c [Homo sapiens] | [16.8](http://blast.ncbi.nlm.nih.gov/Blast.cgi#224994219%23224994219) | 16.8 | 71% | 1095 |
| [NP_001127846.1](http://www.ncbi.nlm.nih.gov/entrez/query.fcgi?cmd=Retrieve&db=Protein&list_uids=197276670&dopt=GenPept&RID=TFNA0JEC01N&log$=prottop&blast_rank=84) | spermatogenesis and centriole associated 1 isoform 2 [Homo sapiens] | [16.8](http://blast.ncbi.nlm.nih.gov/Blast.cgi#197276670%23197276670) | 16.8 | 85% | 1095 |
| [XP_001715866.1](http://www.ncbi.nlm.nih.gov/entrez/query.fcgi?cmd=Retrieve&db=Protein&list_uids=169172540&dopt=GenPept&RID=TFNA0JEC01N&log$=prottop&blast_rank=85) | PREDICTED: hypothetical protein [Homo sapiens] >ref|XP_001715777.1| PREDICTED: hypothetical protein [Homo sapiens] | [16.8](http://blast.ncbi.nlm.nih.gov/Blast.cgi#169172540%23169172540) | 16.8 | 100% | 1095 |
| [NP_619727.2](http://www.ncbi.nlm.nih.gov/entrez/query.fcgi?cmd=Retrieve&db=Protein&list_uids=164419744&dopt=GenPept&RID=TFNA0JEC01N&log$=prottop&blast_rank=86) | nuclear factor of activated T-cells 5 isoform b [Homo sapiens] | [16.8](http://blast.ncbi.nlm.nih.gov/Blast.cgi#164419744%23164419744) | 16.8 | 71% | 1095 |
| [NP_940974.2](http://www.ncbi.nlm.nih.gov/entrez/query.fcgi?cmd=Retrieve&db=Protein&list_uids=197276668&dopt=GenPept&RID=TFNA0JEC01N&log$=prottop&blast_rank=87) | spermatogenesis and centriole associated 1 isoform 1 [Homo sapiens] | [16.8](http://blast.ncbi.nlm.nih.gov/Blast.cgi#197276668%23197276668) | 16.8 | 85% | 1095 |
| [NP_001124171.1](http://www.ncbi.nlm.nih.gov/entrez/query.fcgi?cmd=Retrieve&db=Protein&list_uids=194733747&dopt=GenPept&RID=TFNA0JEC01N&log$=prottop&blast_rank=88) | interactor protein for cytohesin exchange factors 1 isoform 1 [Homo sapiens] >ref|NP_001124172.1| interactor protein for cytohesin exchange factors 1 isoform 1 [Homo sapiens] | [16.8](http://blast.ncbi.nlm.nih.gov/Blast.cgi#194733747%23194733747) | 16.8 | 100% | 1095 |
| [NP_036326.1](http://www.ncbi.nlm.nih.gov/entrez/query.fcgi?cmd=Retrieve&db=Protein&list_uids=149363674&dopt=GenPept&RID=TFNA0JEC01N&log$=prottop&blast_rank=89) | hypothetical protein LOC25758 [Homo sapiens] | [16.8](http://blast.ncbi.nlm.nih.gov/Blast.cgi#149363674%23149363674) | 32.2 | 85% | 1095 |
| [NP_061963.2](http://www.ncbi.nlm.nih.gov/entrez/query.fcgi?cmd=Retrieve&db=Protein&list_uids=149192843&dopt=GenPept&RID=TFNA0JEC01N&log$=prottop&blast_rank=90) | hypothetical protein LOC54627 [Homo sapiens] | [16.8](http://blast.ncbi.nlm.nih.gov/Blast.cgi#149192843%23149192843) | 16.8 | 85% | 1095 |
| [NP_001106649.1](http://www.ncbi.nlm.nih.gov/entrez/query.fcgi?cmd=Retrieve&db=Protein&list_uids=164419746&dopt=GenPept&RID=TFNA0JEC01N&log$=prottop&blast_rank=91) | nuclear factor of activated T-cells 5 isoform d [Homo sapiens] | [16.8](http://blast.ncbi.nlm.nih.gov/Blast.cgi#164419746%23164419746) | 16.8 | 71% | 1095 |
| [NP_055845.1](http://www.ncbi.nlm.nih.gov/entrez/query.fcgi?cmd=Retrieve&db=Protein&list_uids=119874201&dopt=GenPept&RID=TFNA0JEC01N&log$=prottop&blast_rank=92) | protein furry homolog-like [Homo sapiens] | [16.8](http://blast.ncbi.nlm.nih.gov/Blast.cgi#119874201%23119874201) | 16.8 | 71% | 1095 |
| [NP_001120707.1](http://www.ncbi.nlm.nih.gov/entrez/query.fcgi?cmd=Retrieve&db=Protein&list_uids=187829623&dopt=GenPept&RID=TFNA0JEC01N&log$=prottop&blast_rank=93) | GC-rich promoter binding protein 1 isoform 2 [Homo sapiens] | [16.8](http://blast.ncbi.nlm.nih.gov/Blast.cgi#187829623%23187829623) | 27.2 | 100% | 1095 |
| [NP_878918.2](http://www.ncbi.nlm.nih.gov/entrez/query.fcgi?cmd=Retrieve&db=Protein&list_uids=118918407&dopt=GenPept&RID=TFNA0JEC01N&log$=prottop&blast_rank=94) | nesprin-2 isoform 5 [Homo sapiens] | [16.8](http://blast.ncbi.nlm.nih.gov/Blast.cgi#118918407%23118918407) | 67.7 | 100% | 1095 |
| [NP_055995.4](http://www.ncbi.nlm.nih.gov/entrez/query.fcgi?cmd=Retrieve&db=Protein&list_uids=118918403&dopt=GenPept&RID=TFNA0JEC01N&log$=prottop&blast_rank=95) | nesprin-2 isoform 1 [Homo sapiens] | [16.8](http://blast.ncbi.nlm.nih.gov/Blast.cgi#118918403%23118918403) | 56.4 | 100% | 1095 |
| [NP_079279.3](http://www.ncbi.nlm.nih.gov/entrez/query.fcgi?cmd=Retrieve&db=Protein&list_uids=124430557&dopt=GenPept&RID=TFNA0JEC01N&log$=prottop&blast_rank=96) | a disintegrin-like and metalloprotease with thrombospondin type 1 motifs 20 precursor [Homo sapiens] | [16.8](http://blast.ncbi.nlm.nih.gov/Blast.cgi#124430557%23124430557) | 16.8 | 85% | 1095 |
| [NP_002444.2](http://www.ncbi.nlm.nih.gov/entrez/query.fcgi?cmd=Retrieve&db=Protein&list_uids=53729337&dopt=GenPept&RID=TFNA0JEC01N&log$=prottop&blast_rank=97) | mitochondrial translational initiation factor 2 precursor [Homo sapiens] >ref|NP_001005369.1| mitochondrial translational initiation factor 2 precursor [Homo sapiens] | [16.8](http://blast.ncbi.nlm.nih.gov/Blast.cgi#53729337%2353729337) | 16.8 | 71% | 1095 |
| [NP_940867.2](http://www.ncbi.nlm.nih.gov/entrez/query.fcgi?cmd=Retrieve&db=Protein&list_uids=118600963&dopt=GenPept&RID=TFNA0JEC01N&log$=prottop&blast_rank=98) | nik-related protein kinase [Homo sapiens] | [16.8](http://blast.ncbi.nlm.nih.gov/Blast.cgi#118600963%23118600963) | 16.8 | 100% | 1095 |
| [NP_783866.2](http://www.ncbi.nlm.nih.gov/entrez/query.fcgi?cmd=Retrieve&db=Protein&list_uids=110225347&dopt=GenPept&RID=TFNA0JEC01N&log$=prottop&blast_rank=99) | serpin A9 isoform A [Homo sapiens] | [16.8](http://blast.ncbi.nlm.nih.gov/Blast.cgi#110225347%23110225347) | 16.8 | 71% | 1095 |
| [NP_060139.2](http://www.ncbi.nlm.nih.gov/entrez/query.fcgi?cmd=Retrieve&db=Protein&list_uids=58331268&dopt=GenPept&RID=TFNA0JEC01N&log$=prottop&blast_rank=100) | DNA excision repair protein ERCC-6-like [Homo sapiens] | [16.8](http://blast.ncbi.nlm.nih.gov/Blast.cgi#58331268%2358331268) | 31.0 | 71% | 1095 |

| **Accession** | **Proteins with a match to NQHLILSVG petide** | **[Max score](http://blast.ncbi.nlm.nih.gov/Blast.cgi?CMD=Get&ALIGNMENTS=100&ALIGNMENT_VIEW=Pairwise&CDD_SEARCH_STATE=1&DATABASE_SORT=0&DESCRIPTIONS=100&ENTREZ_QUERY=txid9606 %5BORGN%5D&FIRST_QUERY_NUM=0&FORMAT_OBJECT=Alignment&FORMAT_PAGE_TARGET=&FORMAT_TYPE=HTML&GET_SEQUENCE=yes&I_THRESH=&MASK_CHAR=2&MASK_COLOR=1&NEW_VIEW=yes&NUM_OVERVIEW=100&OLD_BLAST=false&PAGE=Proteins&QUERY_INDEX=0&QUERY_NUMBER=0&RESULTS_PAGE_TARGET=&RID=TFNDY55W01N&SHOW_LINKOUT=yes&SHOW_OVERVIEW=yes&STEP_NUMBER=&WORD_SIZE=2&DISPLAY_SORT=1&HSP_SORT=1" \l "sort_mark)** | **[Total score](http://blast.ncbi.nlm.nih.gov/Blast.cgi?CMD=Get&ALIGNMENTS=100&ALIGNMENT_VIEW=Pairwise&CDD_SEARCH_STATE=1&DATABASE_SORT=0&DESCRIPTIONS=100&ENTREZ_QUERY=txid9606 %5BORGN%5D&FIRST_QUERY_NUM=0&FORMAT_OBJECT=Alignment&FORMAT_PAGE_TARGET=&FORMAT_TYPE=HTML&GET_SEQUENCE=yes&I_THRESH=&MASK_CHAR=2&MASK_COLOR=1&NEW_VIEW=yes&NUM_OVERVIEW=100&OLD_BLAST=false&PAGE=Proteins&QUERY_INDEX=0&QUERY_NUMBER=0&RESULTS_PAGE_TARGET=&RID=TFNDY55W01N&SHOW_LINKOUT=yes&SHOW_OVERVIEW=yes&STEP_NUMBER=&WORD_SIZE=2&DISPLAY_SORT=2&HSP_SORT=1" \l "sort_mark)** | **[Query coverage](http://blast.ncbi.nlm.nih.gov/Blast.cgi?CMD=Get&ALIGNMENTS=100&ALIGNMENT_VIEW=Pairwise&CDD_SEARCH_STATE=1&DATABASE_SORT=0&DESCRIPTIONS=100&ENTREZ_QUERY=txid9606 %5BORGN%5D&FIRST_QUERY_NUM=0&FORMAT_OBJECT=Alignment&FORMAT_PAGE_TARGET=&FORMAT_TYPE=HTML&GET_SEQUENCE=yes&I_THRESH=&MASK_CHAR=2&MASK_COLOR=1&NEW_VIEW=yes&NUM_OVERVIEW=100&OLD_BLAST=false&PAGE=Proteins&QUERY_INDEX=0&QUERY_NUMBER=0&RESULTS_PAGE_TARGET=&RID=TFNDY55W01N&SHOW_LINKOUT=yes&SHOW_OVERVIEW=yes&STEP_NUMBER=&WORD_SIZE=2&DISPLAY_SORT=4&HSP_SORT=0" \l "sort_mark)** | **[E value](http://blast.ncbi.nlm.nih.gov/Blast.cgi?CMD=Get&ALIGNMENTS=100&ALIGNMENT_VIEW=Pairwise&CDD_SEARCH_STATE=1&DATABASE_SORT=0&DESCRIPTIONS=100&ENTREZ_QUERY=txid9606 %5BORGN%5D&FIRST_QUERY_NUM=0&FORMAT_OBJECT=Alignment&FORMAT_PAGE_TARGET=&FORMAT_TYPE=HTML&GET_SEQUENCE=yes&I_THRESH=&MASK_CHAR=2&MASK_COLOR=1&NEW_VIEW=yes&NUM_OVERVIEW=100&OLD_BLAST=false&PAGE=Proteins&QUERY_INDEX=0&QUERY_NUMBER=0&RESULTS_PAGE_TARGET=&RID=TFNDY55W01N&SHOW_LINKOUT=yes&SHOW_OVERVIEW=yes&STEP_NUMBER=&WORD_SIZE=2&DISPLAY_SORT=0&HSP_SORT=0" \l "sort_mark)** |
| --- | --- | --- | --- | --- | --- |
| [NP_001164237.1](http://www.ncbi.nlm.nih.gov/entrez/query.fcgi?cmd=Retrieve&db=Protein&list_uids=282847506&dopt=GenPept&RID=TFNDY55W01N&log$=prottop&blast_rank=1) | ligand dependent nuclear receptor corepressor isoform 2 [Homo sapiens] | [22.3](http://blast.ncbi.nlm.nih.gov/Blast.cgi#282847506%23282847506) | 22.3 | 66% | 31 |
| [NP_076941.2](http://www.ncbi.nlm.nih.gov/entrez/query.fcgi?cmd=Retrieve&db=Protein&list_uids=27363458&dopt=GenPept&RID=TFNDY55W01N&log$=prottop&blast_rank=2) | leucine-rich repeat and fibronectin type-III domain-containing protein 4 precursor [Homo sapiens] | [22.3](http://blast.ncbi.nlm.nih.gov/Blast.cgi#27363458%2327363458) | 22.3 | 66% | 31 |
| [NP_001164236.1](http://www.ncbi.nlm.nih.gov/entrez/query.fcgi?cmd=Retrieve&db=Protein&list_uids=282847504&dopt=GenPept&RID=TFNDY55W01N&log$=prottop&blast_rank=3) | ligand dependent nuclear receptor corepressor isoform 1 [Homo sapiens] >ref|NP_115816.2| ligand dependent nuclear receptor corepressor isoform 1 [Homo sapiens] | [22.3](http://blast.ncbi.nlm.nih.gov/Blast.cgi#282847504%23282847504) | 22.3 | 66% | 31 |
| [NP_001092082.1](http://www.ncbi.nlm.nih.gov/entrez/query.fcgi?cmd=Retrieve&db=Protein&list_uids=148762980&dopt=GenPept&RID=TFNDY55W01N&log$=prottop&blast_rank=4) | sialic acid-binding Ig-like lectin 14 precursor [Homo sapiens] | [20.6](http://blast.ncbi.nlm.nih.gov/Blast.cgi#148762980%23148762980) | 20.6 | 77% | 100 |
| [NP_060845.2](http://www.ncbi.nlm.nih.gov/entrez/query.fcgi?cmd=Retrieve&db=Protein&list_uids=40254928&dopt=GenPept&RID=TFNDY55W01N&log$=prottop&blast_rank=5) | zinc transporter ZIP9 [Homo sapiens] | [20.6](http://blast.ncbi.nlm.nih.gov/Blast.cgi#40254928%2340254928) | 20.6 | 66% | 100 |
| [NP_001026.2](http://www.ncbi.nlm.nih.gov/entrez/query.fcgi?cmd=Retrieve&db=Protein&list_uids=112799847&dopt=GenPept&RID=TFNDY55W01N&log$=prottop&blast_rank=6) | cardiac muscle ryanodine receptor [Homo sapiens] | [20.2](http://blast.ncbi.nlm.nih.gov/Blast.cgi#112799847%23112799847) | 36.9 | 55% | 134 |
| [NP_002702.2](http://www.ncbi.nlm.nih.gov/entrez/query.fcgi?cmd=Retrieve&db=Protein&list_uids=167614502&dopt=GenPept&RID=TFNDY55W01N&log$=prottop&blast_rank=7) | protein phosphatase 1 regulatory subunit 3A [Homo sapiens] | [20.2](http://blast.ncbi.nlm.nih.gov/Blast.cgi#167614502%23167614502) | 20.2 | 55% | 134 |
| [NP_001005474.1](http://www.ncbi.nlm.nih.gov/entrez/query.fcgi?cmd=Retrieve&db=Protein&list_uids=53832024&dopt=GenPept&RID=TFNDY55W01N&log$=prottop&blast_rank=8) | NF-kappa-B inhibitor zeta isoform b [Homo sapiens] | [20.2](http://blast.ncbi.nlm.nih.gov/Blast.cgi#53832024%2353832024) | 20.2 | 55% | 134 |
| [NP_113607.1](http://www.ncbi.nlm.nih.gov/entrez/query.fcgi?cmd=Retrieve&db=Protein&list_uids=13899229&dopt=GenPept&RID=TFNDY55W01N&log$=prottop&blast_rank=9) | NF-kappa-B inhibitor zeta isoform a [Homo sapiens] | [20.2](http://blast.ncbi.nlm.nih.gov/Blast.cgi#13899229%2313899229) | 20.2 | 55% | 134 |
| [NP_001026842.2](http://www.ncbi.nlm.nih.gov/entrez/query.fcgi?cmd=Retrieve&db=Protein&list_uids=134133308&dopt=GenPept&RID=TFNDY55W01N&log$=prottop&blast_rank=10) | NADH-cytochrome b5 reductase-like [Homo sapiens] | [19.7](http://blast.ncbi.nlm.nih.gov/Blast.cgi#134133308%23134133308) | 19.7 | 55% | 180 |
| [NP_597676.2](http://www.ncbi.nlm.nih.gov/entrez/query.fcgi?cmd=Retrieve&db=Protein&list_uids=110349713&dopt=GenPept&RID=TFNDY55W01N&log$=prottop&blast_rank=11) | titin isoform novex-1 [Homo sapiens] | [19.7](http://blast.ncbi.nlm.nih.gov/Blast.cgi#110349713%23110349713) | 34.8 | 77% | 180 |
| [NP_006819.2](http://www.ncbi.nlm.nih.gov/entrez/query.fcgi?cmd=Retrieve&db=Protein&list_uids=76880486&dopt=GenPept&RID=TFNDY55W01N&log$=prottop&blast_rank=12) | activating signal cointegrator 1 complex subunit 3 isoform a [Homo sapiens] | [19.7](http://blast.ncbi.nlm.nih.gov/Blast.cgi#76880486%2376880486) | 19.7 | 55% | 180 |
| [NP_054790.2](http://www.ncbi.nlm.nih.gov/entrez/query.fcgi?cmd=Retrieve&db=Protein&list_uids=32307128&dopt=GenPept&RID=TFNDY55W01N&log$=prottop&blast_rank=13) | nuclear receptor coactivator 6 [Homo sapiens] | [19.7](http://blast.ncbi.nlm.nih.gov/Blast.cgi#32307128%2332307128) | 19.7 | 88% | 180 |
| [NP_997274.2](http://www.ncbi.nlm.nih.gov/entrez/query.fcgi?cmd=Retrieve&db=Protein&list_uids=115496700&dopt=GenPept&RID=TFNDY55W01N&log$=prottop&blast_rank=14) | regulator of G-protein signaling 9-binding protein [Homo sapiens] | [19.7](http://blast.ncbi.nlm.nih.gov/Blast.cgi#115496700%23115496700) | 19.7 | 88% | 180 |
| [NP_066363.1](http://www.ncbi.nlm.nih.gov/entrez/query.fcgi?cmd=Retrieve&db=Protein&list_uids=28626521&dopt=GenPept&RID=TFNDY55W01N&log$=prottop&blast_rank=15) | NFX1-type zinc finger-containing protein 1 [Homo sapiens] | [19.7](http://blast.ncbi.nlm.nih.gov/Blast.cgi#28626521%2328626521) | 19.7 | 55% | 180 |
| [XP_002347226.1](http://www.ncbi.nlm.nih.gov/entrez/query.fcgi?cmd=Retrieve&db=Protein&list_uids=239749951&dopt=GenPept&RID=TFNDY55W01N&log$=prottop&blast_rank=16) | PREDICTED: similar to mucin 2 [Homo sapiens] | [19.3](http://blast.ncbi.nlm.nih.gov/Blast.cgi#239749951%23239749951) | 33.5 | 88% | 241 |
| [NP_114157.3](http://www.ncbi.nlm.nih.gov/entrez/query.fcgi?cmd=Retrieve&db=Protein&list_uids=221219020&dopt=GenPept&RID=TFNDY55W01N&log$=prottop&blast_rank=17) | WD repeat-containing protein 87 [Homo sapiens] | [19.3](http://blast.ncbi.nlm.nih.gov/Blast.cgi#221219020%23221219020) | 33.5 | 77% | 241 |
| [NP_001073915.2](http://www.ncbi.nlm.nih.gov/entrez/query.fcgi?cmd=Retrieve&db=Protein&list_uids=148229481&dopt=GenPept&RID=TFNDY55W01N&log$=prottop&blast_rank=18) | hypothetical protein LOC143678 [Homo sapiens] | [19.3](http://blast.ncbi.nlm.nih.gov/Blast.cgi#148229481%23148229481) | 19.3 | 66% | 241 |
| [NP_002448.2](http://www.ncbi.nlm.nih.gov/entrez/query.fcgi?cmd=Retrieve&db=Protein&list_uids=116284392&dopt=GenPept&RID=TFNDY55W01N&log$=prottop&blast_rank=19) | mucin 2 precursor [Homo sapiens] | [19.3](http://blast.ncbi.nlm.nih.gov/Blast.cgi#116284392%23116284392) | 33.5 | 88% | 241 |
| [NP_001121365.1](http://www.ncbi.nlm.nih.gov/entrez/query.fcgi?cmd=Retrieve&db=Protein&list_uids=189217881&dopt=GenPept&RID=TFNDY55W01N&log$=prottop&blast_rank=20) | carcinoembryonic antigen-related cell adhesion molecule 19 isoform 1 [Homo sapiens] | [19.3](http://blast.ncbi.nlm.nih.gov/Blast.cgi#189217881%23189217881) | 19.3 | 88% | 241 |
| [NP_009041.2](http://www.ncbi.nlm.nih.gov/entrez/query.fcgi?cmd=Retrieve&db=Protein&list_uids=21536371&dopt=GenPept&RID=TFNDY55W01N&log$=prottop&blast_rank=21) | telomerase-associated protein 1 [Homo sapiens] | [19.3](http://blast.ncbi.nlm.nih.gov/Blast.cgi#21536371%2321536371) | 19.3 | 66% | 241 |
| [NP_064604.2](http://www.ncbi.nlm.nih.gov/entrez/query.fcgi?cmd=Retrieve&db=Protein&list_uids=45267817&dopt=GenPept&RID=TFNDY55W01N&log$=prottop&blast_rank=22) | carcinoembryonic antigen-related cell adhesion molecule 19 isoform 2 [Homo sapiens] | [19.3](http://blast.ncbi.nlm.nih.gov/Blast.cgi#45267817%2345267817) | 19.3 | 88% | 241 |
| [NP_056273.2](http://www.ncbi.nlm.nih.gov/entrez/query.fcgi?cmd=Retrieve&db=Protein&list_uids=19923424&dopt=GenPept&RID=TFNDY55W01N&log$=prottop&blast_rank=23) | myotubularin-related protein 9 [Homo sapiens] | [19.3](http://blast.ncbi.nlm.nih.gov/Blast.cgi#19923424%2319923424) | 19.3 | 66% | 241 |
| [NP_003746.2](http://www.ncbi.nlm.nih.gov/entrez/query.fcgi?cmd=Retrieve&db=Protein&list_uids=49472822&dopt=GenPept&RID=TFNDY55W01N&log$=prottop&blast_rank=24) | eukaryotic translation initiation factor 3 subunit G [Homo sapiens] | [19.3](http://blast.ncbi.nlm.nih.gov/Blast.cgi#49472822%2349472822) | 19.3 | 66% | 241 |
| [NP_005755.1](http://www.ncbi.nlm.nih.gov/entrez/query.fcgi?cmd=Retrieve&db=Protein&list_uids=5031657&dopt=GenPept&RID=TFNDY55W01N&log$=prottop&blast_rank=25) | PDZK1-interacting protein 1 [Homo sapiens] | [19.3](http://blast.ncbi.nlm.nih.gov/Blast.cgi#5031657%235031657) | 19.3 | 77% | 241 |
| [NP_001152794.1](http://www.ncbi.nlm.nih.gov/entrez/query.fcgi?cmd=Retrieve&db=Protein&list_uids=226693357&dopt=GenPept&RID=TFNDY55W01N&log$=prottop&blast_rank=26) | phospholipase A2, group IVC isoform 2 precursor [Homo sapiens] | [18.9](http://blast.ncbi.nlm.nih.gov/Blast.cgi#226693357%23226693357) | 18.9 | 55% | 324 |
| [NP_001007526.2](http://www.ncbi.nlm.nih.gov/entrez/query.fcgi?cmd=Retrieve&db=Protein&list_uids=169234742&dopt=GenPept&RID=TFNDY55W01N&log$=prottop&blast_rank=27) | NACHT and WD repeat domain-containing protein 1 [Homo sapiens] | [18.9](http://blast.ncbi.nlm.nih.gov/Blast.cgi#169234742%23169234742) | 18.9 | 55% | 324 |
| [XP_001718097.1](http://www.ncbi.nlm.nih.gov/entrez/query.fcgi?cmd=Retrieve&db=Protein&list_uids=169201364&dopt=GenPept&RID=TFNDY55W01N&log$=prottop&blast_rank=28) | PREDICTED: hypothetical protein [Homo sapiens] >ref|XP_001717880.2| PREDICTED: hypothetical protein [Homo sapiens] | [18.9](http://blast.ncbi.nlm.nih.gov/Blast.cgi#169201364%23169201364) | 18.9 | 55% | 324 |
| [NP_057336.3](http://www.ncbi.nlm.nih.gov/entrez/query.fcgi?cmd=Retrieve&db=Protein&list_uids=153792694&dopt=GenPept&RID=TFNDY55W01N&log$=prottop&blast_rank=29) | baculoviral IAP repeat-containing protein 6 [Homo sapiens] | [18.9](http://blast.ncbi.nlm.nih.gov/Blast.cgi#153792694%23153792694) | 35.2 | 66% | 324 |
| [NP_001152795.1](http://www.ncbi.nlm.nih.gov/entrez/query.fcgi?cmd=Retrieve&db=Protein&list_uids=226693363&dopt=GenPept&RID=TFNDY55W01N&log$=prottop&blast_rank=30) | phospholipase A2, group IVC isoform 3 [Homo sapiens] | [18.9](http://blast.ncbi.nlm.nih.gov/Blast.cgi#226693363%23226693363) | 18.9 | 55% | 324 |
| [NP_002510.2](http://www.ncbi.nlm.nih.gov/entrez/query.fcgi?cmd=Retrieve&db=Protein&list_uids=155969714&dopt=GenPept&RID=TFNDY55W01N&log$=prottop&blast_rank=31) | nuclear protein, ataxia-telangiectasia locus [Homo sapiens] | [18.9](http://blast.ncbi.nlm.nih.gov/Blast.cgi#155969714%23155969714) | 33.9 | 100% | 324 |
| [NP_001010875.1](http://www.ncbi.nlm.nih.gov/entrez/query.fcgi?cmd=Retrieve&db=Protein&list_uids=58197562&dopt=GenPept&RID=TFNDY55W01N&log$=prottop&blast_rank=32) | kidney mitochondrial carrier protein 1 [Homo sapiens] | [18.9](http://blast.ncbi.nlm.nih.gov/Blast.cgi#58197562%2358197562) | 18.9 | 55% | 324 |
| [NP_003697.2](http://www.ncbi.nlm.nih.gov/entrez/query.fcgi?cmd=Retrieve&db=Protein&list_uids=226693354&dopt=GenPept&RID=TFNDY55W01N&log$=prottop&blast_rank=33) | phospholipase A2, group IVC isoform 1 precursor [Homo sapiens] | [18.9](http://blast.ncbi.nlm.nih.gov/Blast.cgi#226693354%23226693354) | 18.9 | 55% | 324 |
| [NP_036305.2](http://www.ncbi.nlm.nih.gov/entrez/query.fcgi?cmd=Retrieve&db=Protein&list_uids=34878757&dopt=GenPept&RID=TFNDY55W01N&log$=prottop&blast_rank=34) | F-box only protein 25 isoform 3 [Homo sapiens] | [18.9](http://blast.ncbi.nlm.nih.gov/Blast.cgi#34878757%2334878757) | 29.7 | 77% | 324 |
| [NP_689914.2](http://www.ncbi.nlm.nih.gov/entrez/query.fcgi?cmd=Retrieve&db=Protein&list_uids=31657092&dopt=GenPept&RID=TFNDY55W01N&log$=prottop&blast_rank=35) | ATP-binding cassette sub-family A member 13 [Homo sapiens] | [18.9](http://blast.ncbi.nlm.nih.gov/Blast.cgi#31657092%2331657092) | 18.9 | 55% | 324 |
| [NP_722582.2](http://www.ncbi.nlm.nih.gov/entrez/query.fcgi?cmd=Retrieve&db=Protein&list_uids=61743940&dopt=GenPept&RID=TFNDY55W01N&log$=prottop&blast_rank=36) | probable G-protein coupled receptor 110 isoform 1 [Homo sapiens] | [18.9](http://blast.ncbi.nlm.nih.gov/Blast.cgi#61743940%2361743940) | 33.5 | 88% | 324 |
| [NP_060334.2](http://www.ncbi.nlm.nih.gov/entrez/query.fcgi?cmd=Retrieve&db=Protein&list_uids=70608109&dopt=GenPept&RID=TFNDY55W01N&log$=prottop&blast_rank=37) | integrator complex subunit 8 [Homo sapiens] | [18.9](http://blast.ncbi.nlm.nih.gov/Blast.cgi#70608109%2370608109) | 18.9 | 77% | 324 |
| [NP_904357.1](http://www.ncbi.nlm.nih.gov/entrez/query.fcgi?cmd=Retrieve&db=Protein&list_uids=34878769&dopt=GenPept&RID=TFNDY55W01N&log$=prottop&blast_rank=38) | F-box only protein 25 isoform 1 [Homo sapiens] | [18.9](http://blast.ncbi.nlm.nih.gov/Blast.cgi#34878769%2334878769) | 29.7 | 77% | 324 |
| [NP_005088.1](http://www.ncbi.nlm.nih.gov/entrez/query.fcgi?cmd=Retrieve&db=Protein&list_uids=4826816&dopt=GenPept&RID=TFNDY55W01N&log$=prottop&blast_rank=39) | leucine-rich, glioma inactivated 1 precursor [Homo sapiens] | [18.9](http://blast.ncbi.nlm.nih.gov/Blast.cgi#4826816%234826816) | 18.9 | 55% | 324 |
| [NP_078785.1](http://www.ncbi.nlm.nih.gov/entrez/query.fcgi?cmd=Retrieve&db=Protein&list_uids=13375646&dopt=GenPept&RID=TFNDY55W01N&log$=prottop&blast_rank=40) | leucine-rich repeat and fibronectin type-III domain-containing protein 3 precursor [Homo sapiens] | [18.9](http://blast.ncbi.nlm.nih.gov/Blast.cgi#13375646%2313375646) | 31.4 | 55% | 324 |
| [NP_002664.2](http://www.ncbi.nlm.nih.gov/entrez/query.fcgi?cmd=Retrieve&db=Protein&list_uids=40254442&dopt=GenPept&RID=TFNDY55W01N&log$=prottop&blast_rank=41) | plexin B1 precursor [Homo sapiens] >ref|NP_001123554.1| plexin B1 precursor [Homo sapiens] | [18.9](http://blast.ncbi.nlm.nih.gov/Blast.cgi#40254442%2340254442) | 31.4 | 66% | 324 |
| [NP_003942.1](http://www.ncbi.nlm.nih.gov/entrez/query.fcgi?cmd=Retrieve&db=Protein&list_uids=4507009&dopt=GenPept&RID=TFNDY55W01N&log$=prottop&blast_rank=42) | brain mitochondrial carrier protein 1 isoform UCP5L [Homo sapiens] | [18.9](http://blast.ncbi.nlm.nih.gov/Blast.cgi#4507009%234507009) | 18.9 | 55% | 324 |
| [NP_004455.2](http://www.ncbi.nlm.nih.gov/entrez/query.fcgi?cmd=Retrieve&db=Protein&list_uids=73486655&dopt=GenPept&RID=TFNDY55W01N&log$=prottop&blast_rank=43) | fibroblast growth factor 5 isoform 1 precursor [Homo sapiens] | [18.9](http://blast.ncbi.nlm.nih.gov/Blast.cgi#73486655%2373486655) | 18.9 | 55% | 324 |
| [NP_060646.2](http://www.ncbi.nlm.nih.gov/entrez/query.fcgi?cmd=Retrieve&db=Protein&list_uids=21313638&dopt=GenPept&RID=TFNDY55W01N&log$=prottop&blast_rank=44) | leucine-rich repeat LGI family member 2 precursor [Homo sapiens] | [18.9](http://blast.ncbi.nlm.nih.gov/Blast.cgi#21313638%2321313638) | 18.9 | 55% | 324 |
| [NP_149134.1](http://www.ncbi.nlm.nih.gov/entrez/query.fcgi?cmd=Retrieve&db=Protein&list_uids=15055549&dopt=GenPept&RID=TFNDY55W01N&log$=prottop&blast_rank=45) | fibroblast growth factor 5 isoform 2 precursor [Homo sapiens] | [18.9](http://blast.ncbi.nlm.nih.gov/Blast.cgi#15055549%2315055549) | 18.9 | 55% | 324 |
| [NP_904356.1](http://www.ncbi.nlm.nih.gov/entrez/query.fcgi?cmd=Retrieve&db=Protein&list_uids=34878765&dopt=GenPept&RID=TFNDY55W01N&log$=prottop&blast_rank=46) | F-box only protein 25 isoform 2 [Homo sapiens] | [18.9](http://blast.ncbi.nlm.nih.gov/Blast.cgi#34878765%2334878765) | 29.7 | 77% | 324 |
| [NP_852607.3](http://www.ncbi.nlm.nih.gov/entrez/query.fcgi?cmd=Retrieve&db=Protein&list_uids=198041768&dopt=GenPept&RID=TFNDY55W01N&log$=prottop&blast_rank=47) | leucine-rich repeat-containing protein 70 precursor [Homo sapiens] | [18.9](http://blast.ncbi.nlm.nih.gov/Blast.cgi#198041768%23198041768) | 18.9 | 55% | 324 |
| [NP_073721.1](http://www.ncbi.nlm.nih.gov/entrez/query.fcgi?cmd=Retrieve&db=Protein&list_uids=13259543&dopt=GenPept&RID=TFNDY55W01N&log$=prottop&blast_rank=48) | brain mitochondrial carrier protein 1 isoform UCP5S [Homo sapiens] | [18.9](http://blast.ncbi.nlm.nih.gov/Blast.cgi#13259543%2313259543) | 18.9 | 55% | 324 |
| [NP_570971.1](http://www.ncbi.nlm.nih.gov/entrez/query.fcgi?cmd=Retrieve&db=Protein&list_uids=18702331&dopt=GenPept&RID=TFNDY55W01N&log$=prottop&blast_rank=49) | OTU domain-containing protein 7A [Homo sapiens] | [18.9](http://blast.ncbi.nlm.nih.gov/Blast.cgi#18702331%2318702331) | 18.9 | 55% | 324 |
| [NP_065996.1](http://www.ncbi.nlm.nih.gov/entrez/query.fcgi?cmd=Retrieve&db=Protein&list_uids=191252801&dopt=GenPept&RID=TFNDY55W01N&log$=prottop&blast_rank=50) | protein WDFY4 [Homo sapiens] | [18.5](http://blast.ncbi.nlm.nih.gov/Blast.cgi#191252801%23191252801) | 35.2 | 77% | 434 |
| [NP_077315.2](http://www.ncbi.nlm.nih.gov/entrez/query.fcgi?cmd=Retrieve&db=Protein&list_uids=31543164&dopt=GenPept&RID=TFNDY55W01N&log$=prottop&blast_rank=51) | THO complex subunit 6 homolog isoform 1 [Homo sapiens] | [18.5](http://blast.ncbi.nlm.nih.gov/Blast.cgi#31543164%2331543164) | 18.5 | 88% | 434 |
| [NP_722540.1](http://www.ncbi.nlm.nih.gov/entrez/query.fcgi?cmd=Retrieve&db=Protein&list_uids=24797089&dopt=GenPept&RID=TFNDY55W01N&log$=prottop&blast_rank=52) | peroxisome biogenesis factor 10 isoform 1 [Homo sapiens] | [18.5](http://blast.ncbi.nlm.nih.gov/Blast.cgi#24797089%2324797089) | 18.5 | 77% | 434 |
| [NP_001008485.1](http://www.ncbi.nlm.nih.gov/entrez/query.fcgi?cmd=Retrieve&db=Protein&list_uids=56549673&dopt=GenPept&RID=TFNDY55W01N&log$=prottop&blast_rank=53) | solute carrier family 41 member 3 isoform 1 [Homo sapiens] | [18.5](http://blast.ncbi.nlm.nih.gov/Blast.cgi#56549673%2356549673) | 33.5 | 66% | 434 |
| [NP_001001436.1](http://www.ncbi.nlm.nih.gov/entrez/query.fcgi?cmd=Retrieve&db=Protein&list_uids=47824889&dopt=GenPept&RID=TFNDY55W01N&log$=prottop&blast_rank=54) | hypothetical protein LOC388272 [Homo sapiens] | [18.5](http://blast.ncbi.nlm.nih.gov/Blast.cgi#47824889%2347824889) | 18.5 | 66% | 434 |
| [NP_061897.1](http://www.ncbi.nlm.nih.gov/entrez/query.fcgi?cmd=Retrieve&db=Protein&list_uids=55749742&dopt=GenPept&RID=TFNDY55W01N&log$=prottop&blast_rank=55) | HEAT repeat-containing protein 5B [Homo sapiens] | [18.5](http://blast.ncbi.nlm.nih.gov/Blast.cgi#55749742%2355749742) | 31.8 | 77% | 434 |
| [NP_002608.1](http://www.ncbi.nlm.nih.gov/entrez/query.fcgi?cmd=Retrieve&db=Protein&list_uids=4505715&dopt=GenPept&RID=TFNDY55W01N&log$=prottop&blast_rank=56) | peroxisome biogenesis factor 10 isoform 2 [Homo sapiens] | [18.5](http://blast.ncbi.nlm.nih.gov/Blast.cgi#4505715%234505715) | 18.5 | 77% | 434 |
| [NP_001008486.1](http://www.ncbi.nlm.nih.gov/entrez/query.fcgi?cmd=Retrieve&db=Protein&list_uids=56549675&dopt=GenPept&RID=TFNDY55W01N&log$=prottop&blast_rank=57) | solute carrier family 41 member 3 isoform 3 [Homo sapiens] | [18.5](http://blast.ncbi.nlm.nih.gov/Blast.cgi#56549675%2356549675) | 33.5 | 66% | 434 |
| [NP_055612.2](http://www.ncbi.nlm.nih.gov/entrez/query.fcgi?cmd=Retrieve&db=Protein&list_uids=256600206&dopt=GenPept&RID=TFNDY55W01N&log$=prottop&blast_rank=58) | zinc finger and BTB domain-containing protein 24 isoform 1 [Homo sapiens] | [18.5](http://blast.ncbi.nlm.nih.gov/Blast.cgi#256600206%23256600206) | 18.5 | 66% | 434 |
| [NP_058637.1](http://www.ncbi.nlm.nih.gov/entrez/query.fcgi?cmd=Retrieve&db=Protein&list_uids=8393264&dopt=GenPept&RID=TFNDY55W01N&log$=prottop&blast_rank=59) | delta-like 3 protein isoform 1 precursor [Homo sapiens] | [18.5](http://blast.ncbi.nlm.nih.gov/Blast.cgi#8393264%238393264) | 18.5 | 77% | 434 |
| [NP_003230.1](http://www.ncbi.nlm.nih.gov/entrez/query.fcgi?cmd=Retrieve&db=Protein&list_uids=4507465&dopt=GenPept&RID=TFNDY55W01N&log$=prottop&blast_rank=60) | transforming growth factor, beta 3 precursor [Homo sapiens] | [18.5](http://blast.ncbi.nlm.nih.gov/Blast.cgi#4507465%234507465) | 18.5 | 66% | 434 |
| [XP_002346381.1](http://www.ncbi.nlm.nih.gov/entrez/query.fcgi?cmd=Retrieve&db=Protein&list_uids=239757925&dopt=GenPept&RID=TFNDY55W01N&log$=prottop&blast_rank=61) | PREDICTED: similar to melanoma antigen family C, 2 [Homo sapiens] | [18.0](http://blast.ncbi.nlm.nih.gov/Blast.cgi#239757925%23239757925) | 18.0 | 55% | 583 |
| [XP_002346368.1](http://www.ncbi.nlm.nih.gov/entrez/query.fcgi?cmd=Retrieve&db=Protein&list_uids=239757890&dopt=GenPept&RID=TFNDY55W01N&log$=prottop&blast_rank=62) | PREDICTED: similar to family with sequence similarity 70, member A [Homo sapiens] | [18.0](http://blast.ncbi.nlm.nih.gov/Blast.cgi#239757890%23239757890) | 32.2 | 66% | 583 |
| [XP_001719886.2](http://www.ncbi.nlm.nih.gov/entrez/query.fcgi?cmd=Retrieve&db=Protein&list_uids=239752518&dopt=GenPept&RID=TFNDY55W01N&log$=prottop&blast_rank=63) | PREDICTED: similar to leucine rich repeat containing 50, partial [Homo sapiens] | [18.0](http://blast.ncbi.nlm.nih.gov/Blast.cgi#239752518%23239752518) | 18.0 | 66% | 583 |
| [XP_002343893.1](http://www.ncbi.nlm.nih.gov/entrez/query.fcgi?cmd=Retrieve&db=Protein&list_uids=239746952&dopt=GenPept&RID=TFNDY55W01N&log$=prottop&blast_rank=64) | PREDICTED: similar to melanoma antigen family C, 2 [Homo sapiens] >ref|XP_002348272.1| PREDICTED: similar to melanoma antigen family C, 2 [Homo sapiens] | [18.0](http://blast.ncbi.nlm.nih.gov/Blast.cgi#239746952%23239746952) | 18.0 | 55% | 583 |
| [XP_001716145.2](http://www.ncbi.nlm.nih.gov/entrez/query.fcgi?cmd=Retrieve&db=Protein&list_uids=239744129&dopt=GenPept&RID=TFNDY55W01N&log$=prottop&blast_rank=65) | PREDICTED: hypothetical protein isoform 1 [Homo sapiens] >ref|XP_002343040.1| PREDICTED: hypothetical protein isoform 2 [Homo sapiens] >ref|XP_001716407.2| PREDICTED: hypothetical protein isoform 1 [Homo sapiens] >ref|XP_002347184.1| PREDICTED: hypothetical protein isoform 2 [Homo sapiens] | [18.0](http://blast.ncbi.nlm.nih.gov/Blast.cgi#239744129%23239744129) | 18.0 | 55% | 583 |
| [NP_001153606.1](http://www.ncbi.nlm.nih.gov/entrez/query.fcgi?cmd=Retrieve&db=Protein&list_uids=236462242&dopt=GenPept&RID=TFNDY55W01N&log$=prottop&blast_rank=66) | potassium voltage-gated channel subfamily KQT member 5 isoform 5 [Homo sapiens] | [18.0](http://blast.ncbi.nlm.nih.gov/Blast.cgi#236462242%23236462242) | 18.0 | 55% | 583 |
| [NP_001153604.1](http://www.ncbi.nlm.nih.gov/entrez/query.fcgi?cmd=Retrieve&db=Protein&list_uids=236461942&dopt=GenPept&RID=TFNDY55W01N&log$=prottop&blast_rank=67) | potassium voltage-gated channel subfamily KQT member 5 isoform 3 [Homo sapiens] | [18.0](http://blast.ncbi.nlm.nih.gov/Blast.cgi#236461942%23236461942) | 18.0 | 55% | 583 |
| [NP_001139736.1](http://www.ncbi.nlm.nih.gov/entrez/query.fcgi?cmd=Retrieve&db=Protein&list_uids=226342962&dopt=GenPept&RID=TFNDY55W01N&log$=prottop&blast_rank=68) | synaptotagmin-14 isoform 2 [Homo sapiens] | [18.0](http://blast.ncbi.nlm.nih.gov/Blast.cgi#226342962%23226342962) | 18.0 | 55% | 583 |
| [NP_001139733.1](http://www.ncbi.nlm.nih.gov/entrez/query.fcgi?cmd=Retrieve&db=Protein&list_uids=226342955&dopt=GenPept&RID=TFNDY55W01N&log$=prottop&blast_rank=69) | synaptotagmin-14 isoform 1 [Homo sapiens] | [18.0](http://blast.ncbi.nlm.nih.gov/Blast.cgi#226342955%23226342955) | 18.0 | 55% | 583 |
| [NP_001158137.1](http://www.ncbi.nlm.nih.gov/entrez/query.fcgi?cmd=Retrieve&db=Protein&list_uids=257467654&dopt=GenPept&RID=TFNDY55W01N&log$=prottop&blast_rank=70) | hypothetical protein LOC57670 isoform 2 [Homo sapiens] | [18.0](http://blast.ncbi.nlm.nih.gov/Blast.cgi#257467654%23257467654) | 18.0 | 55% | 583 |
| [NP_065961.2](http://www.ncbi.nlm.nih.gov/entrez/query.fcgi?cmd=Retrieve&db=Protein&list_uids=257467652&dopt=GenPept&RID=TFNDY55W01N&log$=prottop&blast_rank=71) | hypothetical protein LOC57670 isoform 1 [Homo sapiens] | [18.0](http://blast.ncbi.nlm.nih.gov/Blast.cgi#257467652%23257467652) | 18.0 | 55% | 583 |
| [NP_055646.2](http://www.ncbi.nlm.nih.gov/entrez/query.fcgi?cmd=Retrieve&db=Protein&list_uids=257467636&dopt=GenPept&RID=TFNDY55W01N&log$=prottop&blast_rank=72) | lupus brain antigen 1 homolog [Homo sapiens] | [18.0](http://blast.ncbi.nlm.nih.gov/Blast.cgi#257467636%23257467636) | 18.0 | 55% | 583 |
| [NP_001013754.3](http://www.ncbi.nlm.nih.gov/entrez/query.fcgi?cmd=Retrieve&db=Protein&list_uids=257743475&dopt=GenPept&RID=TFNDY55W01N&log$=prottop&blast_rank=73) | patched domain-containing protein C6orf138 [Homo sapiens] | [18.0](http://blast.ncbi.nlm.nih.gov/Blast.cgi#257743475%23257743475) | 18.0 | 55% | 583 |
| [XP_001716567.2](http://www.ncbi.nlm.nih.gov/entrez/query.fcgi?cmd=Retrieve&db=Protein&list_uids=239755328&dopt=GenPept&RID=TFNDY55W01N&log$=prottop&blast_rank=74) | PREDICTED: hypothetical protein isoform 1 [Homo sapiens] >ref|XP_002344531.1| PREDICTED: hypothetical protein isoform 2 [Homo sapiens] | [18.0](http://blast.ncbi.nlm.nih.gov/Blast.cgi#239755328%23239755328) | 18.0 | 55% | 583 |
| [NP_001098015.1](http://www.ncbi.nlm.nih.gov/entrez/query.fcgi?cmd=Retrieve&db=Protein&list_uids=157388935&dopt=GenPept&RID=TFNDY55W01N&log$=prottop&blast_rank=75) | hypothetical protein LOC55026 isoform 3 [Homo sapiens] | [18.0](http://blast.ncbi.nlm.nih.gov/Blast.cgi#157388935%23157388935) | 32.2 | 66% | 583 |
| [NP_001139734.1](http://www.ncbi.nlm.nih.gov/entrez/query.fcgi?cmd=Retrieve&db=Protein&list_uids=226342957&dopt=GenPept&RID=TFNDY55W01N&log$=prottop&blast_rank=76) | synaptotagmin-14 isoform 3 [Homo sapiens] | [18.0](http://blast.ncbi.nlm.nih.gov/Blast.cgi#226342957%23226342957) | 18.0 | 55% | 583 |
| [NP_001093868.1](http://www.ncbi.nlm.nih.gov/entrez/query.fcgi?cmd=Retrieve&db=Protein&list_uids=156086740&dopt=GenPept&RID=TFNDY55W01N&log$=prottop&blast_rank=77) | RAP1 GTPase activating protein 2 isoform 2 [Homo sapiens] | [18.0](http://blast.ncbi.nlm.nih.gov/Blast.cgi#156086740%23156086740) | 18.0 | 55% | 583 |
| [NP_055900.4](http://www.ncbi.nlm.nih.gov/entrez/query.fcgi?cmd=Retrieve&db=Protein&list_uids=156086724&dopt=GenPept&RID=TFNDY55W01N&log$=prottop&blast_rank=78) | RAP1 GTPase activating protein 2 isoform 1 [Homo sapiens] | [18.0](http://blast.ncbi.nlm.nih.gov/Blast.cgi#156086724%23156086724) | 18.0 | 55% | 583 |
| [NP_006662.3](http://www.ncbi.nlm.nih.gov/entrez/query.fcgi?cmd=Retrieve&db=Protein&list_uids=194239697&dopt=GenPept&RID=TFNDY55W01N&log$=prottop&blast_rank=79) | excitatory amino acid transporter 5 [Homo sapiens] | [18.0](http://blast.ncbi.nlm.nih.gov/Blast.cgi#194239697%23194239697) | 18.0 | 55% | 583 |
| [NP_001153605.1](http://www.ncbi.nlm.nih.gov/entrez/query.fcgi?cmd=Retrieve&db=Protein&list_uids=236462079&dopt=GenPept&RID=TFNDY55W01N&log$=prottop&blast_rank=80) | potassium voltage-gated channel subfamily KQT member 5 isoform 4 [Homo sapiens] | [18.0](http://blast.ncbi.nlm.nih.gov/Blast.cgi#236462079%23236462079) | 18.0 | 55% | 583 |
| [NP_004295.2](http://www.ncbi.nlm.nih.gov/entrez/query.fcgi?cmd=Retrieve&db=Protein&list_uids=29029632&dopt=GenPept&RID=TFNDY55W01N&log$=prottop&blast_rank=81) | anaplastic lymphoma kinase precursor [Homo sapiens] | [18.0](http://blast.ncbi.nlm.nih.gov/Blast.cgi#29029632%2329029632) | 30.1 | 66% | 583 |
| [NP_001075956.1](http://www.ncbi.nlm.nih.gov/entrez/query.fcgi?cmd=Retrieve&db=Protein&list_uids=130979214&dopt=GenPept&RID=TFNDY55W01N&log$=prottop&blast_rank=82) | adrenocortical dysplasia protein homolog isoform 3 [Homo sapiens] | [18.0](http://blast.ncbi.nlm.nih.gov/Blast.cgi#130979214%23130979214) | 18.0 | 77% | 583 |
| [NP_001099008.1](http://www.ncbi.nlm.nih.gov/entrez/query.fcgi?cmd=Retrieve&db=Protein&list_uids=157694494&dopt=GenPept&RID=TFNDY55W01N&log$=prottop&blast_rank=83) | myb-binding protein 1A isoform 1 [Homo sapiens] | [18.0](http://blast.ncbi.nlm.nih.gov/Blast.cgi#157694494%23157694494) | 18.0 | 88% | 583 |
| [NP_075065.2](http://www.ncbi.nlm.nih.gov/entrez/query.fcgi?cmd=Retrieve&db=Protein&list_uids=130978962&dopt=GenPept&RID=TFNDY55W01N&log$=prottop&blast_rank=84) | adrenocortical dysplasia protein homolog isoform 2 [Homo sapiens] | [18.0](http://blast.ncbi.nlm.nih.gov/Blast.cgi#130978962%23130978962) | 18.0 | 77% | 583 |
| [NP_001075955.1](http://www.ncbi.nlm.nih.gov/entrez/query.fcgi?cmd=Retrieve&db=Protein&list_uids=130978956&dopt=GenPept&RID=TFNDY55W01N&log$=prottop&blast_rank=85) | adrenocortical dysplasia protein homolog isoform 1 [Homo sapiens] | [18.0](http://blast.ncbi.nlm.nih.gov/Blast.cgi#130978956%23130978956) | 18.0 | 77% | 583 |
| [NP_037521.2](http://www.ncbi.nlm.nih.gov/entrez/query.fcgi?cmd=Retrieve&db=Protein&list_uids=156231351&dopt=GenPept&RID=TFNDY55W01N&log$=prottop&blast_rank=86) | Niemann-Pick C1-like protein 1 isoform 1 precursor [Homo sapiens] | [18.0](http://blast.ncbi.nlm.nih.gov/Blast.cgi#156231351%23156231351) | 46.0 | 77% | 583 |
| [NP_001153602.1](http://www.ncbi.nlm.nih.gov/entrez/query.fcgi?cmd=Retrieve&db=Protein&list_uids=236461719&dopt=GenPept&RID=TFNDY55W01N&log$=prottop&blast_rank=87) | potassium voltage-gated channel subfamily KQT member 5 isoform 2 [Homo sapiens] | [18.0](http://blast.ncbi.nlm.nih.gov/Blast.cgi#236461719%23236461719) | 18.0 | 55% | 583 |
| [NP_057209.3](http://www.ncbi.nlm.nih.gov/entrez/query.fcgi?cmd=Retrieve&db=Protein&list_uids=109134327&dopt=GenPept&RID=TFNDY55W01N&log$=prottop&blast_rank=88) | RING finger and transmembrane domain-containing protein 1 [Homo sapiens] | [18.0](http://blast.ncbi.nlm.nih.gov/Blast.cgi#109134327%23109134327) | 18.0 | 55% | 583 |
| [NP_000092.2](http://www.ncbi.nlm.nih.gov/entrez/query.fcgi?cmd=Retrieve&db=Protein&list_uids=68509914&dopt=GenPept&RID=TFNDY55W01N&log$=prottop&blast_rank=89) | cytochrome b-245 light chain [Homo sapiens] | [18.0](http://blast.ncbi.nlm.nih.gov/Blast.cgi#68509914%2368509914) | 18.0 | 66% | 583 |
| [NP_001078945.1](http://www.ncbi.nlm.nih.gov/entrez/query.fcgi?cmd=Retrieve&db=Protein&list_uids=146262007&dopt=GenPept&RID=TFNDY55W01N&log$=prottop&blast_rank=90) | forkhead box protein D4-like 6 [Homo sapiens] | [18.0](http://blast.ncbi.nlm.nih.gov/Blast.cgi#146262007%23146262007) | 18.0 | 77% | 583 |
| [NP_001098014.1](http://www.ncbi.nlm.nih.gov/entrez/query.fcgi?cmd=Retrieve&db=Protein&list_uids=157388933&dopt=GenPept&RID=TFNDY55W01N&log$=prottop&blast_rank=91) | hypothetical protein LOC55026 isoform 2 [Homo sapiens] | [18.0](http://blast.ncbi.nlm.nih.gov/Blast.cgi#157388933%23157388933) | 32.2 | 66% | 583 |
| [NP_954586.4](http://www.ncbi.nlm.nih.gov/entrez/query.fcgi?cmd=Retrieve&db=Protein&list_uids=157042770&dopt=GenPept&RID=TFNDY55W01N&log$=prottop&blast_rank=92) | forkhead box protein D4-like 2 [Homo sapiens] | [18.0](http://blast.ncbi.nlm.nih.gov/Blast.cgi#157042770%23157042770) | 18.0 | 77% | 583 |
| [NP_954714.2](http://www.ncbi.nlm.nih.gov/entrez/query.fcgi?cmd=Retrieve&db=Protein&list_uids=76880472&dopt=GenPept&RID=TFNDY55W01N&log$=prottop&blast_rank=93) | forkhead box protein D4-like 4 [Homo sapiens] >ref|NP_001092749.1| forkhead box D4-like 2 [Homo sapiens] | [18.0](http://blast.ncbi.nlm.nih.gov/Blast.cgi#76880472%2376880472) | 18.0 | 77% | 583 |
| [NP_694994.2](http://www.ncbi.nlm.nih.gov/entrez/query.fcgi?cmd=Retrieve&db=Protein&list_uids=144226225&dopt=GenPept&RID=TFNDY55W01N&log$=prottop&blast_rank=94) | synaptotagmin-14 isoform 4 [Homo sapiens] | [18.0](http://blast.ncbi.nlm.nih.gov/Blast.cgi#144226225%23144226225) | 18.0 | 55% | 583 |
| [NP_001119806.1](http://www.ncbi.nlm.nih.gov/entrez/query.fcgi?cmd=Retrieve&db=Protein&list_uids=187423902&dopt=GenPept&RID=TFNDY55W01N&log$=prottop&blast_rank=95) | forkhead box protein D4-like 5 [Homo sapiens] | [18.0](http://blast.ncbi.nlm.nih.gov/Blast.cgi#187423902%23187423902) | 18.0 | 77% | 583 |
| [NP_056015.2](http://www.ncbi.nlm.nih.gov/entrez/query.fcgi?cmd=Retrieve&db=Protein&list_uids=155030218&dopt=GenPept&RID=TFNDY55W01N&log$=prottop&blast_rank=96) | sister chromatid cohesion protein PDS5 homolog A isoform 2 [Homo sapiens] | [18.0](http://blast.ncbi.nlm.nih.gov/Blast.cgi#155030218%23155030218) | 18.0 | 66% | 583 |
| [NP_060161.2](http://www.ncbi.nlm.nih.gov/entrez/query.fcgi?cmd=Retrieve&db=Protein&list_uids=55743114&dopt=GenPept&RID=TFNDY55W01N&log$=prottop&blast_rank=97) | leucine-rich repeat-containing protein 49 [Homo sapiens] | [18.0](http://blast.ncbi.nlm.nih.gov/Blast.cgi#55743114%2355743114) | 18.0 | 66% | 583 |
| [NP_060375.4](http://www.ncbi.nlm.nih.gov/entrez/query.fcgi?cmd=Retrieve&db=Protein&list_uids=157388921&dopt=GenPept&RID=TFNDY55W01N&log$=prottop&blast_rank=98) | transmembrane and coiled-coil domain-containing protein 3 precursor [Homo sapiens] | [18.0](http://blast.ncbi.nlm.nih.gov/Blast.cgi#157388921%23157388921) | 18.0 | 55% | 583 |
| [NP_060408.3](http://www.ncbi.nlm.nih.gov/entrez/query.fcgi?cmd=Retrieve&db=Protein&list_uids=157388931&dopt=GenPept&RID=TFNDY55W01N&log$=prottop&blast_rank=99) | hypothetical protein LOC55026 isoform 1 [Homo sapiens] | [18.0](http://blast.ncbi.nlm.nih.gov/Blast.cgi#157388931%23157388931) | 32.2 | 66% | 583 |
| [NP_062816.2](http://www.ncbi.nlm.nih.gov/entrez/query.fcgi?cmd=Retrieve&db=Protein&list_uids=28373065&dopt=GenPept&RID=TFNDY55W01N&log$=prottop&blast_rank=100) | potassium voltage-gated channel subfamily KQT member 5 isoform 1 [Homo sapiens] | [18.0](http://blast.ncbi.nlm.nih.gov/Blast.cgi#28373065%2328373065) | 18.0 | 55% | 583 |

| **Accession** | **Proteins with a match to SIAAAVH petide** | **[Max score](http://blast.ncbi.nlm.nih.gov/Blast.cgi?CMD=Get&ALIGNMENTS=100&ALIGNMENT_VIEW=Pairwise&CDD_SEARCH_STATE=1&DATABASE_SORT=0&DESCRIPTIONS=100&ENTREZ_QUERY=txid9606 %5BORGN%5D&FIRST_QUERY_NUM=0&FORMAT_OBJECT=Alignment&FORMAT_PAGE_TARGET=&FORMAT_TYPE=HTML&GET_SEQUENCE=yes&I_THRESH=&MASK_CHAR=2&MASK_COLOR=1&NEW_VIEW=yes&NUM_OVERVIEW=100&OLD_BLAST=false&PAGE=Proteins&QUERY_INDEX=0&QUERY_NUMBER=0&RESULTS_PAGE_TARGET=&RID=TFNJRBBY01S&SHOW_LINKOUT=yes&SHOW_OVERVIEW=yes&STEP_NUMBER=&WORD_SIZE=2&DISPLAY_SORT=1&HSP_SORT=1" \l "sort_mark)** | **[Total score](http://blast.ncbi.nlm.nih.gov/Blast.cgi?CMD=Get&ALIGNMENTS=100&ALIGNMENT_VIEW=Pairwise&CDD_SEARCH_STATE=1&DATABASE_SORT=0&DESCRIPTIONS=100&ENTREZ_QUERY=txid9606 %5BORGN%5D&FIRST_QUERY_NUM=0&FORMAT_OBJECT=Alignment&FORMAT_PAGE_TARGET=&FORMAT_TYPE=HTML&GET_SEQUENCE=yes&I_THRESH=&MASK_CHAR=2&MASK_COLOR=1&NEW_VIEW=yes&NUM_OVERVIEW=100&OLD_BLAST=false&PAGE=Proteins&QUERY_INDEX=0&QUERY_NUMBER=0&RESULTS_PAGE_TARGET=&RID=TFNJRBBY01S&SHOW_LINKOUT=yes&SHOW_OVERVIEW=yes&STEP_NUMBER=&WORD_SIZE=2&DISPLAY_SORT=2&HSP_SORT=1" \l "sort_mark)** | **[Query coverage](http://blast.ncbi.nlm.nih.gov/Blast.cgi?CMD=Get&ALIGNMENTS=100&ALIGNMENT_VIEW=Pairwise&CDD_SEARCH_STATE=1&DATABASE_SORT=0&DESCRIPTIONS=100&ENTREZ_QUERY=txid9606 %5BORGN%5D&FIRST_QUERY_NUM=0&FORMAT_OBJECT=Alignment&FORMAT_PAGE_TARGET=&FORMAT_TYPE=HTML&GET_SEQUENCE=yes&I_THRESH=&MASK_CHAR=2&MASK_COLOR=1&NEW_VIEW=yes&NUM_OVERVIEW=100&OLD_BLAST=false&PAGE=Proteins&QUERY_INDEX=0&QUERY_NUMBER=0&RESULTS_PAGE_TARGET=&RID=TFNJRBBY01S&SHOW_LINKOUT=yes&SHOW_OVERVIEW=yes&STEP_NUMBER=&WORD_SIZE=2&DISPLAY_SORT=4&HSP_SORT=0" \l "sort_mark)** | **[E value](http://blast.ncbi.nlm.nih.gov/Blast.cgi?CMD=Get&ALIGNMENTS=100&ALIGNMENT_VIEW=Pairwise&CDD_SEARCH_STATE=1&DATABASE_SORT=0&DESCRIPTIONS=100&ENTREZ_QUERY=txid9606 %5BORGN%5D&FIRST_QUERY_NUM=0&FORMAT_OBJECT=Alignment&FORMAT_PAGE_TARGET=&FORMAT_TYPE=HTML&GET_SEQUENCE=yes&I_THRESH=&MASK_CHAR=2&MASK_COLOR=1&NEW_VIEW=yes&NUM_OVERVIEW=100&OLD_BLAST=false&PAGE=Proteins&QUERY_INDEX=0&QUERY_NUMBER=0&RESULTS_PAGE_TARGET=&RID=TFNJRBBY01S&SHOW_LINKOUT=yes&SHOW_OVERVIEW=yes&STEP_NUMBER=&WORD_SIZE=2&DISPLAY_SORT=0&HSP_SORT=0" \l "sort_mark)** |
| --- | --- | --- | --- | --- | --- |
| [NP_001073291.1](http://www.ncbi.nlm.nih.gov/entrez/query.fcgi?cmd=Retrieve&db=Protein&list_uids=119466532&dopt=GenPept&RID=TFNJRBBY01S&log$=prottop&blast_rank=1) | laminin alpha 2 subunit isoform b precursor [Homo sapiens] | [19.7](http://blast.ncbi.nlm.nih.gov/Blast.cgi#119466532%23119466532) | 19.7 | 85% | 140 |
| [NP_877428.1](http://www.ncbi.nlm.nih.gov/entrez/query.fcgi?cmd=Retrieve&db=Protein&list_uids=33383237&dopt=GenPept&RID=TFNJRBBY01S&log$=prottop&blast_rank=2) | protein MCM10 homolog isoform 1 [Homo sapiens] | [19.7](http://blast.ncbi.nlm.nih.gov/Blast.cgi#33383237%2333383237) | 19.7 | 85% | 140 |
| [NP_000417.2](http://www.ncbi.nlm.nih.gov/entrez/query.fcgi?cmd=Retrieve&db=Protein&list_uids=28559088&dopt=GenPept&RID=TFNJRBBY01S&log$=prottop&blast_rank=3) | laminin alpha 2 subunit isoform a precursor [Homo sapiens] | [19.7](http://blast.ncbi.nlm.nih.gov/Blast.cgi#28559088%2328559088) | 19.7 | 85% | 140 |
| [NP_060988.3](http://www.ncbi.nlm.nih.gov/entrez/query.fcgi?cmd=Retrieve&db=Protein&list_uids=33383235&dopt=GenPept&RID=TFNJRBBY01S&log$=prottop&blast_rank=4) | protein MCM10 homolog isoform 2 [Homo sapiens] | [19.7](http://blast.ncbi.nlm.nih.gov/Blast.cgi#33383235%2333383235) | 19.7 | 85% | 140 |
| [XP_001133746.2](http://www.ncbi.nlm.nih.gov/entrez/query.fcgi?cmd=Retrieve&db=Protein&list_uids=239752549&dopt=GenPept&RID=TFNJRBBY01S&log$=prottop&blast_rank=5) | PREDICTED: hypothetical protein [Homo sapiens] | [18.5](http://blast.ncbi.nlm.nih.gov/Blast.cgi#239752549%23239752549) | 18.5 | 85% | 338 |
| [NP_001123553.1](http://www.ncbi.nlm.nih.gov/entrez/query.fcgi?cmd=Retrieve&db=Protein&list_uids=194272176&dopt=GenPept&RID=TFNJRBBY01S&log$=prottop&blast_rank=6) | phospholipase D1 isoform b [Homo sapiens] | [18.5](http://blast.ncbi.nlm.nih.gov/Blast.cgi#194272176%23194272176) | 32.2 | 100% | 338 |
| [NP_002418.1](http://www.ncbi.nlm.nih.gov/entrez/query.fcgi?cmd=Retrieve&db=Protein&list_uids=4505209&dopt=GenPept&RID=TFNJRBBY01S&log$=prottop&blast_rank=7) | matrix metalloproteinase 13 preproprotein [Homo sapiens] | [18.5](http://blast.ncbi.nlm.nih.gov/Blast.cgi#4505209%234505209) | 18.5 | 85% | 338 |
| [NP_002653.1](http://www.ncbi.nlm.nih.gov/entrez/query.fcgi?cmd=Retrieve&db=Protein&list_uids=4505873&dopt=GenPept&RID=TFNJRBBY01S&log$=prottop&blast_rank=8) | phospholipase D1 isoform a [Homo sapiens] | [18.5](http://blast.ncbi.nlm.nih.gov/Blast.cgi#4505873%234505873) | 32.2 | 100% | 338 |
| [NP_001025025.1](http://www.ncbi.nlm.nih.gov/entrez/query.fcgi?cmd=Retrieve&db=Protein&list_uids=71164875&dopt=GenPept&RID=TFNJRBBY01S&log$=prottop&blast_rank=9) | high affinity cAMP-specific and IBMX-insensitive 3',5'-cyclic phosphodiesterase 8B isoform 2 [Homo sapiens] | [18.0](http://blast.ncbi.nlm.nih.gov/Blast.cgi#71164875%2371164875) | 18.0 | 85% | 453 |
| [NP_112185.1](http://www.ncbi.nlm.nih.gov/entrez/query.fcgi?cmd=Retrieve&db=Protein&list_uids=24308309&dopt=GenPept&RID=TFNJRBBY01S&log$=prottop&blast_rank=10) | transmembrane protein 163 [Homo sapiens] | [18.0](http://blast.ncbi.nlm.nih.gov/Blast.cgi#24308309%2324308309) | 18.0 | 100% | 453 |
| [NP_001025024.1](http://www.ncbi.nlm.nih.gov/entrez/query.fcgi?cmd=Retrieve&db=Protein&list_uids=71164873&dopt=GenPept&RID=TFNJRBBY01S&log$=prottop&blast_rank=11) | high affinity cAMP-specific and IBMX-insensitive 3',5'-cyclic phosphodiesterase 8B isoform 4 [Homo sapiens] | [18.0](http://blast.ncbi.nlm.nih.gov/Blast.cgi#71164873%2371164873) | 18.0 | 85% | 453 |
| [NP_003710.1](http://www.ncbi.nlm.nih.gov/entrez/query.fcgi?cmd=Retrieve&db=Protein&list_uids=26006851&dopt=GenPept&RID=TFNJRBBY01S&log$=prottop&blast_rank=12) | high affinity cAMP-specific and IBMX-insensitive 3',5'-cyclic phosphodiesterase 8B isoform 1 [Homo sapiens] | [18.0](http://blast.ncbi.nlm.nih.gov/Blast.cgi#26006851%2326006851) | 18.0 | 85% | 453 |
| [NP_001025023.1](http://www.ncbi.nlm.nih.gov/entrez/query.fcgi?cmd=Retrieve&db=Protein&list_uids=71164869&dopt=GenPept&RID=TFNJRBBY01S&log$=prottop&blast_rank=13) | high affinity cAMP-specific and IBMX-insensitive 3',5'-cyclic phosphodiesterase 8B isoform 5 [Homo sapiens] | [18.0](http://blast.ncbi.nlm.nih.gov/Blast.cgi#71164869%2371164869) | 18.0 | 85% | 453 |
| [NP_001025022.1](http://www.ncbi.nlm.nih.gov/entrez/query.fcgi?cmd=Retrieve&db=Protein&list_uids=71164867&dopt=GenPept&RID=TFNJRBBY01S&log$=prottop&blast_rank=14) | high affinity cAMP-specific and IBMX-insensitive 3',5'-cyclic phosphodiesterase 8B isoform 3 [Homo sapiens] | [18.0](http://blast.ncbi.nlm.nih.gov/Blast.cgi#71164867%2371164867) | 18.0 | 85% | 453 |
| [XP_002344645.1](http://www.ncbi.nlm.nih.gov/entrez/query.fcgi?cmd=Retrieve&db=Protein&list_uids=239755624&dopt=GenPept&RID=TFNJRBBY01S&log$=prottop&blast_rank=15) | PREDICTED: hypothetical protein XP_002344645 [Homo sapiens] | [17.6](http://blast.ncbi.nlm.nih.gov/Blast.cgi#239755624%23239755624) | 17.6 | 71% | 608 |
| [XP_002347282.1](http://www.ncbi.nlm.nih.gov/entrez/query.fcgi?cmd=Retrieve&db=Protein&list_uids=239750127&dopt=GenPept&RID=TFNJRBBY01S&log$=prottop&blast_rank=16) | PREDICTED: hypothetical protein XP_002347282 [Homo sapiens] | [17.6](http://blast.ncbi.nlm.nih.gov/Blast.cgi#239750127%23239750127) | 17.6 | 71% | 608 |
| [XP_002343144.1](http://www.ncbi.nlm.nih.gov/entrez/query.fcgi?cmd=Retrieve&db=Protein&list_uids=239744435&dopt=GenPept&RID=TFNJRBBY01S&log$=prottop&blast_rank=17) | PREDICTED: hypothetical protein XP_002343144 [Homo sapiens] | [17.6](http://blast.ncbi.nlm.nih.gov/Blast.cgi#239744435%23239744435) | 17.6 | 71% | 608 |
| [XP_001717090.2](http://www.ncbi.nlm.nih.gov/entrez/query.fcgi?cmd=Retrieve&db=Protein&list_uids=239742818&dopt=GenPept&RID=TFNJRBBY01S&log$=prottop&blast_rank=18) | PREDICTED: hypothetical protein [Homo sapiens] >ref|XP_001718568.2| PREDICTED: hypothetical protein [Homo sapiens] >ref|XP_001718660.2| PREDICTED: hypothetical protein [Homo sapiens] | [17.6](http://blast.ncbi.nlm.nih.gov/Blast.cgi#239742818%23239742818) | 17.6 | 71% | 608 |
| [XP_002342603.1](http://www.ncbi.nlm.nih.gov/entrez/query.fcgi?cmd=Retrieve&db=Protein&list_uids=239742649&dopt=GenPept&RID=TFNJRBBY01S&log$=prottop&blast_rank=19) | PREDICTED: hypothetical protein XP_002342603 [Homo sapiens] >ref|XP_002346745.1| PREDICTED: hypothetical protein XP_002346745 [Homo sapiens] | [17.6](http://blast.ncbi.nlm.nih.gov/Blast.cgi#239742649%23239742649) | 17.6 | 85% | 608 |
| [NP_001140174.1](http://www.ncbi.nlm.nih.gov/entrez/query.fcgi?cmd=Retrieve&db=Protein&list_uids=226442963&dopt=GenPept&RID=TFNJRBBY01S&log$=prottop&blast_rank=20) | lysine-specific demethylase 5C isoform 2 [Homo sapiens] | [17.6](http://blast.ncbi.nlm.nih.gov/Blast.cgi#226442963%23226442963) | 17.6 | 71% | 608 |
| [NP_001138748.1](http://www.ncbi.nlm.nih.gov/entrez/query.fcgi?cmd=Retrieve&db=Protein&list_uids=223717973&dopt=GenPept&RID=TFNJRBBY01S&log$=prottop&blast_rank=21) | zinc finger protein, Y-linked isoform 3 [Homo sapiens] | [17.6](http://blast.ncbi.nlm.nih.gov/Blast.cgi#223717973%23223717973) | 17.6 | 71% | 608 |
| [XP_001722888.1](http://www.ncbi.nlm.nih.gov/entrez/query.fcgi?cmd=Retrieve&db=Protein&list_uids=169170940&dopt=GenPept&RID=TFNJRBBY01S&log$=prottop&blast_rank=22) | PREDICTED: hypothetical protein [Homo sapiens] >ref|XP_001721215.1| PREDICTED: hypothetical protein [Homo sapiens] >ref|XP_001722943.1| PREDICTED: hypothetical protein [Homo sapiens] >ref|XP_002344334.1| PREDICTED: hypothetical protein [Homo sapiens] | [17.6](http://blast.ncbi.nlm.nih.gov/Blast.cgi#169170940%23169170940) | 17.6 | 71% | 608 |
| [NP_689576.4](http://www.ncbi.nlm.nih.gov/entrez/query.fcgi?cmd=Retrieve&db=Protein&list_uids=168229256&dopt=GenPept&RID=TFNJRBBY01S&log$=prottop&blast_rank=23) | ankyrin repeat and LEM domain-containing protein 1 [Homo sapiens] | [17.6](http://blast.ncbi.nlm.nih.gov/Blast.cgi#168229256%23168229256) | 17.6 | 71% | 608 |
| [NP_612429.2](http://www.ncbi.nlm.nih.gov/entrez/query.fcgi?cmd=Retrieve&db=Protein&list_uids=156766050&dopt=GenPept&RID=TFNJRBBY01S&log$=prottop&blast_rank=24) | AHNAK nucleoprotein 2 [Homo sapiens] | [17.6](http://blast.ncbi.nlm.nih.gov/Blast.cgi#156766050%23156766050) | 17.6 | 100% | 608 |
| [NP_057315.3](http://www.ncbi.nlm.nih.gov/entrez/query.fcgi?cmd=Retrieve&db=Protein&list_uids=149408126&dopt=GenPept&RID=TFNJRBBY01S&log$=prottop&blast_rank=25) | serine/threonine-protein kinase NLK [Homo sapiens] | [17.6](http://blast.ncbi.nlm.nih.gov/Blast.cgi#149408126%23149408126) | 17.6 | 71% | 608 |
| [NP_071401.3](http://www.ncbi.nlm.nih.gov/entrez/query.fcgi?cmd=Retrieve&db=Protein&list_uids=31652264&dopt=GenPept&RID=TFNJRBBY01S&log$=prottop&blast_rank=26) | RNA-binding protein 26 [Homo sapiens] | [17.6](http://blast.ncbi.nlm.nih.gov/Blast.cgi#31652264%2331652264) | 17.6 | 71% | 608 |
| [NP_004178.2](http://www.ncbi.nlm.nih.gov/entrez/query.fcgi?cmd=Retrieve&db=Protein&list_uids=109255243&dopt=GenPept&RID=TFNJRBBY01S&log$=prottop&blast_rank=27) | lysine-specific demethylase 5C isoform 1 [Homo sapiens] | [17.6](http://blast.ncbi.nlm.nih.gov/Blast.cgi#109255243%23109255243) | 17.6 | 71% | 608 |
| [NP_115569.2](http://www.ncbi.nlm.nih.gov/entrez/query.fcgi?cmd=Retrieve&db=Protein&list_uids=38176285&dopt=GenPept&RID=TFNJRBBY01S&log$=prottop&blast_rank=28) | ribonuclease H2 subunit C [Homo sapiens] | [17.6](http://blast.ncbi.nlm.nih.gov/Blast.cgi#38176285%2338176285) | 17.6 | 100% | 608 |
| [NP_115486.1](http://www.ncbi.nlm.nih.gov/entrez/query.fcgi?cmd=Retrieve&db=Protein&list_uids=154146231&dopt=GenPept&RID=TFNJRBBY01S&log$=prottop&blast_rank=29) | DMRT-like family A2 [Homo sapiens] | [17.6](http://blast.ncbi.nlm.nih.gov/Blast.cgi#154146231%23154146231) | 17.6 | 71% | 608 |
| [NP_001161434.1](http://www.ncbi.nlm.nih.gov/entrez/query.fcgi?cmd=Retrieve&db=Protein&list_uids=269784651&dopt=GenPept&RID=TFNJRBBY01S&log$=prottop&blast_rank=30) | prestin isoform e [Homo sapiens] | [17.6](http://blast.ncbi.nlm.nih.gov/Blast.cgi#269784651%23269784651) | 28.0 | 100% | 608 |
| [NP_060509.2](http://www.ncbi.nlm.nih.gov/entrez/query.fcgi?cmd=Retrieve&db=Protein&list_uids=39653317&dopt=GenPept&RID=TFNJRBBY01S&log$=prottop&blast_rank=31) | lysine-specific demethylase 4D [Homo sapiens] | [17.6](http://blast.ncbi.nlm.nih.gov/Blast.cgi#39653317%2339653317) | 17.6 | 71% | 608 |
| [NP_996768.1](http://www.ncbi.nlm.nih.gov/entrez/query.fcgi?cmd=Retrieve&db=Protein&list_uids=45827804&dopt=GenPept&RID=TFNJRBBY01S&log$=prottop&blast_rank=32) | prestin isoform d [Homo sapiens] | [17.6](http://blast.ncbi.nlm.nih.gov/Blast.cgi#45827804%2345827804) | 17.6 | 71% | 608 |
| [NP_996767.1](http://www.ncbi.nlm.nih.gov/entrez/query.fcgi?cmd=Retrieve&db=Protein&list_uids=45827802&dopt=GenPept&RID=TFNJRBBY01S&log$=prottop&blast_rank=33) | prestin isoform c [Homo sapiens] | [17.6](http://blast.ncbi.nlm.nih.gov/Blast.cgi#45827802%2345827802) | 17.6 | 71% | 608 |
| [NP_996766.1](http://www.ncbi.nlm.nih.gov/entrez/query.fcgi?cmd=Retrieve&db=Protein&list_uids=45827800&dopt=GenPept&RID=TFNJRBBY01S&log$=prottop&blast_rank=34) | prestin isoform b [Homo sapiens] | [17.6](http://blast.ncbi.nlm.nih.gov/Blast.cgi#45827800%2345827800) | 17.6 | 71% | 608 |
| [NP_078939.3](http://www.ncbi.nlm.nih.gov/entrez/query.fcgi?cmd=Retrieve&db=Protein&list_uids=47155554&dopt=GenPept&RID=TFNJRBBY01S&log$=prottop&blast_rank=35) | probable aminopeptidase NPEPL1 [Homo sapiens] | [17.6](http://blast.ncbi.nlm.nih.gov/Blast.cgi#47155554%2347155554) | 31.4 | 85% | 608 |
| [NP_787072.2](http://www.ncbi.nlm.nih.gov/entrez/query.fcgi?cmd=Retrieve&db=Protein&list_uids=44921615&dopt=GenPept&RID=TFNJRBBY01S&log$=prottop&blast_rank=36) | exocyst complex 84-kDa subunit [Homo sapiens] | [17.6](http://blast.ncbi.nlm.nih.gov/Blast.cgi#44921615%2344921615) | 17.6 | 71% | 608 |
| [NP_036461.2](http://www.ncbi.nlm.nih.gov/entrez/query.fcgi?cmd=Retrieve&db=Protein&list_uids=52630445&dopt=GenPept&RID=TFNJRBBY01S&log$=prottop&blast_rank=37) | monocyte to macrophage differentiation-associated precursor [Homo sapiens] | [17.6](http://blast.ncbi.nlm.nih.gov/Blast.cgi#52630445%2352630445) | 17.6 | 71% | 608 |
| [NP_909122.1](http://www.ncbi.nlm.nih.gov/entrez/query.fcgi?cmd=Retrieve&db=Protein&list_uids=35493811&dopt=GenPept&RID=TFNJRBBY01S&log$=prottop&blast_rank=38) | RNA-binding protein 39 isoform a [Homo sapiens] | [17.6](http://blast.ncbi.nlm.nih.gov/Blast.cgi#35493811%2335493811) | 17.6 | 85% | 608 |
| [NP_945350.1](http://www.ncbi.nlm.nih.gov/entrez/query.fcgi?cmd=Retrieve&db=Protein&list_uids=39752683&dopt=GenPept&RID=TFNJRBBY01S&log$=prottop&blast_rank=39) | prestin isoform a [Homo sapiens] | [17.6](http://blast.ncbi.nlm.nih.gov/Blast.cgi#39752683%2339752683) | 28.0 | 100% | 608 |
| [NP_071434.2](http://www.ncbi.nlm.nih.gov/entrez/query.fcgi?cmd=Retrieve&db=Protein&list_uids=19923584&dopt=GenPept&RID=TFNJRBBY01S&log$=prottop&blast_rank=40) | modulator of apoptosis 1 [Homo sapiens] | [17.6](http://blast.ncbi.nlm.nih.gov/Blast.cgi#19923584%2319923584) | 17.6 | 85% | 608 |
| [NP_003401.2](http://www.ncbi.nlm.nih.gov/entrez/query.fcgi?cmd=Retrieve&db=Protein&list_uids=71061446&dopt=GenPept&RID=TFNJRBBY01S&log$=prottop&blast_rank=41) | zinc finger X-chromosomal protein [Homo sapiens] | [17.6](http://blast.ncbi.nlm.nih.gov/Blast.cgi#71061446%2371061446) | 17.6 | 71% | 608 |
| [NP_004893.1](http://www.ncbi.nlm.nih.gov/entrez/query.fcgi?cmd=Retrieve&db=Protein&list_uids=4757926&dopt=GenPept&RID=TFNJRBBY01S&log$=prottop&blast_rank=42) | RNA-binding protein 39 isoform b [Homo sapiens] | [17.6](http://blast.ncbi.nlm.nih.gov/Blast.cgi#4757926%234757926) | 17.6 | 85% | 608 |
| [NP_003402.2](http://www.ncbi.nlm.nih.gov/entrez/query.fcgi?cmd=Retrieve&db=Protein&list_uids=33239440&dopt=GenPept&RID=TFNJRBBY01S&log$=prottop&blast_rank=43) | zinc finger protein, Y-linked isoform 1 [Homo sapiens] | [17.6](http://blast.ncbi.nlm.nih.gov/Blast.cgi#33239440%2333239440) | 17.6 | 71% | 608 |
| [NP_000102.1](http://www.ncbi.nlm.nih.gov/entrez/query.fcgi?cmd=Retrieve&db=Protein&list_uids=4557535&dopt=GenPept&RID=TFNJRBBY01S&log$=prottop&blast_rank=44) | chloride anion exchanger [Homo sapiens] | [17.6](http://blast.ncbi.nlm.nih.gov/Blast.cgi#4557535%234557535) | 17.6 | 71% | 608 |
| [XP_002346410.1](http://www.ncbi.nlm.nih.gov/entrez/query.fcgi?cmd=Retrieve&db=Protein&list_uids=239747276&dopt=GenPept&RID=TFNJRBBY01S&log$=prottop&blast_rank=45) | PREDICTED: hypothetical protein XP_002346410 [Homo sapiens] | [17.2](http://blast.ncbi.nlm.nih.gov/Blast.cgi#239747276%23239747276) | 17.2 | 85% | 816 |
| [XP_001718804.1](http://www.ncbi.nlm.nih.gov/entrez/query.fcgi?cmd=Retrieve&db=Protein&list_uids=169214254&dopt=GenPept&RID=TFNJRBBY01S&log$=prottop&blast_rank=46) | PREDICTED: transmembrane protein 221 [Homo sapiens] | [17.2](http://blast.ncbi.nlm.nih.gov/Blast.cgi#169214254%23169214254) | 17.2 | 71% | 816 |
| [XP_001719748.1](http://www.ncbi.nlm.nih.gov/entrez/query.fcgi?cmd=Retrieve&db=Protein&list_uids=169213600&dopt=GenPept&RID=TFNJRBBY01S&log$=prottop&blast_rank=47) | PREDICTED: transmembrane protein 221 [Homo sapiens] | [17.2](http://blast.ncbi.nlm.nih.gov/Blast.cgi#169213600%23169213600) | 17.2 | 71% | 816 |
| [NP_001099047.1](http://www.ncbi.nlm.nih.gov/entrez/query.fcgi?cmd=Retrieve&db=Protein&list_uids=157743280&dopt=GenPept&RID=TFNJRBBY01S&log$=prottop&blast_rank=48) | putative 2-oxo-4-hydroxy-4-carboxy-5-ureidoimidazoline decarboxylase [Homo sapiens] | [17.2](http://blast.ncbi.nlm.nih.gov/Blast.cgi#157743280%23157743280) | 17.2 | 71% | 816 |
| [NP_001073942.1](http://www.ncbi.nlm.nih.gov/entrez/query.fcgi?cmd=Retrieve&db=Protein&list_uids=122937339&dopt=GenPept&RID=TFNJRBBY01S&log$=prottop&blast_rank=49) | major facilitator superfamily domain containing 2B [Homo sapiens] | [17.2](http://blast.ncbi.nlm.nih.gov/Blast.cgi#122937339%23122937339) | 17.2 | 71% | 816 |
| [NP_065681.1](http://www.ncbi.nlm.nih.gov/entrez/query.fcgi?cmd=Retrieve&db=Protein&list_uids=10862701&dopt=GenPept&RID=TFNJRBBY01S&log$=prottop&blast_rank=50) | proto-oncogene tyrosine-protein kinase receptor Ret isoform c [Homo sapiens] | [17.2](http://blast.ncbi.nlm.nih.gov/Blast.cgi#10862701%2310862701) | 17.2 | 71% | 816 |
| [NP_006639.3](http://www.ncbi.nlm.nih.gov/entrez/query.fcgi?cmd=Retrieve&db=Protein&list_uids=32455273&dopt=GenPept&RID=TFNJRBBY01S&log$=prottop&blast_rank=51) | serine/threonine-protein kinase WNK2 [Homo sapiens] | [17.2](http://blast.ncbi.nlm.nih.gov/Blast.cgi#32455273%2332455273) | 28.8 | 85% | 816 |
| [NP_002006.2](http://www.ncbi.nlm.nih.gov/entrez/query.fcgi?cmd=Retrieve&db=Protein&list_uids=9257222&dopt=GenPept&RID=TFNJRBBY01S&log$=prottop&blast_rank=52) | forkhead box protein O1 [Homo sapiens] | [17.2](http://blast.ncbi.nlm.nih.gov/Blast.cgi#9257222%239257222) | 31.0 | 85% | 816 |
| [NP_002664.2](http://www.ncbi.nlm.nih.gov/entrez/query.fcgi?cmd=Retrieve&db=Protein&list_uids=40254442&dopt=GenPept&RID=TFNJRBBY01S&log$=prottop&blast_rank=53) | plexin B1 precursor [Homo sapiens] >ref|NP_001123554.1| plexin B1 precursor [Homo sapiens] | [17.2](http://blast.ncbi.nlm.nih.gov/Blast.cgi#40254442%2340254442) | 31.0 | 71% | 816 |
| [NP_006754.1](http://www.ncbi.nlm.nih.gov/entrez/query.fcgi?cmd=Retrieve&db=Protein&list_uids=5802988&dopt=GenPept&RID=TFNJRBBY01S&log$=prottop&blast_rank=54) | protein BTG2 [Homo sapiens] | [17.2](http://blast.ncbi.nlm.nih.gov/Blast.cgi#5802988%235802988) | 17.2 | 71% | 816 |
| [NP_036392.2](http://www.ncbi.nlm.nih.gov/entrez/query.fcgi?cmd=Retrieve&db=Protein&list_uids=93004078&dopt=GenPept&RID=TFNJRBBY01S&log$=prottop&blast_rank=55) | 2-hydroxyphytanoyl-CoA lyase [Homo sapiens] | [17.2](http://blast.ncbi.nlm.nih.gov/Blast.cgi#93004078%2393004078) | 17.2 | 71% | 816 |
| [NP_077287.1](http://www.ncbi.nlm.nih.gov/entrez/query.fcgi?cmd=Retrieve&db=Protein&list_uids=13236549&dopt=GenPept&RID=TFNJRBBY01S&log$=prottop&blast_rank=56) | UNC93-like protein MFSD11 [Homo sapiens] | [17.2](http://blast.ncbi.nlm.nih.gov/Blast.cgi#13236549%2313236549) | 31.8 | 85% | 816 |
| [NP_001722.1](http://www.ncbi.nlm.nih.gov/entrez/query.fcgi?cmd=Retrieve&db=Protein&list_uids=4502473&dopt=GenPept&RID=TFNJRBBY01S&log$=prottop&blast_rank=57) | protein BTG1 [Homo sapiens] | [17.2](http://blast.ncbi.nlm.nih.gov/Blast.cgi#4502473%234502473) | 17.2 | 71% | 816 |
| [NP_066124.1](http://www.ncbi.nlm.nih.gov/entrez/query.fcgi?cmd=Retrieve&db=Protein&list_uids=10862703&dopt=GenPept&RID=TFNJRBBY01S&log$=prottop&blast_rank=58) | proto-oncogene tyrosine-protein kinase receptor Ret isoform a [Homo sapiens] | [17.2](http://blast.ncbi.nlm.nih.gov/Blast.cgi#10862703%2310862703) | 17.2 | 71% | 816 |
| [NP_543135.1](http://www.ncbi.nlm.nih.gov/entrez/query.fcgi?cmd=Retrieve&db=Protein&list_uids=53850576&dopt=GenPept&RID=TFNJRBBY01S&log$=prottop&blast_rank=59) | olfactory receptor 1K1 [Homo sapiens] | [17.2](http://blast.ncbi.nlm.nih.gov/Blast.cgi#53850576%2353850576) | 17.2 | 71% | 816 |
| [NP_008927.1](http://www.ncbi.nlm.nih.gov/entrez/query.fcgi?cmd=Retrieve&db=Protein&list_uids=27734719&dopt=GenPept&RID=TFNJRBBY01S&log$=prottop&blast_rank=60) | thiamine transporter 1 [Homo sapiens] | [17.2](http://blast.ncbi.nlm.nih.gov/Blast.cgi#27734719%2327734719) | 17.2 | 71% | 816 |
| [NP_001078898.1](http://www.ncbi.nlm.nih.gov/entrez/query.fcgi?cmd=Retrieve&db=Protein&list_uids=146229352&dopt=GenPept&RID=TFNJRBBY01S&log$=prottop&blast_rank=61) | transmembrane protein 213 [Homo sapiens] | [17.2](http://blast.ncbi.nlm.nih.gov/Blast.cgi#146229352%23146229352) | 17.2 | 71% | 816 |
| [NP_001129598.1](http://www.ncbi.nlm.nih.gov/entrez/query.fcgi?cmd=Retrieve&db=Protein&list_uids=209915556&dopt=GenPept&RID=TFNJRBBY01S&log$=prottop&blast_rank=62) | G1/S-specific cyclin-D3 isoform 4 [Homo sapiens] | [16.8](http://blast.ncbi.nlm.nih.gov/Blast.cgi#209915556%23209915556) | 16.8 | 100% | 1095 |
| [NP_001129597.1](http://www.ncbi.nlm.nih.gov/entrez/query.fcgi?cmd=Retrieve&db=Protein&list_uids=209915553&dopt=GenPept&RID=TFNJRBBY01S&log$=prottop&blast_rank=63) | G1/S-specific cyclin-D3 isoform 3 [Homo sapiens] | [16.8](http://blast.ncbi.nlm.nih.gov/Blast.cgi#209915553%23209915553) | 16.8 | 100% | 1095 |
| [NP_001129489.1](http://www.ncbi.nlm.nih.gov/entrez/query.fcgi?cmd=Retrieve&db=Protein&list_uids=209862835&dopt=GenPept&RID=TFNJRBBY01S&log$=prottop&blast_rank=64) | G1/S-specific cyclin-D3 isoform 1 [Homo sapiens] | [16.8](http://blast.ncbi.nlm.nih.gov/Blast.cgi#209862835%23209862835) | 16.8 | 100% | 1095 |
| [NP_055874.2](http://www.ncbi.nlm.nih.gov/entrez/query.fcgi?cmd=Retrieve&db=Protein&list_uids=156938343&dopt=GenPept&RID=TFNJRBBY01S&log$=prottop&blast_rank=65) | talin 2 [Homo sapiens] | [16.8](http://blast.ncbi.nlm.nih.gov/Blast.cgi#156938343%23156938343) | 44.3 | 71% | 1095 |
| [NP_775866.2](http://www.ncbi.nlm.nih.gov/entrez/query.fcgi?cmd=Retrieve&db=Protein&list_uids=157743284&dopt=GenPept&RID=TFNJRBBY01S&log$=prottop&blast_rank=66) | serine/threonine-protein phosphatase 6 regulatory ankyrin repeat subunit C [Homo sapiens] | [16.8](http://blast.ncbi.nlm.nih.gov/Blast.cgi#157743284%23157743284) | 29.7 | 100% | 1095 |
| [XP_001132240.1](http://www.ncbi.nlm.nih.gov/entrez/query.fcgi?cmd=Retrieve&db=Protein&list_uids=113418864&dopt=GenPept&RID=TFNJRBBY01S&log$=prottop&blast_rank=67) | PREDICTED: hypothetical protein [Homo sapiens] >ref|XP_001131545.1| PREDICTED: hypothetical protein [Homo sapiens] >ref|XP_001717386.1| PREDICTED: hypothetical protein [Homo sapiens] >ref|XP_002344439.1| PREDICTED: hypothetical protein [Homo sapiens] | [16.8](http://blast.ncbi.nlm.nih.gov/Blast.cgi#113418864%23113418864) | 16.8 | 71% | 1095 |
| [NP_001070676.1](http://www.ncbi.nlm.nih.gov/entrez/query.fcgi?cmd=Retrieve&db=Protein&list_uids=116256340&dopt=GenPept&RID=TFNJRBBY01S&log$=prottop&blast_rank=68) | protein transport protein Sec31A isoform 3 [Homo sapiens] | [16.8](http://blast.ncbi.nlm.nih.gov/Blast.cgi#116256340%23116256340) | 16.8 | 71% | 1095 |
| [NP_001073922.2](http://www.ncbi.nlm.nih.gov/entrez/query.fcgi?cmd=Retrieve&db=Protein&list_uids=160948599&dopt=GenPept&RID=TFNJRBBY01S&log$=prottop&blast_rank=69) | integrator complex subunit 1 [Homo sapiens] | [16.8](http://blast.ncbi.nlm.nih.gov/Blast.cgi#160948599%23160948599) | 31.0 | 100% | 1095 |
| [NP_001070674.1](http://www.ncbi.nlm.nih.gov/entrez/query.fcgi?cmd=Retrieve&db=Protein&list_uids=116256336&dopt=GenPept&RID=TFNJRBBY01S&log$=prottop&blast_rank=70) | protein transport protein Sec31A isoform 4 [Homo sapiens] | [16.8](http://blast.ncbi.nlm.nih.gov/Blast.cgi#116256336%23116256336) | 16.8 | 71% | 1095 |
| [NP_055676.2](http://www.ncbi.nlm.nih.gov/entrez/query.fcgi?cmd=Retrieve&db=Protein&list_uids=118498343&dopt=GenPept&RID=TFNJRBBY01S&log$=prottop&blast_rank=71) | calcium-transporting ATPase type 2C member 2 [Homo sapiens] | [16.8](http://blast.ncbi.nlm.nih.gov/Blast.cgi#118498343%23118498343) | 16.8 | 85% | 1095 |
| [NP_057295.2](http://www.ncbi.nlm.nih.gov/entrez/query.fcgi?cmd=Retrieve&db=Protein&list_uids=41349441&dopt=GenPept&RID=TFNJRBBY01S&log$=prottop&blast_rank=72) | protein transport protein Sec31A isoform 2 [Homo sapiens] | [16.8](http://blast.ncbi.nlm.nih.gov/Blast.cgi#41349441%2341349441) | 16.8 | 71% | 1095 |
| [NP_068761.4](http://www.ncbi.nlm.nih.gov/entrez/query.fcgi?cmd=Retrieve&db=Protein&list_uids=39995076&dopt=GenPept&RID=TFNJRBBY01S&log$=prottop&blast_rank=73) | hypothetical protein LOC60684 isoform a [Homo sapiens] | [16.8](http://blast.ncbi.nlm.nih.gov/Blast.cgi#39995076%2339995076) | 16.8 | 71% | 1095 |
| [NP_057458.4](http://www.ncbi.nlm.nih.gov/entrez/query.fcgi?cmd=Retrieve&db=Protein&list_uids=22035677&dopt=GenPept&RID=TFNJRBBY01S&log$=prottop&blast_rank=74) | AT-rich interactive domain-containing protein 4B isoform 1 [Homo sapiens] | [16.8](http://blast.ncbi.nlm.nih.gov/Blast.cgi#22035677%2322035677) | 16.8 | 85% | 1095 |
| [NP_002595.1](http://www.ncbi.nlm.nih.gov/entrez/query.fcgi?cmd=Retrieve&db=Protein&list_uids=24429564&dopt=GenPept&RID=TFNJRBBY01S&log$=prottop&blast_rank=75) | high affinity cAMP-specific 3',5'-cyclic phosphodiesterase 7A isoform b [Homo sapiens] | [16.8](http://blast.ncbi.nlm.nih.gov/Blast.cgi#24429564%2324429564) | 16.8 | 85% | 1095 |
| [NP_112739.2](http://www.ncbi.nlm.nih.gov/entrez/query.fcgi?cmd=Retrieve&db=Protein&list_uids=22035679&dopt=GenPept&RID=TFNJRBBY01S&log$=prottop&blast_rank=76) | AT-rich interactive domain-containing protein 4B isoform 2 [Homo sapiens] | [16.8](http://blast.ncbi.nlm.nih.gov/Blast.cgi#22035679%2322035679) | 16.8 | 85% | 1095 |
| [NP_055748.2](http://www.ncbi.nlm.nih.gov/entrez/query.fcgi?cmd=Retrieve&db=Protein&list_uids=41349439&dopt=GenPept&RID=TFNJRBBY01S&log$=prottop&blast_rank=77) | protein transport protein Sec31A isoform 1 [Homo sapiens] >ref|NP_001070675.1| protein transport protein Sec31A isoform 1 [Homo sapiens] | [16.8](http://blast.ncbi.nlm.nih.gov/Blast.cgi#41349439%2341349439) | 16.8 | 71% | 1095 |
| [NP_002594.1](http://www.ncbi.nlm.nih.gov/entrez/query.fcgi?cmd=Retrieve&db=Protein&list_uids=24429566&dopt=GenPept&RID=TFNJRBBY01S&log$=prottop&blast_rank=78) | high affinity cAMP-specific 3',5'-cyclic phosphodiesterase 7A isoform a [Homo sapiens] | [16.8](http://blast.ncbi.nlm.nih.gov/Blast.cgi#24429566%2324429566) | 16.8 | 85% | 1095 |
| [NP_003716.2](http://www.ncbi.nlm.nih.gov/entrez/query.fcgi?cmd=Retrieve&db=Protein&list_uids=19743808&dopt=GenPept&RID=TFNJRBBY01S&log$=prottop&blast_rank=79) | hydroxysteroid (17-beta) dehydrogenase 6 precursor [Homo sapiens] | [16.8](http://blast.ncbi.nlm.nih.gov/Blast.cgi#19743808%2319743808) | 16.8 | 71% | 1095 |
| [NP_570899.1](http://www.ncbi.nlm.nih.gov/entrez/query.fcgi?cmd=Retrieve&db=Protein&list_uids=22027646&dopt=GenPept&RID=TFNJRBBY01S&log$=prottop&blast_rank=80) | angiomotin-like protein 1 [Homo sapiens] | [16.8](http://blast.ncbi.nlm.nih.gov/Blast.cgi#22027646%2322027646) | 16.8 | 71% | 1095 |
| [NP_001751.1](http://www.ncbi.nlm.nih.gov/entrez/query.fcgi?cmd=Retrieve&db=Protein&list_uids=4502619&dopt=GenPept&RID=TFNJRBBY01S&log$=prottop&blast_rank=81) | G1/S-specific cyclin-D3 isoform 2 [Homo sapiens] | [16.8](http://blast.ncbi.nlm.nih.gov/Blast.cgi#4502619%234502619) | 16.8 | 100% | 1095 |
| [NP_006280.3](http://www.ncbi.nlm.nih.gov/entrez/query.fcgi?cmd=Retrieve&db=Protein&list_uids=223029410&dopt=GenPept&RID=TFNJRBBY01S&log$=prottop&blast_rank=82) | talin 1 [Homo sapiens] | [16.8](http://blast.ncbi.nlm.nih.gov/Blast.cgi#223029410%23223029410) | 46.0 | 100% | 1095 |
| [NP_009292.1](http://www.ncbi.nlm.nih.gov/entrez/query.fcgi?cmd=Retrieve&db=Protein&list_uids=6806898&dopt=GenPept&RID=TFNJRBBY01S&log$=prottop&blast_rank=83) | alpha-synuclein isoform NACP112 [Homo sapiens] | [16.8](http://blast.ncbi.nlm.nih.gov/Blast.cgi#6806898%236806898) | 16.8 | 71% | 1095 |
| [NP_005246.2](http://www.ncbi.nlm.nih.gov/entrez/query.fcgi?cmd=Retrieve&db=Protein&list_uids=157384971&dopt=GenPept&RID=TFNJRBBY01S&log$=prottop&blast_rank=84) | cyclin-G-associated kinase [Homo sapiens] | [16.8](http://blast.ncbi.nlm.nih.gov/Blast.cgi#157384971%23157384971) | 31.0 | 100% | 1095 |
| [NP_000336.1](http://www.ncbi.nlm.nih.gov/entrez/query.fcgi?cmd=Retrieve&db=Protein&list_uids=4507109&dopt=GenPept&RID=TFNJRBBY01S&log$=prottop&blast_rank=85) | alpha-synuclein isoform NACP140 [Homo sapiens] >ref|NP_001139526.1| alpha-synuclein isoform NACP140 [Homo sapiens] >ref|NP_001139527.1| alpha-synuclein isoform NACP140 [Homo sapiens] | [16.8](http://blast.ncbi.nlm.nih.gov/Blast.cgi#4507109%234507109) | 16.8 | 71% | 1095 |
| [NP_001158095.1](http://www.ncbi.nlm.nih.gov/entrez/query.fcgi?cmd=Retrieve&db=Protein&list_uids=257467482&dopt=GenPept&RID=TFNJRBBY01S&log$=prottop&blast_rank=86) | protein argonaute-2 isoform 2 [Homo sapiens] | [16.3](http://blast.ncbi.nlm.nih.gov/Blast.cgi#257467482%23257467482) | 16.3 | 85% | 1469 |
| [NP_060113.2](http://www.ncbi.nlm.nih.gov/entrez/query.fcgi?cmd=Retrieve&db=Protein&list_uids=158508476&dopt=GenPept&RID=TFNJRBBY01S&log$=prottop&blast_rank=87) | MBT domain-containing protein 1 [Homo sapiens] | [16.3](http://blast.ncbi.nlm.nih.gov/Blast.cgi#158508476%23158508476) | 16.3 | 85% | 1469 |
| [NP_061852.2](http://www.ncbi.nlm.nih.gov/entrez/query.fcgi?cmd=Retrieve&db=Protein&list_uids=148612809&dopt=GenPept&RID=TFNJRBBY01S&log$=prottop&blast_rank=88) | WNK lysine deficient protein kinase 1 [Homo sapiens] | [16.3](http://blast.ncbi.nlm.nih.gov/Blast.cgi#148612809%23148612809) | 16.3 | 85% | 1469 |
| [NP_031371.3](http://www.ncbi.nlm.nih.gov/entrez/query.fcgi?cmd=Retrieve&db=Protein&list_uids=138175817&dopt=GenPept&RID=TFNJRBBY01S&log$=prottop&blast_rank=89) | zinc finger protein 236 [Homo sapiens] | [16.3](http://blast.ncbi.nlm.nih.gov/Blast.cgi#138175817%23138175817) | 16.3 | 100% | 1469 |
| [NP_001018865.2](http://www.ncbi.nlm.nih.gov/entrez/query.fcgi?cmd=Retrieve&db=Protein&list_uids=170014680&dopt=GenPept&RID=TFNJRBBY01S&log$=prottop&blast_rank=90) | IQ calmodulin-binding motif-containing protein 1 isoform c [Homo sapiens] | [16.3](http://blast.ncbi.nlm.nih.gov/Blast.cgi#170014680%23170014680) | 16.3 | 85% | 1469 |
| [NP_001837.2](http://www.ncbi.nlm.nih.gov/entrez/query.fcgi?cmd=Retrieve&db=Protein&list_uids=116256354&dopt=GenPept&RID=TFNJRBBY01S&log$=prottop&blast_rank=91) | alpha 2 type IV collagen preproprotein [Homo sapiens] | [16.3](http://blast.ncbi.nlm.nih.gov/Blast.cgi#116256354%23116256354) | 16.3 | 85% | 1469 |
| [NP_060269.3](http://www.ncbi.nlm.nih.gov/entrez/query.fcgi?cmd=Retrieve&db=Protein&list_uids=93277080&dopt=GenPept&RID=TFNJRBBY01S&log$=prottop&blast_rank=92) | hypothetical protein LOC54916 [Homo sapiens] | [16.3](http://blast.ncbi.nlm.nih.gov/Blast.cgi#93277080%2393277080) | 16.3 | 85% | 1469 |
| [NP_112730.2](http://www.ncbi.nlm.nih.gov/entrez/query.fcgi?cmd=Retrieve&db=Protein&list_uids=89142733&dopt=GenPept&RID=TFNJRBBY01S&log$=prottop&blast_rank=93) | alpha 3 type IV collagen isoform 2 precursor [Homo sapiens] | [16.3](http://blast.ncbi.nlm.nih.gov/Blast.cgi#89142733%2389142733) | 16.3 | 85% | 1469 |
| [NP_958831.1](http://www.ncbi.nlm.nih.gov/entrez/query.fcgi?cmd=Retrieve&db=Protein&list_uids=41393608&dopt=GenPept&RID=TFNJRBBY01S&log$=prottop&blast_rank=94) | reticulon-3 isoform b [Homo sapiens] | [16.3](http://blast.ncbi.nlm.nih.gov/Blast.cgi#41393608%2341393608) | 43.1 | 100% | 1469 |
| [NP_803171.1](http://www.ncbi.nlm.nih.gov/entrez/query.fcgi?cmd=Retrieve&db=Protein&list_uids=29337286&dopt=GenPept&RID=TFNJRBBY01S&log$=prottop&blast_rank=95) | protein argonaute-3 isoform b [Homo sapiens] | [16.3](http://blast.ncbi.nlm.nih.gov/Blast.cgi#29337286%2329337286) | 16.3 | 85% | 1469 |
| [NP_777616.1](http://www.ncbi.nlm.nih.gov/entrez/query.fcgi?cmd=Retrieve&db=Protein&list_uids=28373111&dopt=GenPept&RID=TFNJRBBY01S&log$=prottop&blast_rank=96) | ATPase, Ca++ transporting, ubiquitous isoform c [Homo sapiens] >ref|NP_777618.1| ATPase, Ca++ transporting, ubiquitous isoform c [Homo sapiens] | [16.3](http://blast.ncbi.nlm.nih.gov/Blast.cgi#28373111%2328373111) | 16.3 | 85% | 1469 |
| [NP_005164.2](http://www.ncbi.nlm.nih.gov/entrez/query.fcgi?cmd=Retrieve&db=Protein&list_uids=28373103&dopt=GenPept&RID=TFNJRBBY01S&log$=prottop&blast_rank=97) | ATPase, Ca++ transporting, ubiquitous isoform a [Homo sapiens] | [16.3](http://blast.ncbi.nlm.nih.gov/Blast.cgi#28373103%2328373103) | 16.3 | 85% | 1469 |
| [NP_000082.2](http://www.ncbi.nlm.nih.gov/entrez/query.fcgi?cmd=Retrieve&db=Protein&list_uids=89142730&dopt=GenPept&RID=TFNJRBBY01S&log$=prottop&blast_rank=98) | alpha 3 type IV collagen isoform 1 precursor [Homo sapiens] | [16.3](http://blast.ncbi.nlm.nih.gov/Blast.cgi#89142730%2389142730) | 16.3 | 85% | 1469 |
| [NP_777613.1](http://www.ncbi.nlm.nih.gov/entrez/query.fcgi?cmd=Retrieve&db=Protein&list_uids=28373105&dopt=GenPept&RID=TFNJRBBY01S&log$=prottop&blast_rank=99) | ATPase, Ca++ transporting, ubiquitous isoform e [Homo sapiens] | [16.3](http://blast.ncbi.nlm.nih.gov/Blast.cgi#28373105%2328373105) | 16.3 | 85% | 1469 |
| [NP_777617.1](http://www.ncbi.nlm.nih.gov/entrez/query.fcgi?cmd=Retrieve&db=Protein&list_uids=28373113&dopt=GenPept&RID=TFNJRBBY01S&log$=prottop&blast_rank=100) | ATPase, Ca++ transporting, ubiquitous isoform f [Homo sapiens] | [16.3](http://blast.ncbi.nlm.nih.gov/Blast.cgi#28373113%2328373113) | 16.3 | 85% | 1469 |

| **Accession** | **Proteins with a match to RANKEPAT peptide** | **[Max score](http://blast.ncbi.nlm.nih.gov/Blast.cgi?CMD=Get&ALIGNMENTS=100&ALIGNMENT_VIEW=Pairwise&CDD_SEARCH_STATE=1&DATABASE_SORT=0&DESCRIPTIONS=100&ENTREZ_QUERY=txid9606 %5BORGN%5D&FIRST_QUERY_NUM=0&FORMAT_OBJECT=Alignment&FORMAT_PAGE_TARGET=&FORMAT_TYPE=HTML&GET_SEQUENCE=yes&I_THRESH=&MASK_CHAR=2&MASK_COLOR=1&NEW_VIEW=yes&NUM_OVERVIEW=100&OLD_BLAST=false&PAGE=Proteins&QUERY_INDEX=0&QUERY_NUMBER=0&RESULTS_PAGE_TARGET=&RID=TFNP80JC01N&SHOW_LINKOUT=yes&SHOW_OVERVIEW=yes&STEP_NUMBER=&WORD_SIZE=2&DISPLAY_SORT=1&HSP_SORT=1" \l "sort_mark)** | **[Total score](http://blast.ncbi.nlm.nih.gov/Blast.cgi?CMD=Get&ALIGNMENTS=100&ALIGNMENT_VIEW=Pairwise&CDD_SEARCH_STATE=1&DATABASE_SORT=0&DESCRIPTIONS=100&ENTREZ_QUERY=txid9606 %5BORGN%5D&FIRST_QUERY_NUM=0&FORMAT_OBJECT=Alignment&FORMAT_PAGE_TARGET=&FORMAT_TYPE=HTML&GET_SEQUENCE=yes&I_THRESH=&MASK_CHAR=2&MASK_COLOR=1&NEW_VIEW=yes&NUM_OVERVIEW=100&OLD_BLAST=false&PAGE=Proteins&QUERY_INDEX=0&QUERY_NUMBER=0&RESULTS_PAGE_TARGET=&RID=TFNP80JC01N&SHOW_LINKOUT=yes&SHOW_OVERVIEW=yes&STEP_NUMBER=&WORD_SIZE=2&DISPLAY_SORT=2&HSP_SORT=1" \l "sort_mark)** | **[Query coverage](http://blast.ncbi.nlm.nih.gov/Blast.cgi?CMD=Get&ALIGNMENTS=100&ALIGNMENT_VIEW=Pairwise&CDD_SEARCH_STATE=1&DATABASE_SORT=0&DESCRIPTIONS=100&ENTREZ_QUERY=txid9606 %5BORGN%5D&FIRST_QUERY_NUM=0&FORMAT_OBJECT=Alignment&FORMAT_PAGE_TARGET=&FORMAT_TYPE=HTML&GET_SEQUENCE=yes&I_THRESH=&MASK_CHAR=2&MASK_COLOR=1&NEW_VIEW=yes&NUM_OVERVIEW=100&OLD_BLAST=false&PAGE=Proteins&QUERY_INDEX=0&QUERY_NUMBER=0&RESULTS_PAGE_TARGET=&RID=TFNP80JC01N&SHOW_LINKOUT=yes&SHOW_OVERVIEW=yes&STEP_NUMBER=&WORD_SIZE=2&DISPLAY_SORT=4&HSP_SORT=0" \l "sort_mark)** | **[E value](http://blast.ncbi.nlm.nih.gov/Blast.cgi?CMD=Get&ALIGNMENTS=100&ALIGNMENT_VIEW=Pairwise&CDD_SEARCH_STATE=1&DATABASE_SORT=0&DESCRIPTIONS=100&ENTREZ_QUERY=txid9606 %5BORGN%5D&FIRST_QUERY_NUM=0&FORMAT_OBJECT=Alignment&FORMAT_PAGE_TARGET=&FORMAT_TYPE=HTML&GET_SEQUENCE=yes&I_THRESH=&MASK_CHAR=2&MASK_COLOR=1&NEW_VIEW=yes&NUM_OVERVIEW=100&OLD_BLAST=false&PAGE=Proteins&QUERY_INDEX=0&QUERY_NUMBER=0&RESULTS_PAGE_TARGET=&RID=TFNP80JC01N&SHOW_LINKOUT=yes&SHOW_OVERVIEW=yes&STEP_NUMBER=&WORD_SIZE=2&DISPLAY_SORT=0&HSP_SORT=0" \l "sort_mark)** |
| --- | --- | --- | --- | --- | --- |
| [NP_596869.3](http://www.ncbi.nlm.nih.gov/entrez/query.fcgi?cmd=Retrieve&db=Protein&list_uids=110349719&dopt=GenPept&RID=TFNP80JC01N&log$=prottop&blast_rank=1) | titin isoform N2-A [Homo sapiens] | [19.3](http://blast.ncbi.nlm.nih.gov/Blast.cgi#110349719%23110349719) | 181 | 100% | 214 |
| [NP_001155056.1](http://www.ncbi.nlm.nih.gov/entrez/query.fcgi?cmd=Retrieve&db=Protein&list_uids=239787844&dopt=GenPept&RID=TFNP80JC01N&log$=prottop&blast_rank=2) | testis-expressed sequence 10 protein isoform 2 [Homo sapiens] | [18.9](http://blast.ncbi.nlm.nih.gov/Blast.cgi#239787844%23239787844) | 18.9 | 62% | 288 |
| [NP_001106849.1](http://www.ncbi.nlm.nih.gov/entrez/query.fcgi?cmd=Retrieve&db=Protein&list_uids=164607124&dopt=GenPept&RID=TFNP80JC01N&log$=prottop&blast_rank=3) | fanconi anemia group I protein isoform 1 [Homo sapiens] | [18.9](http://blast.ncbi.nlm.nih.gov/Blast.cgi#164607124%23164607124) | 18.9 | 62% | 288 |
| [NP_004738.3](http://www.ncbi.nlm.nih.gov/entrez/query.fcgi?cmd=Retrieve&db=Protein&list_uids=95089461&dopt=GenPept&RID=TFNP80JC01N&log$=prottop&blast_rank=4) | disks large homolog 5 [Homo sapiens] | [18.9](http://blast.ncbi.nlm.nih.gov/Blast.cgi#95089461%2395089461) | 18.9 | 62% | 288 |
| [NP_001139438.1](http://www.ncbi.nlm.nih.gov/entrez/query.fcgi?cmd=Retrieve&db=Protein&list_uids=225543215&dopt=GenPept&RID=TFNP80JC01N&log$=prottop&blast_rank=5) | antigen KI-67 isoform 2 [Homo sapiens] | [18.9](http://blast.ncbi.nlm.nih.gov/Blast.cgi#225543215%23225543215) | 60.2 | 87% | 288 |
| [NP_002408.3](http://www.ncbi.nlm.nih.gov/entrez/query.fcgi?cmd=Retrieve&db=Protein&list_uids=103472005&dopt=GenPept&RID=TFNP80JC01N&log$=prottop&blast_rank=6) | antigen KI-67 isoform 1 [Homo sapiens] | [18.9](http://blast.ncbi.nlm.nih.gov/Blast.cgi#103472005%23103472005) | 60.2 | 87% | 288 |
| [NP_060663.2](http://www.ncbi.nlm.nih.gov/entrez/query.fcgi?cmd=Retrieve&db=Protein&list_uids=82830440&dopt=GenPept&RID=TFNP80JC01N&log$=prottop&blast_rank=7) | fanconi anemia group I protein isoform 2 [Homo sapiens] | [18.9](http://blast.ncbi.nlm.nih.gov/Blast.cgi#82830440%2382830440) | 18.9 | 62% | 288 |
| [NP_060868.2](http://www.ncbi.nlm.nih.gov/entrez/query.fcgi?cmd=Retrieve&db=Protein&list_uids=54112397&dopt=GenPept&RID=TFNP80JC01N&log$=prottop&blast_rank=8) | voltage-dependent calcium channel subunit alpha-2/delta-3 [Homo sapiens] | [18.9](http://blast.ncbi.nlm.nih.gov/Blast.cgi#54112397%2354112397) | 35.2 | 87% | 288 |
| [NP_001019782.1](http://www.ncbi.nlm.nih.gov/entrez/query.fcgi?cmd=Retrieve&db=Protein&list_uids=66912176&dopt=GenPept&RID=TFNP80JC01N&log$=prottop&blast_rank=9) | leucine-rich repeat-containing protein 66 [Homo sapiens] | [18.9](http://blast.ncbi.nlm.nih.gov/Blast.cgi#66912176%2366912176) | 18.9 | 62% | 288 |
| [NP_060216.2](http://www.ncbi.nlm.nih.gov/entrez/query.fcgi?cmd=Retrieve&db=Protein&list_uids=239787838&dopt=GenPept&RID=TFNP80JC01N&log$=prottop&blast_rank=10) | testis-expressed sequence 10 protein isoform 1 [Homo sapiens] | [18.9](http://blast.ncbi.nlm.nih.gov/Blast.cgi#239787838%23239787838) | 18.9 | 62% | 288 |
| [NP_003795.2](http://www.ncbi.nlm.nih.gov/entrez/query.fcgi?cmd=Retrieve&db=Protein&list_uids=57242761&dopt=GenPept&RID=TFNP80JC01N&log$=prottop&blast_rank=11) | receptor-interacting serine/threonine-protein kinase 1 [Homo sapiens] | [18.9](http://blast.ncbi.nlm.nih.gov/Blast.cgi#57242761%2357242761) | 18.9 | 62% | 288 |
| [NP_006384.1](http://www.ncbi.nlm.nih.gov/entrez/query.fcgi?cmd=Retrieve&db=Protein&list_uids=5453758&dopt=GenPept&RID=TFNP80JC01N&log$=prottop&blast_rank=12) | nebulette sarcomeric isoform [Homo sapiens] | [18.9](http://blast.ncbi.nlm.nih.gov/Blast.cgi#5453758%235453758) | 18.9 | 62% | 288 |
| [NP_078861.1](http://www.ncbi.nlm.nih.gov/entrez/query.fcgi?cmd=Retrieve&db=Protein&list_uids=13375770&dopt=GenPept&RID=TFNP80JC01N&log$=prottop&blast_rank=13) | armadillo repeat-containing protein 7 [Homo sapiens] | [18.9](http://blast.ncbi.nlm.nih.gov/Blast.cgi#13375770%2313375770) | 18.9 | 62% | 288 |
| [NP_001158136.1](http://www.ncbi.nlm.nih.gov/entrez/query.fcgi?cmd=Retrieve&db=Protein&list_uids=257467648&dopt=GenPept&RID=TFNP80JC01N&log$=prottop&blast_rank=14) | microtubule-associated serine/threonine-protein kinase 4 isoform c [Homo sapiens] | [18.5](http://blast.ncbi.nlm.nih.gov/Blast.cgi#257467648%23257467648) | 18.5 | 62% | 386 |
| [NP_009231.2](http://www.ncbi.nlm.nih.gov/entrez/query.fcgi?cmd=Retrieve&db=Protein&list_uids=237681119&dopt=GenPept&RID=TFNP80JC01N&log$=prottop&blast_rank=15) | breast cancer type 1 susceptibility protein isoform 2 [Homo sapiens] | [18.5](http://blast.ncbi.nlm.nih.gov/Blast.cgi#237681119%23237681119) | 34.8 | 75% | 386 |
| [NP_009228.2](http://www.ncbi.nlm.nih.gov/entrez/query.fcgi?cmd=Retrieve&db=Protein&list_uids=237681121&dopt=GenPept&RID=TFNP80JC01N&log$=prottop&blast_rank=16) | breast cancer type 1 susceptibility protein isoform 3 [Homo sapiens] | [18.5](http://blast.ncbi.nlm.nih.gov/Blast.cgi#237681121%23237681121) | 34.8 | 75% | 386 |
| [NP_001094889.1](http://www.ncbi.nlm.nih.gov/entrez/query.fcgi?cmd=Retrieve&db=Protein&list_uids=210031154&dopt=GenPept&RID=TFNP80JC01N&log$=prottop&blast_rank=17) | zinc finger protein 541 [Homo sapiens] | [18.5](http://blast.ncbi.nlm.nih.gov/Blast.cgi#210031154%23210031154) | 18.5 | 62% | 386 |
| [NP_055998.1](http://www.ncbi.nlm.nih.gov/entrez/query.fcgi?cmd=Retrieve&db=Protein&list_uids=148727255&dopt=GenPept&RID=TFNP80JC01N&log$=prottop&blast_rank=18) | microtubule-associated serine/threonine-protein kinase 4 isoform a [Homo sapiens] | [18.5](http://blast.ncbi.nlm.nih.gov/Blast.cgi#148727255%23148727255) | 18.5 | 62% | 386 |
| [NP_653303.2](http://www.ncbi.nlm.nih.gov/entrez/query.fcgi?cmd=Retrieve&db=Protein&list_uids=163965441&dopt=GenPept&RID=TFNP80JC01N&log$=prottop&blast_rank=19) | leucine-rich repeat-containing protein C10orf92 [Homo sapiens] | [18.5](http://blast.ncbi.nlm.nih.gov/Blast.cgi#163965441%23163965441) | 30.1 | 100% | 386 |
| [NP_055787.1](http://www.ncbi.nlm.nih.gov/entrez/query.fcgi?cmd=Retrieve&db=Protein&list_uids=14149657&dopt=GenPept&RID=TFNP80JC01N&log$=prottop&blast_rank=20) | transcription factor 25 [Homo sapiens] | [18.5](http://blast.ncbi.nlm.nih.gov/Blast.cgi#14149657%2314149657) | 18.5 | 62% | 386 |
| [NP_009225.1](http://www.ncbi.nlm.nih.gov/entrez/query.fcgi?cmd=Retrieve&db=Protein&list_uids=6552299&dopt=GenPept&RID=TFNP80JC01N&log$=prottop&blast_rank=21) | breast cancer type 1 susceptibility protein isoform 1 [Homo sapiens] | [18.5](http://blast.ncbi.nlm.nih.gov/Blast.cgi#6552299%236552299) | 34.8 | 75% | 386 |
| [NP_002684.1](http://www.ncbi.nlm.nih.gov/entrez/query.fcgi?cmd=Retrieve&db=Protein&list_uids=4505937&dopt=GenPept&RID=TFNP80JC01N&log$=prottop&blast_rank=22) | DNA-directed DNA polymerase gamma [Homo sapiens] >ref|NP_001119603.1| DNA-directed DNA polymerase gamma [Homo sapiens] | [18.5](http://blast.ncbi.nlm.nih.gov/Blast.cgi#4505937%234505937) | 18.5 | 62% | 386 |
| [NP_055917.1](http://www.ncbi.nlm.nih.gov/entrez/query.fcgi?cmd=Retrieve&db=Protein&list_uids=23510323&dopt=GenPept&RID=TFNP80JC01N&log$=prottop&blast_rank=23) | nephroretinin [Homo sapiens] | [18.5](http://blast.ncbi.nlm.nih.gov/Blast.cgi#23510323%2323510323) | 18.5 | 75% | 386 |
| [XP_001716416.1](http://www.ncbi.nlm.nih.gov/entrez/query.fcgi?cmd=Retrieve&db=Protein&list_uids=169211554&dopt=GenPept&RID=TFNP80JC01N&log$=prottop&blast_rank=24) | PREDICTED: hypothetical protein [Homo sapiens] >ref|XP_001726833.1| PREDICTED: hypothetical protein [Homo sapiens] | [18.0](http://blast.ncbi.nlm.nih.gov/Blast.cgi#169211554%23169211554) | 18.0 | 75% | 518 |
| [XP_001721730.1](http://www.ncbi.nlm.nih.gov/entrez/query.fcgi?cmd=Retrieve&db=Protein&list_uids=169211178&dopt=GenPept&RID=TFNP80JC01N&log$=prottop&blast_rank=25) | PREDICTED: hypothetical protein [Homo sapiens] | [18.0](http://blast.ncbi.nlm.nih.gov/Blast.cgi#169211178%23169211178) | 18.0 | 75% | 518 |
| [NP_001096637.1](http://www.ncbi.nlm.nih.gov/entrez/query.fcgi?cmd=Retrieve&db=Protein&list_uids=157266269&dopt=GenPept&RID=TFNP80JC01N&log$=prottop&blast_rank=26) | GATA-like protein 1 [Homo sapiens] | [18.0](http://blast.ncbi.nlm.nih.gov/Blast.cgi#157266269%23157266269) | 18.0 | 87% | 518 |
| [NP_079052.2](http://www.ncbi.nlm.nih.gov/entrez/query.fcgi?cmd=Retrieve&db=Protein&list_uids=148368962&dopt=GenPept&RID=TFNP80JC01N&log$=prottop&blast_rank=27) | tyrosine-protein kinase SgK269 [Homo sapiens] | [18.0](http://blast.ncbi.nlm.nih.gov/Blast.cgi#148368962%23148368962) | 31.8 | 75% | 518 |
| [NP_055712.1](http://www.ncbi.nlm.nih.gov/entrez/query.fcgi?cmd=Retrieve&db=Protein&list_uids=7662384&dopt=GenPept&RID=TFNP80JC01N&log$=prottop&blast_rank=28) | zinc finger protein 652 [Homo sapiens] >ref|NP_001138837.1| zinc finger protein 652 [Homo sapiens] | [18.0](http://blast.ncbi.nlm.nih.gov/Blast.cgi#7662384%237662384) | 18.0 | 75% | 518 |
| [NP_001036002.1](http://www.ncbi.nlm.nih.gov/entrez/query.fcgi?cmd=Retrieve&db=Protein&list_uids=110227626&dopt=GenPept&RID=TFNP80JC01N&log$=prottop&blast_rank=29) | sodium/hydrogen exchanger 6 isoform a [Homo sapiens] | [17.6](http://blast.ncbi.nlm.nih.gov/Blast.cgi#110227626%23110227626) | 17.6 | 75% | 695 |
| [NP_660279.1](http://www.ncbi.nlm.nih.gov/entrez/query.fcgi?cmd=Retrieve&db=Protein&list_uids=21687139&dopt=GenPept&RID=TFNP80JC01N&log$=prottop&blast_rank=30) | UDP-GlcNAc:betaGal beta-1,3-N-acetylglucosaminyltransferase 7 [Homo sapiens] | [17.6](http://blast.ncbi.nlm.nih.gov/Blast.cgi#21687139%2321687139) | 17.6 | 75% | 695 |
| [NP_003895.1](http://www.ncbi.nlm.nih.gov/entrez/query.fcgi?cmd=Retrieve&db=Protein&list_uids=4508021&dopt=GenPept&RID=TFNP80JC01N&log$=prottop&blast_rank=31) | zinc finger protein 259 [Homo sapiens] | [17.6](http://blast.ncbi.nlm.nih.gov/Blast.cgi#4508021%234508021) | 17.6 | 100% | 695 |
| [NP_006350.1](http://www.ncbi.nlm.nih.gov/entrez/query.fcgi?cmd=Retrieve&db=Protein&list_uids=5454070&dopt=GenPept&RID=TFNP80JC01N&log$=prottop&blast_rank=32) | sodium/hydrogen exchanger 6 isoform b [Homo sapiens] | [17.6](http://blast.ncbi.nlm.nih.gov/Blast.cgi#5454070%235454070) | 17.6 | 75% | 695 |
| [NP_062540.2](http://www.ncbi.nlm.nih.gov/entrez/query.fcgi?cmd=Retrieve&db=Protein&list_uids=62241040&dopt=GenPept&RID=TFNP80JC01N&log$=prottop&blast_rank=33) | leucine rich repeat containing 8 family, member A [Homo sapiens] >ref|NP_001120716.1| leucine rich repeat containing 8 family, member A [Homo sapiens] >ref|NP_001120717.1| leucine rich repeat containing 8 family, member A [Homo sapiens] | [17.6](http://blast.ncbi.nlm.nih.gov/Blast.cgi#62241040%2362241040) | 17.6 | 87% | 695 |
| [NP_065760.1](http://www.ncbi.nlm.nih.gov/entrez/query.fcgi?cmd=Retrieve&db=Protein&list_uids=146219841&dopt=GenPept&RID=TFNP80JC01N&log$=prottop&blast_rank=34) | PNMA-like protein 2 [Homo sapiens] | [17.2](http://blast.ncbi.nlm.nih.gov/Blast.cgi#146219841%23146219841) | 17.2 | 75% | 932 |
| [NP_000932.3](http://www.ncbi.nlm.nih.gov/entrez/query.fcgi?cmd=Retrieve&db=Protein&list_uids=127139033&dopt=GenPept&RID=TFNP80JC01N&log$=prottop&blast_rank=35) | NADPH--cytochrome P450 reductase [Homo sapiens] | [17.2](http://blast.ncbi.nlm.nih.gov/Blast.cgi#127139033%23127139033) | 29.3 | 100% | 932 |
| [NP_001923.2](http://www.ncbi.nlm.nih.gov/entrez/query.fcgi?cmd=Retrieve&db=Protein&list_uids=21536464&dopt=GenPept&RID=TFNP80JC01N&log$=prottop&blast_rank=36) | MAGUK p55 subfamily member 3 [Homo sapiens] | [17.2](http://blast.ncbi.nlm.nih.gov/Blast.cgi#21536464%2321536464) | 17.2 | 75% | 932 |
| [NP_006017.1](http://www.ncbi.nlm.nih.gov/entrez/query.fcgi?cmd=Retrieve&db=Protein&list_uids=5174449&dopt=GenPept&RID=TFNP80JC01N&log$=prottop&blast_rank=37) | histone H1x [Homo sapiens] | [17.2](http://blast.ncbi.nlm.nih.gov/Blast.cgi#5174449%235174449) | 17.2 | 87% | 932 |
| [NP_055731.2](http://www.ncbi.nlm.nih.gov/entrez/query.fcgi?cmd=Retrieve&db=Protein&list_uids=38016937&dopt=GenPept&RID=TFNP80JC01N&log$=prottop&blast_rank=38) | lemur tyrosine kinase 2 precursor [Homo sapiens] | [17.2](http://blast.ncbi.nlm.nih.gov/Blast.cgi#38016937%2338016937) | 32.2 | 100% | 932 |
| [NP_001129416.1](http://www.ncbi.nlm.nih.gov/entrez/query.fcgi?cmd=Retrieve&db=Protein&list_uids=209863002&dopt=GenPept&RID=TFNP80JC01N&log$=prottop&blast_rank=39) | MAP kinase-activating death domain protein isoform j [Homo sapiens] | [16.8](http://blast.ncbi.nlm.nih.gov/Blast.cgi#209863002%23209863002) | 16.8 | 75% | 1251 |
| [NP_778236.2](http://www.ncbi.nlm.nih.gov/entrez/query.fcgi?cmd=Retrieve&db=Protein&list_uids=154759257&dopt=GenPept&RID=TFNP80JC01N&log$=prottop&blast_rank=40) | ATP-dependent RNA helicase DDX51 [Homo sapiens] | [16.8](http://blast.ncbi.nlm.nih.gov/Blast.cgi#154759257%23154759257) | 16.8 | 87% | 1251 |
| [NP_569828.2](http://www.ncbi.nlm.nih.gov/entrez/query.fcgi?cmd=Retrieve&db=Protein&list_uids=209862979&dopt=GenPept&RID=TFNP80JC01N&log$=prottop&blast_rank=41) | MAP kinase-activating death domain protein isoform c [Homo sapiens] | [16.8](http://blast.ncbi.nlm.nih.gov/Blast.cgi#209862979%23209862979) | 16.8 | 75% | 1251 |
| [NP_569827.2](http://www.ncbi.nlm.nih.gov/entrez/query.fcgi?cmd=Retrieve&db=Protein&list_uids=209862975&dopt=GenPept&RID=TFNP80JC01N&log$=prottop&blast_rank=42) | MAP kinase-activating death domain protein isoform b [Homo sapiens] | [16.8](http://blast.ncbi.nlm.nih.gov/Blast.cgi#209862975%23209862975) | 16.8 | 75% | 1251 |
| [NP_001129415.1](http://www.ncbi.nlm.nih.gov/entrez/query.fcgi?cmd=Retrieve&db=Protein&list_uids=209862998&dopt=GenPept&RID=TFNP80JC01N&log$=prottop&blast_rank=43) | MAP kinase-activating death domain protein isoform i [Homo sapiens] | [16.8](http://blast.ncbi.nlm.nih.gov/Blast.cgi#209862998%23209862998) | 16.8 | 75% | 1251 |
| [NP_003673.3](http://www.ncbi.nlm.nih.gov/entrez/query.fcgi?cmd=Retrieve&db=Protein&list_uids=209862994&dopt=GenPept&RID=TFNP80JC01N&log$=prottop&blast_rank=44) | MAP kinase-activating death domain protein isoform d [Homo sapiens] | [16.8](http://blast.ncbi.nlm.nih.gov/Blast.cgi#209862994%23209862994) | 16.8 | 75% | 1251 |
| [NP_569830.2](http://www.ncbi.nlm.nih.gov/entrez/query.fcgi?cmd=Retrieve&db=Protein&list_uids=209862987&dopt=GenPept&RID=TFNP80JC01N&log$=prottop&blast_rank=45) | MAP kinase-activating death domain protein isoform f [Homo sapiens] | [16.8](http://blast.ncbi.nlm.nih.gov/Blast.cgi#209862987%23209862987) | 16.8 | 75% | 1251 |
| [NP_569829.2](http://www.ncbi.nlm.nih.gov/entrez/query.fcgi?cmd=Retrieve&db=Protein&list_uids=209862983&dopt=GenPept&RID=TFNP80JC01N&log$=prottop&blast_rank=46) | MAP kinase-activating death domain protein isoform e [Homo sapiens] | [16.8](http://blast.ncbi.nlm.nih.gov/Blast.cgi#209862983%23209862983) | 16.8 | 75% | 1251 |
| [NP_002842.2](http://www.ncbi.nlm.nih.gov/entrez/query.fcgi?cmd=Retrieve&db=Protein&list_uids=91208428&dopt=GenPept&RID=TFNP80JC01N&log$=prottop&blast_rank=47) | protein tyrosine phosphatase, receptor-type, zeta1 precursor [Homo sapiens] | [16.8](http://blast.ncbi.nlm.nih.gov/Blast.cgi#91208428%2391208428) | 16.8 | 75% | 1251 |
| [NP_003449.2](http://www.ncbi.nlm.nih.gov/entrez/query.fcgi?cmd=Retrieve&db=Protein&list_uids=91208420&dopt=GenPept&RID=TFNP80JC01N&log$=prottop&blast_rank=48) | protein bassoon [Homo sapiens] | [16.8](http://blast.ncbi.nlm.nih.gov/Blast.cgi#91208420%2391208420) | 16.8 | 100% | 1251 |
| [NP_031361.2](http://www.ncbi.nlm.nih.gov/entrez/query.fcgi?cmd=Retrieve&db=Protein&list_uids=90669194&dopt=GenPept&RID=TFNP80JC01N&log$=prottop&blast_rank=49) | deleted in lung and esophageal cancer protein 1 isoform DLEC1-N1 [Homo sapiens] | [16.8](http://blast.ncbi.nlm.nih.gov/Blast.cgi#90669194%2390669194) | 16.8 | 75% | 1251 |
| [NP_031363.2](http://www.ncbi.nlm.nih.gov/entrez/query.fcgi?cmd=Retrieve&db=Protein&list_uids=90669228&dopt=GenPept&RID=TFNP80JC01N&log$=prottop&blast_rank=50) | deleted in lung and esophageal cancer protein 1 isoform DLEC1-S3 [Homo sapiens] | [16.8](http://blast.ncbi.nlm.nih.gov/Blast.cgi#90669228%2390669228) | 16.8 | 75% | 1251 |
| [NP_940862.2](http://www.ncbi.nlm.nih.gov/entrez/query.fcgi?cmd=Retrieve&db=Protein&list_uids=157671915&dopt=GenPept&RID=TFNP80JC01N&log$=prottop&blast_rank=51) | guanylate-binding protein 6 [Homo sapiens] | [16.8](http://blast.ncbi.nlm.nih.gov/Blast.cgi#157671915%23157671915) | 16.8 | 87% | 1251 |
| [NP_569826.2](http://www.ncbi.nlm.nih.gov/entrez/query.fcgi?cmd=Retrieve&db=Protein&list_uids=209862971&dopt=GenPept&RID=TFNP80JC01N&log$=prottop&blast_rank=52) | MAP kinase-activating death domain protein isoform a [Homo sapiens] | [16.8](http://blast.ncbi.nlm.nih.gov/Blast.cgi#209862971%23209862971) | 16.8 | 75% | 1251 |
| [NP_569831.1](http://www.ncbi.nlm.nih.gov/entrez/query.fcgi?cmd=Retrieve&db=Protein&list_uids=18860873&dopt=GenPept&RID=TFNP80JC01N&log$=prottop&blast_rank=53) | MAP kinase-activating death domain protein isoform g [Homo sapiens] | [16.8](http://blast.ncbi.nlm.nih.gov/Blast.cgi#18860873%2318860873) | 16.8 | 75% | 1251 |
| [NP_569832.2](http://www.ncbi.nlm.nih.gov/entrez/query.fcgi?cmd=Retrieve&db=Protein&list_uids=209571517&dopt=GenPept&RID=TFNP80JC01N&log$=prottop&blast_rank=54) | MAP kinase-activating death domain protein isoform h [Homo sapiens] | [16.8](http://blast.ncbi.nlm.nih.gov/Blast.cgi#209571517%23209571517) | 16.8 | 75% | 1251 |
| [NP_065716.1](http://www.ncbi.nlm.nih.gov/entrez/query.fcgi?cmd=Retrieve&db=Protein&list_uids=15042969&dopt=GenPept&RID=TFNP80JC01N&log$=prottop&blast_rank=55) | transmembrane protein 27 precursor [Homo sapiens] | [16.8](http://blast.ncbi.nlm.nih.gov/Blast.cgi#15042969%2315042969) | 16.8 | 75% | 1251 |
| [NP_065978.1](http://www.ncbi.nlm.nih.gov/entrez/query.fcgi?cmd=Retrieve&db=Protein&list_uids=24308257&dopt=GenPept&RID=TFNP80JC01N&log$=prottop&blast_rank=56) | synaptic vesicle membrane protein VAT-1 homolog-like [Homo sapiens] | [16.8](http://blast.ncbi.nlm.nih.gov/Blast.cgi#24308257%2324308257) | 16.8 | 75% | 1251 |
| [NP_057224.2](http://www.ncbi.nlm.nih.gov/entrez/query.fcgi?cmd=Retrieve&db=Protein&list_uids=56676375&dopt=GenPept&RID=TFNP80JC01N&log$=prottop&blast_rank=57) | tubulin polymerization-promoting protein family member 3 [Homo sapiens] >ref|NP_057048.2| tubulin polymerization-promoting protein family member 3 [Homo sapiens] | [16.8](http://blast.ncbi.nlm.nih.gov/Blast.cgi#56676375%2356676375) | 16.8 | 75% | 1251 |
| [NP_006358.1](http://www.ncbi.nlm.nih.gov/entrez/query.fcgi?cmd=Retrieve&db=Protein&list_uids=5453595&dopt=GenPept&RID=TFNP80JC01N&log$=prottop&blast_rank=58) | adenylyl cyclase-associated protein 1 [Homo sapiens] >ref|NP_001099000.1| adenylyl cyclase-associated protein 1 [Homo sapiens] | [16.8](http://blast.ncbi.nlm.nih.gov/Blast.cgi#5453595%235453595) | 16.8 | 87% | 1251 |
| [NP_000026.2](http://www.ncbi.nlm.nih.gov/entrez/query.fcgi?cmd=Retrieve&db=Protein&list_uids=40354205&dopt=GenPept&RID=TFNP80JC01N&log$=prottop&blast_rank=59) | fructose-bisphosphate aldolase B [Homo sapiens] | [16.8](http://blast.ncbi.nlm.nih.gov/Blast.cgi#40354205%2340354205) | 16.8 | 87% | 1251 |
| [NP_006779.1](http://www.ncbi.nlm.nih.gov/entrez/query.fcgi?cmd=Retrieve&db=Protein&list_uids=5803145&dopt=GenPept&RID=TFNP80JC01N&log$=prottop&blast_rank=60) | ralA binding protein 1 [Homo sapiens] | [16.8](http://blast.ncbi.nlm.nih.gov/Blast.cgi#5803145%235803145) | 16.8 | 75% | 1251 |
| [NP_689963.2](http://www.ncbi.nlm.nih.gov/entrez/query.fcgi?cmd=Retrieve&db=Protein&list_uids=40255133&dopt=GenPept&RID=TFNP80JC01N&log$=prottop&blast_rank=61) | cadherin-like protein 28 precursor [Homo sapiens] | [16.8](http://blast.ncbi.nlm.nih.gov/Blast.cgi#40255133%2340255133) | 16.8 | 75% | 1251 |
| [NP_001163887.1](http://www.ncbi.nlm.nih.gov/entrez/query.fcgi?cmd=Retrieve&db=Protein&list_uids=282165810&dopt=GenPept&RID=TFNP80JC01N&log$=prottop&blast_rank=62) | tight junction protein ZO-2 isoform 3 [Homo sapiens] | [16.3](http://blast.ncbi.nlm.nih.gov/Blast.cgi#282165810%23282165810) | 16.3 | 50% | 1679 |
| [NP_001163886.1](http://www.ncbi.nlm.nih.gov/entrez/query.fcgi?cmd=Retrieve&db=Protein&list_uids=282165804&dopt=GenPept&RID=TFNP80JC01N&log$=prottop&blast_rank=63) | tight junction protein ZO-2 isoform 4 [Homo sapiens] | [16.3](http://blast.ncbi.nlm.nih.gov/Blast.cgi#282165804%23282165804) | 16.3 | 50% | 1679 |
| [NP_001163885.1](http://www.ncbi.nlm.nih.gov/entrez/query.fcgi?cmd=Retrieve&db=Protein&list_uids=282165800&dopt=GenPept&RID=TFNP80JC01N&log$=prottop&blast_rank=64) | tight junction protein ZO-2 isoform 5 [Homo sapiens] | [16.3](http://blast.ncbi.nlm.nih.gov/Blast.cgi#282165800%23282165800) | 16.3 | 50% | 1679 |
| [NP_115518.3](http://www.ncbi.nlm.nih.gov/entrez/query.fcgi?cmd=Retrieve&db=Protein&list_uids=255003833&dopt=GenPept&RID=TFNP80JC01N&log$=prottop&blast_rank=65) | centrosomal protein of 192 kDa [Homo sapiens] | [16.3](http://blast.ncbi.nlm.nih.gov/Blast.cgi#255003833%23255003833) | 16.3 | 50% | 1679 |
| [XP_002345680.1](http://www.ncbi.nlm.nih.gov/entrez/query.fcgi?cmd=Retrieve&db=Protein&list_uids=239753500&dopt=GenPept&RID=TFNP80JC01N&log$=prottop&blast_rank=66) | PREDICTED: hypothetical protein [Homo sapiens] | [16.3](http://blast.ncbi.nlm.nih.gov/Blast.cgi#239753500%23239753500) | 16.3 | 75% | 1679 |
| [XP_942663.3](http://www.ncbi.nlm.nih.gov/entrez/query.fcgi?cmd=Retrieve&db=Protein&list_uids=239751865&dopt=GenPept&RID=TFNP80JC01N&log$=prottop&blast_rank=67) | PREDICTED: hypothetical LOC646508 [Homo sapiens] | [16.3](http://blast.ncbi.nlm.nih.gov/Blast.cgi#239751865%23239751865) | 31.8 | 62% | 1679 |
| [XP_002346919.1](http://www.ncbi.nlm.nih.gov/entrez/query.fcgi?cmd=Retrieve&db=Protein&list_uids=239749124&dopt=GenPept&RID=TFNP80JC01N&log$=prottop&blast_rank=68) | PREDICTED: zinc finger protein 716 [Homo sapiens] | [16.3](http://blast.ncbi.nlm.nih.gov/Blast.cgi#239749124%23239749124) | 16.3 | 50% | 1679 |
| [XP_002346459.1](http://www.ncbi.nlm.nih.gov/entrez/query.fcgi?cmd=Retrieve&db=Protein&list_uids=239747859&dopt=GenPept&RID=TFNP80JC01N&log$=prottop&blast_rank=69) | PREDICTED: hypothetical protein [Homo sapiens] | [16.3](http://blast.ncbi.nlm.nih.gov/Blast.cgi#239747859%23239747859) | 32.7 | 62% | 1679 |
| [XP_002342359.1](http://www.ncbi.nlm.nih.gov/entrez/query.fcgi?cmd=Retrieve&db=Protein&list_uids=239741944&dopt=GenPept&RID=TFNP80JC01N&log$=prottop&blast_rank=70) | PREDICTED: hypothetical protein XP_002342359 [Homo sapiens] >ref|XP_002346521.1| PREDICTED: hypothetical protein XP_002346521 [Homo sapiens] | [16.3](http://blast.ncbi.nlm.nih.gov/Blast.cgi#239741944%23239741944) | 16.3 | 75% | 1679 |
| [XP_002342309.1](http://www.ncbi.nlm.nih.gov/entrez/query.fcgi?cmd=Retrieve&db=Protein&list_uids=239741741&dopt=GenPept&RID=TFNP80JC01N&log$=prottop&blast_rank=71) | PREDICTED: hypothetical protein XP_002342309 [Homo sapiens] | [16.3](http://blast.ncbi.nlm.nih.gov/Blast.cgi#239741741%23239741741) | 16.3 | 50% | 1679 |
| [XP_002346034.1](http://www.ncbi.nlm.nih.gov/entrez/query.fcgi?cmd=Retrieve&db=Protein&list_uids=239754572&dopt=GenPept&RID=TFNP80JC01N&log$=prottop&blast_rank=72) | PREDICTED: zinc finger protein 716 [Homo sapiens] | [16.3](http://blast.ncbi.nlm.nih.gov/Blast.cgi#239754572%23239754572) | 16.3 | 50% | 1679 |
| [XP_002344355.1](http://www.ncbi.nlm.nih.gov/entrez/query.fcgi?cmd=Retrieve&db=Protein&list_uids=239508840&dopt=GenPept&RID=TFNP80JC01N&log$=prottop&blast_rank=73) | PREDICTED: zinc finger protein 716 [Homo sapiens] >ref|XP_002342768.1| PREDICTED: zinc finger protein 716 [Homo sapiens] | [16.3](http://blast.ncbi.nlm.nih.gov/Blast.cgi#239508840%23239508840) | 16.3 | 50% | 1679 |
| [NP_699194.2](http://www.ncbi.nlm.nih.gov/entrez/query.fcgi?cmd=Retrieve&db=Protein&list_uids=224586910&dopt=GenPept&RID=TFNP80JC01N&log$=prottop&blast_rank=74) | zinc finger protein 679 [Homo sapiens] | [16.3](http://blast.ncbi.nlm.nih.gov/Blast.cgi#224586910%23224586910) | 16.3 | 50% | 1679 |
| [NP_056667.2](http://www.ncbi.nlm.nih.gov/entrez/query.fcgi?cmd=Retrieve&db=Protein&list_uids=221316634&dopt=GenPept&RID=TFNP80JC01N&log$=prottop&blast_rank=75) | LIM domain only protein 7 isoform 2 [Homo sapiens] | [16.3](http://blast.ncbi.nlm.nih.gov/Blast.cgi#221316634%23221316634) | 16.3 | 50% | 1679 |
| [NP_001128476.1](http://www.ncbi.nlm.nih.gov/entrez/query.fcgi?cmd=Retrieve&db=Protein&list_uids=201862518&dopt=GenPept&RID=TFNP80JC01N&log$=prottop&blast_rank=76) | dnaJ homolog subfamily B member 5 isoform 2 [Homo sapiens] | [16.3](http://blast.ncbi.nlm.nih.gov/Blast.cgi#201862518%23201862518) | 16.3 | 50% | 1679 |
| [NP_659444.2](http://www.ncbi.nlm.nih.gov/entrez/query.fcgi?cmd=Retrieve&db=Protein&list_uids=194018484&dopt=GenPept&RID=TFNP80JC01N&log$=prottop&blast_rank=77) | NACHT, LRR and PYD domains-containing protein 11 [Homo sapiens] | [16.3](http://blast.ncbi.nlm.nih.gov/Blast.cgi#194018484%23194018484) | 16.3 | 50% | 1679 |
| [NP_001119580.1](http://www.ncbi.nlm.nih.gov/entrez/query.fcgi?cmd=Retrieve&db=Protein&list_uids=186910319&dopt=GenPept&RID=TFNP80JC01N&log$=prottop&blast_rank=78) | solute carrier family 12 member 3 isoform 3 [Homo sapiens] | [16.3](http://blast.ncbi.nlm.nih.gov/Blast.cgi#186910319%23186910319) | 16.3 | 50% | 1679 |
| [NP_001119579.1](http://www.ncbi.nlm.nih.gov/entrez/query.fcgi?cmd=Retrieve&db=Protein&list_uids=186910317&dopt=GenPept&RID=TFNP80JC01N&log$=prottop&blast_rank=79) | solute carrier family 12 member 3 isoform 2 [Homo sapiens] | [16.3](http://blast.ncbi.nlm.nih.gov/Blast.cgi#186910317%23186910317) | 16.3 | 50% | 1679 |
| [NP_000330.2](http://www.ncbi.nlm.nih.gov/entrez/query.fcgi?cmd=Retrieve&db=Protein&list_uids=186910315&dopt=GenPept&RID=TFNP80JC01N&log$=prottop&blast_rank=80) | solute carrier family 12 member 3 isoform 1 [Homo sapiens] | [16.3](http://blast.ncbi.nlm.nih.gov/Blast.cgi#186910315%23186910315) | 16.3 | 50% | 1679 |
| [NP_001116314.1](http://www.ncbi.nlm.nih.gov/entrez/query.fcgi?cmd=Retrieve&db=Protein&list_uids=170932526&dopt=GenPept&RID=TFNP80JC01N&log$=prottop&blast_rank=81) | nuclear receptor coactivator 7 isoform 2 [Homo sapiens] | [16.3](http://blast.ncbi.nlm.nih.gov/Blast.cgi#170932526%23170932526) | 16.3 | 50% | 1679 |
| [XP_001719513.1](http://www.ncbi.nlm.nih.gov/entrez/query.fcgi?cmd=Retrieve&db=Protein&list_uids=169218211&dopt=GenPept&RID=TFNP80JC01N&log$=prottop&blast_rank=82) | PREDICTED: similar to plectin 1, partial [Homo sapiens] | [16.3](http://blast.ncbi.nlm.nih.gov/Blast.cgi#169218211%23169218211) | 16.3 | 62% | 1679 |
| [XP_001724598.1](http://www.ncbi.nlm.nih.gov/entrez/query.fcgi?cmd=Retrieve&db=Protein&list_uids=169179790&dopt=GenPept&RID=TFNP80JC01N&log$=prottop&blast_rank=83) | PREDICTED: similar to potassium channel tetramerisation domain containing 9 [Homo sapiens] >ref|XP_001715936.1| PREDICTED: similar to potassium channel tetramerisation domain containing 9 [Homo sapiens] | [16.3](http://blast.ncbi.nlm.nih.gov/Blast.cgi#169179790%23169179790) | 16.3 | 50% | 1679 |
| [XP_001722670.1](http://www.ncbi.nlm.nih.gov/entrez/query.fcgi?cmd=Retrieve&db=Protein&list_uids=169179702&dopt=GenPept&RID=TFNP80JC01N&log$=prottop&blast_rank=84) | PREDICTED: hypothetical protein [Homo sapiens] >ref|XP_001725190.1| PREDICTED: hypothetical protein [Homo sapiens] | [16.3](http://blast.ncbi.nlm.nih.gov/Blast.cgi#169179702%23169179702) | 16.3 | 87% | 1679 |
| [XP_002344379.1](http://www.ncbi.nlm.nih.gov/entrez/query.fcgi?cmd=Retrieve&db=Protein&list_uids=239508879&dopt=GenPept&RID=TFNP80JC01N&log$=prottop&blast_rank=85) | PREDICTED: similar to zinc finger protein 208 [Homo sapiens] | [16.3](http://blast.ncbi.nlm.nih.gov/Blast.cgi#239508879%23239508879) | 16.3 | 50% | 1679 |
| [NP_001152996.1](http://www.ncbi.nlm.nih.gov/entrez/query.fcgi?cmd=Retrieve&db=Protein&list_uids=226958483&dopt=GenPept&RID=TFNP80JC01N&log$=prottop&blast_rank=86) | putative zinc finger protein 735 [Homo sapiens] | [16.3](http://blast.ncbi.nlm.nih.gov/Blast.cgi#226958483%23226958483) | 16.3 | 50% | 1679 |
| [XP_934528.2](http://www.ncbi.nlm.nih.gov/entrez/query.fcgi?cmd=Retrieve&db=Protein&list_uids=169213419&dopt=GenPept&RID=TFNP80JC01N&log$=prottop&blast_rank=87) | PREDICTED: hypothetical LOC646508 [Homo sapiens] >ref|XP_001718825.1| PREDICTED: hypothetical LOC646508 [Homo sapiens] | [16.3](http://blast.ncbi.nlm.nih.gov/Blast.cgi#169213419%23169213419) | 31.8 | 62% | 1679 |
| [XP_001726086.1](http://www.ncbi.nlm.nih.gov/entrez/query.fcgi?cmd=Retrieve&db=Protein&list_uids=169180181&dopt=GenPept&RID=TFNP80JC01N&log$=prottop&blast_rank=88) | PREDICTED: similar to potassium channel tetramerisation domain containing 9 [Homo sapiens] | [16.3](http://blast.ncbi.nlm.nih.gov/Blast.cgi#169180181%23169180181) | 16.3 | 50% | 1679 |
| [NP_008832.2](http://www.ncbi.nlm.nih.gov/entrez/query.fcgi?cmd=Retrieve&db=Protein&list_uids=156119615&dopt=GenPept&RID=TFNP80JC01N&log$=prottop&blast_rank=89) | myosin-IXa [Homo sapiens] | [16.3](http://blast.ncbi.nlm.nih.gov/Blast.cgi#156119615%23156119615) | 16.3 | 50% | 1679 |
| [NP_001164101.1](http://www.ncbi.nlm.nih.gov/entrez/query.fcgi?cmd=Retrieve&db=Protein&list_uids=282165706&dopt=GenPept&RID=TFNP80JC01N&log$=prottop&blast_rank=90) | tight junction protein ZO-2 isoform 6 [Homo sapiens] | [16.3](http://blast.ncbi.nlm.nih.gov/Blast.cgi#282165706%23282165706) | 16.3 | 50% | 1679 |
| [NP_071934.3](http://www.ncbi.nlm.nih.gov/entrez/query.fcgi?cmd=Retrieve&db=Protein&list_uids=149999380&dopt=GenPept&RID=TFNP80JC01N&log$=prottop&blast_rank=91) | inverted formin-2 isoform 1 [Homo sapiens] | [16.3](http://blast.ncbi.nlm.nih.gov/Blast.cgi#149999380%23149999380) | 28.8 | 75% | 1679 |
| [NP_001026884.3](http://www.ncbi.nlm.nih.gov/entrez/query.fcgi?cmd=Retrieve&db=Protein&list_uids=149999378&dopt=GenPept&RID=TFNP80JC01N&log$=prottop&blast_rank=92) | inverted formin-2 isoform 2 [Homo sapiens] | [16.3](http://blast.ncbi.nlm.nih.gov/Blast.cgi#149999378%23149999378) | 28.8 | 75% | 1679 |
| [NP_001091971.1](http://www.ncbi.nlm.nih.gov/entrez/query.fcgi?cmd=Retrieve&db=Protein&list_uids=148612857&dopt=GenPept&RID=TFNP80JC01N&log$=prottop&blast_rank=93) | sickle tail protein homolog isoform 3 [Homo sapiens] | [16.3](http://blast.ncbi.nlm.nih.gov/Blast.cgi#148612857%23148612857) | 31.4 | 87% | 1679 |
| [NP_055855.2](http://www.ncbi.nlm.nih.gov/entrez/query.fcgi?cmd=Retrieve&db=Protein&list_uids=121583483&dopt=GenPept&RID=TFNP80JC01N&log$=prottop&blast_rank=94) | 1-phosphatidylinositol-3-phosphate 5-kinase isoform 2 [Homo sapiens] | [16.3](http://blast.ncbi.nlm.nih.gov/Blast.cgi#121583483%23121583483) | 16.3 | 75% | 1679 |
| [NP_001001786.2](http://www.ncbi.nlm.nih.gov/entrez/query.fcgi?cmd=Retrieve&db=Protein&list_uids=221316605&dopt=GenPept&RID=TFNP80JC01N&log$=prottop&blast_rank=95) | BH3-like motif-containing cell death inducer [Homo sapiens] | [16.3](http://blast.ncbi.nlm.nih.gov/Blast.cgi#221316605%23221316605) | 16.3 | 50% | 1679 |
| [NP_060766.5](http://www.ncbi.nlm.nih.gov/entrez/query.fcgi?cmd=Retrieve&db=Protein&list_uids=239735605&dopt=GenPept&RID=TFNP80JC01N&log$=prottop&blast_rank=96) | leucine-rich repeat-containing protein 36 isoform 1 [Homo sapiens] | [16.3](http://blast.ncbi.nlm.nih.gov/Blast.cgi#239735605%23239735605) | 16.3 | 50% | 1679 |
| [NP_055845.1](http://www.ncbi.nlm.nih.gov/entrez/query.fcgi?cmd=Retrieve&db=Protein&list_uids=119874201&dopt=GenPept&RID=TFNP80JC01N&log$=prottop&blast_rank=97) | protein furry homolog-like [Homo sapiens] | [16.3](http://blast.ncbi.nlm.nih.gov/Blast.cgi#119874201%23119874201) | 16.3 | 50% | 1679 |
| [NP_001136197.1](http://www.ncbi.nlm.nih.gov/entrez/query.fcgi?cmd=Retrieve&db=Protein&list_uids=218505680&dopt=GenPept&RID=TFNP80JC01N&log$=prottop&blast_rank=98) | exoribonuclease 2 isoform 1 [Homo sapiens] | [16.3](http://blast.ncbi.nlm.nih.gov/Blast.cgi#218505680%23218505680) | 16.3 | 50% | 1679 |
| [NP_004875.2](http://www.ncbi.nlm.nih.gov/entrez/query.fcgi?cmd=Retrieve&db=Protein&list_uids=134244585&dopt=GenPept&RID=TFNP80JC01N&log$=prottop&blast_rank=99) | putative neuronal cell adhesion molecule precursor [Homo sapiens] | [16.3](http://blast.ncbi.nlm.nih.gov/Blast.cgi#134244585%23134244585) | 16.3 | 75% | 1679 |
| [NP_000827.2](http://www.ncbi.nlm.nih.gov/entrez/query.fcgi?cmd=Retrieve&db=Protein&list_uids=153946391&dopt=GenPept&RID=TFNP80JC01N&log$=prottop&blast_rank=100) | glutamate [NMDA] receptor subunit epsilon-4 precursor [Homo sapiens] | [16.3](http://blast.ncbi.nlm.nih.gov/Blast.cgi#153946391%23153946391) | 16.3 | 62% | 1679 |
